# Draft *de novo* transcriptome assembly and proteome characterization of the electric lobe of *Tetronarce californica*: a molecular tool for the study of cholinergic neurotransmission in the electric organ

**DOI:** 10.1186/s12864-017-3890-4

**Published:** 2017-08-14

**Authors:** Maria Stavrianakou, Ricardo Perez, Cheng Wu, Matthew S. Sachs, Rodolfo Aramayo, Mark Harlow

**Affiliations:** Department of Biology, Texas A&M University, 3258 TAMU, College Station, 77843-3258 USA

**Keywords:** *Torpedo californica*, Transcriptome assembly, Trinity assembly, Assembly clustering, Cholinergic neurotransmission, Fish genomes, Transporters, Reverse blast hit, Transporter classification database

## Abstract

**Background:**

The electric organ of *Tetronarce californica* (an electric ray formerly known as *Torpedo californica*) is a classic preparation for biochemical studies of cholinergic neurotransmission. To broaden the usefulness of this preparation, we have performed a transcriptome assembly of the presynaptic component of the electric organ (the electric lobe). We combined our assembled transcriptome with a previous transcriptome of the postsynaptic electric organ, to define a MetaProteome containing pre- and post-synaptic components of the electric organ.

**Results:**

Sequencing yielded 102 million paired-end 100 bp reads. *De novo* Trinity assembly was performed at Kmer 25 (default) and Kmers 27, 29, and 31. Trinity, generated around 103,000 transcripts, and 78,000 genes per assembly. Assemblies were evaluated based on the number of bases/transcripts assembled, RSEM-EVAL scores and informational content and completeness. We found that different assemblies scored differently according to the evaluation criteria used, and that while each individual assembly contained unique information, much of the assembly information was shared by all assemblies. To generate the presynaptic transcriptome (electric lobe), while capturing all information, assemblies were first clustered and then combined with postsynaptic transcripts (electric organ) downloaded from NCBI. The completness of the resulting clustered predicted MetaProteome was rigorously evaluated by comparing its information against the predicted proteomes from *Homo sapiens*, *Callorhinchus milli,* and the Transporter Classification Database (TCDB).

**Conclusions:**

In summary, we obtained a MetaProteome containing 92%, 88.5%, and 66% of the expected set of ultra-conserved sequences (i.e., BUSCOs), expected to be found for Eukaryotes, Metazoa, and Vertebrata, respectively. We cross-annotated the conserved set of proteins shared between the *T. californica* MetaProteome and the proteomes of *H. sapiens* and *C. milli*, using the *H. sapiens* genome as a reference. This information was used to predict the position in human pathways of the conserved members of the *T. californica* MetaProteome. We found proteins not detected before in *T. californica*, corresponding to processes involved in synaptic vesicle biology. Finally, we identified 42 transporter proteins in TCDB that were detected by the *T. californica* MetaProteome (electric fish) and not selected by a control proteome consisting of the combined proteomes of 12 widely diverse non-electric fishes by Reverse-Blast-Hit Blast. Combined, the information provided here is not only a unique tool for the study of cholinergic neurotransmission, but it is also a starting point for understanding the evolution of early vertebrates.

**Electronic supplementary material:**

The online version of this article (doi:10.1186/s12864-017-3890-4) contains supplementary material, which is available to authorized users.

## Background

**Fig. 1:**
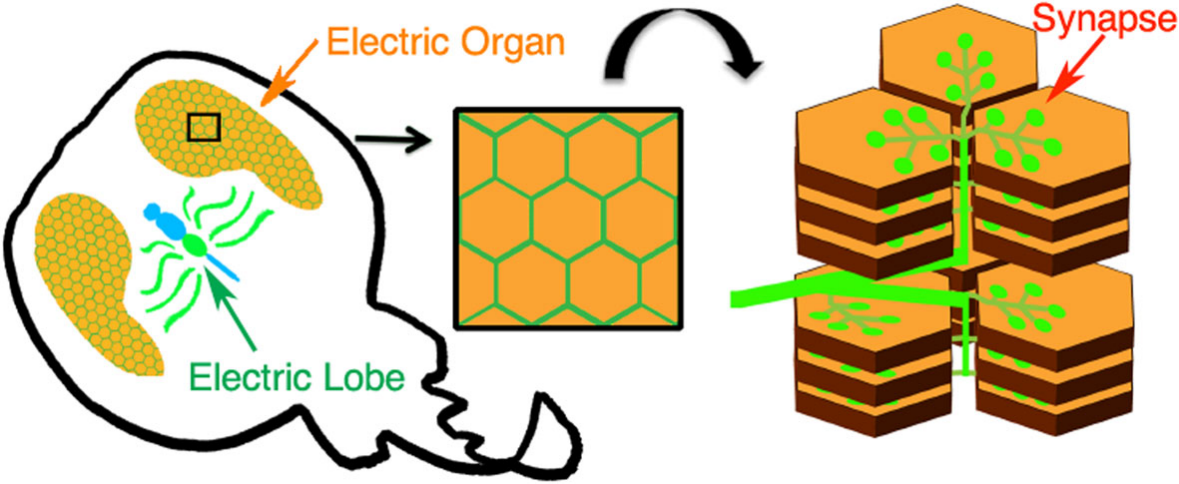
Cartoon depicting the electric lobe and the electric organ of *T. californica*. The electric lobe of *T. californica* resides within the central nervous system (CNS; Blue and Green, electric lobe: *Green arrow*) and possesses four paired electromotor nerves that project from the electric lobe of the CNS to the electric organ (*Orange arrow*) of the peripheral nervous system (PNS). From the surface, the electroplaque cells of the electric lobe appear as a honeycomb. Viewed from the side the electroplaque cells appear as large, pancake-like stacks. Electromotor nerves (*green*) branch into individual nerve fibers, and form synapses (*red arrow*) on the surface of electroplaque cells

Electric rays have a long history of scientific inquiry, dating back to ancient times [1]. The strong narcotizing powers that Aristotle and Plutarch discussed are the result of high voltage shocks (50-600 volts) that the rays can produce in a pair of specialized organs, termed electric organs, on either side of the rays’ bodies (Fig. 1). In fish, electric organs are thought to have evolved independently from primordial muscle tissue at least six or more times [2]. The organs are developmentally derived from an enlargement of the vertebrate neuromuscular junction (NMJ). In humans, NMJs are usually 30 microns in size, and occupy less than 0.1% of the muscle cell’s surface. In the rays, the electric organs begin life as muscle cells; however during development, the muscle cells lose their contractile apparatus and morphologically change into disc-like electroplaque cells that are approximately 1 cm in diameter and 10 microns in depth. The entire surface of one side of each disc is innervated by cholinergic presynaptic boutons; thus, a single electroplaque possesses 100,000 times more presynaptic innervation than a vertebrate muscle cell. The massive hypertrification of this synapse has made it a powerful model for the biochemical and physiological study of cholinergic nerve impulse transmission [3] of the synapse. A single ray, such as *Tetronarce californica*, can provide over 1 kg of tissue highly enriched in both pre and postsynaptic proteins involved in cholinergic transmission.

Molecular genomics tools have been rather limited for the rays. To date, only the transcriptome of the postsynaptic electric organ of *T. californica* has been studied [4, 5], and no organismal genomic sequences are yet available for these animals. In the ray, the cholinergic neurons that innervate the electric organ reside in a specialized pair of lobes within the central nervous system, termed the electric lobes (Fig. 1). The lack of a transcriptome from the presynaptic cholinergic neurons, located in the electric lobe, hinders our ability to study the presynaptic components of the synapse, and more generally, the lack of genomic and transcriptome information hinders the study of the evolution and ecology of these early vertebrates. To address this deficit, we have isolated mRNAs from the electric lobe of *T. californica*, and applied a Next Generation Sequencing (NGS) approach to provide the first transcriptome of the cholinergic presynaptic neurons that innervate the peripheral electric organs.

## Results and discussion

### Transcriptome assembly

**Fig. 2:**
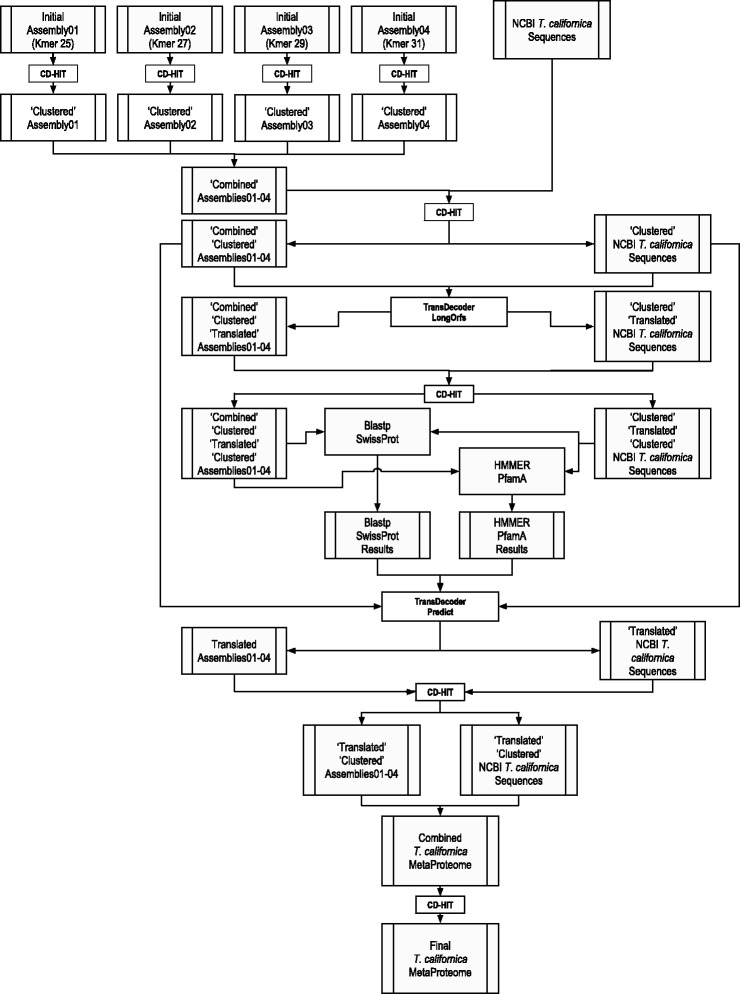
General Strategy. The logical outline of the main steps involved in assembly, clustering and Open Reading Frame (ORF) prediction of the *T. californica* electric lobe transcriptome and how this data was processed and combined with NCBI *T. californica* sequences is presented

We used a dataset of 102,431,406 paired reads, which, after Read Quality Control (RQC) represented approximately 96% of our initial dataset (106,453,074 reads. Additional file 1: Figure S01). We opted to use Trinity for assembly due to the robustness of the software, its well documented ability to resolve splice alternates, and to produce less duplicates [6, 7]. We performed assemblies at Kmers 25 (Trinity’s default, Assembly01), 27 (Assembly02), 29 (Assembly03), and 31 (Assembly04) (Fig. 2 and see Additional file 2).

### Transcriptome assembly evaluation

Evaluating a *de novo* transcriptome assembly is a challenging problem. Before accepting either one of our assemblies as the best one, and use it to predict a proteome, we first tested if any one of them contained all the information present in the other assemblies. We did this using the following criteria: 1) Number of bases assembled, 2) Number of transcripts assembled, 3) RSEM-EVAL scores, 4) Informational content, and 5) Informational completeness (BUSCO analysis).

We found that the total number of bases assembled in Assemblies01-to-04 was roughly equivalent, but not identical (Table 1). Using Kmer 25 (Assembly01), Trinity assembled approximately 6,795,400 bp more than Assembly04 (Kmer 31), but only 55,562 bp and 2,723,975 bp more than Assembly02 (Kmer 27) and Assembly03 (Kmer 29), respectively. Assembly01 also assembled the highest number of transcripts or transfragments (i.e., 104,902). Specifically, 58, 1,733, and 4,034 more transfragments than Assemblies 02, 03, and 04, respectively. Correcting for the number of isoforms assembled, Assembly01 similarly produced 614, 1,873, and 3,097 more genes than Assemblies 02, 03, and 04, respectively. Despite the marked differences in Assembly capabilities at different Kmers, the observed ratio of transcripts/genes was roughly 1.32 for all assemblies, suggesting that for Trinity, the number of isoforms assembled with this dataset remained constant using different Kmers. According to these criteria Assembly01 is the best assembly.

**Table 1:** Trinity assemblies

Parameters	Trinity					Trinity + CD-HIT-EST			
Assembly (Kmer)	Assembled Bases	Transcripts^a^	Genes^b^	Score^c^		Assembled Bases	Transcripts^a^	Genes^b^	Score^c^
01 (25)	132,763,501	104,902	79,707	-6105288705.62		122,806,861	95,964	72,213	-6115264651.77
02 (27)	132,707,939	104,844	79,093	-6053954533.82		122,869,910	95,906	71,537	-6110650415.91
03 (29)	130,039,526	103,169	77,834	-5967748885.21		120,327,821	94,415	70,473	-5976932627.27
04 (31)	125,968,101	100,868	76,610	-5932951277.04		116,388,010	92,306	69,295	-5939550282.37

^a^Number of Transcripts

^b^Number of Genes

^c^Detonate RSEM-EVAL Score

Next, we used Detonate [8], a package whose algorithm is designed for assessing true assembly. It does this by a reference-free evaluation method based on a novel probabilistic model that depends only on an assembly and the RNA-seq reads used for its construction. Using RSEM-EVAL, a component of the Detonate package, we scored our different assemblies (Table 1). We found that Assembly04 had a higher RSEM-EVAL score than any of the other assemblies. In fact Assembly01 rank was outperformed by Assemblies 02, 03, and 04. Clearly, neither the number of assembled bases, number of transcripts, nor number of genes correlated with RSEM-EVAL scores. Examination of the Detonate RSEM-EVAL results revealed both an inverse correlation between the number of contigs with no reads aligned to, and a direct correlation between the RSEM-EVAL scores and the number of alignable reads (Additional file 3: Table S01). Therefore, according to this criterion, Assembly04 is the best assembly.

In order to simplify these assemblies, we reduced sequence redundancy by applying the clustering algorithm optimized by CD-HIT [2, 9–13]. CD-HIT-EST has the potential of merging partially assembled transcripts (i.e., transfragments) into the longest assembled related sequence. We used stringent parameters (i.e., 100% sequence identity). We observed a reduction in the complexity of our assemblies (Table 1 and Additional file 3: Table S01). Comparing the Trinity + CD-HIT-EST assemblies to the original initial Trinity assemblies, we observed that the number of assembled bases in the Trinity + CD-HIT-EST assemblies was approximately 92% of the number of assembled bases in their corresponding original Trinity assemblies. Similarly, again we observed that Trinity + CD-HIT-EST assemblies contained approximately between 90% to 91.5% of the number of transcripts and genes when compared to their corresponding non-clustered original Trinity assemblies. The observed reduction was proportional for all four assemblies. Again, Trinity + CD-HIT-EST Assembly01 outperformed all other assemblies on the number of assembled bases, transcripts and genes. Importantly, as observed for the unclustered Trinity assemblies, the best clustered Trinity + CD-HIT-EST assembly, as determined by Detonate, was Assembly04 (Table 1). The clustered or ’flattened’ Assembly01 Detonate’s rank was, again, outperformed by clustered Assemblies 02, 03, and 04, even after applying the CD-HIT-EST algorithm, which resulted in a reduction of the assemblies’ complexity.

Looking at the clustered assemblies, we observed a direct correlation between the number of alignable reads and RSEM-EVAL scores (i.e., the more alignable reads, the better the RSEM-EVAL score). We also observed an inverse correlation between the number of contigs with no reads aligned to and RSEM-EVAL scores (i.e., the lower number of contig with no reads aligned, the higher the RSEM-EVAL score)(Additional file 3: Table S01). In all cases, RSEM-EVAL scores consistently pointed to the same best assembly regardless of the redundancy of the sample. This last result is important as it underscores the ability of Detonate RSEM-EVAL to correct for these duplications by its prior modeling of assemblies algorithm. Finally, it is worth noting that in all cases more than 87% of the reads that entered assembly mapped to the different transcriptome assemblies and that the best assembly as called by Detonate RSEM-EVAL has the highest number of mapped reads (Additional file 3: Table S01).

Next, we further evaluated these assemblies by looking at their ’informational’ content. We detected differences between assemblies by estimating the full-length transcript ’coverage’ of the different assembled transcripts, or as we prefer to call them, transfragments, when compared to the Uniprot_Sprot protein database with Blastx [14, 15]. We selected Uniprot_Sprot because this is a high quality database [16–19]. We started by running blastx using as a ’query’ transfragments corresponding to different assemblies and as ’subject’ proteins in Uniprot_Sprot database. We used a stringent E-Value (1e-20) and retrieved only the best hit for each alignment (i.e., max-target-seqs=1). Results were then processed using ‘analyze_blastPlus_topHit_coverage’, a script provided with the Trinity package, and summarized in Additional file 3: Table S02 and displayed in Additional file 1: Figure S02. We found on average 5,582 full length transcripts for all four original Trinity assemblies and, on average, 5,556 full length transcripts for all four Trinity + CD-HIT-EST assemblies. Looking just at the CD-HIT-EST processed assemblies we found that 9,783 transcripts (average of all four ‘flattened’ assemblies) covered 60% or higher length percentage of the proteins present in Uniprot_Sprot. The equivalent number for the Trinity + CD-HIT-EST assemblies was 9,880 transcripts. We were unable to observe marked differences between assemblies, as they all looked similar (see Additional file 1: Figure S02). Given that the Trinity assemblies clustered with CD-HIT-EST are less complex while retaining the same sequence information, we decided to concentrate on these ‘flattened’ or ‘non-redundant’ assemblies (that from now on will simply be called Assembly01, Assembly02, Assembly03, and Assembly04).

Aiming at detecting differences between assemblies, we performed the same comparison but this time from the subject or ‘database’ point of view. We asked if the assembled transfragments from each assembly could identify the same protein present in the database. We did this by using Uniprot_Sprot protein identifiers present in the ‘w_pct_hit_length’ output files of the ‘analyze_blastPlus_topHit_coverage’ script run before and then compared these results between assemblies. More specifically, we eliminated common Uniprot_Sprot identifiers present in the different assemblies and then counted the total number of different IDs that are present in one assembly (e.g., Assembly01) but absent in another (e.g., Assembly02). These results are summarized in Table 2. We observed that every assembly had informational content not present in other assemblies. In other words, each assembly assembled transfragments not assembled by other assemblies. For example, Assembly01 lacked 567, 780, and 889 protein hits (a total of 2,236) identified by Assemblies02, 03, and 04, respectively. By this criteria, Assembly04 (best assembly according to Detonate) was the most incomplete, lacking a total of 3,613 hits. In contrast, Assembly02 had a higher database-hit performance (or lower total missing hits) than any of the other assemblies. We concluded that Assembly02 has the highest ‘informational content’ when compared to the other assemblies.

**Table 2:** Informational assembly content comparison against Uniprot_Sprot database

Assembly (Kmer)					
Assembly (Kmer)	01 (25)	02 (27)	03 (29)	04 (31)	Total hits missing
01 (25)	0	567	780	889	2,236
02 (27)	808	0	579	763	2,150
03 (29)	1,188	746	0	540	2,474
04 (31)	1,579	1,212	822	0	3,613

Numbers represent hits present in one assembly (e.g., Assembly01 - Y axes) that are not present in the second assembly (e.g., Assembly02 - X axes). For example Assembly02 has 567, 746 and 1,212 hits not present in Assemblies 01, 03, and 04, respectively

To further understand the informational difference between the different assemblies, we performed a ‘between assemblies’ comparison. We did this in two different ways. First, we started by comparing the informational content of a given assembly (e.g., Assembly01) with that of another assembly (e.g., Assembly02). For this we used the CD-HIT-2D clustering algorithm, that compares two datasets and reports their differences. We performed a total of 16 clustering calculations (e.g., Assembly01 versus Assemblies 01 to 04), using as controls Assemblies 01 to 04 compared to themselves. These results are summarized in Table 3. Control clustering worked as expected producing zero information content differences for all four self calculations. However, we found differences in all our other experimental calculations. Again, Assembly02 by far had the most ’informational content’ as evidenced by the lower number (when compared to the other assemblies) of hits missing (Table 3), confirming previous observations (see above). Assembly02 lacked a total of 129,469 transfragments. In contrast, Assembly04, the best assembly according to Detonate score, lacked 159,752 total transfragments or, 30,283 more transfragments when compared to Assembly02. The ’best’ assembly according to its informational content was Assembly02, followed by Assemblies 03, 01, and 04, respectively. Given that these results evaluate actual sequence content, not just number of assembled transfragments, we think they are significant as they underscore the high variability intrinsically present in any assembly project, not just transcriptome assembly.

**Table 3:** Informational ’Between assemblies’ content comparison

Assembly (Kmer)					
Assembly (Kmer)	01 (25)	02 (27)	03 (29)	04 (31)	Total Hits Missing
01 (25)	0	43,431	47,710	50,034	141,175
02 (27)	43,553	0	40,938	44,978	129,469
03 (29)	53,099	44,554	0	39,412	137,065
04 (31)	60,668	54,740	44,344	0	159,752

Numbers represent transfragments present in one assembly (e.g., Assembly01 - Y axes) that are not present in the second assembly (e.g., Assembly02 - X axes). For example Assembly01 lacks 43,431, 47,710, and 50,034 transfragments present in Assemblies 02, 03, and 04, respectively

**Fig. 3:**
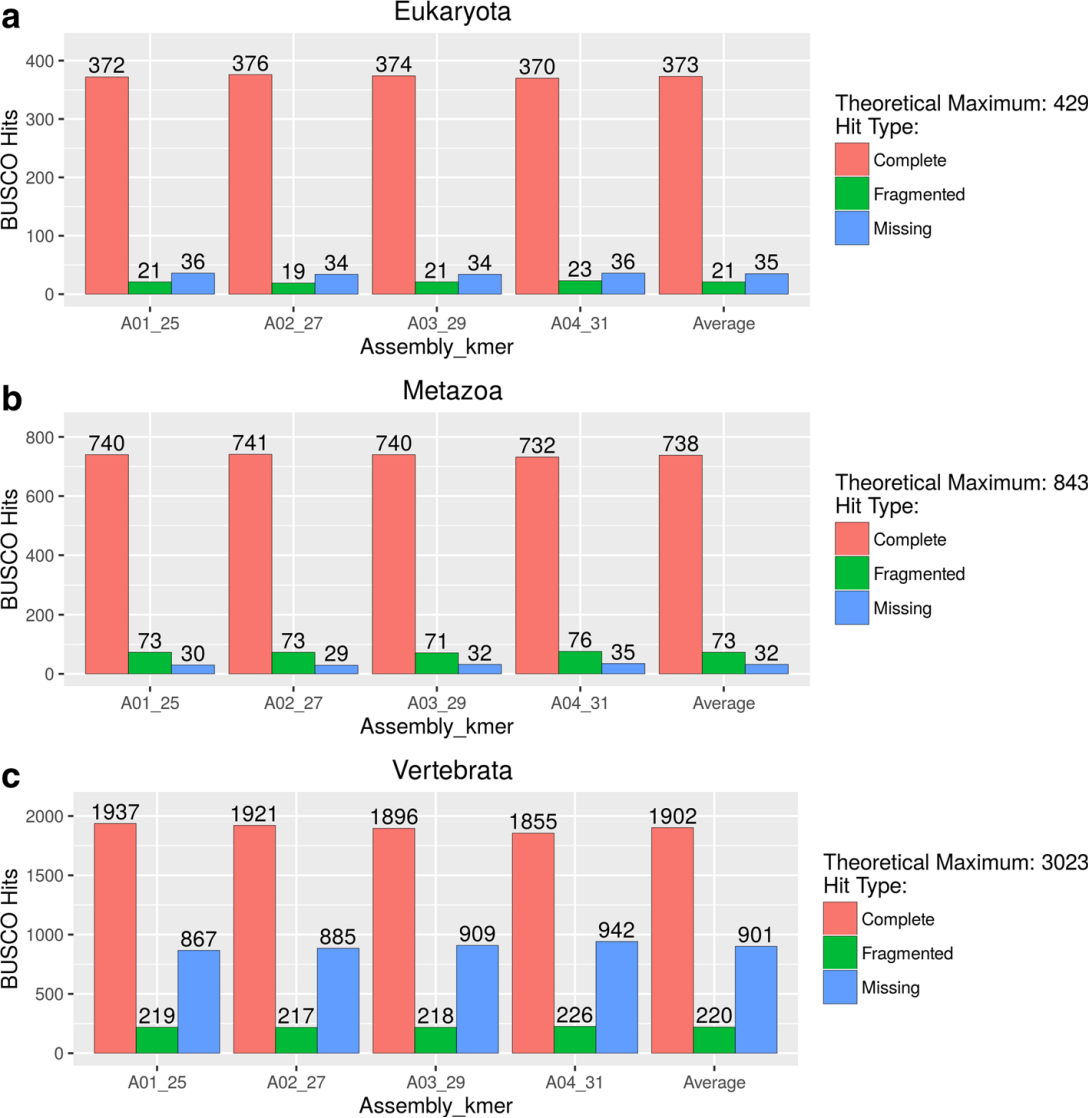
BUSCO Analysis of the Trinity + CD-HIT-EST Assemblies. Histograms of BUSCOs hits abundance detected in Assembly01 (25 Kmer – A01-25), Assembly02 (27 Kmer – A02-27), Assembly03 (29 Kmer – A03-29), Assembly04 (31 Kmer – A04-31) and the average values of Assemblies01-04 Average is presented for Eukaryota (Panel **a** ), Metazoa (Panel **b** ) and Vertebrata (Panel **c** )

Finally, we assessed the ’completeness’ of our ’flattened’ assemblies using the Benchmarking Universal Single-Copy Orthologs (BUSCO) program, using three major phylogenetic clades as measure (Eukaryota, Metazoa, and Vertebrata, Fig. 3 and Additional file 3: Table S03). BUSCO queries the OrthDB database [20], searching for highly conserved sequences present in a given clade. For example, we expected to find 429, 843, and 3,023 sets of conserved sequences in Eukaryotes, Metazoa, and Vertebrata, respectively. Looking at the Eukaryotic lineage, on average, our assemblies contained 373 complete BUSCO hits. This represents 87% of the expected set of 429 hits. Results for the Metazoa and Vertebrata lineages contain an average total of 738 and 1,902 hits, respectively, which represents 87.5% and 62.9% of the expected sets of 843 and 3,023 hits for Metazoa and Vertebrata, respectively. Given that we are looking at the assembly of transcripts corresponding to a highly specialized electric lobe, we think, these results are consistent with the hypothesis that the data in question are representative. Note that we have not taken into consideration the presence of fragmented hits in our analysis, which would enhance our results. Also, note that we clearly have missing hits for all clades. In the absence of genome assembly data, the significance of this last finding is impossible to evaluate. Finally, the data obtained with BUSCO was equivalent for all assemblies.

Based on these results it is hard to select a single “best assembly”. First, we were unable to select a given assembly based on the RSEM-EVAL score. Second, we found that each one of the individual assemblies obtained had informational content not present in the other assemblies. This ’informational-content’ difference was evident when the different assemblies were queried against standard databases. We used a ’complexity-reduction’ strategy designed to capture the unique information generated by the different assemblies by clustering them using very stringent parameters (i.e., 100% ID). This resulted in a substantial redundancy reduction, while preserving new information. Our results emphasize the need to evaluate assembly results critically and not just accept a given assembly as the best assembly using a single parameter. Results here obtained emphasize the complexity of transcriptome assembly. Even when using one of the best transcriptome assemblers available to date (i.e. Trinity), the complexity of transcriptome assembly should not be underestimated. Results coming from a single assembly must be taken with caution. While it has been established that assemblies at different Kmers generates a potentially larger set of assembled transfragments and elegant solutions have been proposed [21–27], finding a unified final solution to this problem is still an area of active investigation.

### Defining the conserved proteome of the electric organ

The main motivation for this work was to identify key presynaptic proteins of the electric lobe that are important for the structure and function of the fish electric organ, and to combine these with proteins identified in previous transcriptomes of the postsynaptic organ (see Fig. 1). We defined the conserved proteome in three general steps: 1) The longest open reading frame (ORF) of each assembled transfragment was extracted, making sure not to leave behind any conserved and/or potentially functional smaller ORFs. 2) All available public records corresponding to *T. californica* deposited in the NCBI (GeneBank), were downloaded and processed. 3) Potential proteins sequences identified in steps 1 and 2 above were combined and characterized (see Fig. 2 for a general outline).

#### 1. Defining the assembled presynaptic electric lobe proteome

To define our assembled proteome, without leaving behind any information, we first combined Assemblies 01, 02, 03, and 04 into a single file. This combined assembly, called ’Combined-Assemblies01-04’ (Fig. 2), contained a total of 378,591 assembled transfragments. This file was then ‘flattened’ using CD-HIT-EST (at 100% ID) to generate a file (Combined-Clustered-Assemblies01-04) containing 180,840 transfragments (or 47.8% of the original sequences). We then used ‘TransDecoder.LongOrfs’ to extract the longest ORF from each transfragment. The resulting file (Combined-Clustered-Translated-Assemblies01-04) had 1,057,426 proteins. The complexity of this file was reduced to 211,589 proteins with CD-HIT (100% ID). The final file (Combined-Clustered-Translated-Clustered-Assemblies01-04) was then used as a ’query’ for blastp searches against Uniprot_Sprot and HMMER searches against PfamA. The final Assembled Proteome file was generated by running ’TransDecoder.Predict’, using the Combined-Clustered-Assemblies01-04 file and the results of both Blastp and Pfam searches to generate a set of 124,536 predicted assembled proteins. The nucleotide sequences corresponding to these predicted proteins were screened for those that were shorter than 200 bp. We found the presence of 2,896 sequences smaller than 200 bp. Although the 2,896 sequences were used in downstream analyses, they were separated for sequence submission (see Availability of Data Materials for details). The final file contained 121,640 predicted assembled proteins (Translated-Assemblies01-04. Fig. 2).

#### 2. Defining the publicly available postsynaptic electric organ proteome

After downloading all currently available sequences from NCBI, we followed the same logic outlined above. We started by processing 10,185 transcripts (file NCBI-Tcalifornica-Sequences) that clustered into 9,099 unique transcripts (Clustered-NCBI-Tcalifornica-Sequences). We obtained 18,404 peptides after ‘TransDecoder.LongOrfs’ translation (to get Clustered-Translated-NCBI-Tcalifornica-Sequences). This set produced 13,129 peptides after CD-HIT (Clustered-Translated-Clustered-NCBI-Tcalifornica-Sequences). The resulting peptides were then used for both Blastp and Pfam searches. Finally, we extracted a set of 6,490 proteins after running ‘TransDecoder.Predict’ (Translated-NCBI-Tcalifornica-Sequences).

#### 3. Defining the MetaProteome

The MetaProteome of the electric organ was defined by combining files Translated-Assemblies01-04 + Translated-NCBI-Tcalifornica-Sequences to generate ‘Combined-Tcalifornica-MetaProteome’, containing a total of 128,130 proteins. This combined file was then further clustered to generate a final file called Combined-Clustered-Tcalifornica-MetaProteome containing a set of 74,195 predicted proteins. The resulting final set of 74,195 predicted proteins was defined as our MetaProteome (see Additional file 4: for details). In summary, of the 74,195 predicted MetaProteomic proteins, 70,338 proteins (98.8%) come from the presynaptic transcriptome, while only 3,857 proteins (5.2%) originated in the postsynaptic transcriptome.

### Evaluationg the MetaProteome

**Fig. 4:**
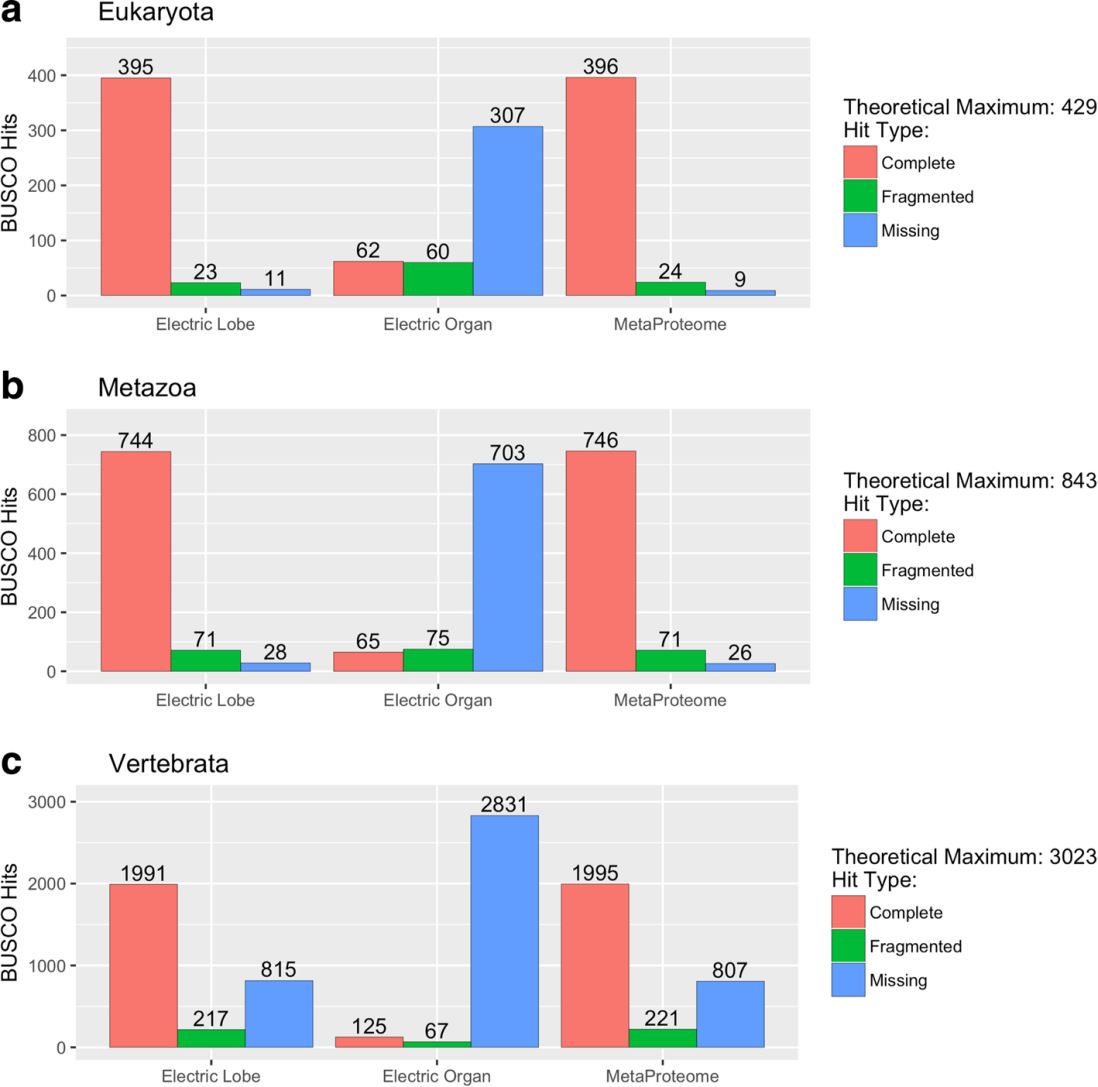
BUSCO Analysis of the *Tetronarce californica* Proteome. Histograms of BUSCOs hits abundance detected in Electric Lobe, Electric Organ, and MetaProteome datasets is presented for Eukaryota (Panel **a** ), Metazoa (Panel **b** ) and Vertebrata (Panel **c** )

The completeness of these different proteomes was assessed using BUSCO (as described before). These results are presented in Fig. 4 and summarized in Additional file 3: Table S04. Comparing the informational content of the electric lobe (presynaptic) versus that of the electric organ (postsynaptic) for Eukaryota, Metazoa, and Vertebrata lineage BUSCOs, we observed that the lack of 11 versus 307, 28 versus 703, and 815 versus 2,831 Eukaryota, Vertebrata, and Metazoa BUSCOs, respectively. We conclude that most of the MetaProteome information presented here originated from our assembled presynaptic transcriptome. The MetaProteome contains 92%, 88.5%, and 66.9% of the expected BUSCOs corresponding to the Eukaryota, Metazoa, and Vertebrata clades, respectively. These results are consistent with the hypothesis that the MetaProteome dataset is nearly complete for highly conserved genes and thus is likely to be representative of this particular tissue and developmental stage.

### Analysis of the MetaProteome against *Uniprot_Sprot* database

To determine the full-length ’coverage’ of the MetaProteome, we performed a Blastp search against Uniprot_Sprot. We wanted to determine how many of the Metaproteome sequences were complete. We observed an increase in the total number of hits to the database (from 15,929 to 17,615; compare Additional file 3: Tables S02 with S05), as expected. Similarly, the total number of Uniprot_Sprot proteins with a coverage percentage of 60% or more, jumped from 9,783 to 10,642 (859 more). We extracted the UniProt Identifiers of proteins who had a coverage of 70% or higher (9,600. Additional file 5). Using these IDs and the Panther database [28–31], we identified the Gene-Ontology (GO) terms associated with these proteins. Looking at the ‘Pathway’ category, we found enrichment in signaling pathways like Gonadotropin-releasing hormone receptor, CCRK, Wnt, Integrin, and Huntington disease pathways. In the ’Protein Class’ category, the highest percentage hits was against terms associated with RNA binding proteins like translation factors, mRNA processing, and ribosomal proteins. In addition, we observed enrichment associated with macromolecular complexes of the nervous system, for example tubulin, the SNARE proteins, Vesicle Coat proteins, among others. We also observed enrichment in terms associated with phosphoprotein phosphatase hydrolase activity and non-membrane spanning protein tyrosine kinase activity. We observed enrichment for the Alzheimer disease-amyloid secretase pathway. These observations support the notion that the electric organ is a good system to study the biochemistry of important signaling pathways. The compiled MetaProteome thus provides a good starting point to understand the biology of how the electric organ works (Additional file 5).

### Evolutionary analysis of the MetaProteome

To understand the biology of the *T. californica* electric organ MetaProteome from an evolutionary point of view, we performed two broadly different analysis. In the first one, the MetaProteome was compared against the proteomes of largely different genomes: *H. sapiens* and *C. milli* (Elephant Shark). In the second one, transporter proteins were detected in the MetaProteome and the combined proteomes of 12 fish genomes (Additional file 3: Table S06), by comparing them against the Transporter Classification Database (TCDB) [32–35].

### 1. Comparative analysis of the MetaProteome against the human and elephant shark genomes

**Fig. 5:**
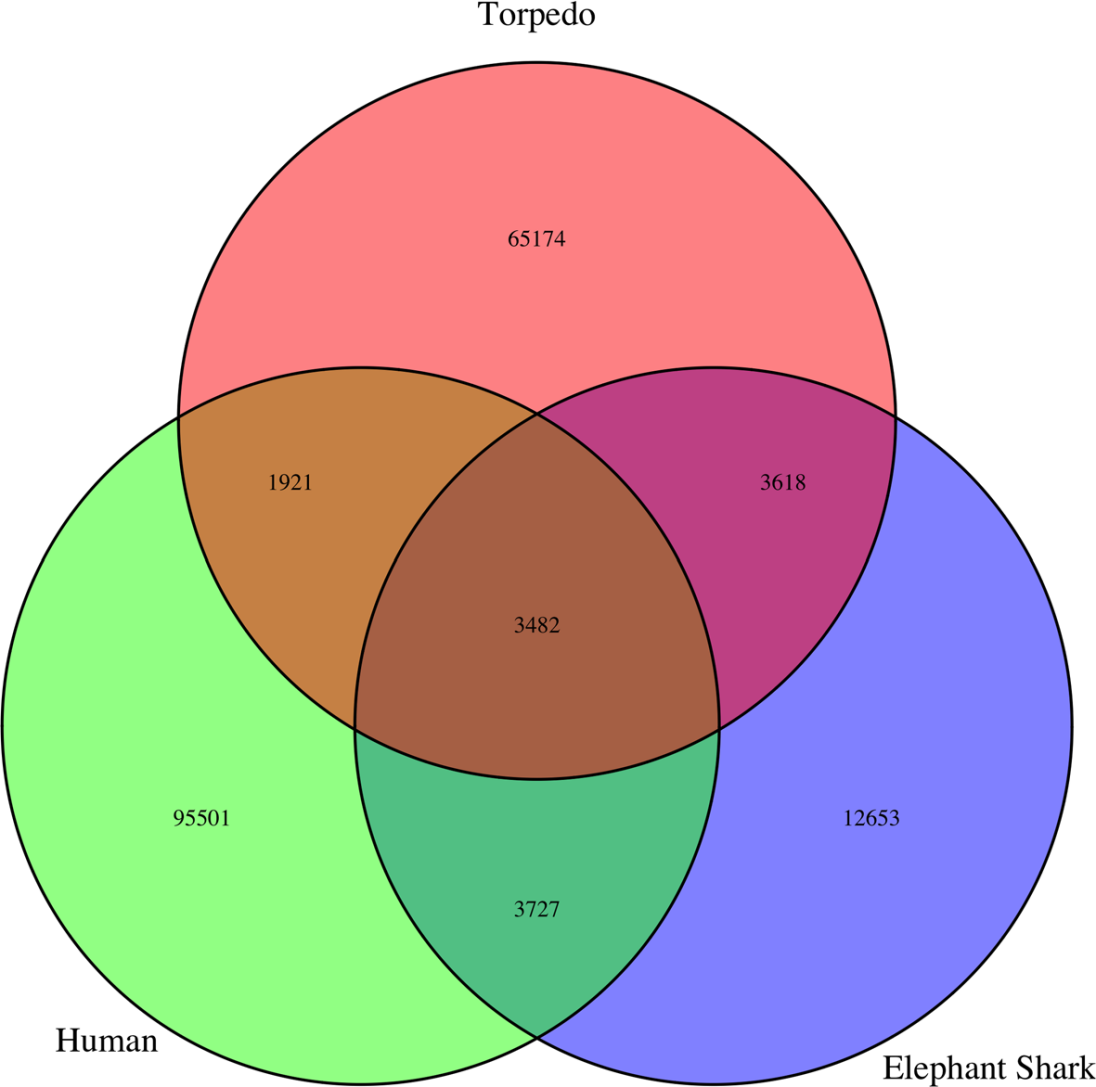
Relationships Between Proteomes from *T. californica*, *H. sapiens* and *C. milli*. Venn diagram of the intersection of the protein datasets corresponding to *T. californica* (Torpedo), *H. sapiens* (Human), and *C. milli* (Elephant Shark) is presented. Numbers represent proteins shared by these datasets (see Additional file 3: Table S07 for details)

The MetaProteome was compared against the *H. sapiens* and *C. milli* (Elephant Shark) [27] genomes (see Additional file 6: (see AF06A and AF06B for details). This last genome was selected because like *T. californica*, *C. milli* are jawed vertebrates (gnathostomes) that have boneless skeleton made of a tough elastic substance, but unlike *T. californica*, they are *Holocephali* (i.e., they have ’complete heads’) and do not have a flat body. We performed three related protein-comparison experiments: First, we determined the set of *T. californica* proteins homologous to *H. sapiens*. Second, we determined the set of *T. californica* proteins homologous to *C. milli*. Finally, we determined the set of *H. sapiens* proteins related to *C. milli*. In all cases we used the reciprocal blast hit algorithm (RBH-Blast) designed to identify orthologous protein pairs between two genomes [14]. To ensure the future reproducibility of these results, we started by first reducing the complexity of the starting proteomes using CD-HIT at 100% Identity. This resulted in a reduction of the *H. sapiens* proteome dataset from 151,569 to 104,631 proteins. Similarly, we obtained a reduction of the *C. milli* dataset from 28,237 to 23,480 proteins. In all cases we run RBH-Blast looking for matching pairs having 60% or higher identity (ID) and 50% or higher overlap or coverage. Results from these experiments are presented in Fig. 5 and summarized in Additional file 3: Table S07.

The number of orthologous pairs found between *T. californica* and *C. milli* was 7,100, between *T. californica* and *H. sapiens* was 5,403, and between *H. sapiens*, and *C. milli* was 7,209 (Additional file 7, Additional file 8, and Additional file 9). The number of orthologous pairs found to be uniquely shared between *T. californica* and *C. milli* (i.e., excluding *H. sapiens*) was 3,618, between *T. californica* and *H. sapiens* (i.e., excluding *C. milli*) was 1,921, and between *H. sapiens* and *C. milli* (i.e., excluding *T. californica*) was 3,727 (Additional file 10, Additional file 11, and Additional file 12). We calculated that 3,482 proteins define the common set shared by all three datasets (Additional file 13).

Searching for extremely conserved proteins among those corresponding to the intersection of these three datasets (i.e., having 100% coverage), we found one, and only one protein, belonging to the Serine/Threonine-protein Phosphatase 4 Regulatory Subunit 3B, a 849 aa protein, essential for cell division that is also involved in regulation of gluconeogenesis, lipid metabolism, and protein dephosphorylation (sp|Q5MIZ7|P4R3B_HUMAN) [36, 37]. Not only was this the only common protein found, but it was also the only one shared at this level of homology between the *T. californica* and the *C. milli* datasets. Interestingly, we detected 1,061 such conserved proteins shared between *H. sapiens* and *C. milli* (Additional file 14). The majority of these proteins (i.e., 81%) can also be detected in the intersection between *H. sapiens* and *T. californica*, when the coverage is lowered to 90%. Among the 5,403 proteins shared between *H. sapiens* and *T. californica*, we find that 3,224 (i.e., 60%) have a coverage of at least 90% (see Table 4). These results are consistent with *H. sapiens* and *C. milli* having a more recent last-common ancestor than the one shared between *H. sapiens* and *T. californica*. This notion is also in agreement with the higher number of proteins shared between *H. sapiens* and *C. milli*, than the number shared between *H. sapiens* and *T. californica*. The GO terms associated with proteins shared by these three species were retrieved and found to be enriched in RNA metabolism, sugar metabolism, and energy production. These points to processes that have been conserved through evolution in all these three species (Additional file 15: and Additional file 3: Table S07).

**Table 4:** Percentage conservation of proteins between proteomes of *T. californica* and *H. sapiens*

Hit coverage percentage:	100	99-95	94-90	89-80	79-70	69-60	59-50	Total
Number of hits:	1	2,380	843	910	469	410	390	5,406

Comparing and contrasting GO terms retrieved by the set of proteins present only in the *H. sapiens* versus *T. californica* (not *C. milli*) with those present in the *H. sapiens* versus *C. milli* (not *T. californica*) (Additional file 16 and Additional file 17, respectively), we find a distinctive pattern: the *H. sapiens* versus *T. californica* set is highly enriched in terms associated with Axon guidance mediated by Slit/Robo, *Beta3* adrenergic receptor signaling pathway, Opioid prodynorphin pathway, Opioid proopiomelanocortin pathway, Metabotropic glutamate receptor group II pathway, and Muscarinic acetylcholine receptor 2 and 4 signaling pathway, among others (Additional file 16). In contrast, terms associated with the *H. sapiens* versus *C. milli* set are distinctively enriched with terms related to Alzheimer disease-presenilin pathway, Cadherin signaling pathway, and Heterotrimeric G-protein signaling pathway-Gi *alpha* and Gs *alpha* mediated pathway (Additional file 17). Similarly, among the protein classes observed in the *H. sapiens* versus *T. californica* only set, we detect acetyltransferase, G-protein, membrane traffic protein and RNA binding, whereas the terms we observed in the *H. sapiens* versus *C. milli* set are enriched in homeobox -related helix-turn-helix transcription factors and ion channel transporters. Overall there is a clear pattern that favors terms associated with RNA binding, vesicle, membrane, and synaptic signalling in the protein set shared only by *H. sapiens* and *T. californica* versus *wnt/frizzled*, phototransduction in those proteins shared by *H. sapiens* and *C. milli*.

**Fig. 6:**
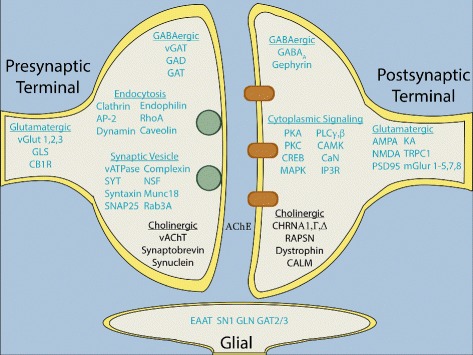
Synaptic Signalling Pathways Identified in MetaProteome of *T. californica*. Predicted presynaptic terminal, postsynaptic terminal, and glial proteins present in the *T. californica* Metaproteome, are shown in blue. These newly identified proteins include the majority of proteins involved in synaptic vesicle exocytosis and endocytosis and proteins specific to glutamatergic and GABAergic synapses, as well as proteins specific to glial and postsynaptic signalling pathways. Previously available synaptic signalling pathways, consisting primarily of the cholinergic pathways of the electric organ, shown in black. These *T. californica* proteins were identified as having at least 60% identity and 50% coverage to their *H. sapiens* counterparts

Given that the value of the information generated by any sequencing and/or assembly project is directly related to the degree of information-associated (or annotation) of the sequences in question, we classified and organized the distribution of the proteins shared between *H. sapiens*, *T. californica*, and *C. milli*, in well annotated Human biochemical pathways. First, we extracted Uniprot identifiers corresponding to the intersections of: 1) *H. sapiens* with *T. californica*, without *C. milli* (Additional file 11) 2) *H. sapiens* with *C. milli*, without *T. californica* (Additional file 12); 3) *H. sapiens* with *T. californica* (Additional file 8); and 4) *H. sapiens* with *C. milli* (Additional file 9). Second, these IDs were then mapped to human pathways using the Kyoto Encyclopedia of Genes and Genomes (KEGG) [38], and results of these mappings were displayed in Additional files 18, 19, 20, and 21, respectively. The large amount of information obtained represents a first guide to the distribution of the *T. californica* MetaProteome proteins in *H. sapiens* metabolic and developmental pathways. We used this information to compile a list of human pathways involved in neurological processes and determined the proteins present in these pathways (Table 5). Comparing the informational content of our own assembled presynaptic with the previously identified postsynaptic transcriptome, we found that most of the information contained in the MetaProteome originated from our assembled presynaptic transcriptome. We also found hits against important neurological and metabolic human pathways (see Table 5 and selected Figures in Additional file 22). We compiled a list of Synaptic and Glial proteins present in the electric organ. We found proteins not detected before, corresponding to processes involved in synaptic vesicle exocytosis/endocytosis and proteins specific to glutamatergic and GABAergic synapses, in addition to proteins specific to glial and postsynaptic signalling pathways (Table 5 and Fig. 6).

**Table 5:** Number of *T. californica* proteins present in important Neurobiological human pathways

	Transcriptome		
Pathway	Meta	Pre-synaptic	Post-synaptic
Metabolic pathways	460	445	18
Endocytosis	108	105	5
Alzheimer’s disease	66	61	5
Parkinson’s disease	58	52	6
Dopaminergic synapse	57	57	ND^a^
Axon guidance	54	54	ND^a^
Glutamatergic synapse	50	50	ND^a^
Neurotrophin signaling pathway	50	50	ND^a^
GABAergic synapse	42	41	1
Cholinergic synapse	39	39	ND^a^
Synaptic vesicle cycle	35	33	ND^a^
Amyotrophic lateral sclerosis (ALS)	22	22	ND^a^
SNARE interactions in vesicular transport	22	21	ND^a^

^a^Not detected

### 2. Analysis of the Proteomes of *T. californica* and Twelve Fish Genomes Against the Transporter Classification Database (TCDB)

Electricity generated by the electric organ of *T. californica* is the result of a highly synchronized neurotransmitter-mediated depolarization. To identify transporter proteins potentially involved in this massive cellular depolarization, we queried the MetaProteome of *T. californica* (an electric fish) and the combined proteomes of 12 evolutionarily diverse non-electric fishes (Additional file 3: Table S06), against the Transporter Classification DataBase (TCDB). We selected the TCDB database because it contains an exhaustive and well-curated list of transporter proteins [32–35]. Our starting hypothesis was that our MetaProteome should be enriched in a set of transporters preferentially found in the electric fish. This analysis was performed in three steps: 1) Our query sequences (i.e., the *T. californica* MetaProteome and the proteomes of the 12 individual fish) were first clustered (using CD-HIT, as described before). The individually-clustered fish proteomes were then combined into a single file and clustered again. The resulting file (12-Fish-CD-HIT) containing all the unique information encoded by the 12 fish genomes selected, contained 326,455 unique proteins. 2) A similar sequence duplication removal strategy was followed to process the 14,961 transporter proteins in the TCDB database that, after clustering, were reduced to 14,901 proteins. 3) We then performed two different RBH-Blasts looking for matching pairs having 60% or higher identity and 50% or higher coverage, between the *T. californica* and the 12-Fish-CD-HIT proteomes, each against TCDB. Results from these experiments are summarized in Table 6 and presented in Additional file 6: see AF06F, AF06G and Additional file 23. The combined 12-Fish-CD-HIT proteome identified a total of 923 transporters, while the *T. californica* MetaProteome identified 417 transporters (i.e., 6.2% and 2.8% of the TCDB, respectively). Both the 12-Fish-CD-HIT and *T. californica* proteomes had 375 hits in common. Among the 38 classes of transporters present in the TCDB database, we found a higher number of hits (i.e., five hits or more) against 15 well-defined Eukaryotic transporter classes (Table 6). We found that, in general, both the *T. californica* and the 12-Fish-CD-HIT proteomes showed the same trend in the number of hits against a given defined transporter class. By far, the highest number of hits against observed was against the ’Porters’ class, followed by hits against the *Alpha*-Type Channels, Auxiliary Transport Proteins and P-P Bond Hydrolysis Driven Transporters (Table 6).

**Table 6:** Distribution of Eukaryotic transporter proteins present in *T. californica* and combined fish Proteomes

Classes of transporters^a^	Combined fish Proteome^b^	*T. californica* Proteome^b^	Families specific To *T. californica*
Porters (uniporters, symporters, antiporters)	331	141	15
Alpha type channels	235	85	5
Auxillary transport proteins	114	70	7
P-P-Bond-Hydrolysis-Driven transporters	108	62	2
Recognized transporters of unknown mechanism	74	26	2
Putative transport proteins	63	43	9
Membrame-Bounded channels	59	41	0
Oxidoreduction-Driven transportes^c^	50	35	0
Vesicle fusion pores	20	14	0
Pore-Forming toxins	18	8	1
Transmembrane 1-electron transfer carriers	15	5	0
Paracellular channels	12	4	0
Acyl CoA ligase-coupled transporters	6	4	0
Beta-Barrel porins	6	6	0
Others^d^	9	6	1

^a^According to the Transporter Classification Database (http://tcdb.org)

^b^Number of RBH-Hits Identified by Reverse-Blast-Hit(RBH) at 60% Identity and 50% Coverage to TCDB

^c^An unusual over-representation of hits corresponding to the Bovine UniProt Annotations was observed for this category (i.e., 41 for Combined Fish and 28 for *T. californica*)

^d^Transporter Classes having two or less hits were grouped into this category. They include: Ribosomally synthesized protein/peptide toxins/agonists that target channels and carriers, Transcompartment Lipid Carrier, Cell Fusion Pores, Choline/EthanolaminePhosphotransferase 1, Polysaccharide Synthase/Exporters and Holins

We then asked if the *T. californica* MetaProteome detected transporter proteins present in the TCDB dataset, not detected by the 12-Fish proteome. We found 42 such hits (Table 6; Additional file 23). The majority of the hits were in the Porters (uniporters, symporters, antiporters) class, including proteins such as the *Excitatory Amino Acid Transporter 1* (*EAAT1*), which is important in the uptake of the excitatory neurotransmitter glutamate [39], and the *Chloride Channel protein 2* (*ClC-2*) [40], which modulates neuronal excitability. Five *Alpha*-Type Channels were unique to *T. californica*, including the *Voltage-dependent L-type calcium channel subunit 1* (*Cav1.2*) [41], and ligand gated channels, such as the *Glutamate* [*NMDA*], *receptor subunit epsilon-3* (*NR2C*) [42], and the *GABA(A) receptor subunit beta-1* [43]. Finally, among the representative transporters detected only by *T. californica*, we found two proteins that have been previously identified in the Marbled electric ray *T. marmorata - Dispanin*, which is a type of auxiliary transport protein, and the proton conducting portion of the *vacuolar-ATPase*, the *V-type proton ATPase* 16 kDa proteolipid subunit [44].

The *T. californica*-specific hits described here (i.e., signal) were found despite the use of a combined 12-Fish proteome (i.e., noise) composed of 12 largely evolutionarily diverse organisms ranging from bony vertebrates (Euteleostomi like Coelacanth (Latimeria), Spotted gar (Lepisosteus), Zebrafish (Danio), Blind Cave Fish (Astyanax), Atlantic Cod (Gadus), Southern Platyfish (Xiphophorus), Japanese Medaka (Oryzias), Nile Tilapia (Oreochromis), Fugu (Takifugu), Amazon molly (Poecilia), Three-spined Stickleback (Gasterosteus), and Spotted Green Pufferfish (Tetraodon). Many of these organisms (i.e., the Euteleostomi), arguably are closer to *H. sapiens* than *T. californica*. Also, note that of the 42 transporters identified, only 22 of them were present in the set of proteins shared by *C. milli*, *H. sapiens*, and *T. californica* and 12 were present in the set shared by both *H. sapiens* and *T. californica*, and absent in *C. milli*. This suggests that these transporters are specific to *T. californica* (an electric fish) and are not just present in the Chondrichthyes. Finding *Dispanin*, a *Torpedo* protein, validated both the assembly and protein prediction strategy employed. The RBH-Blast strategy used here is very specific, as it establishes a one-to-one relationship between two datasets. RBH-Blast establishes that the hits observed are mutual, both from the query and from the subject (or database) point of view. Importantly, these results do not argue against nor discard the existence of homologous proteins present in the 12-Fish proteome. These results do establish, however, that in a RBH-Blast between the *T. californica* and current components of the TCDB-database, the *T. californica* transporter proteins selected, showed higher performance both at the level of Identity and Coverage, than the proteins of the combined 12-Fish-Proteome set used in this work. As a result, we were able to identify transporter proteins not identified by the other fish proteomes. In summary, we find that the set of transporters preferentially identified by the *T. californica* MetaProteome is highly enriched in proteins that play key roles in important neurological processes. These results are consistent with electric fish having a set of transporters that have been ’optimized’ to respond and recover quickly from a massive cellular depolarization. These results are also consistent with the idea that the genomes of organisms like *H. sapiens* and *Mus musculus*, in addition to other transporters, also posses the set of such ’electrically-optimized’ transporters or derivatives of them.

## Conclusions

We have assembled a *de novo* transcriptome corresponding to the electric lobe of *T. californica*. We critically evaluated the quality of our assemblies using ‘Industry Standard’ methods. We found a high degree of variability between assemblies produced at different Kmers. Neither the number of transcripts assembled nor the value of the Detonate scores calculated gave us a definitive prediction for best assembly. In contrast, we observed that the only reliable parameter for assembly evaluation was related to the information content of the assembly in question, when compared to a standard database. We also generated a non-redundant set of transcripts by combining the transcriptome of the electric lobe with previous transcriptome of the electric organ, and determined those predicted proteins having high homology against the genomes of both *H. sapiens* and *C. milli*. Finally, we mapped and cross-annotated these highly-conserved predicted proteins against the well annotated Human biochemical and developmental pathways. We also identified transporter proteins present in the *T. californica* MetaProteome and in a MetaProteome set corresponding to the proteomes of 12-Fish genomes and identified a set of important transporters that were only detected by the *T. californica* MetaProteome. The combined information provides not only a unique tool for the study of cholinergic neurotransmission, but also a starting point for understanding the biology of early vertebrates, as well as, the biology of strongly electric fish, such as *T. californica*.

## Methods

### Poly(A)^+^ RNA purification, cDNA library preparation, and sequencing

The electric lobe from a female marine ray *Tetronarce californica* (Aquatic Research Consultants; San Pedro, CA) was dissected from the central nervous system, and total RNA was isolated from the frozen tissue as described [45]. RNA concentration was determined using a Nanodrop spectrophotometer and quality assessed by denaturing gel electrophoresis in formaldehyde gels and Northern analyses [45]. Poly(A)^+^RNA was purified using Poly(A)Purist MAG Kit (Ambion) and further cleaned up with Turbo DNase (Ambion) and Terminator-5’-phosphate-dependent exonuclease (Epicentre) to remove any trace amount of DNA and rRNA. cDNA was synthesized from purified poly(A)^+^mRNA using random hexamer oligonucleotide or oligo dT as primer. Synthesized cDNA was then sheared into small pieces of 100 to 800 bp in length using a Biorupter. The fragmented cDNA was prepared for Illumina sequencing using TruSeq Sample Prep Kit (Illumina). Paired-end sequencing (100 bp) of the cDNA libraries was performed on a HiSeq 2500 instrument (Illumina).

### *De novo* transcriptome assembly

The description of the software, version number and origin (when applicable) and representative commands and databases used are presented in Additional file 2.

#### Reads quality control (RQC)

Briefly, reads quality score was initially evaluated using FastQC. The first and last nucleotides of every read were trimmed using fastx_trimmer (FASTX-Toolkit). Similarly, sequencing artifacts were removed using fastx_artifacts_filter (FASTX-Toolkit). Sequencing adaptors were then removed using Cutadapt [46]. Reads were then trimmed with fastq_quality_trimmer (FASTX-Toolkit) to remove any nucleotide with a quality threshold lower than 20. Reads with a minimal length of 40 nucleotides (after trimming) were discarded. The read size distributions before and after RQC are shown in Additional file 2.

#### Transcriptome assembly

Assembly was performed using Trinity at four different Kmers (25, 27, 29, and 31). All assemblies were performed with the ’jaccard_clip’ flag on (see Additional file 2 for details).

#### Post-assembly processing

We used: CD-HIT [2, 9–13], Detonate [8], BUSCO [20], Blast+ [14, 15], and BlastRBH [14]. The commands used for each program and the software version are described in Additional file 2.

#### Assembly experimental strategy

The general Assembly experimental strategy used is presented in Fig. 2.

#### Other essential tools

We used GNU-Parallel for code parallelization [47] and Kent Tools [48, 49] for data processing, extensively during all steps of this work.

## Additional files

Additional file 1 Supplemental Figures: File containing Figure S01-to-S02. (PDF 349 kb)

Additional file 2 Summary Of The Software And Commands Used In This Work. (PDF 158 kb)

Additional file 3 Supplemental Tables: File containing Table S01-to-S07. (PDF 128 kb)

Additional file 4 *T. californica* MetaTranscriptome. Transcripts IDs and their origin. (XLSX 2806 kb)

Additional file 5 Cross-Annotation and GO Analysis of the SwissProt proteins that have a coverage of 70% or higher to *T. cali* MetaORFeome proteins. (XLSX 855 kb)

Additional file 6 Proteome IDs of Blast Results and Reverse Blast Hit Results. (XLSX 2887 kb)

Additional file 7 Cross-Annotated *T. californica* Transcripts Present in *C. milli*. (XLSX 2201 kb)

Additional file 8 Cross-Annotated *T. californica* Transcripts Present in *H. sapiens*. (XLSX 3276 kb)

Additional file 9 Cross-Annotated Proteins Present in *H. sapiens* and *C. milli*. (XLSX 3880 kb)

Additional file 10 Cross-Annotated *T. californica* Transcripts Present in *C. milli* Not Present in *H. sapiens*. (XLSX 269 kb)

Additional file 11 Cross-Annotated *T. californica* Transcripts Present in *H. sapiens* Not Present in *C. milli*. (XLSX 1075 kb)

Additional file 12 Cross-Annotated Proteins Present in *H. sapiens* and *C. milli* Not Present in *T. californica*. (XLSX 1976 kb)

Additional file 13 Cross-Annotated *T. californica* Transcripts Present in *H. sapiens* and *C. milli*. (XLSX 2211 kb)

Additional file 14 Cross-Annotated conserved SwissProt proteins shared between *H. sapiens*, *T. californica* and *C. milli* that have a coverage of 100% between *H. sapiens* and *C. Milli*. (XLSX 99.2 kb)

Additional file 15 Set10 – GO Biological Processes Complete. (XLSX 47 KB)

Additional file 16 Set08 – GO Biological Processes Complete. (XLSX 24.6 kb)

Additional file 17 Set09 – GO Biological Processes Complete. (XLSX 41.2 kb)

Additional file 18 KEGG Analysis. Intersection of *H. sapiens* With *T. californica* Without *C. milii* (set 8). See Additional file 3: TableS07 for details. (PDF 10649 kb)

Additional file 19 KEGG Analysis. Intersection of *H. sapiens* With *C. milli* Without *T. californica* (set 9). See Additional file 3: TableS07 for details. (PDF 11366 kb)

Additional file 20 KEGG Analysis. Intersection of *H. sapiens* With *T. californica* (set 12). See Additional file 3: TableS07 for details. (PDF 11366 kb)

Additional file 21 KEGG Analysis. Intersection of *H. sapiens* With *C. milli* (set 13). See Additional file 3: TableS07 for details. (PDF 11366 kb)

Additional file 22 Selected KEGG Figures corresponding to pathways presented in Table 5. (XLSX 1198 kb)

Additional file 23 Data Corresponding to the Eukaryotic Transporter Proteins Only Detected by *T. californica*. (XLSX 41.3 kb)

Additional file 24 Fasta File Containing Nucleotide Sequences Shorter Than 200 bp That Are Part Of The MetaProteome. (FA 791 kb)

Additional file 25 Fasta File Containing Predicted Peptide Sequences From The Nucleotide Sequences Shorter Than 200 bp That Are Part Of The MetaProteome. (FA 889 kb)
