# Supplementary figures and images for: Draft de novo transcriptome assembly and proteome characterization of the electric lobe of Tetronarce californica: a molecular tool for the study of cholinergic neurotransmission in the electric organ
Source: BMC Genomics. 2017 Aug 14;18:611. doi: 10.1186/s12864-017-3890-4 (PMC5557070; doi:10.1186/s12864-017-3890-4)

Figure S01

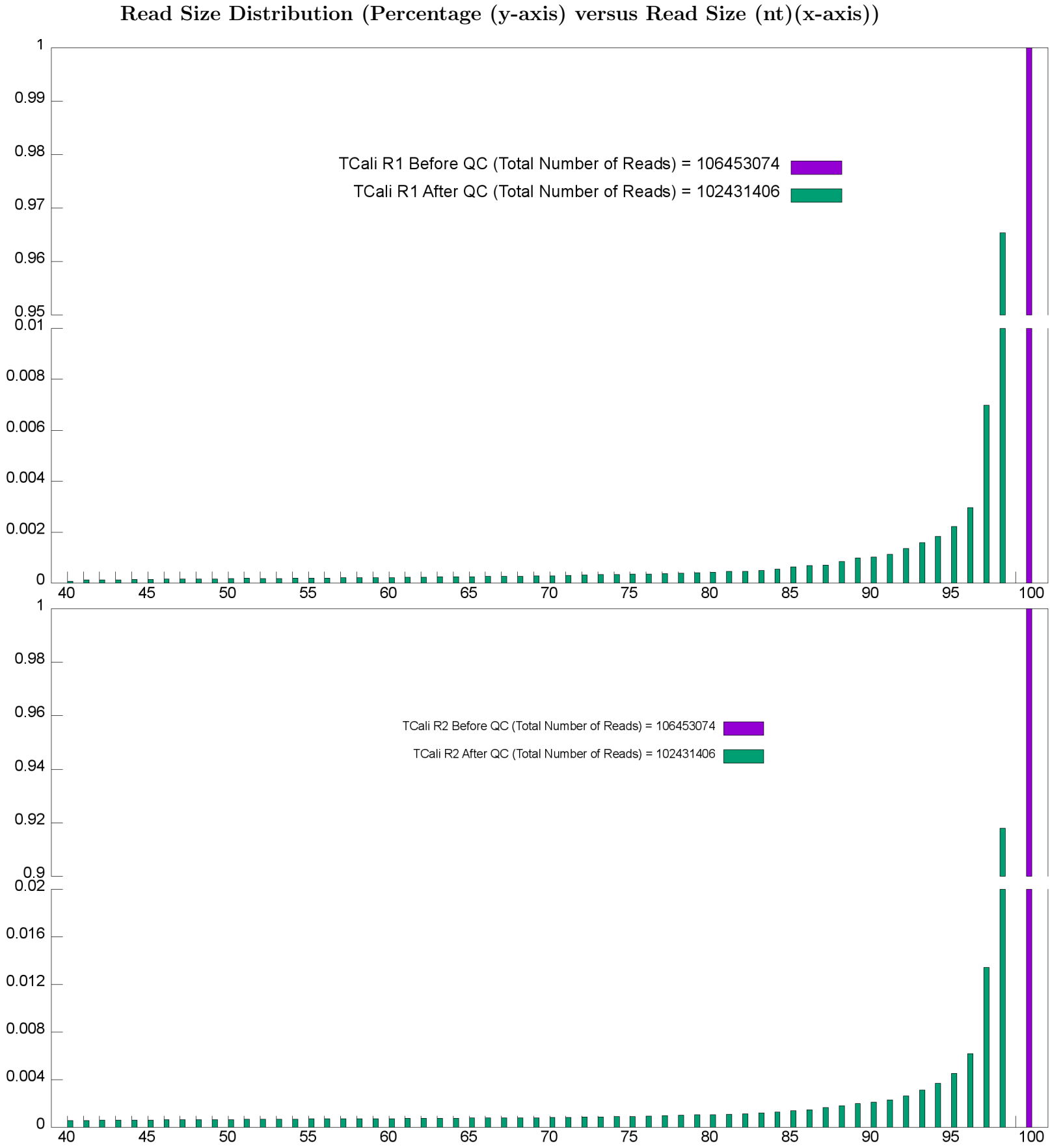

Figure S02: Full Length Transcript Analysis Against Uniprot\_Sprot

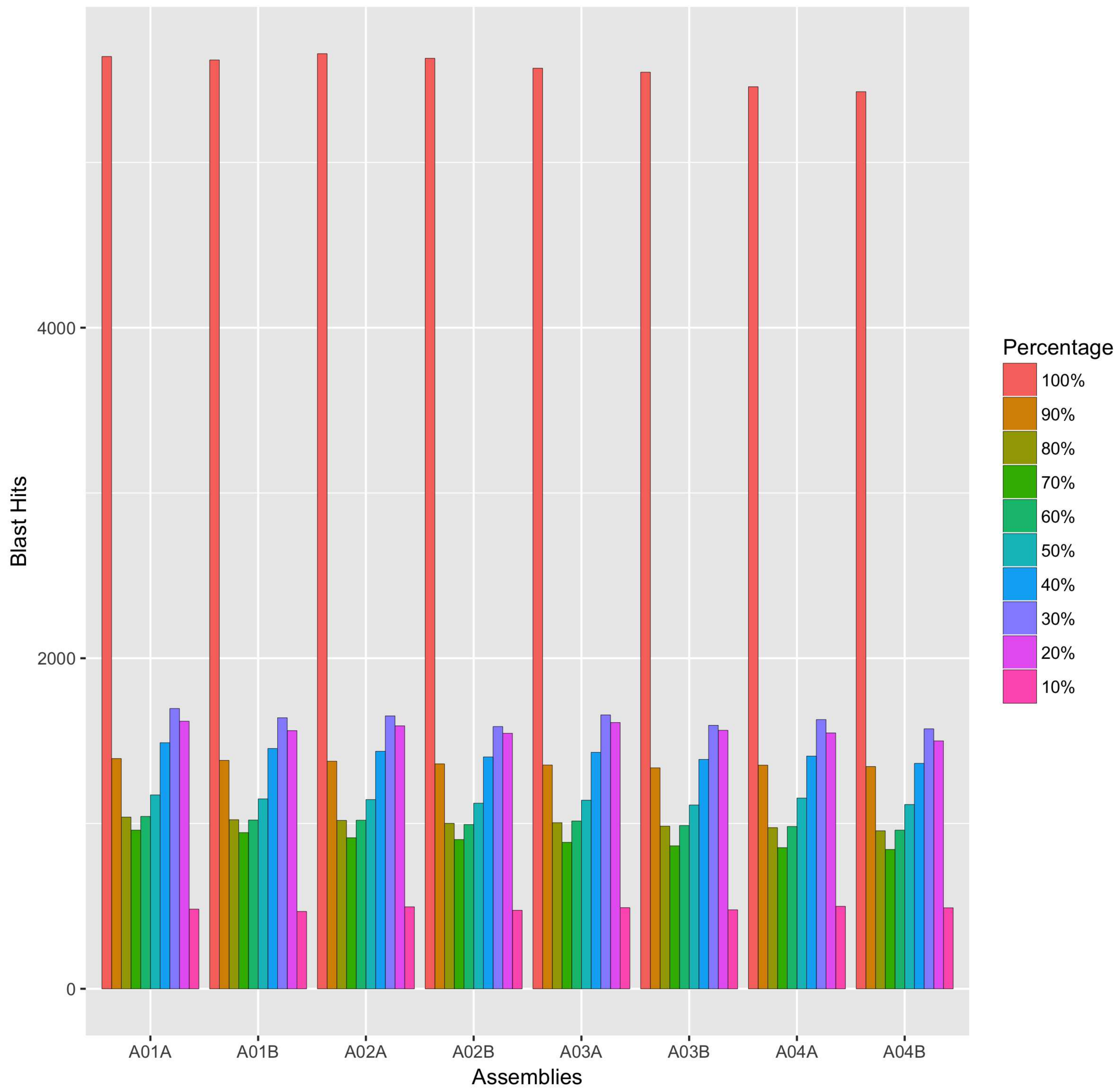

Supplement: Supplementary file 1 — Supplemental Figures: File containing Figure S01-to-S02. (PDF 349 kb) [file 12864_2017_3890_MOESM1_ESM.pdf]
