# Supplementary material for: Draft de novo transcriptome assembly and proteome characterization of the electric lobe of Tetronarce californica: a molecular tool for the study of cholinergic neurotransmission in the electric organ
Source: BMC Genomics. 2017 Aug 14;18:611. doi: 10.1186/s12864-017-3890-4 (PMC5557070; doi:10.1186/s12864-017-3890-4)
Supplement: Supplementary file 2 — Summary Of The Software And Commands Used In This Work. (PDF 158 kb) [file 12864_2017_3890_MOESM2_ESM.pdf]

Contents

1 Additional File 02 **2**

1.1 **FASTQC** 2
1.2 **FASTX-TOOLKIT** 3
1.3 **CUTADAPT** 4
1.4 **TRINITY** 5
1.5 **CD-HIT** 7
1.6 **DETONATE** 9
1.7 **BUSCO** 10
1.8 **TRANSDECODER** 11
1.9 **BLAST** 12
1.10 **HMMER** 13
1.11 **BLAST-RBH** 14
1.12 **DATABASES-USED** 15

1 Additional File 02

1.1 FASTQC

- `http://www.bioinformatics.bbsrc.ac.uk/projects/fastqc/`
- Version: v0.11.2
- Command:

fastqc \
InputFile \
&» \
OutputFile

1.2 FASTX-TOOLKIT

- `http://hannonlab.cshl.edu/fastx-toolkit/index.html`
- Version: v0.0.13
- Command-01:

```
fastx-trimmer \
-t 1 \
-Q 33 \
-i \
InputFile \
-o \
OutputFile \
1 » \
LogFile;
```

- Command-02:

```
fastx-trimmer \
-f 2 \
-Q 33 \
-i \
InputFile \
-o \
OutputFile \
1 » \
LogFile;
```

- Command-03:

```
fastx-artifacts-filter \
-v \
-Q 33 \
-i \
InputFile \
-o \
OutputFile \
1» \
LogFile;
```

- Command-04:

```
fastq-quality-trimmer \
-v \
-t 20 \
-l 40 \
-Q 33 \
-i \
InputFile \
-o \
OutputFile \
1» \
LogFile;
```

1.3 CUTADAPT

- `https://cutadapt.readthedocs.io/en/stable/`
- Version: v1.7.1
- Command:

```
cutadapt \
-O 9 \
-b 5'-AdaptorSequence-3' \
InputFile \
1> \
OutputFile \
2» \
LogFile;
```

1.4 TRINITY

- https://github.com/trinityrnaseq/trinityrnaseq/wiki
- Version: v2.1.1
- Command-01:

```
Trinity \
–seqType fq \
–max-memory 200G \
–left InputReadsFile.R1.fastq \
–right InputReadsFileR2.fastq \
–SS-lib-type RF \
–CPU 1 \
–min-contig-length 400 \
–full-cleanup \
–KMER-SIZE x; where x=25,27,29 or 31 \
–jaccard-clip \
–output OutputFile.TinityVersion.KMerSize.Jaccard
```

- Command-02:

```
bowtie-PE-separate-then-join.pl \
–seqType fq \
–left InputReadsFile.R1.fastq \
–right InputReadsFile.R2.fastq \
–target InputAssemblyFile \
–aligner bowtie \
– \
-p 1 \
-t \
-a \
–best \
–strata \
&> zlog &;
```

- Command-03:

```
SAM-nameSorted-to-uniq-count-stats.pl \
bowtie-out/bowtie-out.nameSorted.bam \
> bowtie-out.results \
&> zlog &
```

- Command-04:

```
analyze-blastPlus-topHit-coverage.pl \
OutputName.outfmt6 \
InputFile \
Database (uniprot-sprot) \
&> zlog &
```

- Command-05:

```
blast-outfmt6-group-segments.pl \
InputAssemblyFile.vs.uniprot-sprot.blastx.outfmt6 \
InputAssemblyFile \
Database (uniprot-sprot) \
> \
InputAssemblyFile.vs.uniprot-sprot.blastx.outfmt6.grouped \
&> zlog &
```

1.5 CD-HIT

- `http://weizhongli-lab.org/cd-hit/`
- Version: v4.6
- Command-01:

```
cd-hit \
-i \
InputFile \
-o \
OutputFile \
-c 1.00 \
-n 5 \
-p 1 \
-g 1 \
-T 1 \
-d 40 \
-M 0 \
&> \
LogFile
```

- Command-02:

```
cd-hit-est \
-i \
InputFile \
-o \
OutputFile \
-c 1.00 \
-n 8 \
-r 1 \
-p 1 \
-g 1 \
-T 1 \
-d 40 \
-M 0 \
&> \
LogFile
```

- Command-03:

```
cd-hit-2d \
-i InputFile01 \
-i2 InputFile02 \
-o OutputFile \
-c 1.0 \
-n 5 \
-d 0 \
-M 0 \
-T 1 \
&> LogFile;
```

1.6 DETONATE

- http://deweylab.biostat.wisc.edu/detonate/
- Version: v1.10
- Command-01:

```
rsem-eval-calculate-score \
-p 1 \
–time \
–paired-end \
InputReadsFile.R1.fastq \
InputReadsFile.R2.fastq \
InputFile (i.e.,OutputFile.TinityVersion.KMerSize.Jaccard) \
OutputFilesPrefix \
FragmentLength;
```

1.7 BUSCO

- `http://busco.ezlab.org/`
- Version: v1.1b1
- Command-01:

```
BUSCO-v1.1b1.py \
-in InputFile \
–mode trans \
–species human \
–cpu 1 \
–lineage eukaryota \
-o OutputFile \
-f \
&> LogFile;
```

- Command-02:

```
BUSCO-v1.1b1.py \
-in InputFile \
–mode trans \
–species human \
–cpu 1 \
–lineage metazoa \
-o OutputFile \
-f \
&> LogFile;
```

- Command-03:

```
BUSCO-v1.1b1.py \
-in InputFile \
–mode trans \
–species human \
–cpu 1 \
–lineage vertebrata \
-o OutputFile \
-f \
&> LogFile;
```

1.8 TRANSDECODER

- http://transdecoder.github.io/
- Version:
- Command-01:

```
TransDecoder.LongOrfs \
-t \
-m 60 \
InputFile \
&> LogFile;
```

- Command-02:

```
TransDecoder.Predict \
-t InputAssemblyFile.fa \
–retain-pfam-hits InputPFAMSearchFile.domtblout \
–retain-blastp-hits InputBlastpSearchFile.outfmt6 \
–retain-long-orfs 900 \
–single-best-orf \
–cpu 1 \
&> LogFile;
```

1.9 BLAST

- ftp://ftp.ncbi.nlm.nih.gov/blast/executables/blast+/2.2.31/
- Version: v2.2.31+
- Command-01:

```
makeblastdb \
-in uniprot-sprot.fasta \
-dbtype prot \
```

- Command-02:

```
blastx \
-query InputFile \
-db uniprot-sprot.fa \
-out OutputFile.outfmt6
-evalue 1e-20 \
-num-threads 1 \
-max-target-seqs 1 \
-outfmt 6 \
```

- Command-03:

```
blastp \
-query InputFile.pep \
-db DataBaseFile (uniprot-sprot) \
-out OutputFile.DataBaseFile.vs.uniprot-sprot.blastp.outfmt6 \
-max-target-seqs 1 \
-outfmt 6 \
-evalue 1e-5 \
-num-threads 1 \
&> OutputFile.DataBaseFile.vs.uniprot-sprot.blastp.outfmt6.log &
```

- Command-04:

```
rpsblast \
-query InputFile.pep \
-db DataBaseFile (Cdd) \
-out OutputFile.DataBaseFile.vs.Cdd.rpsblast.outfmt6 \
-max-target-seqs 1 \
-outfmt 6 \
-evalue 1e-5 \
-num-threads 1 \
&> OutputFile.DataBaseFile.vs.Cdd.rpsblast.outfmt6.log &
```

- Command-05:

```
blast-formatter \
-archive InputFile.outfmt11 \
-outfmt OutFormat \
-max-target-seqs 1 \
-out OutputFile.outfmt6 \
&> OutputFile.outfmt6.log &
```

1.10 HMMER

- `http://hmmer.org/`
- Version: 3.1b1
- Command-01:

```
hmmscan \
–cpu 1 \
–domtblout \
OutputFile.pep.pfam.domtblout \
Pfam-A.hmm \
InputFile.pep \
&> OutputFile.pep.pfam.domtblout.log &
```

1.11 BLAST-RBH

- `https://github.com/peterjc/galaxy-blast/tree/master/tools/blast-rbh`
- Version: v0.1.8
- Command-01:

```
python blast-rbh.py \
-a prot \
-t blastp \
–nr \
-i 100.0 \
-c 50.0 \
./InputFile01.pep \
./InputFile02.pep \
-o OutputFile
```

1.12 DATABASES-USED

	Database	Origin	NumerOfRecords	Downloaded
01	*Homo sapiens*	Uniprot[1]	151569	2016-03-01
02	*Callorhinchus milii*	GenBank[2]	28237	2016-07-01
03	PFAM	NCBI[3]		2016-06-27
04	CDD	NCBI[4]		2016-06-27
05	UniProtSProt	Uniprot[5]	551385	2016-06-27
06	KEGG Mapper	KEGG[6]		
07	Panther	Panther[7]		
08	TCDB	TCDB[8]	14961	2017-03-01

1. http://www.uniprot.org/
2. http://www.ncbi.nlm.nih.gov/genome/?term=Callorhinchus+milii
3. ftp://ftp.ncbi.nih.gov/pub/mmdb/cdd/little_endian/
4. ftp://ftp.ncbi.nih.gov/pub/mmdb/cdd/little_endian/
5. ftp://ftp.uniprot.org/pub/databases/uniprot/current_release/knowledgebase/complete/uniprot_sprot.fasta.gz
6. http://www.kegg.jp/kegg/tool/map_pathway2.html
7. http://pantherdb.org/
8. http://tcdb.org/
