# Supplementary material for: Draft de novo transcriptome assembly and proteome characterization of the electric lobe of Tetronarce californica: a molecular tool for the study of cholinergic neurotransmission in the electric organ
Source: BMC Genomics. 2017 Aug 14;18:611. doi: 10.1186/s12864-017-3890-4 (PMC5557070; doi:10.1186/s12864-017-3890-4)
Supplement: Supplementary file 3 — Supplemental Tables: File containing Table S01-to-S07. (PDF 128 kb) [file 12864_2017_3890_MOESM3_ESM.pdf]

Table S01

Detonate RSEM-EVAL Evaluation Results

	Assembly (Kmer)	Read Representation in Assembly (%)[1]	Score[2]	Contigs with no reads aligned to[2]	Number of alignable reads[2]
Trinity	01 (25)	87.4	-6105288705.62	9018	89540171
	02 (27)	87.6	-6053954533.82	8812	89767591
	03 (29)	87.9	-5967748885.21	8059	90125791
	04 (31)	88.2	-5932951277.04	7620	90413054
Trinity + CD-HIT-EST	01 (25)	87.4	-6115264651.77	7295	89529692
	02 (27)	87.6	-6110650415.91	7118	89744478
	03 (29)	87.9	-5976932627.27	6563	90117377
	04 (31)	88.2	-5939550282.37	6073	90404499

[1]Calculated as a fraction of the total paired reads that entered assembly (i.e., 102,431,406 reads)
[2]As determined by Detonate RSEM-EVAL

Table S02

Full Length Transcript Analysis Against `Uniprot_Sprot`

	**Assembly (Kmer)**	**Hit Coverage Percentage**:	**100**	**90**	**80**	**70**	**60**	**50**	**40**	**30**	**20**	**10**	**Total**
Trinity	01 (25)	Number of Hits[1]:	5641	1393	1039	960	1043	1173	1489	1696	1619	482	16535
	02 (27)		5658	1377	1019	914	1020	1145	1437	1651	1591	496	16308
	03 (29)		5570	1354	1005	886	1015	1141	1431	1657	1611	491	16161
	04 (31)		5458	1353	975	854	982	1154	1408	1629	1548	499	15860
		Average:	5582	1369	1010	904	1015	1153	1441	1658	1592	492	16216
Trinity + CD-HIT-EST	01 (25)		5620	1382	1023	945	1021	1149	1454	1640	1562	468	16264
	02 (27)		5630	1361	1001	903	994	1123	1403	1587	1546	475	16023
	03 (29)		5546	1337	984	865	988	1112	1388	1594	1564	478	15856
	04 (31)		5428	1345	956	843	960	1115	1364	1573	1500	490	15574
		Average:	5556	1356	991	889	991	1125	1402	1598	1543	478	15929

[1]Only the single best matching Trinity transcript is reported for each top matching database entry at that given identity percentage

Table S03

BUSCO Analysis of the Trinity + CD-HIT-EST Assemblies

Assemblies	BUSCOs[1]								
	Complete	Fragmented	Missing	Complete	Fragmented	Missing	Complete	Fragmented	Missing
Assembly (Kmer)	Eukaryota (429)			Metazoa (843)			Vertebrata (3023)		
01 (25)	372	21	36	740	73	30	1937	219	867
02 (27)	376	19	34	741	73	29	1921	217	885
03 (29)	374	21	34	740	71	32	1896	218	909
04 (31)	370	23	36	732	76	35	1855	226	942
Average:	373	21	35	738	73	32	1902	220	901

[1]Calculated using the 'trans' mode

Table S04

Tetronarce californica Proteome

		BUSCOs[1]								
Datasets	Proteins	Complete	Fragmented	Missing	Complete	Fragmented	Missing	Complete	Fragmented	Missing
		Eukaryota (429)			Metazoa (843)			Vertebrata (3023)		
Electric Lobe[3]	70338	395	23	11	744	71	28	1991	217	815
Electric Organ[4]	3857	62	60	307	65	75	703	125	67	2831
MetaAssembly[3]	74195	396	24	9	746	71	26	1995	221	807

[1]Calculated using the 'OGS' mode. [2]Unique Transcripts. [3]This work. [4]Nazarian *et al.*

Table S05

Full Length *T. californica* Proteome Analysis Against `Unirot_Sprot`

	Hit Coverage Percentage:	100	90	80	70	60	50	40	30	20	10	Total
Proteome	Number of Hits[1]:	6438	1260	1003	904	1037	1205	1515	1820	1763	670	17615

[1]Only the single best matching Trinity transcript is reported for each top matching database entry at that given identity percentage

Table S06

Fish Genomes Used In This Study

Common Name	Scientific Name	Number of Proteins	Taxonomy ID	Database Name	Ensembl Link
Amazon molly	*Poecilia formosa*	29970	48698	Poecilia-formosa-5.1.2	`http://www.ensembl.org/Poecilia_formosa/Info/Index`
Cave fish	*Astyanax mexicanus*	23607	7994	AstMex102	`http://www.ensembl.org/Astyanax_mexicanus/Info/Index`
Cod	*Gadus morhua*	22014	8049	gadMor1	`http://www.ensembl.org/Gadus_morhua/Info/Index`
Coelacanth	*Latimeria chalumnae*	23213	7897	LatCha1	`http://www.ensembl.org/Latimeria_chalumnae/Info/Index`
Fugu	*Takifugu rubripes*	47575	31033	FUGU 4.0	`http://www.ensembl.org/Takifugu_rubripes/Info/Index`
Medaka	*Oryzias latipes*	24433	8090	HdrR	`http://www.ensembl.org/Oryzias_latipes/Info/Index`
Platyfish	*Xiphophorus maculatus*	20443	8083	Xipmac4.4.2	`http://www.ensembl.org/Xiphophorus_maculatus/Info/Index`
Spotted gar	*Lepisosteus oculatus*	22287	7918	LepOcu1	`http://www.ensembl.org/Lepisosteus_oculatus/Info/Index`
Stickleback	*Gasterosteus aculeatus*	26621	69293	BROAD S1	`http://www.ensembl.org/Gasterosteus_aculeatus/Info/Index`
Tetraodon	*Tetraodon nigroviridis*	22938	99883	TETRAODON 8.0	`http://www.ensembl.org/Tetraodon_nigroviridis/Info/Index`
Tilapia	*Oreochromis niloticus*	26590	8128	Orenil1.0	`http://www.ensembl.org/Oreochromis_niloticus/Info/Index`
Zebrafish	*Danio rerio*	38096	7955	GRCz10	`http://www.ensembl.org/Danio_rerio/Info/Index`

Table S07

Venn Diagram Set Naming Convention

Set	Set Content Definition	No Proteins	Additional File
01	*Tetronarce californica*	74195	AF04
02	*Homo Sapiens*	104631	AF06A
03	*Callorhinchus milii*	23480	AF06B
04	*Tetronarce californica* ∩ ¬ *Homo Sapiens* ∩ ¬ *Callorhinchus milii*	65174	–
05	¬ *Tetronarce californica* ∩ *Homo Sapiens* ∩ ¬ *Callorhinchus milii*	95501	–
06	¬ *Tetronarce californica* ∩ ¬ *Homo Sapiens* ∩ *Callorhinchus milii*	12653	–
07	*Tetronarce californica* ∩ ¬ *Homo Sapiens* ∩ *Callorhinchus milii*	3618	AF10
08	*Tetronarce californica* ∩ *Homo Sapiens* ∩ ¬ *Callorhinchus milii*	1921	AF11
09	¬ *Tetronarce californica* ∩ *Homo Sapiens* ∩ *Callorhinchus milii*	3727	AF12
10	*Tetronarce californica* ∩ *Homo Sapiens* ∩ *Callorhinchus milii*	3482	AF13
11	*Tetronarce californica* ∩ *Callorhinchus milii*	7100	AF07
12	*Tetronarce californica* ∩ *Homo Sapiens*	5403	AF08
13	*Homo Sapiens* ∩ Callorhinchus milii	7209	AF09
