# Supplementary material for: Draft de novo transcriptome assembly and proteome characterization of the electric lobe of Tetronarce californica: a molecular tool for the study of cholinergic neurotransmission in the electric organ
Source: BMC Genomics. 2017 Aug 14;18:611. doi: 10.1186/s12864-017-3890-4 (PMC5557070; doi:10.1186/s12864-017-3890-4)
Supplement: Supplementary file 18 — KEGG Analysis. Intersection of H. sapiens With T. californica Without C. milii (set 8). See Additional file 3: TableS07 for details. (PDF 10649 kb) [file 12864_2017_3890_MOESM18_ESM.pdf]

Intersection of *H. sapiens* With *T. californica* Without *C. milii*

Set08: Reverse-Blast-Hit (RBH)-Blast at 60% Identity + 50% Coverage

Rodolfo Aramayo

April 30, 2017

Contents

1 Endocytosis **36**
- 1.1 Human Pathway: HSA04144 36
- 1.2 Number of Hits: 31 36
- 1.3 Legend: 36

2 Pathways in cancer **37**
- 2.1 Human Pathway: HSA05200 37
- 2.2 Number of Hits: 30 37
- 2.3 Legend: 37

3 PI3K-Akt signaling pathway **38**
- 3.1 Human Pathway: HSA04151 38
- 3.2 Number of Hits: 29 38
- 3.3 Legend: 38

4 Focal adhesion **39**
- 4.1 Human Pathway: HSA04510 39
- 4.2 Number of Hits: 26 39
- 4.3 Legend: 39

5 HTLV-I infection **40**
- 5.1 Human Pathway: HSA05166 40
- 5.2 Number of Hits: 23 40
- 5.3 Legend: 41

6 MAPK signaling pathway **41**
6.1 Human Pathway: HSA04010 41
6.2 Number of Hits: 23 41
6.3 Legend: 41

7 Ras signaling pathway **42**
7.1 Human Pathway: HSA04014 42
7.2 Number of Hits: 23 42
7.3 Legend: 42

8 Alzheimer's disease **43**
8.1 Human Pathway: HSA05010 43
8.2 Number of Hits: 22 43
8.3 Legend: 43

9 RNA transport **44**
9.1 Human Pathway: HSA03013 44
9.2 Number of Hits: 21 44
9.3 Legend: 44

10 Non-alcoholic fatty liver disease (NAFLD) **45**
10.1 Human Pathway: HSA04932 45
10.2 Number of Hits: 20 45
10.3 Legend: 45

11 Axon guidance **46**
11.1 Human Pathway: HSA04360 46
11.2 Number of Hits: 20 46
11.3 Legend: 46

12 Oxytocin signaling pathway **47**
12.1 Human Pathway: HSA04921 47
12.2 Number of Hits: 20 47
12.3 Legend: 47

13 Regulation of actin cytoskeleton **48**
13.1 Human Pathway: HSA04810 48
13.2 Number of Hits: 20 48
13.3 Legend: 48

14 Huntington's disease **49**
14.1 Human Pathway: HSA05016 49
14.2 Number of Hits: 20 49
14.3 Legend: 49

15 Insulin resistance **50**
15.1 Human Pathway: HSA04931 50
15.2 Number of Hits: 19 50
15.3 Legend: 50

16 cAMP signaling pathway **51**
16.1 Human Pathway: HSA04024 51
16.2 Number of Hits: 19 51
16.3 Legend: 51

17 Ubiquitin mediated proteolysis **52**
17.1 Human Pathway: HSA04120 52
17.2 Number of Hits: 18 52
17.3 Legend: 52

18 Insulin signaling pathway **53**
18.1 Human Pathway: HSA04910 53
18.2 Number of Hits: 18 53
18.3 Legend: 53

19 Ribosome **54**
19.1 Human Pathway: HSA03010 54
19.2 Number of Hits: 18 54
19.3 Legend: 54

20 Chemokine signaling pathway **55**
20.1 Human Pathway: HSA04062 55
20.2 Number of Hits: 18 55
20.3 Legend: 55

21 AMPK signaling pathway **56**
21.1 Human Pathway: HSA04152 56
21.2 Number of Hits: 18 56
21.3 Legend: 56

22 cGMP-PKG signaling pathway **57**
22.1 Human Pathway: HSA04022 57
22.2 Number of Hits: 18 57
22.3 Legend: 57

23 Spliceosome **58**
23.1 Human Pathway: HSA03040 58
23.2 Number of Hits: 18 58
23.3 Legend: 58

24 Proteoglycans in cancer **59**
24.1 Human Pathway: HSA05205 59
24.2 Number of Hits: 18 59
24.3 Legend: 59

25 Influenza A **60**
25.1 Human Pathway: HSA05164 60
25.2 Number of Hits: 17 60
25.3 Legend: 60

26 Hippo signaling pathway **61**
26.1 Human Pathway: HSA04390 61
26.2 Number of Hits: 17 61
26.3 Legend: 61

27 Neuroactive ligand-receptor interaction **62**
27.1 Human Pathway: HSA04080 62
27.2 Number of Hits: 17 62
27.3 Legend: 62

28 Adrenergic signaling in cardiomyocytes **63**
28.1 Human Pathway: HSA04261 63
28.2 Number of Hits: 16 63
28.3 Legend: 63

29 Wnt signaling pathway **64**
29.1 Human Pathway: HSA04310 64
29.2 Number of Hits: 16 64
29.3 Legend: 64

30 Purine metabolism **65**
30.1 Human Pathway: HSA00230 65
30.2 Number of Hits: 16 65
30.3 Legend: 65

31 Neurotrophin signaling pathway **66**
31.1 Human Pathway: HSA04722 66
31.2 Number of Hits: 16 66
31.3 Legend: 66

32 Rap1 signaling pathway **67**
32.1 Human Pathway: HSA04015 67
32.2 Number of Hits: 16 67
32.3 Legend: 67

33 Dopaminergic synapse **68**
33.1 Human Pathway: HSA04728 68
33.2 Number of Hits: 16 68
33.3 Legend: 68

34 Longevity regulating pathway **69**
34.1 Human Pathway: HSA04211 69
34.2 Number of Hits: 15 69
34.3 Legend: 69

35 Parkinson's disease **70**
35.1 Human Pathway: HSA05012 70
35.2 Number of Hits: 15 70
35.3 Legend: 70

36 mRNA surveillance pathway **71**
36.1 Human Pathway: HSA03015 71
36.2 Number of Hits: 15 71
36.3 Legend: 71

37 Cholinergic synapse **72**
37.1 Human Pathway: HSA04725 72
37.2 Number of Hits: 15 72
37.3 Legend: 72

38 Glutamatergic synapse **73**
38.1 Human Pathway: HSA04724 73
38.2 Number of Hits: 15 73
38.3 Legend: 73

39 Oxidative phosphorylation **74**
39.1 Human Pathway: HSA00190 74
39.2 Number of Hits: 14 74
39.3 Legend: 74

40 FoxO signaling pathway **75**
40.1 Human Pathway: HSA04068 75
40.2 Number of Hits: 14 75
40.3 Legend: 75

41 Thyroid hormone signaling pathway **76**
41.1 Human Pathway: HSA04919 76
41.2 Number of Hits: 14 76
41.3 Legend: 76

42 Morphine addiction **77**
42.1 Human Pathway: HSA05032 77
42.2 Number of Hits: 14 77
42.3 Legend: 77

43 GABAergic synapse **78**
43.1 Human Pathway: HSA04727 78
43.2 Number of Hits: 14 78
43.3 Legend: 78

44 Apoptosis **79**
44.1 Human Pathway: HSA04210 79
44.2 Number of Hits: 14 79
44.3 Legend: 79

45 Epstein-Barr virus infection **80**
45.1 Human Pathway: HSA05169 80
45.2 Number of Hits: 14 80
45.3 Legend: 80

46 Renal cell carcinoma **81**
46.1 Human Pathway: HSA05211 81
46.2 Number of Hits: 14 81
46.3 Legend: 81

47 Cell cycle **82**
47.1 Human Pathway: HSA04110 82
47.2 Number of Hits: 14 82
47.3 Legend: 82

48 Circadian entrainment **83**
48.1 Human Pathway: HSA04713 83
48.2 Number of Hits: 14 83
48.3 Legend: 83

49 Bacterial invasion of epithelial cells **84**
49.1 Human Pathway: HSA05100 84
49.2 Number of Hits: 13 84
49.3 Legend: 84

50 Calcium signaling pathway **85**
50.1 Human Pathway: HSA04020 85
50.2 Number of Hits: 13 85
50.3 Legend: 85

51 Alcoholism **86**
51.1 Human Pathway: HSA05034 86
51.2 Number of Hits: 13 86
51.3 Legend: 86

52 Viral carcinogenesis **87**
52.1 Human Pathway: HSA05203 87
52.2 Number of Hits: 13 87
52.3 Legend: 87

53 Phagosome **88**
53.1 Human Pathway: HSA04145 88
53.2 Number of Hits: 12 88
53.3 Legend: 88

54 Tight junction **89**
54.1 Human Pathway: HSA04530 89
54.2 Number of Hits: 12 89
54.3 Legend: 89

55 Phospholipase D signaling pathway **90**
55.1 Human Pathway: HSA04072 90
55.2 Number of Hits: 12 90
55.3 Legend: 90

56 mTOR signaling pathway **91**
56.1 Human Pathway: HSA04150 91
56.2 Number of Hits: 12 91
56.3 Legend: 91

57 Valine, leucine and isoleucine degradation **92**
57.1 Human Pathway: HSA00280 92
57.2 Number of Hits: 12 92
57.3 Legend: 93

58 Fc gamma R-mediated phagocytosis **93**
58.1 Human Pathway: HSA04666 93
58.2 Number of Hits: 12 93
58.3 Legend: 93

59 Protein processing in endoplasmic reticulum **94**
59.1 Human Pathway: HSA04141 94
59.2 Number of Hits: 12 94
59.3 Legend: 94

60 Shigellosis **95**
60.1 Human Pathway: HSA05131 95
60.2 Number of Hits: 11 95
60.3 Legend: 95

61 Sphingolipid signaling pathway **96**
61.1 Human Pathway: HSA04071 96
61.2 Number of Hits: 11 96
61.3 Legend: 96

62 Herpes simplex infection **97**
62.1 Human Pathway: HSA05168 97
62.2 Number of Hits: 11 97
62.3 Legend: 97

63 Pyrimidine metabolism **98**
63.1 Human Pathway: HSA00240 98
63.2 Number of Hits: 11 98
63.3 Legend: 98

64 Hepatitis B **99**
64.1 Human Pathway: HSA05161 99
64.2 Number of Hits: 11 99
64.3 Legend: 99

65 Oocyte meiosis **100**
65.1 Human Pathway: HSA04114 100
65.2 Number of Hits: 11 100
65.3 Legend: 100

66 Glucagon signaling pathway **101**
66.1 Human Pathway: HSA04922 101
66.2 Number of Hits: 11 101
66.3 Legend: 101

67 Salmonella infection **102**
67.1 Human Pathway: HSA05132 102
67.2 Number of Hits: 11 102
67.3 Legend: 102

68 Retrograde endocannabinoid signaling **103**
68.1 Human Pathway: HSA04723 103
68.2 Number of Hits: 11 103
68.3 Legend: 103

69 Cell adhesion molecules (CAMs) **104**
69.1 Human Pathway: HSA04514 104
69.2 Number of Hits: 10 104
69.3 Legend: 104

70 T cell receptor signaling pathway **105**
70.1 Human Pathway: HSA04660 105
70.2 Number of Hits: 10 105
70.3 Legend: 105

71 B cell receptor signaling pathway **106**
71.1 Human Pathway: HSA04662 106
71.2 Number of Hits: 10 106
71.3 Legend: 106

72 Adipocytokine signaling pathway **107**
72.1 Human Pathway: HSA04920 107
72.2 Number of Hits: 10 107
72.3 Legend: 107

73 Signaling pathways regulating pluripotency of stem cells **108**
73.1 Human Pathway: HSA04550 108
73.2 Number of Hits: 10 108
73.3 Legend: 108

74 HIF-1 signaling pathway **109**
74.1 Human Pathway: HSA04066 109
74.2 Number of Hits: 10 109
74.3 Legend: 109

75 Toxoplasmosis **110**
75.1 Human Pathway: HSA05145 110
75.2 Number of Hits: 10 110
75.3 Legend: 110

76 Carbon metabolism **111**
76.1 Human Pathway: HSA01200 111
76.2 Number of Hits: 10 111
76.3 Legend: 111

77 Serotonergic synapse **112**
77.1 Human Pathway: HSA04726 112
77.2 Number of Hits: 10 112
77.3 Legend: 112

78 Longevity regulating pathway - multiple species **113**
78.1 Human Pathway: HSA04213 113
78.2 Number of Hits: 10 113
78.3 Legend: 113

79 ErbB signaling pathway **114**
79.1 Human Pathway: HSA04012 114
79.2 Number of Hits: 10 114
79.3 Legend: 114

80 Choline metabolism in cancer **115**
80.1 Human Pathway: HSA05231 115
80.2 Number of Hits: 10 115
80.3 Legend: 115

81 Lysosome **116**
81.1 Human Pathway: HSA04142 116
81.2 Number of Hits: 10 116
81.3 Legend: 116

82 Platelet activation **117**
82.1 Human Pathway: HSA04611 117
82.2 Number of Hits: 10 117
82.3 Legend: 117

83 ECM-receptor interaction **118**
83.1 Human Pathway: HSA04512 118
83.2 Number of Hits: 9 118
83.3 Legend: 118

84 Pancreatic secretion **119**
84.1 Human Pathway: HSA04972 119
84.2 Number of Hits: 9 119
84.3 Legend: 119

85 Leukocyte transendothelial migration **120**
85.1 Human Pathway: HSA04670 120
85.2 Number of Hits: 9 120
85.3 Legend: 120

86 Osteoclast differentiation **121**
86.1 Human Pathway: HSA04380 121
86.2 Number of Hits: 9 121
86.3 Legend: 121

87 Pancreatic cancer **122**
87.1 Human Pathway: HSA05212 122
87.2 Number of Hits: 9 122
87.3 Legend: 122

88 TNF signaling pathway **123**
88.1 Human Pathway: HSA04668 123
88.2 Number of Hits: 9 123
88.3 Legend: 123

89 Small cell lung cancer **124**
89.1 Human Pathway: HSA05222 124
89.2 Number of Hits: 9 124
89.3 Legend: 124

90 Fc epsilon RI signaling pathway **125**
90.1 Human Pathway: HSA04664 125
90.2 Number of Hits: 9 125
90.3 Legend: 125

91 Glycolysis / Gluconeogenesis **126**
91.1 Human Pathway: HSA00010 126
91.2 Number of Hits: 9 126
91.3 Legend: 127

92 AGE-RAGE signaling pathway in diabetic complications **127**
92.1 Human Pathway: HSA04933 127
92.2 Number of Hits: 9 127
92.3 Legend: 127

93 Viral myocarditis **128**
93.1 Human Pathway: HSA05416 128
93.2 Number of Hits: 8 128
93.3 Legend: 128

94 Endocrine and other factor-regulated calcium reabsorption 129
94.1 Human Pathway: HSA04961 129
94.2 Number of Hits: 8 129
94.3 Legend: 129

95 Estrogen signaling pathway 130
95.1 Human Pathway: HSA04915 130
95.2 Number of Hits: 8 130
95.3 Legend: 130

96 Natural killer cell mediated cytotoxicity 131
96.1 Human Pathway: HSA04650 131
96.2 Number of Hits: 8 131
96.3 Legend: 131

97 Chronic myeloid leukemia 132
97.1 Human Pathway: HSA05220 132
97.2 Number of Hits: 8 132
97.3 Legend: 132

98 Fatty acid degradation 133
98.1 Human Pathway: HSA00071 133
98.2 Number of Hits: 8 133
98.3 Legend: 133

99 Lysine degradation 134
99.1 Human Pathway: HSA00310 134
99.2 Number of Hits: 8 134
99.3 Legend: 134

100 RNA degradation 135
100.1 Human Pathway: HSA03018 135
100.2 Number of Hits: 8 135
100.3 Legend: 135

101 Biosynthesis of amino acids 136
101.1 Human Pathway: HSA01230 136
101.2 Number of Hits: 8 136
101.3 Legend: 136

102 Insulin secretion **137**
102.1 Human Pathway: HSA04911 137
102.2 Number of Hits: 8 137
102.3 Legend: 137

103 Hypertrophic cardiomyopathy (HCM) **138**
103.1 Human Pathway: HSA05410 138
103.2 Number of Hits: 8 138
103.3 Legend: 138

104 Phosphatidylinositol signaling system **139**
104.1 Human Pathway: HSA04070 139
104.2 Number of Hits: 8 139
104.3 Legend: 139

105 Vascular smooth muscle contraction **140**
105.1 Human Pathway: HSA04270 140
105.2 Number of Hits: 8 140
105.3 Legend: 140

106 Toll-like receptor signaling pathway **141**
106.1 Human Pathway: HSA04620 141
106.2 Number of Hits: 8 141
106.3 Legend: 141

107 Jak-STAT signaling pathway **142**
107.1 Human Pathway: HSA04630 142
107.2 Number of Hits: 8 142
107.3 Legend: 142

108 Aldosterone synthesis and secretion **143**
108.1 Human Pathway: HSA04925 143
108.2 Number of Hits: 8 143
108.3 Legend: 143

109 Amphetamine addiction **144**
109.1 Human Pathway: HSA05031 144
109.2 Number of Hits: 8 144
109.3 Legend: 144

110 Synaptic vesicle cycle **145**
110.1 Human Pathway: HSA04721 145
110.2 Number of Hits: 8 145
110.3 Legend: 145

111 Cysteine and methionine metabolism **146**
111.1 Human Pathway: HSA00270 146
111.2 Number of Hits: 8 146
111.3 Legend: 146

112 MicroRNAs in cancer **147**
112.1 Human Pathway: HSA05206 147
112.2 Number of Hits: 8 147
112.3 Legend: 147

113 Inflammatory mediator regulation of TRP channels **148**
113.1 Human Pathway: HSA04750 148
113.2 Number of Hits: 8 148
113.3 Legend: 148

114 Cardiac muscle contraction **149**
114.1 Human Pathway: HSA04260 149
114.2 Number of Hits: 8 149
114.3 Legend: 149

115 Amoebiasis **150**
115.1 Human Pathway: HSA05146 150
115.2 Number of Hits: 8 150
115.3 Legend: 150

116 Dilated cardiomyopathy **151**
116.1 Human Pathway: HSA05414 151
116.2 Number of Hits: 7 151
116.3 Legend: 151

117 Progesterone-mediated oocyte maturation **152**
117.1 Human Pathway: HSA04914 152
117.2 Number of Hits: 7 152
117.3 Legend: 152

118 Protein digestion and absorption 153
118.1 Human Pathway: HSA04974 153
118.2 Number of Hits: 7 153
118.3 Legend: 153

119 Melanogenesis 154
119.1 Human Pathway: HSA04916 154
119.2 Number of Hits: 7 154
119.3 Legend: 154

120 Colorectal cancer 155
120.1 Human Pathway: HSA05210 155
120.2 Number of Hits: 7 155
120.3 Legend: 155

121 DNA replication 156
121.1 Human Pathway: HSA03030 156
121.2 Number of Hits: 7 156
121.3 Legend: 156

122 Fatty acid metabolism 157
122.1 Human Pathway: HSA01212 157
122.2 Number of Hits: 7 157
122.3 Legend: 158

123 Circadian rhythm 158
123.1 Human Pathway: HSA04710 158
123.2 Number of Hits: 7 158
123.3 Legend: 158

124 Amino sugar and nucleotide sugar metabolism 159
124.1 Human Pathway: HSA00520 159
124.2 Number of Hits: 7 159
124.3 Legend: 160

125 Basal transcription factors 160
125.1 Human Pathway: HSA03022 160
125.2 Number of Hits: 7 160
125.3 Legend: 161

126 TGF-beta signaling pathway **161**
126.1 Human Pathway: HSA04350 161
126.2 Number of Hits: 7 161
126.3 Legend: 161

127 Peroxisome **162**
127.1 Human Pathway: HSA04146 162
127.2 Number of Hits: 7 162
127.3 Legend: 162

128 Epithelial cell signaling in Helicobacter pylori infection **163**
128.1 Human Pathway: HSA05120 163
128.2 Number of Hits: 7 163
128.3 Legend: 163

129 Starch and sucrose metabolism **164**
129.1 Human Pathway: HSA00500 164
129.2 Number of Hits: 7 164
129.3 Legend: 164

130 Glycerophospholipid metabolism **165**
130.1 Human Pathway: HSA00564 165
130.2 Number of Hits: 7 165
130.3 Legend: 165

131 Gap junction **166**
131.1 Human Pathway: HSA04540 166
131.2 Number of Hits: 7 166
131.3 Legend: 166

132 Tuberculosis **167**
132.1 Human Pathway: HSA05152 167
132.2 Number of Hits: 7 167
132.3 Legend: 167

133 Transcriptional misregulation in cancer **168**
133.1 Human Pathway: HSA05202 168
133.2 Number of Hits: 7 168
133.3 Legend: 168

134 Prolactin signaling pathway **169**
134.1 Human Pathway: HSA04917 169
134.2 Number of Hits: 7 169
134.3 Legend: 169

135 Glycerolipid metabolism **170**
135.1 Human Pathway: HSA00561 170
135.2 Number of Hits: 7 170
135.3 Legend: 170

136 Non-small cell lung cancer **171**
136.1 Human Pathway: HSA05223 171
136.2 Number of Hits: 7 171
136.3 Legend: 171

137 Pentose and glucuronate interconversions **172**
137.1 Human Pathway: HSA00040 172
137.2 Number of Hits: 7 172
137.3 Legend: 172

138 Central carbon metabolism in cancer **173**
138.1 Human Pathway: HSA05230 173
138.2 Number of Hits: 7 173
138.3 Legend: 173

139 Arrhythmogenic right ventricular cardiomyopathy (ARVC) **174**
139.1 Human Pathway: HSA05412 174
139.2 Number of Hits: 7 174
139.3 Legend: 174

140 p53 signaling pathway **175**
140.1 Human Pathway: HSA04115 175
140.2 Number of Hits: 6 175
140.3 Legend: 175

141 Long-term potentiation **176**
141.1 Human Pathway: HSA04720 176
141.2 Number of Hits: 6 176
141.3 Legend: 176

142VEGF signaling pathway **177**
142.1Human Pathway: HSA04370 177
142.2Number of Hits: 6 177
142.3Legend: 177

143Measles **178**
143.1Human Pathway: HSA05162 178
143.2Number of Hits: 6 178
143.3Legend: 179

144Propanoate metabolism **179**
144.1Human Pathway: HSA00640 179
144.2Number of Hits: 6 179
144.3Legend: 179

145Cocaine addiction **180**
145.1Human Pathway: HSA05030 180
145.2Number of Hits: 6 180
145.3Legend: 180

146Adherens junction **181**
146.1Human Pathway: HSA04520 181
146.2Number of Hits: 6 181
146.3Legend: 181

147Glioma **182**
147.1Human Pathway: HSA05214 182
147.2Number of Hits: 6 182
147.3Legend: 182

148SNARE interactions in vesicular transport **183**
148.1Human Pathway: HSA04130 183
148.2Number of Hits: 6 183
148.3Legend: 183

149Terpenoid backbone biosynthesis **184**
149.1Human Pathway: HSA00900 184
149.2Number of Hits: 6 184
149.3Legend: 185

150 EGFR tyrosine kinase inhibitor resistance **185**
150.1 Human Pathway: HSA01521 185
150.2 Number of Hits: 6 185
150.3 Legend: 185

151 Drug metabolism - other enzymes **186**
151.1 Human Pathway: HSA00983 186
151.2 Number of Hits: 6 186
151.3 Legend: 186

152 Acute myeloid leukemia **187**
152.1 Human Pathway: HSA05221 187
152.2 Number of Hits: 6 187
152.3 Legend: 187

153 Hedgehog signaling pathway **188**
153.1 Human Pathway: HSA04340 188
153.2 Number of Hits: 6 188
153.3 Legend: 188

154 Chagas disease (American trypanosomiasis) **189**
154.1 Human Pathway: HSA05142 189
154.2 Number of Hits: 6 189
154.3 Legend: 189

155 Amyotrophic lateral sclerosis (ALS) **190**
155.1 Human Pathway: HSA05014 190
155.2 Number of Hits: 6 190
155.3 Legend: 190

156 Prostate cancer **191**
156.1 Human Pathway: HSA05215 191
156.2 Number of Hits: 6 191
156.3 Legend: 191

157 Hepatitis C **192**
157.1 Human Pathway: HSA05160 192
157.2 Number of Hits: 6 192
157.3 Legend: 192

158 Nicotine addiction **193**
158.1 Human Pathway: HSA05033 193
158.2 Number of Hits: 6 193
158.3 Legend: 193

159 Vasopressin-regulated water reabsorption **194**
159.1 Human Pathway: HSA04962 194
159.2 Number of Hits: 6 194
159.3 Legend: 194

160 Regulation of lipolysis in adipocytes **195**
160.1 Human Pathway: HSA04923 195
160.2 Number of Hits: 6 195
160.3 Legend: 195

161 PPAR signaling pathway **196**
161.1 Human Pathway: HSA03320 196
161.2 Number of Hits: 6 196
161.3 Legend: 196

162 Galactose metabolism **197**
162.1 Human Pathway: HSA00052 197
162.2 Number of Hits: 5 197
162.3 Legend: 197

163 Salivary secretion **198**
163.1 Human Pathway: HSA04970 198
163.2 Number of Hits: 5 198
163.3 Legend: 198

164 GnRH signaling pathway **199**
164.1 Human Pathway: HSA04912 199
164.2 Number of Hits: 5 199
164.3 Legend: 199

165 Endometrial cancer **200**
165.1 Human Pathway: HSA05213 200
165.2 Number of Hits: 5 200
165.3 Legend: 200

166 Base excision repair **201**
166.1 Human Pathway: HSA03410 201
166.2 Number of Hits: 5 201
166.3 Legend: 201

167 NF-kappa B signaling pathway **202**
167.1 Human Pathway: HSA04064 202
167.2 Number of Hits: 5 202
167.3 Legend: 202

168 Inositol phosphate metabolism **203**
168.1 Human Pathway: HSA00562 203
168.2 Number of Hits: 5 203
168.3 Legend: 203

169 Carbohydrate digestion and absorption **204**
169.1 Human Pathway: HSA04973 204
169.2 Number of Hits: 5 204
169.3 Legend: 204

170 Aminoacyl-tRNA biosynthesis **205**
170.1 Human Pathway: HSA00970 205
170.2 Number of Hits: 5 205
170.3 Legend: 205

171 Proteasome **206**
171.1 Human Pathway: HSA03050 206
171.2 Number of Hits: 5 206
171.3 Legend: 206

172 Glyoxylate and dicarboxylate metabolism **207**
172.1 Human Pathway: HSA00630 207
172.2 Number of Hits: 5 207
172.3 Legend: 207

173 Olfactory transduction **208**
173.1 Human Pathway: HSA04740 208
173.2 Number of Hits: 5 208
173.3 Legend: 208

174 Pyruvate metabolism **209**
174.1 Human Pathway: HSA00620 209
174.2 Number of Hits: 5 209
174.3 Legend: 209

175 Type II diabetes mellitus **210**
175.1 Human Pathway: HSA04930 210
175.2 Number of Hits: 4 210
175.3 Legend: 210

176 Synthesis and degradation of ketone bodies **211**
176.1 Human Pathway: HSA00072 211
176.2 Number of Hits: 4 211
176.3 Legend: 211

177 Alanine, aspartate and glutamate metabolism **212**
177.1 Human Pathway: HSA00250 212
177.2 Number of Hits: 4 212
177.3 Legend: 212

178 Notch signaling pathway **213**
178.1 Human Pathway: HSA04330 213
178.2 Number of Hits: 4 213
178.3 Legend: 213

179 Melanoma **214**
179.1 Human Pathway: HSA05218 214
179.2 Number of Hits: 4 214
179.3 Legend: 214

180 Ribosome biogenesis in eukaryotes **215**
180.1 Human Pathway: HSA03008 215
180.2 Number of Hits: 4 215
180.3 Legend: 216

181 Sphingolipid metabolism **216**
181.1 Human Pathway: HSA00600 216
181.2 Number of Hits: 4 216
181.3 Legend: 216

182 Arachidonic acid metabolism 217
182.1 Human Pathway: HSA00590 217
182.2 Number of Hits: 4 217
182.3 Legend: 217

183 Butanoate metabolism 218
183.1 Human Pathway: HSA00650 218
183.2 Number of Hits: 4 218
183.3 Legend: 218

184 Homologous recombination 219
184.1 Human Pathway: HSA03440 219
184.2 Number of Hits: 4 219
184.3 Legend: 219

185 Cytosolic DNA-sensing pathway 220
185.1 Human Pathway: HSA04623 220
185.2 Number of Hits: 4 220
185.3 Legend: 220

186 Gastric acid secretion 221
186.1 Human Pathway: HSA04971 221
186.2 Number of Hits: 4 221
186.3 Legend: 221

187 Tryptophan metabolism 222
187.1 Human Pathway: HSA00380 222
187.2 Number of Hits: 4 222
187.3 Legend: 222

188 Nucleotide excision repair 223
188.1 Human Pathway: HSA03420 223
188.2 Number of Hits: 4 223
188.3 Legend: 223

189 Cytokine-cytokine receptor interaction 224
189.1 Human Pathway: HSA04060 224
189.2 Number of Hits: 4 224
189.3 Legend: 224

190 Fructose and mannose metabolism **225**
190.1 Human Pathway: HSA00051 225
190.2 Number of Hits: 4 225
190.3 Legend: 225

191 Platinum drug resistance **226**
191.1 Human Pathway: HSA01524 226
191.2 Number of Hits: 4 226
191.3 Legend: 226

192 beta-Alanine metabolism **227**
192.1 Human Pathway: HSA00410 227
192.2 Number of Hits: 4 227
192.3 Legend: 227

193 Bile secretion **228**
193.1 Human Pathway: HSA04976 228
193.2 Number of Hits: 4 228
193.3 Legend: 228

194 Pathogenic Escherichia coli infection **229**
194.1 Human Pathway: HSA05130 229
194.2 Number of Hits: 4 229
194.3 Legend: 229

195 Glycine, serine and threonine metabolism **230**
195.1 Human Pathway: HSA00260 230
195.2 Number of Hits: 4 230
195.3 Legend: 230

196 Hippo signaling pathway -multiple species **231**
196.1 Human Pathway: HSA04392 231
196.2 Number of Hits: 4 231
196.3 Legend: 231

197 Arginine and proline metabolism **232**
197.1 Human Pathway: HSA00330 232
197.2 Number of Hits: 4 232
197.3 Legend: 232

198 RNA polymerase 233
198.1 Human Pathway: HSA03020 233
198.2 Number of Hits: 4 233
198.3 Legend: 233

199 Pentose phosphate pathway 234
199.1 Human Pathway: HSA00030 234
199.2 Number of Hits: 4 234
199.3 Legend: 234

200 Mineral absorption 235
200.1 Human Pathway: HSA04978 235
200.2 Number of Hits: 3 235
200.3 Legend: 235

201 Legionellosis 236
201.1 Human Pathway: HSA05134 236
201.2 Number of Hits: 3 236
201.3 Legend: 236

202 Porphyrin and chlorophyll metabolism 237
202.1 Human Pathway: HSA00860 237
202.2 Number of Hits: 3 237
202.3 Legend: 237

203 Glycosaminoglycan biosynthesis - heparan sulfate / heparin 238
203.1 Human Pathway: HSA00534 238
203.2 Number of Hits: 3 238
203.3 Legend: 238

204 Renin secretion 239
204.1 Human Pathway: HSA04924 239
204.2 Number of Hits: 3 239
204.3 Legend: 239

205 Tyrosine metabolism 240
205.1 Human Pathway: HSA00350 240
205.2 Number of Hits: 3 240
205.3 Legend: 240

206 Steroid biosynthesis **241**
206.1 Human Pathway: HSA00100 241
206.2 Number of Hits: 3 241
206.3 Legend: 241

207 Thyroid hormone synthesis **242**
207.1 Human Pathway: HSA04918 242
207.2 Number of Hits: 3 242
207.3 Legend: 242

208 Folate biosynthesis **243**
208.1 Human Pathway: HSA00790 243
208.2 Number of Hits: 3 243
208.3 Legend: 243

209 Basal cell carcinoma **244**
209.1 Human Pathway: HSA05217 244
209.2 Number of Hits: 3 244
209.3 Legend: 244

210 N-Glycan biosynthesis **245**
210.1 Human Pathway: HSA00510 245
210.2 Number of Hits: 3 245
210.3 Legend: 246

211 Glutathione metabolism **246**
211.1 Human Pathway: HSA00480 246
211.2 Number of Hits: 3 246
211.3 Legend: 247

212 Long-term depression **247**
212.1 Human Pathway: HSA04730 247
212.2 Number of Hits: 3 247
212.3 Legend: 247

213 Regulation of autophagy **248**
213.1 Human Pathway: HSA04140 248
213.2 Number of Hits: 3 248
213.3 Legend: 248

214 Fatty acid elongation 249
214.1 Human Pathway: HSA00062 249
214.2 Number of Hits: 3 249
214.3 Legend: 250

215 Aldosterone-regulated sodium reabsorption 250
215.1 Human Pathway: HSA04960 250
215.2 Number of Hits: 3 250
215.3 Legend: 251

216 2-Oxocarboxylic acid metabolism 251
216.1 Human Pathway: HSA01210 251
216.2 Number of Hits: 3 251
216.3 Legend: 252

217 Antigen processing and presentation 252
217.1 Human Pathway: HSA04612 252
217.2 Number of Hits: 3 252
217.3 Legend: 252

218 Ascorbate and aldarate metabolism 253
218.1 Human Pathway: HSA00053 253
218.2 Number of Hits: 3 253
218.3 Legend: 253

219 Fat digestion and absorption 254
219.1 Human Pathway: HSA04975 254
219.2 Number of Hits: 3 254
219.3 Legend: 254

220 Prion diseases 255
220.1 Human Pathway: HSA05020 255
220.2 Number of Hits: 3 255
220.3 Legend: 255

221 Bladder cancer 256
221.1 Human Pathway: HSA05219 256
221.2 Number of Hits: 2 256
221.3 Legend: 256

222 RIG-I-like receptor signaling pathway 257
222.1 Human Pathway: HSA04622 257
222.2 Number of Hits: 2 257
222.3 Legend: 257

223 Leishmaniasis 258
223.1 Human Pathway: HSA05140 258
223.2 Number of Hits: 2 258
223.3 Legend: 258

224 Nicotinate and nicotinamide metabolism 259
224.1 Human Pathway: HSA00760 259
224.2 Number of Hits: 2 259
224.3 Legend: 259

225 Metabolism of xenobiotics by cytochrome P450 260
225.1 Human Pathway: HSA00980 260
225.2 Number of Hits: 2 260
225.3 Legend: 260

226 Citrate cycle (TCA cycle) 261
226.1 Human Pathway: HSA00020 261
226.2 Number of Hits: 2 261
226.3 Legend: 261

227 Protein export 262
227.1 Human Pathway: HSA03060 262
227.2 Number of Hits: 2 262
227.3 Legend: 262

228 Biosynthesis of unsaturated fatty acids 263
228.1 Human Pathway: HSA01040 263
228.2 Number of Hits: 2 263
228.3 Legend: 263

229 Other types of O-glycan biosynthesis 264
229.1 Human Pathway: HSA00514 264
229.2 Number of Hits: 2 264
229.3 Legend: 264

230 NOD-like receptor signaling pathway 265
230.1 Human Pathway: HSA04621 265
230.2 Number of Hits: 2 265
230.3 Legend: 265

231 One carbon pool by folate 266
231.1 Human Pathway: HSA00670 266
231.2 Number of Hits: 2 266
231.3 Legend: 266

232 Mismatch repair 267
232.1 Human Pathway: HSA03430 267
232.2 Number of Hits: 2 267
232.3 Legend: 267

233 Pantothenate and CoA biosynthesis 268
233.1 Human Pathway: HSA00770 268
233.2 Number of Hits: 2 268
233.3 Legend: 268

234 Rheumatoid arthritis 269
234.1 Human Pathway: HSA05323 269
234.2 Number of Hits: 2 269
234.3 Legend: 269

235 Malaria 270
235.1 Human Pathway: HSA05144 270
235.2 Number of Hits: 2 270
235.3 Legend: 270

236 Histidine metabolism 271
236.1 Human Pathway: HSA00340 271
236.2 Number of Hits: 2 271
236.3 Legend: 271

237 Glycosaminoglycan biosynthesis - chondroitin sulfate / dermatan sulfate 272
237.1 Human Pathway: HSA00532 272
237.2 Number of Hits: 2 272
237.3 Legend: 272

238 Glycosaminoglycan biosynthesis - keratan sulfate 273
238.1 Human Pathway: HSA00533 273
238.2 Number of Hits: 2 273
238.3 Legend: 273

239 Vibrio cholerae infection 274
239.1 Human Pathway: HSA05110 274
239.2 Number of Hits: 2 274
239.3 Legend: 274

240 Ether lipid metabolism 275
240.1 Human Pathway: HSA00565 275
240.2 Number of Hits: 2 275
240.3 Legend: 275

241 African trypanosomiasis 276
241.1 Human Pathway: HSA05143 276
241.2 Number of Hits: 2 276
241.3 Legend: 276

242 Fanconi anemia pathway 277
242.1 Human Pathway: HSA03460 277
242.2 Number of Hits: 2 277
242.3 Legend: 278

243 Thyroid cancer 278
243.1 Human Pathway: HSA05216 278
243.2 Number of Hits: 2 278
243.3 Legend: 278

244 Valine, leucine and isoleucine biosynthesis 279
244.1 Human Pathway: HSA00290 279
244.2 Number of Hits: 1 279
244.3 Legend: 279

245 Collecting duct acid secretion 280
245.1 Human Pathway: HSA04966 280
245.2 Number of Hits: 1 280
245.3 Legend: 281

246 Linoleic acid metabolism **281**
246.1 Human Pathway: HSA00591 281
246.2 Number of Hits: 1 281
246.3 Legend: 281

247 Pertussis **282**
247.1 Human Pathway: HSA05133 282
247.2 Number of Hits: 1 282
247.3 Legend: 282

248 Glycosphingolipid biosynthesis - ganglio series **283**
248.1 Human Pathway: HSA00604 283
248.2 Number of Hits: 1 283
248.3 Legend: 284

249 Retinol metabolism **284**
249.1 Human Pathway: HSA00830 284
249.2 Number of Hits: 1 284
249.3 Legend: 284

250 Primary immunodeficiency **285**
250.1 Human Pathway: HSA05340 285
250.2 Number of Hits: 1 285
250.3 Legend: 285

251 Glycosylphosphatidylinositol(GPI)-anchor biosynthesis **286**
251.1 Human Pathway: HSA00563 286
251.2 Number of Hits: 1 286
251.3 Legend: 287

252 Maturity onset diabetes of the young **288**
252.1 Human Pathway: HSA04950 288
252.2 Number of Hits: 1 288
252.3 Legend: 289

253 Non-homologous end-joining **289**
253.1 Human Pathway: HSA03450 289
253.2 Number of Hits: 1 289
253.3 Legend: 289

254 Taste transduction **290**
254.1 Human Pathway: HSA04742 290
254.2 Number of Hits: 1 290
254.3 Legend: 290

255 Sulfur metabolism **291**
255.1 Human Pathway: HSA00920 291
255.2 Number of Hits: 1 291
255.3 Legend: 291

256 Chemical carcinogenesis **293**
256.1 Human Pathway: HSA05204 293
256.2 Number of Hits: 1 293
256.3 Legend: 294

257 Dorso-ventral axis formation **294**
257.1 Human Pathway: HSA04320 294
257.2 Number of Hits: 1 294
257.3 Legend: 294

258 Glycosaminoglycan degradation **295**
258.1 Human Pathway: HSA00531 295
258.2 Number of Hits: 1 295
258.3 Legend: 295

259 Proximal tubule bicarbonate reclamation **296**
259.1 Human Pathway: HSA04964 296
259.2 Number of Hits: 1 296
259.3 Legend: 297

260 Glycosphingolipid biosynthesis - lacto and neolacto series **298**
260.1 Human Pathway: HSA00601 298
260.2 Number of Hits: 1 298
260.3 Legend: 299

261 Type I diabetes mellitus **299**
261.1 Human Pathway: HSA04940 299
261.2 Number of Hits: 1 299
261.3 Legend: 300

262 Fatty acid biosynthesis **300**
262.1 Human Pathway: HSA00061 300
262.2 Number of Hits: 1 300
262.3 Legend: 300

263 Systemic lupus erythematosus **301**
263.1 Human Pathway: HSA05322 301
263.2 Number of Hits: 1 301
263.3 Legend: 301

264 Taurine and hypotaurine metabolism **302**
264.1 Human Pathway: HSA00430 302
264.2 Number of Hits: 1 302
264.3 Legend: 302

265 Arginine biosynthesis **303**
265.1 Human Pathway: HSA00220 303
265.2 Number of Hits: 1 303
265.3 Legend: 303

266 Hematopoietic cell lineage **304**
266.1 Human Pathway: HSA04640 304
266.2 Number of Hits: 1 304
266.3 Legend: 305

267 Intestinal immune network for IgA production **305**
267.1 Human Pathway: HSA04672 305
267.2 Number of Hits: 1 305
267.3 Legend: 305

268 Other glycan degradation **306**
268.1 Human Pathway: HSA00511 306
268.2 Number of Hits: 1 306
268.3 Legend: 306

269 Phenylalanine, tyrosine and tryptophan biosynthesis **307**
269.1 Human Pathway: HSA00400 307
269.2 Number of Hits: 1 307
269.3 Legend: 307

270 Glycosphingolipid biosynthesis - globo series **308**
270.1 Human Pathway: HSA00603 308
270.2 Number of Hits: 1 308
270.3 Legend: 309

271 alpha-Linolenic acid metabolism **309**
271.1 Human Pathway: HSA00592 309
271.2 Number of Hits: 1 309
271.3 Legend: 310

272 Ovarian steroidogenesis **310**
272.1 Human Pathway: HSA04913 310
272.2 Number of Hits: 1 310
272.3 Legend: 311

273 Phenylalanine metabolism **311**
273.1 Human Pathway: HSA00360 311
273.2 Number of Hits: 1 311
273.3 Legend: 311

274 Phototransduction **312**
274.1 Human Pathway: HSA04744 312
274.2 Number of Hits: 1 312
274.3 Legend: 312

275 Selenocompound metabolism **313**
275.1 Human Pathway: HSA00450 313
275.2 Number of Hits: 1 313
275.3 Legend: 313

1 Endocytosis

1.1 Human Pathway: HSA04144

1.2 Number of Hits: 31

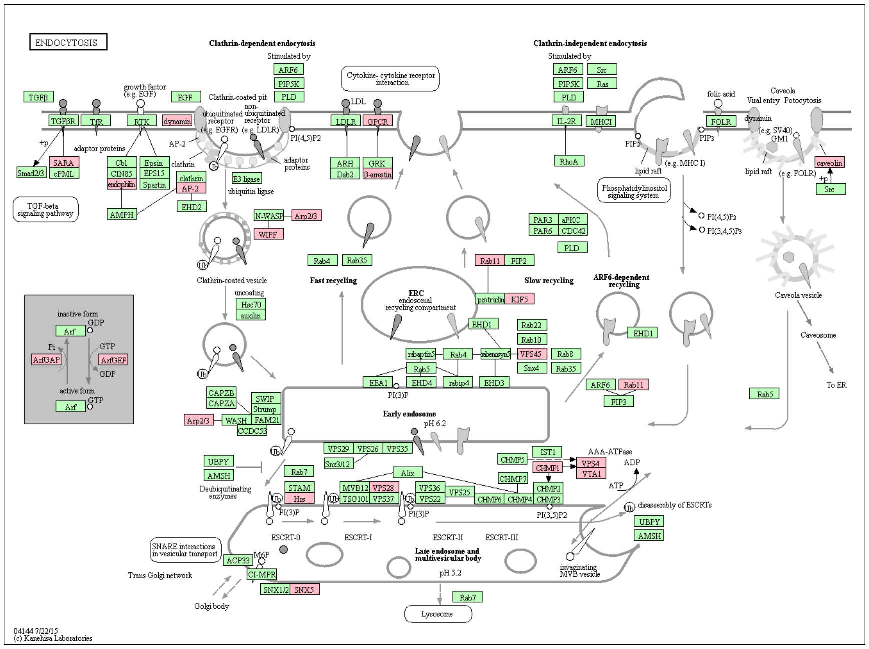

1.3 Legend:

RBH-Blast at 60% Identity + 50% Coverage
Green = Hit in *H. sapiens*
Red = Hit in *H. sapiens* and *T. californica*
White = Not in *H. sapiens*

2 Pathways in cancer

2.1 Human Pathway: HSA05200

2.2 Number of Hits: 30

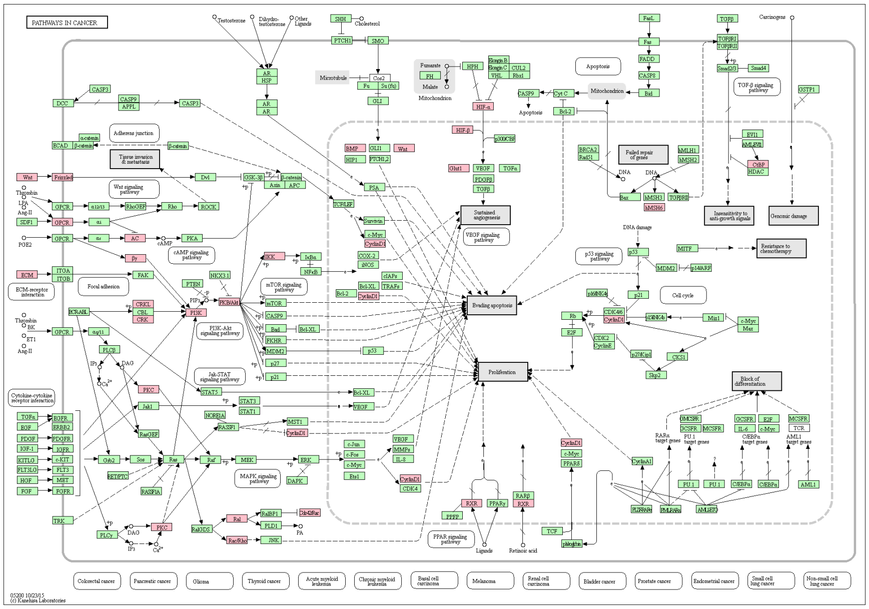

2.3 Legend:

RBH-Blast at 60% Identity + 50% Coverage
Green = Hit in *H. sapiens*
Red = Hit in *H. sapiens* and *T. californica*
White = Not in *H. sapiens*

3 PI3K-Akt signaling pathway

3.1 Human Pathway: HSA04151

3.2 Number of Hits: 29

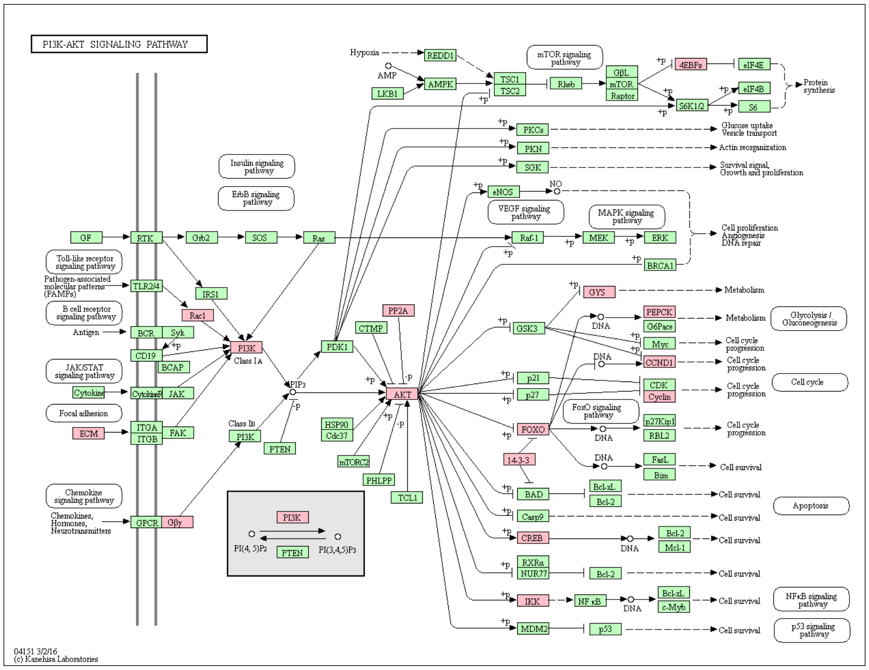

3.3 Legend:

RBH-Blast at 60% Identity + 50% Coverage
Green = Hit in *H. sapiens*
Red = Hit in *H. sapiens* and *T. californica*
White = Not in *H. sapiens*

4 Focal adhesion

4.1 Human Pathway: HSA04510

4.2 Number of Hits: 26

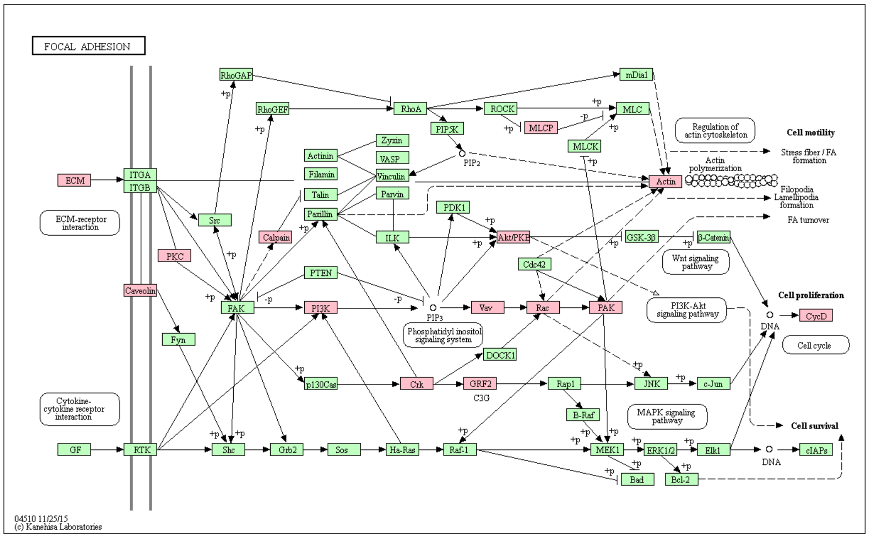

4.3 Legend:

RBH-Blast at 60% Identity + 50% Coverage
Green = Hit in *H. sapiens*
Red = Hit in *H. sapiens* and *T. californica*
White = Not in *H. sapiens*

5 HTLV-I infection

5.1 Human Pathway: HSA05166

5.2 Number of Hits: 23

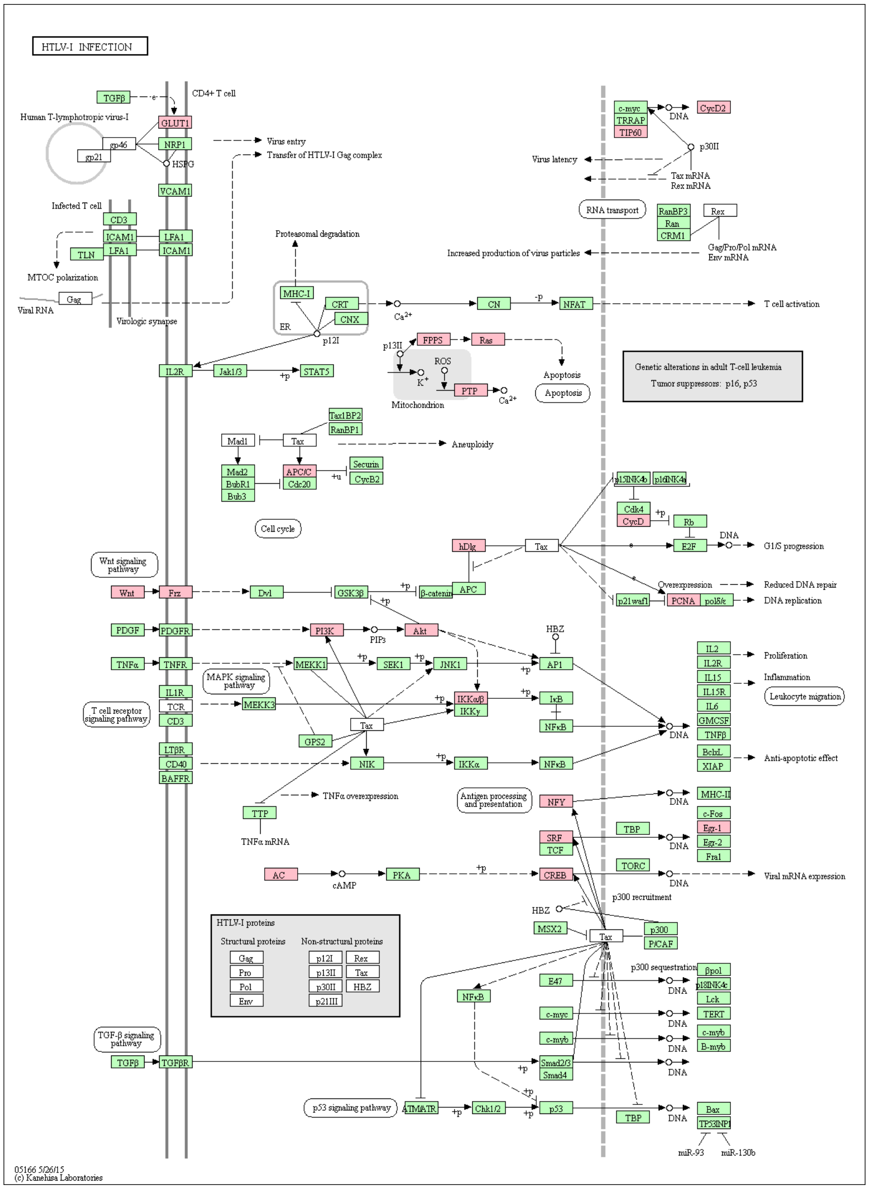

5.3 Legend:

RBH-Blast at 60% Identity + 50% Coverage
Green = Hit in *H. sapiens*
Red = Hit in *H. sapiens* and *T. californica*
White = Not in *H. sapiens*

---

6 MAPK signaling pathway

6.1 Human Pathway: HSA04010

6.2 Number of Hits: 23

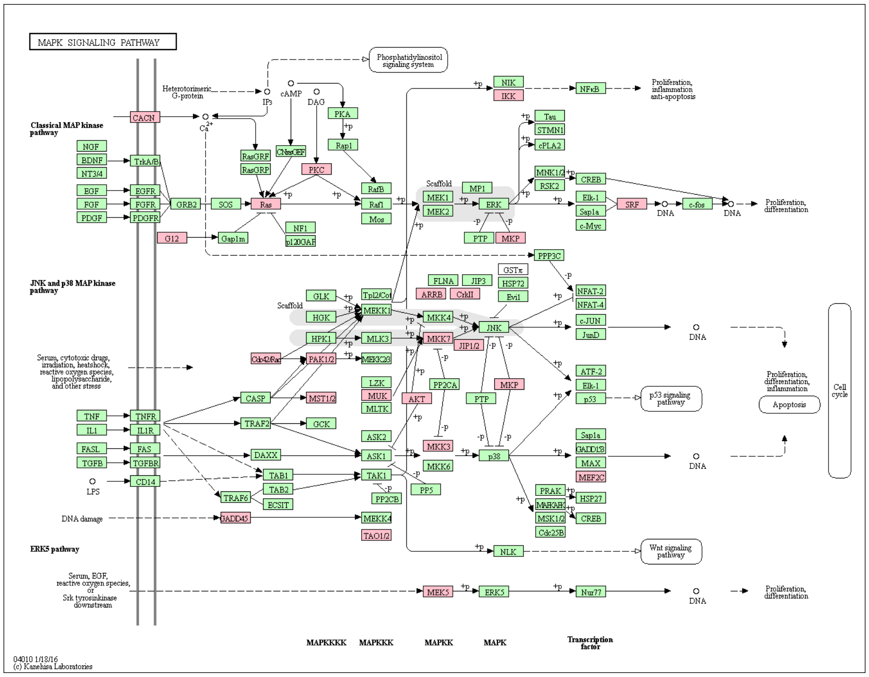

6.3 Legend:

RBH-Blast at 60% Identity + 50% Coverage
Green = Hit in *H. sapiens*
Red = Hit in *H. sapiens* and *T. californica*
White = Not in *H. sapiens*

---

7 Ras signaling pathway

7.1 Human Pathway: HSA04014

7.2 Number of Hits: 23

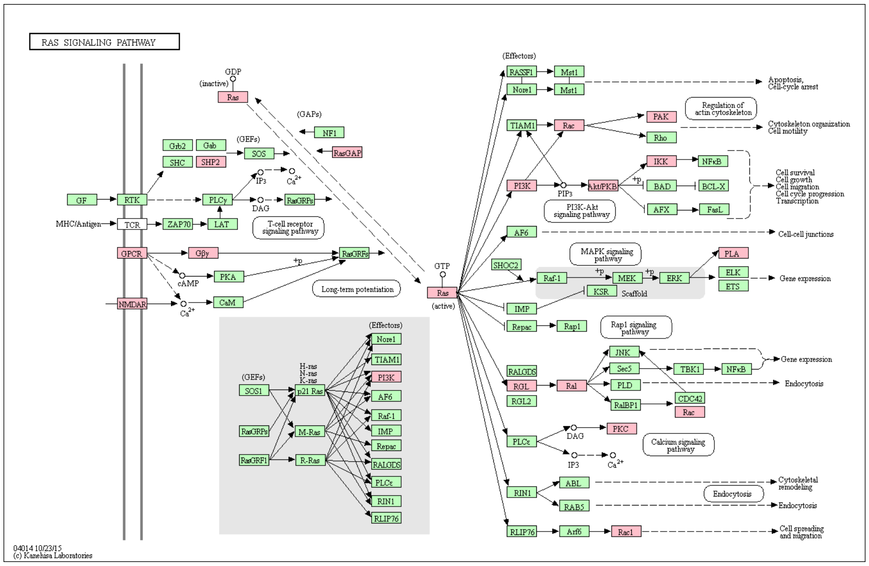

7.3 Legend:

RBH-Blast at 60% Identity + 50% Coverage
Green = Hit in *H. sapiens*
Red = Hit in *H. sapiens* and *T. californica*
White = Not in *H. sapiens*

8 Alzheimer's disease

8.1 Human Pathway: HSA05010

8.2 Number of Hits: 22

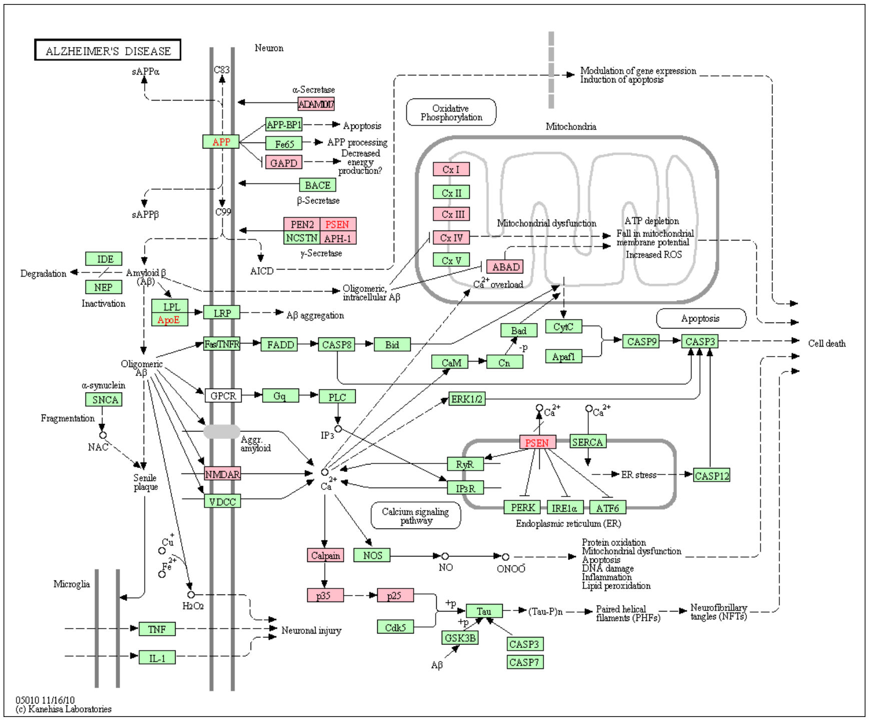

8.3 Legend:

RBH-Blast at 60% Identity + 50% Coverage
Green = Hit in *H. sapiens*
Red = Hit in *H. sapiens* and *T. californica*
White = Not in *H. sapiens*

9 RNA transport

9.1 Human Pathway: HSA03013

9.2 Number of Hits: 21

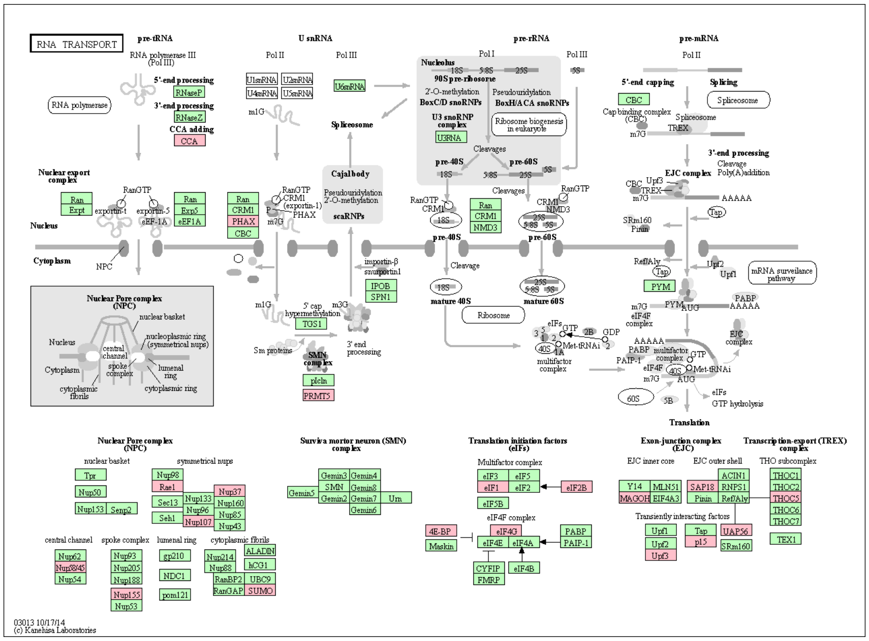

9.3 Legend:

RBH-Blast at 60% Identity + 50% Coverage
Green = Hit in *H. sapiens*
Red = Hit in *H. sapiens* and *T. californica*
White = Not in *H. sapiens*

10 Non-alcoholic fatty liver disease (NAFLD)

10.1 Human Pathway: HSA04932

10.2 Number of Hits: 20

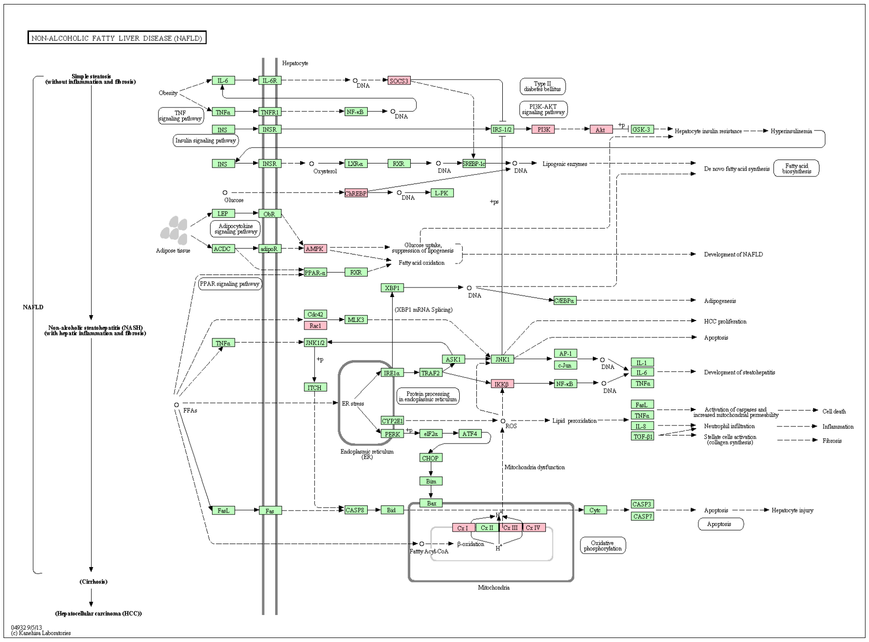

10.3 Legend:

RBH-Blast at 60% Identity + 50% Coverage
Green = Hit in *H. sapiens*
Red = Hit in *H. sapiens* and *T. californica*
White = Not in *H. sapiens*

11 Axon guidance

11.1 Human Pathway: HSA04360

11.2 Number of Hits: 20

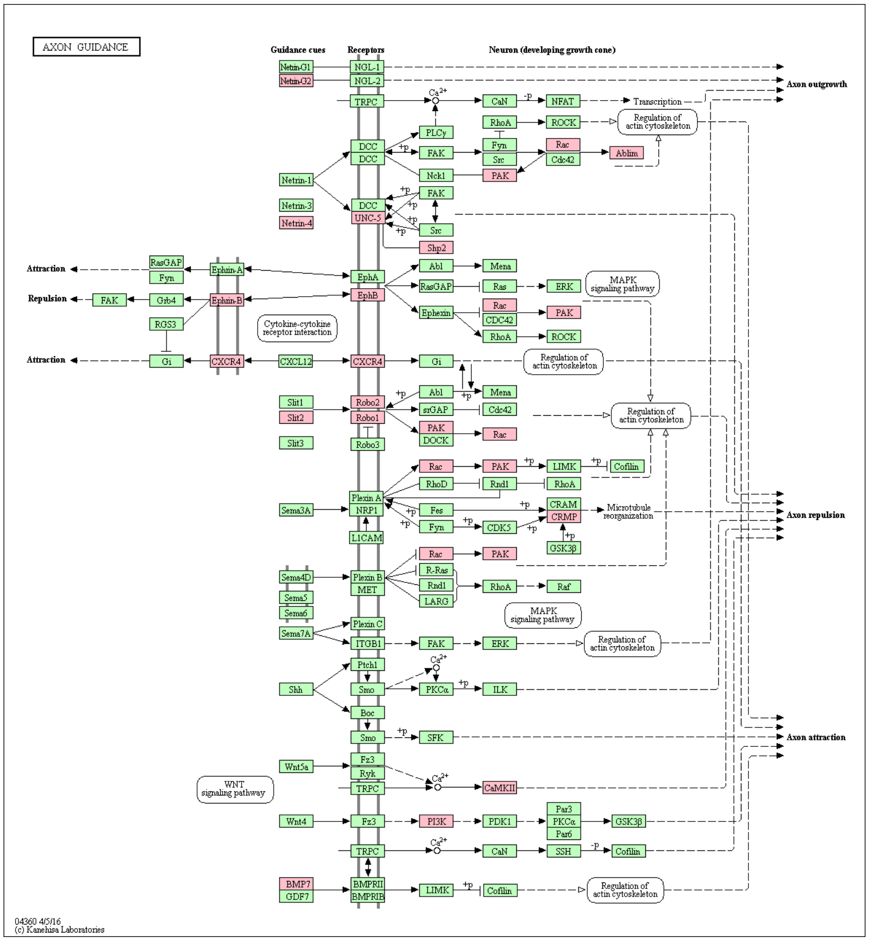

11.3 Legend:

RBH-Blast at 60% Identity + 50% Coverage
Green = Hit in *H. sapiens*
Red = Hit in *H. sapiens* and *T. californica*
White = Not in *H. sapiens*

12 Oxytocin signaling pathway

12.1 Human Pathway: HSA04921

12.2 Number of Hits: 20

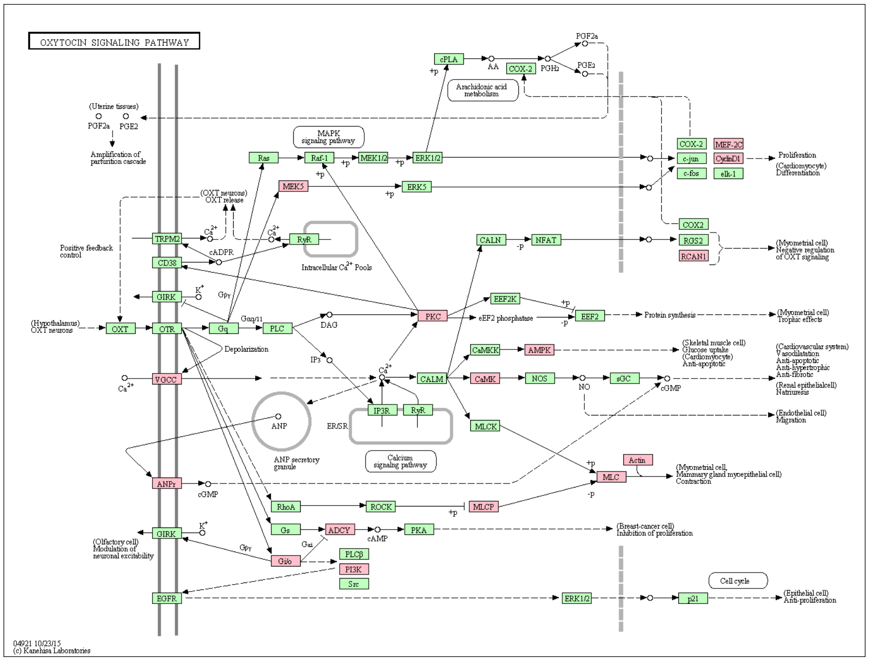

12.3 Legend:

RBH-Blast at 60% Identity + 50% Coverage
Green = Hit in *H. sapiens*
Red = Hit in *H. sapiens* and *T. californica*
White = Not in *H. sapiens*

13 Regulation of actin cytoskeleton

13.1 Human Pathway: HSA04810

13.2 Number of Hits: 20

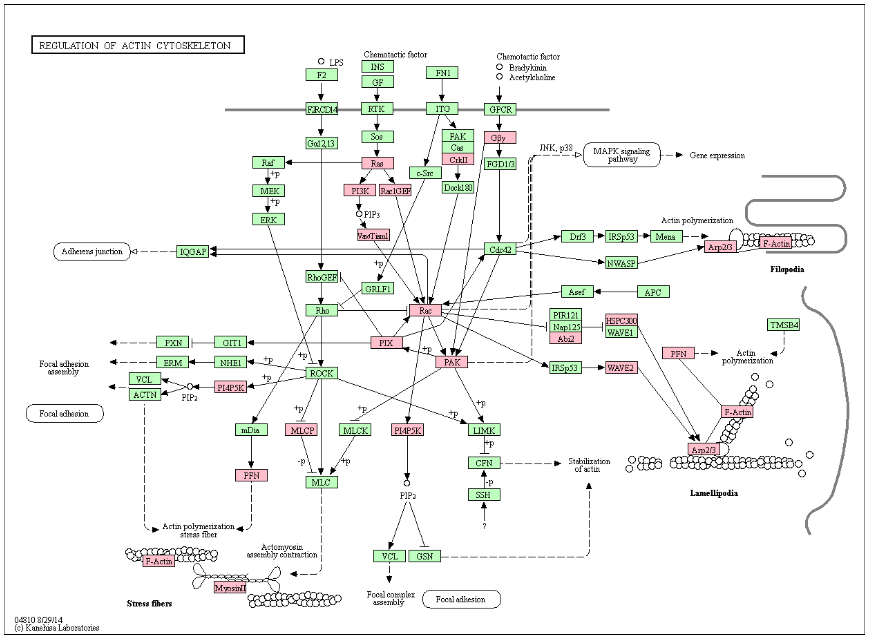

13.3 Legend:

RBH-Blast at 60% Identity + 50% Coverage
Green = Hit in *H. sapiens*
Red = Hit in *H. sapiens* and *T. californica*
White = Not in *H. sapiens*

14 Huntington's disease

14.1 Human Pathway: HSA05016

14.2 Number of Hits: 20

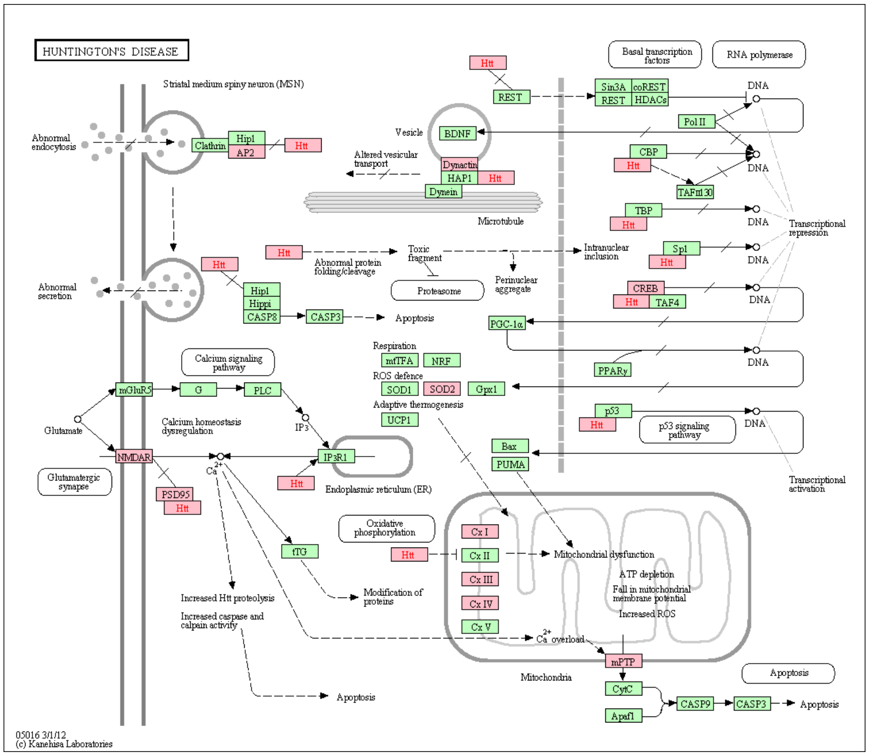

14.3 Legend:

RBH-Blast at 60% Identity + 50% Coverage
Green = Hit in *H. sapiens*
Red = Hit in *H. sapiens* and *T. californica*
White = Not in *H. sapiens*

15 Insulin resistance

15.1 Human Pathway: HSA04931

15.2 Number of Hits: 19

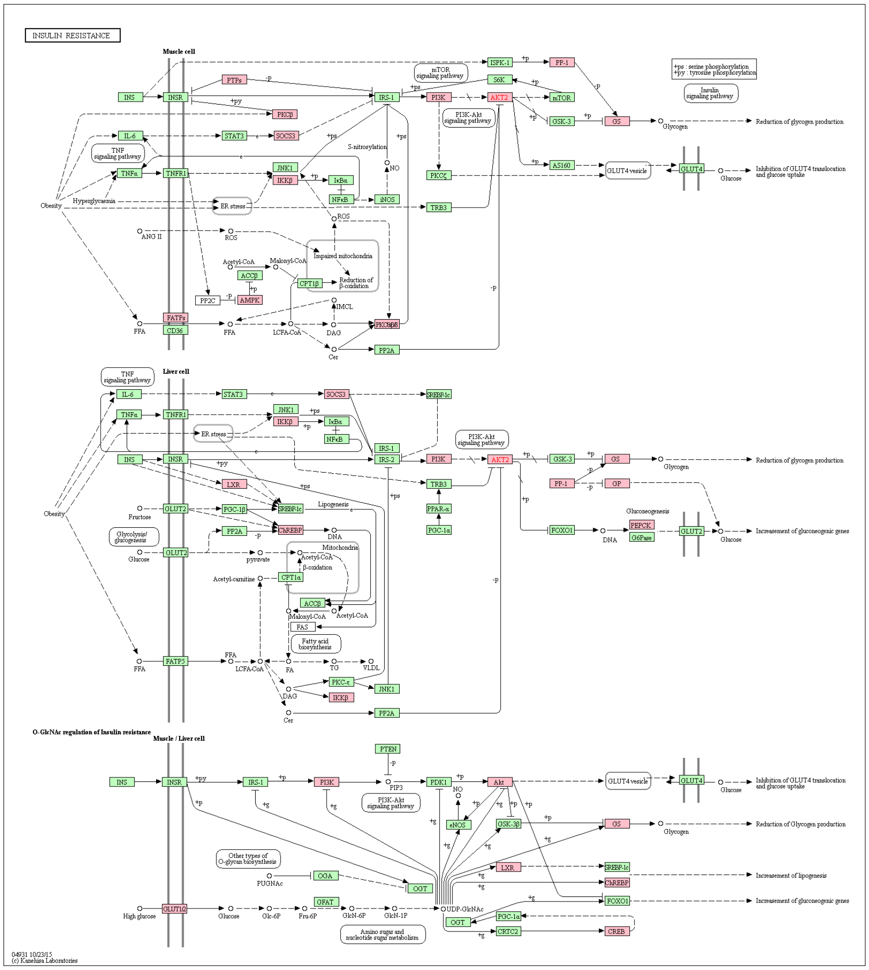

15.3 Legend:

RBH-Blast at 60% Identity + 50% Coverage
Green = Hit in *H. sapiens*
Red = Hit in *H. sapiens* and *T. californica*
White = Not in *H. sapiens*

16 cAMP signaling pathway

16.1 Human Pathway: HSA04024

16.2 Number of Hits: 19

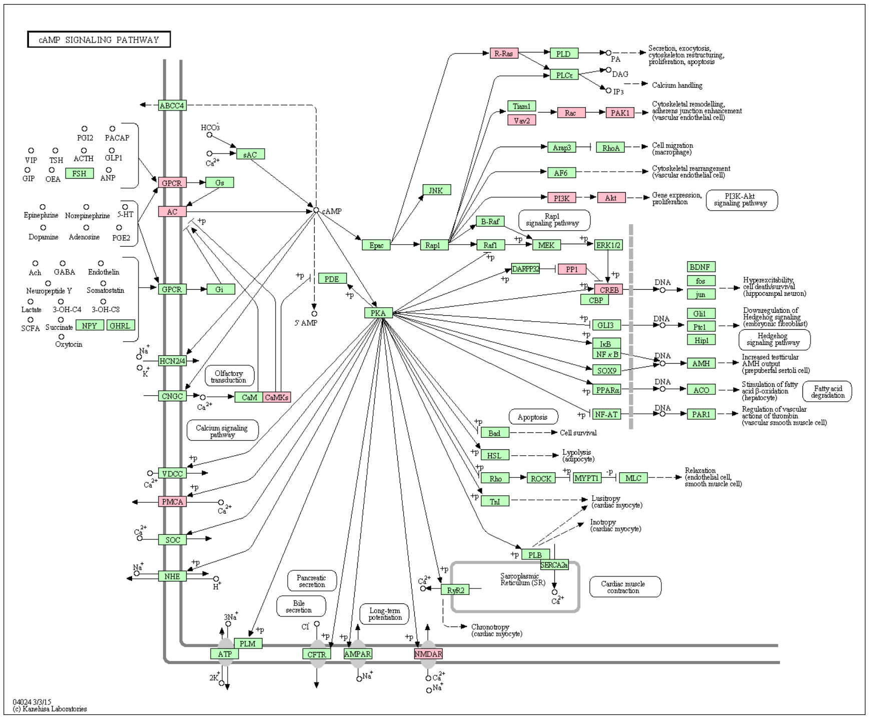

16.3 Legend:

RBH-Blast at 60% Identity + 50% Coverage
Green = Hit in *H. sapiens*
Red = Hit in *H. sapiens* and *T. californica*
White = Not in *H. sapiens*

17 Ubiquitin mediated proteolysis

17.1 Human Pathway: HSA04120

17.2 Number of Hits: 18

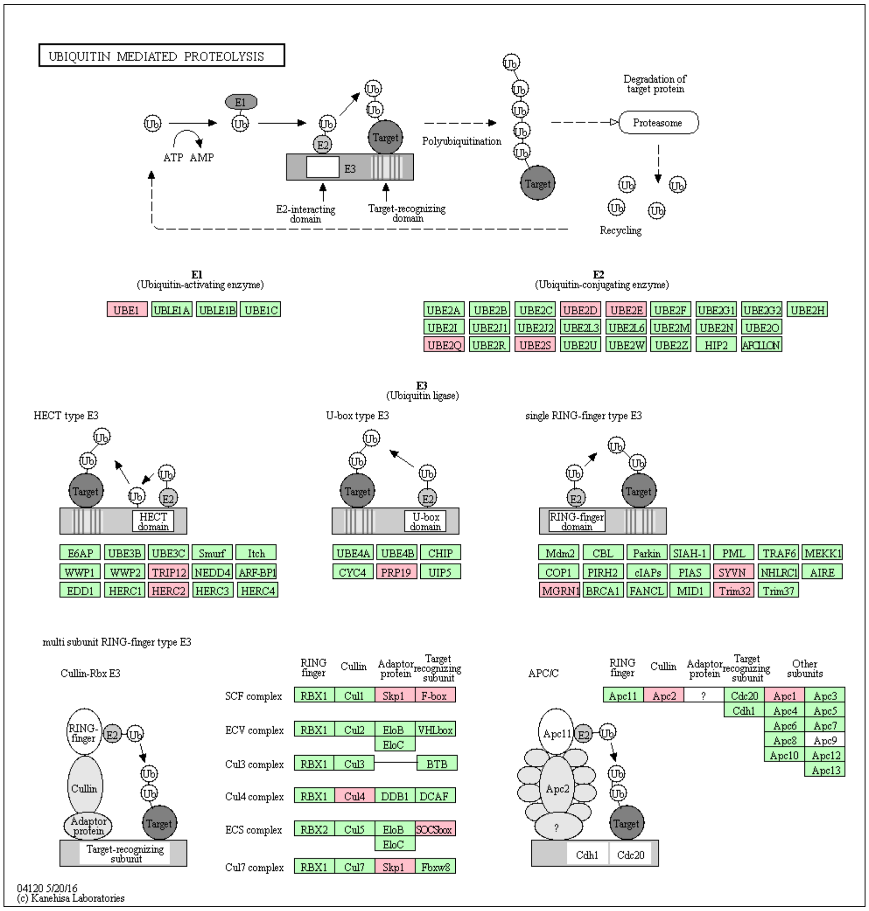

17.3 Legend:

RBH-Blast at 60% Identity + 50% Coverage
Green = Hit in *H. sapiens*
Red = Hit in *H. sapiens* and *T. californica*
White = Not in *H. sapiens*

18 Insulin signaling pathway

18.1 Human Pathway: HSA04910

18.2 Number of Hits: 18

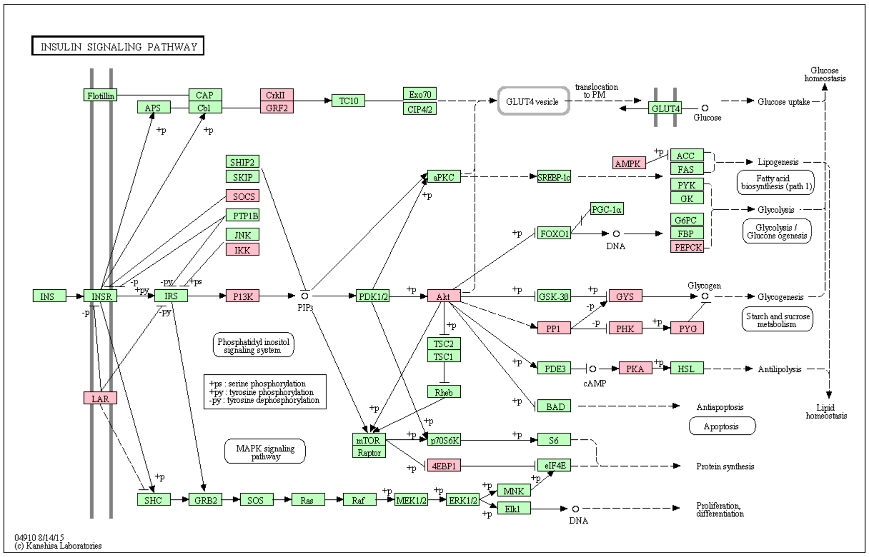

18.3 Legend:

RBH-Blast at 60% Identity + 50% Coverage
Green = Hit in *H. sapiens*
Red = Hit in *H. sapiens* and *T. californica*
White = Not in *H. sapiens*

19 Ribosome

19.1 Human Pathway: HSA03010

19.2 Number of Hits: 18

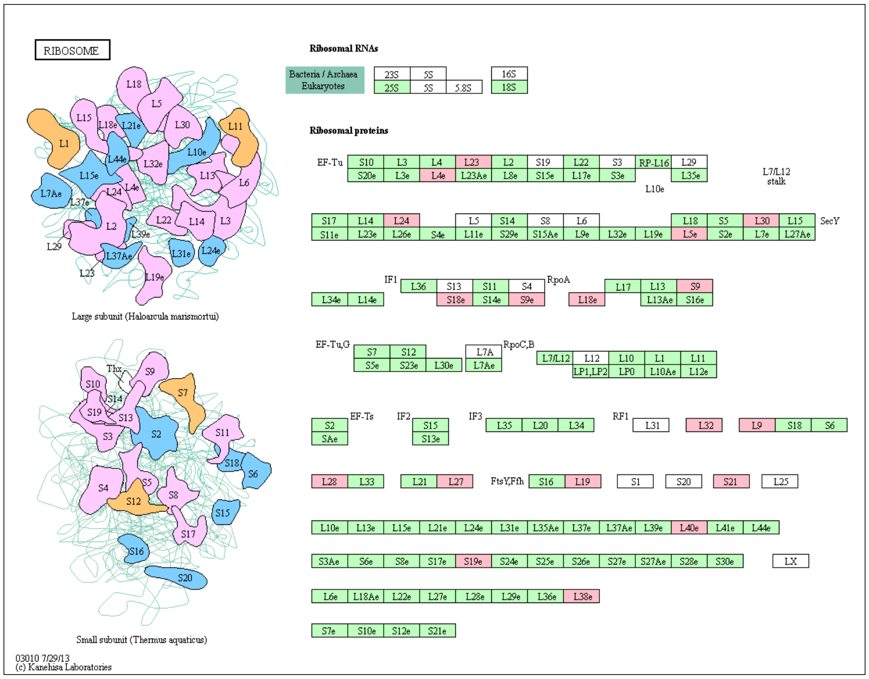

19.3 Legend:

RBH-Blast at 60% Identity + 50% Coverage
Green = Hit in *H. sapiens*
Red = Hit in *H. sapiens* and *T. californica*
White = Not in *H. sapiens*

20 Chemokine signaling pathway

20.1 Human Pathway: HSA04062

20.2 Number of Hits: 18

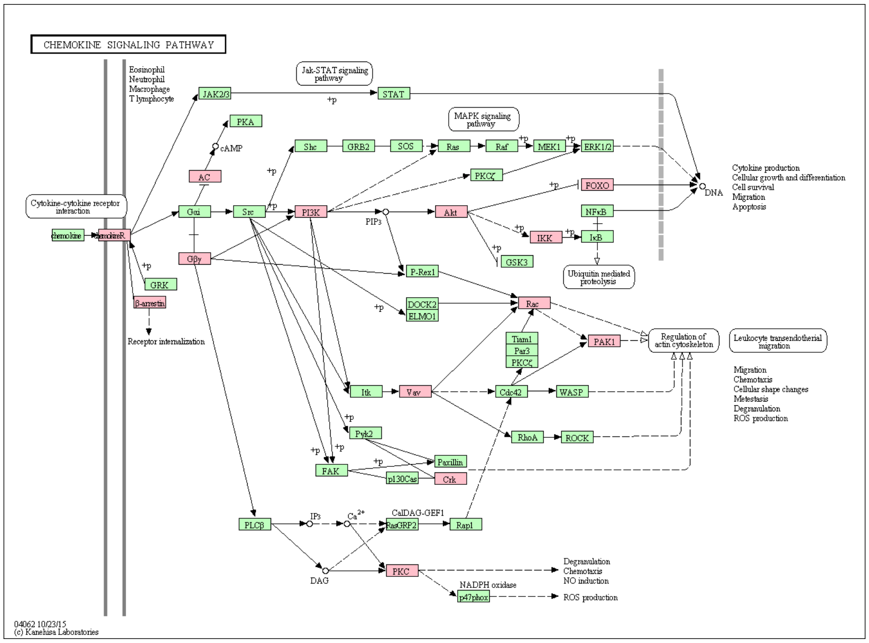

20.3 Legend:

RBH-Blast at 60% Identity + 50% Coverage
Green = Hit in *H. sapiens*
Red = Hit in *H. sapiens* and *T. californica*
White = Not in *H. sapiens*

21 AMPK signaling pathway

21.1 Human Pathway: HSA04152

21.2 Number of Hits: 18

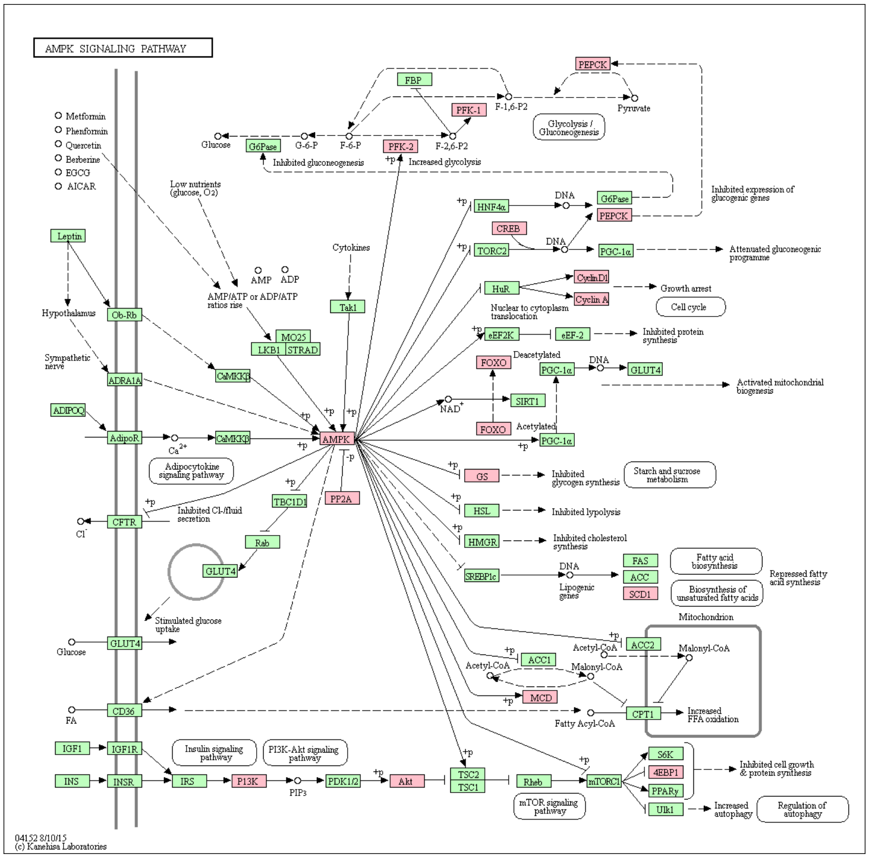

21.3 Legend:

RBH-Blast at 60% Identity + 50% Coverage
Green = Hit in *H. sapiens*
Red = Hit in *H. sapiens* and *T. californica*
White = Not in *H. sapiens*

22 cGMP-PKG signaling pathway

22.1 Human Pathway: HSA04022

22.2 Number of Hits: 18

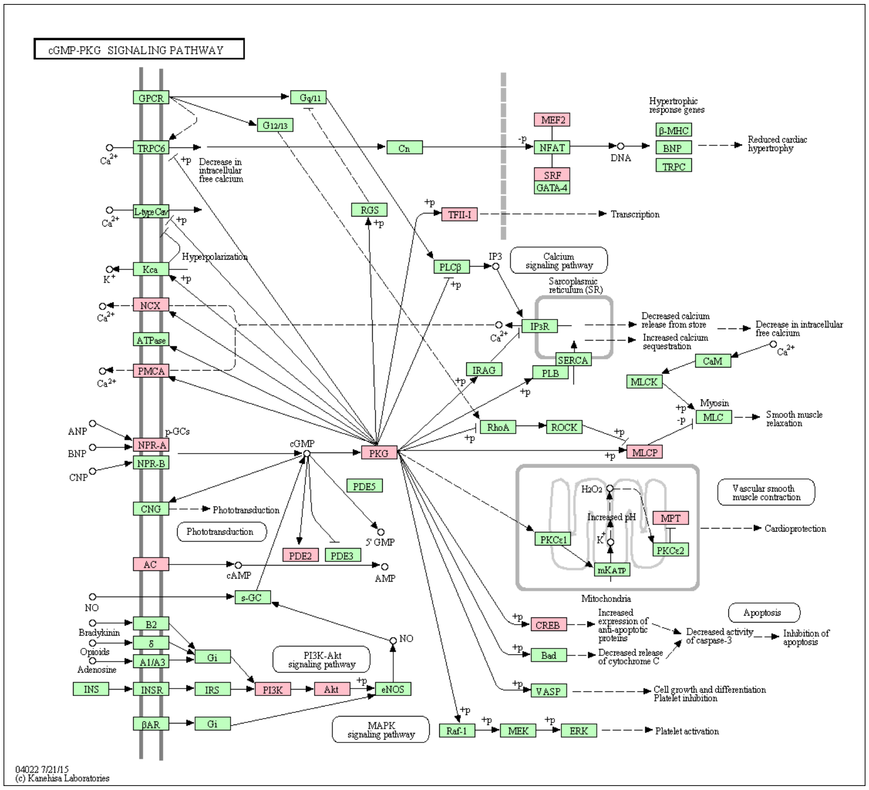

22.3 Legend:

RBH-Blast at 60% Identity + 50% Coverage
Green = Hit in *H. sapiens*
Red = Hit in *H. sapiens* and *T. californica*
White = Not in *H. sapiens*

23 Spliceosome

23.1 Human Pathway: HSA03040

23.2 Number of Hits: 18

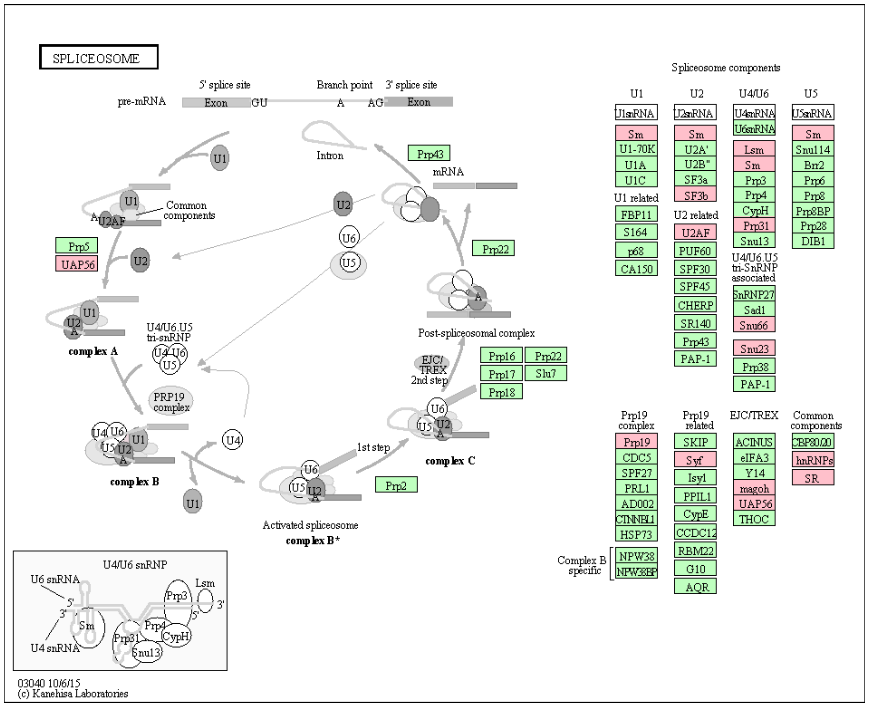

23.3 Legend:

RBH-Blast at 60% Identity + 50% Coverage
Green = Hit in *H. sapiens*
Red = Hit in *H. sapiens* and *T. californica*
White = Not in *H. sapiens*

24 Proteoglycans in cancer

24.1 Human Pathway: HSA05205

24.2 Number of Hits: 18

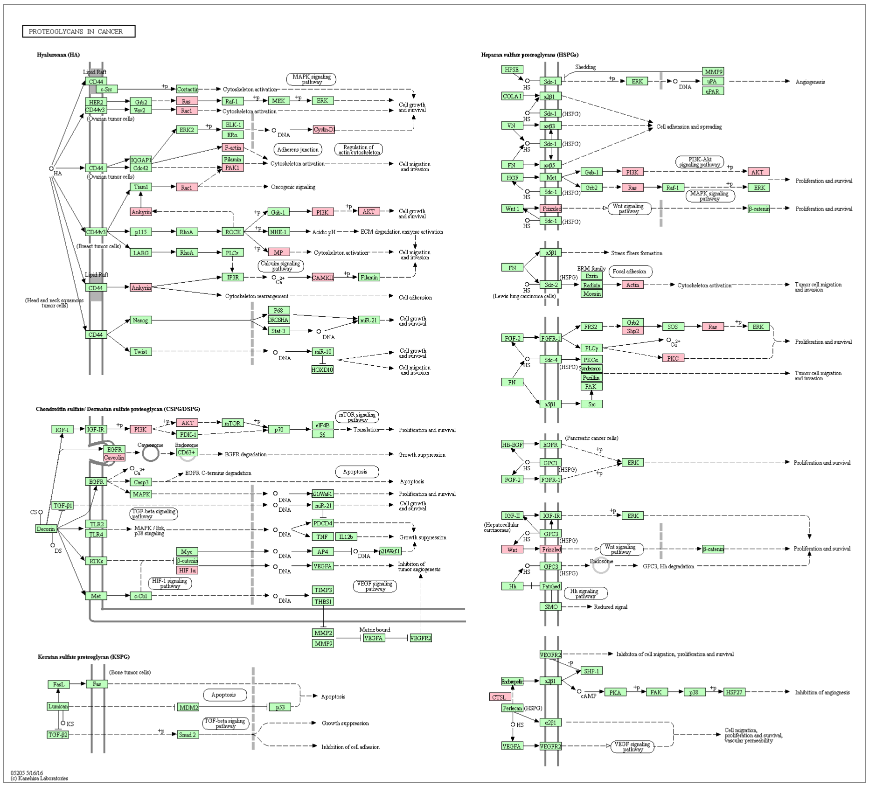

24.3 Legend:

RBH-Blast at 60% Identity + 50% Coverage
Green = Hit in *H. sapiens*
Red = Hit in *H. sapiens* and *T. californica*
White = Not in *H. sapiens*

25 Influenza A

25.1 Human Pathway: HSA05164

25.2 Number of Hits: 17

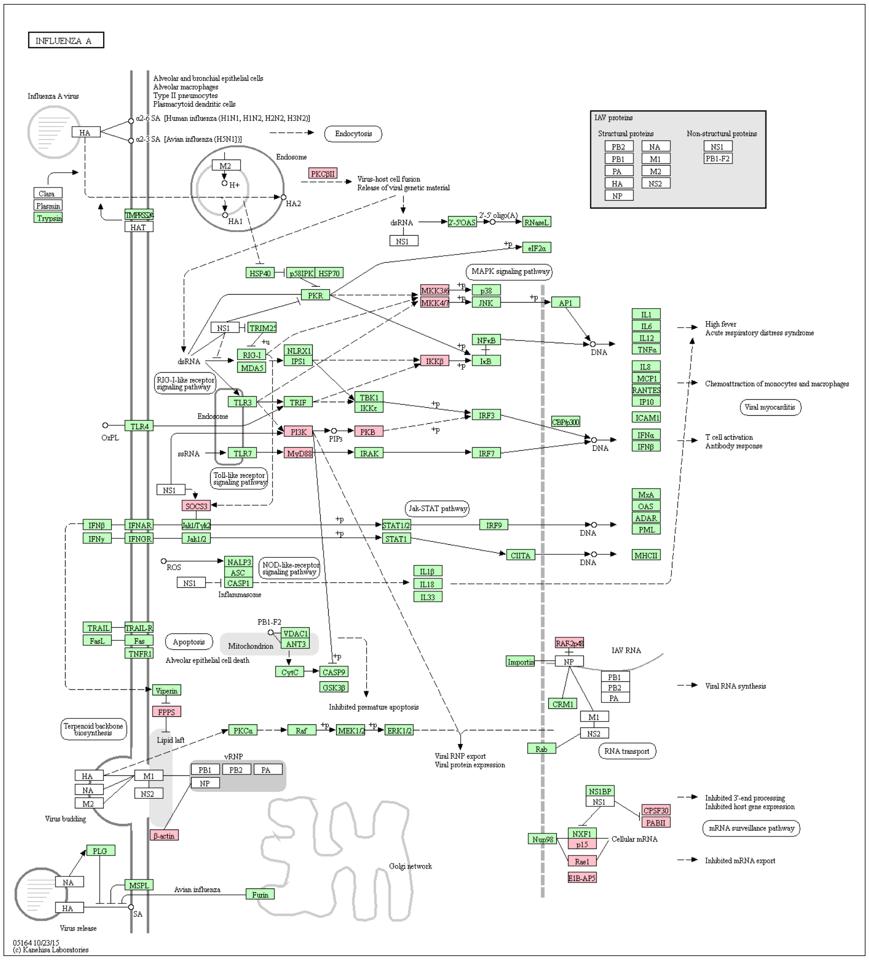

25.3 Legend:

RBH-Blast at 60% Identity + 50% Coverage
Green = Hit in *H. sapiens*
Red = Hit in *H. sapiens* and *T. californica*
White = Not in *H. sapiens*

26 Hippo signaling pathway

26.1 Human Pathway: HSA04390

26.2 Number of Hits: 17

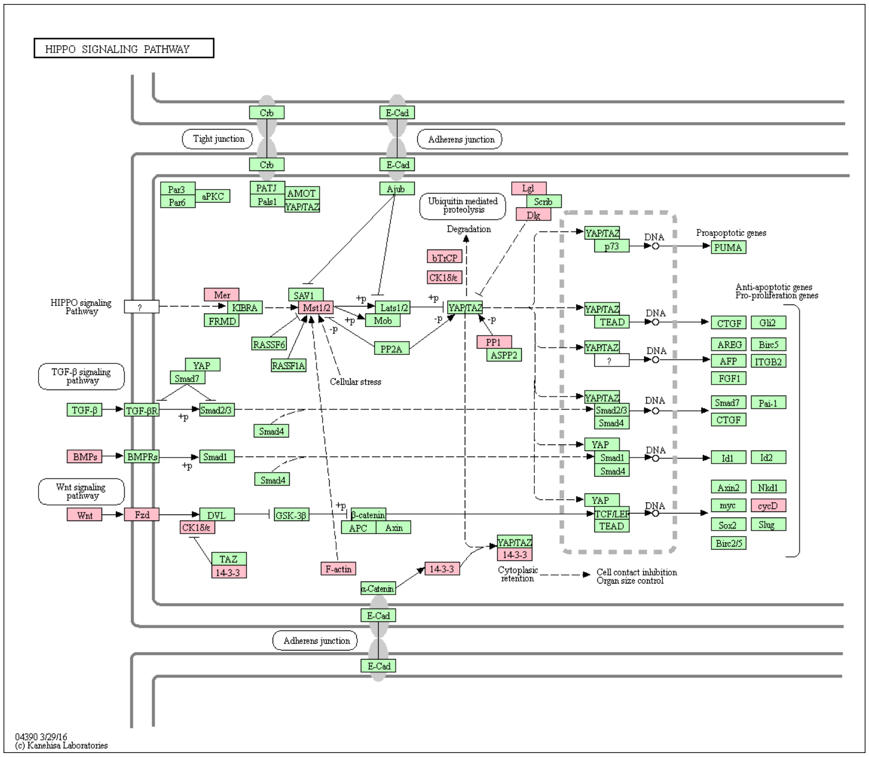

26.3 Legend:

RBH-Blast at 60% Identity + 50% Coverage
Green = Hit in *H. sapiens*
Red = Hit in *H. sapiens* and *T. californica*
White = Not in *H. sapiens*

27 Neuroactive ligand-receptor interaction

27.1 Human Pathway: HSA04080

27.2 Number of Hits: 17

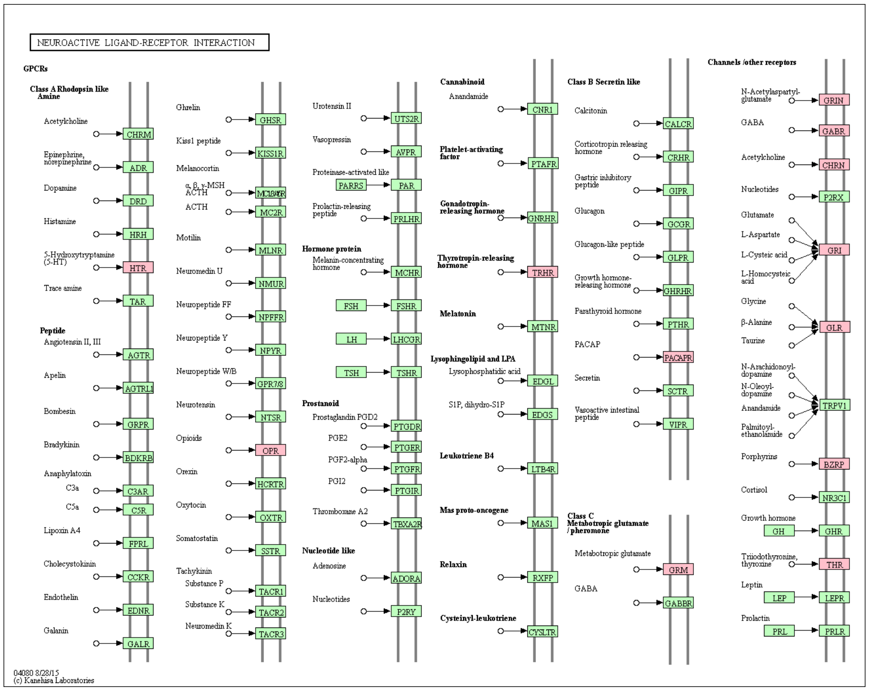

27.3 Legend:

RBH-Blast at 60% Identity + 50% Coverage
Green = Hit in *H. sapiens*
Red = Hit in *H. sapiens* and *T. californica*
White = Not in *H. sapiens*

28 Adrenergic signaling in cardiomyocytes

28.1 Human Pathway: HSA04261

28.2 Number of Hits: 16

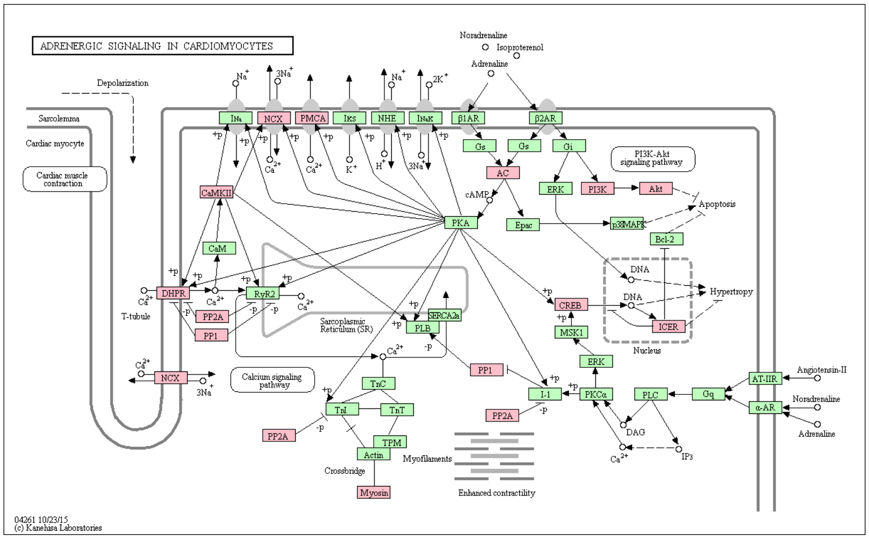

28.3 Legend:

RBH-Blast at 60% Identity + 50% Coverage
Green = Hit in *H. sapiens*
Red = Hit in *H. sapiens* and *T. californica*
White = Not in *H. sapiens*

29 Wnt signaling pathway

29.1 Human Pathway: HSA04310

29.2 Number of Hits: 16

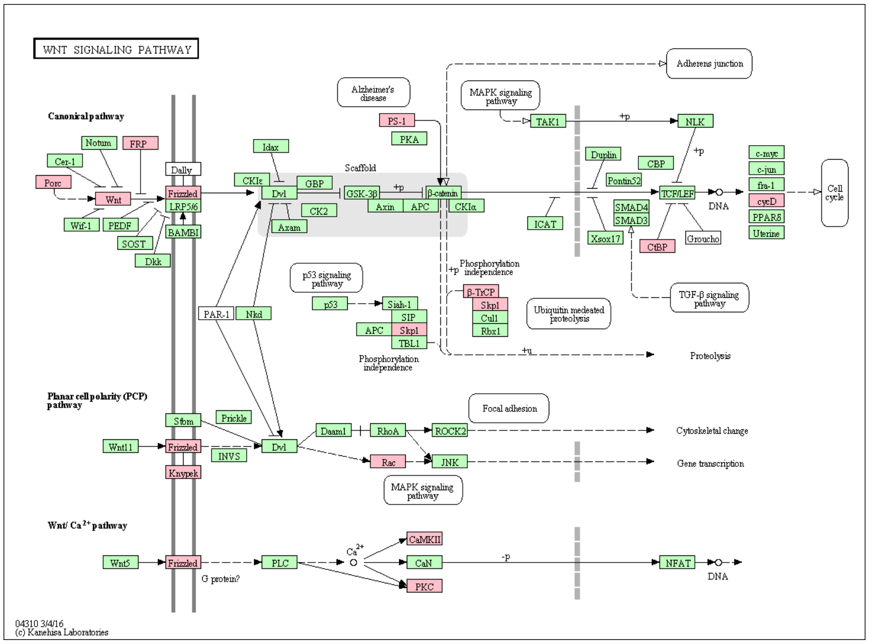

29.3 Legend:

RBH-Blast at 60% Identity + 50% Coverage
Green = Hit in *H. sapiens*
Red = Hit in *H. sapiens* and *T. californica*
White = Not in *H. sapiens*

30 Purine metabolism

30.1 Human Pathway: HSA00230

30.2 Number of Hits: 16

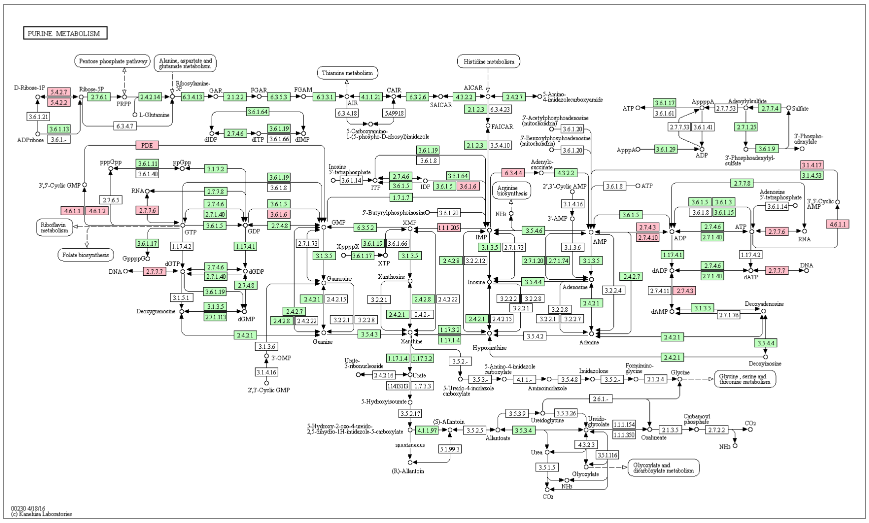

30.3 Legend:

RBH-Blast at 60% Identity + 50% Coverage
Green = Hit in *H. sapiens*
Red = Hit in *H. sapiens* and *T. californica*
White = Not in *H. sapiens*

31 Neurotrophin signaling pathway

31.1 Human Pathway: HSA04722

31.2 Number of Hits: 16

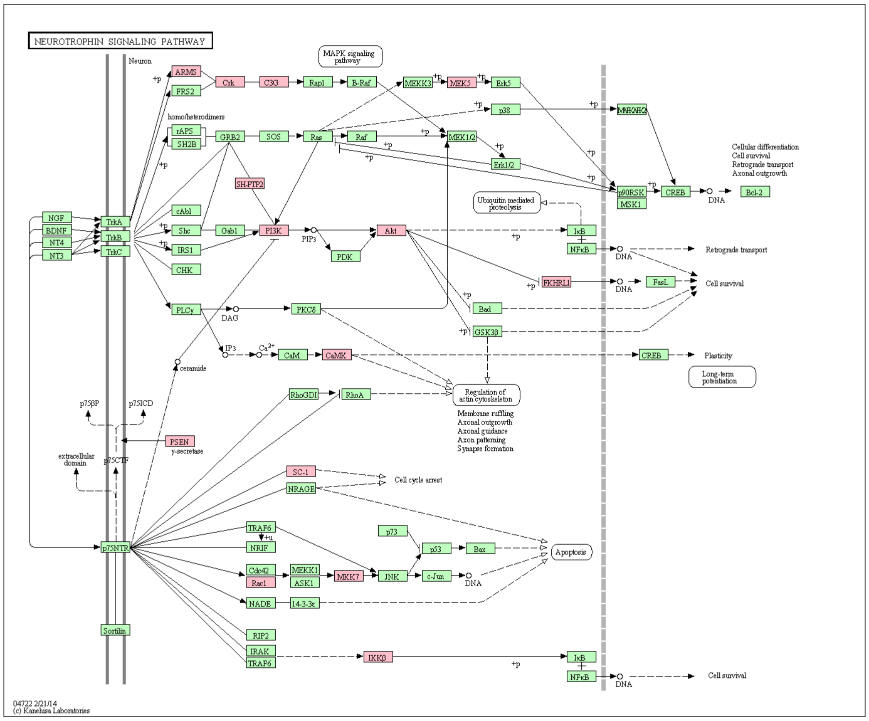

31.3 Legend:

RBH-Blast at 60% Identity + 50% Coverage
Green = Hit in *H. sapiens*
Red = Hit in *H. sapiens* and *T. californica*
White = Not in *H. sapiens*

32 Rap1 signaling pathway

32.1 Human Pathway: HSA04015

32.2 Number of Hits: 16

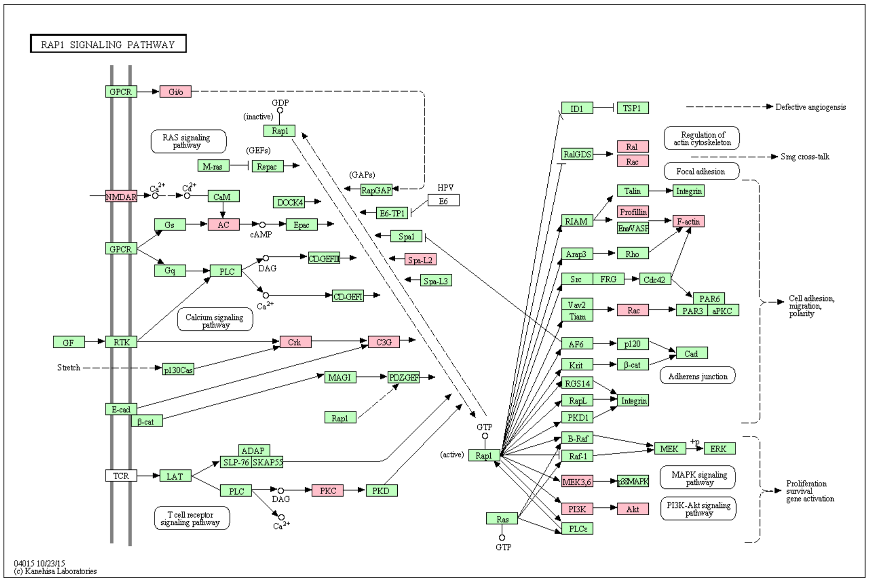

32.3 Legend:

RBH-Blast at 60% Identity + 50% Coverage
Green = Hit in *H. sapiens*
Red = Hit in *H. sapiens* and *T. californica*
White = Not in *H. sapiens*

33 Dopaminergic synapse

33.1 Human Pathway: HSA04728

33.2 Number of Hits: 16

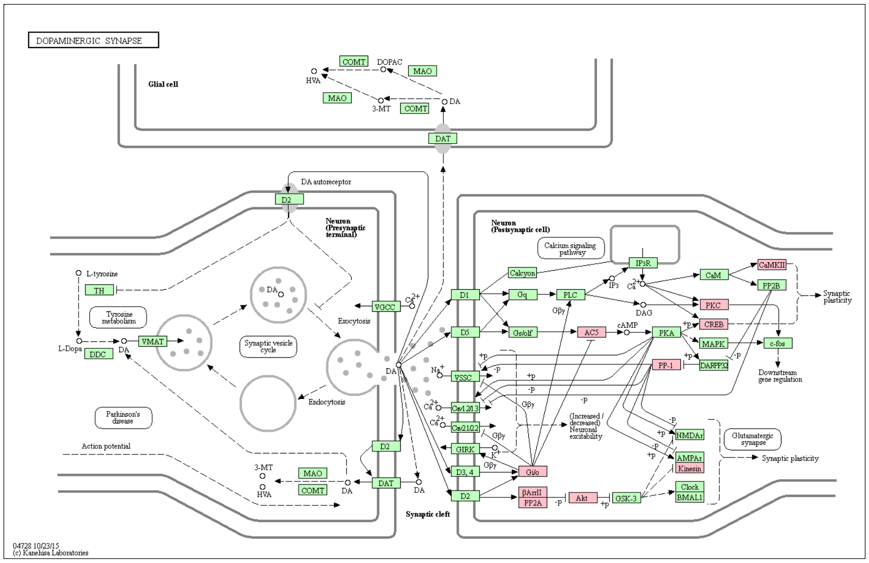

33.3 Legend:

RBH-Blast at 60% Identity + 50% Coverage
Green = Hit in *H. sapiens*
Red = Hit in *H. sapiens* and *T. californica*
White = Not in *H. sapiens*

34 Longevity regulating pathway

34.1 Human Pathway: HSA04211

34.2 Number of Hits: 15

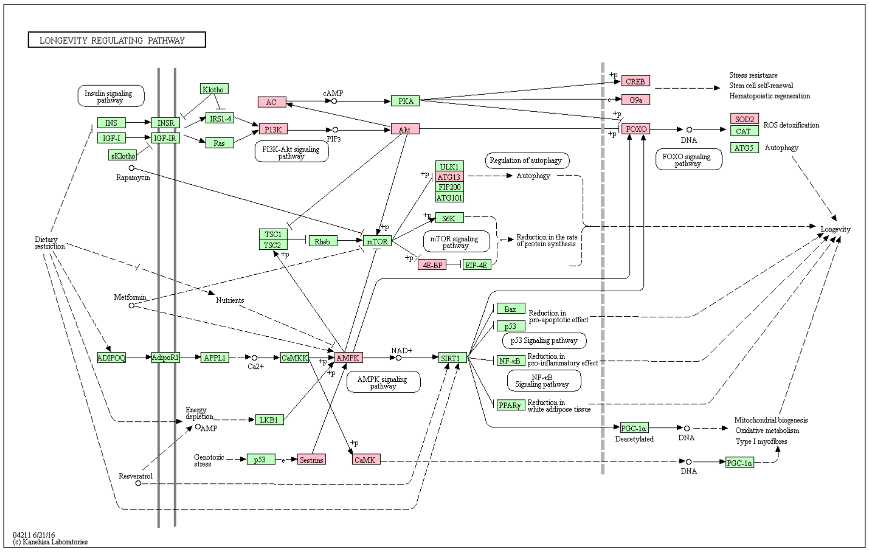

34.3 Legend:

RBH-Blast at 60% Identity + 50% Coverage
Green = Hit in *H. sapiens*
Red = Hit in *H. sapiens* and *T. californica*
White = Not in *H. sapiens*

35 Parkinson's disease

35.1 Human Pathway: HSA05012

35.2 Number of Hits: 15

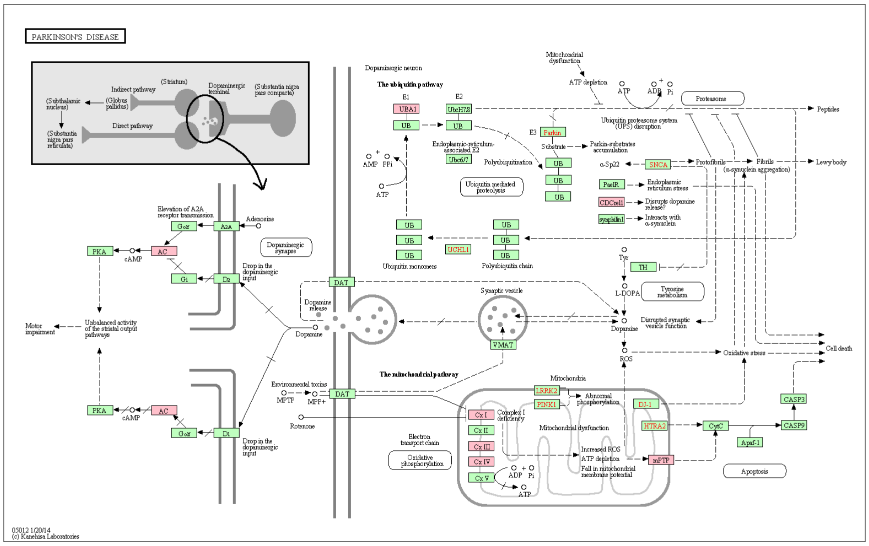

35.3 Legend:

RBH-Blast at 60% Identity + 50% Coverage
Green = Hit in *H. sapiens*
Red = Hit in *H. sapiens* and *T. californica*
White = Not in *H. sapiens*

36 mRNA surveillance pathway

36.1 Human Pathway: HSA03015

36.2 Number of Hits: 15

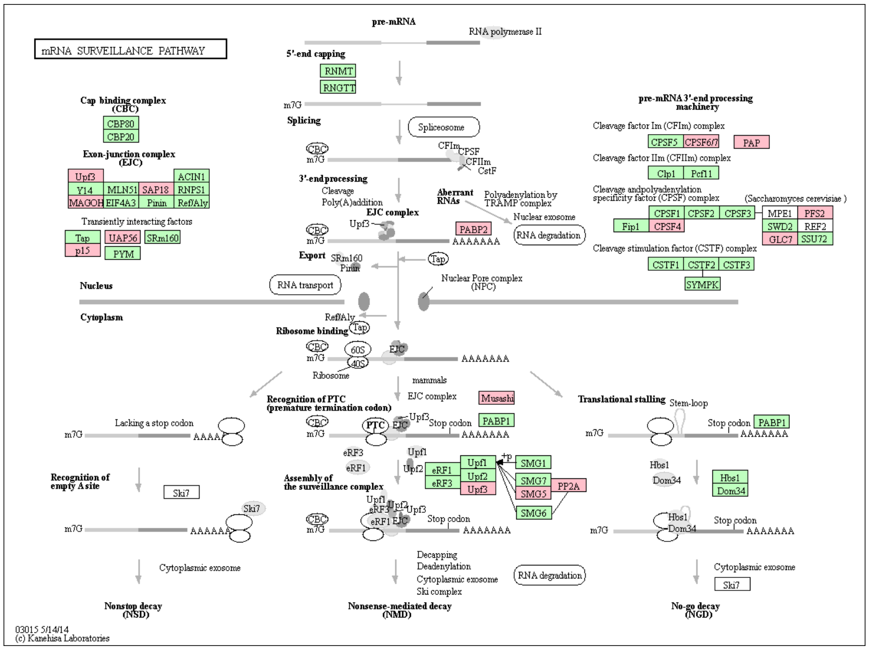

36.3 Legend:

RBH-Blast at 60% Identity + 50% Coverage
Green = Hit in *H. sapiens*
Red = Hit in *H. sapiens* and *T. californica*
White = Not in *H. sapiens*

37 Cholinergic synapse

37.1 Human Pathway: HSA04725

37.2 Number of Hits: 15

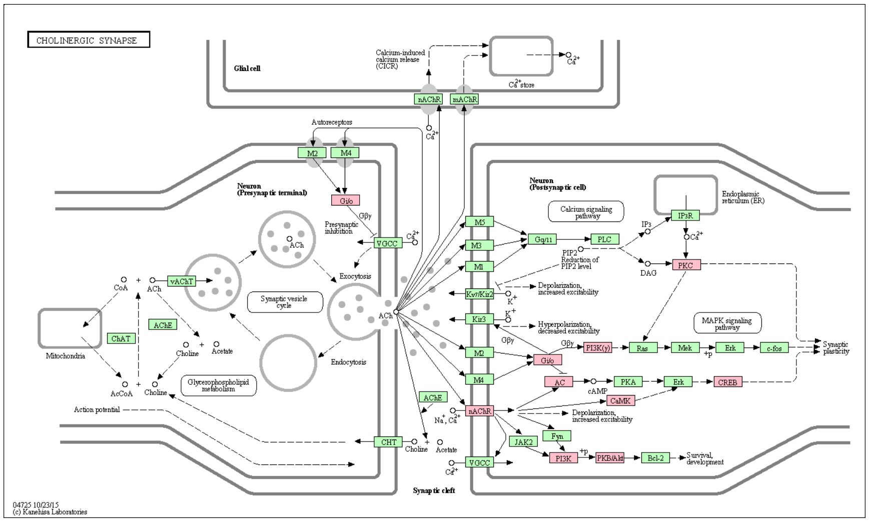

37.3 Legend:

RBH-Blast at 60% Identity + 50% Coverage
Green = Hit in *H. sapiens*
Red = Hit in *H. sapiens* and *T. californica*
White = Not in *H. sapiens*

38 Glutamatergic synapse

38.1 Human Pathway: HSA04724

38.2 Number of Hits: 15

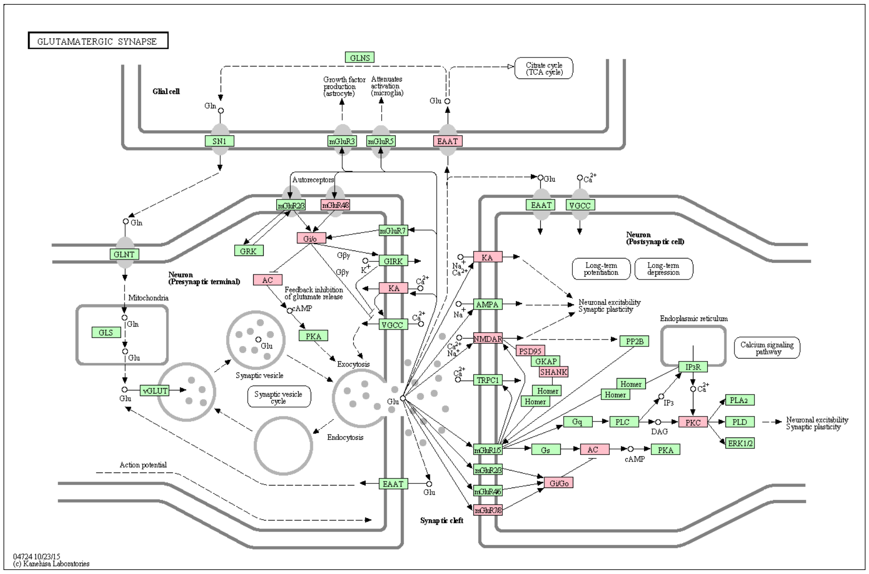

38.3 Legend:

RBH-Blast at 60% Identity + 50% Coverage
Green = Hit in *H. sapiens*
Red = Hit in *H. sapiens* and *T. californica*
White = Not in *H. sapiens*

39 Oxidative phosphorylation

39.1 Human Pathway: HSA00190

39.2 Number of Hits: 14

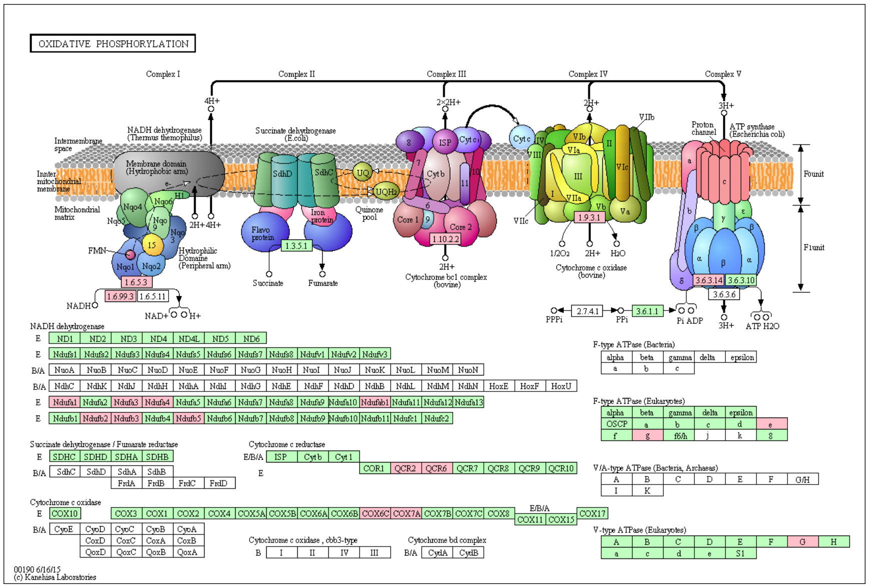

39.3 Legend:

RBH-Blast at 60% Identity + 50% Coverage
Green = Hit in *H. sapiens*
Red = Hit in *H. sapiens* and *T. californica*
White = Not in *H. sapiens*

40 FoxO signaling pathway

40.1 Human Pathway: HSA04068

40.2 Number of Hits: 14

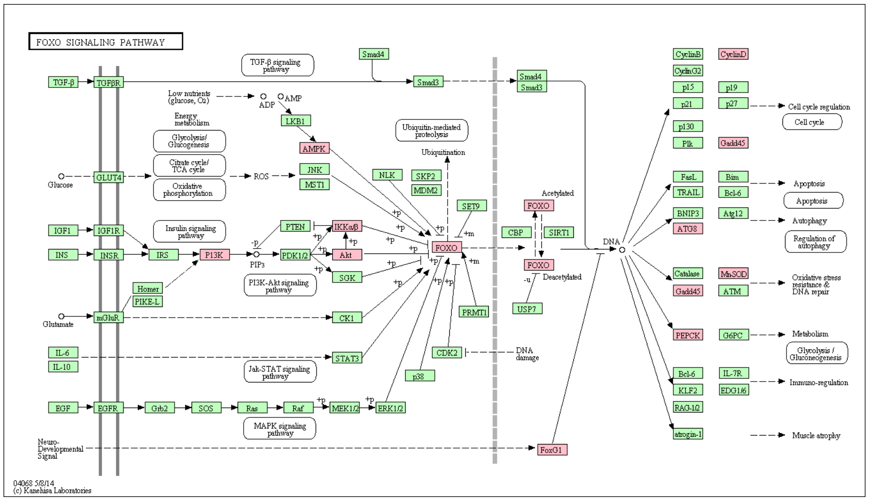

40.3 Legend:

RBH-Blast at 60% Identity + 50% Coverage
Green = Hit in *H. sapiens*
Red = Hit in *H. sapiens* and *T. californica*
White = Not in *H. sapiens*

41 Thyroid hormone signaling pathway

41.1 Human Pathway: HSA04919

41.2 Number of Hits: 14

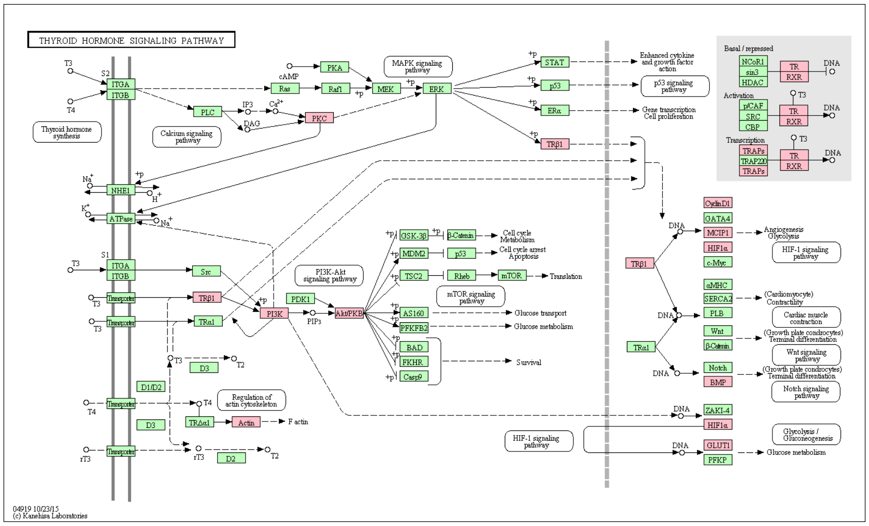

41.3 Legend:

RBH-Blast at 60% Identity + 50% Coverage
Green = Hit in *H. sapiens*
Red = Hit in *H. sapiens* and *T. californica*
White = Not in *H. sapiens*

42 Morphine addiction

42.1 Human Pathway: HSA05032

42.2 Number of Hits: 14

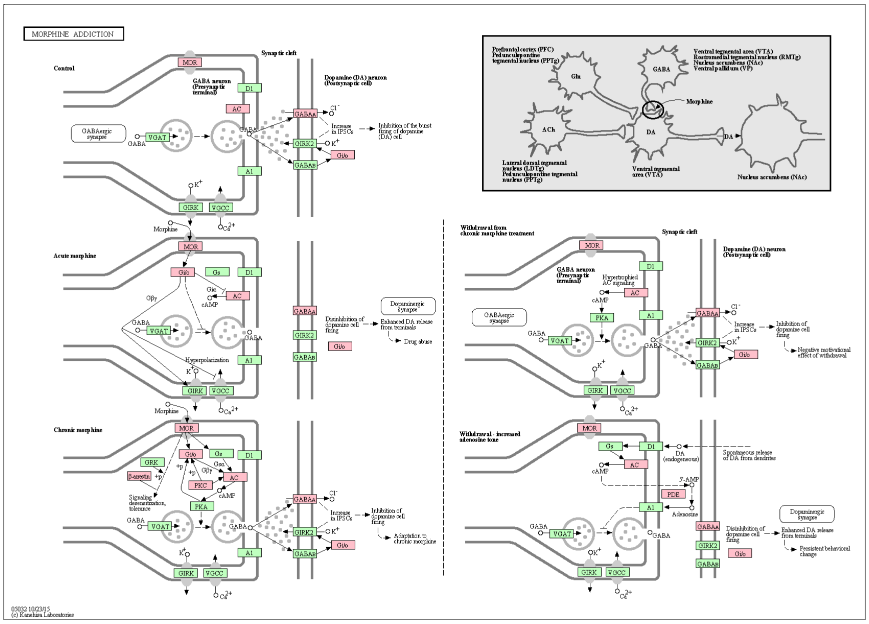

42.3 Legend:

RBH-Blast at 60% Identity + 50% Coverage
Green = Hit in *H. sapiens*
Red = Hit in *H. sapiens* and *T. californica*
White = Not in *H. sapiens*

43 GABAergic synapse

43.1 Human Pathway: HSA04727

43.2 Number of Hits: 14

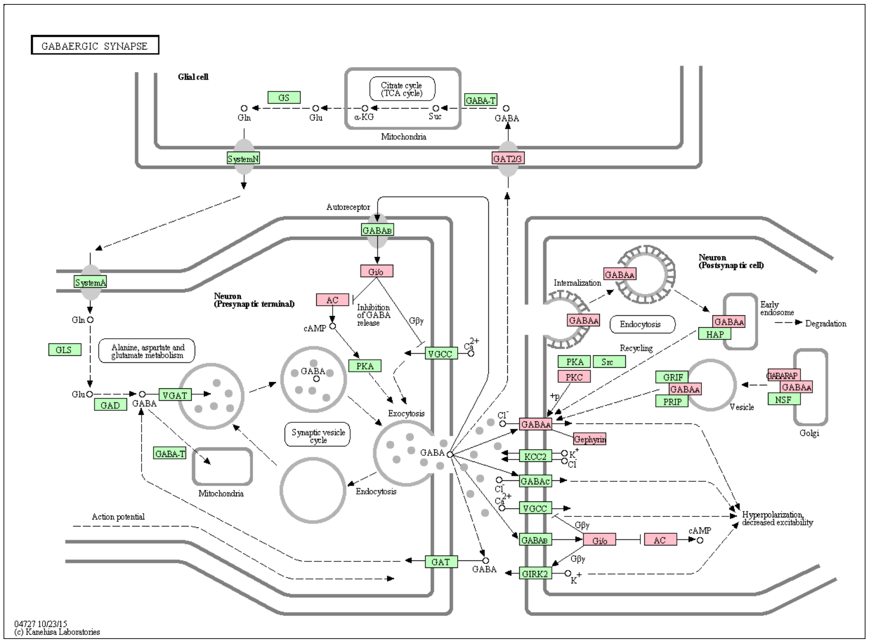

43.3 Legend:

RBH-Blast at 60% Identity + 50% Coverage
Green = Hit in *H. sapiens*
Red = Hit in *H. sapiens* and *T. californica*
White = Not in *H. sapiens*

44 Apoptosis

44.1 Human Pathway: HSA04210

44.2 Number of Hits: 14

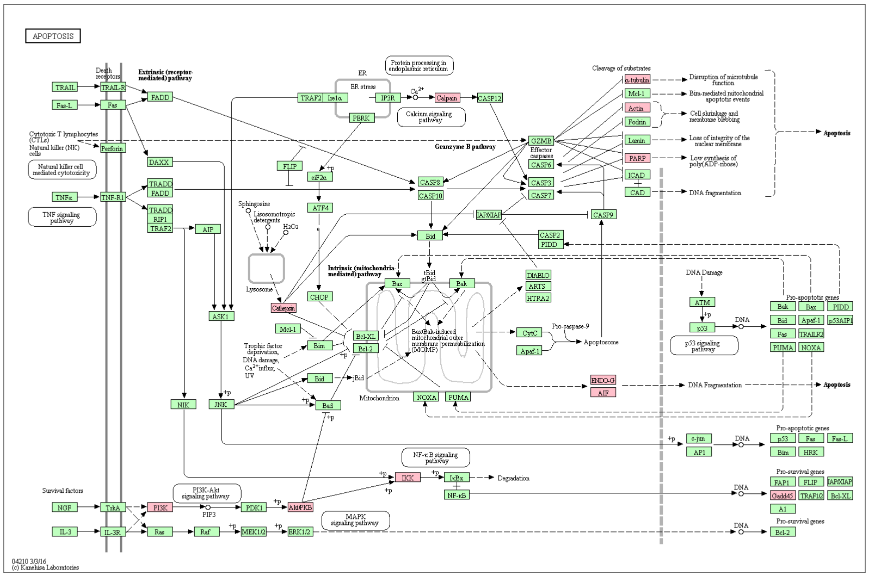

44.3 Legend:

RBH-Blast at 60% Identity + 50% Coverage
Green = Hit in *H. sapiens*
Red = Hit in *H. sapiens* and *T. californica*
White = Not in *H. sapiens*

45 Epstein-Barr virus infection

45.1 Human Pathway: HSA05169

45.2 Number of Hits: 14

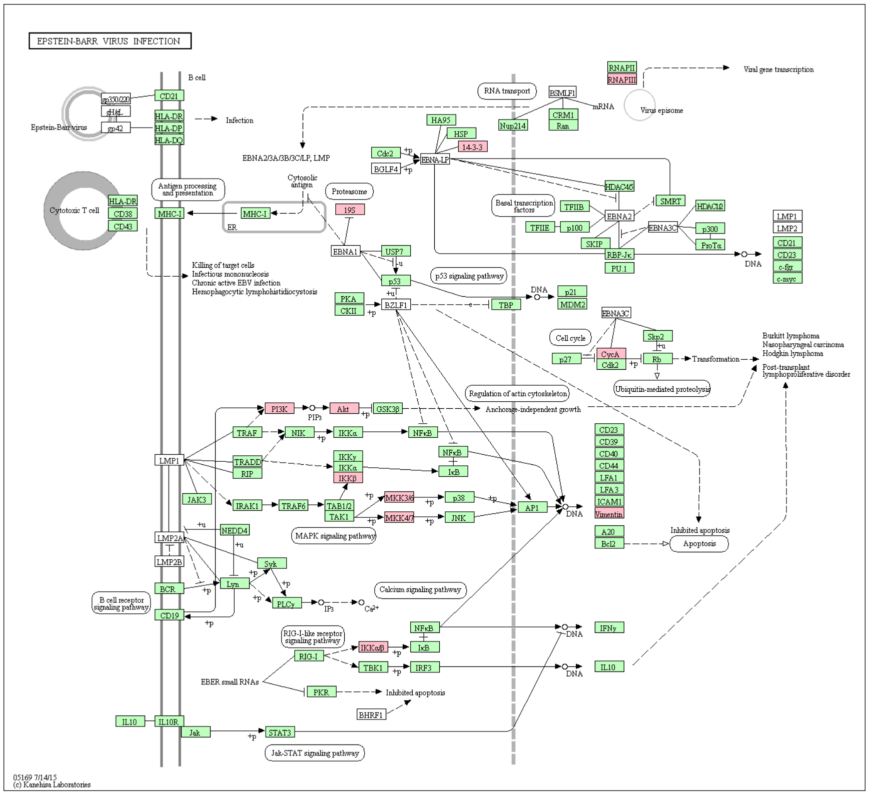

45.3 Legend:

RBH-Blast at 60% Identity + 50% Coverage
Green = Hit in *H. sapiens*
Red = Hit in *H. sapiens* and *T. californica*
White = Not in *H. sapiens*

46 Renal cell carcinoma

46.1 Human Pathway: HSA05211

46.2 Number of Hits: 14

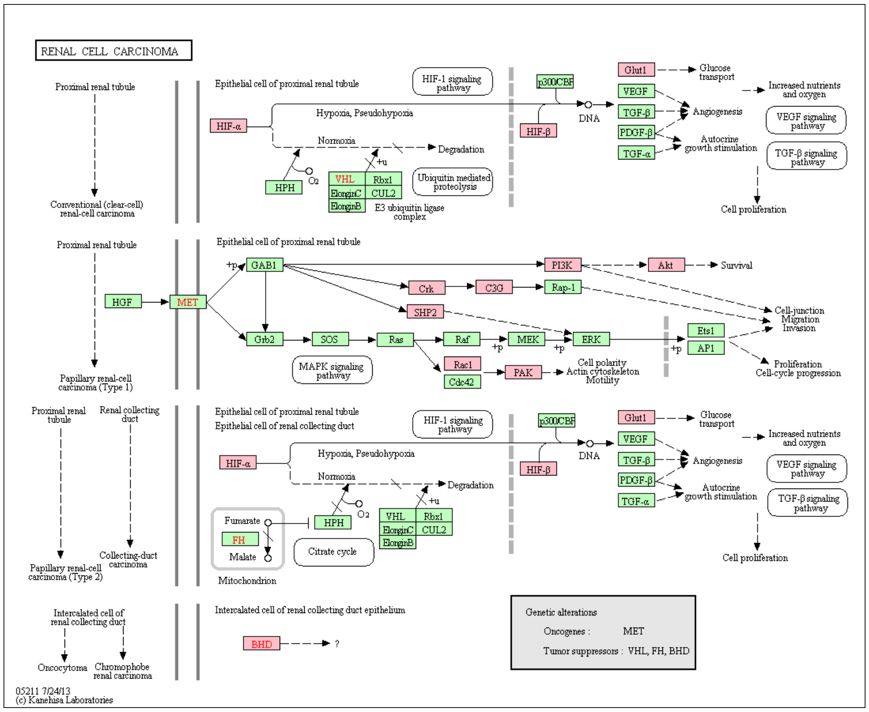

46.3 Legend:

RBH-Blast at 60% Identity + 50% Coverage
Green = Hit in *H. sapiens*
Red = Hit in *H. sapiens* and *T. californica*
White = Not in *H. sapiens*

47 Cell cycle

47.1 Human Pathway: HSA04110

47.2 Number of Hits: 14

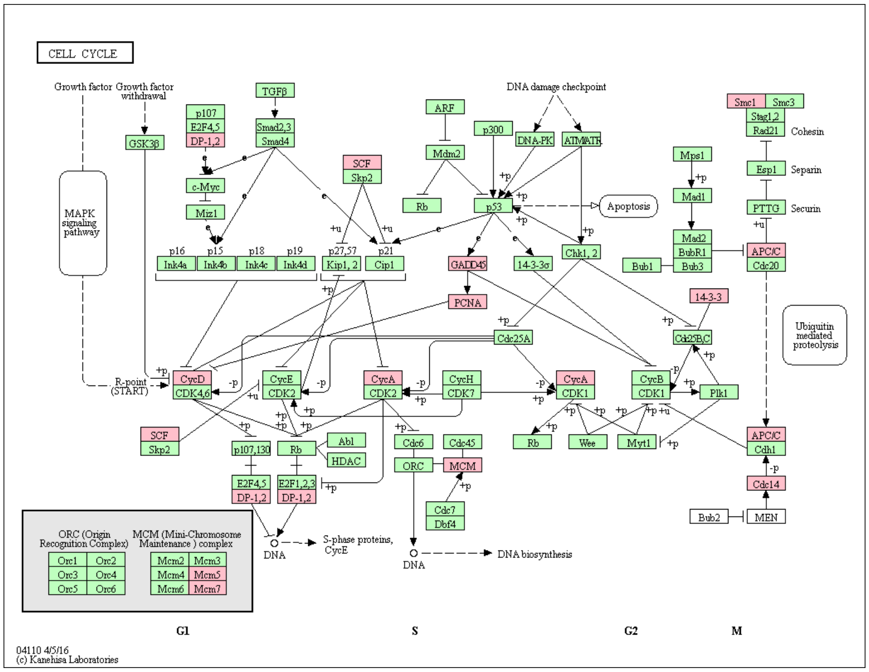

47.3 Legend:

RBH-Blast at 60% Identity + 50% Coverage
Green = Hit in *H. sapiens*
Red = Hit in *H. sapiens* and *T. californica*
White = Not in *H. sapiens*

48 Circadian entrainment

48.1 Human Pathway: HSA04713

48.2 Number of Hits: 14

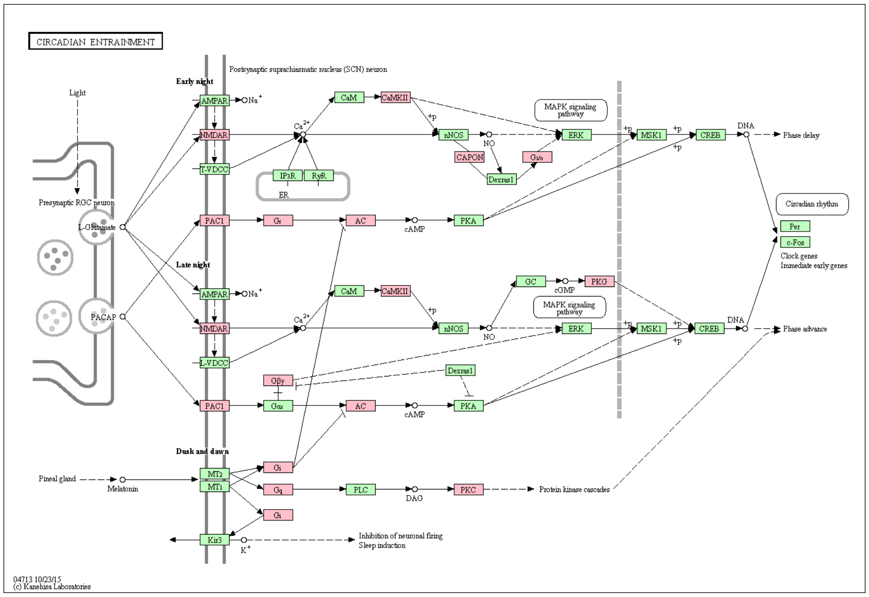

48.3 Legend:

RBH-Blast at 60% Identity + 50% Coverage
Green = Hit in *H. sapiens*
Red = Hit in *H. sapiens* and *T. californica*
White = Not in *H. sapiens*

49 Bacterial invasion of epithelial cells

49.1 Human Pathway: HSA05100

49.2 Number of Hits: 13

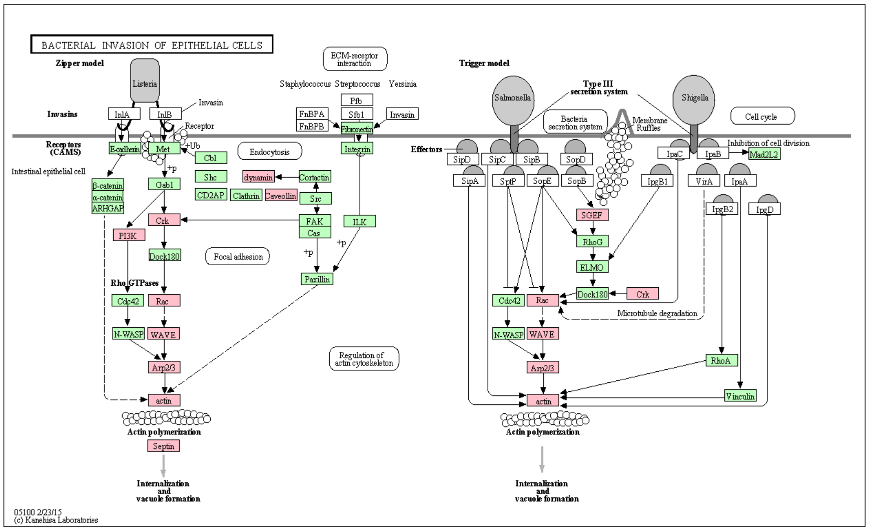

49.3 Legend:

RBH-Blast at 60% Identity + 50% Coverage
Green = Hit in *H. sapiens*
Red = Hit in *H. sapiens* and *T. californica*
White = Not in *H. sapiens*

50 Calcium signaling pathway

50.1 Human Pathway: HSA04020

50.2 Number of Hits: 13

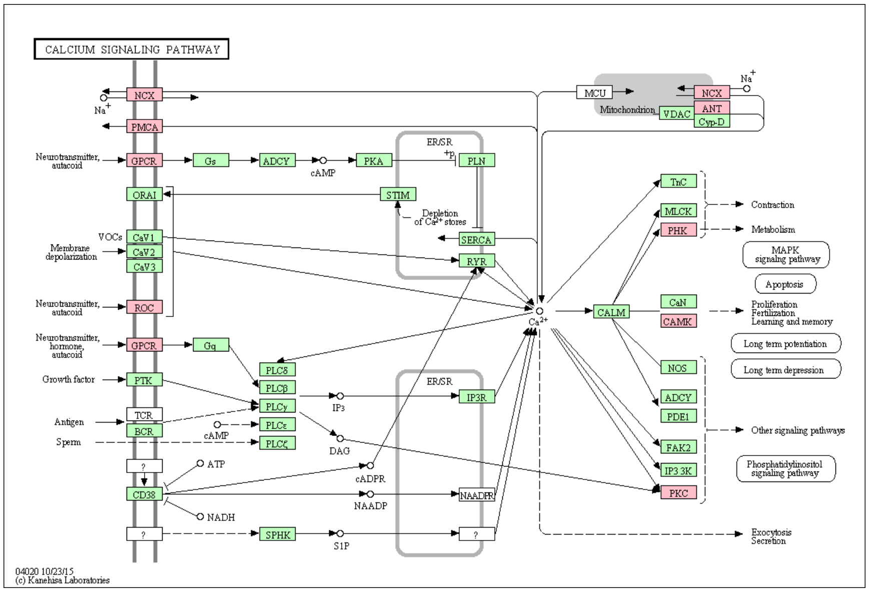

50.3 Legend:

RBH-Blast at 60% Identity + 50% Coverage
Green = Hit in *H. sapiens*
Red = Hit in *H. sapiens* and *T. californica*
White = Not in *H. sapiens*

51 Alcoholism

51.1 Human Pathway: HSA05034

51.2 Number of Hits: 13

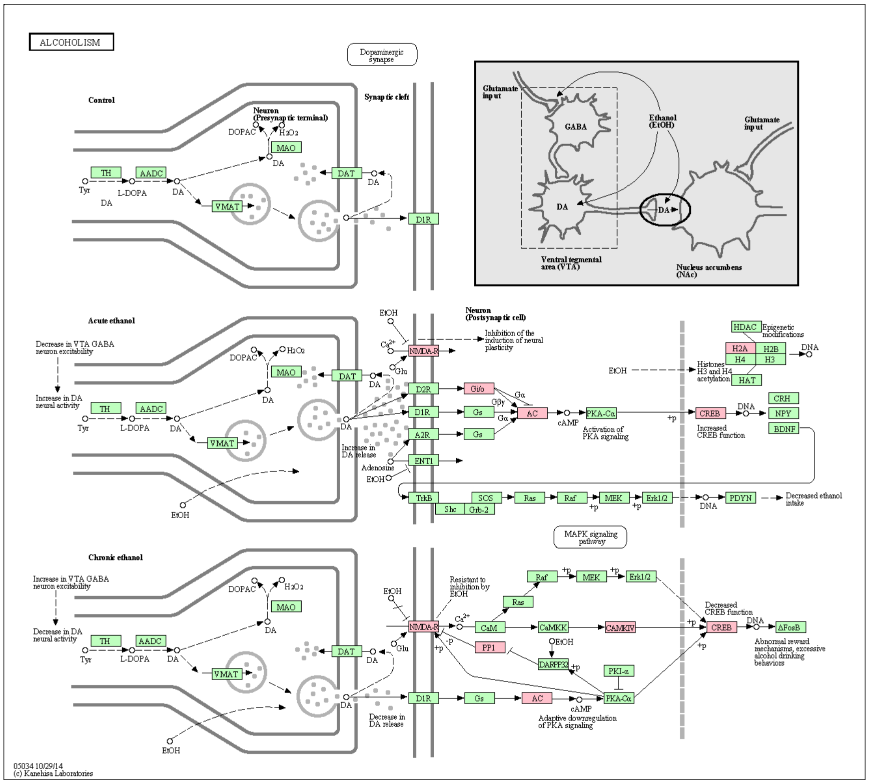

51.3 Legend:

RBH-Blast at 60% Identity + 50% Coverage
Green = Hit in *H. sapiens*
Red = Hit in *H. sapiens* and *T. californica*
White = Not in *H. sapiens*

52 Viral carcinogenesis

52.1 Human Pathway: HSA05203

52.2 Number of Hits: 13

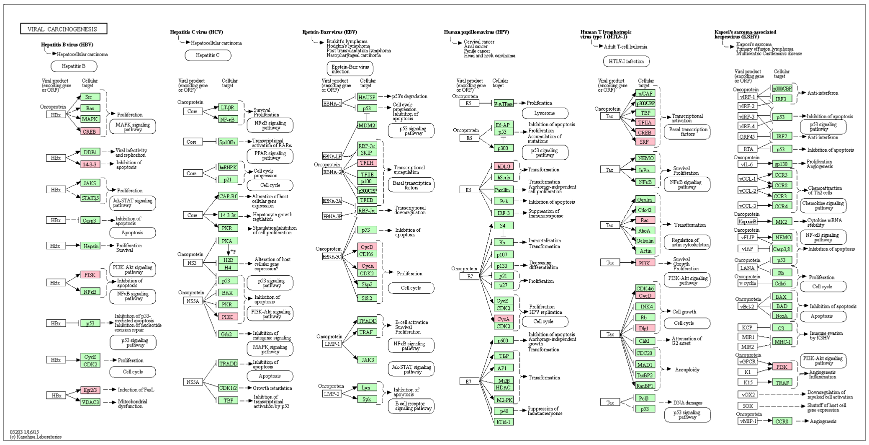

52.3 Legend:

RBH-Blast at 60% Identity + 50% Coverage
Green = Hit in *H. sapiens*
Red = Hit in *H. sapiens* and *T. californica*
White = Not in *H. sapiens*

53 Phagosome

53.1 Human Pathway: HSA04145

53.2 Number of Hits: 12

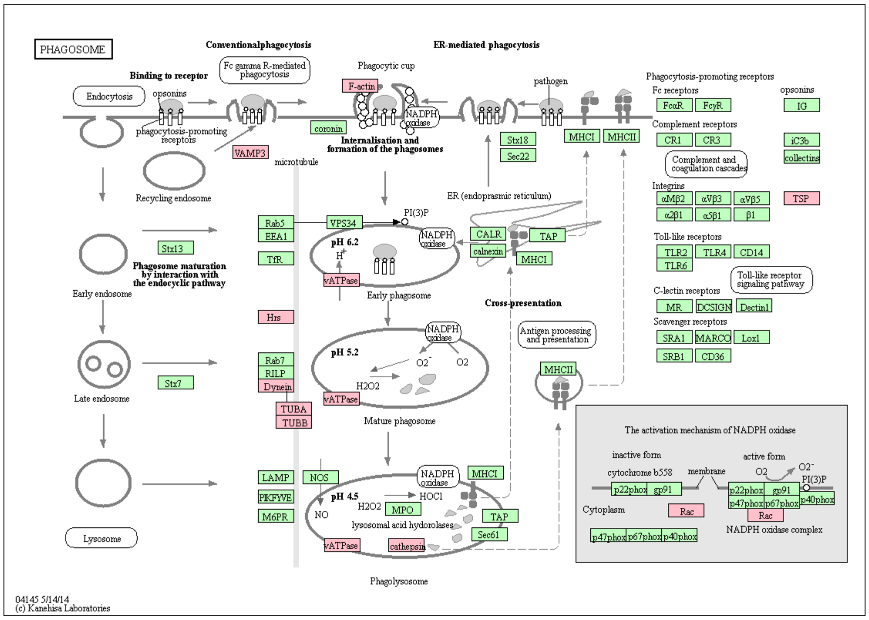

53.3 Legend:

RBH-Blast at 60% Identity + 50% Coverage
Green = Hit in *H. sapiens*
Red = Hit in *H. sapiens* and *T. californica*
White = Not in *H. sapiens*

54 Tight junction

54.1 Human Pathway: HSA04530

54.2 Number of Hits: 12

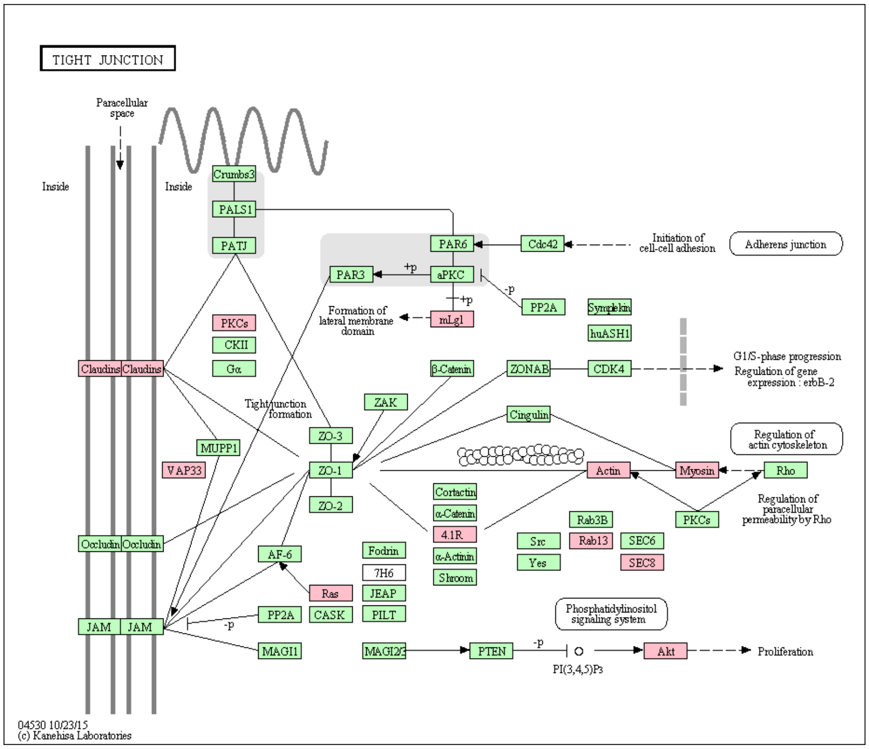

54.3 Legend:

RBH-Blast at 60% Identity + 50% Coverage
Green = Hit in *H. sapiens*
Red = Hit in *H. sapiens* and *T. californica*
White = Not in *H. sapiens*

55 Phospholipase D signaling pathway

55.1 Human Pathway: HSA04072

55.2 Number of Hits: 12

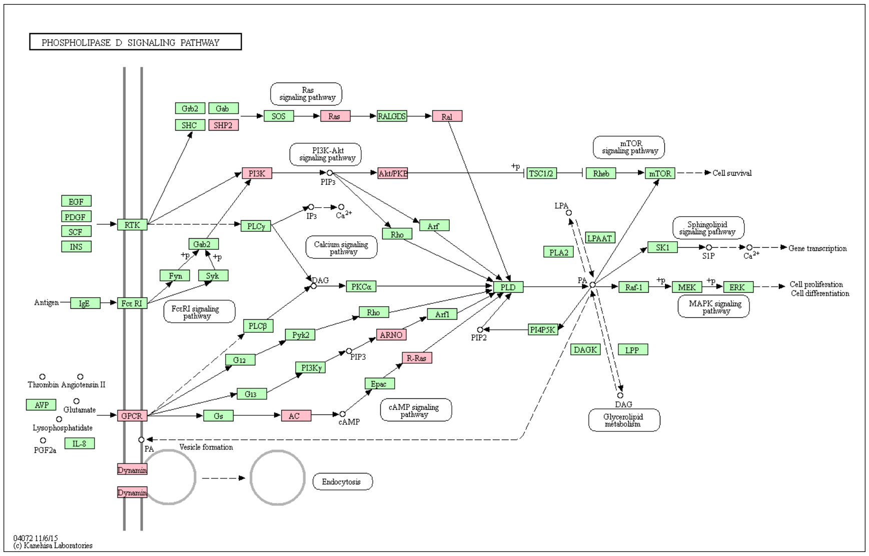

55.3 Legend:

RBH-Blast at 60% Identity + 50% Coverage
Green = Hit in *H. sapiens*
Red = Hit in *H. sapiens* and *T. californica*
White = Not in *H. sapiens*

56 mTOR signaling pathway

56.1 Human Pathway: HSA04150

56.2 Number of Hits: 12

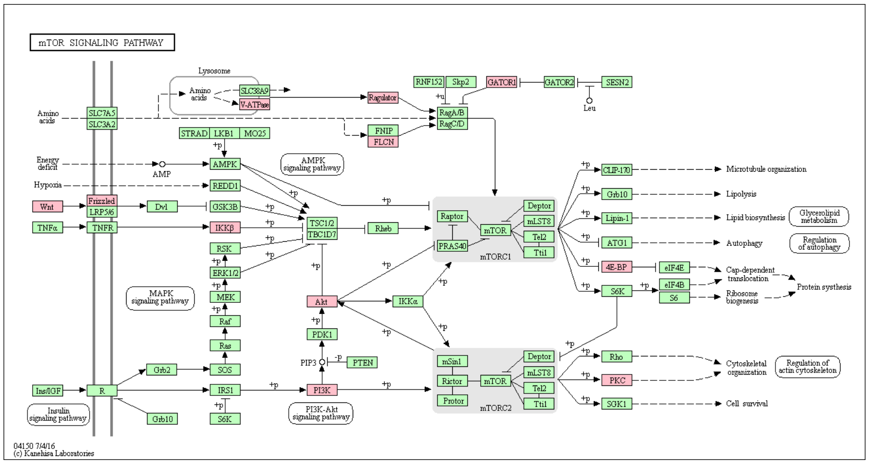

56.3 Legend:

RBH-Blast at 60% Identity + 50% Coverage
Green = Hit in *H. sapiens*
Red = Hit in *H. sapiens* and *T. californica*
White = Not in *H. sapiens*

57 Valine, leucine and isoleucine degradation

57.1 Human Pathway: HSA00280

57.2 Number of Hits: 12

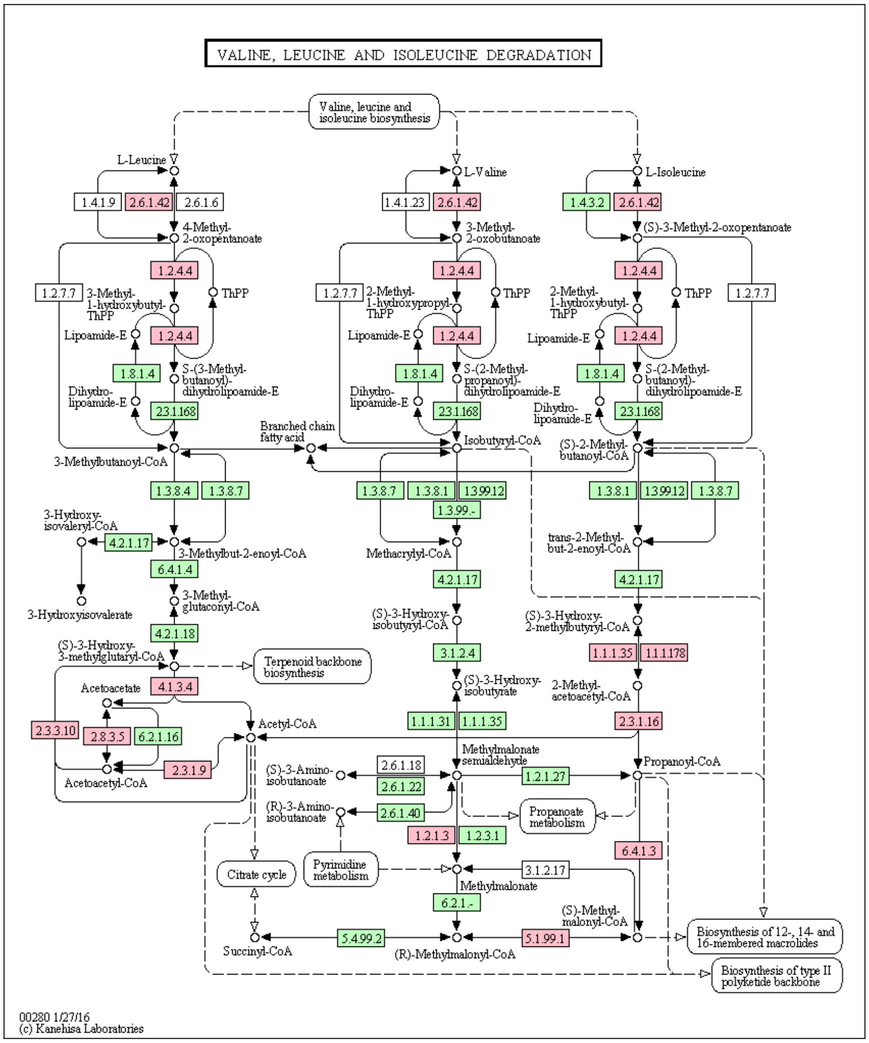

57.3 Legend:

RBH-Blast at 60% Identity + 50% Coverage
Green = Hit in *H. sapiens*
Red = Hit in *H. sapiens* and *T. californica*
White = Not in *H. sapiens*

---

58 Fc gamma R-mediated phagocytosis

58.1 Human Pathway: HSA04666

58.2 Number of Hits: 12

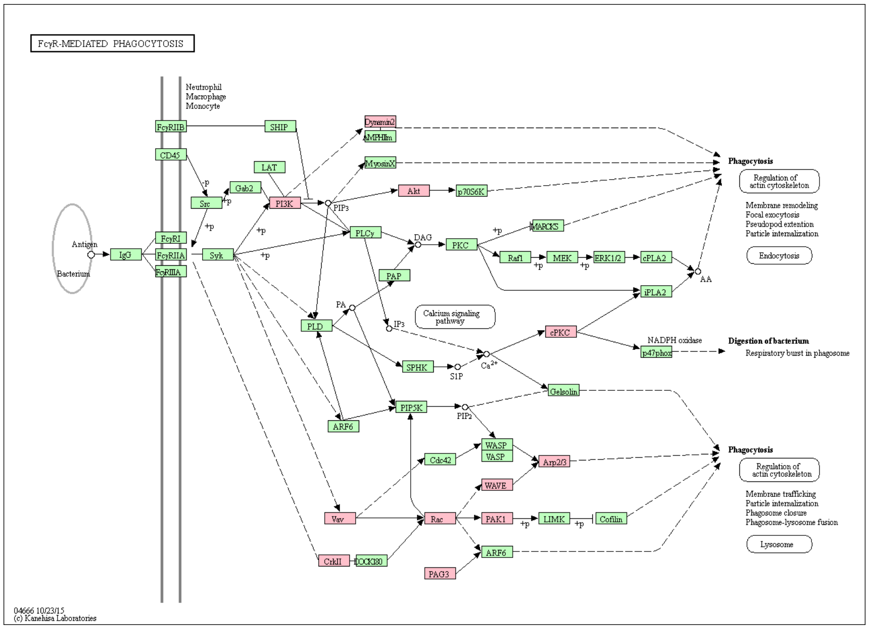

58.3 Legend:

RBH-Blast at 60% Identity + 50% Coverage
Green = Hit in *H. sapiens*
Red = Hit in *H. sapiens* and *T. californica*
White = Not in *H. sapiens*

---

59 Protein processing in endoplasmic reticulum

59.1 Human Pathway: HSA04141

59.2 Number of Hits: 12

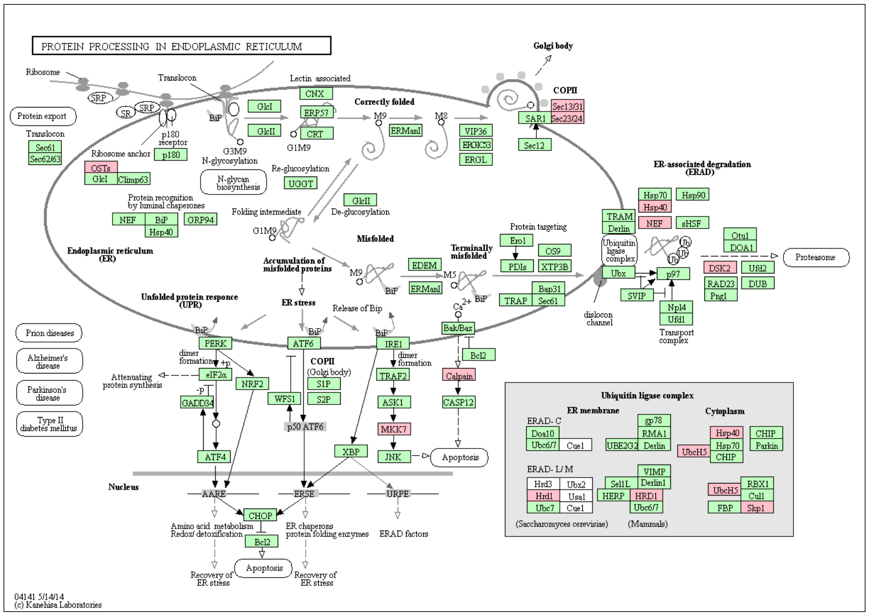

59.3 Legend:

RBH-Blast at 60% Identity + 50% Coverage
Green = Hit in *H. sapiens*
Red = Hit in *H. sapiens* and *T. californica*
White = Not in *H. sapiens*

60 Shigellosis

60.1 Human Pathway: HSA05131

60.2 Number of Hits: 11

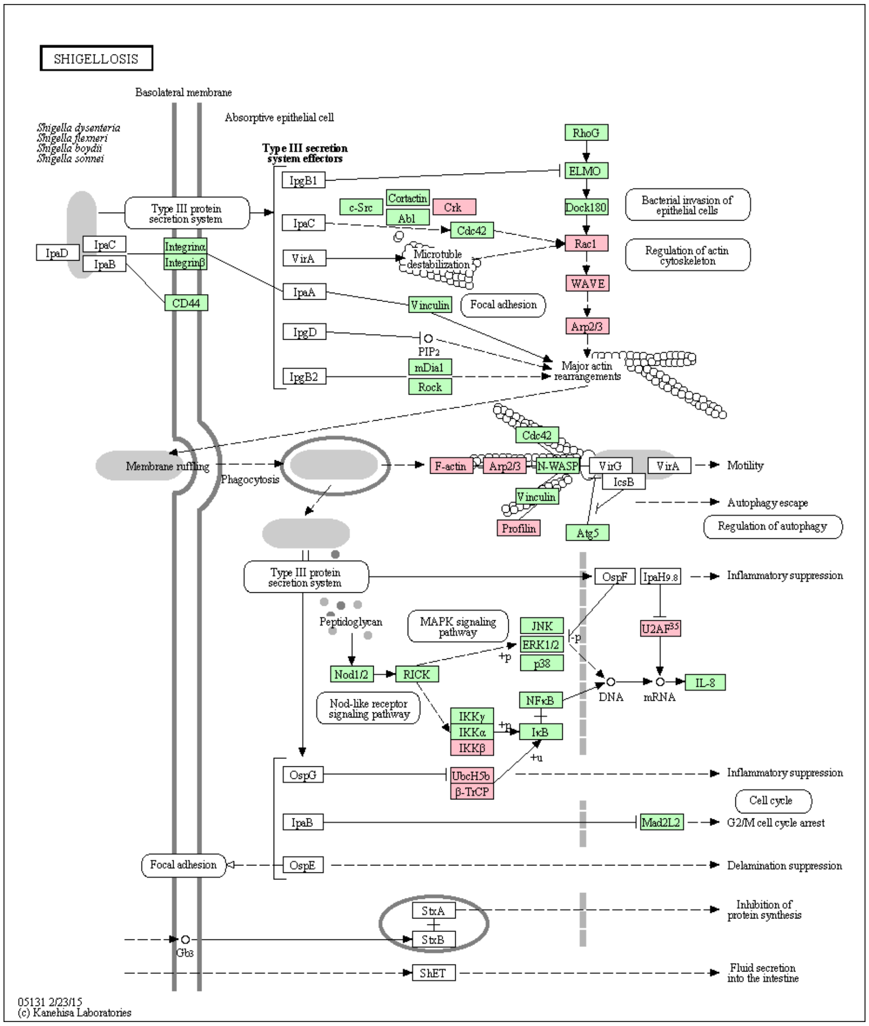

60.3 Legend:

RBH-Blast at 60% Identity + 50% Coverage
Green = Hit in *H. sapiens*
Red = Hit in *H. sapiens* and *T. californica*
White = Not in *H. sapiens*

61 Sphingolipid signaling pathway

61.1 Human Pathway: HSA04071

61.2 Number of Hits: 11

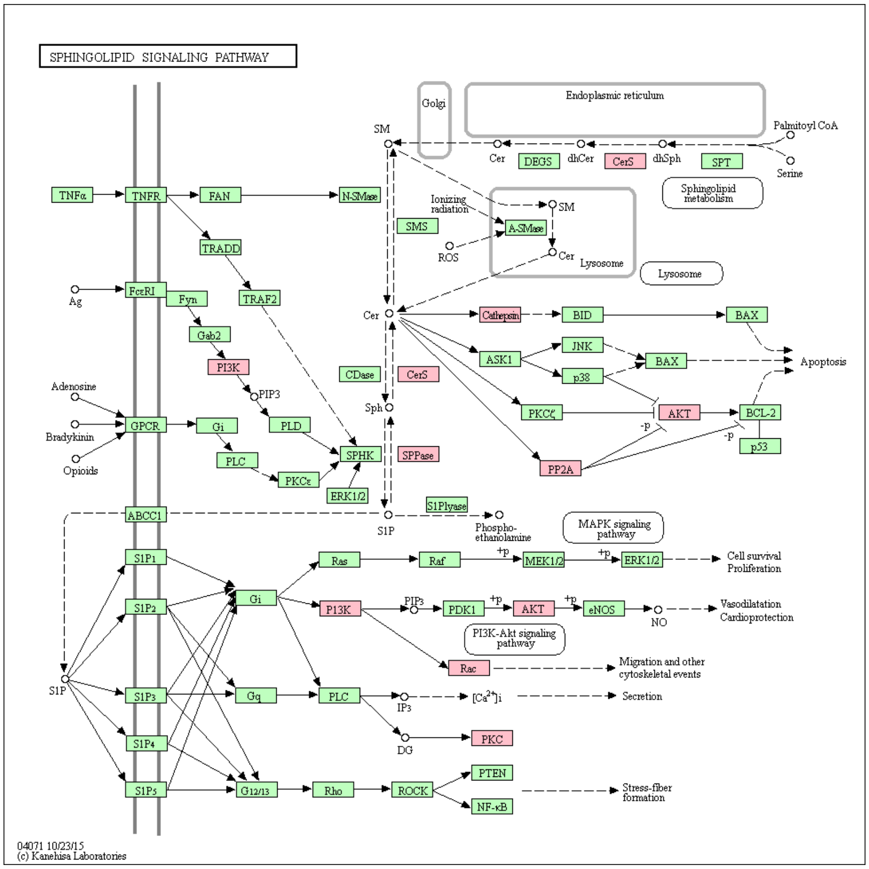

61.3 Legend:

RBH-Blast at 60% Identity + 50% Coverage
Green = Hit in *H. sapiens*
Red = Hit in *H. sapiens* and *T. californica*
White = Not in *H. sapiens*

62 Herpes simplex infection

62.1 Human Pathway: HSA05168

62.2 Number of Hits: 11

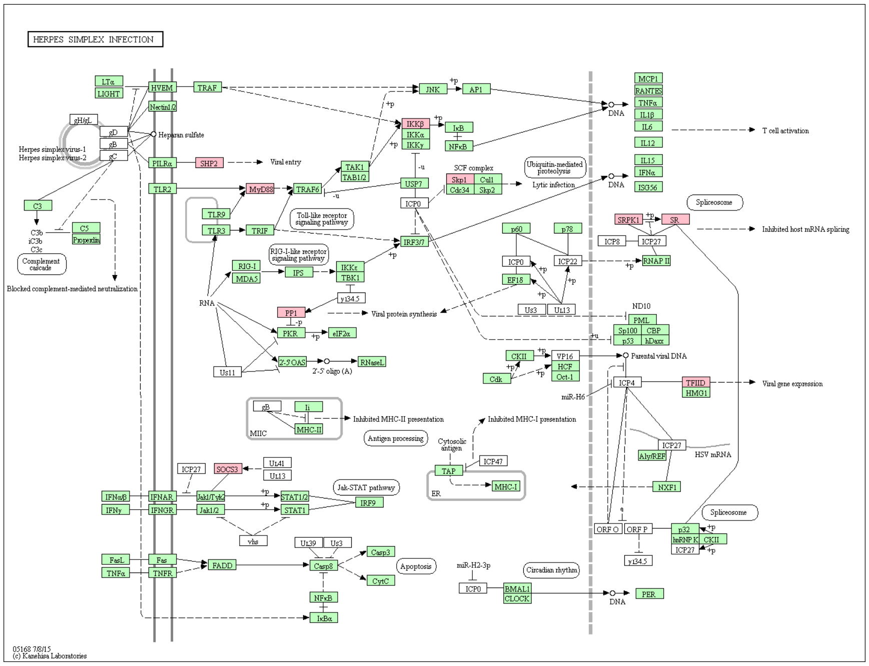

62.3 Legend:

RBH-Blast at 60% Identity + 50% Coverage
Green = Hit in *H. sapiens*
Red = Hit in *H. sapiens* and *T. californica*
White = Not in *H. sapiens*

63 Pyrimidine metabolism

63.1 Human Pathway: HSA00240

63.2 Number of Hits: 11

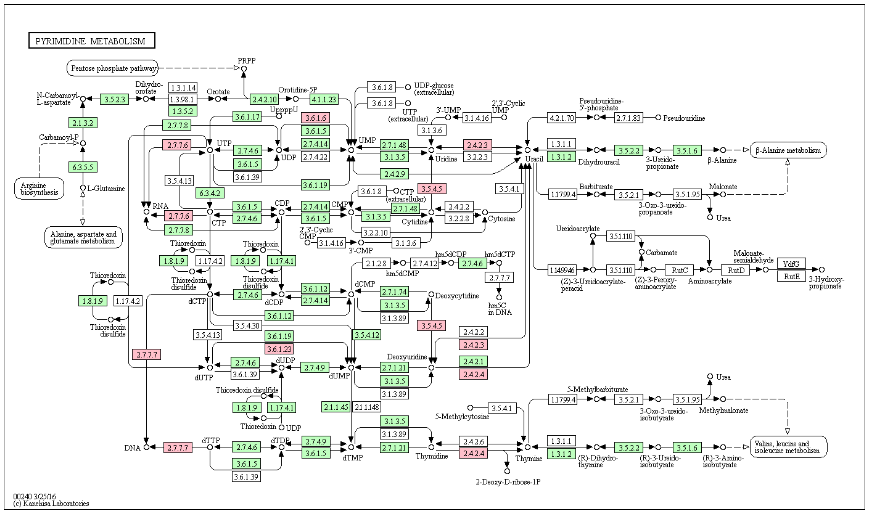

63.3 Legend:

RBH-Blast at 60% Identity + 50% Coverage
Green = Hit in *H. sapiens*
Red = Hit in *H. sapiens* and *T. californica*
White = Not in *H. sapiens*

64 Hepatitis B

64.1 Human Pathway: HSA05161

64.2 Number of Hits: 11

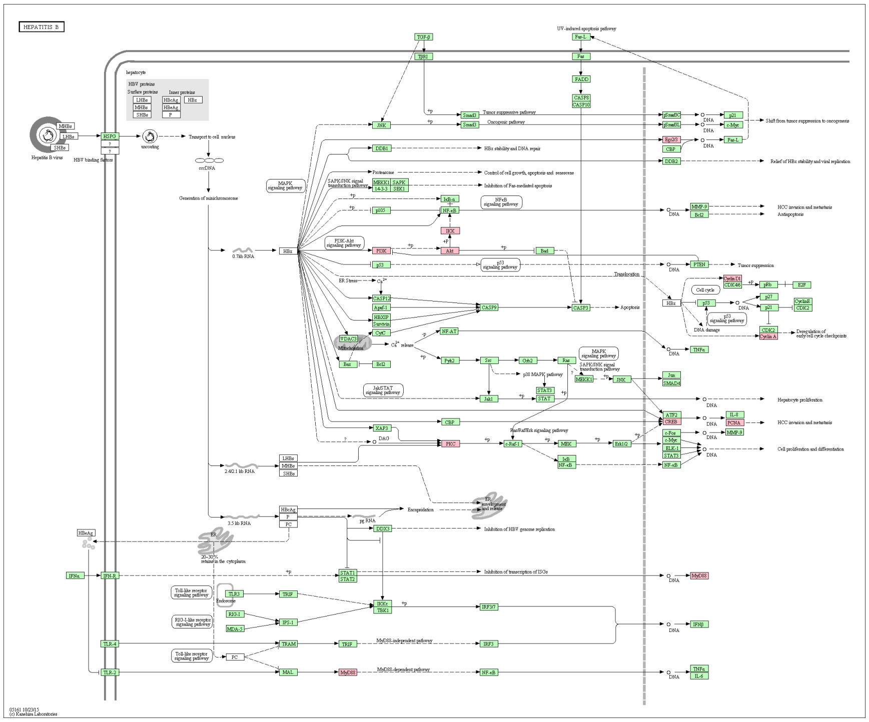

64.3 Legend:

RBH-Blast at 60% Identity + 50% Coverage
Green = Hit in *H. sapiens*
Red = Hit in *H. sapiens* and *T. californica*
White = Not in *H. sapiens*

65 Oocyte meiosis

65.1 Human Pathway: HSA04114

65.2 Number of Hits: 11

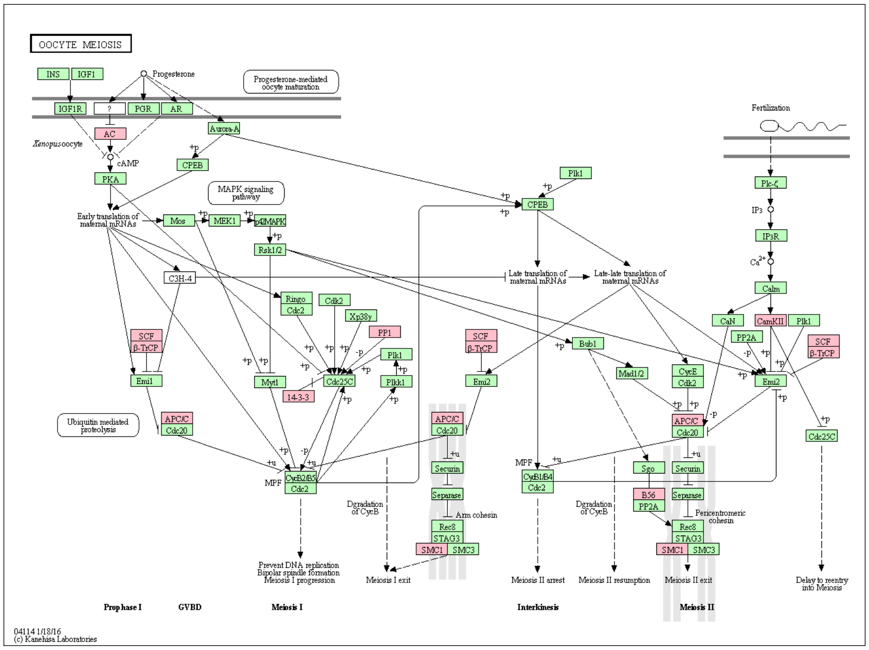

65.3 Legend:

RBH-Blast at 60% Identity + 50% Coverage
Green = Hit in *H. sapiens*
Red = Hit in *H. sapiens* and *T. californica*
White = Not in *H. sapiens*

66 Glucagon signaling pathway

66.1 Human Pathway: HSA04922

66.2 Number of Hits: 11

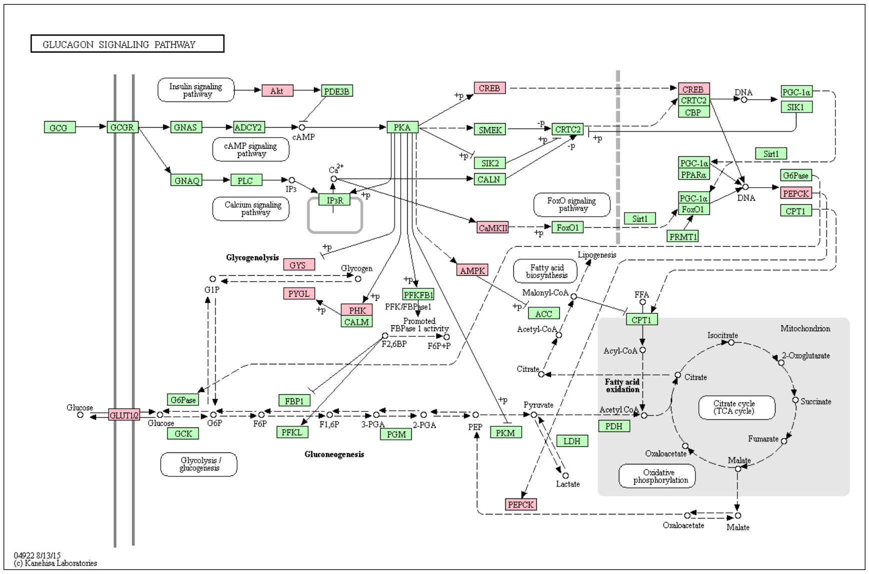

66.3 Legend:

RBH-Blast at 60% Identity + 50% Coverage
Green = Hit in *H. sapiens*
Red = Hit in *H. sapiens* and *T. californica*
White = Not in *H. sapiens*

67 Salmonella infection

67.1 Human Pathway: HSA05132

67.2 Number of Hits: 11

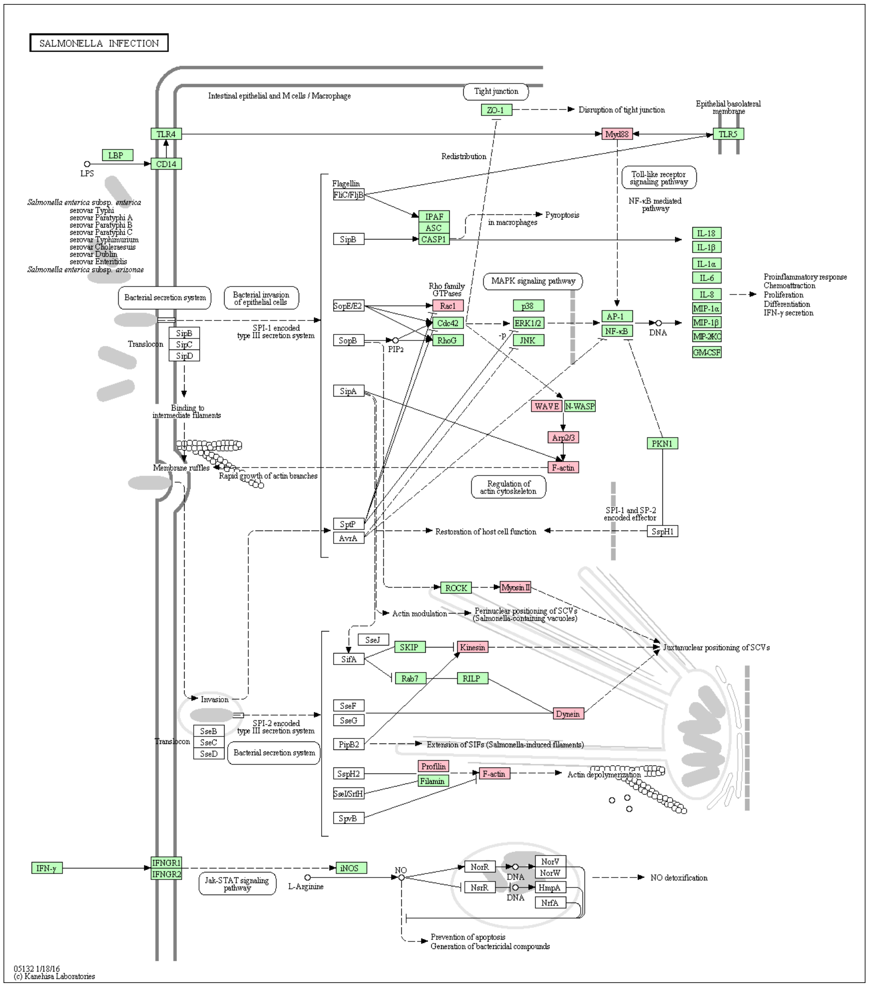

67.3 Legend:

RBH-Blast at 60% Identity + 50% Coverage
Green = Hit in *H. sapiens*
Red = Hit in *H. sapiens* and *T. californica*
White = Not in *H. sapiens*

68 Retrograde endocannabinoid signaling

68.1 Human Pathway: HSA04723

68.2 Number of Hits: 11

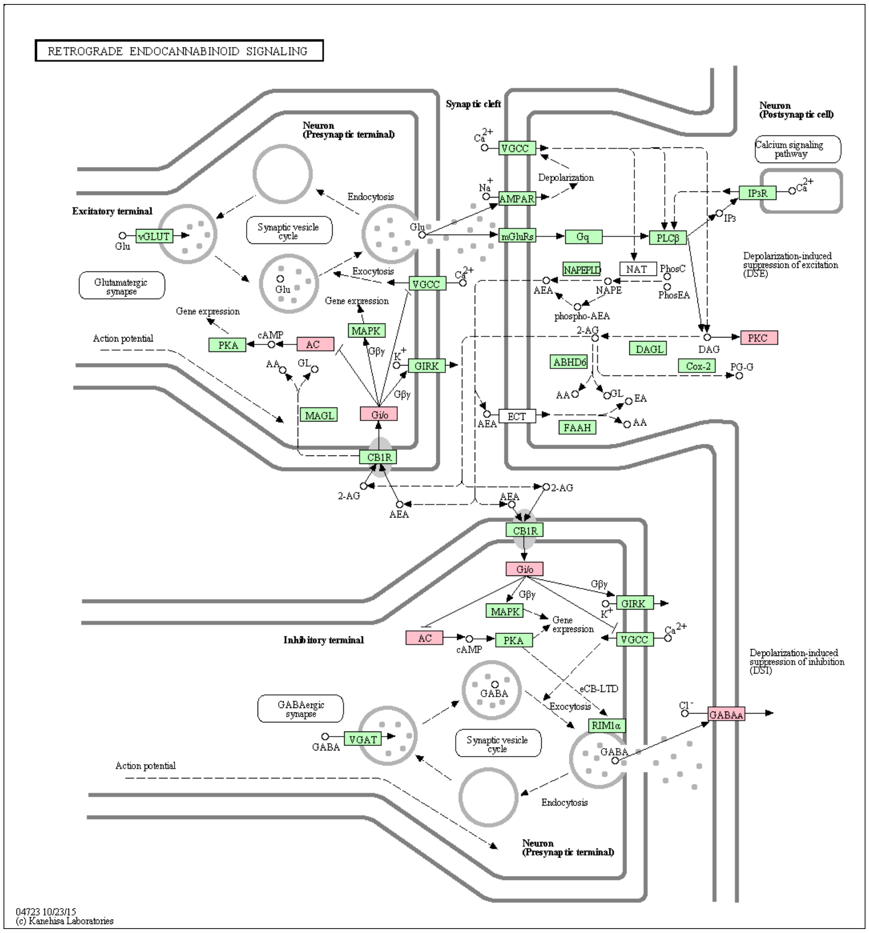

68.3 Legend:

RBH-Blast at 60% Identity + 50% Coverage
Green = Hit in *H. sapiens*
Red = Hit in *H. sapiens* and *T. californica*
White = Not in *H. sapiens*

69 Cell adhesion molecules (CAMs)

69.1 Human Pathway: HSA04514

69.2 Number of Hits: 10

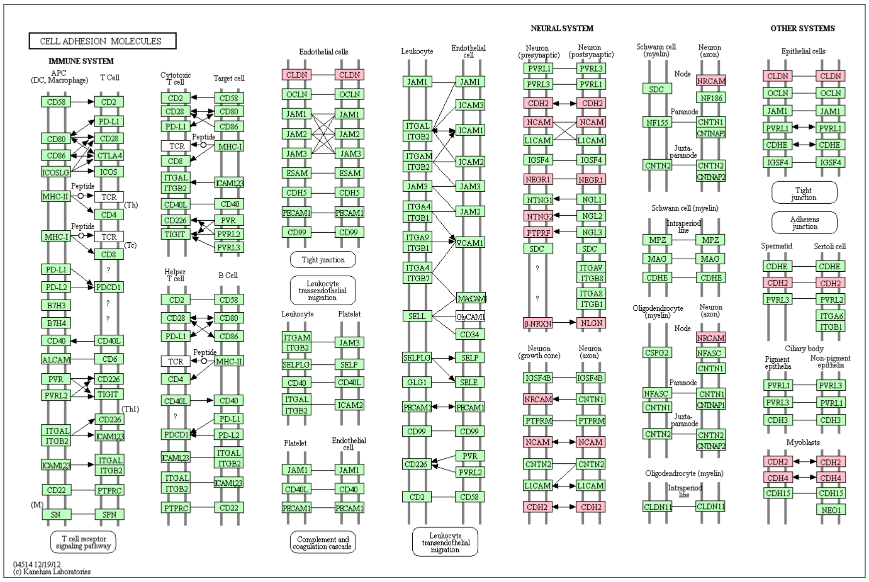

69.3 Legend:

RBH-Blast at 60% Identity + 50% Coverage
Green = Hit in *H. sapiens*
Red = Hit in *H. sapiens* and *T. californica*
White = Not in *H. sapiens*

70 T cell receptor signaling pathway

70.1 Human Pathway: HSA04660

70.2 Number of Hits: 10

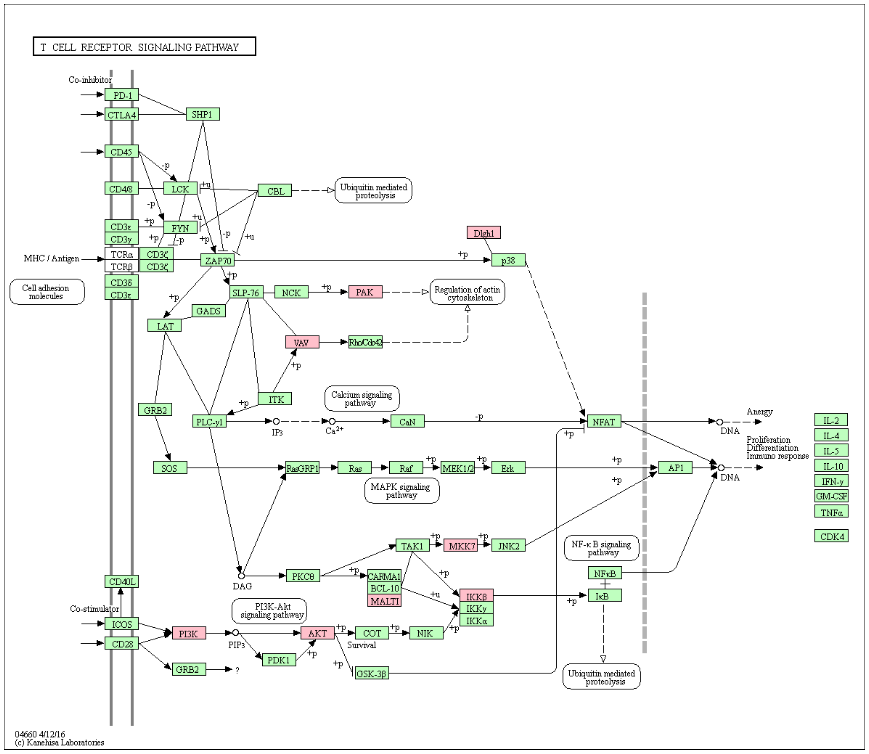

70.3 Legend:

RBH-Blast at 60% Identity + 50% Coverage
Green = Hit in *H. sapiens*
Red = Hit in *H. sapiens* and *T. californica*
White = Not in *H. sapiens*

71 B cell receptor signaling pathway

71.1 Human Pathway: HSA04662

71.2 Number of Hits: 10

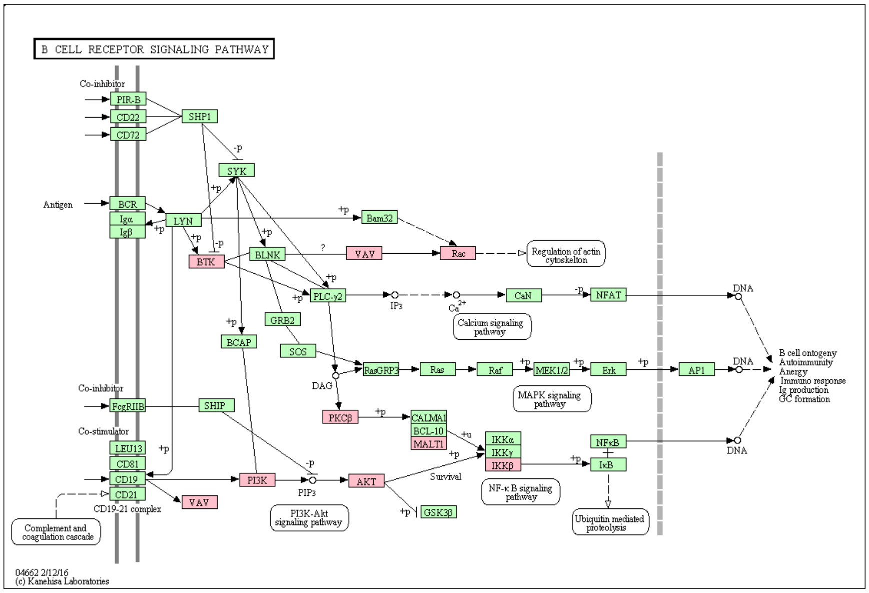

71.3 Legend:

RBH-Blast at 60% Identity + 50% Coverage
Green = Hit in *H. sapiens*
Red = Hit in *H. sapiens* and *T. californica*
White = Not in *H. sapiens*

72 Adipocytokine signaling pathway

72.1 Human Pathway: HSA04920

72.2 Number of Hits: 10

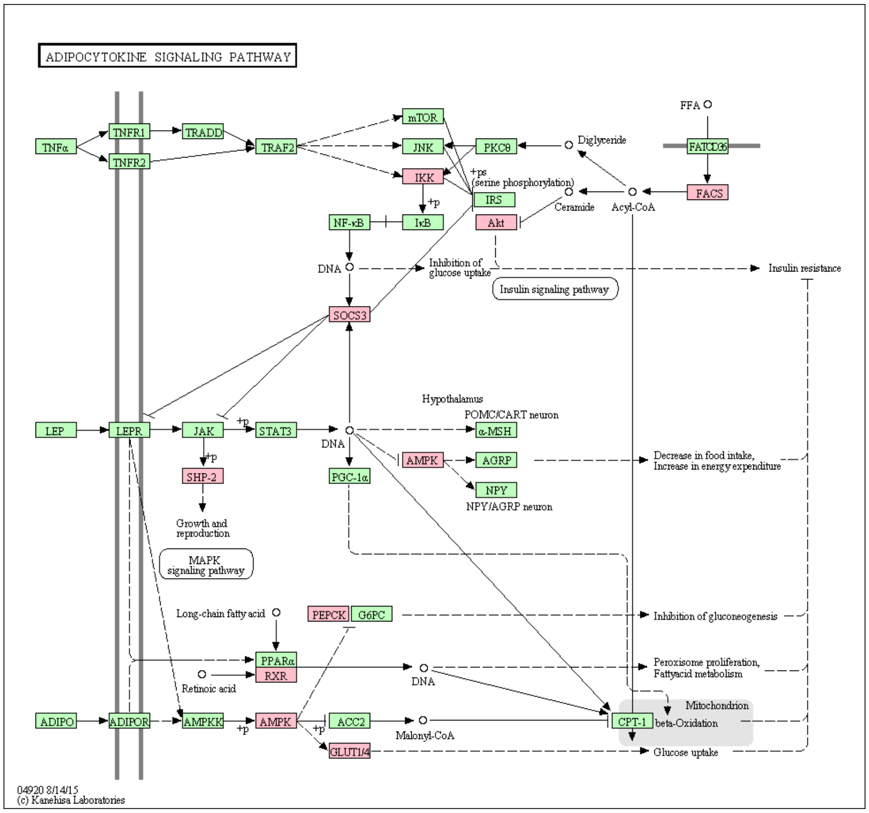

72.3 Legend:

RBH-Blast at 60% Identity + 50% Coverage
Green = Hit in *H. sapiens*
Red = Hit in *H. sapiens* and *T. californica*
White = Not in *H. sapiens*

73 Signaling pathways regulating pluripotency of stem cells

73.1 Human Pathway: HSA04550

73.2 Number of Hits: 10

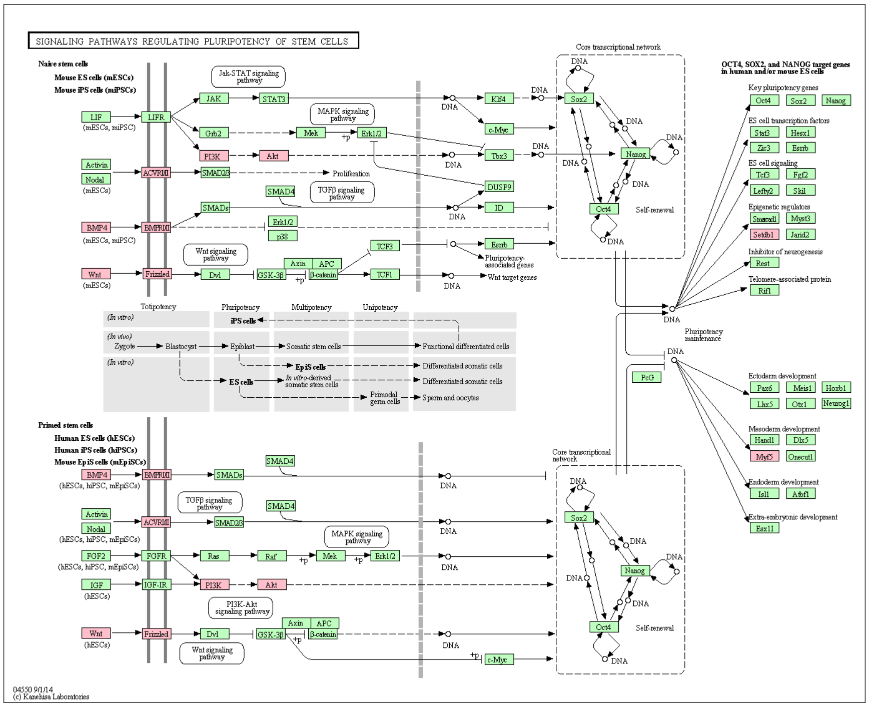

73.3 Legend:

RBH-Blast at 60% Identity + 50% Coverage
Green = Hit in *H. sapiens*
Red = Hit in *H. sapiens* and *T. californica*
White = Not in *H. sapiens*

74 HIF-1 signaling pathway

74.1 Human Pathway: HSA04066

74.2 Number of Hits: 10

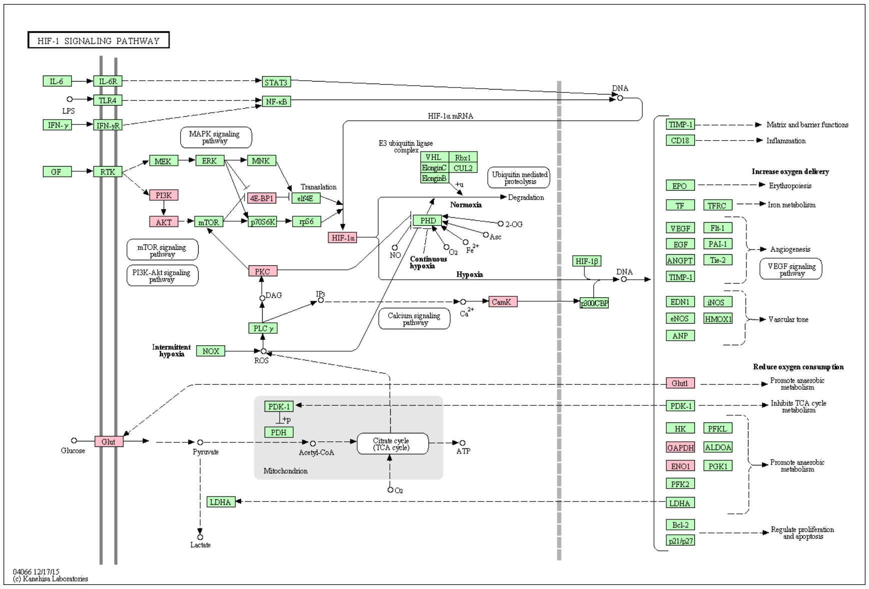

74.3 Legend:

RBH-Blast at 60% Identity + 50% Coverage
Green = Hit in *H. sapiens*
Red = Hit in *H. sapiens* and *T. californica*
White = Not in *H. sapiens*

75 Toxoplasmosis

75.1 Human Pathway: HSA05145

75.2 Number of Hits: 10

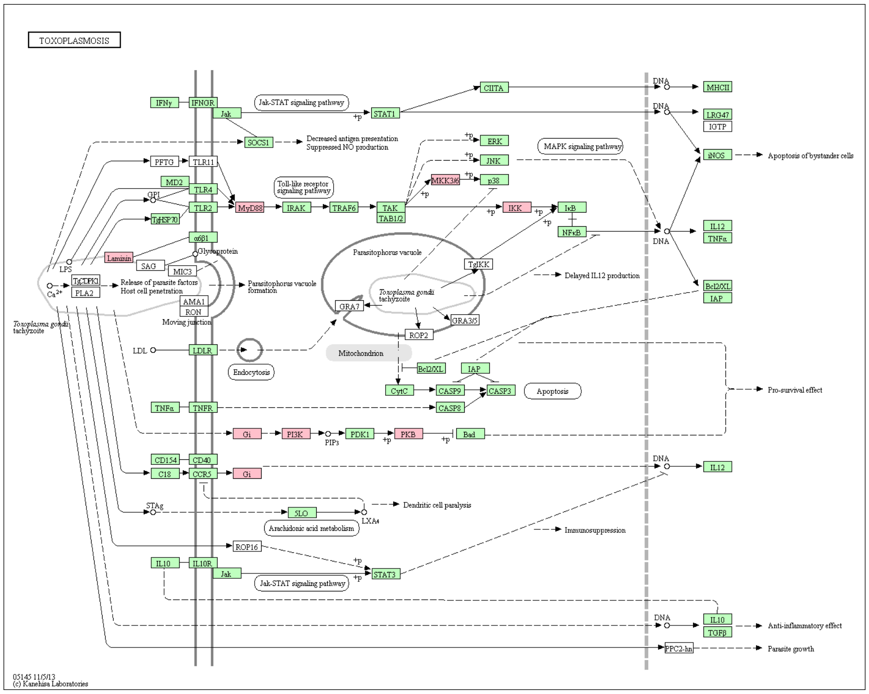

75.3 Legend:

RBH-Blast at 60% Identity + 50% Coverage
Green = Hit in *H. sapiens*
Red = Hit in *H. sapiens* and *T. californica*
White = Not in *H. sapiens*

76 Carbon metabolism

76.1 Human Pathway: HSA01200

76.2 Number of Hits: 10

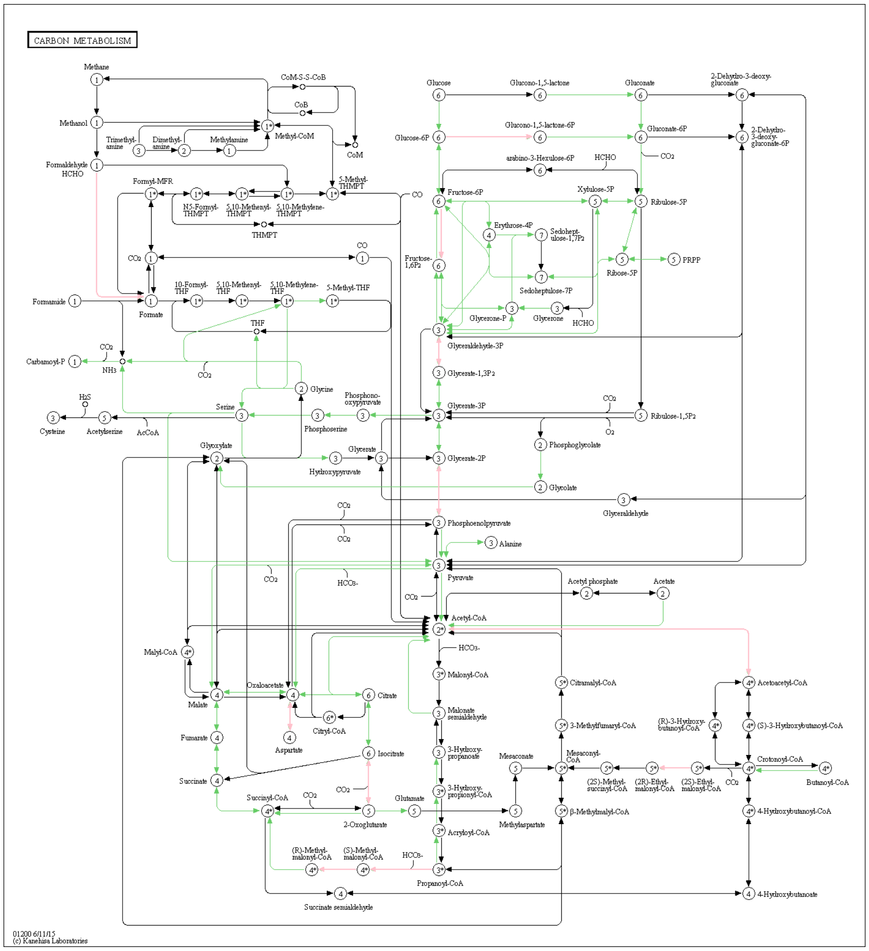

76.3 Legend:

RBH-Blast at 60% Identity + 50% Coverage
Green = Hit in *H. sapiens*
Red = Hit in *H. sapiens* and *T. californica*
White = Not in *H. sapiens*

77 Serotonergic synapse

77.1 Human Pathway: HSA04726

77.2 Number of Hits: 10

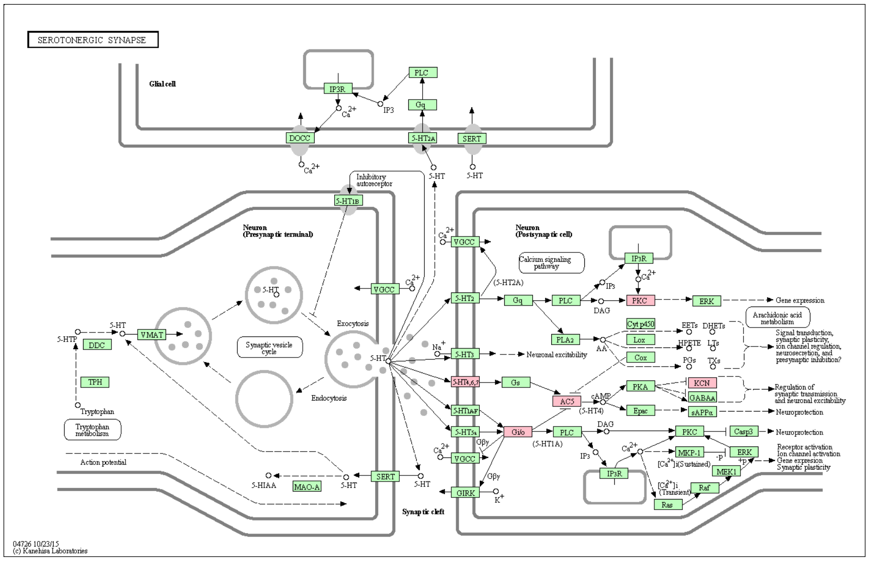

77.3 Legend:

RBH-Blast at 60% Identity + 50% Coverage
Green = Hit in *H. sapiens*
Red = Hit in *H. sapiens* and *T. californica*
White = Not in *H. sapiens*

78 Longevity regulating pathway - multiple species

78.1 Human Pathway: HSA04213

78.2 Number of Hits: 10

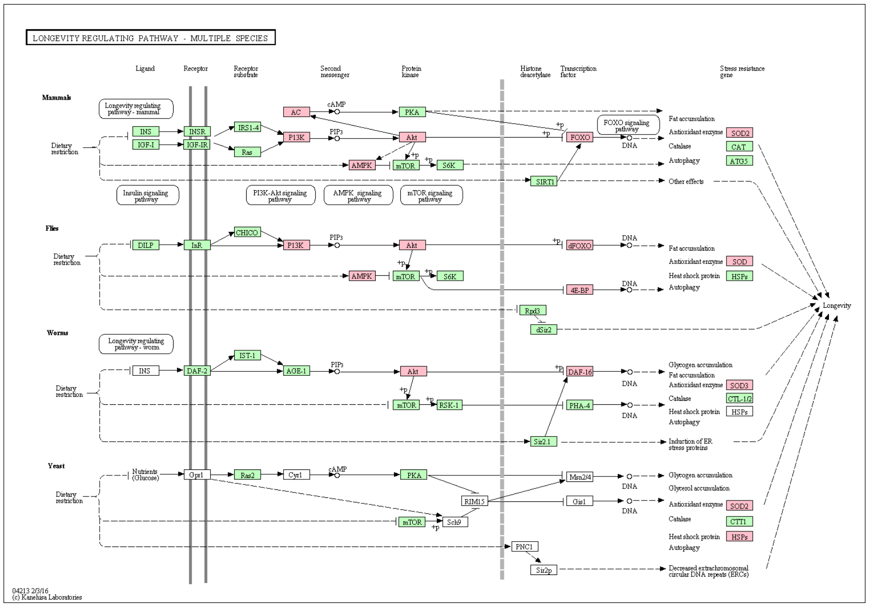

78.3 Legend:

RBH-Blast at 60% Identity + 50% Coverage
Green = Hit in *H. sapiens*
Red = Hit in *H. sapiens* and *T. californica*
White = Not in *H. sapiens*

79 ErbB signaling pathway

79.1 Human Pathway: HSA04012

79.2 Number of Hits: 10

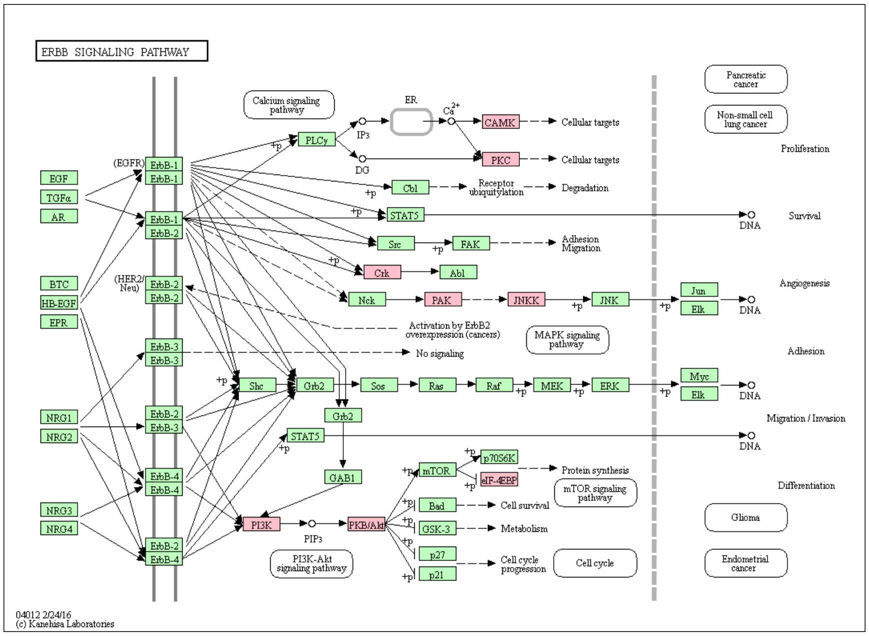

79.3 Legend:

RBH-Blast at 60% Identity + 50% Coverage
Green = Hit in *H. sapiens*
Red = Hit in *H. sapiens* and *T. californica*
White = Not in *H. sapiens*

80 Choline metabolism in cancer

80.1 Human Pathway: HSA05231

80.2 Number of Hits: 10

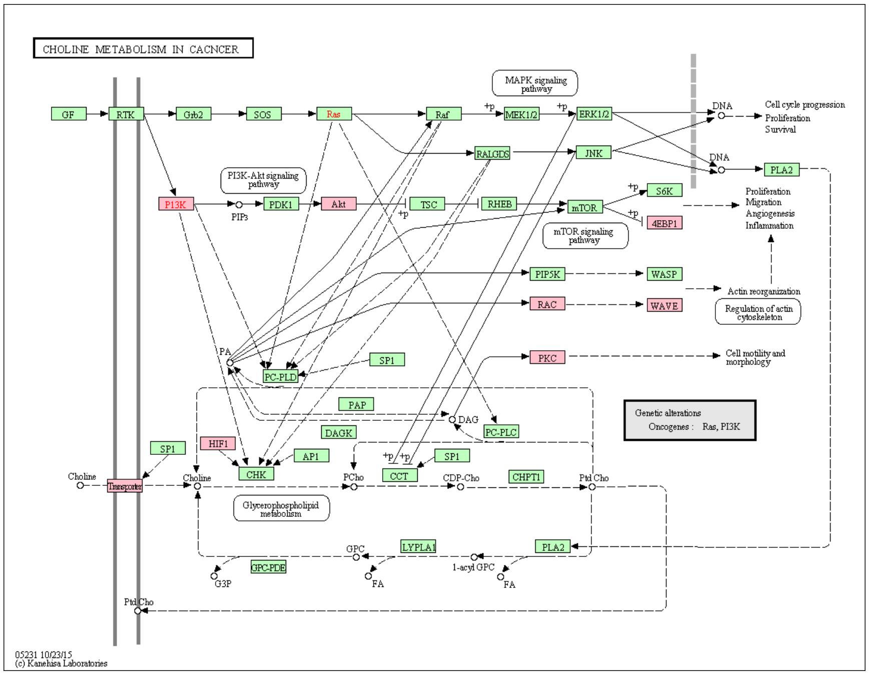

80.3 Legend:

RBH-Blast at 60% Identity + 50% Coverage
Green = Hit in *H. sapiens*
Red = Hit in *H. sapiens* and *T. californica*
White = Not in *H. sapiens*

81 Lysosome

81.1 Human Pathway: HSA04142

81.2 Number of Hits: 10

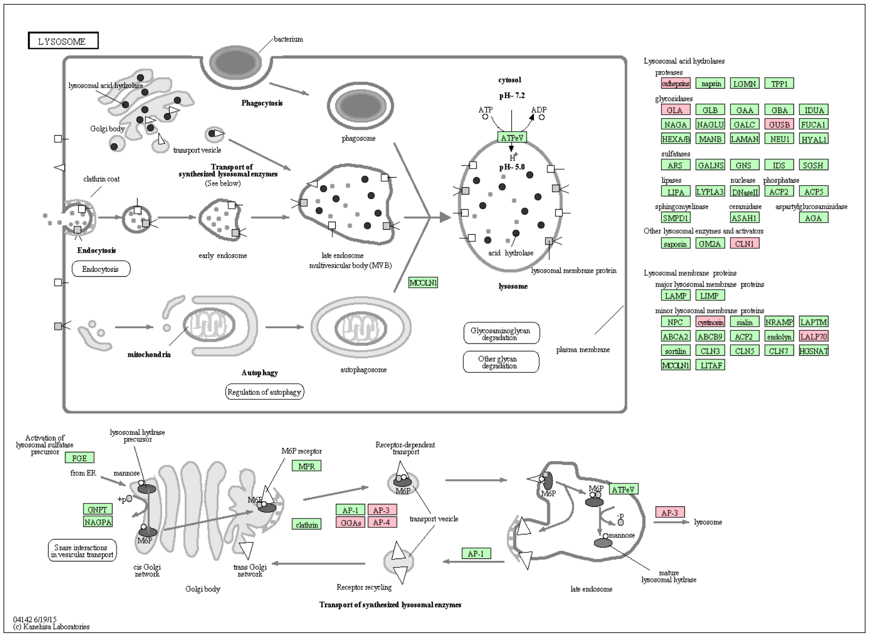

81.3 Legend:

RBH-Blast at 60% Identity + 50% Coverage
Green = Hit in *H. sapiens*
Red = Hit in *H. sapiens* and *T. californica*
White = Not in *H. sapiens*

82 Platelet activation

82.1 Human Pathway: HSA04611

82.2 Number of Hits: 10

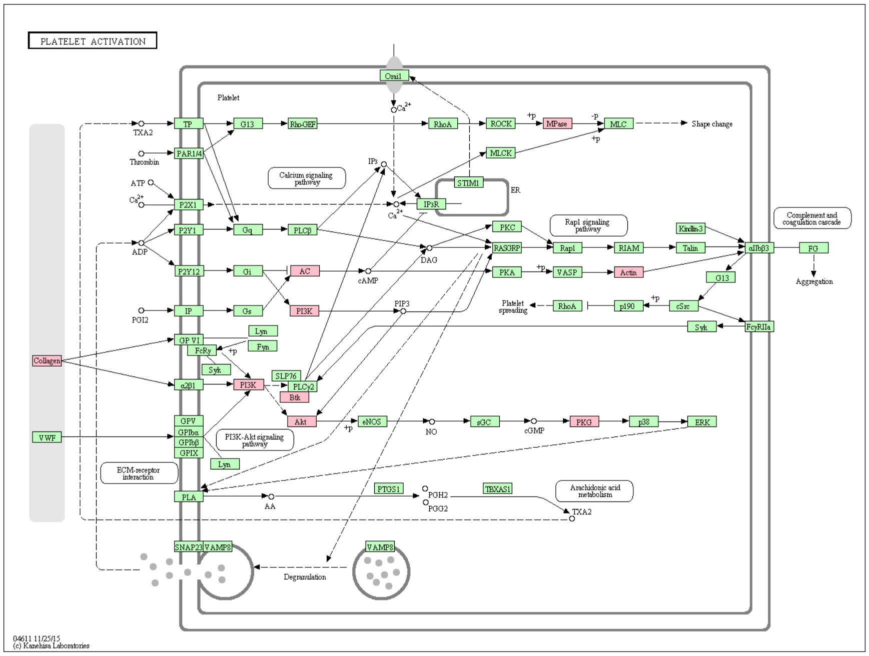

82.3 Legend:

RBH-Blast at 60% Identity + 50% Coverage
Green = Hit in *H. sapiens*
Red = Hit in *H. sapiens* and *T. californica*
White = Not in *H. sapiens*

83 ECM-receptor interaction

83.1 Human Pathway: HSA04512

83.2 Number of Hits: 9

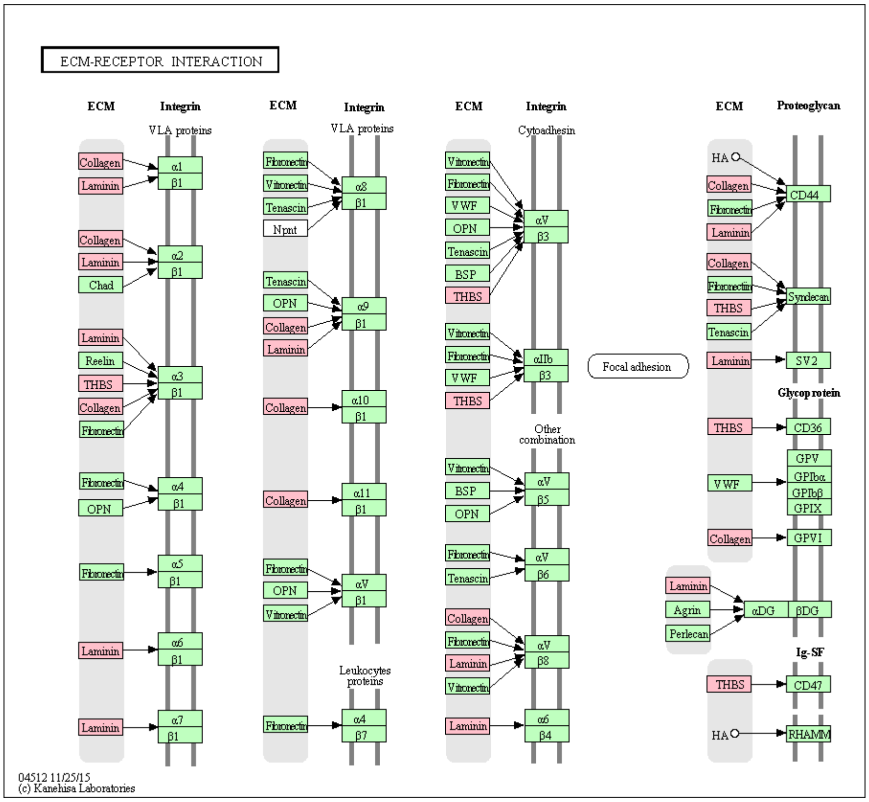

83.3 Legend:

RBH-Blast at 60% Identity + 50% Coverage
Green = Hit in *H. sapiens*
Red = Hit in *H. sapiens* and *T. californica*
White = Not in *H. sapiens*

84 Pancreatic secretion

84.1 Human Pathway: HSA04972

84.2 Number of Hits: 9

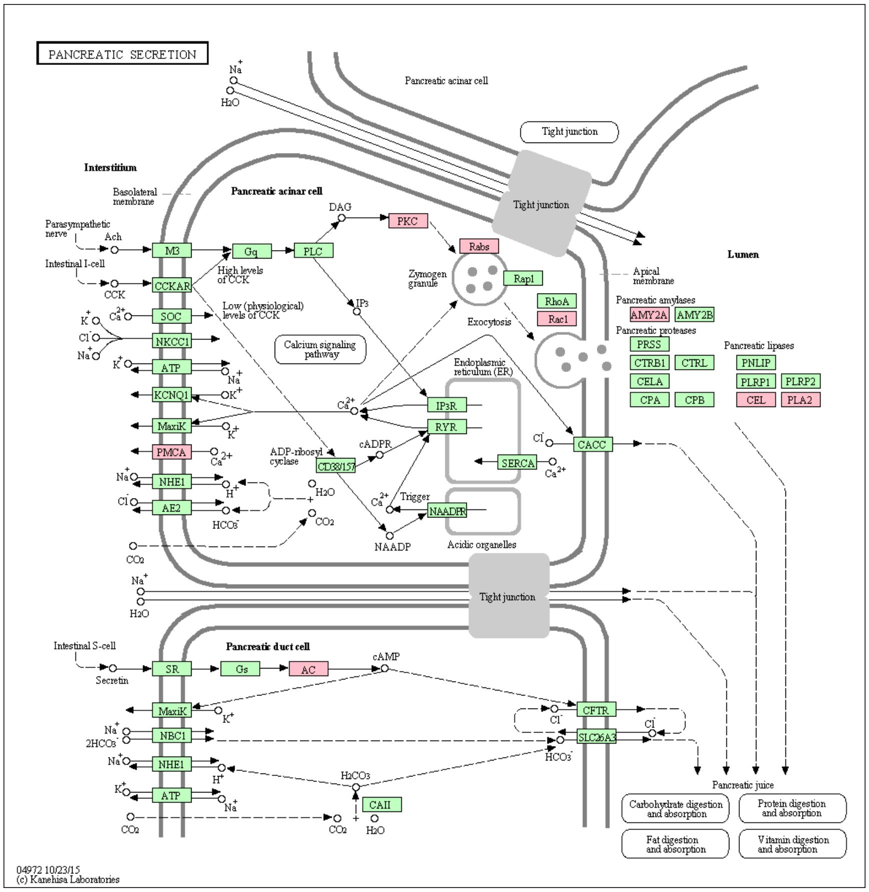

84.3 Legend:

RBH-Blast at 60% Identity + 50% Coverage
Green = Hit in *H. sapiens*
Red = Hit in *H. sapiens* and *T. californica*
White = Not in *H. sapiens*

85 Leukocyte transendothelial migration

85.1 Human Pathway: HSA04670

85.2 Number of Hits: 9

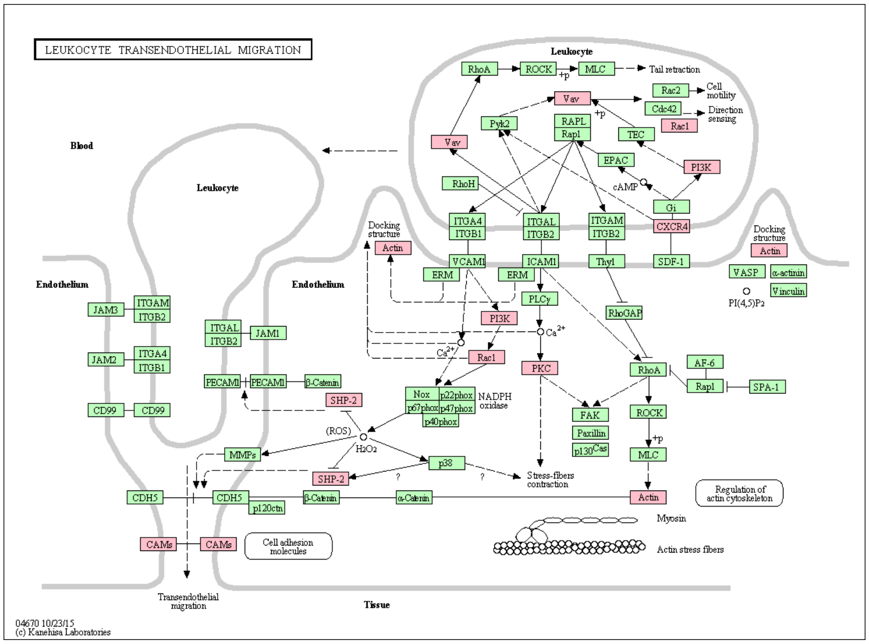

85.3 Legend:

RBH-Blast at 60% Identity + 50% Coverage
Green = Hit in *H. sapiens*
Red = Hit in *H. sapiens* and *T. californica*
White = Not in *H. sapiens*

86 Osteoclast differentiation

86.1 Human Pathway: HSA04380

86.2 Number of Hits: 9

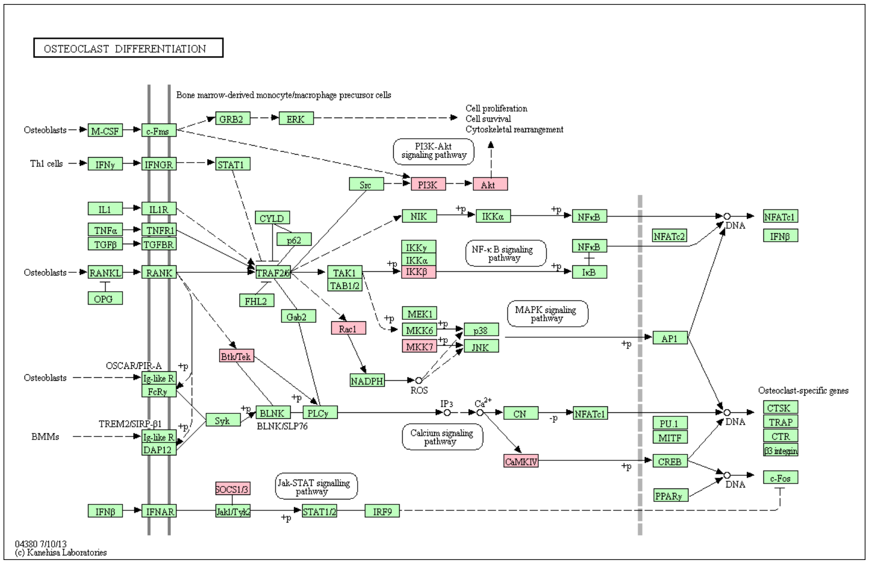

86.3 Legend:

RBH-Blast at 60% Identity + 50% Coverage
Green = Hit in *H. sapiens*
Red = Hit in *H. sapiens* and *T. californica*
White = Not in *H. sapiens*

87 Pancreatic cancer

87.1 Human Pathway: HSA05212

87.2 Number of Hits: 9

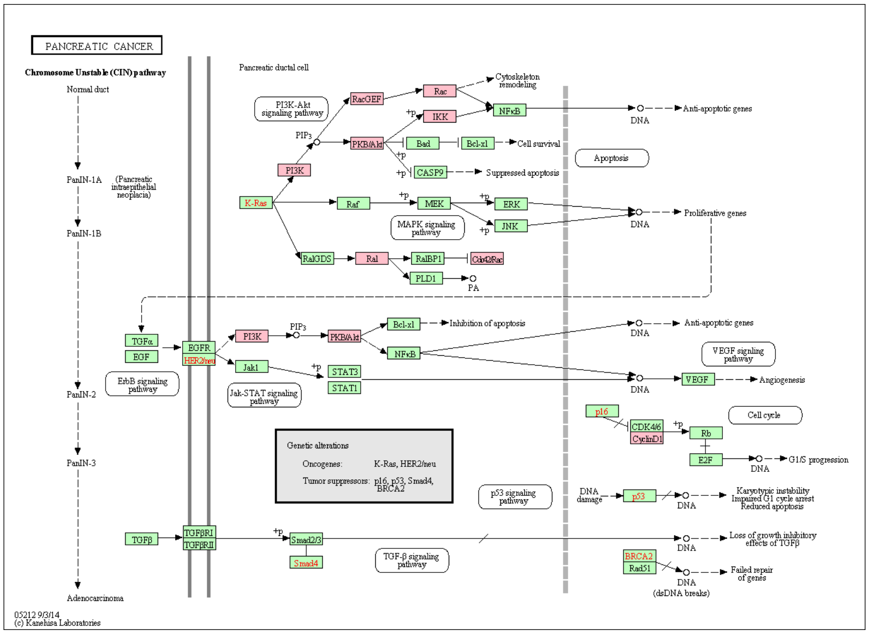

87.3 Legend:

RBH-Blast at 60% Identity + 50% Coverage
Green = Hit in *H. sapiens*
Red = Hit in *H. sapiens* and *T. californica*
White = Not in *H. sapiens*

88 TNF signaling pathway

88.1 Human Pathway: HSA04668

88.2 Number of Hits: 9

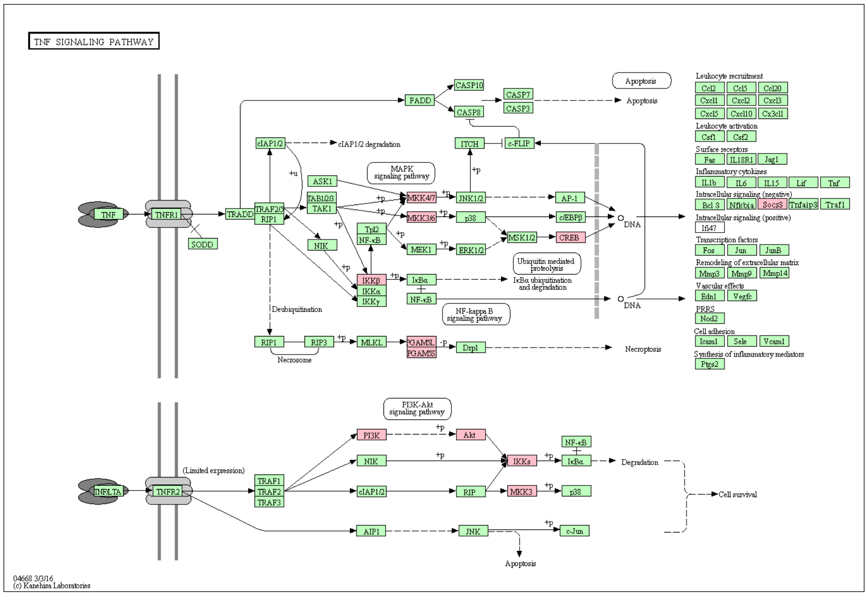

88.3 Legend:

RBH-Blast at 60% Identity + 50% Coverage
Green = Hit in *H. sapiens*
Red = Hit in *H. sapiens* and *T. californica*
White = Not in *H. sapiens*

89 Small cell lung cancer

89.1 Human Pathway: HSA05222

89.2 Number of Hits: 9

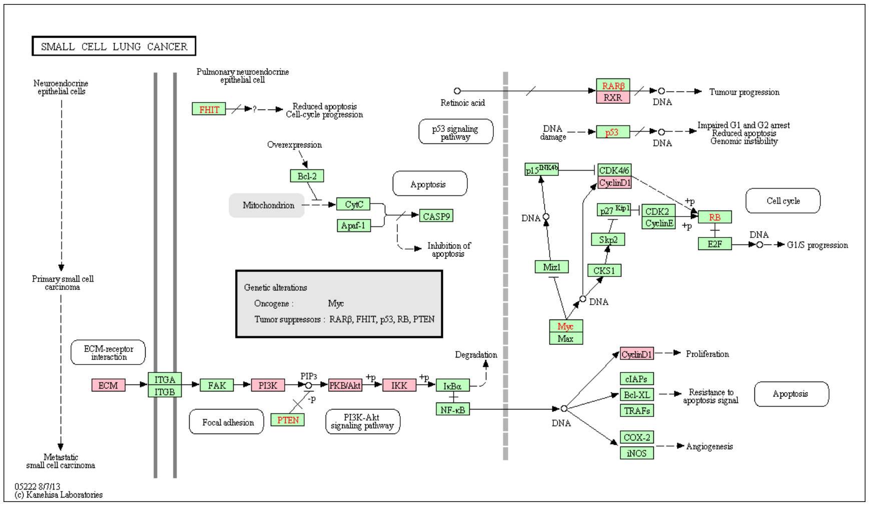

89.3 Legend:

RBH-Blast at 60% Identity + 50% Coverage
Green = Hit in *H. sapiens*
Red = Hit in *H. sapiens* and *T. californica*
White = Not in *H. sapiens*

90 Fc epsilon RI signaling pathway

90.1 Human Pathway: HSA04664

90.2 Number of Hits: 9

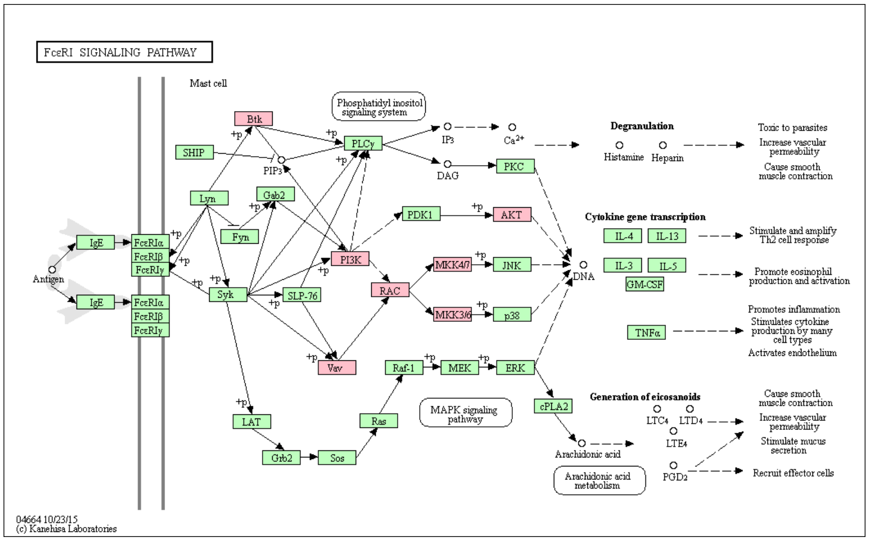

90.3 Legend:

RBH-Blast at 60% Identity + 50% Coverage
Green = Hit in *H. sapiens*
Red = Hit in *H. sapiens* and *T. californica*
White = Not in *H. sapiens*

91 Glycolysis / Gluconeogenesis

91.1 Human Pathway: HSA00010

91.2 Number of Hits: 9

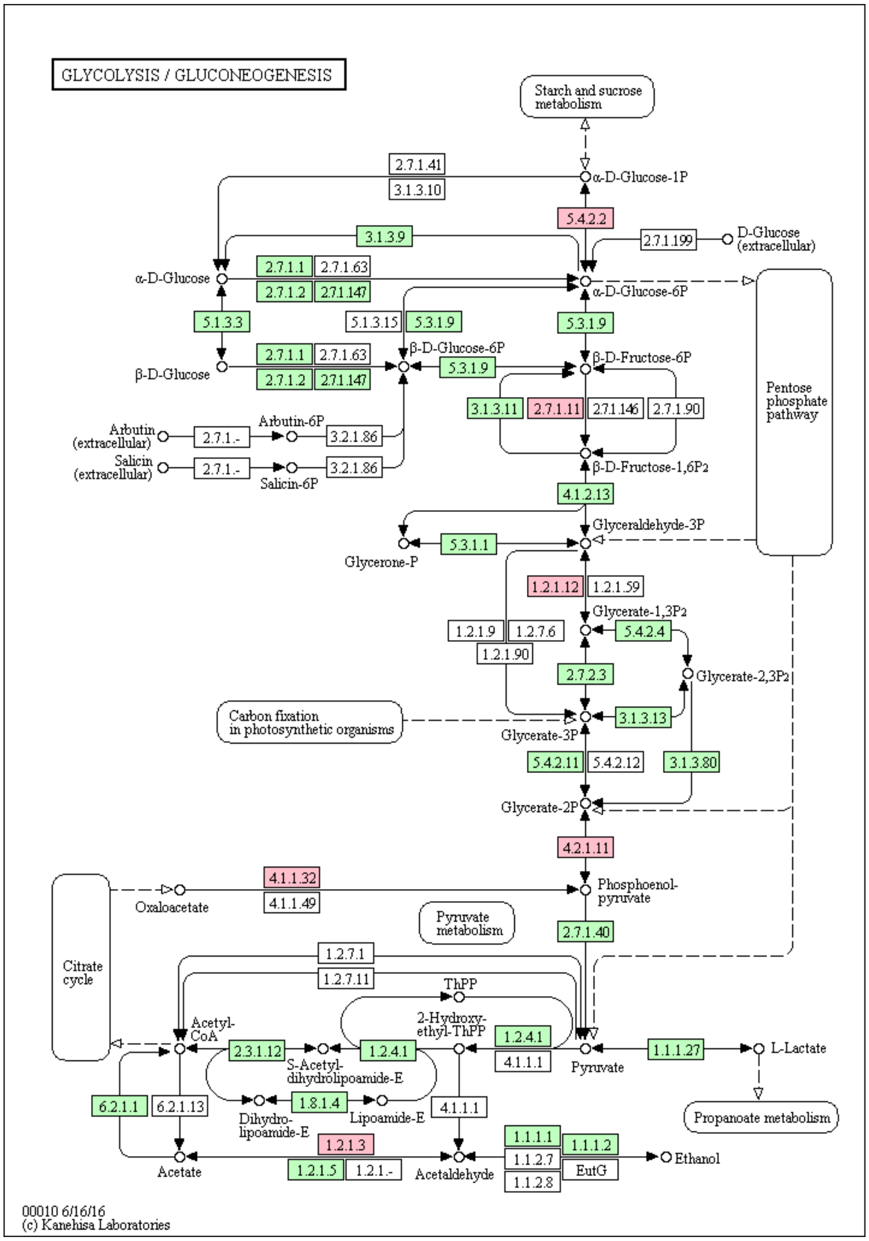

91.3 Legend:

RBH-Blast at 60% Identity + 50% Coverage
Green = Hit in *H. sapiens*
Red = Hit in *H. sapiens* and *T. californica*
White = Not in *H. sapiens*

---

92 AGE-RAGE signaling pathway in diabetic complications

92.1 Human Pathway: HSA04933

92.2 Number of Hits: 9

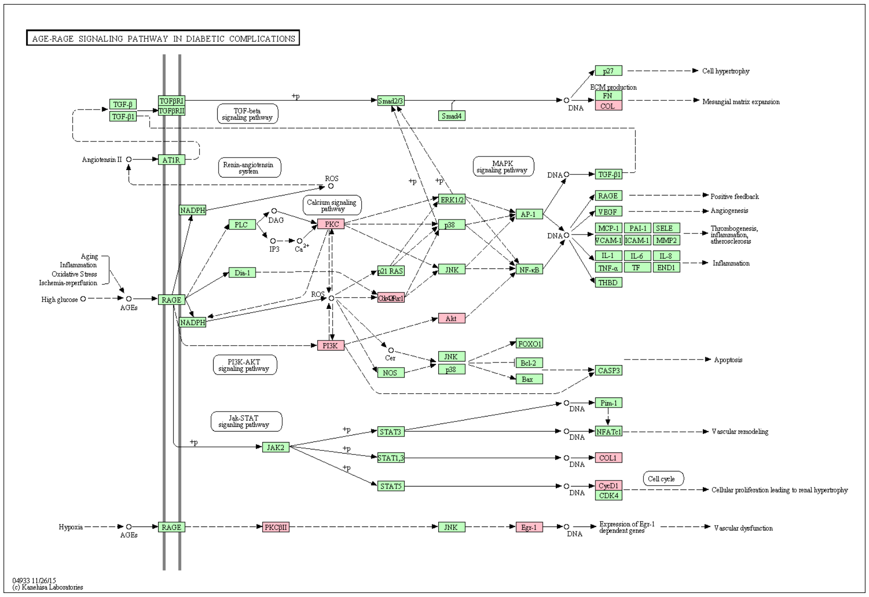

92.3 Legend:

RBH-Blast at 60% Identity + 50% Coverage
Green = Hit in *H. sapiens*
Red = Hit in *H. sapiens* and *T. californica*
White = Not in *H. sapiens*

---

93 Viral myocarditis

93.1 Human Pathway: HSA05416

93.2 Number of Hits: 8

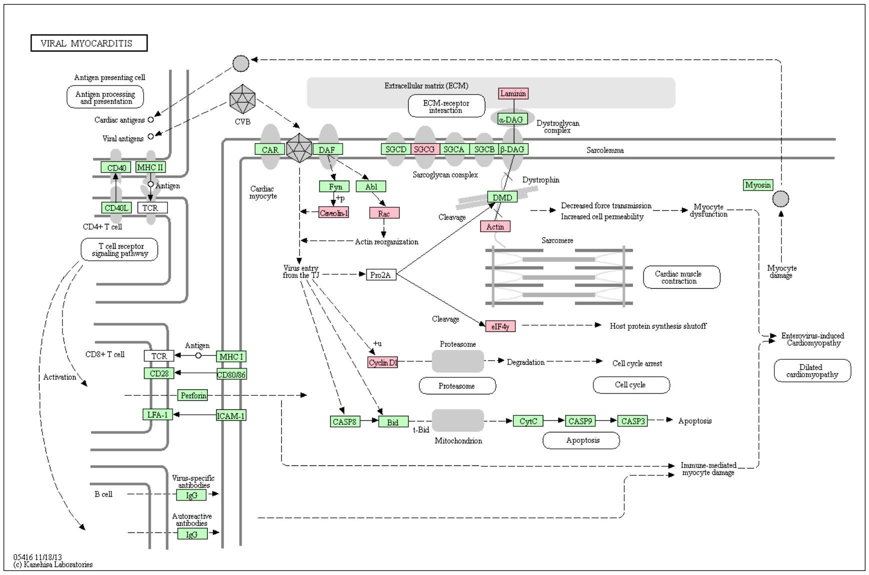

93.3 Legend:

RBH-Blast at 60% Identity + 50% Coverage
Green = Hit in *H. sapiens*
Red = Hit in *H. sapiens* and *T. californica*
White = Not in *H. sapiens*

94 Endocrine and other factor-regulated calcium reabsorption

94.1 Human Pathway: HSA04961

94.2 Number of Hits: 8

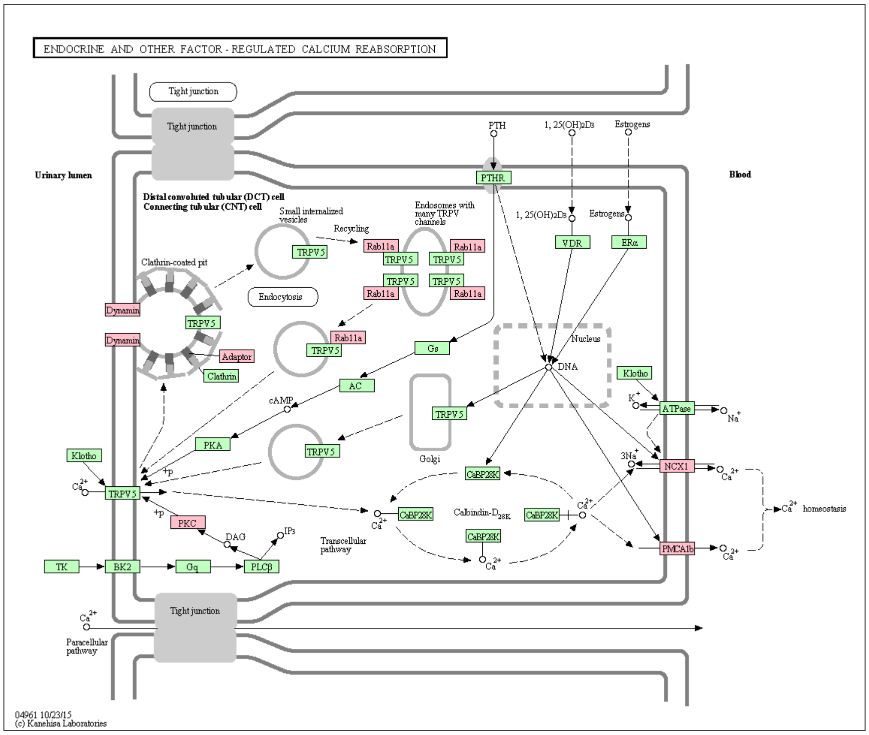

94.3 Legend:

RBH-Blast at 60% Identity + 50% Coverage
Green = Hit in *H. sapiens*
Red = Hit in *H. sapiens* and *T. californica*
White = Not in *H. sapiens*

95 Estrogen signaling pathway

95.1 Human Pathway: HSA04915

95.2 Number of Hits: 8

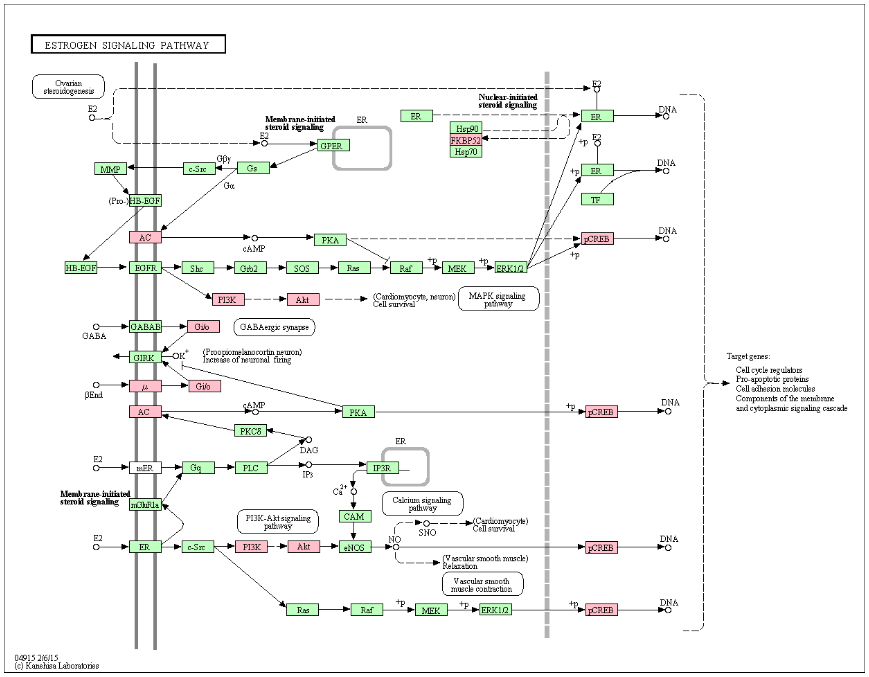

95.3 Legend:

RBH-Blast at 60% Identity + 50% Coverage
Green = Hit in *H. sapiens*
Red = Hit in *H. sapiens* and *T. californica*
White = Not in *H. sapiens*

96 Natural killer cell mediated cytotoxicity

96.1 Human Pathway: HSA04650

96.2 Number of Hits: 8

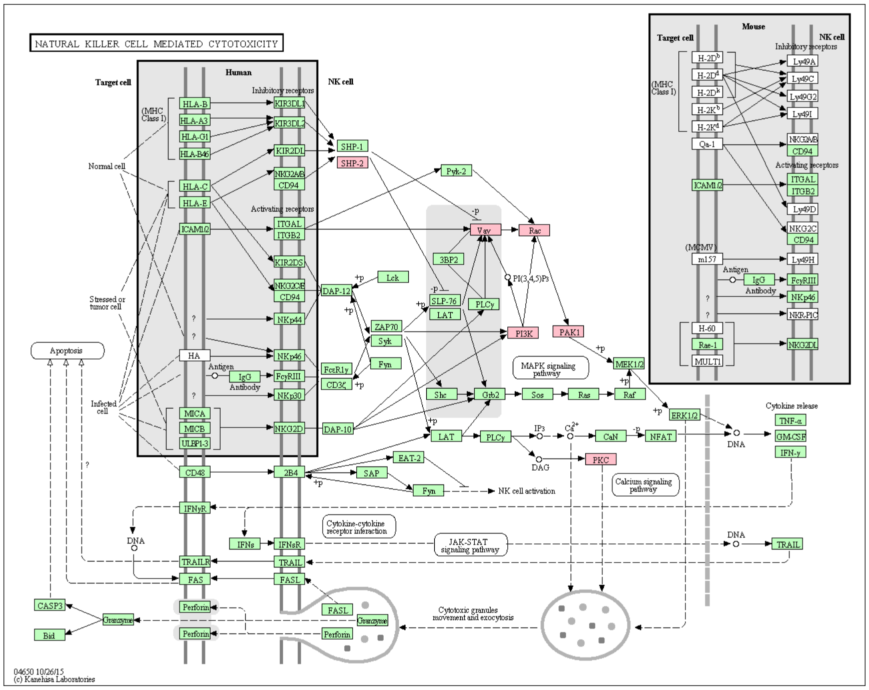

96.3 Legend:

RBH-Blast at 60% Identity + 50% Coverage
Green = Hit in *H. sapiens*
Red = Hit in *H. sapiens* and *T. californica*
White = Not in *H. sapiens*

97 Chronic myeloid leukemia

97.1 Human Pathway: HSA05220

97.2 Number of Hits: 8

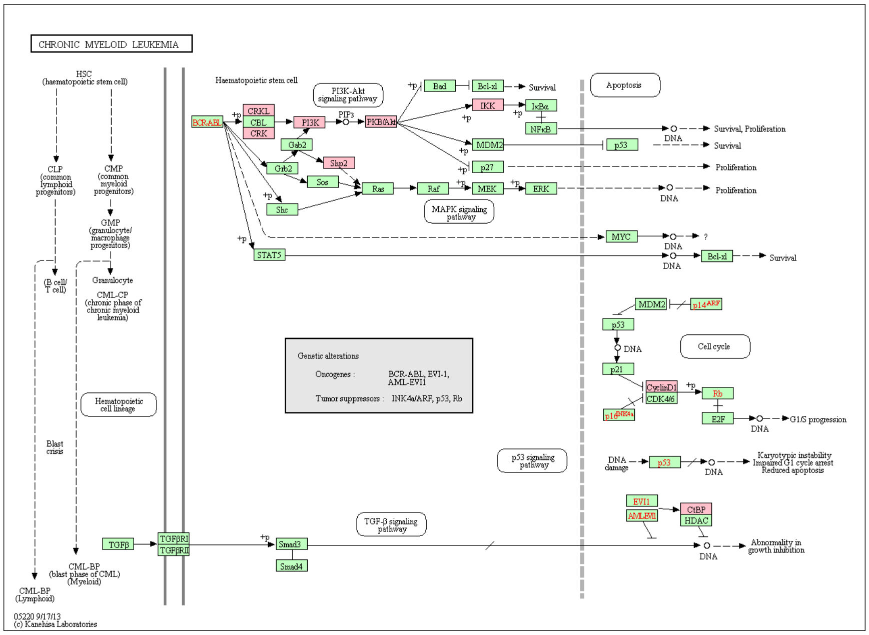

97.3 Legend:

RBH-Blast at 60% Identity + 50% Coverage
Green = Hit in *H. sapiens*
Red = Hit in *H. sapiens* and *T. californica*
White = Not in *H. sapiens*

98 Fatty acid degradation

98.1 Human Pathway: HSA00071

98.2 Number of Hits: 8

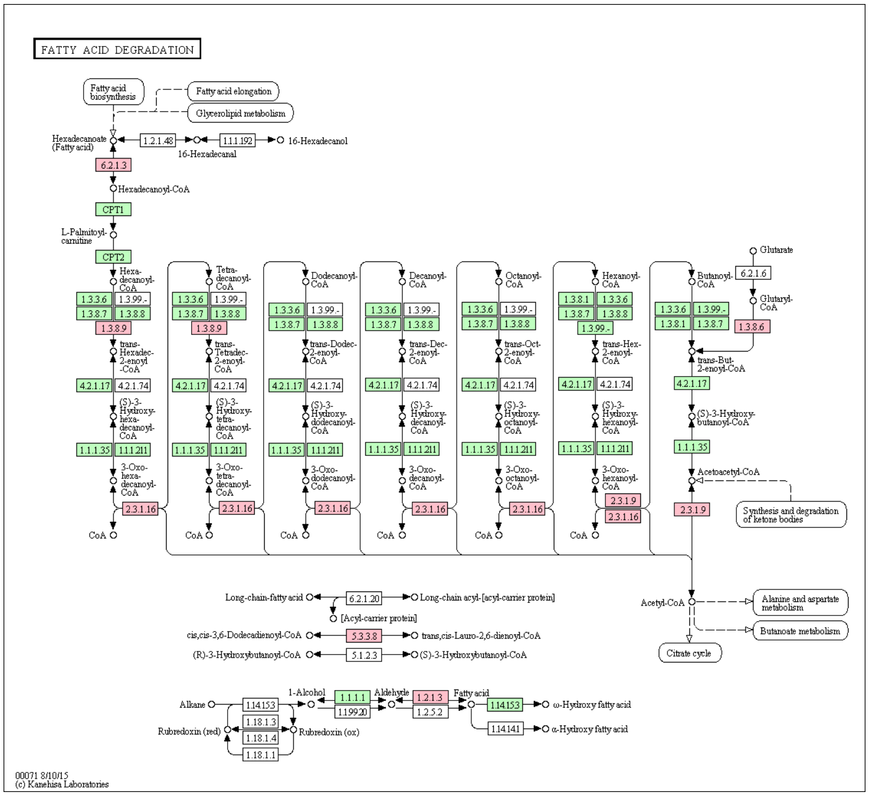

98.3 Legend:

RBH-Blast at 60% Identity + 50% Coverage
Green = Hit in *H. sapiens*
Red = Hit in *H. sapiens* and *T. californica*
White = Not in *H. sapiens*

99 Lysine degradation

99.1 Human Pathway: HSA00310

99.2 Number of Hits: 8

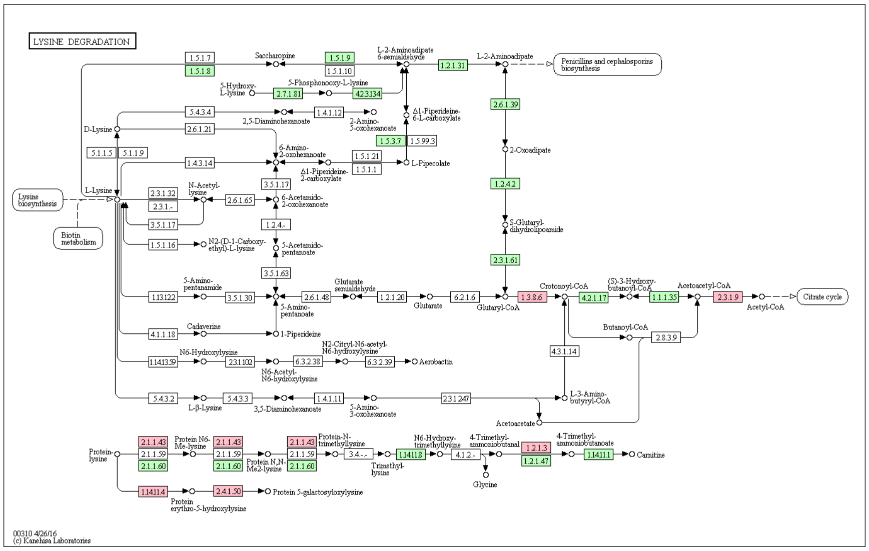

99.3 Legend:

RBH-Blast at 60% Identity + 50% Coverage
Green = Hit in *H. sapiens*
Red = Hit in *H. sapiens* and *T. californica*
White = Not in *H. sapiens*

100 RNA degradation

100.1 Human Pathway: HSA03018

100.2 Number of Hits: 8

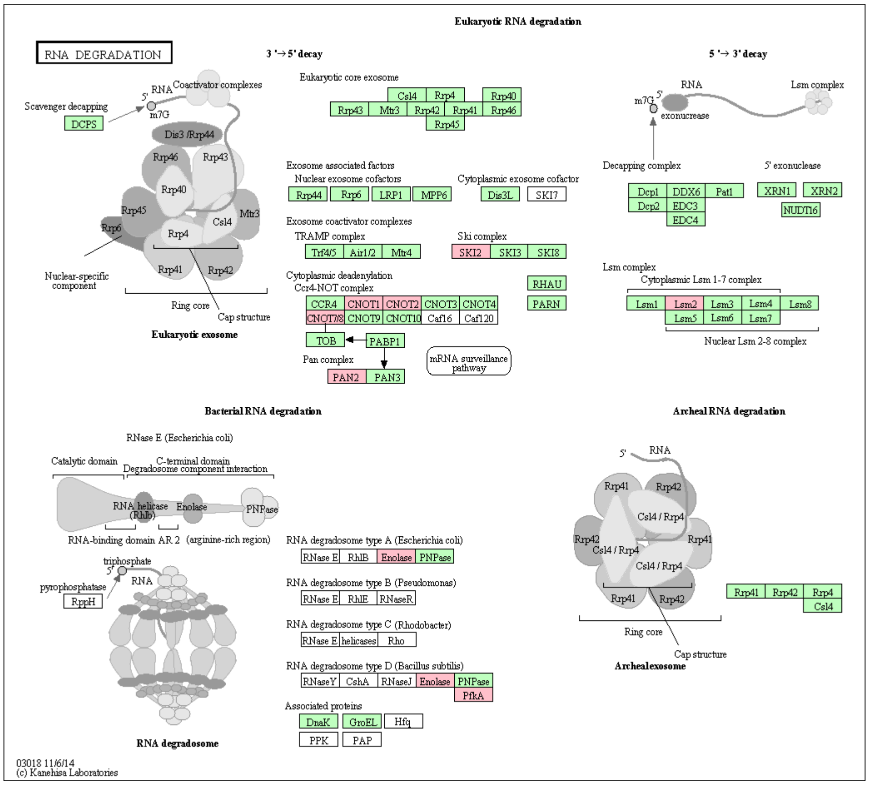

100.3 Legend:

RBH-Blast at 60% Identity + 50% Coverage
Green = Hit in *H. sapiens*
Red = Hit in *H. sapiens* and *T. californica*
White = Not in *H. sapiens*

101 Biosynthesis of amino acids

101.1 Human Pathway: HSA01230

101.2 Number of Hits: 8

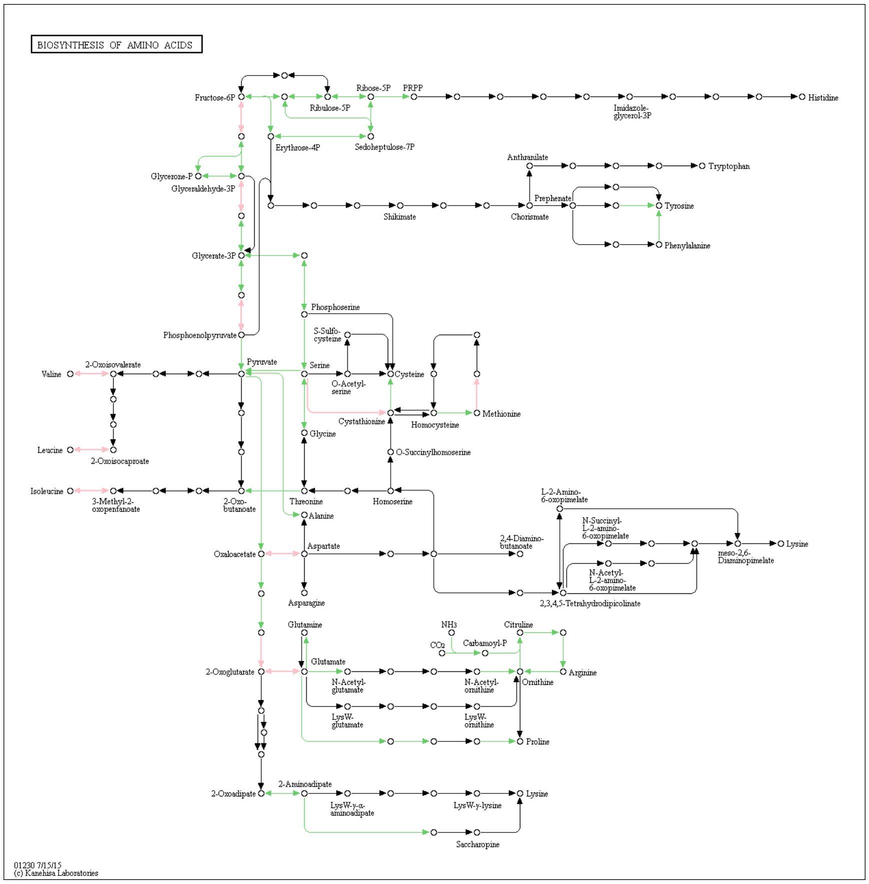

101.3 Legend:

RBH-Blast at 60% Identity + 50% Coverage
Green = Hit in *H. sapiens*
Red = Hit in *H. sapiens* and *T. californica*
White = Not in *H. sapiens*

102 Insulin secretion

102.1 Human Pathway: HSA04911

102.2 Number of Hits: 8

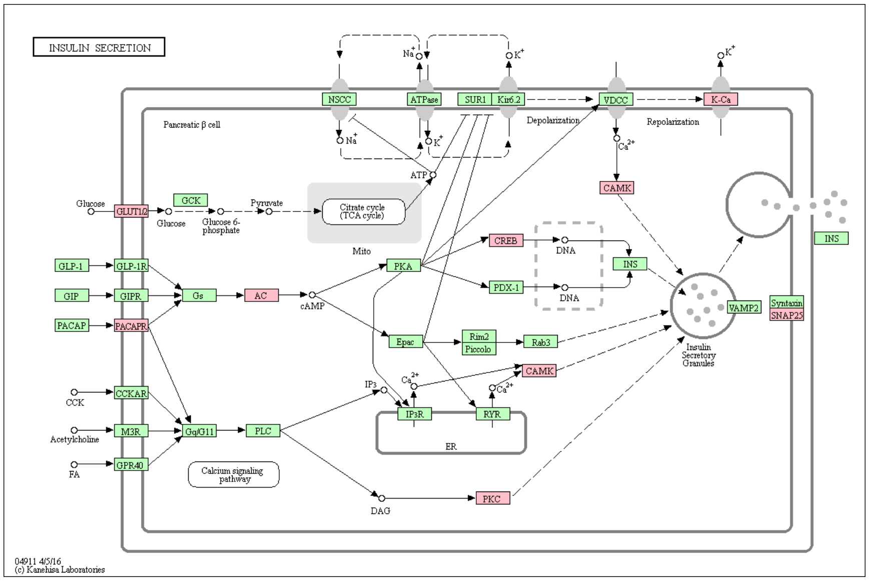

102.3 Legend:

RBH-Blast at 60% Identity + 50% Coverage
Green = Hit in *H. sapiens*
Red = Hit in *H. sapiens* and *T. californica*
White = Not in *H. sapiens*

103 Hypertrophic cardiomyopathy (HCM)

103.1 Human Pathway: HSA05410

103.2 Number of Hits: 8

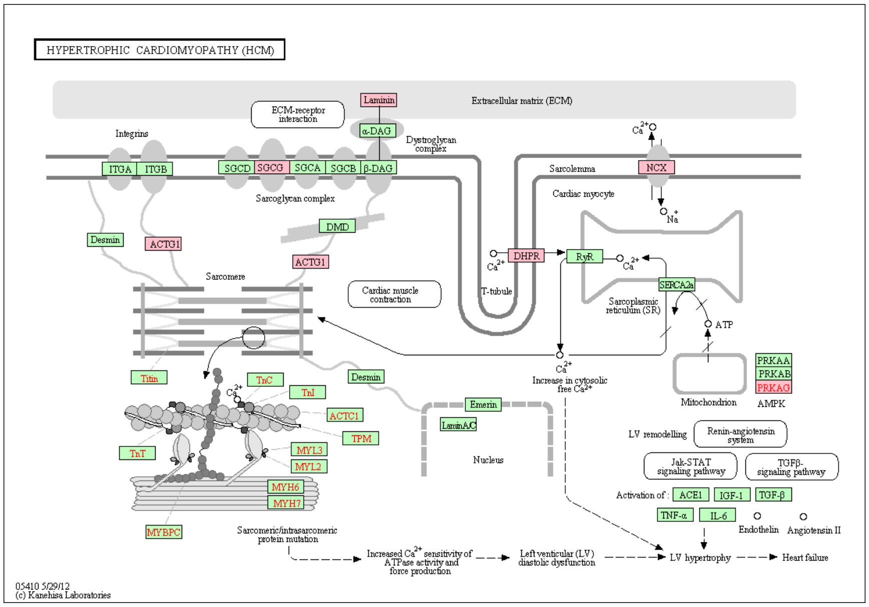

103.3 Legend:

RBH-Blast at 60% Identity + 50% Coverage
Green = Hit in *H. sapiens*
Red = Hit in *H. sapiens* and *T. californica*
White = Not in *H. sapiens*

104 Phosphatidylinositol signaling system

104.1 Human Pathway: HSA04070

104.2 Number of Hits: 8

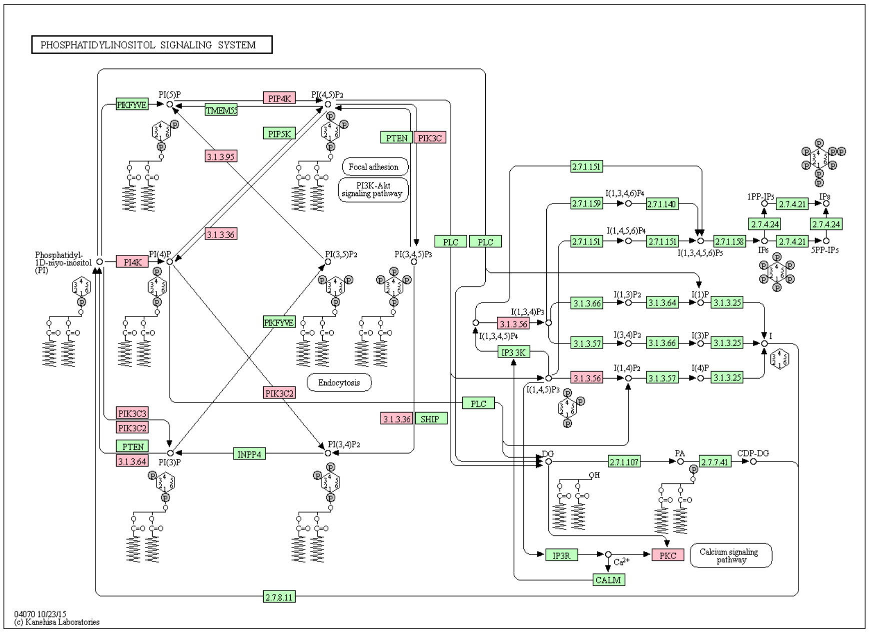

104.3 Legend:

RBH-Blast at 60% Identity + 50% Coverage
Green = Hit in *H. sapiens*
Red = Hit in *H. sapiens* and *T. californica*
White = Not in *H. sapiens*

105 Vascular smooth muscle contraction

105.1 Human Pathway: HSA04270

105.2 Number of Hits: 8

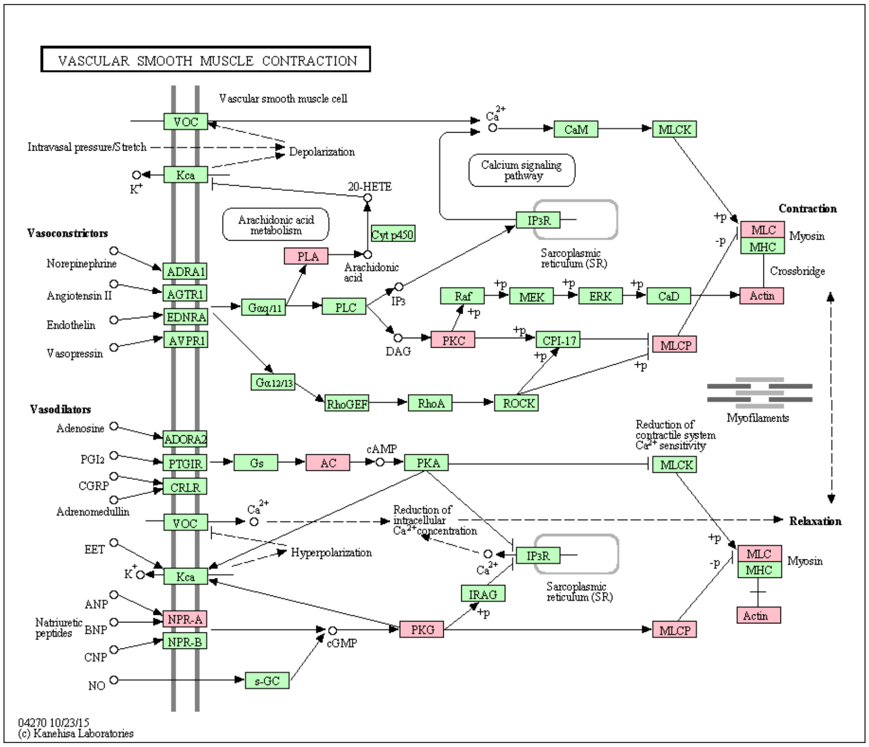

105.3 Legend:

RBH-Blast at 60% Identity + 50% Coverage
Green = Hit in *H. sapiens*
Red = Hit in *H. sapiens* and *T. californica*
White = Not in *H. sapiens*

106 Toll-like receptor signaling pathway

106.1 Human Pathway: HSA04620

106.2 Number of Hits: 8

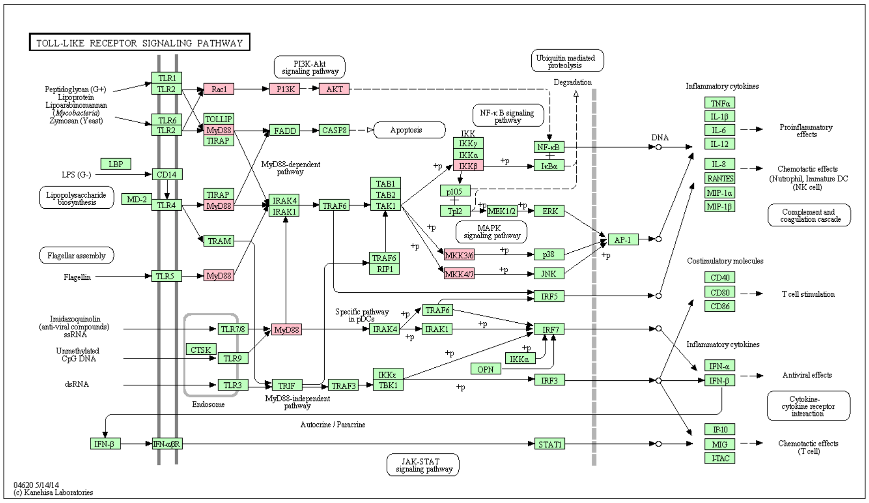

106.3 Legend:

RBH-Blast at 60% Identity + 50% Coverage
Green = Hit in *H. sapiens*
Red = Hit in *H. sapiens* and *T. californica*
White = Not in *H. sapiens*

107 Jak-STAT signaling pathway

107.1 Human Pathway: HSA04630

107.2 Number of Hits: 8

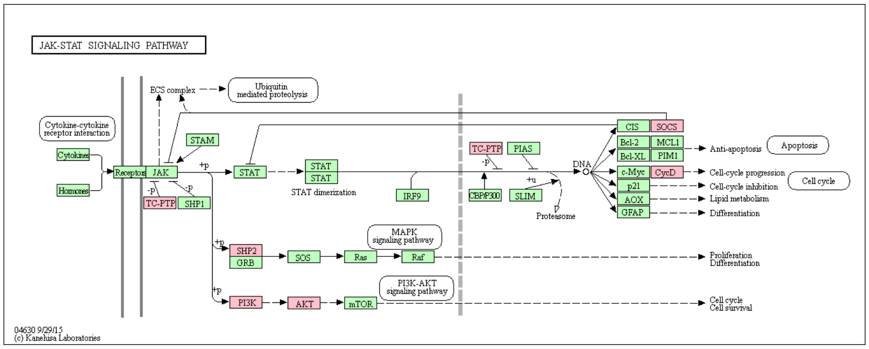

107.3 Legend:

RBH-Blast at 60% Identity + 50% Coverage
Green = Hit in *H. sapiens*
Red = Hit in *H. sapiens* and *T. californica*
White = Not in *H. sapiens*

108 Aldosterone synthesis and secretion

108.1 Human Pathway: HSA04925

108.2 Number of Hits: 8

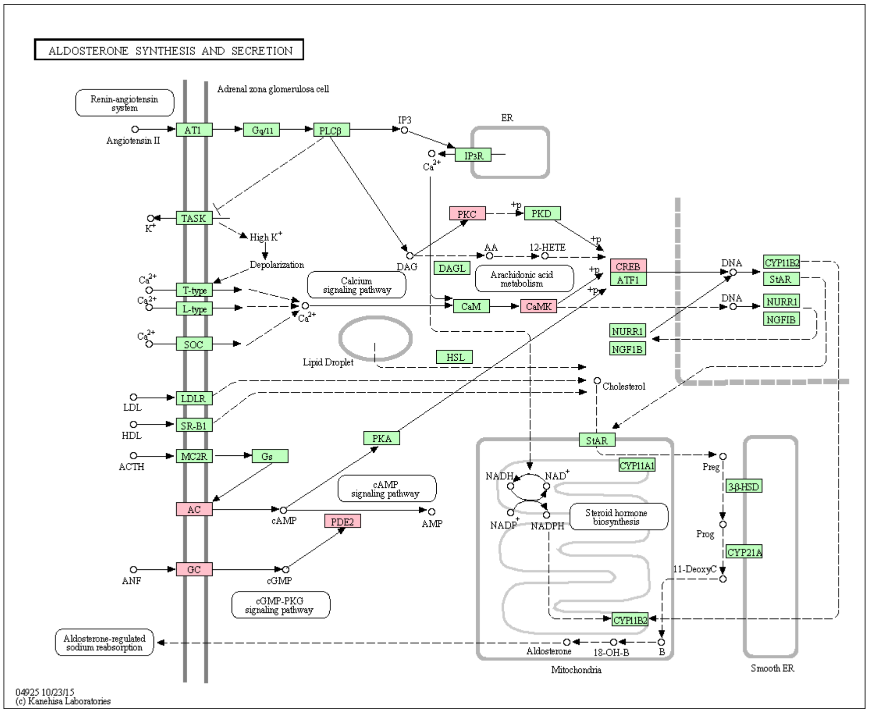

108.3 Legend:

RBH-Blast at 60% Identity + 50% Coverage
Green = Hit in *H. sapiens*
Red = Hit in *H. sapiens* and *T. californica*
White = Not in *H. sapiens*

109 Amphetamine addiction

109.1 Human Pathway: HSA05031

109.2 Number of Hits: 8

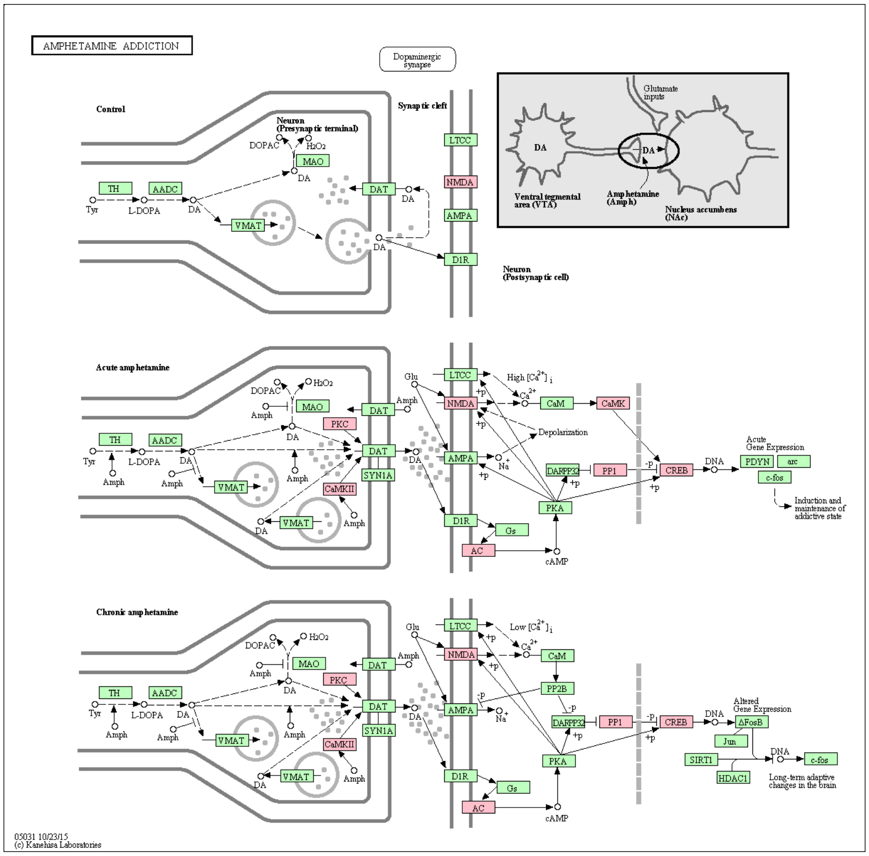

109.3 Legend:

RBH-Blast at 60% Identity + 50% Coverage
Green = Hit in *H. sapiens*
Red = Hit in *H. sapiens* and *T. californica*
White = Not in *H. sapiens*

110 Synaptic vesicle cycle

110.1 Human Pathway: HSA04721

110.2 Number of Hits: 8

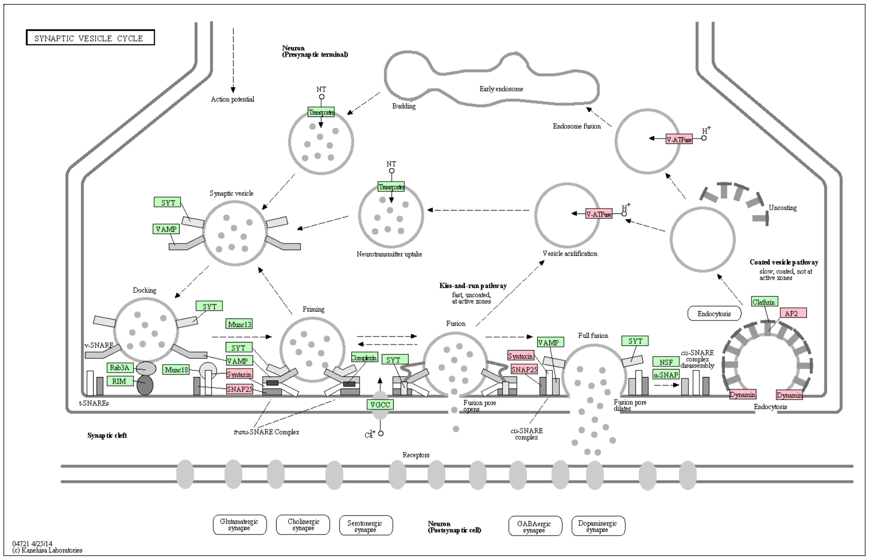

110.3 Legend:

RBH-Blast at 60% Identity + 50% Coverage
Green = Hit in *H. sapiens*
Red = Hit in *H. sapiens* and *T. californica*
White = Not in *H. sapiens*

111 Cysteine and methionine metabolism

111.1 Human Pathway: HSA00270

111.2 Number of Hits: 8

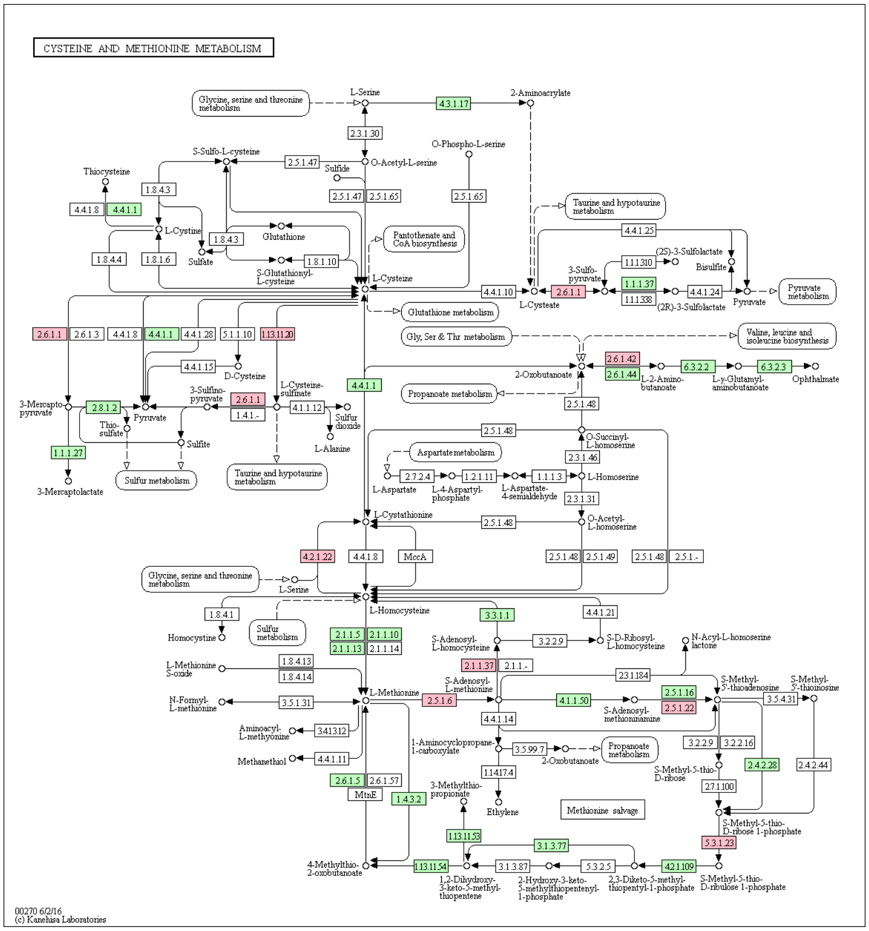

111.3 Legend:

RBH-Blast at 60% Identity + 50% Coverage
Green = Hit in *H. sapiens*
Red = Hit in *H. sapiens* and *T. californica*
White = Not in *H. sapiens*

112 MicroRNAs in cancer

112.1 Human Pathway: HSA05206

112.2 Number of Hits: 8

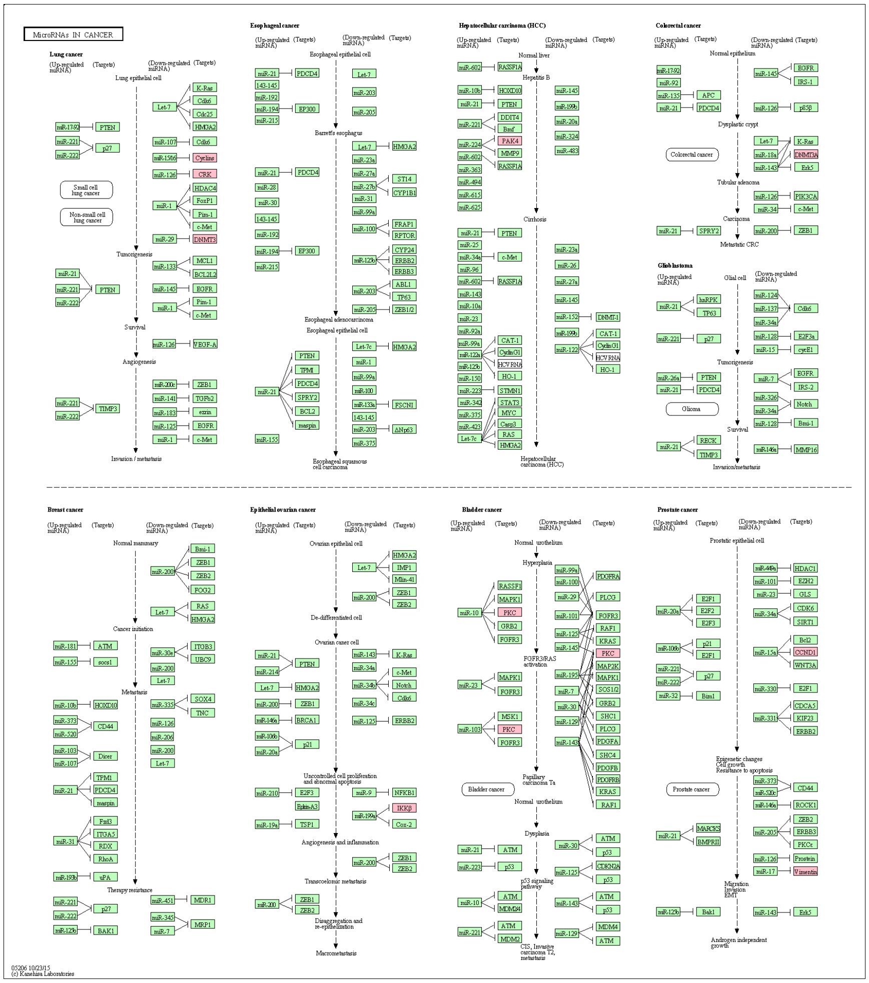

112.3 Legend:

RBH-Blast at 60% Identity + 50% Coverage
Green = Hit in *H. sapiens*
Red = Hit in *H. sapiens* and *T. californica*
White = Not in *H. sapiens*

113 Inflammatory mediator regulation of TRP channels

113.1 Human Pathway: HSA04750

113.2 Number of Hits: 8

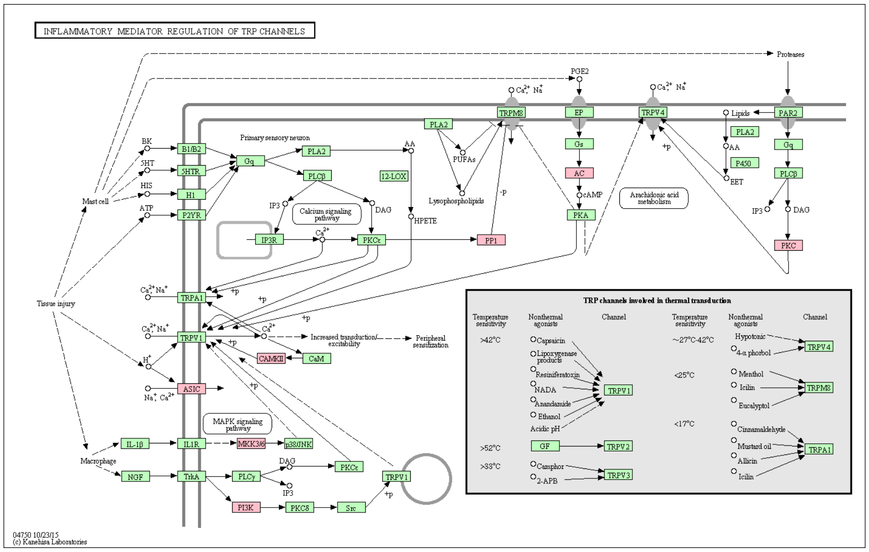

113.3 Legend:

RBH-Blast at 60% Identity + 50% Coverage
Green = Hit in *H. sapiens*
Red = Hit in *H. sapiens* and *T. californica*
White = Not in *H. sapiens*

114 Cardiac muscle contraction

114.1 Human Pathway: HSA04260

114.2 Number of Hits: 8

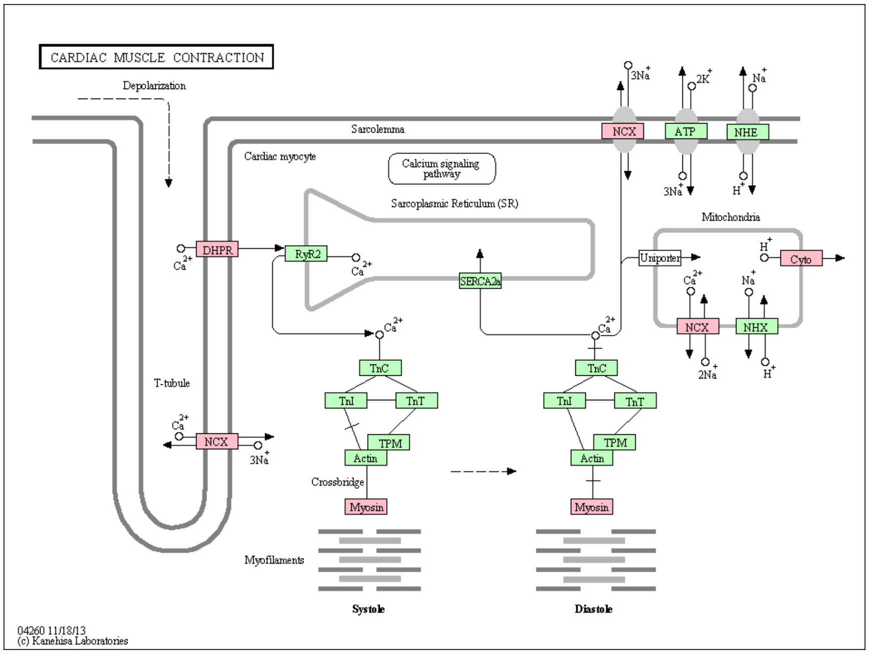

114.3 Legend:

RBH-Blast at 60% Identity + 50% Coverage
Green = Hit in *H. sapiens*
Red = Hit in *H. sapiens* and *T. californica*
White = Not in *H. sapiens*

115 Amoebiasis

115.1 Human Pathway: HSA05146

115.2 Number of Hits: 8

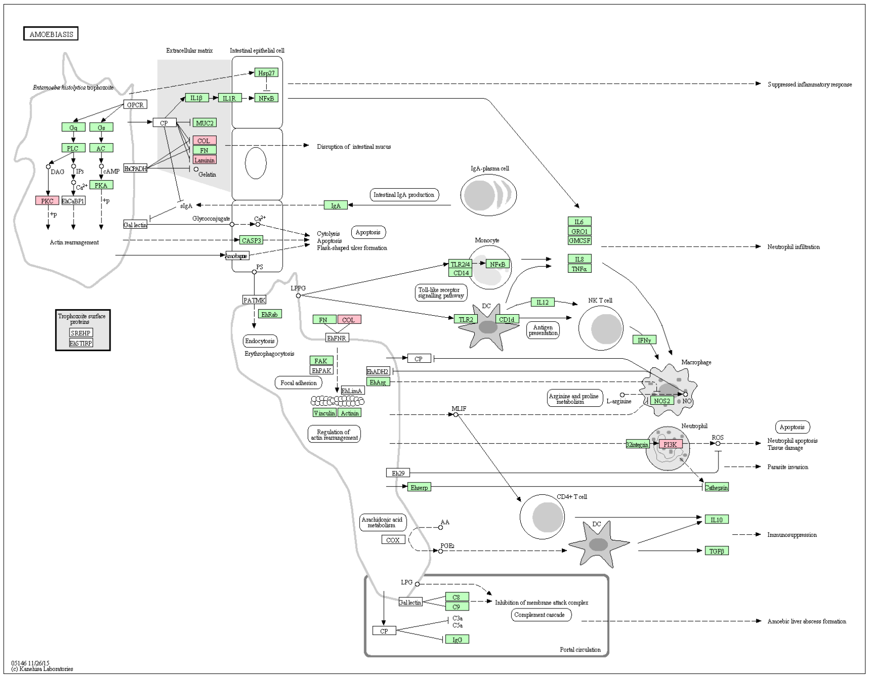

115.3 Legend:

RBH-Blast at 60% Identity + 50% Coverage
Green = Hit in *H. sapiens*
Red = Hit in *H. sapiens* and *T. californica*
White = Not in *H. sapiens*

116 Dilated cardiomyopathy

116.1 Human Pathway: HSA05414

116.2 Number of Hits: 7

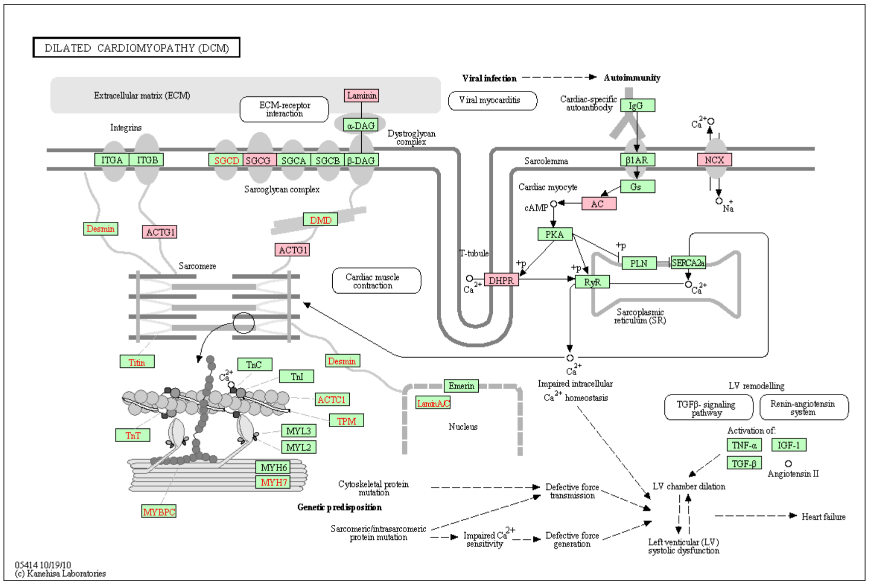

116.3 Legend:

RBH-Blast at 60% Identity + 50% Coverage
Green = Hit in *H. sapiens*
Red = Hit in *H. sapiens* and *T. californica*
White = Not in *H. sapiens*

117 Progesterone-mediated oocyte maturation

117.1 Human Pathway: HSA04914

117.2 Number of Hits: 7

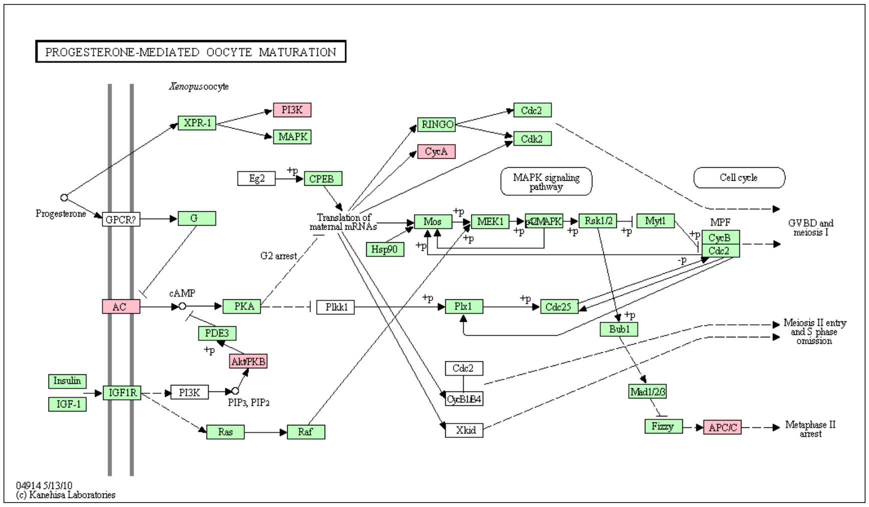

117.3 Legend:

RBH-Blast at 60% Identity + 50% Coverage
Green = Hit in *H. sapiens*
Red = Hit in *H. sapiens* and *T. californica*
White = Not in *H. sapiens*

118 Protein digestion and absorption

118.1 Human Pathway: HSA04974

118.2 Number of Hits: 7

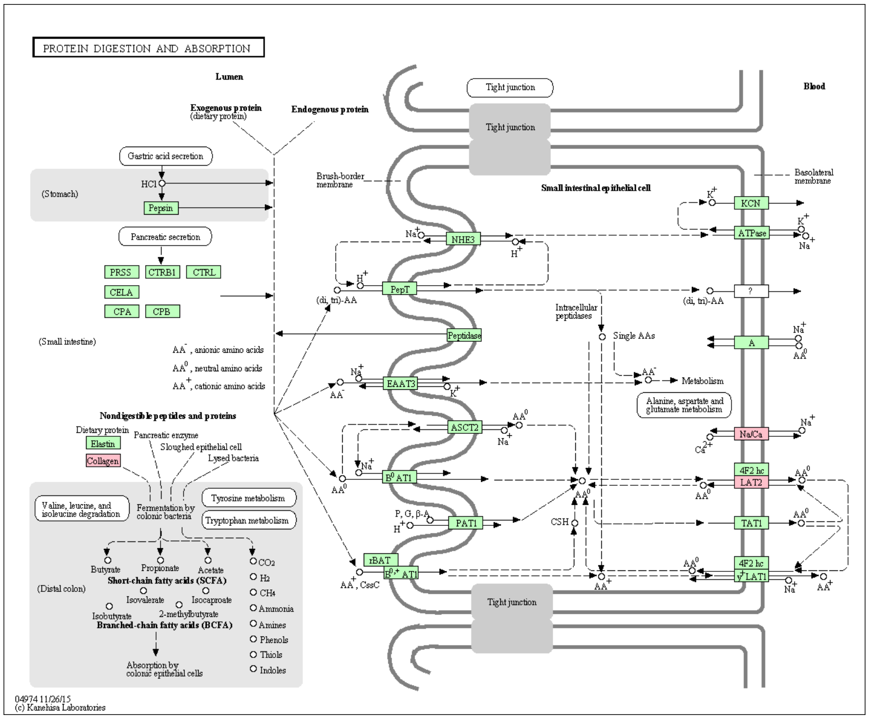

118.3 Legend:

RBH-Blast at 60% Identity + 50% Coverage
Green = Hit in *H. sapiens*
Red = Hit in *H. sapiens* and *T. californica*
White = Not in *H. sapiens*

119 Melanogenesis

119.1 Human Pathway: HSA04916

119.2 Number of Hits: 7

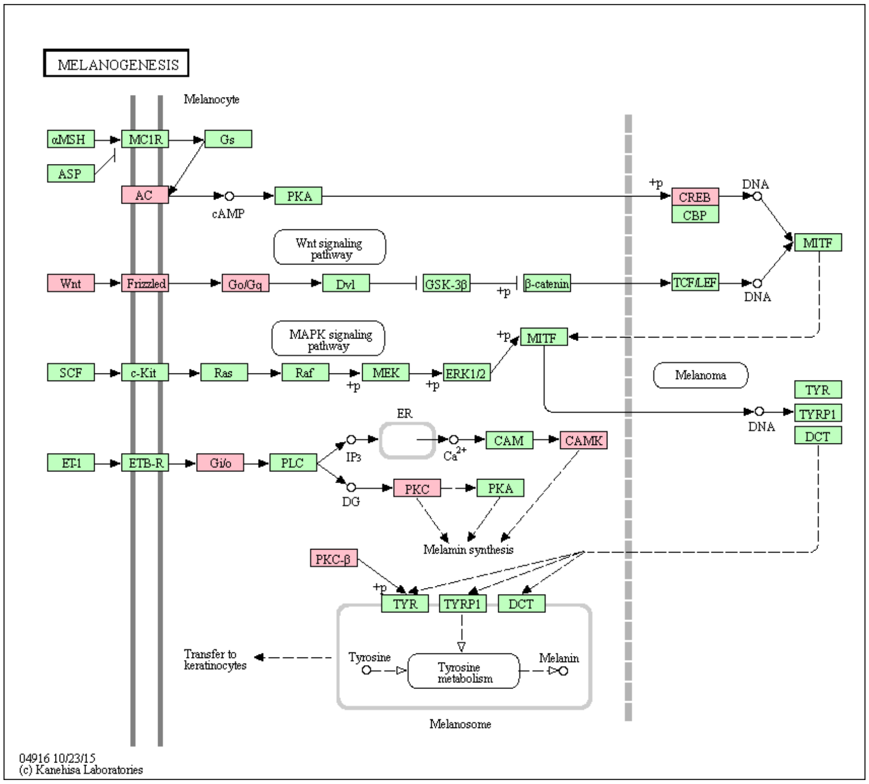

119.3 Legend:

RBH-Blast at 60% Identity + 50% Coverage
Green = Hit in *H. sapiens*
Red = Hit in *H. sapiens* and *T. californica*
White = Not in *H. sapiens*

120 Colorectal cancer

120.1 Human Pathway: HSA05210

120.2 Number of Hits: 7

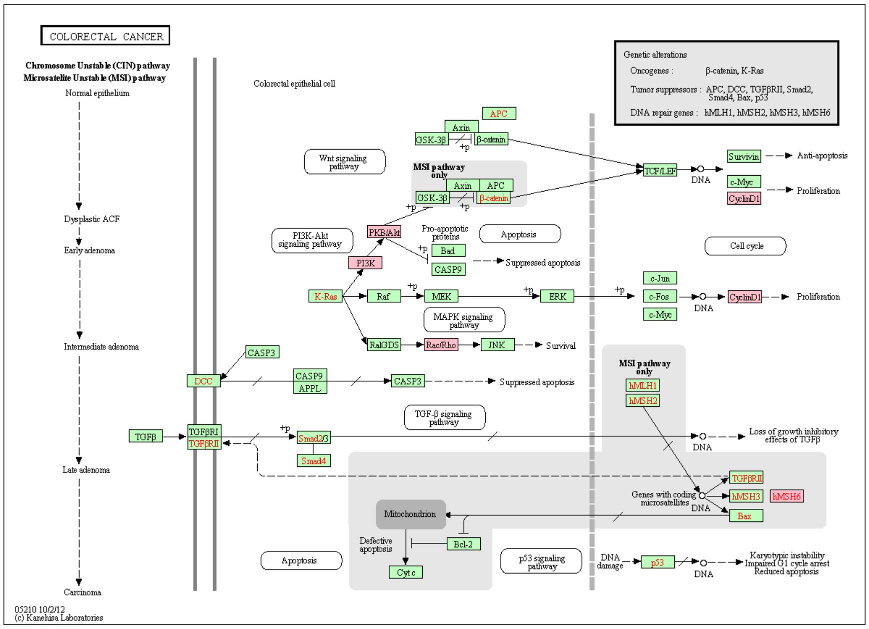

120.3 Legend:

RBH-Blast at 60% Identity + 50% Coverage
Green = Hit in *H. sapiens*
Red = Hit in *H. sapiens* and *T. californica*
White = Not in *H. sapiens*

121 DNA replication

121.1 Human Pathway: HSA03030

121.2 Number of Hits: 7

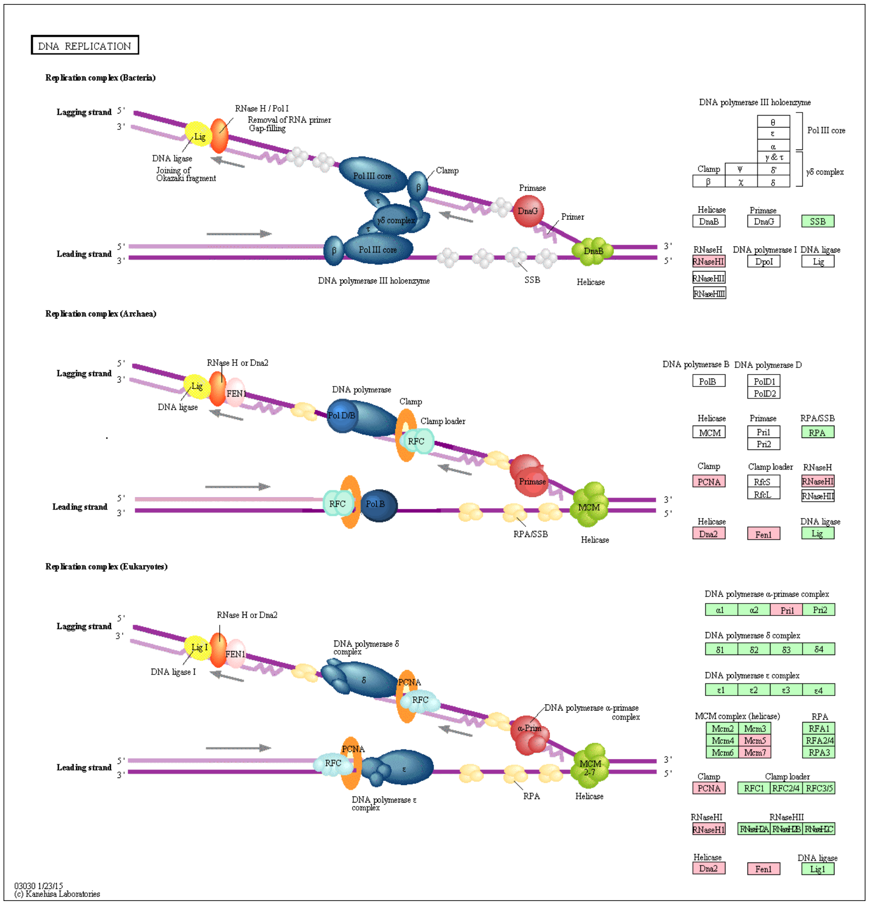

121.3 Legend:

RBH-Blast at 60% Identity + 50% Coverage
Green = Hit in *H. sapiens*
Red = Hit in *H. sapiens* and *T. californica*
White = Not in *H. sapiens*

122 Fatty acid metabolism

122.1 Human Pathway: HSA01212

122.2 Number of Hits: 7

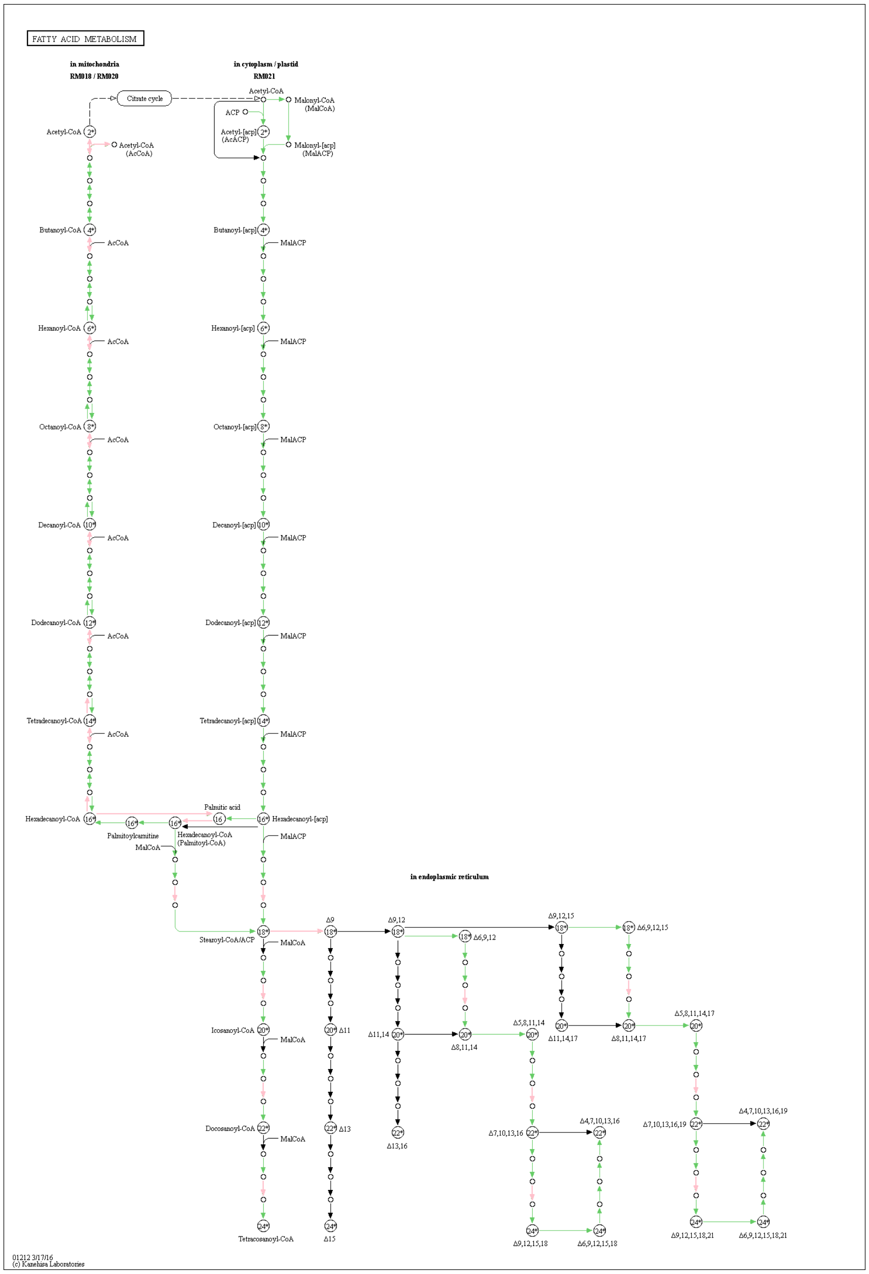

122.3 Legend:

RBH-Blast at 60% Identity + 50% Coverage
Green = Hit in *H. sapiens*
Red = Hit in *H. sapiens* and *T. californica*
White = Not in *H. sapiens*

---

123 Circadian rhythm

123.1 Human Pathway: HSA04710

123.2 Number of Hits: 7

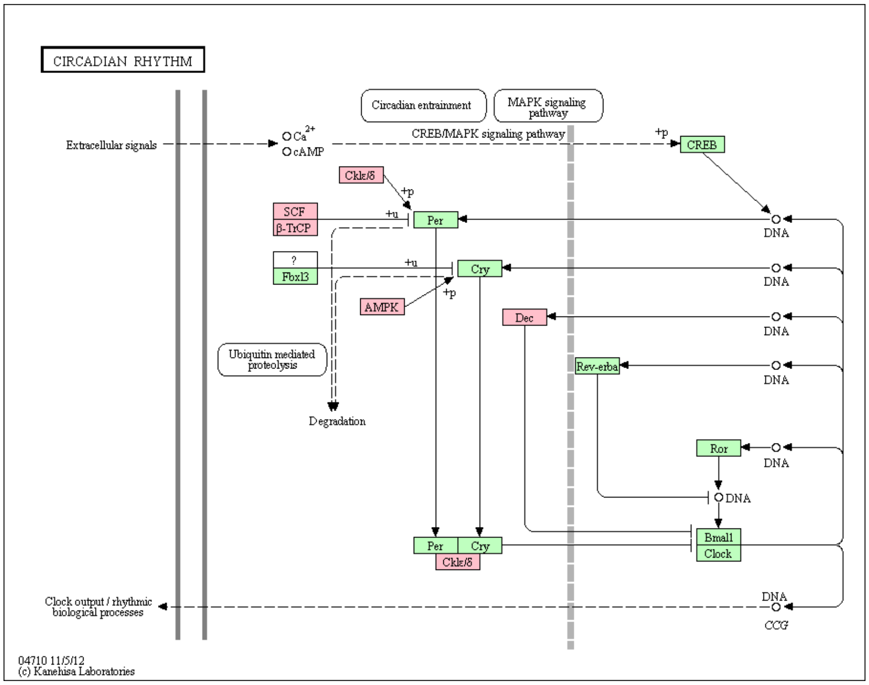

123.3 Legend:

RBH-Blast at 60% Identity + 50% Coverage
Green = Hit in *H. sapiens*
Red = Hit in *H. sapiens* and *T. californica*
White = Not in *H. sapiens*

---

124 Amino sugar and nucleotide sugar metabolism

124.1 Human Pathway: HSA00520

124.2 Number of Hits: 7

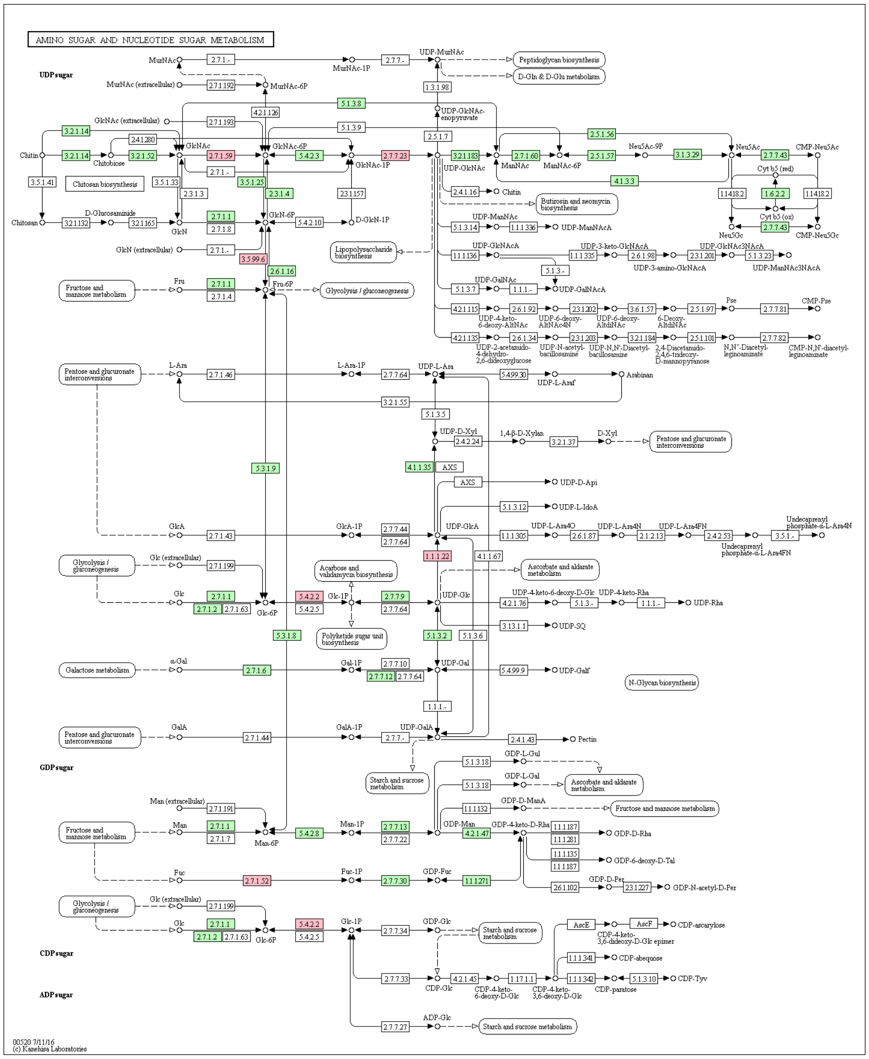

124.3 Legend:

RBH-Blast at 60% Identity + 50% Coverage
Green = Hit in *H. sapiens*
Red = Hit in *H. sapiens* and *T. californica*
White = Not in *H. sapiens*

---

125 Basal transcription factors

125.1 Human Pathway: HSA03022

125.2 Number of Hits: 7

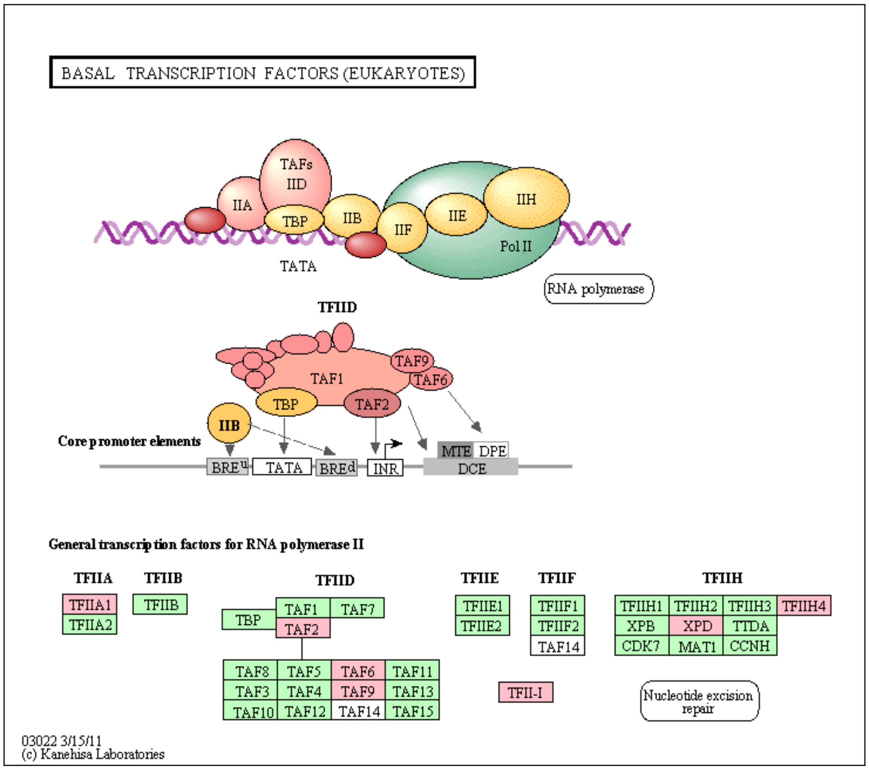

125.3 Legend:

RBH-Blast at 60% Identity + 50% Coverage
Green = Hit in *H. sapiens*
Red = Hit in *H. sapiens* and *T. californica*
White = Not in *H. sapiens*

126 TGF-beta signaling pathway

126.1 Human Pathway: HSA04350

126.2 Number of Hits: 7

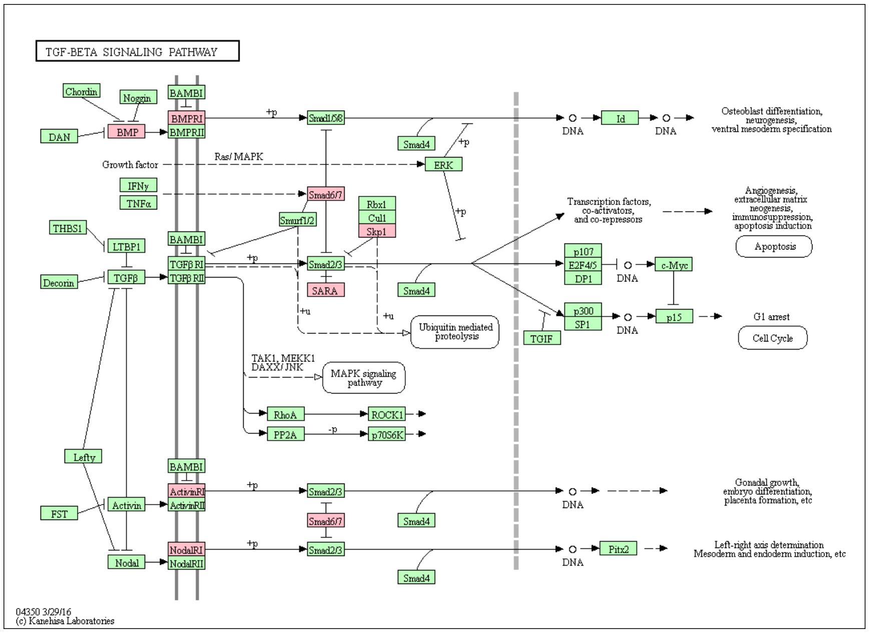

126.3 Legend:

RBH-Blast at 60% Identity + 50% Coverage
Green = Hit in *H. sapiens*
Red = Hit in *H. sapiens* and *T. californica*
White = Not in *H. sapiens*

127 Peroxisome

127.1 Human Pathway: HSA04146

127.2 Number of Hits: 7

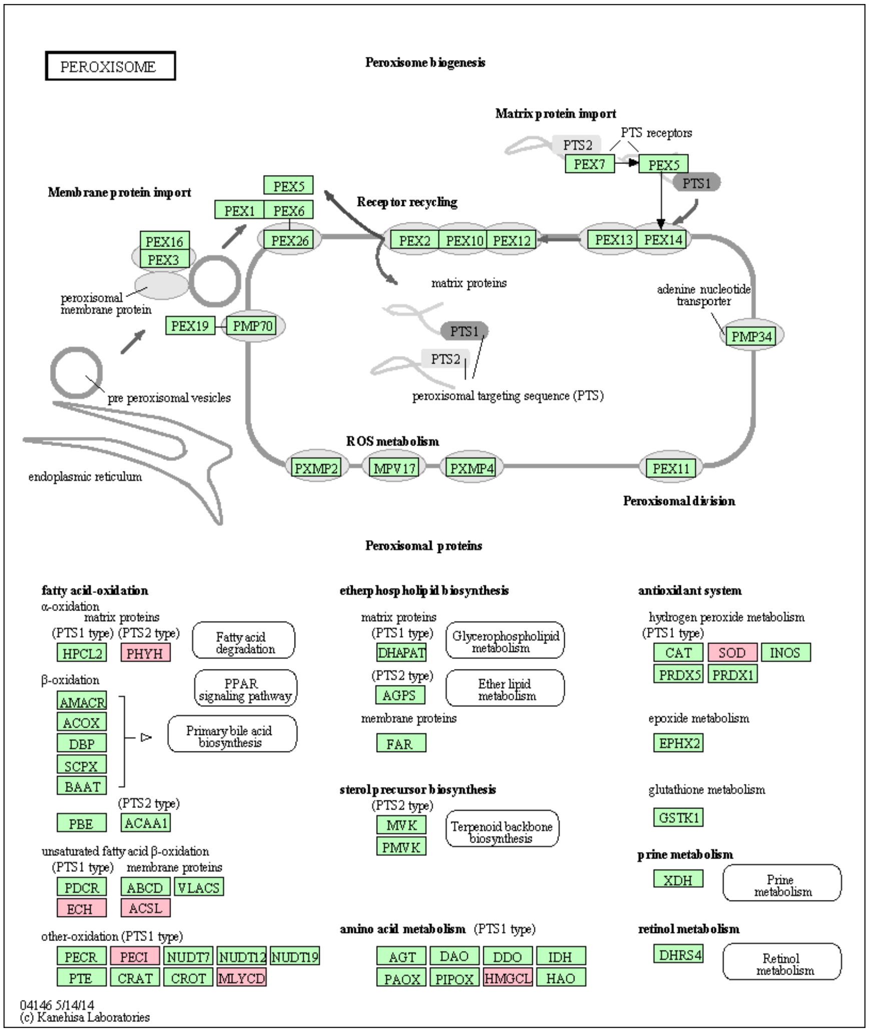

127.3 Legend:

RBH-Blast at 60% Identity + 50% Coverage
Green = Hit in *H. sapiens*
Red = Hit in *H. sapiens* and *T. californica*
White = Not in *H. sapiens*

128 Epithelial cell signaling in Helicobacter pylori infection

128.1 Human Pathway: HSA05120

128.2 Number of Hits: 7

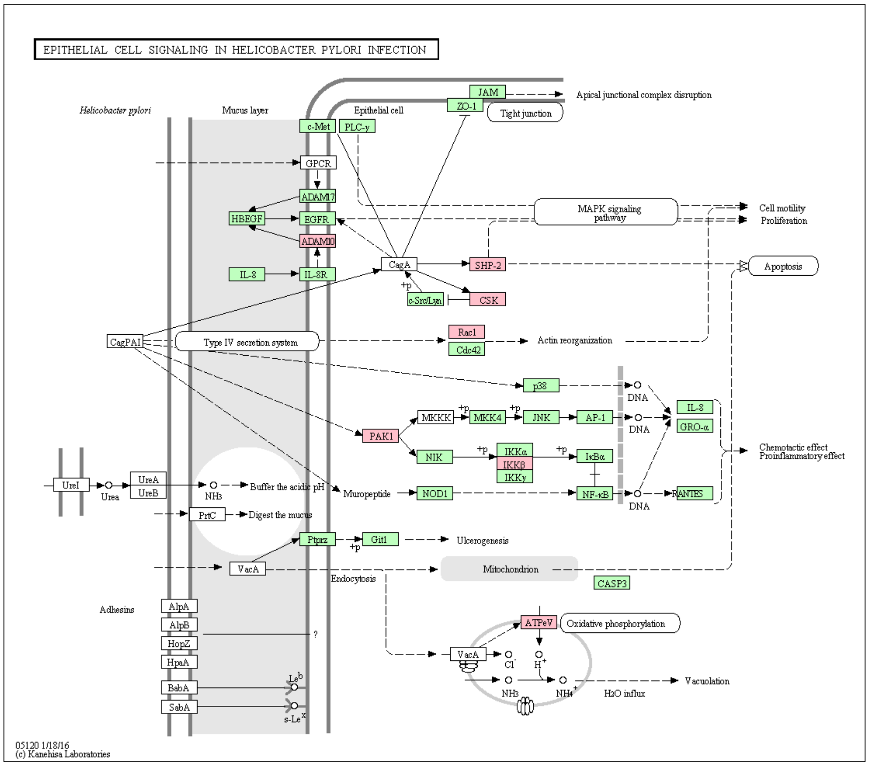

128.3 Legend:

RBH-Blast at 60% Identity + 50% Coverage
Green = Hit in *H. sapiens*
Red = Hit in *H. sapiens* and *T. californica*
White = Not in *H. sapiens*

129 Starch and sucrose metabolism

129.1 Human Pathway: HSA00500

129.2 Number of Hits: 7

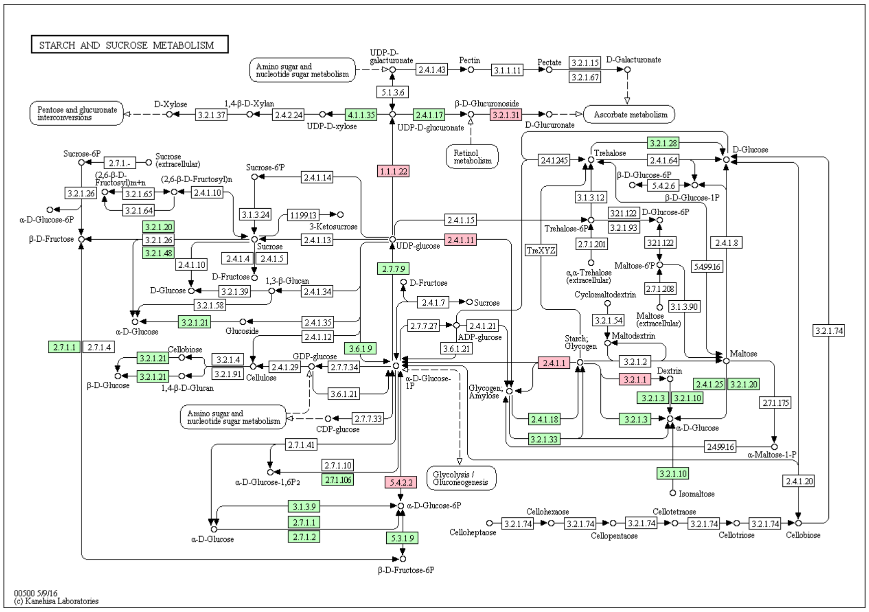

129.3 Legend:

RBH-Blast at 60% Identity + 50% Coverage
Green = Hit in *H. sapiens*
Red = Hit in *H. sapiens* and *T. californica*
White = Not in *H. sapiens*

130 Glycerophospholipid metabolism

130.1 Human Pathway: HSA00564

130.2 Number of Hits: 7

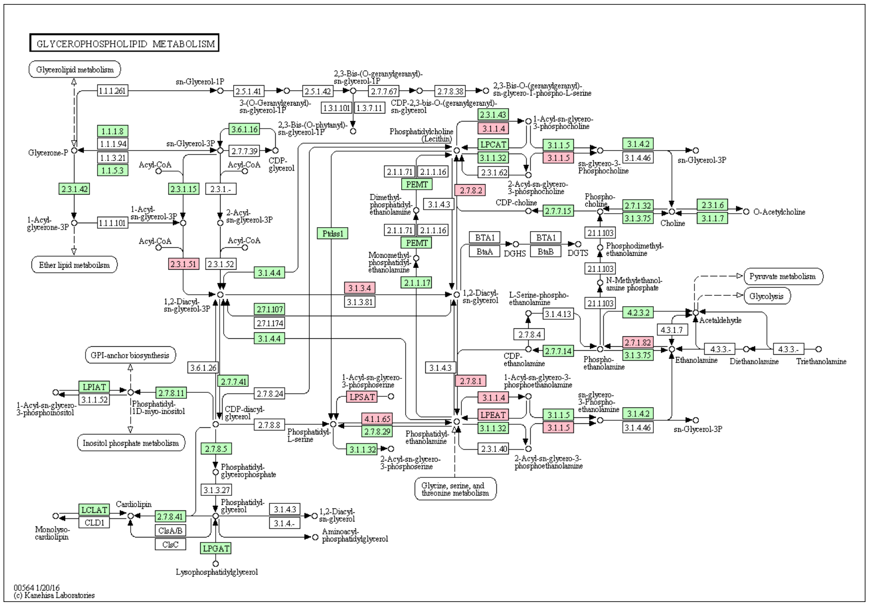

130.3 Legend:

RBH-Blast at 60% Identity + 50% Coverage
Green = Hit in *H. sapiens*
Red = Hit in *H. sapiens* and *T. californica*
White = Not in *H. sapiens*

131 Gap junction

131.1 Human Pathway: HSA04540

131.2 Number of Hits: 7

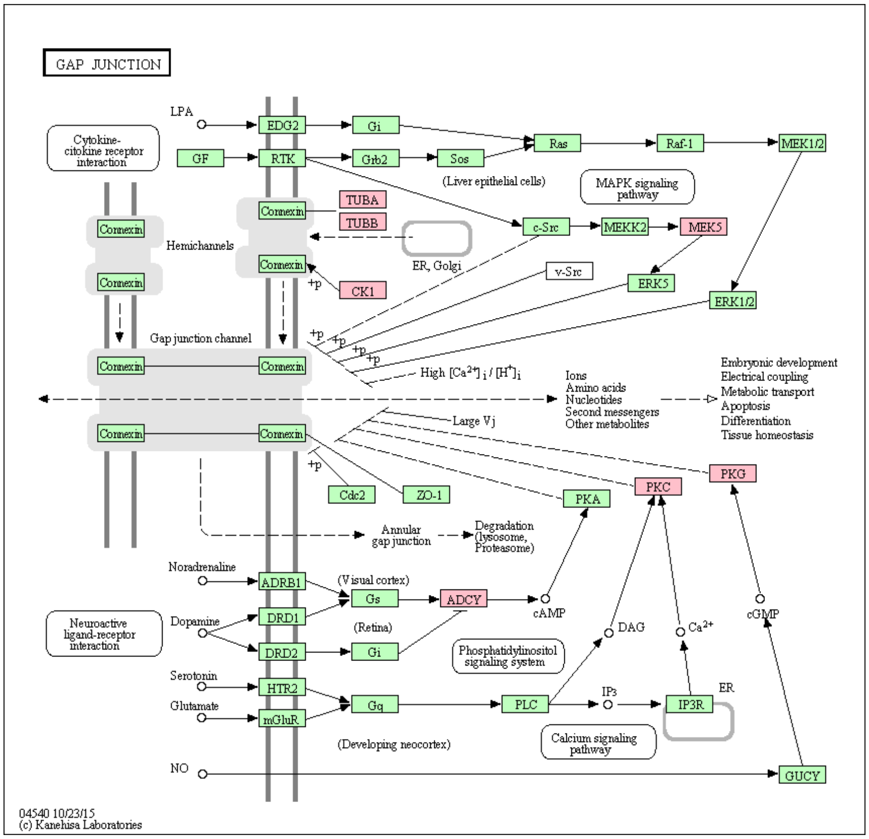

131.3 Legend:

RBH-Blast at 60% Identity + 50% Coverage
Green = Hit in *H. sapiens*
Red = Hit in *H. sapiens* and *T. californica*
White = Not in *H. sapiens*

132 Tuberculosis

132.1 Human Pathway: HSA05152

132.2 Number of Hits: 7

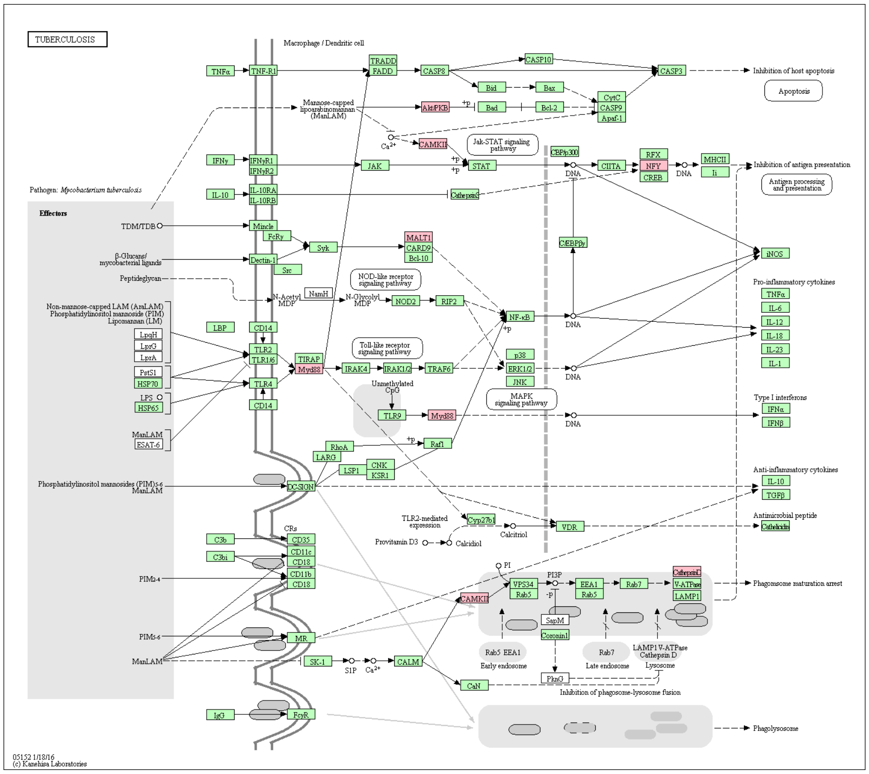

132.3 Legend:

RBH-Blast at 60% Identity + 50% Coverage
Green = Hit in *H. sapiens*
Red = Hit in *H. sapiens* and *T. californica*
White = Not in *H. sapiens*

133 Transcriptional misregulation in cancer

133.1 Human Pathway: HSA05202

133.2 Number of Hits: 7

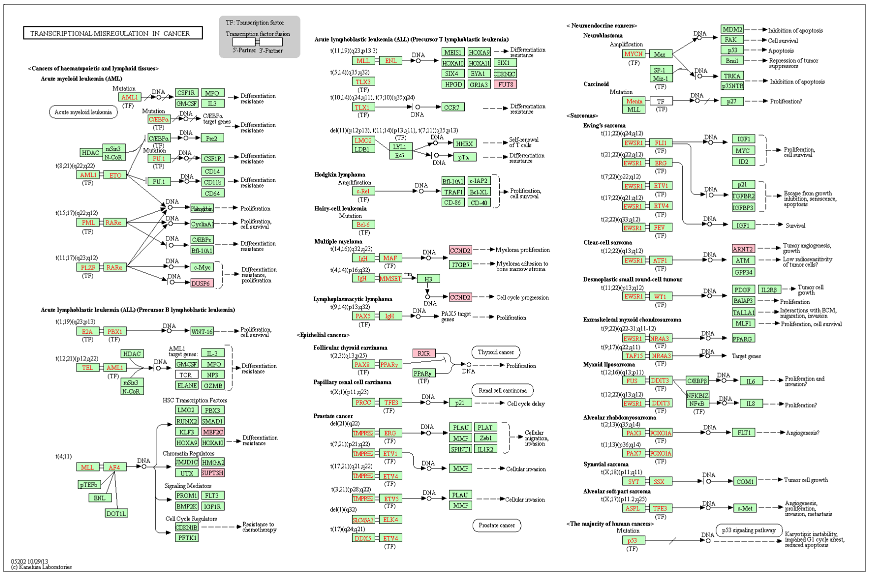

133.3 Legend:

RBH-Blast at 60% Identity + 50% Coverage
Green = Hit in *H. sapiens*
Red = Hit in *H. sapiens* and *T. californica*
White = Not in *H. sapiens*

134 Prolactin signaling pathway

134.1 Human Pathway: HSA04917

134.2 Number of Hits: 7

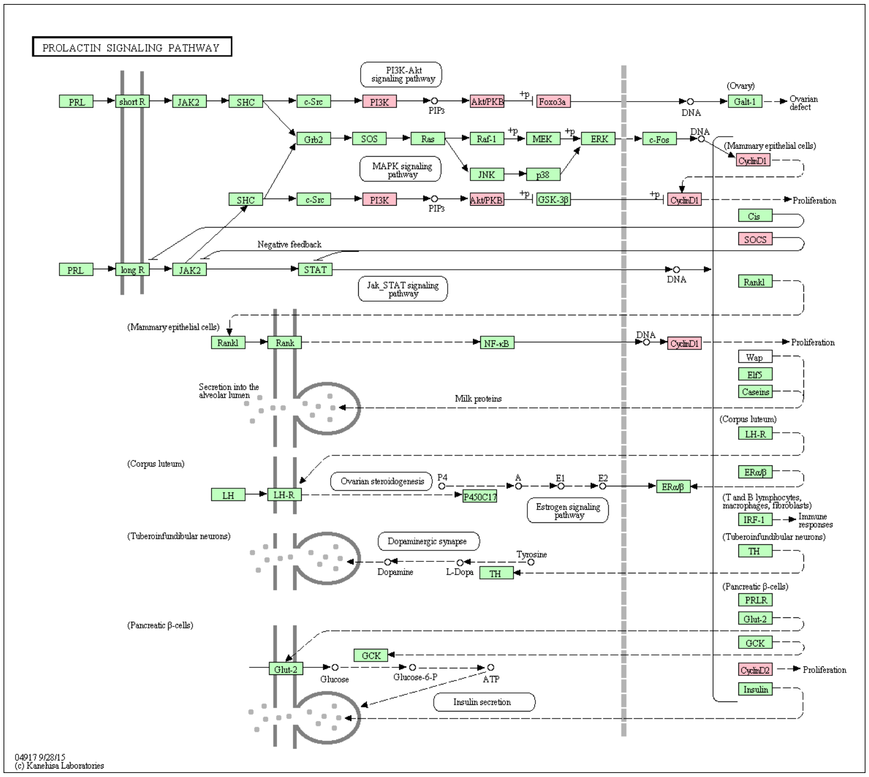

134.3 Legend:

RBH-Blast at 60% Identity + 50% Coverage
Green = Hit in *H. sapiens*
Red = Hit in *H. sapiens* and *T. californica*
White = Not in *H. sapiens*

135 Glycerolipid metabolism

135.1 Human Pathway: HSA00561

135.2 Number of Hits: 7

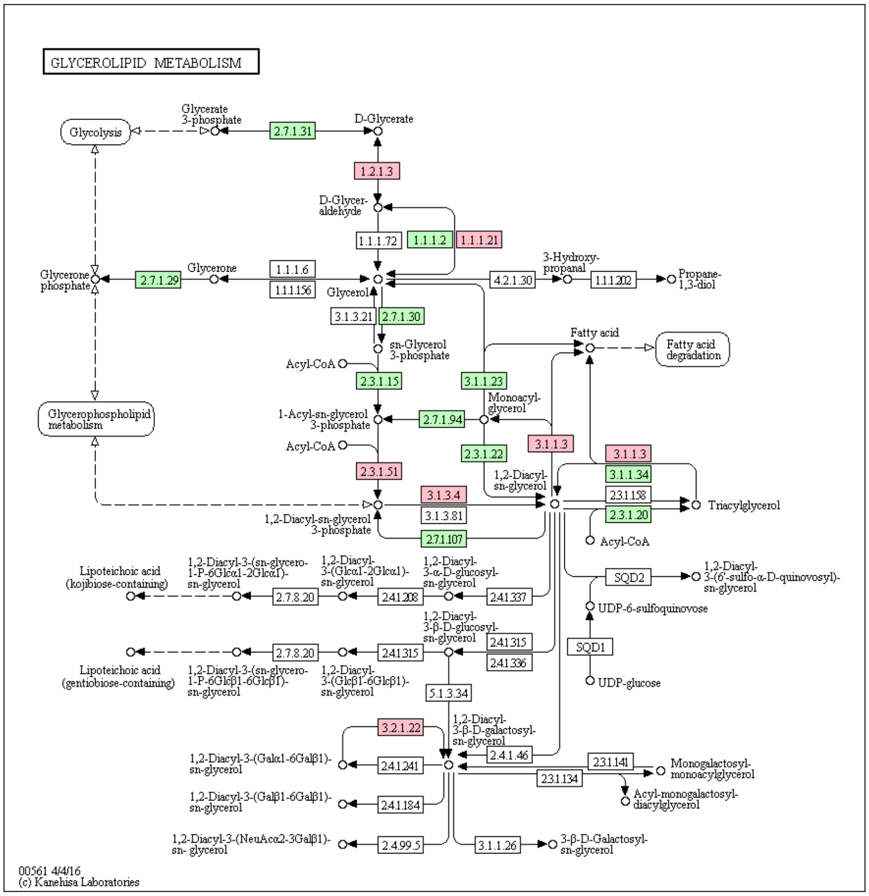

135.3 Legend:

RBH-Blast at 60% Identity + 50% Coverage
Green = Hit in *H. sapiens*
Red = Hit in *H. sapiens* and *T. californica*
White = Not in *H. sapiens*

136 Non-small cell lung cancer

136.1 Human Pathway: HSA05223

136.2 Number of Hits: 7

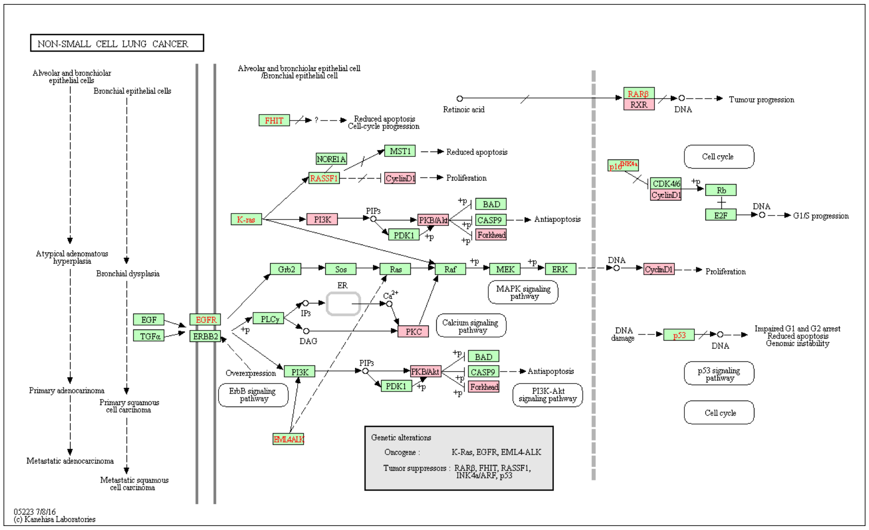

136.3 Legend:

RBH-Blast at 60% Identity + 50% Coverage
Green = Hit in *H. sapiens*
Red = Hit in *H. sapiens* and *T. californica*
White = Not in *H. sapiens*

137 Pentose and glucuronate interconversions

137.1 Human Pathway: HSA00040

137.2 Number of Hits: 7

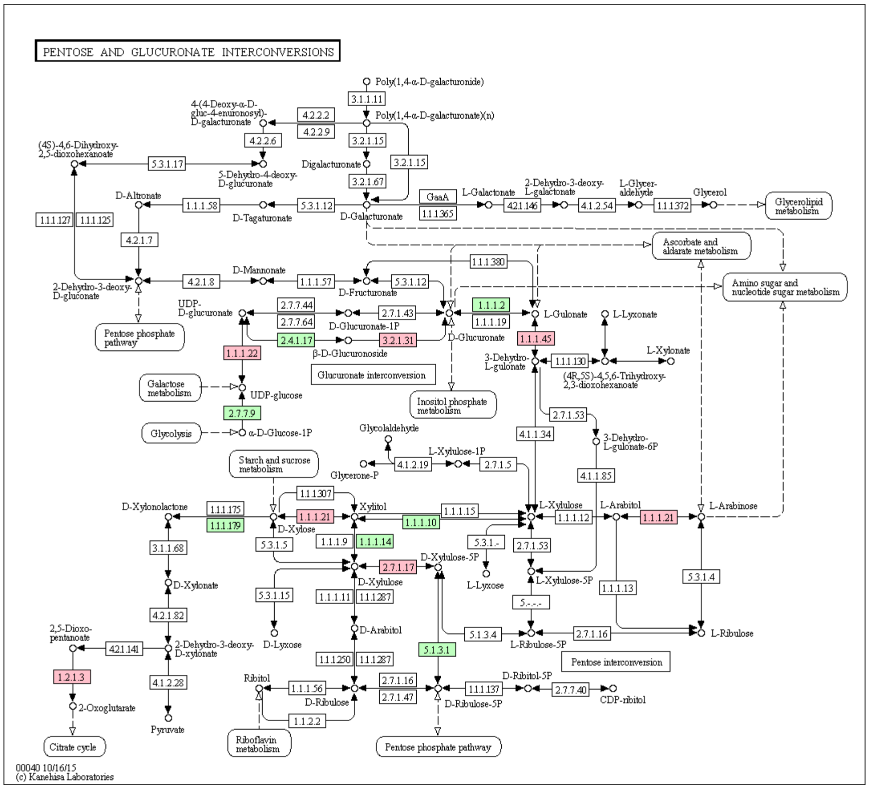

137.3 Legend:

RBH-Blast at 60% Identity + 50% Coverage
Green = Hit in *H. sapiens*
Red = Hit in *H. sapiens* and *T. californica*
White = Not in *H. sapiens*

138 Central carbon metabolism in cancer

138.1 Human Pathway: HSA05230

138.2 Number of Hits: 7

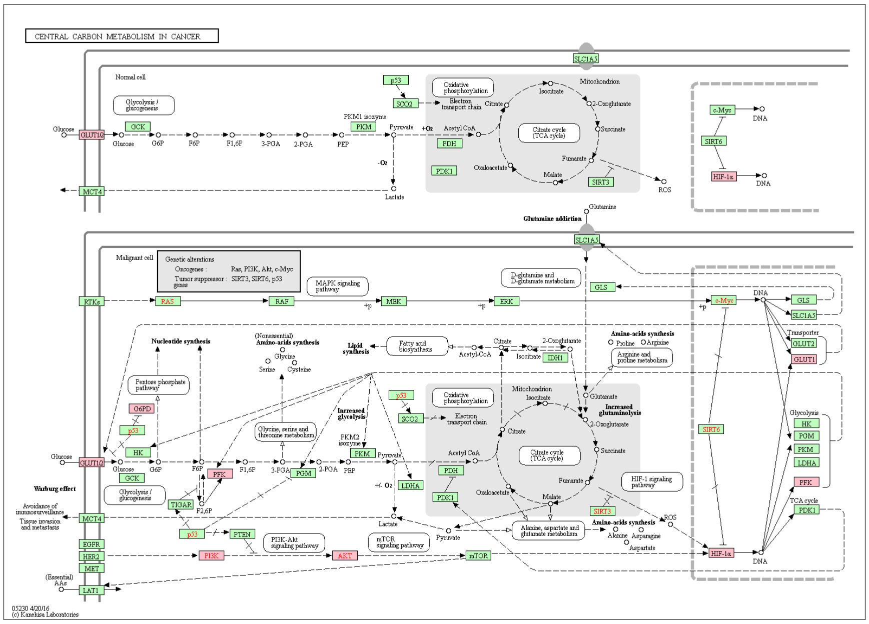

138.3 Legend:

RBH-Blast at 60% Identity + 50% Coverage
Green = Hit in *H. sapiens*
Red = Hit in *H. sapiens* and *T. californica*
White = Not in *H. sapiens*

139 Arrhythmogenic right ventricular cardiomyopathy (ARVC)

139.1 Human Pathway: HSA05412

139.2 Number of Hits: 7

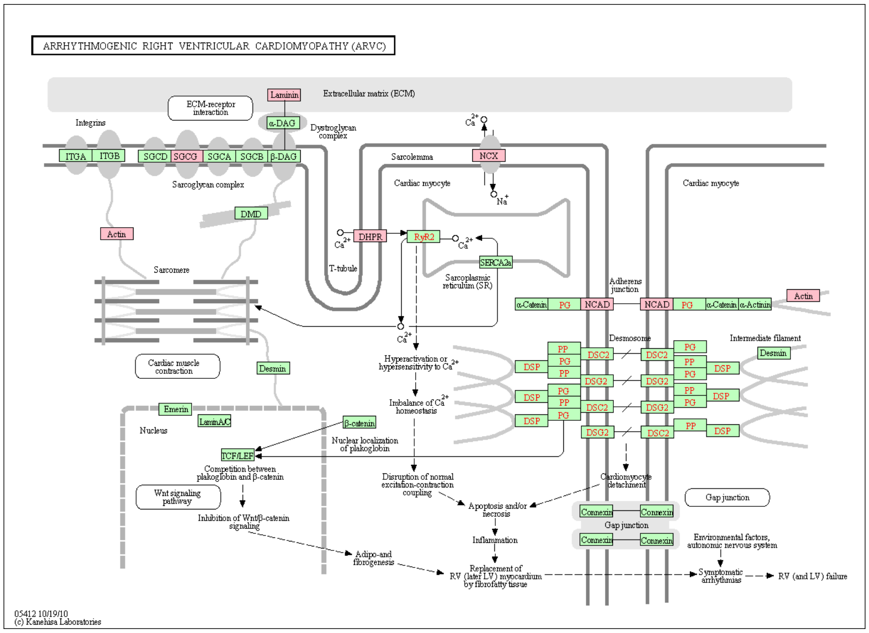

139.3 Legend:

RBH-Blast at 60% Identity + 50% Coverage
Green = Hit in *H. sapiens*
Red = Hit in *H. sapiens* and *T. californica*
White = Not in *H. sapiens*

140 p53 signaling pathway

140.1 Human Pathway: HSA04115

140.2 Number of Hits: 6

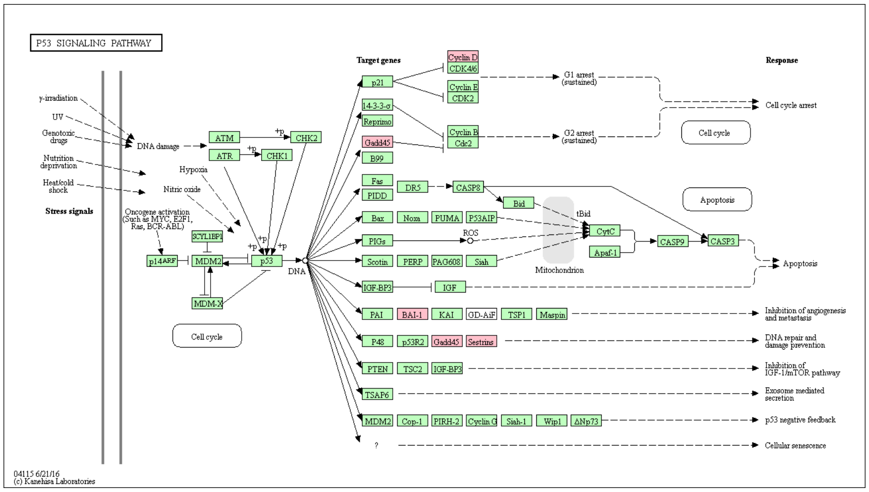

140.3 Legend:

RBH-Blast at 60% Identity + 50% Coverage
Green = Hit in *H. sapiens*
Red = Hit in *H. sapiens* and *T. californica*
White = Not in *H. sapiens*

141 Long-term potentiation

141.1 Human Pathway: HSA04720

141.2 Number of Hits: 6

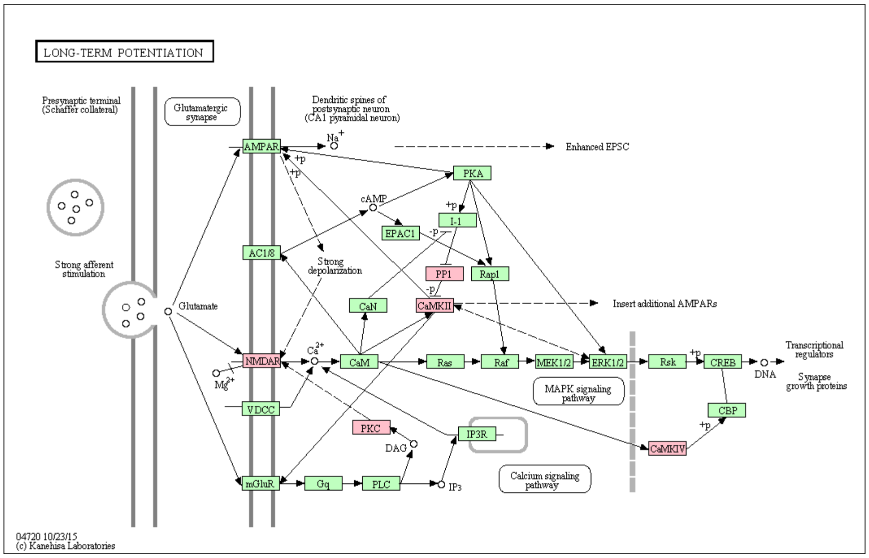

141.3 Legend:

RBH-Blast at 60% Identity + 50% Coverage
Green = Hit in *H. sapiens*
Red = Hit in *H. sapiens* and *T. californica*
White = Not in *H. sapiens*

142 VEGF signaling pathway

142.1 Human Pathway: HSA04370

142.2 Number of Hits: 6

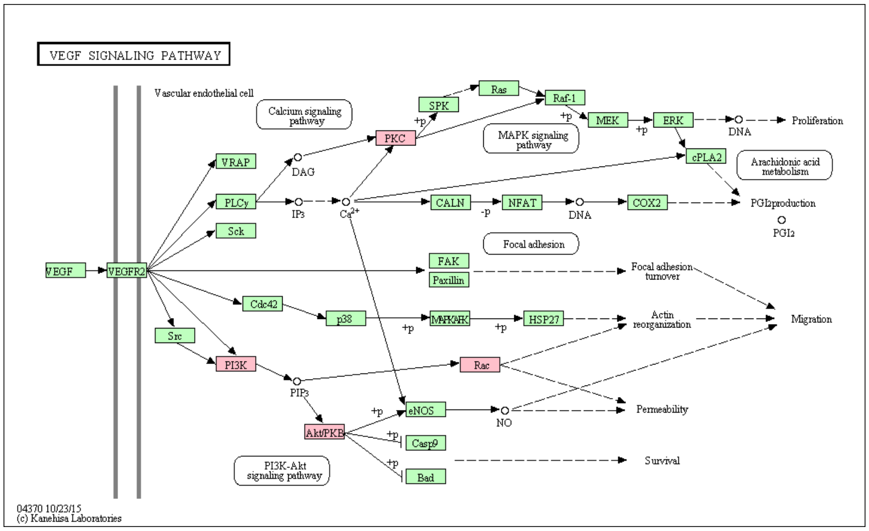

142.3 Legend:

RBH-Blast at 60% Identity + 50% Coverage
Green = Hit in *H. sapiens*
Red = Hit in *H. sapiens* and *T. californica*
White = Not in *H. sapiens*

143 Measles

143.1 Human Pathway: HSA05162

143.2 Number of Hits: 6

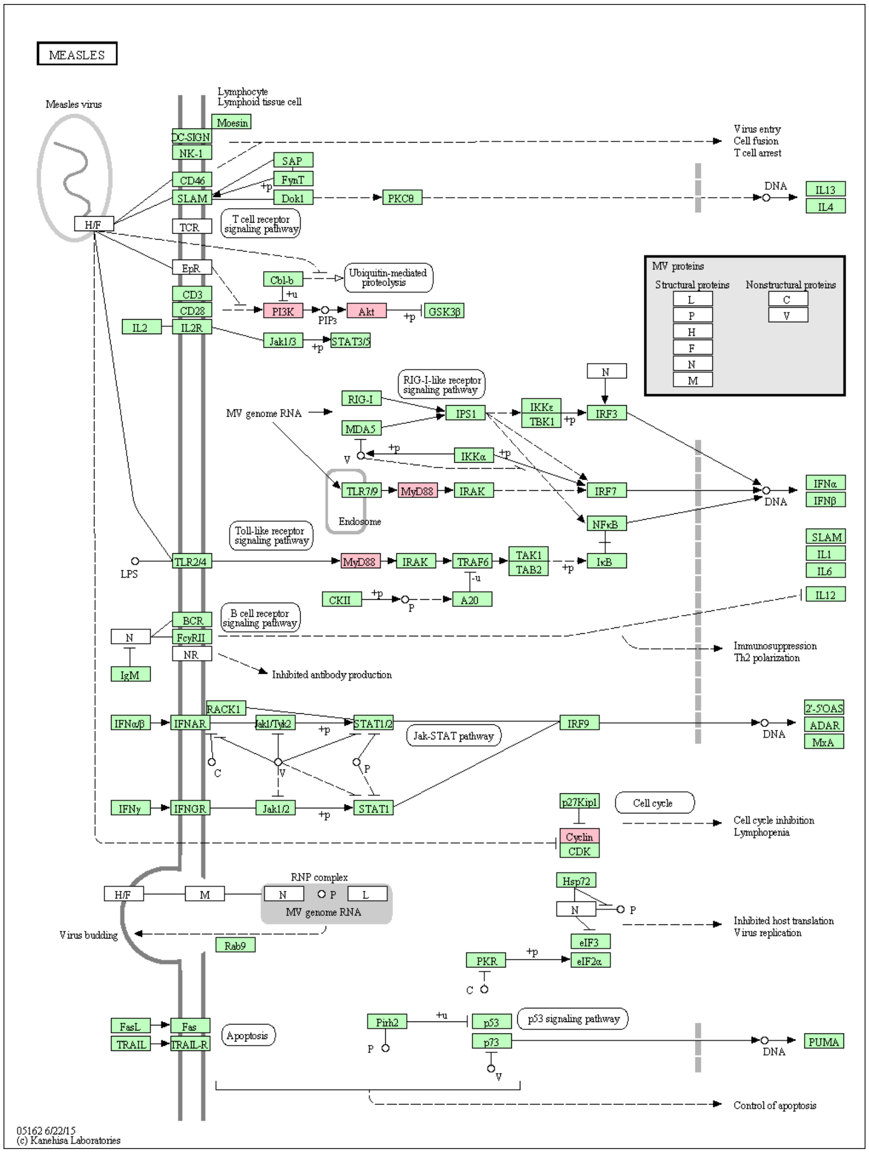

143.3 Legend:

RBH-Blast at 60% Identity + 50% Coverage
Green = Hit in *H. sapiens*
Red = Hit in *H. sapiens* and *T. californica*
White = Not in *H. sapiens*

144 Propanoate metabolism

144.1 Human Pathway: HSA00640

144.2 Number of Hits: 6

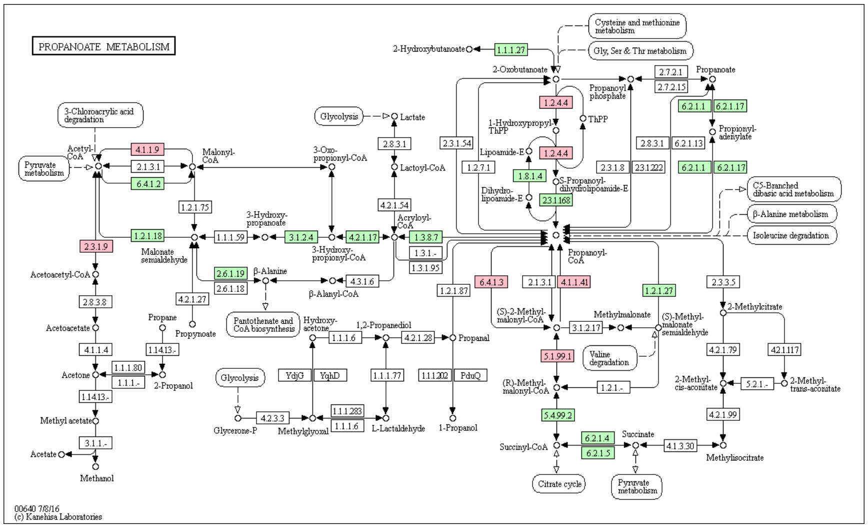

144.3 Legend:

RBH-Blast at 60% Identity + 50% Coverage
Green = Hit in *H. sapiens*
Red = Hit in *H. sapiens* and *T. californica*
White = Not in *H. sapiens*

145 Cocaine addiction

145.1 Human Pathway: HSA05030

145.2 Number of Hits: 6

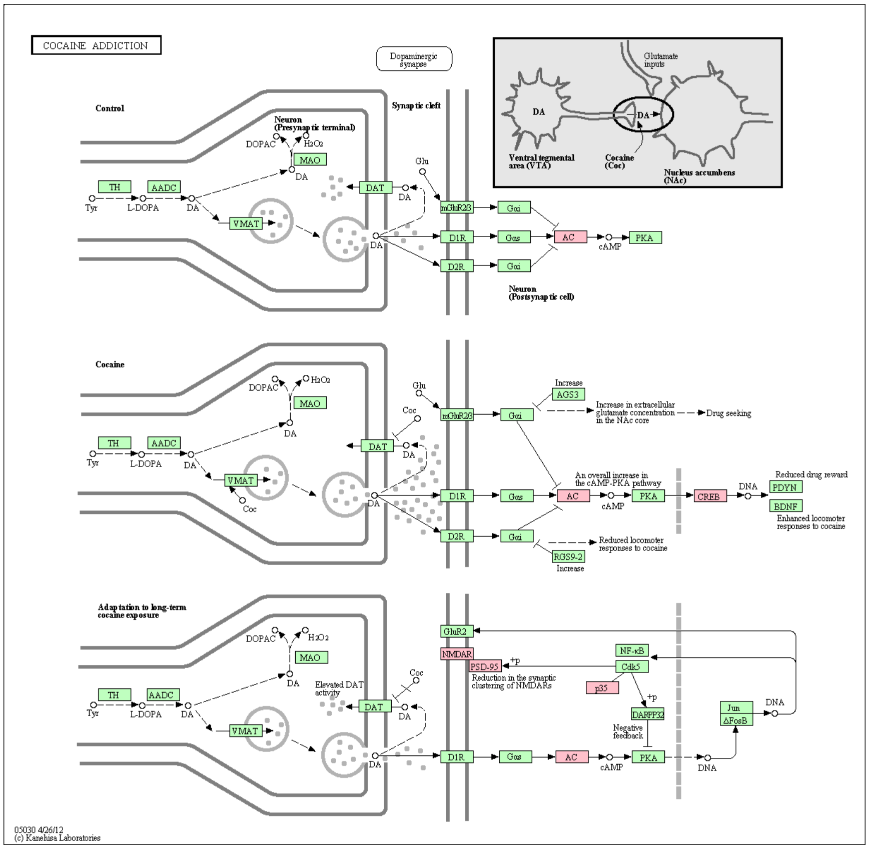

145.3 Legend:

RBH-Blast at 60% Identity + 50% Coverage
Green = Hit in *H. sapiens*
Red = Hit in *H. sapiens* and *T. californica*
White = Not in *H. sapiens*

146 Adherens junction

146.1 Human Pathway: HSA04520

146.2 Number of Hits: 6

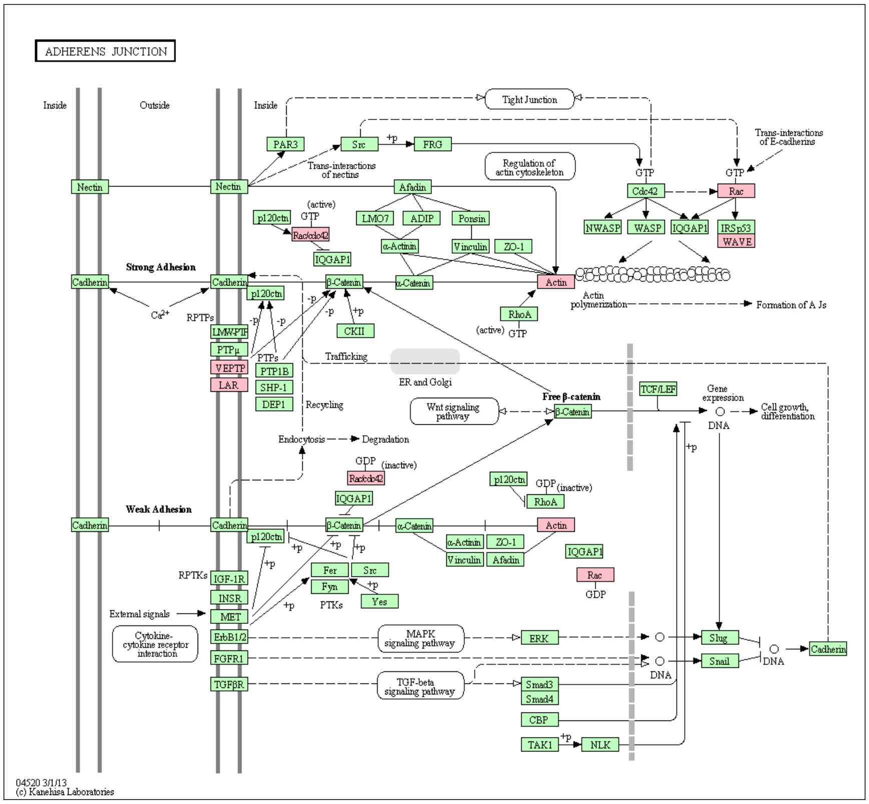

146.3 Legend:

RBH-Blast at 60% Identity + 50% Coverage
Green = Hit in *H. sapiens*
Red = Hit in *H. sapiens* and *T. californica*
White = Not in *H. sapiens*

147 Glioma

147.1 Human Pathway: HSA05214

147.2 Number of Hits: 6

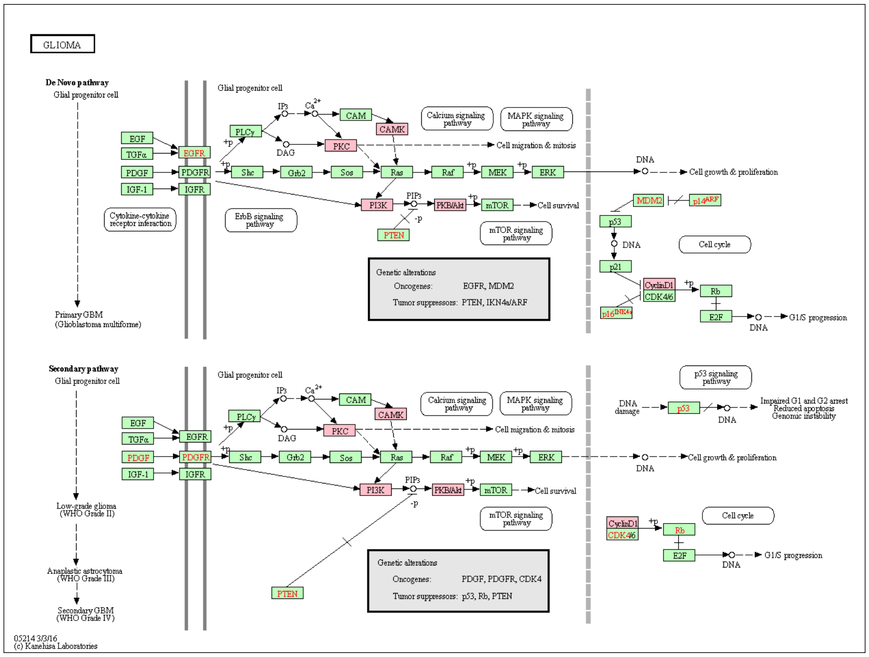

147.3 Legend:

RBH-Blast at 60% Identity + 50% Coverage
Green = Hit in *H. sapiens*
Red = Hit in *H. sapiens* and *T. californica*
White = Not in *H. sapiens*

148 SNARE interactions in vesicular transport

148.1 Human Pathway: HSA04130

148.2 Number of Hits: 6

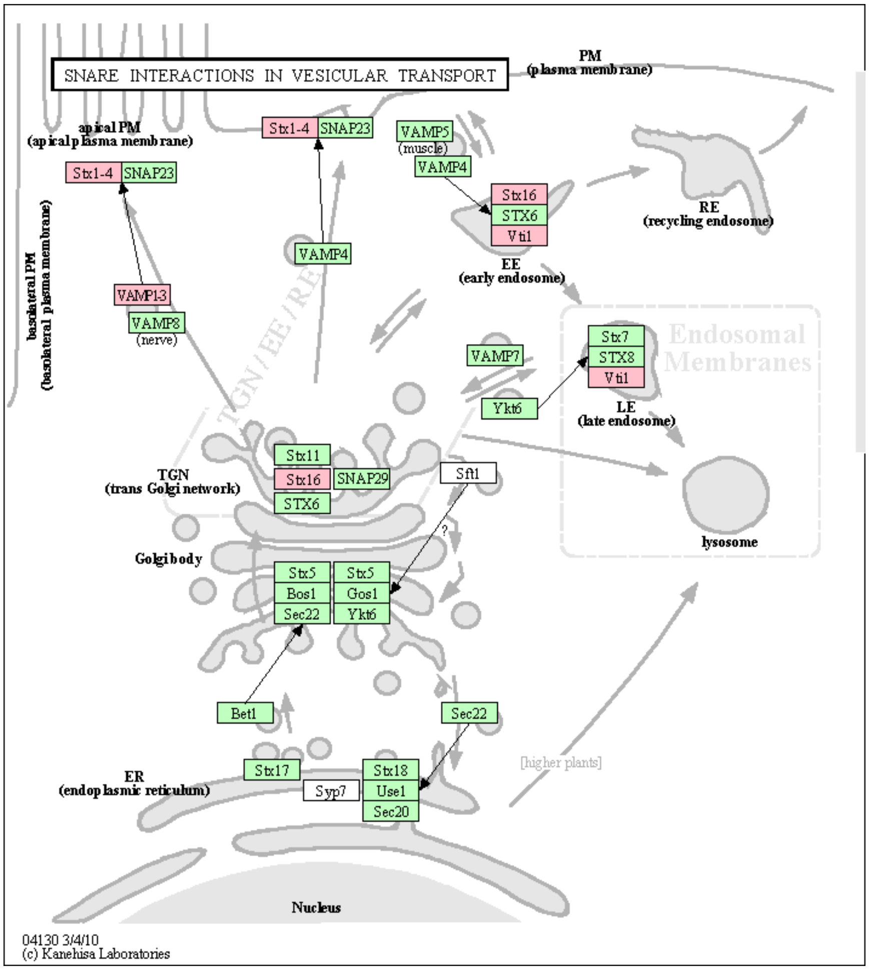

148.3 Legend:

RBH-Blast at 60% Identity + 50% Coverage
Green = Hit in *H. sapiens*
Red = Hit in *H. sapiens* and *T. californica*
White = Not in *H. sapiens*

149 Terpenoid backbone biosynthesis

149.1 Human Pathway: HSA00900

149.2 Number of Hits: 6

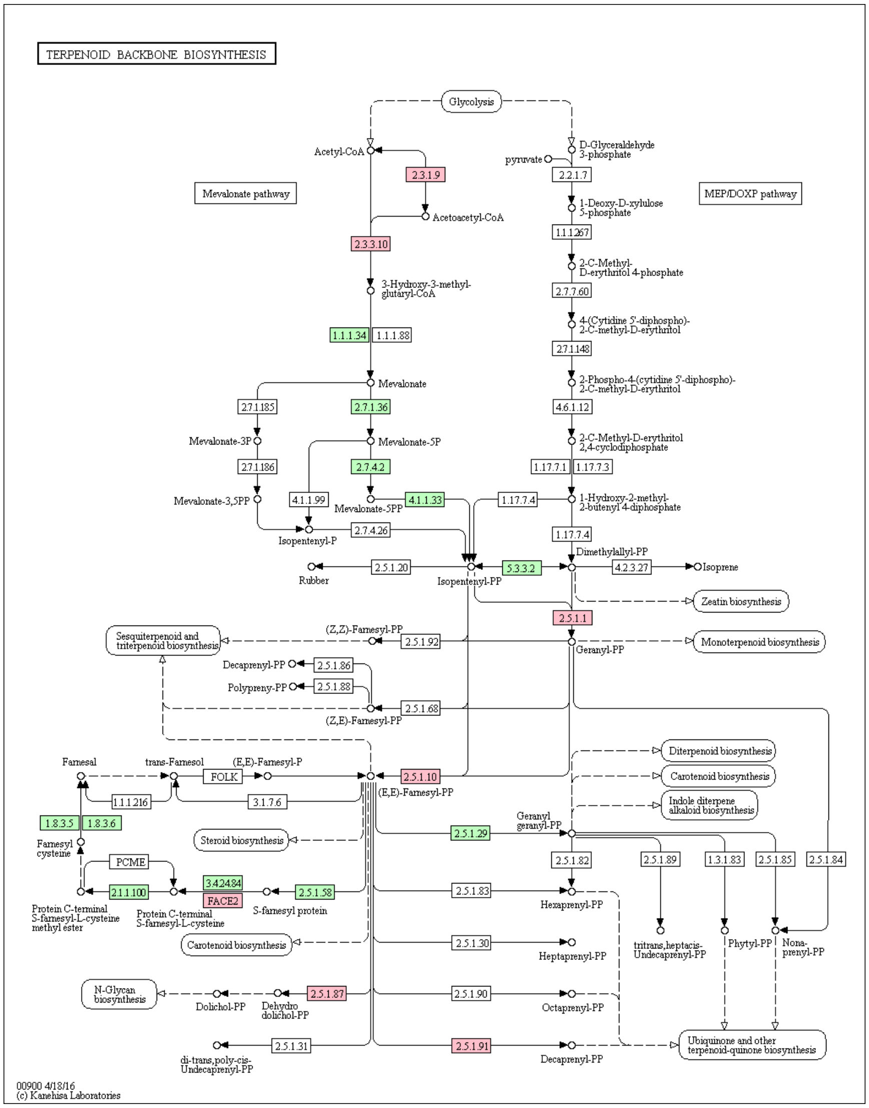

149.3 Legend:

RBH-Blast at 60% Identity + 50% Coverage
Green = Hit in *H. sapiens*
Red = Hit in *H. sapiens* and *T. californica*
White = Not in *H. sapiens*

150 EGFR tyrosine kinase inhibitor resistance

150.1 Human Pathway: HSA01521

150.2 Number of Hits: 6

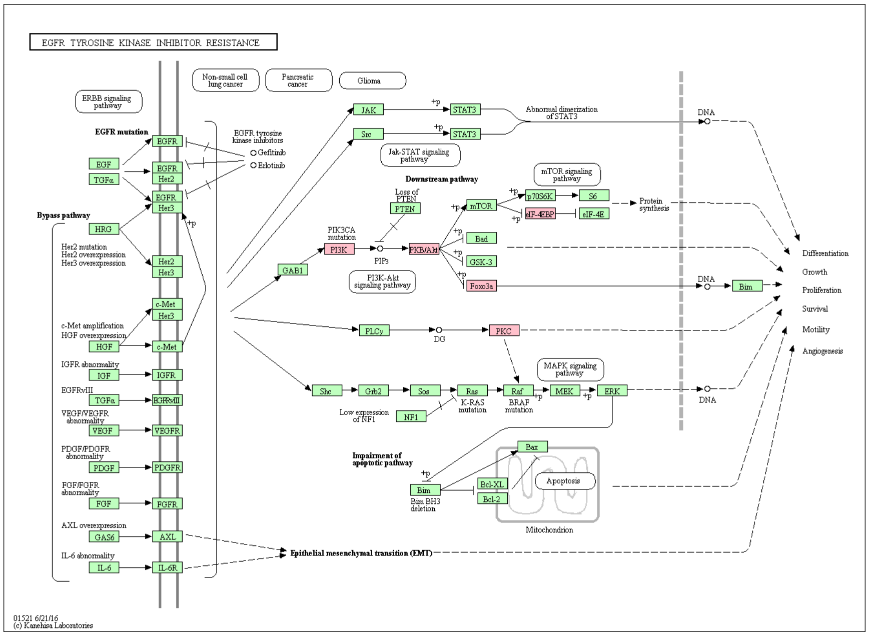

150.3 Legend:

RBH-Blast at 60% Identity + 50% Coverage
Green = Hit in *H. sapiens*
Red = Hit in *H. sapiens* and *T. californica*
White = Not in *H. sapiens*

151 Drug metabolism - other enzymes

151.1 Human Pathway: HSA00983

151.2 Number of Hits: 6

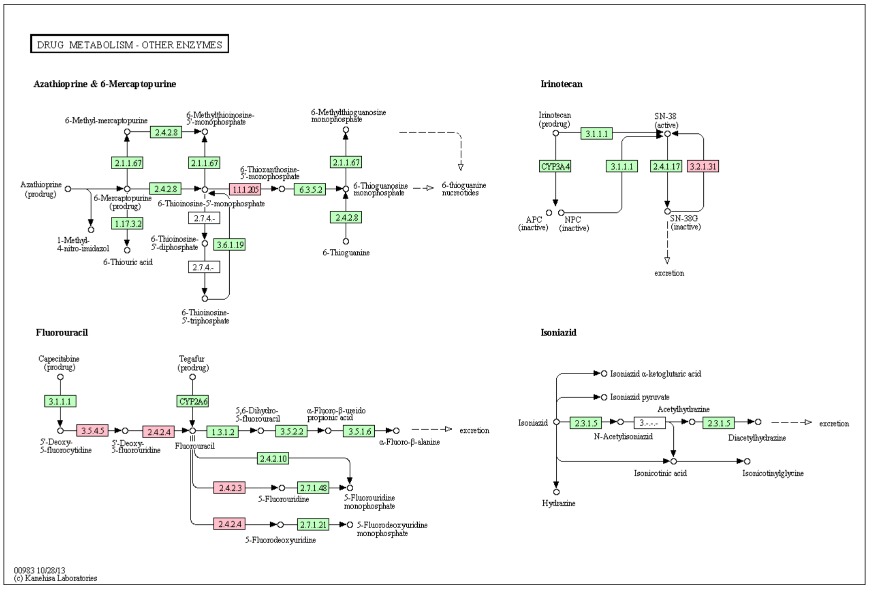

151.3 Legend:

RBH-Blast at 60% Identity + 50% Coverage
Green = Hit in *H. sapiens*
Red = Hit in *H. sapiens* and *T. californica*
White = Not in *H. sapiens*

152 Acute myeloid leukemia

152.1 Human Pathway: HSA05221

152.2 Number of Hits: 6

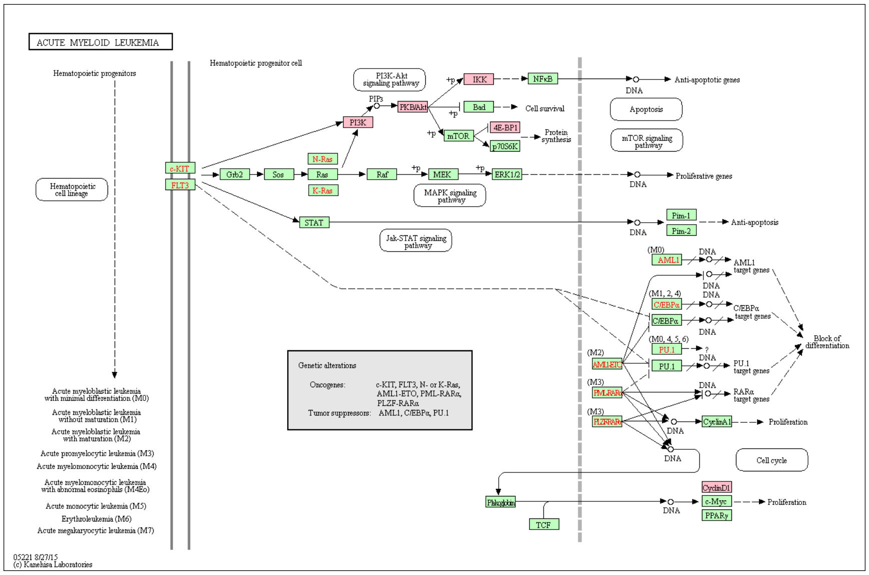

152.3 Legend:

RBH-Blast at 60% Identity + 50% Coverage
Green = Hit in *H. sapiens*
Red = Hit in *H. sapiens* and *T. californica*
White = Not in *H. sapiens*

153 Hedgehog signaling pathway

153.1 Human Pathway: HSA04340

153.2 Number of Hits: 6

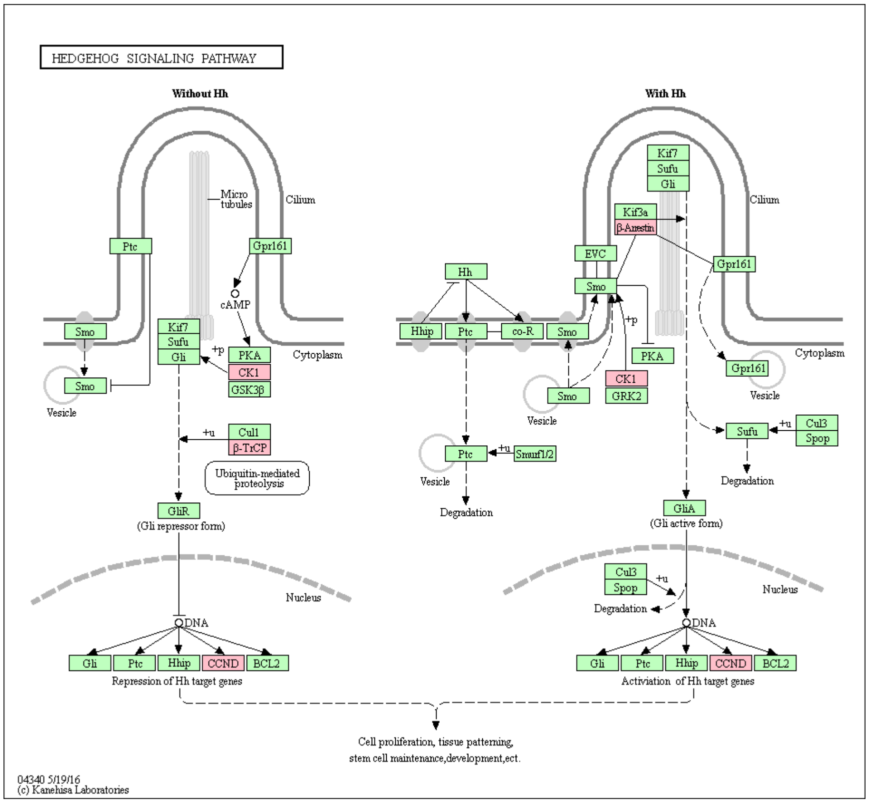

153.3 Legend:

RBH-Blast at 60% Identity + 50% Coverage
Green = Hit in *H. sapiens*
Red = Hit in *H. sapiens* and *T. californica*
White = Not in *H. sapiens*

154 Chagas disease (American trypanosomiasis)

154.1 Human Pathway: HSA05142

154.2 Number of Hits: 6

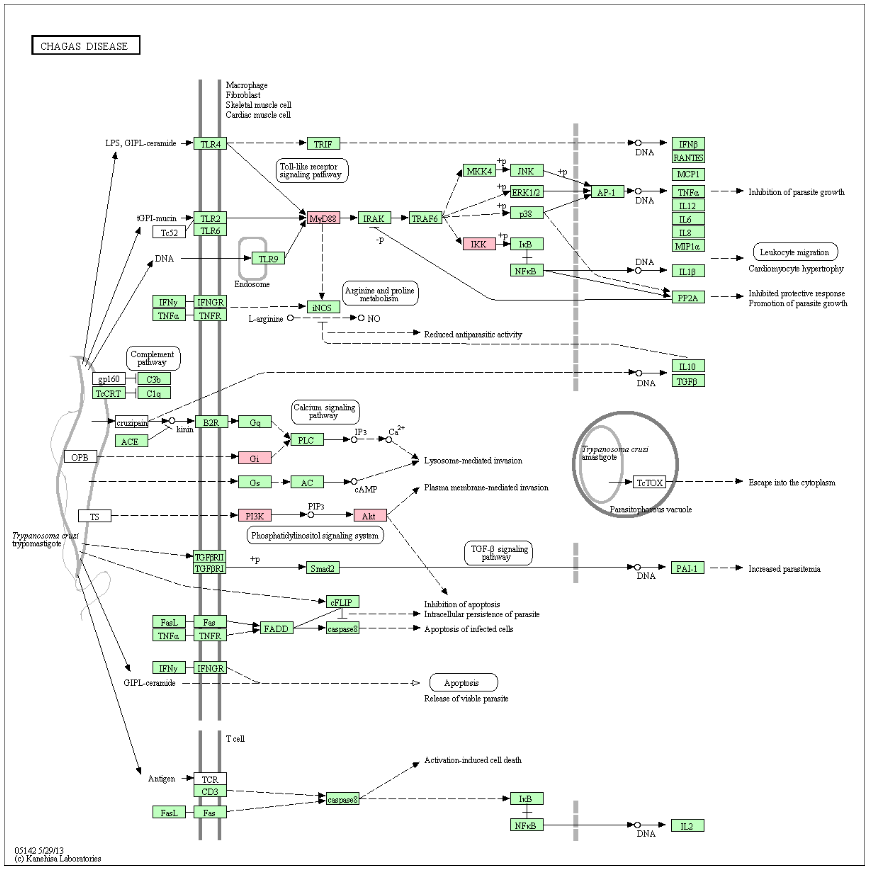

154.3 Legend:

RBH-Blast at 60% Identity + 50% Coverage
Green = Hit in *H. sapiens*
Red = Hit in *H. sapiens* and *T. californica*
White = Not in *H. sapiens*

155 Amyotrophic lateral sclerosis (ALS)

155.1 Human Pathway: HSA05014

155.2 Number of Hits: 6

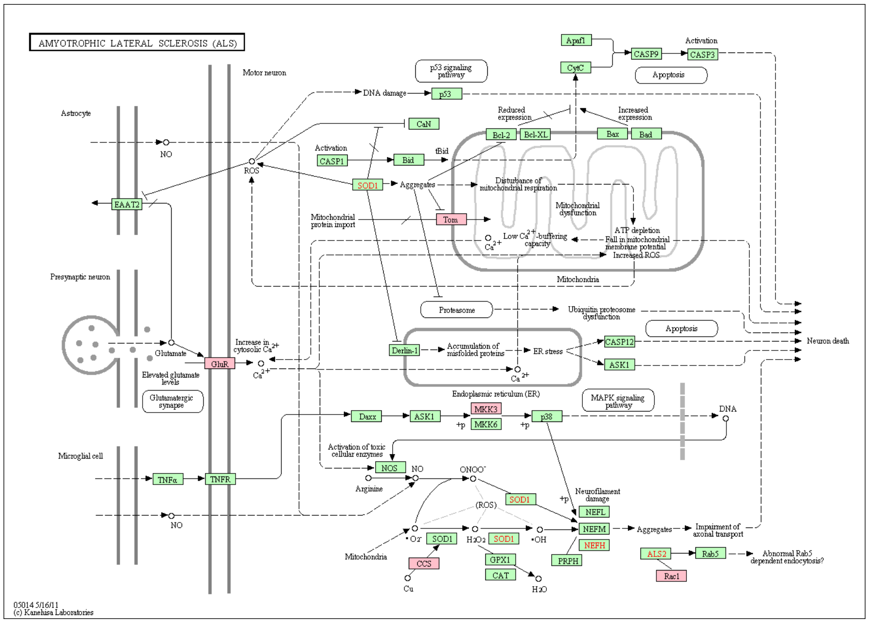

155.3 Legend:

RBH-Blast at 60% Identity + 50% Coverage
Green = Hit in *H. sapiens*
Red = Hit in *H. sapiens* and *T. californica*
White = Not in *H. sapiens*

156 Prostate cancer

156.1 Human Pathway: HSA05215

156.2 Number of Hits: 6

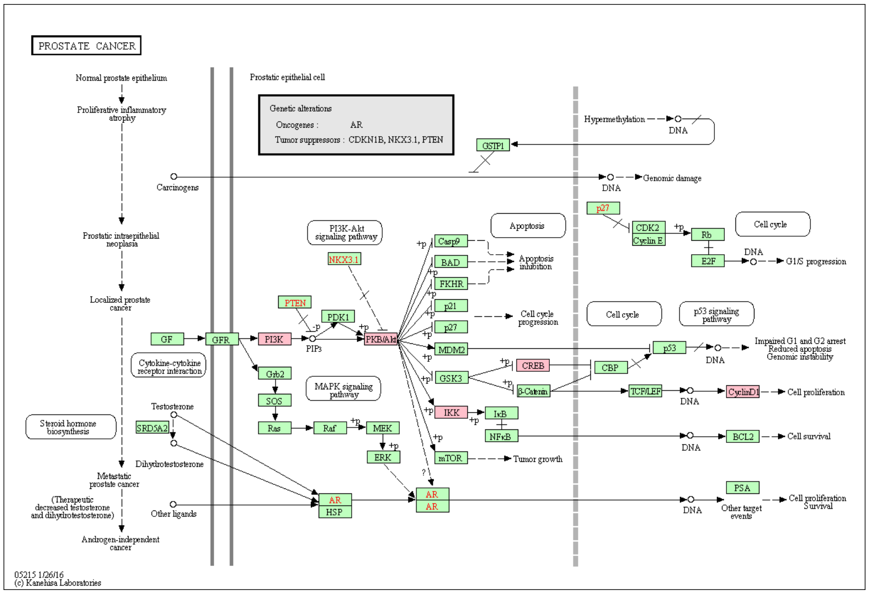

156.3 Legend:

RBH-Blast at 60% Identity + 50% Coverage
Green = Hit in *H. sapiens*
Red = Hit in *H. sapiens* and *T. californica*
White = Not in *H. sapiens*

157 Hepatitis C

157.1 Human Pathway: HSA05160

157.2 Number of Hits: 6

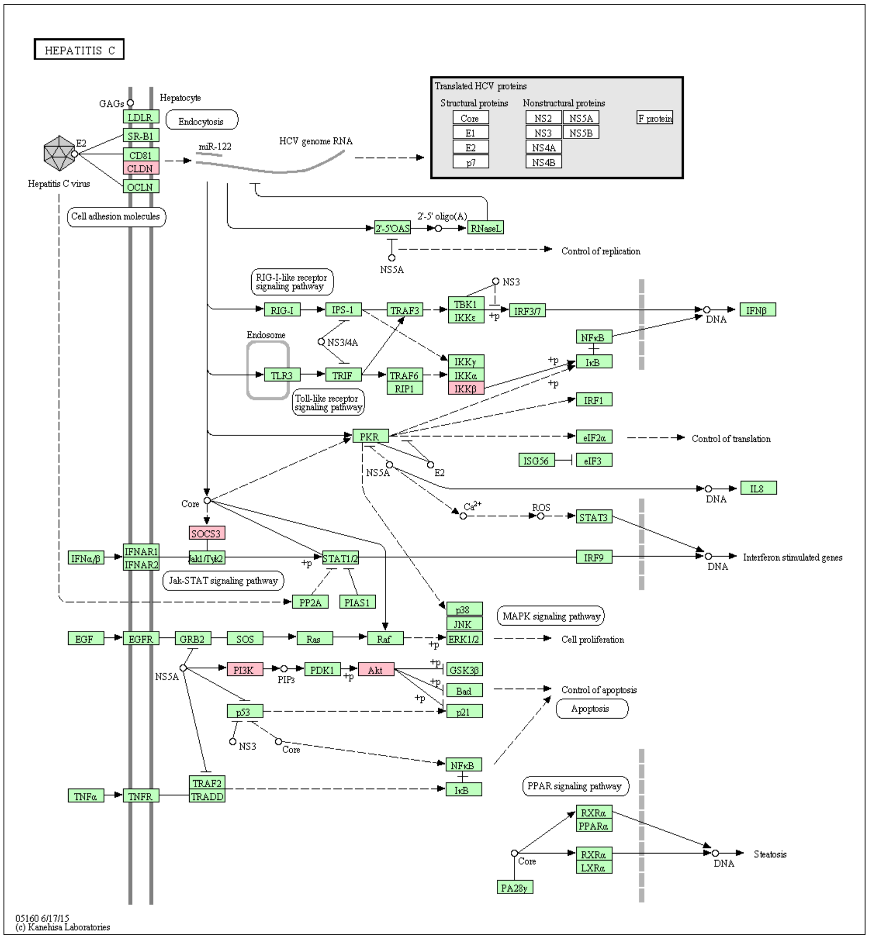

157.3 Legend:

RBH-Blast at 60% Identity + 50% Coverage
Green = Hit in *H. sapiens*
Red = Hit in *H. sapiens* and *T. californica*
White = Not in *H. sapiens*

158 Nicotine addiction

158.1 Human Pathway: HSA05033

158.2 Number of Hits: 6

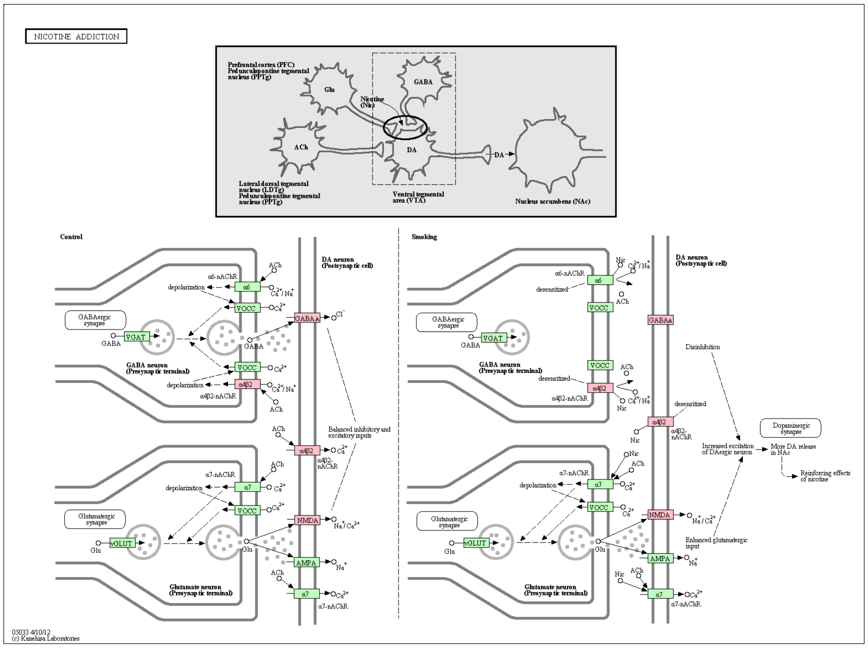

158.3 Legend:

RBH-Blast at 60% Identity + 50% Coverage
Green = Hit in *H. sapiens*
Red = Hit in *H. sapiens* and *T. californica*
White = Not in *H. sapiens*

159 Vasopressin-regulated water reabsorption

159.1 Human Pathway: HSA04962

159.2 Number of Hits: 6

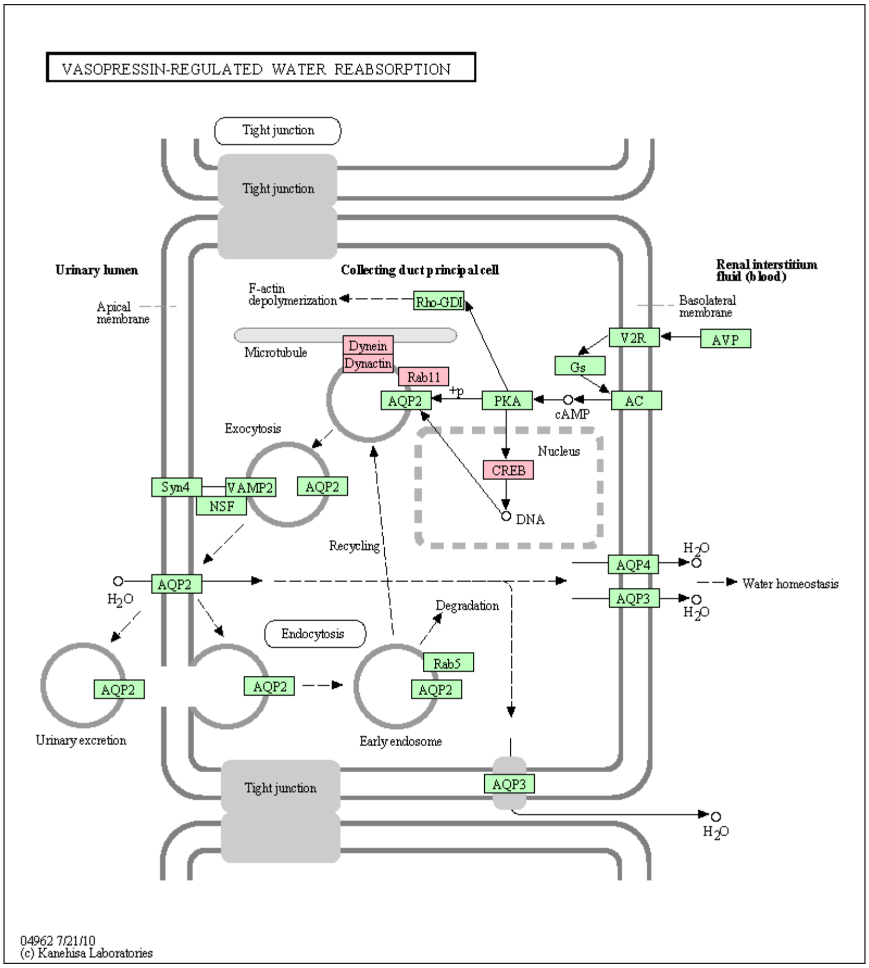

159.3 Legend:

RBH-Blast at 60% Identity + 50% Coverage
Green = Hit in *H. sapiens*
Red = Hit in *H. sapiens* and *T. californica*
White = Not in *H. sapiens*

160 Regulation of lipolysis in adipocytes

160.1 Human Pathway: HSA04923

160.2 Number of Hits: 6

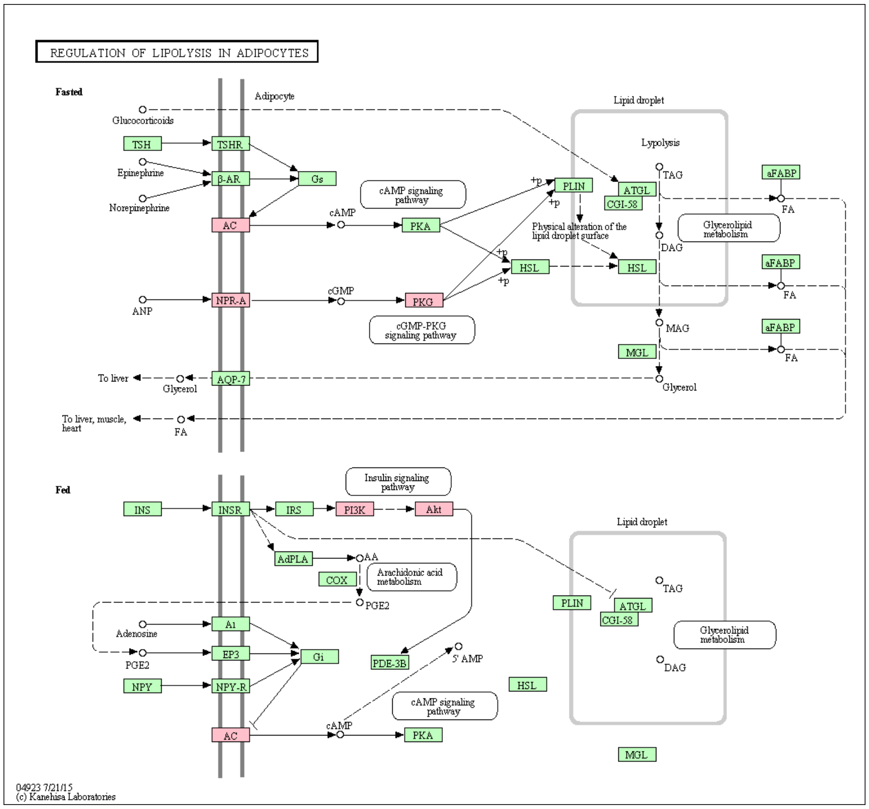

160.3 Legend:

RBH-Blast at 60% Identity + 50% Coverage
Green = Hit in *H. sapiens*
Red = Hit in *H. sapiens* and *T. californica*
White = Not in *H. sapiens*

161 PPAR signaling pathway

161.1 Human Pathway: HSA03320

161.2 Number of Hits: 6

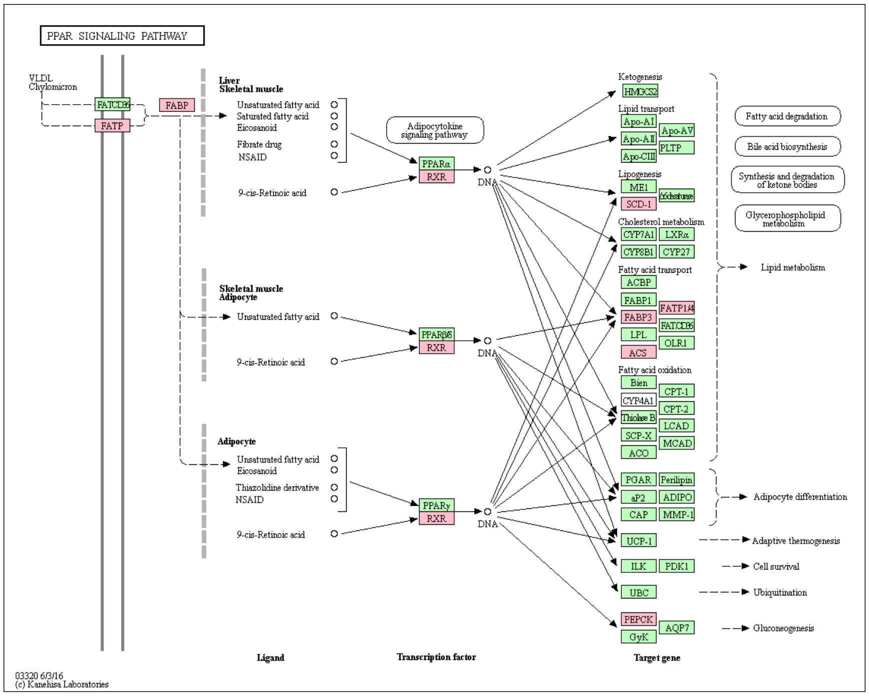

161.3 Legend:

RBH-Blast at 60% Identity + 50% Coverage
Green = Hit in *H. sapiens*
Red = Hit in *H. sapiens* and *T. californica*
White = Not in *H. sapiens*

162 Galactose metabolism

162.1 Human Pathway: HSA00052

162.2 Number of Hits: 5

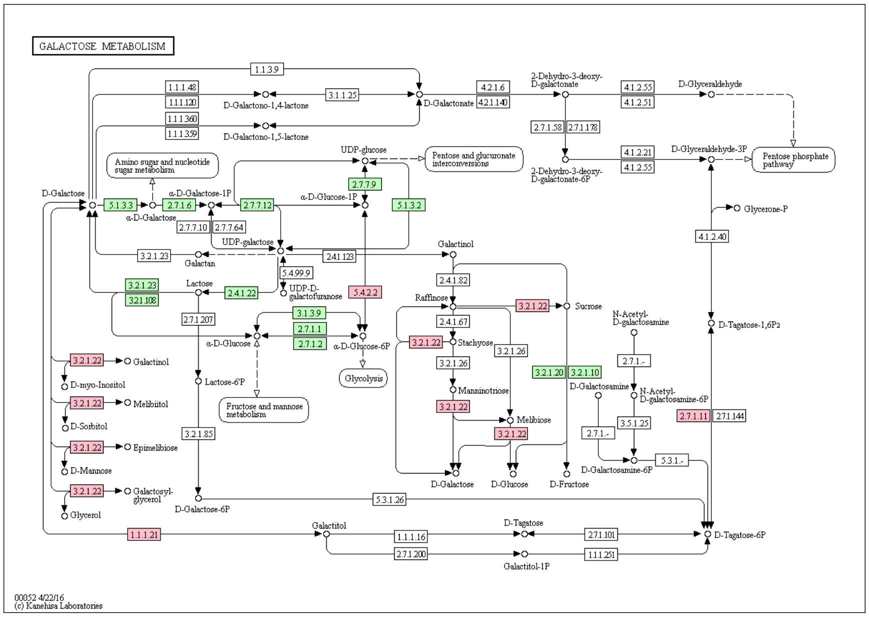

162.3 Legend:

RBH-Blast at 60% Identity + 50% Coverage
Green = Hit in *H. sapiens*
Red = Hit in *H. sapiens* and *T. californica*
White = Not in *H. sapiens*

163 Salivary secretion

163.1 Human Pathway: HSA04970

163.2 Number of Hits: 5

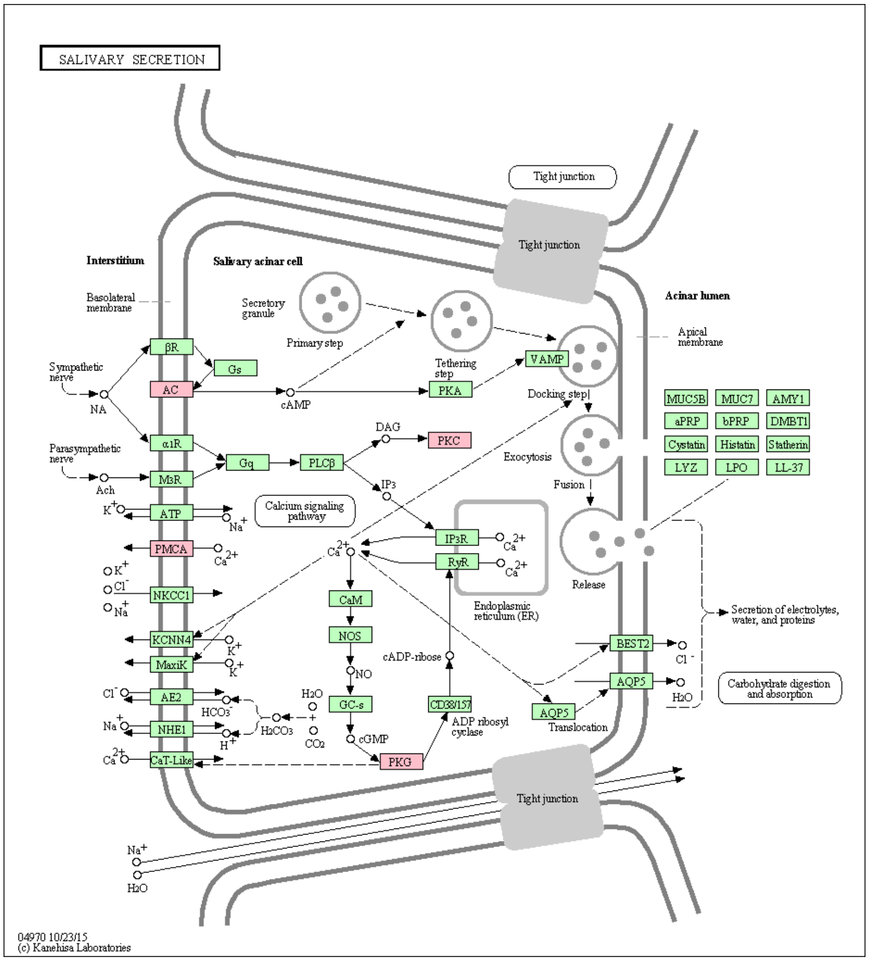

163.3 Legend:

RBH-Blast at 60% Identity + 50% Coverage
Green = Hit in *H. sapiens*
Red = Hit in *H. sapiens* and *T. californica*
White = Not in *H. sapiens*

164 GnRH signaling pathway

164.1 Human Pathway: HSA04912

164.2 Number of Hits: 5

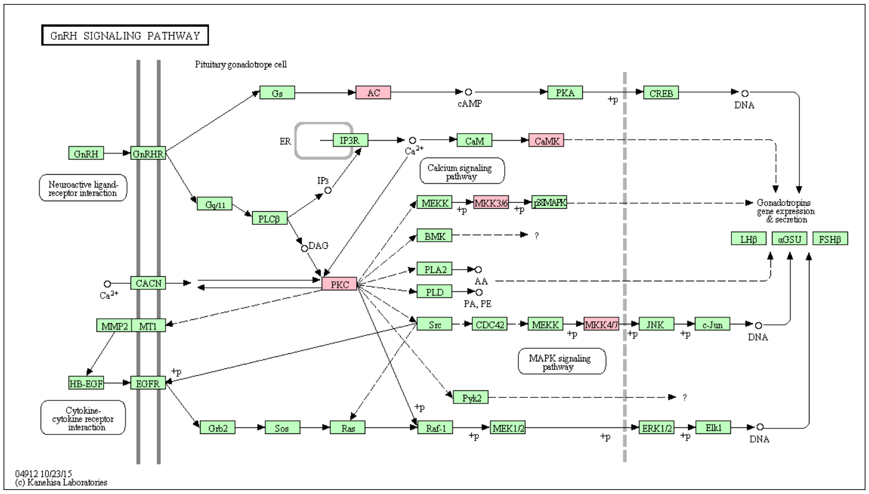

164.3 Legend:

RBH-Blast at 60% Identity + 50% Coverage
Green = Hit in *H. sapiens*
Red = Hit in *H. sapiens* and *T. californica*
White = Not in *H. sapiens*

165 Endometrial cancer

165.1 Human Pathway: HSA05213

165.2 Number of Hits: 5

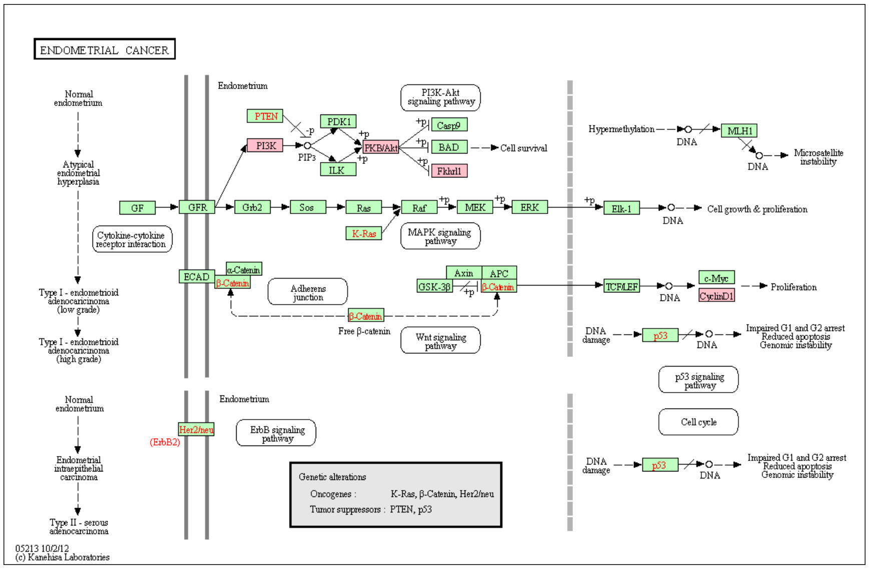

165.3 Legend:

RBH-Blast at 60% Identity + 50% Coverage
Green = Hit in *H. sapiens*
Red = Hit in *H. sapiens* and *T. californica*
White = Not in *H. sapiens*

166 Base excision repair

166.1 Human Pathway: HSA03410

166.2 Number of Hits: 5

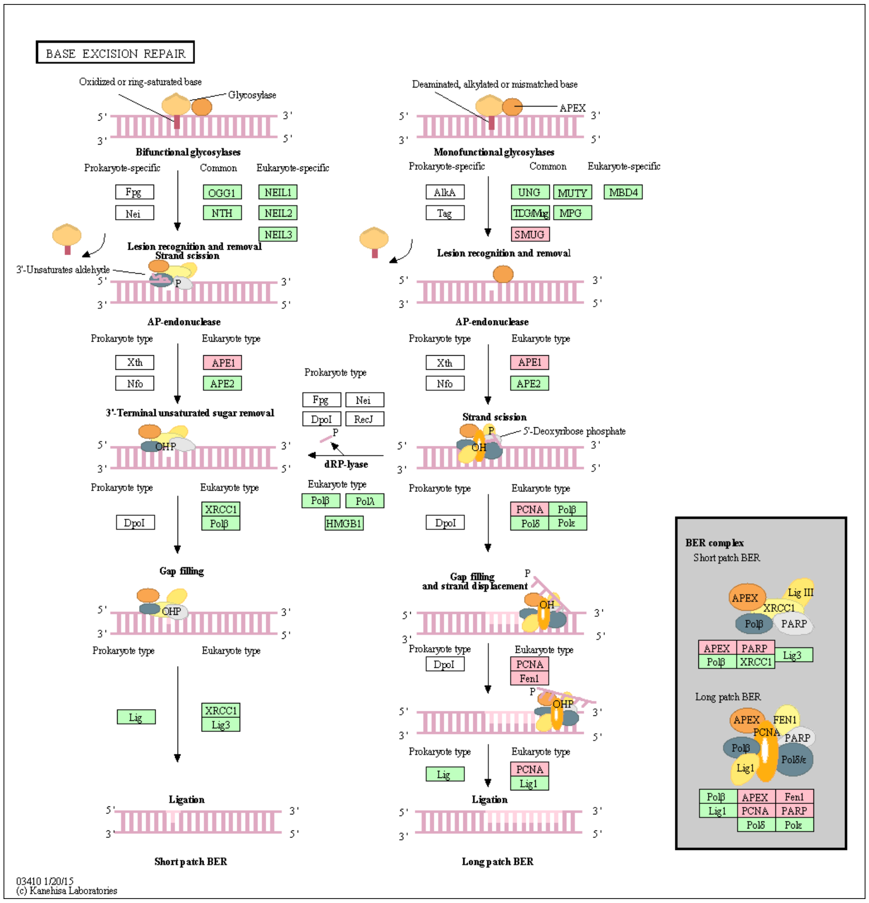

166.3 Legend:

RBH-Blast at 60% Identity + 50% Coverage
Green = Hit in *H. sapiens*
Red = Hit in *H. sapiens* and *T. californica*
White = Not in *H. sapiens*

167 NF-kappa B signaling pathway

167.1 Human Pathway: HSA04064

167.2 Number of Hits: 5

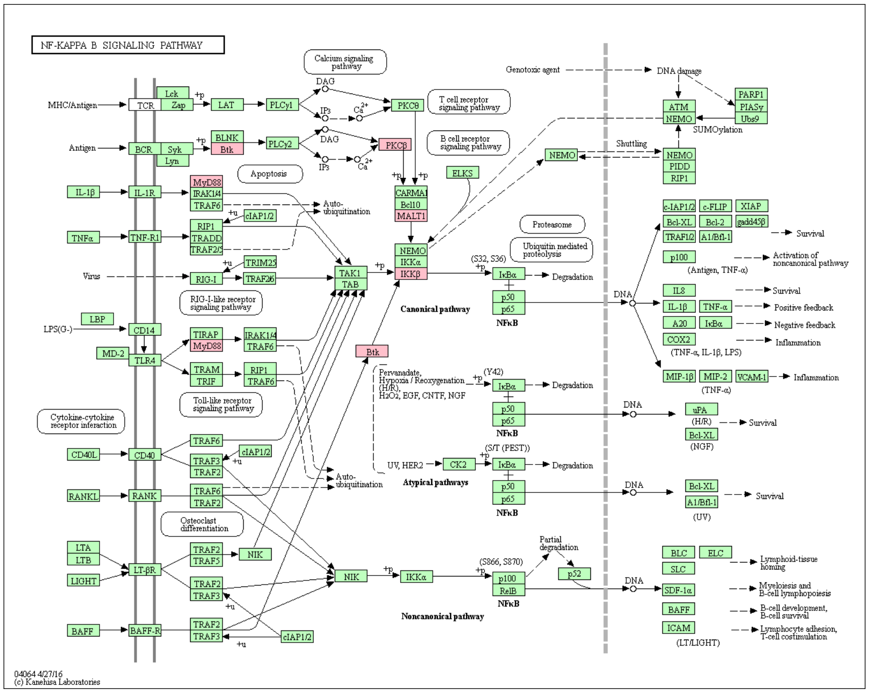

167.3 Legend:

RBH-Blast at 60% Identity + 50% Coverage
Green = Hit in *H. sapiens*
Red = Hit in *H. sapiens* and *T. californica*
White = Not in *H. sapiens*

168 Inositol phosphate metabolism

168.1 Human Pathway: HSA00562

168.2 Number of Hits: 5

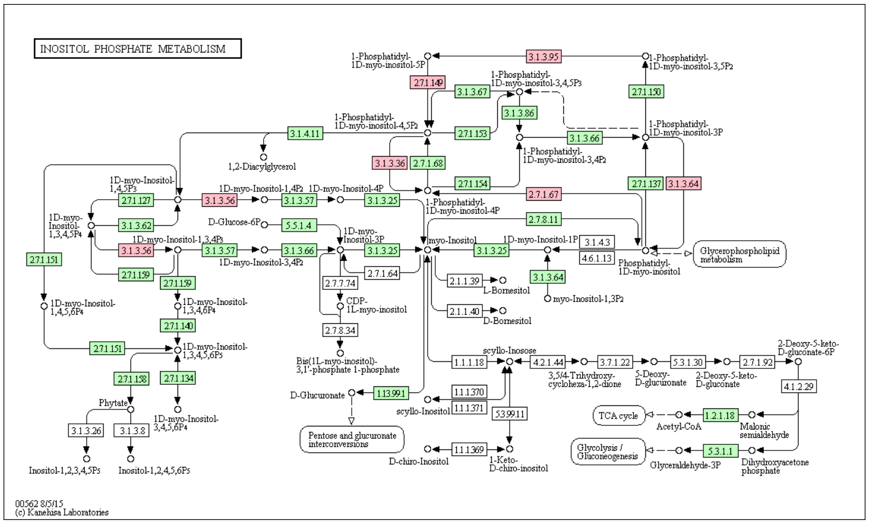

168.3 Legend:

RBH-Blast at 60% Identity + 50% Coverage
Green = Hit in *H. sapiens*
Red = Hit in *H. sapiens* and *T. californica*
White = Not in *H. sapiens*

169 Carbohydrate digestion and absorption

169.1 Human Pathway: HSA04973

169.2 Number of Hits: 5

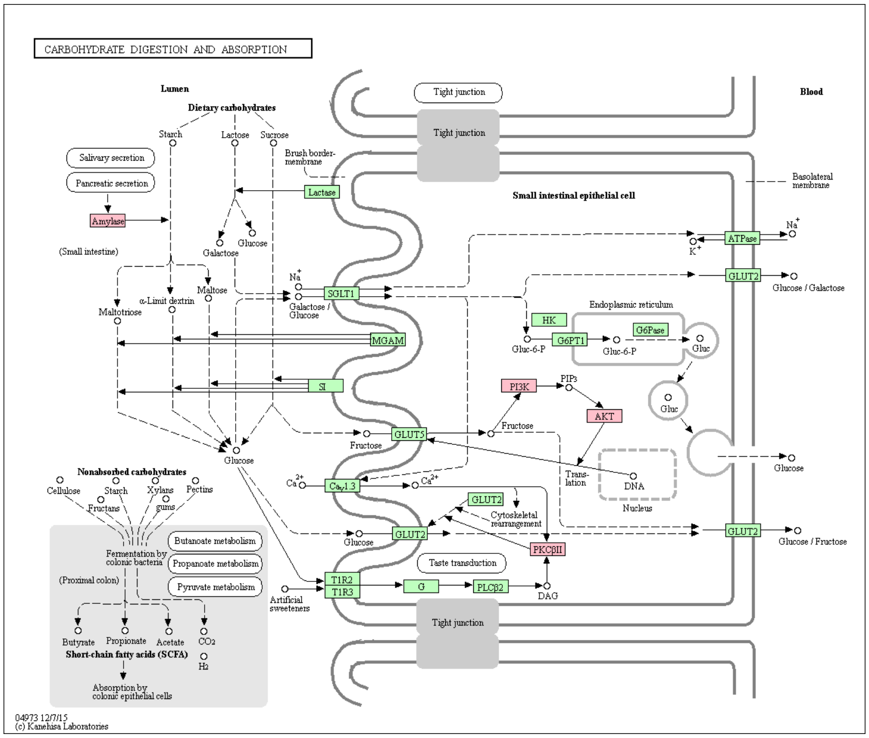

169.3 Legend:

RBH-Blast at 60% Identity + 50% Coverage
Green = Hit in *H. sapiens*
Red = Hit in *H. sapiens* and *T. californica*
White = Not in *H. sapiens*

170 Aminoacyl-tRNA biosynthesis

170.1 Human Pathway: HSA00970

170.2 Number of Hits: 5

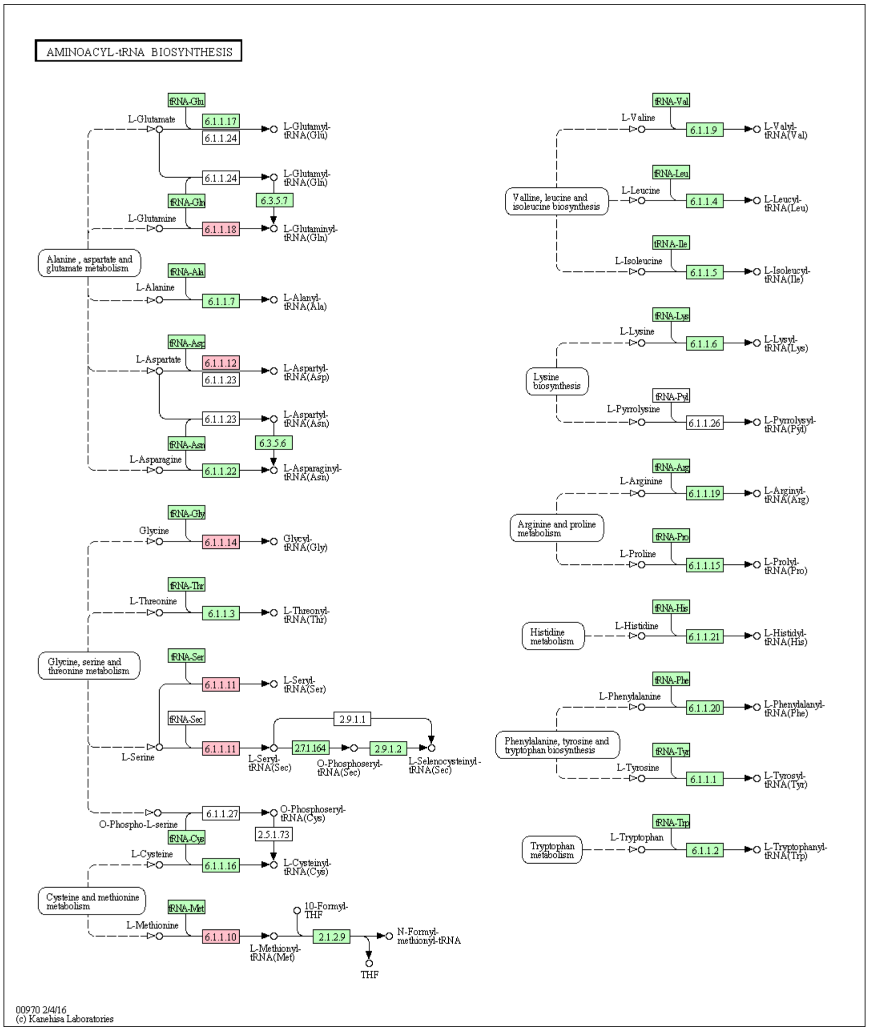

170.3 Legend:

RBH-Blast at 60% Identity + 50% Coverage
Green = Hit in *H. sapiens*
Red = Hit in *H. sapiens* and *T. californica*
White = Not in *H. sapiens*

171 Proteasome

171.1 Human Pathway: HSA03050

171.2 Number of Hits: 5

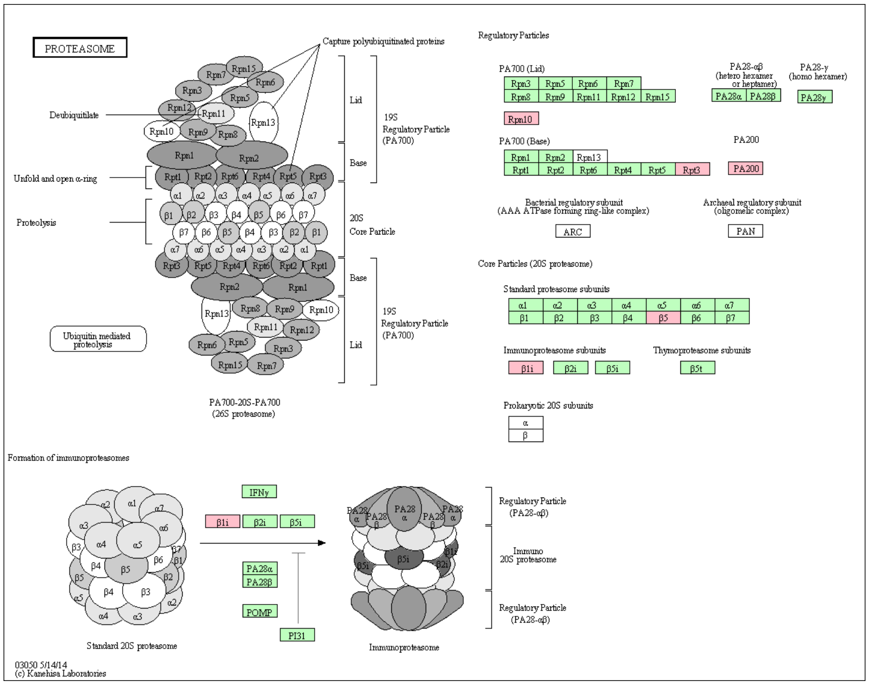

171.3 Legend:

RBH-Blast at 60% Identity + 50% Coverage
Green = Hit in *H. sapiens*
Red = Hit in *H. sapiens* and *T. californica*
White = Not in *H. sapiens*

172 Glyoxylate and dicarboxylate metabolism

172.1 Human Pathway: HSA00630

172.2 Number of Hits: 5

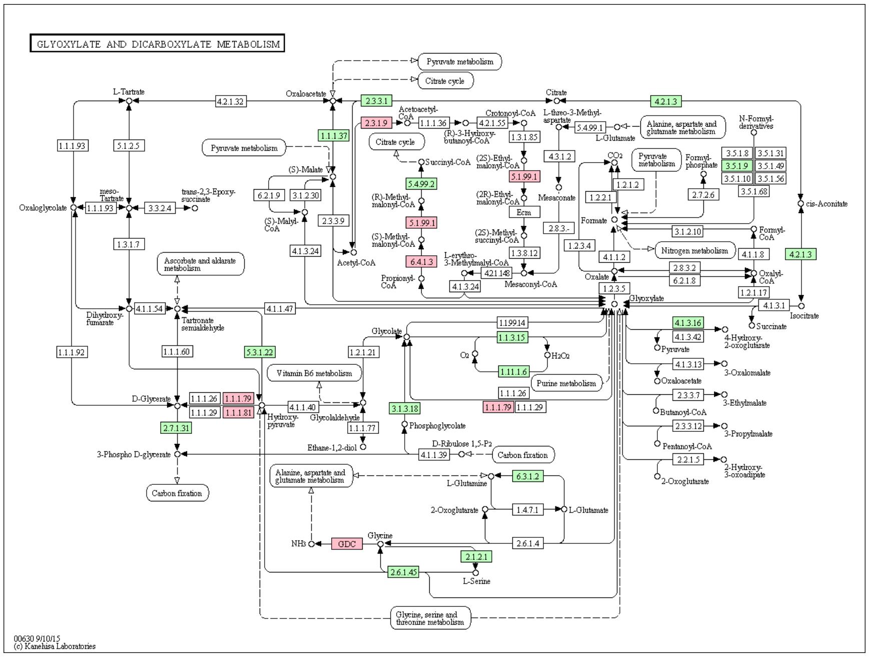

172.3 Legend:

RBH-Blast at 60% Identity + 50% Coverage
Green = Hit in *H. sapiens*
Red = Hit in *H. sapiens* and *T. californica*
White = Not in *H. sapiens*

173 Olfactory transduction

173.1 Human Pathway: HSA04740

173.2 Number of Hits: 5

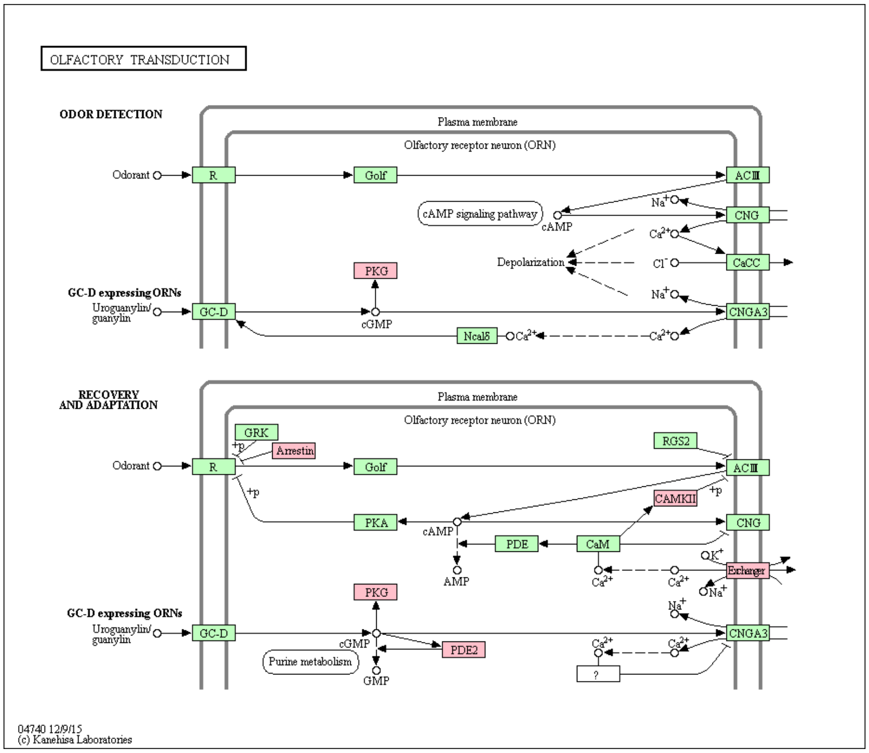

173.3 Legend:

RBH-Blast at 60% Identity + 50% Coverage
Green = Hit in *H. sapiens*
Red = Hit in *H. sapiens* and *T. californica*
White = Not in *H. sapiens*

174 Pyruvate metabolism

174.1 Human Pathway: HSA00620

174.2 Number of Hits: 5

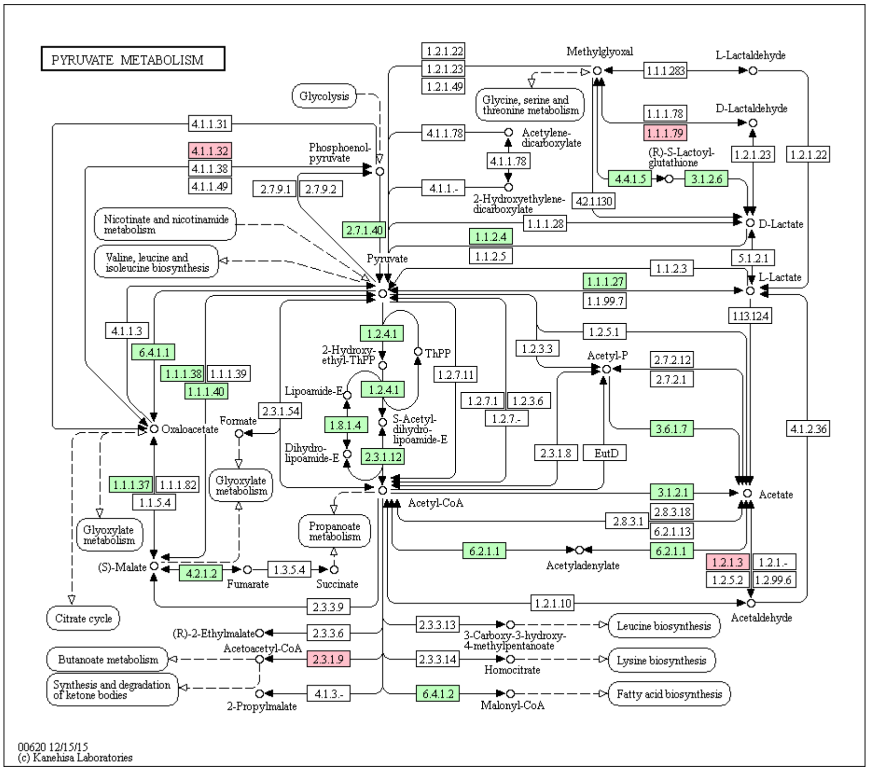

174.3 Legend:

RBH-Blast at 60% Identity + 50% Coverage
Green = Hit in *H. sapiens*
Red = Hit in *H. sapiens* and *T. californica*
White = Not in *H. sapiens*

175 Type II diabetes mellitus

175.1 Human Pathway: HSA04930

175.2 Number of Hits: 4

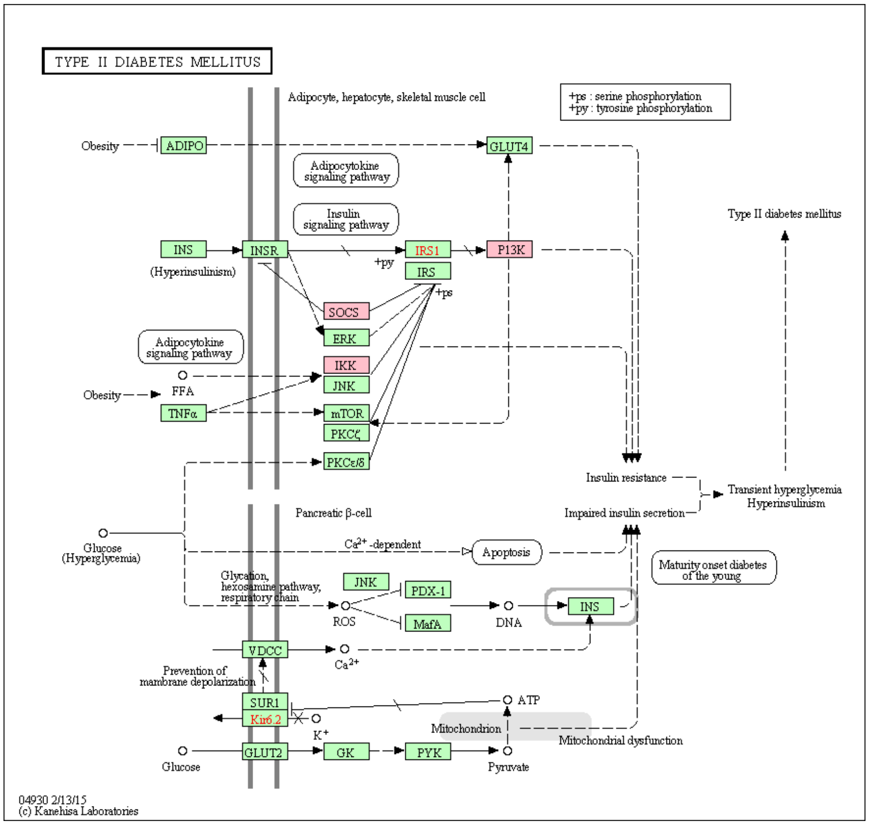

175.3 Legend:

RBH-Blast at 60% Identity + 50% Coverage
Green = Hit in *H. sapiens*
Red = Hit in *H. sapiens* and *T. californica*
White = Not in *H. sapiens*

176 Synthesis and degradation of ketone bodies

176.1 Human Pathway: HSA00072

176.2 Number of Hits: 4

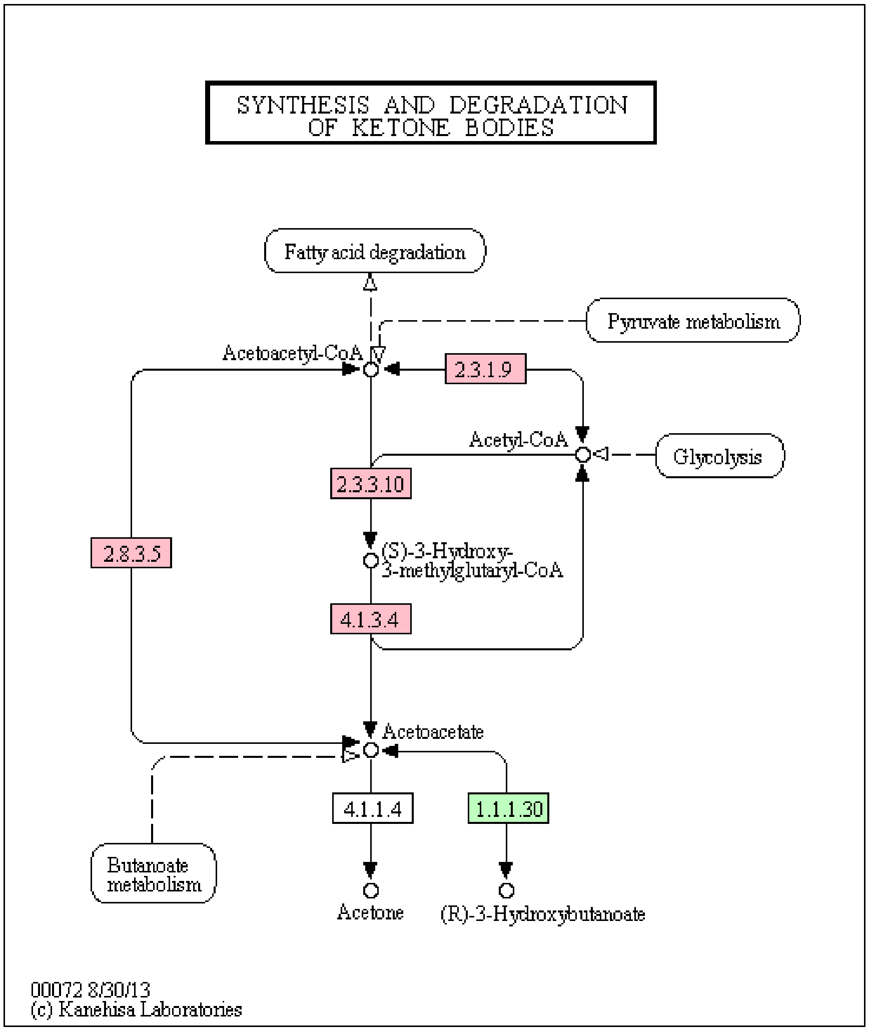

176.3 Legend:

RBH-Blast at 60% Identity + 50% Coverage
Green = Hit in *H. sapiens*
Red = Hit in *H. sapiens* and *T. californica*
White = Not in *H. sapiens*

177 Alanine, aspartate and glutamate metabolism

177.1 Human Pathway: HSA00250

177.2 Number of Hits: 4

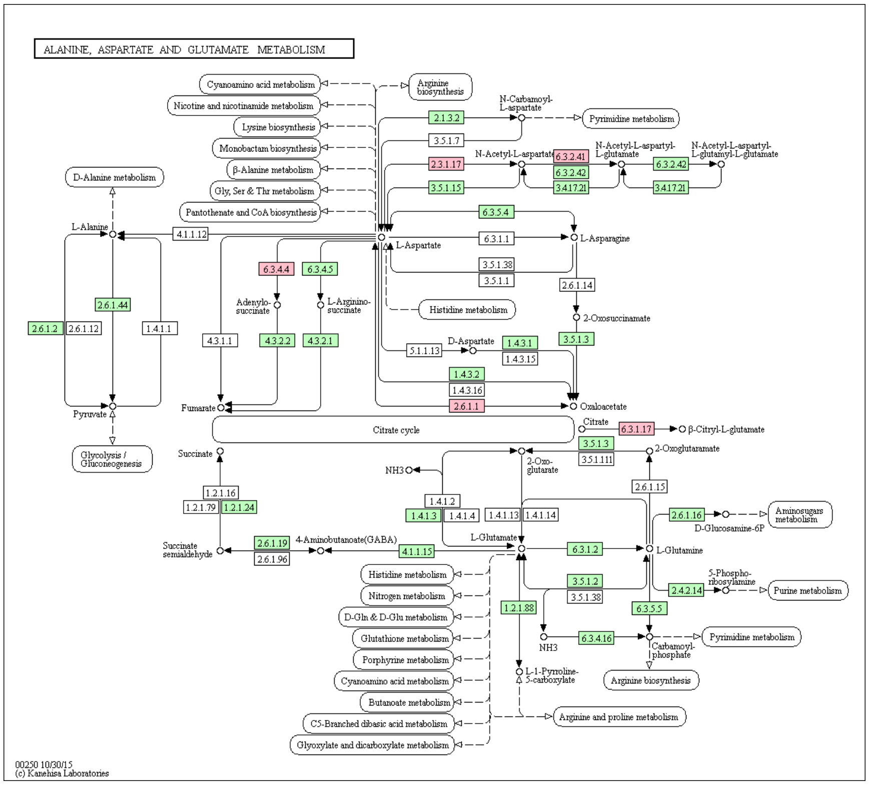

177.3 Legend:

RBH-Blast at 60% Identity + 50% Coverage
Green = Hit in *H. sapiens*
Red = Hit in *H. sapiens* and *T. californica*
White = Not in *H. sapiens*

178 Notch signaling pathway

178.1 Human Pathway: HSA04330

178.2 Number of Hits: 4

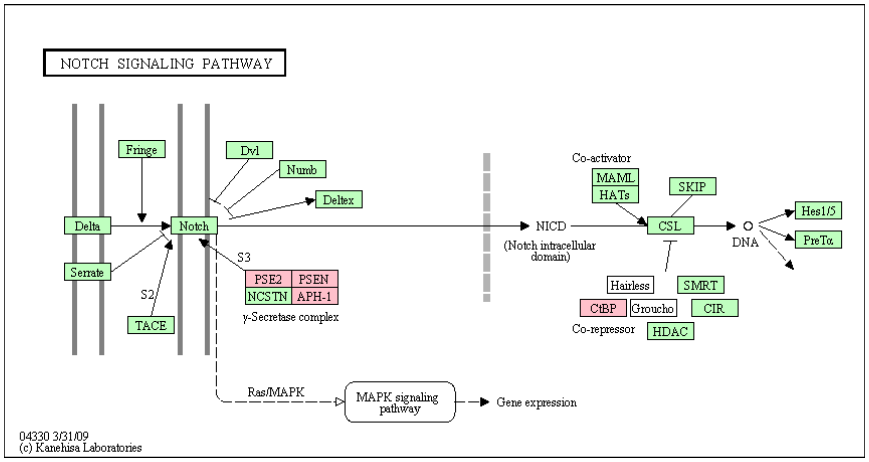

178.3 Legend:

RBH-Blast at 60% Identity + 50% Coverage
Green = Hit in *H. sapiens*
Red = Hit in *H. sapiens* and *T. californica*
White = Not in *H. sapiens*

179 Melanoma

179.1 Human Pathway: HSA05218

179.2 Number of Hits: 4

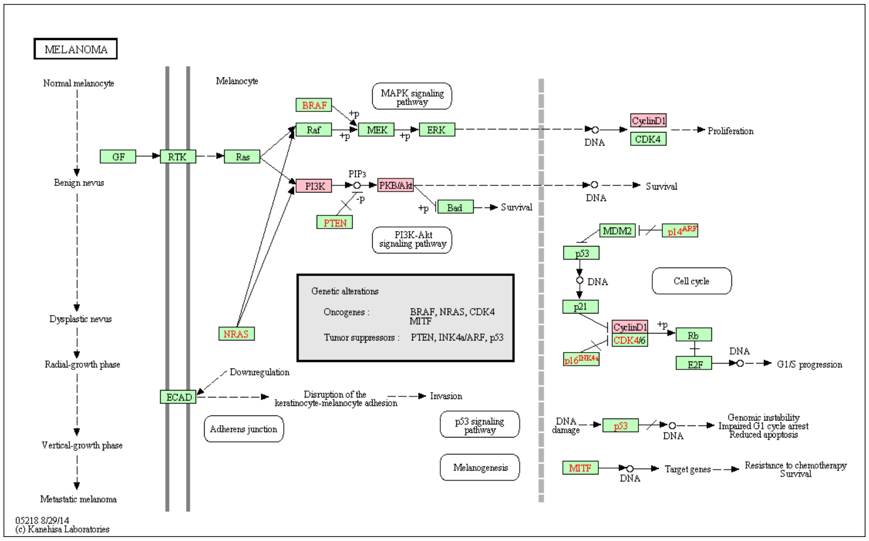

179.3 Legend:

RBH-Blast at 60% Identity + 50% Coverage
Green = Hit in *H. sapiens*
Red = Hit in *H. sapiens* and *T. californica*
White = Not in *H. sapiens*

180 Ribosome biogenesis in eukaryotes

180.1 Human Pathway: HSA03008

180.2 Number of Hits: 4

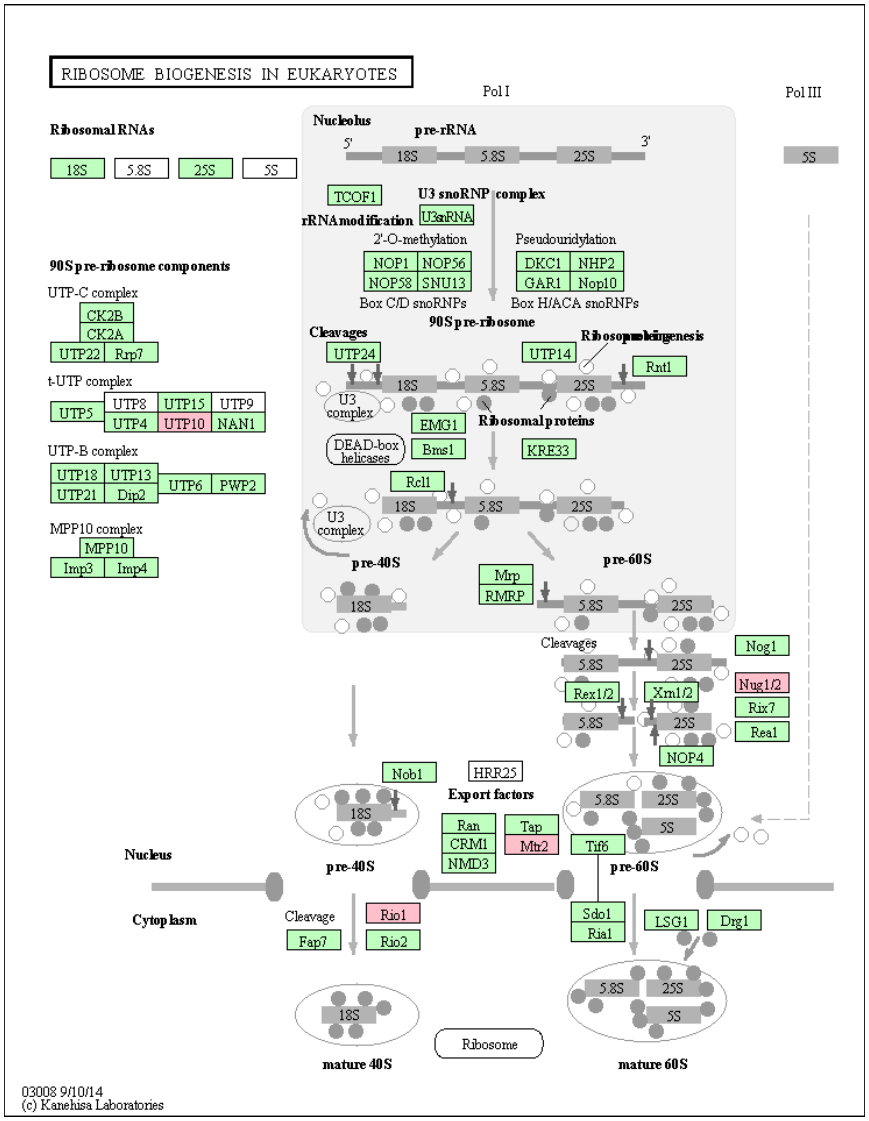

180.3 Legend:

RBH-Blast at 60% Identity + 50% Coverage
Green = Hit in *H. sapiens*
Red = Hit in *H. sapiens* and *T. californica*
White = Not in *H. sapiens*

181 Sphingolipid metabolism

181.1 Human Pathway: HSA00600

181.2 Number of Hits: 4

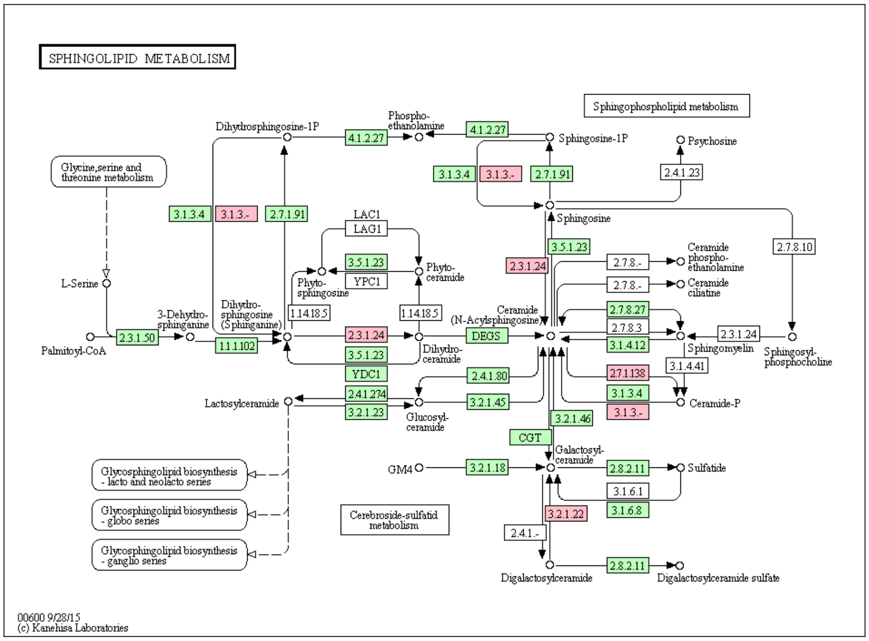

181.3 Legend:

RBH-Blast at 60% Identity + 50% Coverage
Green = Hit in *H. sapiens*
Red = Hit in *H. sapiens* and *T. californica*
White = Not in *H. sapiens*

182 Arachidonic acid metabolism

182.1 Human Pathway: HSA00590

182.2 Number of Hits: 4

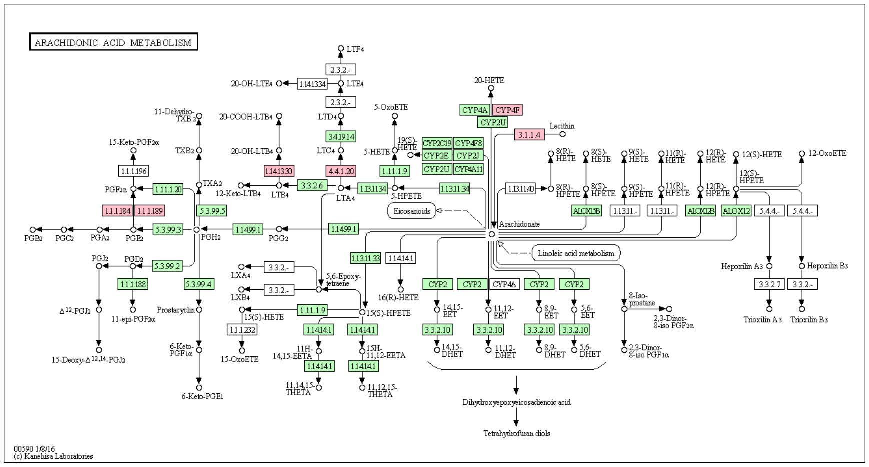

182.3 Legend:

RBH-Blast at 60% Identity + 50% Coverage
Green = Hit in *H. sapiens*
Red = Hit in *H. sapiens* and *T. californica*
White = Not in *H. sapiens*

183 Butanoate metabolism

183.1 Human Pathway: HSA00650

183.2 Number of Hits: 4

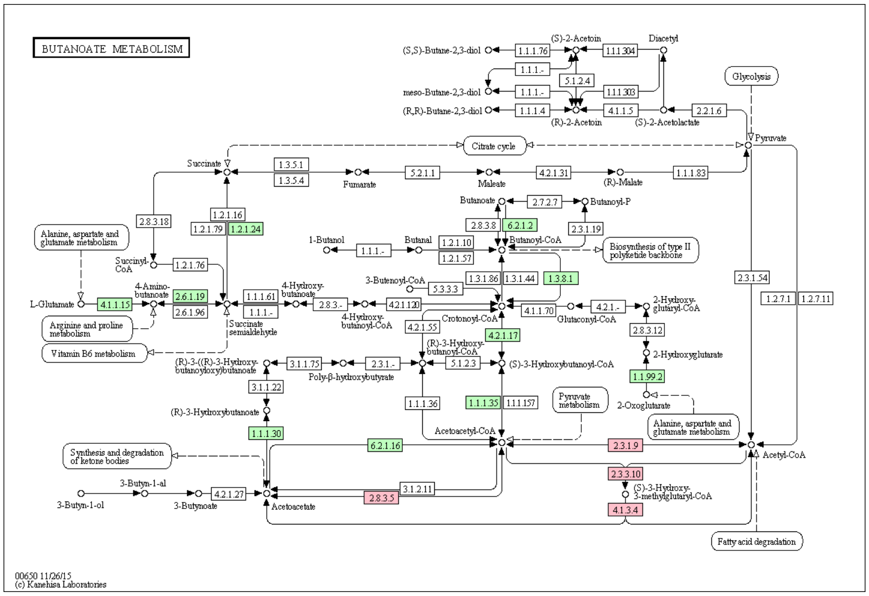

183.3 Legend:

RBH-Blast at 60% Identity + 50% Coverage
Green = Hit in *H. sapiens*
Red = Hit in *H. sapiens* and *T. californica*
White = Not in *H. sapiens*

184 Homologous recombination

184.1 Human Pathway: HSA03440

184.2 Number of Hits: 4

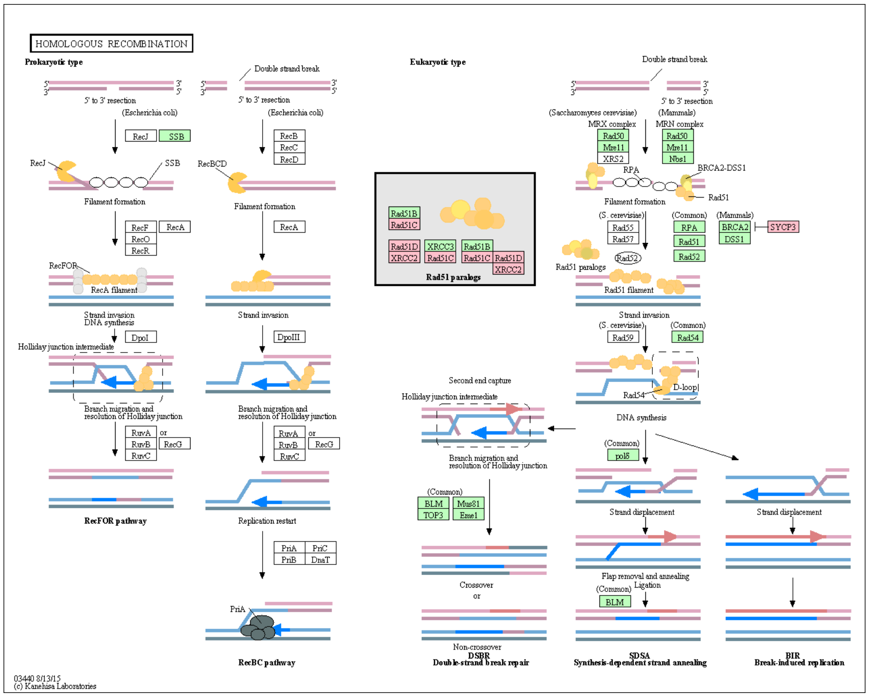

184.3 Legend:

RBH-Blast at 60% Identity + 50% Coverage
Green = Hit in *H. sapiens*
Red = Hit in *H. sapiens* and *T. californica*
White = Not in *H. sapiens*

185 Cytosolic DNA-sensing pathway

185.1 Human Pathway: HSA04623

185.2 Number of Hits: 4

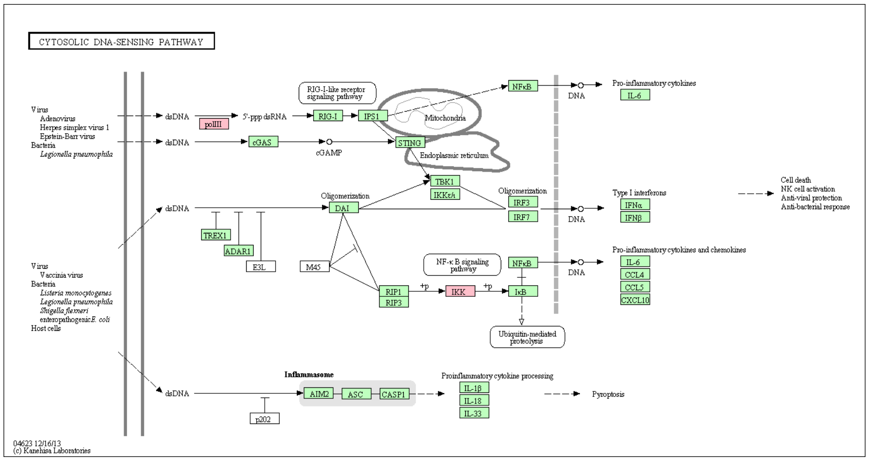

185.3 Legend:

RBH-Blast at 60% Identity + 50% Coverage
Green = Hit in *H. sapiens*
Red = Hit in *H. sapiens* and *T. californica*
White = Not in *H. sapiens*

186 Gastric acid secretion

186.1 Human Pathway: HSA04971

186.2 Number of Hits: 4

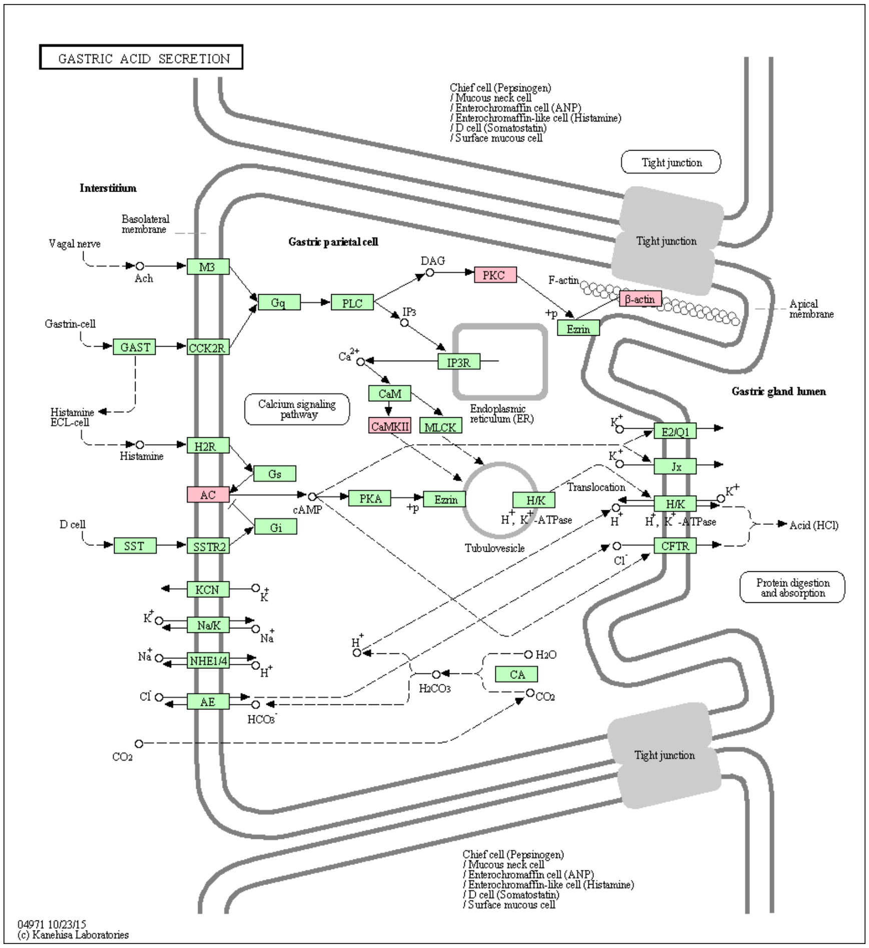

186.3 Legend:

RBH-Blast at 60% Identity + 50% Coverage
Green = Hit in *H. sapiens*
Red = Hit in *H. sapiens* and *T. californica*
White = Not in *H. sapiens*

187 Tryptophan metabolism

187.1 Human Pathway: HSA00380

187.2 Number of Hits: 4

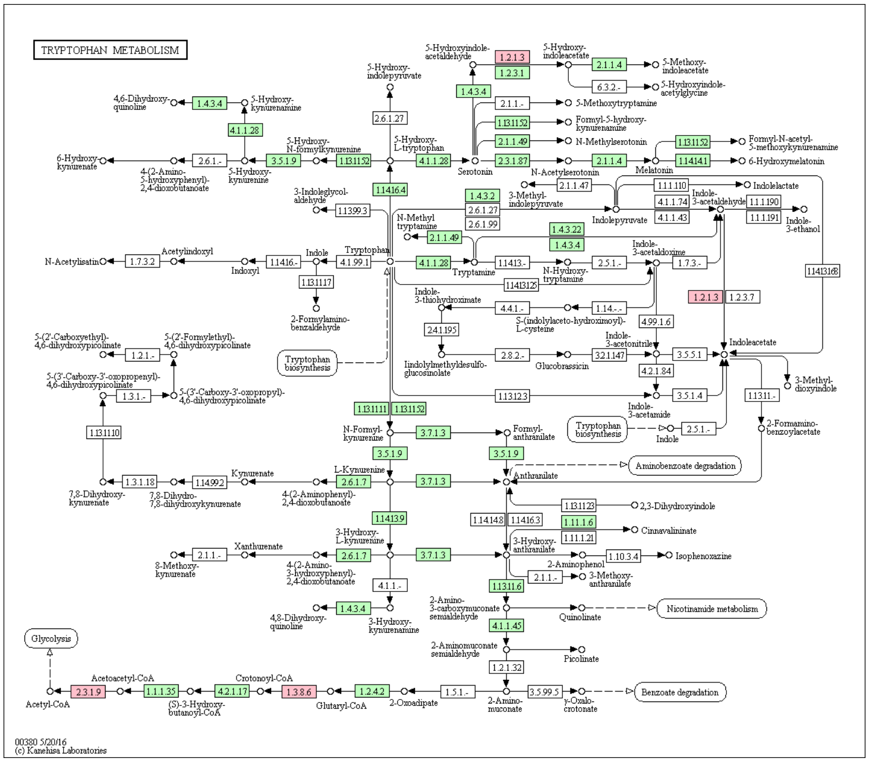

187.3 Legend:

RBH-Blast at 60% Identity + 50% Coverage
Green = Hit in *H. sapiens*
Red = Hit in *H. sapiens* and *T. californica*
White = Not in *H. sapiens*

188 Nucleotide excision repair

188.1 Human Pathway: HSA03420

188.2 Number of Hits: 4

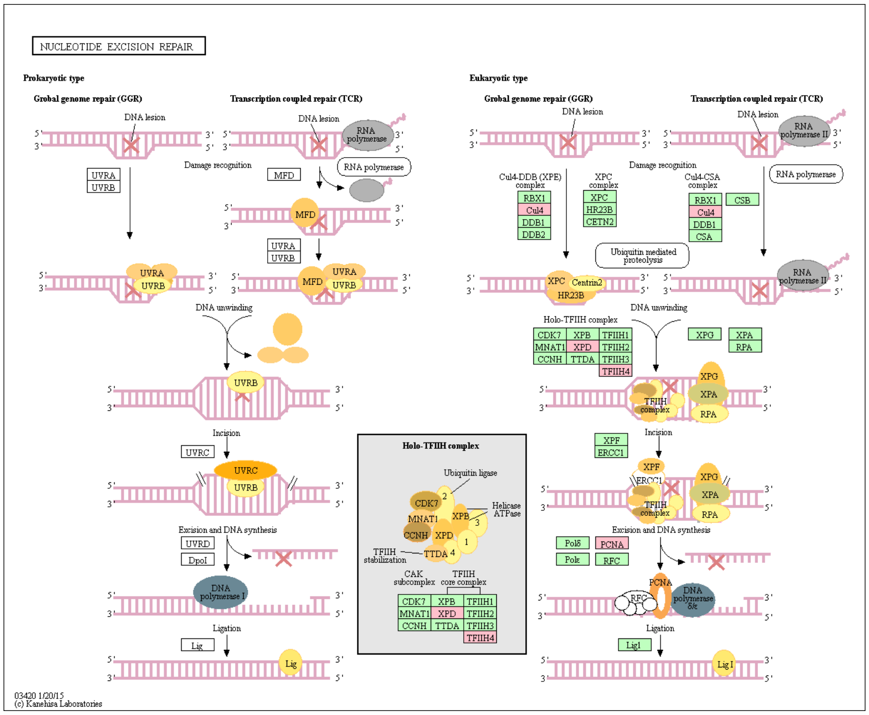

188.3 Legend:

RBH-Blast at 60% Identity + 50% Coverage
Green = Hit in *H. sapiens*
Red = Hit in *H. sapiens* and *T. californica*
White = Not in *H. sapiens*

189 Cytokine-cytokine receptor interaction

189.1 Human Pathway: HSA04060

189.2 Number of Hits: 4

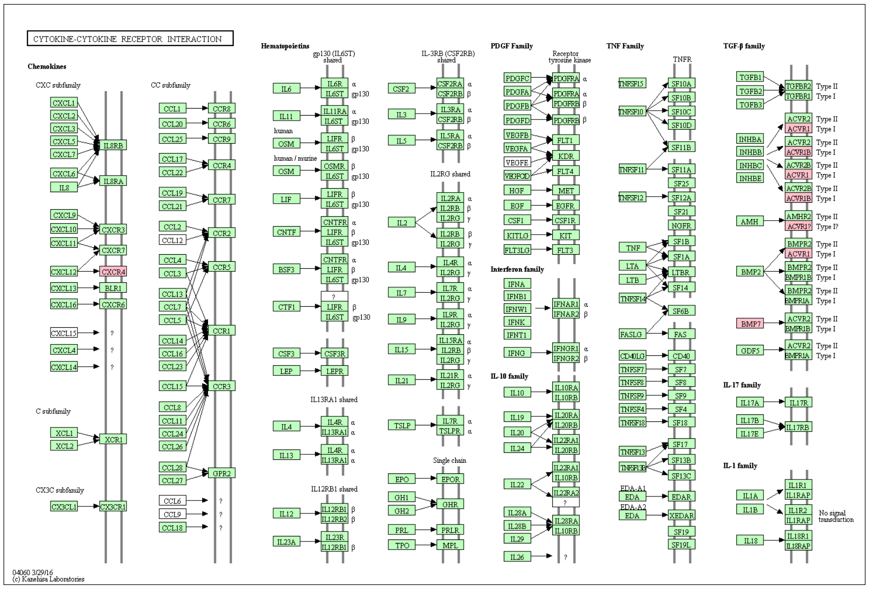

189.3 Legend:

RBH-Blast at 60% Identity + 50% Coverage
Green = Hit in *H. sapiens*
Red = Hit in *H. sapiens* and *T. californica*
White = Not in *H. sapiens*

190 Fructose and mannose metabolism

190.1 Human Pathway: HSA00051

190.2 Number of Hits: 4

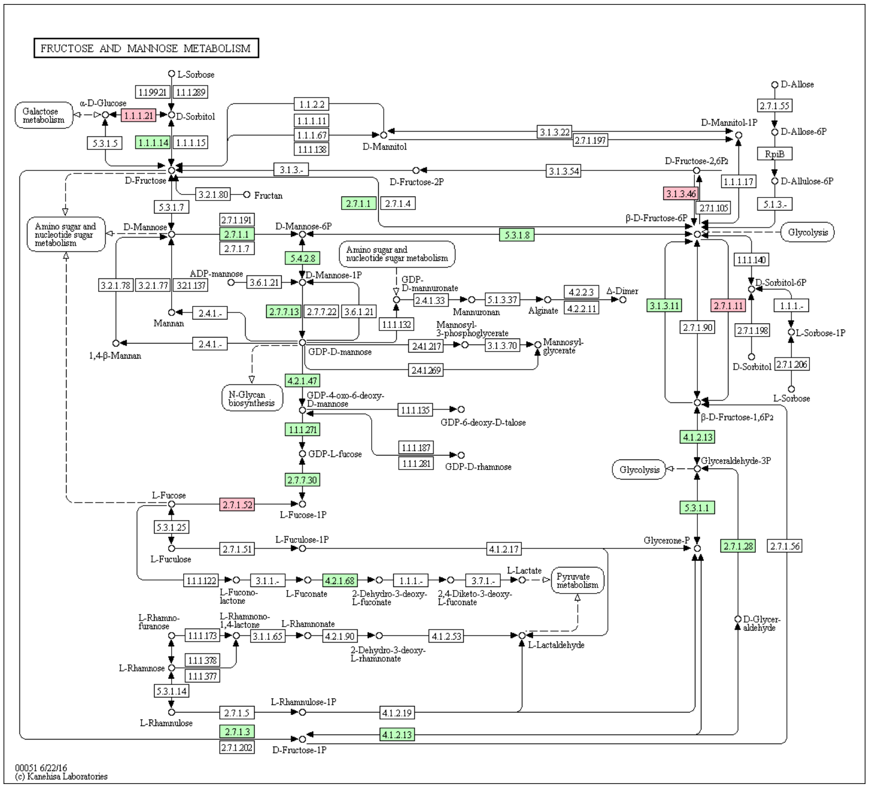

190.3 Legend:

RBH-Blast at 60% Identity + 50% Coverage
Green = Hit in *H. sapiens*
Red = Hit in *H. sapiens* and *T. californica*
White = Not in *H. sapiens*

191 Platinum drug resistance

191.1 Human Pathway: HSA01524

191.2 Number of Hits: 4

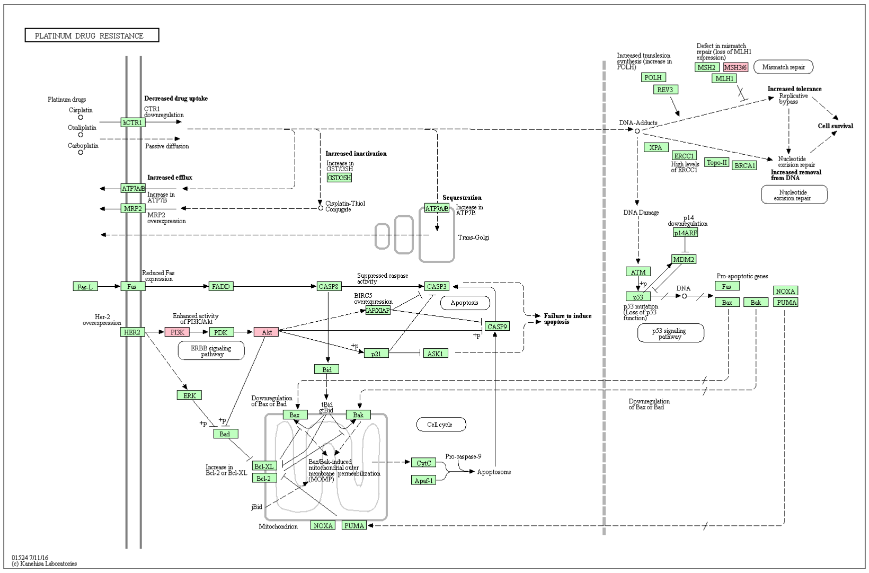

191.3 Legend:

RBH-Blast at 60% Identity + 50% Coverage
Green = Hit in *H. sapiens*
Red = Hit in *H. sapiens* and *T. californica*
White = Not in *H. sapiens*

192 beta-Alanine metabolism

192.1 Human Pathway: HSA00410

192.2 Number of Hits: 4

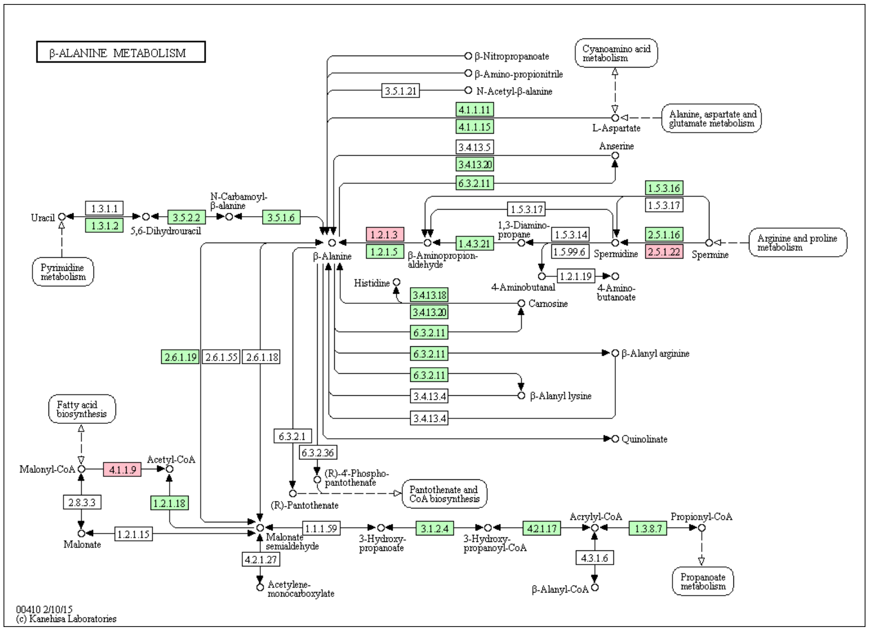

192.3 Legend:

RBH-Blast at 60% Identity + 50% Coverage
Green = Hit in *H. sapiens*
Red = Hit in *H. sapiens* and *T. californica*
White = Not in *H. sapiens*

193 Bile secretion

193.1 Human Pathway: HSA04976

193.2 Number of Hits: 4

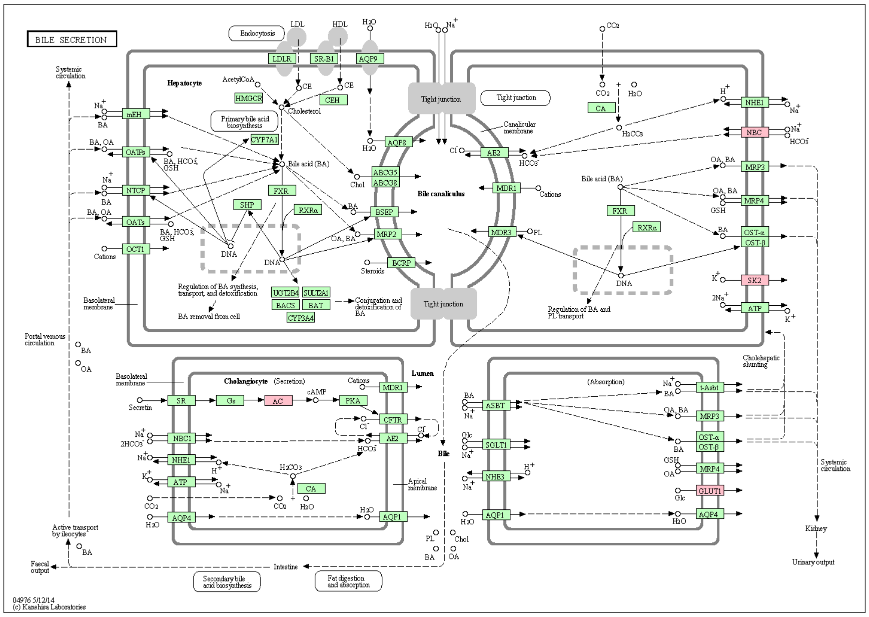

193.3 Legend:

RBH-Blast at 60% Identity + 50% Coverage
Green = Hit in *H. sapiens*
Red = Hit in *H. sapiens* and *T. californica*
White = Not in *H. sapiens*

194 Pathogenic Escherichia coli infection

194.1 Human Pathway: HSA05130

194.2 Number of Hits: 4

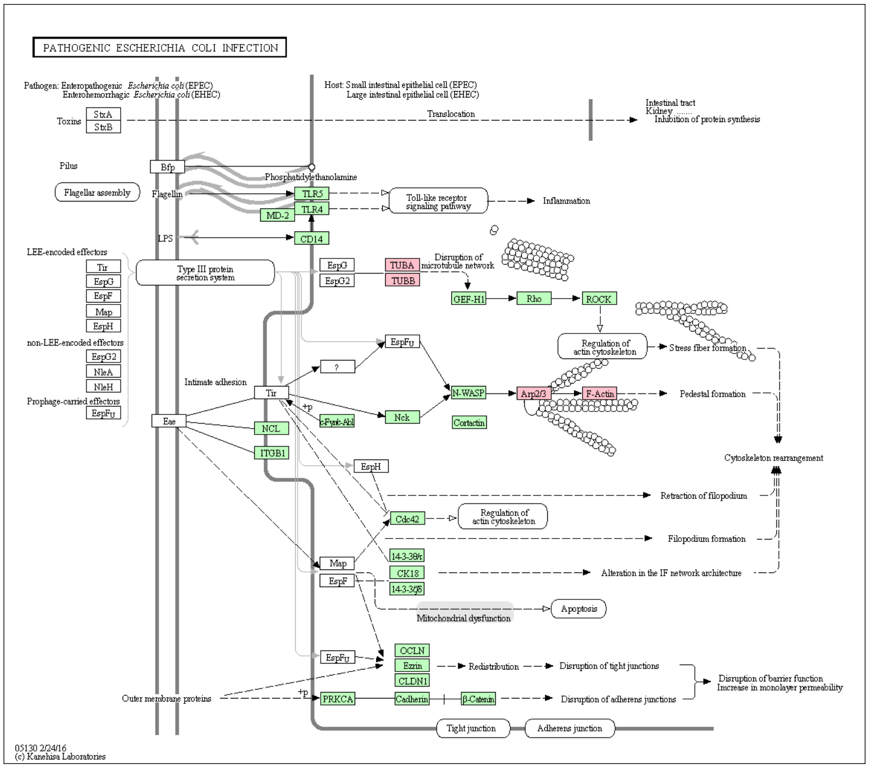

194.3 Legend:

RBH-Blast at 60% Identity + 50% Coverage
Green = Hit in *H. sapiens*
Red = Hit in *H. sapiens* and *T. californica*
White = Not in *H. sapiens*

195 Glycine, serine and threonine metabolism

195.1 Human Pathway: HSA00260

195.2 Number of Hits: 4

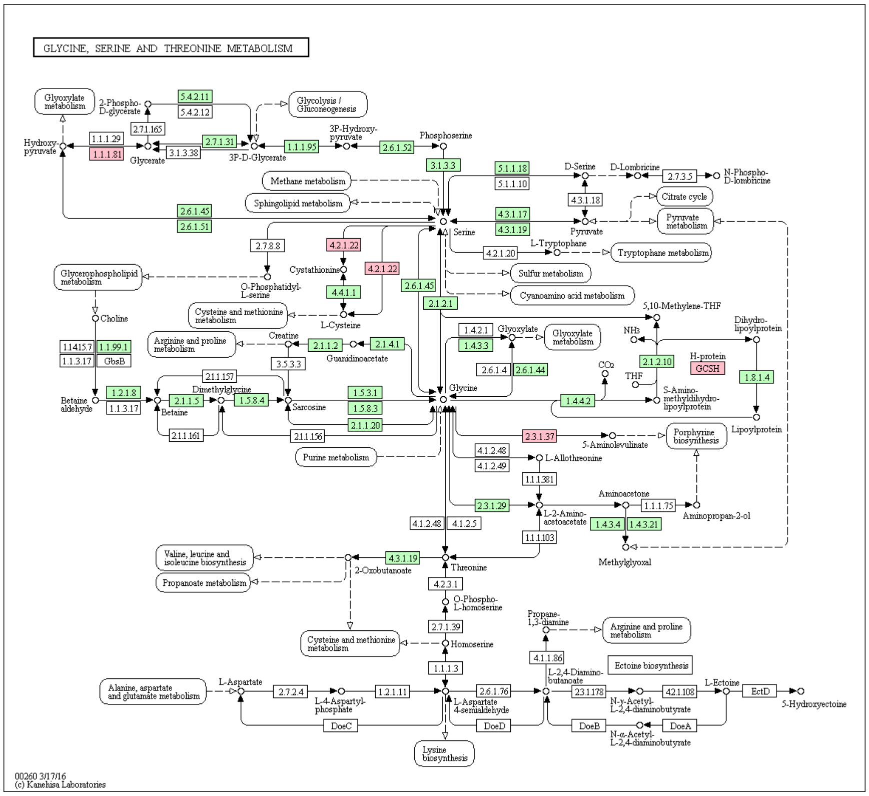

195.3 Legend:

RBH-Blast at 60% Identity + 50% Coverage
Green = Hit in *H. sapiens*
Red = Hit in *H. sapiens* and *T. californica*
White = Not in *H. sapiens*

196 Hippo signaling pathway -multiple species

196.1 Human Pathway: HSA04392

196.2 Number of Hits: 4

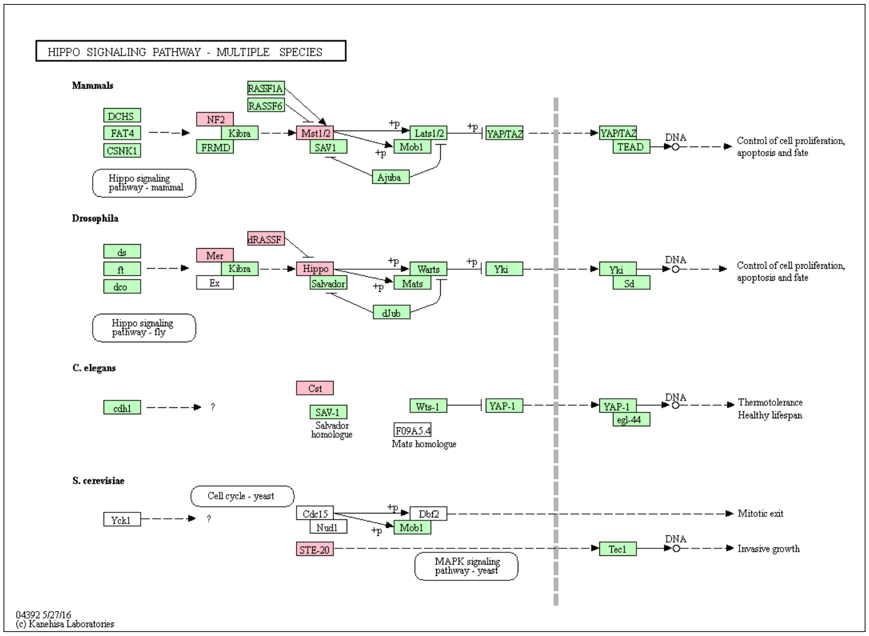

196.3 Legend:

RBH-Blast at 60% Identity + 50% Coverage
Green = Hit in *H. sapiens*
Red = Hit in *H. sapiens* and *T. californica*
White = Not in *H. sapiens*

197 Arginine and proline metabolism

197.1 Human Pathway: HSA00330

197.2 Number of Hits: 4

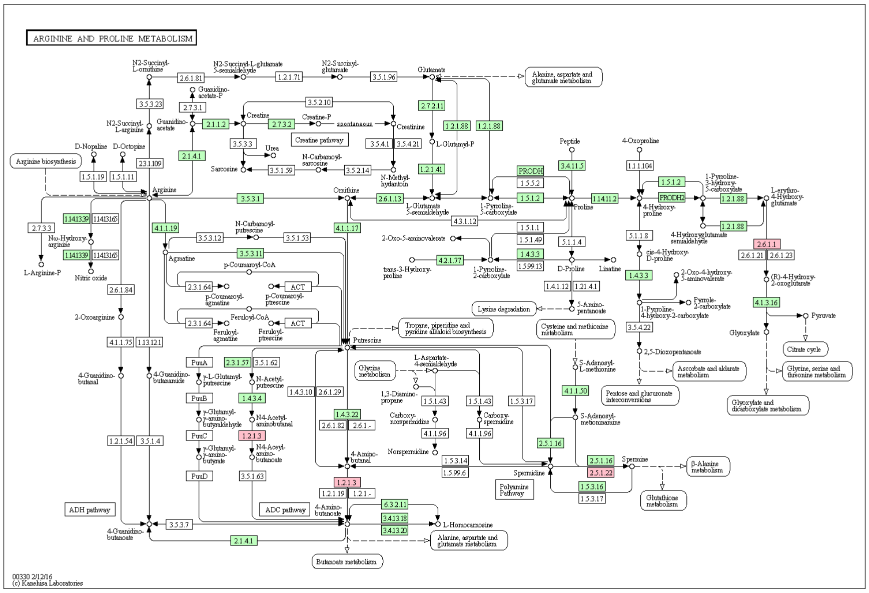

197.3 Legend:

RBH-Blast at 60% Identity + 50% Coverage
Green = Hit in *H. sapiens*
Red = Hit in *H. sapiens* and *T. californica*
White = Not in *H. sapiens*

198 RNA polymerase

198.1 Human Pathway: HSA03020

198.2 Number of Hits: 4

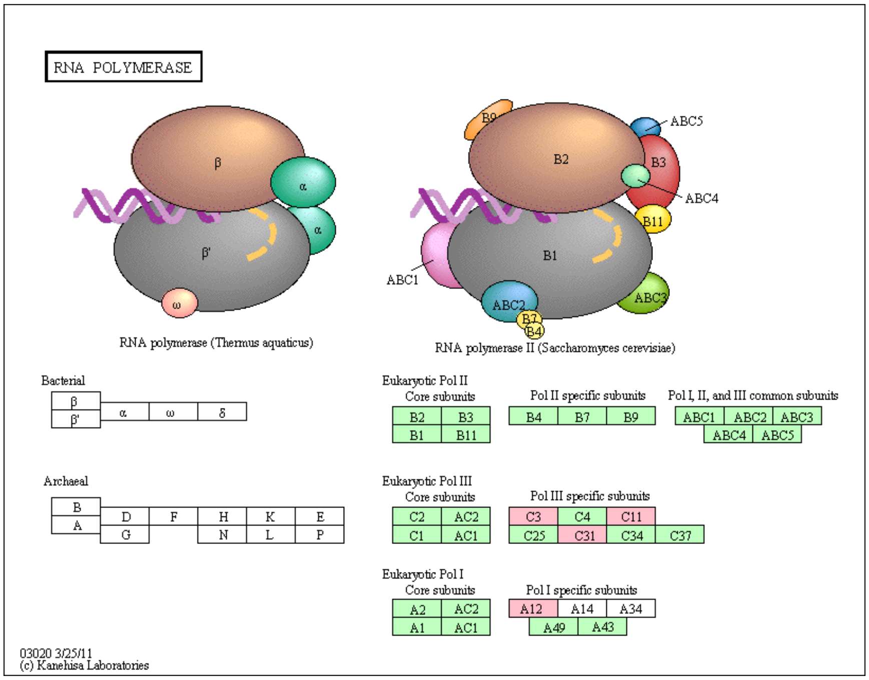

198.3 Legend:

RBH-Blast at 60% Identity + 50% Coverage
Green = Hit in *H. sapiens*
Red = Hit in *H. sapiens* and *T. californica*
White = Not in *H. sapiens*

199 Pentose phosphate pathway

199.1 Human Pathway: HSA00030

199.2 Number of Hits: 4

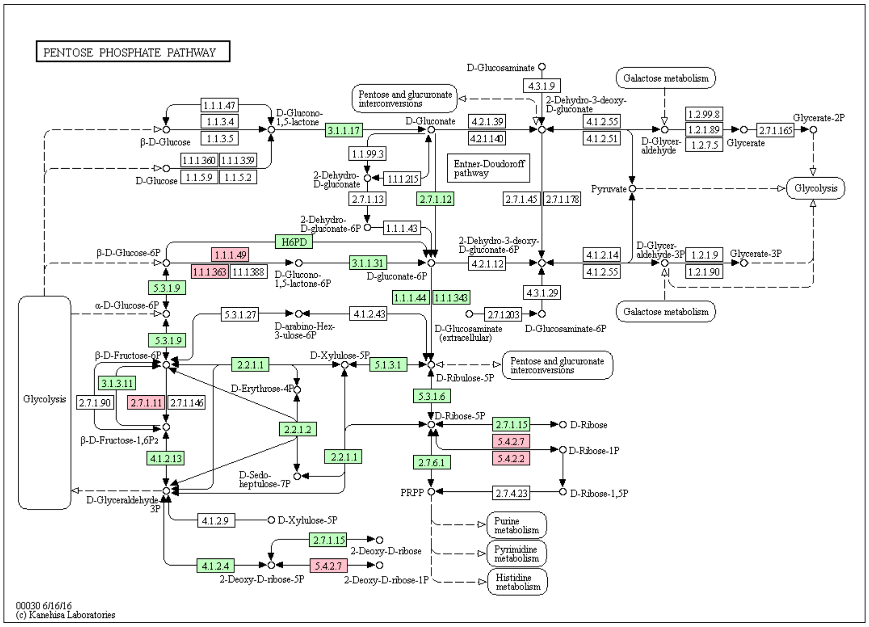

199.3 Legend:

RBH-Blast at 60% Identity + 50% Coverage
Green = Hit in *H. sapiens*
Red = Hit in *H. sapiens* and *T. californica*
White = Not in *H. sapiens*

200 Mineral absorption

200.1 Human Pathway: HSA04978

200.2 Number of Hits: 3

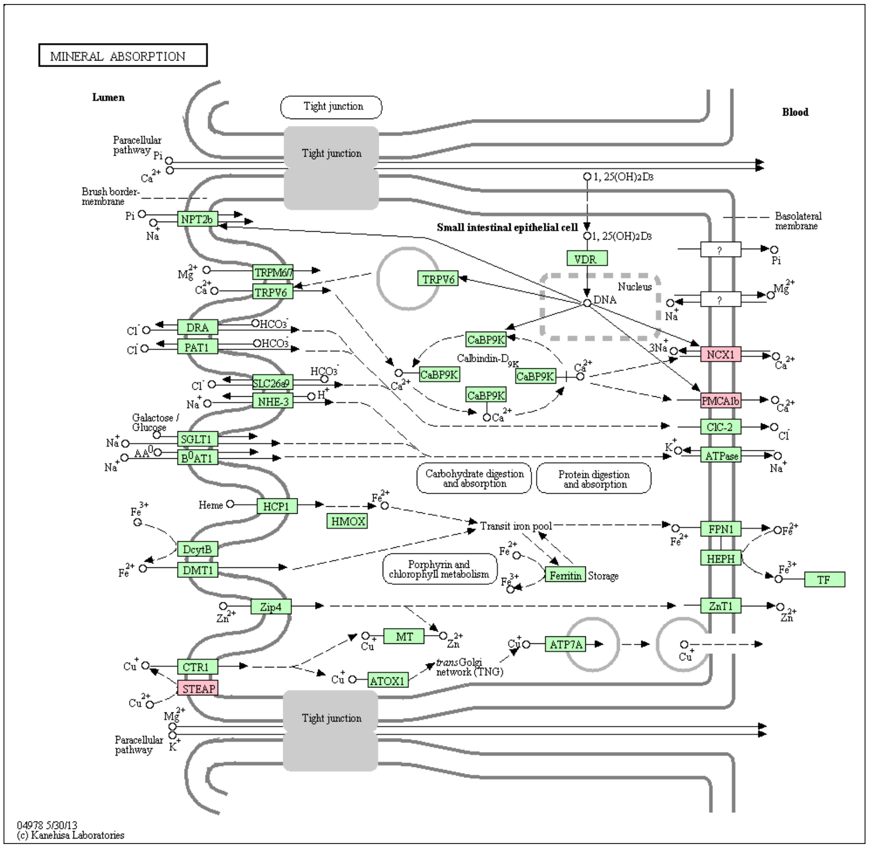

200.3 Legend:

RBH-Blast at 60% Identity + 50% Coverage
Green = Hit in *H. sapiens*
Red = Hit in *H. sapiens* and *T. californica*
White = Not in *H. sapiens*

201 Legionellosis

201.1 Human Pathway: HSA05134

201.2 Number of Hits: 3

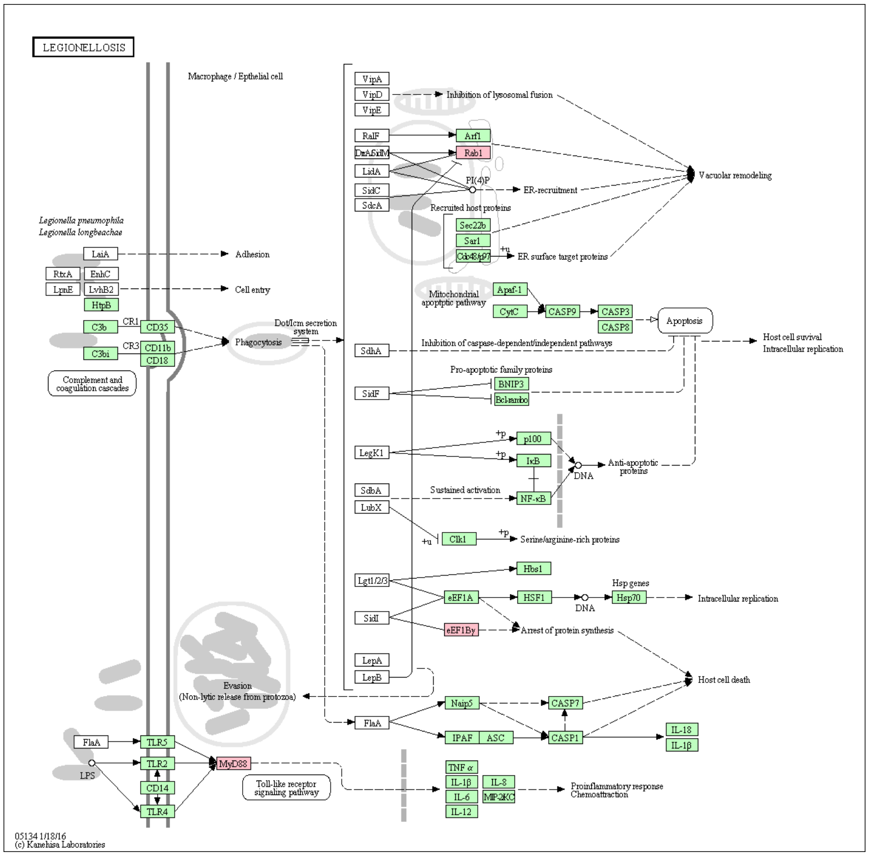

201.3 Legend:

RBH-Blast at 60% Identity + 50% Coverage
Green = Hit in *H. sapiens*
Red = Hit in *H. sapiens* and *T. californica*
White = Not in *H. sapiens*

202 Porphyrin and chlorophyll metabolism

202.1 Human Pathway: HSA00860

202.2 Number of Hits: 3

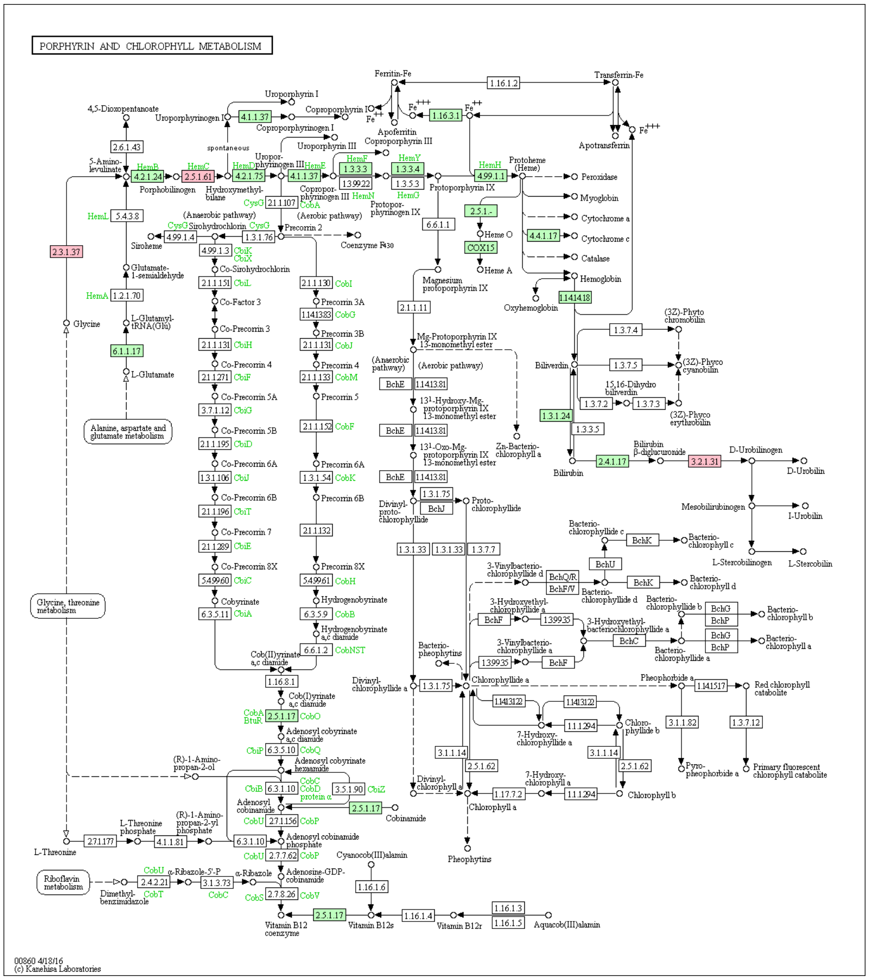

202.3 Legend:

RBH-Blast at 60% Identity + 50% Coverage
Green = Hit in *H. sapiens*
Red = Hit in *H. sapiens* and *T. californica*
White = Not in *H. sapiens*

203 Glycosaminoglycan biosynthesis - heparan sulfate / heparin

203.1 Human Pathway: HSA00534

203.2 Number of Hits: 3

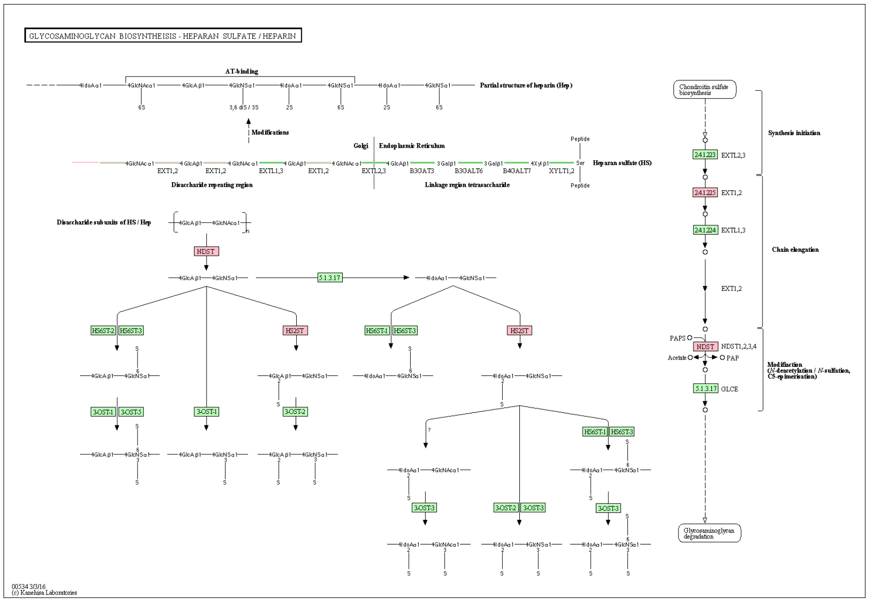

203.3 Legend:

RBH-Blast at 60% Identity + 50% Coverage
Green = Hit in *H. sapiens*
Red = Hit in *H. sapiens* and *T. californica*
White = Not in *H. sapiens*

204 Renin secretion

204.1 Human Pathway: HSA04924

204.2 Number of Hits: 3

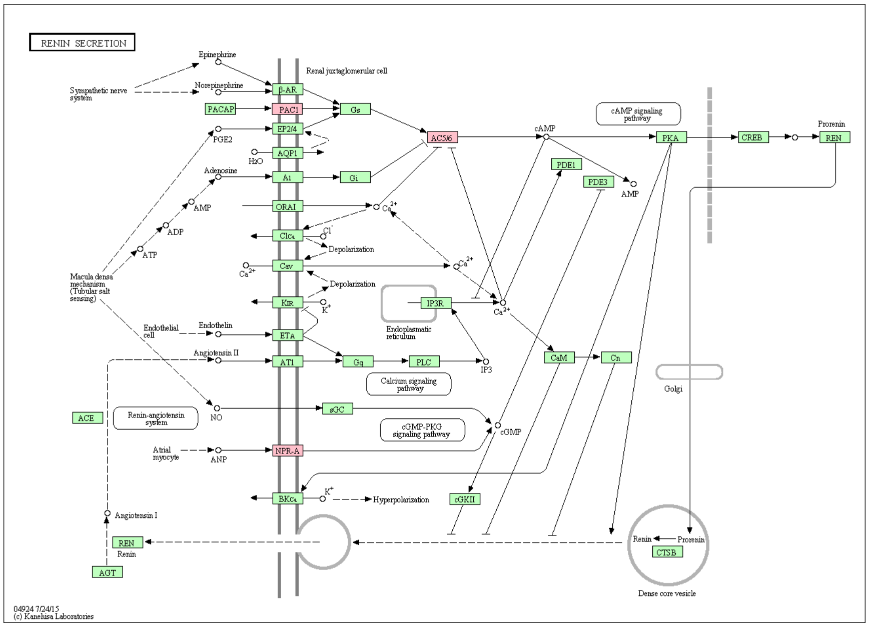

204.3 Legend:

RBH-Blast at 60% Identity + 50% Coverage
Green = Hit in *H. sapiens*
Red = Hit in *H. sapiens* and *T. californica*
White = Not in *H. sapiens*

205 Tyrosine metabolism

205.1 Human Pathway: HSA00350

205.2 Number of Hits: 3

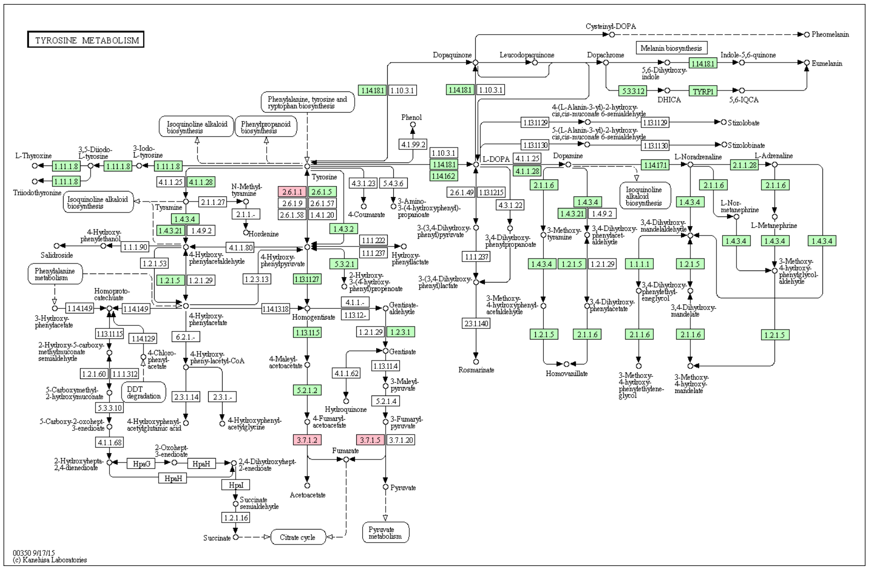

205.3 Legend:

RBH-Blast at 60% Identity + 50% Coverage
Green = Hit in *H. sapiens*
Red = Hit in *H. sapiens* and *T. californica*
White = Not in *H. sapiens*

206 Steroid biosynthesis

206.1 Human Pathway: HSA00100

206.2 Number of Hits: 3

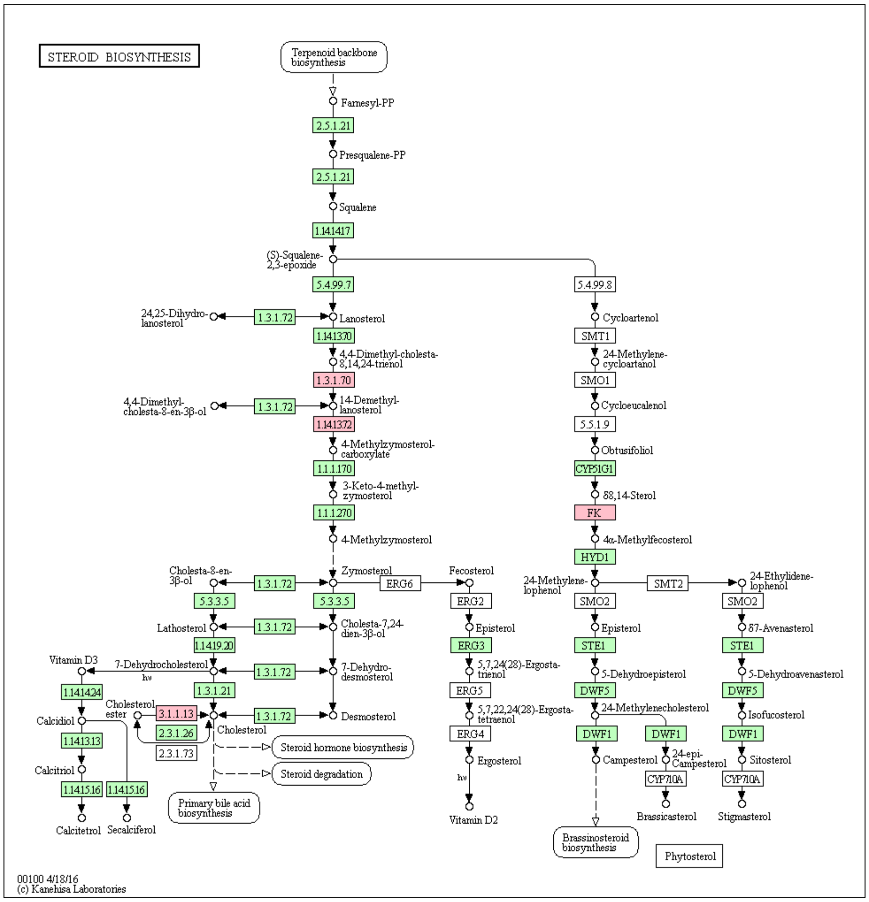

206.3 Legend:

RBH-Blast at 60% Identity + 50% Coverage
Green = Hit in *H. sapiens*
Red = Hit in *H. sapiens* and *T. californica*
White = Not in *H. sapiens*

207 Thyroid hormone synthesis

207.1 Human Pathway: HSA04918

207.2 Number of Hits: 3

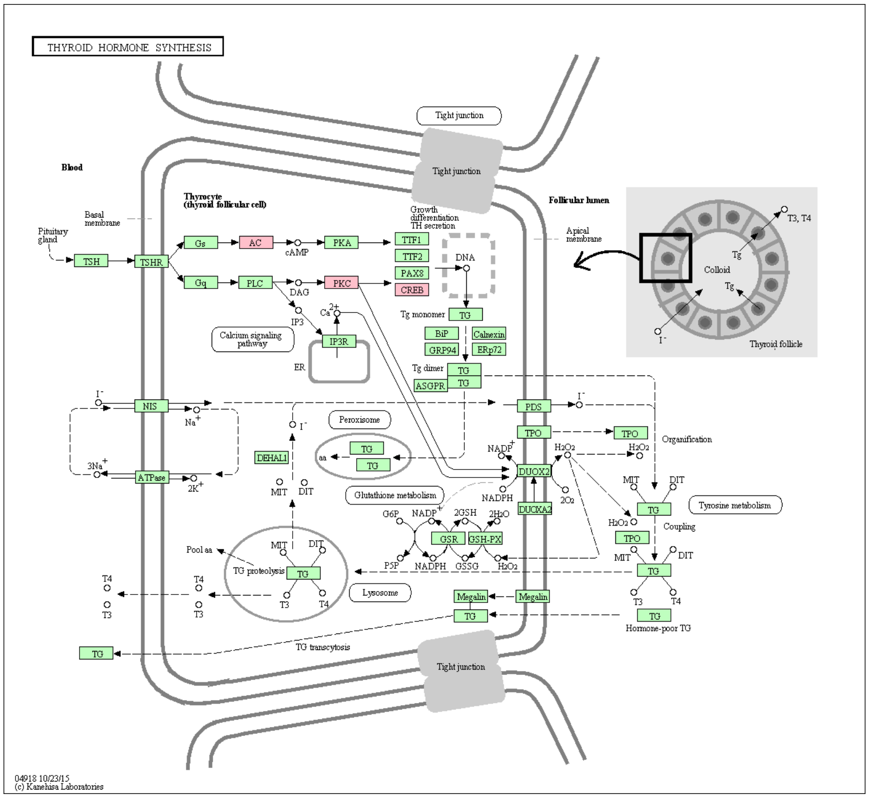

207.3 Legend:

RBH-Blast at 60% Identity + 50% Coverage
Green = Hit in *H. sapiens*
Red = Hit in *H. sapiens* and *T. californica*
White = Not in *H. sapiens*

208 Folate biosynthesis

208.1 Human Pathway: HSA00790

208.2 Number of Hits: 3

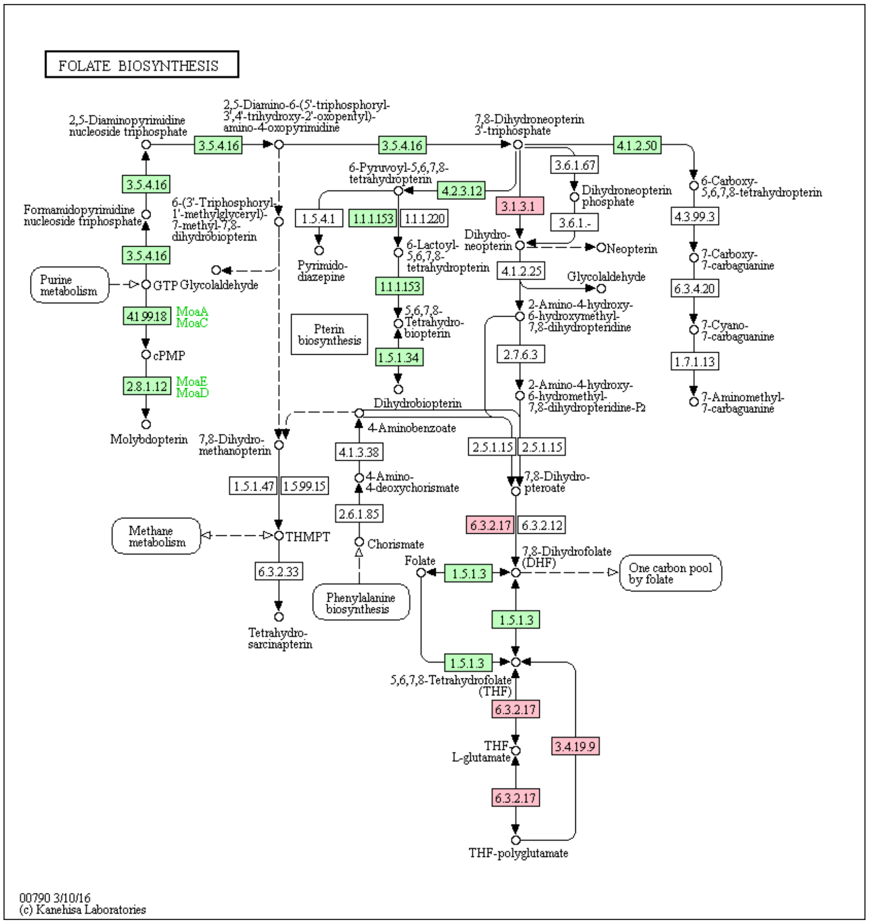

208.3 Legend:

RBH-Blast at 60% Identity + 50% Coverage
Green = Hit in *H. sapiens*
Red = Hit in *H. sapiens* and *T. californica*
White = Not in *H. sapiens*

209 Basal cell carcinoma

209.1 Human Pathway: HSA05217

209.2 Number of Hits: 3

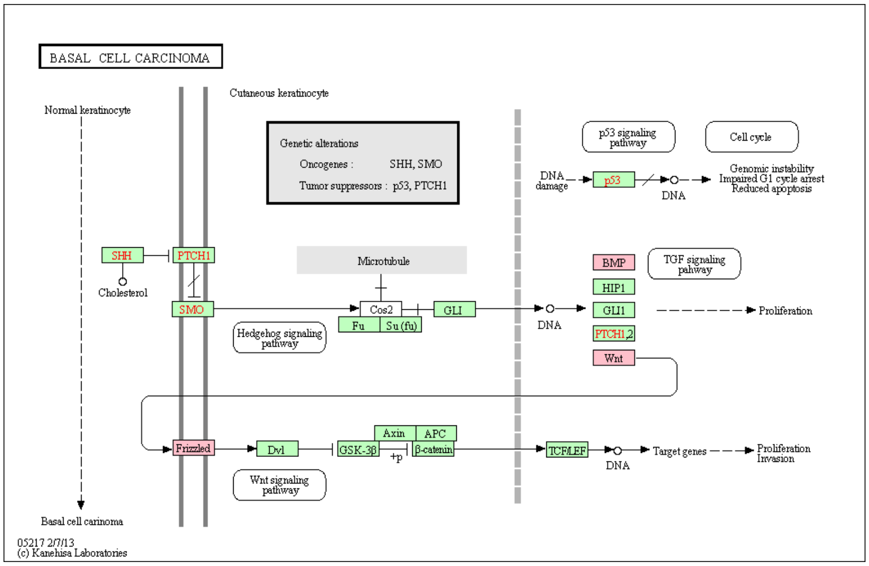

209.3 Legend:

RBH-Blast at 60% Identity + 50% Coverage
Green = Hit in *H. sapiens*
Red = Hit in *H. sapiens* and *T. californica*
White = Not in *H. sapiens*

210 N-Glycan biosynthesis

210.1 Human Pathway: HSA00510

210.2 Number of Hits: 3

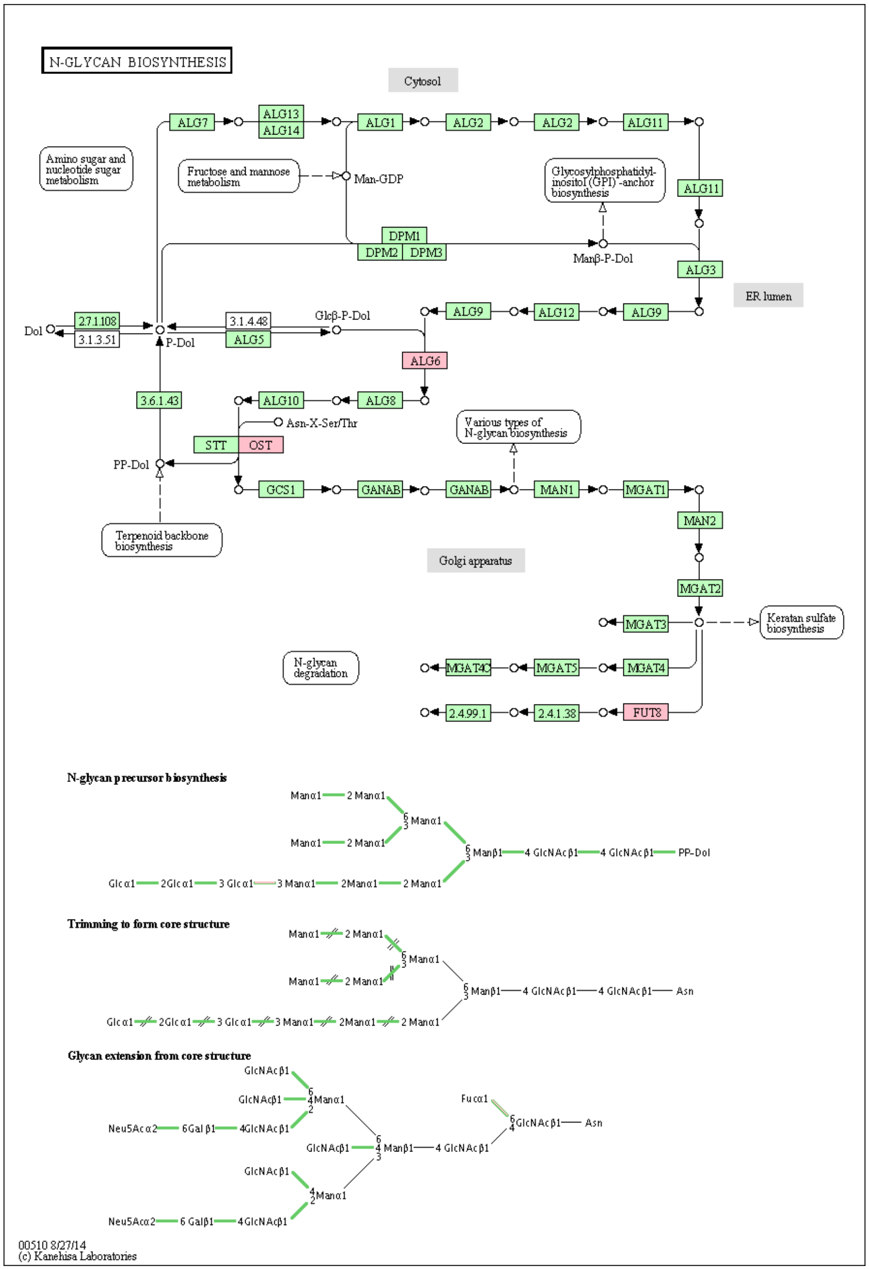

210.3 Legend:

RBH-Blast at 60% Identity + 50% Coverage
Green = Hit in *H. sapiens*
Red = Hit in *H. sapiens* and *T. californica*
White = Not in *H. sapiens*

---

211 Glutathione metabolism

211.1 Human Pathway: HSA00480

211.2 Number of Hits: 3

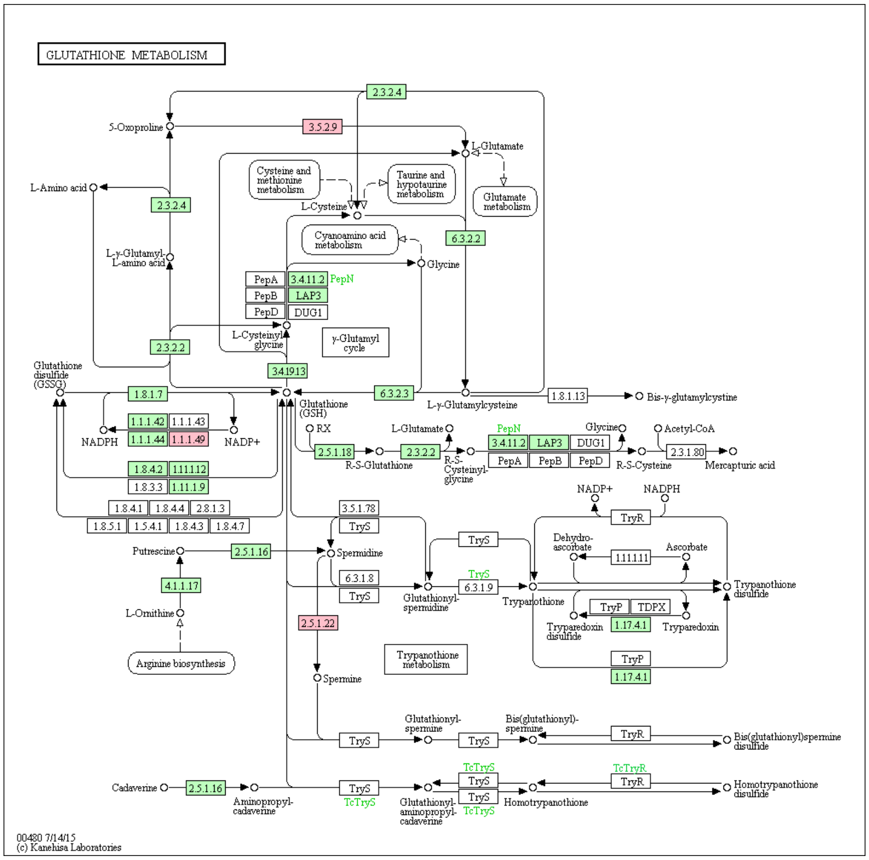

211.3 Legend:

RBH-Blast at 60% Identity + 50% Coverage
Green = Hit in *H. sapiens*
Red = Hit in *H. sapiens* and *T. californica*
White = Not in *H. sapiens*

212 Long-term depression

212.1 Human Pathway: HSA04730

212.2 Number of Hits: 3

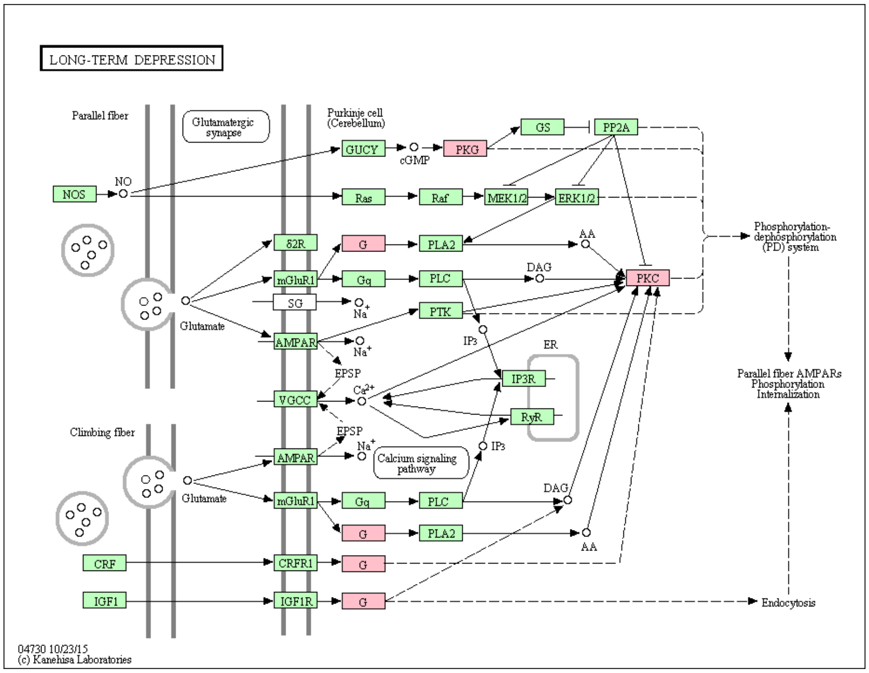

212.3 Legend:

RBH-Blast at 60% Identity + 50% Coverage
Green = Hit in *H. sapiens*
Red = Hit in *H. sapiens* and *T. californica*
White = Not in *H. sapiens*

213 Regulation of autophagy

213.1 Human Pathway: HSA04140

213.2 Number of Hits: 3

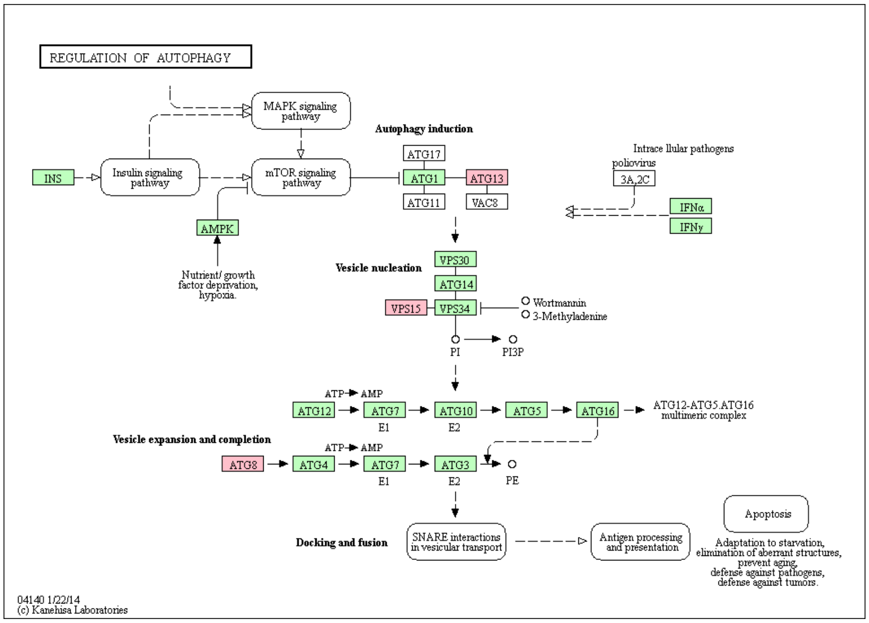

213.3 Legend:

RBH-Blast at 60% Identity + 50% Coverage
Green = Hit in *H. sapiens*
Red = Hit in *H. sapiens* and *T. californica*
White = Not in *H. sapiens*

214 Fatty acid elongation

214.1 Human Pathway: HSA00062

214.2 Number of Hits: 3

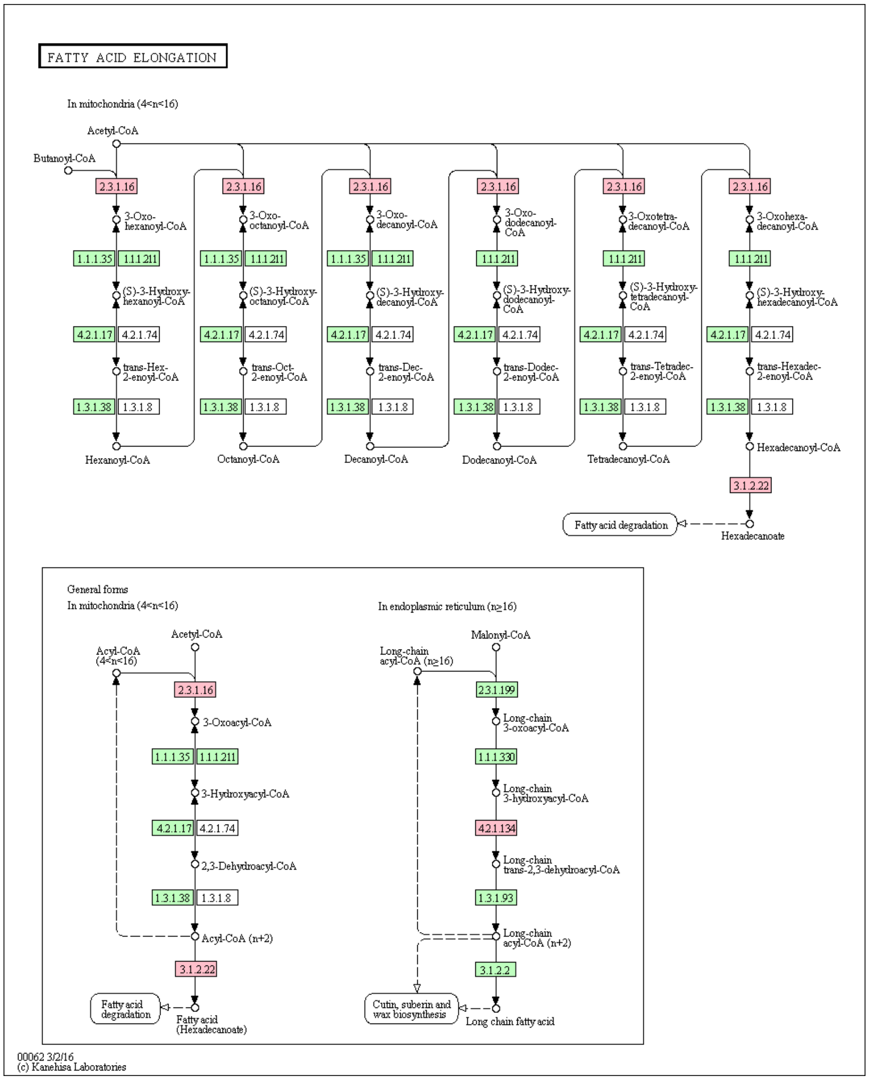

214.3 Legend:

RBH-Blast at 60% Identity + 50% Coverage
Green = Hit in *H. sapiens*
Red = Hit in *H. sapiens* and *T. californica*
White = Not in *H. sapiens*

---

215 Aldosterone-regulated sodium reabsorption

215.1 Human Pathway: HSA04960

215.2 Number of Hits: 3

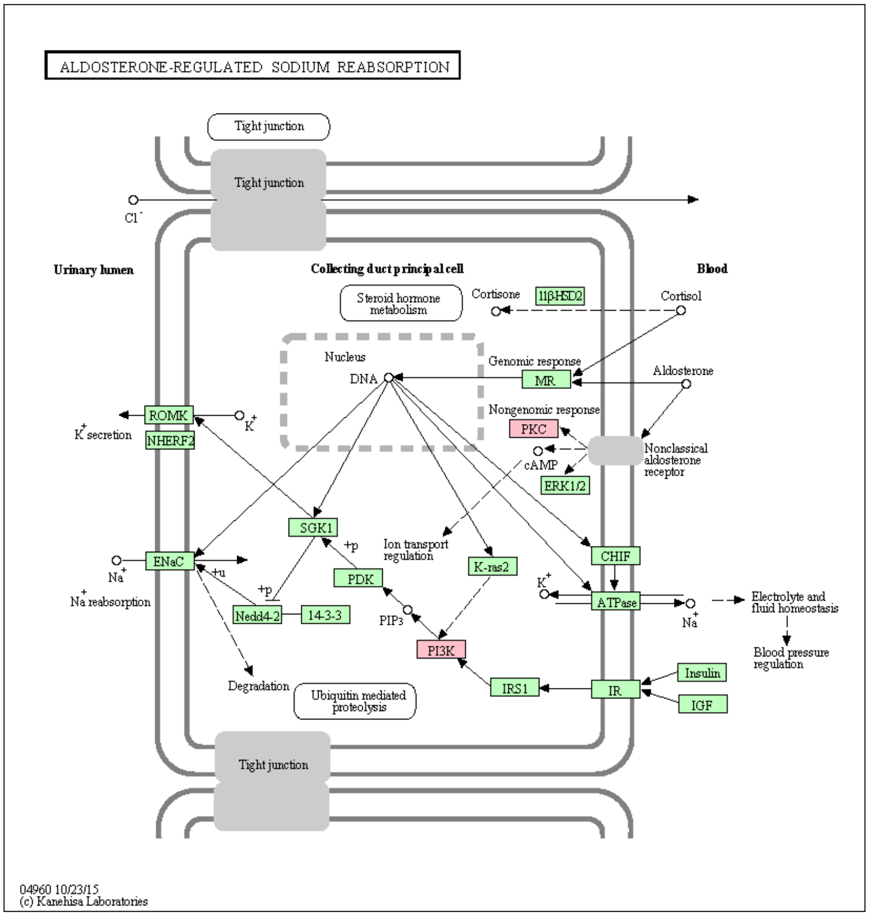

215.3 Legend:

RBH-Blast at 60% Identity + 50% Coverage
Green = Hit in *H. sapiens*
Red = Hit in *H. sapiens* and *T. californica*
White = Not in *H. sapiens*

---

216 2-Oxocarboxylic acid metabolism

216.1 Human Pathway: HSA01210

216.2 Number of Hits: 3

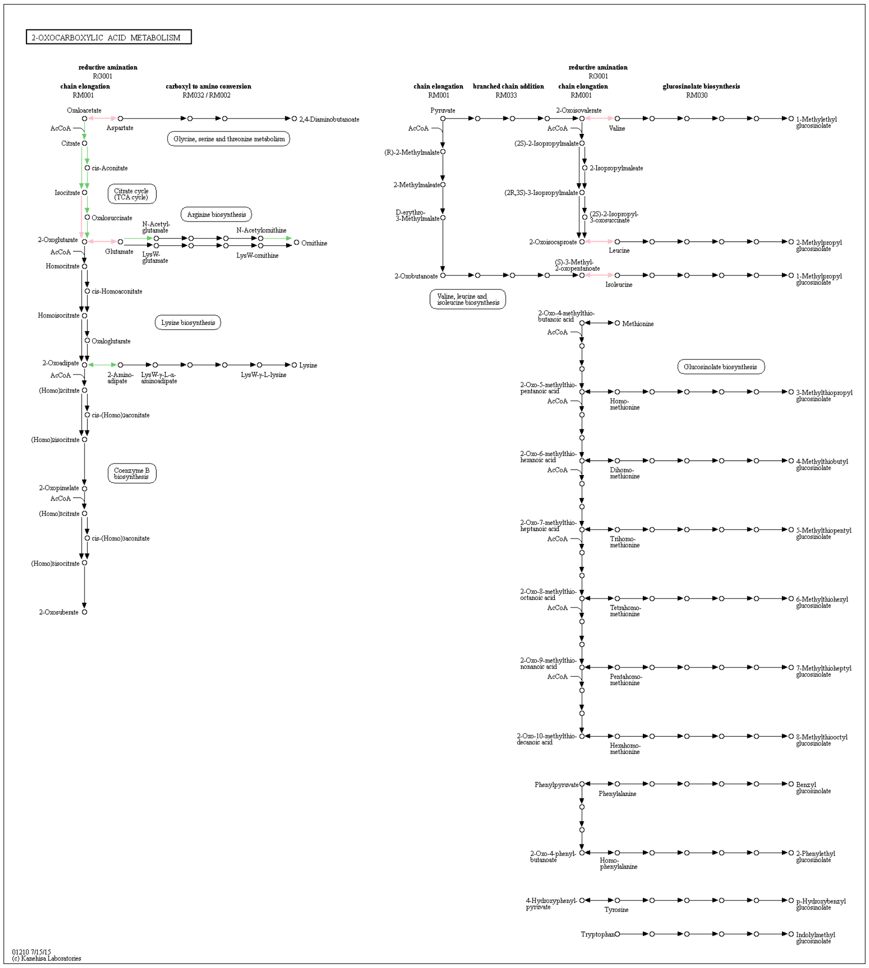

216.3 Legend:

RBH-Blast at 60% Identity + 50% Coverage
Green = Hit in *H. sapiens*
Red = Hit in *H. sapiens* and *T. californica*
White = Not in *H. sapiens*

---

217 Antigen processing and presentation

217.1 Human Pathway: HSA04612

217.2 Number of Hits: 3

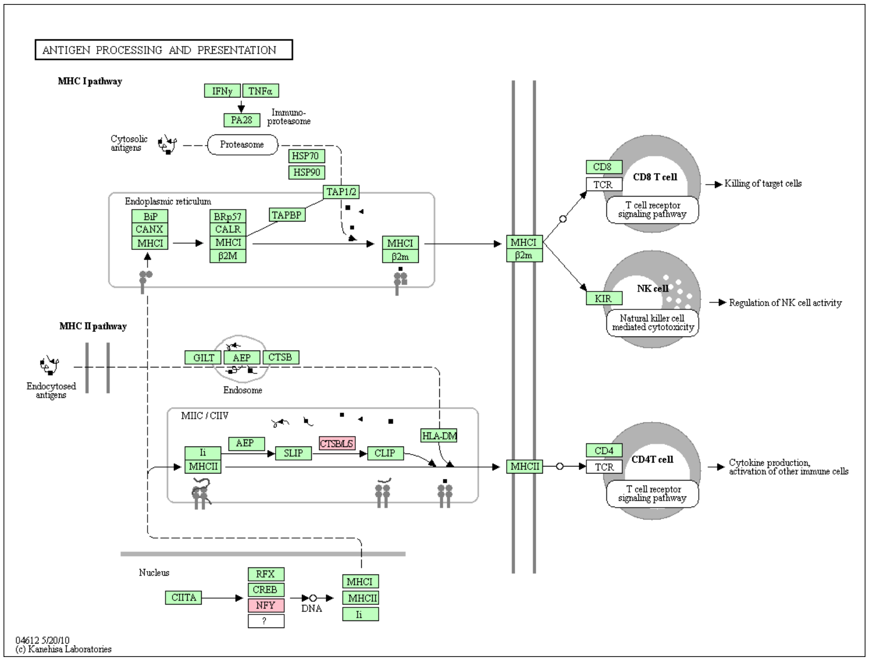

217.3 Legend:

RBH-Blast at 60% Identity + 50% Coverage
Green = Hit in *H. sapiens*
Red = Hit in *H. sapiens* and *T. californica*
White = Not in *H. sapiens*

---

218 Ascorbate and aldarate metabolism

218.1 Human Pathway: HSA00053

218.2 Number of Hits: 3

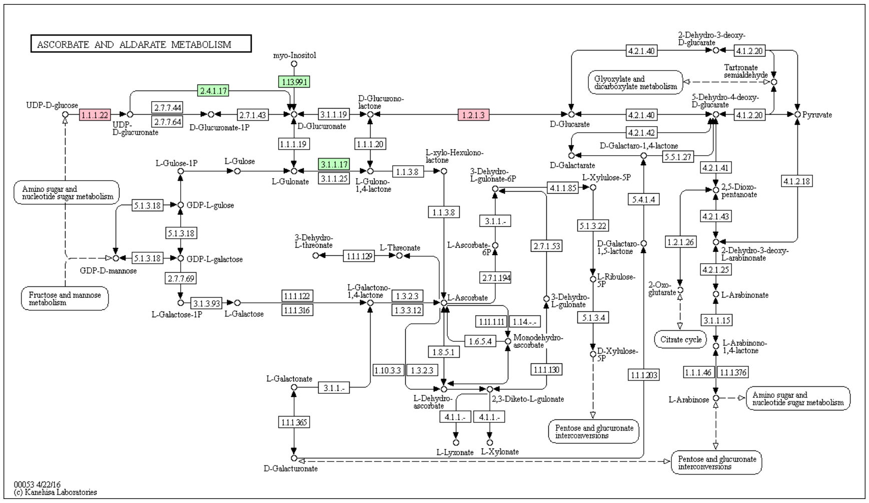

218.3 Legend:

RBH-Blast at 60% Identity + 50% Coverage
Green = Hit in *H. sapiens*
Red = Hit in *H. sapiens* and *T. californica*
White = Not in *H. sapiens*

219 Fat digestion and absorption

219.1 Human Pathway: HSA04975

219.2 Number of Hits: 3

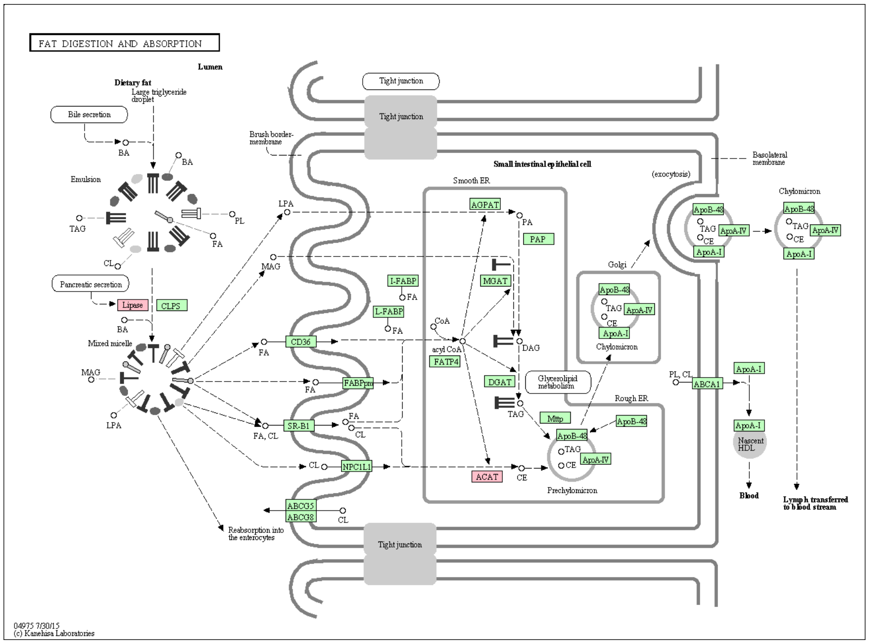

219.3 Legend:

RBH-Blast at 60% Identity + 50% Coverage
Green = Hit in *H. sapiens*
Red = Hit in *H. sapiens* and *T. californica*
White = Not in *H. sapiens*

220 Prion diseases

220.1 Human Pathway: HSA05020

220.2 Number of Hits: 3

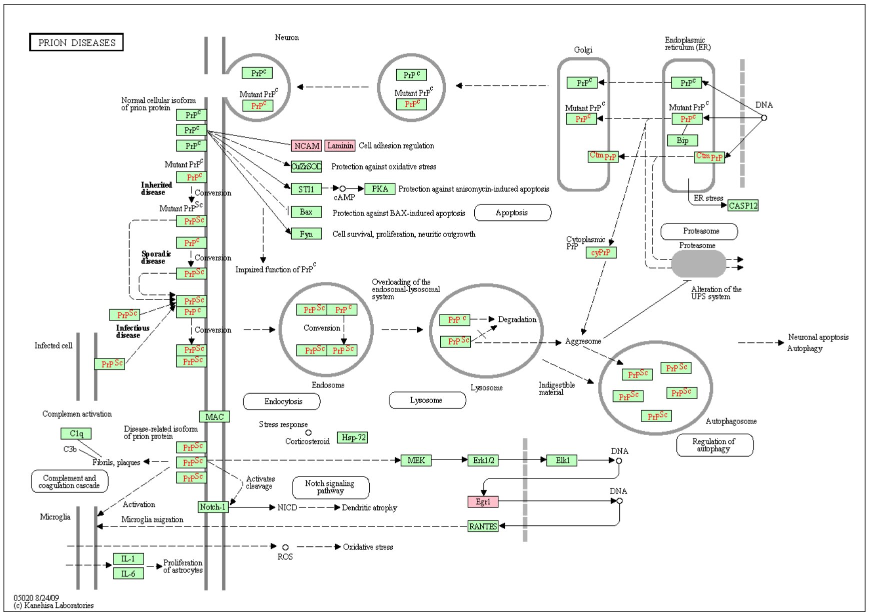

220.3 Legend:

RBH-Blast at 60% Identity + 50% Coverage
Green = Hit in *H. sapiens*
Red = Hit in *H. sapiens* and *T. californica*
White = Not in *H. sapiens*

221 Bladder cancer

221.1 Human Pathway: HSA05219

221.2 Number of Hits: 2

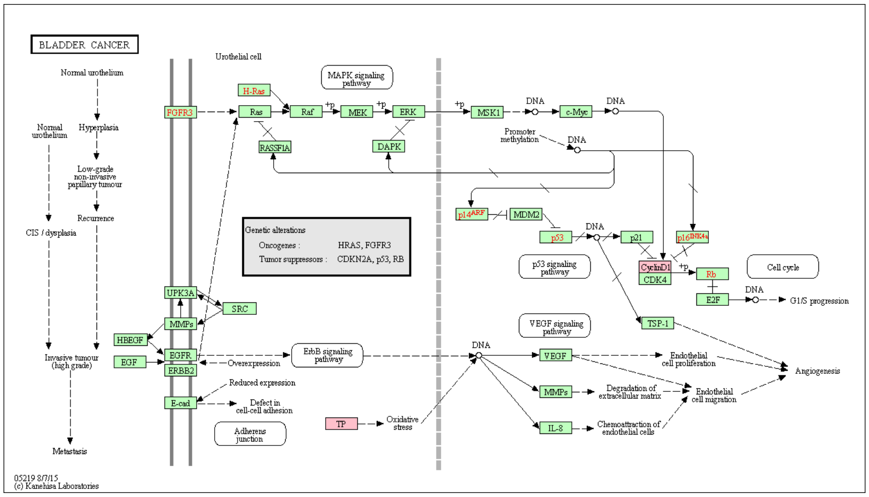

221.3 Legend:

RBH-Blast at 60% Identity + 50% Coverage
Green = Hit in *H. sapiens*
Red = Hit in *H. sapiens* and *T. californica*
White = Not in *H. sapiens*

222 RIG-I-like receptor signaling pathway

222.1 Human Pathway: HSA04622

222.2 Number of Hits: 2

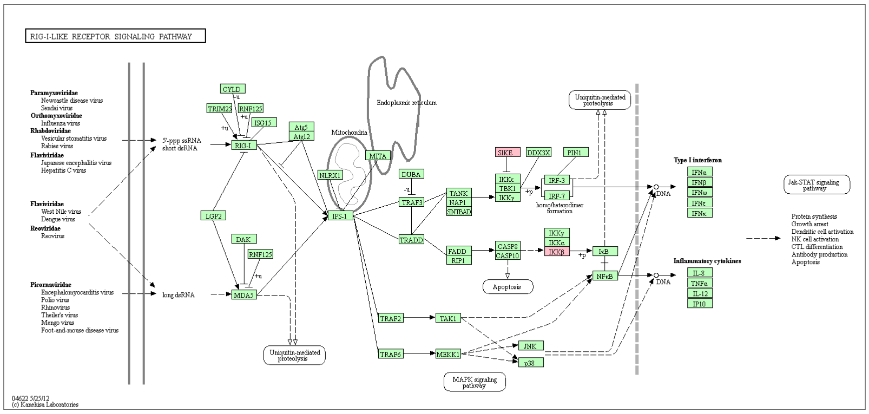

222.3 Legend:

RBH-Blast at 60% Identity + 50% Coverage
Green = Hit in *H. sapiens*
Red = Hit in *H. sapiens* and *T. californica*
White = Not in *H. sapiens*

223 Leishmaniasis

223.1 Human Pathway: HSA05140

223.2 Number of Hits: 2

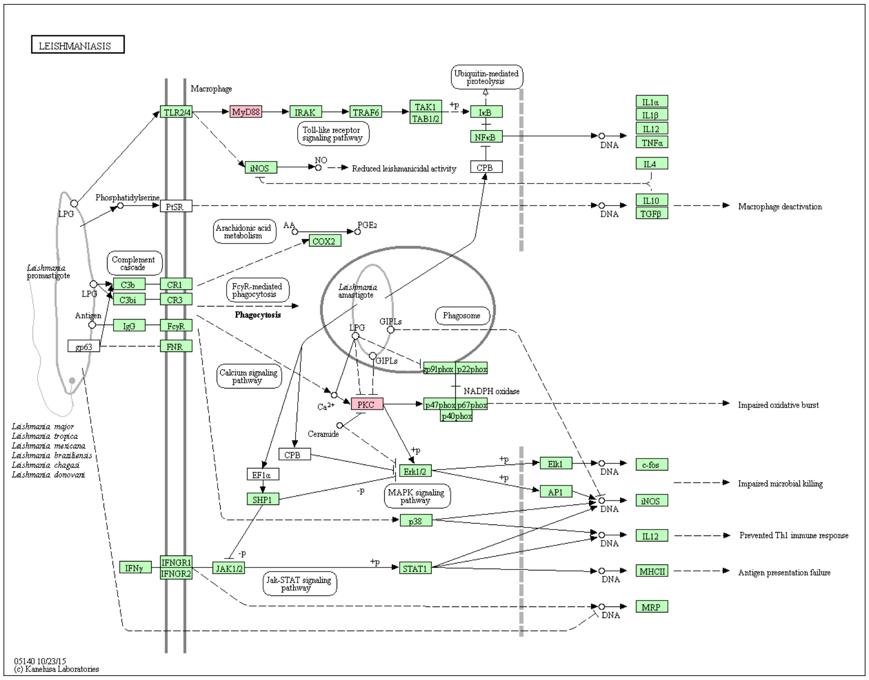

223.3 Legend:

RBH-Blast at 60% Identity + 50% Coverage
Green = Hit in *H. sapiens*
Red = Hit in *H. sapiens* and *T. californica*
White = Not in *H. sapiens*

224 Nicotinate and nicotinamide metabolism

224.1 Human Pathway: HSA00760

224.2 Number of Hits: 2

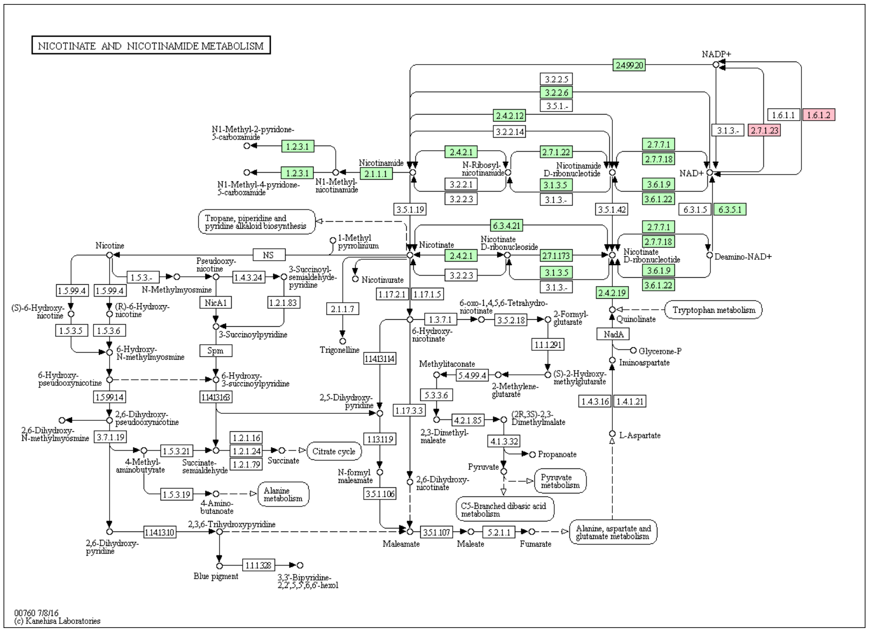

224.3 Legend:

RBH-Blast at 60% Identity + 50% Coverage
Green = Hit in *H. sapiens*
Red = Hit in *H. sapiens* and *T. californica*
White = Not in *H. sapiens*

225 Metabolism of xenobiotics by cytochrome P450

225.1 Human Pathway: HSA00980

225.2 Number of Hits: 2

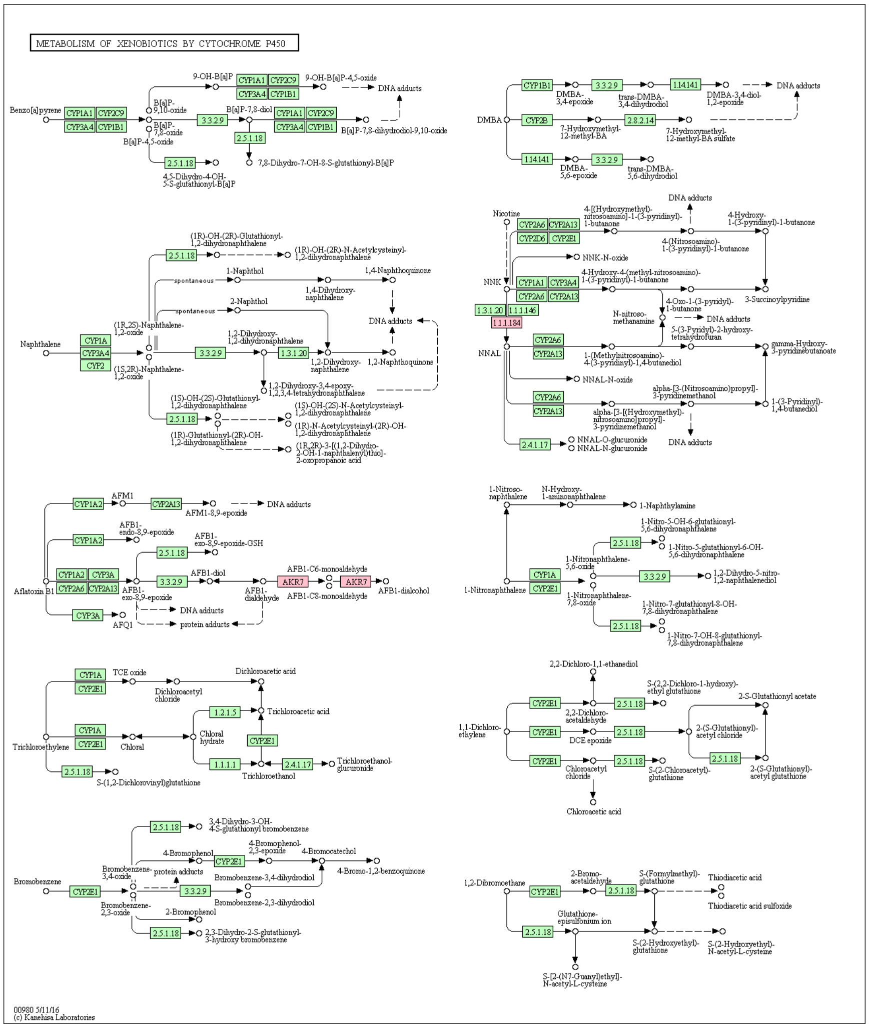

225.3 Legend:

RBH-Blast at 60% Identity + 50% Coverage
Green = Hit in *H. sapiens*
Red = Hit in *H. sapiens* and *T. californica*
White = Not in *H. sapiens*

226 Citrate cycle (TCA cycle)

226.1 Human Pathway: HSA00020

226.2 Number of Hits: 2

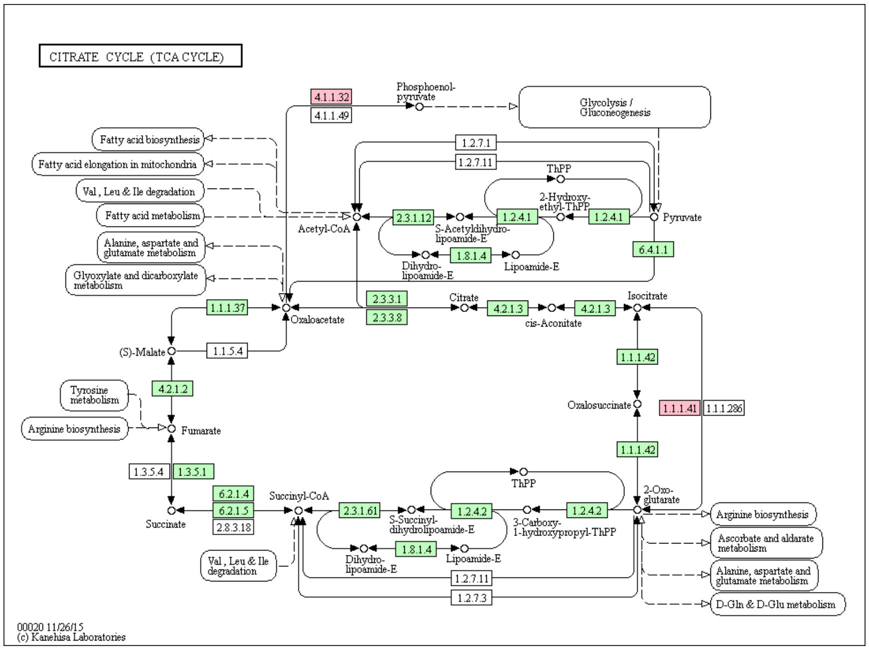

226.3 Legend:

RBH-Blast at 60% Identity + 50% Coverage
Green = Hit in *H. sapiens*
Red = Hit in *H. sapiens* and *T. californica*
White = Not in *H. sapiens*

227 Protein export

227.1 Human Pathway: HSA03060

227.2 Number of Hits: 2

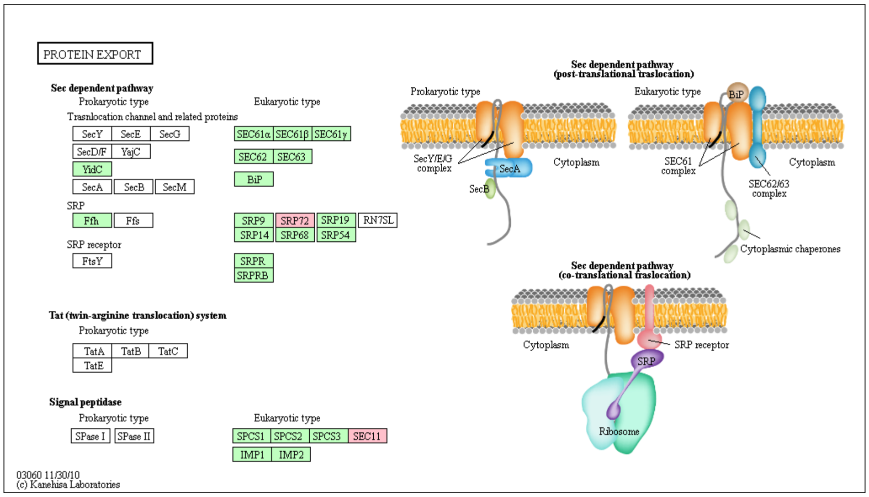

227.3 Legend:

RBH-Blast at 60% Identity + 50% Coverage
Green = Hit in *H. sapiens*
Red = Hit in *H. sapiens* and *T. californica*
White = Not in *H. sapiens*

228 Biosynthesis of unsaturated fatty acids

228.1 Human Pathway: HSA01040

228.2 Number of Hits: 2

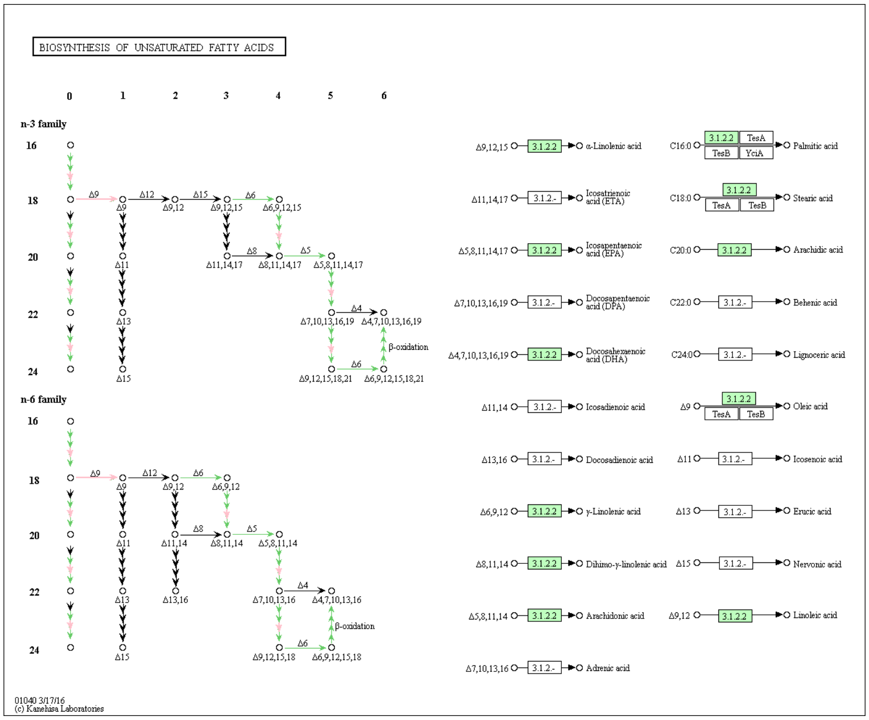

228.3 Legend:

RBH-Blast at 60% Identity + 50% Coverage
Green = Hit in *H. sapiens*
Red = Hit in *H. sapiens* and *T. californica*
White = Not in *H. sapiens*

229 Other types of O-glycan biosynthesis

229.1 Human Pathway: HSA00514

229.2 Number of Hits: 2

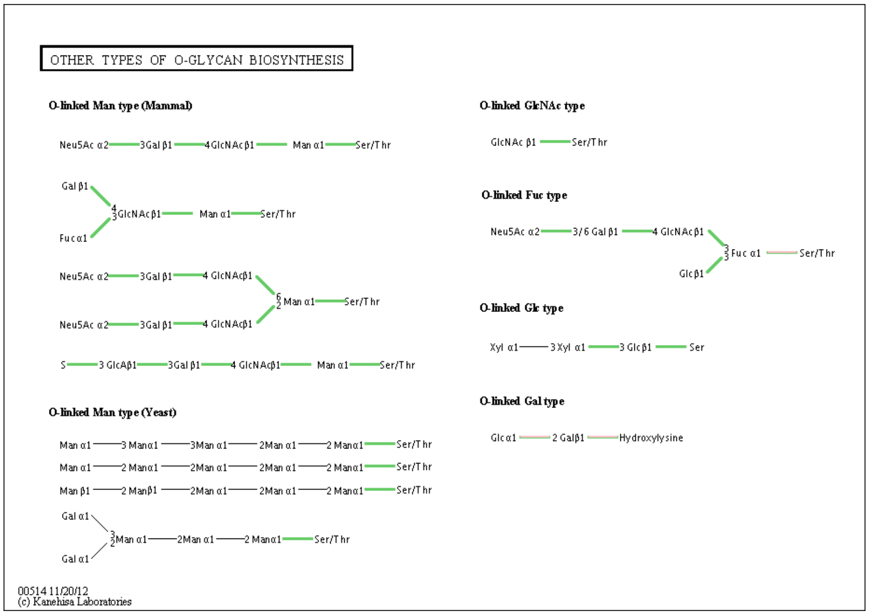

229.3 Legend:

RBH-Blast at 60% Identity + 50% Coverage
Green = Hit in *H. sapiens*
Red = Hit in *H. sapiens* and *T. californica*
White = Not in *H. sapiens*

230 NOD-like receptor signaling pathway

230.1 Human Pathway: HSA04621

230.2 Number of Hits: 2

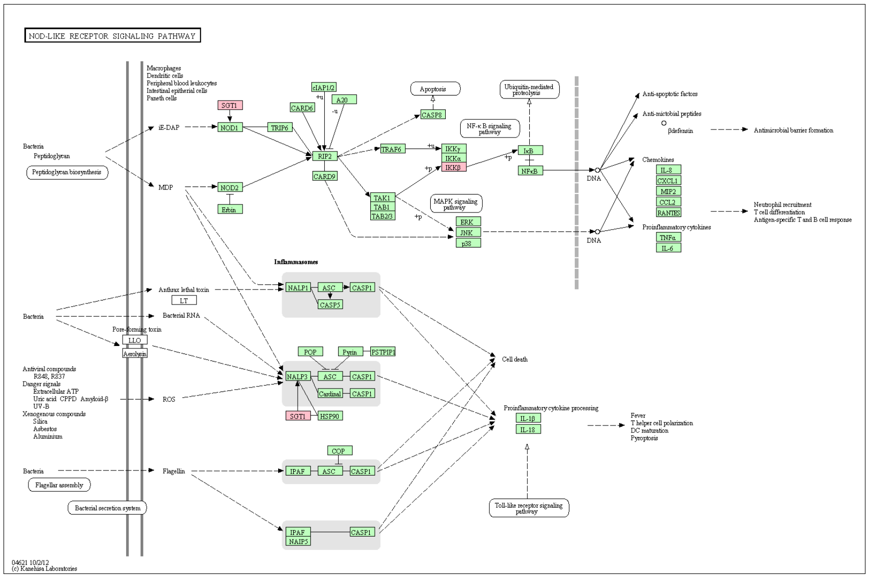

230.3 Legend:

RBH-Blast at 60% Identity + 50% Coverage
Green = Hit in *H. sapiens*
Red = Hit in *H. sapiens* and *T. californica*
White = Not in *H. sapiens*

231 One carbon pool by folate

231.1 Human Pathway: HSA00670

231.2 Number of Hits: 2

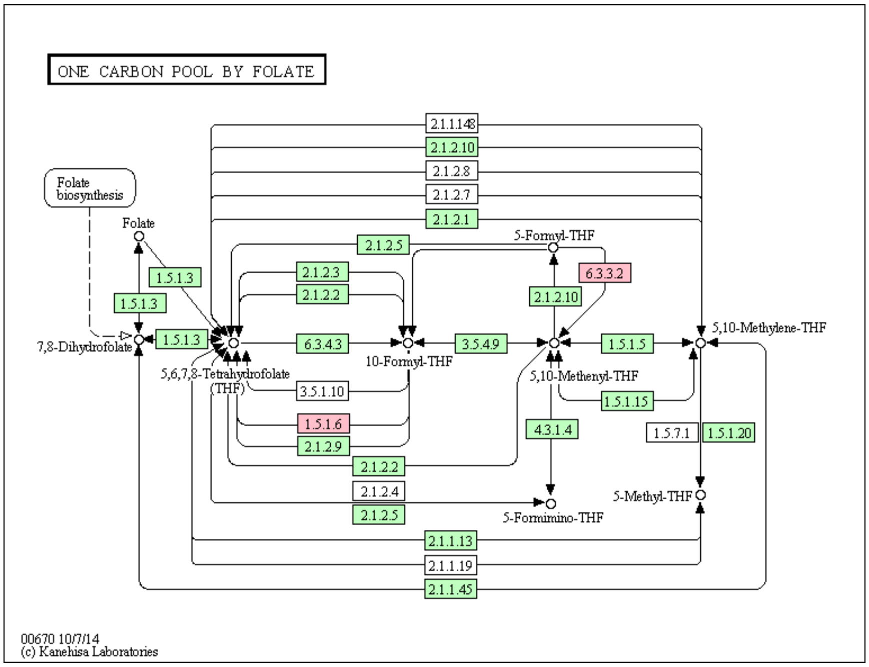

231.3 Legend:

RBH-Blast at 60% Identity + 50% Coverage
Green = Hit in *H. sapiens*
Red = Hit in *H. sapiens* and *T. californica*
White = Not in *H. sapiens*

232 Mismatch repair

232.1 Human Pathway: HSA03430

232.2 Number of Hits: 2

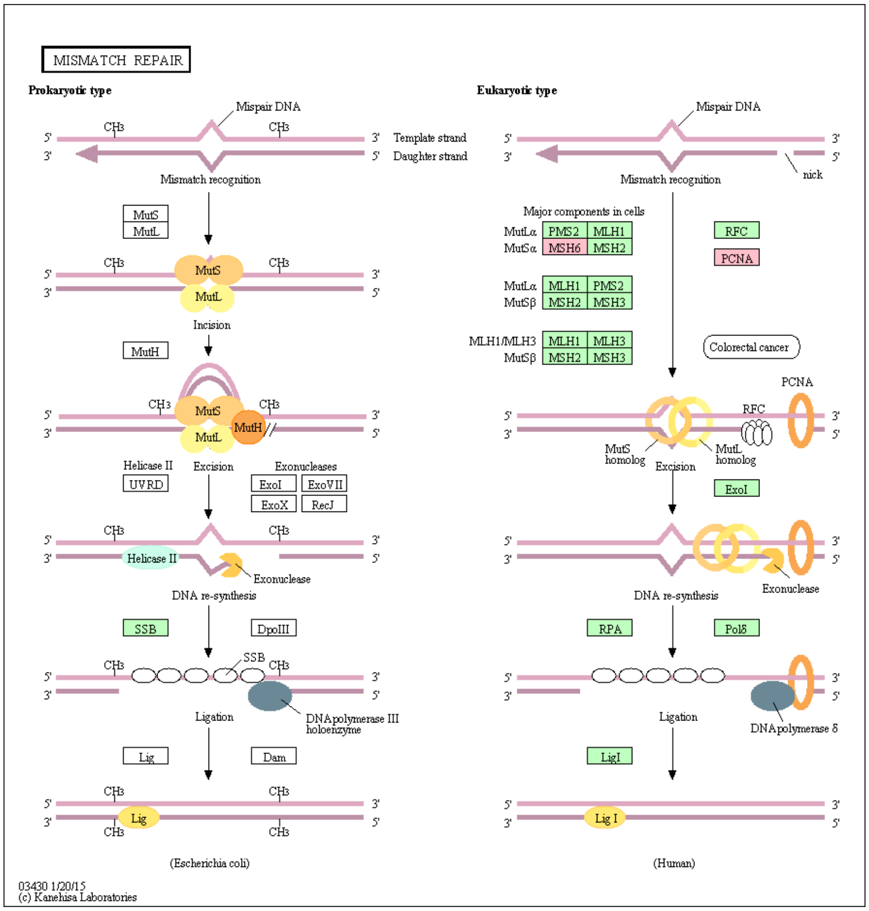

232.3 Legend:

RBH-Blast at 60% Identity + 50% Coverage
Green = Hit in *H. sapiens*
Red = Hit in *H. sapiens* and *T. californica*
White = Not in *H. sapiens*

233 Pantothenate and CoA biosynthesis

233.1 Human Pathway: HSA00770

233.2 Number of Hits: 2

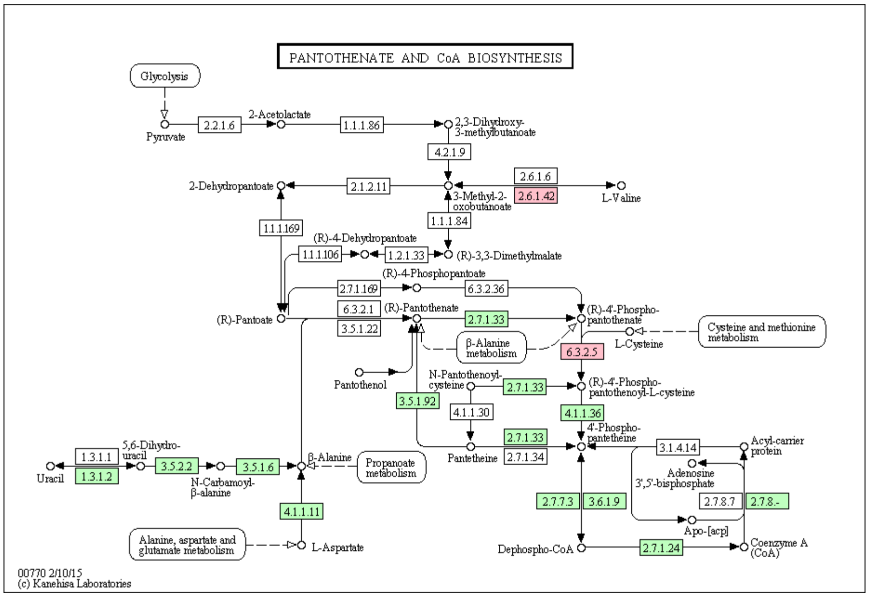

233.3 Legend:

RBH-Blast at 60% Identity + 50% Coverage
Green = Hit in *H. sapiens*
Red = Hit in *H. sapiens* and *T. californica*
White = Not in *H. sapiens*

234 Rheumatoid arthritis

234.1 Human Pathway: HSA05323

234.2 Number of Hits: 2

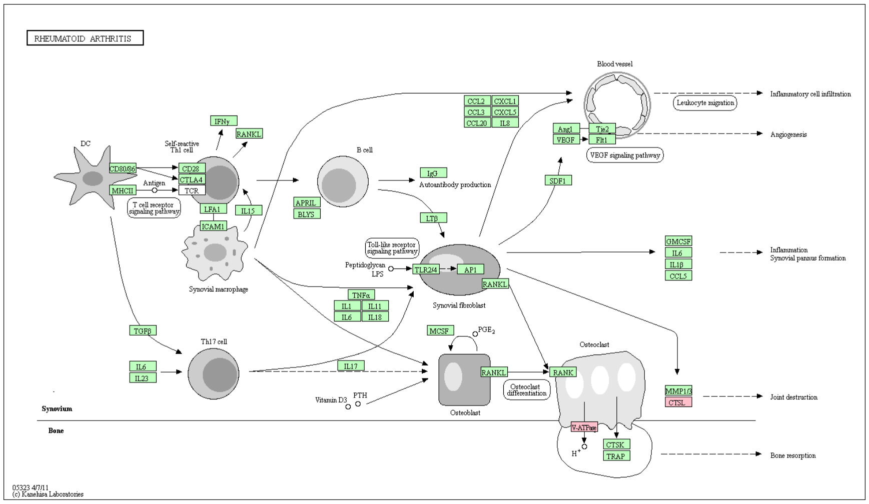

234.3 Legend:

RBH-Blast at 60% Identity + 50% Coverage
Green = Hit in *H. sapiens*
Red = Hit in *H. sapiens* and *T. californica*
White = Not in *H. sapiens*

235 Malaria

235.1 Human Pathway: HSA05144

235.2 Number of Hits: 2

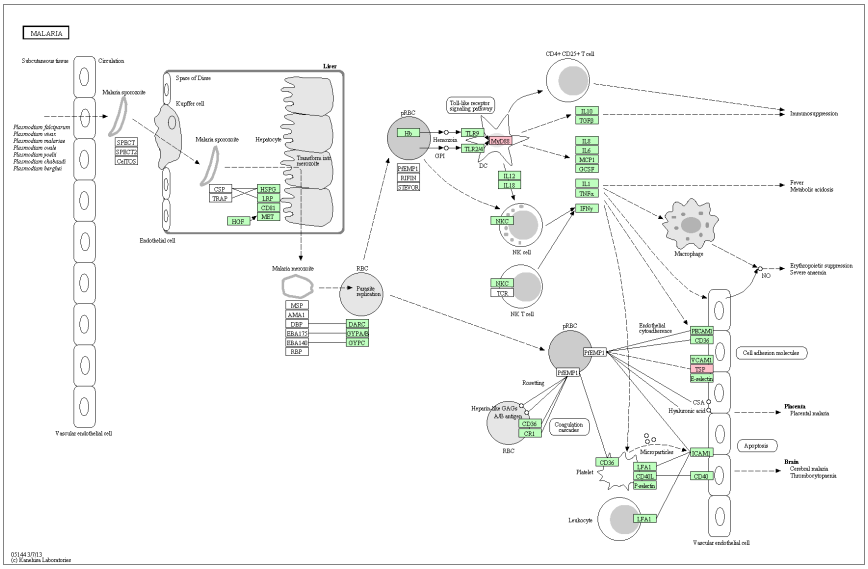

235.3 Legend:

RBH-Blast at 60% Identity + 50% Coverage
Green = Hit in *H. sapiens*
Red = Hit in *H. sapiens* and *T. californica*
White = Not in *H. sapiens*

236 Histidine metabolism

236.1 Human Pathway: HSA00340

236.2 Number of Hits: 2

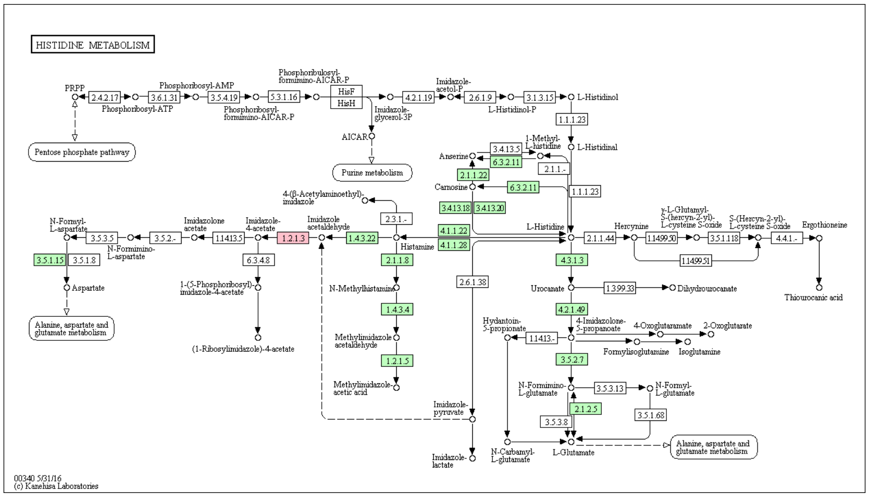

236.3 Legend:

RBH-Blast at 60% Identity + 50% Coverage
Green = Hit in *H. sapiens*
Red = Hit in *H. sapiens* and *T. californica*
White = Not in *H. sapiens*

237 Glycosaminoglycan biosynthesis - chondroitin sulfate / dermatan sulfate

237.1 Human Pathway: HSA00532

237.2 Number of Hits: 2

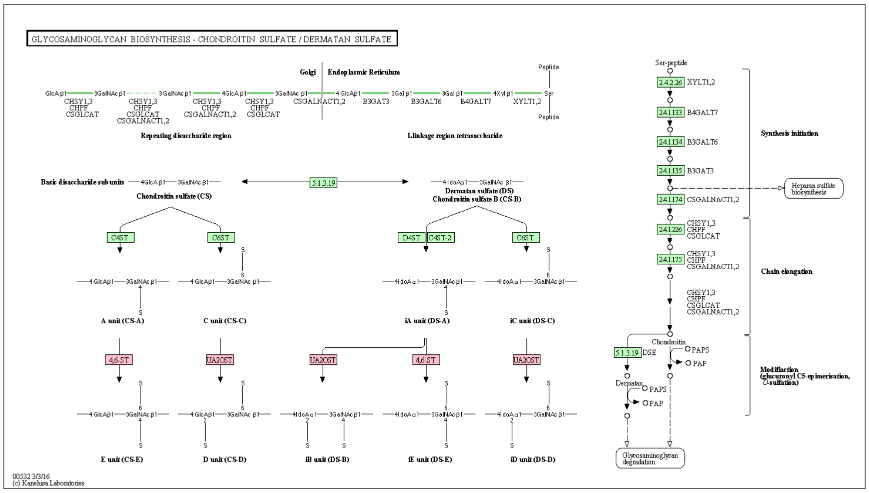

237.3 Legend:

RBH-Blast at 60% Identity + 50% Coverage
Green = Hit in *H. sapiens*
Red = Hit in *H. sapiens* and *T. californica*
White = Not in *H. sapiens*

238 Glycosaminoglycan biosynthesis - keratan sulfate

238.1 Human Pathway: HSA00533

238.2 Number of Hits: 2

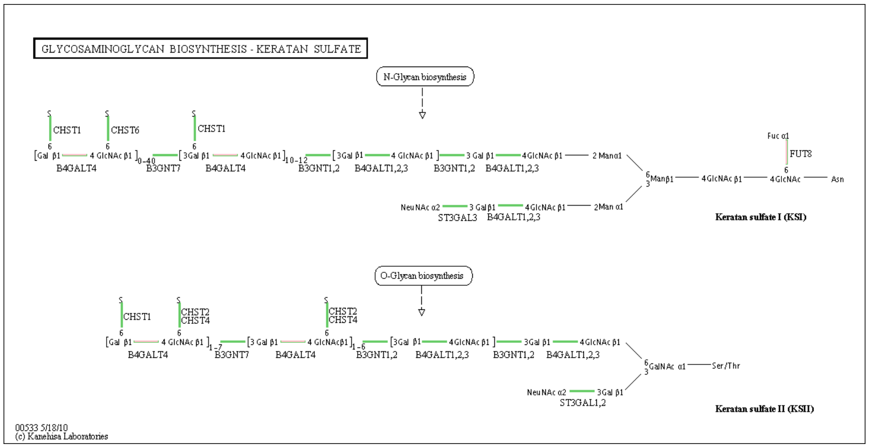

238.3 Legend:

RBH-Blast at 60% Identity + 50% Coverage
Green = Hit in *H. sapiens*
Red = Hit in *H. sapiens* and *T. californica*
White = Not in *H. sapiens*

239 Vibrio cholerae infection

239.1 Human Pathway: HSA05110

239.2 Number of Hits: 2

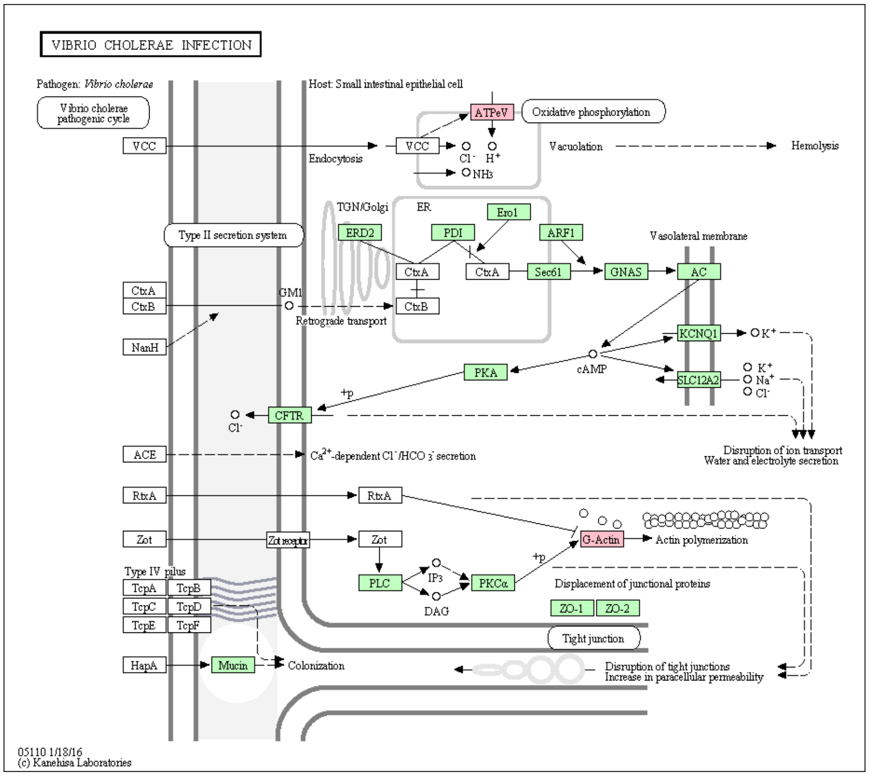

239.3 Legend:

RBH-Blast at 60% Identity + 50% Coverage
Green = Hit in *H. sapiens*
Red = Hit in *H. sapiens* and *T. californica*
White = Not in *H. sapiens*

240 Ether lipid metabolism

240.1 Human Pathway: HSA00565

240.2 Number of Hits: 2

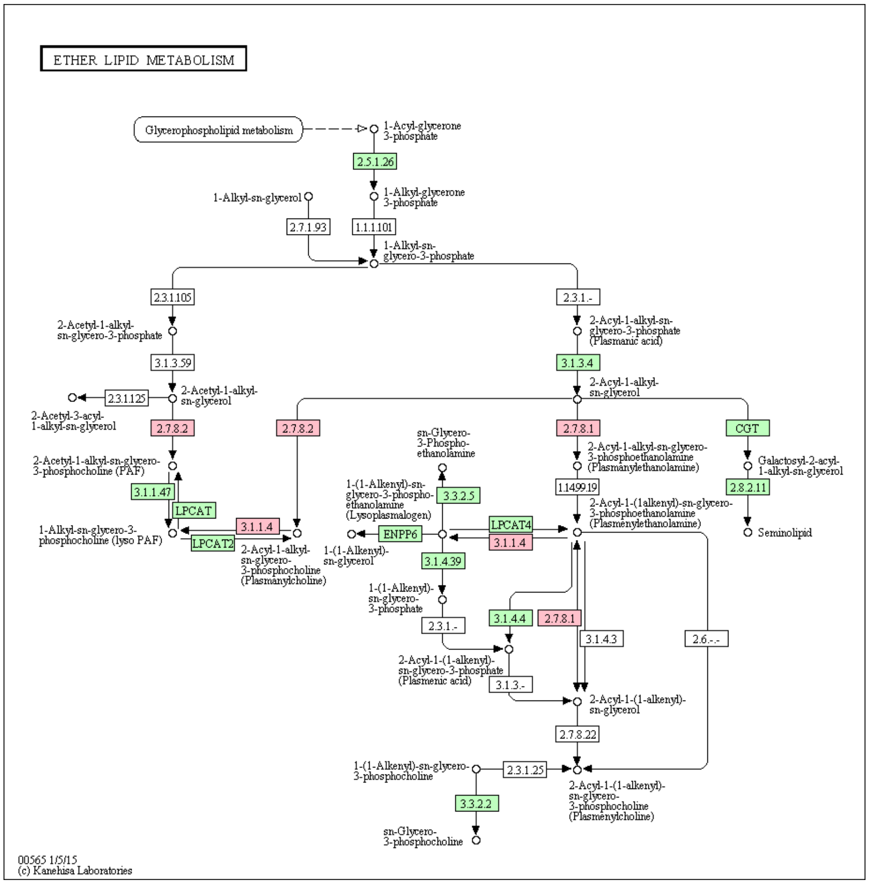

240.3 Legend:

RBH-Blast at 60% Identity + 50% Coverage
Green = Hit in *H. sapiens*
Red = Hit in *H. sapiens* and *T. californica*
White = Not in *H. sapiens*

241 African trypanosomiasis

241.1 Human Pathway: HSA05143

241.2 Number of Hits: 2

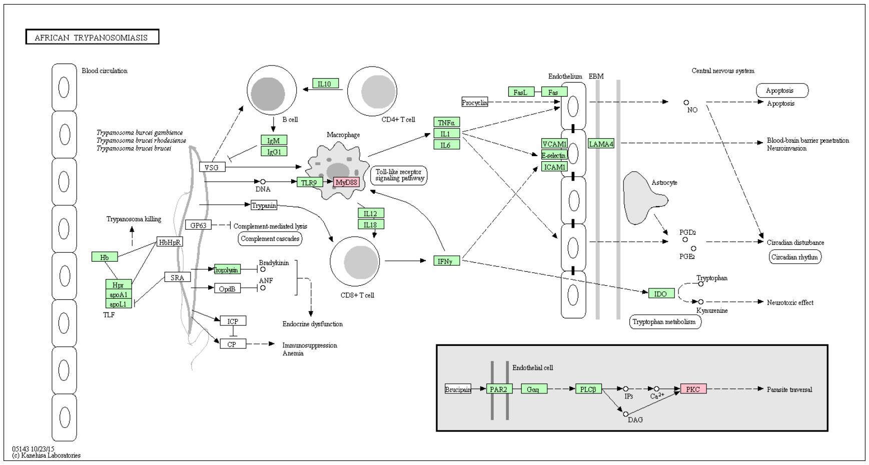

241.3 Legend:

RBH-Blast at 60% Identity + 50% Coverage
Green = Hit in *H. sapiens*
Red = Hit in *H. sapiens* and *T. californica*
White = Not in *H. sapiens*

242 Fanconi anemia pathway

242.1 Human Pathway: HSA03460

242.2 Number of Hits: 2

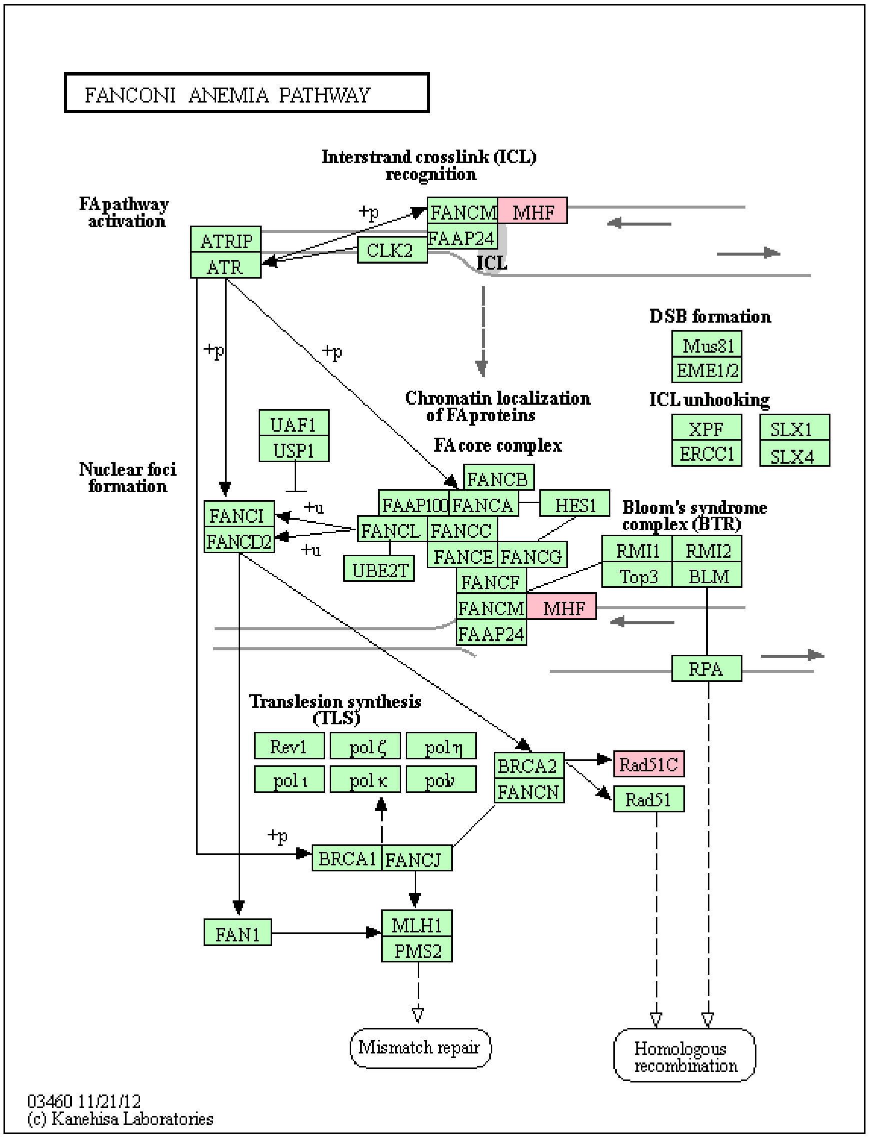

242.3 Legend:

RBH-Blast at 60% Identity + 50% Coverage
Green = Hit in *H. sapiens*
Red = Hit in *H. sapiens* and *T. californica*
White = Not in *H. sapiens*

---

243 Thyroid cancer

243.1 Human Pathway: HSA05216

243.2 Number of Hits: 2

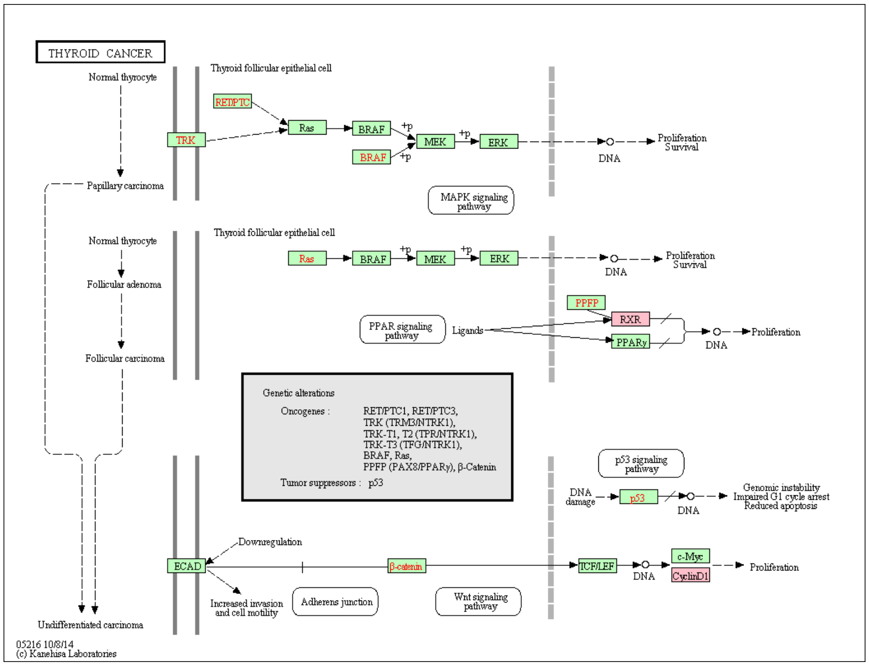

243.3 Legend:

RBH-Blast at 60% Identity + 50% Coverage
Green = Hit in *H. sapiens*
Red = Hit in *H. sapiens* and *T. californica*
White = Not in *H. sapiens*

---

244 Valine, leucine and isoleucine biosynthesis

244.1 Human Pathway: HSA00290

244.2 Number of Hits: 1

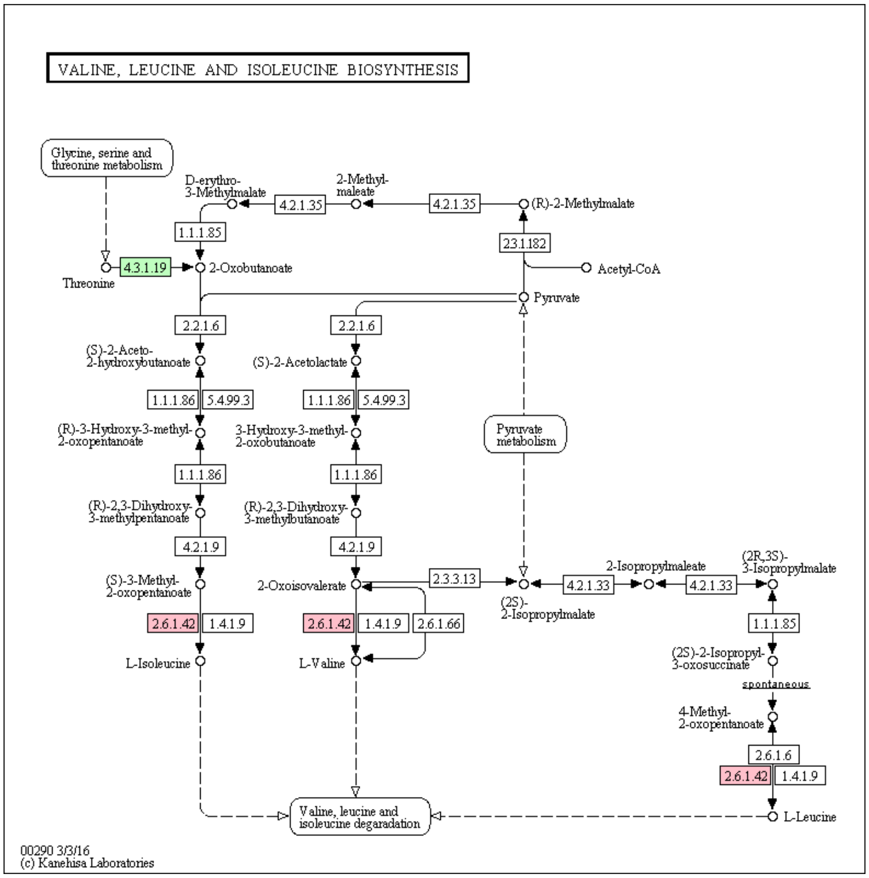

244.3 Legend:

RBH-Blast at 60% Identity + 50% Coverage
Green = Hit in *H. sapiens*
Red = Hit in *H. sapiens* and *T. californica*
White = Not in *H. sapiens*

245 Collecting duct acid secretion

245.1 Human Pathway: HSA04966

245.2 Number of Hits: 1

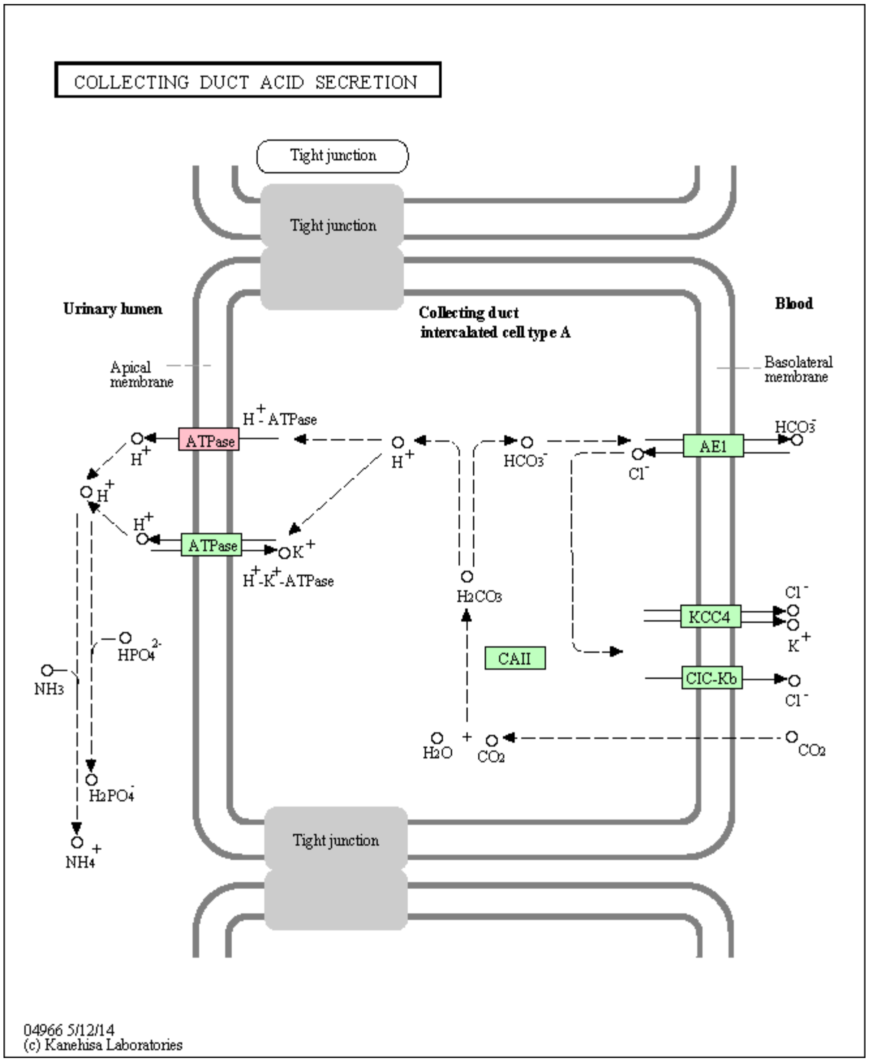

245.3 Legend:

RBH-Blast at 60% Identity + 50% Coverage
Green = Hit in *H. sapiens*
Red = Hit in *H. sapiens* and *T. californica*
White = Not in *H. sapiens*

246 Linoleic acid metabolism

246.1 Human Pathway: HSA00591

246.2 Number of Hits: 1

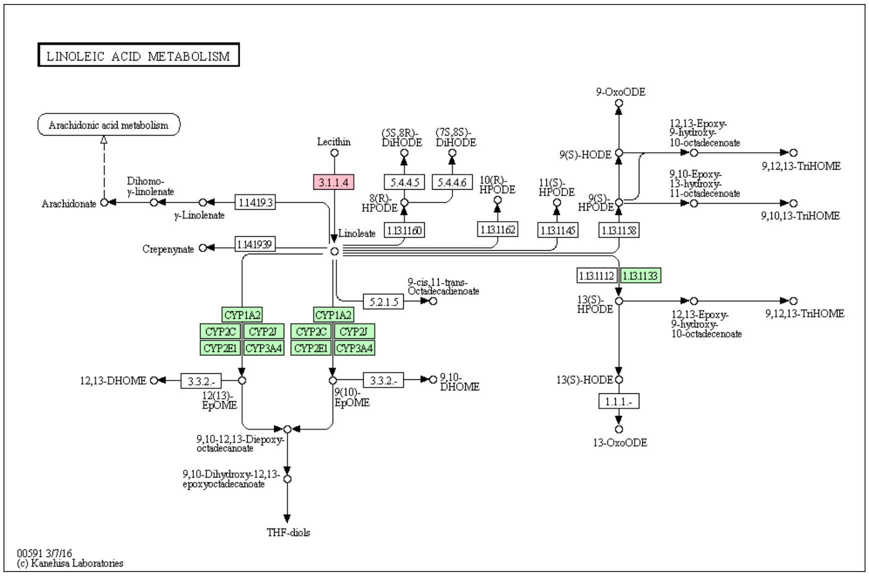

246.3 Legend:

RBH-Blast at 60% Identity + 50% Coverage
Green = Hit in *H. sapiens*
Red = Hit in *H. sapiens* and *T. californica*
White = Not in *H. sapiens*

247 Pertussis

247.1 Human Pathway: HSA05133

247.2 Number of Hits: 1

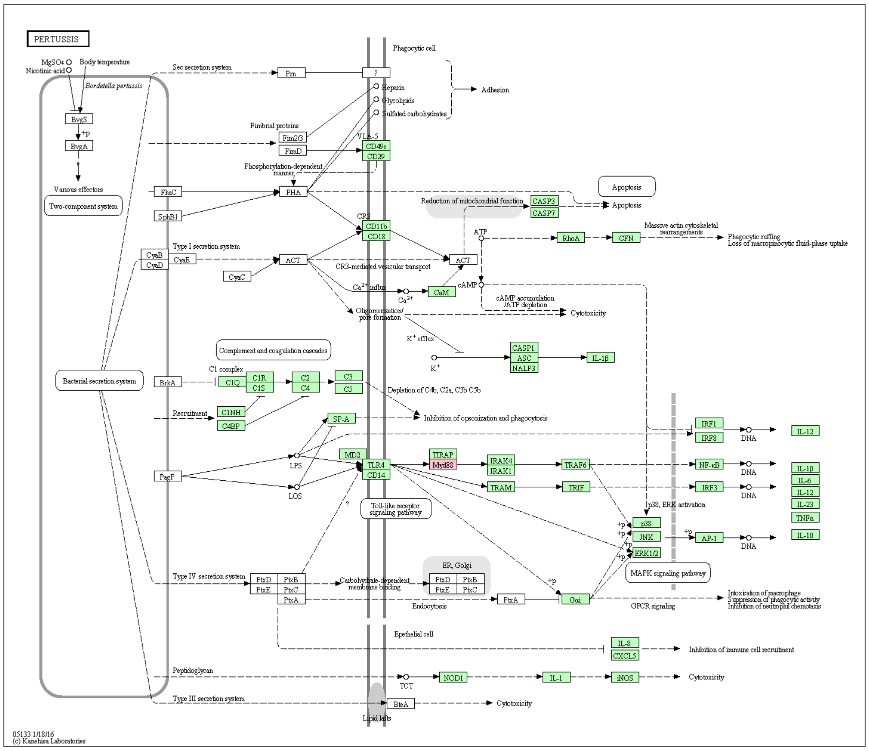

247.3 Legend:

RBH-Blast at 60% Identity + 50% Coverage
Green = Hit in *H. sapiens*
Red = Hit in *H. sapiens* and *T. californica*
White = Not in *H. sapiens*

248 Glycosphingolipid biosynthesis - ganglio series

248.1 Human Pathway: HSA00604

248.2 Number of Hits: 1

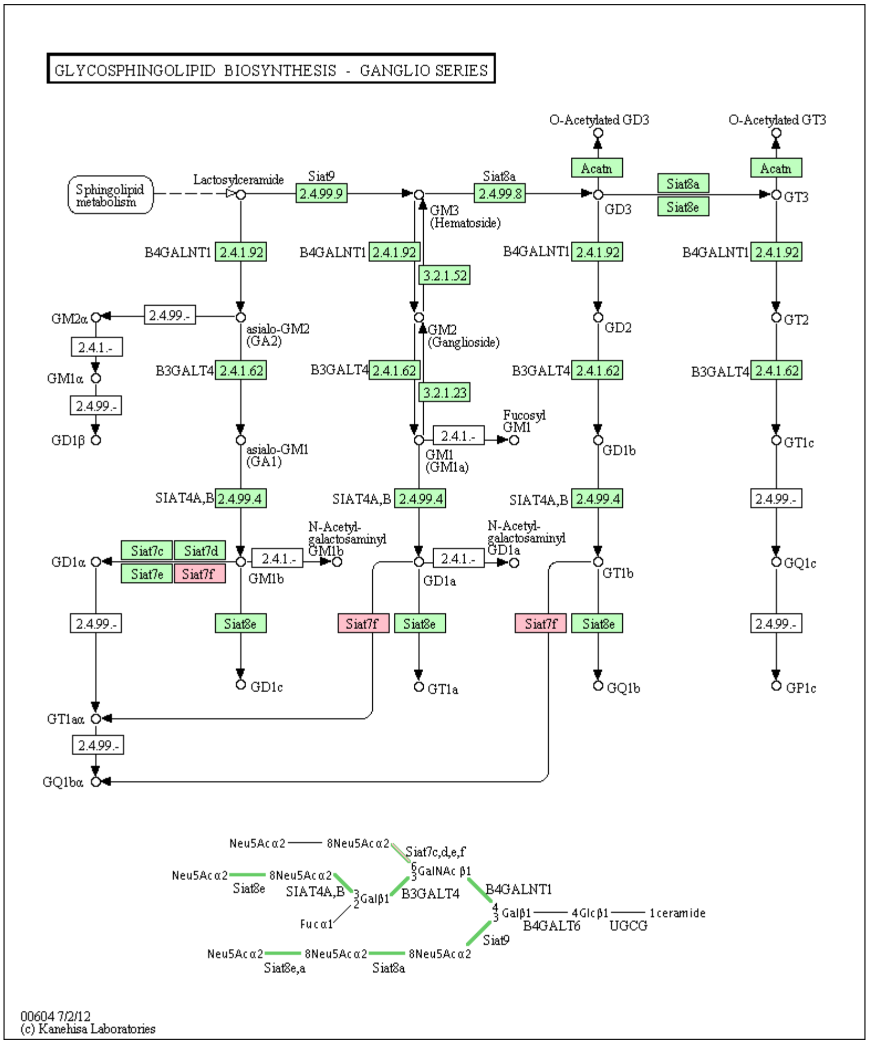

248.3 Legend:

RBH-Blast at 60% Identity + 50% Coverage
Green = Hit in *H. sapiens*
Red = Hit in *H. sapiens* and *T. californica*
White = Not in *H. sapiens*

---

249 Retinol metabolism

249.1 Human Pathway: HSA00830

249.2 Number of Hits: 1

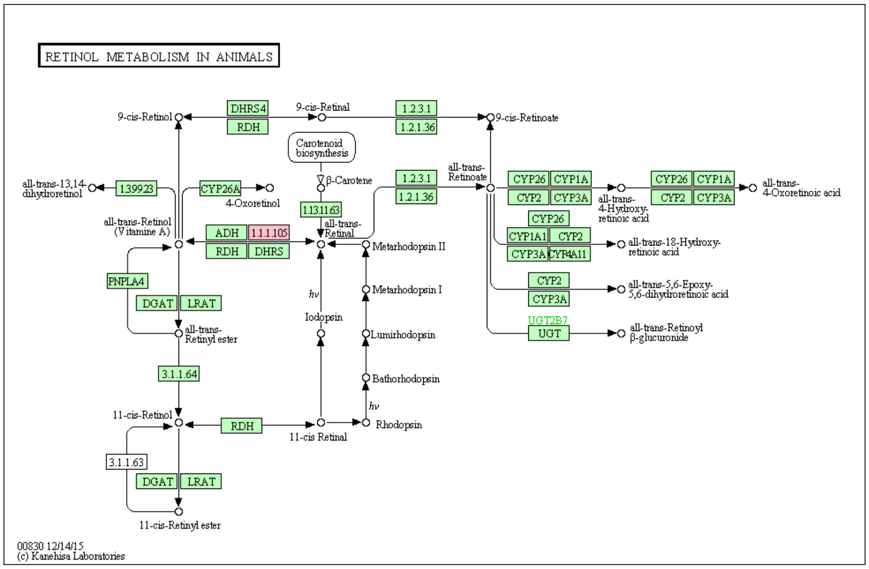

249.3 Legend:

RBH-Blast at 60% Identity + 50% Coverage
Green = Hit in *H. sapiens*
Red = Hit in *H. sapiens* and *T. californica*
White = Not in *H. sapiens*

---

250 Primary immunodeficiency

250.1 Human Pathway: HSA05340

250.2 Number of Hits: 1

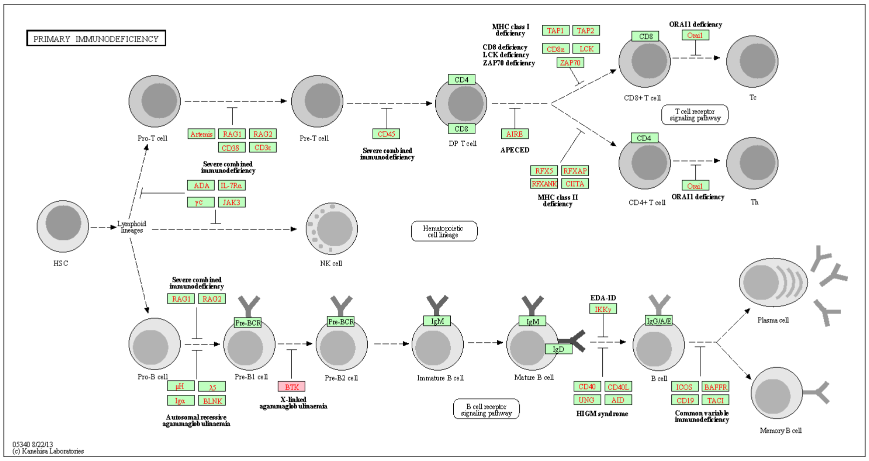

250.3 Legend:

RBH-Blast at 60% Identity + 50% Coverage
Green = Hit in *H. sapiens*
Red = Hit in *H. sapiens* and *T. californica*
White = Not in *H. sapiens*

251 Glycosylphosphatidylinositol(GPI)-anchor biosynthesis

251.1 Human Pathway: HSA00563

251.2 Number of Hits: 1

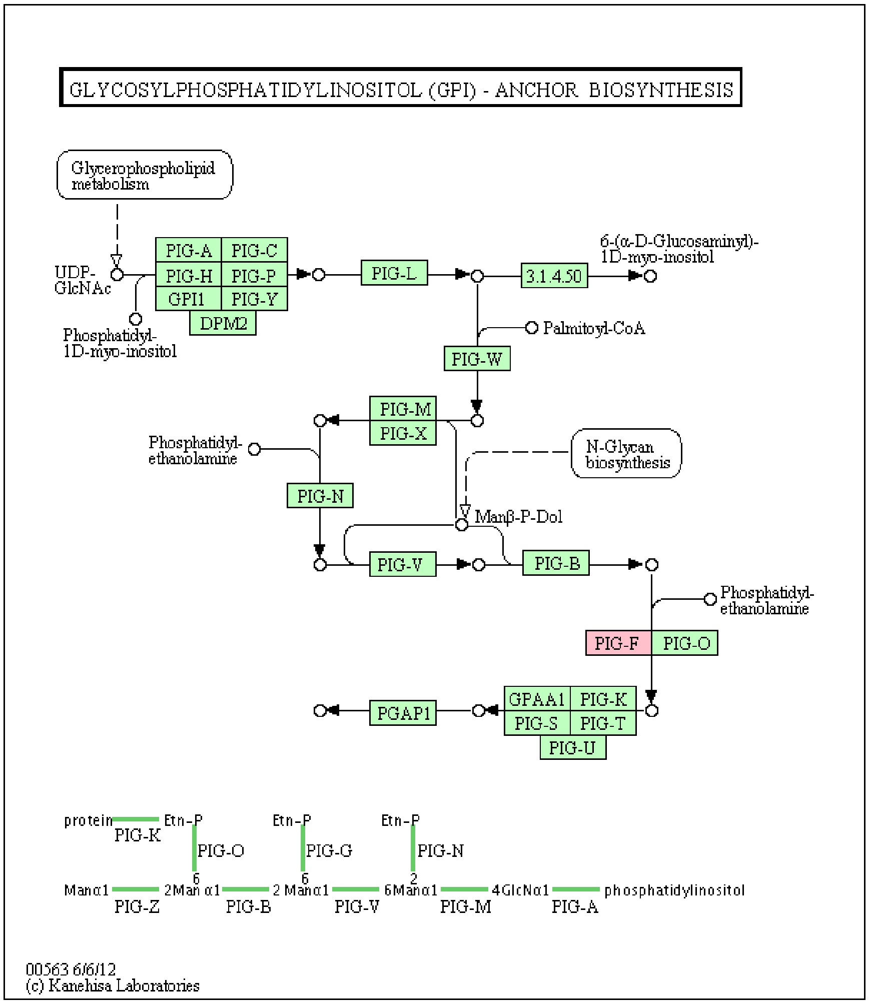

251.3 Legend:

RBH-Blast at 60% Identity + 50% Coverage
Green = Hit in *H. sapiens*
Red = Hit in *H. sapiens* and *T. californica*
White = Not in *H. sapiens*

252 Maturity onset diabetes of the young

252.1 Human Pathway: HSA04950

252.2 Number of Hits: 1

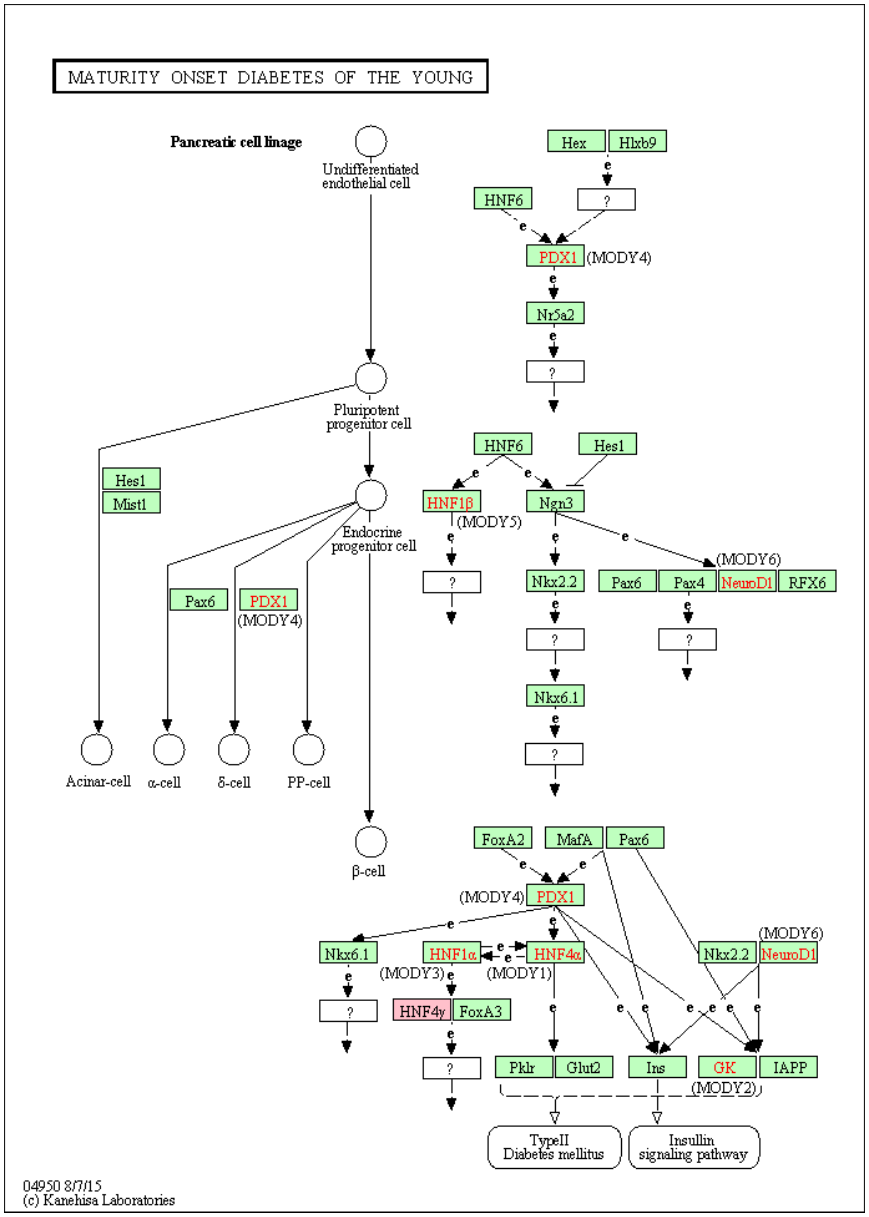

252.3 Legend:

RBH-Blast at 60% Identity + 50% Coverage
Green = Hit in *H. sapiens*
Red = Hit in *H. sapiens* and *T. californica*
White = Not in *H. sapiens*

253 Non-homologous end-joining

253.1 Human Pathway: HSA03450

253.2 Number of Hits: 1

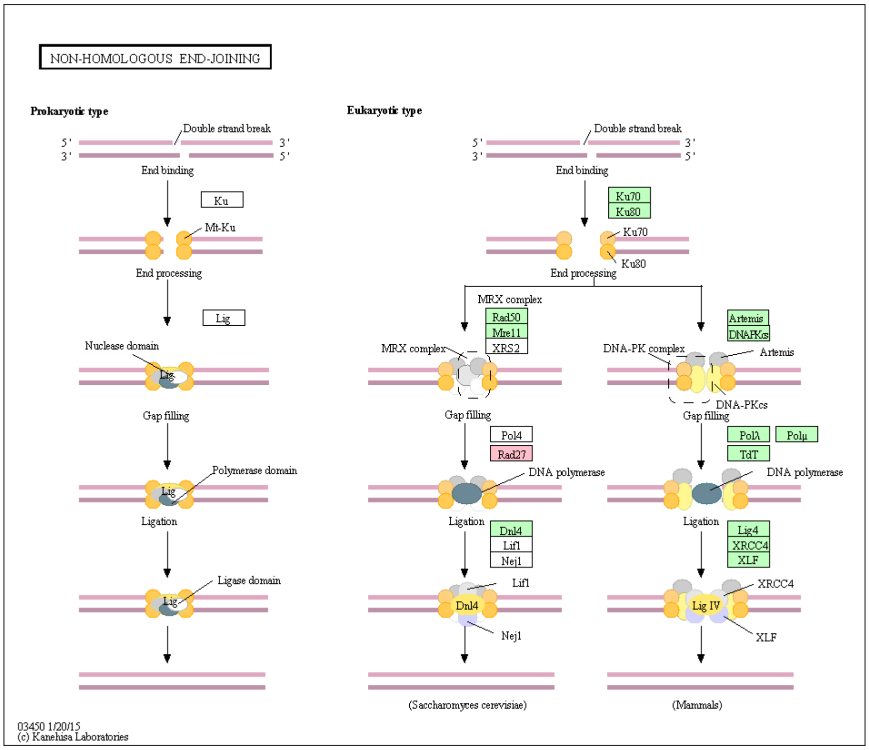

253.3 Legend:

RBH-Blast at 60% Identity + 50% Coverage
Green = Hit in *H. sapiens*
Red = Hit in *H. sapiens* and *T. californica*
White = Not in *H. sapiens*

254 Taste transduction

254.1 Human Pathway: HSA04742

254.2 Number of Hits: 1

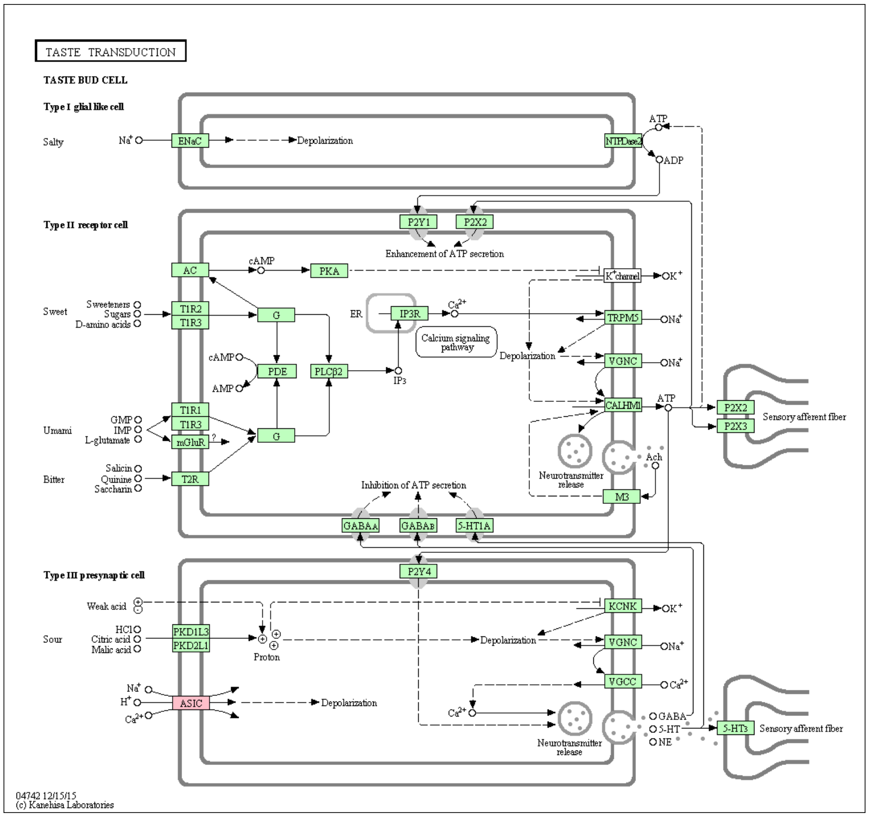

254.3 Legend:

RBH-Blast at 60% Identity + 50% Coverage
Green = Hit in *H. sapiens*
Red = Hit in *H. sapiens* and *T. californica*
White = Not in *H. sapiens*

255 Sulfur metabolism

255.1 Human Pathway: HSA00920

255.2 Number of Hits: 1

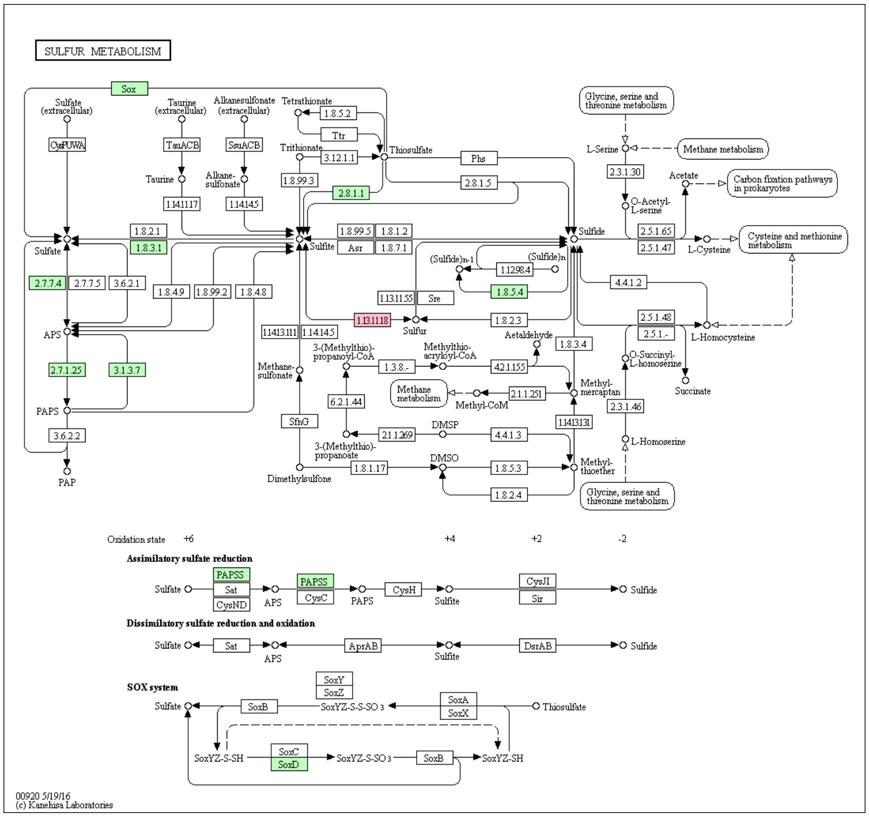

255.3 Legend:

RBH-Blast at 60% Identity + 50% Coverage
Green = Hit in *H. sapiens*
Red = Hit in *H. sapiens* and *T. californica*
White = Not in *H. sapiens*

256 Chemical carcinogenesis

256.1 Human Pathway: HSA05204

256.2 Number of Hits: 1

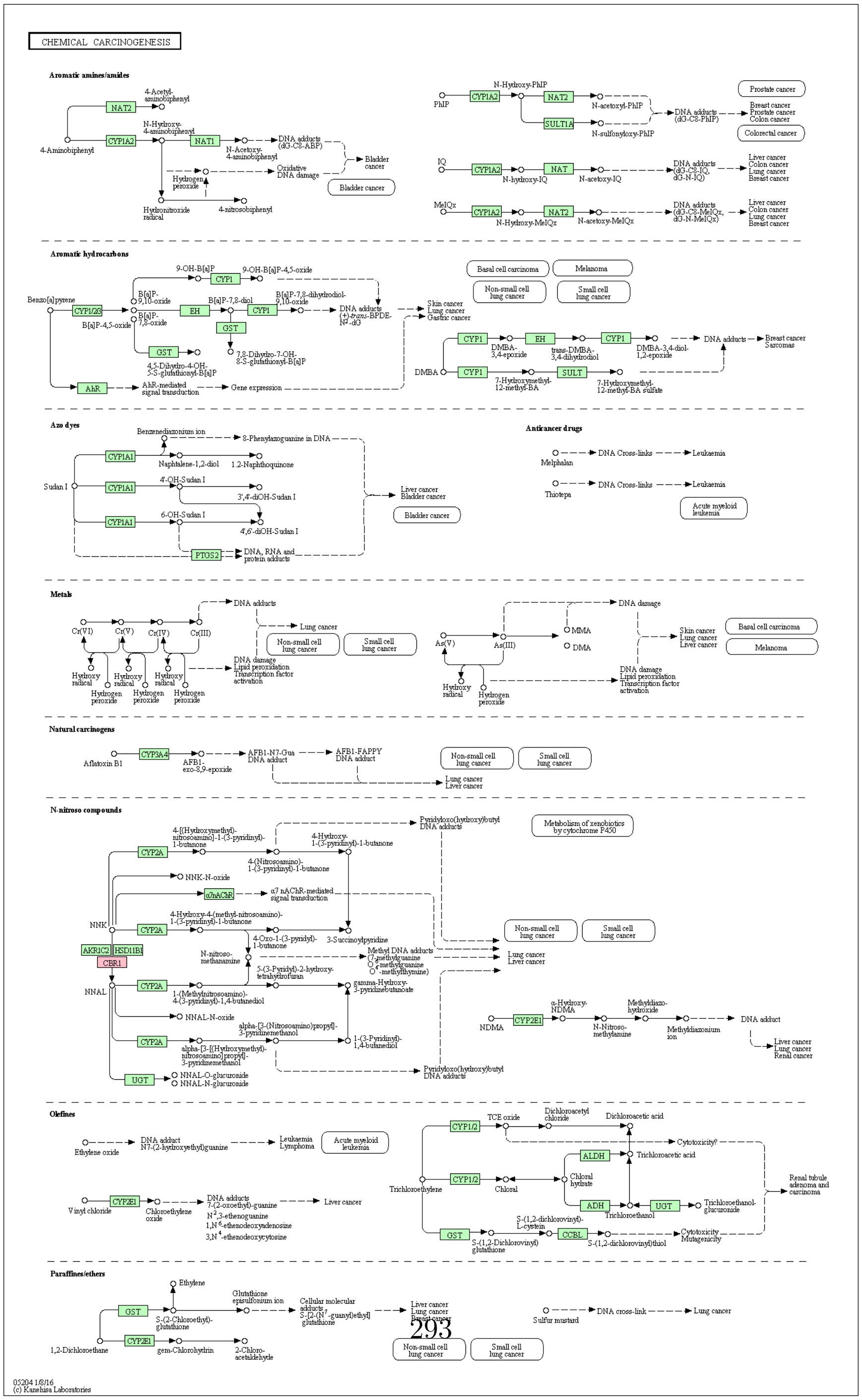

256.3 Legend:

RBH-Blast at 60% Identity + 50% Coverage
Green = Hit in *H. sapiens*
Red = Hit in *H. sapiens* and *T. californica*
White = Not in *H. sapiens*

---

257 Dorso-ventral axis formation

257.1 Human Pathway: HSA04320

257.2 Number of Hits: 1

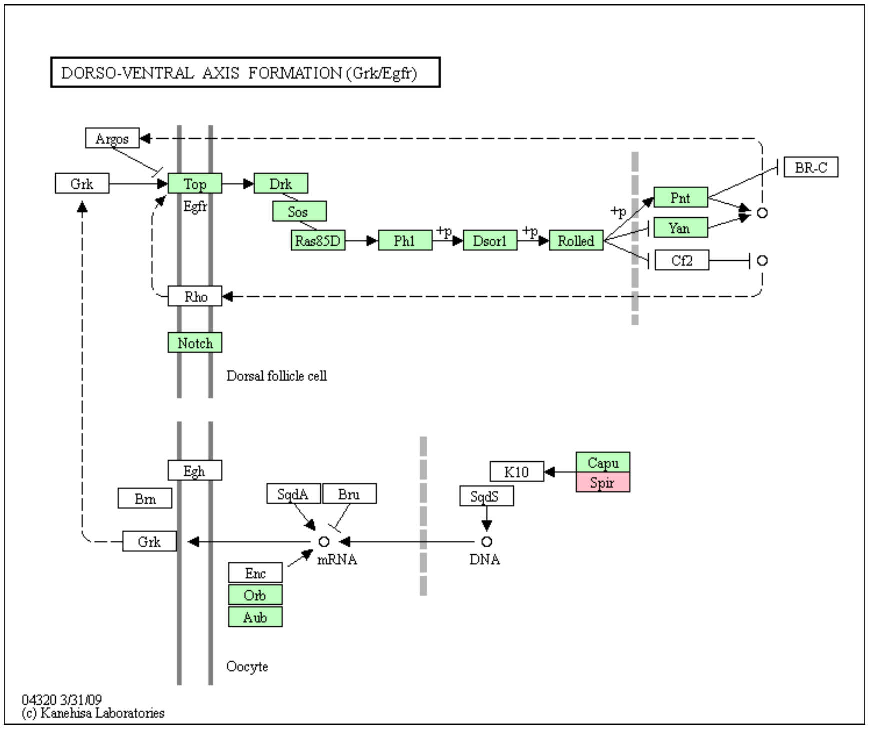

257.3 Legend:

RBH-Blast at 60% Identity + 50% Coverage
Green = Hit in *H. sapiens*
Red = Hit in *H. sapiens* and *T. californica*
White = Not in *H. sapiens*

258 Glycosaminoglycan degradation

258.1 Human Pathway: HSA00531

258.2 Number of Hits: 1

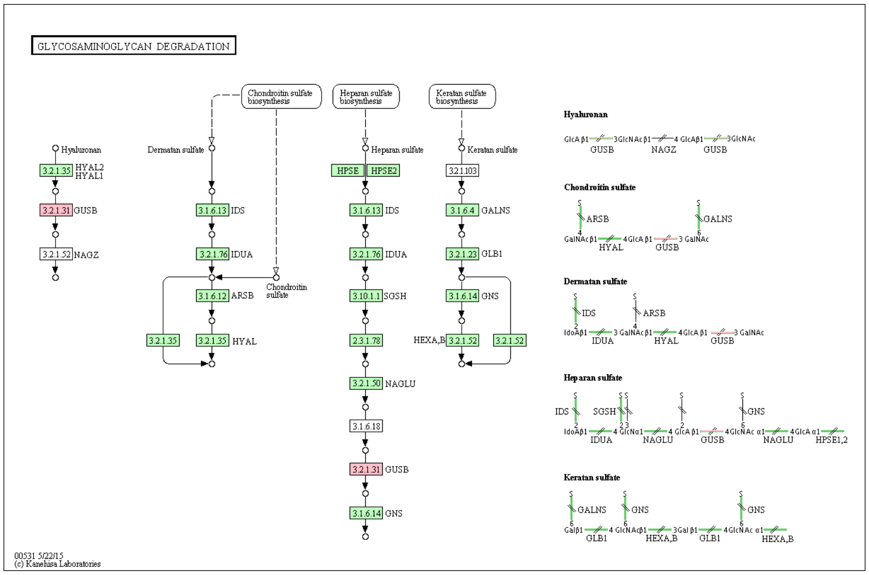

258.3 Legend:

RBH-Blast at 60% Identity + 50% Coverage
Green = Hit in *H. sapiens*
Red = Hit in *H. sapiens* and *T. californica*
White = Not in *H. sapiens*

259 Proximal tubule bicarbonate reclamation

259.1 Human Pathway: HSA04964

259.2 Number of Hits: 1

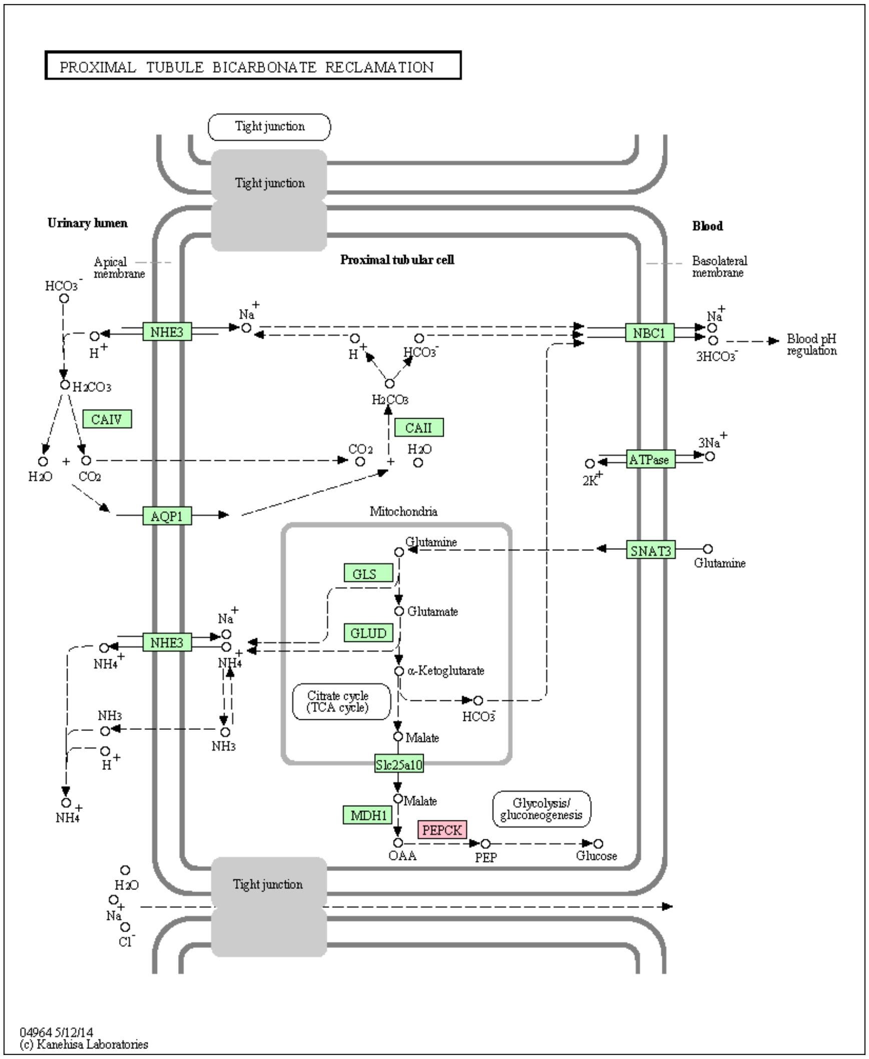

259.3 Legend:

RBH-Blast at 60% Identity + 50% Coverage
Green = Hit in *H. sapiens*
Red = Hit in *H. sapiens* and *T. californica*
White = Not in *H. sapiens*

260 Glycosphingolipid biosynthesis - lacto and neolacto series

260.1 Human Pathway: HSA00601

260.2 Number of Hits: 1

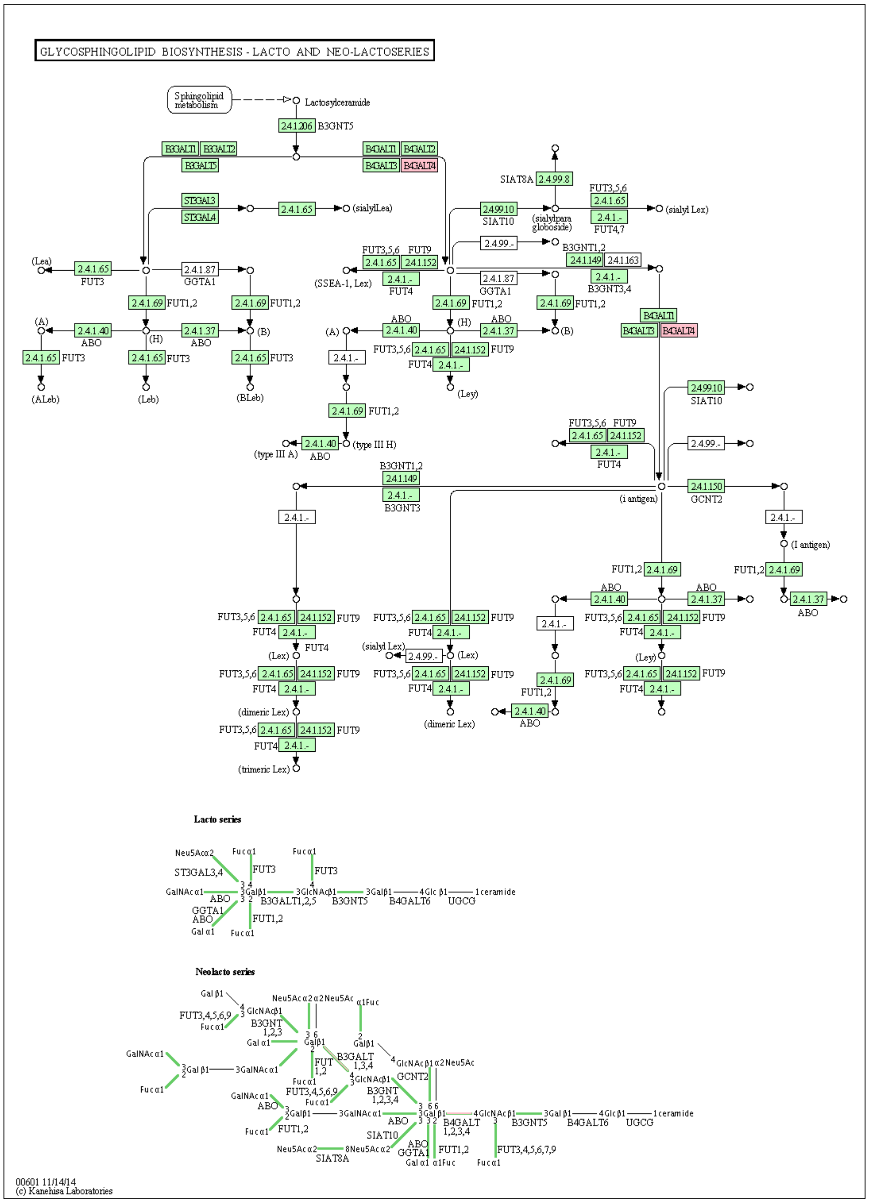

260.3 Legend:

RBH-Blast at 60% Identity + 50% Coverage
Green = Hit in *H. sapiens*
Red = Hit in *H. sapiens* and *T. californica*
White = Not in *H. sapiens*

---

261 Type I diabetes mellitus

261.1 Human Pathway: HSA04940

261.2 Number of Hits: 1

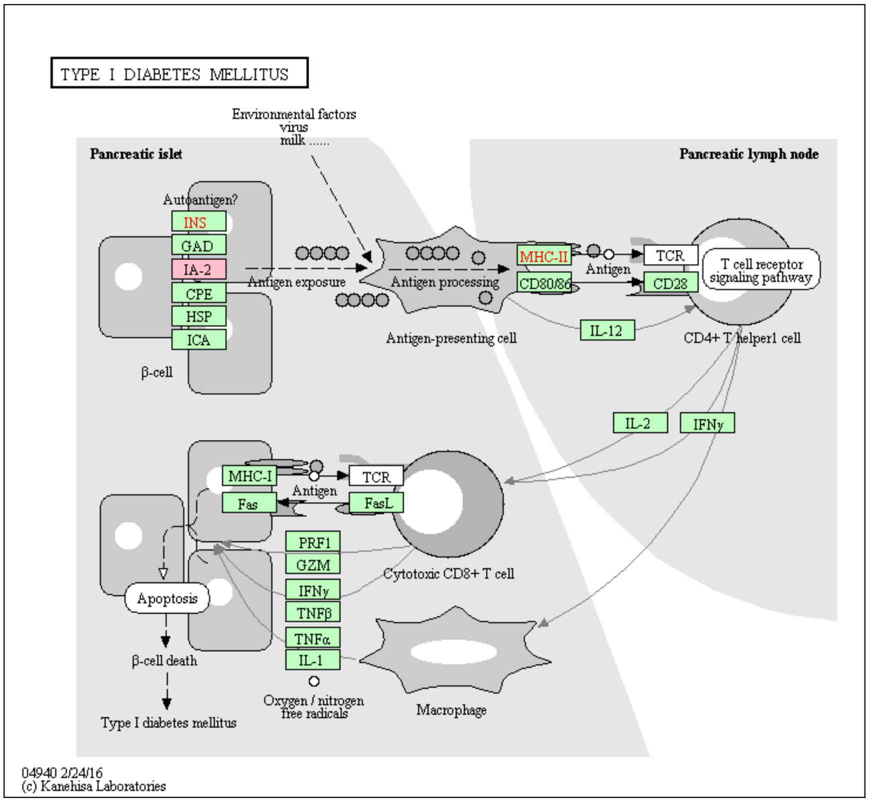

261.3 Legend:

RBH-Blast at 60% Identity + 50% Coverage
Green = Hit in *H. sapiens*
Red = Hit in *H. sapiens* and *T. californica*
White = Not in *H. sapiens*

262 Fatty acid biosynthesis

262.1 Human Pathway: HSA00061

262.2 Number of Hits: 1

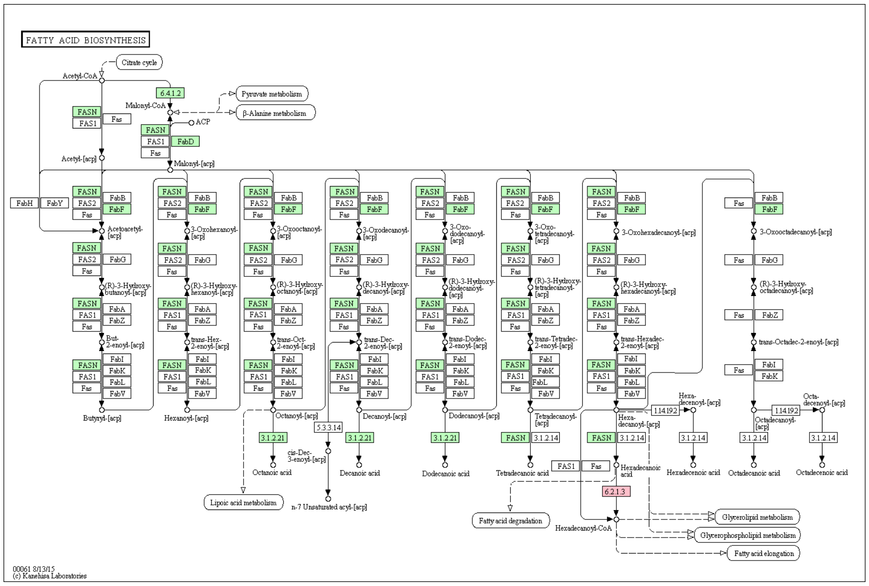

262.3 Legend:

RBH-Blast at 60% Identity + 50% Coverage
Green = Hit in *H. sapiens*
Red = Hit in *H. sapiens* and *T. californica*
White = Not in *H. sapiens*

263 Systemic lupus erythematosus

263.1 Human Pathway: HSA05322

263.2 Number of Hits: 1

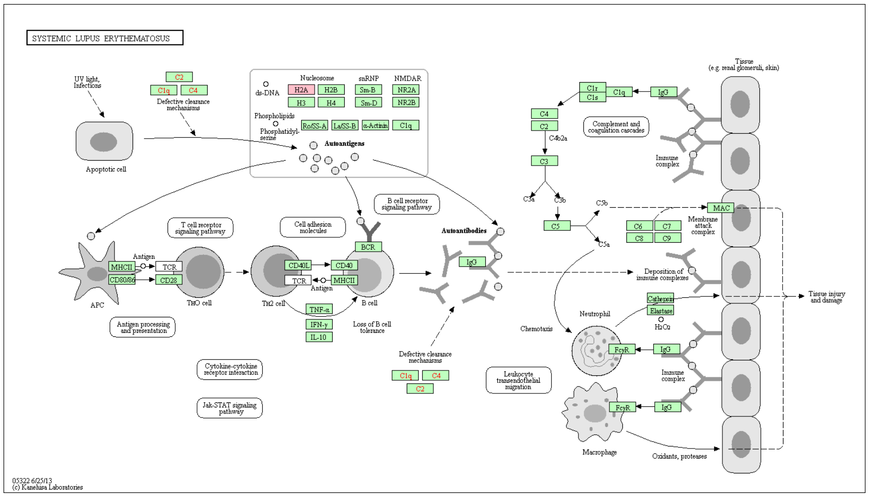

263.3 Legend:

RBH-Blast at 60% Identity + 50% Coverage
Green = Hit in *H. sapiens*
Red = Hit in *H. sapiens* and *T. californica*
White = Not in *H. sapiens*

264 Taurine and hypotaurine metabolism

264.1 Human Pathway: HSA00430

264.2 Number of Hits: 1

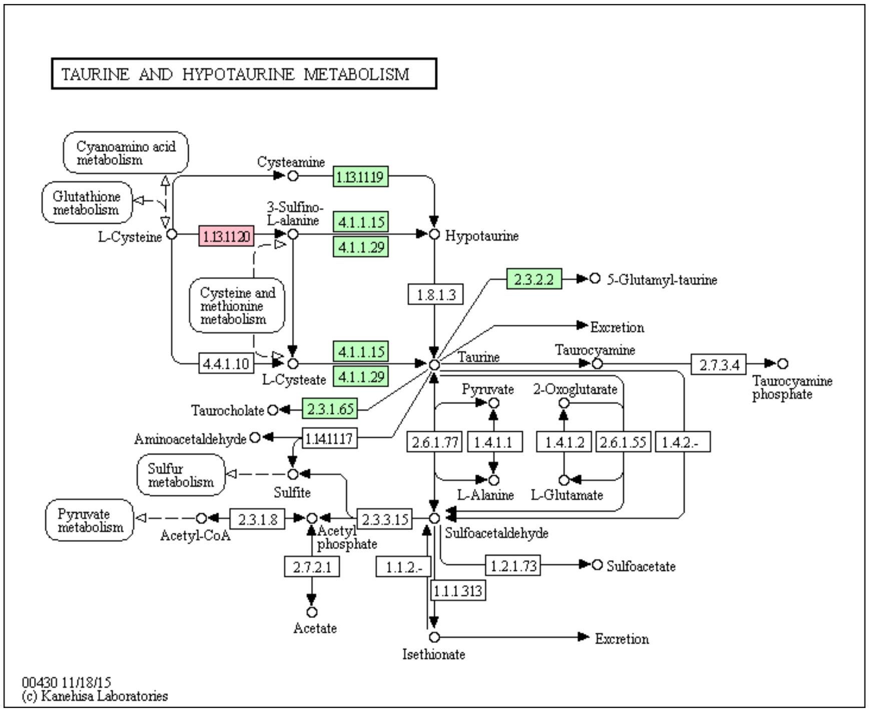

264.3 Legend:

RBH-Blast at 60% Identity + 50% Coverage
Green = Hit in *H. sapiens*
Red = Hit in *H. sapiens* and *T. californica*
White = Not in *H. sapiens*

265 Arginine biosynthesis

265.1 Human Pathway: HSA00220

265.2 Number of Hits: 1

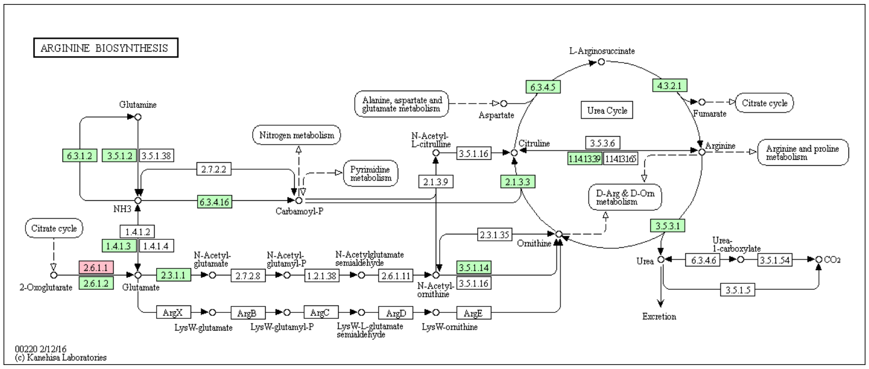

265.3 Legend:

RBH-Blast at 60% Identity + 50% Coverage
Green = Hit in *H. sapiens*
Red = Hit in *H. sapiens* and *T. californica*
White = Not in *H. sapiens*

266 Hematopoietic cell lineage

266.1 Human Pathway: HSA04640

266.2 Number of Hits: 1

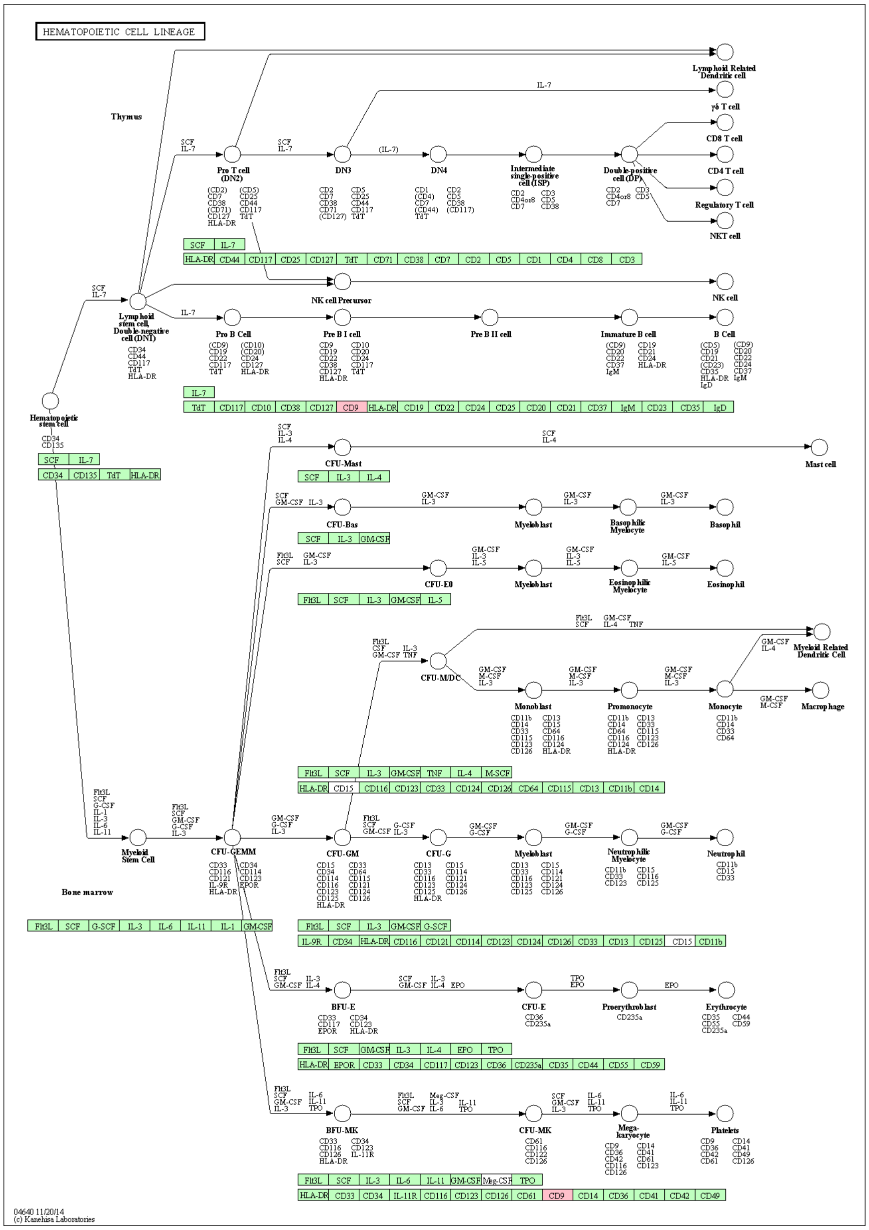

266.3 Legend:

RBH-Blast at 60% Identity + 50% Coverage
Green = Hit in *H. sapiens*
Red = Hit in *H. sapiens* and *T. californica*
White = Not in *H. sapiens*

267 Intestinal immune network for IgA production

267.1 Human Pathway: HSA04672

267.2 Number of Hits: 1

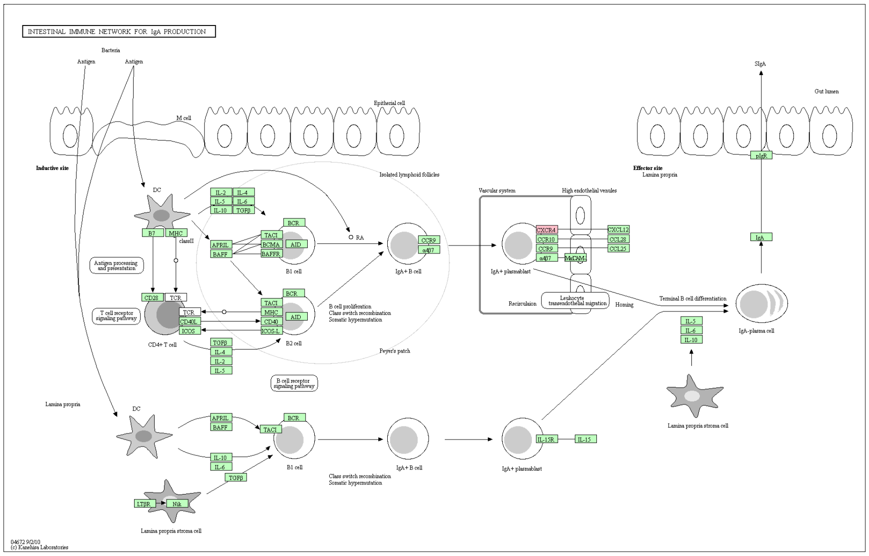

267.3 Legend:

RBH-Blast at 60% Identity + 50% Coverage
Green = Hit in *H. sapiens*
Red = Hit in *H. sapiens* and *T. californica*
White = Not in *H. sapiens*

268 Other glycan degradation

268.1 Human Pathway: HSA00511

268.2 Number of Hits: 1

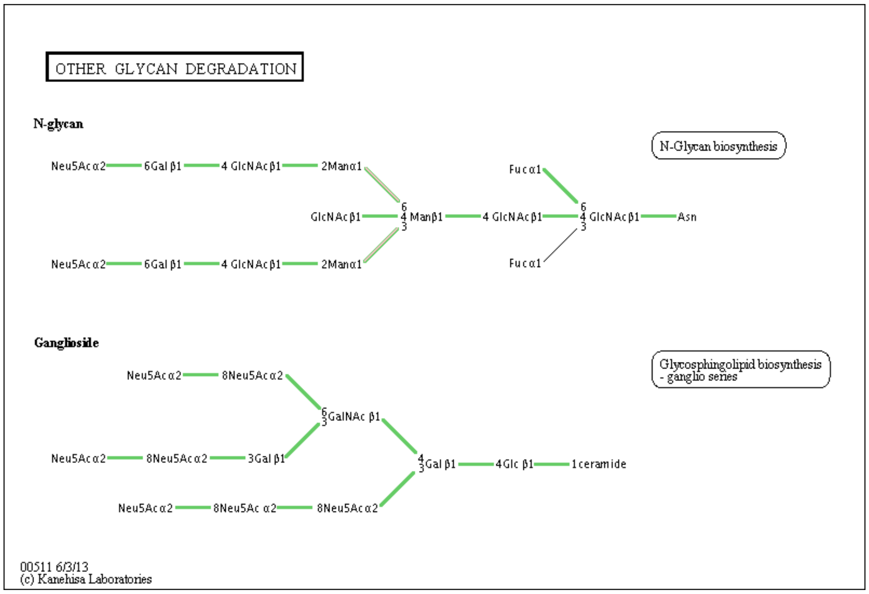

268.3 Legend:

RBH-Blast at 60% Identity + 50% Coverage
Green = Hit in *H. sapiens*
Red = Hit in *H. sapiens* and *T. californica*
White = Not in *H. sapiens*

269 Phenylalanine, tyrosine and tryptophan biosynthesis

269.1 Human Pathway: HSA00400

269.2 Number of Hits: 1

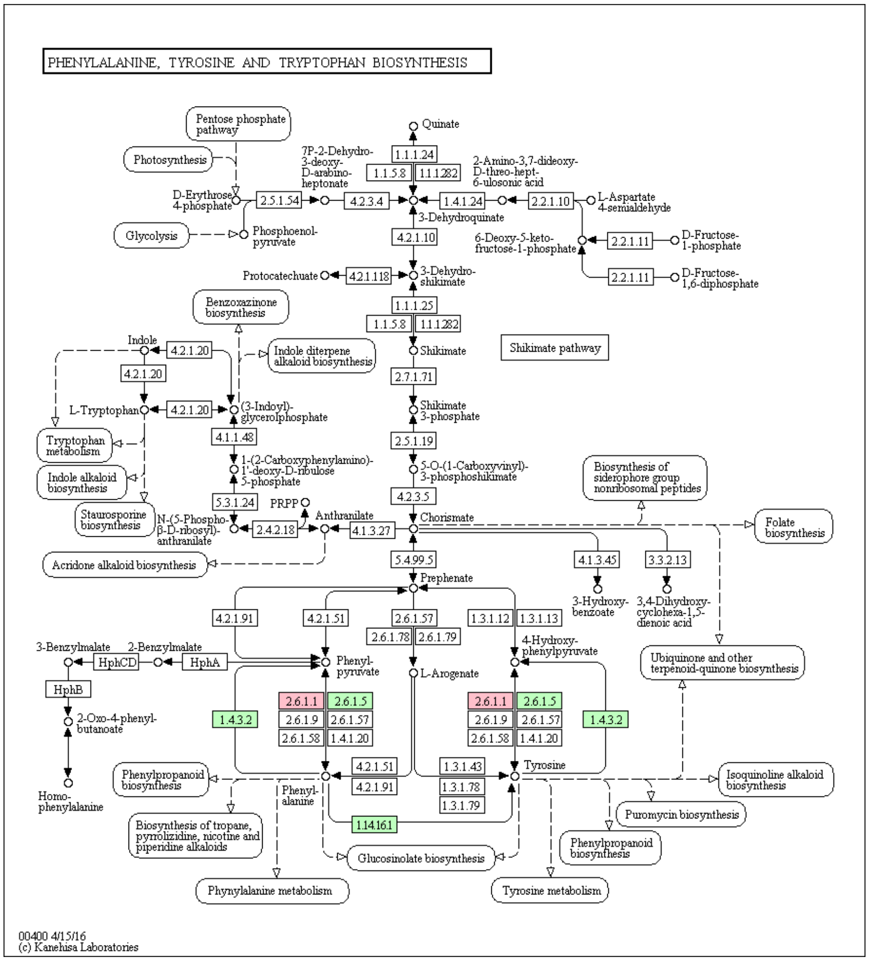

269.3 Legend:

RBH-Blast at 60% Identity + 50% Coverage
Green = Hit in *H. sapiens*
Red = Hit in *H. sapiens* and *T. californica*
White = Not in *H. sapiens*

270 Glycosphingolipid biosynthesis - globo series

270.1 Human Pathway: HSA00603

270.2 Number of Hits: 1

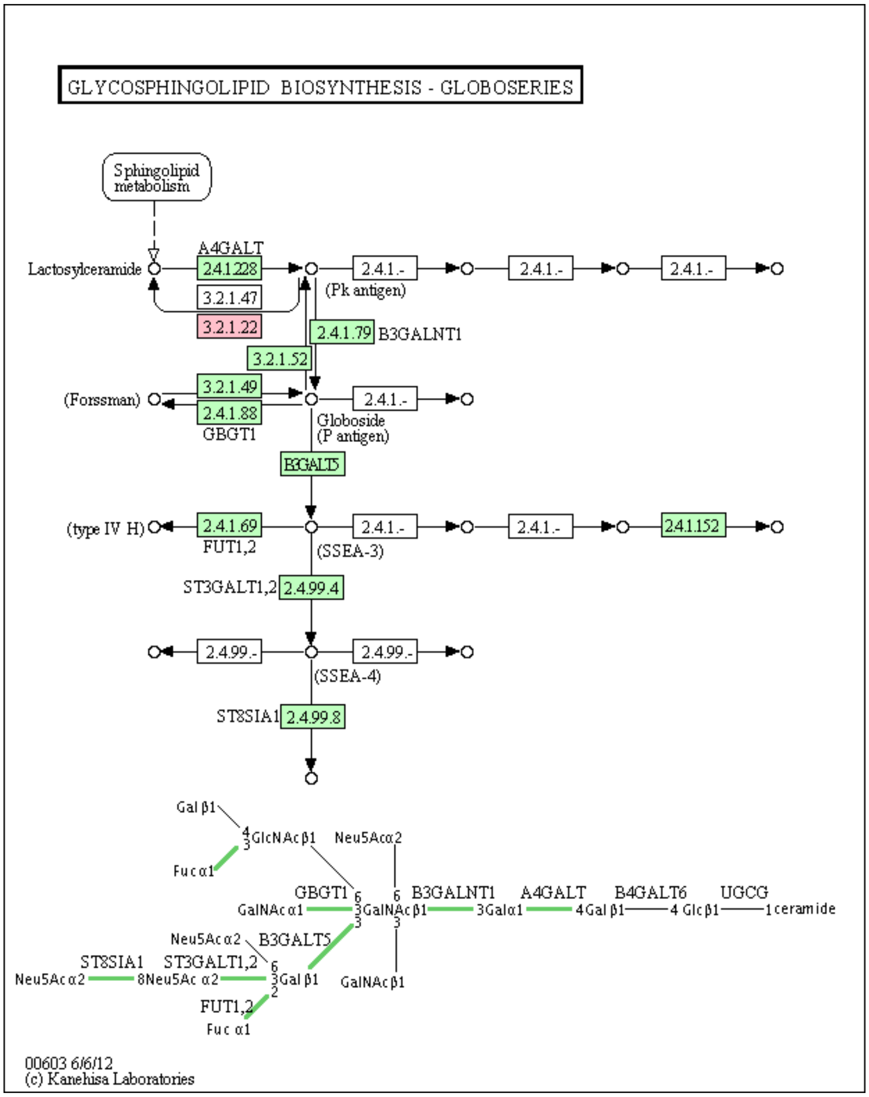

270.3 Legend:

RBH-Blast at 60% Identity + 50% Coverage
Green = Hit in *H. sapiens*
Red = Hit in *H. sapiens* and *T. californica*
White = Not in *H. sapiens*

---

271 alpha-Linolenic acid metabolism

271.1 Human Pathway: HSA00592

271.2 Number of Hits: 1

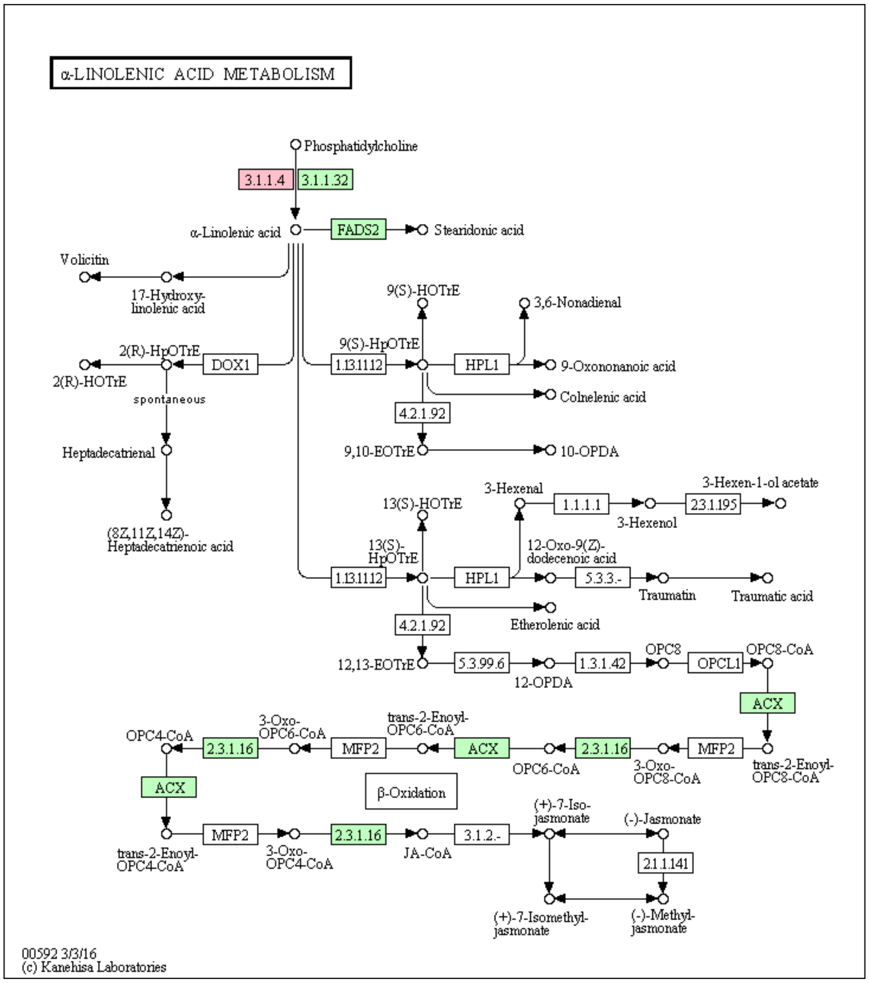

271.3 Legend:

RBH-Blast at 60% Identity + 50% Coverage
Green = Hit in *H. sapiens*
Red = Hit in *H. sapiens* and *T. californica*
White = Not in *H. sapiens*

---

272 Ovarian steroidogenesis

272.1 Human Pathway: HSA04913

272.2 Number of Hits: 1

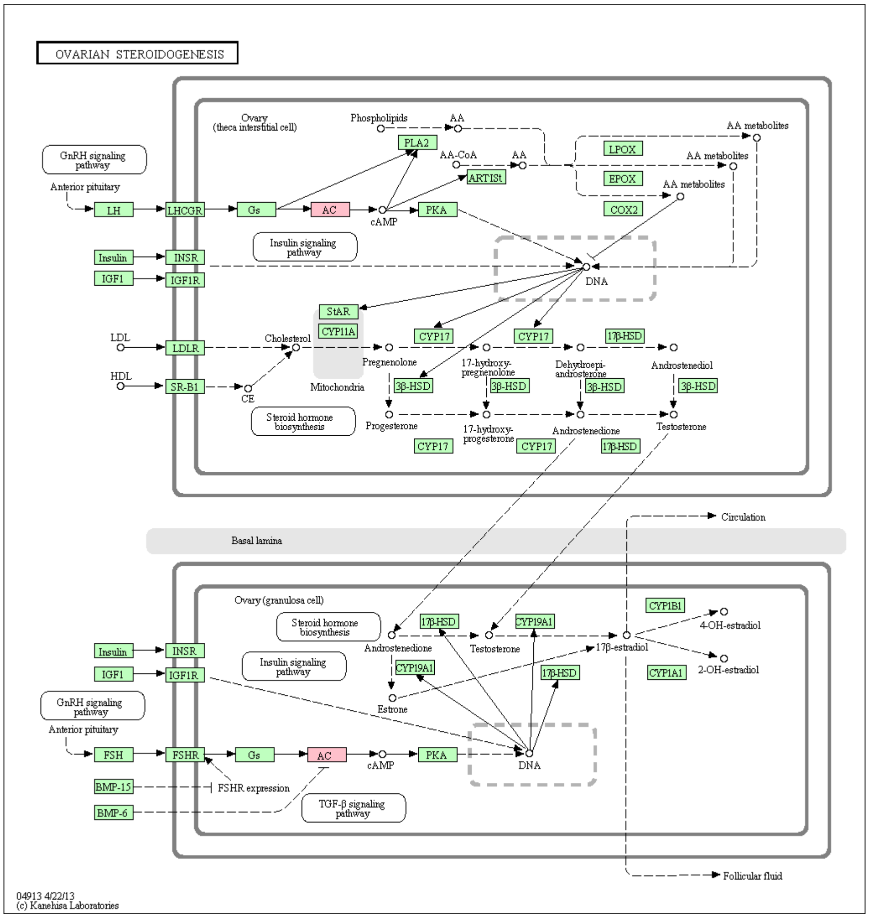

272.3 Legend:

RBH-Blast at 60% Identity + 50% Coverage
Green = Hit in *H. sapiens*
Red = Hit in *H. sapiens* and *T. californica*
White = Not in *H. sapiens*

273 Phenylalanine metabolism

273.1 Human Pathway: HSA00360

273.2 Number of Hits: 1

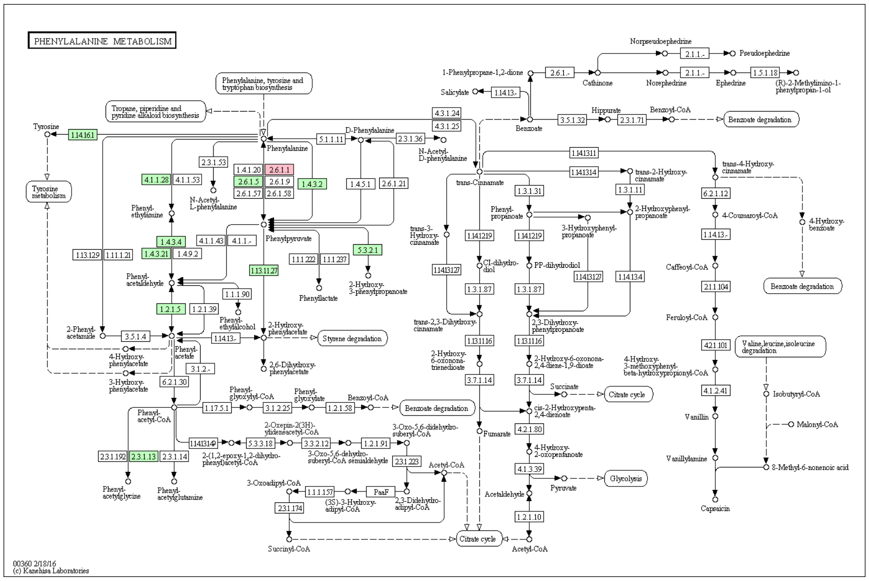

273.3 Legend:

RBH-Blast at 60% Identity + 50% Coverage
Green = Hit in *H. sapiens*
Red = Hit in *H. sapiens* and *T. californica*
White = Not in *H. sapiens*

274 Phototransduction

274.1 Human Pathway: HSA04744

274.2 Number of Hits: 1

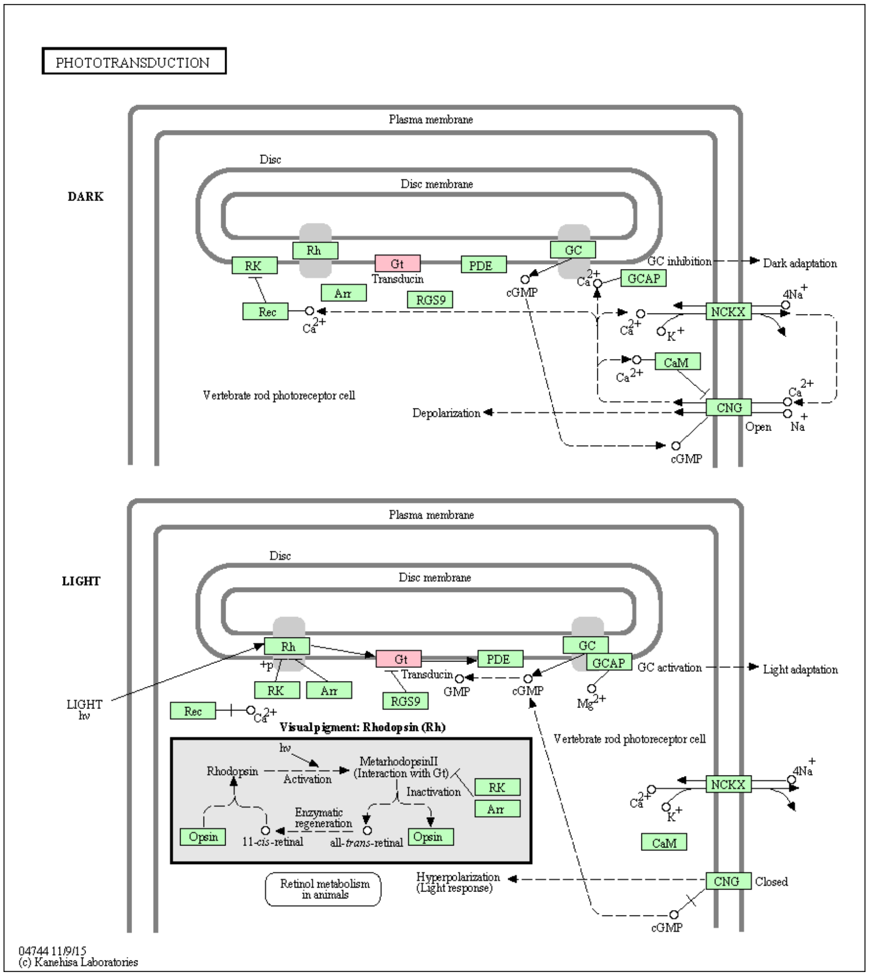

274.3 Legend:

RBH-Blast at 60% Identity + 50% Coverage
Green = Hit in *H. sapiens*
Red = Hit in *H. sapiens* and *T. californica*
White = Not in *H. sapiens*

275 Selenocompound metabolism

275.1 Human Pathway: HSA00450

275.2 Number of Hits: 1

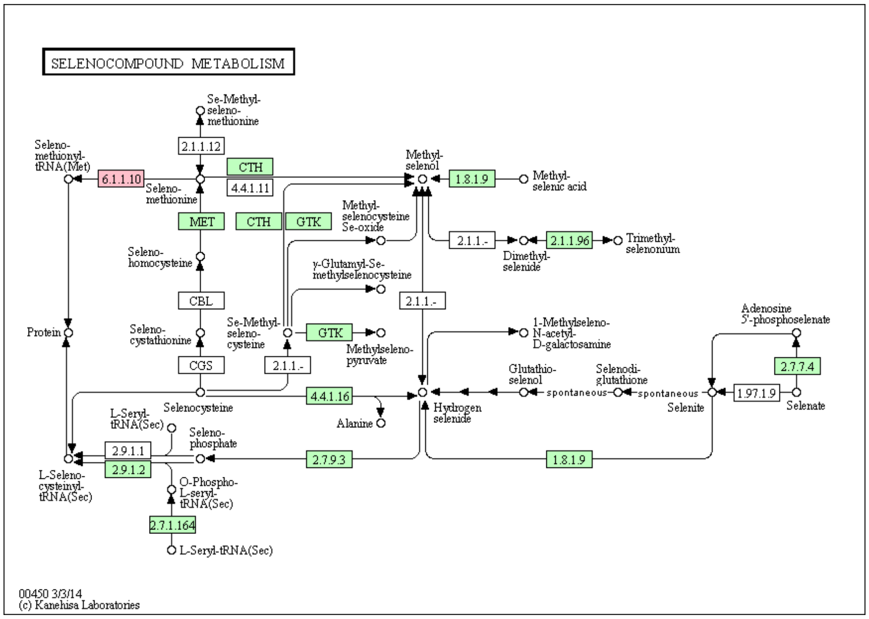

275.3 Legend:

RBH-Blast at 60% Identity + 50% Coverage
Green = Hit in *H. sapiens*
Red = Hit in *H. sapiens* and *T. californica*
White = Not in *H. sapiens*
