# Supplementary material for: Draft de novo transcriptome assembly and proteome characterization of the electric lobe of Tetronarce californica: a molecular tool for the study of cholinergic neurotransmission in the electric organ
Source: BMC Genomics. 2017 Aug 14;18:611. doi: 10.1186/s12864-017-3890-4 (PMC5557070; doi:10.1186/s12864-017-3890-4)
Supplement: Supplementary file 19 — KEGG Analysis. Intersection of H. sapiens With C. milli Without T. californica (set 9). See Additional file 3: TableS07 for details. (PDF 11366 kb) [file 12864_2017_3890_MOESM19_ESM.pdf]

Intersection of *H. sapiens* With *C. milli* Without *T. californica*

Set09: Reverse-Blast-Hit (RBH)-Blast at 60% Identity + 50% Coverage

Rodolfo Aramayo

April 30, 2017

Contents

1 Pathways in cancer **38**
1.1 Human Pathway: HSA05200 38
1.2 Number of Hits: 99 38
1.3 Legend: 38

2 Neuroactive ligand-receptor interaction **39**
2.1 Human Pathway: HSA04080 39
2.2 Number of Hits: 72 39
2.3 Legend: 39

3 cAMP signaling pathway **40**
3.1 Human Pathway: HSA04024 40
3.2 Number of Hits: 63 40
3.3 Legend: 40

4 HTLV-I infection **41**
4.1 Human Pathway: HSA05166 41
4.2 Number of Hits: 61 41
4.3 Legend: 42

5 PI3K-Akt signaling pathway **42**
5.1 Human Pathway: HSA04151 42
5.2 Number of Hits: 58 42
5.3 Legend: 42

6 Ras signaling pathway **43**
6.1 Human Pathway: HSA04014 43
6.2 Number of Hits: 57 43
6.3 Legend: 43

7 Rap1 signaling pathway **44**
7.1 Human Pathway: HSA04015 44
7.2 Number of Hits: 55 44
7.3 Legend: 44

8 Proteoglycans in cancer **45**
8.1 Human Pathway: HSA05205 45
8.2 Number of Hits: 54 45
8.3 Legend: 45

9 Axon guidance **46**
9.1 Human Pathway: HSA04360 46
9.2 Number of Hits: 53 46
9.3 Legend: 46

10 MAPK signaling pathway **47**
10.1 Human Pathway: HSA04010 47
10.2 Number of Hits: 52 47
10.3 Legend: 47

11 Calcium signaling pathway **48**
11.1 Human Pathway: HSA04020 48
11.2 Number of Hits: 51 48
11.3 Legend: 48

12 Signaling pathways regulating pluripotency of stem cells **49**
12.1 Human Pathway: HSA04550 49
12.2 Number of Hits: 48 49
12.3 Legend: 49

13 mTOR signaling pathway **50**
13.1 Human Pathway: HSA04150 50
13.2 Number of Hits: 47 50
13.3 Legend: 50

14 Hippo signaling pathway **51**
14.1 Human Pathway: HSA04390 51
14.2 Number of Hits: 46 51
14.3 Legend: 51

15 Regulation of actin cytoskeleton **52**
15.1 Human Pathway: HSA04810 52
15.2 Number of Hits: 46 52
15.3 Legend: 52

16 Wnt signaling pathway **53**
16.1 Human Pathway: HSA04310 53
16.2 Number of Hits: 43 53
16.3 Legend: 53

17 Oxytocin signaling pathway **54**
17.1 Human Pathway: HSA04921 54
17.2 Number of Hits: 40 54
17.3 Legend: 54

18 Focal adhesion **55**
18.1 Human Pathway: HSA04510 55
18.2 Number of Hits: 40 55
18.3 Legend: 55

19 MicroRNAs in cancer **56**
19.1 Human Pathway: HSA05206 56
19.2 Number of Hits: 39 56
19.3 Legend: 56

20 Purine metabolism **57**
20.1 Human Pathway: HSA00230 57
20.2 Number of Hits: 39 57
20.3 Legend: 57

21 Endocytosis **58**
21.1 Human Pathway: HSA04144 58
21.2 Number of Hits: 39 58
21.3 Legend: 58

22 Huntington's disease **59**
22.1 Human Pathway: HSA05016 59
22.2 Number of Hits: 38 59
22.3 Legend: 59

23 cGMP-PKG signaling pathway **60**
23.1 Human Pathway: HSA04022 60
23.2 Number of Hits: 38 60
23.3 Legend: 60

24 FoxO signaling pathway **61**
24.1 Human Pathway: HSA04068 61
24.2 Number of Hits: 37 61
24.3 Legend: 61

25 Insulin signaling pathway **62**
25.1 Human Pathway: HSA04910 62
25.2 Number of Hits: 35 62
25.3 Legend: 62

26 Viral carcinogenesis **63**
26.1 Human Pathway: HSA05203 63
26.2 Number of Hits: 34 63
26.3 Legend: 63

27 Phospholipase D signaling pathway **64**
27.1 Human Pathway: HSA04072 64
27.2 Number of Hits: 34 64
27.3 Legend: 64

28 Alzheimer's disease **65**
28.1 Human Pathway: HSA05010 65
28.2 Number of Hits: 34 65
28.3 Legend: 65

29 Thyroid hormone signaling pathway **66**
29.1 Human Pathway: HSA04919 66
29.2 Number of Hits: 34 66
29.3 Legend: 66

30 Chemokine signaling pathway **67**
30.1 Human Pathway: HSA04062 67
30.2 Number of Hits: 34 67
30.3 Legend: 67

31 Sphingolipid signaling pathway **68**
31.1 Human Pathway: HSA04071 68
31.2 Number of Hits: 34 68
31.3 Legend: 68

32 Adrenergic signaling in cardiomyocytes **69**
32.1 Human Pathway: HSA04261 69
32.2 Number of Hits: 34 69
32.3 Legend: 69

33 AMPK signaling pathway **70**
33.1 Human Pathway: HSA04152 70
33.2 Number of Hits: 33 70
33.3 Legend: 70

34 Neurotrophin signaling pathway **71**
34.1 Human Pathway: HSA04722 71
34.2 Number of Hits: 33 71
34.3 Legend: 71

35 Melanogenesis **72**
35.1 Human Pathway: HSA04916 72
35.2 Number of Hits: 32 72
35.3 Legend: 72

36 Cholinergic synapse **73**
36.1 Human Pathway: HSA04725 73
36.2 Number of Hits: 32 73
36.3 Legend: 73

37 Transcriptional misregulation in cancer **74**
37.1 Human Pathway: HSA05202 74
37.2 Number of Hits: 30 74
37.3 Legend: 74

38 Dopaminergic synapse **75**
38.1 Human Pathway: HSA04728 75
38.2 Number of Hits: 30 75
38.3 Legend: 75

39 Serotonergic synapse **76**
39.1 Human Pathway: HSA04726 76
39.2 Number of Hits: 28 76
39.3 Legend: 76

40 Epstein-Barr virus infection **77**
40.1 Human Pathway: HSA05169 77
40.2 Number of Hits: 28 77
40.3 Legend: 77

41 Retrograde endocannabinoid signaling **78**
41.1 Human Pathway: HSA04723 78
41.2 Number of Hits: 28 78
41.3 Legend: 78

42 Apoptosis **79**
42.1 Human Pathway: HSA04210 79
42.2 Number of Hits: 28 79
42.3 Legend: 79

43 Insulin resistance **80**
43.1 Human Pathway: HSA04931 80
43.2 Number of Hits: 27 80
43.3 Legend: 80

44 Cell cycle **81**
44.1 Human Pathway: HSA04110 81
44.2 Number of Hits: 27 81
44.3 Legend: 81

45 Circadian entrainment **82**
45.1 Human Pathway: HSA04713 82
45.2 Number of Hits: 27 82
45.3 Legend: 82

46 Alcoholism **83**
46.1 Human Pathway: HSA05034 83
46.2 Number of Hits: 27 83
46.3 Legend: 83

47 Leukocyte transendothelial migration **84**
47.1 Human Pathway: HSA04670 84
47.2 Number of Hits: 27 84
47.3 Legend: 84

48 Glucagon signaling pathway **85**
48.1 Human Pathway: HSA04922 85
48.2 Number of Hits: 27 85
48.3 Legend: 85

49 Glutamatergic synapse **86**
49.1 Human Pathway: HSA04724 86
49.2 Number of Hits: 27 86
49.3 Legend: 86

50 Tight junction **87**
50.1 Human Pathway: HSA04530 87
50.2 Number of Hits: 26 87
50.3 Legend: 87

51 Spliceosome **88**
51.1 Human Pathway: HSA03040 88
51.2 Number of Hits: 25 88
51.3 Legend: 88

52 Gap junction **89**
52.1 Human Pathway: HSA04540 89
52.2 Number of Hits: 25 89
52.3 Legend: 89

53 Morphine addiction **90**
53.1 Human Pathway: HSA05032 90
53.2 Number of Hits: 25 90
53.3 Legend: 90

54 Basal cell carcinoma **91**
54.1 Human Pathway: HSA05217 91
54.2 Number of Hits: 25 91
54.3 Legend: 91

55 Longevity regulating pathway **92**
55.1 Human Pathway: HSA04211 92
55.2 Number of Hits: 25 92
55.3 Legend: 92

56 Non-alcoholic fatty liver disease (NAFLD) **93**
56.1 Human Pathway: HSA04932 93
56.2 Number of Hits: 25 93
56.3 Legend: 93

57 Ubiquitin mediated proteolysis **94**
57.1 Human Pathway: HSA04120 94
57.2 Number of Hits: 25 94
57.3 Legend: 94

58 Protein processing in endoplasmic reticulum **95**
58.1 Human Pathway: HSA04141 95
58.2 Number of Hits: 25 95
58.3 Legend: 95

59 Prostate cancer **96**
59.1 Human Pathway: HSA05215 96
59.2 Number of Hits: 24 96
59.3 Legend: 96

60 Hepatitis C **97**
60.1 Human Pathway: HSA05160 97
60.2 Number of Hits: 24 97
60.3 Legend: 97

61 RNA transport **98**
61.1 Human Pathway: HSA03013 98
61.2 Number of Hits: 24 98
61.3 Legend: 98

62 Lysosome **99**
62.1 Human Pathway: HSA04142 99
62.2 Number of Hits: 24 99
62.3 Legend: 99

63 Platelet activation **100**
63.1 Human Pathway: HSA04611 100
63.2 Number of Hits: 24 100
63.3 Legend: 100

64 Hepatitis B **101**
64.1 Human Pathway: HSA05161 101
64.2 Number of Hits: 24 101
64.3 Legend: 101

65 Estrogen signaling pathway **102**
65.1 Human Pathway: HSA04915 102
65.2 Number of Hits: 24 102
65.3 Legend: 102

66 Vascular smooth muscle contraction **103**
66.1 Human Pathway: HSA04270 103
66.2 Number of Hits: 23 103
66.3 Legend: 103

67 Inflammatory mediator regulation of TRP channels **104**
67.1 Human Pathway: HSA04750 104
67.2 Number of Hits: 23 104
67.3 Legend: 104

68 Phosphatidylinositol signaling system **105**
68.1 Human Pathway: HSA04070 105
68.2 Number of Hits: 23 105
68.3 Legend: 105

69 AGE-RAGE signaling pathway in diabetic complications **106**
69.1 Human Pathway: HSA04933 106
69.2 Number of Hits: 23 106
69.3 Legend: 106

70 GnRH signaling pathway **107**
70.1 Human Pathway: HSA04912 107
70.2 Number of Hits: 22 107
70.3 Legend: 107

71 Renal cell carcinoma **108**
71.1 Human Pathway: HSA05211 108
71.2 Number of Hits: 22 108
71.3 Legend: 108

72 Progesterone-mediated oocyte maturation **109**
72.1 Human Pathway: HSA04914 109
72.2 Number of Hits: 22 109
72.3 Legend: 109

73 Prolactin signaling pathway **110**
73.1 Human Pathway: HSA04917 110
73.2 Number of Hits: 22 110
73.3 Legend: 110

74 Influenza A **111**
74.1 Human Pathway: HSA05164 111
74.2 Number of Hits: 21 111
74.3 Legend: 111

75 Parkinson's disease **112**
75.1 Human Pathway: HSA05012 112
75.2 Number of Hits: 21 112
75.3 Legend: 112

76 Chronic myeloid leukemia **113**
76.1 Human Pathway: HSA05220 113
76.2 Number of Hits: 21 113
76.3 Legend: 113

77 Osteoclast differentiation **114**
77.1 Human Pathway: HSA04380 114
77.2 Number of Hits: 21 114
77.3 Legend: 114

78 Longevity regulating pathway - multiple species 115
78.1 Human Pathway: HSA04213 115
78.2 Number of Hits: 21 115
78.3 Legend: 115

79 Choline metabolism in cancer 116
79.1 Human Pathway: HSA05231 116
79.2 Number of Hits: 21 116
79.3 Legend: 116

80 T cell receptor signaling pathway 117
80.1 Human Pathway: HSA04660 117
80.2 Number of Hits: 21 117
80.3 Legend: 117

81 Glycerophospholipid metabolism 118
81.1 Human Pathway: HSA00564 118
81.2 Number of Hits: 21 118
81.3 Legend: 118

82 Adherens junction 119
82.1 Human Pathway: HSA04520 119
82.2 Number of Hits: 20 119
82.3 Legend: 119

83 EGFR tyrosine kinase inhibitor resistance 120
83.1 Human Pathway: HSA01521 120
83.2 Number of Hits: 20 120
83.3 Legend: 120

84 HIF-1 signaling pathway 121
84.1 Human Pathway: HSA04066 121
84.2 Number of Hits: 20 121
84.3 Legend: 121

85 Melanoma 122
85.1 Human Pathway: HSA05218 122
85.2 Number of Hits: 20 122
85.3 Legend: 122

86 GABAergic synapse **123**
86.1 Human Pathway: HSA04727 123
86.2 Number of Hits: 20 123
86.3 Legend: 123

87 Pyrimidine metabolism **124**
87.1 Human Pathway: HSA00240 124
87.2 Number of Hits: 20 124
87.3 Legend: 124

88 Pancreatic secretion **125**
88.1 Human Pathway: HSA04972 125
88.2 Number of Hits: 20 125
88.3 Legend: 125

89 ErbB signaling pathway **126**
89.1 Human Pathway: HSA04012 126
89.2 Number of Hits: 20 126
89.3 Legend: 126

90 Chagas disease (American trypanosomiasis) **127**
90.1 Human Pathway: HSA05142 127
90.2 Number of Hits: 19 127
90.3 Legend: 127

91 Fc gamma R-mediated phagocytosis **128**
91.1 Human Pathway: HSA04666 128
91.2 Number of Hits: 19 128
91.3 Legend: 128

92 Jak-STAT signaling pathway **129**
92.1 Human Pathway: HSA04630 129
92.2 Number of Hits: 19 129
92.3 Legend: 129

93 Long-term depression **130**
93.1 Human Pathway: HSA04730 130
93.2 Number of Hits: 19 130
93.3 Legend: 130

94 Pancreatic cancer 131
94.1 Human Pathway: HSA05212 131
94.2 Number of Hits: 18 131
94.3 Legend: 131

95 Peroxisome 132
95.1 Human Pathway: HSA04146 132
95.2 Number of Hits: 18 132
95.3 Legend: 132

96 Glioma 133
96.1 Human Pathway: HSA05214 133
96.2 Number of Hits: 18 133
96.3 Legend: 133

97 Long-term potentiation 134
97.1 Human Pathway: HSA04720 134
97.2 Number of Hits: 18 134
97.3 Legend: 134

98 Ribosome 135
98.1 Human Pathway: HSA03010 135
98.2 Number of Hits: 18 135
98.3 Legend: 135

99 Hypertrophic cardiomyopathy (HCM) 136
99.1 Human Pathway: HSA05410 136
99.2 Number of Hits: 18 136
99.3 Legend: 136

100 Phagosome 137
100.1 Human Pathway: HSA04145 137
100.2 Number of Hits: 18 137
100.3 Legend: 137

101 Bile secretion 138
101.1 Human Pathway: HSA04976 138
101.2 Number of Hits: 18 138
101.3 Legend: 138

102 Dilated cardiomyopathy 139
102.1 Human Pathway: HSA05414 139
102.2 Number of Hits: 18 139
102.3 Legend: 139

103 Tuberculosis 140
103.1 Human Pathway: HSA05152 140
103.2 Number of Hits: 18 140
103.3 Legend: 140

104 Toxoplasmosis 141
104.1 Human Pathway: HSA05145 141
104.2 Number of Hits: 18 141
104.3 Legend: 141

105 Oxidative phosphorylation 142
105.1 Human Pathway: HSA00190 142
105.2 Number of Hits: 18 142
105.3 Legend: 142

106 mRNA surveillance pathway 143
106.1 Human Pathway: HSA03015 143
106.2 Number of Hits: 17 143
106.3 Legend: 143

107 Regulation of lipolysis in adipocytes 144
107.1 Human Pathway: HSA04923 144
107.2 Number of Hits: 17 144
107.3 Legend: 144

108 Acute myeloid leukemia 145
108.1 Human Pathway: HSA05221 145
108.2 Number of Hits: 17 145
108.3 Legend: 145

109 Measles 146
109.1 Human Pathway: HSA05162 146
109.2 Number of Hits: 17 146
109.3 Legend: 147

110 Endometrial cancer **147**
110.1 Human Pathway: HSA05213 147
110.2 Number of Hits: 17 147
110.3 Legend: 147

111 Small cell lung cancer **148**
111.1 Human Pathway: HSA05222 148
111.2 Number of Hits: 17 148
111.3 Legend: 148

112 Non-small cell lung cancer **149**
112.1 Human Pathway: HSA05223 149
112.2 Number of Hits: 17 149
112.3 Legend: 149

113 Insulin secretion **150**
113.1 Human Pathway: HSA04911 150
113.2 Number of Hits: 17 150
113.3 Legend: 150

114 TNF signaling pathway **151**
114.1 Human Pathway: HSA04668 151
114.2 Number of Hits: 17 151
114.3 Legend: 151

115 Salivary secretion **152**
115.1 Human Pathway: HSA04970 152
115.2 Number of Hits: 17 152
115.3 Legend: 152

116 Gastric acid secretion **153**
116.1 Human Pathway: HSA04971 153
116.2 Number of Hits: 17 153
116.3 Legend: 153

117 Nicotine addiction **154**
117.1 Human Pathway: HSA05033 154
117.2 Number of Hits: 17 154
117.3 Legend: 154

118 Inositol phosphate metabolism **155**
118.1 Human Pathway: HSA00562 155
118.2 Number of Hits: 17 155
118.3 Legend: 155

119 Adipocytokine signaling pathway **156**
119.1 Human Pathway: HSA04920 156
119.2 Number of Hits: 17 156
119.3 Legend: 156

120 Fc epsilon RI signaling pathway **157**
120.1 Human Pathway: HSA04664 157
120.2 Number of Hits: 16 157
120.3 Legend: 157

121 Cell adhesion molecules (CAMs) **158**
121.1 Human Pathway: HSA04514 158
121.2 Number of Hits: 16 158
121.3 Legend: 158

122 Herpes simplex infection **159**
122.1 Human Pathway: HSA05168 159
122.2 Number of Hits: 16 159
122.3 Legend: 159

123 Arrhythmogenic right ventricular cardiomyopathy (ARVC) **160**
123.1 Human Pathway: HSA05412 160
123.2 Number of Hits: 16 160
123.3 Legend: 160

124 Notch signaling pathway **161**
124.1 Human Pathway: HSA04330 161
124.2 Number of Hits: 16 161
124.3 Legend: 161

125 Oocyte meiosis **162**
125.1 Human Pathway: HSA04114 162
125.2 Number of Hits: 16 162
125.3 Legend: 162

126 B cell receptor signaling pathway 163
126.1 Human Pathway: HSA04662 163
126.2 Number of Hits: 16 163
126.3 Legend: 163

127 p53 signaling pathway 164
127.1 Human Pathway: HSA04115 164
127.2 Number of Hits: 16 164
127.3 Legend: 164

128 TGF-beta signaling pathway 165
128.1 Human Pathway: HSA04350 165
128.2 Number of Hits: 16 165
128.3 Legend: 165

129 Natural killer cell mediated cytotoxicity 166
129.1 Human Pathway: HSA04650 166
129.2 Number of Hits: 16 166
129.3 Legend: 166

130 Taste transduction 167
130.1 Human Pathway: HSA04742 167
130.2 Number of Hits: 16 167
130.3 Legend: 167

131 N-Glycan biosynthesis 168
131.1 Human Pathway: HSA00510 168
131.2 Number of Hits: 15 168
131.3 Legend: 169

132 Renin secretion 169
132.1 Human Pathway: HSA04924 169
132.2 Number of Hits: 15 169
132.3 Legend: 169

133 Amoebiasis 170
133.1 Human Pathway: HSA05146 170
133.2 Number of Hits: 15 170
133.3 Legend: 170

134 Type II diabetes mellitus **171**
134.1 Human Pathway: HSA04930 171
134.2 Number of Hits: 15 171
134.3 Legend: 171

135 Sphingolipid metabolism **172**
135.1 Human Pathway: HSA00600 172
135.2 Number of Hits: 15 172
135.3 Legend: 172

136 Aldosterone synthesis and secretion **173**
136.1 Human Pathway: HSA04925 173
136.2 Number of Hits: 15 173
136.3 Legend: 173

137 Protein digestion and absorption **174**
137.1 Human Pathway: HSA04974 174
137.2 Number of Hits: 15 174
137.3 Legend: 174

138 Epithelial cell signaling in Helicobacter pylori infection **175**
138.1 Human Pathway: HSA05120 175
138.2 Number of Hits: 15 175
138.3 Legend: 175

139 Salmonella infection **176**
139.1 Human Pathway: HSA05132 176
139.2 Number of Hits: 15 176
139.3 Legend: 176

140 Colorectal cancer **177**
140.1 Human Pathway: HSA05210 177
140.2 Number of Hits: 15 177
140.3 Legend: 177

141 Fatty acid metabolism **178**
141.1 Human Pathway: HSA01212 178
141.2 Number of Hits: 15 178
141.3 Legend: 179

142 Thyroid hormone synthesis 179
142.1 Human Pathway: HSA04918 179
142.2 Number of Hits: 15 179
142.3 Legend: 180

143 Synaptic vesicle cycle 180
143.1 Human Pathway: HSA04721 180
143.2 Number of Hits: 14 180
143.3 Legend: 180

144 Bacterial invasion of epithelial cells 181
144.1 Human Pathway: HSA05100 181
144.2 Number of Hits: 14 181
144.3 Legend: 181

145 Glycerolipid metabolism 182
145.1 Human Pathway: HSA00561 182
145.2 Number of Hits: 14 182
145.3 Legend: 182

146 Cardiac muscle contraction 183
146.1 Human Pathway: HSA04260 183
146.2 Number of Hits: 14 183
146.3 Legend: 183

147 Cysteine and methionine metabolism 184
147.1 Human Pathway: HSA00270 184
147.2 Number of Hits: 14 184
147.3 Legend: 184

148 Amphetamine addiction 185
148.1 Human Pathway: HSA05031 185
148.2 Number of Hits: 14 185
148.3 Legend: 185

149 ABC transporters 186
149.1 Human Pathway: HSA02010 186
149.2 Number of Hits: 13 186
149.3 Legend: 187

150 Arginine and proline metabolism **187**
150.1 Human Pathway: HSA00330 187
150.2 Number of Hits: 13 187
150.3 Legend: 187

151 Central carbon metabolism in cancer **188**
151.1 Human Pathway: HSA05230 188
151.2 Number of Hits: 13 188
151.3 Legend: 188

152 Toll-like receptor signaling pathway **189**
152.1 Human Pathway: HSA04620 189
152.2 Number of Hits: 13 189
152.3 Legend: 189

153 Phototransduction **190**
153.1 Human Pathway: HSA04744 190
153.2 Number of Hits: 13 190
153.3 Legend: 190

154 PPAR signaling pathway **191**
154.1 Human Pathway: HSA03320 191
154.2 Number of Hits: 13 191
154.3 Legend: 191

155 Dorso-ventral axis formation **192**
155.1 Human Pathway: HSA04320 192
155.2 Number of Hits: 13 192
155.3 Legend: 192

156 Cocaine addiction **193**
156.1 Human Pathway: HSA05030 193
156.2 Number of Hits: 13 193
156.3 Legend: 193

157 NF-kappa B signaling pathway **194**
157.1 Human Pathway: HSA04064 194
157.2 Number of Hits: 12 194
157.3 Legend: 194

158 Glycosaminoglycan biosynthesis - heparan sulfate / heparin 195
158.1 Human Pathway: HSA00534 195
158.2 Number of Hits: 12 195
158.3 Legend: 195

159 Carbon metabolism 196
159.1 Human Pathway: HSA01200 196
159.2 Number of Hits: 12 196
159.3 Legend: 196

160 RNA degradation 197
160.1 Human Pathway: HSA03018 197
160.2 Number of Hits: 12 197
160.3 Legend: 197

161 Carbohydrate digestion and absorption 198
161.1 Human Pathway: HSA04973 198
161.2 Number of Hits: 11 198
161.3 Legend: 198

162 VEGF signaling pathway 199
162.1 Human Pathway: HSA04370 199
162.2 Number of Hits: 11 199
162.3 Legend: 199

163 Chemical carcinogenesis 201
163.1 Human Pathway: HSA05204 201
163.2 Number of Hits: 11 201
163.3 Legend: 202

164 Platinum drug resistance 202
164.1 Human Pathway: HSA01524 202
164.2 Number of Hits: 11 202
164.3 Legend: 202

165 ECM-receptor interaction 203
165.1 Human Pathway: HSA04512 203
165.2 Number of Hits: 11 203
165.3 Legend: 203

166 Amino sugar and nucleotide sugar metabolism **204**
166.1 Human Pathway: HSA00520 204
166.2 Number of Hits: 11 204
166.3 Legend: 205

167 Vibrio cholerae infection **205**
167.1 Human Pathway: HSA05110 205
167.2 Number of Hits: 11 205
167.3 Legend: 206

168 Shigellosis **207**
168.1 Human Pathway: HSA05131 207
168.2 Number of Hits: 10 207
168.3 Legend: 207

169 Cytokine-cytokine receptor interaction **208**
169.1 Human Pathway: HSA04060 208
169.2 Number of Hits: 10 208
169.3 Legend: 208

170 Ribosome biogenesis in eukaryotes **209**
170.1 Human Pathway: HSA03008 209
170.2 Number of Hits: 10 209
170.3 Legend: 210

171 Mucin type O-Glycan biosynthesis **212**
171.1 Human Pathway: HSA00512 212
171.2 Number of Hits: 10 212
171.3 Legend: 213

172 Starch and sucrose metabolism **213**
172.1 Human Pathway: HSA00500 213
172.2 Number of Hits: 10 213
172.3 Legend: 213

173 Glycosaminoglycan biosynthesis - chondroitin sulfate / dermatan sulfate **214**
173.1 Human Pathway: HSA00532 214
173.2 Number of Hits: 10 214
173.3 Legend: 214

174 Metabolism of xenobiotics by cytochrome P450 **215**
174.1 Human Pathway: HSA00980 215
174.2 Number of Hits: 9 215
174.3 Legend: 215

175 Tyrosine metabolism **216**
175.1 Human Pathway: HSA00350 216
175.2 Number of Hits: 9 216
175.3 Legend: 216

176 Amyotrophic lateral sclerosis (ALS) **217**
176.1 Human Pathway: HSA05014 217
176.2 Number of Hits: 9 217
176.3 Legend: 217

177 Retinol metabolism **218**
177.1 Human Pathway: HSA00830 218
177.2 Number of Hits: 9 218
177.3 Legend: 218

178 Ovarian steroidogenesis **219**
178.1 Human Pathway: HSA04913 219
178.2 Number of Hits: 9 219
178.3 Legend: 219

179 Glutathione metabolism **220**
179.1 Human Pathway: HSA00480 220
179.2 Number of Hits: 9 220
179.3 Legend: 220

180 Olfactory transduction **221**
180.1 Human Pathway: HSA04740 221
180.2 Number of Hits: 9 221
180.3 Legend: 221

181 Tryptophan metabolism **222**
181.1 Human Pathway: HSA00380 222
181.2 Number of Hits: 9 222
181.3 Legend: 222

182 Bladder cancer **223**
182.1 Human Pathway: HSA05219 223
182.2 Number of Hits: 9 223
182.3 Legend: 223

183 Drug metabolism - other enzymes **224**
183.1 Human Pathway: HSA00983 224
183.2 Number of Hits: 9 224
183.3 Legend: 224

184 DNA replication **225**
184.1 Human Pathway: HSA03030 225
184.2 Number of Hits: 9 225
184.3 Legend: 225

185 Hedgehog signaling pathway **226**
185.1 Human Pathway: HSA04340 226
185.2 Number of Hits: 9 226
185.3 Legend: 226

186 Glycine, serine and threonine metabolism **227**
186.1 Human Pathway: HSA00260 227
186.2 Number of Hits: 9 227
186.3 Legend: 227

187 Biosynthesis of amino acids **228**
187.1 Human Pathway: HSA01230 228
187.2 Number of Hits: 8 228
187.3 Legend: 228

188 Fatty acid degradation **229**
188.1 Human Pathway: HSA00071 229
188.2 Number of Hits: 8 229
188.3 Legend: 229

189 Alanine, aspartate and glutamate metabolism **230**
189.1 Human Pathway: HSA00250 230
189.2 Number of Hits: 8 230
189.3 Legend: 230

190 Pyruvate metabolism **231**
190.1 Human Pathway: HSA00620 231
190.2 Number of Hits: 8 231
190.3 Legend: 231

191 Aldosterone-regulated sodium reabsorption **232**
191.1 Human Pathway: HSA04960 232
191.2 Number of Hits: 8 232
191.3 Legend: 232

192 Drug metabolism - cytochrome P450 **233**
192.1 Human Pathway: HSA00982 233
192.2 Number of Hits: 8 233
192.3 Legend: 233

193 Vasopressin-regulated water reabsorption **234**
193.1 Human Pathway: HSA04962 234
193.2 Number of Hits: 8 234
193.3 Legend: 234

194 Lysine degradation **235**
194.1 Human Pathway: HSA00310 235
194.2 Number of Hits: 8 235
194.3 Legend: 235

195 Aminoacyl-tRNA biosynthesis **236**
195.1 Human Pathway: HSA00970 236
195.2 Number of Hits: 8 236
195.3 Legend: 236

196 Glycolysis / Gluconeogenesis **238**
196.1 Human Pathway: HSA00010 238
196.2 Number of Hits: 8 238
196.3 Legend: 239

197 Glycosylphosphatidylinositol(GPI)-anchor biosynthesis **240**
197.1 Human Pathway: HSA00563 240
197.2 Number of Hits: 8 240
197.3 Legend: 241

198 Nucleotide excision repair **241**
198.1 Human Pathway: HSA03420 241
198.2 Number of Hits: 8 241
198.3 Legend: 241

199 Arachidonic acid metabolism **242**
199.1 Human Pathway: HSA00590 242
199.2 Number of Hits: 8 242
199.3 Legend: 242

200 NOD-like receptor signaling pathway **243**
200.1 Human Pathway: HSA04621 243
200.2 Number of Hits: 8 243
200.3 Legend: 243

201 Fanconi anemia pathway **244**
201.1 Human Pathway: HSA03460 244
201.2 Number of Hits: 8 244
201.3 Legend: 245

202 Base excision repair **245**
202.1 Human Pathway: HSA03410 245
202.2 Number of Hits: 8 245
202.3 Legend: 246

203 Mineral absorption **246**
203.1 Human Pathway: HSA04978 246
203.2 Number of Hits: 8 246
203.3 Legend: 247

204 Valine, leucine and isoleucine degradation **248**
204.1 Human Pathway: HSA00280 248
204.2 Number of Hits: 7 248
204.3 Legend: 249

205 Basal transcription factors **249**
205.1 Human Pathway: HSA03022 249
205.2 Number of Hits: 7 249
205.3 Legend: 250

206 Leishmaniasis **250**
206.1 Human Pathway: HSA05140 250
206.2 Number of Hits: 7 250
206.3 Legend: 250

207 Steroid hormone biosynthesis **251**
207.1 Human Pathway: HSA00140 251
207.2 Number of Hits: 7 251
207.3 Legend: 251

208 beta-Alanine metabolism **252**
208.1 Human Pathway: HSA00410 252
208.2 Number of Hits: 7 252
208.3 Legend: 252

209 Hippo signaling pathway -multiple species **253**
209.1 Human Pathway: HSA04392 253
209.2 Number of Hits: 7 253
209.3 Legend: 253

210 RIG-I-like receptor signaling pathway **254**
210.1 Human Pathway: HSA04622 254
210.2 Number of Hits: 7 254
210.3 Legend: 254

211 Circadian rhythm **255**
211.1 Human Pathway: HSA04710 255
211.2 Number of Hits: 7 255
211.3 Legend: 255

212 Pertussis **256**
212.1 Human Pathway: HSA05133 256
212.2 Number of Hits: 7 256
212.3 Legend: 256

213 Histidine metabolism **257**
213.1 Human Pathway: HSA00340 257
213.2 Number of Hits: 7 257
213.3 Legend: 257

214 Phenylalanine metabolism **258**
214.1 Human Pathway: HSA00360 258
214.2 Number of Hits: 6 258
214.3 Legend: 258

215 Rheumatoid arthritis **259**
215.1 Human Pathway: HSA05323 259
215.2 Number of Hits: 6 259
215.3 Legend: 259

216 Maturity onset diabetes of the young **260**
216.1 Human Pathway: HSA04950 260
216.2 Number of Hits: 6 260
216.3 Legend: 261

217 Fatty acid elongation **262**
217.1 Human Pathway: HSA00062 262
217.2 Number of Hits: 6 262
217.3 Legend: 263

218 Galactose metabolism **263**
218.1 Human Pathway: HSA00052 263
218.2 Number of Hits: 6 263
218.3 Legend: 263

219 Steroid biosynthesis **264**
219.1 Human Pathway: HSA00100 264
219.2 Number of Hits: 6 264
219.3 Legend: 264

220 Fat digestion and absorption **265**
220.1 Human Pathway: HSA04975 265
220.2 Number of Hits: 6 265
220.3 Legend: 265

221 Glyoxylate and dicarboxylate metabolism **266**
221.1 Human Pathway: HSA00630 266
221.2 Number of Hits: 6 266
221.3 Legend: 266

222 Inflammatory bowel disease (IBD) 267
222.1 Human Pathway: HSA05321 267
222.2 Number of Hits: 6 267
222.3 Legend: 267

223 Pathogenic Escherichia coli infection 268
223.1 Human Pathway: HSA05130 268
223.2 Number of Hits: 5 268
223.3 Legend: 268

224 Fructose and mannose metabolism 269
224.1 Human Pathway: HSA00051 269
224.2 Number of Hits: 5 269
224.3 Legend: 269

225 Primary immunodeficiency 270
225.1 Human Pathway: HSA05340 270
225.2 Number of Hits: 5 270
225.3 Legend: 270

226 Ascorbate and aldarate metabolism 271
226.1 Human Pathway: HSA00053 271
226.2 Number of Hits: 5 271
226.3 Legend: 271

227 Regulation of autophagy 272
227.1 Human Pathway: HSA04140 272
227.2 Number of Hits: 5 272
227.3 Legend: 272

228 Endocrine and other factor-regulated calcium reabsorption 273
228.1 Human Pathway: HSA04961 273
228.2 Number of Hits: 5 273
228.3 Legend: 273

229 Pentose phosphate pathway 274
229.1 Human Pathway: HSA00030 274
229.2 Number of Hits: 5 274
229.3 Legend: 274

230 Fatty acid biosynthesis **275**
230.1 Human Pathway: HSA00061 275
230.2 Number of Hits: 5 275
230.3 Legend: 275

231 Systemic lupus erythematosus **276**
231.1 Human Pathway: HSA05322 276
231.2 Number of Hits: 5 276
231.3 Legend: 276

232 Biosynthesis of unsaturated fatty acids **277**
232.1 Human Pathway: HSA01040 277
232.2 Number of Hits: 5 277
232.3 Legend: 277

233 Propanoate metabolism **278**
233.1 Human Pathway: HSA00640 278
233.2 Number of Hits: 5 278
233.3 Legend: 278

234 Collecting duct acid secretion **279**
234.1 Human Pathway: HSA04966 279
234.2 Number of Hits: 5 279
234.3 Legend: 280

235 Thyroid cancer **280**
235.1 Human Pathway: HSA05216 280
235.2 Number of Hits: 5 280
235.3 Legend: 280

236 Glycosphingolipid biosynthesis - ganglio series **281**
236.1 Human Pathway: HSA00604 281
236.2 Number of Hits: 5 281
236.3 Legend: 282

237 Ether lipid metabolism **282**
237.1 Human Pathway: HSA00565 282
237.2 Number of Hits: 5 282
237.3 Legend: 283

238 Arginine biosynthesis **283**
238.1 Human Pathway: HSA00220 283
238.2 Number of Hits: 4 283
238.3 Legend: 283

239 Legionellosis **284**
239.1 Human Pathway: HSA05134 284
239.2 Number of Hits: 4 284
239.3 Legend: 284

240 Glycosphingolipid biosynthesis - lacto and neolacto series **285**
240.1 Human Pathway: HSA00601 285
240.2 Number of Hits: 4 285
240.3 Legend: 286

241 Citrate cycle (TCA cycle) **286**
241.1 Human Pathway: HSA00020 286
241.2 Number of Hits: 4 286
241.3 Legend: 286

242 Pantothenate and CoA biosynthesis **287**
242.1 Human Pathway: HSA00770 287
242.2 Number of Hits: 4 287
242.3 Legend: 287

243 Pentose and glucuronate interconversions **288**
243.1 Human Pathway: HSA00040 288
243.2 Number of Hits: 4 288
243.3 Legend: 288

244 Other types of O-glycan biosynthesis **289**
244.1 Human Pathway: HSA00514 289
244.2 Number of Hits: 4 289
244.3 Legend: 289

245 Vitamin digestion and absorption **290**
245.1 Human Pathway: HSA04977 290
245.2 Number of Hits: 4 290
245.3 Legend: 290

246 Porphyrin and chlorophyll metabolism **291**
246.1 Human Pathway: HSA00860 291
246.2 Number of Hits: 4 291
246.3 Legend: 291

247 SNARE interactions in vesicular transport **292**
247.1 Human Pathway: HSA04130 292
247.2 Number of Hits: 4 292
247.3 Legend: 292

248 Homologous recombination **293**
248.1 Human Pathway: HSA03440 293
248.2 Number of Hits: 4 293
248.3 Legend: 293

249 Proteasome **294**
249.1 Human Pathway: HSA03050 294
249.2 Number of Hits: 4 294
249.3 Legend: 294

250 Apoptosis - multiple species **295**
250.1 Human Pathway: HSA04215 295
250.2 Number of Hits: 4 295
250.3 Legend: 295

251 Glycosaminoglycan degradation **296**
251.1 Human Pathway: HSA00531 296
251.2 Number of Hits: 3 296
251.3 Legend: 296

252 RNA polymerase **297**
252.1 Human Pathway: HSA03020 297
252.2 Number of Hits: 3 297
252.3 Legend: 297

253 Protein export **298**
253.1 Human Pathway: HSA03060 298
253.2 Number of Hits: 3 298
253.3 Legend: 298

254 Terpenoid backbone biosynthesis **299**
254.1 Human Pathway: HSA00900 299
254.2 Number of Hits: 3 299
254.3 Legend: 300

255 Non-homologous end-joining **300**
255.1 Human Pathway: HSA03450 300
255.2 Number of Hits: 3 300
255.3 Legend: 300

256 Viral myocarditis **301**
256.1 Human Pathway: HSA05416 301
256.2 Number of Hits: 3 301
256.3 Legend: 301

257 Mismatch repair **302**
257.1 Human Pathway: HSA03430 302
257.2 Number of Hits: 3 302
257.3 Legend: 302

258 Butanoate metabolism **303**
258.1 Human Pathway: HSA00650 303
258.2 Number of Hits: 3 303
258.3 Legend: 303

259 Antigen processing and presentation **304**
259.1 Human Pathway: HSA04612 304
259.2 Number of Hits: 3 304
259.3 Legend: 304

260 Proximal tubule bicarbonate reclamation **305**
260.1 Human Pathway: HSA04964 305
260.2 Number of Hits: 3 305
260.3 Legend: 306

261 African trypanosomiasis **306**
261.1 Human Pathway: HSA05143 306
261.2 Number of Hits: 3 306
261.3 Legend: 306

262 Cytosolic DNA-sensing pathway **307**
262.1 Human Pathway: HSA04623 307
262.2 Number of Hits: 3 307
262.3 Legend: 307

263 Other glycan degradation **308**
263.1 Human Pathway: HSA00511 308
263.2 Number of Hits: 3 308
263.3 Legend: 308

264 Renin-angiotensin system **309**
264.1 Human Pathway: HSA04614 309
264.2 Number of Hits: 2 309
264.3 Legend: 309

265 Sulfur relay system **310**
265.1 Human Pathway: HSA04122 310
265.2 Number of Hits: 2 310
265.3 Legend: 310

266 Ubiquinone and other terpenoid-quinone biosynthesis **311**
266.1 Human Pathway: HSA00130 311
266.2 Number of Hits: 2 311
266.3 Legend: 312

267 One carbon pool by folate **312**
267.1 Human Pathway: HSA00670 312
267.2 Number of Hits: 2 312
267.3 Legend: 312

268 Prion diseases **313**
268.1 Human Pathway: HSA05020 313
268.2 Number of Hits: 2 313
268.3 Legend: 313

269 Nicotinate and nicotinamide metabolism **314**
269.1 Human Pathway: HSA00760 314
269.2 Number of Hits: 2 314
269.3 Legend: 314

270 Synthesis and degradation of ketone bodies **315**
270.1 Human Pathway: HSA00072 315
270.2 Number of Hits: 2 315
270.3 Legend: 315

271 Nitrogen metabolism **316**
271.1 Human Pathway: HSA00910 316
271.2 Number of Hits: 2 316
271.3 Legend: 316

272 Selenocompound metabolism **317**
272.1 Human Pathway: HSA00450 317
272.2 Number of Hits: 2 317
272.3 Legend: 317

273 Primary bile acid biosynthesis **319**
273.1 Human Pathway: HSA00120 319
273.2 Number of Hits: 2 319
273.3 Legend: 320

274 Taurine and hypotaurine metabolism **320**
274.1 Human Pathway: HSA00430 320
274.2 Number of Hits: 2 320
274.3 Legend: 320

275 alpha-Linolenic acid metabolism **321**
275.1 Human Pathway: HSA00592 321
275.2 Number of Hits: 2 321
275.3 Legend: 321

276 Folate biosynthesis **322**
276.1 Human Pathway: HSA00790 322
276.2 Number of Hits: 2 322
276.3 Legend: 322

277 Caffeine metabolism **323**
277.1 Human Pathway: HSA00232 323
277.2 Number of Hits: 1 323
277.3 Legend: 323

278 Phenylalanine, tyrosine and tryptophan biosynthesis 324
278.1 Human Pathway: HSA00400 . . . 324
278.2 Number of Hits: 1 . . . 324
278.3 Legend: . . . 324

279 Malaria 325
279.1 Human Pathway: HSA05144 . . . 325
279.2 Number of Hits: 1 . . . 325
279.3 Legend: . . . 325

280 Type I diabetes mellitus 326
280.1 Human Pathway: HSA04940 . . . 326
280.2 Number of Hits: 1 . . . 326
280.3 Legend: . . . 326

281 Glycosaminoglycan biosynthesis - keratan sulfate 327
281.1 Human Pathway: HSA00533 . . . 327
281.2 Number of Hits: 1 . . . 327
281.3 Legend: . . . 327

282 Valine, leucine and isoleucine biosynthesis 328
282.1 Human Pathway: HSA00290 . . . 328
282.2 Number of Hits: 1 . . . 328
282.3 Legend: . . . 328

283 2-Oxocarboxylic acid metabolism 329
283.1 Human Pathway: HSA01210 . . . 329
283.2 Number of Hits: 1 . . . 329
283.3 Legend: . . . 329

284 D-Arginine and D-ornithine metabolism 330
284.1 Human Pathway: HSA00472 . . . 330
284.2 Number of Hits: 1 . . . 330
284.3 Legend: . . . 330

285 Butirosin and neomycin biosynthesis 331
285.1 Human Pathway: HSA00524 . . . 331
285.2 Number of Hits: 1 . . . 331
285.3 Legend: . . . 331

286 Biotin metabolism **332**
286.1 Human Pathway: HSA00780 332
286.2 Number of Hits: 1 332
286.3 Legend: 333

287 Thiamine metabolism **333**
287.1 Human Pathway: HSA00730 333
287.2 Number of Hits: 1 333
287.3 Legend: 333

288 Hematopoietic cell lineage **334**
288.1 Human Pathway: HSA04640 334
288.2 Number of Hits: 1 334
288.3 Legend: 335

289 Glycosphingolipid biosynthesis - globo series **336**
289.1 Human Pathway: HSA00603 336
289.2 Number of Hits: 1 336
289.3 Legend: 337

290 Linoleic acid metabolism **337**
290.1 Human Pathway: HSA00591 337
290.2 Number of Hits: 1 337
290.3 Legend: 337

291 Autoimmune thyroid disease **338**
291.1 Human Pathway: HSA05320 338
291.2 Number of Hits: 1 338
291.3 Legend: 338

292 Riboflavin metabolism **339**
292.1 Human Pathway: HSA00740 339
292.2 Number of Hits: 1 339
292.3 Legend: 339

1 Pathways in cancer

1.1 Human Pathway: HSA05200

1.2 Number of Hits: 99

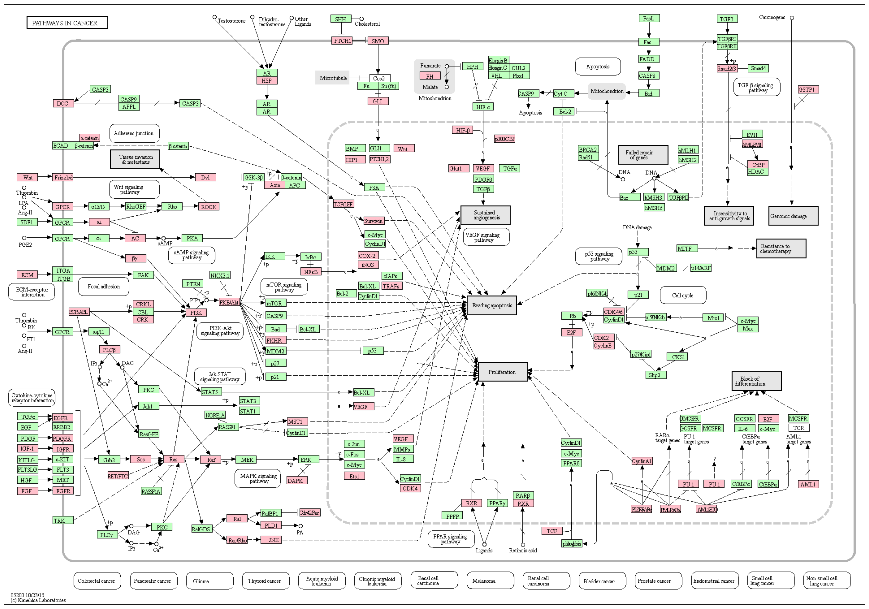

1.3 Legend:

RBH-Blast at 60% Identity + 50% Coverage
Green = Hit in *H. sapiens*
Red = Hit in *H. sapiens* and *C. milli*
White = Not in *H. sapiens*

2 Neuroactive ligand-receptor interaction

2.1 Human Pathway: HSA04080

2.2 Number of Hits: 72

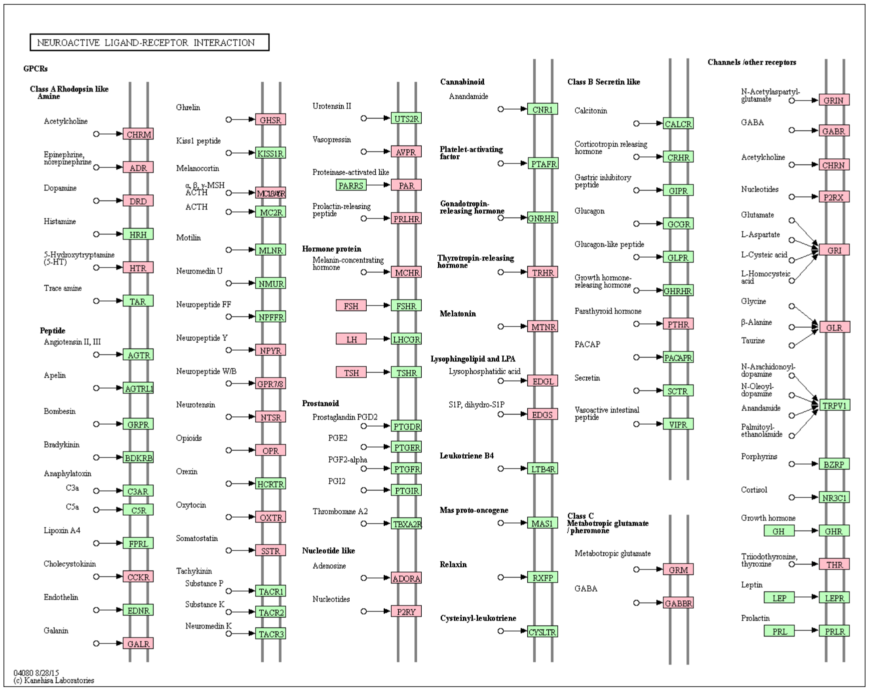

2.3 Legend:

RBH-Blast at 60% Identity + 50% Coverage
Green = Hit in *H. sapiens*
Red = Hit in *H. sapiens* and *C. milli*
White = Not in *H. sapiens*

3 cAMP signaling pathway

3.1 Human Pathway: HSA04024

3.2 Number of Hits: 63

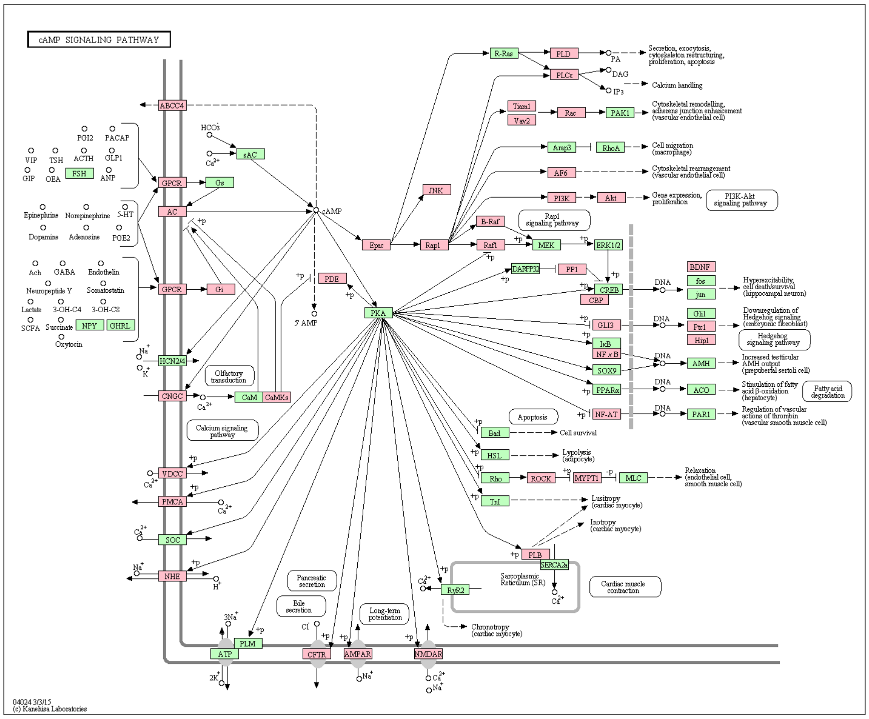

3.3 Legend:

RBH-Blast at 60% Identity + 50% Coverage
Green = Hit in *H. sapiens*
Red = Hit in *H. sapiens* and *C. milli*
White = Not in *H. sapiens*

4 HTLV-I infection

4.1 Human Pathway: HSA05166

4.2 Number of Hits: 61

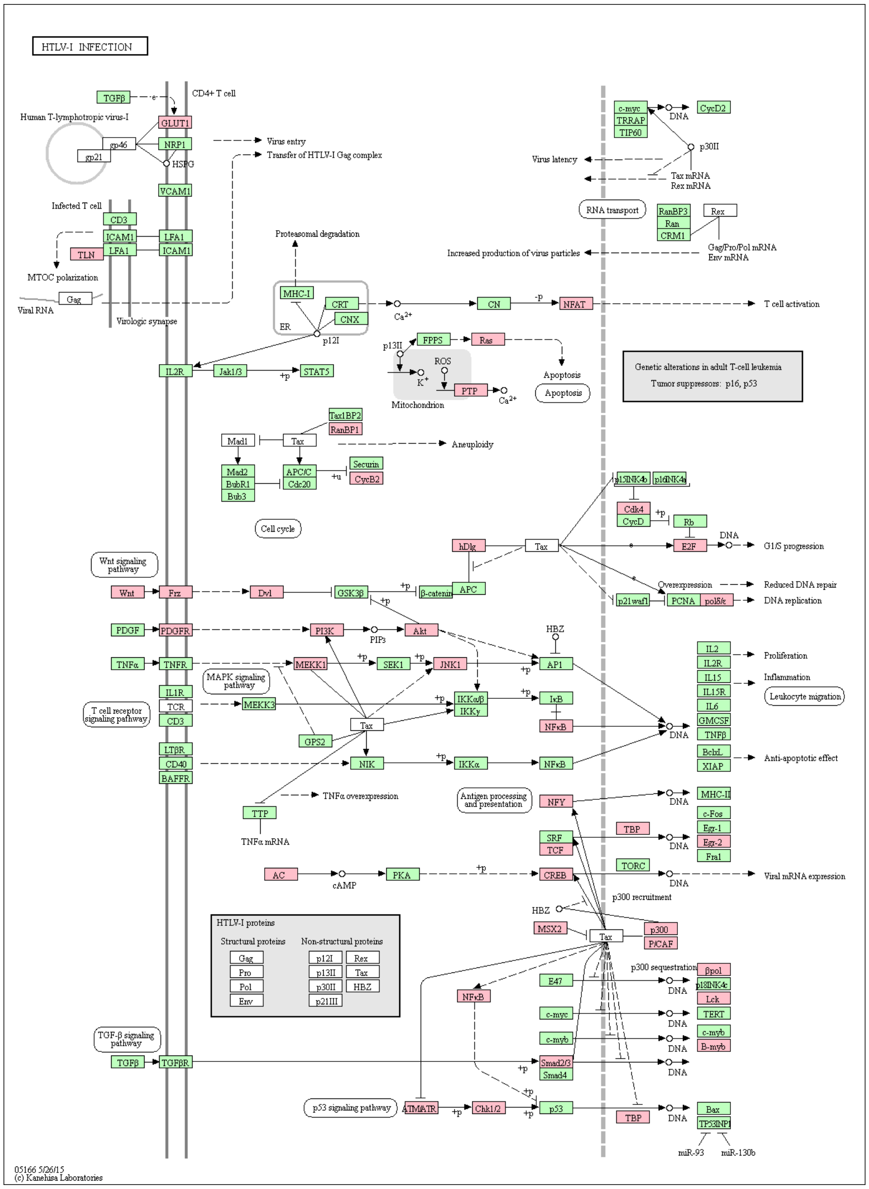

4.3 Legend:

RBH-Blast at 60% Identity + 50% Coverage
Green = Hit in *H. sapiens*
Red = Hit in *H. sapiens* and *C. milli*
White = Not in *H. sapiens*

---

5 PI3K-Akt signaling pathway

5.1 Human Pathway: HSA04151

5.2 Number of Hits: 58

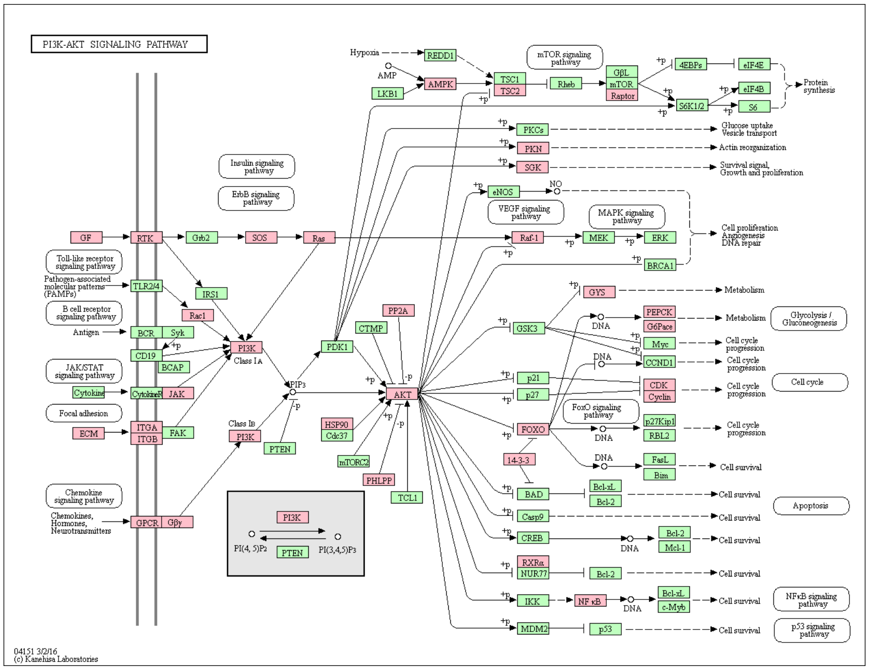

5.3 Legend:

RBH-Blast at 60% Identity + 50% Coverage
Green = Hit in *H. sapiens*
Red = Hit in *H. sapiens* and *C. milli*
White = Not in *H. sapiens*

---

6 Ras signaling pathway

6.1 Human Pathway: HSA04014

6.2 Number of Hits: 57

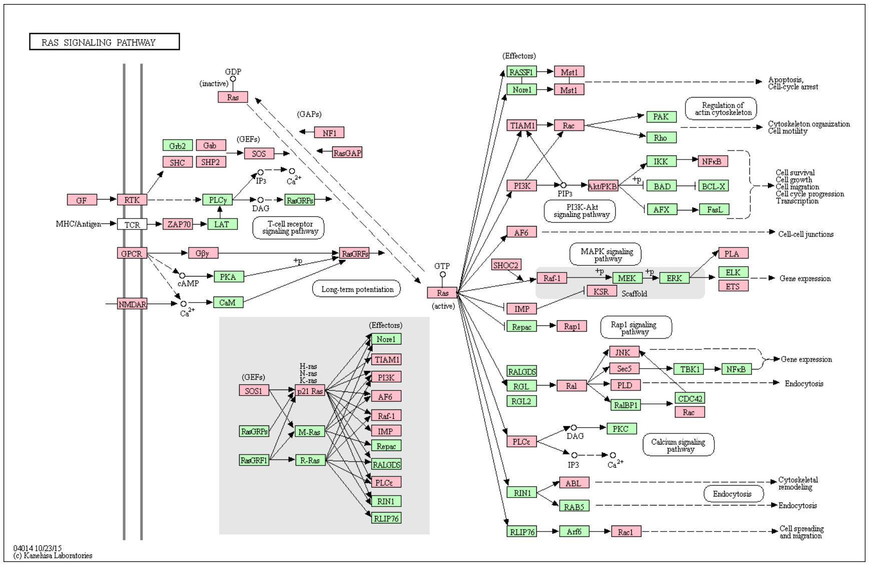

6.3 Legend:

RBH-Blast at 60% Identity + 50% Coverage
Green = Hit in *H. sapiens*
Red = Hit in *H. sapiens* and *C. milli*
White = Not in *H. sapiens*

7 Rap1 signaling pathway

7.1 Human Pathway: HSA04015

7.2 Number of Hits: 55

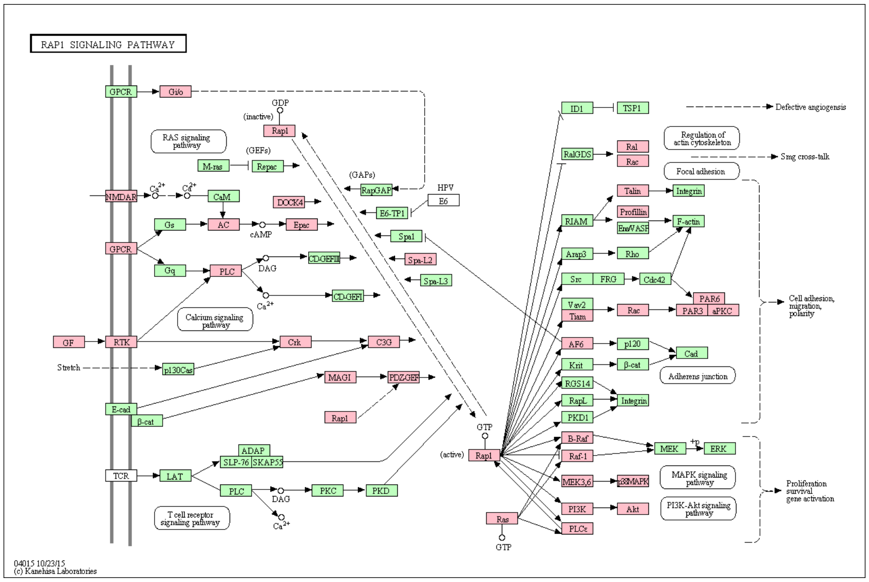

7.3 Legend:

RBH-Blast at 60% Identity + 50% Coverage
Green = Hit in *H. sapiens*
Red = Hit in *H. sapiens* and *C. milli*
White = Not in *H. sapiens*

8 Proteoglycans in cancer

8.1 Human Pathway: HSA05205

8.2 Number of Hits: 54

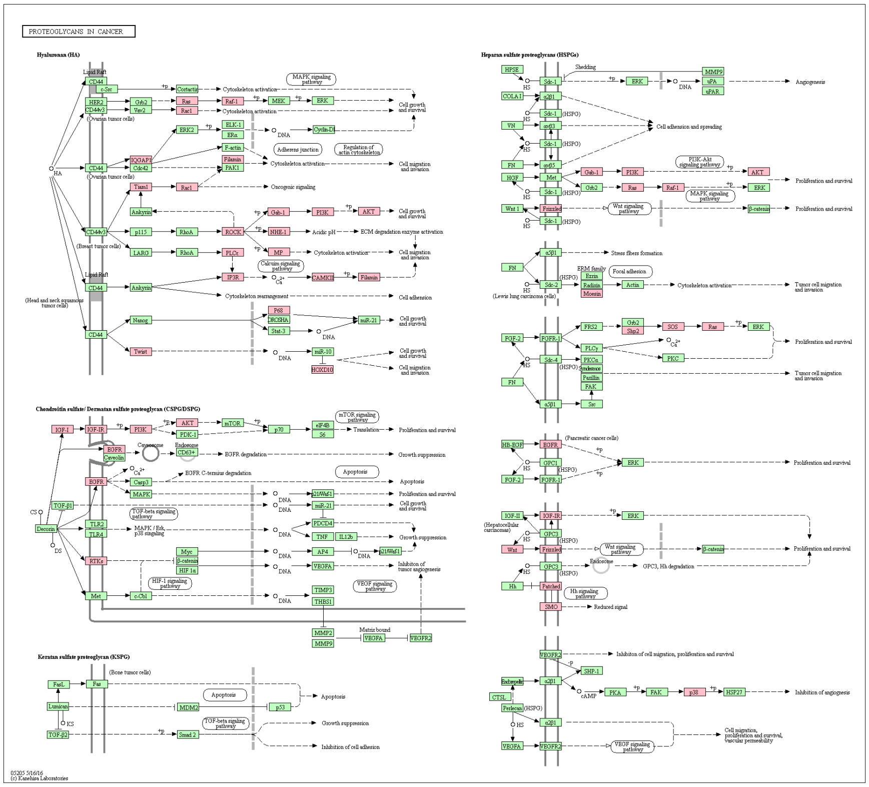

8.3 Legend:

RBH-Blast at 60% Identity + 50% Coverage
Green = Hit in *H. sapiens*
Red = Hit in *H. sapiens* and *C. milli*
White = Not in *H. sapiens*

9 Axon guidance

9.1 Human Pathway: HSA04360

9.2 Number of Hits: 53

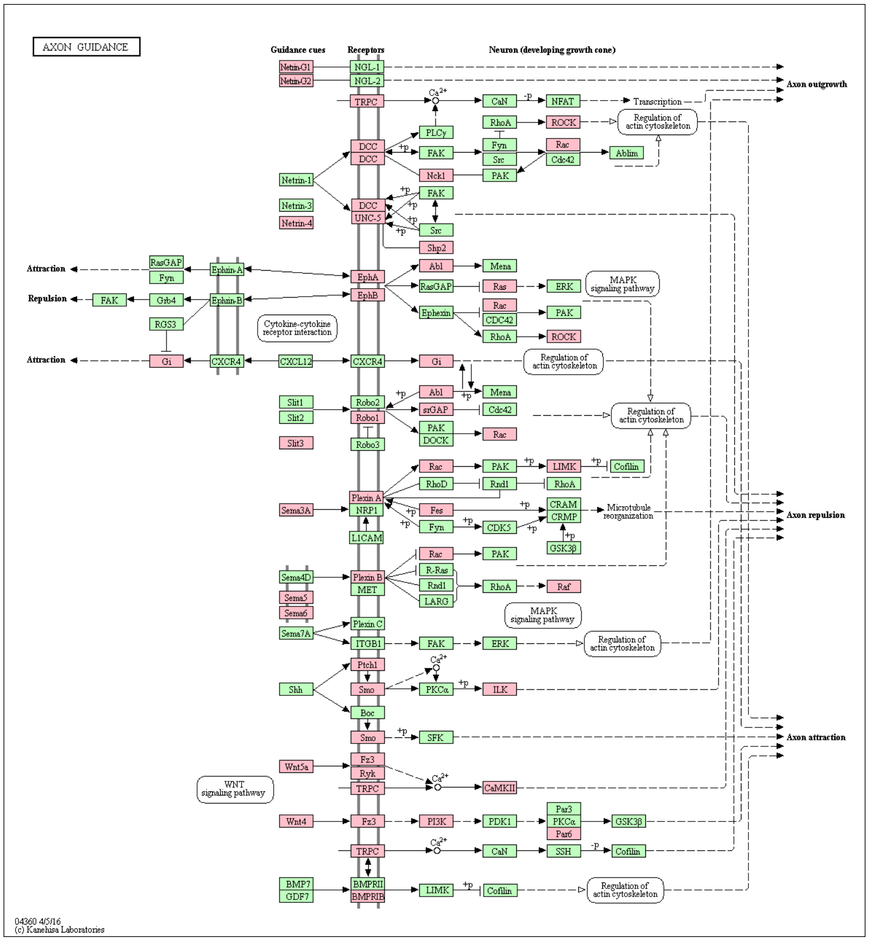

9.3 Legend:

RBH-Blast at 60% Identity + 50% Coverage
Green = Hit in *H. sapiens*
Red = Hit in *H. sapiens* and *C. milli*
White = Not in *H. sapiens*

10 MAPK signaling pathway

10.1 Human Pathway: HSA04010

10.2 Number of Hits: 52

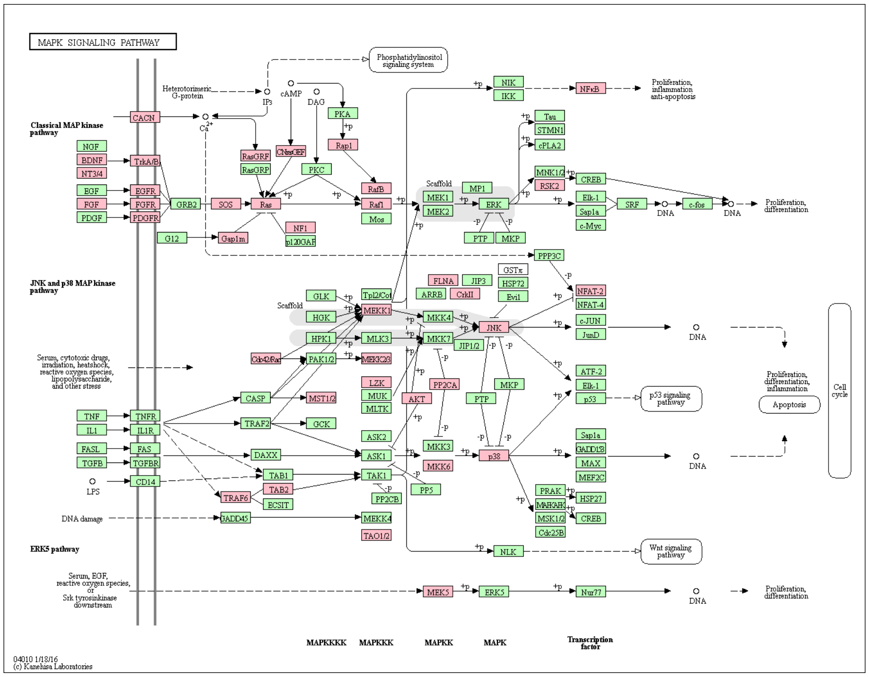

10.3 Legend:

RBH-Blast at 60% Identity + 50% Coverage
Green = Hit in *H. sapiens*
Red = Hit in *H. sapiens* and *C. milli*
White = Not in *H. sapiens*

11 Calcium signaling pathway

11.1 Human Pathway: HSA04020

11.2 Number of Hits: 51

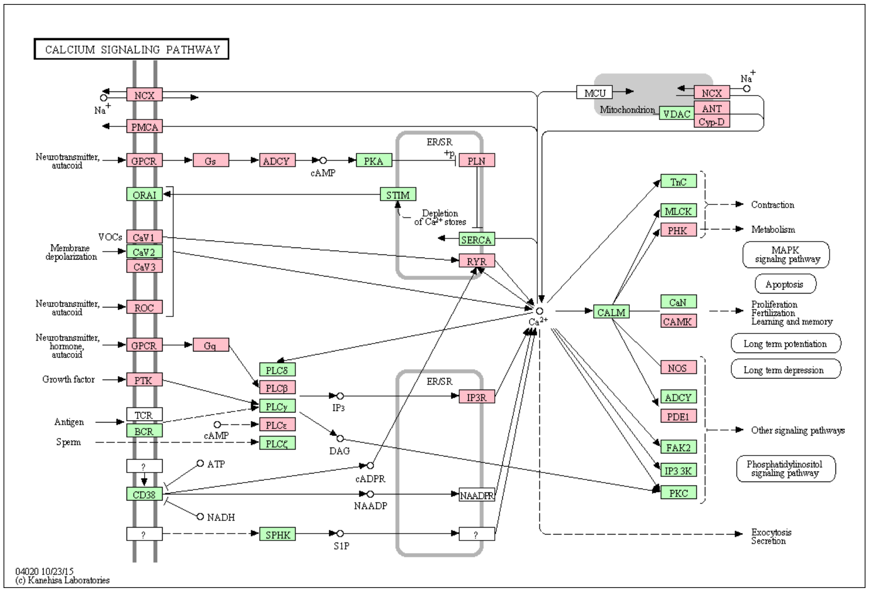

11.3 Legend:

RBH-Blast at 60% Identity + 50% Coverage
Green = Hit in *H. sapiens*
Red = Hit in *H. sapiens* and *C. milli*
White = Not in *H. sapiens*

12 Signaling pathways regulating pluripotency of stem cells

12.1 Human Pathway: HSA04550

12.2 Number of Hits: 48

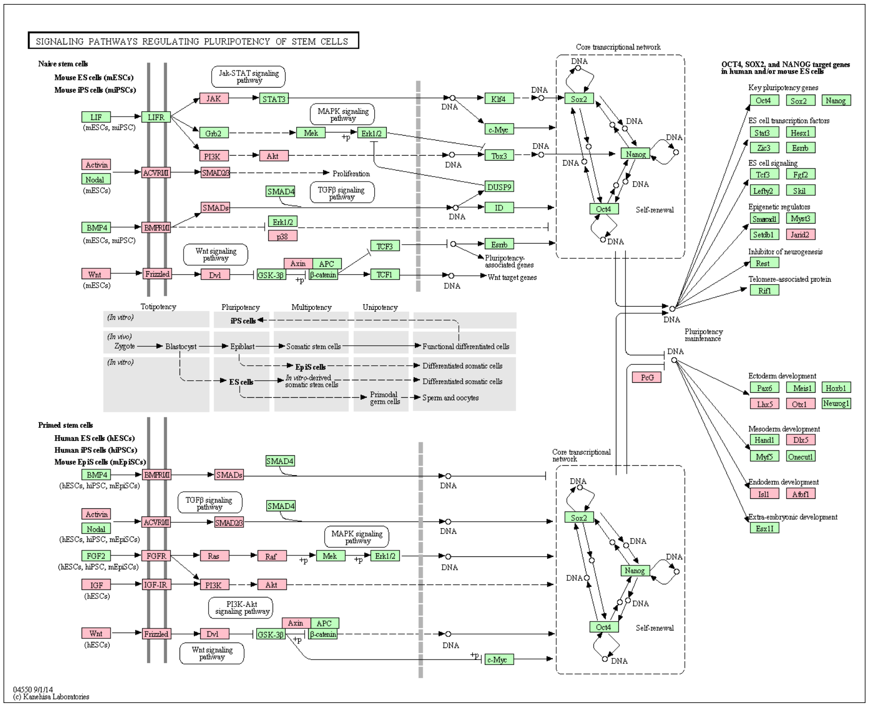

12.3 Legend:

RBH-Blast at 60% Identity + 50% Coverage
Green = Hit in *H. sapiens*
Red = Hit in *H. sapiens* and *C. milli*
White = Not in *H. sapiens*

13 mTOR signaling pathway

13.1 Human Pathway: HSA04150

13.2 Number of Hits: 47

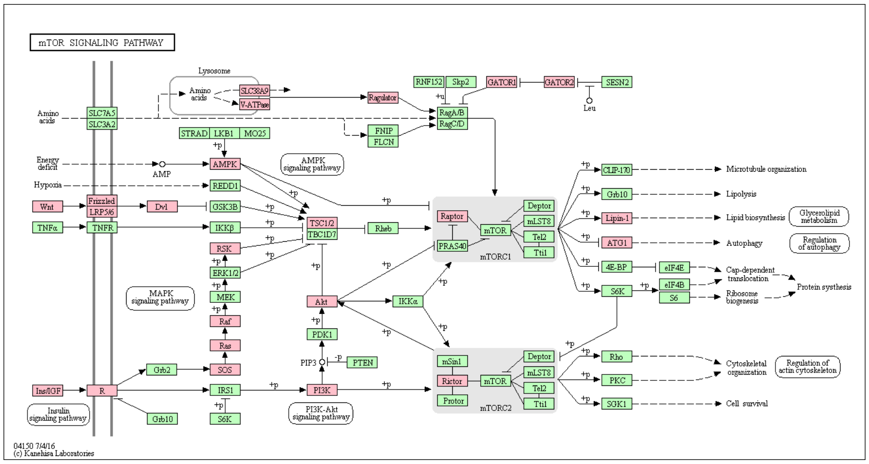

13.3 Legend:

RBH-Blast at 60% Identity + 50% Coverage
Green = Hit in *H. sapiens*
Red = Hit in *H. sapiens* and *C. milli*
White = Not in *H. sapiens*

14 Hippo signaling pathway

14.1 Human Pathway: HSA04390

14.2 Number of Hits: 46

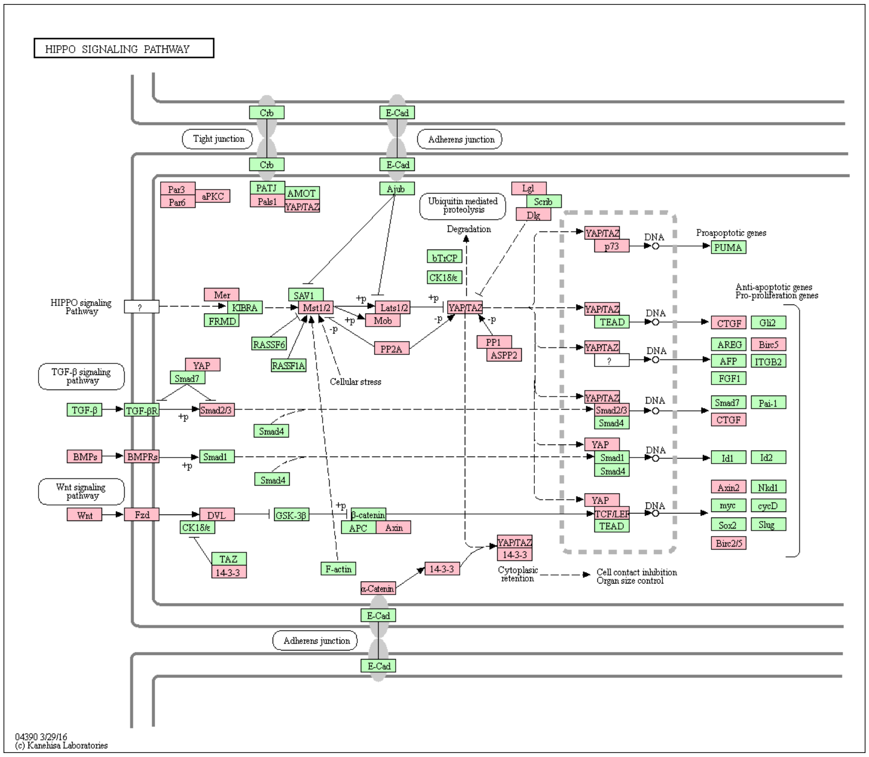

14.3 Legend:

RBH-Blast at 60% Identity + 50% Coverage
Green = Hit in *H. sapiens*
Red = Hit in *H. sapiens* and *C. milli*
White = Not in *H. sapiens*

15 Regulation of actin cytoskeleton

15.1 Human Pathway: HSA04810

15.2 Number of Hits: 46

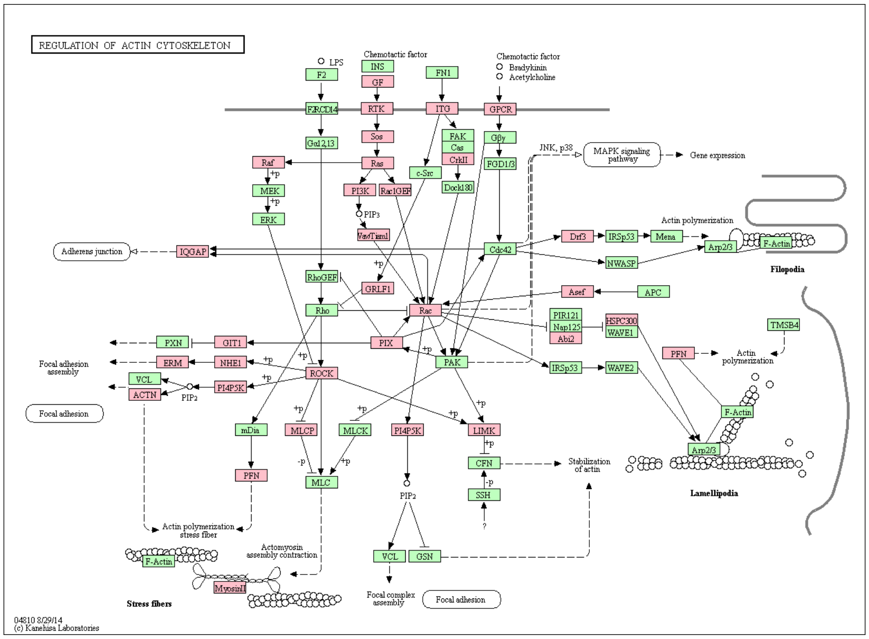

15.3 Legend:

RBH-Blast at 60% Identity + 50% Coverage
Green = Hit in *H. sapiens*
Red = Hit in *H. sapiens* and *C. milli*
White = Not in *H. sapiens*

16 Wnt signaling pathway

16.1 Human Pathway: HSA04310

16.2 Number of Hits: 43

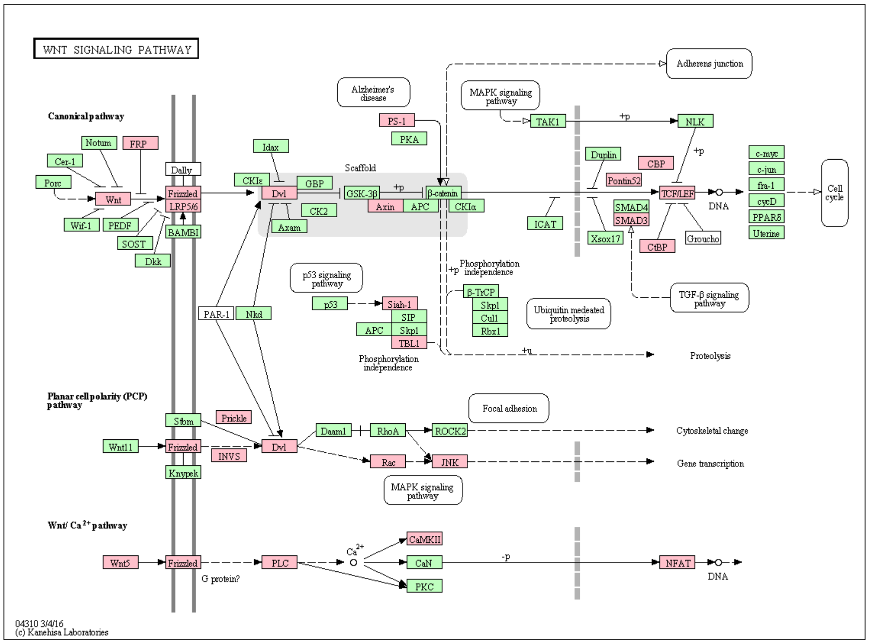

16.3 Legend:

RBH-Blast at 60% Identity + 50% Coverage
Green = Hit in *H. sapiens*
Red = Hit in *H. sapiens* and *C. milli*
White = Not in *H. sapiens*

17 Oxytocin signaling pathway

17.1 Human Pathway: HSA04921

17.2 Number of Hits: 40

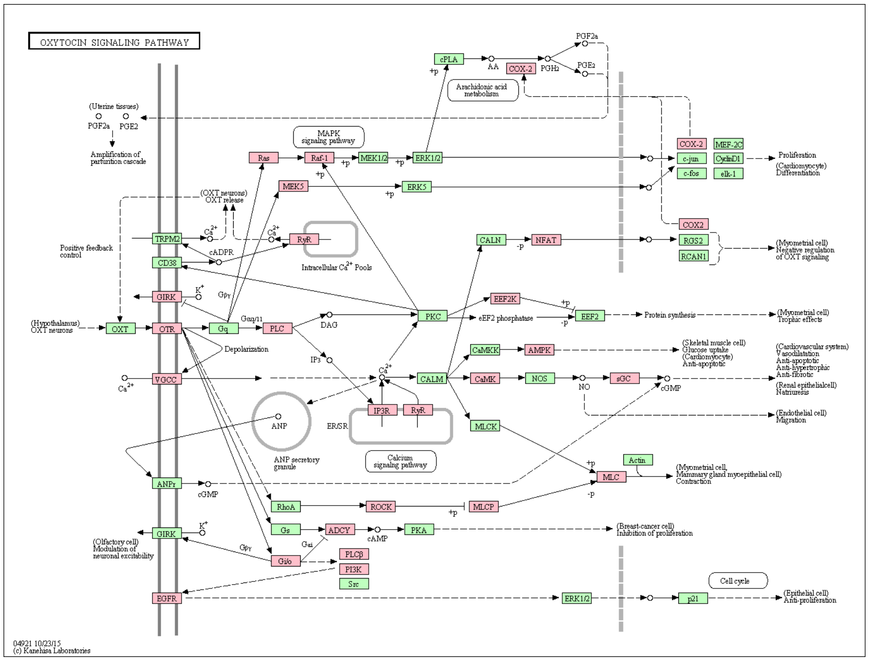

17.3 Legend:

RBH-Blast at 60% Identity + 50% Coverage
Green = Hit in *H. sapiens*
Red = Hit in *H. sapiens* and *C. milli*
White = Not in *H. sapiens*

18 Focal adhesion

18.1 Human Pathway: HSA04510

18.2 Number of Hits: 40

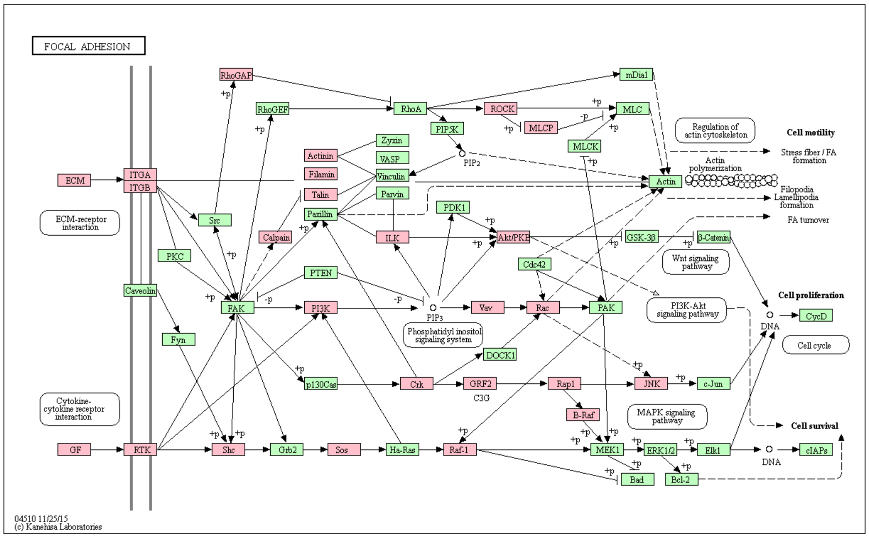

18.3 Legend:

RBH-Blast at 60% Identity + 50% Coverage
Green = Hit in *H. sapiens*
Red = Hit in *H. sapiens* and *C. milli*
White = Not in *H. sapiens*

19 MicroRNAs in cancer

19.1 Human Pathway: HSA05206

19.2 Number of Hits: 39

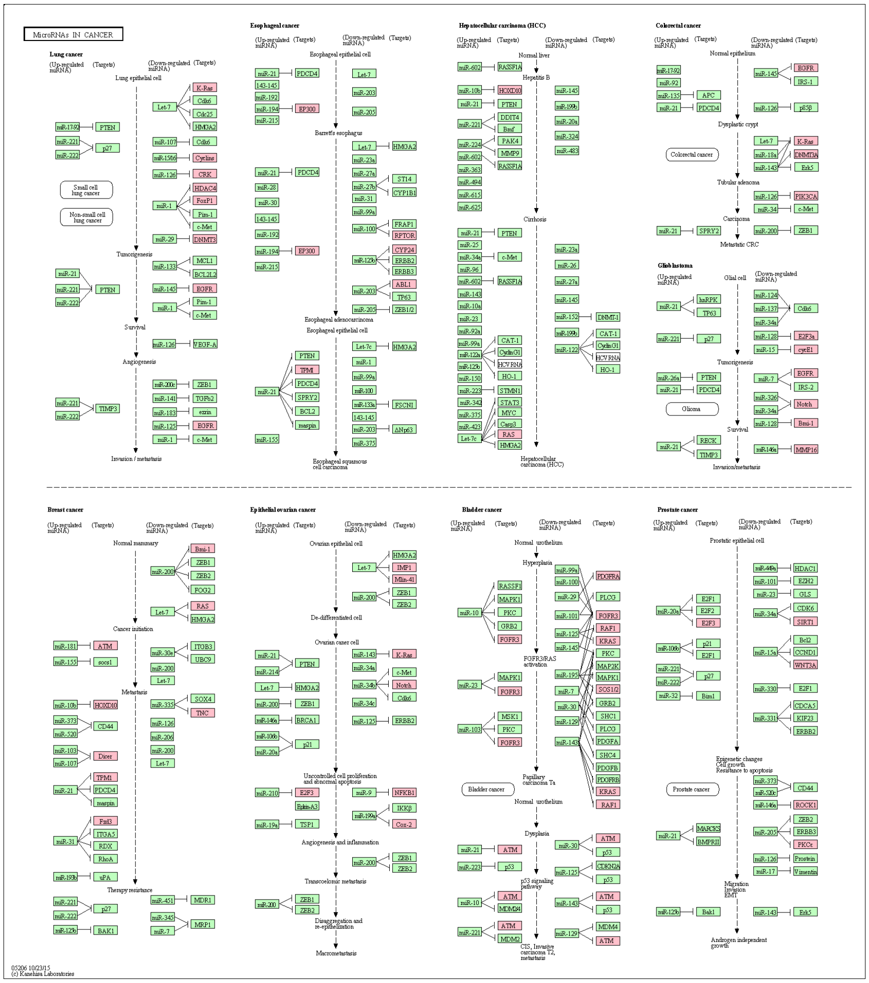

19.3 Legend:

RBH-Blast at 60% Identity + 50% Coverage
Green = Hit in *H. sapiens*
Red = Hit in *H. sapiens* and *C. milli*
White = Not in *H. sapiens*

20 Purine metabolism

20.1 Human Pathway: HSA00230

20.2 Number of Hits: 39

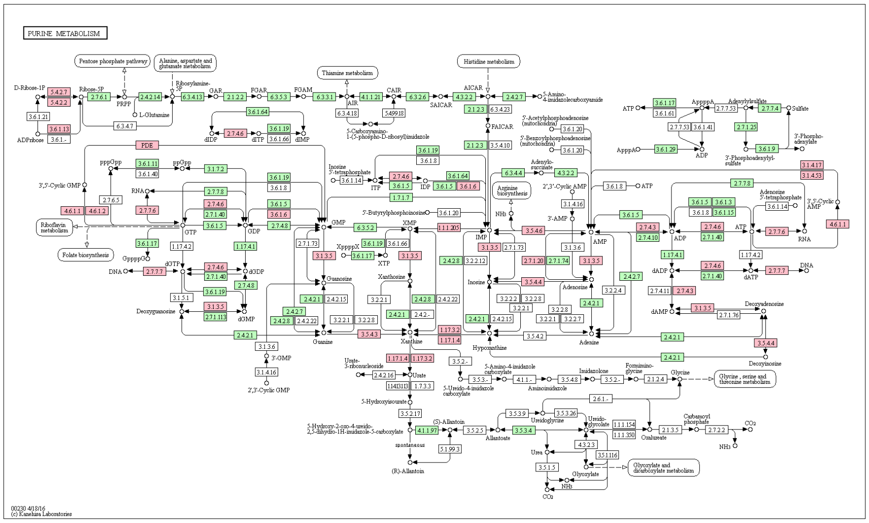

20.3 Legend:

RBH-Blast at 60% Identity + 50% Coverage
Green = Hit in *H. sapiens*
Red = Hit in *H. sapiens* and *C. milli*
White = Not in *H. sapiens*

21 Endocytosis

21.1 Human Pathway: HSA04144

21.2 Number of Hits: 39

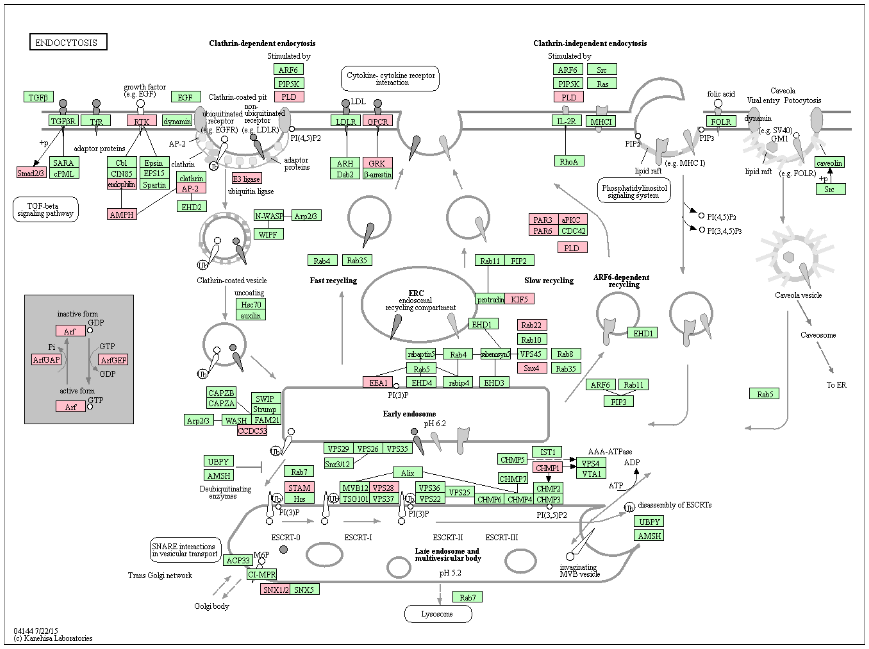

21.3 Legend:

RBH-Blast at 60% Identity + 50% Coverage
Green = Hit in *H. sapiens*
Red = Hit in *H. sapiens* and *C. milli*
White = Not in *H. sapiens*

22 Huntington's disease

22.1 Human Pathway: HSA05016

22.2 Number of Hits: 38

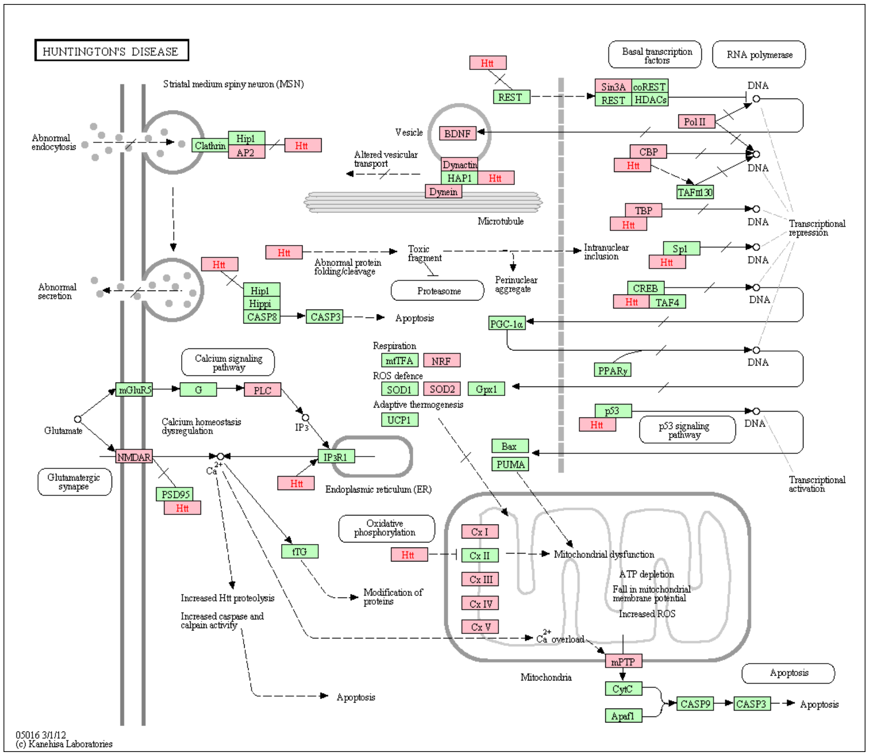

22.3 Legend:

RBH-Blast at 60% Identity + 50% Coverage
Green = Hit in *H. sapiens*
Red = Hit in *H. sapiens* and *C. milli*
White = Not in *H. sapiens*

23 cGMP-PKG signaling pathway

23.1 Human Pathway: HSA04022

23.2 Number of Hits: 38

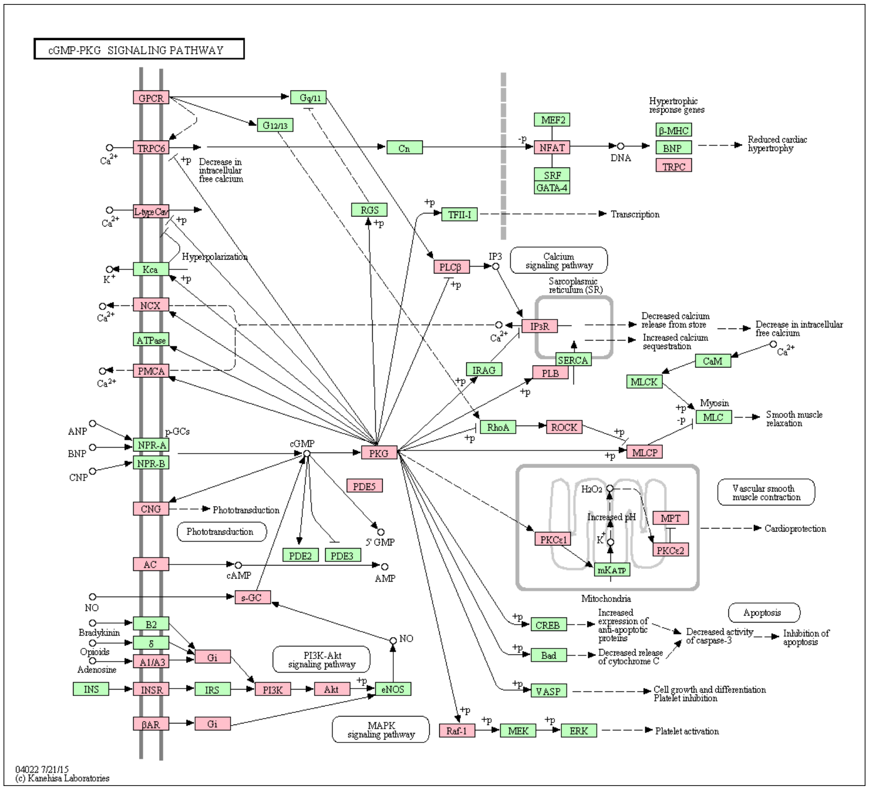

23.3 Legend:

RBH-Blast at 60% Identity + 50% Coverage
Green = Hit in *H. sapiens*
Red = Hit in *H. sapiens* and *C. milli*
White = Not in *H. sapiens*

24 FoxO signaling pathway

24.1 Human Pathway: HSA04068

24.2 Number of Hits: 37

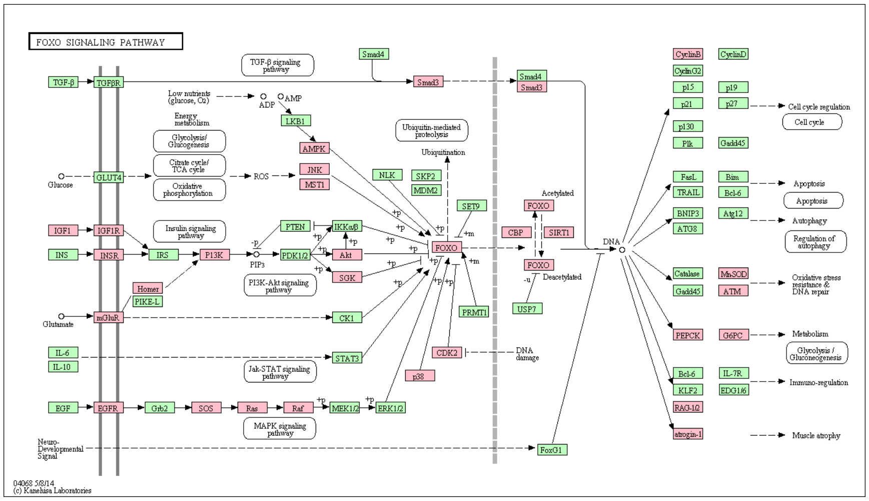

24.3 Legend:

RBH-Blast at 60% Identity + 50% Coverage
Green = Hit in *H. sapiens*
Red = Hit in *H. sapiens* and *C. milli*
White = Not in *H. sapiens*

25 Insulin signaling pathway

25.1 Human Pathway: HSA04910

25.2 Number of Hits: 35

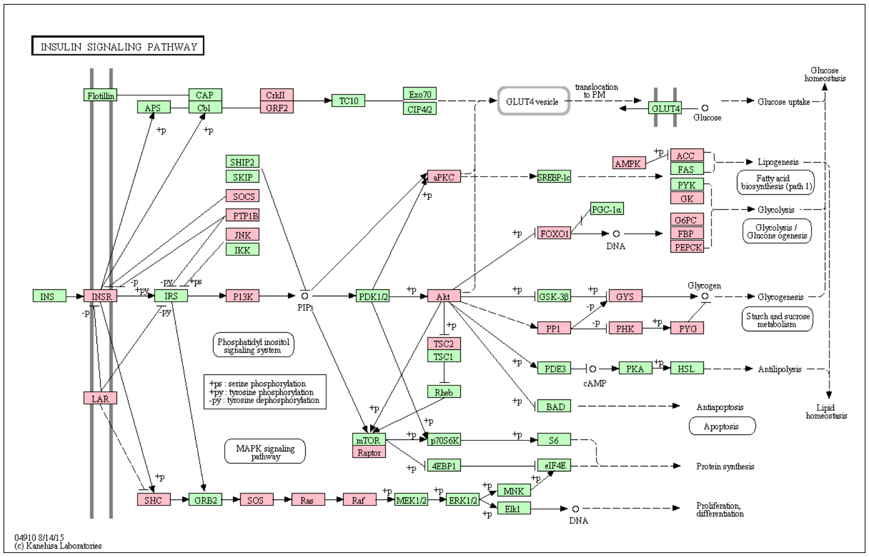

25.3 Legend:

RBH-Blast at 60% Identity + 50% Coverage
Green = Hit in *H. sapiens*
Red = Hit in *H. sapiens* and *C. milli*
White = Not in *H. sapiens*

26 Viral carcinogenesis

26.1 Human Pathway: HSA05203

26.2 Number of Hits: 34

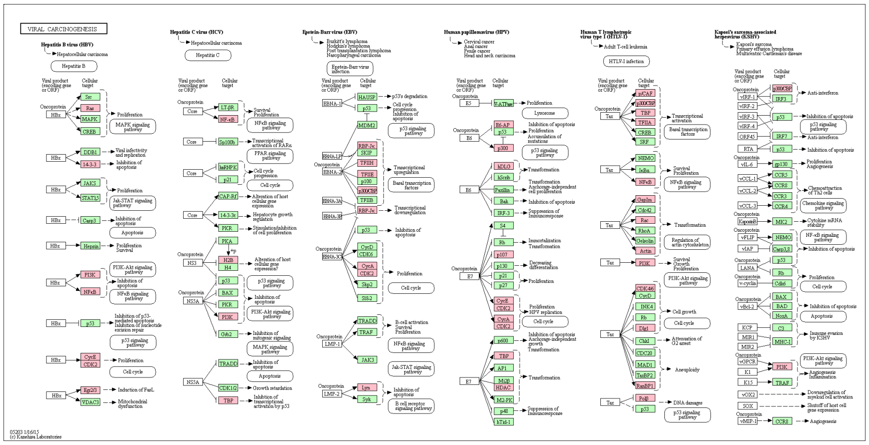

26.3 Legend:

RBH-Blast at 60% Identity + 50% Coverage
Green = Hit in *H. sapiens*
Red = Hit in *H. sapiens* and *C. milli*
White = Not in *H. sapiens*

27 Phospholipase D signaling pathway

27.1 Human Pathway: HSA04072

27.2 Number of Hits: 34

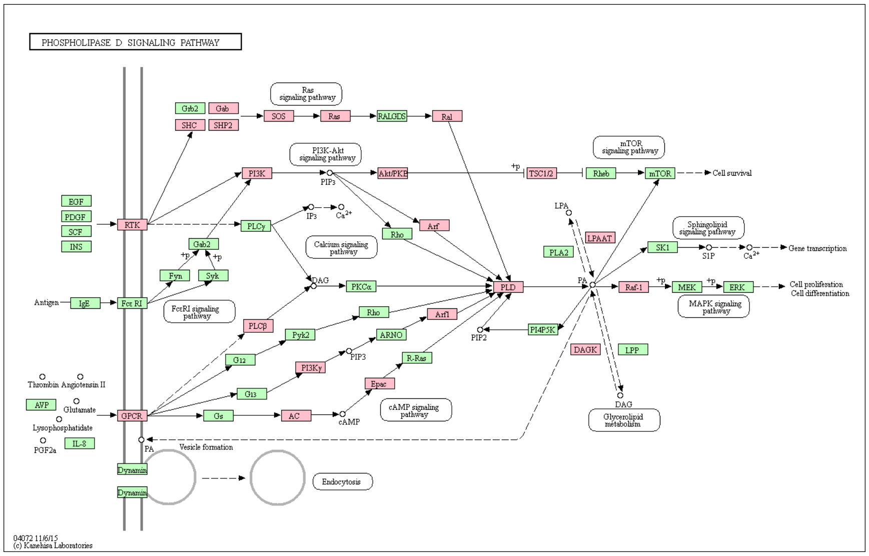

27.3 Legend:

RBH-Blast at 60% Identity + 50% Coverage
Green = Hit in *H. sapiens*
Red = Hit in *H. sapiens* and *C. milli*
White = Not in *H. sapiens*

28 Alzheimer's disease

28.1 Human Pathway: HSA05010

28.2 Number of Hits: 34

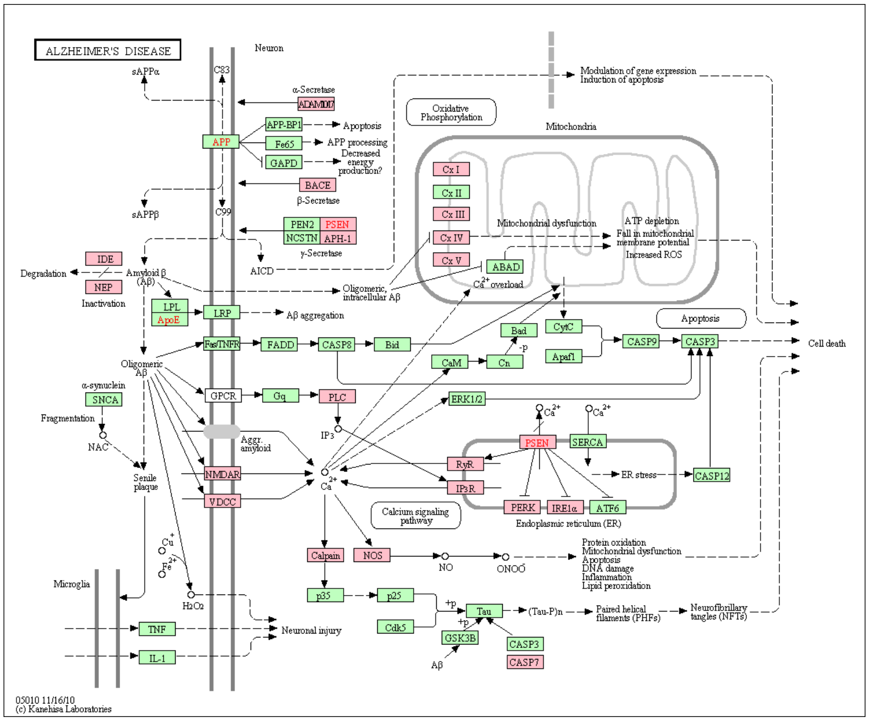

28.3 Legend:

RBH-Blast at 60% Identity + 50% Coverage
Green = Hit in *H. sapiens*
Red = Hit in *H. sapiens* and *C. milli*
White = Not in *H. sapiens*

29 Thyroid hormone signaling pathway

29.1 Human Pathway: HSA04919

29.2 Number of Hits: 34

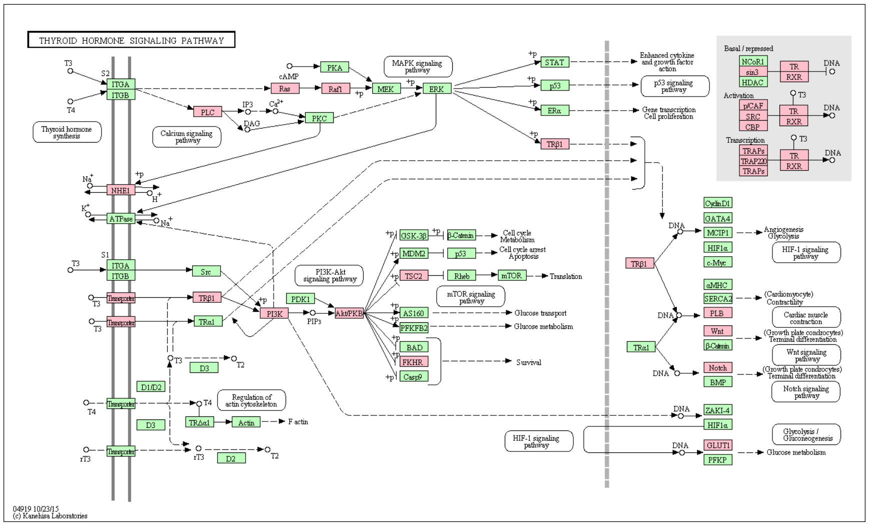

29.3 Legend:

RBH-Blast at 60% Identity + 50% Coverage
Green = Hit in *H. sapiens*
Red = Hit in *H. sapiens* and *C. milli*
White = Not in *H. sapiens*

30 Chemokine signaling pathway

30.1 Human Pathway: HSA04062

30.2 Number of Hits: 34

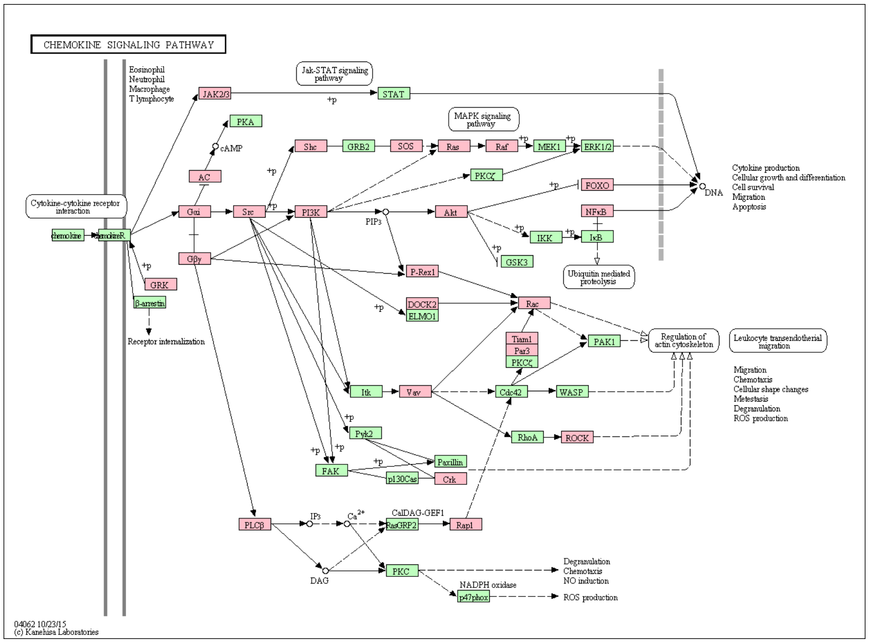

30.3 Legend:

RBH-Blast at 60% Identity + 50% Coverage
Green = Hit in *H. sapiens*
Red = Hit in *H. sapiens* and *C. milli*
White = Not in *H. sapiens*

31 Sphingolipid signaling pathway

31.1 Human Pathway: HSA04071

31.2 Number of Hits: 34

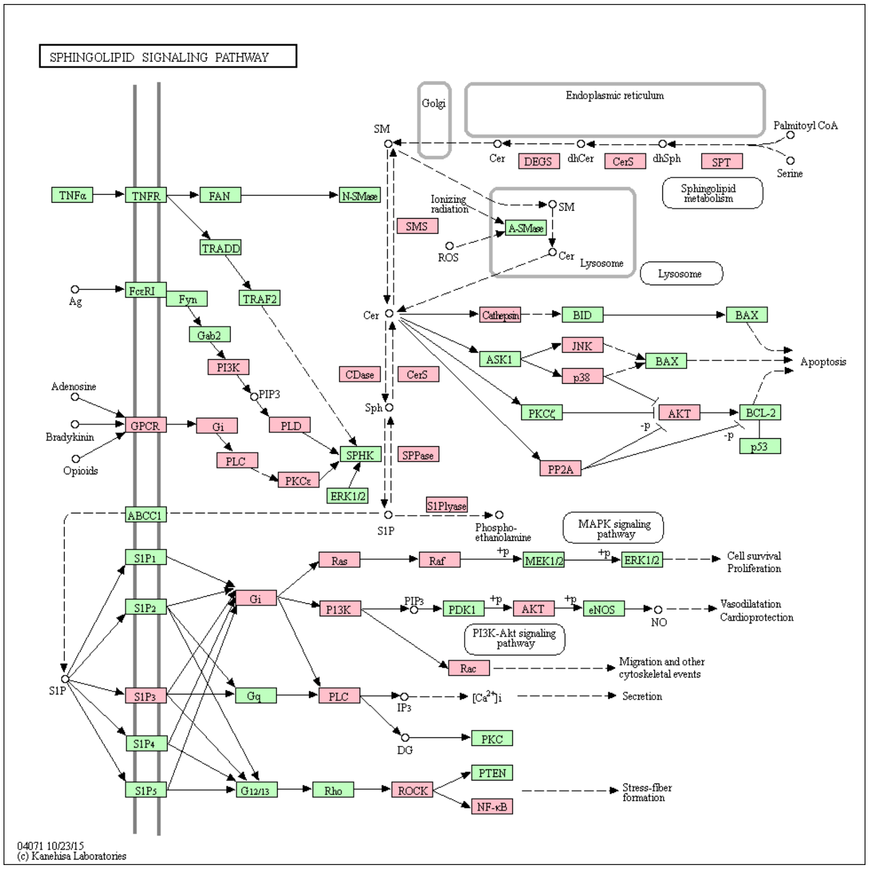

31.3 Legend:

RBH-Blast at 60% Identity + 50% Coverage
Green = Hit in *H. sapiens*
Red = Hit in *H. sapiens* and *C. milli*
White = Not in *H. sapiens*

32 Adrenergic signaling in cardiomyocytes

32.1 Human Pathway: HSA04261

32.2 Number of Hits: 34

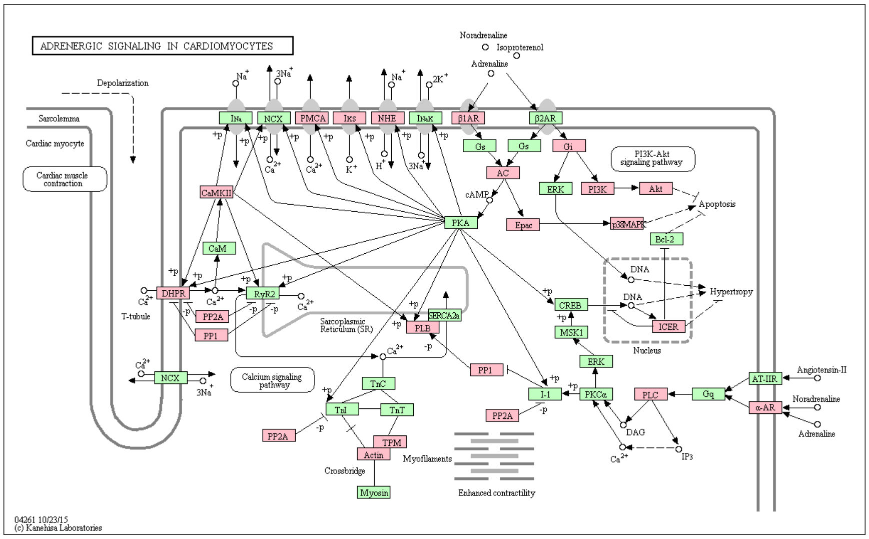

32.3 Legend:

RBH-Blast at 60% Identity + 50% Coverage
Green = Hit in *H. sapiens*
Red = Hit in *H. sapiens* and *C. milli*
White = Not in *H. sapiens*

33 AMPK signaling pathway

33.1 Human Pathway: HSA04152

33.2 Number of Hits: 33

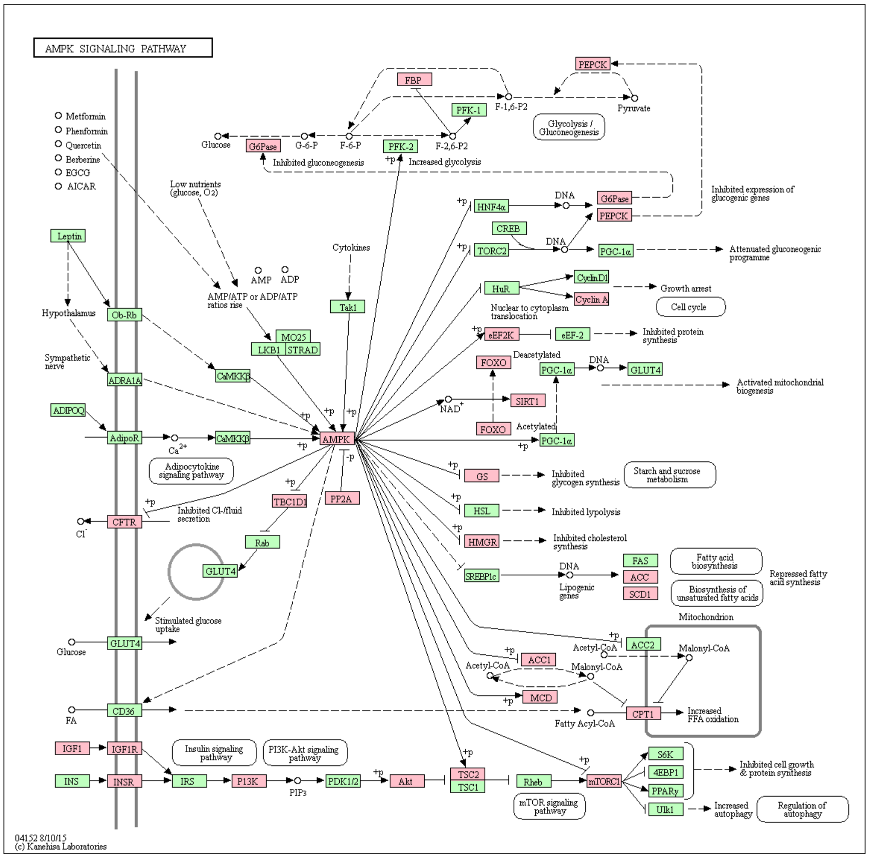

33.3 Legend:

RBH-Blast at 60% Identity + 50% Coverage
Green = Hit in *H. sapiens*
Red = Hit in *H. sapiens* and *C. milli*
White = Not in *H. sapiens*

34 Neurotrophin signaling pathway

34.1 Human Pathway: HSA04722

34.2 Number of Hits: 33

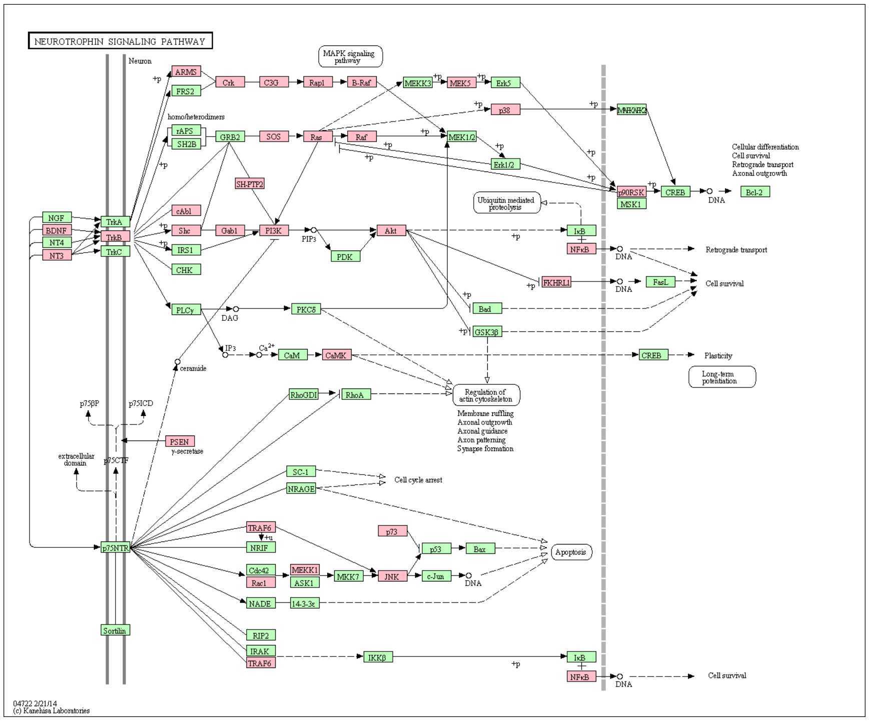

34.3 Legend:

RBH-Blast at 60% Identity + 50% Coverage
Green = Hit in *H. sapiens*
Red = Hit in *H. sapiens* and *C. milli*
White = Not in *H. sapiens*

35 Melanogenesis

35.1 Human Pathway: HSA04916

35.2 Number of Hits: 32

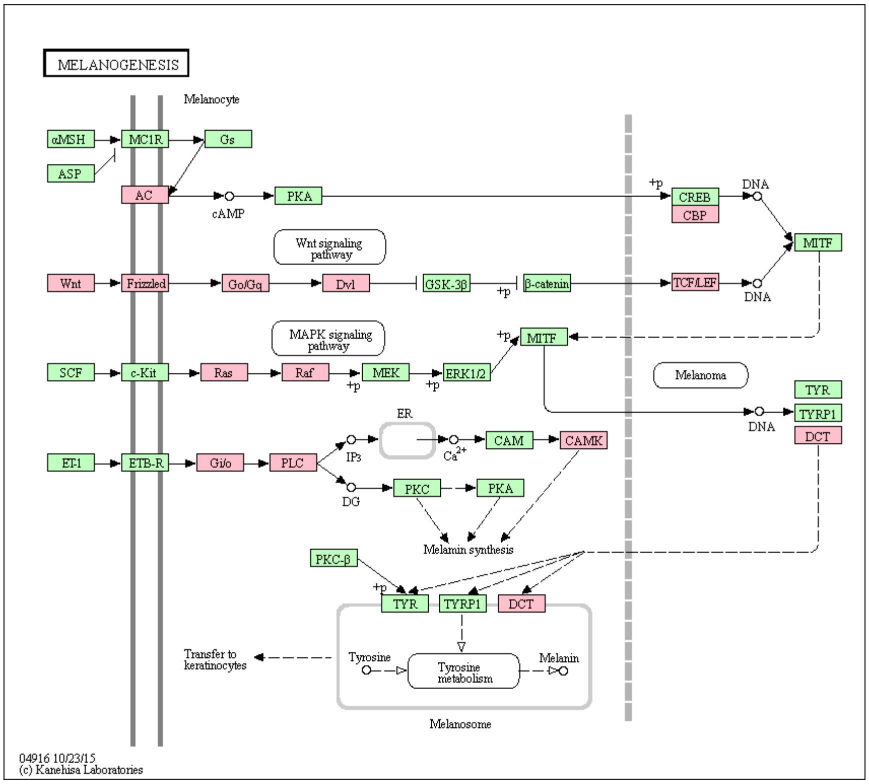

35.3 Legend:

RBH-Blast at 60% Identity + 50% Coverage
Green = Hit in *H. sapiens*
Red = Hit in *H. sapiens* and *C. milli*
White = Not in *H. sapiens*

36 Cholinergic synapse

36.1 Human Pathway: HSA04725

36.2 Number of Hits: 32

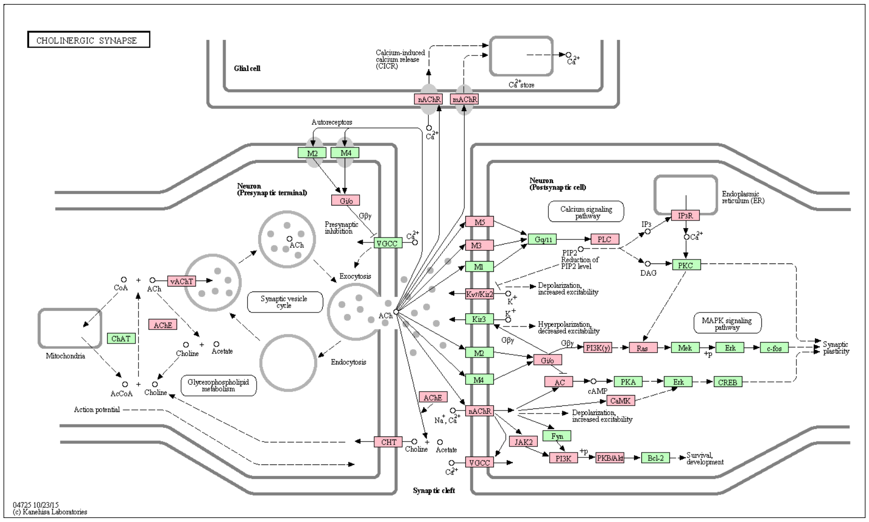

36.3 Legend:

RBH-Blast at 60% Identity + 50% Coverage
Green = Hit in *H. sapiens*
Red = Hit in *H. sapiens* and *C. milli*
White = Not in *H. sapiens*

37 Transcriptional misregulation in cancer

37.1 Human Pathway: HSA05202

37.2 Number of Hits: 30

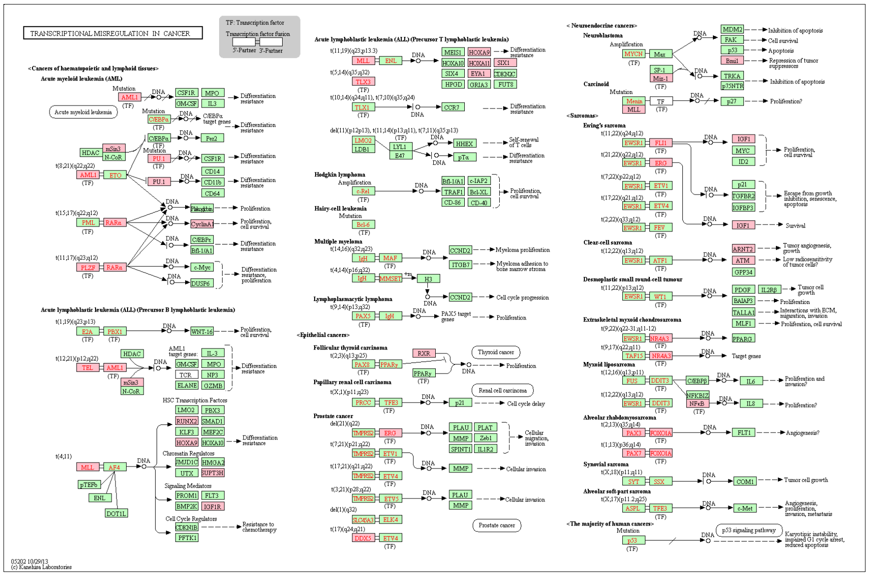

37.3 Legend:

RBH-Blast at 60% Identity + 50% Coverage
Green = Hit in *H. sapiens*
Red = Hit in *H. sapiens* and *C. milli*
White = Not in *H. sapiens*

38 Dopaminergic synapse

38.1 Human Pathway: HSA04728

38.2 Number of Hits: 30

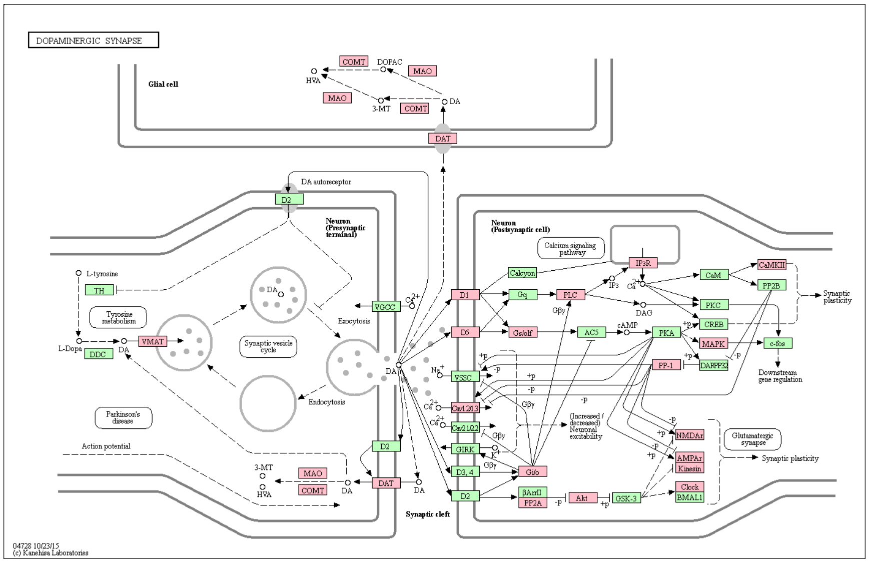

38.3 Legend:

RBH-Blast at 60% Identity + 50% Coverage
Green = Hit in *H. sapiens*
Red = Hit in *H. sapiens* and *C. milli*
White = Not in *H. sapiens*

39 Serotonergic synapse

39.1 Human Pathway: HSA04726

39.2 Number of Hits: 28

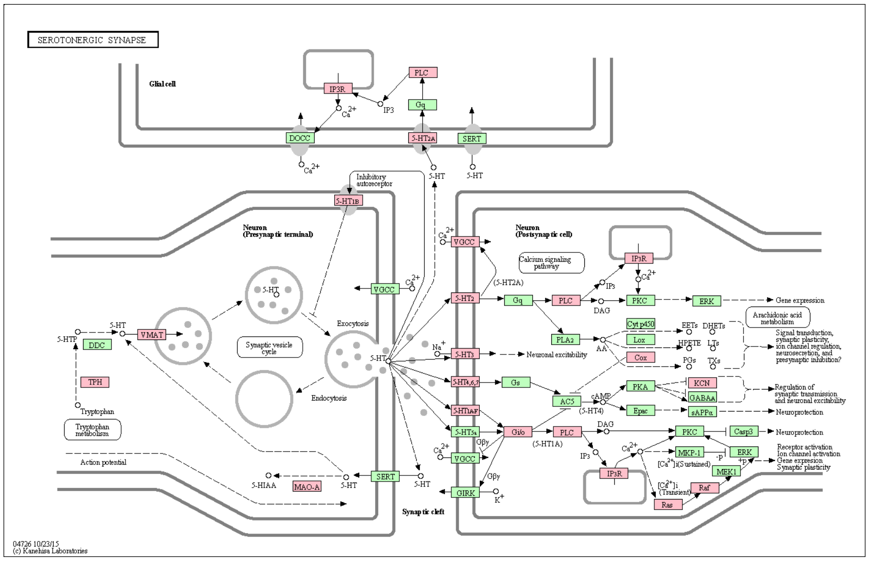

39.3 Legend:

RBH-Blast at 60% Identity + 50% Coverage
Green = Hit in *H. sapiens*
Red = Hit in *H. sapiens* and *C. milli*
White = Not in *H. sapiens*

40 Epstein-Barr virus infection

40.1 Human Pathway: HSA05169

40.2 Number of Hits: 28

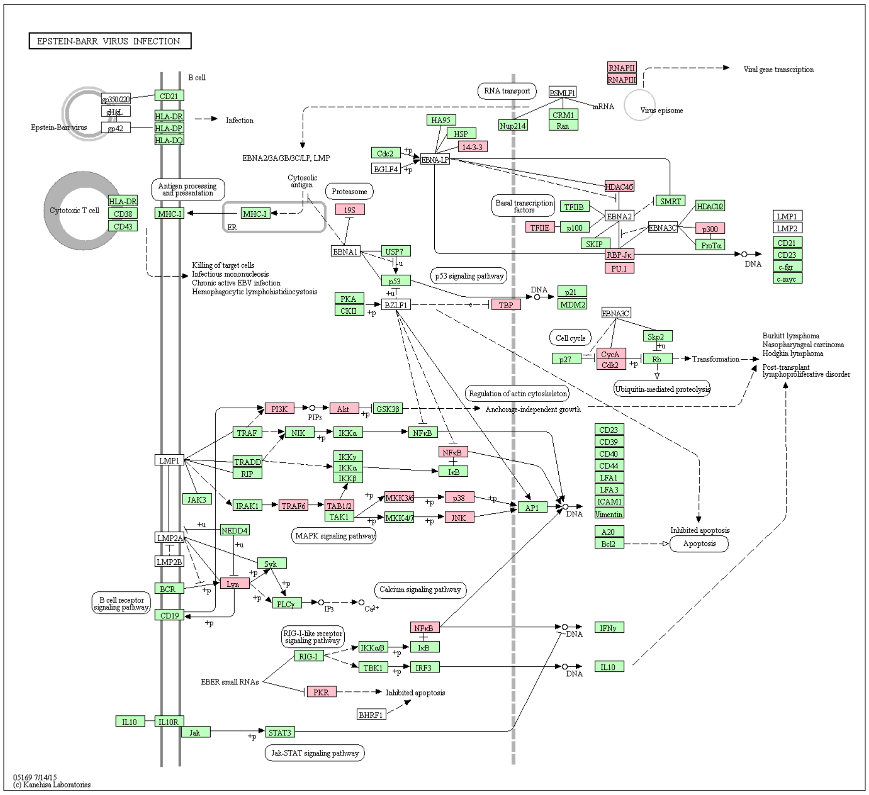

40.3 Legend:

RBH-Blast at 60% Identity + 50% Coverage
Green = Hit in *H. sapiens*
Red = Hit in *H. sapiens* and *C. milli*
White = Not in *H. sapiens*

41 Retrograde endocannabinoid signaling

41.1 Human Pathway: HSA04723

41.2 Number of Hits: 28

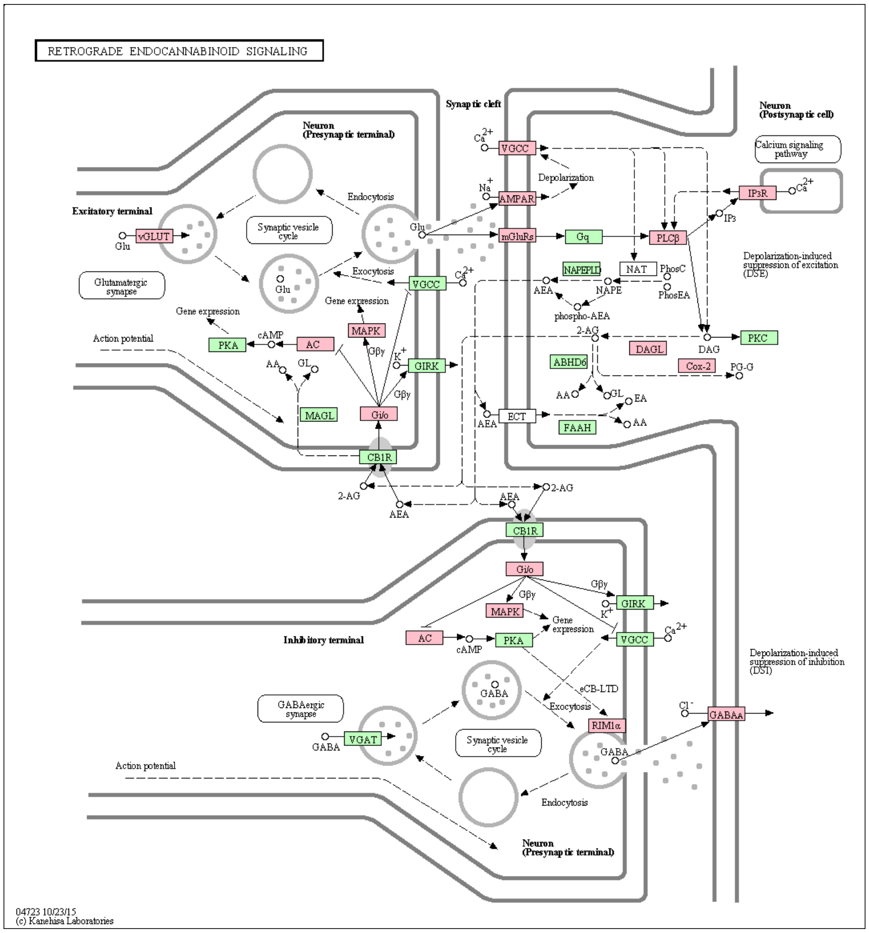

41.3 Legend:

RBH-Blast at 60% Identity + 50% Coverage
Green = Hit in *H. sapiens*
Red = Hit in *H. sapiens* and *C. milli*
White = Not in *H. sapiens*

42 Apoptosis

42.1 Human Pathway: HSA04210

42.2 Number of Hits: 28

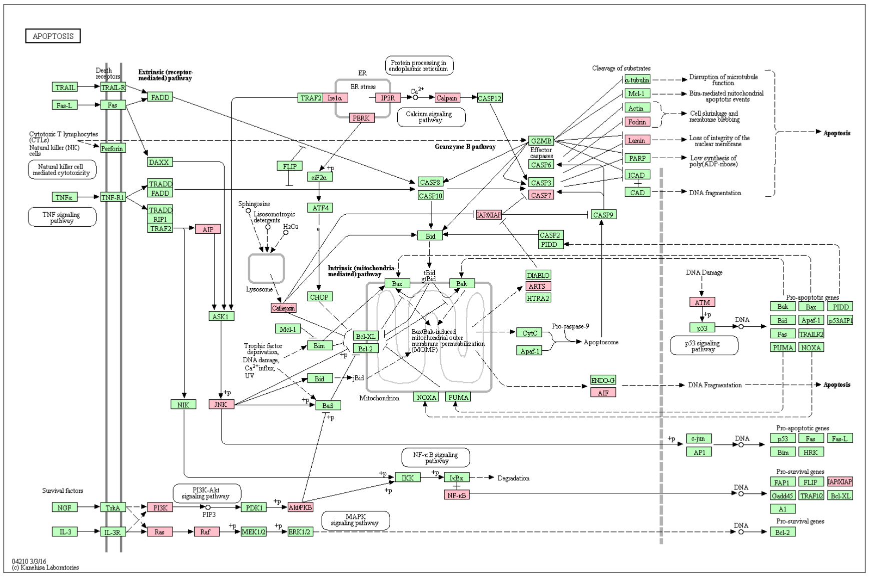

42.3 Legend:

RBH-Blast at 60% Identity + 50% Coverage
Green = Hit in *H. sapiens*
Red = Hit in *H. sapiens* and *C. milli*
White = Not in *H. sapiens*

43 Insulin resistance

43.1 Human Pathway: HSA04931

43.2 Number of Hits: 27

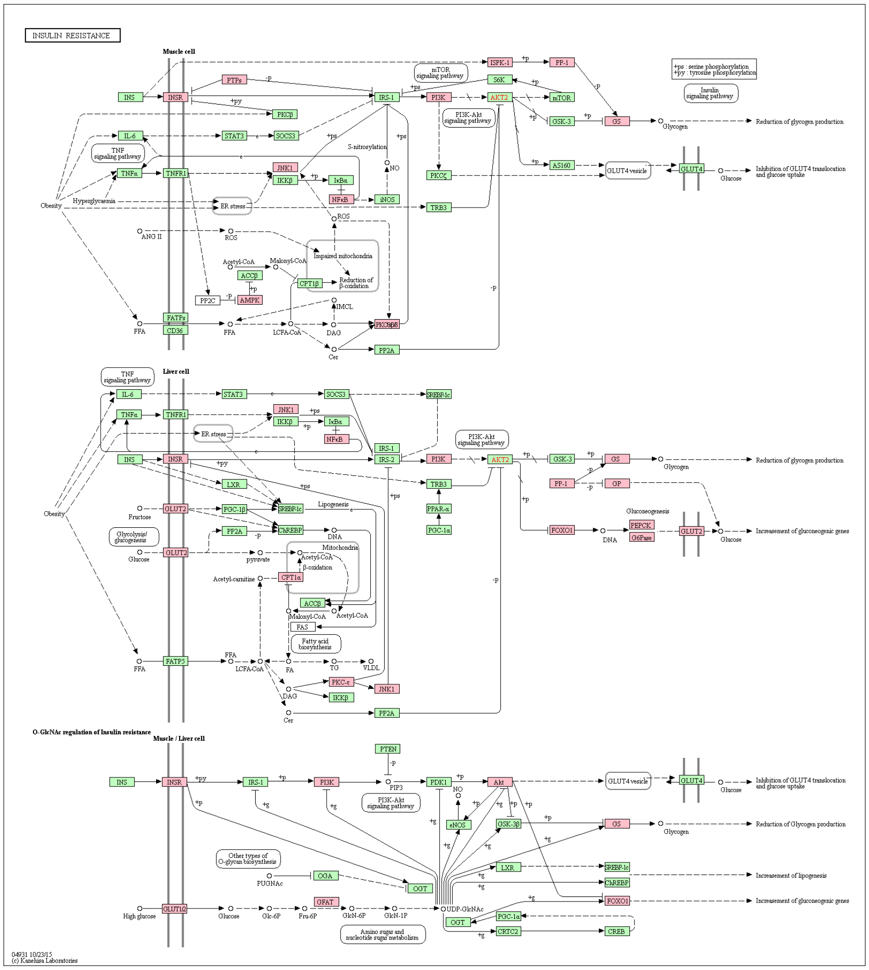

43.3 Legend:

RBH-Blast at 60% Identity + 50% Coverage
Green = Hit in *H. sapiens*
Red = Hit in *H. sapiens* and *C. milli*
White = Not in *H. sapiens*

44 Cell cycle

44.1 Human Pathway: HSA04110

44.2 Number of Hits: 27

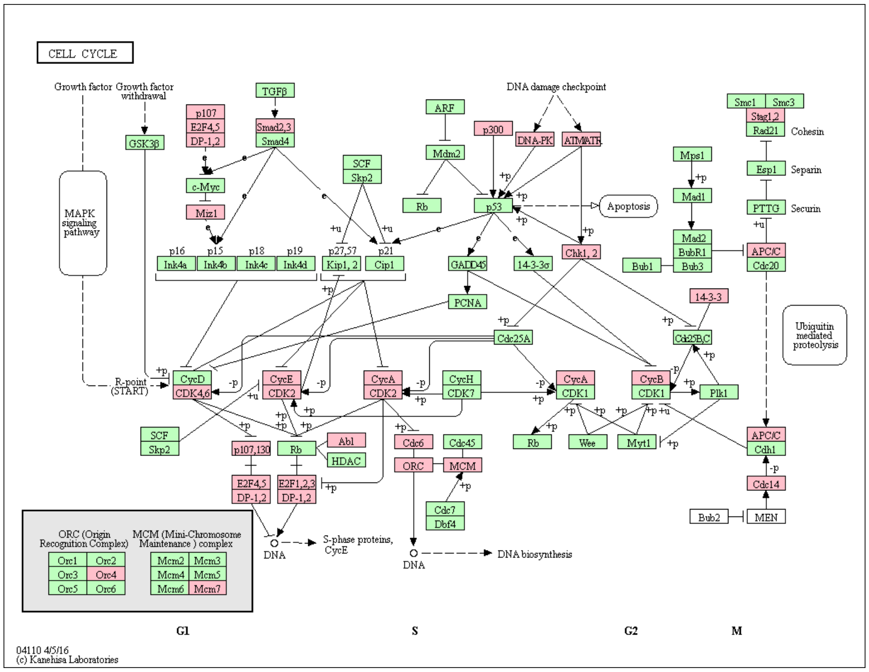

44.3 Legend:

RBH-Blast at 60% Identity + 50% Coverage
Green = Hit in *H. sapiens*
Red = Hit in *H. sapiens* and *C. milli*
White = Not in *H. sapiens*

45 Circadian entrainment

45.1 Human Pathway: HSA04713

45.2 Number of Hits: 27

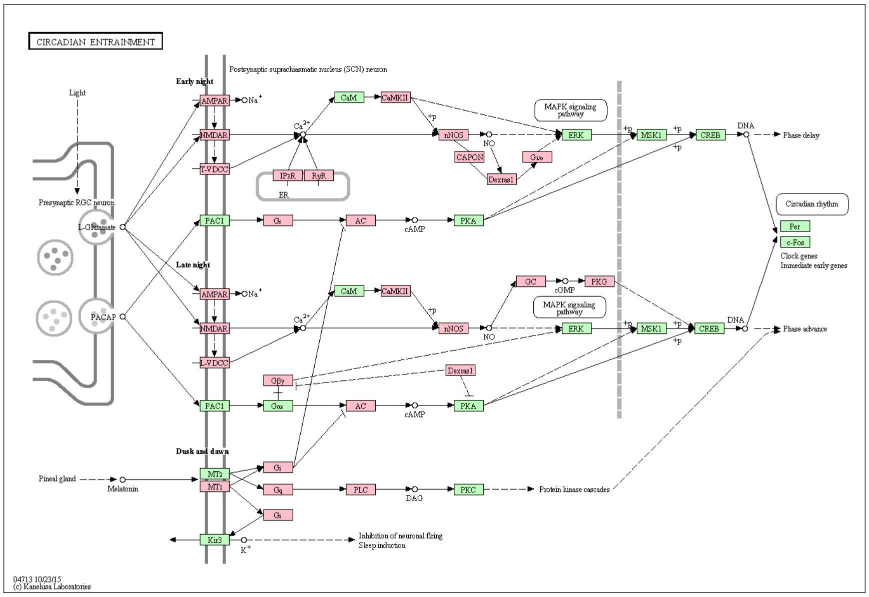

45.3 Legend:

RBH-Blast at 60% Identity + 50% Coverage
Green = Hit in *H. sapiens*
Red = Hit in *H. sapiens* and *C. milli*
White = Not in *H. sapiens*

46 Alcoholism

46.1 Human Pathway: HSA05034

46.2 Number of Hits: 27

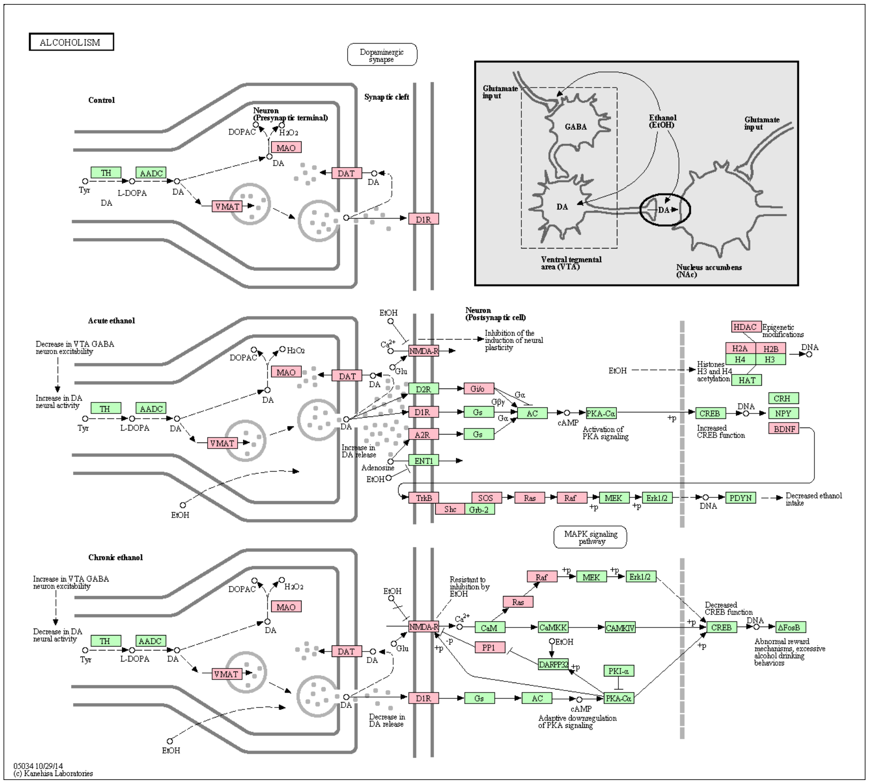

46.3 Legend:

RBH-Blast at 60% Identity + 50% Coverage
Green = Hit in *H. sapiens*
Red = Hit in *H. sapiens* and *C. milli*
White = Not in *H. sapiens*

47 Leukocyte transendothelial migration

47.1 Human Pathway: HSA04670

47.2 Number of Hits: 27

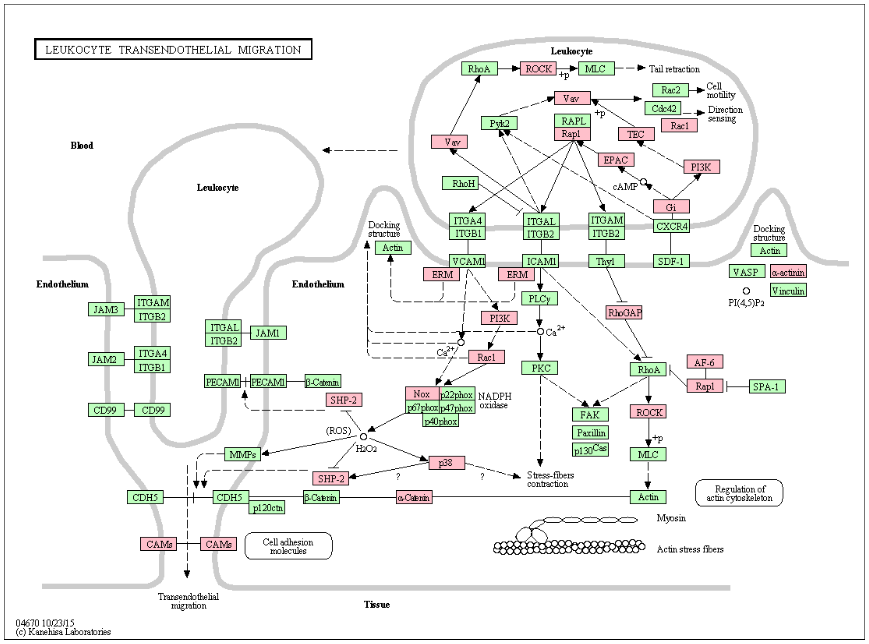

47.3 Legend:

RBH-Blast at 60% Identity + 50% Coverage
Green = Hit in *H. sapiens*
Red = Hit in *H. sapiens* and *C. milli*
White = Not in *H. sapiens*

48 Glucagon signaling pathway

48.1 Human Pathway: HSA04922

48.2 Number of Hits: 27

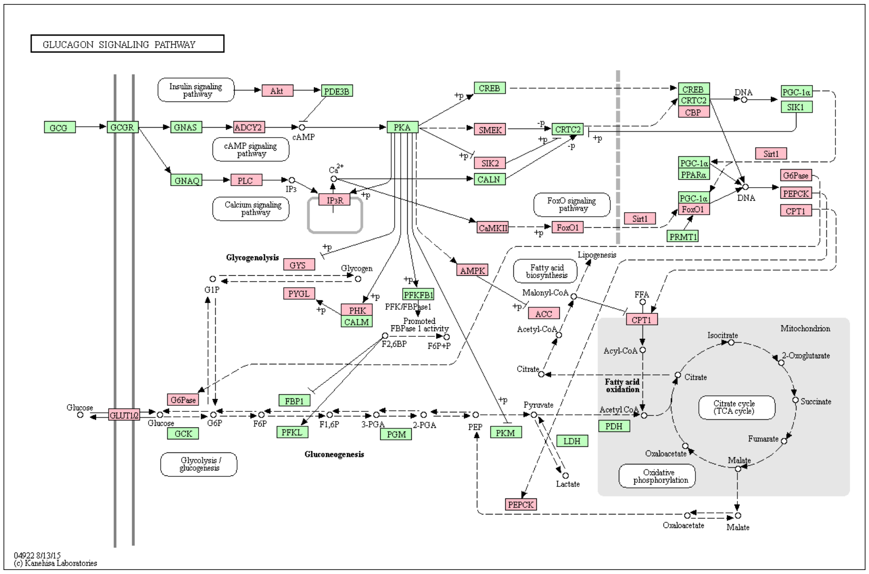

48.3 Legend:

RBH-Blast at 60% Identity + 50% Coverage
Green = Hit in *H. sapiens*
Red = Hit in *H. sapiens* and *C. milli*
White = Not in *H. sapiens*

49 Glutamatergic synapse

49.1 Human Pathway: HSA04724

49.2 Number of Hits: 27

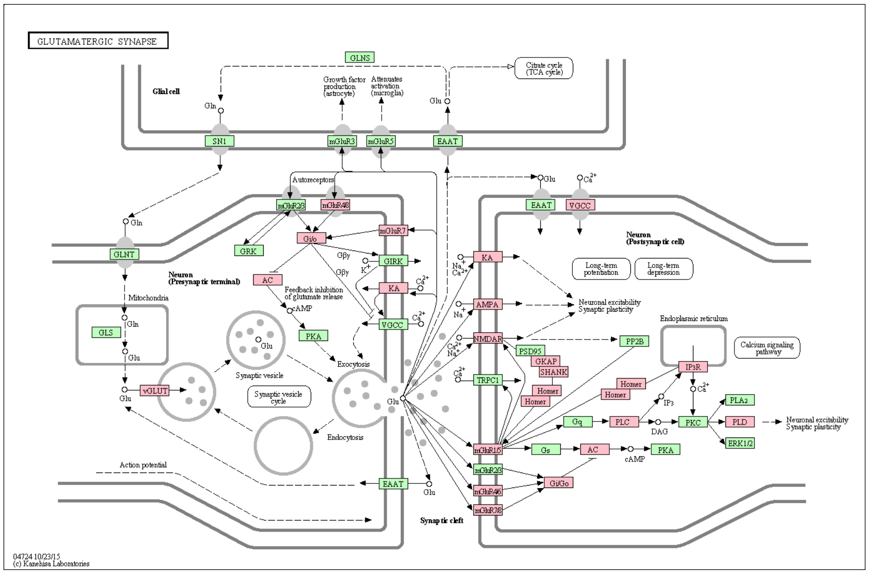

49.3 Legend:

RBH-Blast at 60% Identity + 50% Coverage
Green = Hit in *H. sapiens*
Red = Hit in *H. sapiens* and *C. milli*
White = Not in *H. sapiens*

50 Tight junction

50.1 Human Pathway: HSA04530

50.2 Number of Hits: 26

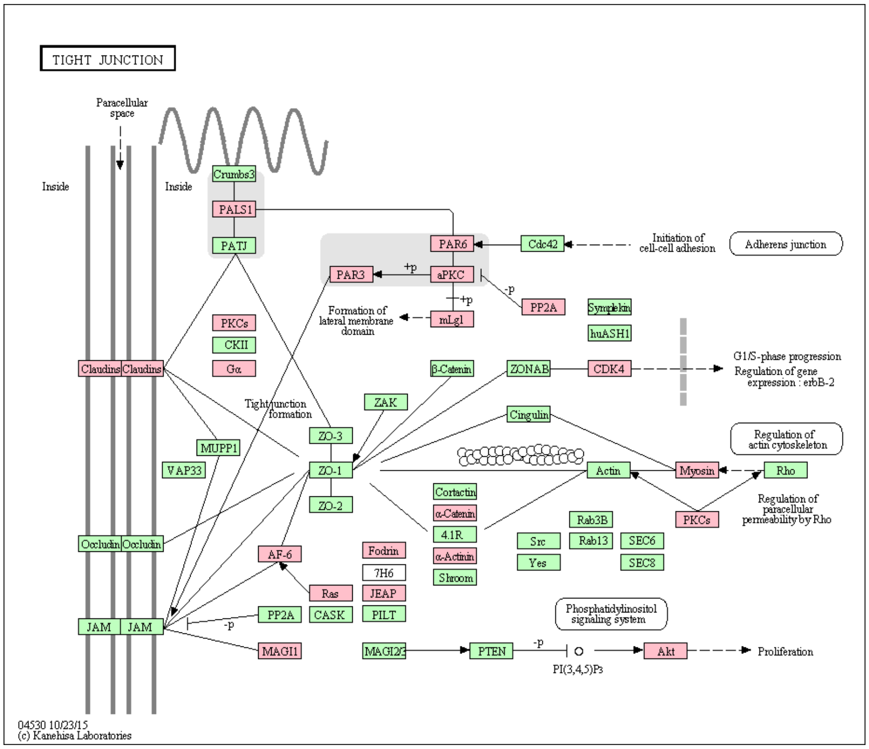

50.3 Legend:

RBH-Blast at 60% Identity + 50% Coverage
Green = Hit in *H. sapiens*
Red = Hit in *H. sapiens* and *C. milli*
White = Not in *H. sapiens*

51 Spliceosome

51.1 Human Pathway: HSA03040

51.2 Number of Hits: 25

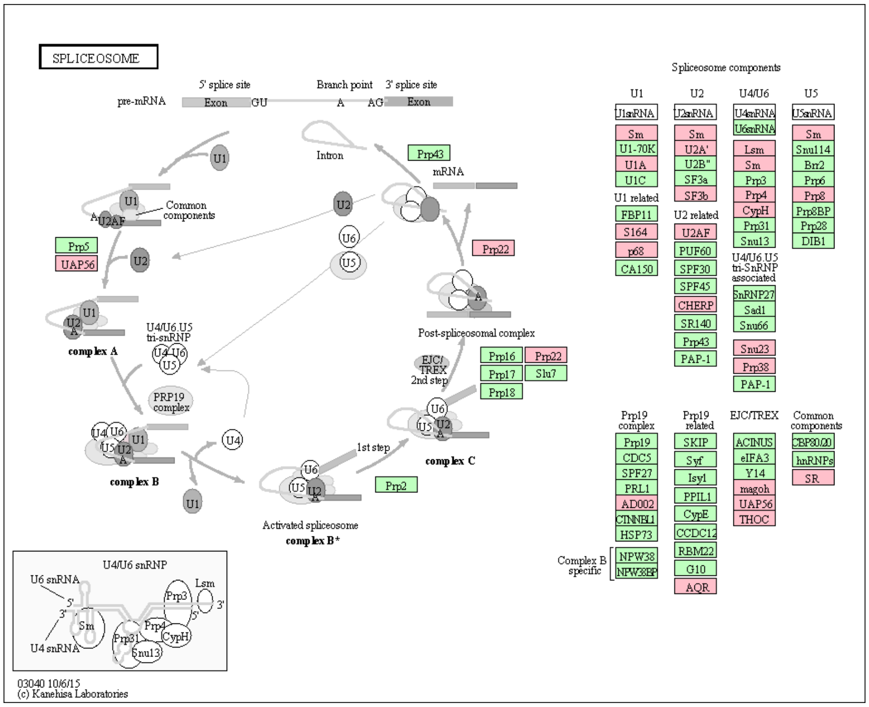

51.3 Legend:

RBH-Blast at 60% Identity + 50% Coverage
Green = Hit in *H. sapiens*
Red = Hit in *H. sapiens* and *C. milli*
White = Not in *H. sapiens*

52 Gap junction

52.1 Human Pathway: HSA04540

52.2 Number of Hits: 25

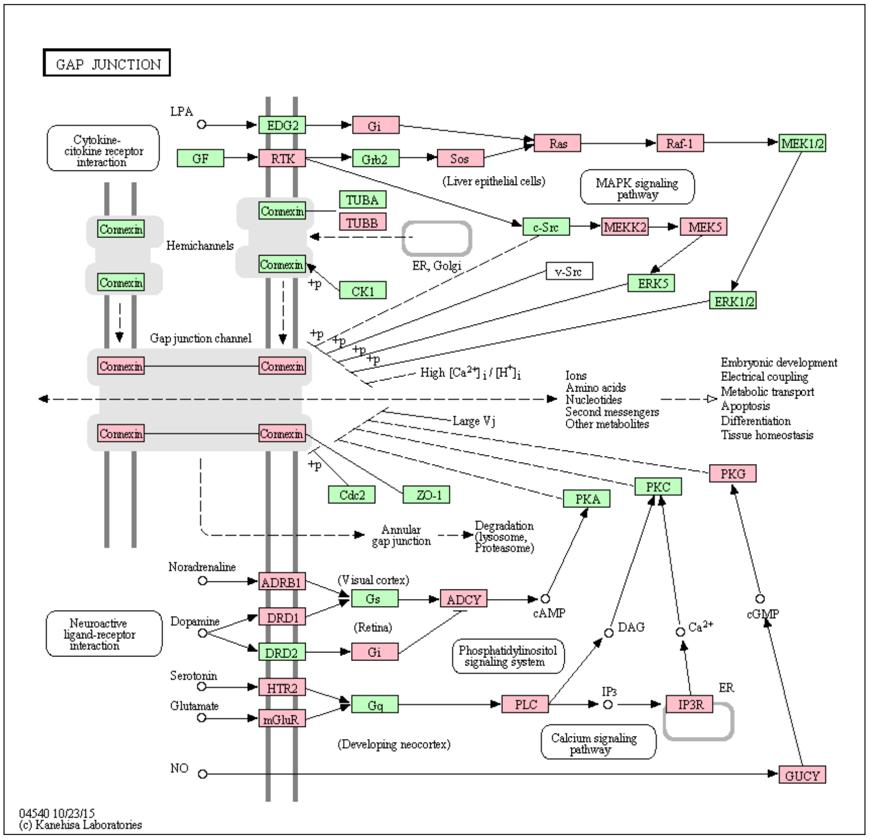

52.3 Legend:

RBH-Blast at 60% Identity + 50% Coverage
Green = Hit in *H. sapiens*
Red = Hit in *H. sapiens* and *C. milli*
White = Not in *H. sapiens*

53 Morphine addiction

53.1 Human Pathway: HSA05032

53.2 Number of Hits: 25

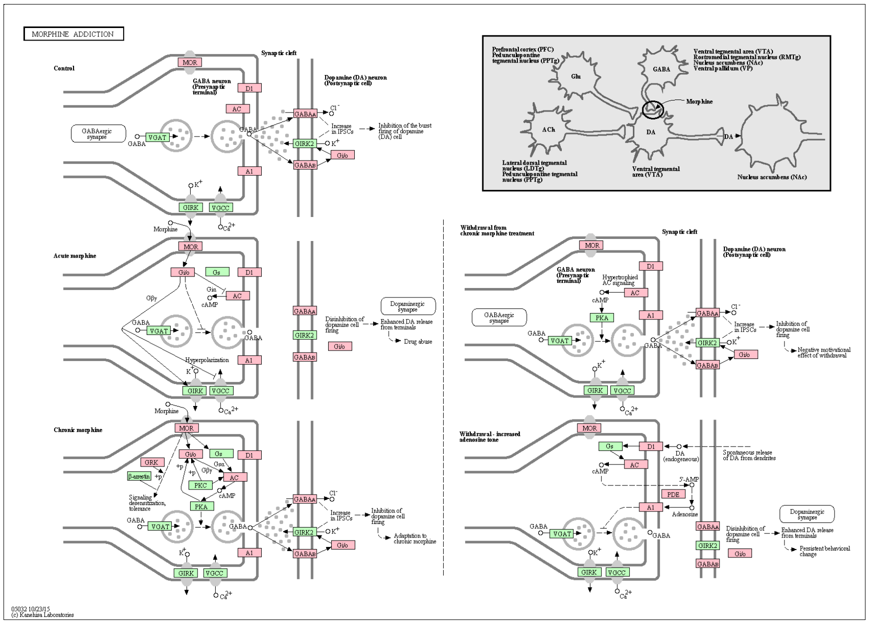

53.3 Legend:

RBH-Blast at 60% Identity + 50% Coverage
Green = Hit in *H. sapiens*
Red = Hit in *H. sapiens* and *C. milli*
White = Not in *H. sapiens*

54 Basal cell carcinoma

54.1 Human Pathway: HSA05217

54.2 Number of Hits: 25

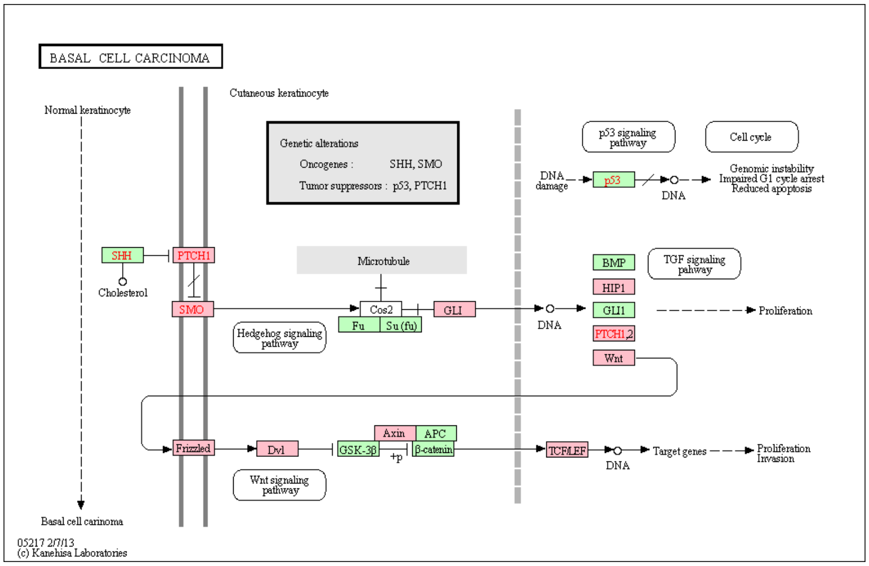

54.3 Legend:

RBH-Blast at 60% Identity + 50% Coverage
Green = Hit in *H. sapiens*
Red = Hit in *H. sapiens* and *C. milli*
White = Not in *H. sapiens*

55 Longevity regulating pathway

55.1 Human Pathway: HSA04211

55.2 Number of Hits: 25

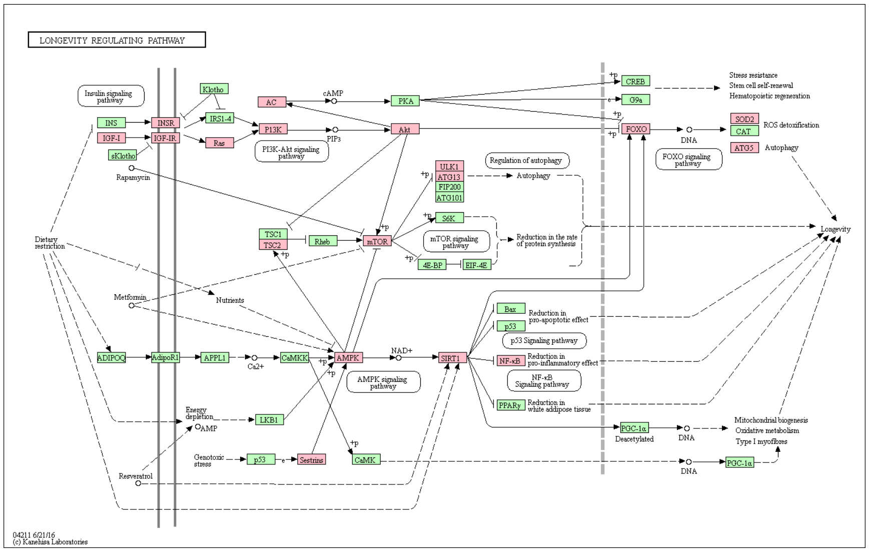

55.3 Legend:

RBH-Blast at 60% Identity + 50% Coverage
Green = Hit in *H. sapiens*
Red = Hit in *H. sapiens* and *C. milli*
White = Not in *H. sapiens*

56 Non-alcoholic fatty liver disease (NAFLD)

56.1 Human Pathway: HSA04932

56.2 Number of Hits: 25

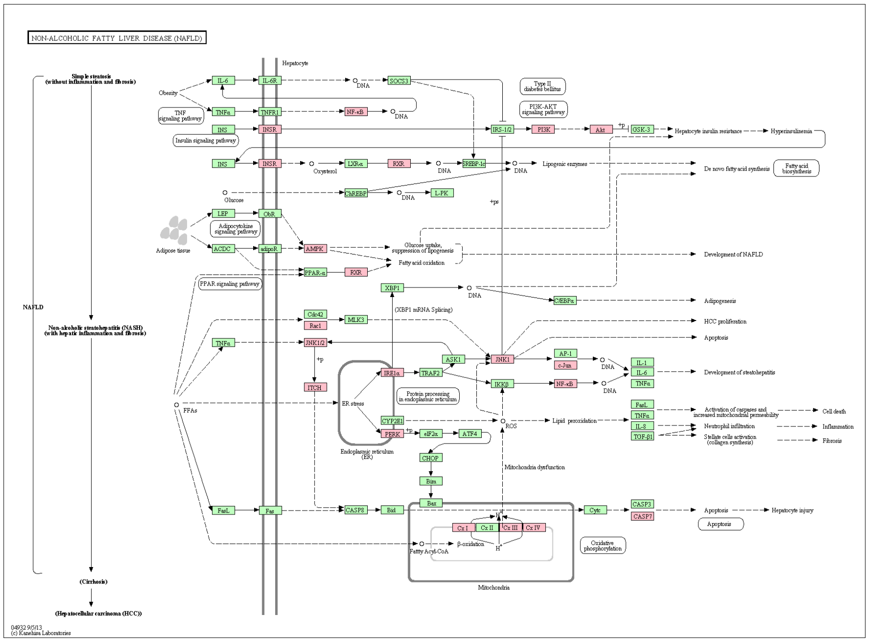

56.3 Legend:

RBH-Blast at 60% Identity + 50% Coverage
Green = Hit in *H. sapiens*
Red = Hit in *H. sapiens* and *C. milli*
White = Not in *H. sapiens*

57 Ubiquitin mediated proteolysis

57.1 Human Pathway: HSA04120

57.2 Number of Hits: 25

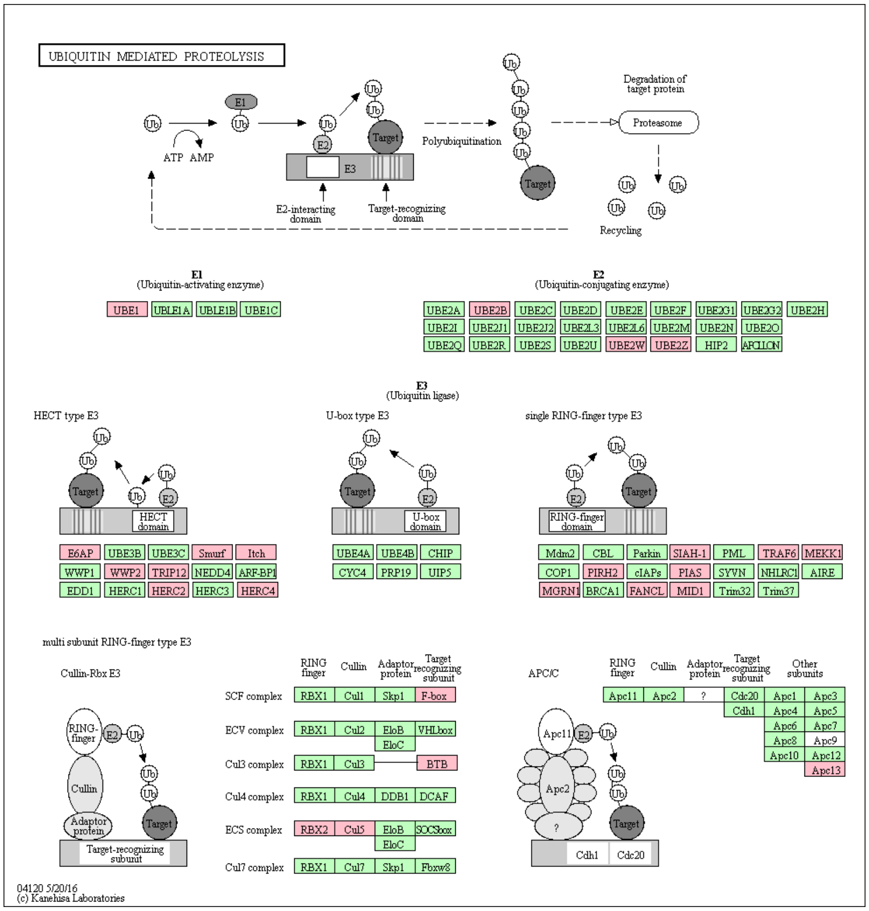

57.3 Legend:

RBH-Blast at 60% Identity + 50% Coverage
Green = Hit in *H. sapiens*
Red = Hit in *H. sapiens* and *C. milli*
White = Not in *H. sapiens*

58 Protein processing in endoplasmic reticulum

58.1 Human Pathway: HSA04141

58.2 Number of Hits: 25

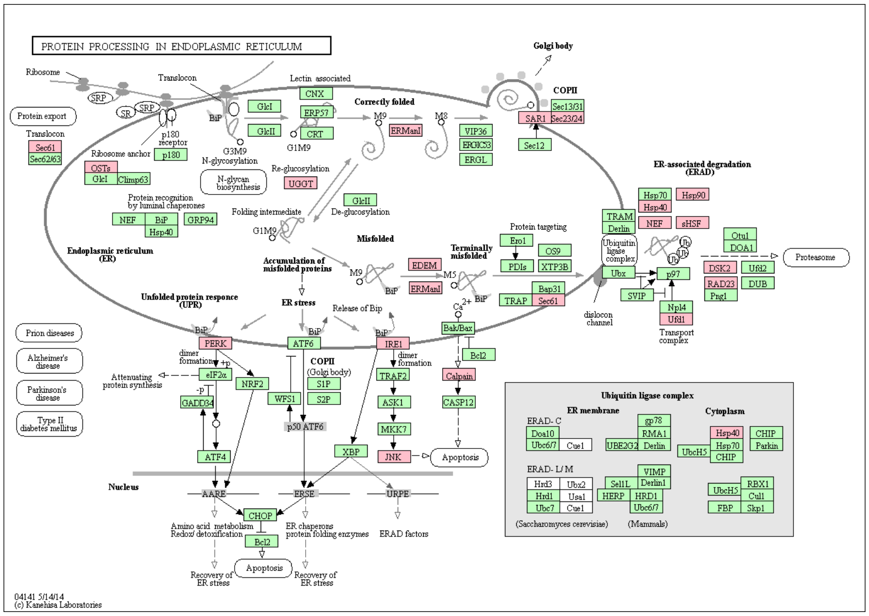

58.3 Legend:

RBH-Blast at 60% Identity + 50% Coverage
Green = Hit in *H. sapiens*
Red = Hit in *H. sapiens* and *C. milli*
White = Not in *H. sapiens*

59 Prostate cancer

59.1 Human Pathway: HSA05215

59.2 Number of Hits: 24

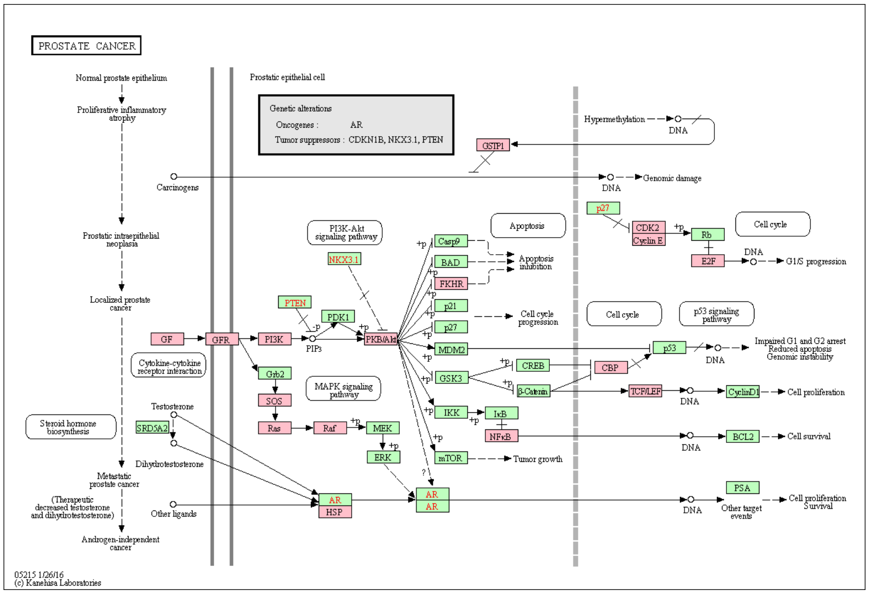

59.3 Legend:

RBH-Blast at 60% Identity + 50% Coverage
Green = Hit in *H. sapiens*
Red = Hit in *H. sapiens* and *C. milli*
White = Not in *H. sapiens*

60 Hepatitis C

60.1 Human Pathway: HSA05160

60.2 Number of Hits: 24

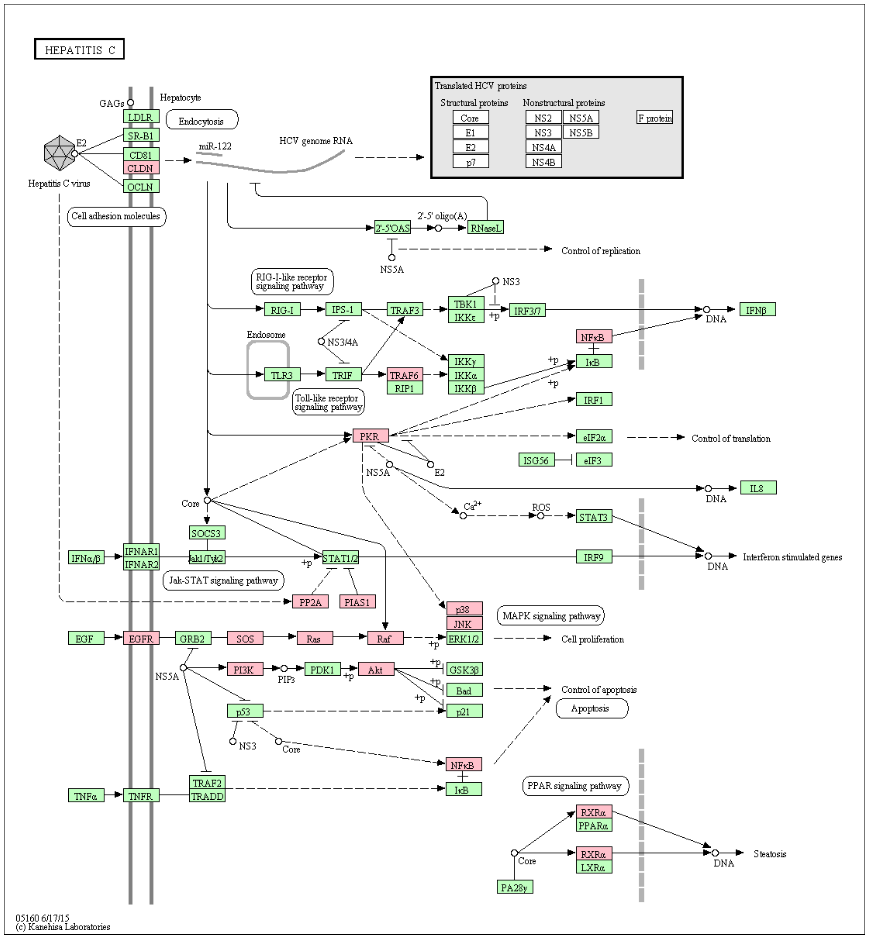

60.3 Legend:

RBH-Blast at 60% Identity + 50% Coverage
Green = Hit in *H. sapiens*
Red = Hit in *H. sapiens* and *C. milli*
White = Not in *H. sapiens*

61 RNA transport

61.1 Human Pathway: HSA03013

61.2 Number of Hits: 24

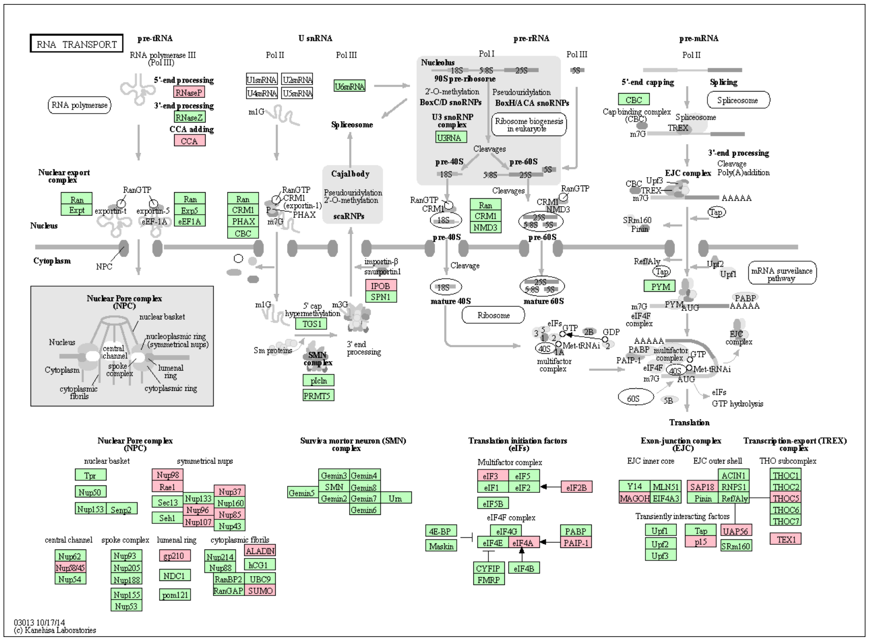

61.3 Legend:

RBH-Blast at 60% Identity + 50% Coverage
Green = Hit in *H. sapiens*
Red = Hit in *H. sapiens* and *C. milli*
White = Not in *H. sapiens*

62 Lysosome

62.1 Human Pathway: HSA04142

62.2 Number of Hits: 24

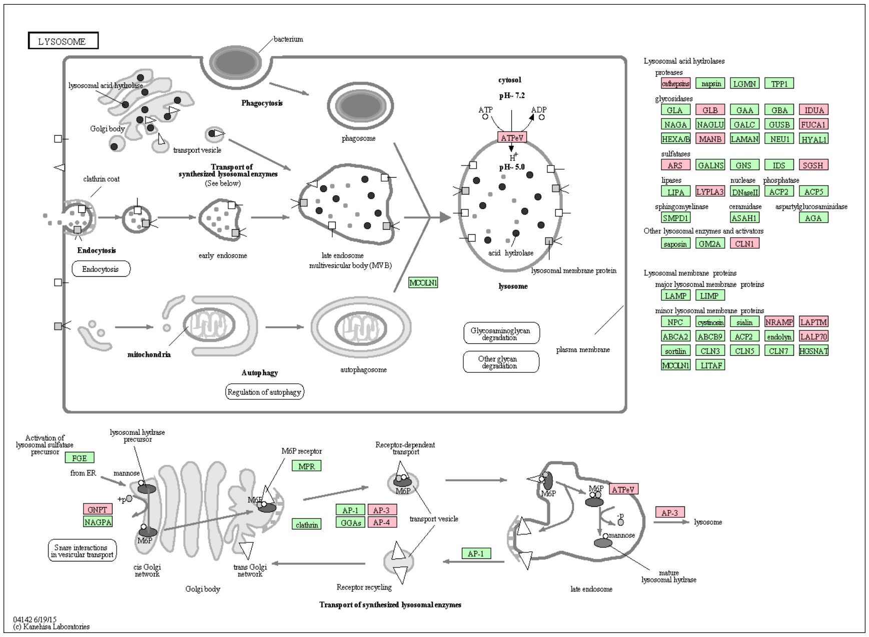

62.3 Legend:

RBH-Blast at 60% Identity + 50% Coverage
Green = Hit in *H. sapiens*
Red = Hit in *H. sapiens* and *C. milli*
White = Not in *H. sapiens*

63 Platelet activation

63.1 Human Pathway: HSA04611

63.2 Number of Hits: 24

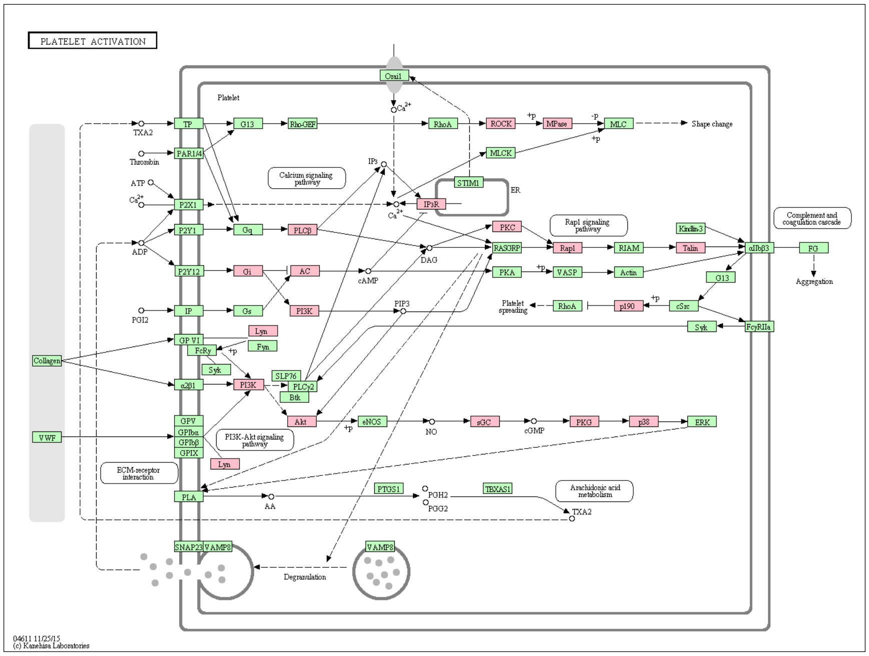

63.3 Legend:

RBH-Blast at 60% Identity + 50% Coverage
Green = Hit in *H. sapiens*
Red = Hit in *H. sapiens* and *C. milli*
White = Not in *H. sapiens*

64 Hepatitis B

64.1 Human Pathway: HSA05161

64.2 Number of Hits: 24

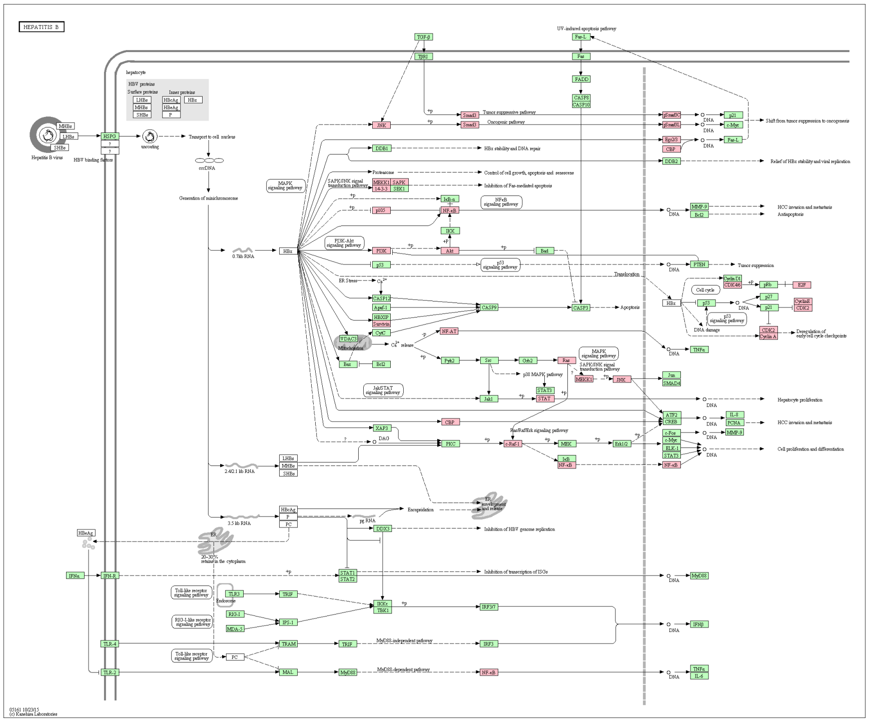

64.3 Legend:

RBH-Blast at 60% Identity + 50% Coverage
Green = Hit in *H. sapiens*
Red = Hit in *H. sapiens* and *C. milli*
White = Not in *H. sapiens*

65 Estrogen signaling pathway

65.1 Human Pathway: HSA04915

65.2 Number of Hits: 24

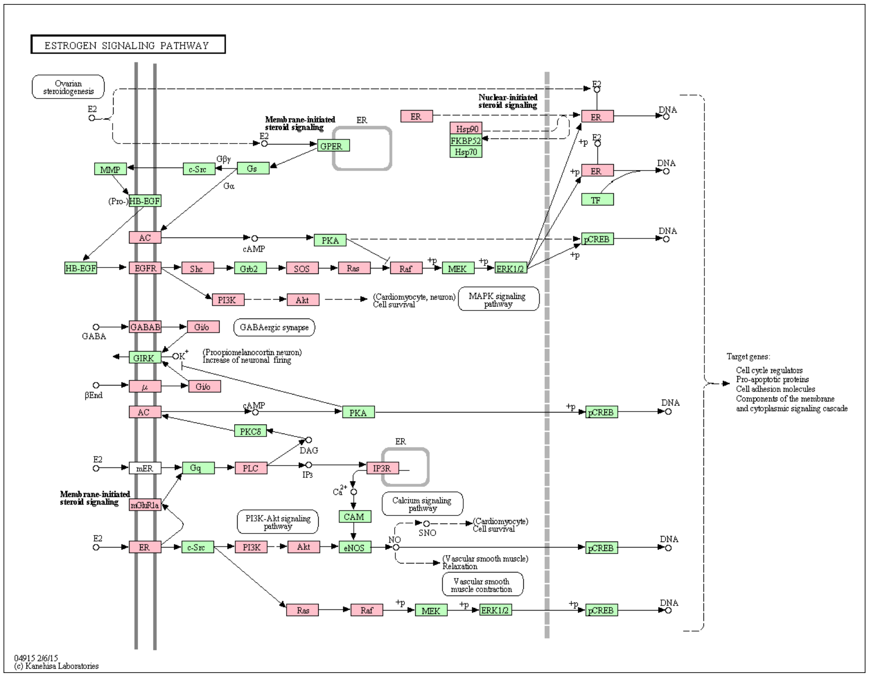

65.3 Legend:

RBH-Blast at 60% Identity + 50% Coverage
Green = Hit in *H. sapiens*
Red = Hit in *H. sapiens* and *C. milli*
White = Not in *H. sapiens*

66 Vascular smooth muscle contraction

66.1 Human Pathway: HSA04270

66.2 Number of Hits: 23

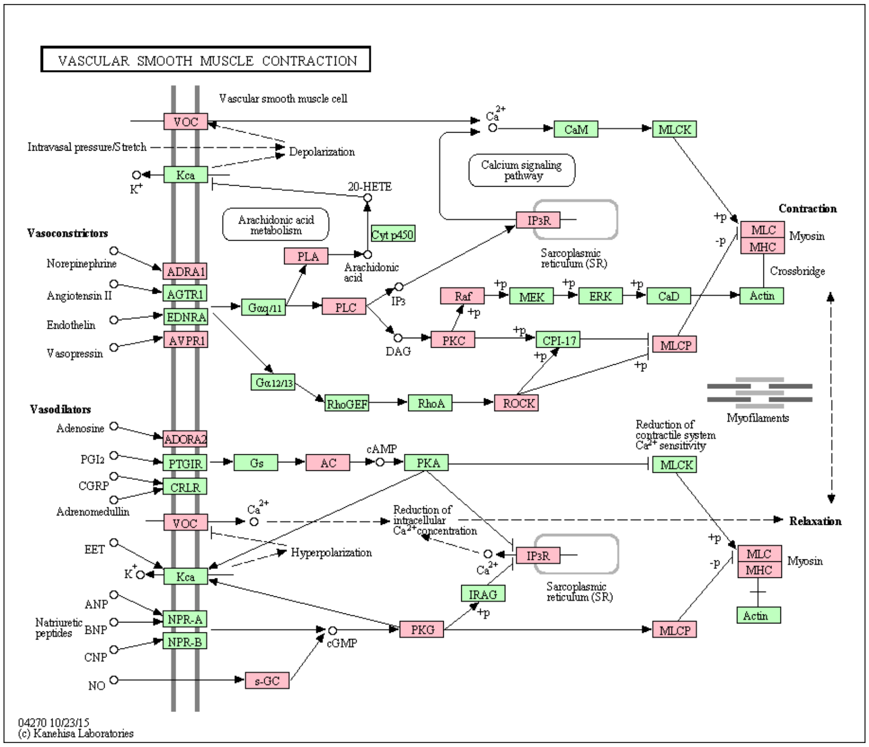

66.3 Legend:

RBH-Blast at 60% Identity + 50% Coverage
Green = Hit in *H. sapiens*
Red = Hit in *H. sapiens* and *C. milli*
White = Not in *H. sapiens*

67 Inflammatory mediator regulation of TRP channels

67.1 Human Pathway: HSA04750

67.2 Number of Hits: 23

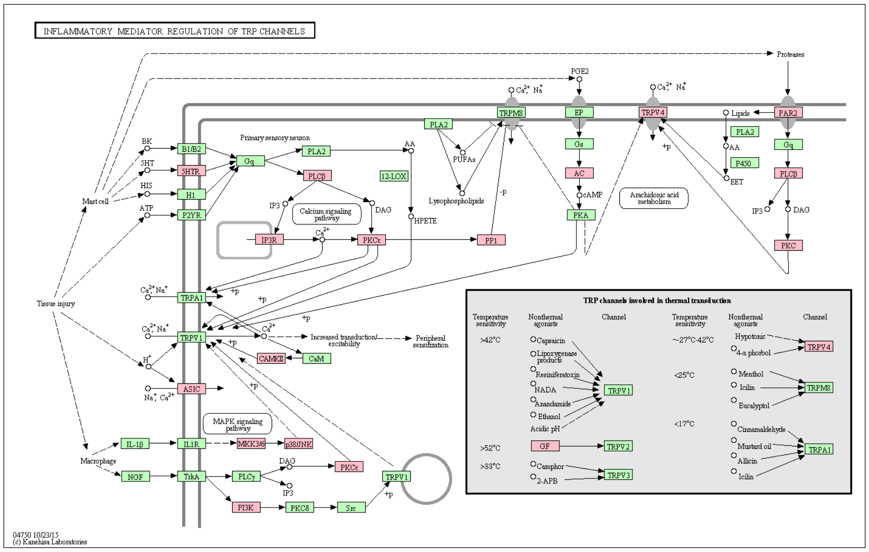

67.3 Legend:

RBH-Blast at 60% Identity + 50% Coverage
Green = Hit in *H. sapiens*
Red = Hit in *H. sapiens* and *C. milli*
White = Not in *H. sapiens*

68 Phosphatidylinositol signaling system

68.1 Human Pathway: HSA04070

68.2 Number of Hits: 23

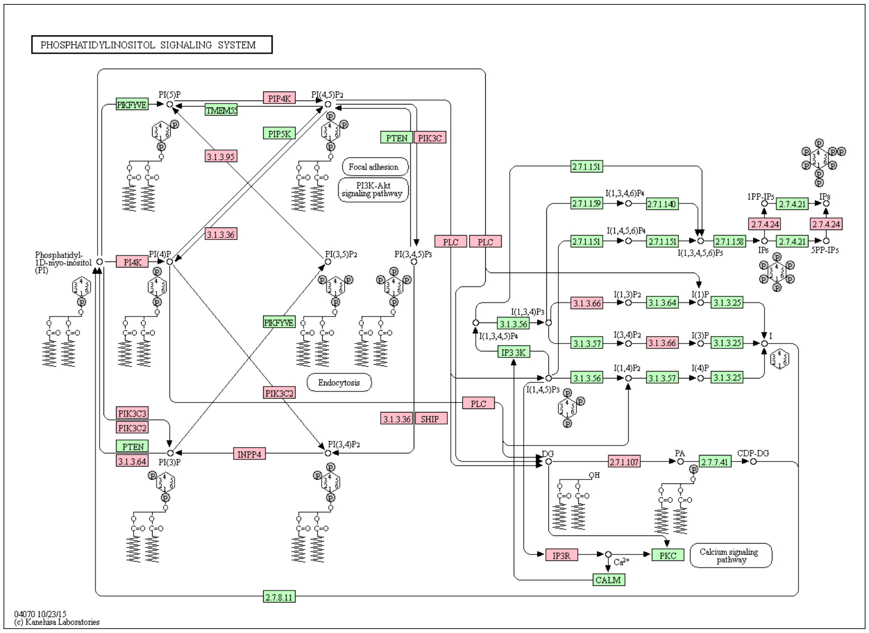

68.3 Legend:

RBH-Blast at 60% Identity + 50% Coverage
Green = Hit in *H. sapiens*
Red = Hit in *H. sapiens* and *C. milli*
White = Not in *H. sapiens*

69 AGE-RAGE signaling pathway in diabetic complications

69.1 Human Pathway: HSA04933

69.2 Number of Hits: 23

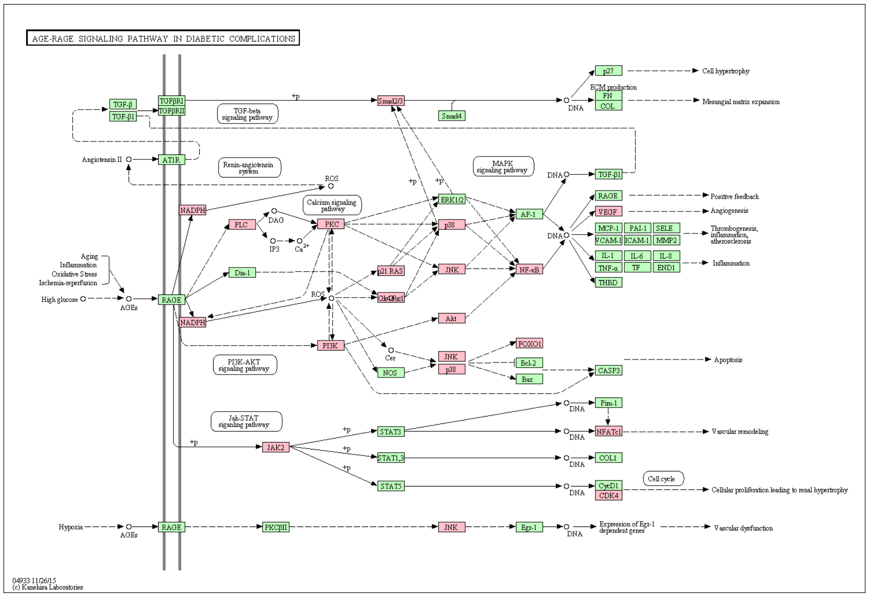

69.3 Legend:

RBH-Blast at 60% Identity + 50% Coverage
Green = Hit in *H. sapiens*
Red = Hit in *H. sapiens* and *C. milli*
White = Not in *H. sapiens*

70 GnRH signaling pathway

70.1 Human Pathway: HSA04912

70.2 Number of Hits: 22

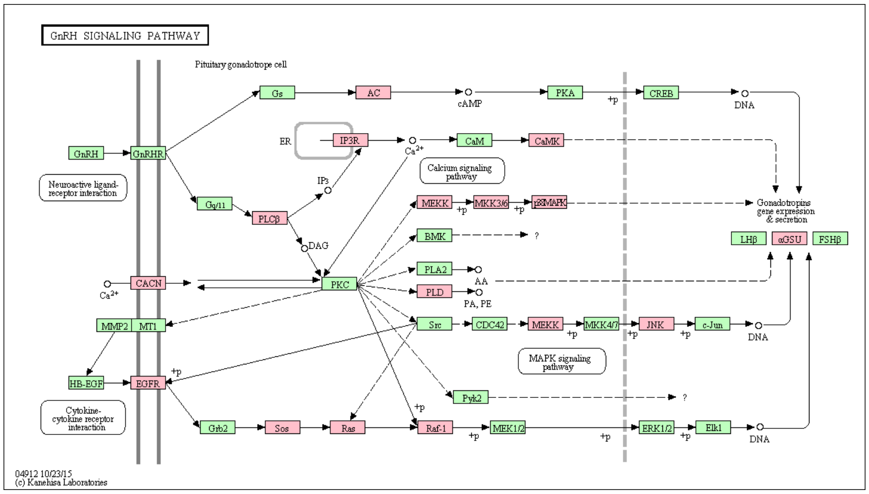

70.3 Legend:

RBH-Blast at 60% Identity + 50% Coverage
Green = Hit in *H. sapiens*
Red = Hit in *H. sapiens* and *C. milli*
White = Not in *H. sapiens*

71 Renal cell carcinoma

71.1 Human Pathway: HSA05211

71.2 Number of Hits: 22

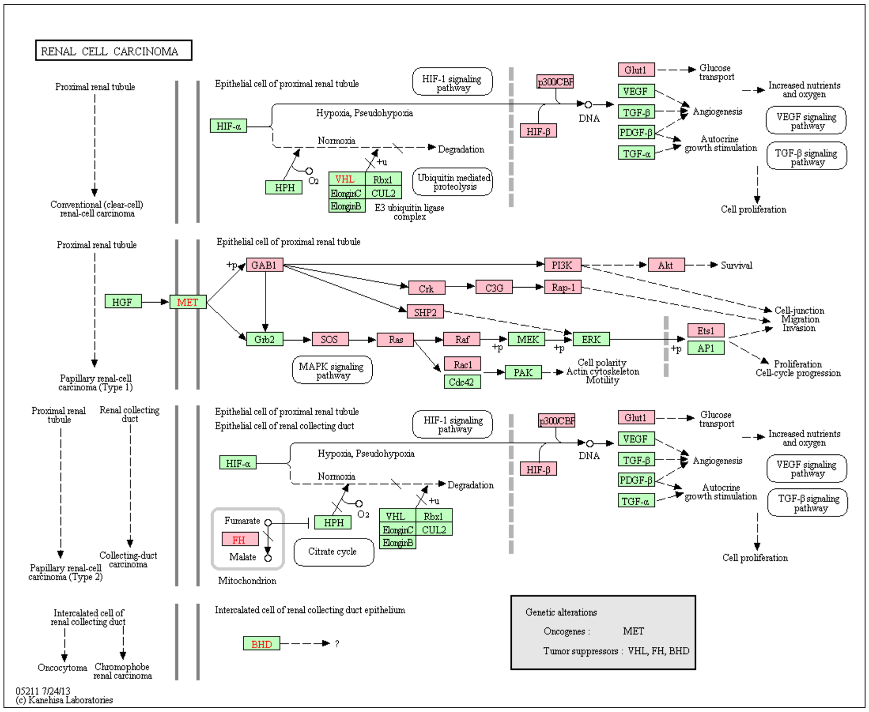

71.3 Legend:

RBH-Blast at 60% Identity + 50% Coverage
Green = Hit in *H. sapiens*
Red = Hit in *H. sapiens* and *C. milli*
White = Not in *H. sapiens*

72 Progesterone-mediated oocyte maturation

72.1 Human Pathway: HSA04914

72.2 Number of Hits: 22

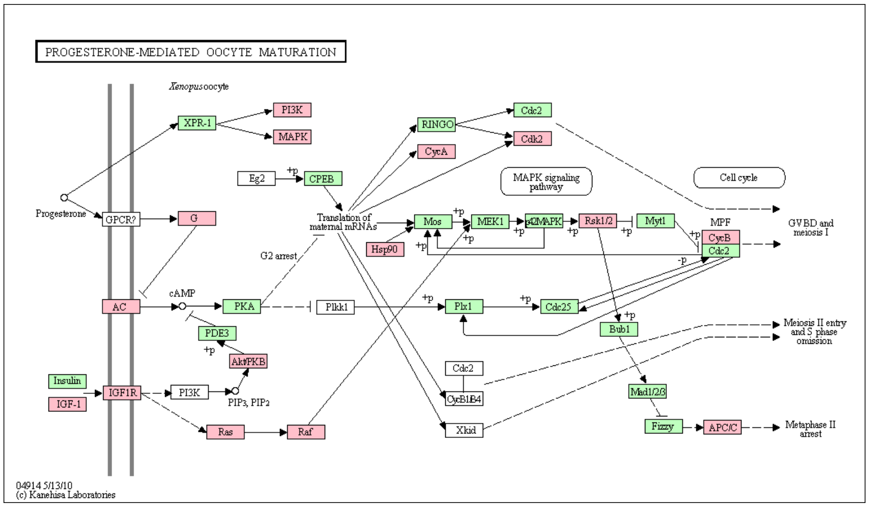

72.3 Legend:

RBH-Blast at 60% Identity + 50% Coverage
Green = Hit in *H. sapiens*
Red = Hit in *H. sapiens* and *C. milli*
White = Not in *H. sapiens*

73 Prolactin signaling pathway

73.1 Human Pathway: HSA04917

73.2 Number of Hits: 22

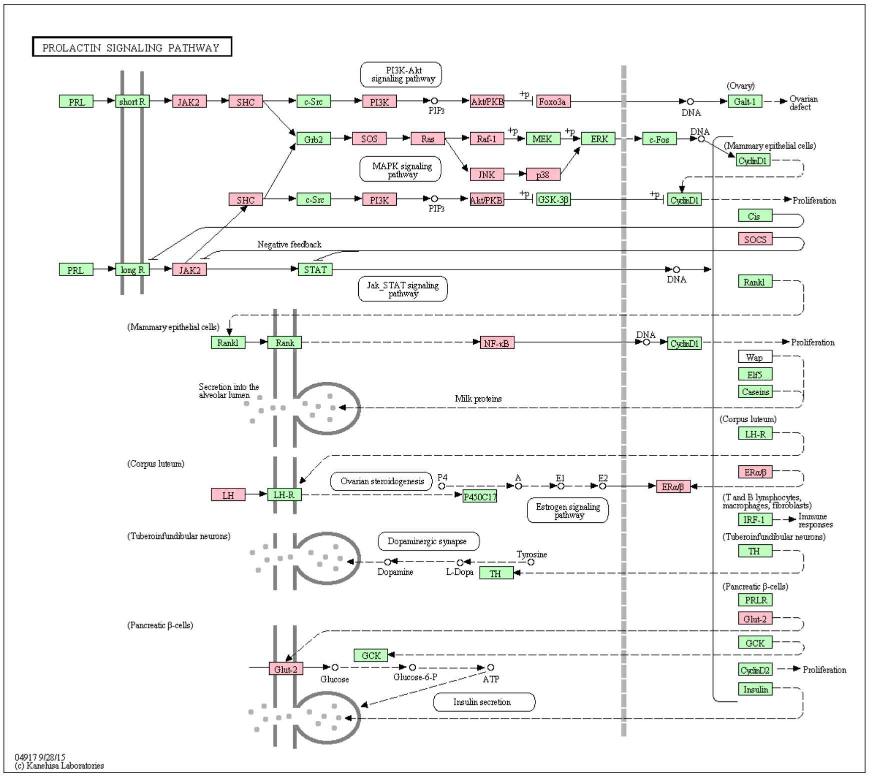

73.3 Legend:

RBH-Blast at 60% Identity + 50% Coverage
Green = Hit in *H. sapiens*
Red = Hit in *H. sapiens* and *C. milli*
White = Not in *H. sapiens*

74 Influenza A

74.1 Human Pathway: HSA05164

74.2 Number of Hits: 21

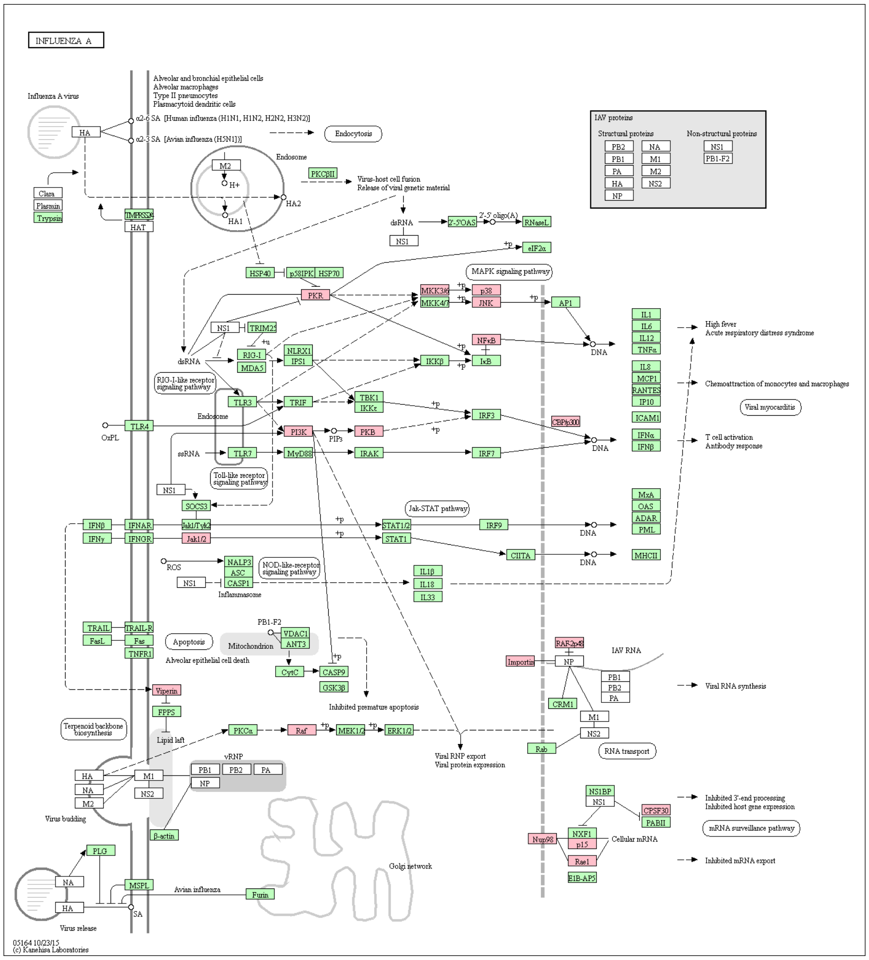

74.3 Legend:

RBH-Blast at 60% Identity + 50% Coverage
Green = Hit in *H. sapiens*
Red = Hit in *H. sapiens* and *C. milli*
White = Not in *H. sapiens*

75 Parkinson's disease

75.1 Human Pathway: HSA05012

75.2 Number of Hits: 21

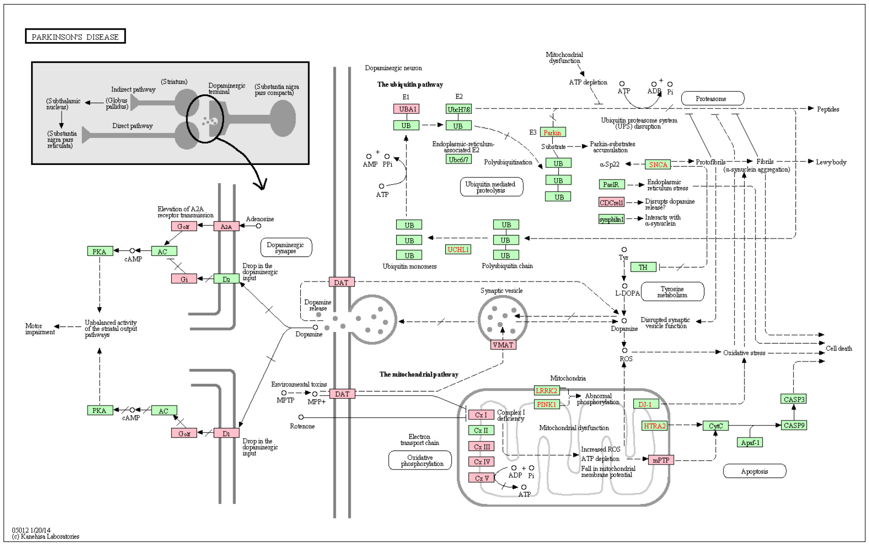

75.3 Legend:

RBH-Blast at 60% Identity + 50% Coverage
Green = Hit in *H. sapiens*
Red = Hit in *H. sapiens* and *C. milli*
White = Not in *H. sapiens*

76 Chronic myeloid leukemia

76.1 Human Pathway: HSA05220

76.2 Number of Hits: 21

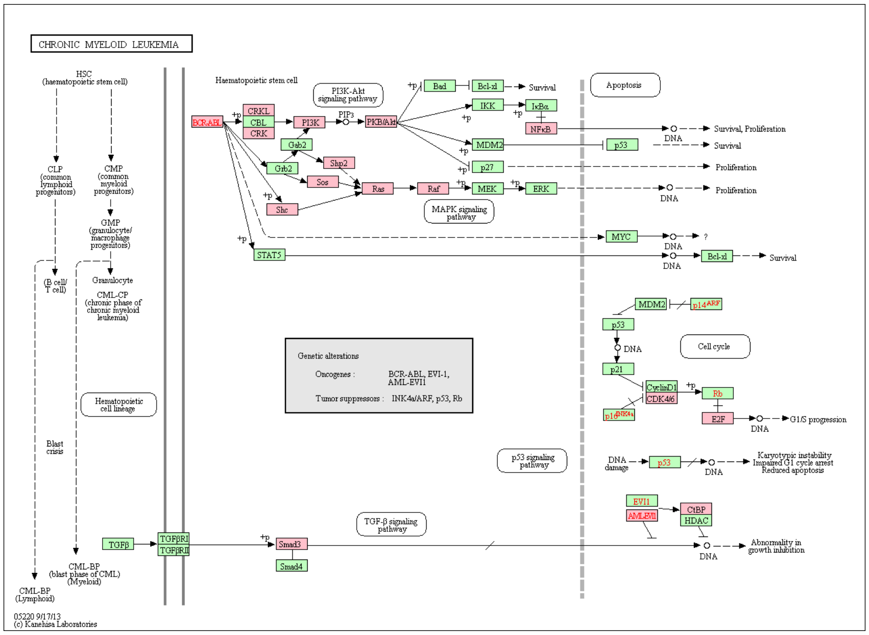

76.3 Legend:

RBH-Blast at 60% Identity + 50% Coverage
Green = Hit in *H. sapiens*
Red = Hit in *H. sapiens* and *C. milli*
White = Not in *H. sapiens*

77 Osteoclast differentiation

77.1 Human Pathway: HSA04380

77.2 Number of Hits: 21

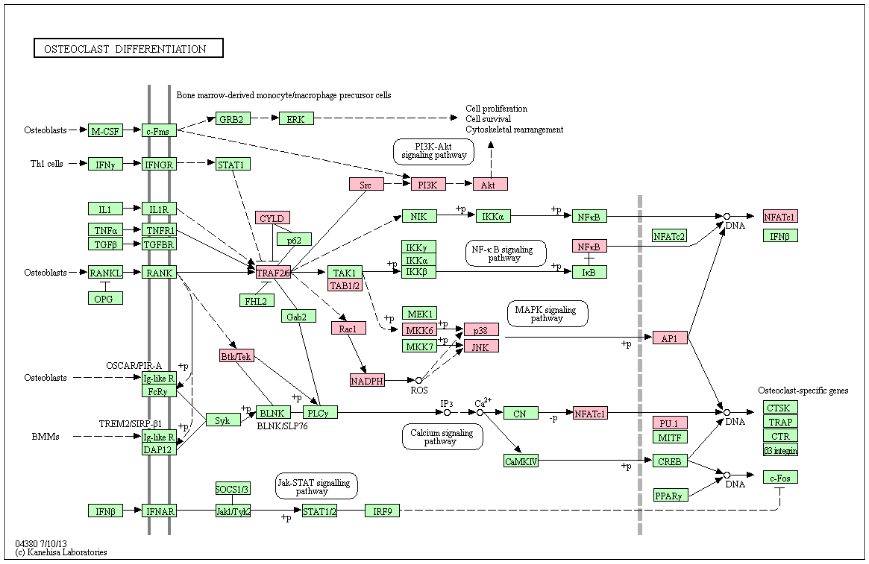

77.3 Legend:

RBH-Blast at 60% Identity + 50% Coverage
Green = Hit in *H. sapiens*
Red = Hit in *H. sapiens* and *C. milli*
White = Not in *H. sapiens*

78 Longevity regulating pathway - multiple species

78.1 Human Pathway: HSA04213

78.2 Number of Hits: 21

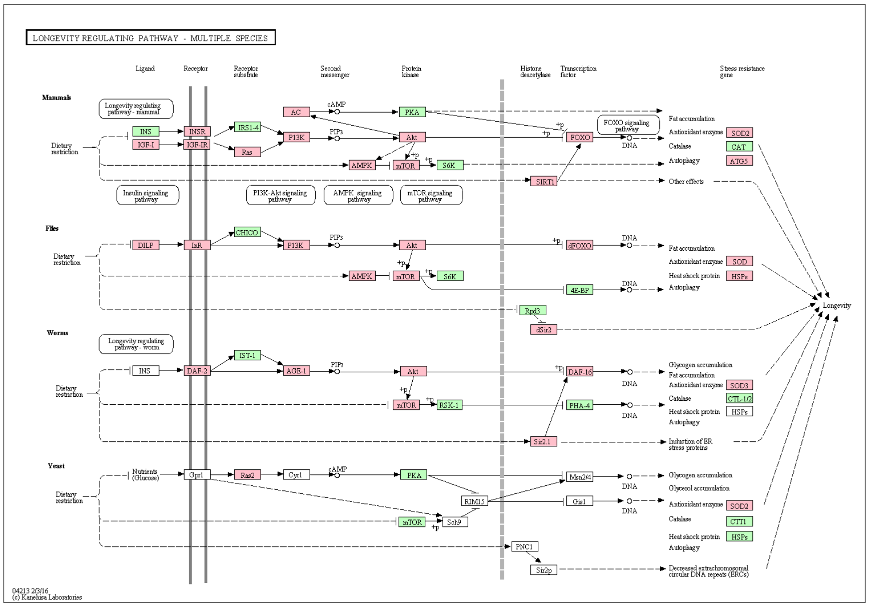

78.3 Legend:

RBH-Blast at 60% Identity + 50% Coverage
Green = Hit in *H. sapiens*
Red = Hit in *H. sapiens* and *C. milli*
White = Not in *H. sapiens*

79 Choline metabolism in cancer

79.1 Human Pathway: HSA05231

79.2 Number of Hits: 21

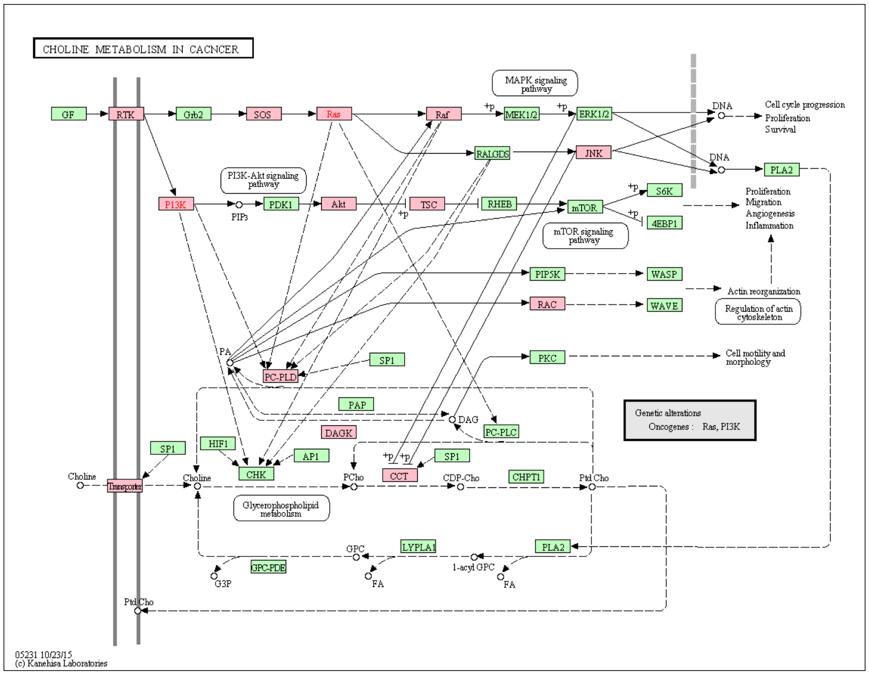

79.3 Legend:

RBH-Blast at 60% Identity + 50% Coverage
Green = Hit in *H. sapiens*
Red = Hit in *H. sapiens* and *C. milli*
White = Not in *H. sapiens*

80 T cell receptor signaling pathway

80.1 Human Pathway: HSA04660

80.2 Number of Hits: 21

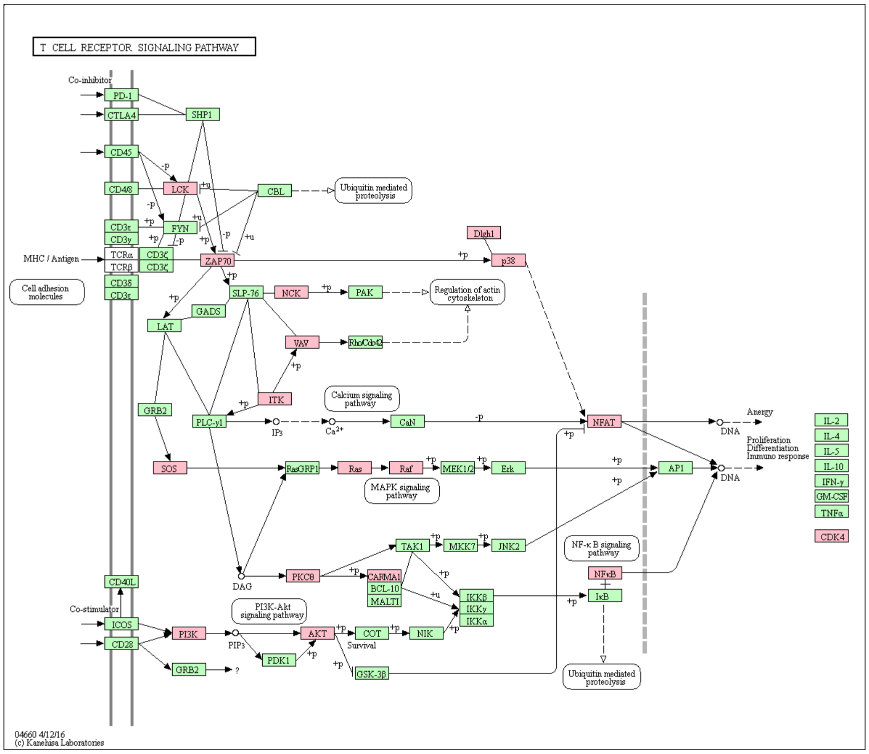

80.3 Legend:

RBH-Blast at 60% Identity + 50% Coverage
Green = Hit in *H. sapiens*
Red = Hit in *H. sapiens* and *C. milli*
White = Not in *H. sapiens*

81 Glycerophospholipid metabolism

81.1 Human Pathway: HSA00564

81.2 Number of Hits: 21

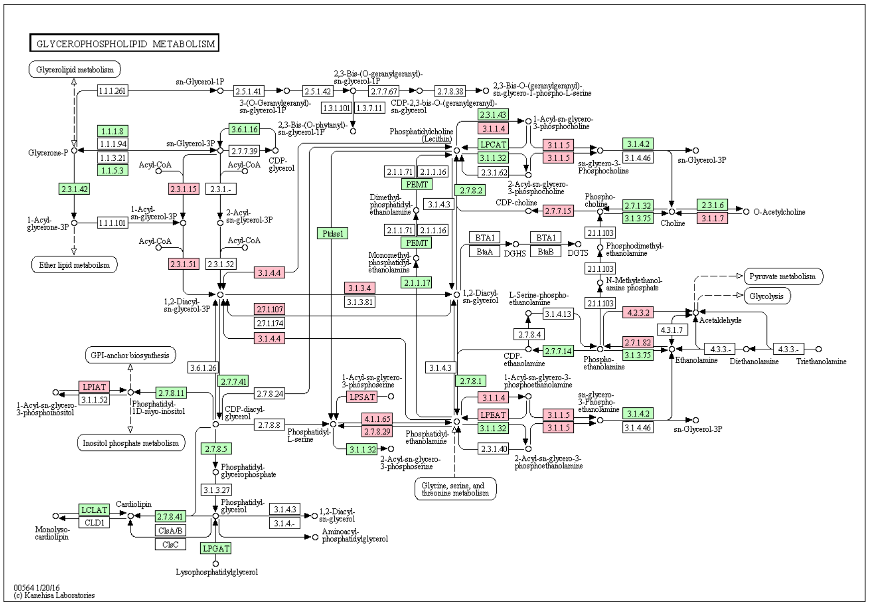

81.3 Legend:

RBH-Blast at 60% Identity + 50% Coverage
Green = Hit in *H. sapiens*
Red = Hit in *H. sapiens* and *C. milli*
White = Not in *H. sapiens*

82 Adherens junction

82.1 Human Pathway: HSA04520

82.2 Number of Hits: 20

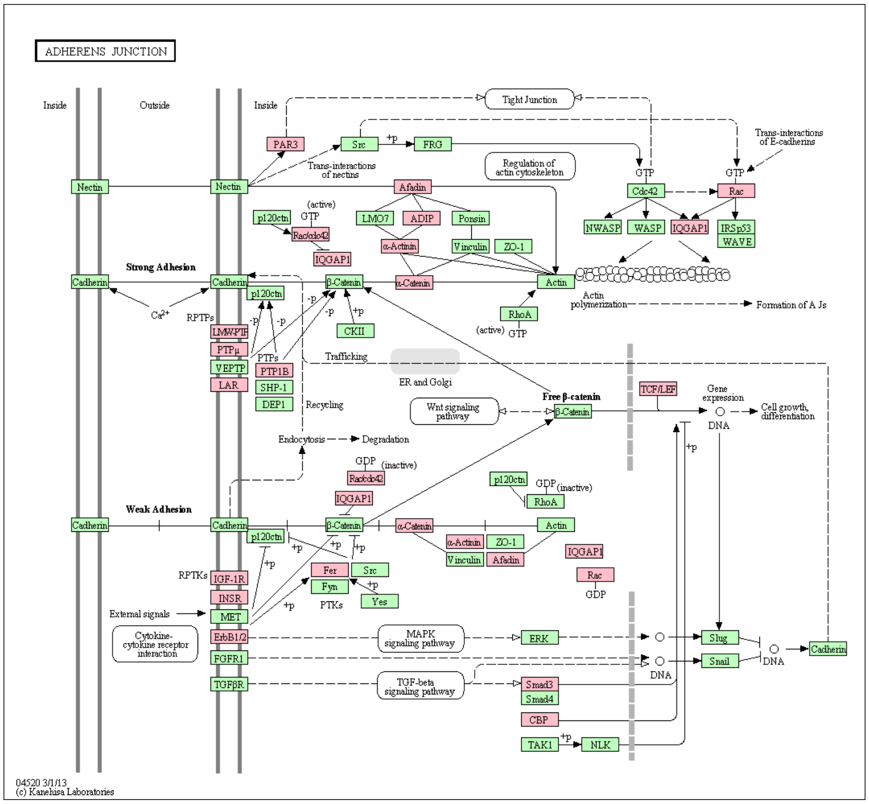

82.3 Legend:

RBH-Blast at 60% Identity + 50% Coverage
Green = Hit in *H. sapiens*
Red = Hit in *H. sapiens* and *C. milli*
White = Not in *H. sapiens*

83 EGFR tyrosine kinase inhibitor resistance

83.1 Human Pathway: HSA01521

83.2 Number of Hits: 20

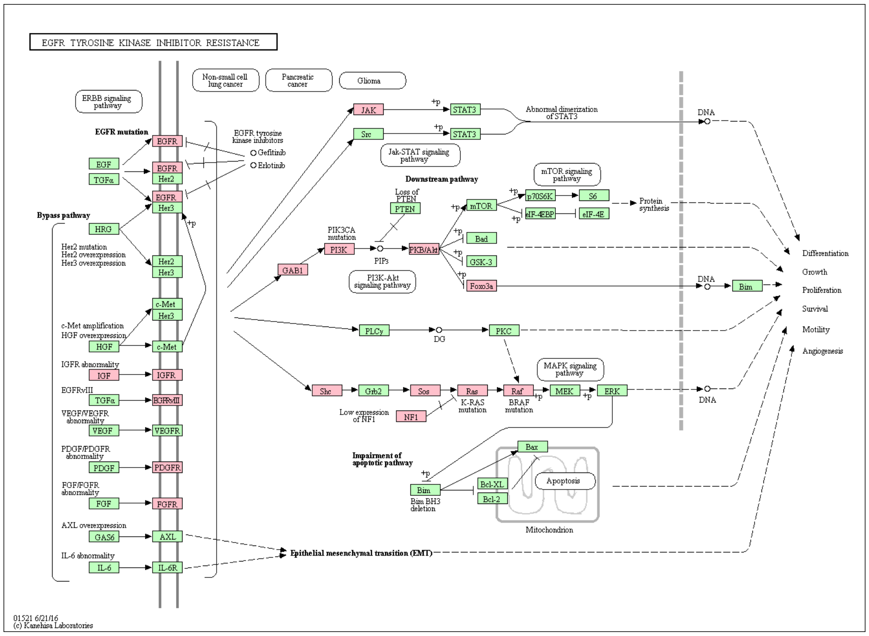

83.3 Legend:

RBH-Blast at 60% Identity + 50% Coverage
Green = Hit in *H. sapiens*
Red = Hit in *H. sapiens* and *C. milli*
White = Not in *H. sapiens*

84 HIF-1 signaling pathway

84.1 Human Pathway: HSA04066

84.2 Number of Hits: 20

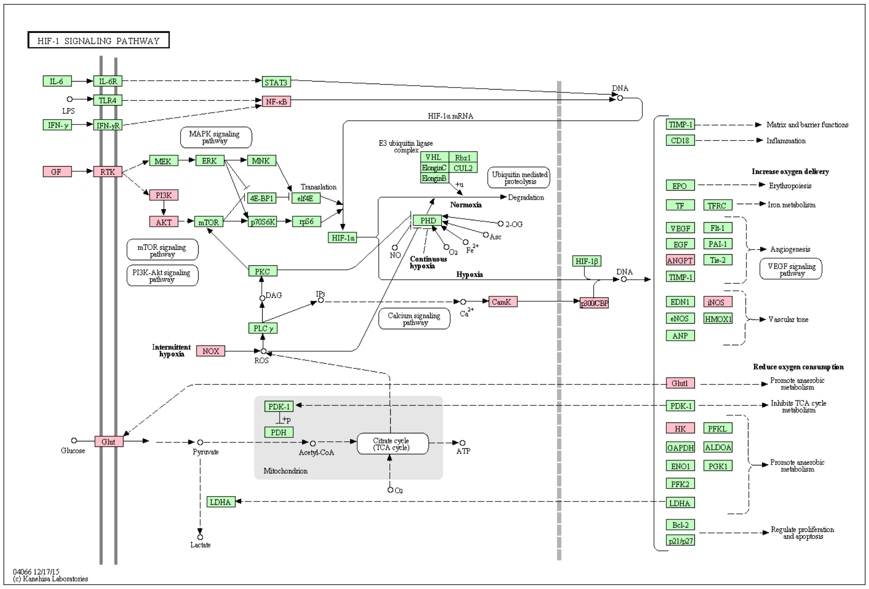

84.3 Legend:

RBH-Blast at 60% Identity + 50% Coverage
Green = Hit in *H. sapiens*
Red = Hit in *H. sapiens* and *C. milli*
White = Not in *H. sapiens*

85 Melanoma

85.1 Human Pathway: HSA05218

85.2 Number of Hits: 20

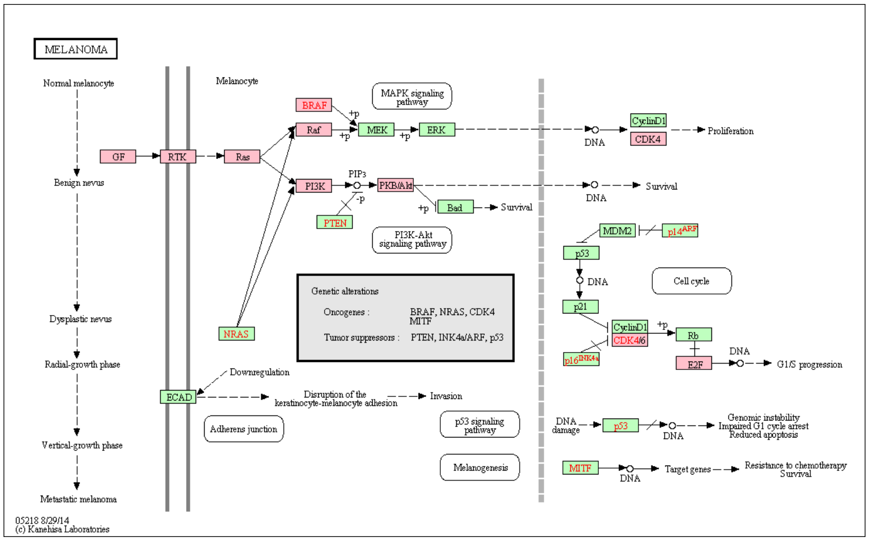

85.3 Legend:

RBH-Blast at 60% Identity + 50% Coverage
Green = Hit in *H. sapiens*
Red = Hit in *H. sapiens* and *C. milli*
White = Not in *H. sapiens*

86 GABAergic synapse

86.1 Human Pathway: HSA04727

86.2 Number of Hits: 20

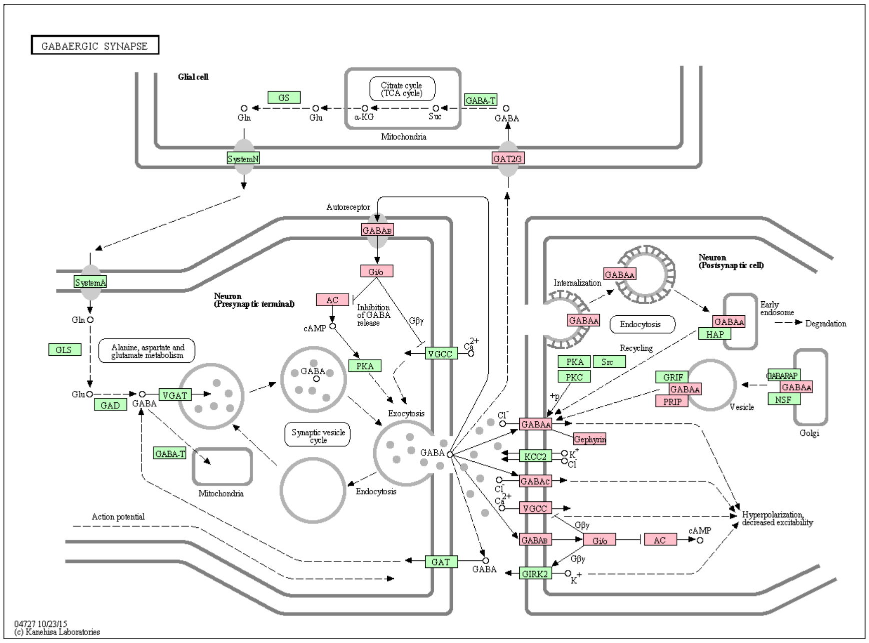

86.3 Legend:

RBH-Blast at 60% Identity + 50% Coverage
Green = Hit in *H. sapiens*
Red = Hit in *H. sapiens* and *C. milli*
White = Not in *H. sapiens*

87 Pyrimidine metabolism

87.1 Human Pathway: HSA00240

87.2 Number of Hits: 20

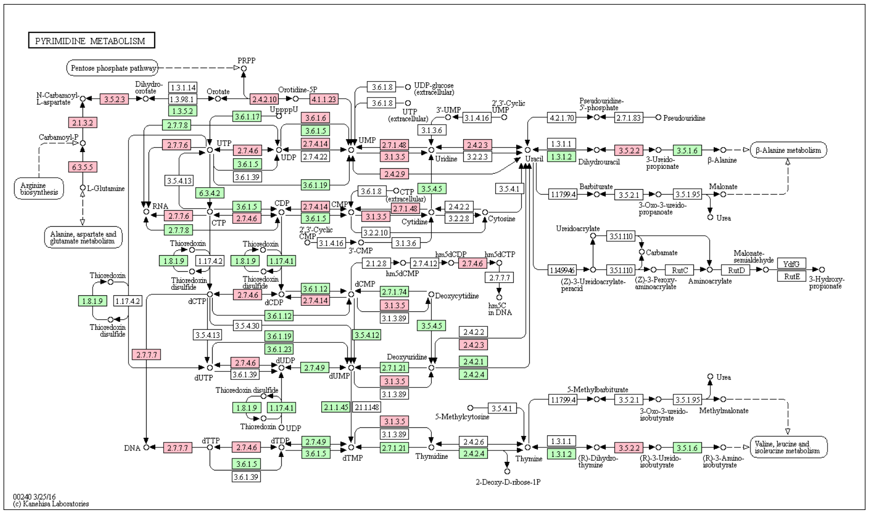

87.3 Legend:

RBH-Blast at 60% Identity + 50% Coverage
Green = Hit in *H. sapiens*
Red = Hit in *H. sapiens* and *C. milli*
White = Not in *H. sapiens*

88 Pancreatic secretion

88.1 Human Pathway: HSA04972

88.2 Number of Hits: 20

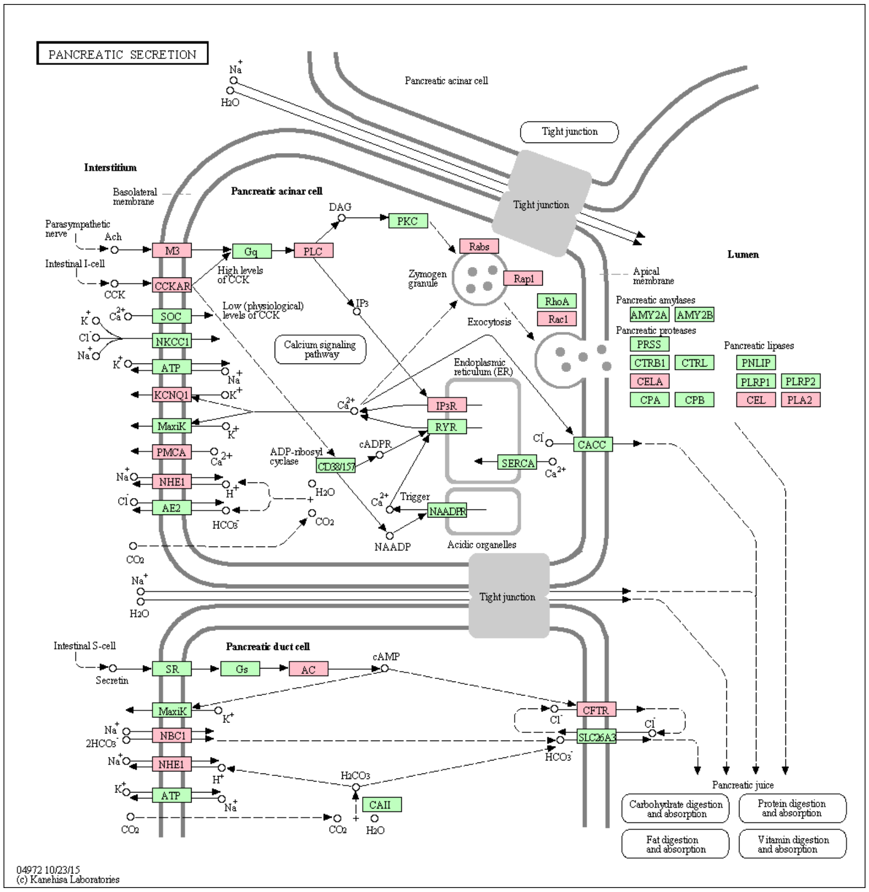

88.3 Legend:

RBH-Blast at 60% Identity + 50% Coverage
Green = Hit in *H. sapiens*
Red = Hit in *H. sapiens* and *C. milli*
White = Not in *H. sapiens*

89 ErbB signaling pathway

89.1 Human Pathway: HSA04012

89.2 Number of Hits: 20

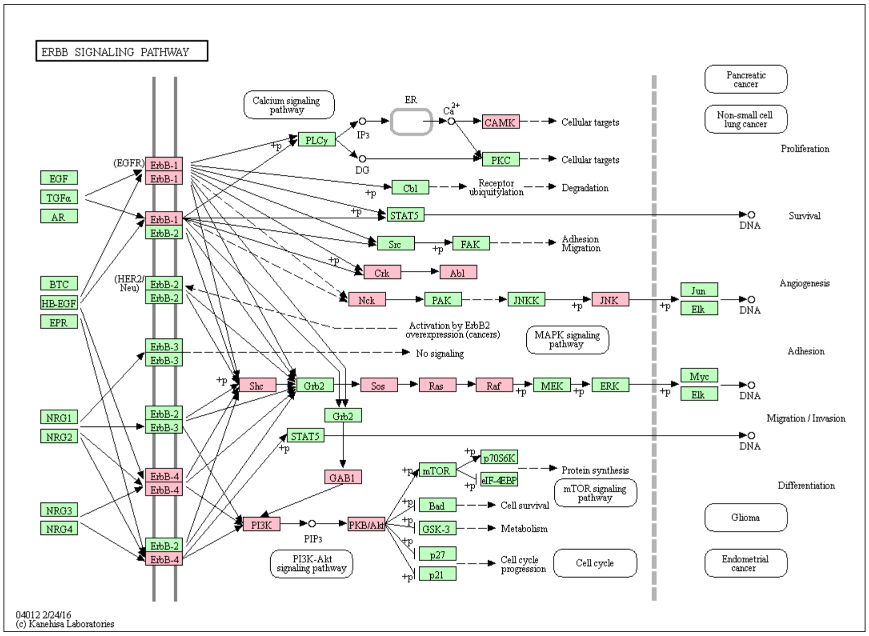

89.3 Legend:

RBH-Blast at 60% Identity + 50% Coverage
Green = Hit in *H. sapiens*
Red = Hit in *H. sapiens* and *C. milli*
White = Not in *H. sapiens*

90 Chagas disease (American trypanosomiasis)

90.1 Human Pathway: HSA05142

90.2 Number of Hits: 19

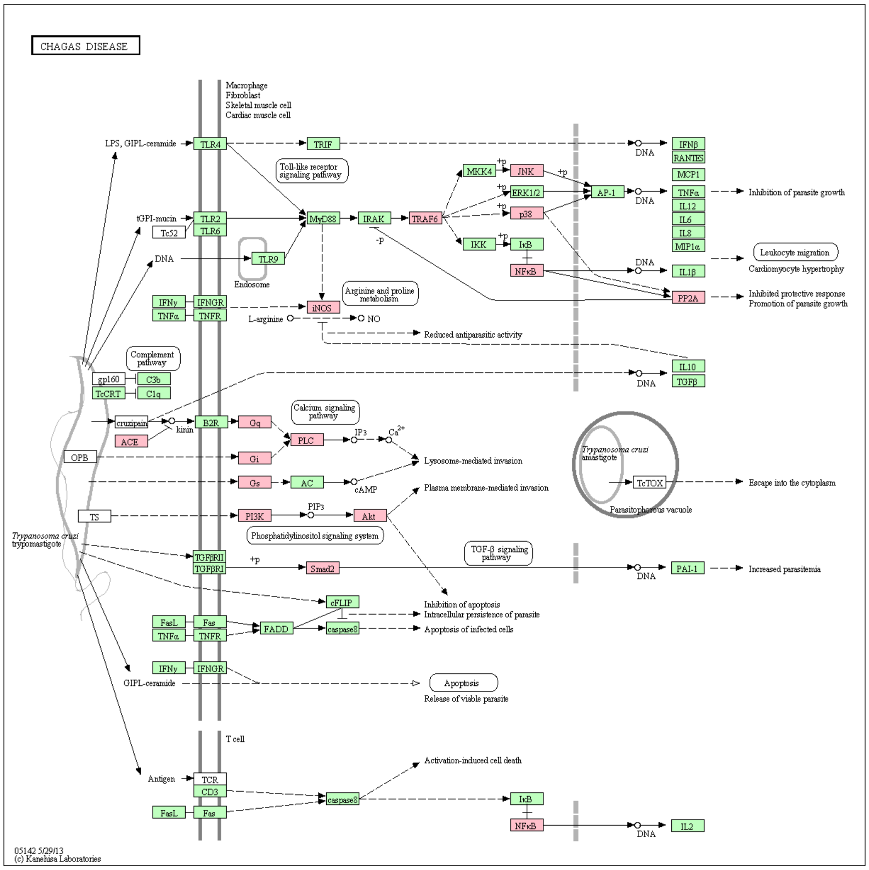

90.3 Legend:

RBH-Blast at 60% Identity + 50% Coverage
Green = Hit in *H. sapiens*
Red = Hit in *H. sapiens* and *C. milli*
White = Not in *H. sapiens*

91 Fc gamma R-mediated phagocytosis

91.1 Human Pathway: HSA04666

91.2 Number of Hits: 19

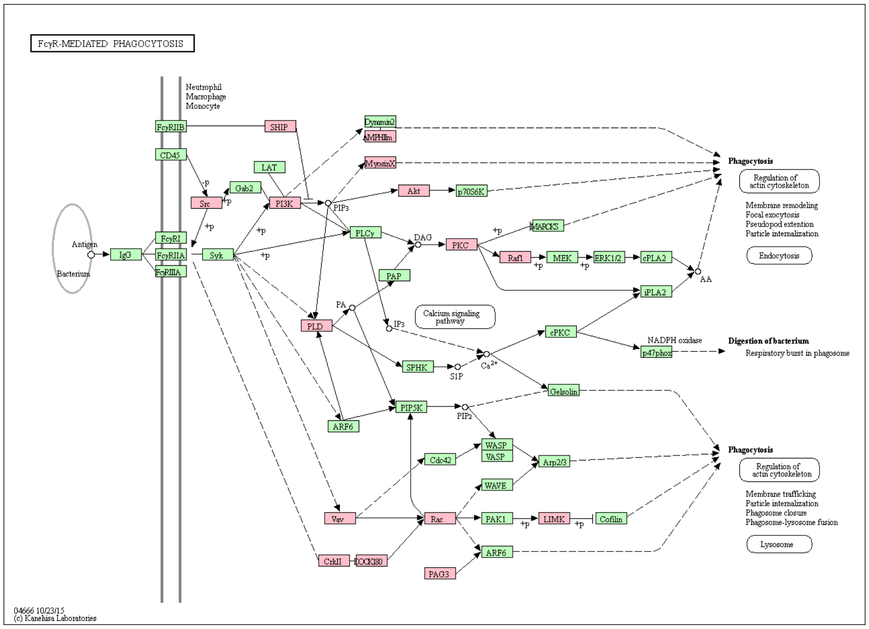

91.3 Legend:

RBH-Blast at 60% Identity + 50% Coverage
Green = Hit in *H. sapiens*
Red = Hit in *H. sapiens* and *C. milli*
White = Not in *H. sapiens*

92 Jak-STAT signaling pathway

92.1 Human Pathway: HSA04630

92.2 Number of Hits: 19

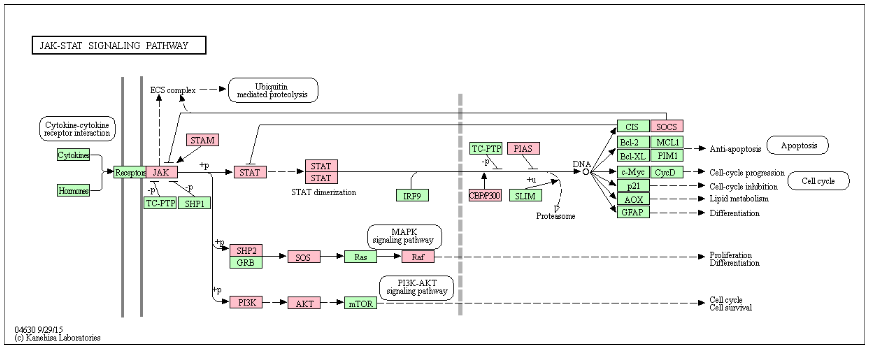

92.3 Legend:

RBH-Blast at 60% Identity + 50% Coverage
Green = Hit in *H. sapiens*
Red = Hit in *H. sapiens* and *C. milli*
White = Not in *H. sapiens*

93 Long-term depression

93.1 Human Pathway: HSA04730

93.2 Number of Hits: 19

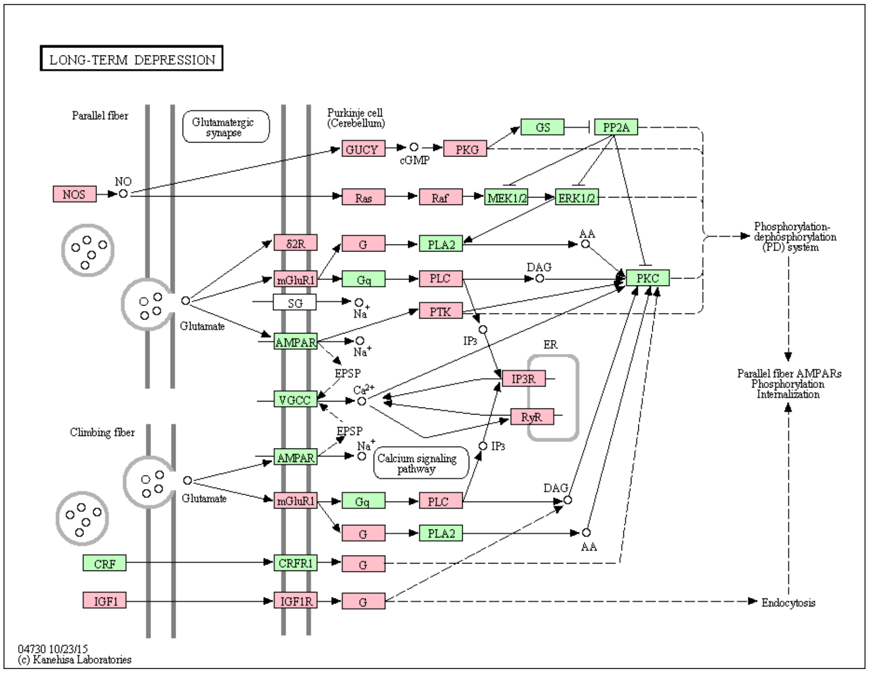

93.3 Legend:

RBH-Blast at 60% Identity + 50% Coverage
Green = Hit in *H. sapiens*
Red = Hit in *H. sapiens* and *C. milli*
White = Not in *H. sapiens*

94 Pancreatic cancer

94.1 Human Pathway: HSA05212

94.2 Number of Hits: 18

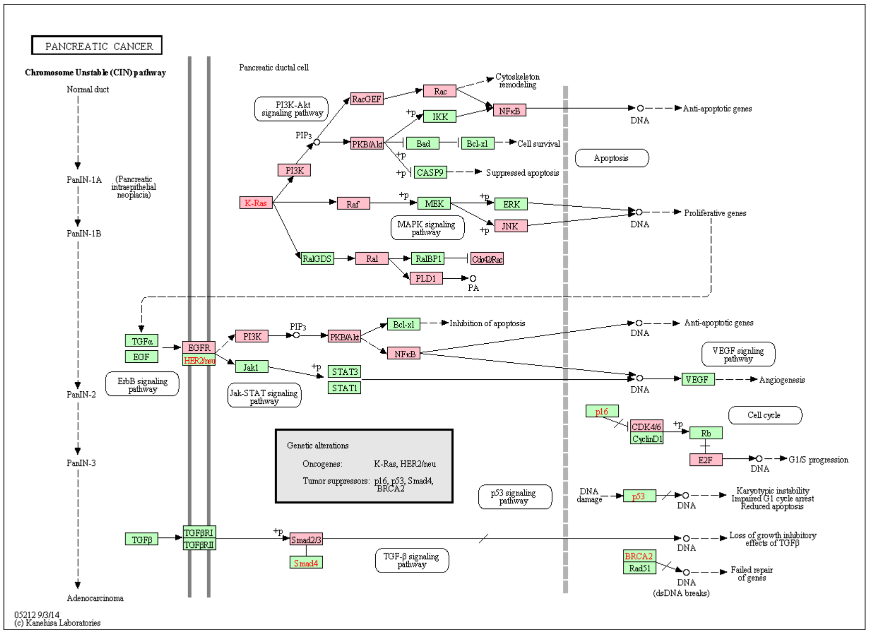

94.3 Legend:

RBH-Blast at 60% Identity + 50% Coverage
Green = Hit in *H. sapiens*
Red = Hit in *H. sapiens* and *C. milli*
White = Not in *H. sapiens*

95 Peroxisome

95.1 Human Pathway: HSA04146

95.2 Number of Hits: 18

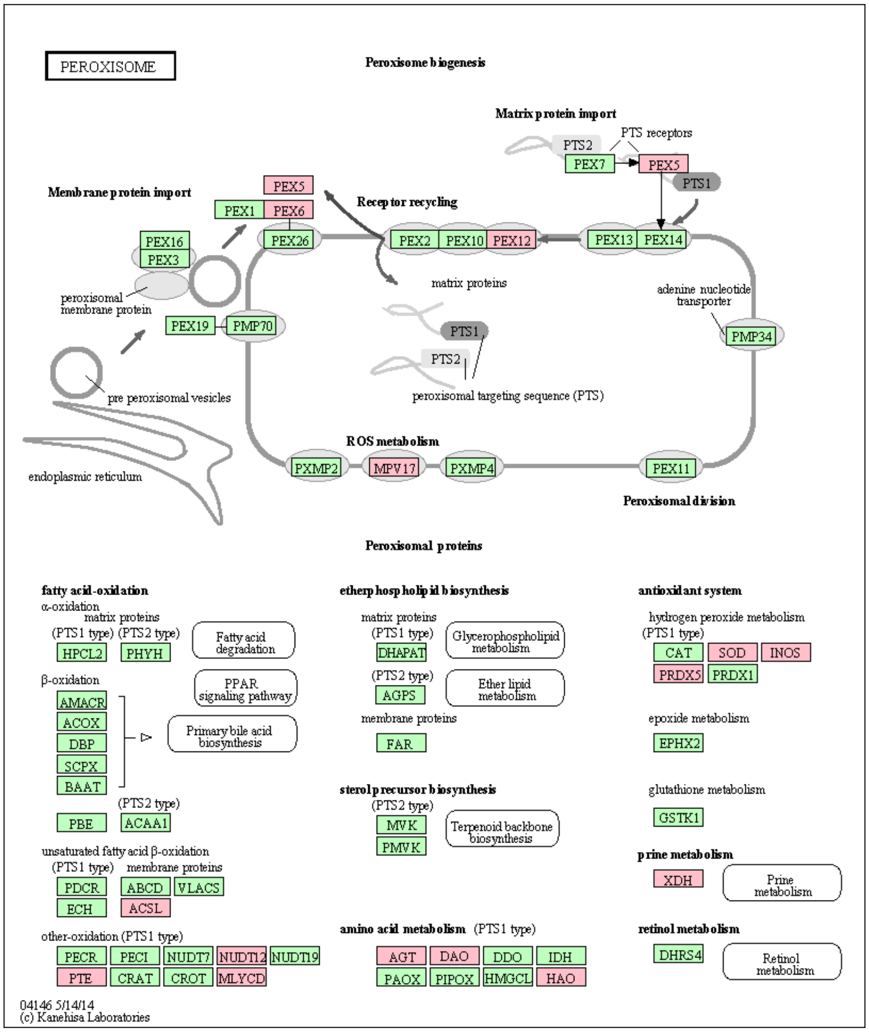

95.3 Legend:

RBH-Blast at 60% Identity + 50% Coverage
Green = Hit in *H. sapiens*
Red = Hit in *H. sapiens* and *C. milli*
White = Not in *H. sapiens*

96 Glioma

96.1 Human Pathway: HSA05214

96.2 Number of Hits: 18

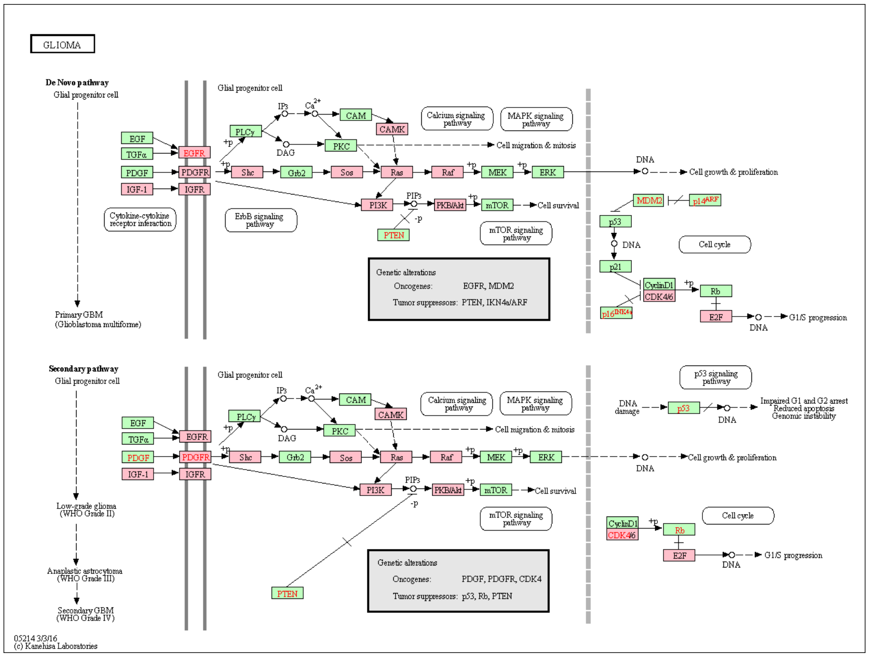

96.3 Legend:

RBH-Blast at 60% Identity + 50% Coverage
Green = Hit in *H. sapiens*
Red = Hit in *H. sapiens* and *C. milli*
White = Not in *H. sapiens*

97 Long-term potentiation

97.1 Human Pathway: HSA04720

97.2 Number of Hits: 18

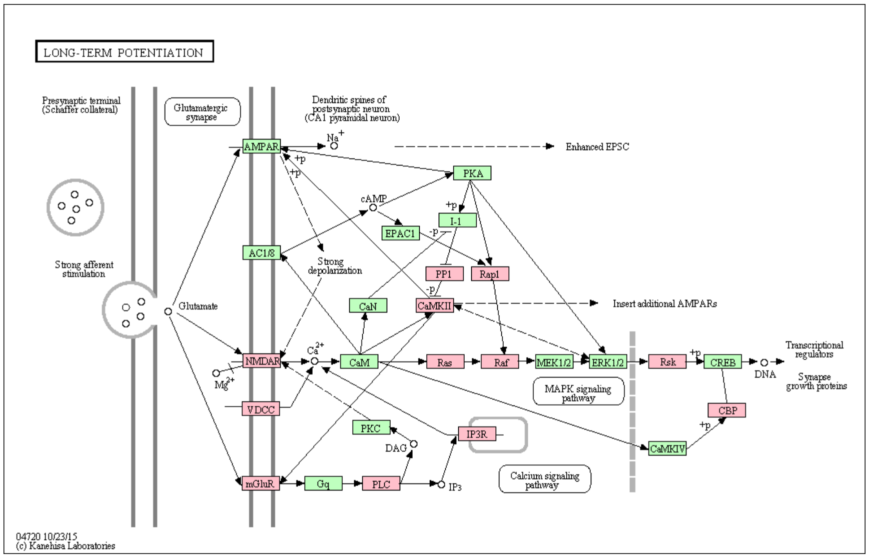

97.3 Legend:

RBH-Blast at 60% Identity + 50% Coverage
Green = Hit in *H. sapiens*
Red = Hit in *H. sapiens* and *C. milli*
White = Not in *H. sapiens*

98 Ribosome

98.1 Human Pathway: HSA03010

98.2 Number of Hits: 18

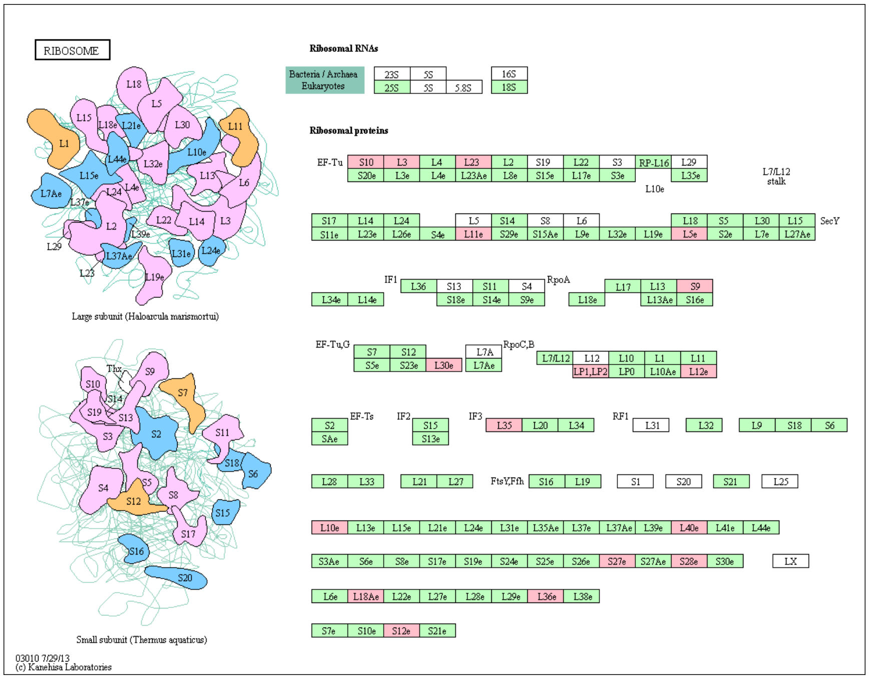

98.3 Legend:

RBH-Blast at 60% Identity + 50% Coverage
Green = Hit in *H. sapiens*
Red = Hit in *H. sapiens* and *C. milli*
White = Not in *H. sapiens*

99 Hypertrophic cardiomyopathy (HCM)

99.1 Human Pathway: HSA05410

99.2 Number of Hits: 18

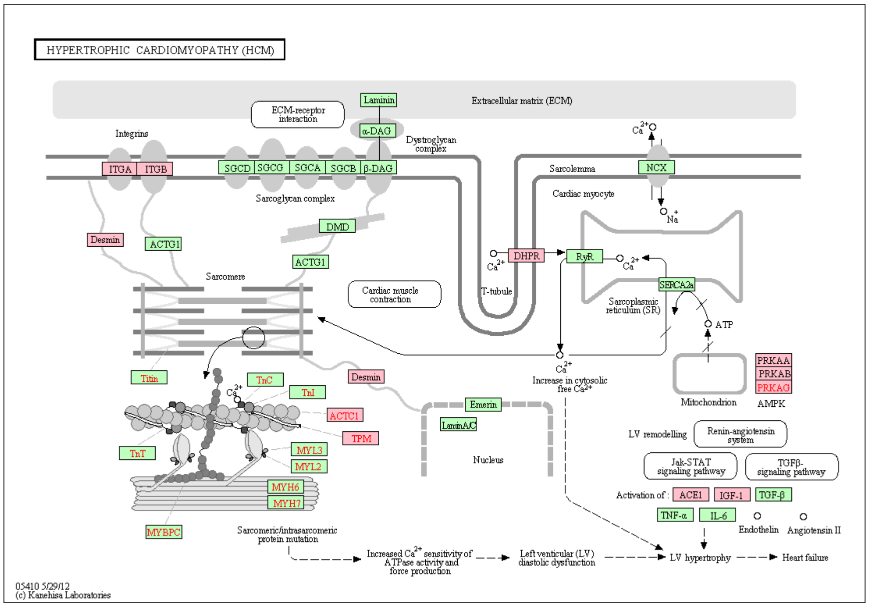

99.3 Legend:

RBH-Blast at 60% Identity + 50% Coverage
Green = Hit in *H. sapiens*
Red = Hit in *H. sapiens* and *C. milli*
White = Not in *H. sapiens*

100 Phagosome

100.1 Human Pathway: HSA04145

100.2 Number of Hits: 18

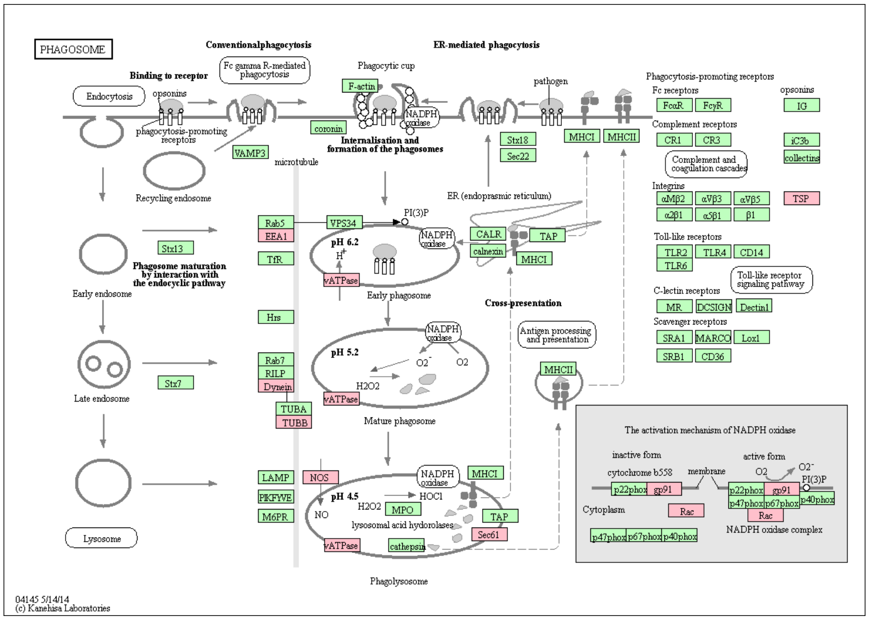

100.3 Legend:

RBH-Blast at 60% Identity + 50% Coverage
Green = Hit in *H. sapiens*
Red = Hit in *H. sapiens* and *C. milli*
White = Not in *H. sapiens*

101 Bile secretion

101.1 Human Pathway: HSA04976

101.2 Number of Hits: 18

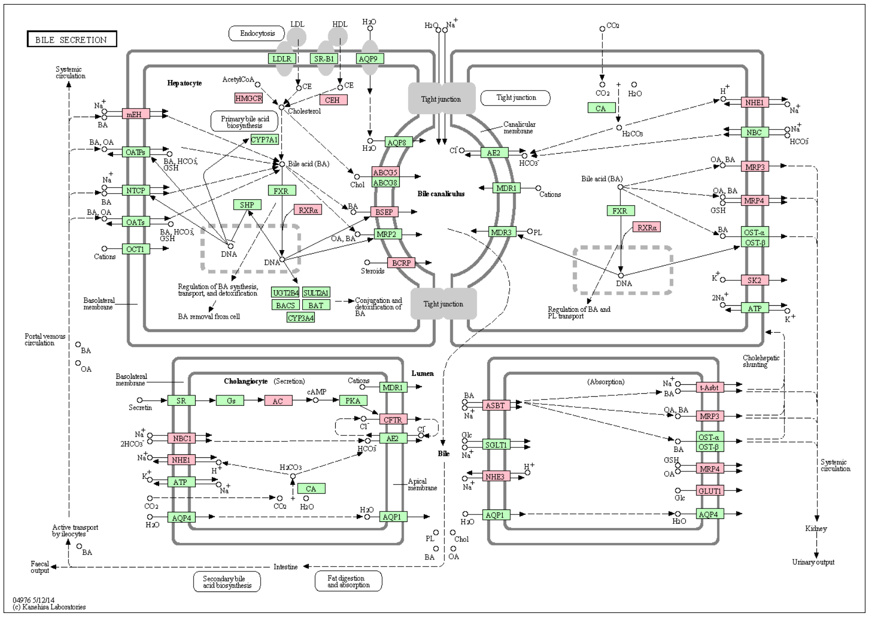

101.3 Legend:

RBH-Blast at 60% Identity + 50% Coverage
Green = Hit in *H. sapiens*
Red = Hit in *H. sapiens* and *C. milli*
White = Not in *H. sapiens*

102 Dilated cardiomyopathy

102.1 Human Pathway: HSA05414

102.2 Number of Hits: 18

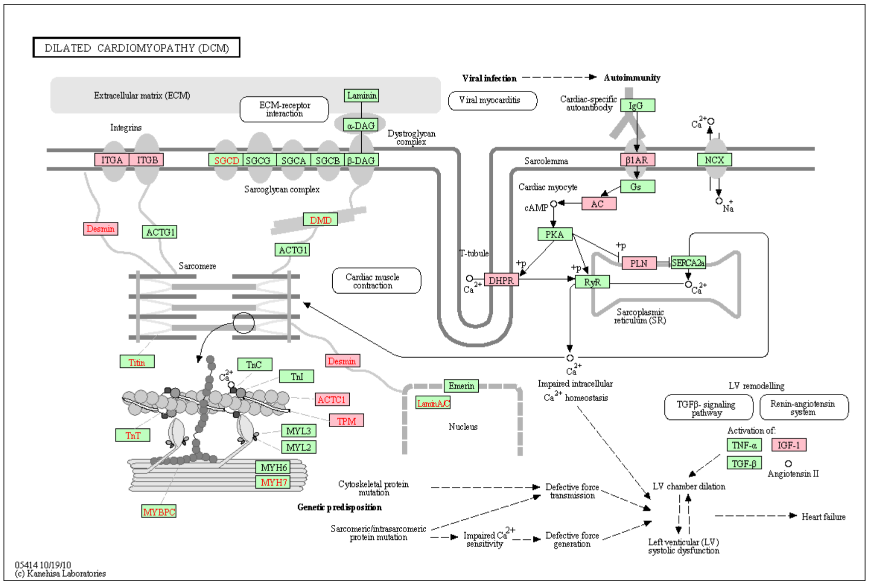

102.3 Legend:

RBH-Blast at 60% Identity + 50% Coverage
Green = Hit in *H. sapiens*
Red = Hit in *H. sapiens* and *C. milli*
White = Not in *H. sapiens*

103 Tuberculosis

103.1 Human Pathway: HSA05152

103.2 Number of Hits: 18

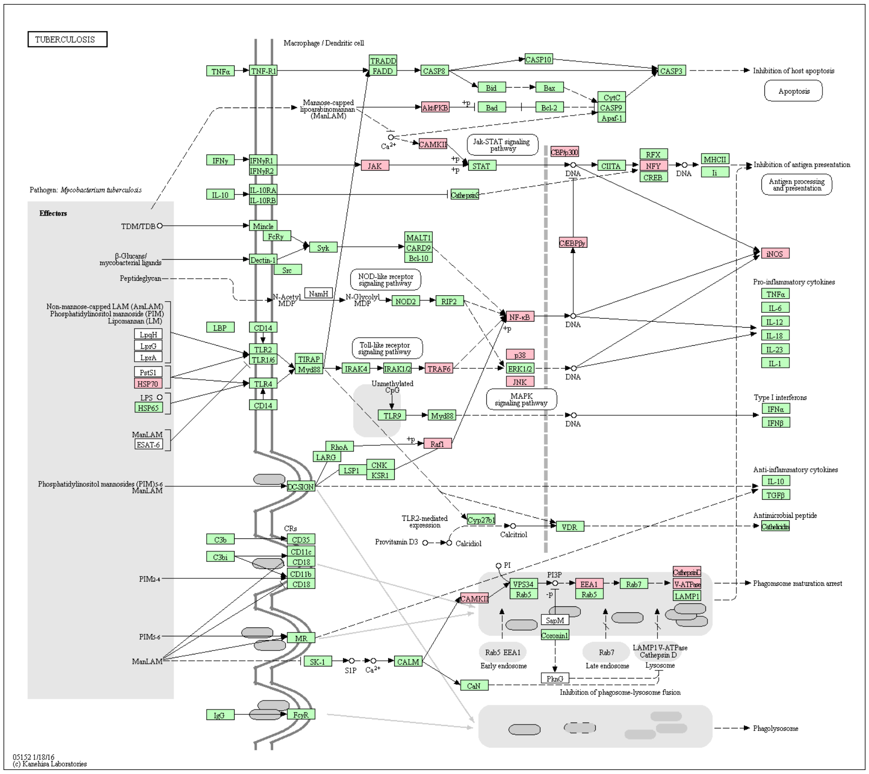

103.3 Legend:

RBH-Blast at 60% Identity + 50% Coverage
Green = Hit in *H. sapiens*
Red = Hit in *H. sapiens* and *C. milli*
White = Not in *H. sapiens*

104 Toxoplasmosis

104.1 Human Pathway: HSA05145

104.2 Number of Hits: 18

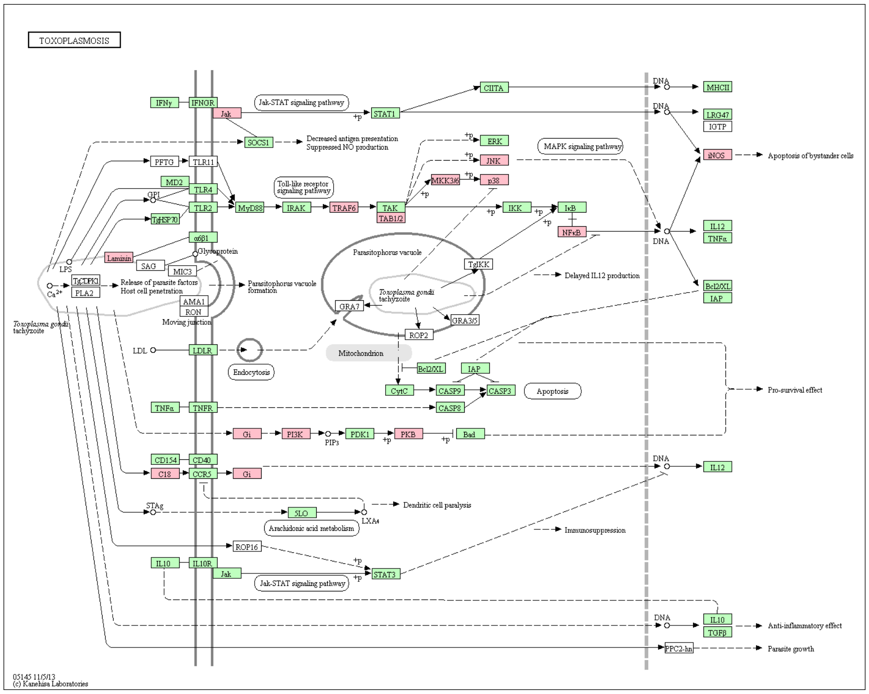

104.3 Legend:

RBH-Blast at 60% Identity + 50% Coverage
Green = Hit in *H. sapiens*
Red = Hit in *H. sapiens* and *C. milli*
White = Not in *H. sapiens*

105 Oxidative phosphorylation

105.1 Human Pathway: HSA00190

105.2 Number of Hits: 18

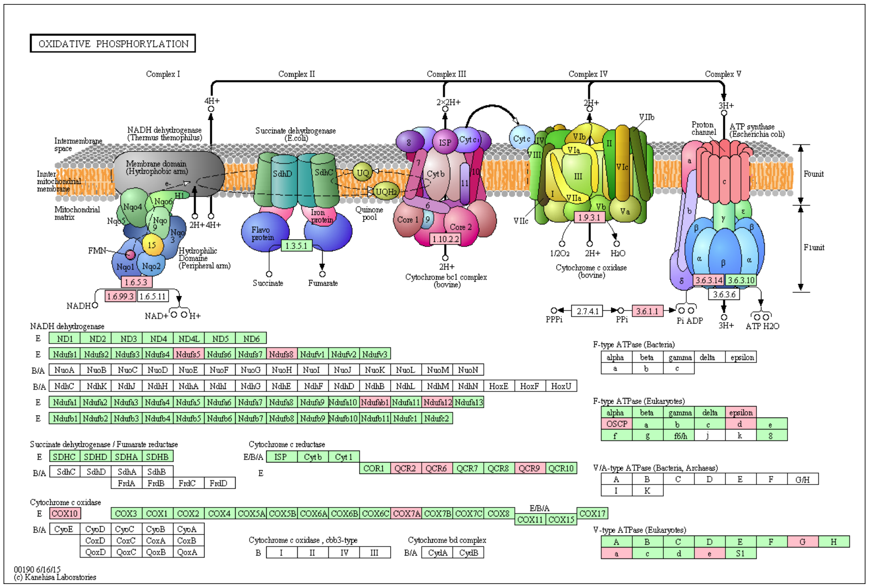

105.3 Legend:

RBH-Blast at 60% Identity + 50% Coverage
Green = Hit in *H. sapiens*
Red = Hit in *H. sapiens* and *C. milli*
White = Not in *H. sapiens*

106 mRNA surveillance pathway

106.1 Human Pathway: HSA03015

106.2 Number of Hits: 17

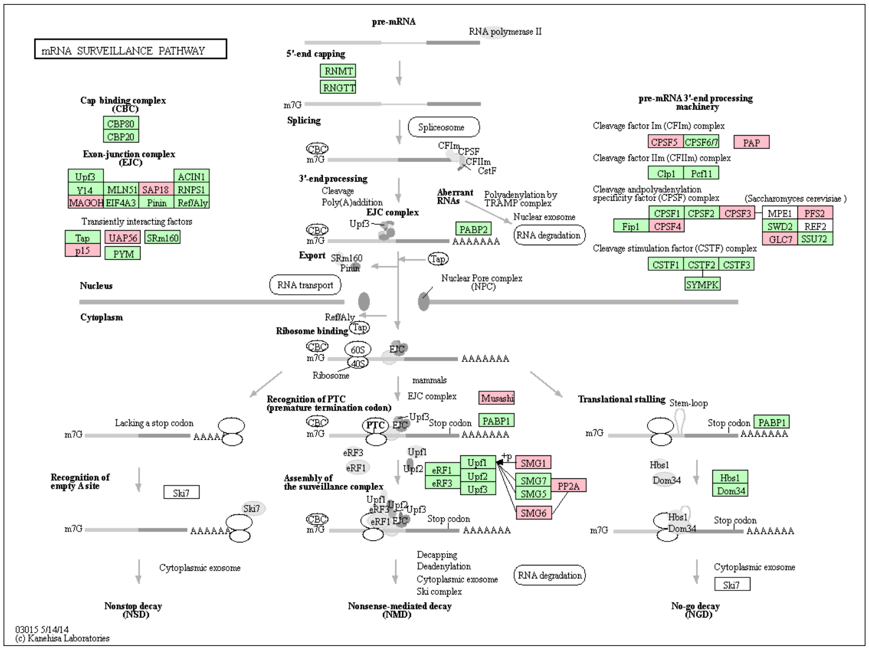

106.3 Legend:

RBH-Blast at 60% Identity + 50% Coverage
Green = Hit in *H. sapiens*
Red = Hit in *H. sapiens* and *C. milli*
White = Not in *H. sapiens*

107 Regulation of lipolysis in adipocytes

107.1 Human Pathway: HSA04923

107.2 Number of Hits: 17

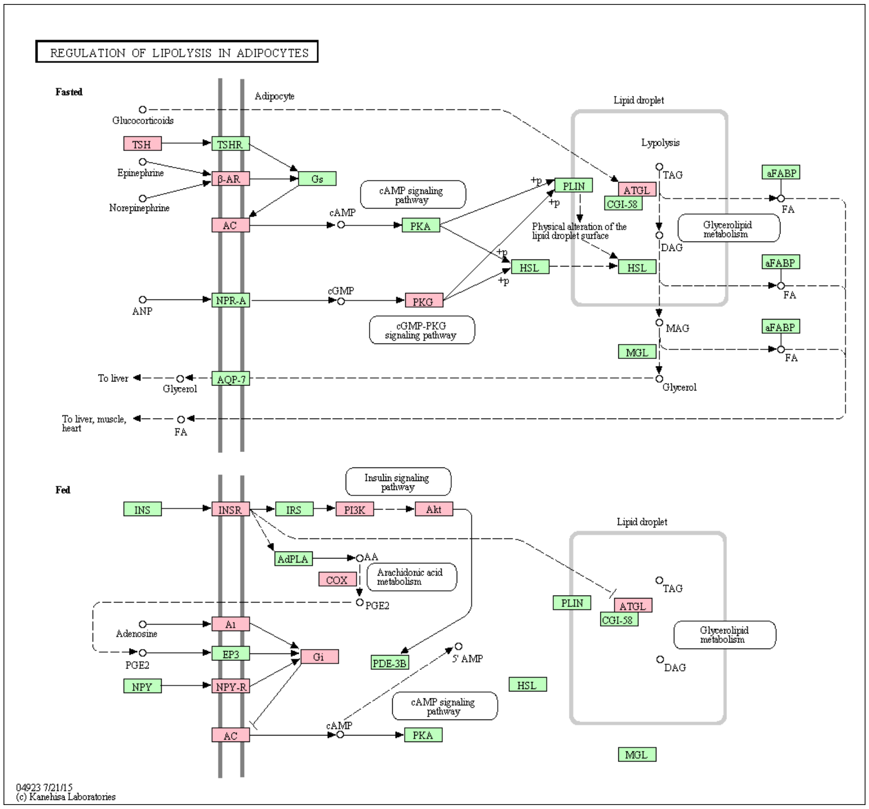

107.3 Legend:

RBH-Blast at 60% Identity + 50% Coverage
Green = Hit in *H. sapiens*
Red = Hit in *H. sapiens* and *C. milli*
White = Not in *H. sapiens*

108 Acute myeloid leukemia

108.1 Human Pathway: HSA05221

108.2 Number of Hits: 17

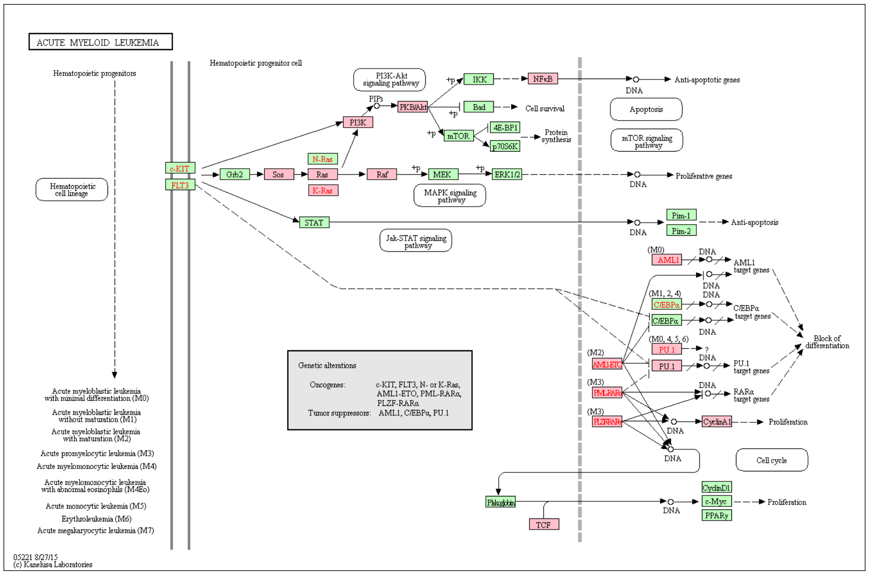

108.3 Legend:

RBH-Blast at 60% Identity + 50% Coverage
Green = Hit in *H. sapiens*
Red = Hit in *H. sapiens* and *C. milli*
White = Not in *H. sapiens*

109 Measles

109.1 Human Pathway: HSA05162

109.2 Number of Hits: 17

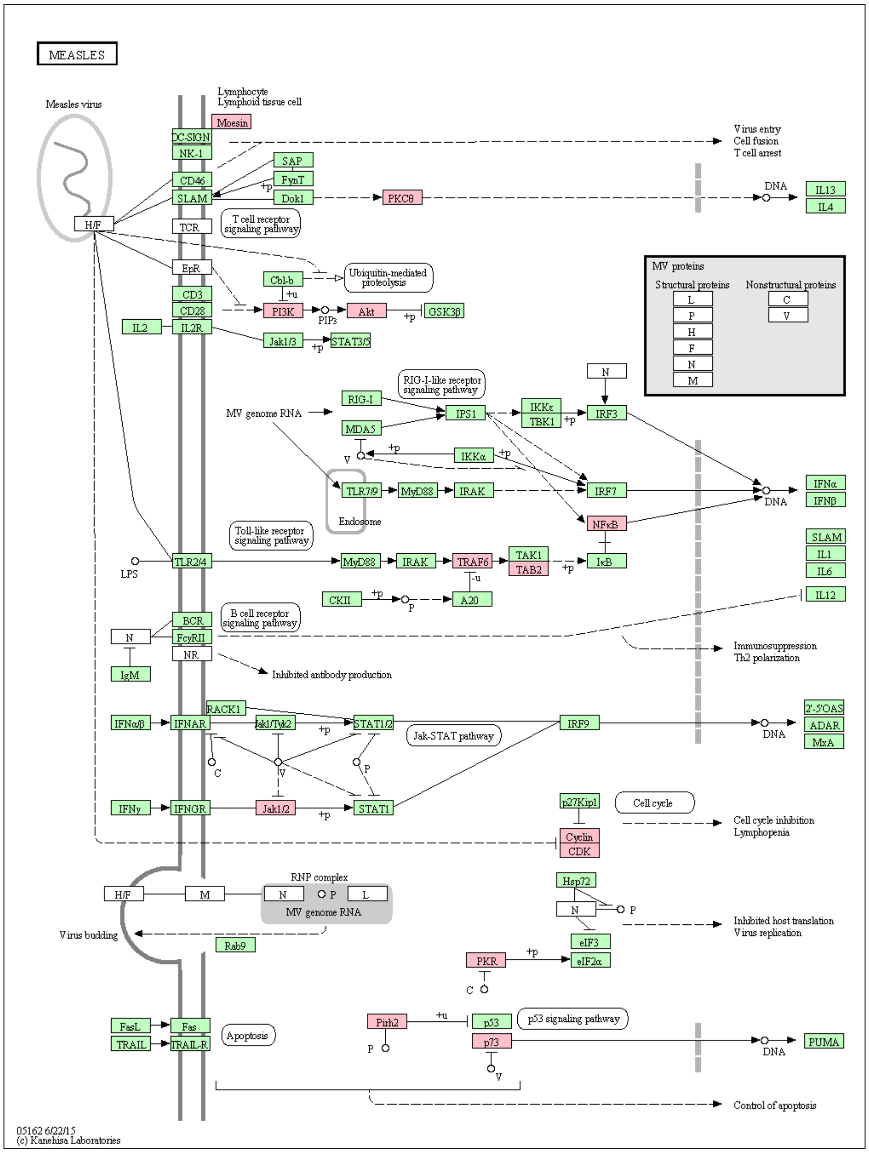

109.3 Legend:

RBH-Blast at 60% Identity + 50% Coverage
Green = Hit in *H. sapiens*
Red = Hit in *H. sapiens* and *C. milli*
White = Not in *H. sapiens*

---

110 Endometrial cancer

110.1 Human Pathway: HSA05213

110.2 Number of Hits: 17

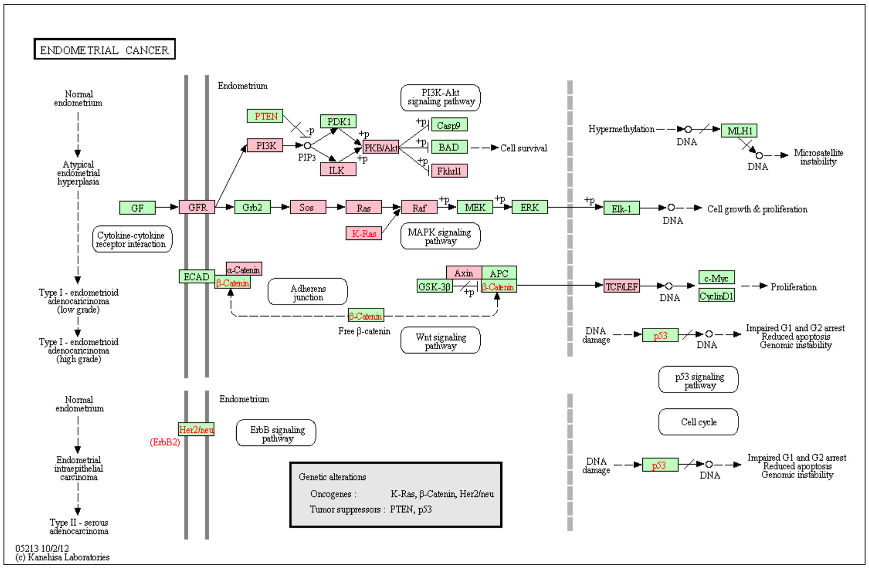

110.3 Legend:

RBH-Blast at 60% Identity + 50% Coverage
Green = Hit in *H. sapiens*
Red = Hit in *H. sapiens* and *C. milli*
White = Not in *H. sapiens*

---

111 Small cell lung cancer

111.1 Human Pathway: HSA05222

111.2 Number of Hits: 17

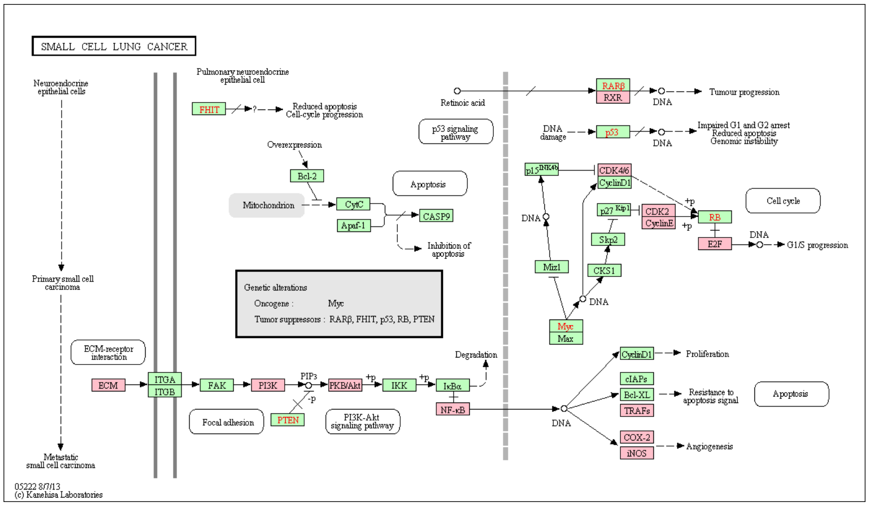

111.3 Legend:

RBH-Blast at 60% Identity + 50% Coverage
Green = Hit in *H. sapiens*
Red = Hit in *H. sapiens* and *C. milli*
White = Not in *H. sapiens*

112 Non-small cell lung cancer

112.1 Human Pathway: HSA05223

112.2 Number of Hits: 17

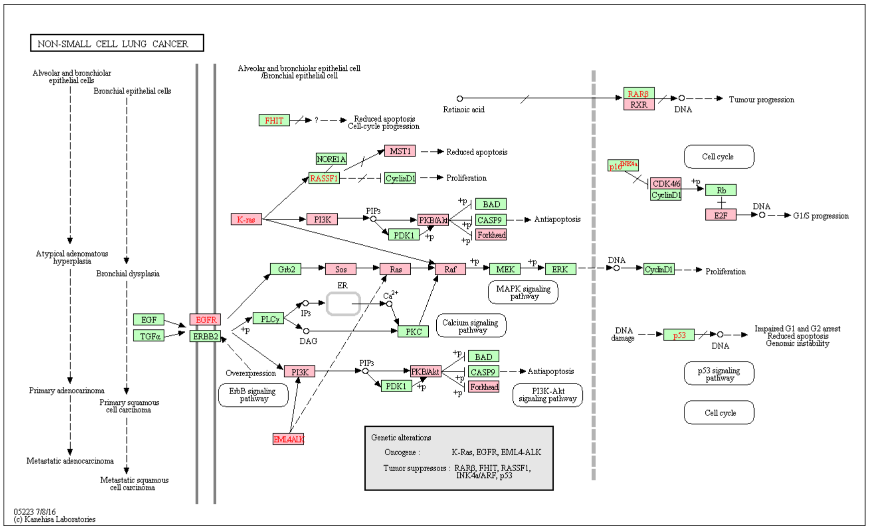

112.3 Legend:

RBH-Blast at 60% Identity + 50% Coverage
Green = Hit in *H. sapiens*
Red = Hit in *H. sapiens* and *C. milli*
White = Not in *H. sapiens*

113 Insulin secretion

113.1 Human Pathway: HSA04911

113.2 Number of Hits: 17

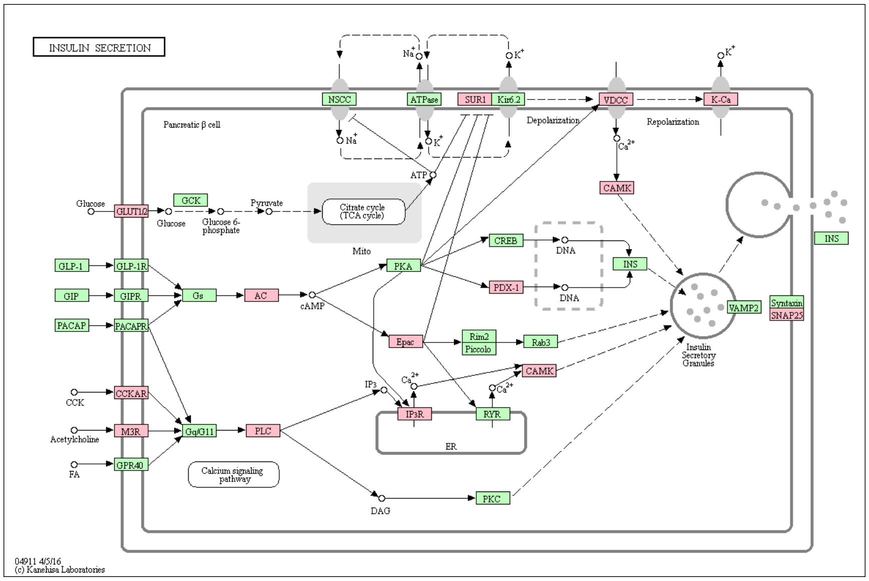

113.3 Legend:

RBH-Blast at 60% Identity + 50% Coverage
Green = Hit in *H. sapiens*
Red = Hit in *H. sapiens* and *C. milli*
White = Not in *H. sapiens*

114 TNF signaling pathway

114.1 Human Pathway: HSA04668

114.2 Number of Hits: 17

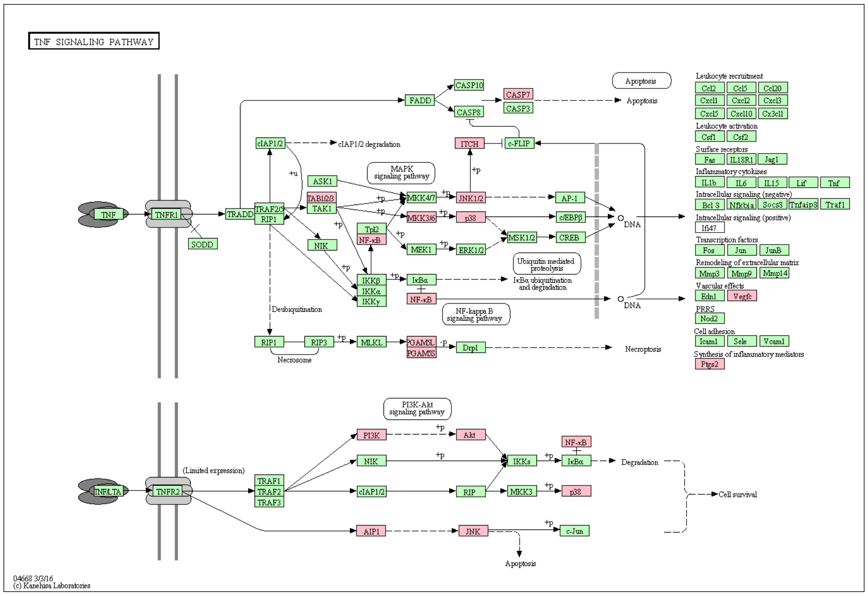

114.3 Legend:

RBH-Blast at 60% Identity + 50% Coverage
Green = Hit in *H. sapiens*
Red = Hit in *H. sapiens* and *C. milli*
White = Not in *H. sapiens*

115 Salivary secretion

115.1 Human Pathway: HSA04970

115.2 Number of Hits: 17

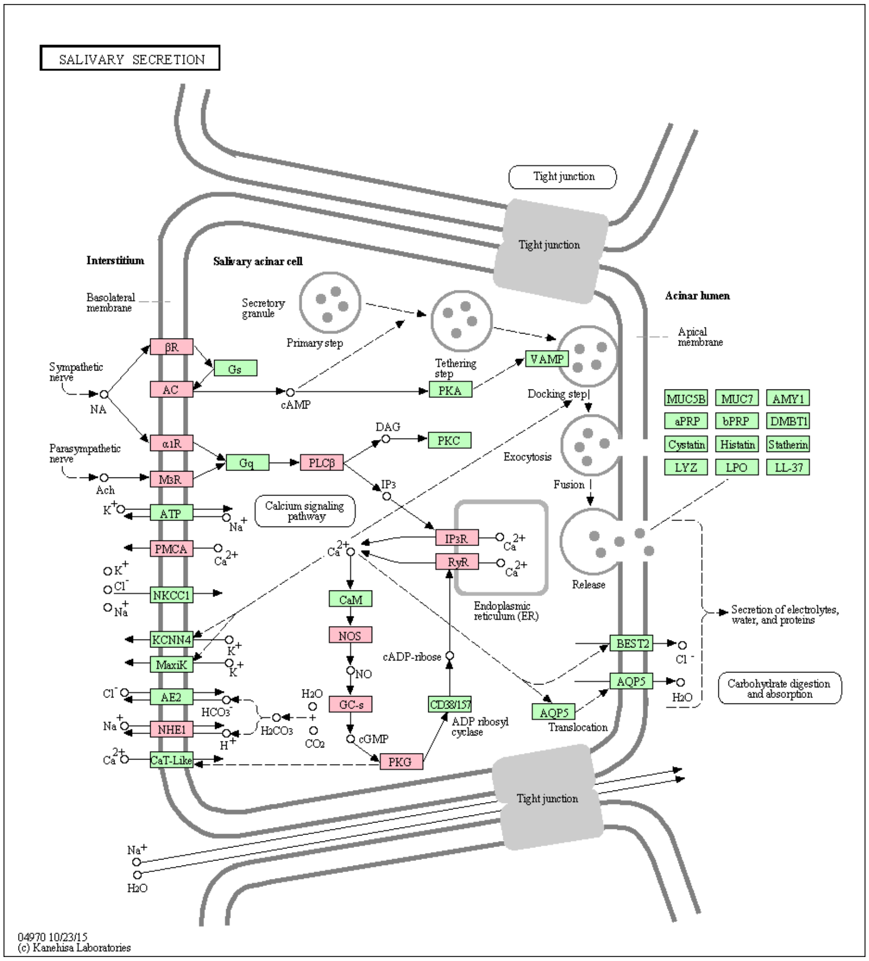

115.3 Legend:

RBH-Blast at 60% Identity + 50% Coverage
Green = Hit in *H. sapiens*
Red = Hit in *H. sapiens* and *C. milli*
White = Not in *H. sapiens*

116 Gastric acid secretion

116.1 Human Pathway: HSA04971

116.2 Number of Hits: 17

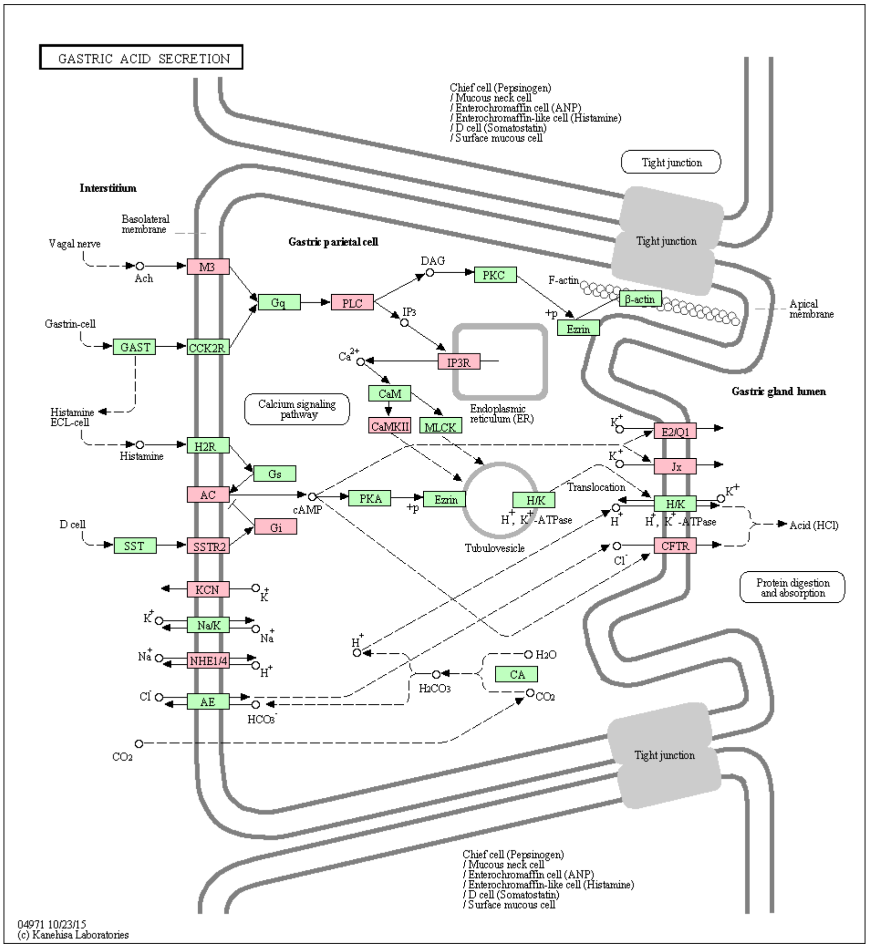

116.3 Legend:

RBH-Blast at 60% Identity + 50% Coverage
Green = Hit in *H. sapiens*
Red = Hit in *H. sapiens* and *C. milli*
White = Not in *H. sapiens*

117 Nicotine addiction

117.1 Human Pathway: HSA05033

117.2 Number of Hits: 17

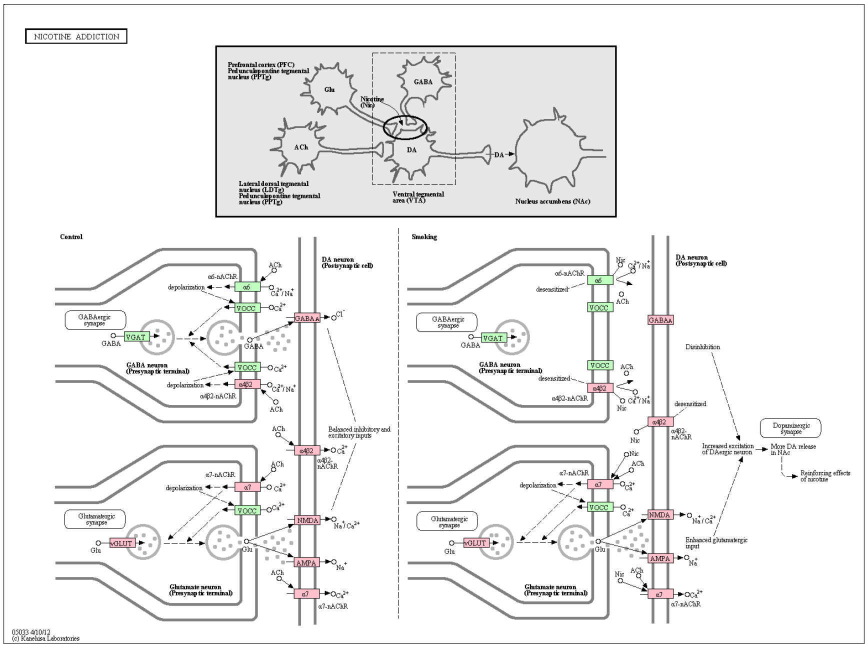

117.3 Legend:

RBH-Blast at 60% Identity + 50% Coverage
Green = Hit in *H. sapiens*
Red = Hit in *H. sapiens* and *C. milli*
White = Not in *H. sapiens*

118 Inositol phosphate metabolism

118.1 Human Pathway: HSA00562

118.2 Number of Hits: 17

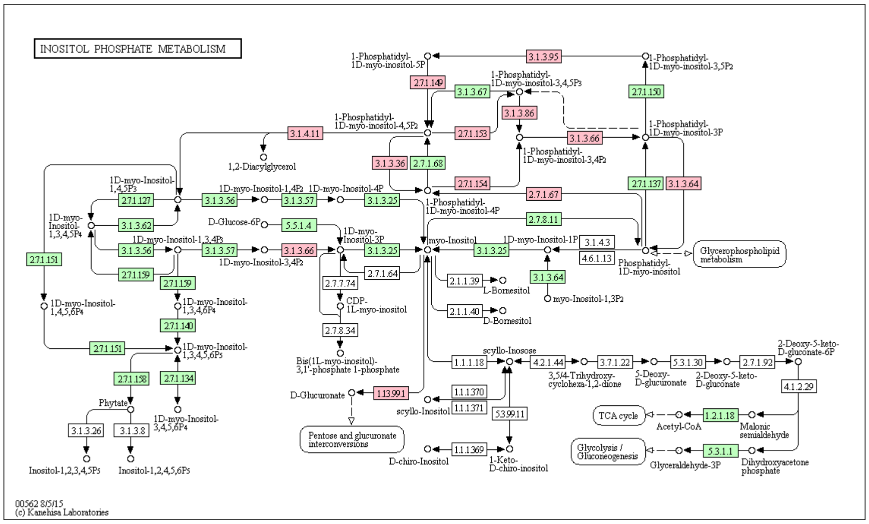

118.3 Legend:

RBH-Blast at 60% Identity + 50% Coverage
Green = Hit in *H. sapiens*
Red = Hit in *H. sapiens* and *C. milli*
White = Not in *H. sapiens*

119 Adipocytokine signaling pathway

119.1 Human Pathway: HSA04920

119.2 Number of Hits: 17

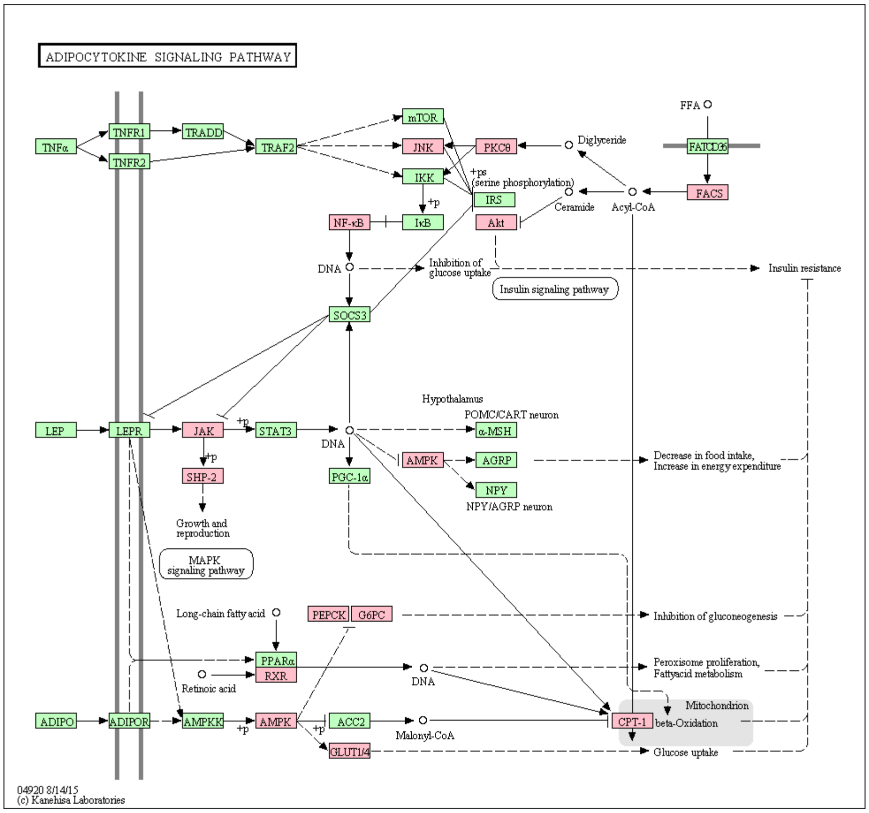

119.3 Legend:

RBH-Blast at 60% Identity + 50% Coverage
Green = Hit in *H. sapiens*
Red = Hit in *H. sapiens* and *C. milli*
White = Not in *H. sapiens*

120 Fc epsilon RI signaling pathway

120.1 Human Pathway: HSA04664

120.2 Number of Hits: 16

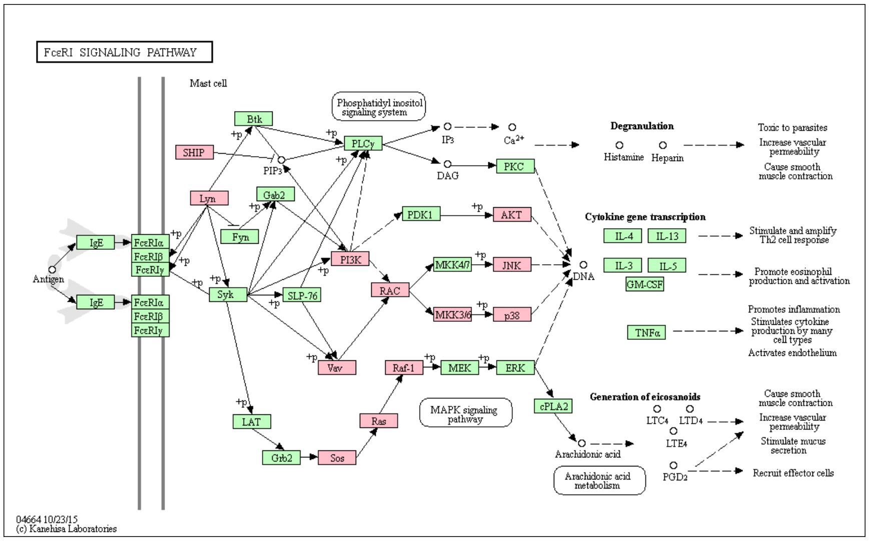

120.3 Legend:

RBH-Blast at 60% Identity + 50% Coverage
Green = Hit in *H. sapiens*
Red = Hit in *H. sapiens* and *C. milli*
White = Not in *H. sapiens*

121 Cell adhesion molecules (CAMs)

121.1 Human Pathway: HSA04514

121.2 Number of Hits: 16

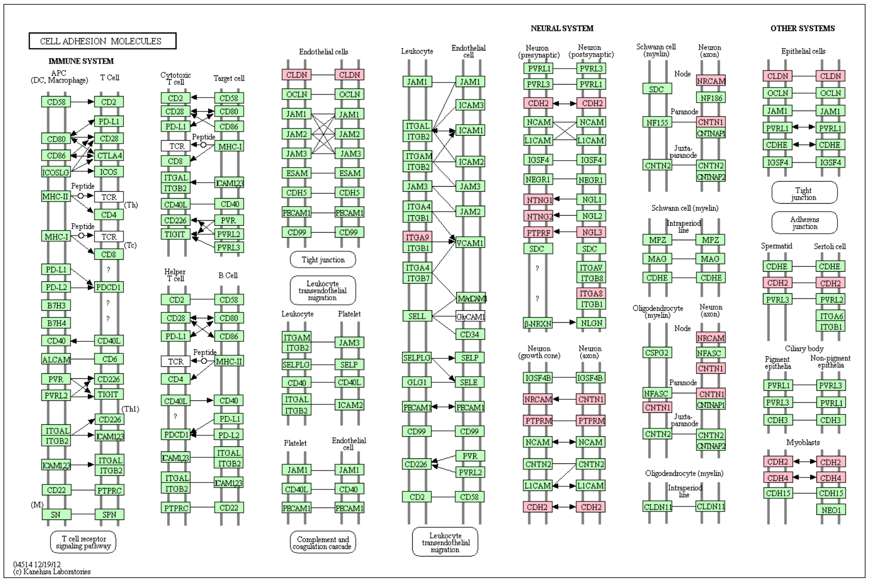

121.3 Legend:

RBH-Blast at 60% Identity + 50% Coverage
Green = Hit in *H. sapiens*
Red = Hit in *H. sapiens* and *C. milli*
White = Not in *H. sapiens*

122 Herpes simplex infection

122.1 Human Pathway: HSA05168

122.2 Number of Hits: 16

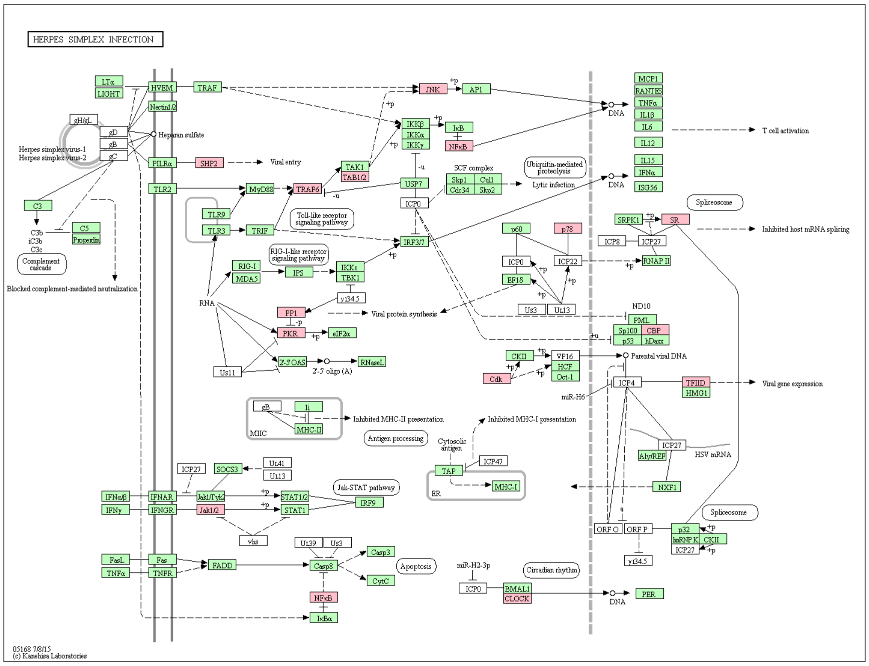

122.3 Legend:

RBH-Blast at 60% Identity + 50% Coverage
Green = Hit in *H. sapiens*
Red = Hit in *H. sapiens* and *C. milli*
White = Not in *H. sapiens*

123 Arrhythmogenic right ventricular cardiomyopathy (ARVC)

123.1 Human Pathway: HSA05412

123.2 Number of Hits: 16

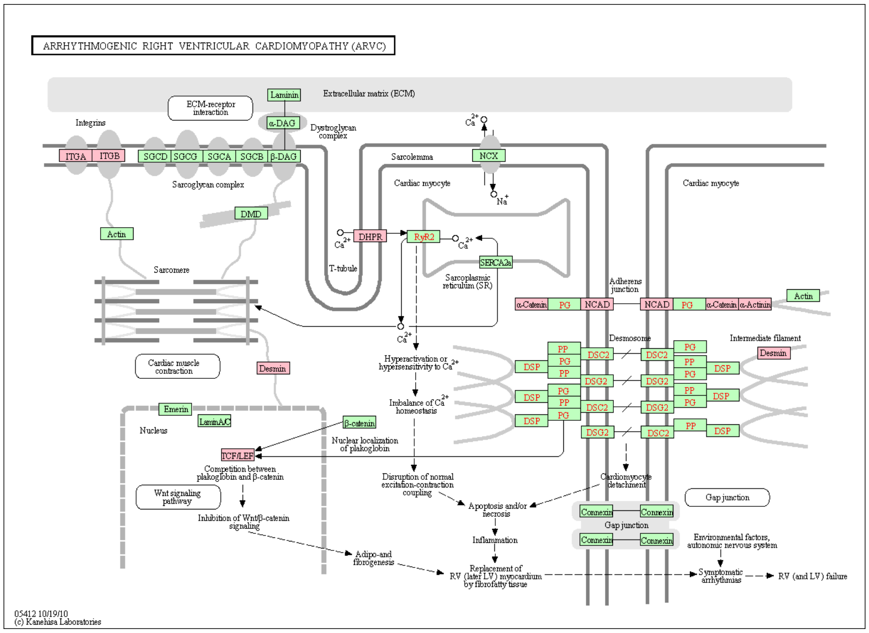

123.3 Legend:

RBH-Blast at 60% Identity + 50% Coverage
Green = Hit in *H. sapiens*
Red = Hit in *H. sapiens* and *C. milli*
White = Not in *H. sapiens*

124 Notch signaling pathway

124.1 Human Pathway: HSA04330

124.2 Number of Hits: 16

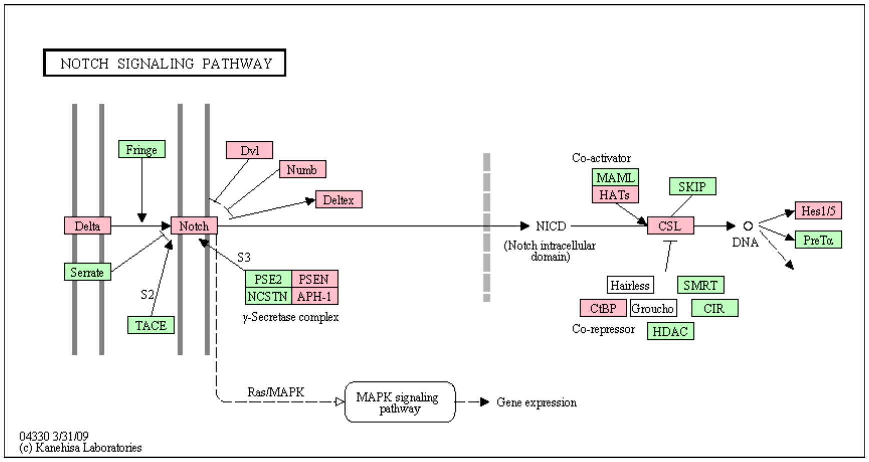

124.3 Legend:

RBH-Blast at 60% Identity + 50% Coverage
Green = Hit in *H. sapiens*
Red = Hit in *H. sapiens* and *C. milli*
White = Not in *H. sapiens*

125 Oocyte meiosis

125.1 Human Pathway: HSA04114

125.2 Number of Hits: 16

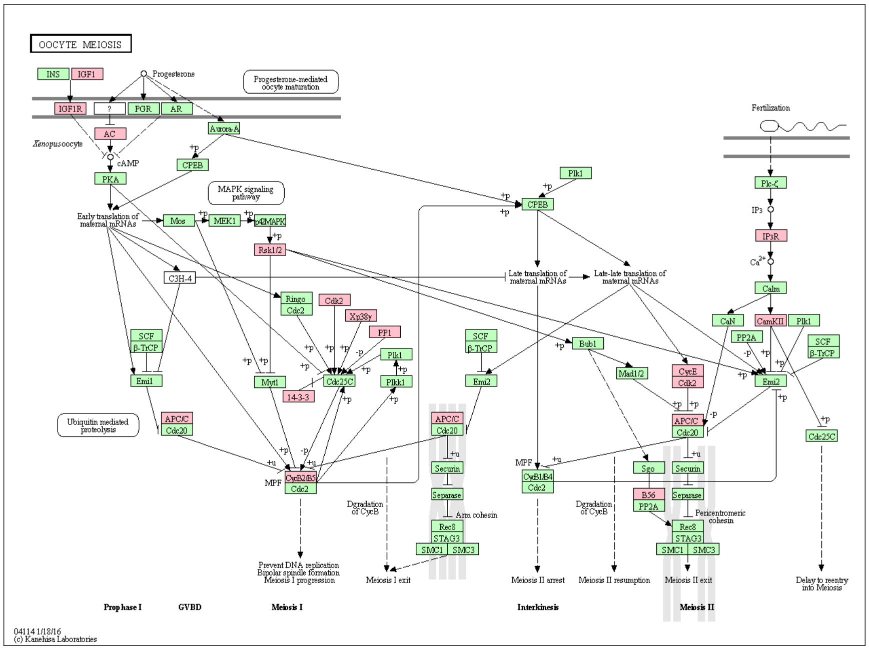

125.3 Legend:

RBH-Blast at 60% Identity + 50% Coverage
Green = Hit in *H. sapiens*
Red = Hit in *H. sapiens* and *C. milli*
White = Not in *H. sapiens*

126 B cell receptor signaling pathway

126.1 Human Pathway: HSA04662

126.2 Number of Hits: 16

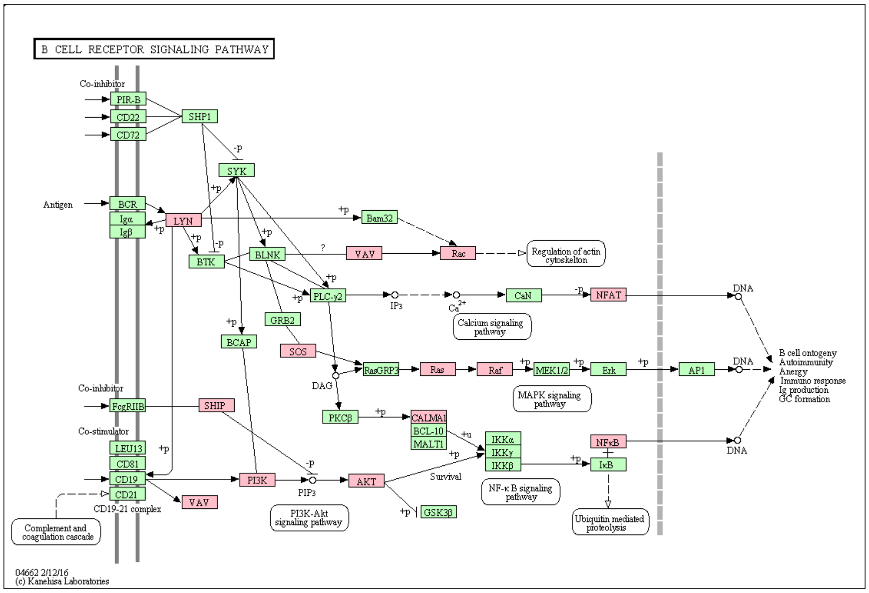

126.3 Legend:

RBH-Blast at 60% Identity + 50% Coverage
Green = Hit in *H. sapiens*
Red = Hit in *H. sapiens* and *C. milli*
White = Not in *H. sapiens*

127 p53 signaling pathway

127.1 Human Pathway: HSA04115

127.2 Number of Hits: 16

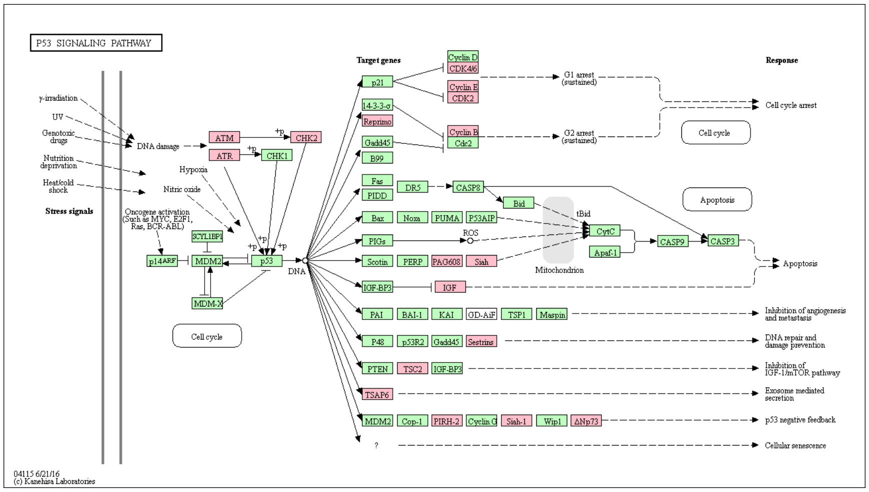

127.3 Legend:

RBH-Blast at 60% Identity + 50% Coverage
Green = Hit in *H. sapiens*
Red = Hit in *H. sapiens* and *C. milli*
White = Not in *H. sapiens*

128 TGF-beta signaling pathway

128.1 Human Pathway: HSA04350

128.2 Number of Hits: 16

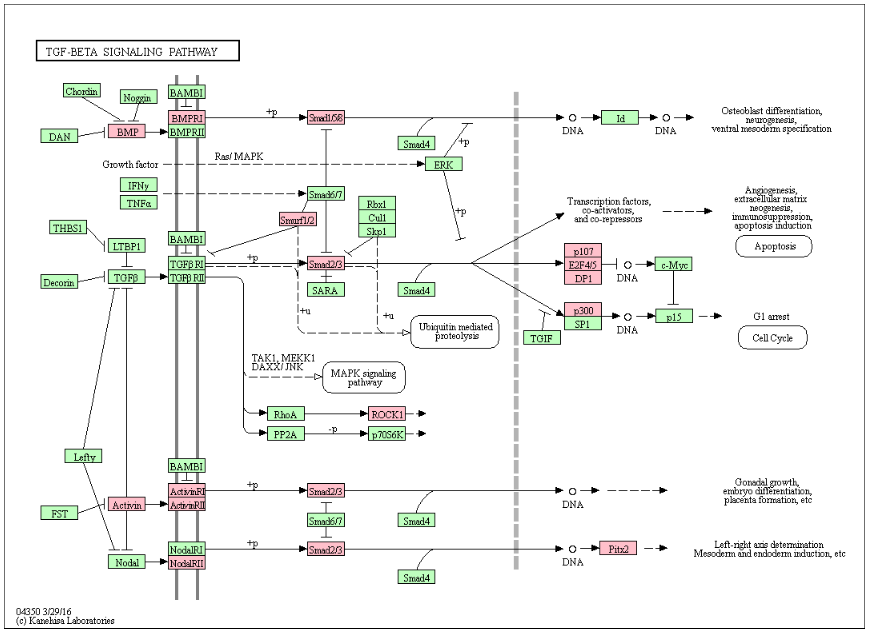

128.3 Legend:

RBH-Blast at 60% Identity + 50% Coverage
Green = Hit in *H. sapiens*
Red = Hit in *H. sapiens* and *C. milli*
White = Not in *H. sapiens*

129 Natural killer cell mediated cytotoxicity

129.1 Human Pathway: HSA04650

129.2 Number of Hits: 16

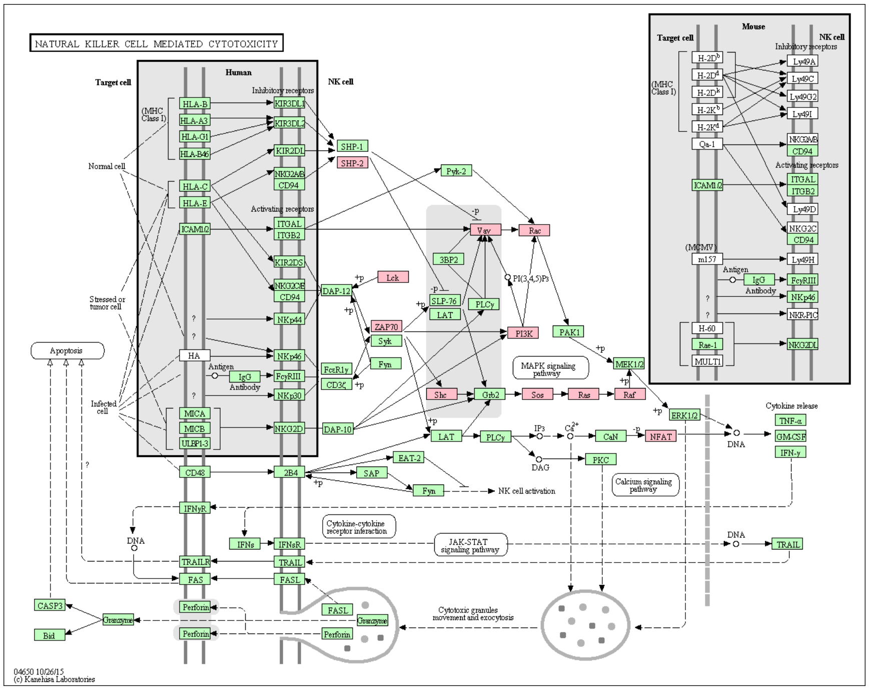

129.3 Legend:

RBH-Blast at 60% Identity + 50% Coverage
Green = Hit in *H. sapiens*
Red = Hit in *H. sapiens* and *C. milli*
White = Not in *H. sapiens*

130 Taste transduction

130.1 Human Pathway: HSA04742

130.2 Number of Hits: 16

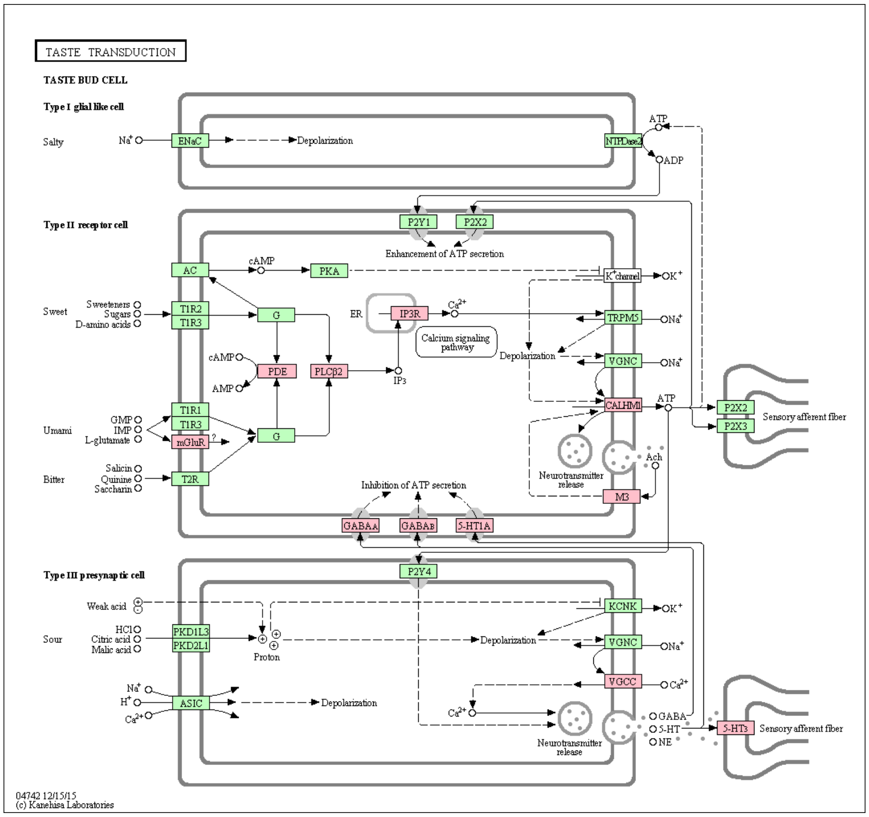

130.3 Legend:

RBH-Blast at 60% Identity + 50% Coverage
Green = Hit in *H. sapiens*
Red = Hit in *H. sapiens* and *C. milli*
White = Not in *H. sapiens*

131 N-Glycan biosynthesis

131.1 Human Pathway: HSA00510

131.2 Number of Hits: 15

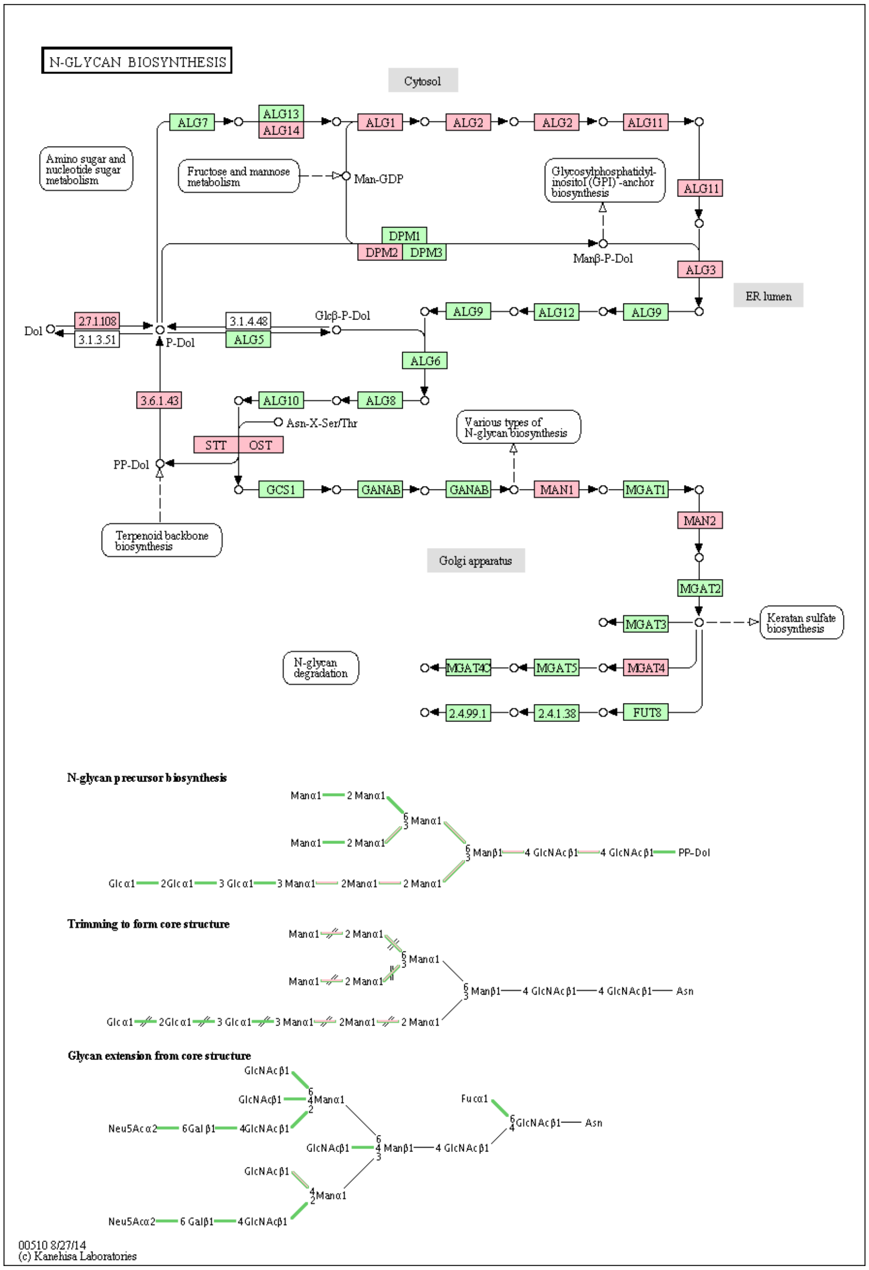

131.3 Legend:

RBH-Blast at 60% Identity + 50% Coverage
Green = Hit in *H. sapiens*
Red = Hit in *H. sapiens* and *C. milli*
White = Not in *H. sapiens*

---

132 Renin secretion

132.1 Human Pathway: HSA04924

132.2 Number of Hits: 15

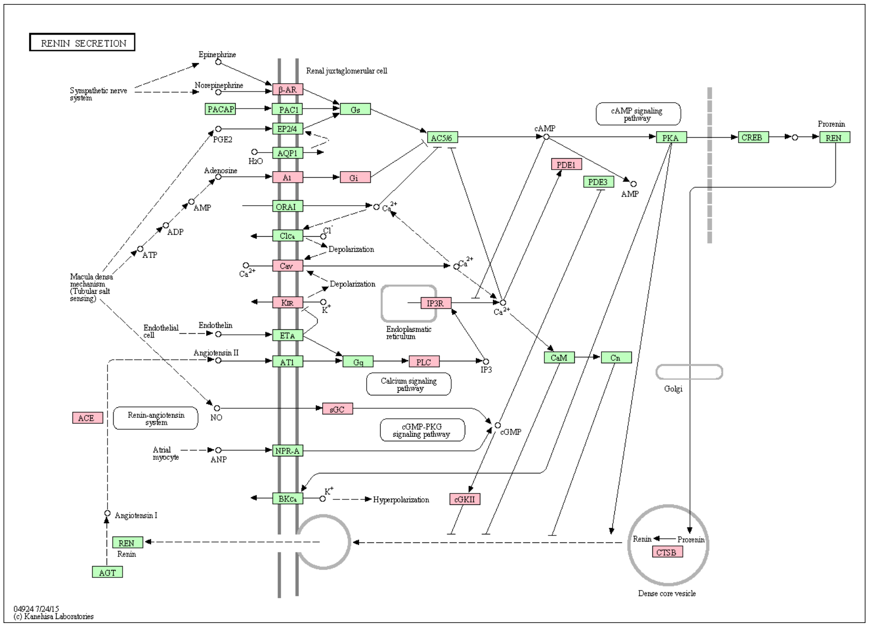

132.3 Legend:

RBH-Blast at 60% Identity + 50% Coverage
Green = Hit in *H. sapiens*
Red = Hit in *H. sapiens* and *C. milli*
White = Not in *H. sapiens*

---

133 Amoebiasis

133.1 Human Pathway: HSA05146

133.2 Number of Hits: 15

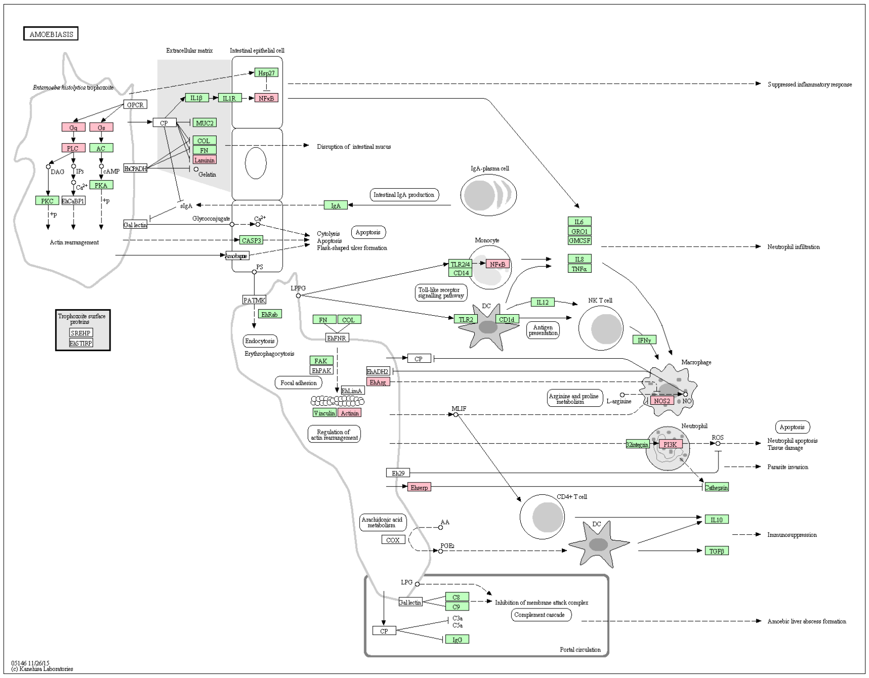

133.3 Legend:

RBH-Blast at 60% Identity + 50% Coverage
Green = Hit in *H. sapiens*
Red = Hit in *H. sapiens* and *C. milli*
White = Not in *H. sapiens*

134 Type II diabetes mellitus

134.1 Human Pathway: HSA04930

134.2 Number of Hits: 15

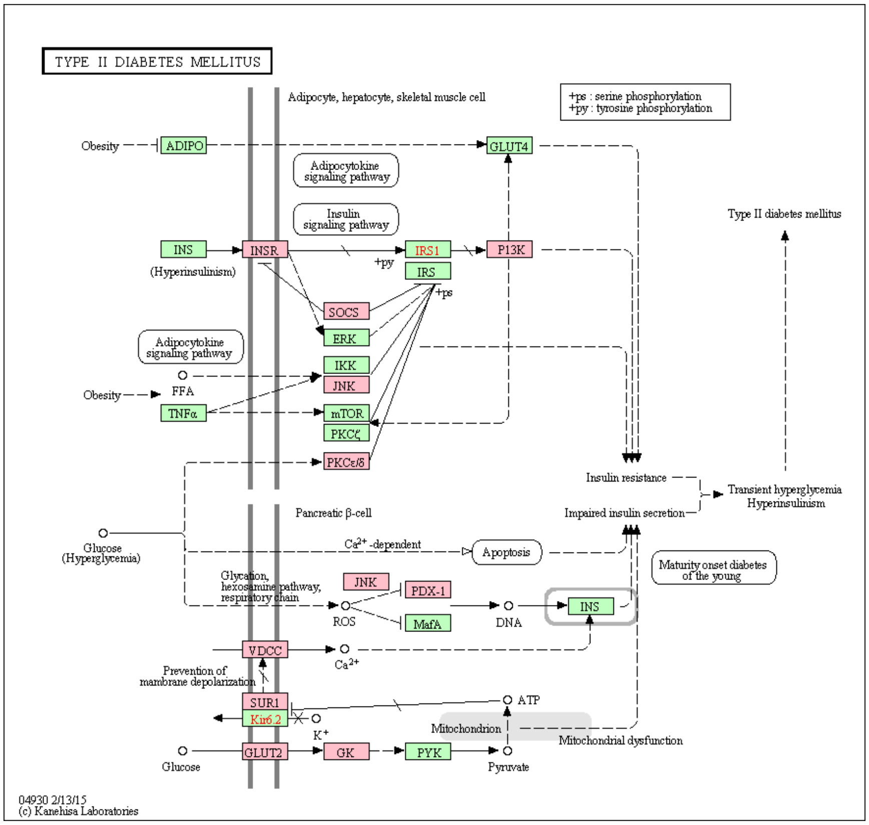

134.3 Legend:

RBH-Blast at 60% Identity + 50% Coverage
Green = Hit in *H. sapiens*
Red = Hit in *H. sapiens* and *C. milli*
White = Not in *H. sapiens*

135 Sphingolipid metabolism

135.1 Human Pathway: HSA00600

135.2 Number of Hits: 15

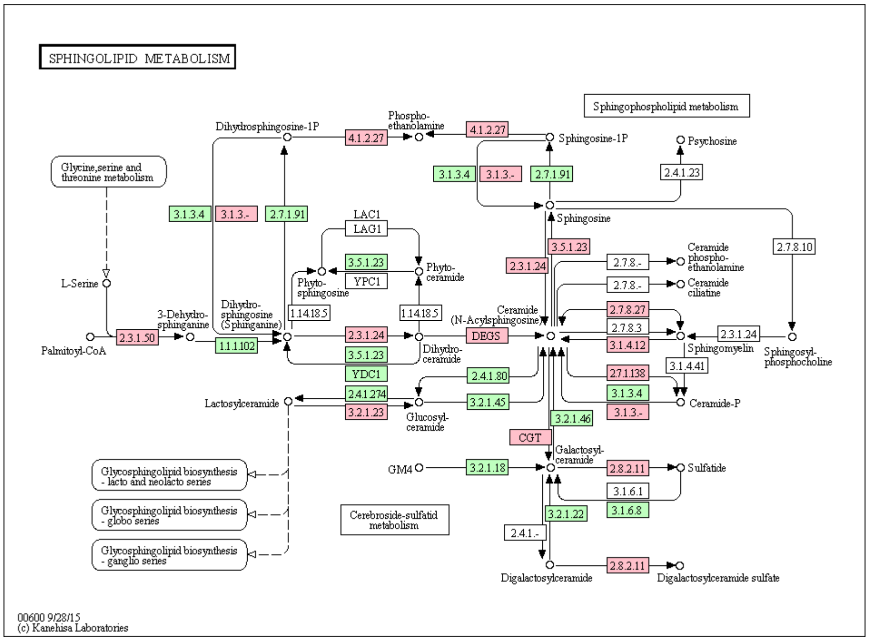

135.3 Legend:

RBH-Blast at 60% Identity + 50% Coverage
Green = Hit in *H. sapiens*
Red = Hit in *H. sapiens* and *C. milli*
White = Not in *H. sapiens*

136 Aldosterone synthesis and secretion

136.1 Human Pathway: HSA04925

136.2 Number of Hits: 15

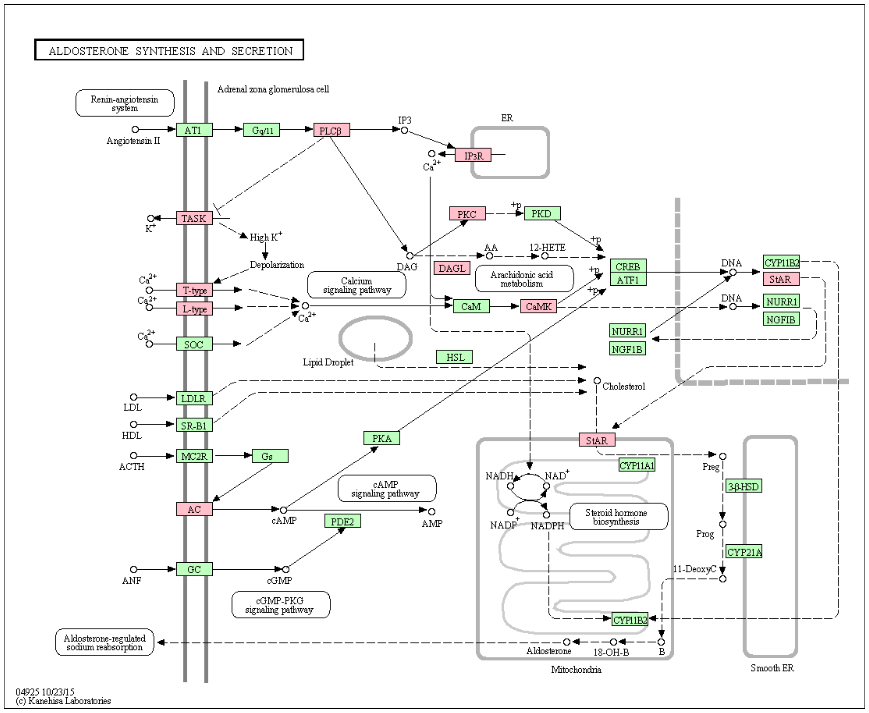

136.3 Legend:

RBH-Blast at 60% Identity + 50% Coverage
Green = Hit in *H. sapiens*
Red = Hit in *H. sapiens* and *C. milli*
White = Not in *H. sapiens*

137 Protein digestion and absorption

137.1 Human Pathway: HSA04974

137.2 Number of Hits: 15

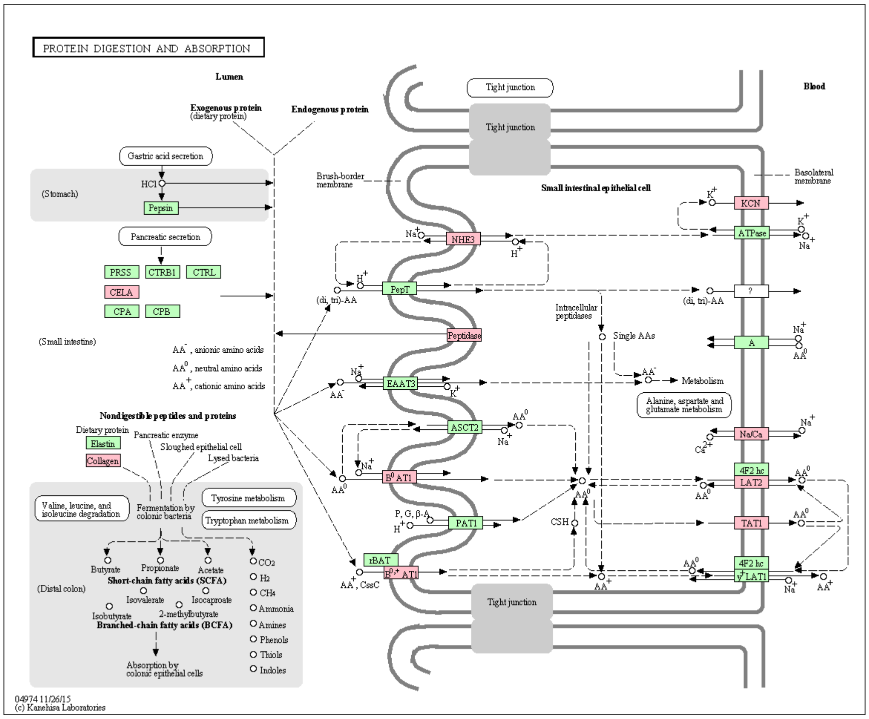

137.3 Legend:

RBH-Blast at 60% Identity + 50% Coverage
Green = Hit in *H. sapiens*
Red = Hit in *H. sapiens* and *C. milli*
White = Not in *H. sapiens*

138 Epithelial cell signaling in Helicobacter pylori infection

138.1 Human Pathway: HSA05120

138.2 Number of Hits: 15

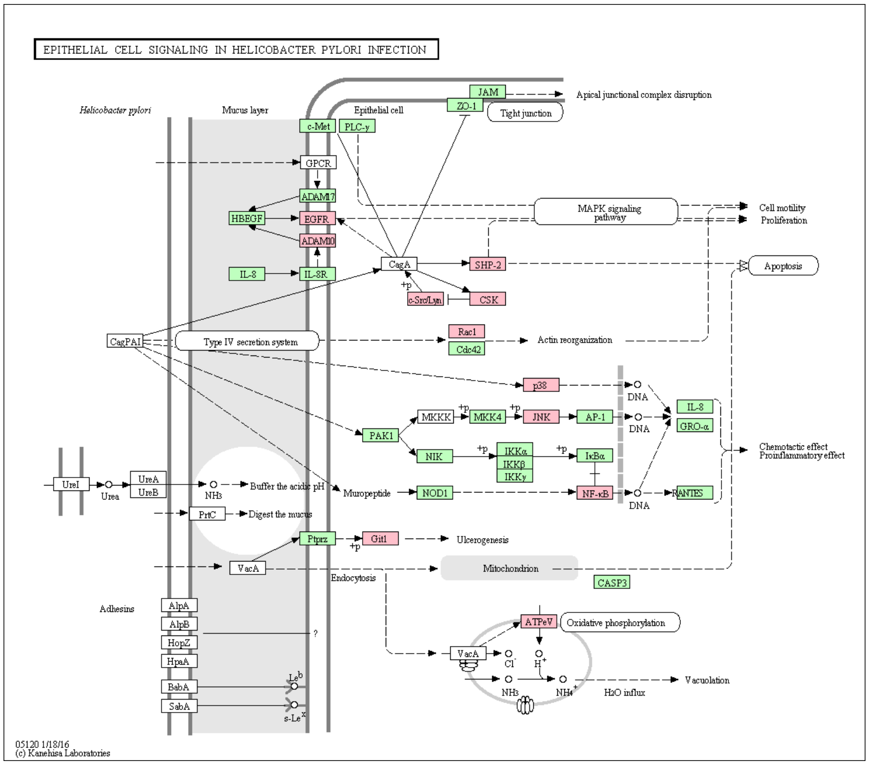

138.3 Legend:

RBH-Blast at 60% Identity + 50% Coverage
Green = Hit in *H. sapiens*
Red = Hit in *H. sapiens* and *C. milli*
White = Not in *H. sapiens*

139 Salmonella infection

139.1 Human Pathway: HSA05132

139.2 Number of Hits: 15

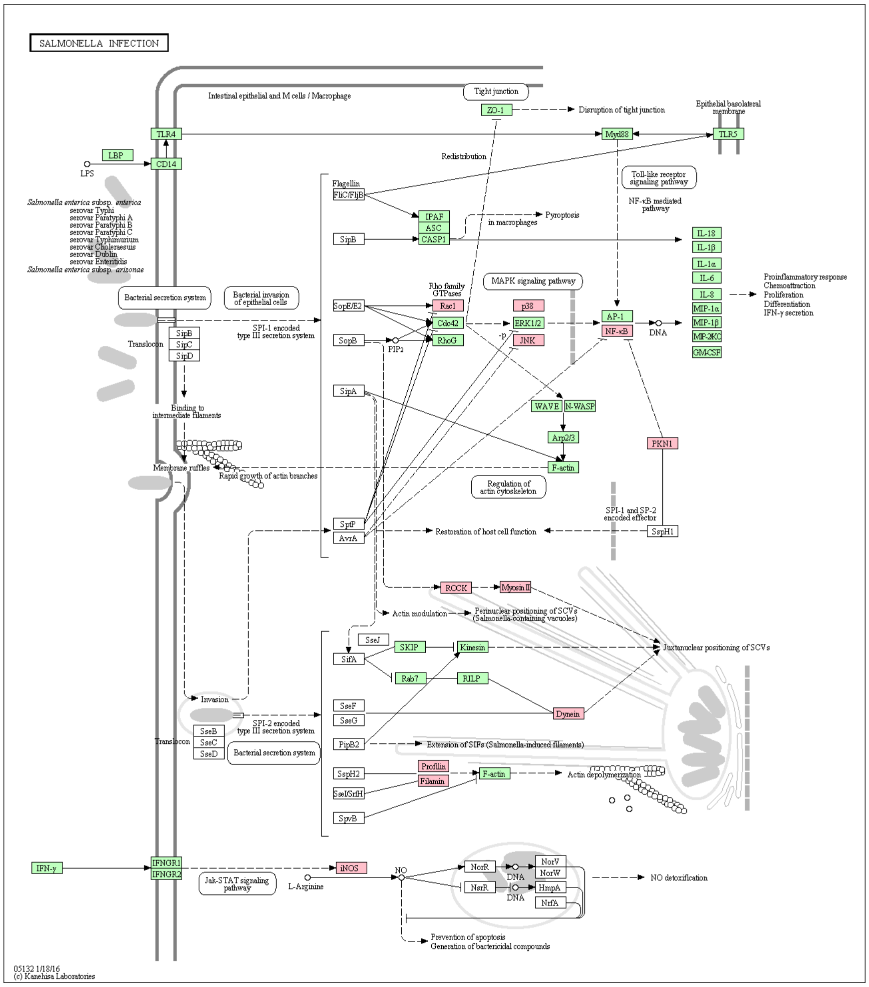

139.3 Legend:

RBH-Blast at 60% Identity + 50% Coverage
Green = Hit in *H. sapiens*
Red = Hit in *H. sapiens* and *C. milli*
White = Not in *H. sapiens*

140 Colorectal cancer

140.1 Human Pathway: HSA05210

140.2 Number of Hits: 15

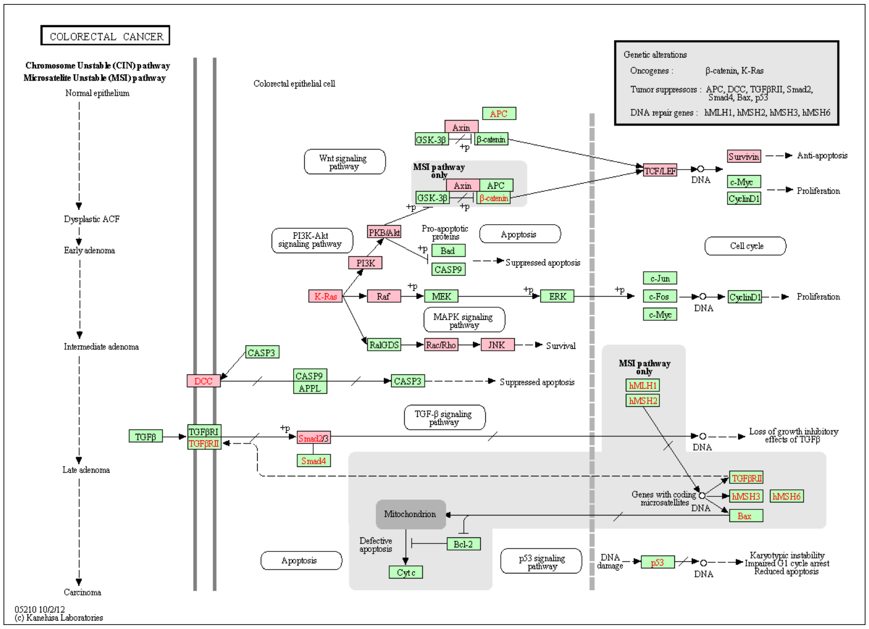

140.3 Legend:

RBH-Blast at 60% Identity + 50% Coverage
Green = Hit in *H. sapiens*
Red = Hit in *H. sapiens* and *C. milli*
White = Not in *H. sapiens*

141 Fatty acid metabolism

141.1 Human Pathway: HSA01212

141.2 Number of Hits: 15

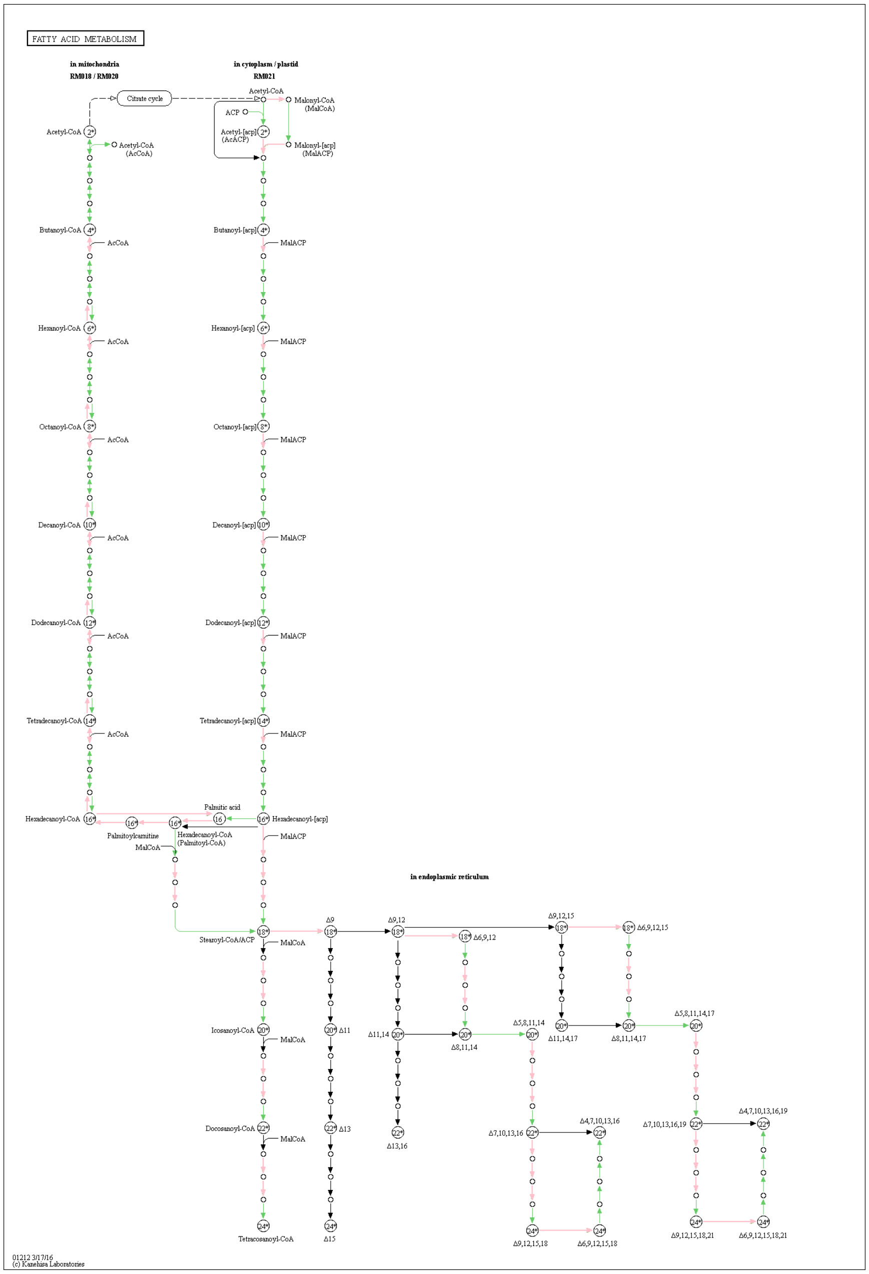

141.3 Legend:

RBH-Blast at 60% Identity + 50% Coverage
Green = Hit in *H. sapiens*
Red = Hit in *H. sapiens* and *C. milli*
White = Not in *H. sapiens*

142 Thyroid hormone synthesis

142.1 Human Pathway: HSA04918

142.2 Number of Hits: 15

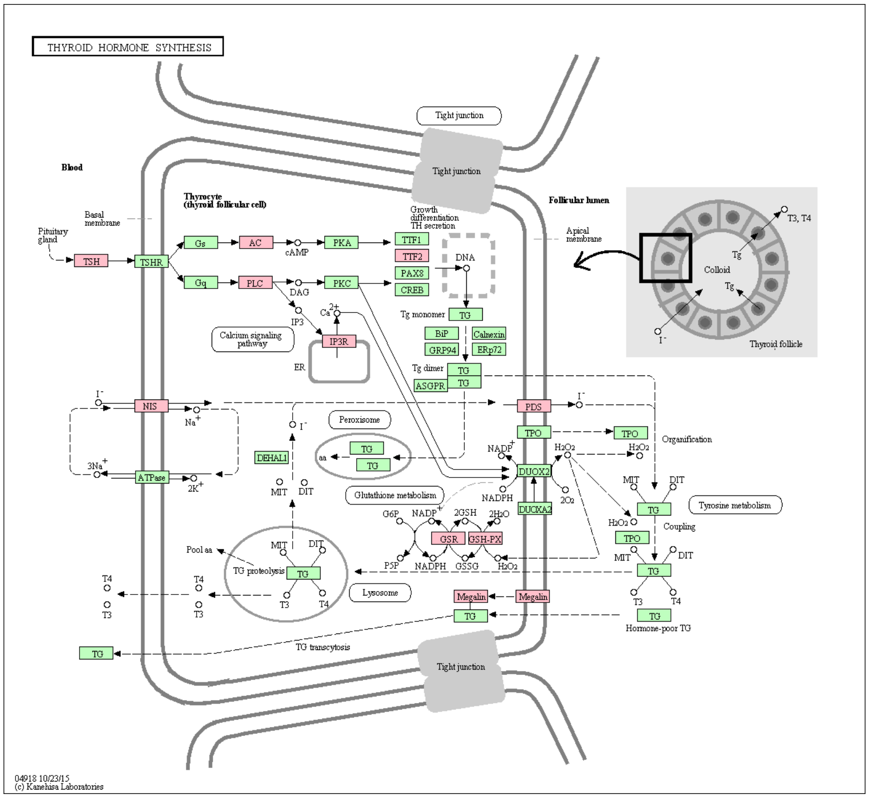

142.3 Legend:

RBH-Blast at 60% Identity + 50% Coverage
Green = Hit in *H. sapiens*
Red = Hit in *H. sapiens* and *C. milli*
White = Not in *H. sapiens*

143 Synaptic vesicle cycle

143.1 Human Pathway: HSA04721

143.2 Number of Hits: 14

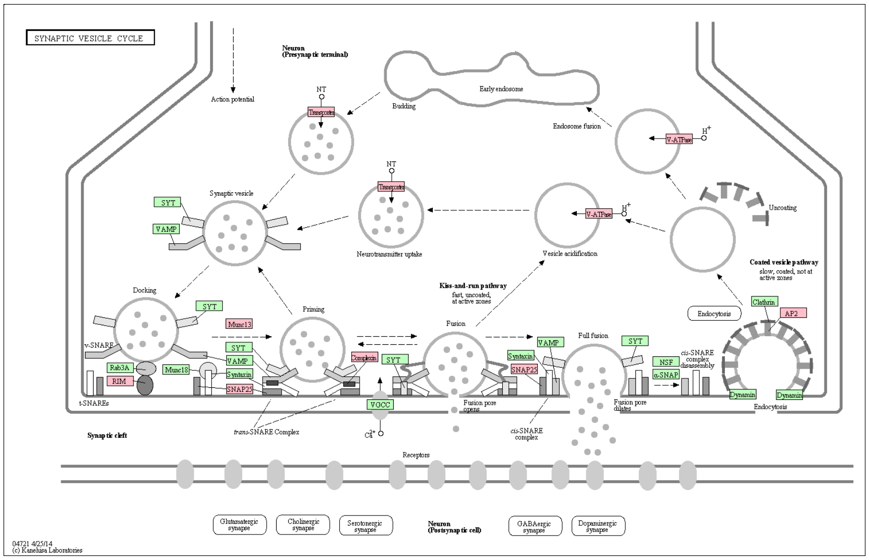

143.3 Legend:

RBH-Blast at 60% Identity + 50% Coverage
Green = Hit in *H. sapiens*
Red = Hit in *H. sapiens* and *C. milli*
White = Not in *H. sapiens*

144 Bacterial invasion of epithelial cells

144.1 Human Pathway: HSA05100

144.2 Number of Hits: 14

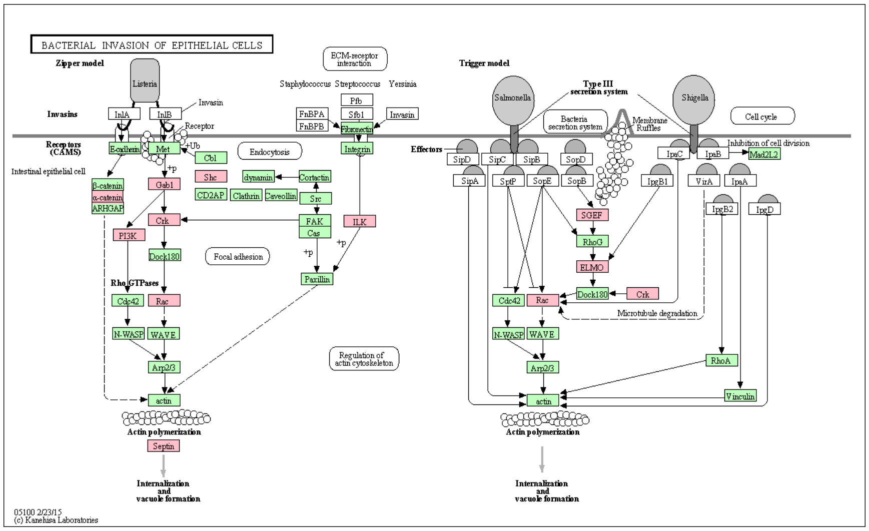

144.3 Legend:

RBH-Blast at 60% Identity + 50% Coverage
Green = Hit in *H. sapiens*
Red = Hit in *H. sapiens* and *C. milli*
White = Not in *H. sapiens*

145 Glycerolipid metabolism

145.1 Human Pathway: HSA00561

145.2 Number of Hits: 14

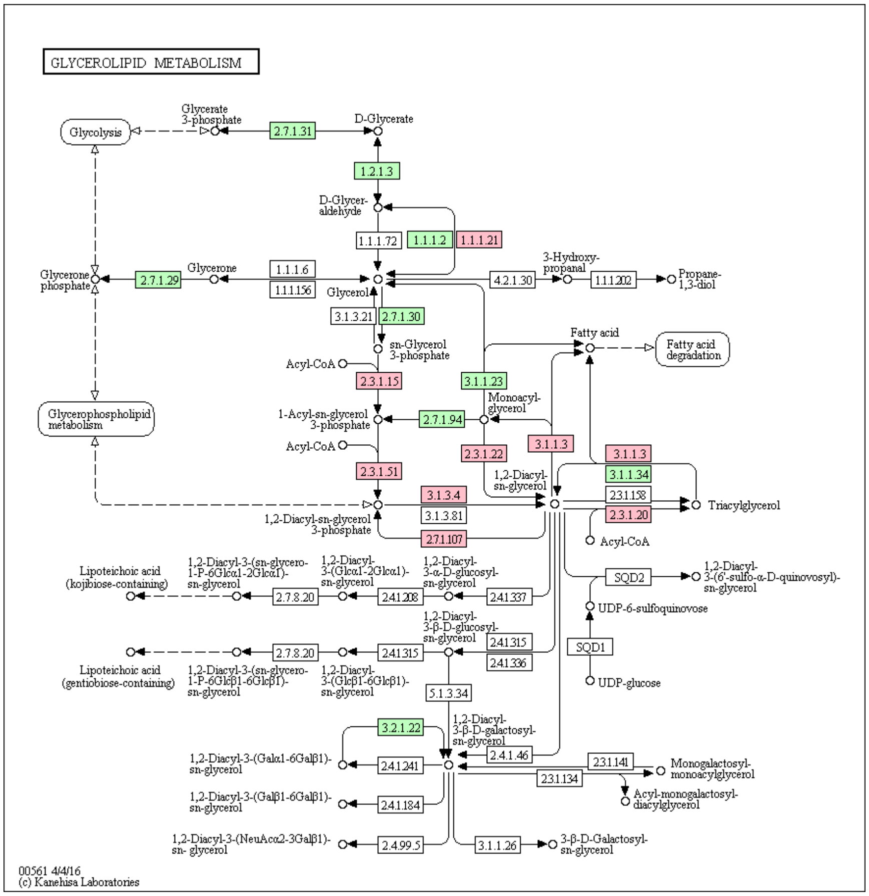

145.3 Legend:

RBH-Blast at 60% Identity + 50% Coverage
Green = Hit in *H. sapiens*
Red = Hit in *H. sapiens* and *C. milli*
White = Not in *H. sapiens*

146 Cardiac muscle contraction

146.1 Human Pathway: HSA04260

146.2 Number of Hits: 14

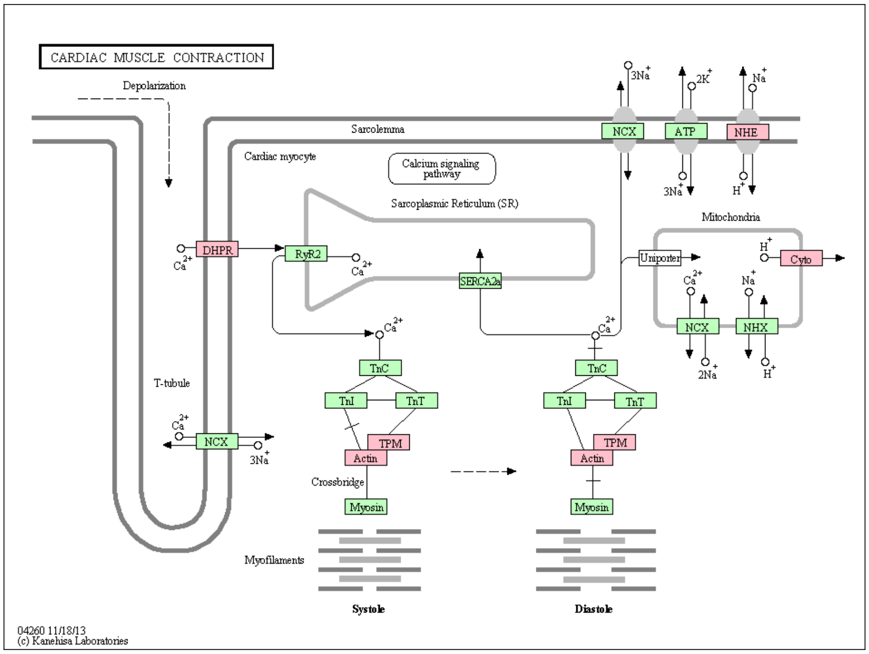

146.3 Legend:

RBH-Blast at 60% Identity + 50% Coverage
Green = Hit in *H. sapiens*
Red = Hit in *H. sapiens* and *C. milli*
White = Not in *H. sapiens*

147 Cysteine and methionine metabolism

147.1 Human Pathway: HSA00270

147.2 Number of Hits: 14

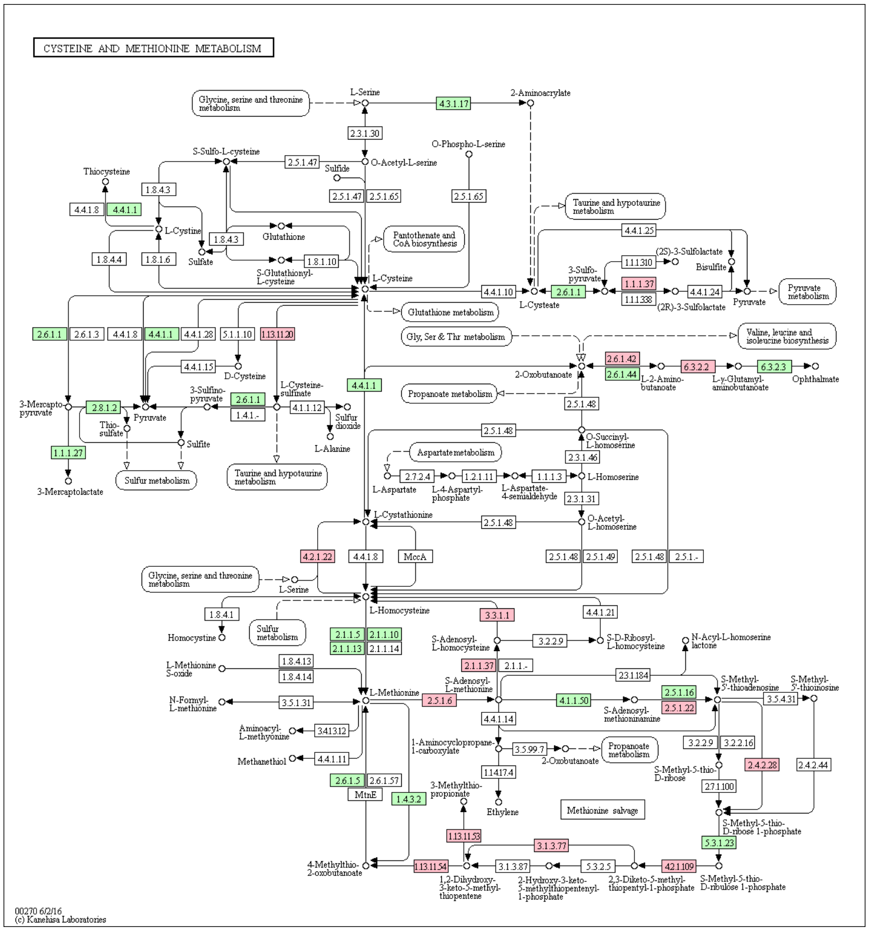

147.3 Legend:

RBH-Blast at 60% Identity + 50% Coverage
Green = Hit in *H. sapiens*
Red = Hit in *H. sapiens* and *C. milli*
White = Not in *H. sapiens*

148 Amphetamine addiction

148.1 Human Pathway: HSA05031

148.2 Number of Hits: 14

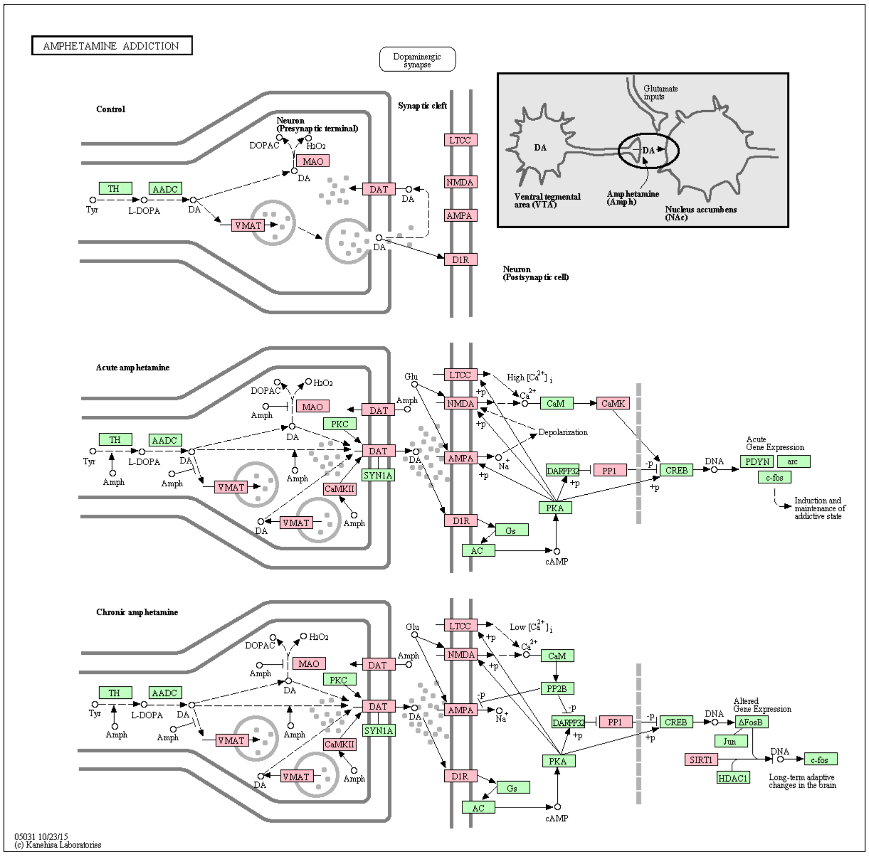

148.3 Legend:

RBH-Blast at 60% Identity + 50% Coverage
Green = Hit in *H. sapiens*
Red = Hit in *H. sapiens* and *C. milli*
White = Not in *H. sapiens*

149 ABC transporters

149.1 Human Pathway: HSA02010

149.2 Number of Hits: 13

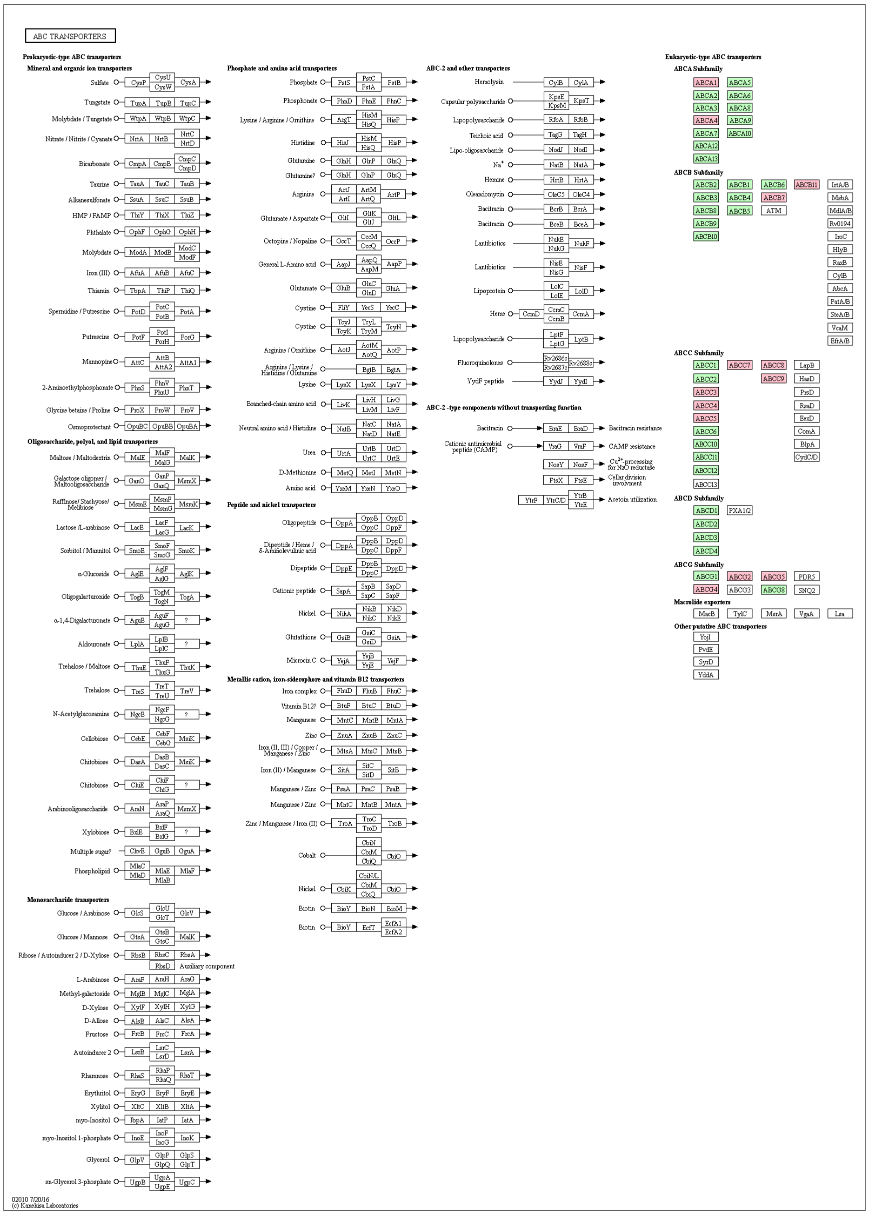

149.3 Legend:

RBH-Blast at 60% Identity + 50% Coverage
Green = Hit in *H. sapiens*
Red = Hit in *H. sapiens* and *C. milli*
White = Not in *H. sapiens*

---

150 Arginine and proline metabolism

150.1 Human Pathway: HSA00330

150.2 Number of Hits: 13

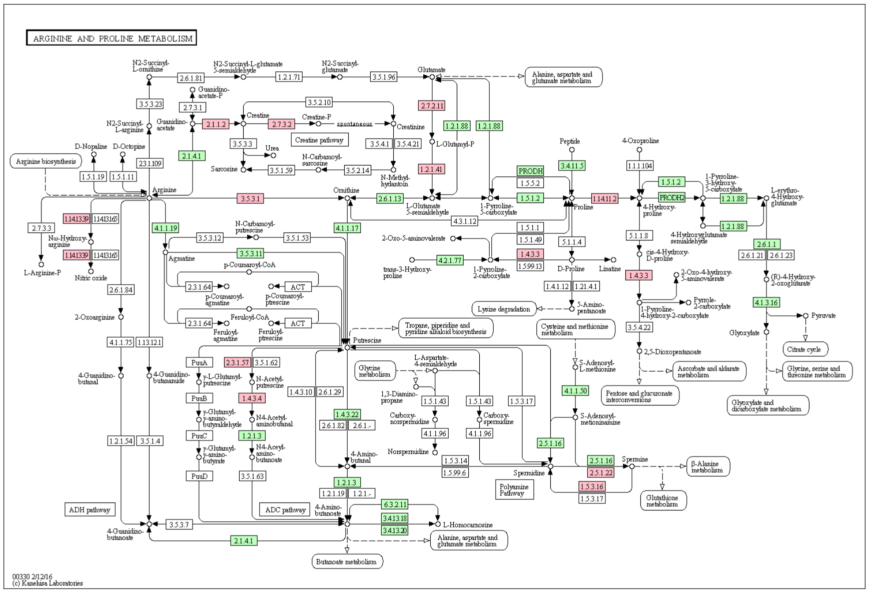

150.3 Legend:

RBH-Blast at 60% Identity + 50% Coverage
Green = Hit in *H. sapiens*
Red = Hit in *H. sapiens* and *C. milli*
White = Not in *H. sapiens*

---

151 Central carbon metabolism in cancer

151.1 Human Pathway: HSA05230

151.2 Number of Hits: 13

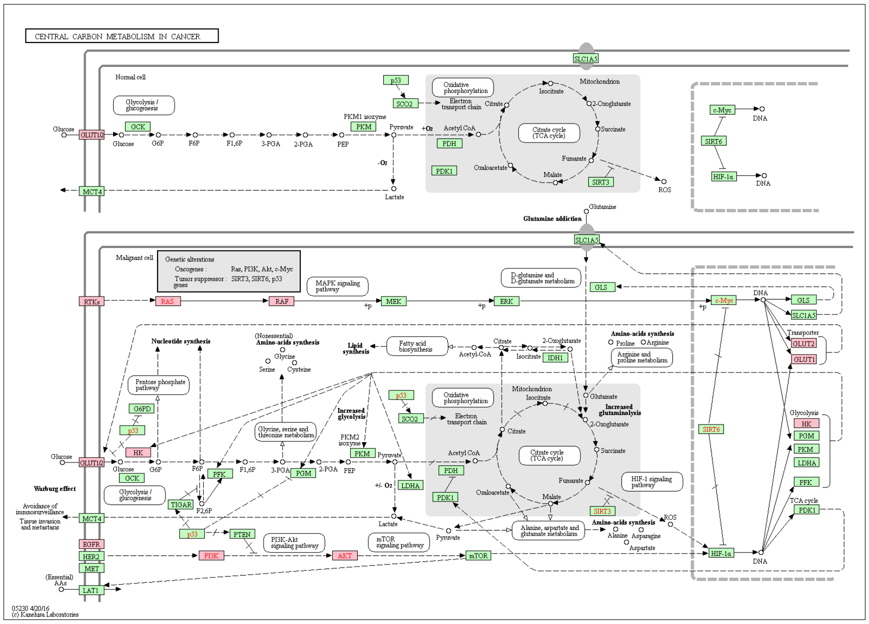

151.3 Legend:

RBH-Blast at 60% Identity + 50% Coverage
Green = Hit in *H. sapiens*
Red = Hit in *H. sapiens* and *C. milli*
White = Not in *H. sapiens*

152 Toll-like receptor signaling pathway

152.1 Human Pathway: HSA04620

152.2 Number of Hits: 13

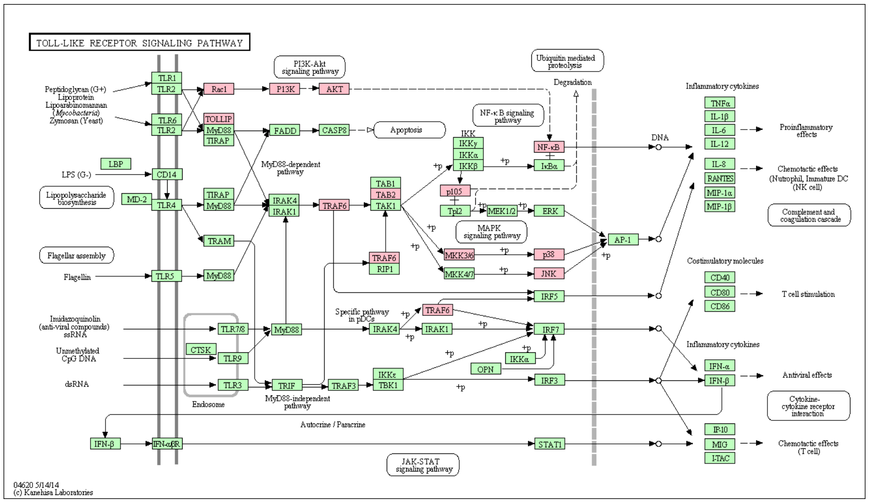

152.3 Legend:

RBH-Blast at 60% Identity + 50% Coverage
Green = Hit in *H. sapiens*
Red = Hit in *H. sapiens* and *C. milli*
White = Not in *H. sapiens*

153 Phototransduction

153.1 Human Pathway: HSA04744

153.2 Number of Hits: 13

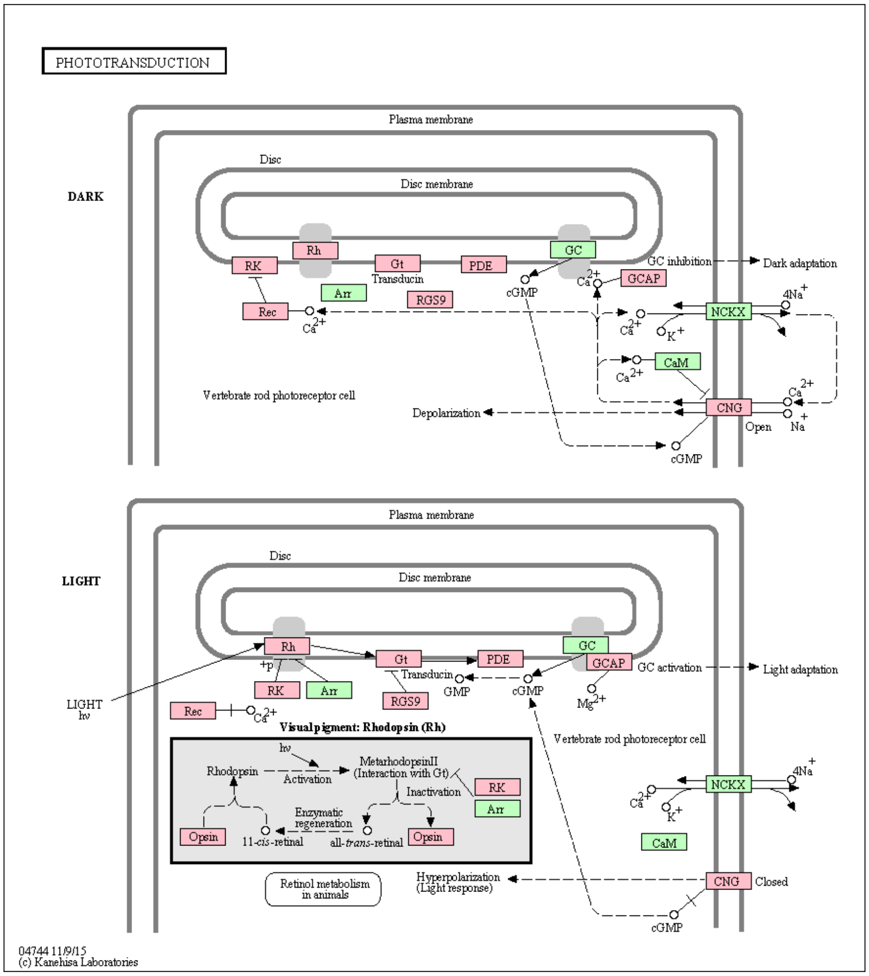

153.3 Legend:

RBH-Blast at 60% Identity + 50% Coverage
Green = Hit in *H. sapiens*
Red = Hit in *H. sapiens* and *C. milli*
White = Not in *H. sapiens*

154 PPAR signaling pathway

154.1 Human Pathway: HSA03320

154.2 Number of Hits: 13

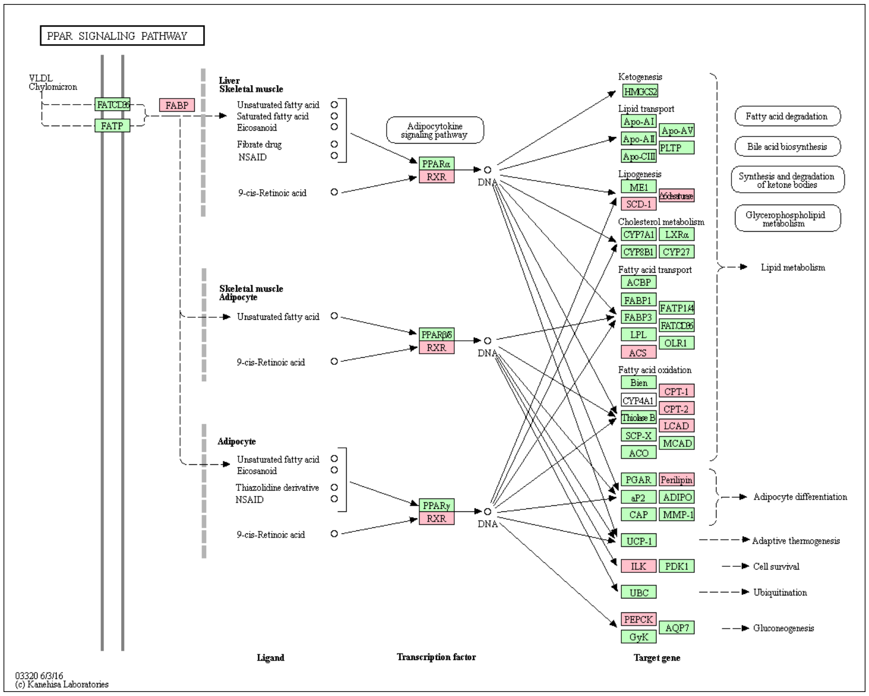

154.3 Legend:

RBH-Blast at 60% Identity + 50% Coverage
Green = Hit in *H. sapiens*
Red = Hit in *H. sapiens* and *C. milli*
White = Not in *H. sapiens*

155 Dorso-ventral axis formation

155.1 Human Pathway: HSA04320

155.2 Number of Hits: 13

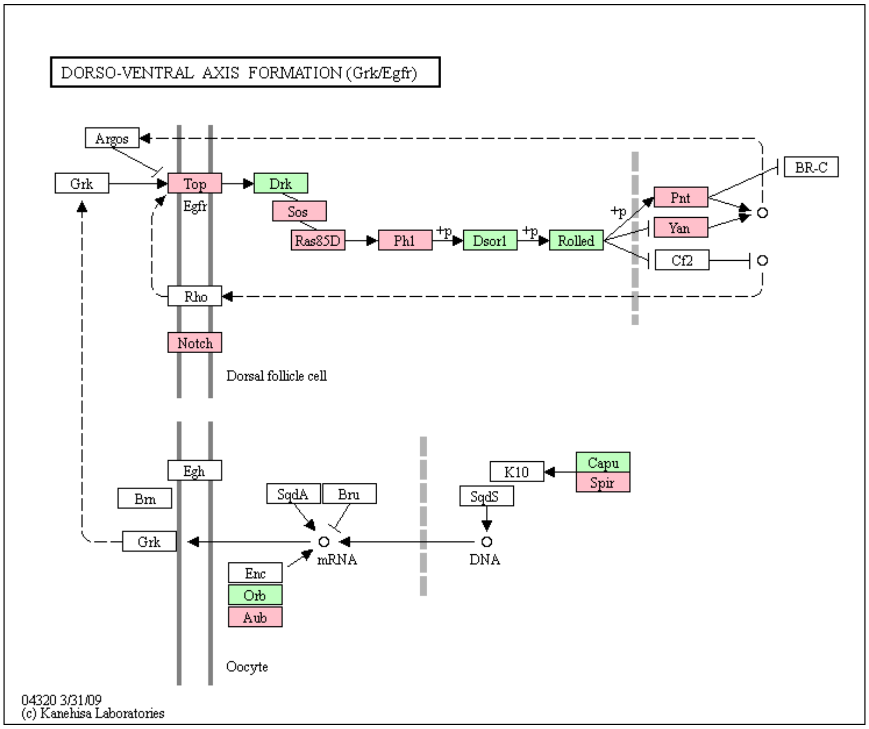

155.3 Legend:

RBH-Blast at 60% Identity + 50% Coverage
Green = Hit in *H. sapiens*
Red = Hit in *H. sapiens* and *C. milli*
White = Not in *H. sapiens*

156 Cocaine addiction

156.1 Human Pathway: HSA05030

156.2 Number of Hits: 13

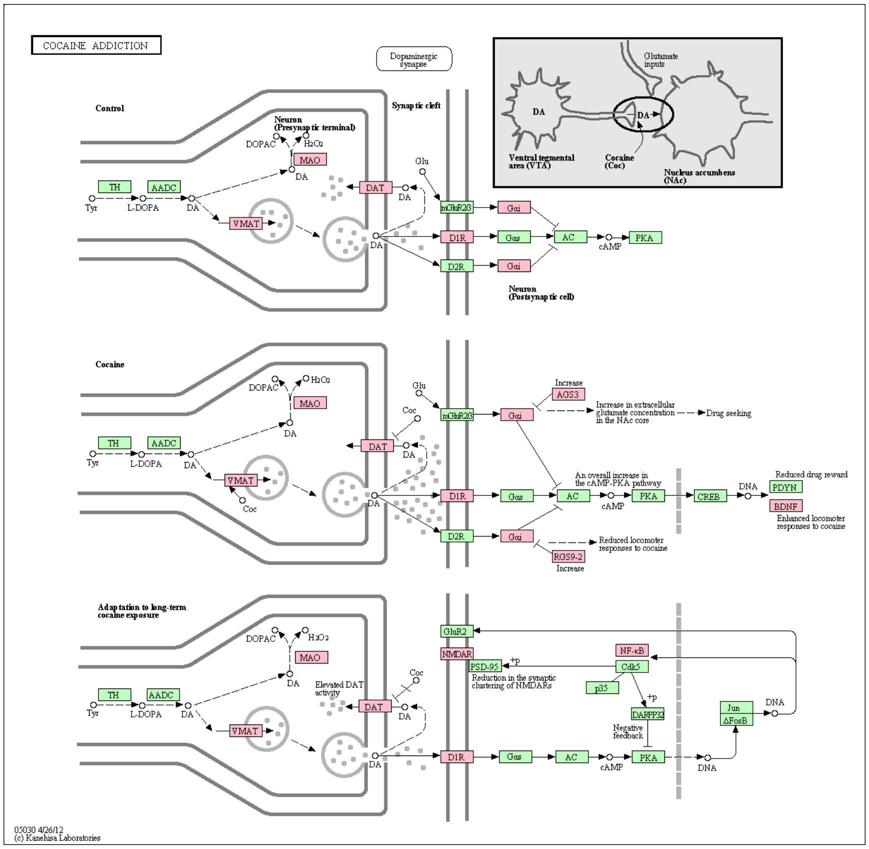

156.3 Legend:

RBH-Blast at 60% Identity + 50% Coverage
Green = Hit in *H. sapiens*
Red = Hit in *H. sapiens* and *C. milli*
White = Not in *H. sapiens*

157 NF-kappa B signaling pathway

157.1 Human Pathway: HSA04064

157.2 Number of Hits: 12

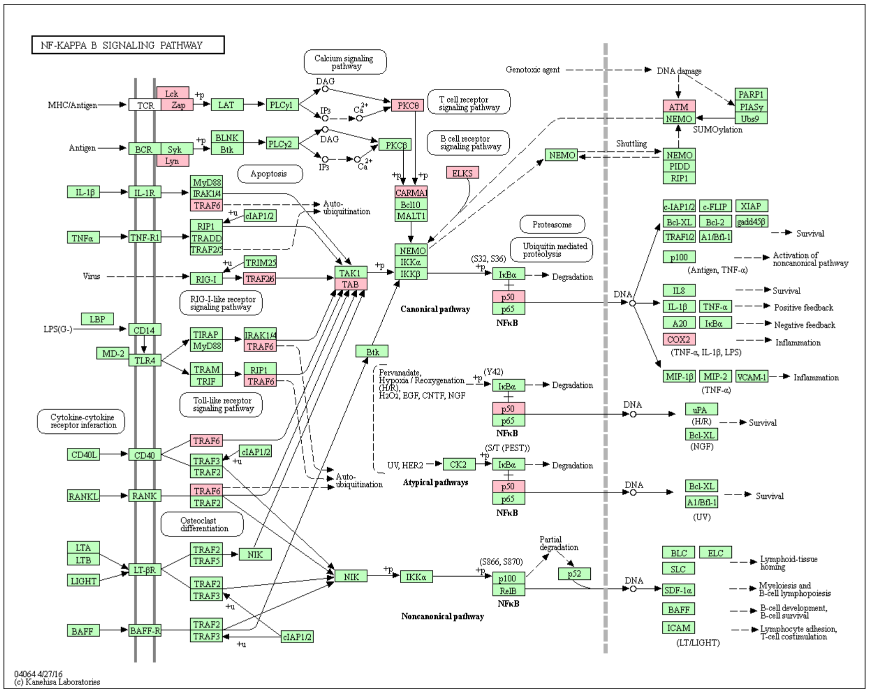

157.3 Legend:

RBH-Blast at 60% Identity + 50% Coverage
Green = Hit in *H. sapiens*
Red = Hit in *H. sapiens* and *C. milli*
White = Not in *H. sapiens*

158 Glycosaminoglycan biosynthesis - heparan sulfate / heparin

158.1 Human Pathway: HSA00534

158.2 Number of Hits: 12

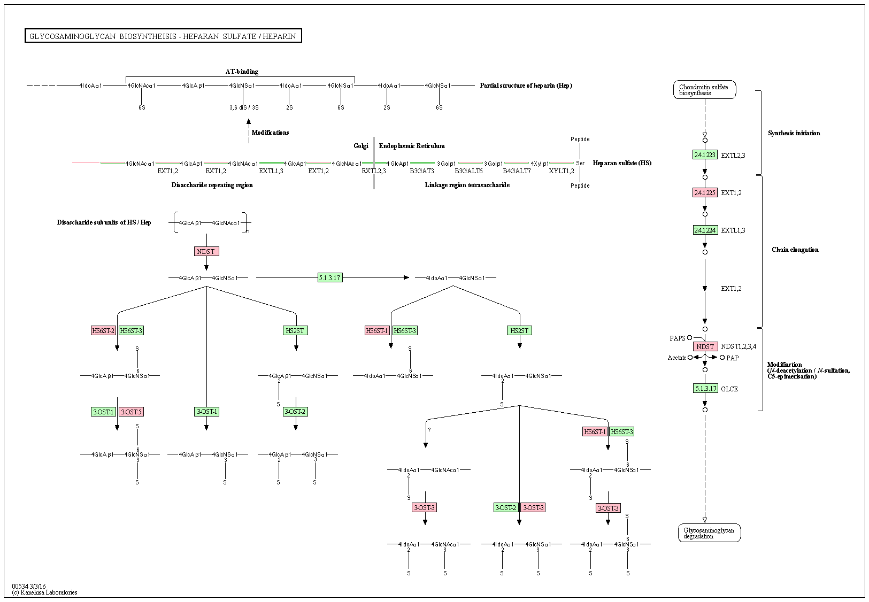

158.3 Legend:

RBH-Blast at 60% Identity + 50% Coverage
Green = Hit in *H. sapiens*
Red = Hit in *H. sapiens* and *C. milli*
White = Not in *H. sapiens*

159 Carbon metabolism

159.1 Human Pathway: HSA01200

159.2 Number of Hits: 12

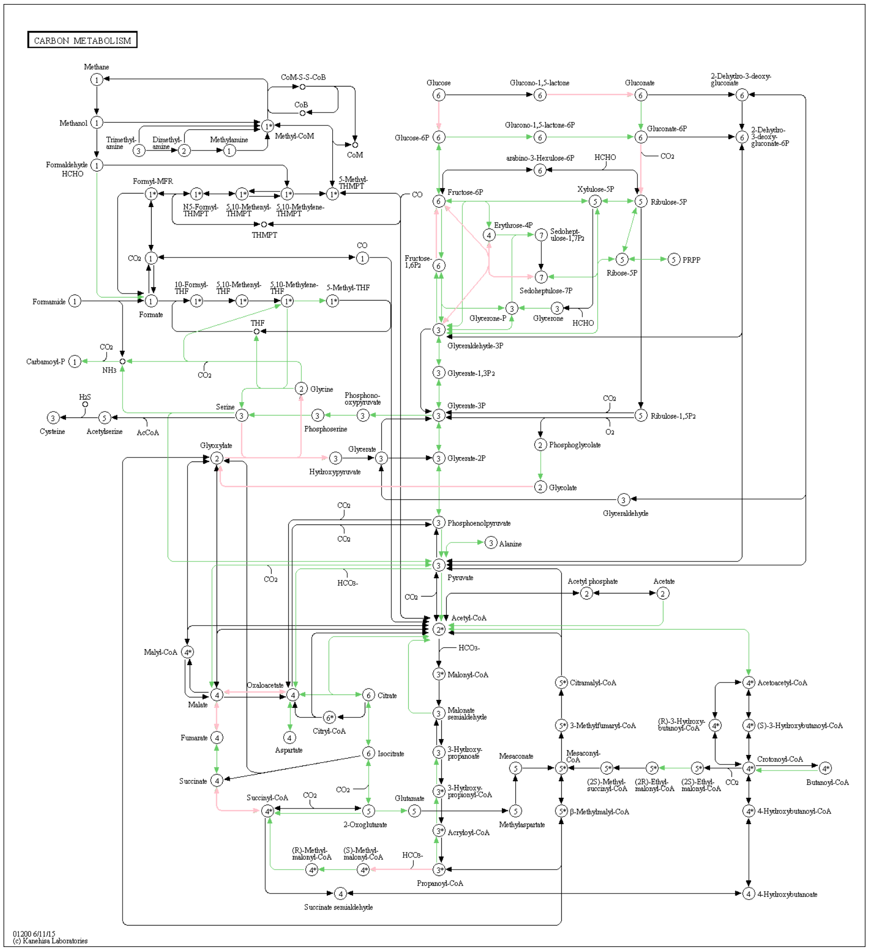

159.3 Legend:

RBH-Blast at 60% Identity + 50% Coverage
Green = Hit in *H. sapiens*
Red = Hit in *H. sapiens* and *C. milli*
White = Not in *H. sapiens*

160 RNA degradation

160.1 Human Pathway: HSA03018

160.2 Number of Hits: 12

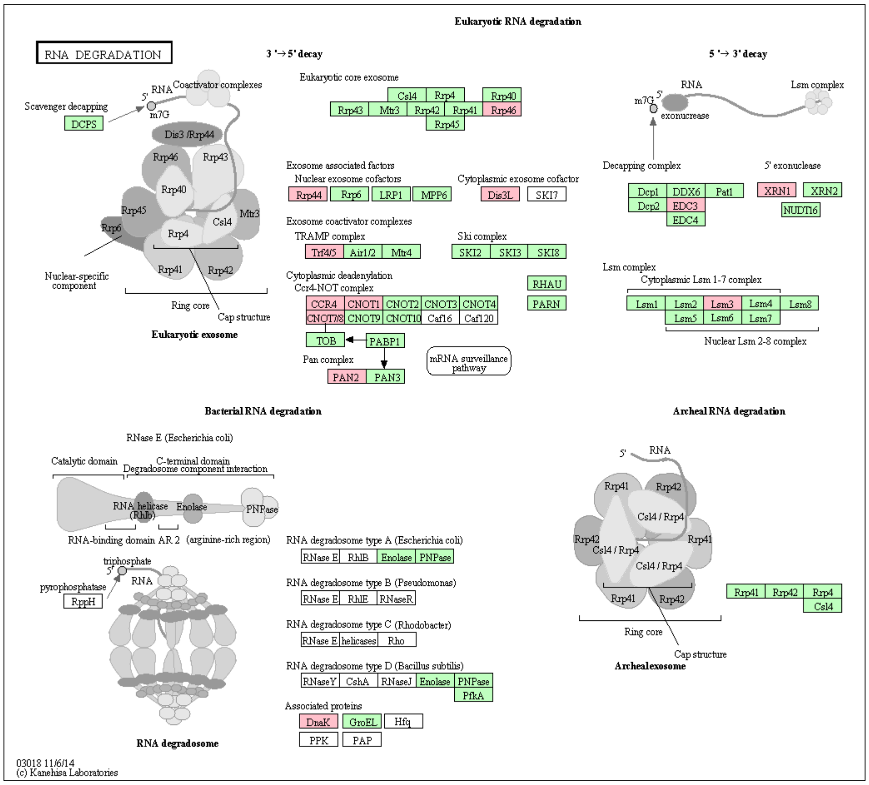

160.3 Legend:

RBH-Blast at 60% Identity + 50% Coverage
Green = Hit in *H. sapiens*
Red = Hit in *H. sapiens* and *C. milli*
White = Not in *H. sapiens*

161 Carbohydrate digestion and absorption

161.1 Human Pathway: HSA04973

161.2 Number of Hits: 11

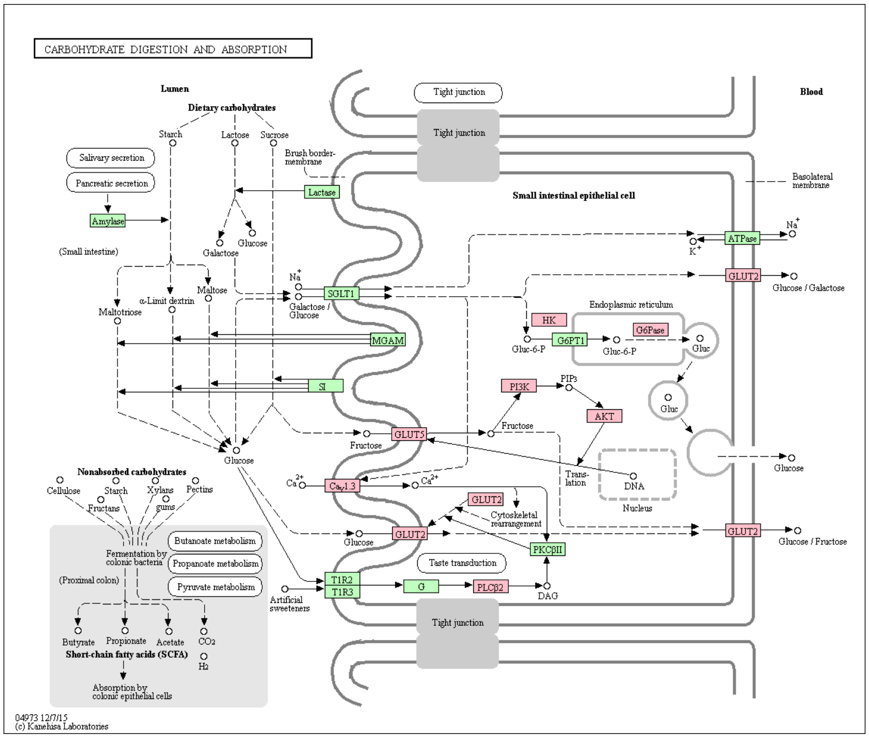

161.3 Legend:

RBH-Blast at 60% Identity + 50% Coverage
Green = Hit in *H. sapiens*
Red = Hit in *H. sapiens* and *C. milli*
White = Not in *H. sapiens*

162 VEGF signaling pathway

162.1 Human Pathway: HSA04370

162.2 Number of Hits: 11

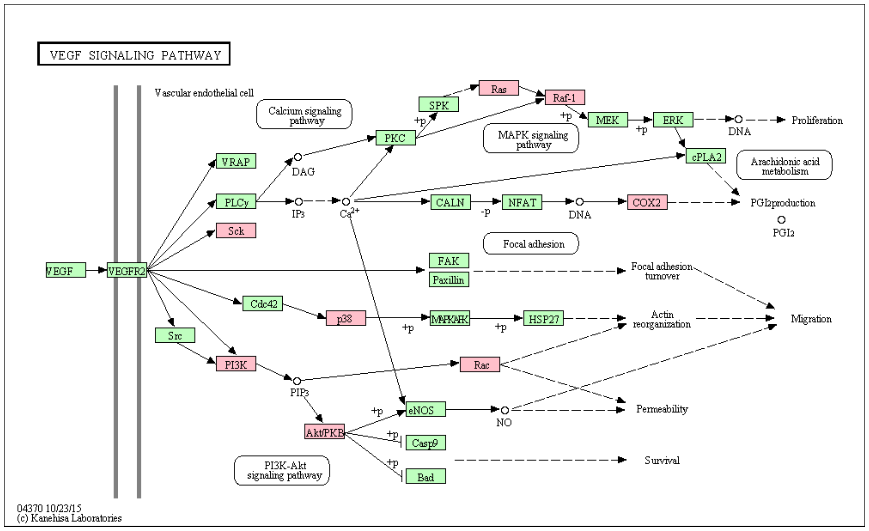

162.3 Legend:

RBH-Blast at 60% Identity + 50% Coverage
Green = Hit in *H. sapiens*
Red = Hit in *H. sapiens* and *C. milli*
White = Not in *H. sapiens*

163 Chemical carcinogenesis

163.1 Human Pathway: HSA05204

163.2 Number of Hits: 11

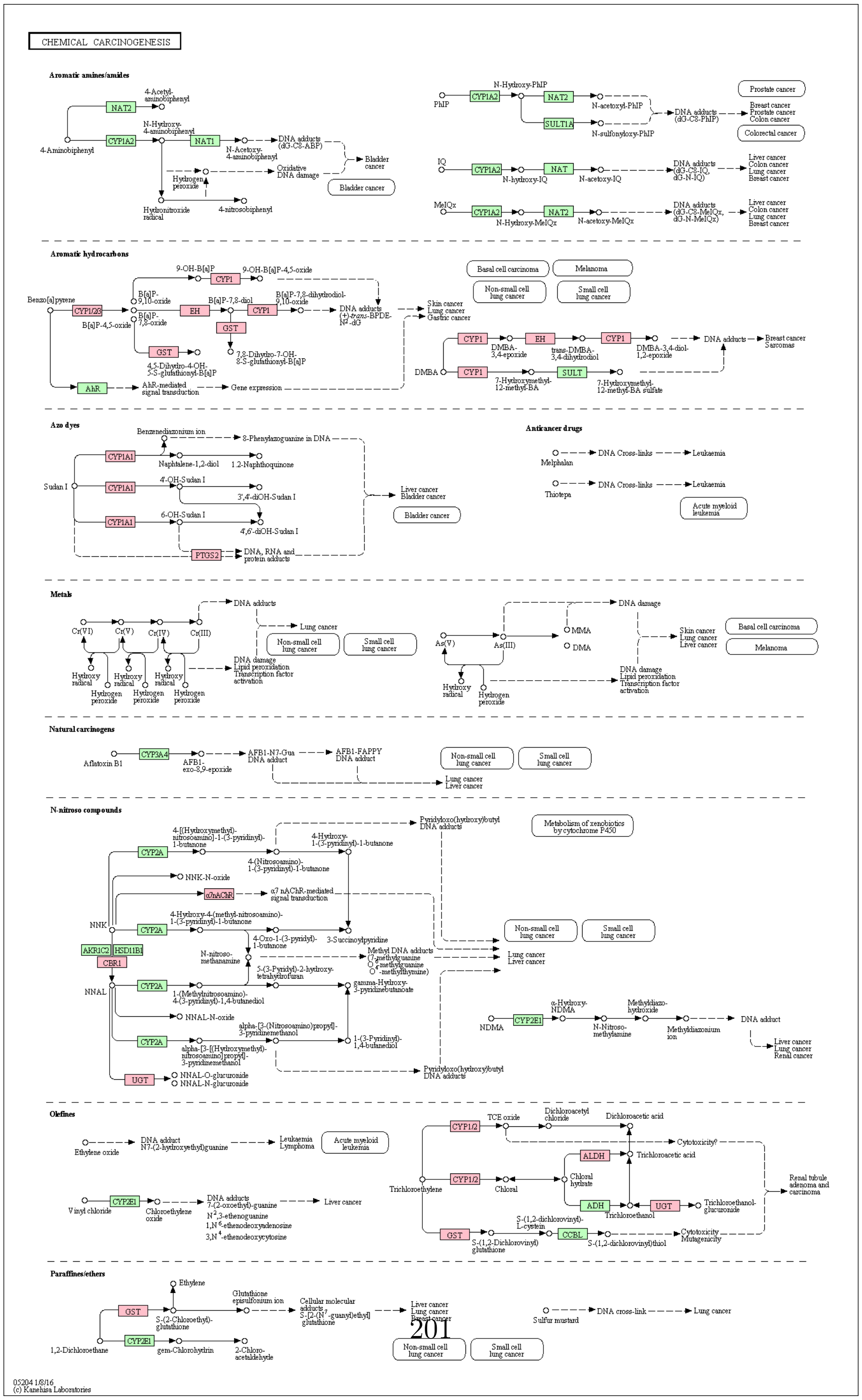

163.3 Legend:

RBH-Blast at 60% Identity + 50% Coverage
Green = Hit in *H. sapiens*
Red = Hit in *H. sapiens* and *C. milli*
White = Not in *H. sapiens*

---

164 Platinum drug resistance

164.1 Human Pathway: HSA01524

164.2 Number of Hits: 11

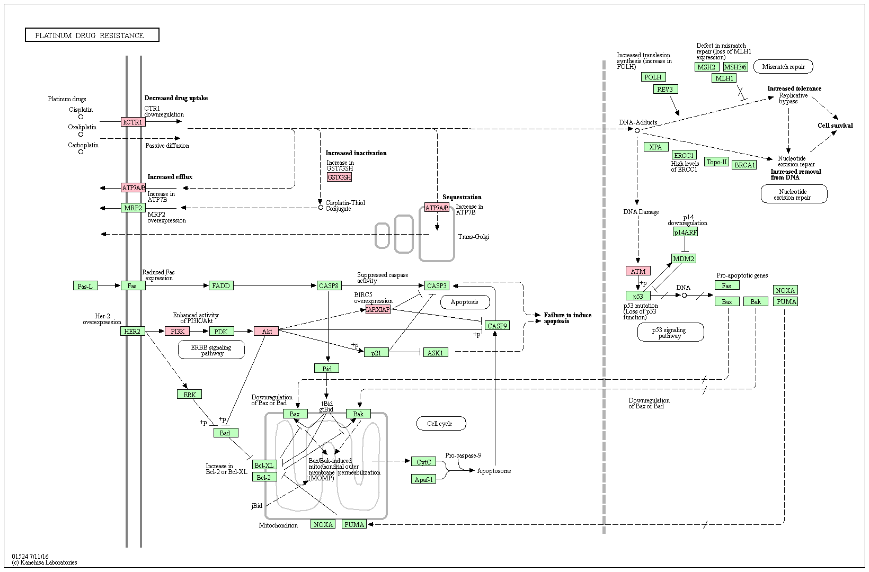

164.3 Legend:

RBH-Blast at 60% Identity + 50% Coverage
Green = Hit in *H. sapiens*
Red = Hit in *H. sapiens* and *C. milli*
White = Not in *H. sapiens*

---

165 ECM-receptor interaction

165.1 Human Pathway: HSA04512

165.2 Number of Hits: 11

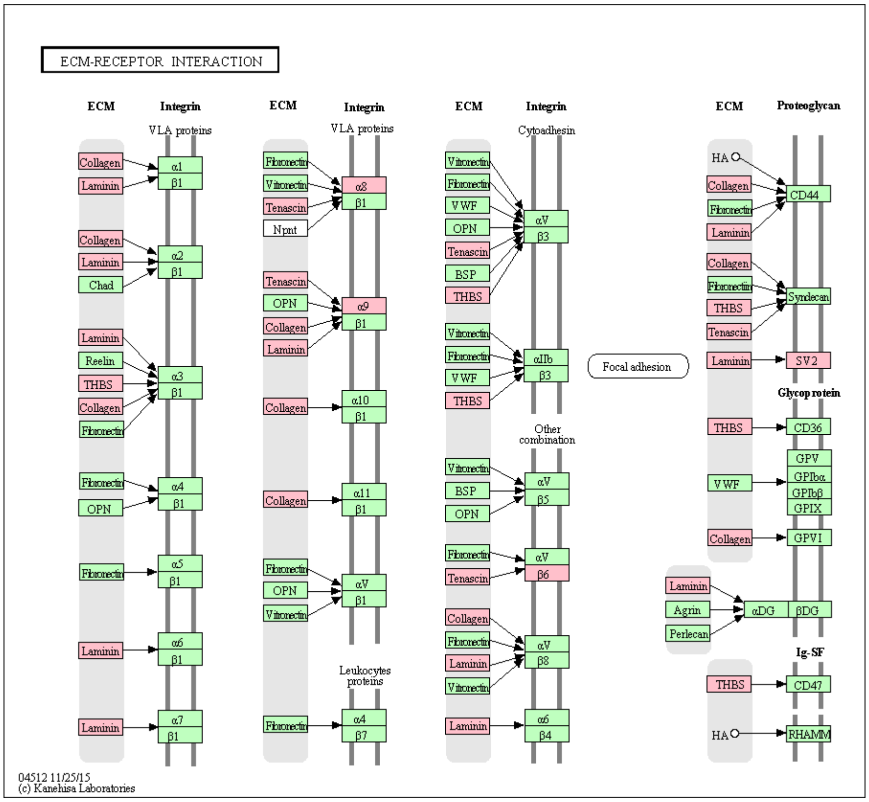

165.3 Legend:

RBH-Blast at 60% Identity + 50% Coverage
Green = Hit in *H. sapiens*
Red = Hit in *H. sapiens* and *C. milli*
White = Not in *H. sapiens*

166 Amino sugar and nucleotide sugar metabolism

166.1 Human Pathway: HSA00520

166.2 Number of Hits: 11

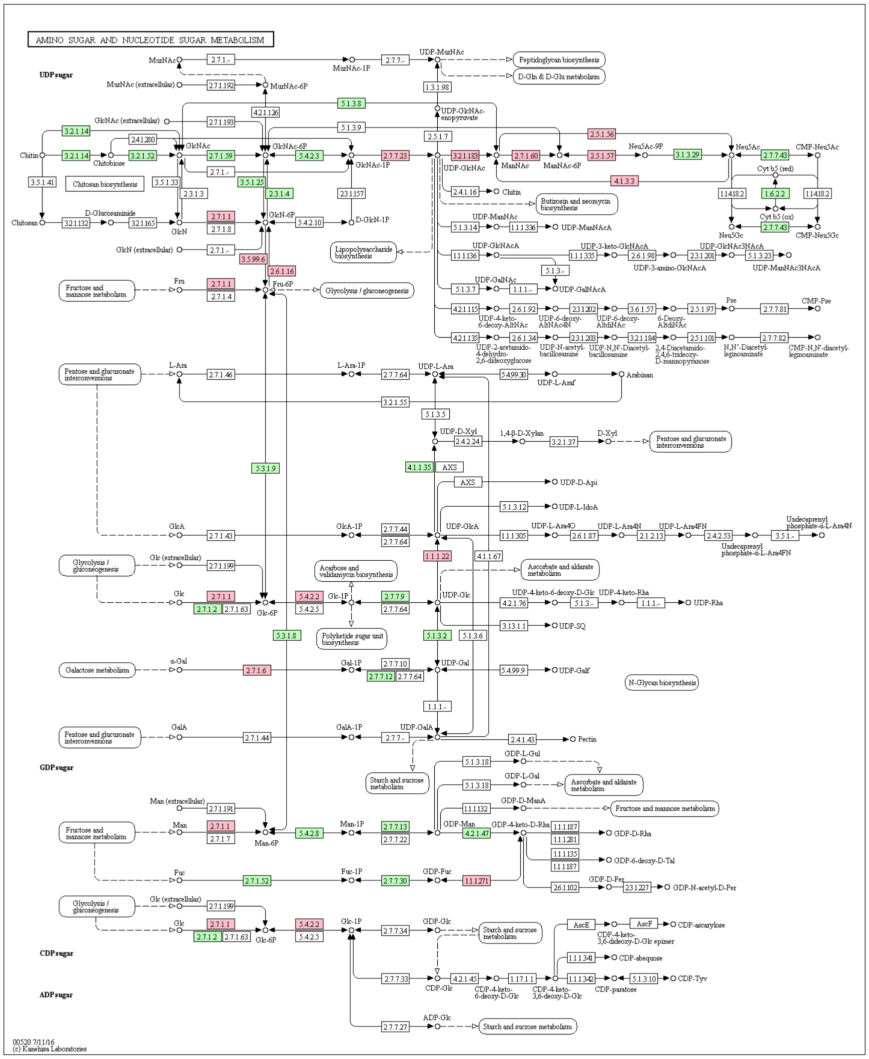

166.3 Legend:

RBH-Blast at 60% Identity + 50% Coverage
Green = Hit in *H. sapiens*
Red = Hit in *H. sapiens* and *C. milli*
White = Not in *H. sapiens*

167 Vibrio cholerae infection

167.1 Human Pathway: HSA05110

167.2 Number of Hits: 11

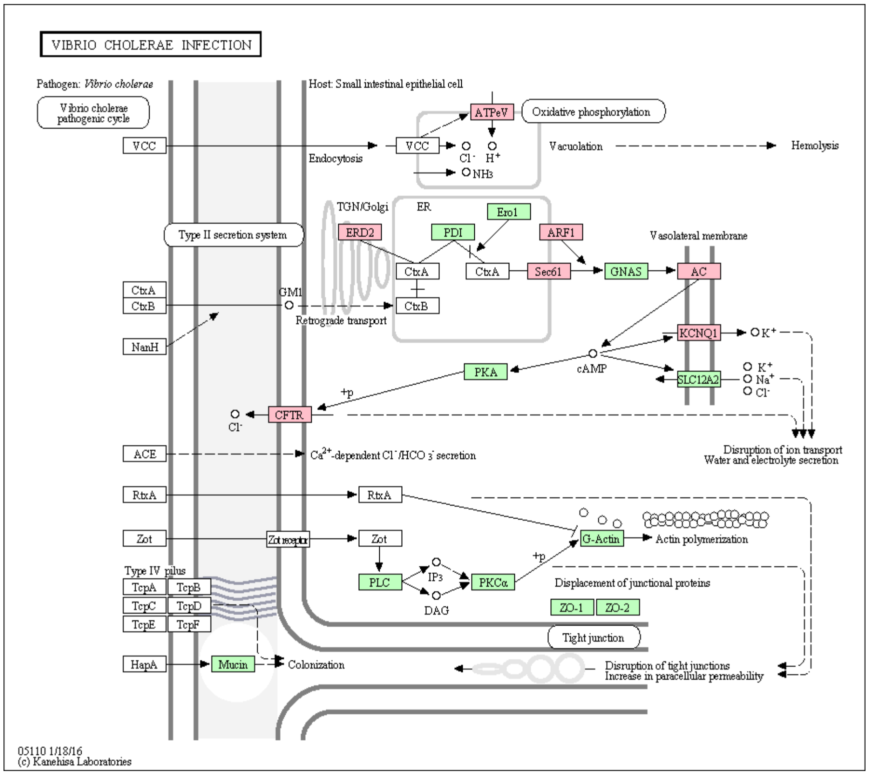

167.3 Legend:

RBH-Blast at 60% Identity + 50% Coverage
Green = Hit in *H. sapiens*
Red = Hit in *H. sapiens* and *C. milli*
White = Not in *H. sapiens*

168 Shigellosis

168.1 Human Pathway: HSA05131

168.2 Number of Hits: 10

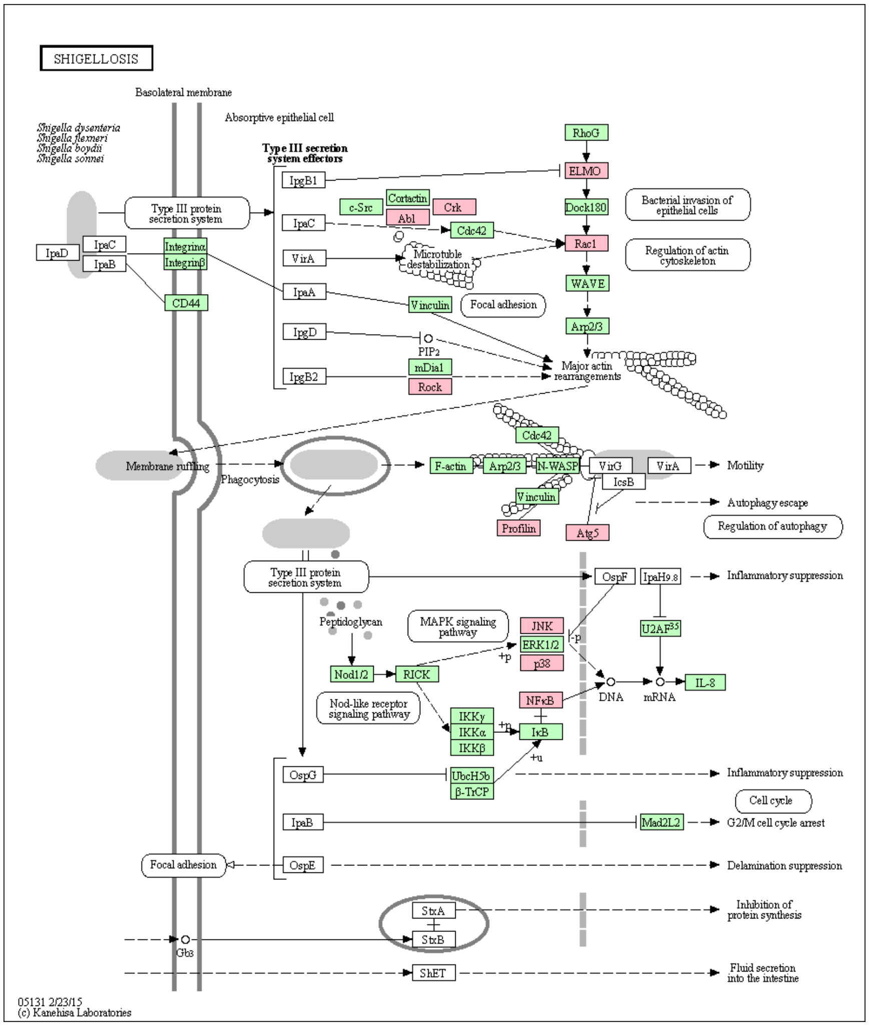

168.3 Legend:

RBH-Blast at 60% Identity + 50% Coverage
Green = Hit in *H. sapiens*
Red = Hit in *H. sapiens* and *C. milli*
White = Not in *H. sapiens*

169 Cytokine-cytokine receptor interaction

169.1 Human Pathway: HSA04060

169.2 Number of Hits: 10

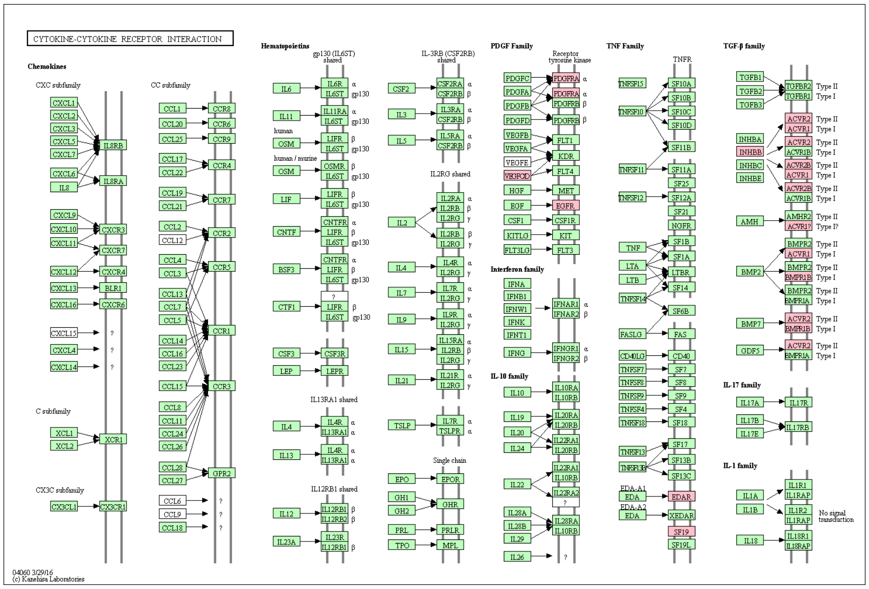

169.3 Legend:

RBH-Blast at 60% Identity + 50% Coverage
Green = Hit in *H. sapiens*
Red = Hit in *H. sapiens* and *C. milli*
White = Not in *H. sapiens*

170 Ribosome biogenesis in eukaryotes

170.1 Human Pathway: HSA03008

170.2 Number of Hits: 10

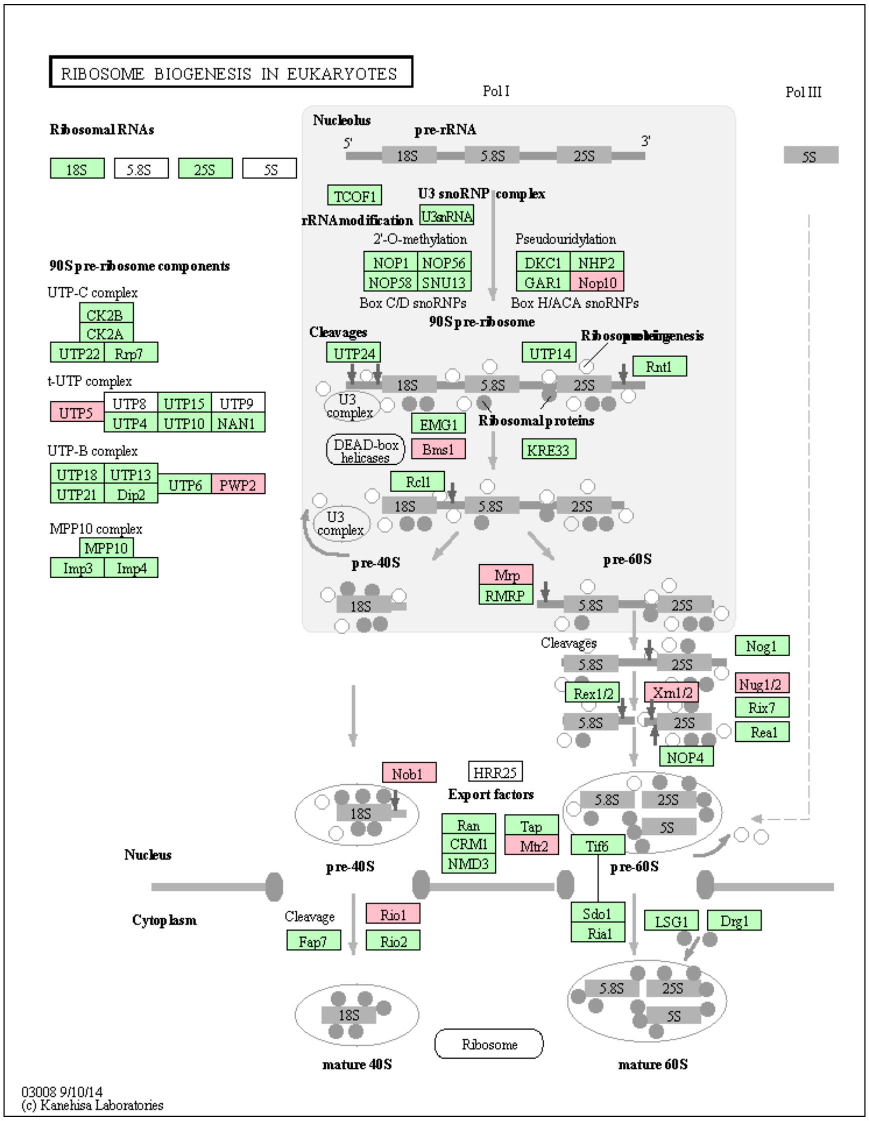

170.3 Legend:

RBH-Blast at 60% Identity + 50% Coverage
Green = Hit in *H. sapiens*
Red = Hit in *H. sapiens* and *C. milli*
White = Not in *H. sapiens*

171 Mucin type O-Glycan biosynthesis

171.1 Human Pathway: HSA00512

171.2 Number of Hits: 10

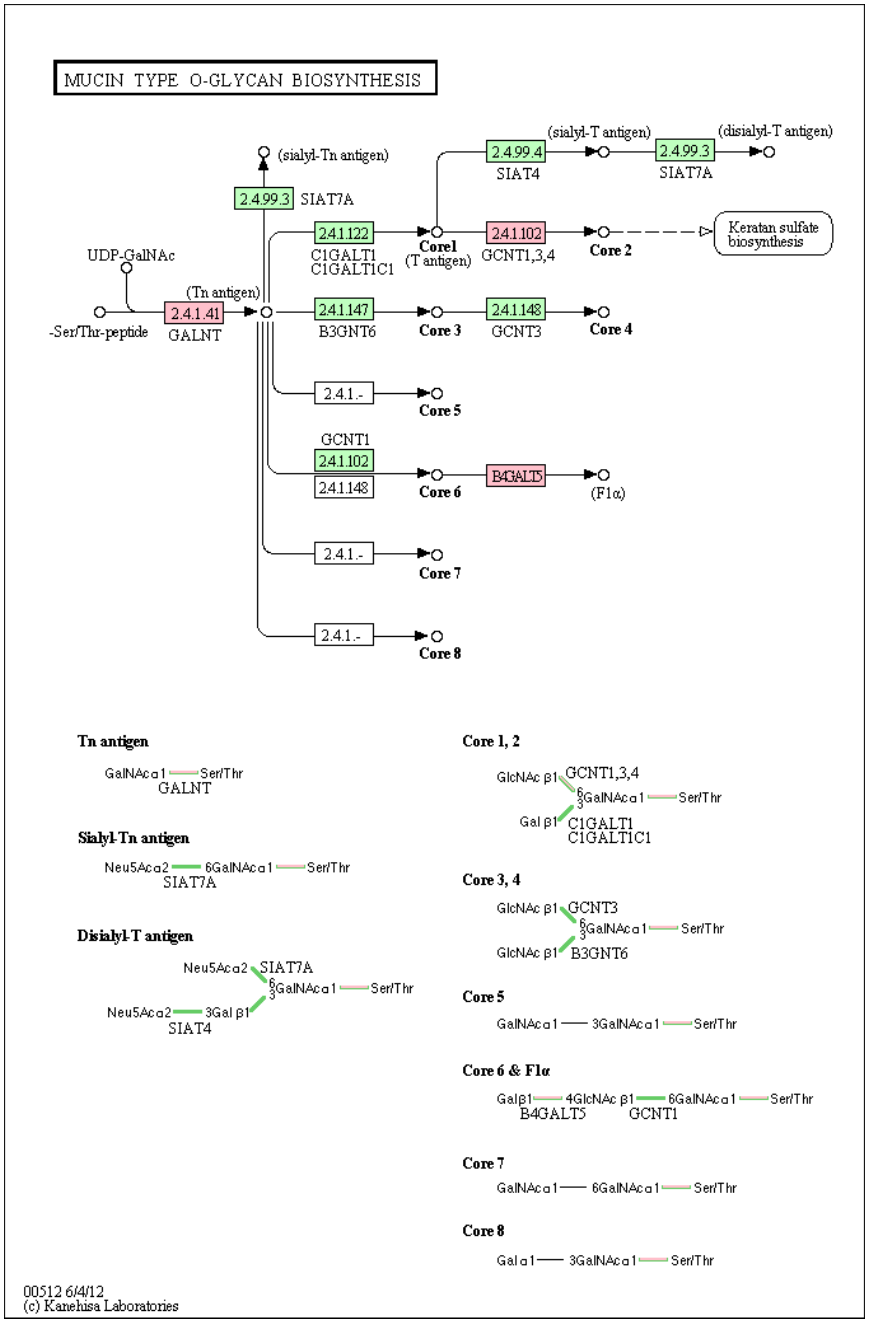

171.3 Legend:

RBH-Blast at 60% Identity + 50% Coverage
Green = Hit in *H. sapiens*
Red = Hit in *H. sapiens* and *C. milli*
White = Not in *H. sapiens*

---

172 Starch and sucrose metabolism

172.1 Human Pathway: HSA00500

172.2 Number of Hits: 10

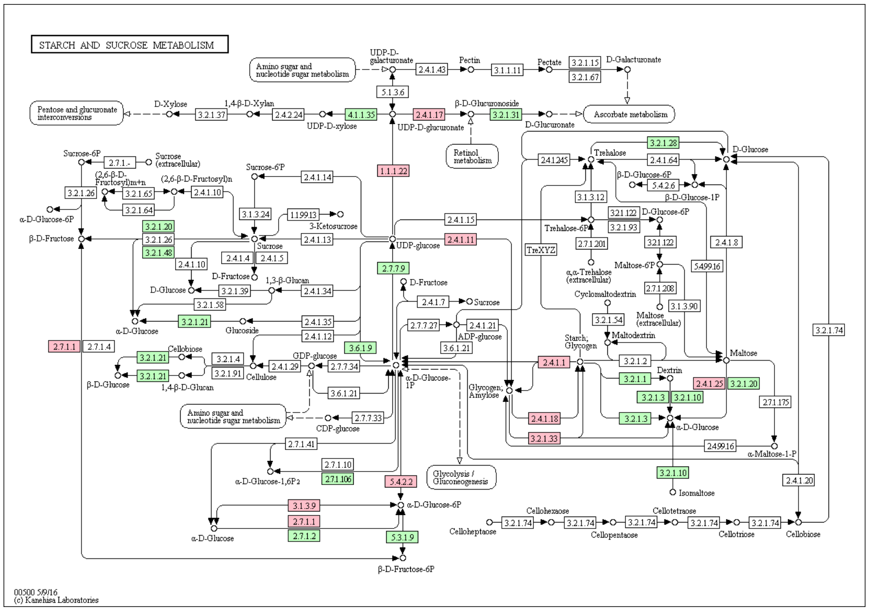

172.3 Legend:

RBH-Blast at 60% Identity + 50% Coverage
Green = Hit in *H. sapiens*
Red = Hit in *H. sapiens* and *C. milli*
White = Not in *H. sapiens*

---

173 Glycosaminoglycan biosynthesis - chondroitin sulfate / dermatan sulfate

173.1 Human Pathway: HSA00532

173.2 Number of Hits: 10

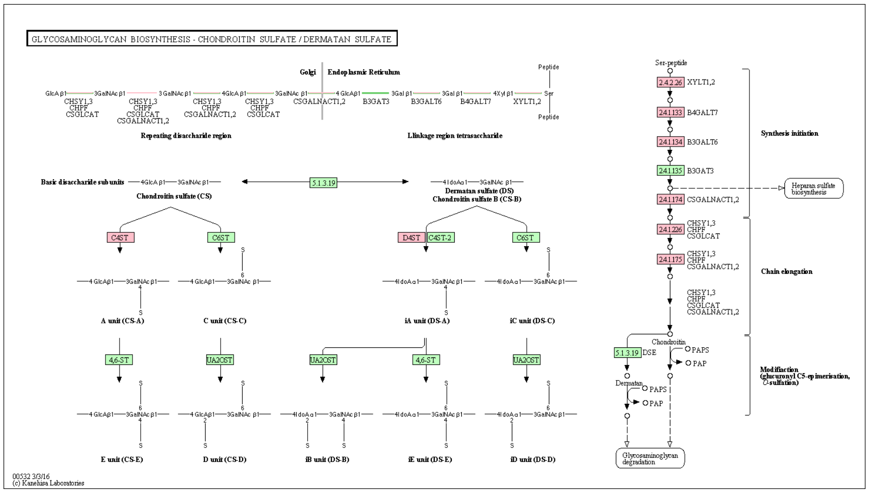

173.3 Legend:

RBH-Blast at 60% Identity + 50% Coverage
Green = Hit in *H. sapiens*
Red = Hit in *H. sapiens* and *C. milli*
White = Not in *H. sapiens*

174 Metabolism of xenobiotics by cytochrome P450

174.1 Human Pathway: HSA00980

174.2 Number of Hits: 9

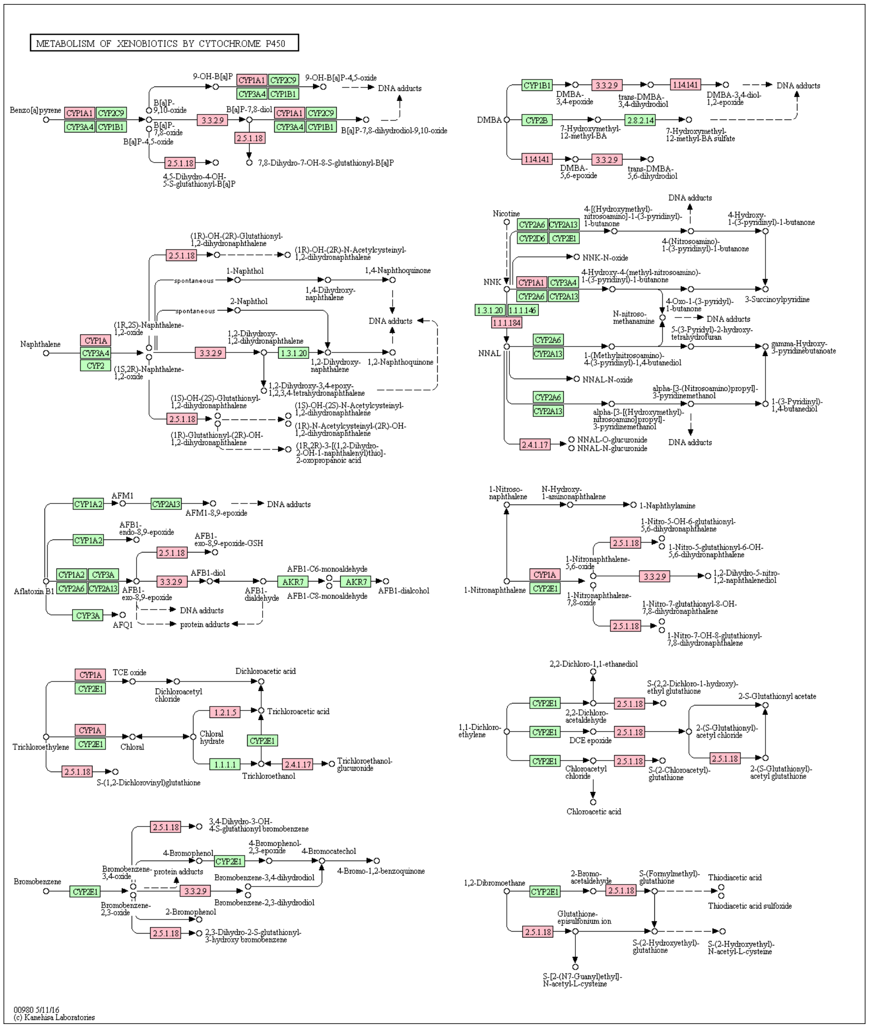

174.3 Legend:

RBH-Blast at 60% Identity + 50% Coverage
Green = Hit in *H. sapiens*
Red = Hit in *H. sapiens* and *C. milli*
White = Not in *H. sapiens*

175 Tyrosine metabolism

175.1 Human Pathway: HSA00350

175.2 Number of Hits: 9

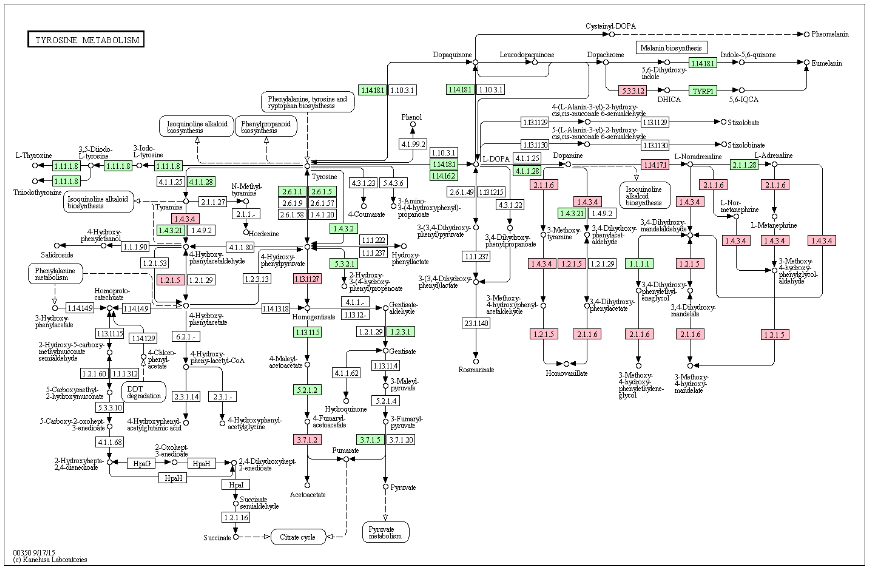

175.3 Legend:

RBH-Blast at 60% Identity + 50% Coverage
Green = Hit in *H. sapiens*
Red = Hit in *H. sapiens* and *C. milli*
White = Not in *H. sapiens*

176 Amyotrophic lateral sclerosis (ALS)

176.1 Human Pathway: HSA05014

176.2 Number of Hits: 9

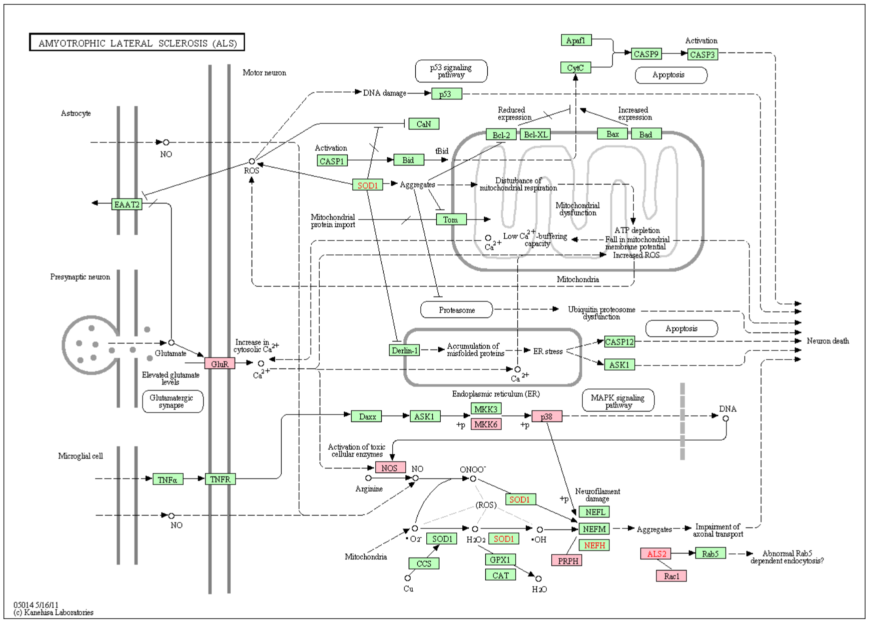

176.3 Legend:

RBH-Blast at 60% Identity + 50% Coverage
Green = Hit in *H. sapiens*
Red = Hit in *H. sapiens* and *C. milli*
White = Not in *H. sapiens*

177 Retinol metabolism

177.1 Human Pathway: HSA00830

177.2 Number of Hits: 9

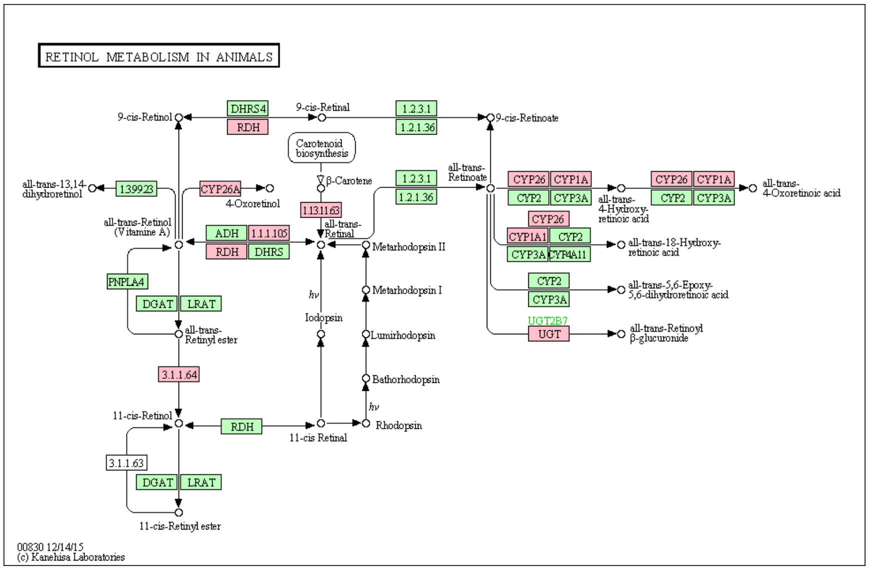

177.3 Legend:

RBH-Blast at 60% Identity + 50% Coverage
Green = Hit in *H. sapiens*
Red = Hit in *H. sapiens* and *C. milli*
White = Not in *H. sapiens*

178 Ovarian steroidogenesis

178.1 Human Pathway: HSA04913

178.2 Number of Hits: 9

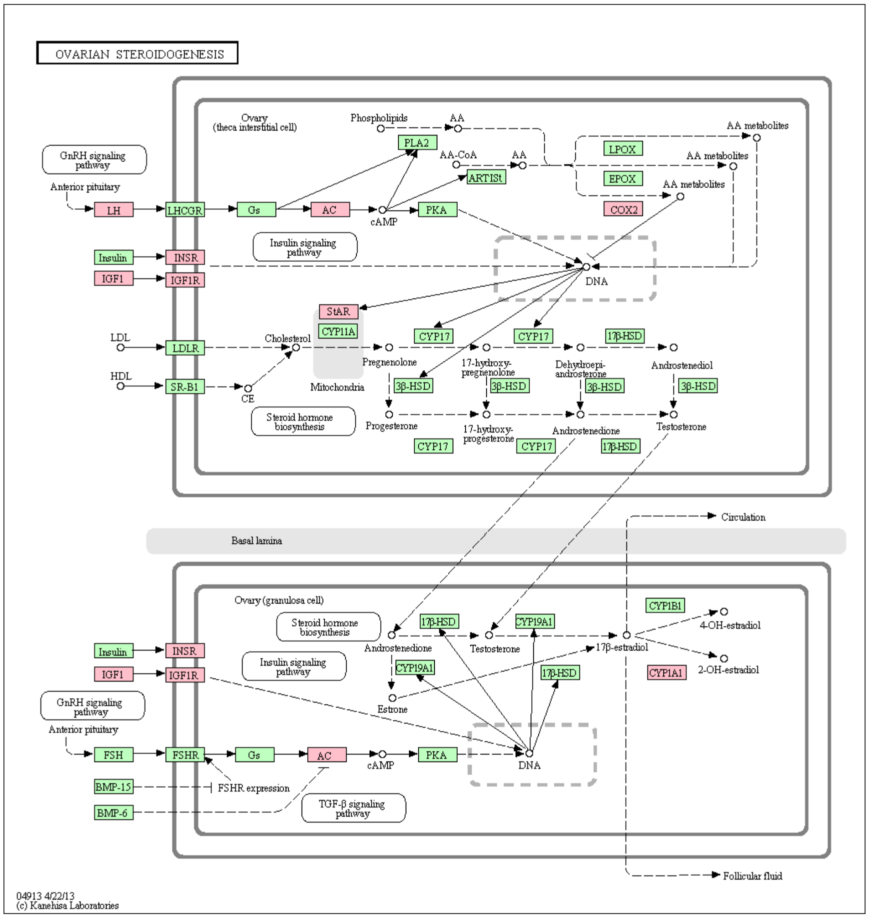

178.3 Legend:

RBH-Blast at 60% Identity + 50% Coverage
Green = Hit in *H. sapiens*
Red = Hit in *H. sapiens* and *C. milli*
White = Not in *H. sapiens*

179 Glutathione metabolism

179.1 Human Pathway: HSA00480

179.2 Number of Hits: 9

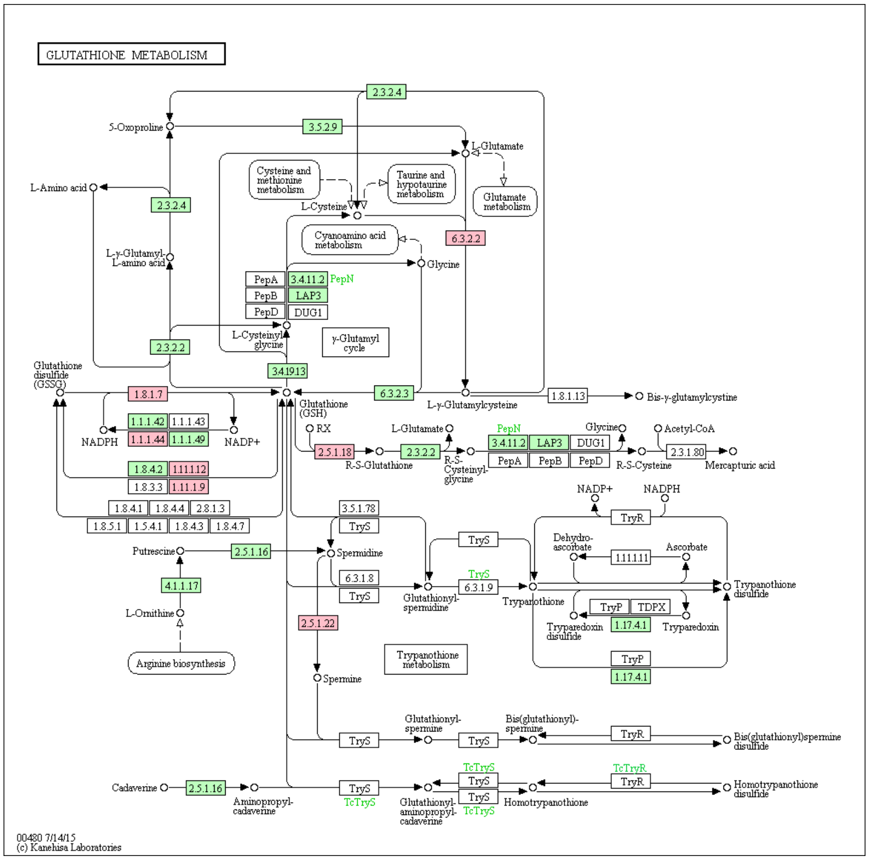

179.3 Legend:

RBH-Blast at 60% Identity + 50% Coverage
Green = Hit in *H. sapiens*
Red = Hit in *H. sapiens* and *C. milli*
White = Not in *H. sapiens*

180 Olfactory transduction

180.1 Human Pathway: HSA04740

180.2 Number of Hits: 9

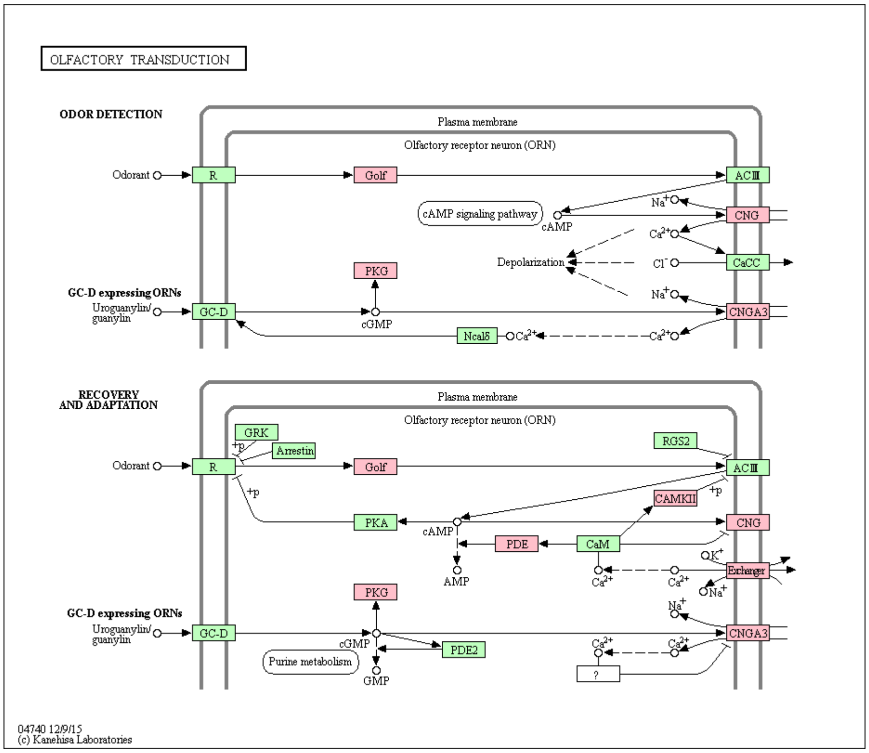

180.3 Legend:

RBH-Blast at 60% Identity + 50% Coverage
Green = Hit in *H. sapiens*
Red = Hit in *H. sapiens* and *C. milli*
White = Not in *H. sapiens*

181 Tryptophan metabolism

181.1 Human Pathway: HSA00380

181.2 Number of Hits: 9

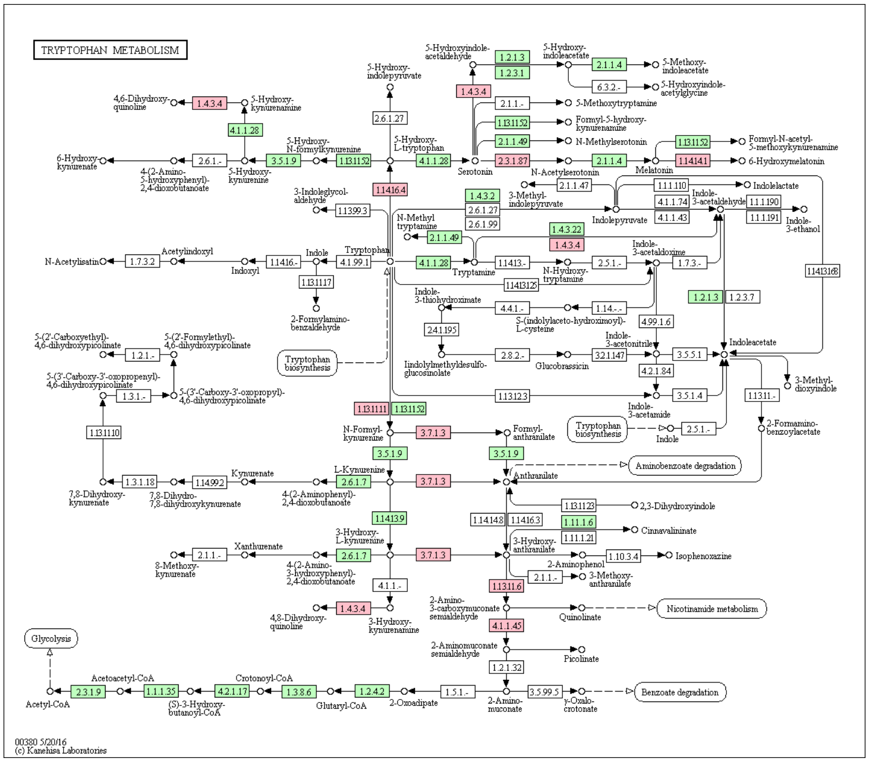

181.3 Legend:

RBH-Blast at 60% Identity + 50% Coverage
Green = Hit in *H. sapiens*
Red = Hit in *H. sapiens* and *C. milli*
White = Not in *H. sapiens*

182 Bladder cancer

182.1 Human Pathway: HSA05219

182.2 Number of Hits: 9

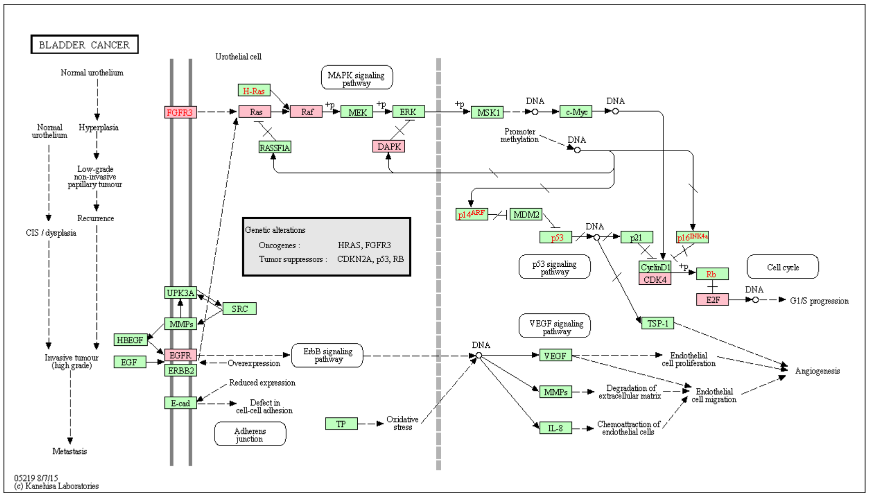

182.3 Legend:

RBH-Blast at 60% Identity + 50% Coverage
Green = Hit in *H. sapiens*
Red = Hit in *H. sapiens* and *C. milli*
White = Not in *H. sapiens*

183 Drug metabolism - other enzymes

183.1 Human Pathway: HSA00983

183.2 Number of Hits: 9

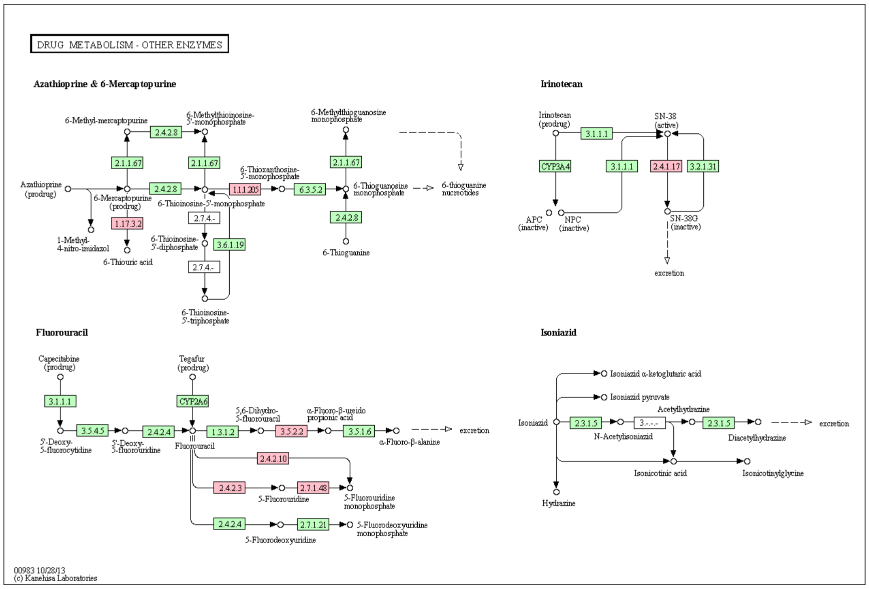

183.3 Legend:

RBH-Blast at 60% Identity + 50% Coverage
Green = Hit in *H. sapiens*
Red = Hit in *H. sapiens* and *C. milli*
White = Not in *H. sapiens*

184 DNA replication

184.1 Human Pathway: HSA03030

184.2 Number of Hits: 9

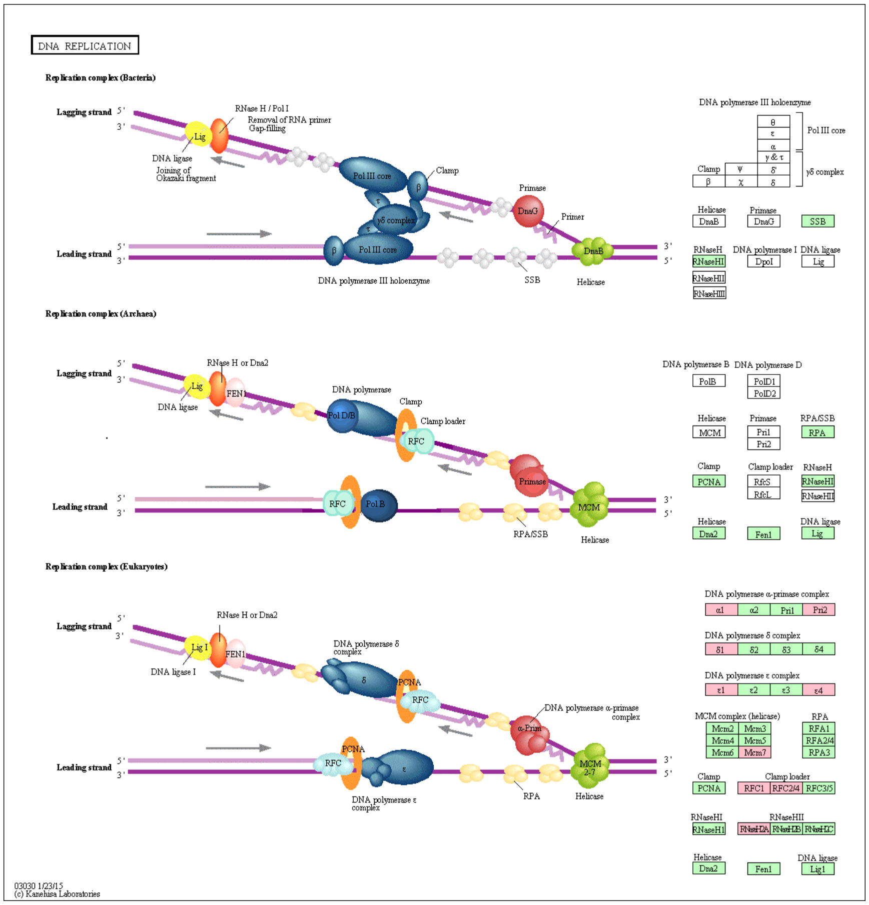

184.3 Legend:

RBH-Blast at 60% Identity + 50% Coverage
Green = Hit in *H. sapiens*
Red = Hit in *H. sapiens* and *C. milli*
White = Not in *H. sapiens*

185 Hedgehog signaling pathway

185.1 Human Pathway: HSA04340

185.2 Number of Hits: 9

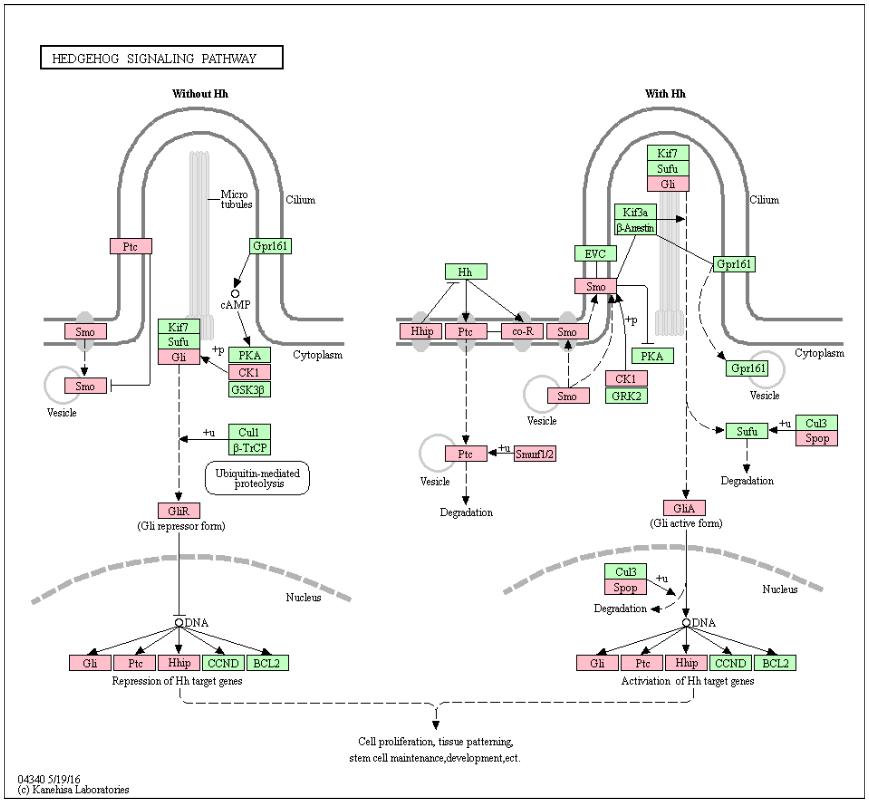

185.3 Legend:

RBH-Blast at 60% Identity + 50% Coverage
Green = Hit in *H. sapiens*
Red = Hit in *H. sapiens* and *C. milli*
White = Not in *H. sapiens*

186 Glycine, serine and threonine metabolism

186.1 Human Pathway: HSA00260

186.2 Number of Hits: 9

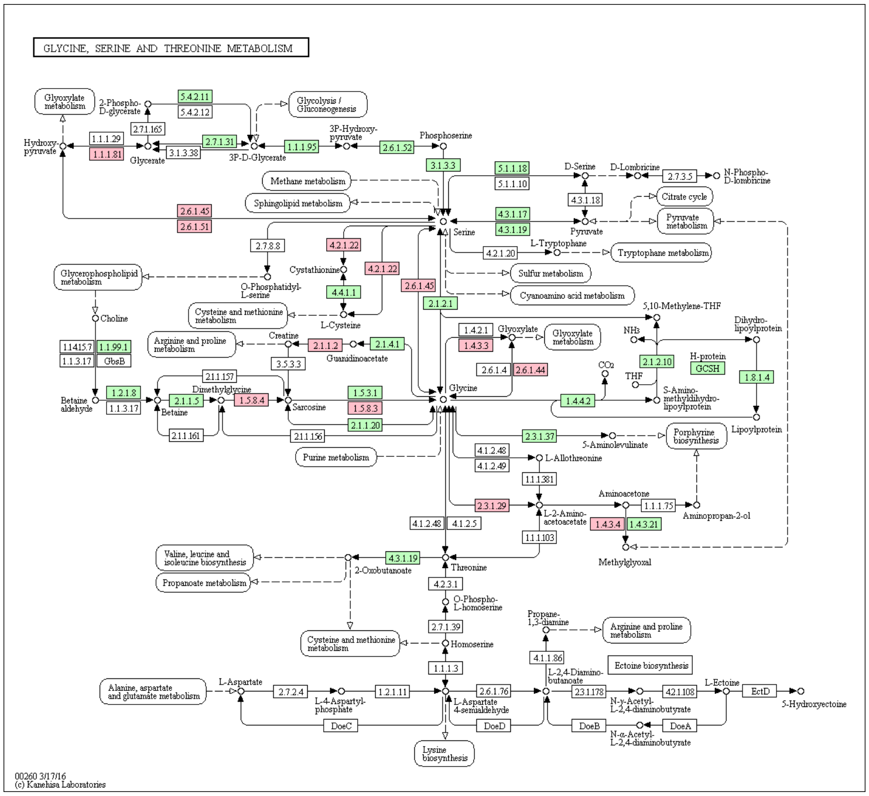

186.3 Legend:

RBH-Blast at 60% Identity + 50% Coverage
Green = Hit in *H. sapiens*
Red = Hit in *H. sapiens* and *C. milli*
White = Not in *H. sapiens*

187 Biosynthesis of amino acids

187.1 Human Pathway: HSA01230

187.2 Number of Hits: 8

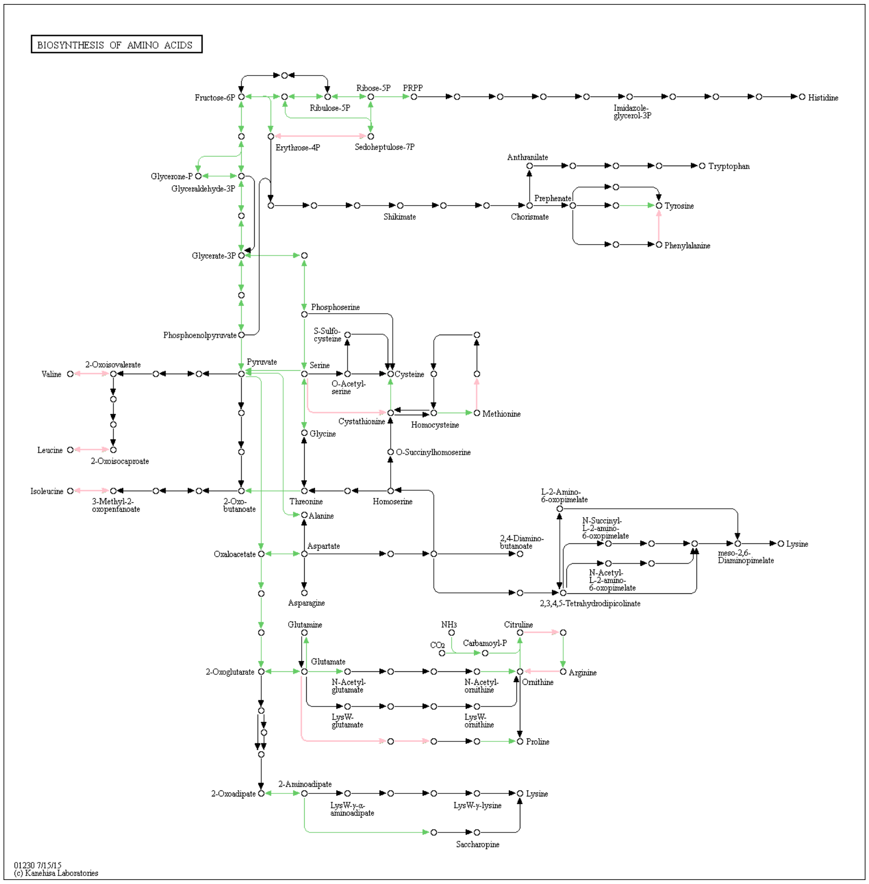

187.3 Legend:

RBH-Blast at 60% Identity + 50% Coverage
Green = Hit in *H. sapiens*
Red = Hit in *H. sapiens* and *C. milli*
White = Not in *H. sapiens*

188 Fatty acid degradation

188.1 Human Pathway: HSA00071

188.2 Number of Hits: 8

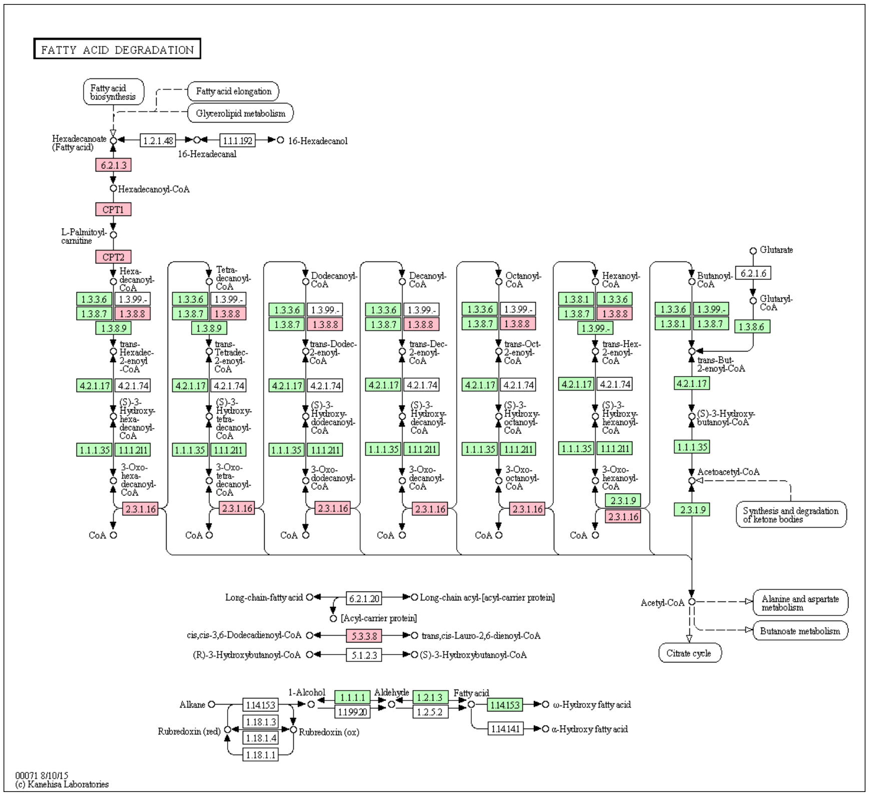

188.3 Legend:

RBH-Blast at 60% Identity + 50% Coverage
Green = Hit in *H. sapiens*
Red = Hit in *H. sapiens* and *C. milli*
White = Not in *H. sapiens*

189 Alanine, aspartate and glutamate metabolism

189.1 Human Pathway: HSA00250

189.2 Number of Hits: 8

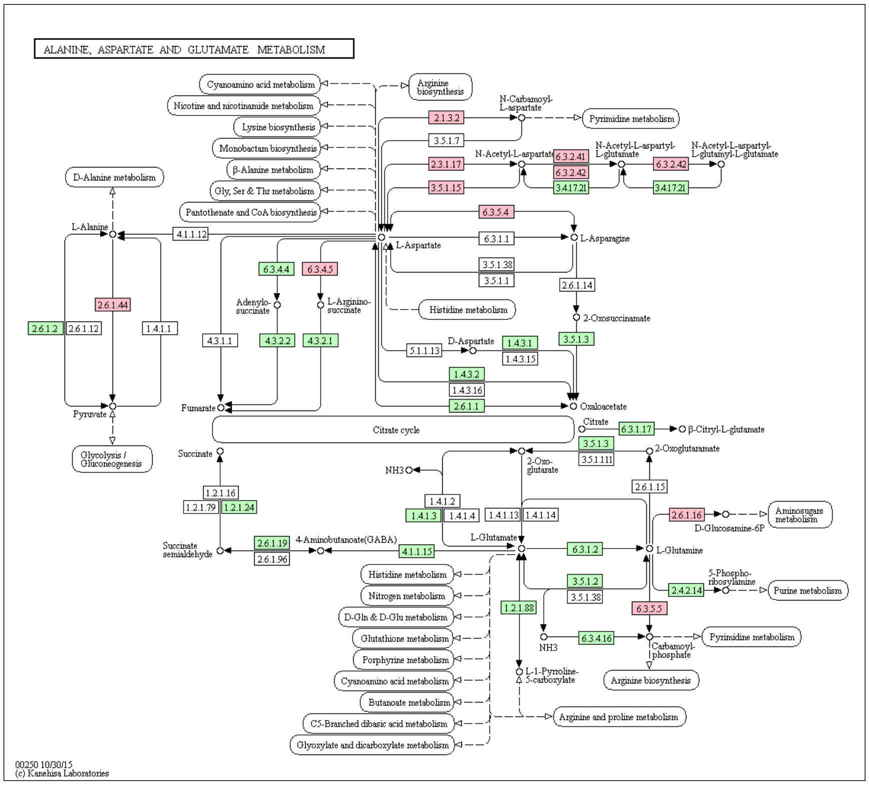

189.3 Legend:

RBH-Blast at 60% Identity + 50% Coverage
Green = Hit in *H. sapiens*
Red = Hit in *H. sapiens* and *C. milli*
White = Not in *H. sapiens*

190 Pyruvate metabolism

190.1 Human Pathway: HSA00620

190.2 Number of Hits: 8

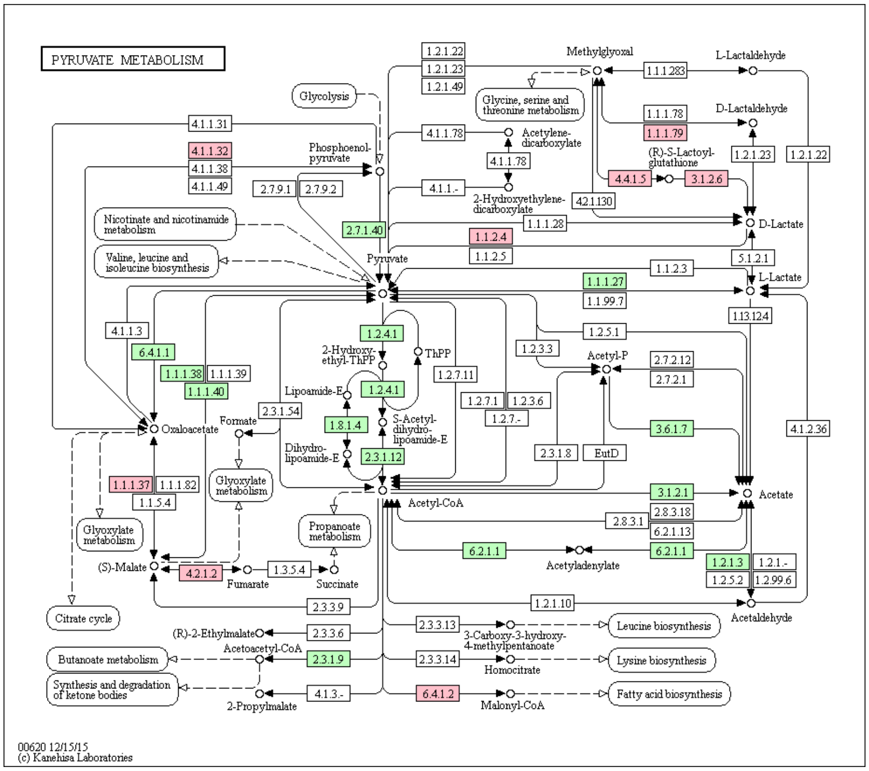

190.3 Legend:

RBH-Blast at 60% Identity + 50% Coverage
Green = Hit in *H. sapiens*
Red = Hit in *H. sapiens* and *C. milli*
White = Not in *H. sapiens*

191 Aldosterone-regulated sodium reabsorption

191.1 Human Pathway: HSA04960

191.2 Number of Hits: 8

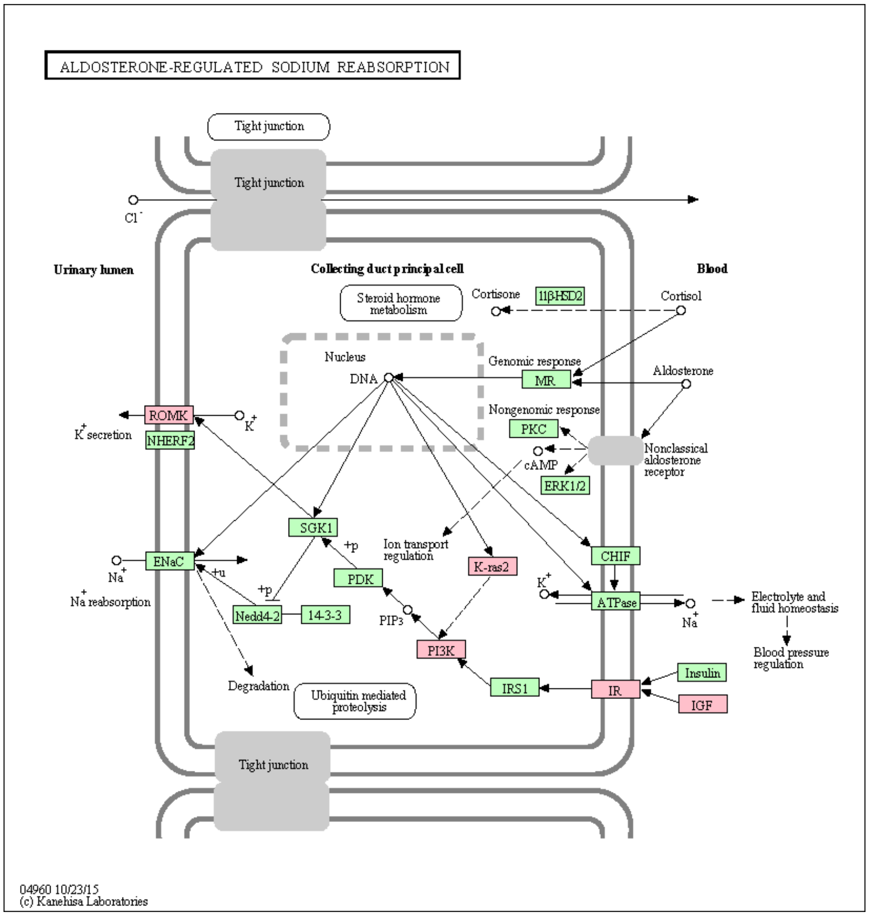

191.3 Legend:

RBH-Blast at 60% Identity + 50% Coverage
Green = Hit in *H. sapiens*
Red = Hit in *H. sapiens* and *C. milli*
White = Not in *H. sapiens*

192 Drug metabolism - cytochrome P450

192.1 Human Pathway: HSA00982

192.2 Number of Hits: 8

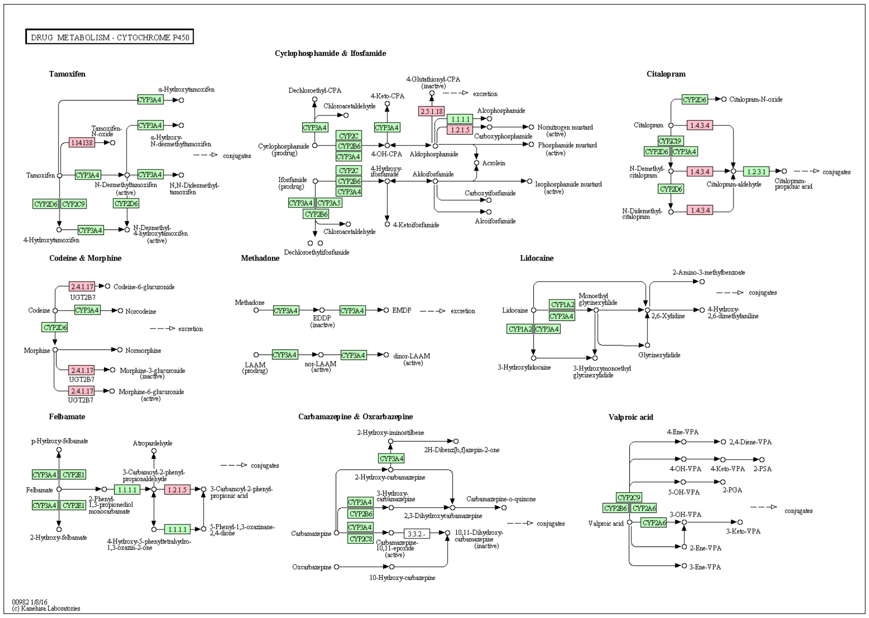

192.3 Legend:

RBH-Blast at 60% Identity + 50% Coverage
Green = Hit in *H. sapiens*
Red = Hit in *H. sapiens* and *C. milli*
White = Not in *H. sapiens*

193 Vasopressin-regulated water reabsorption

193.1 Human Pathway: HSA04962

193.2 Number of Hits: 8

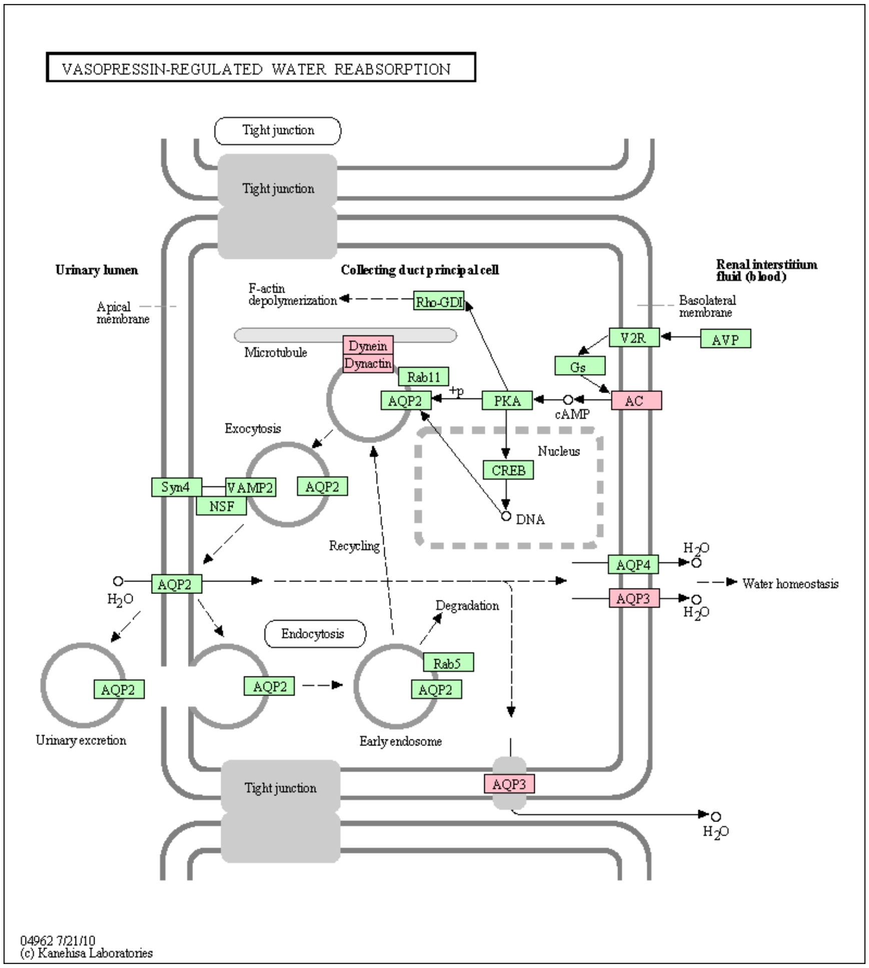

193.3 Legend:

RBH-Blast at 60% Identity + 50% Coverage
Green = Hit in *H. sapiens*
Red = Hit in *H. sapiens* and *C. milli*
White = Not in *H. sapiens*

194 Lysine degradation

194.1 Human Pathway: HSA00310

194.2 Number of Hits: 8

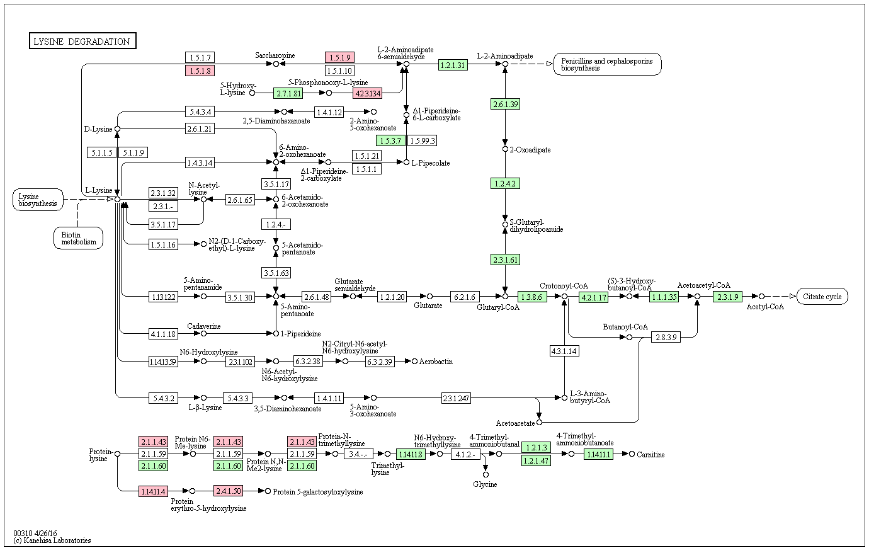

194.3 Legend:

RBH-Blast at 60% Identity + 50% Coverage
Green = Hit in *H. sapiens*
Red = Hit in *H. sapiens* and *C. milli*
White = Not in *H. sapiens*

195 Aminoacyl-tRNA biosynthesis

195.1 Human Pathway: HSA00970

195.2 Number of Hits: 8

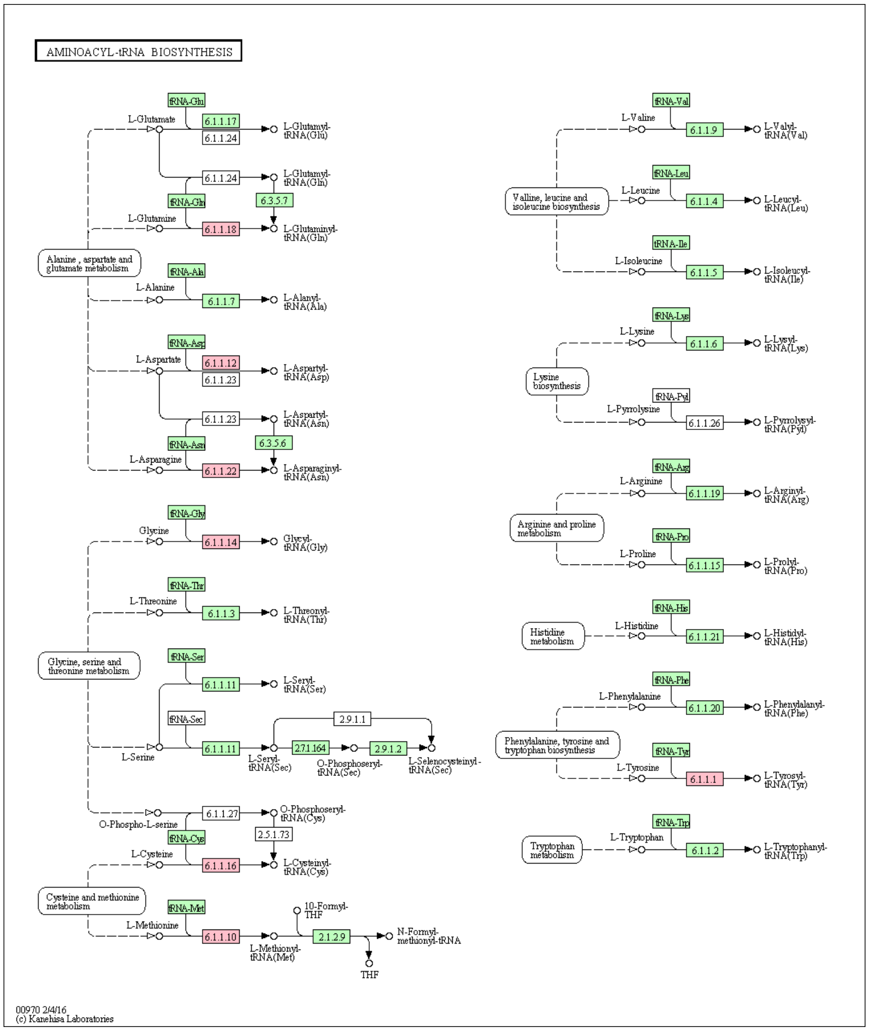

195.3 Legend:

RBH-Blast at 60% Identity + 50% Coverage
Green = Hit in *H. sapiens*
Red = Hit in *H. sapiens* and *C. milli*
White = Not in *H. sapiens*

196 Glycolysis / Gluconeogenesis

196.1 Human Pathway: HSA00010

196.2 Number of Hits: 8

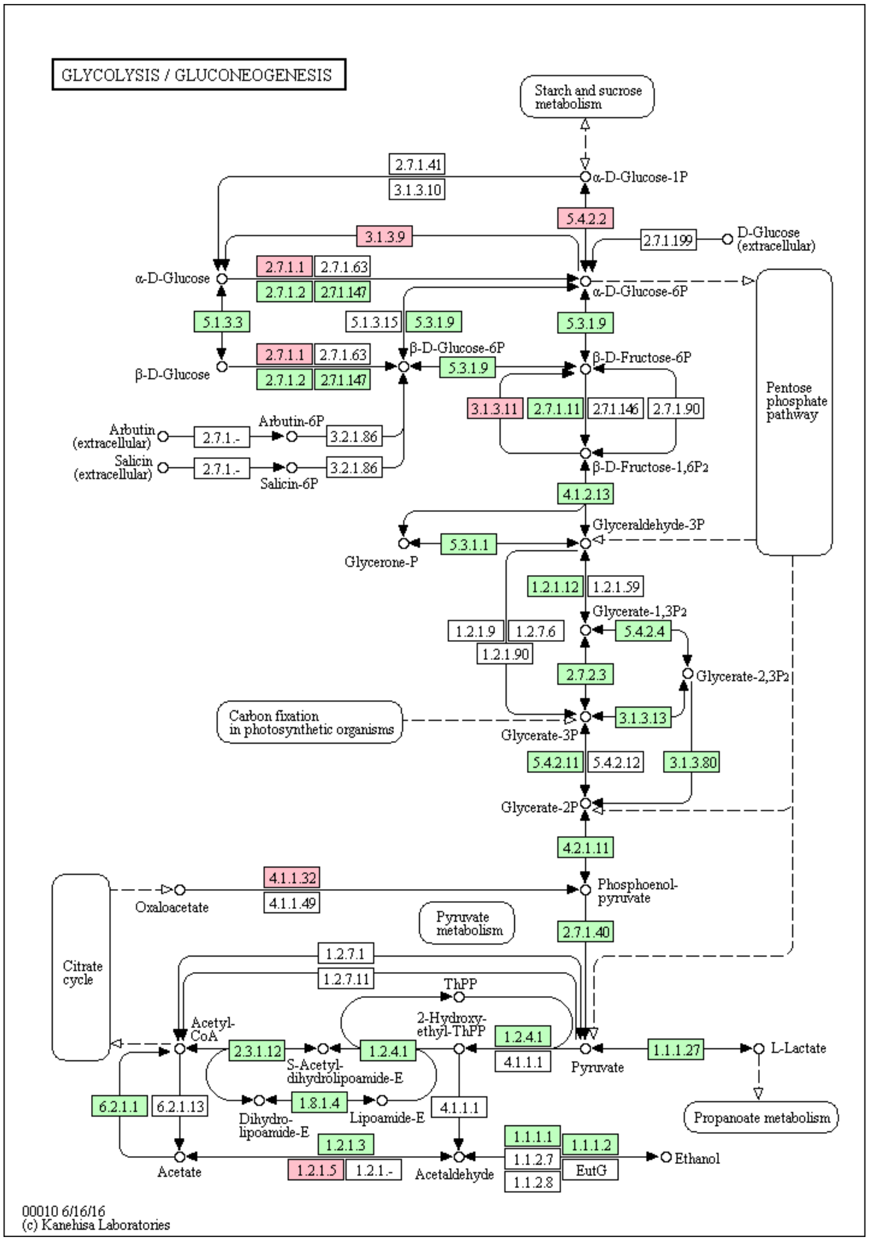

196.3 Legend:

RBH-Blast at 60% Identity + 50% Coverage
Green = Hit in *H. sapiens*
Red = Hit in *H. sapiens* and *C. milli*
White = Not in *H. sapiens*

197 Glycosylphosphatidylinositol(GPI)-anchor biosynthesis

197.1 Human Pathway: HSA00563

197.2 Number of Hits: 8

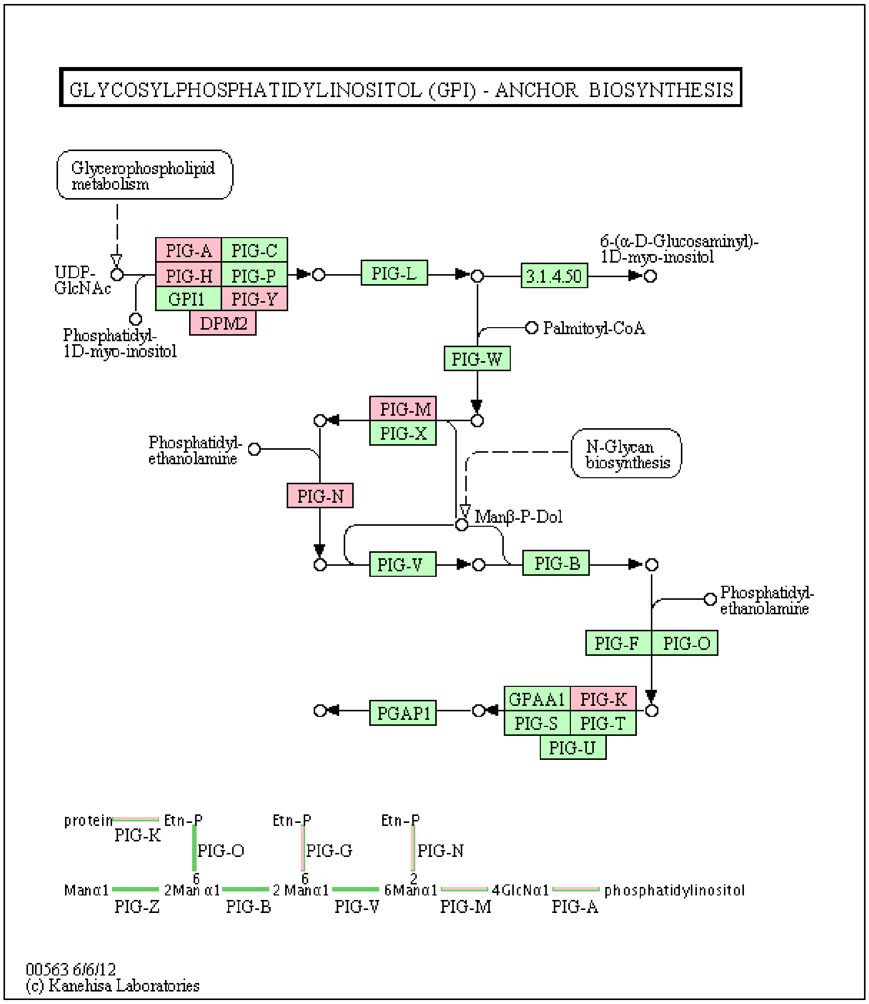

197.3 Legend:

RBH-Blast at 60% Identity + 50% Coverage
Green = Hit in *H. sapiens*
Red = Hit in *H. sapiens* and *C. milli*
White = Not in *H. sapiens*

198 Nucleotide excision repair

198.1 Human Pathway: HSA03420

198.2 Number of Hits: 8

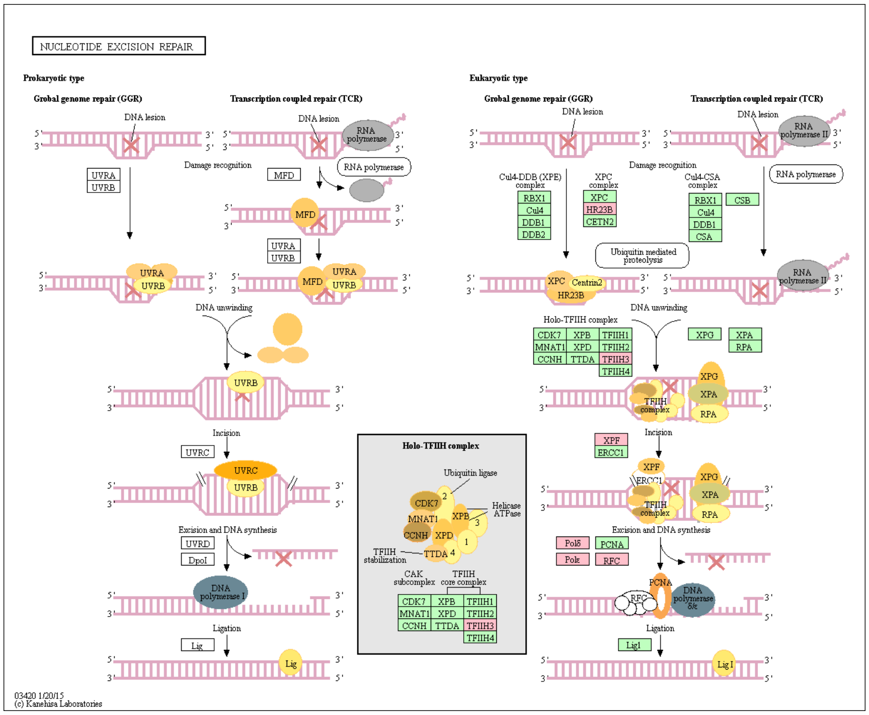

198.3 Legend:

RBH-Blast at 60% Identity + 50% Coverage
Green = Hit in *H. sapiens*
Red = Hit in *H. sapiens* and *C. milli*
White = Not in *H. sapiens*

199 Arachidonic acid metabolism

199.1 Human Pathway: HSA00590

199.2 Number of Hits: 8

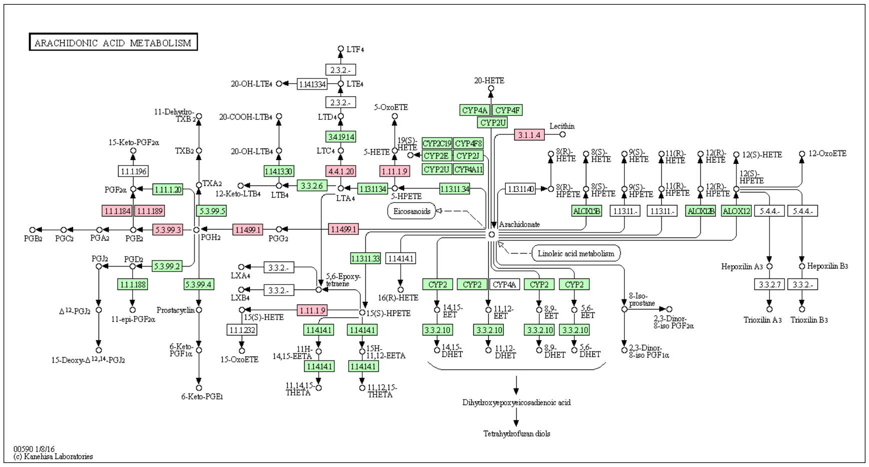

199.3 Legend:

RBH-Blast at 60% Identity + 50% Coverage
Green = Hit in *H. sapiens*
Red = Hit in *H. sapiens* and *C. milli*
White = Not in *H. sapiens*

200 NOD-like receptor signaling pathway

200.1 Human Pathway: HSA04621

200.2 Number of Hits: 8

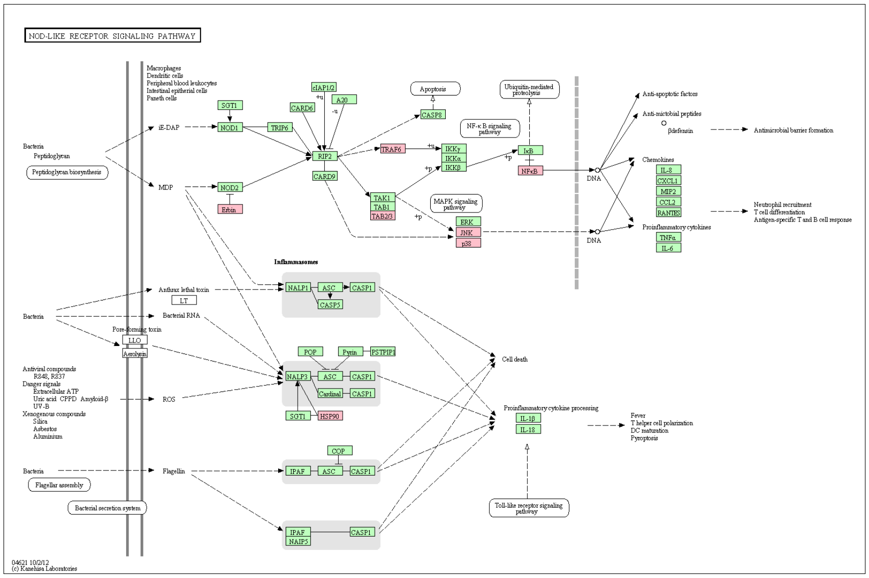

200.3 Legend:

RBH-Blast at 60% Identity + 50% Coverage
Green = Hit in *H. sapiens*
Red = Hit in *H. sapiens* and *C. milli*
White = Not in *H. sapiens*

201 Fanconi anemia pathway

201.1 Human Pathway: HSA03460

201.2 Number of Hits: 8

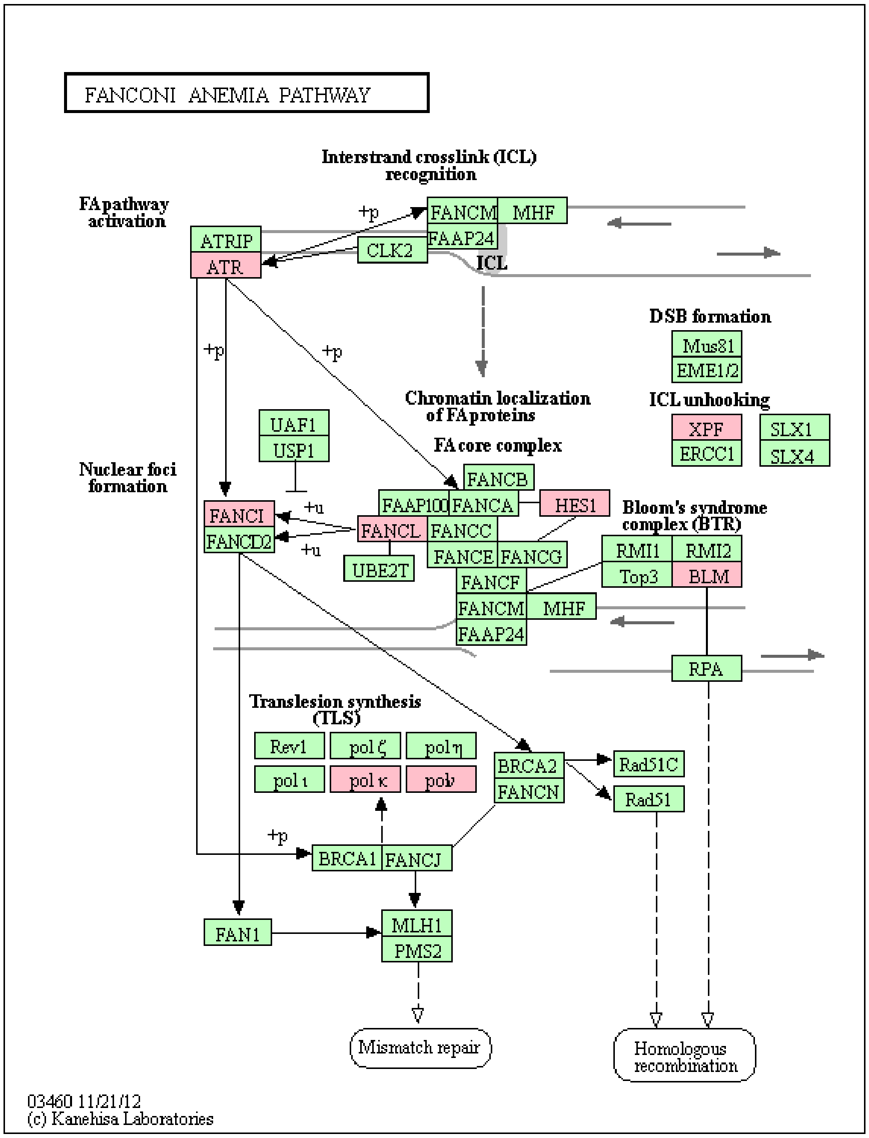

201.3 Legend:

RBH-Blast at 60% Identity + 50% Coverage
Green = Hit in *H. sapiens*
Red = Hit in *H. sapiens* and *C. milli*
White = Not in *H. sapiens*

202 Base excision repair

202.1 Human Pathway: HSA03410

202.2 Number of Hits: 8

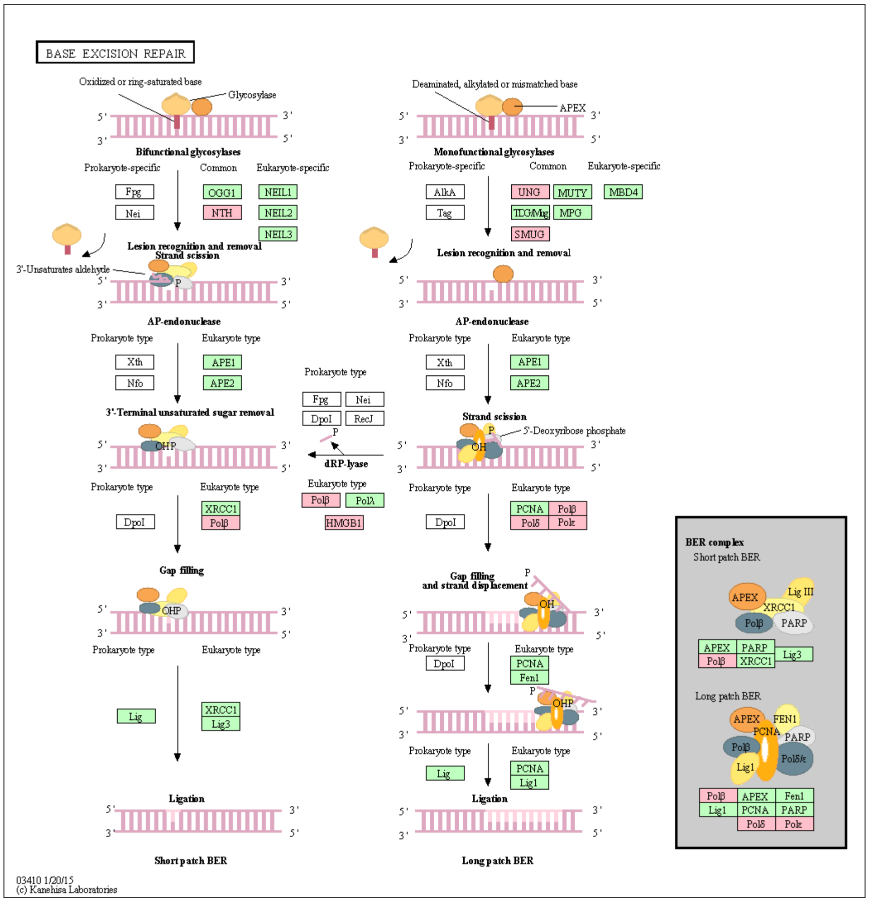

202.3 Legend:

RBH-Blast at 60% Identity + 50% Coverage
Green = Hit in *H. sapiens*
Red = Hit in *H. sapiens* and *C. milli*
White = Not in *H. sapiens*

---

203 Mineral absorption

203.1 Human Pathway: HSA04978

203.2 Number of Hits: 8

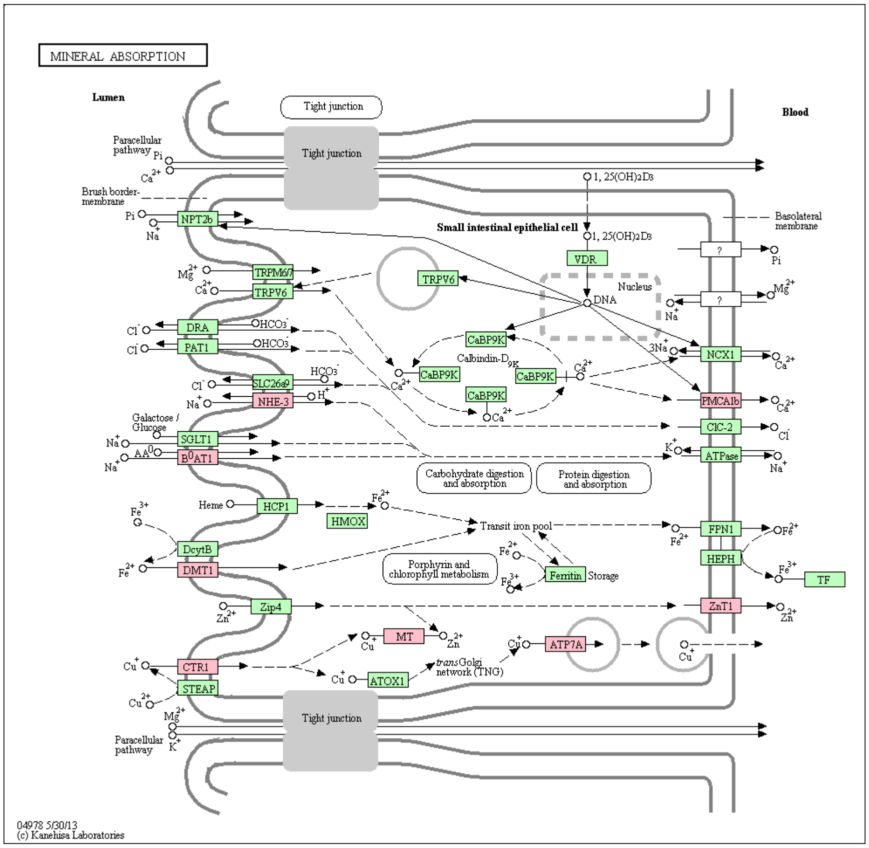

203.3 Legend:

RBH-Blast at 60% Identity + 50% Coverage
Green = Hit in *H. sapiens*
Red = Hit in *H. sapiens* and *C. milli*
White = Not in *H. sapiens*

204 Valine, leucine and isoleucine degradation

204.1 Human Pathway: HSA00280

204.2 Number of Hits: 7

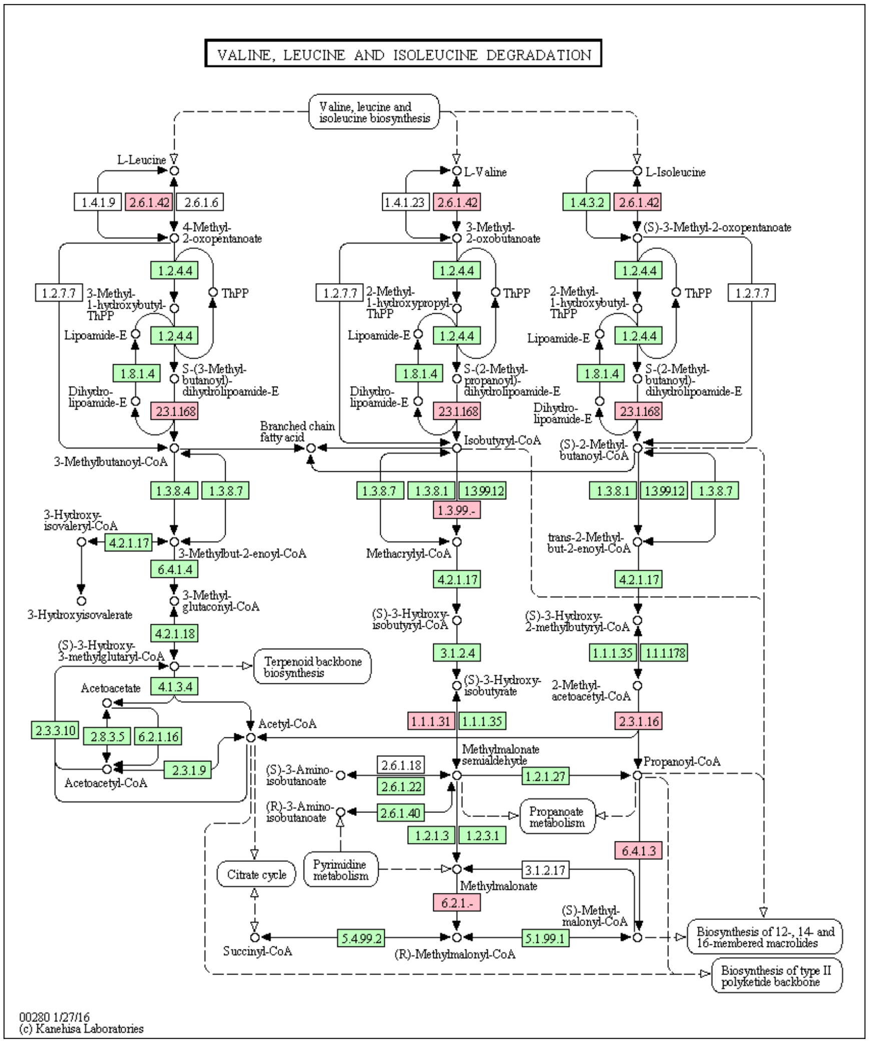

204.3 Legend:

RBH-Blast at 60% Identity + 50% Coverage
Green = Hit in *H. sapiens*
Red = Hit in *H. sapiens* and *C. milli*
White = Not in *H. sapiens*

---

205 Basal transcription factors

205.1 Human Pathway: HSA03022

205.2 Number of Hits: 7

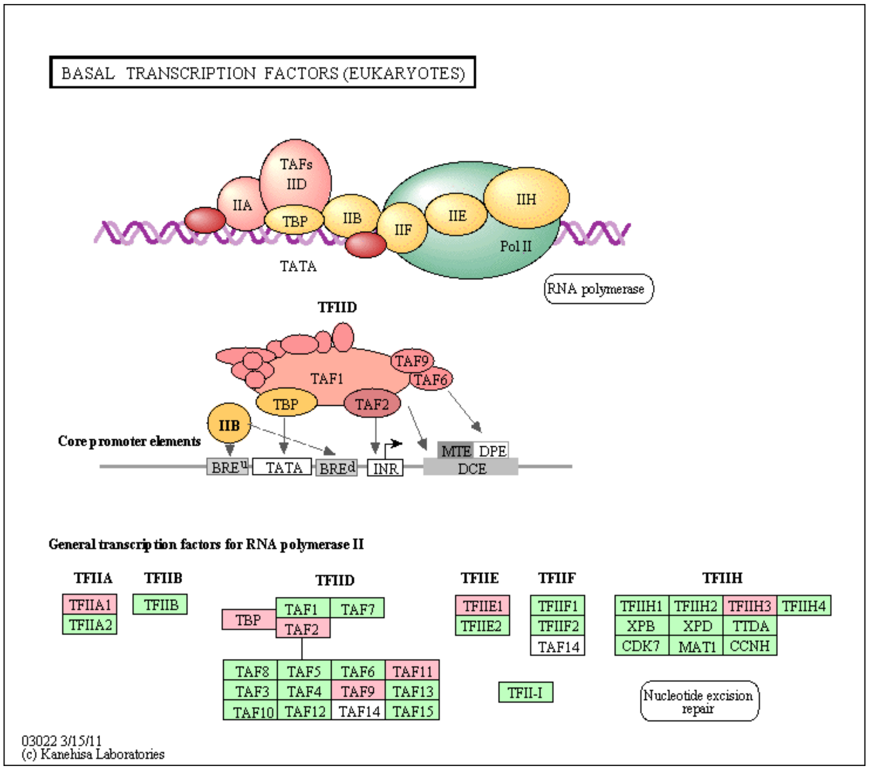

205.3 Legend:

RBH-Blast at 60% Identity + 50% Coverage
Green = Hit in *H. sapiens*
Red = Hit in *H. sapiens* and *C. milli*
White = Not in *H. sapiens*

206 Leishmaniasis

206.1 Human Pathway: HSA05140

206.2 Number of Hits: 7

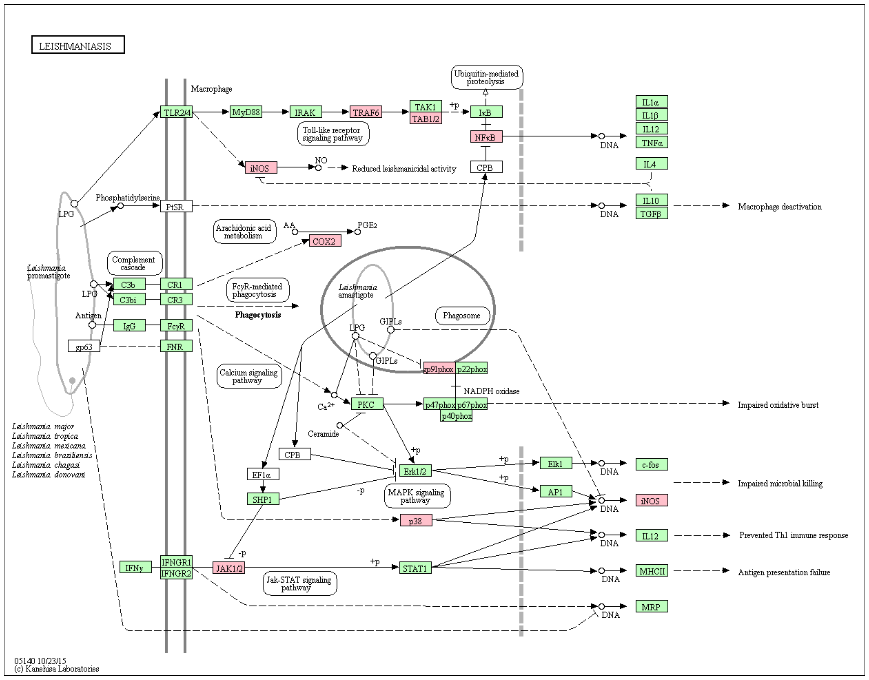

206.3 Legend:

RBH-Blast at 60% Identity + 50% Coverage
Green = Hit in *H. sapiens*
Red = Hit in *H. sapiens* and *C. milli*
White = Not in *H. sapiens*

207 Steroid hormone biosynthesis

207.1 Human Pathway: HSA00140

207.2 Number of Hits: 7

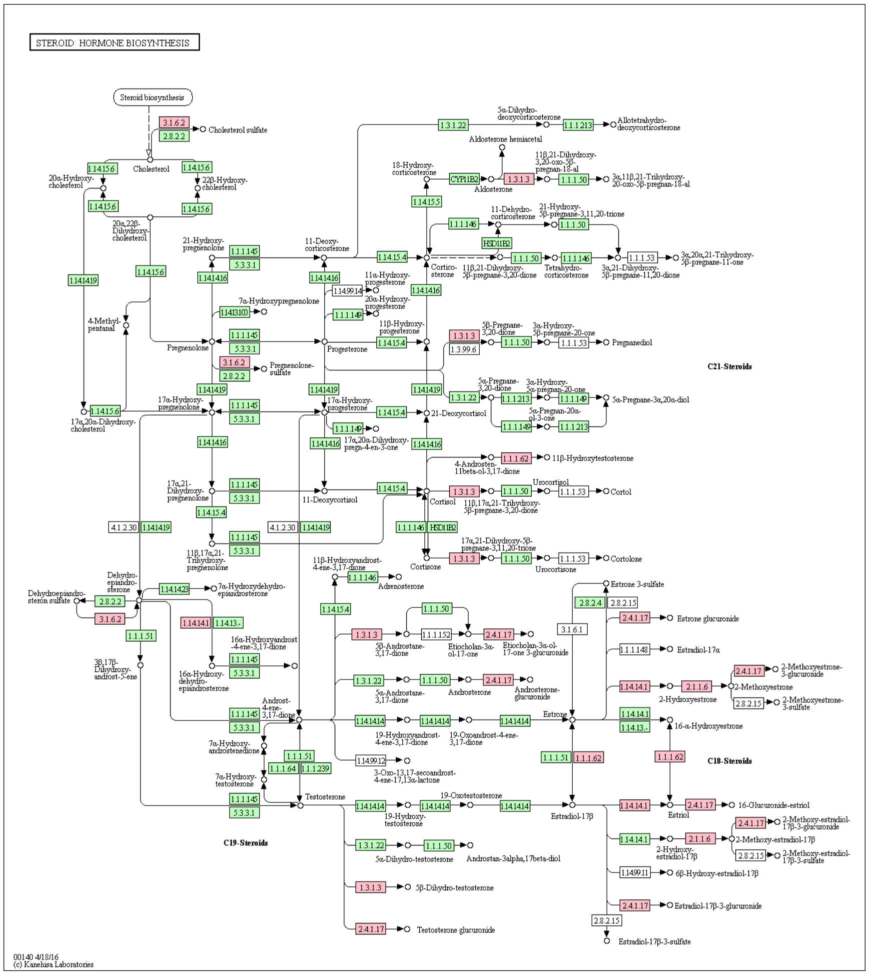

207.3 Legend:

RBH-Blast at 60% Identity + 50% Coverage
Green = Hit in *H. sapiens*
Red = Hit in *H. sapiens* and *C. milli*
White = Not in *H. sapiens*

208 beta-Alanine metabolism

208.1 Human Pathway: HSA00410

208.2 Number of Hits: 7

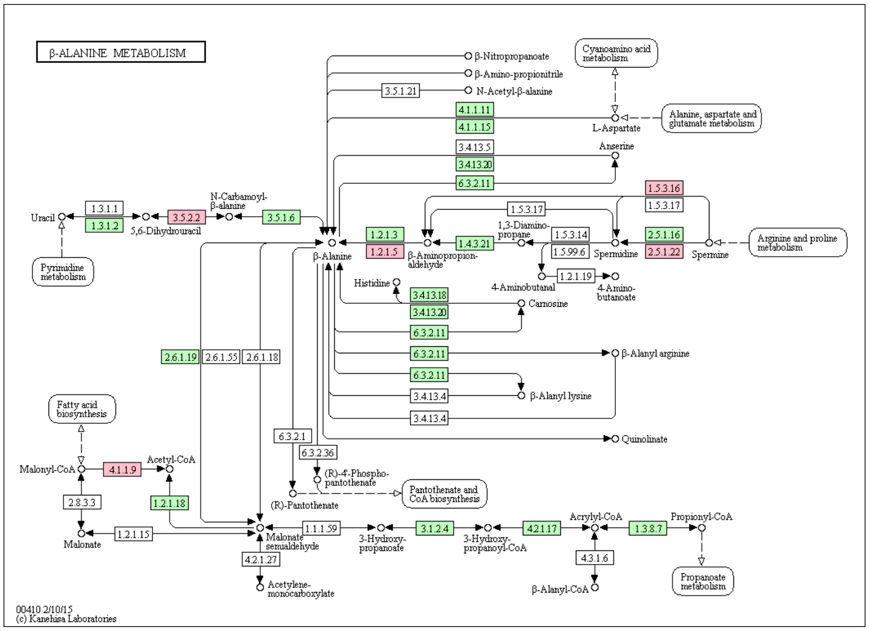

208.3 Legend:

RBH-Blast at 60% Identity + 50% Coverage
Green = Hit in *H. sapiens*
Red = Hit in *H. sapiens* and *C. milli*
White = Not in *H. sapiens*

209 Hippo signaling pathway -multiple species

209.1 Human Pathway: HSA04392

209.2 Number of Hits: 7

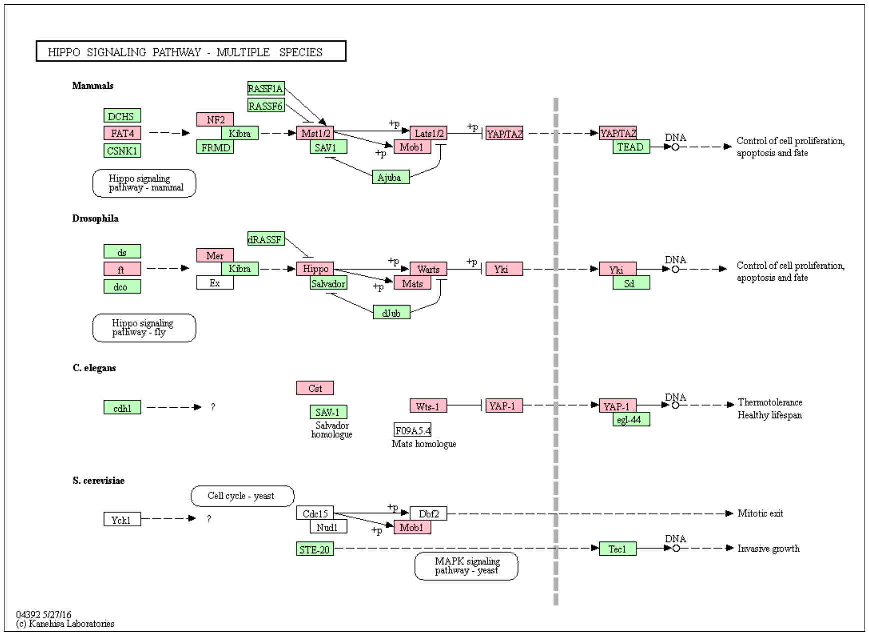

209.3 Legend:

RBH-Blast at 60% Identity + 50% Coverage
Green = Hit in *H. sapiens*
Red = Hit in *H. sapiens* and *C. milli*
White = Not in *H. sapiens*

210 RIG-I-like receptor signaling pathway

210.1 Human Pathway: HSA04622

210.2 Number of Hits: 7

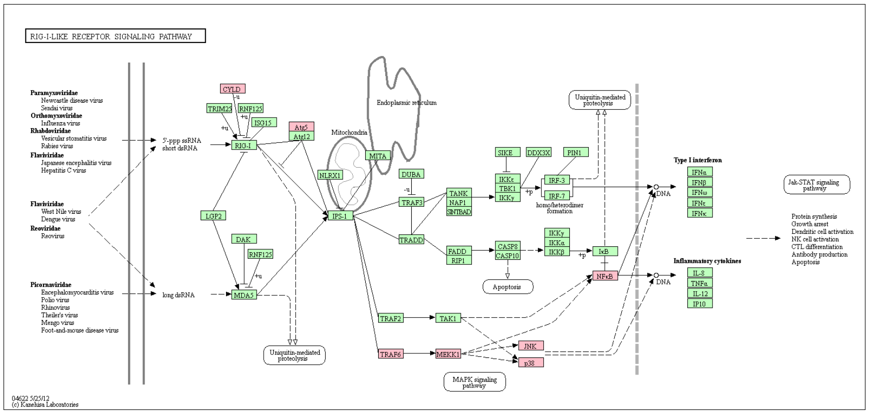

210.3 Legend:

RBH-Blast at 60% Identity + 50% Coverage
Green = Hit in *H. sapiens*
Red = Hit in *H. sapiens* and *C. milli*
White = Not in *H. sapiens*

211 Circadian rhythm

211.1 Human Pathway: HSA04710

211.2 Number of Hits: 7

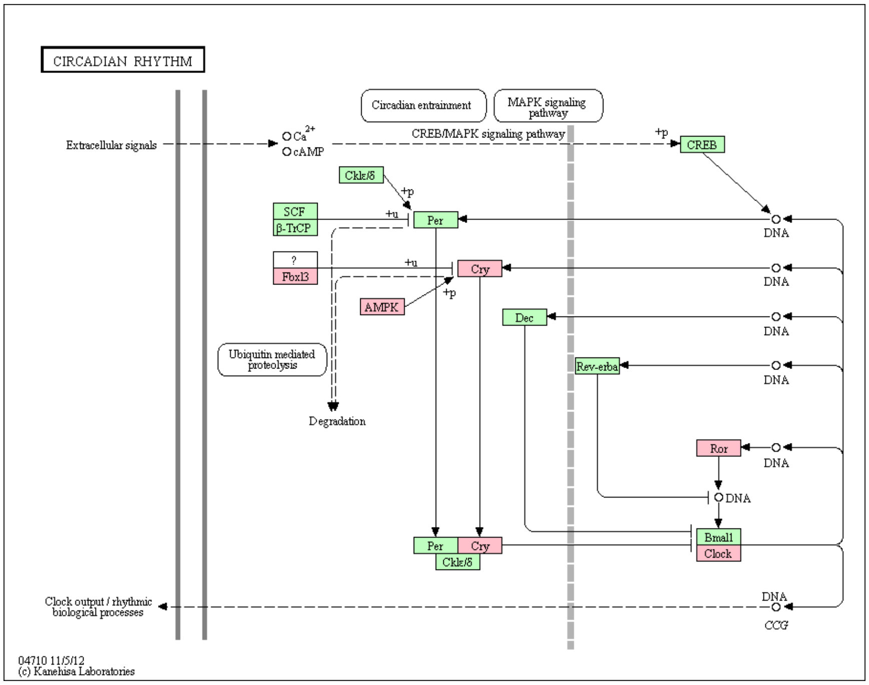

211.3 Legend:

RBH-Blast at 60% Identity + 50% Coverage
Green = Hit in *H. sapiens*
Red = Hit in *H. sapiens* and *C. milli*
White = Not in *H. sapiens*

212 Pertussis

212.1 Human Pathway: HSA05133

212.2 Number of Hits: 7

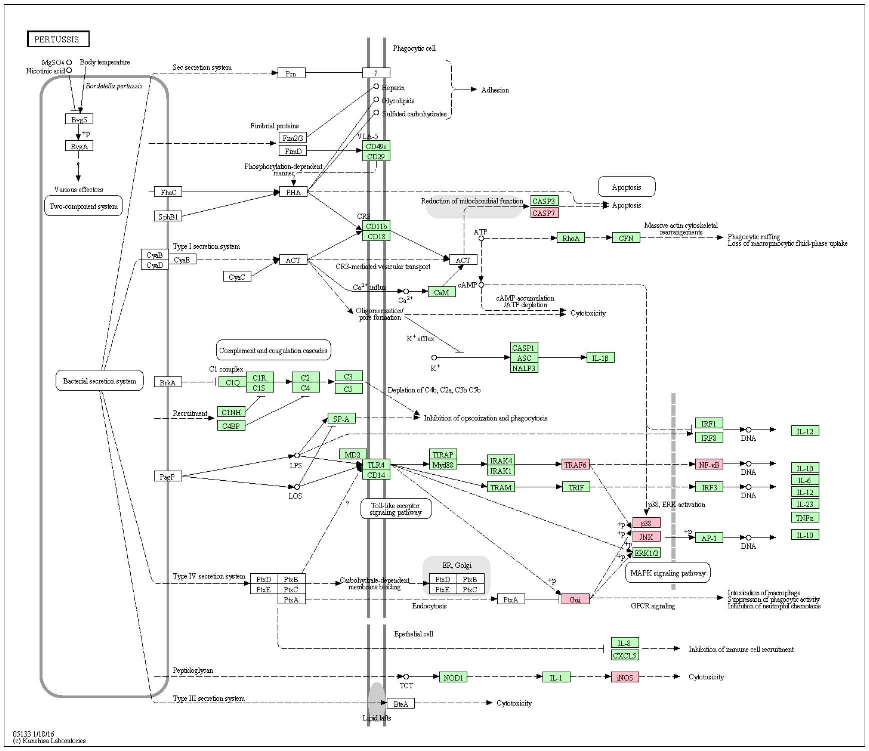

212.3 Legend:

RBH-Blast at 60% Identity + 50% Coverage
Green = Hit in *H. sapiens*
Red = Hit in *H. sapiens* and *C. milli*
White = Not in *H. sapiens*

213 Histidine metabolism

213.1 Human Pathway: HSA00340

213.2 Number of Hits: 7

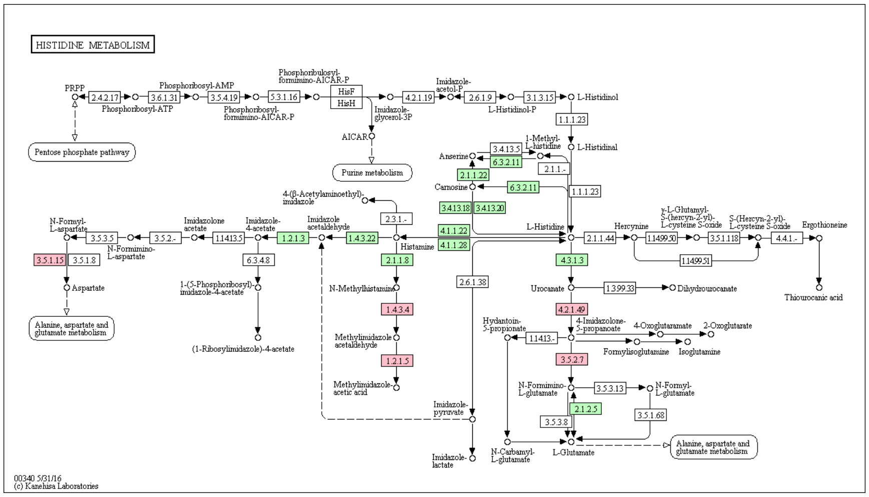

213.3 Legend:

RBH-Blast at 60% Identity + 50% Coverage
Green = Hit in *H. sapiens*
Red = Hit in *H. sapiens* and *C. milli*
White = Not in *H. sapiens*

214 Phenylalanine metabolism

214.1 Human Pathway: HSA00360

214.2 Number of Hits: 6

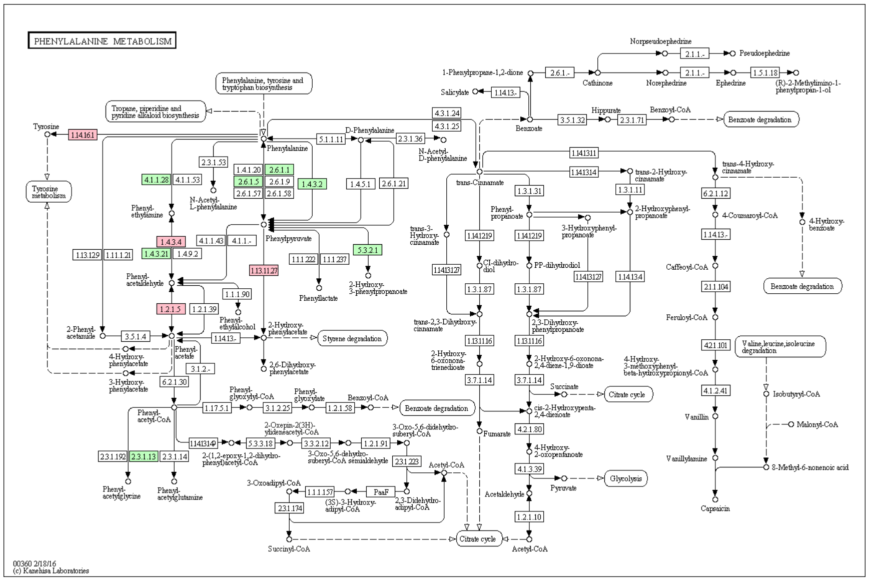

214.3 Legend:

RBH-Blast at 60% Identity + 50% Coverage
Green = Hit in *H. sapiens*
Red = Hit in *H. sapiens* and *C. milli*
White = Not in *H. sapiens*

215 Rheumatoid arthritis

215.1 Human Pathway: HSA05323

215.2 Number of Hits: 6

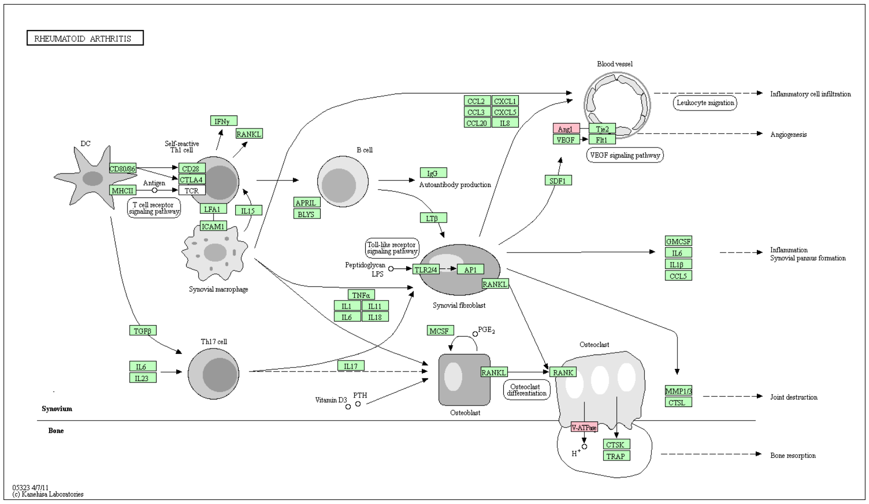

215.3 Legend:

RBH-Blast at 60% Identity + 50% Coverage
Green = Hit in *H. sapiens*
Red = Hit in *H. sapiens* and *C. milli*
White = Not in *H. sapiens*

216 Maturity onset diabetes of the young

216.1 Human Pathway: HSA04950

216.2 Number of Hits: 6

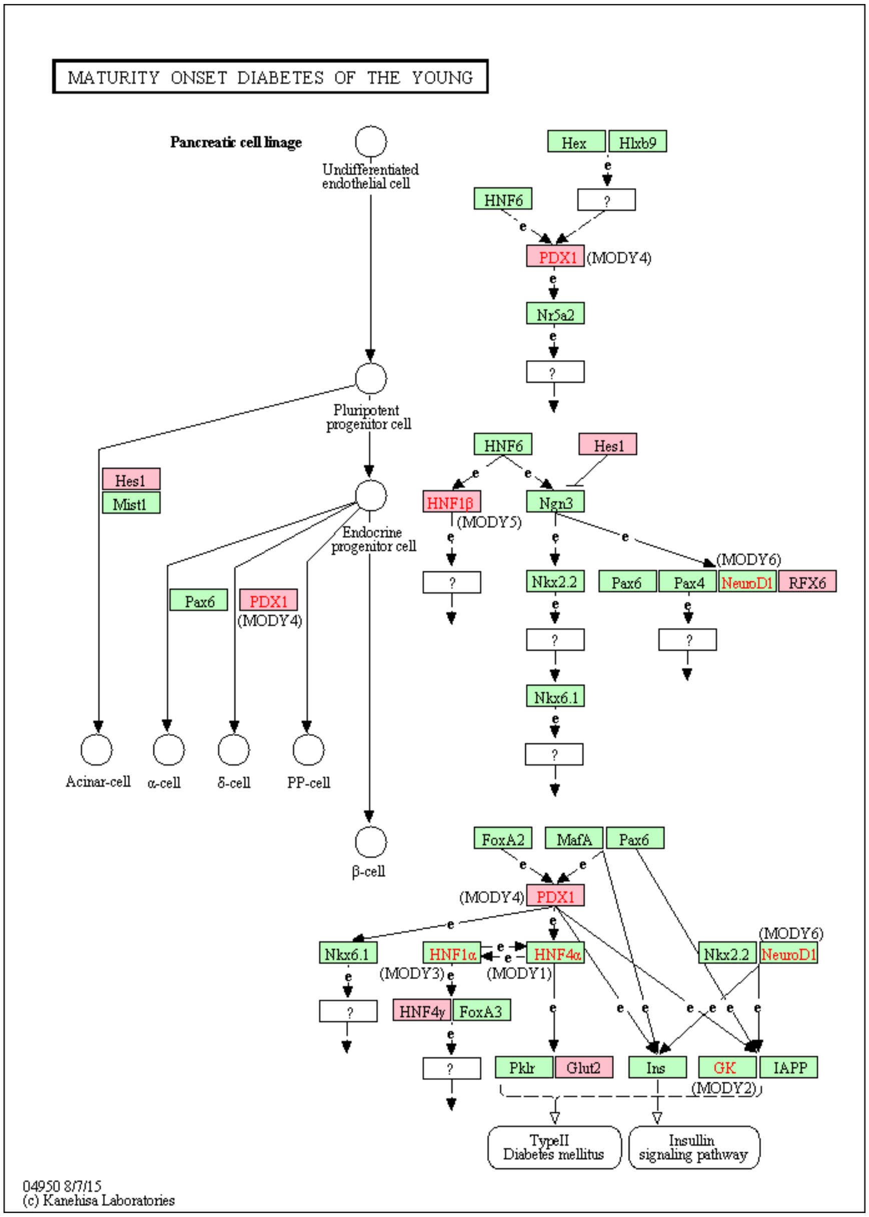

216.3 Legend:

RBH-Blast at 60% Identity + 50% Coverage
Green = Hit in *H. sapiens*
Red = Hit in *H. sapiens* and *C. milli*
White = Not in *H. sapiens*

217 Fatty acid elongation

217.1 Human Pathway: HSA00062

217.2 Number of Hits: 6

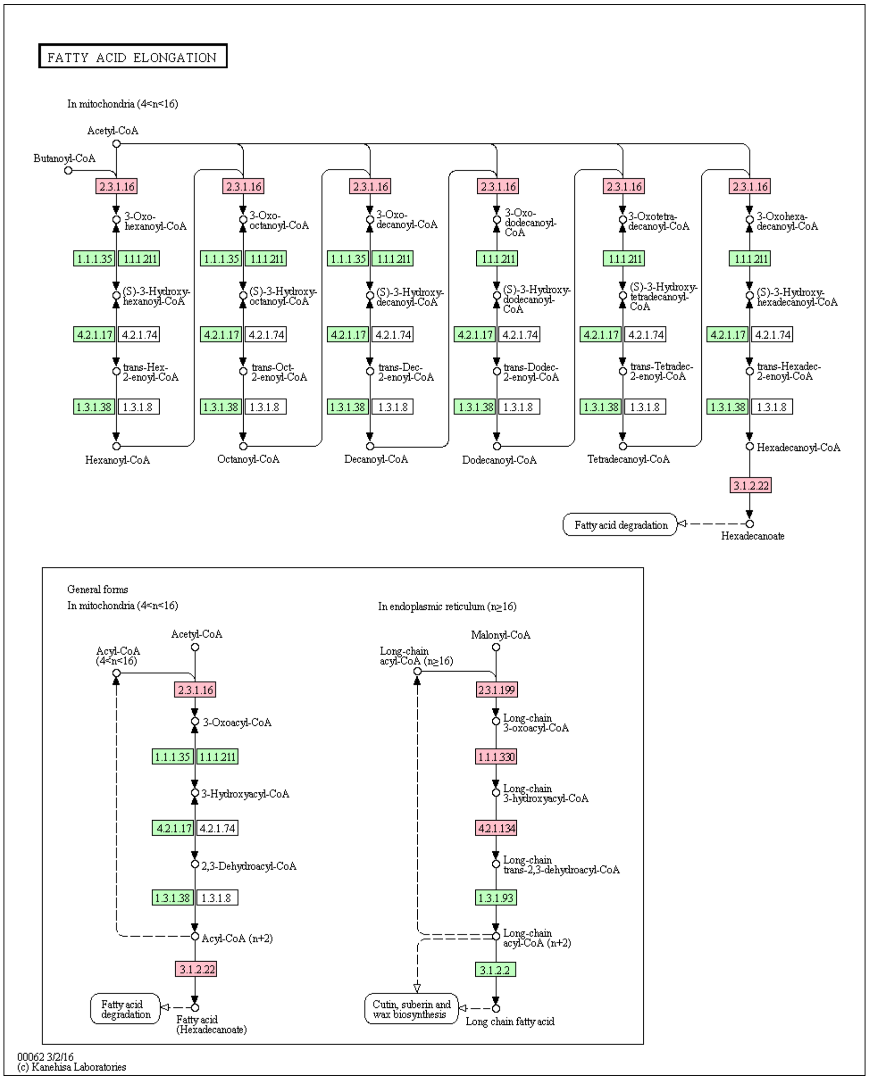

217.3 Legend:

RBH-Blast at 60% Identity + 50% Coverage
Green = Hit in *H. sapiens*
Red = Hit in *H. sapiens* and *C. milli*
White = Not in *H. sapiens*

---

218 Galactose metabolism

218.1 Human Pathway: HSA00052

218.2 Number of Hits: 6

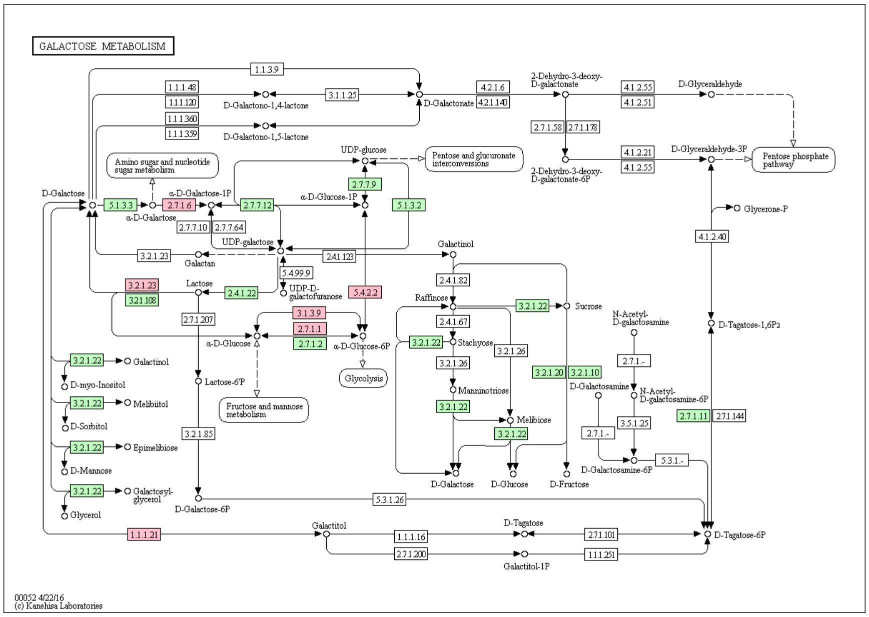

218.3 Legend:

RBH-Blast at 60% Identity + 50% Coverage
Green = Hit in *H. sapiens*
Red = Hit in *H. sapiens* and *C. milli*
White = Not in *H. sapiens*

---

219 Steroid biosynthesis

219.1 Human Pathway: HSA00100

219.2 Number of Hits: 6

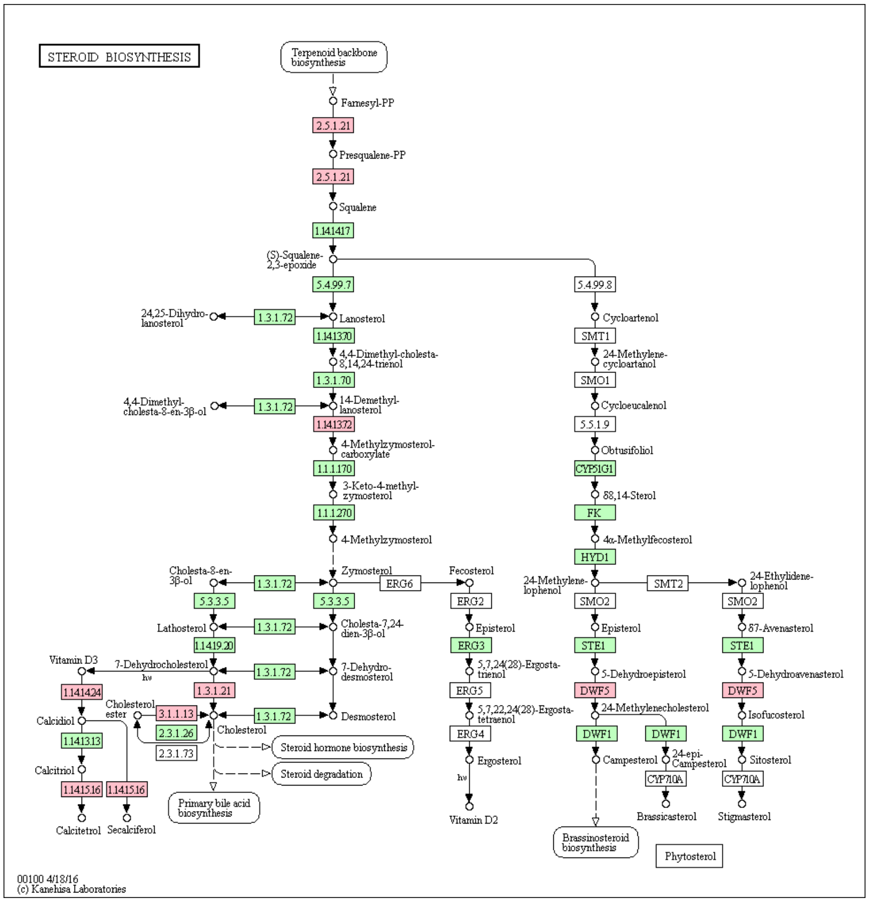

219.3 Legend:

RBH-Blast at 60% Identity + 50% Coverage
Green = Hit in *H. sapiens*
Red = Hit in *H. sapiens* and *C. milli*
White = Not in *H. sapiens*

220 Fat digestion and absorption

220.1 Human Pathway: HSA04975

220.2 Number of Hits: 6

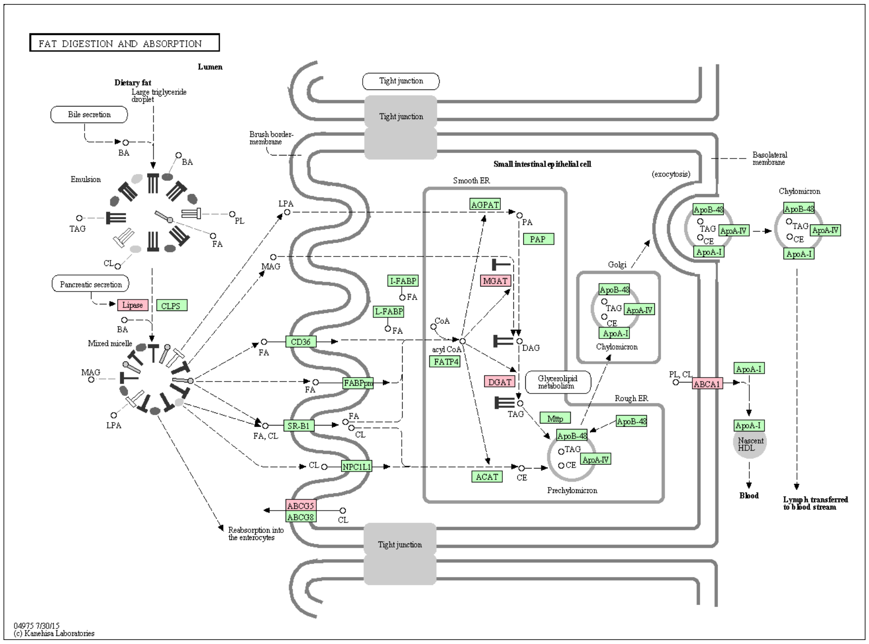

220.3 Legend:

RBH-Blast at 60% Identity + 50% Coverage
Green = Hit in *H. sapiens*
Red = Hit in *H. sapiens* and *C. milli*
White = Not in *H. sapiens*

221 Glyoxylate and dicarboxylate metabolism

221.1 Human Pathway: HSA00630

221.2 Number of Hits: 6

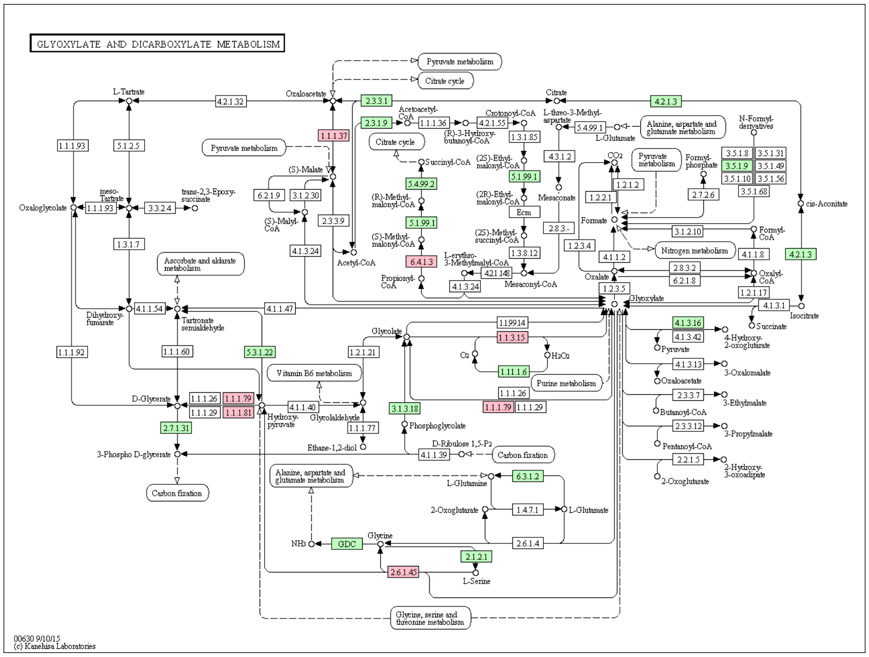

221.3 Legend:

RBH-Blast at 60% Identity + 50% Coverage
Green = Hit in *H. sapiens*
Red = Hit in *H. sapiens* and *C. milli*
White = Not in *H. sapiens*

222 Inflammatory bowel disease (IBD)

222.1 Human Pathway: HSA05321

222.2 Number of Hits: 6

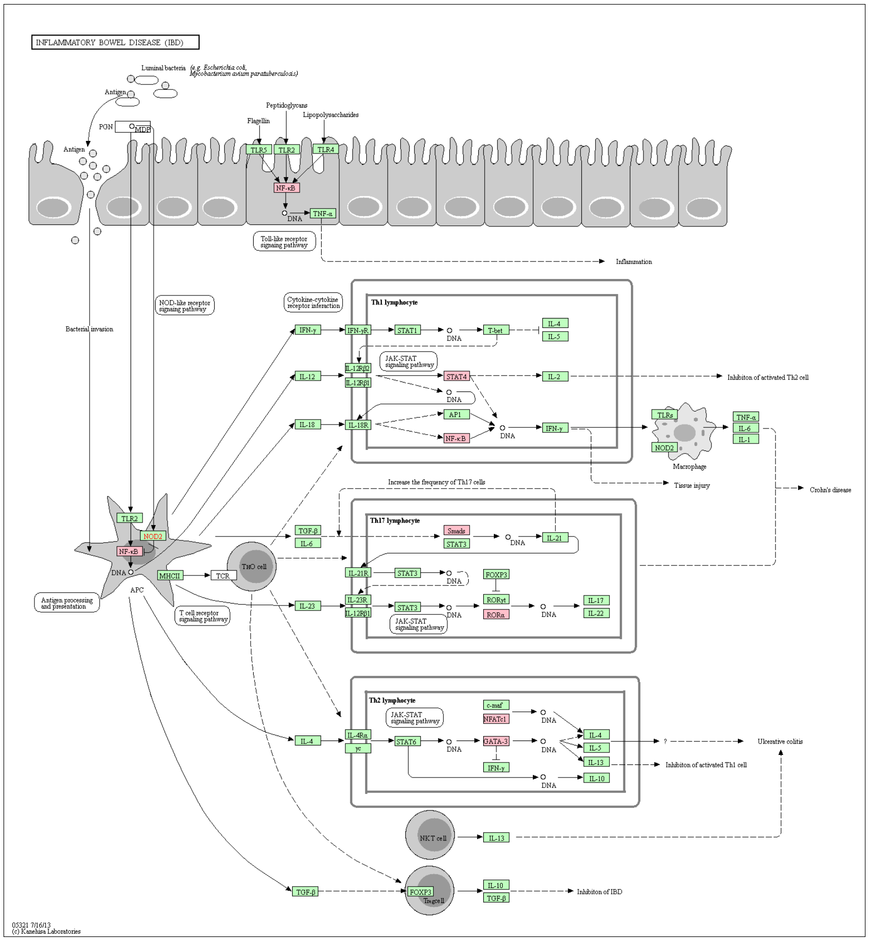

222.3 Legend:

RBH-Blast at 60% Identity + 50% Coverage
Green = Hit in *H. sapiens*
Red = Hit in *H. sapiens* and *C. milli*
White = Not in *H. sapiens*

223 Pathogenic Escherichia coli infection

223.1 Human Pathway: HSA05130

223.2 Number of Hits: 5

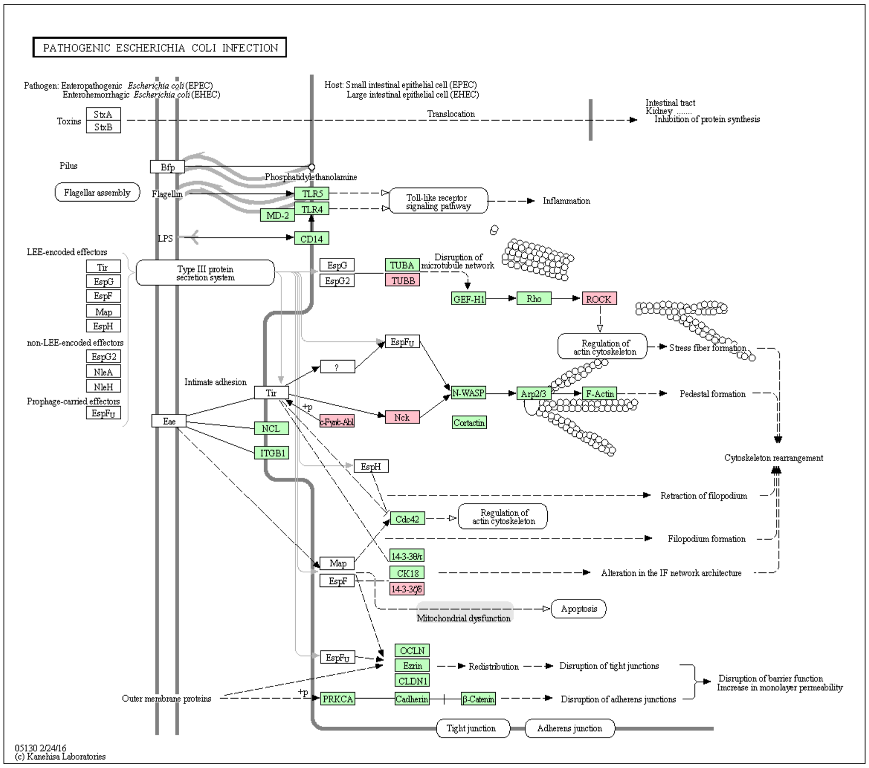

223.3 Legend:

RBH-Blast at 60% Identity + 50% Coverage
Green = Hit in *H. sapiens*
Red = Hit in *H. sapiens* and *C. milli*
White = Not in *H. sapiens*

224 Fructose and mannose metabolism

224.1 Human Pathway: HSA00051

224.2 Number of Hits: 5

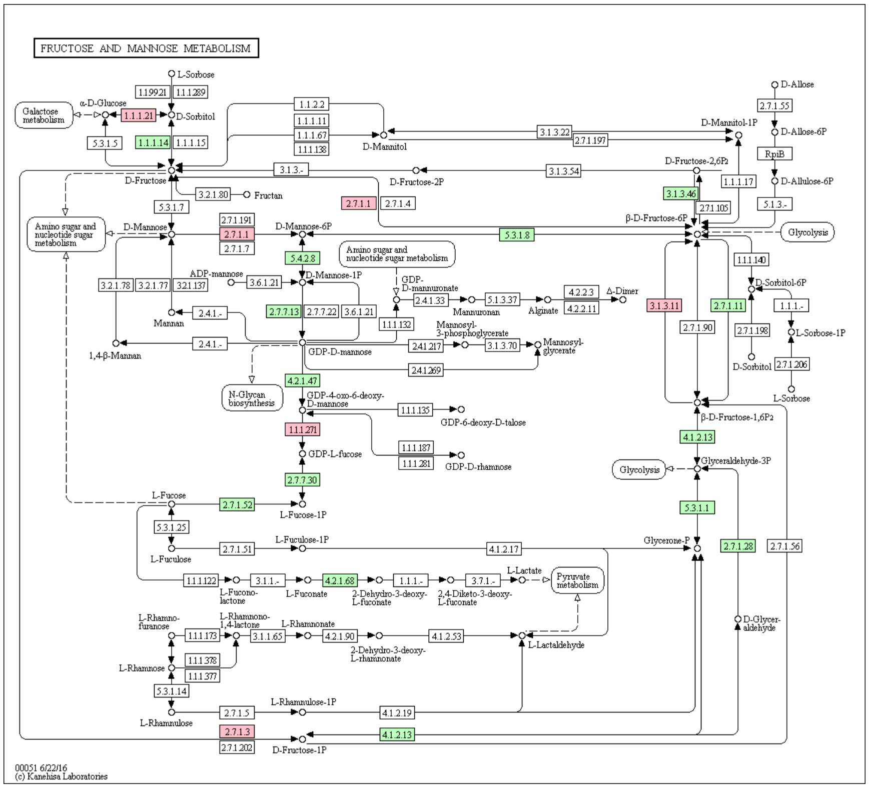

224.3 Legend:

RBH-Blast at 60% Identity + 50% Coverage
Green = Hit in *H. sapiens*
Red = Hit in *H. sapiens* and *C. milli*
White = Not in *H. sapiens*

225 Primary immunodeficiency

225.1 Human Pathway: HSA05340

225.2 Number of Hits: 5

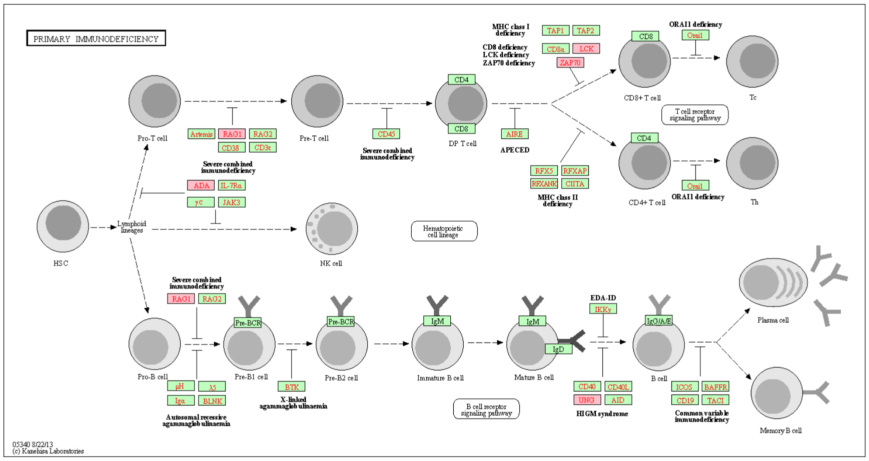

225.3 Legend:

RBH-Blast at 60% Identity + 50% Coverage
Green = Hit in *H. sapiens*
Red = Hit in *H. sapiens* and *C. milli*
White = Not in *H. sapiens*

226 Ascorbate and aldarate metabolism

226.1 Human Pathway: HSA00053

226.2 Number of Hits: 5

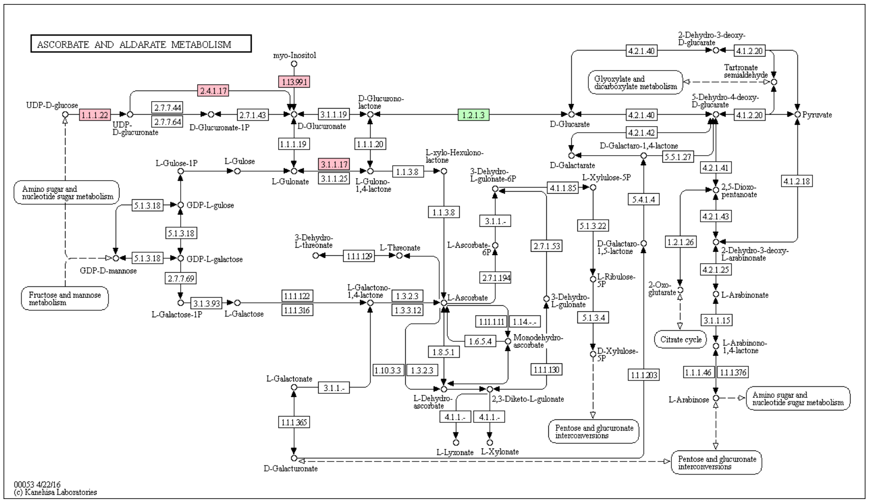

226.3 Legend:

RBH-Blast at 60% Identity + 50% Coverage
Green = Hit in *H. sapiens*
Red = Hit in *H. sapiens* and *C. milli*
White = Not in *H. sapiens*

227 Regulation of autophagy

227.1 Human Pathway: HSA04140

227.2 Number of Hits: 5

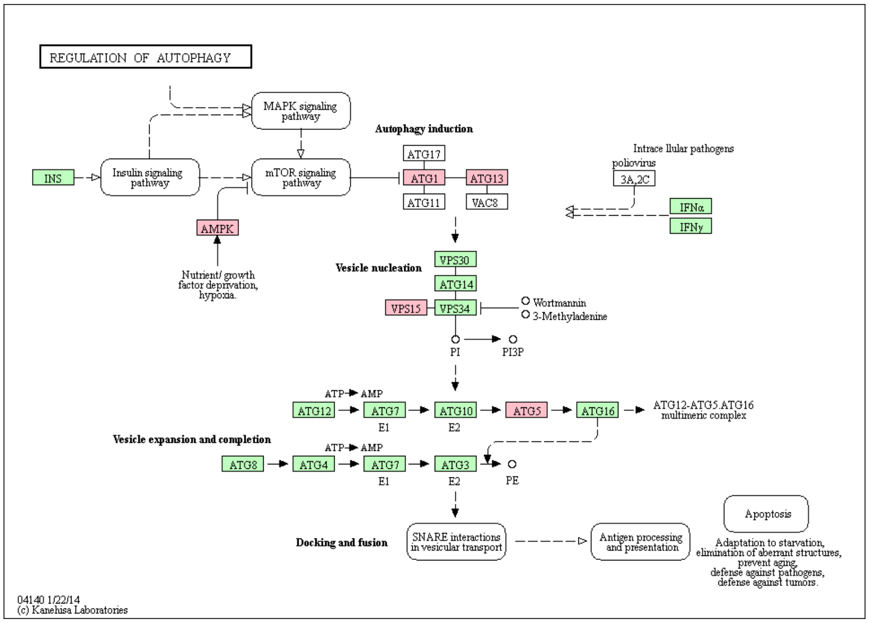

227.3 Legend:

RBH-Blast at 60% Identity + 50% Coverage
Green = Hit in *H. sapiens*
Red = Hit in *H. sapiens* and *C. milli*
White = Not in *H. sapiens*

228 Endocrine and other factor-regulated calcium reabsorption

228.1 Human Pathway: HSA04961

228.2 Number of Hits: 5

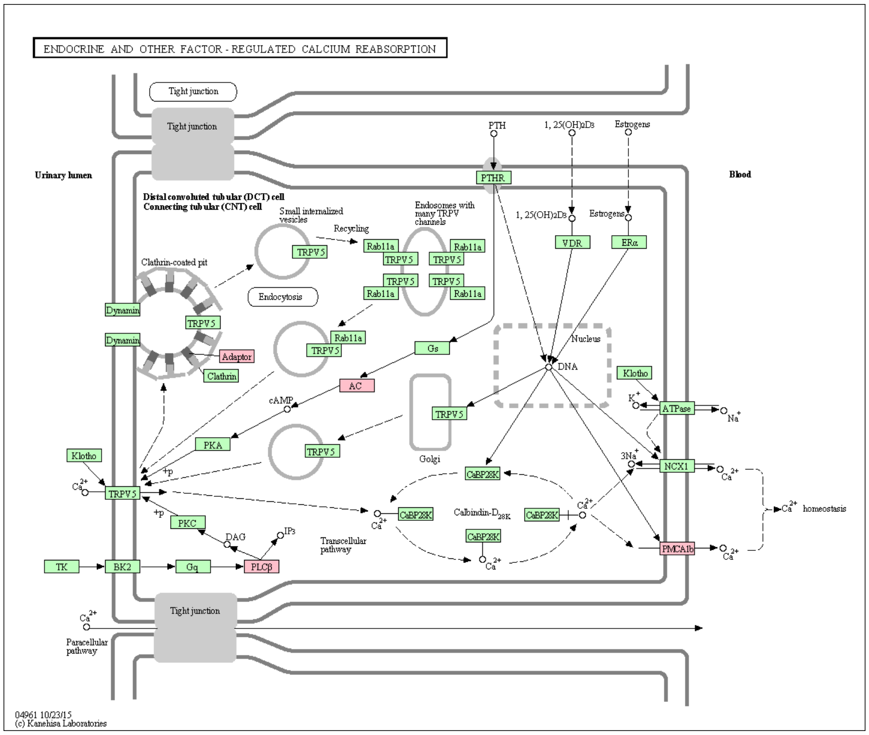

228.3 Legend:

RBH-Blast at 60% Identity + 50% Coverage
Green = Hit in *H. sapiens*
Red = Hit in *H. sapiens* and *C. milli*
White = Not in *H. sapiens*

229 Pentose phosphate pathway

229.1 Human Pathway: HSA00030

229.2 Number of Hits: 5

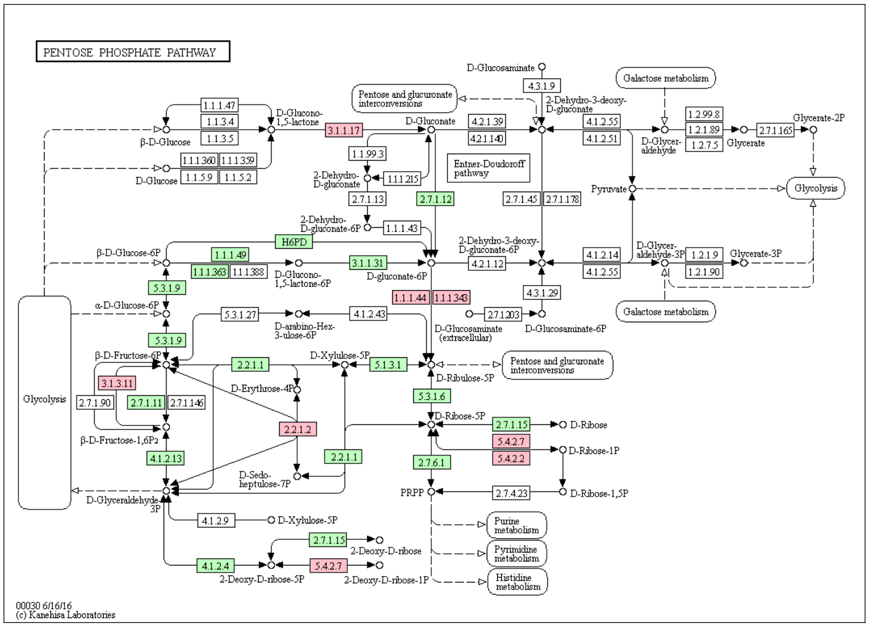

229.3 Legend:

RBH-Blast at 60% Identity + 50% Coverage
Green = Hit in *H. sapiens*
Red = Hit in *H. sapiens* and *C. milli*
White = Not in *H. sapiens*

230 Fatty acid biosynthesis

230.1 Human Pathway: HSA00061

230.2 Number of Hits: 5

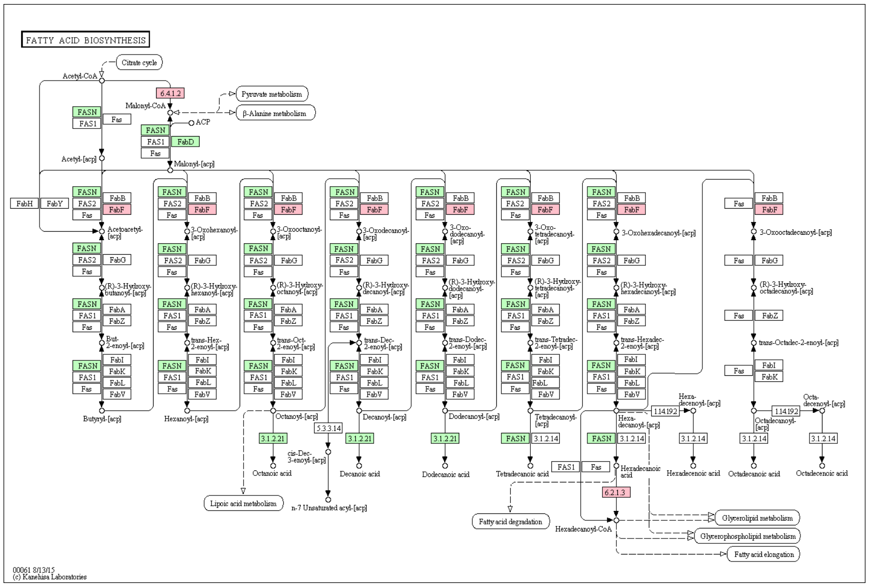

230.3 Legend:

RBH-Blast at 60% Identity + 50% Coverage
Green = Hit in *H. sapiens*
Red = Hit in *H. sapiens* and *C. milli*
White = Not in *H. sapiens*

231 Systemic lupus erythematosus

231.1 Human Pathway: HSA05322

231.2 Number of Hits: 5

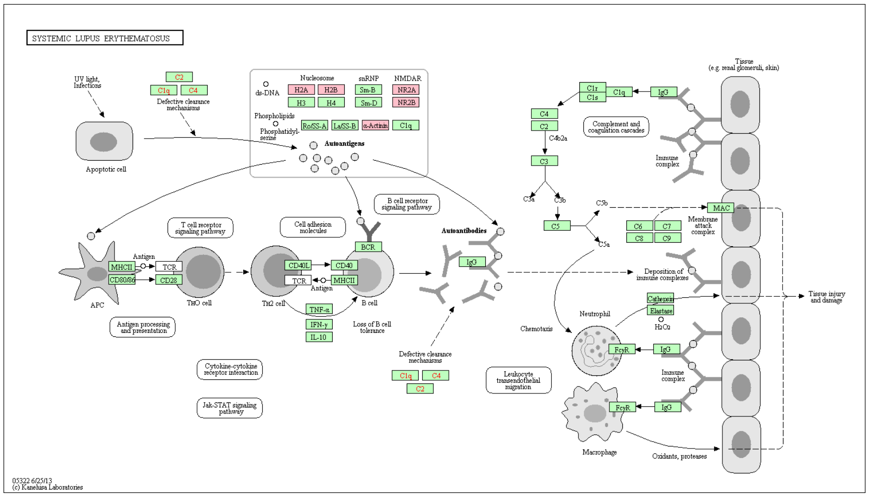

231.3 Legend:

RBH-Blast at 60% Identity + 50% Coverage
Green = Hit in *H. sapiens*
Red = Hit in *H. sapiens* and *C. milli*
White = Not in *H. sapiens*

232 Biosynthesis of unsaturated fatty acids

232.1 Human Pathway: HSA01040

232.2 Number of Hits: 5

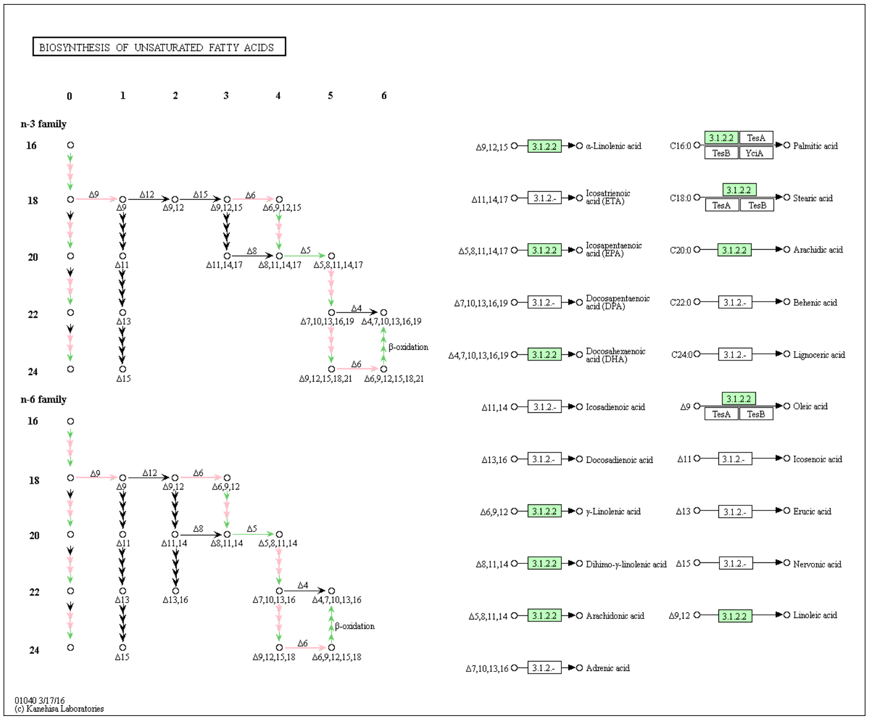

232.3 Legend:

RBH-Blast at 60% Identity + 50% Coverage
Green = Hit in *H. sapiens*
Red = Hit in *H. sapiens* and *C. milli*
White = Not in *H. sapiens*

233 Propanoate metabolism

233.1 Human Pathway: HSA00640

233.2 Number of Hits: 5

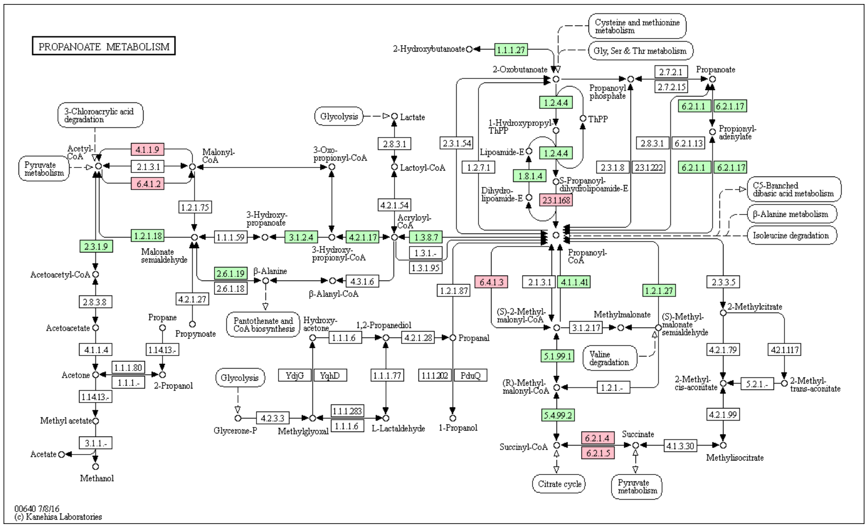

233.3 Legend:

RBH-Blast at 60% Identity + 50% Coverage
Green = Hit in *H. sapiens*
Red = Hit in *H. sapiens* and *C. milli*
White = Not in *H. sapiens*

234 Collecting duct acid secretion

234.1 Human Pathway: HSA04966

234.2 Number of Hits: 5

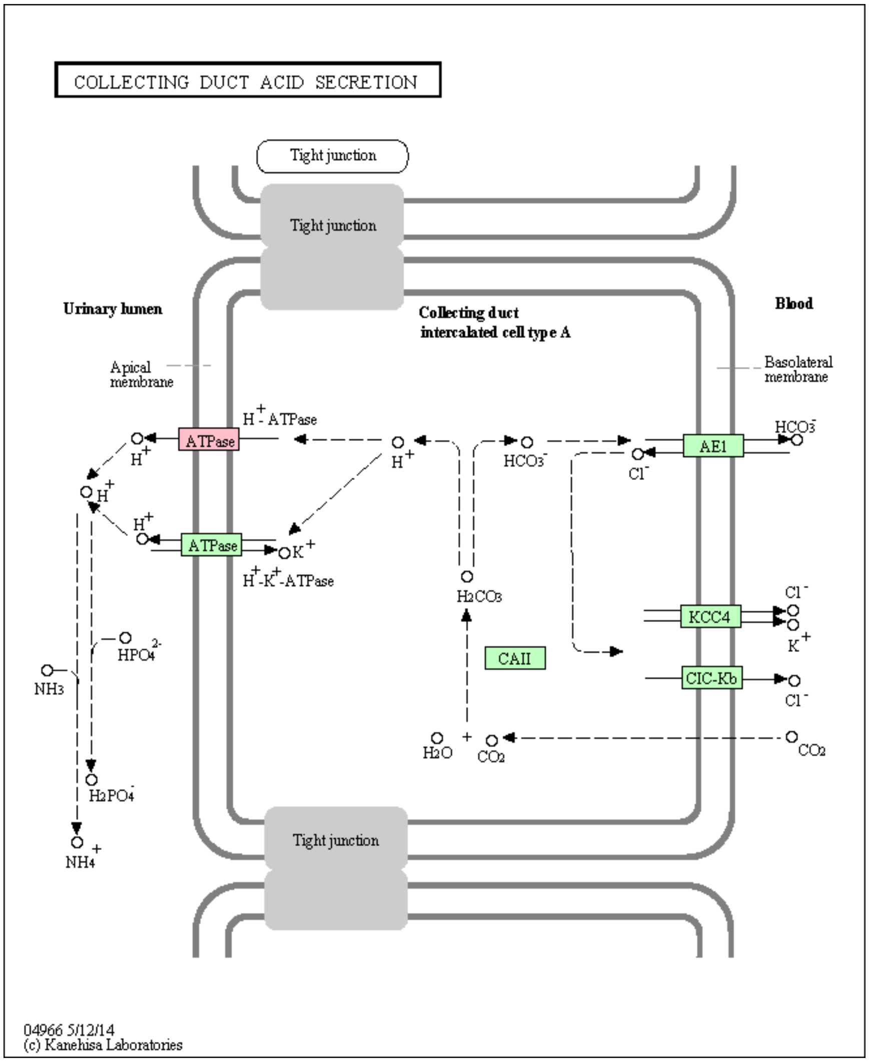

234.3 Legend:

RBH-Blast at 60% Identity + 50% Coverage
Green = Hit in *H. sapiens*
Red = Hit in *H. sapiens* and *C. milli*
White = Not in *H. sapiens*

---

235 Thyroid cancer

235.1 Human Pathway: HSA05216

235.2 Number of Hits: 5

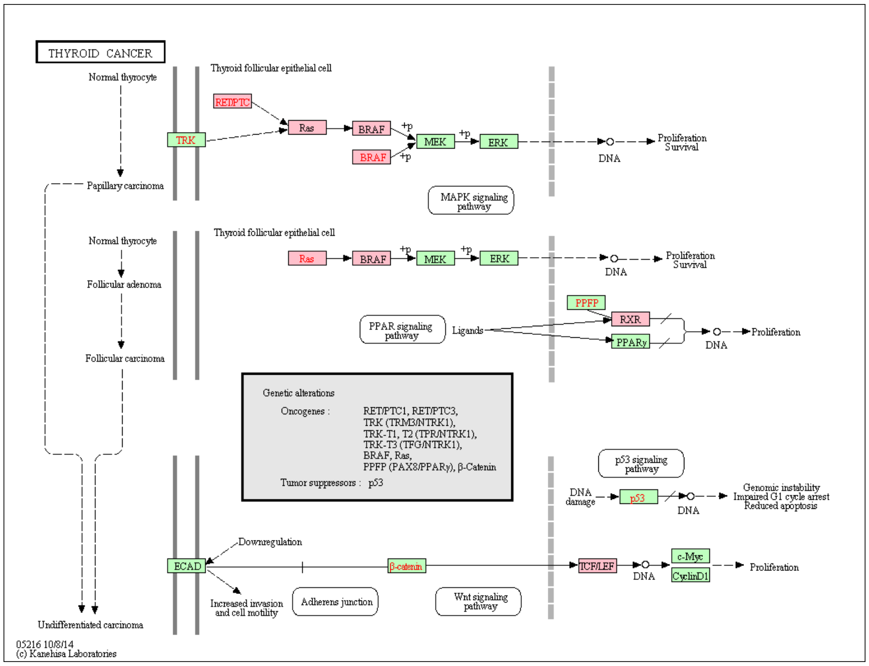

235.3 Legend:

RBH-Blast at 60% Identity + 50% Coverage
Green = Hit in *H. sapiens*
Red = Hit in *H. sapiens* and *C. milli*
White = Not in *H. sapiens*

---

236 Glycosphingolipid biosynthesis - ganglio series

236.1 Human Pathway: HSA00604

236.2 Number of Hits: 5

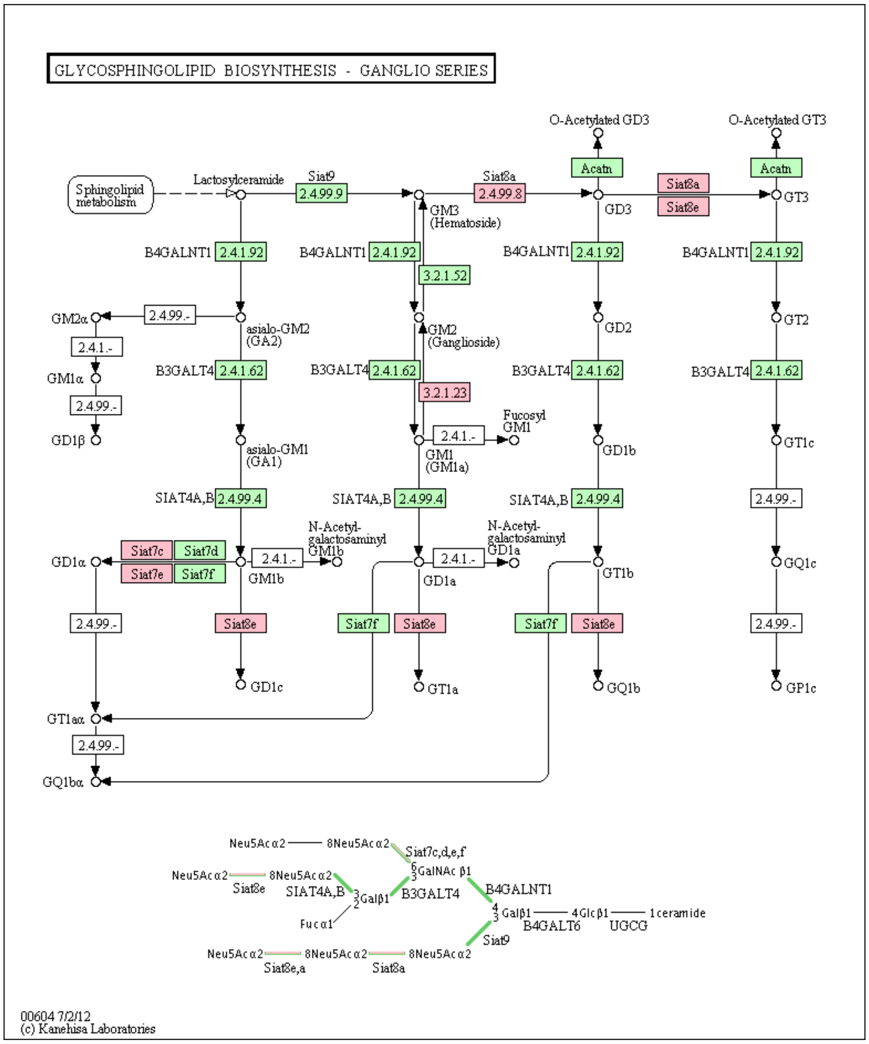

236.3 Legend:

RBH-Blast at 60% Identity + 50% Coverage
Green = Hit in *H. sapiens*
Red = Hit in *H. sapiens* and *C. milli*
White = Not in *H. sapiens*

---

237 Ether lipid metabolism

237.1 Human Pathway: HSA00565

237.2 Number of Hits: 5

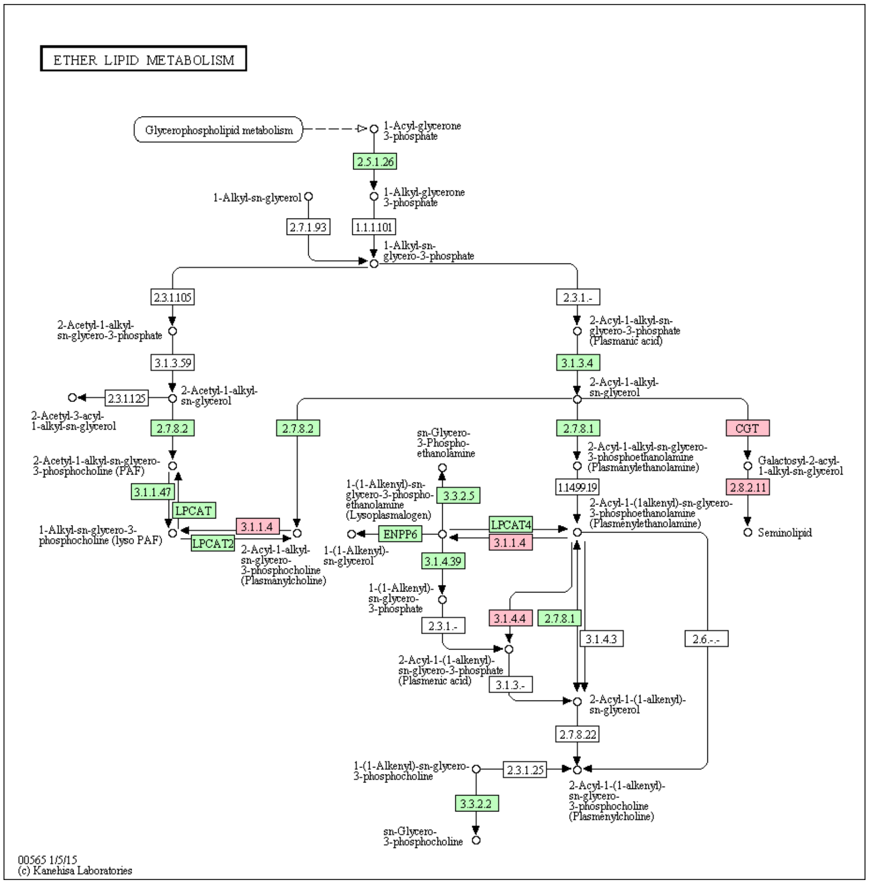

237.3 Legend:

RBH-Blast at 60% Identity + 50% Coverage
Green = Hit in *H. sapiens*
Red = Hit in *H. sapiens* and *C. milli*
White = Not in *H. sapiens*

238 Arginine biosynthesis

238.1 Human Pathway: HSA00220

238.2 Number of Hits: 4

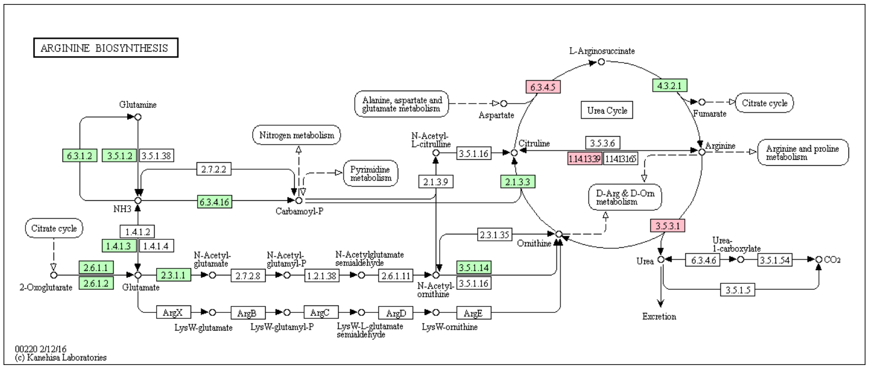

238.3 Legend:

RBH-Blast at 60% Identity + 50% Coverage
Green = Hit in *H. sapiens*
Red = Hit in *H. sapiens* and *C. milli*
White = Not in *H. sapiens*

239 Legionellosis

239.1 Human Pathway: HSA05134

239.2 Number of Hits: 4

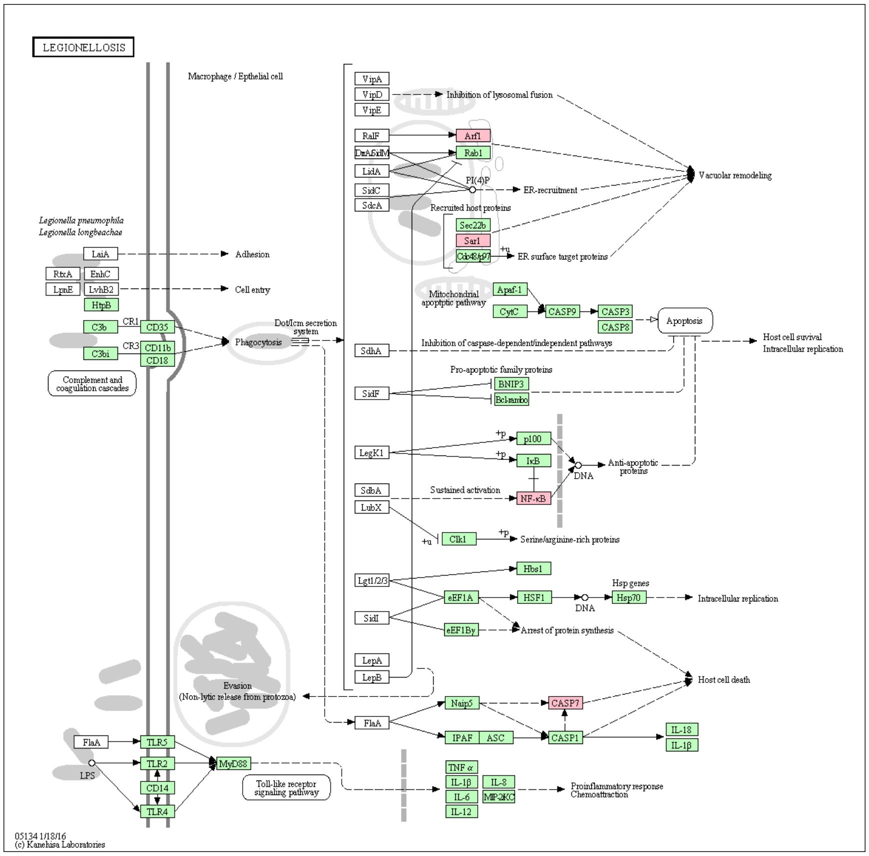

239.3 Legend:

RBH-Blast at 60% Identity + 50% Coverage
Green = Hit in *H. sapiens*
Red = Hit in *H. sapiens* and *C. milli*
White = Not in *H. sapiens*

240 Glycosphingolipid biosynthesis - lacto and neolacto series

240.1 Human Pathway: HSA00601

240.2 Number of Hits: 4

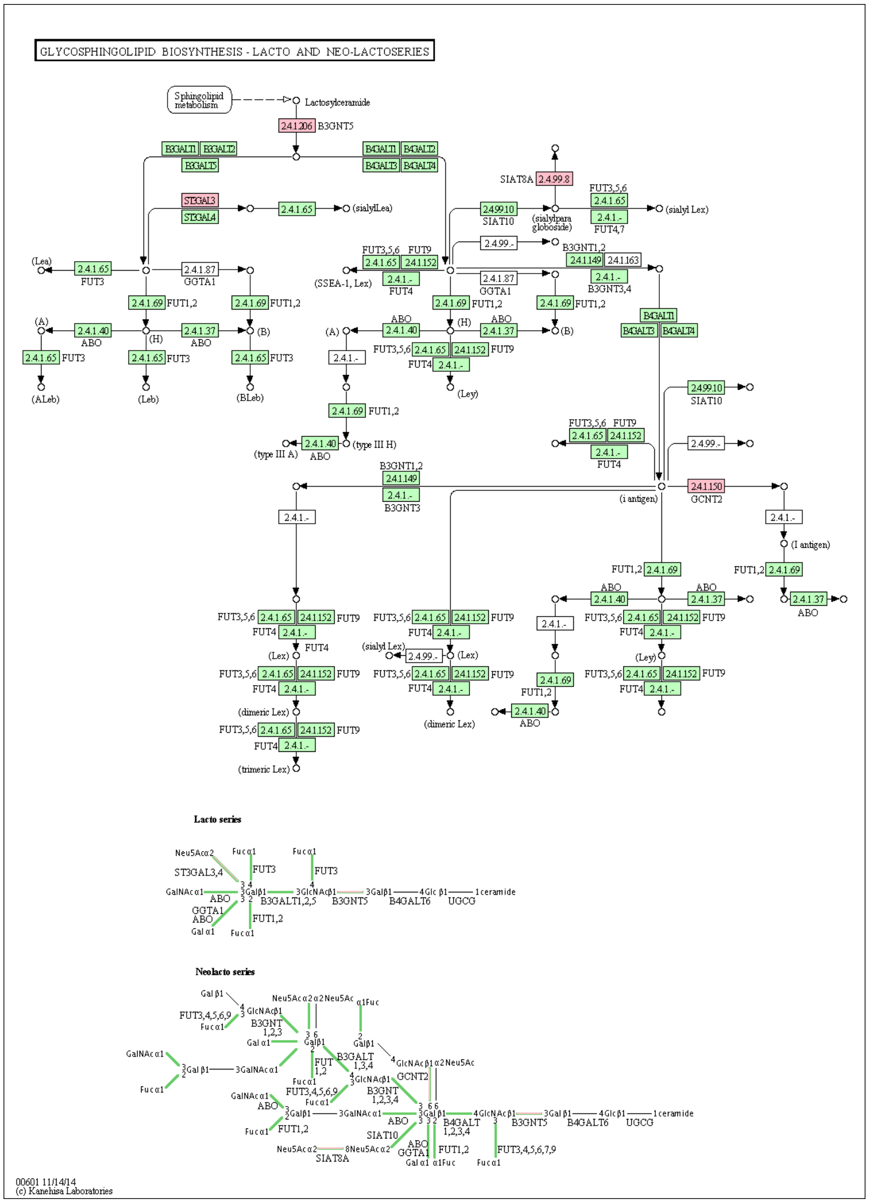

240.3 Legend:

RBH-Blast at 60% Identity + 50% Coverage
Green = Hit in *H. sapiens*
Red = Hit in *H. sapiens* and *C. milli*
White = Not in *H. sapiens*

---

241 Citrate cycle (TCA cycle)

241.1 Human Pathway: HSA00020

241.2 Number of Hits: 4

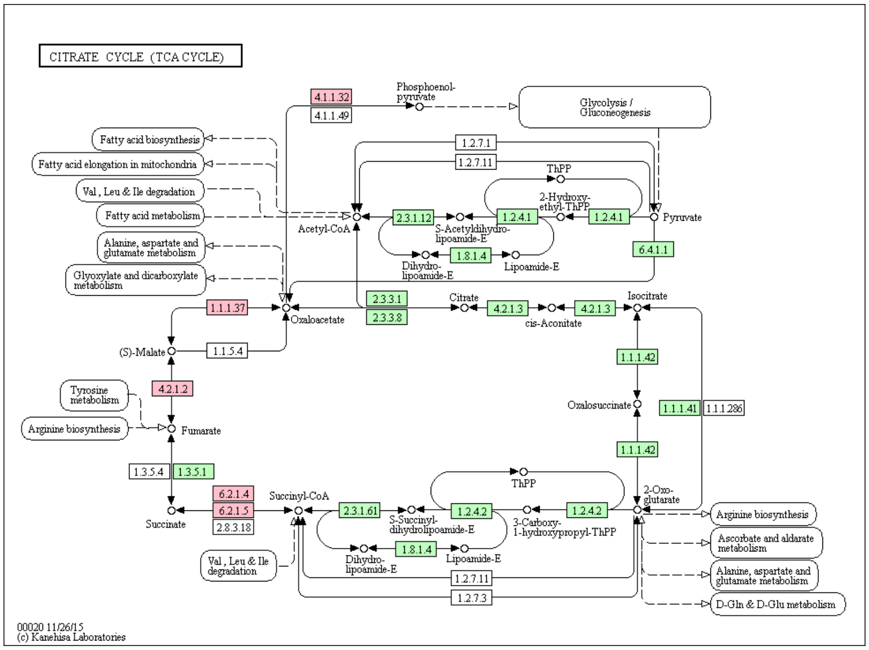

241.3 Legend:

RBH-Blast at 60% Identity + 50% Coverage
Green = Hit in *H. sapiens*
Red = Hit in *H. sapiens* and *C. milli*
White = Not in *H. sapiens*

---

242 Pantothenate and CoA biosynthesis

242.1 Human Pathway: HSA00770

242.2 Number of Hits: 4

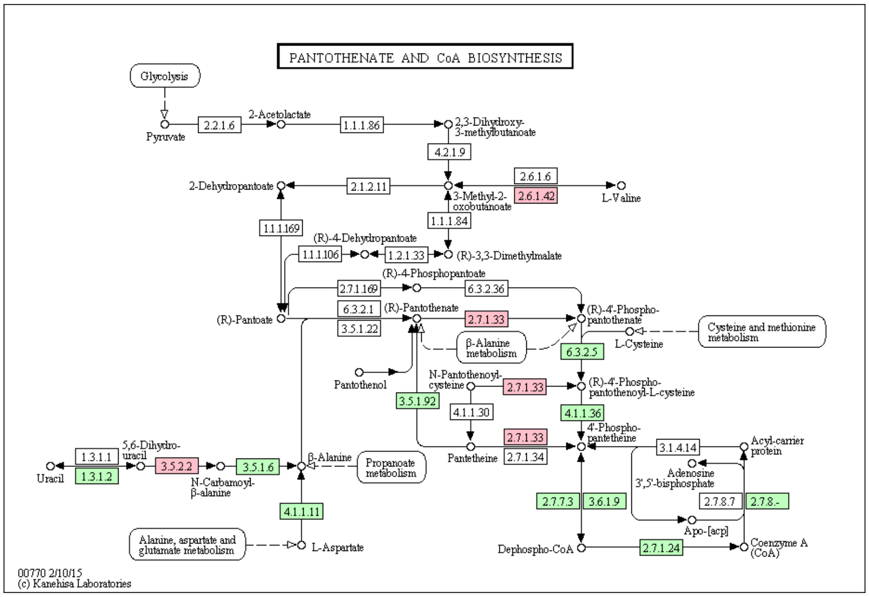

242.3 Legend:

RBH-Blast at 60% Identity + 50% Coverage
Green = Hit in *H. sapiens*
Red = Hit in *H. sapiens* and *C. milli*
White = Not in *H. sapiens*

243 Pentose and glucuronate interconversions

243.1 Human Pathway: HSA00040

243.2 Number of Hits: 4

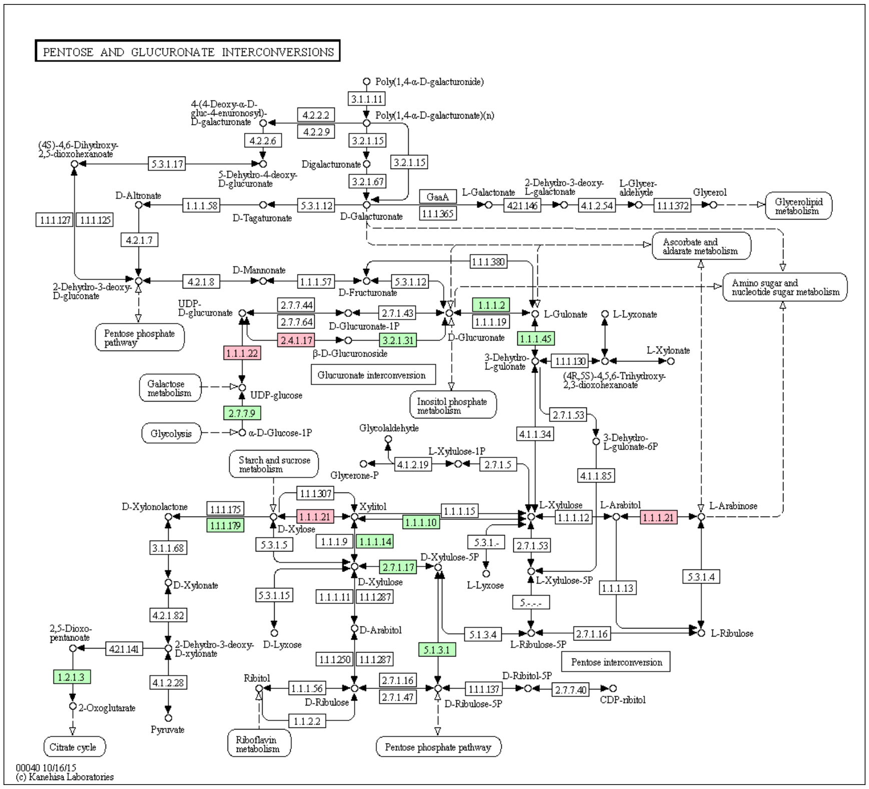

243.3 Legend:

RBH-Blast at 60% Identity + 50% Coverage
Green = Hit in *H. sapiens*
Red = Hit in *H. sapiens* and *C. milli*
White = Not in *H. sapiens*

244 Other types of O-glycan biosynthesis

244.1 Human Pathway: HSA00514

244.2 Number of Hits: 4

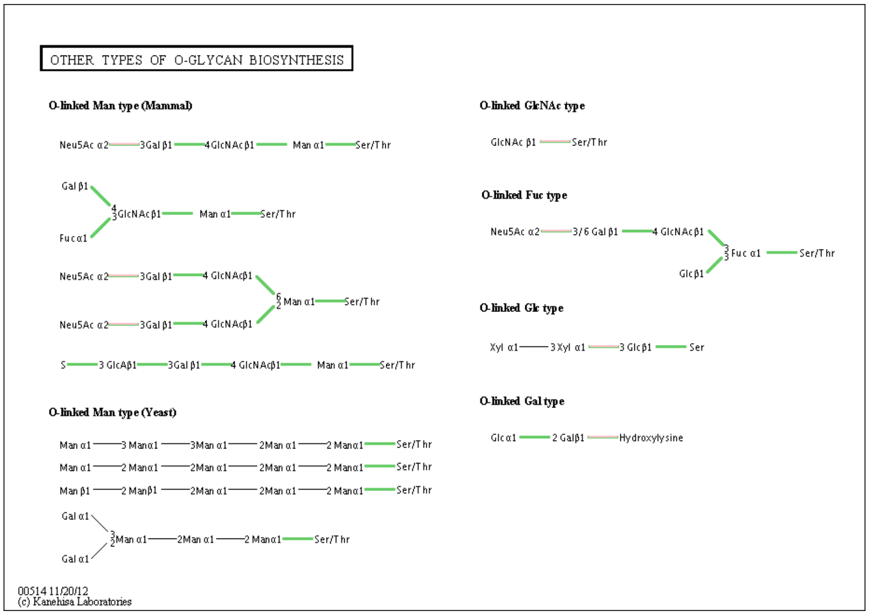

244.3 Legend:

RBH-Blast at 60% Identity + 50% Coverage
Green = Hit in *H. sapiens*
Red = Hit in *H. sapiens* and *C. milli*
White = Not in *H. sapiens*

245 Vitamin digestion and absorption

245.1 Human Pathway: HSA04977

245.2 Number of Hits: 4

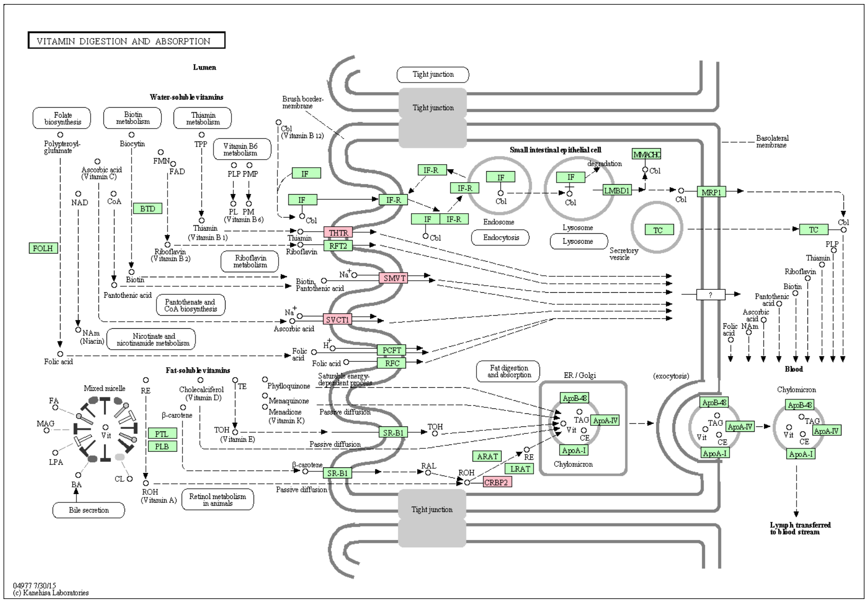

245.3 Legend:

RBH-Blast at 60% Identity + 50% Coverage
Green = Hit in *H. sapiens*
Red = Hit in *H. sapiens* and *C. milli*
White = Not in *H. sapiens*

246 Porphyrin and chlorophyll metabolism

246.1 Human Pathway: HSA00860

246.2 Number of Hits: 4

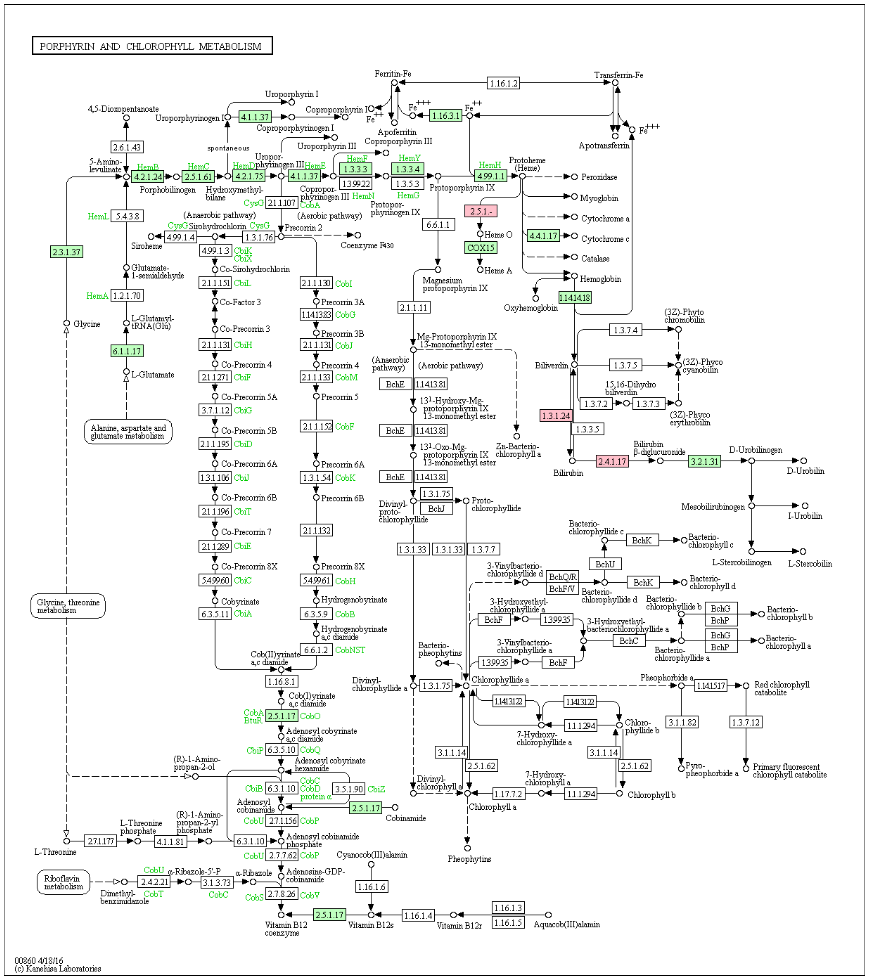

246.3 Legend:

RBH-Blast at 60% Identity + 50% Coverage
Green = Hit in *H. sapiens*
Red = Hit in *H. sapiens* and *C. milli*
White = Not in *H. sapiens*

247 SNARE interactions in vesicular transport

247.1 Human Pathway: HSA04130

247.2 Number of Hits: 4

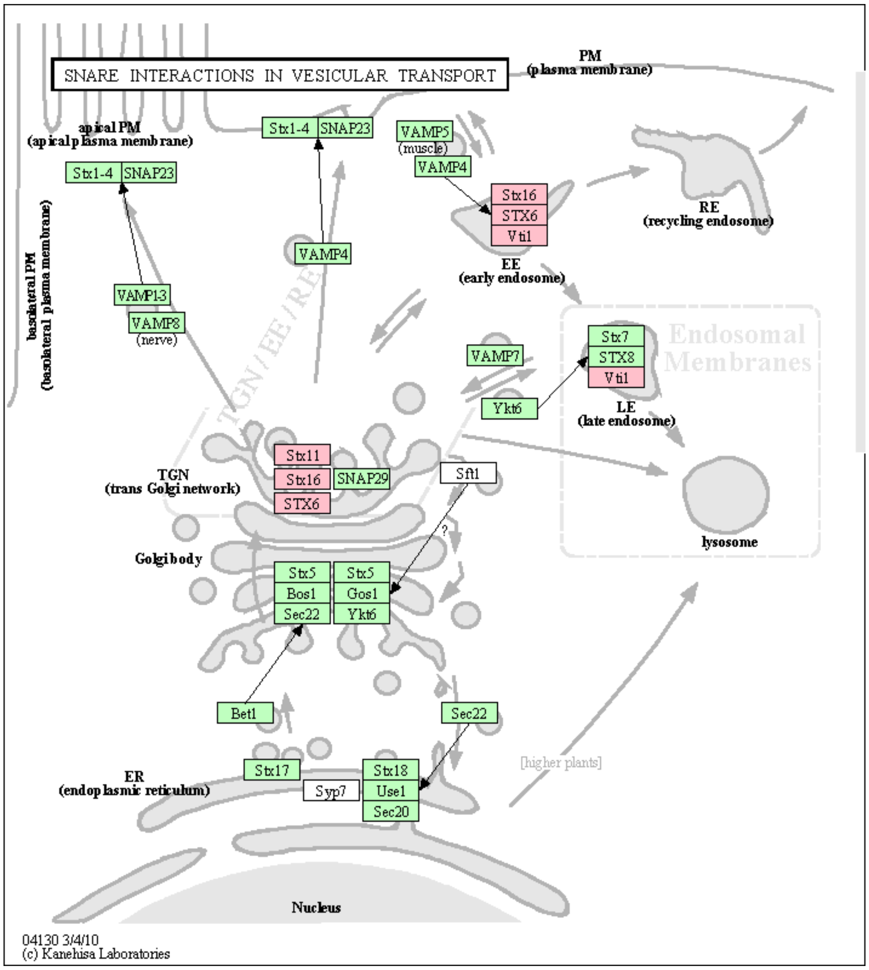

247.3 Legend:

RBH-Blast at 60% Identity + 50% Coverage
Green = Hit in *H. sapiens*
Red = Hit in *H. sapiens* and *C. milli*
White = Not in *H. sapiens*

248 Homologous recombination

248.1 Human Pathway: HSA03440

248.2 Number of Hits: 4

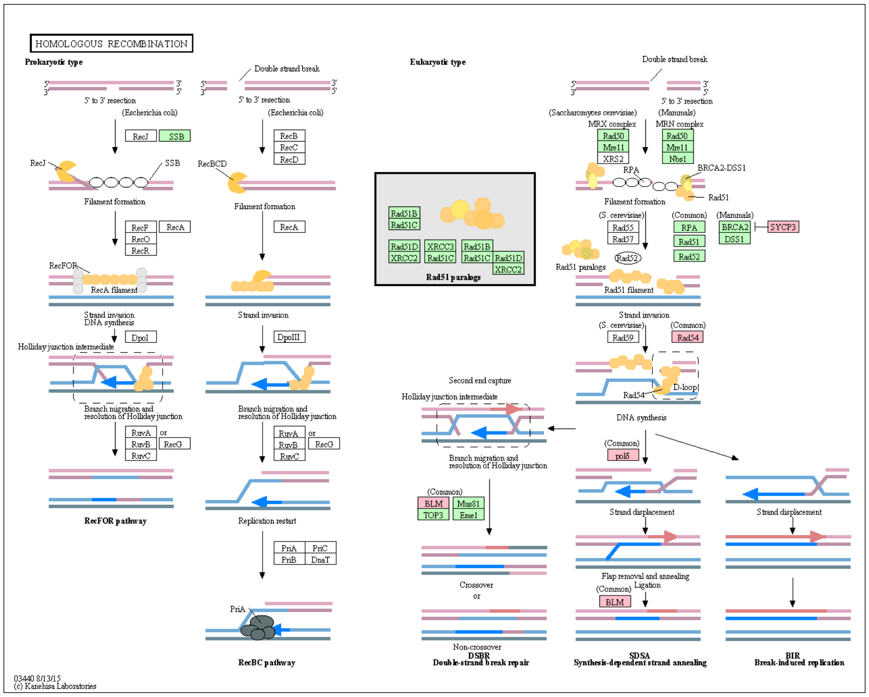

248.3 Legend:

RBH-Blast at 60% Identity + 50% Coverage
Green = Hit in *H. sapiens*
Red = Hit in *H. sapiens* and *C. milli*
White = Not in *H. sapiens*

249 Proteasome

249.1 Human Pathway: HSA03050

249.2 Number of Hits: 4

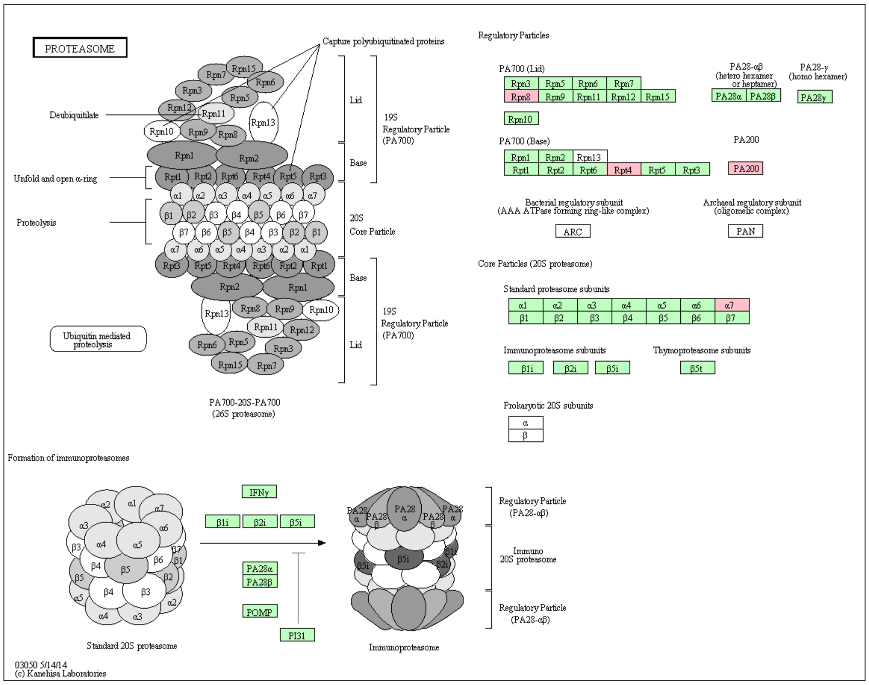

249.3 Legend:

RBH-Blast at 60% Identity + 50% Coverage
Green = Hit in *H. sapiens*
Red = Hit in *H. sapiens* and *C. milli*
White = Not in *H. sapiens*

250 Apoptosis - multiple species

250.1 Human Pathway: HSA04215

250.2 Number of Hits: 4

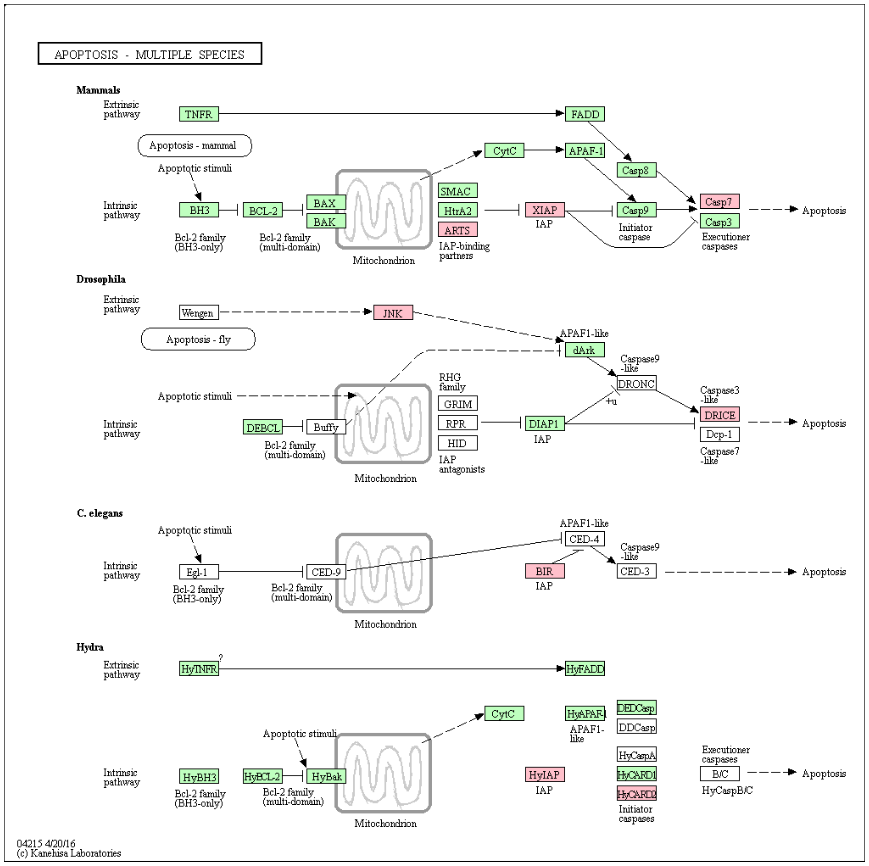

250.3 Legend:

RBH-Blast at 60% Identity + 50% Coverage
Green = Hit in *H. sapiens*
Red = Hit in *H. sapiens* and *C. milli*
White = Not in *H. sapiens*

251 Glycosaminoglycan degradation

251.1 Human Pathway: HSA00531

251.2 Number of Hits: 3

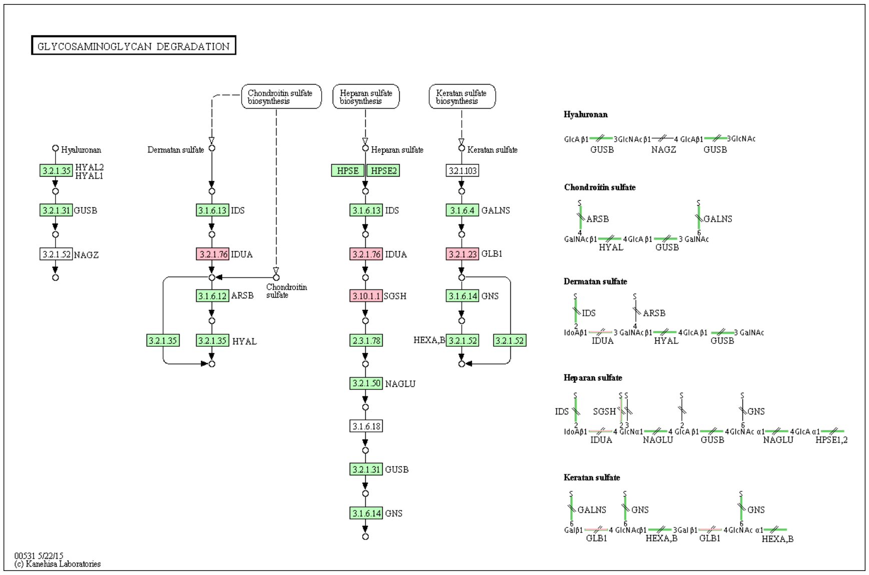

251.3 Legend:

RBH-Blast at 60% Identity + 50% Coverage
Green = Hit in *H. sapiens*
Red = Hit in *H. sapiens* and *C. milli*
White = Not in *H. sapiens*

252 RNA polymerase

252.1 Human Pathway: HSA03020

252.2 Number of Hits: 3

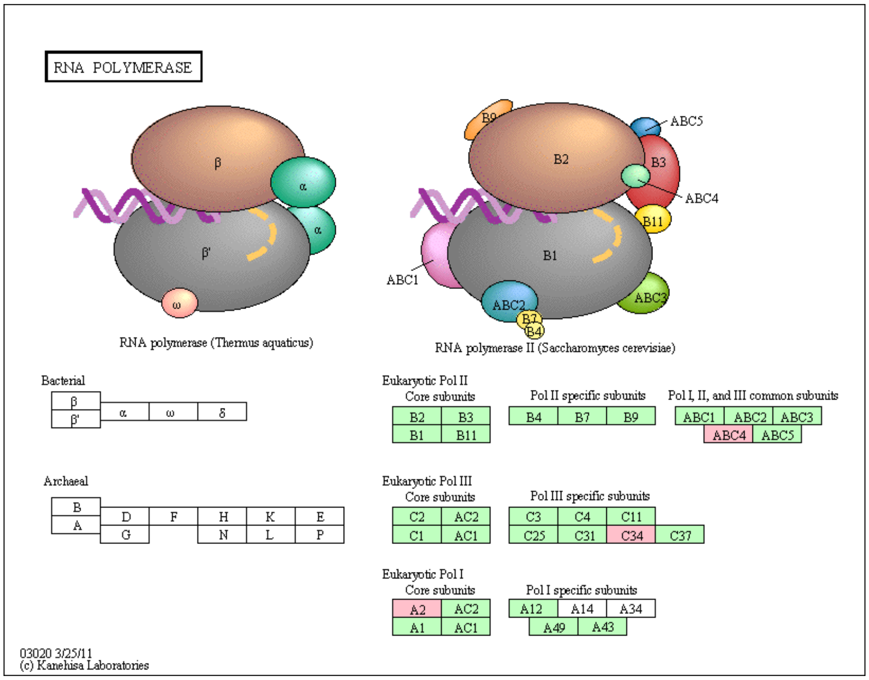

252.3 Legend:

RBH-Blast at 60% Identity + 50% Coverage
Green = Hit in *H. sapiens*
Red = Hit in *H. sapiens* and *C. milli*
White = Not in *H. sapiens*

253 Protein export

253.1 Human Pathway: HSA03060

253.2 Number of Hits: 3

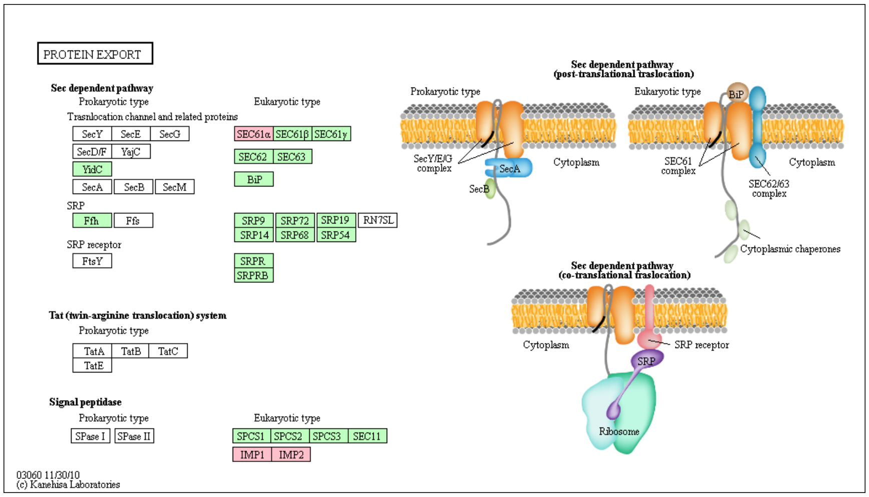

253.3 Legend:

RBH-Blast at 60% Identity + 50% Coverage
Green = Hit in *H. sapiens*
Red = Hit in *H. sapiens* and *C. milli*
White = Not in *H. sapiens*

254 Terpenoid backbone biosynthesis

254.1 Human Pathway: HSA00900

254.2 Number of Hits: 3

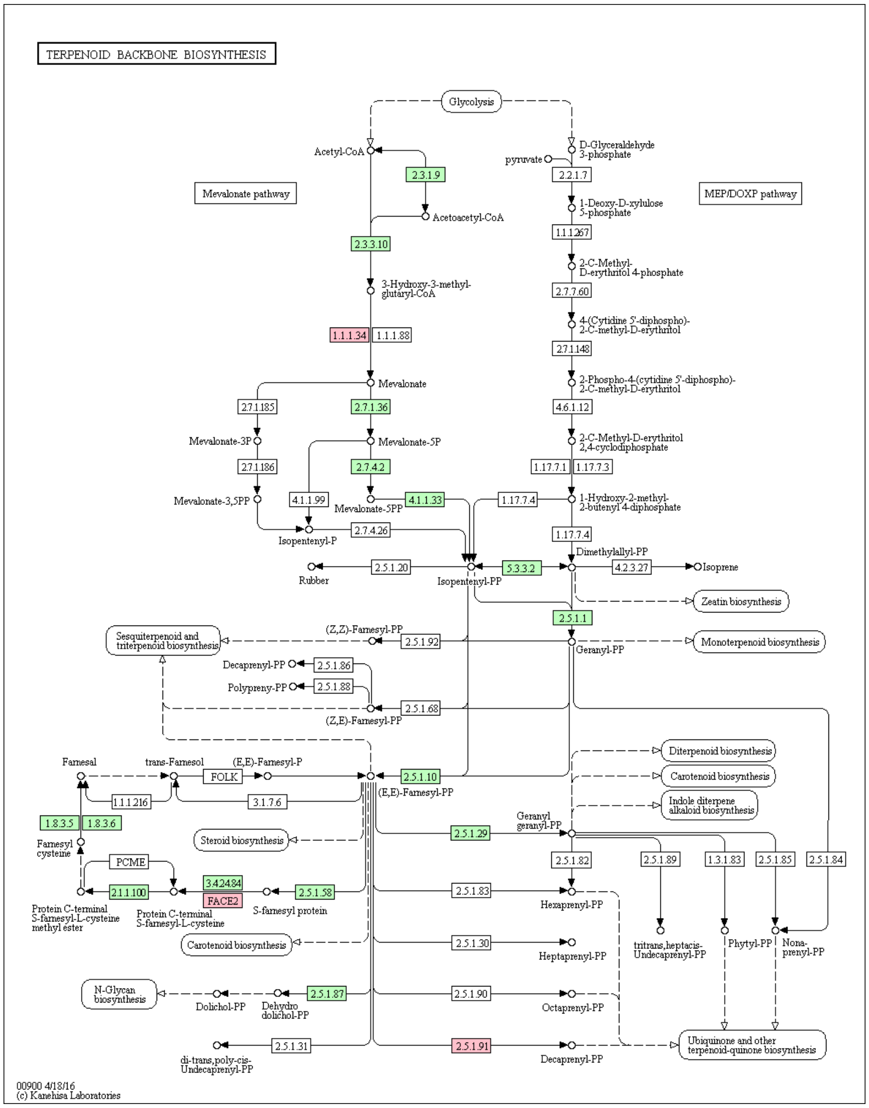

254.3 Legend:

RBH-Blast at 60% Identity + 50% Coverage
Green = Hit in *H. sapiens*
Red = Hit in *H. sapiens* and *C. milli*
White = Not in *H. sapiens*

---

255 Non-homologous end-joining

255.1 Human Pathway: HSA03450

255.2 Number of Hits: 3

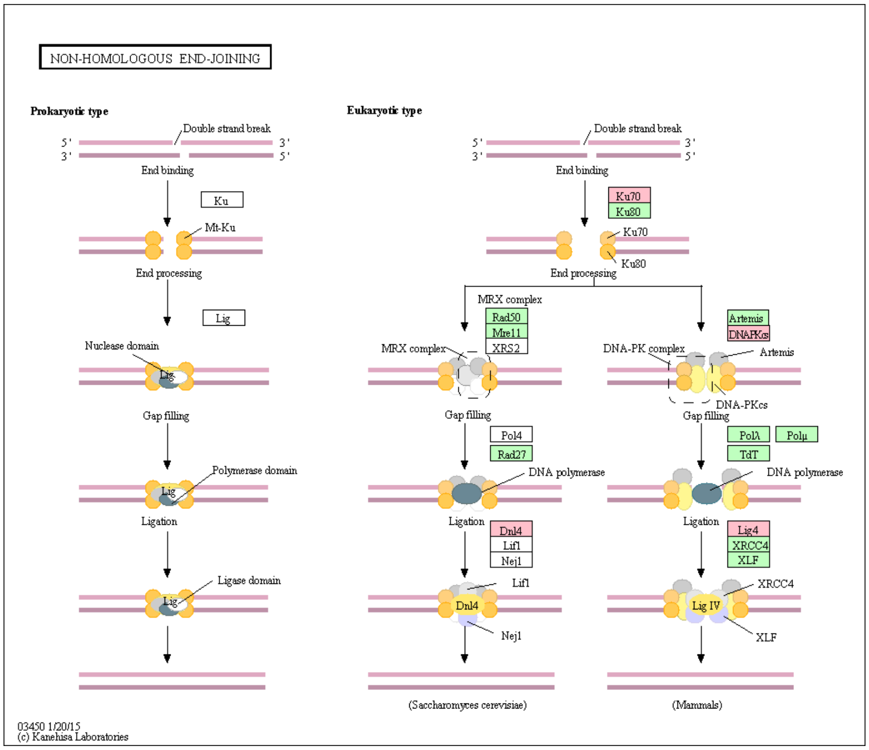

255.3 Legend:

RBH-Blast at 60% Identity + 50% Coverage
Green = Hit in *H. sapiens*
Red = Hit in *H. sapiens* and *C. milli*
White = Not in *H. sapiens*

256 Viral myocarditis

256.1 Human Pathway: HSA05416

256.2 Number of Hits: 3

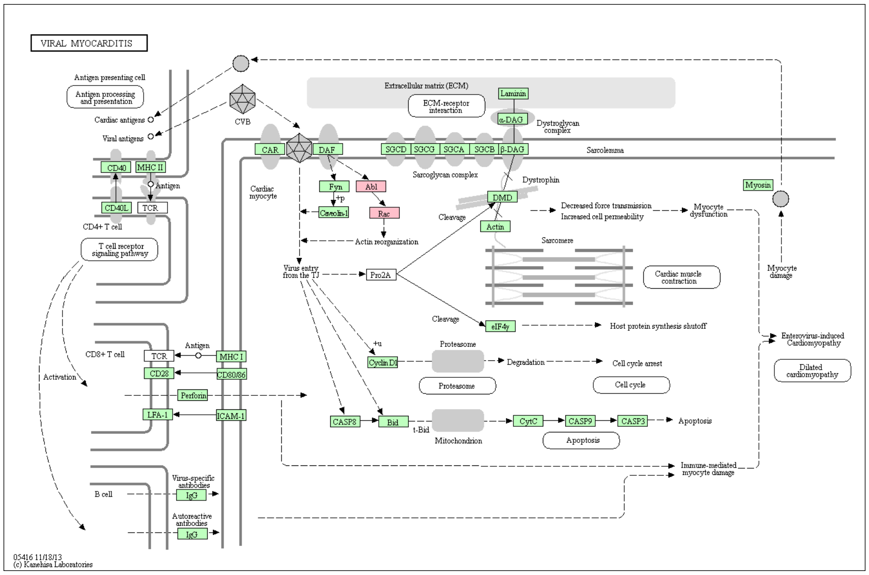

256.3 Legend:

RBH-Blast at 60% Identity + 50% Coverage
Green = Hit in *H. sapiens*
Red = Hit in *H. sapiens* and *C. milli*
White = Not in *H. sapiens*

257 Mismatch repair

257.1 Human Pathway: HSA03430

257.2 Number of Hits: 3

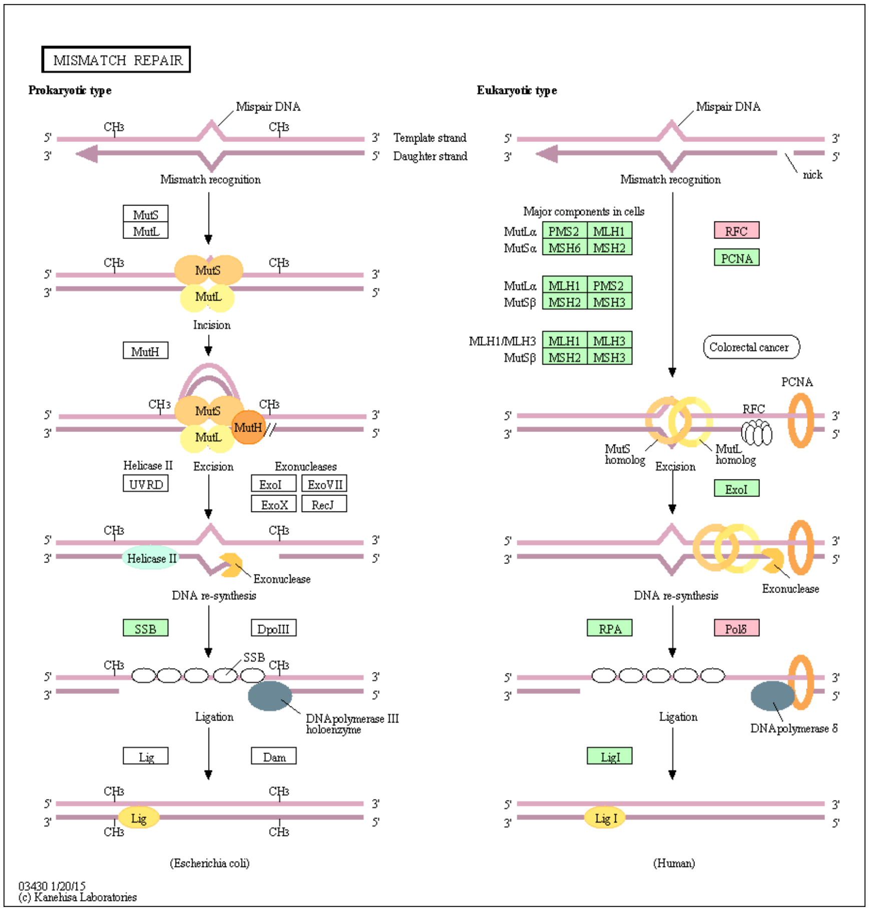

257.3 Legend:

RBH-Blast at 60% Identity + 50% Coverage
Green = Hit in *H. sapiens*
Red = Hit in *H. sapiens* and *C. milli*
White = Not in *H. sapiens*

258 Butanoate metabolism

258.1 Human Pathway: HSA00650

258.2 Number of Hits: 3

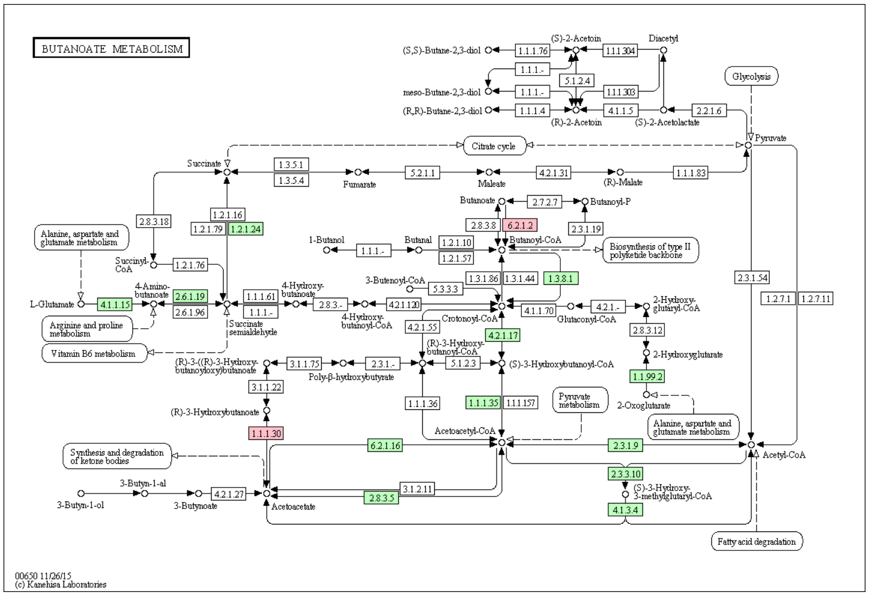

258.3 Legend:

RBH-Blast at 60% Identity + 50% Coverage
Green = Hit in *H. sapiens*
Red = Hit in *H. sapiens* and *C. milli*
White = Not in *H. sapiens*

259 Antigen processing and presentation

259.1 Human Pathway: HSA04612

259.2 Number of Hits: 3

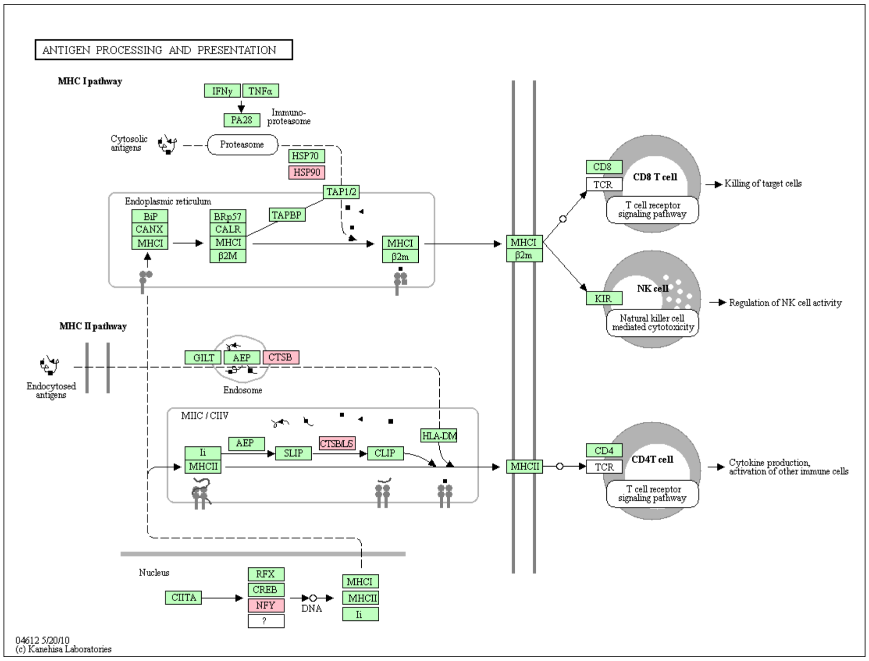

259.3 Legend:

RBH-Blast at 60% Identity + 50% Coverage
Green = Hit in *H. sapiens*
Red = Hit in *H. sapiens* and *C. milli*
White = Not in *H. sapiens*

260 Proximal tubule bicarbonate reclamation

260.1 Human Pathway: HSA04964

260.2 Number of Hits: 3

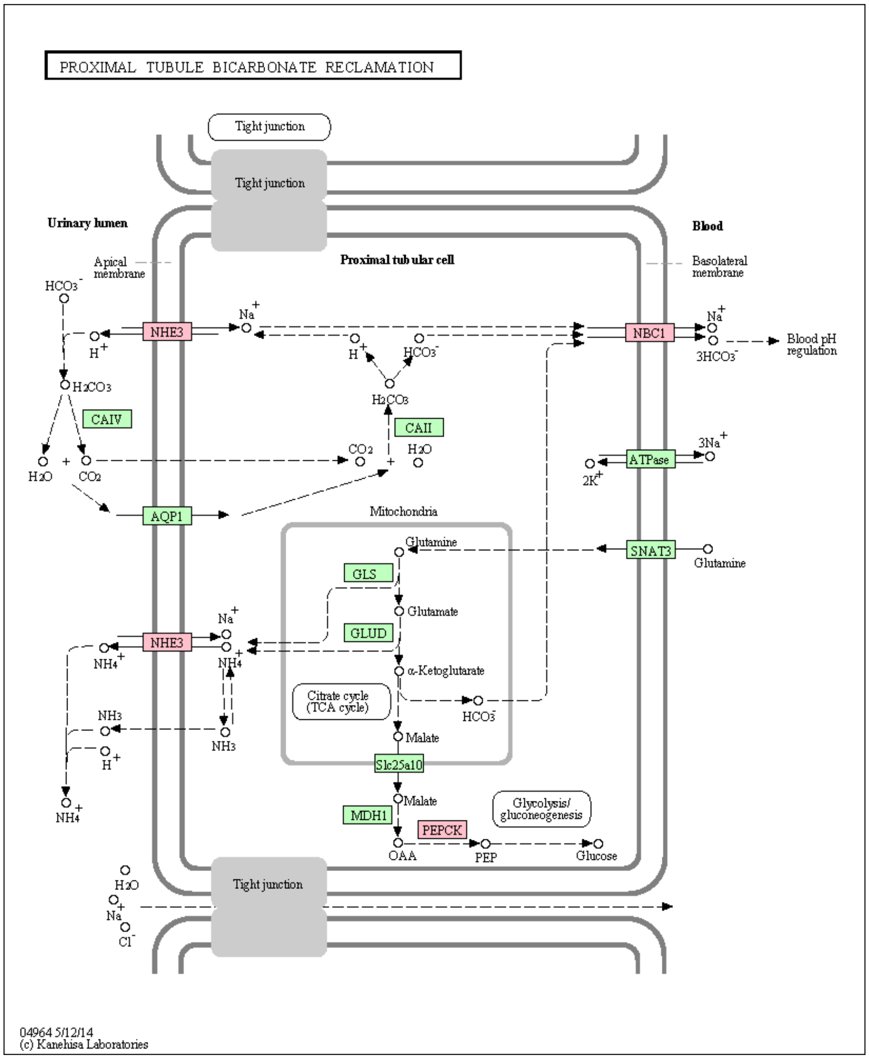

260.3 Legend:

RBH-Blast at 60% Identity + 50% Coverage
Green = Hit in *H. sapiens*
Red = Hit in *H. sapiens* and *C. milli*
White = Not in *H. sapiens*

---

261 African trypanosomiasis

261.1 Human Pathway: HSA05143

261.2 Number of Hits: 3

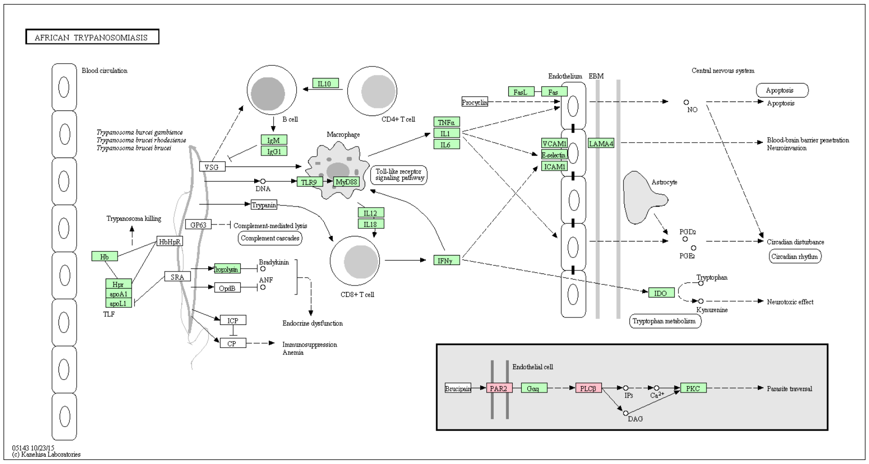

261.3 Legend:

RBH-Blast at 60% Identity + 50% Coverage
Green = Hit in *H. sapiens*
Red = Hit in *H. sapiens* and *C. milli*
White = Not in *H. sapiens*

---

262 Cytosolic DNA-sensing pathway

262.1 Human Pathway: HSA04623

262.2 Number of Hits: 3

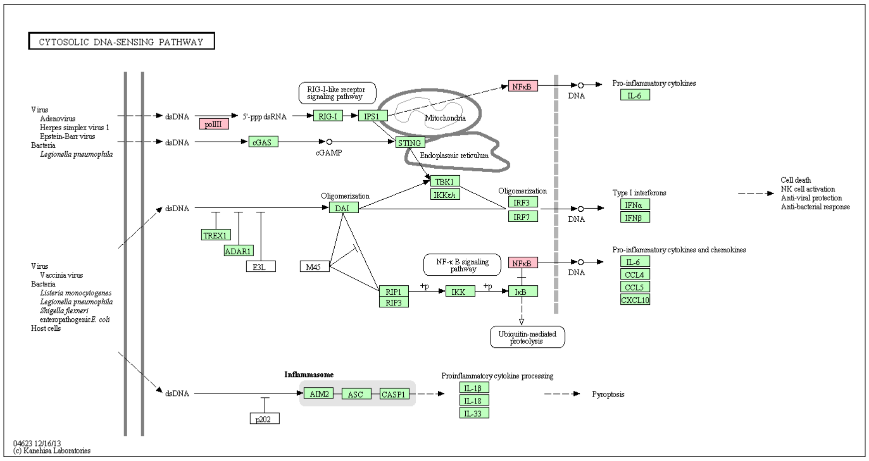

262.3 Legend:

RBH-Blast at 60% Identity + 50% Coverage
Green = Hit in *H. sapiens*
Red = Hit in *H. sapiens* and *C. milli*
White = Not in *H. sapiens*

263 Other glycan degradation

263.1 Human Pathway: HSA00511

263.2 Number of Hits: 3

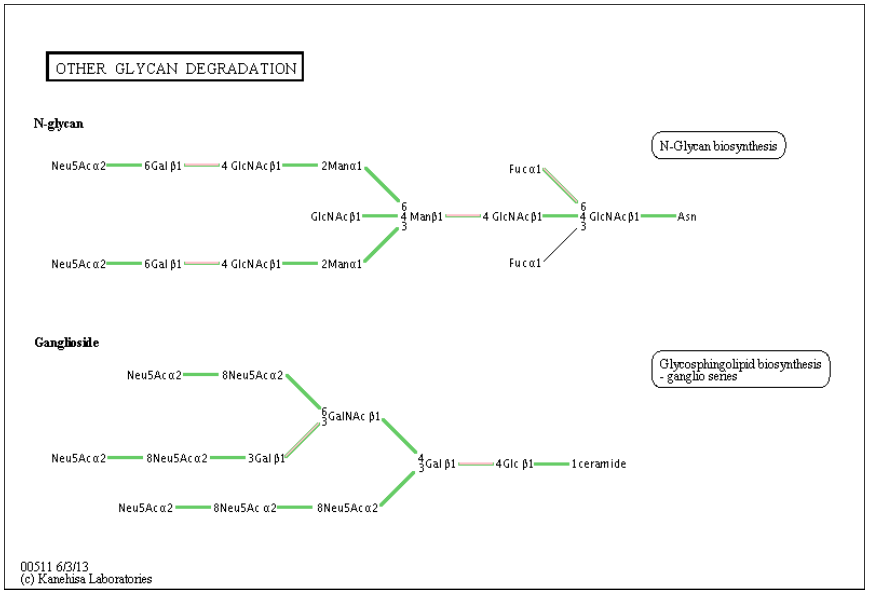

263.3 Legend:

RBH-Blast at 60% Identity + 50% Coverage
Green = Hit in *H. sapiens*
Red = Hit in *H. sapiens* and *C. milli*
White = Not in *H. sapiens*

264 Renin-angiotensin system

264.1 Human Pathway: HSA04614

264.2 Number of Hits: 2

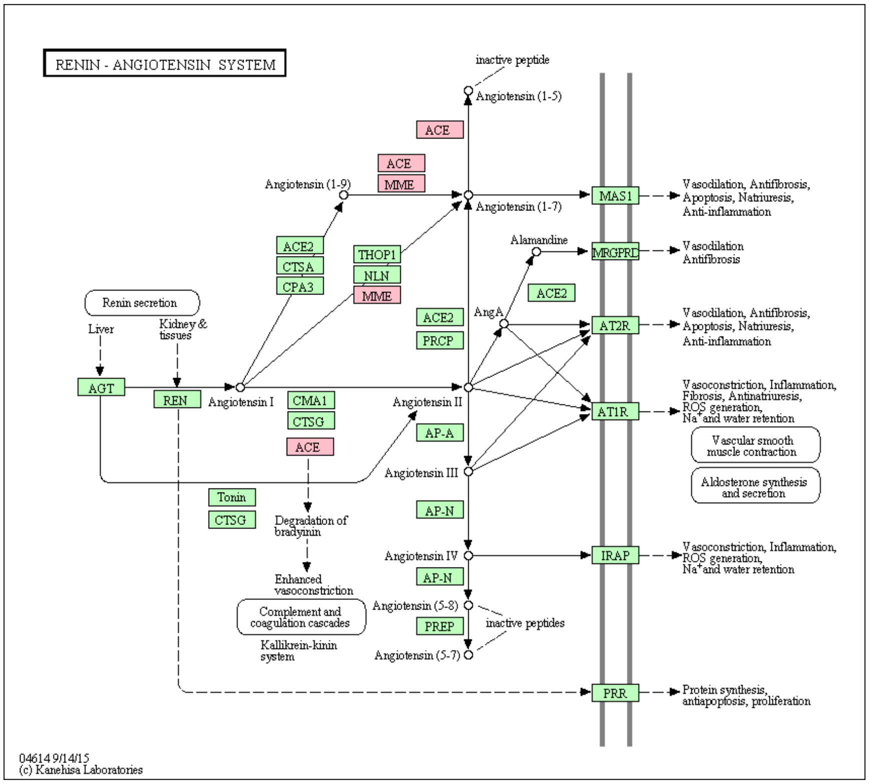

264.3 Legend:

RBH-Blast at 60% Identity + 50% Coverage
Green = Hit in *H. sapiens*
Red = Hit in *H. sapiens* and *C. milli*
White = Not in *H. sapiens*

265 Sulfur relay system

265.1 Human Pathway: HSA04122

265.2 Number of Hits: 2

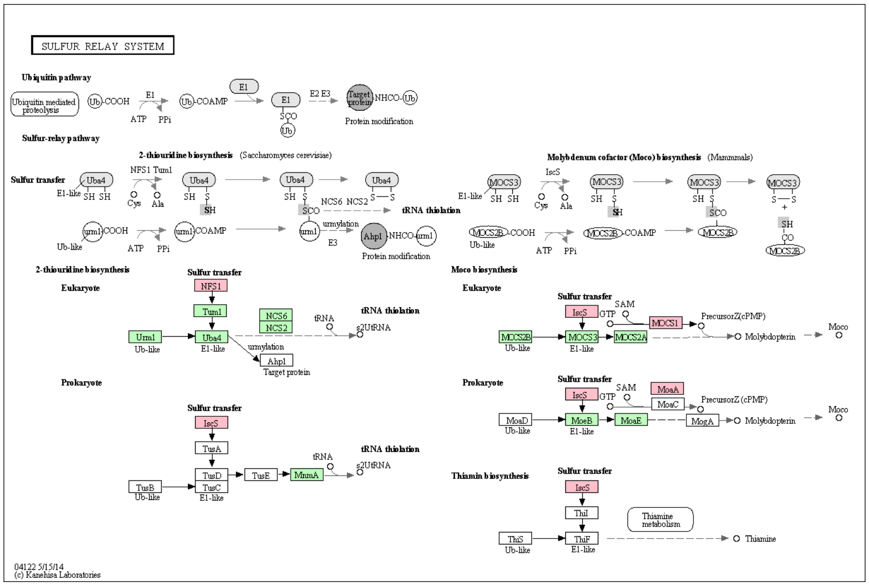

265.3 Legend:

RBH-Blast at 60% Identity + 50% Coverage
Green = Hit in *H. sapiens*
Red = Hit in *H. sapiens* and *C. milli*
White = Not in *H. sapiens*

266 Ubiquinone and other terpenoid-quinone biosynthesis

266.1 Human Pathway: HSA00130

266.2 Number of Hits: 2

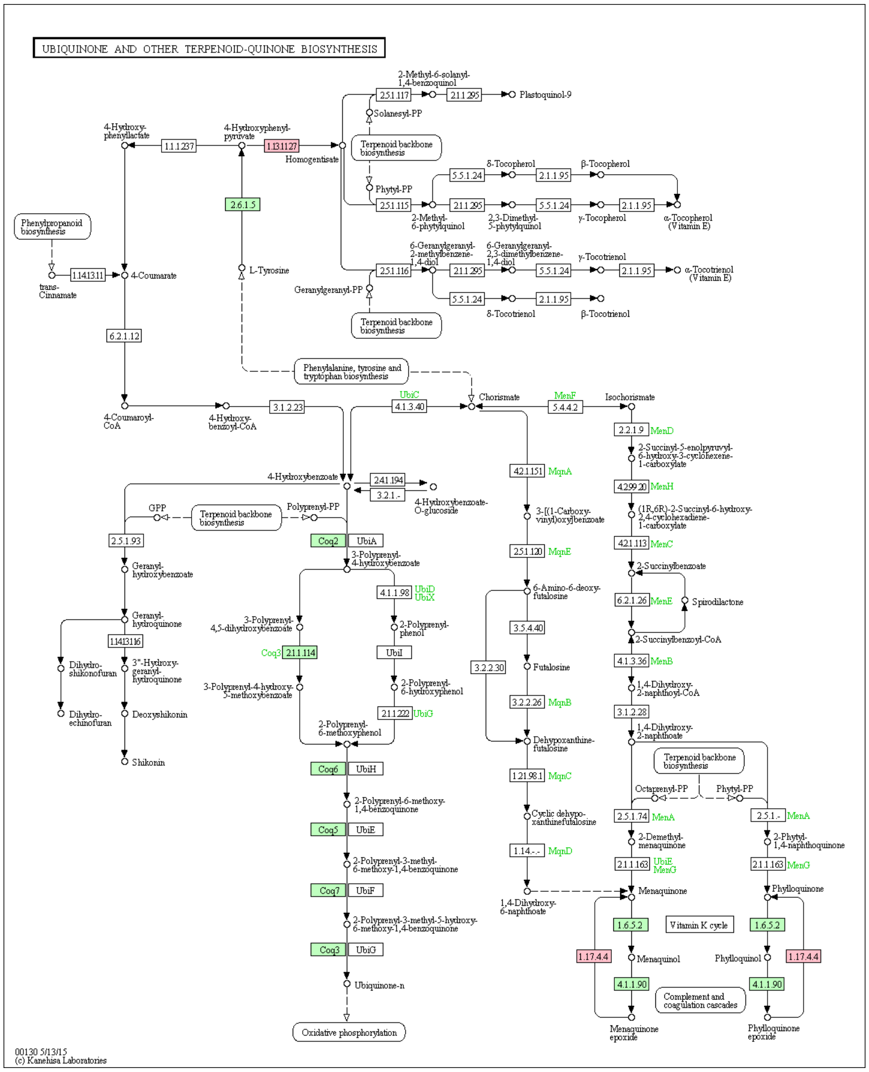

266.3 Legend:

RBH-Blast at 60% Identity + 50% Coverage
Green = Hit in *H. sapiens*
Red = Hit in *H. sapiens* and *C. milli*
White = Not in *H. sapiens*

267 One carbon pool by folate

267.1 Human Pathway: HSA00670

267.2 Number of Hits: 2

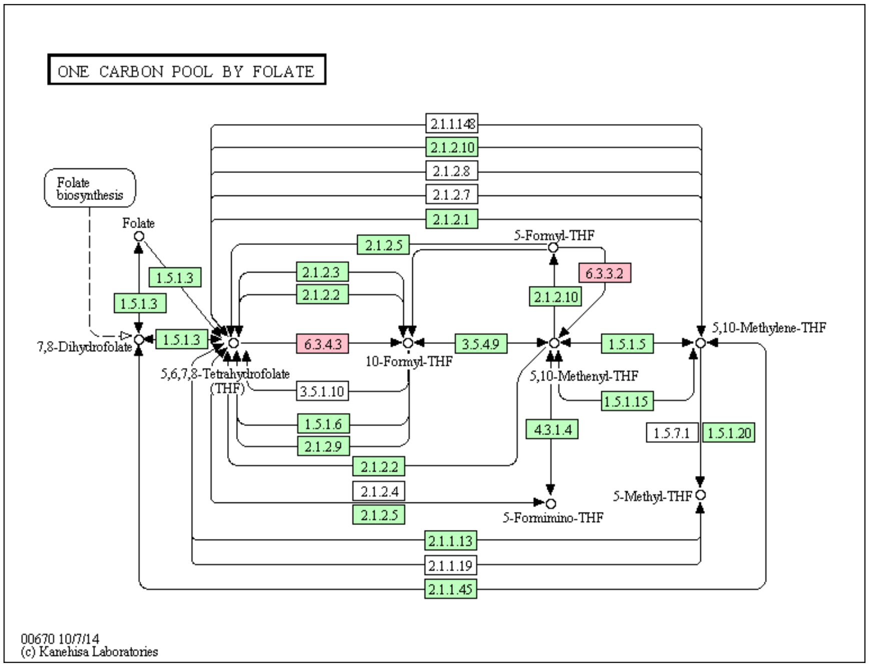

267.3 Legend:

RBH-Blast at 60% Identity + 50% Coverage
Green = Hit in *H. sapiens*
Red = Hit in *H. sapiens* and *C. milli*
White = Not in *H. sapiens*

268 Prion diseases

268.1 Human Pathway: HSA05020

268.2 Number of Hits: 2

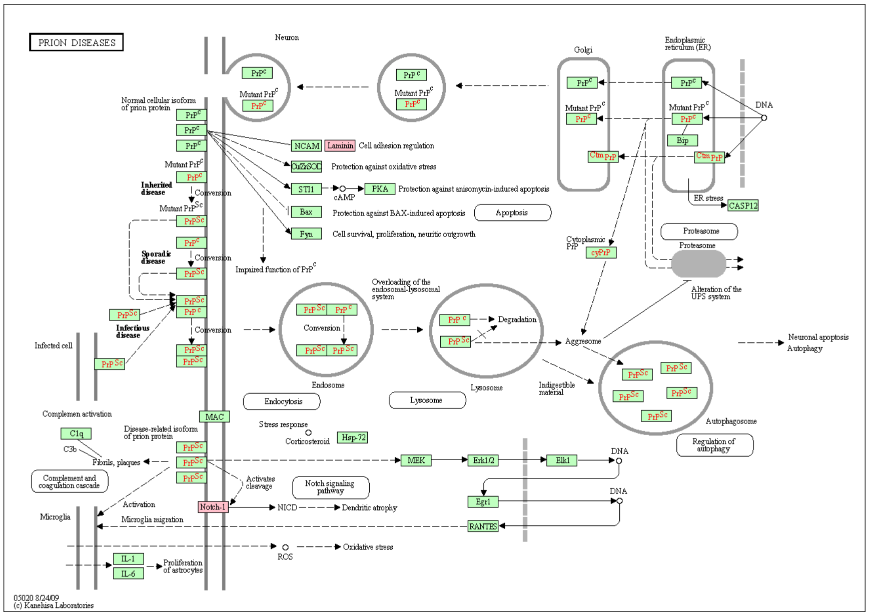

268.3 Legend:

RBH-Blast at 60% Identity + 50% Coverage
Green = Hit in *H. sapiens*
Red = Hit in *H. sapiens* and *C. milli*
White = Not in *H. sapiens*

269 Nicotinate and nicotinamide metabolism

269.1 Human Pathway: HSA00760

269.2 Number of Hits: 2

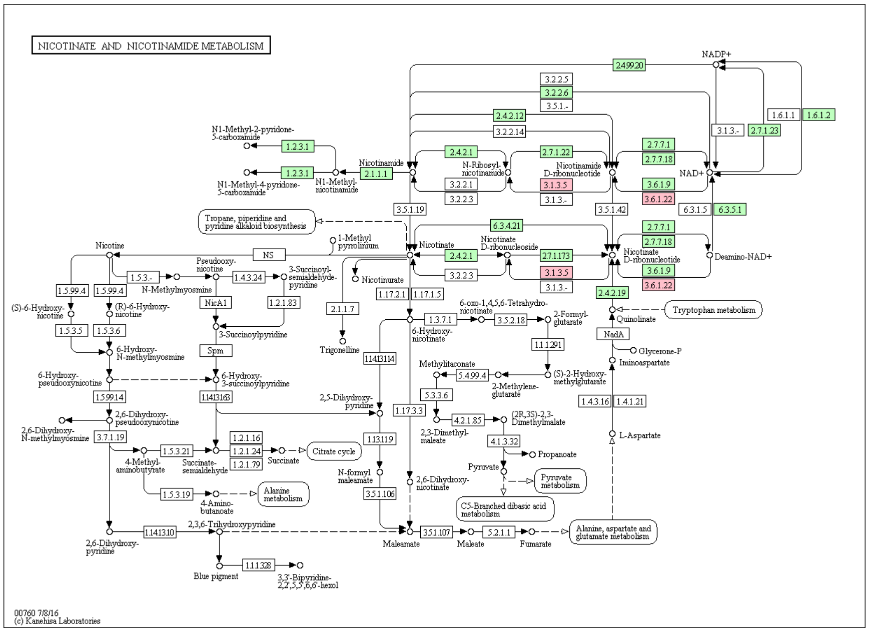

269.3 Legend:

RBH-Blast at 60% Identity + 50% Coverage
Green = Hit in *H. sapiens*
Red = Hit in *H. sapiens* and *C. milli*
White = Not in *H. sapiens*

270 Synthesis and degradation of ketone bodies

270.1 Human Pathway: HSA00072

270.2 Number of Hits: 2

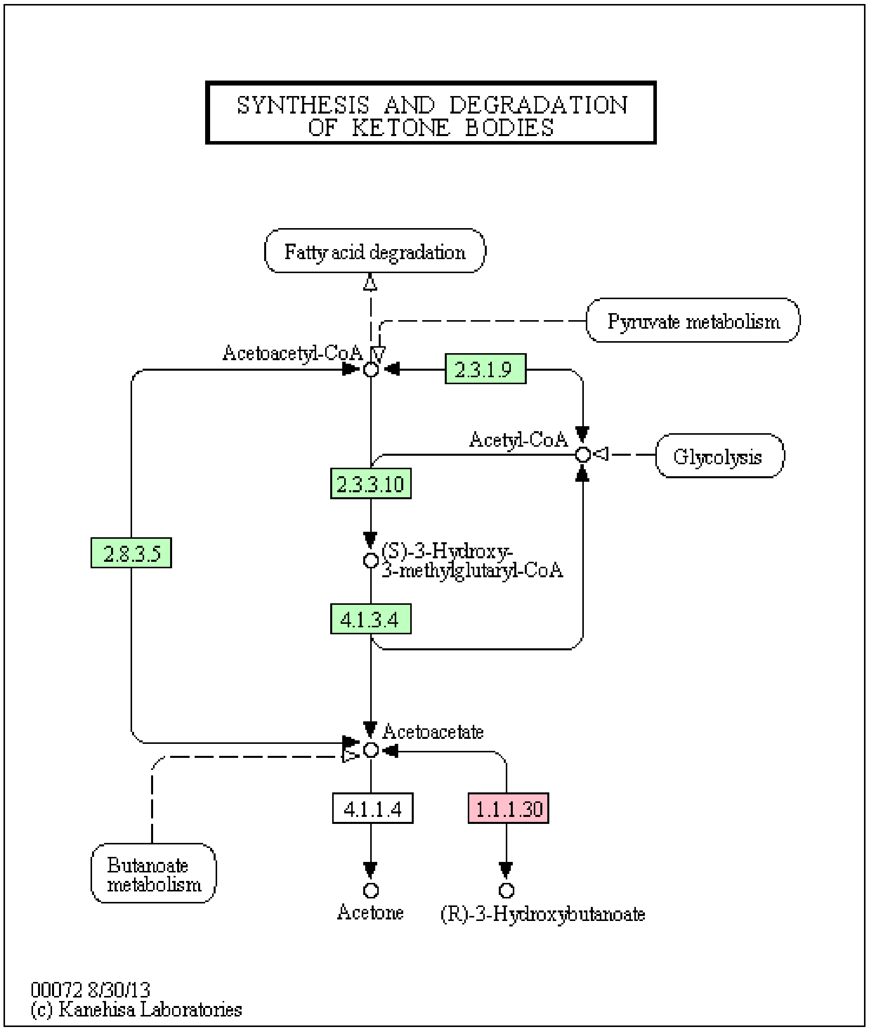

270.3 Legend:

RBH-Blast at 60% Identity + 50% Coverage
Green = Hit in *H. sapiens*
Red = Hit in *H. sapiens* and *C. milli*
White = Not in *H. sapiens*

271 Nitrogen metabolism

271.1 Human Pathway: HSA00910

271.2 Number of Hits: 2

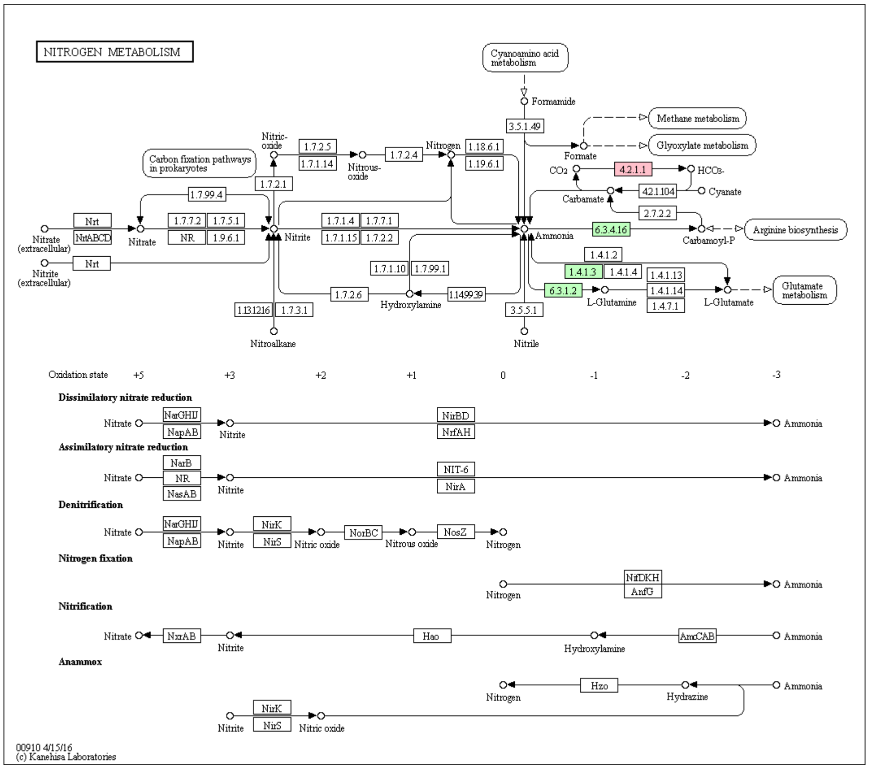

271.3 Legend:

RBH-Blast at 60% Identity + 50% Coverage
Green = Hit in *H. sapiens*
Red = Hit in *H. sapiens* and *C. milli*
White = Not in *H. sapiens*

272 Selenocompound metabolism

272.1 Human Pathway: HSA00450

272.2 Number of Hits: 2

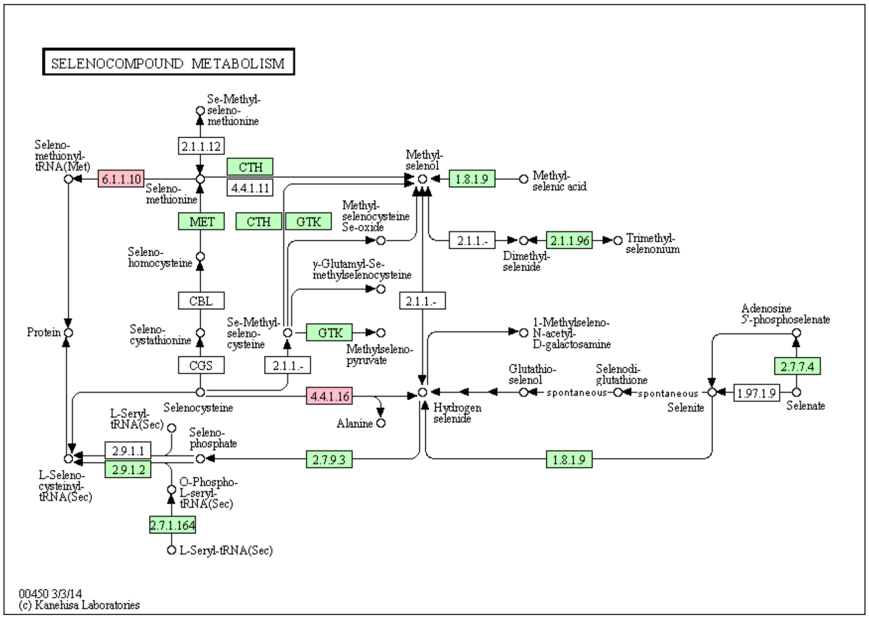

272.3 Legend:

RBH-Blast at 60% Identity + 50% Coverage
Green = Hit in *H. sapiens*
Red = Hit in *H. sapiens* and *C. milli*
White = Not in *H. sapiens*

273 Primary bile acid biosynthesis

273.1 Human Pathway: HSA00120

273.2 Number of Hits: 2

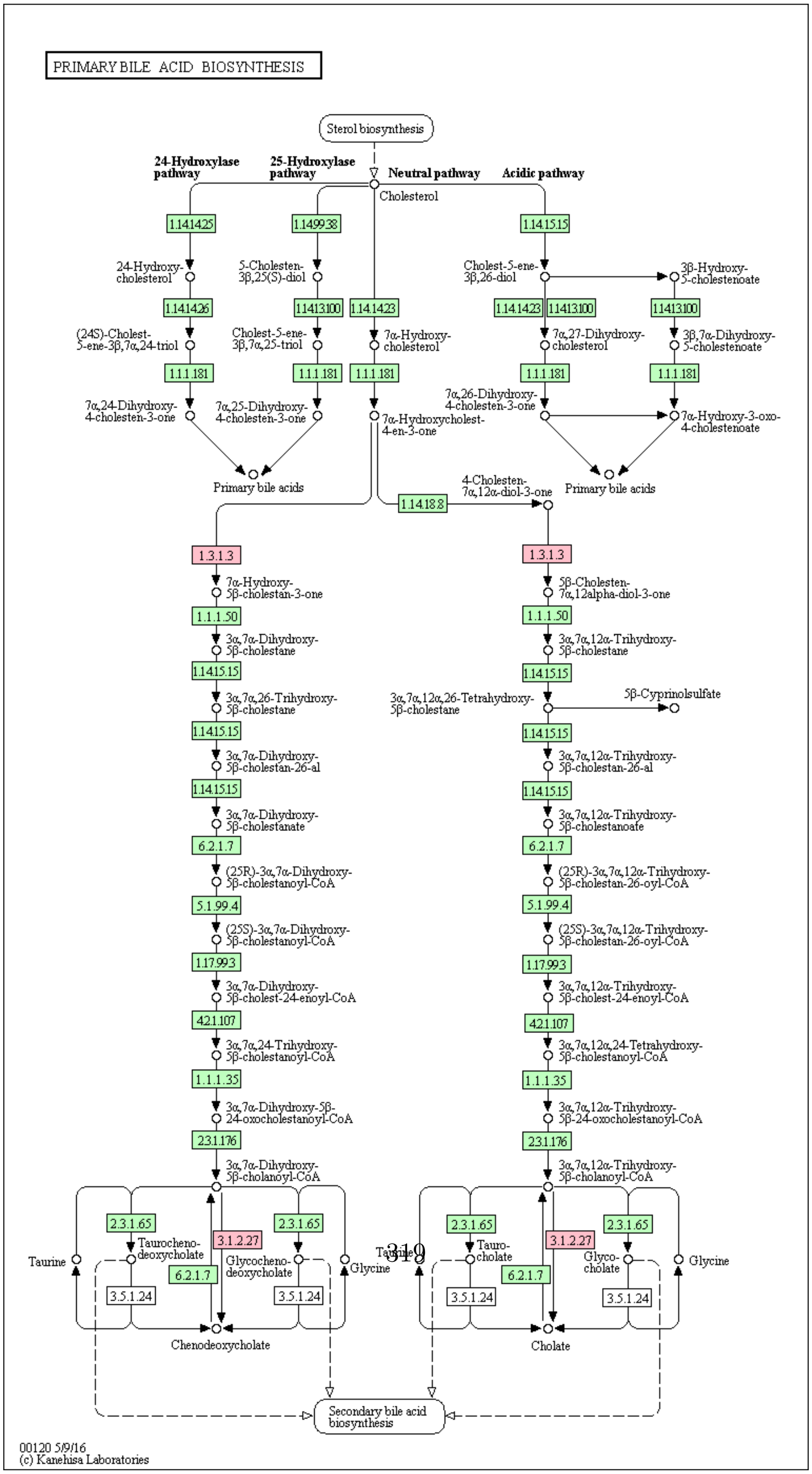

273.3 Legend:

RBH-Blast at 60% Identity + 50% Coverage
Green = Hit in *H. sapiens*
Red = Hit in *H. sapiens* and *C. milli*
White = Not in *H. sapiens*

274 Taurine and hypotaurine metabolism

274.1 Human Pathway: HSA00430

274.2 Number of Hits: 2

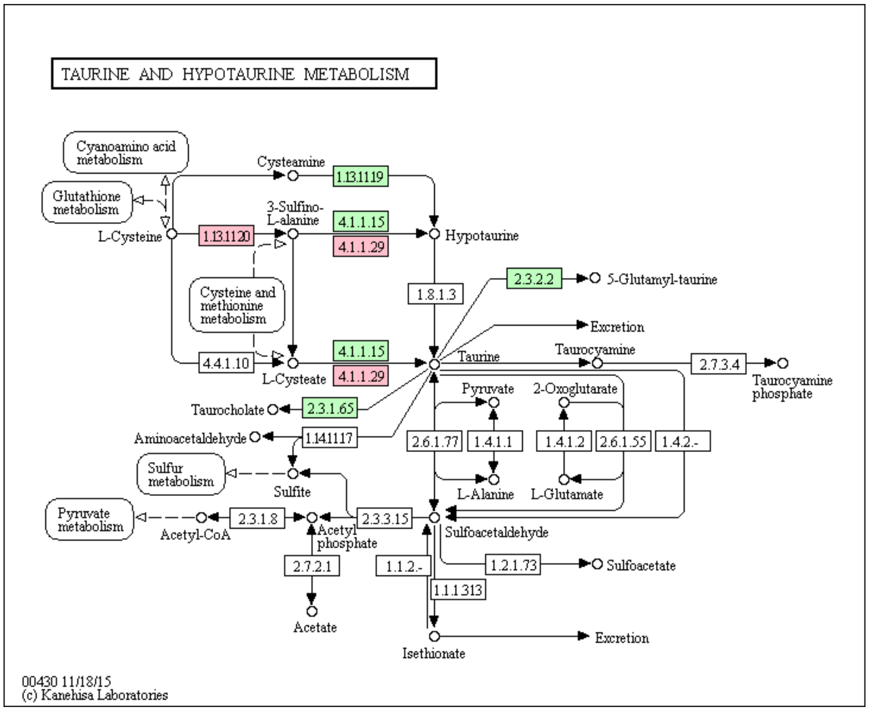

274.3 Legend:

RBH-Blast at 60% Identity + 50% Coverage
Green = Hit in *H. sapiens*
Red = Hit in *H. sapiens* and *C. milli*
White = Not in *H. sapiens*

275 alpha-Linolenic acid metabolism

275.1 Human Pathway: HSA00592

275.2 Number of Hits: 2

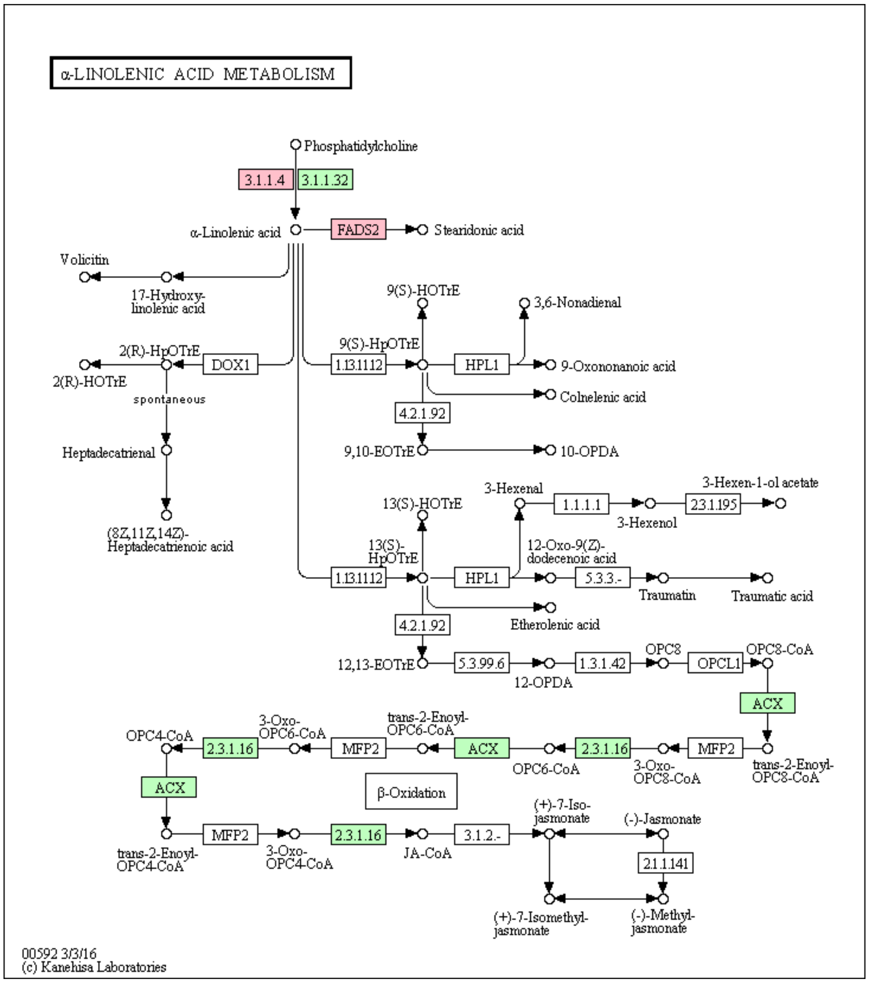

275.3 Legend:

RBH-Blast at 60% Identity + 50% Coverage
Green = Hit in *H. sapiens*
Red = Hit in *H. sapiens* and *C. milli*
White = Not in *H. sapiens*

276 Folate biosynthesis

276.1 Human Pathway: HSA00790

276.2 Number of Hits: 2

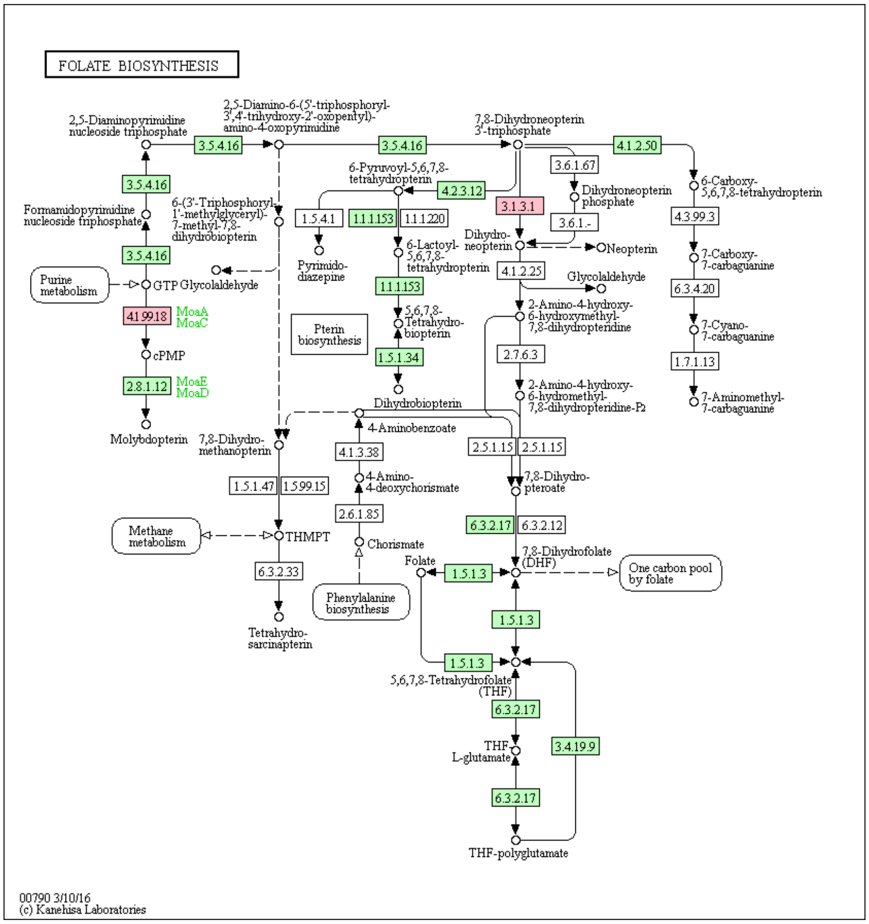

276.3 Legend:

RBH-Blast at 60% Identity + 50% Coverage
Green = Hit in *H. sapiens*
Red = Hit in *H. sapiens* and *C. milli*
White = Not in *H. sapiens*

277 Caffeine metabolism

277.1 Human Pathway: HSA00232

277.2 Number of Hits: 1

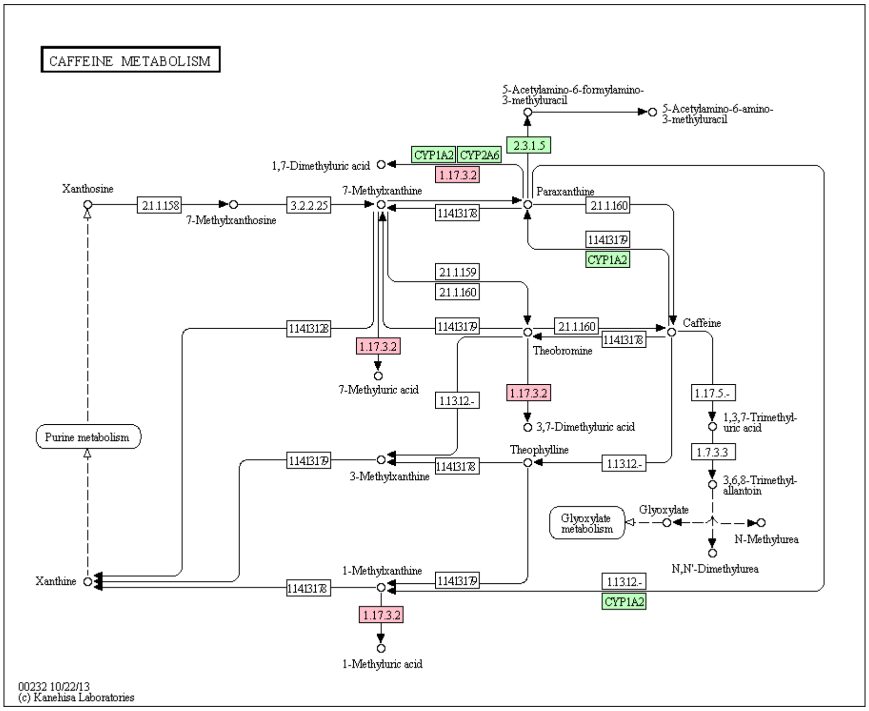

277.3 Legend:

RBH-Blast at 60% Identity + 50% Coverage
Green = Hit in *H. sapiens*
Red = Hit in *H. sapiens* and *C. milli*
White = Not in *H. sapiens*

278 Phenylalanine, tyrosine and tryptophan biosynthesis

278.1 Human Pathway: HSA00400

278.2 Number of Hits: 1

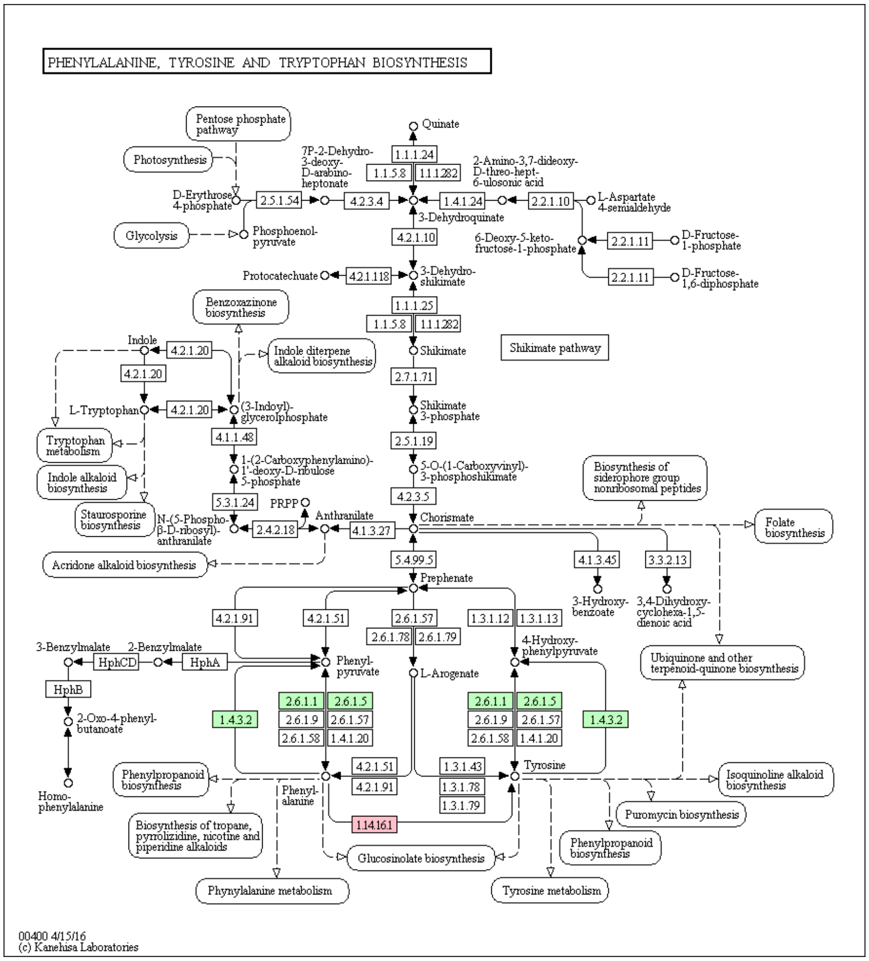

278.3 Legend:

RBH-Blast at 60% Identity + 50% Coverage
Green = Hit in *H. sapiens*
Red = Hit in *H. sapiens* and *C. milli*
White = Not in *H. sapiens*

279 Malaria

279.1 Human Pathway: HSA05144

279.2 Number of Hits: 1

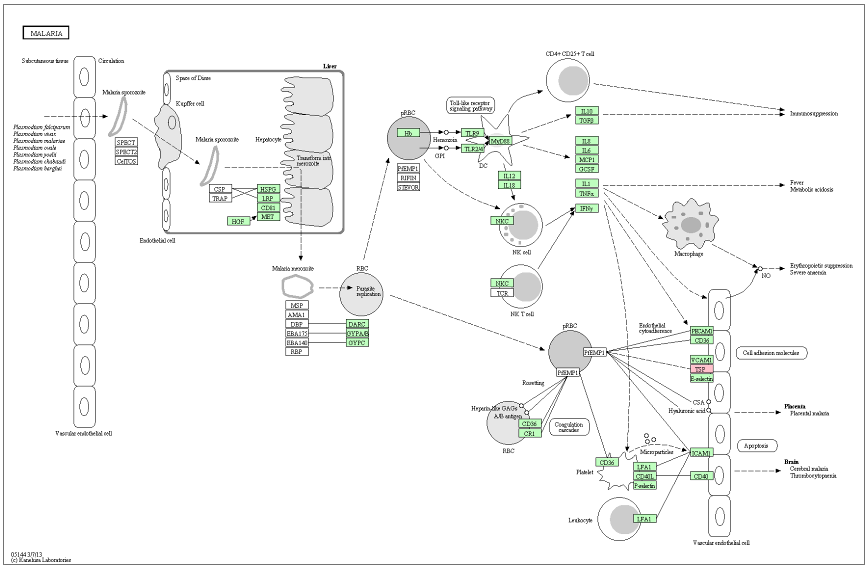

279.3 Legend:

RBH-Blast at 60% Identity + 50% Coverage
Green = Hit in *H. sapiens*
Red = Hit in *H. sapiens* and *C. milli*
White = Not in *H. sapiens*

280 Type I diabetes mellitus

280.1 Human Pathway: HSA04940

280.2 Number of Hits: 1

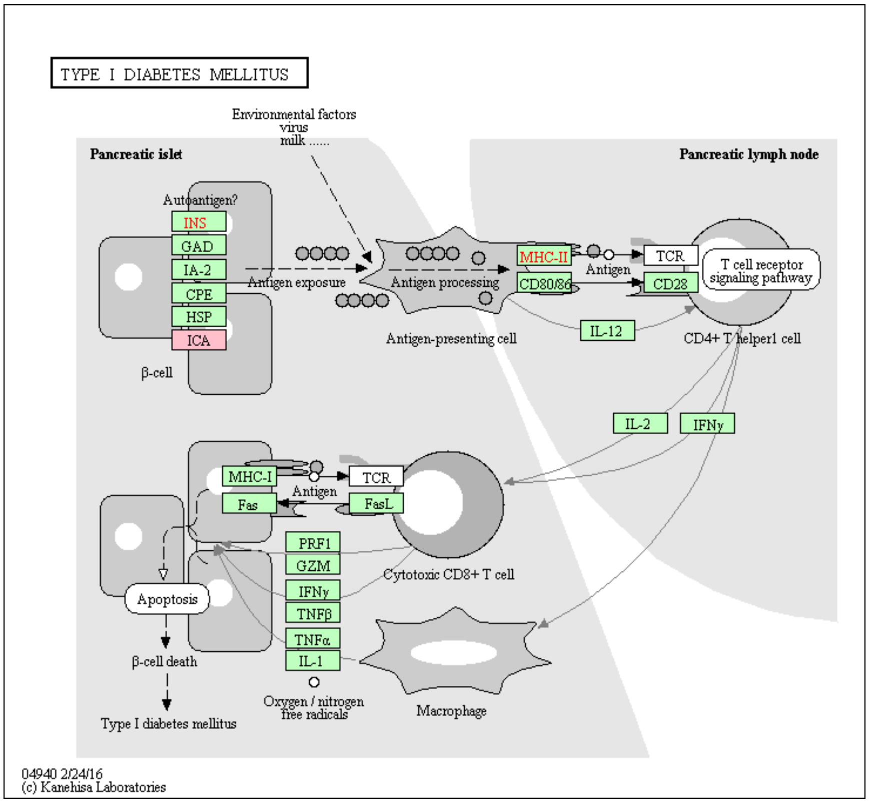

280.3 Legend:

RBH-Blast at 60% Identity + 50% Coverage
Green = Hit in *H. sapiens*
Red = Hit in *H. sapiens* and *C. milli*
White = Not in *H. sapiens*

281 Glycosaminoglycan biosynthesis - keratan sulfate

281.1 Human Pathway: HSA00533

281.2 Number of Hits: 1

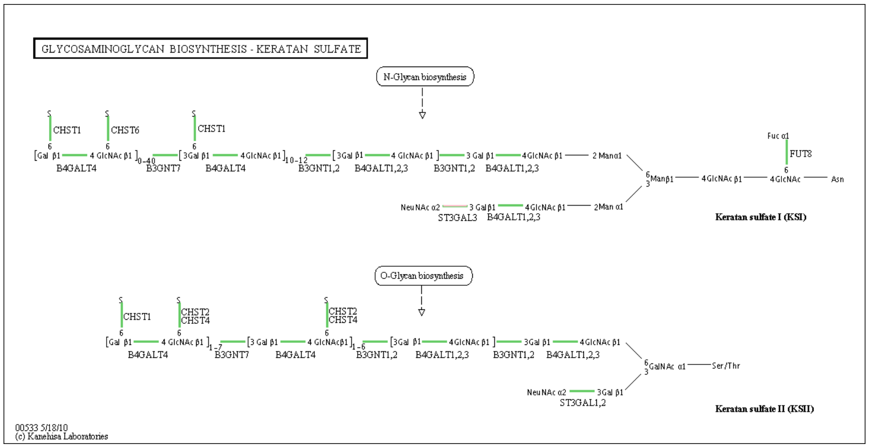

281.3 Legend:

RBH-Blast at 60% Identity + 50% Coverage
Green = Hit in *H. sapiens*
Red = Hit in *H. sapiens* and *C. milli*
White = Not in *H. sapiens*

282 Valine, leucine and isoleucine biosynthesis

282.1 Human Pathway: HSA00290

282.2 Number of Hits: 1

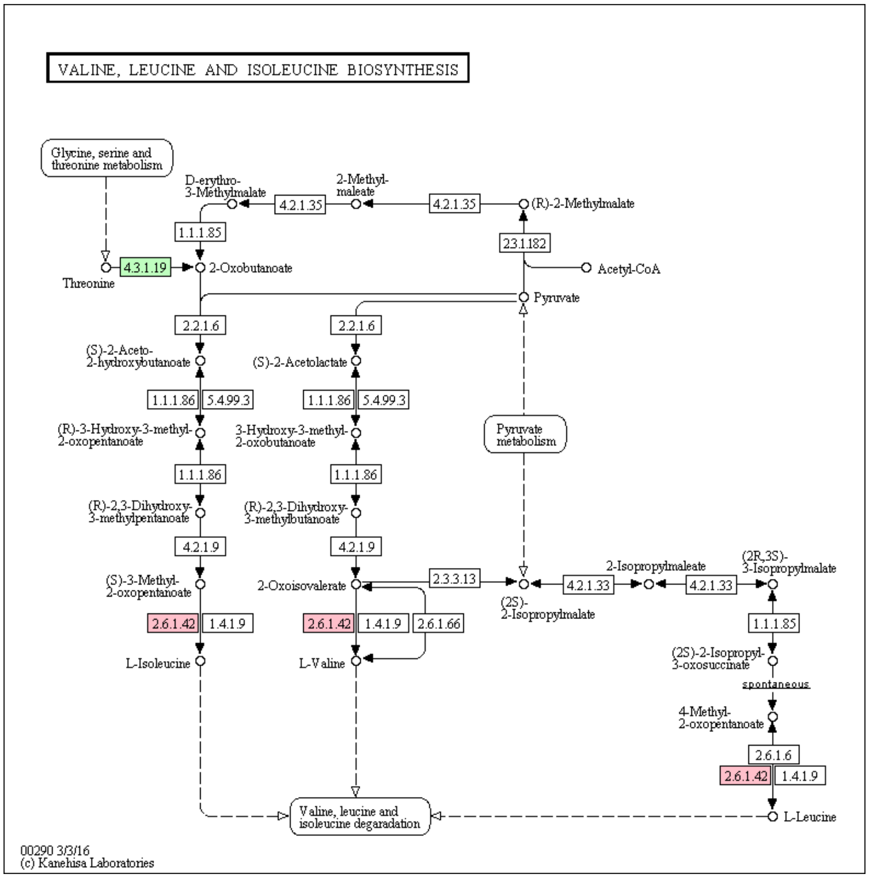

282.3 Legend:

RBH-Blast at 60% Identity + 50% Coverage
Green = Hit in *H. sapiens*
Red = Hit in *H. sapiens* and *C. milli*
White = Not in *H. sapiens*

283 2-Oxocarboxylic acid metabolism

283.1 Human Pathway: HSA01210

283.2 Number of Hits: 1

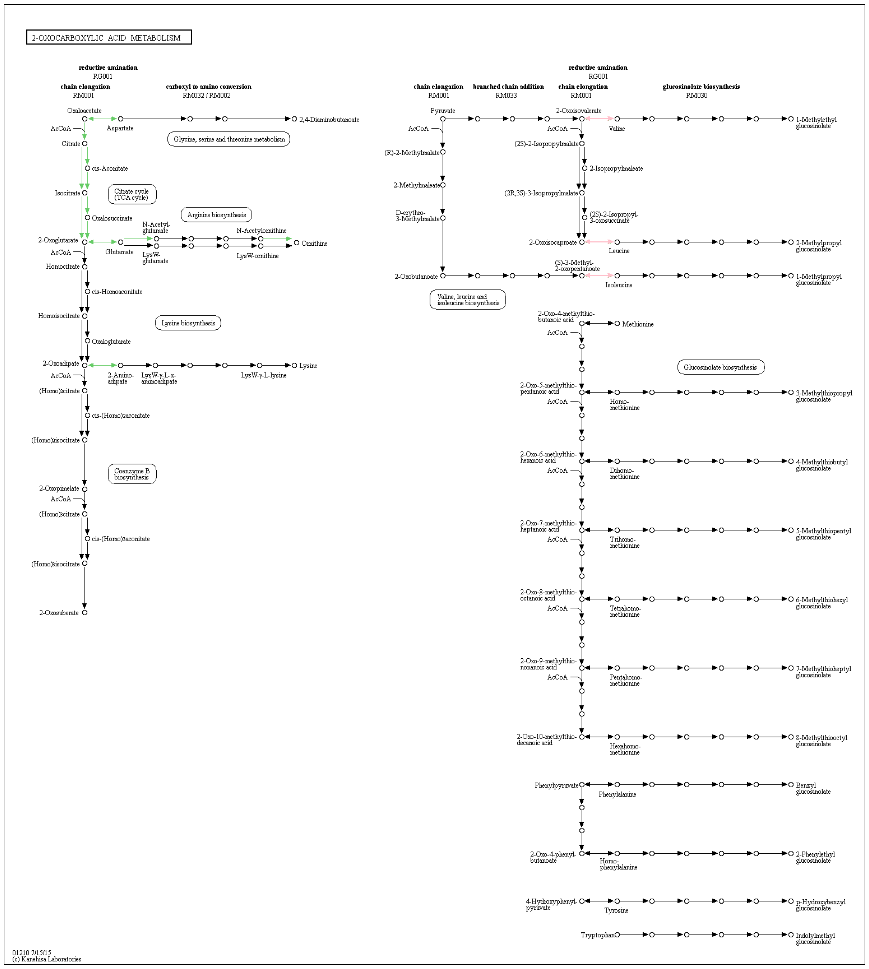

283.3 Legend:

RBH-Blast at 60% Identity + 50% Coverage
Green = Hit in *H. sapiens*
Red = Hit in *H. sapiens* and *C. milli*
White = Not in *H. sapiens*

284 D-Arginine and D-ornithine metabolism

284.1 Human Pathway: HSA00472

284.2 Number of Hits: 1

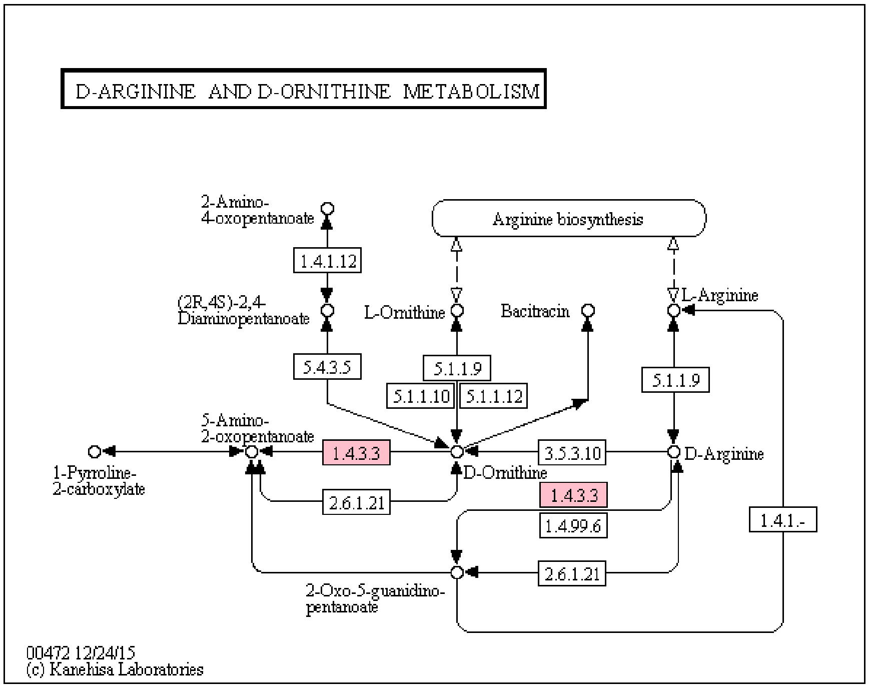

284.3 Legend:

RBH-Blast at 60% Identity + 50% Coverage
Green = Hit in *H. sapiens*
Red = Hit in *H. sapiens* and *C. milli*
White = Not in *H. sapiens*

285 Butirosin and neomycin biosynthesis

285.1 Human Pathway: HSA00524

285.2 Number of Hits: 1

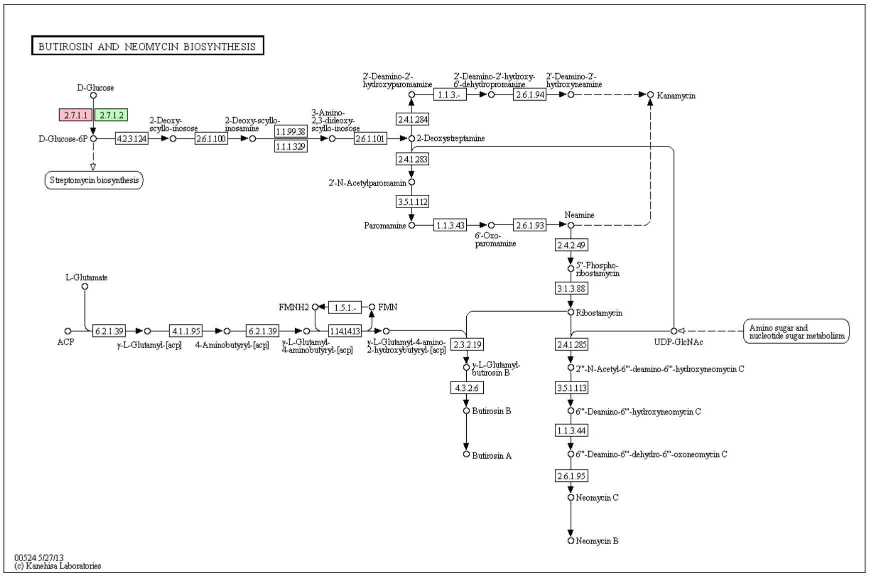

285.3 Legend:

RBH-Blast at 60% Identity + 50% Coverage
Green = Hit in *H. sapiens*
Red = Hit in *H. sapiens* and *C. milli*
White = Not in *H. sapiens*

286 Biotin metabolism

286.1 Human Pathway: HSA00780

286.2 Number of Hits: 1

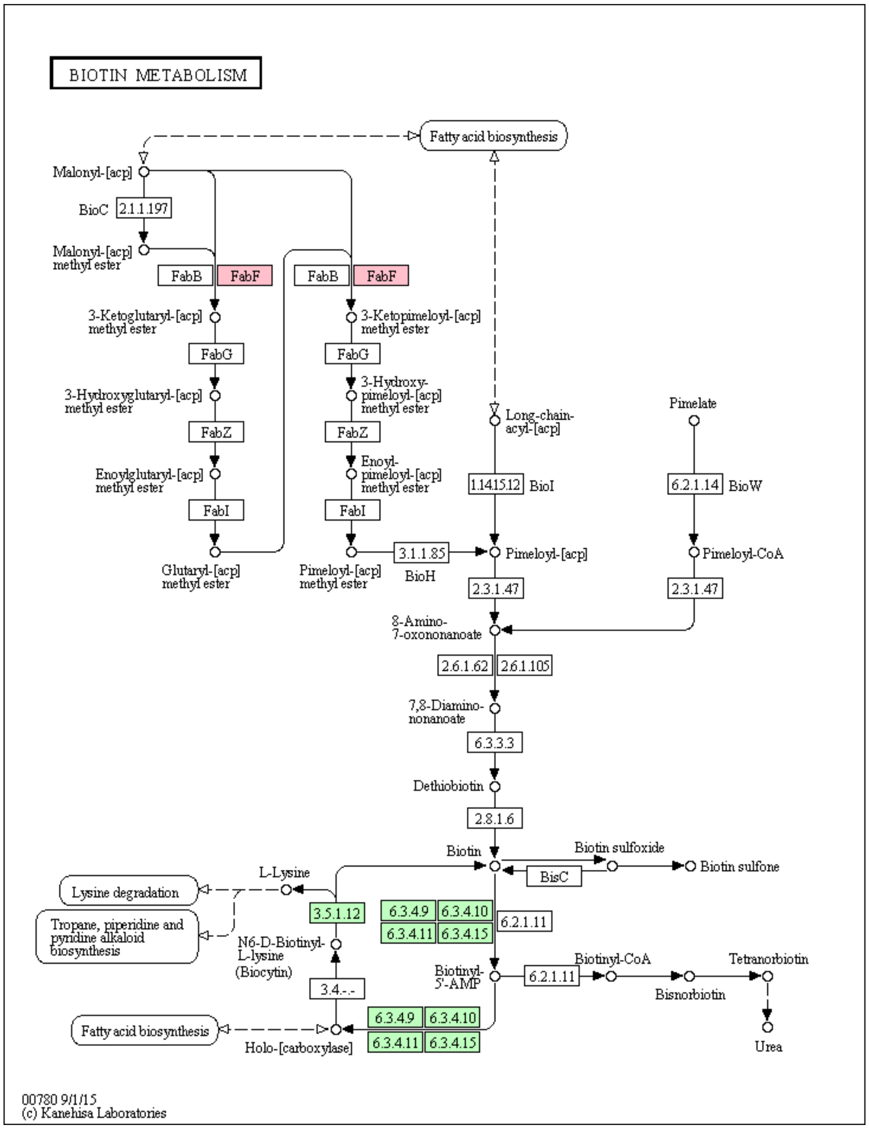

286.3 Legend:

RBH-Blast at 60% Identity + 50% Coverage
Green = Hit in *H. sapiens*
Red = Hit in *H. sapiens* and *C. milli*
White = Not in *H. sapiens*

---

287 Thiamine metabolism

287.1 Human Pathway: HSA00730

287.2 Number of Hits: 1

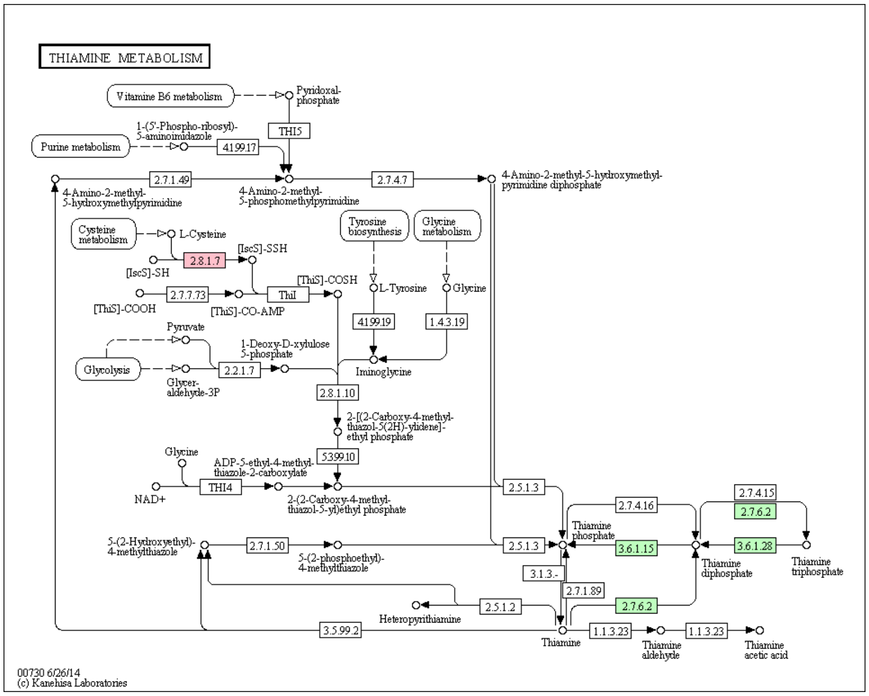

287.3 Legend:

RBH-Blast at 60% Identity + 50% Coverage
Green = Hit in *H. sapiens*
Red = Hit in *H. sapiens* and *C. milli*
White = Not in *H. sapiens*

---

288 Hematopoietic cell lineage

288.1 Human Pathway: HSA04640

288.2 Number of Hits: 1

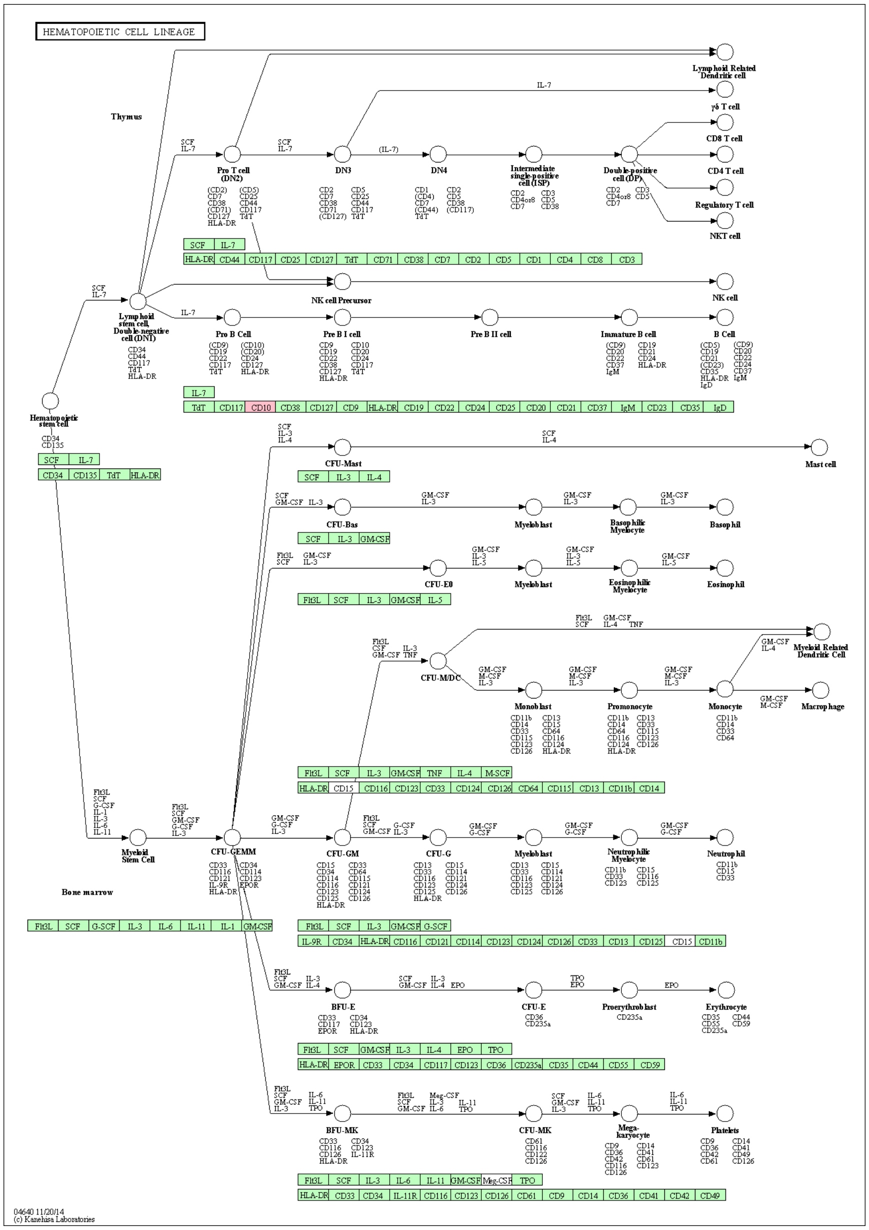

288.3 Legend:

RBH-Blast at 60% Identity + 50% Coverage
Green = Hit in *H. sapiens*
Red = Hit in *H. sapiens* and *C. milli*
White = Not in *H. sapiens*

289 Glycosphingolipid biosynthesis - globo series

289.1 Human Pathway: HSA00603

289.2 Number of Hits: 1

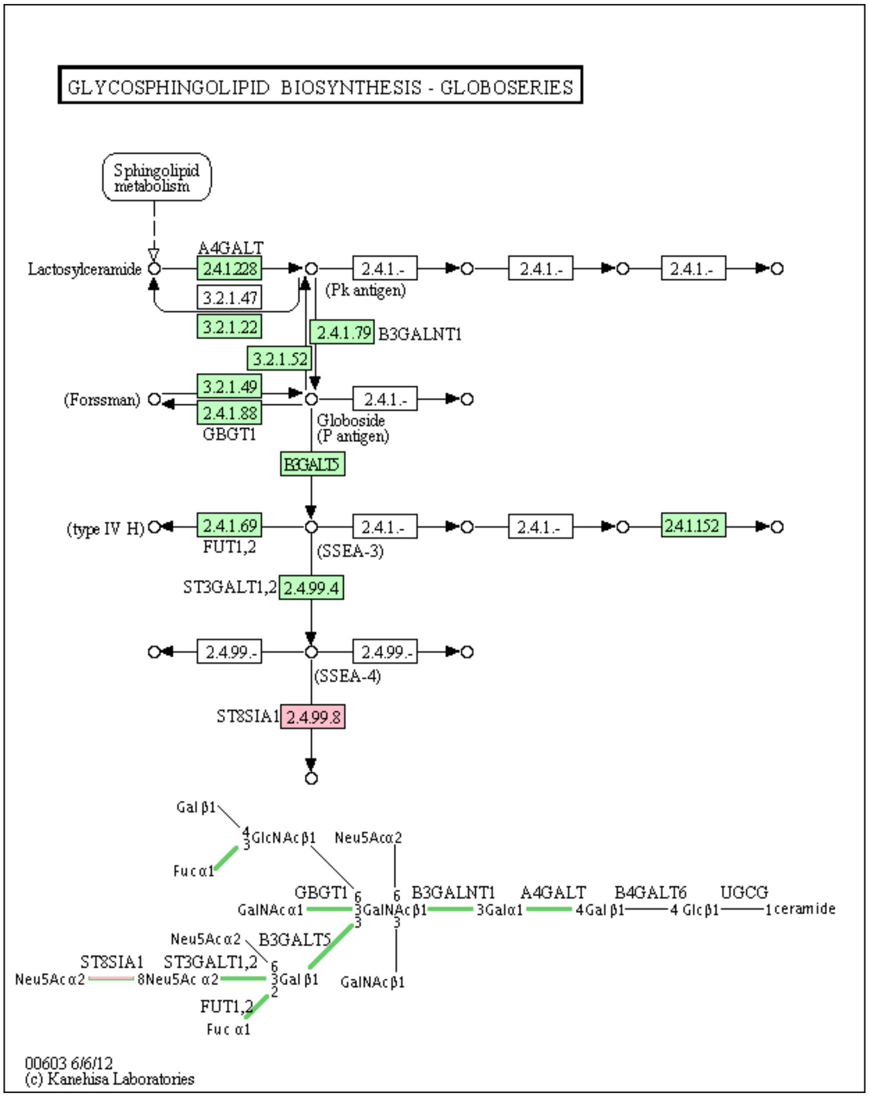

289.3 Legend:

RBH-Blast at 60% Identity + 50% Coverage
Green = Hit in *H. sapiens*
Red = Hit in *H. sapiens* and *C. milli*
White = Not in *H. sapiens*

---

290 Linoleic acid metabolism

290.1 Human Pathway: HSA00591

290.2 Number of Hits: 1

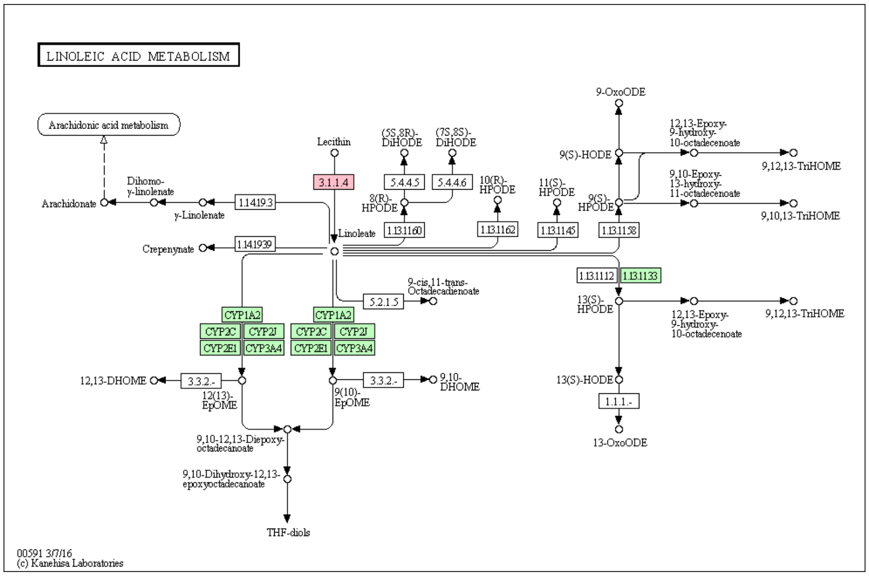

290.3 Legend:

RBH-Blast at 60% Identity + 50% Coverage
Green = Hit in *H. sapiens*
Red = Hit in *H. sapiens* and *C. milli*
White = Not in *H. sapiens*

---

291 Autoimmune thyroid disease

291.1 Human Pathway: HSA05320

291.2 Number of Hits: 1

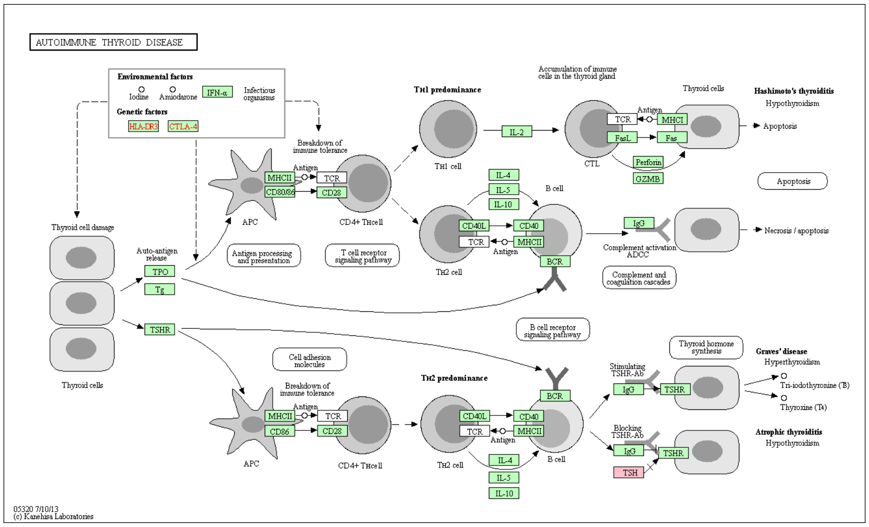

291.3 Legend:

RBH-Blast at 60% Identity + 50% Coverage
Green = Hit in *H. sapiens*
Red = Hit in *H. sapiens* and *C. milli*
White = Not in *H. sapiens*

292 Riboflavin metabolism

292.1 Human Pathway: HSA00740

292.2 Number of Hits: 1

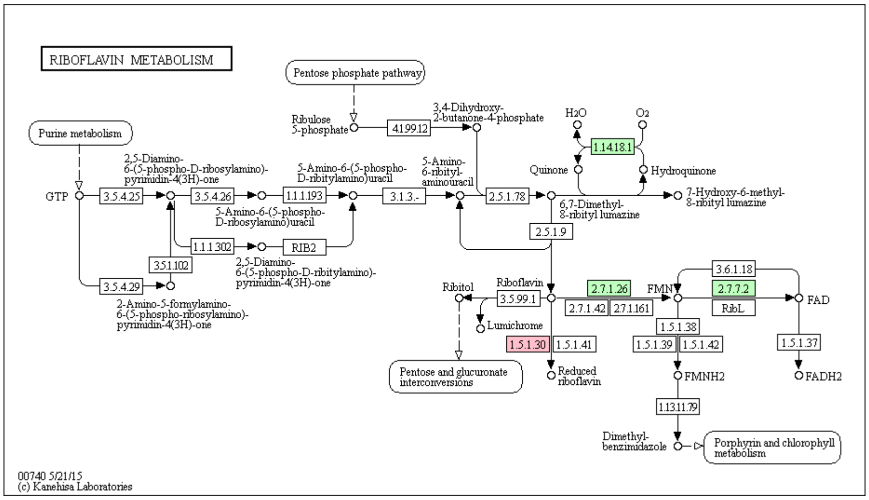

292.3 Legend:

RBH-Blast at 60% Identity + 50% Coverage
Green = Hit in *H. sapiens*
Red = Hit in *H. sapiens* and *C. milli*
White = Not in *H. sapiens*
