# Supplementary material for: Draft de novo transcriptome assembly and proteome characterization of the electric lobe of Tetronarce californica: a molecular tool for the study of cholinergic neurotransmission in the electric organ
Source: BMC Genomics. 2017 Aug 14;18:611. doi: 10.1186/s12864-017-3890-4 (PMC5557070; doi:10.1186/s12864-017-3890-4)
Supplement: Supplementary file 20 — KEGG Analysis. Intersection of H. sapiens With T. californica (set 12). See Additional file 3: TableS07 for details. (PDF 11366 kb) [file 12864_2017_3890_MOESM20_ESM.pdf]

Intersection of *H. sapiens* With *T. californica*

Set12: Reverse-Blast-Hit (RBH)-Blast at 60% Identity + 50% Coverage

Rodolfo Aramayo

April 30, 2017

Contents

1 Pathways in cancer **38**

1.1 Human Pathway: HSA05200 38

1.2 Number of Hits: 113 38

1.3 Legend: 38

2 Endocytosis **39**

2.1 Human Pathway: HSA04144 39

2.2 Number of Hits: 108 39

2.3 Legend: 39

3 MAPK signaling pathway **40**

3.1 Human Pathway: HSA04010 40

3.2 Number of Hits: 90 40

3.3 Legend: 40

4 PI3K-Akt signaling pathway **41**

4.1 Human Pathway: HSA04151 41

4.2 Number of Hits: 86 41

4.3 Legend: 41

5 Protein processing in endoplasmic reticulum **42**

5.1 Human Pathway: HSA04141 42

5.2 Number of Hits: 83 42

5.3 Legend: 42

6 Ribosome **43**
6.1 Human Pathway: HSA03010 43
6.2 Number of Hits: 80 43
6.3 Legend: 43

7 Epstein-Barr virus infection **44**
7.1 Human Pathway: HSA05169 44
7.2 Number of Hits: 76 44
7.3 Legend: 44

8 Huntington's disease **45**
8.1 Human Pathway: HSA05016 45
8.2 Number of Hits: 75 45
8.3 Legend: 45

9 Ubiquitin mediated proteolysis **46**
9.1 Human Pathway: HSA04120 46
9.2 Number of Hits: 73 46
9.3 Legend: 46

10 Regulation of actin cytoskeleton **47**
10.1 Human Pathway: HSA04810 47
10.2 Number of Hits: 72 47
10.3 Legend: 47

11 HTLV-I infection **48**
11.1 Human Pathway: HSA05166 48
11.2 Number of Hits: 72 48
11.3 Legend: 49

12 Purine metabolism **49**
12.1 Human Pathway: HSA00230 49
12.2 Number of Hits: 71 49
12.3 Legend: 49

13 Spliceosome **50**
13.1 Human Pathway: HSA03040 50
13.2 Number of Hits: 71 50
13.3 Legend: 50

14 Ras signaling pathway **51**
14.1 Human Pathway: HSA04014 51
14.2 Number of Hits: 70 51
14.3 Legend: 51

15 RNA transport **52**
15.1 Human Pathway: HSA03013 52
15.2 Number of Hits: 68 52
15.3 Legend: 52

16 Focal adhesion **53**
16.1 Human Pathway: HSA04510 53
16.2 Number of Hits: 66 53
16.3 Legend: 53

17 Alzheimer's disease **54**
17.1 Human Pathway: HSA05010 54
17.2 Number of Hits: 65 54
17.3 Legend: 54

18 Rap1 signaling pathway **55**
18.1 Human Pathway: HSA04015 55
18.2 Number of Hits: 64 55
18.3 Legend: 55

19 Viral carcinogenesis **56**
19.1 Human Pathway: HSA05203 56
19.2 Number of Hits: 61 56
19.3 Legend: 56

20 Proteoglycans in cancer **57**
20.1 Human Pathway: HSA05205 57
20.2 Number of Hits: 60 57
20.3 Legend: 57

21 mTOR signaling pathway **58**
21.1 Human Pathway: HSA04150 58
21.2 Number of Hits: 60 58
21.3 Legend: 58

22 Carbon metabolism **59**
22.1 Human Pathway: HSA01200 59
22.2 Number of Hits: 60 59
22.3 Legend: 59

23 Parkinson's disease **60**
23.1 Human Pathway: HSA05012 60
23.2 Number of Hits: 58 60
23.3 Legend: 60

24 Non-alcoholic fatty liver disease (NAFLD) **61**
24.1 Human Pathway: HSA04932 61
24.2 Number of Hits: 57 61
24.3 Legend: 61

25 Chemokine signaling pathway **62**
25.1 Human Pathway: HSA04062 62
25.2 Number of Hits: 56 62
25.3 Legend: 62

26 Dopaminergic synapse **63**
26.1 Human Pathway: HSA04728 63
26.2 Number of Hits: 56 63
26.3 Legend: 63

27 Oxytocin signaling pathway **64**
27.1 Human Pathway: HSA04921 64
27.2 Number of Hits: 56 64
27.3 Legend: 64

28 Oxidative phosphorylation **65**
28.1 Human Pathway: HSA00190 65
28.2 Number of Hits: 56 65
28.3 Legend: 65

29 cAMP signaling pathway **66**
29.1 Human Pathway: HSA04024 66
29.2 Number of Hits: 54 66
29.3 Legend: 66

30 Axon guidance **67**
30.1 Human Pathway: HSA04360 67
30.2 Number of Hits: 53 67
30.3 Legend: 67

31 Adrenergic signaling in cardiomyocytes **68**
31.1 Human Pathway: HSA04261 68
31.2 Number of Hits: 52 68
31.3 Legend: 68

32 FoxO signaling pathway **69**
32.1 Human Pathway: HSA04068 69
32.2 Number of Hits: 51 69
32.3 Legend: 69

33 Glutamatergic synapse **70**
33.1 Human Pathway: HSA04724 70
33.2 Number of Hits: 50 70
33.3 Legend: 70

34 cGMP-PKG signaling pathway **71**
34.1 Human Pathway: HSA04022 71
34.2 Number of Hits: 50 71
34.3 Legend: 71

35 AMPK signaling pathway **72**
35.1 Human Pathway: HSA04152 72
35.2 Number of Hits: 50 72
35.3 Legend: 72

36 Insulin signaling pathway **73**
36.1 Human Pathway: HSA04910 73
36.2 Number of Hits: 50 73
36.3 Legend: 73

37 Cell cycle **74**
37.1 Human Pathway: HSA04110 74
37.2 Number of Hits: 50 74
37.3 Legend: 74

38 Neurotrophin signaling pathway **75**
38.1 Human Pathway: HSA04722 75
38.2 Number of Hits: 49 75
38.3 Legend: 75

39 Oocyte meiosis **76**
39.1 Human Pathway: HSA04114 76
39.2 Number of Hits: 49 76
39.3 Legend: 76

40 Sphingolipid signaling pathway **77**
40.1 Human Pathway: HSA04071 77
40.2 Number of Hits: 48 77
40.3 Legend: 77

41 Retrograde endocannabinoid signaling **78**
41.1 Human Pathway: HSA04723 78
41.2 Number of Hits: 48 78
41.3 Legend: 78

42 Pyrimidine metabolism **79**
42.1 Human Pathway: HSA00240 79
42.2 Number of Hits: 47 79
42.3 Legend: 79

43 Phospholipase D signaling pathway **80**
43.1 Human Pathway: HSA04072 80
43.2 Number of Hits: 47 80
43.3 Legend: 80

44 Wnt signaling pathway **81**
44.1 Human Pathway: HSA04310 81
44.2 Number of Hits: 47 81
44.3 Legend: 81

45 Tight junction **82**
45.1 Human Pathway: HSA04530 82
45.2 Number of Hits: 46 82
45.3 Legend: 82

46 Neuroactive ligand-receptor interaction **83**
46.1 Human Pathway: HSA04080 83
46.2 Number of Hits: 45 83
46.3 Legend: 83

47 Phagosome **84**
47.1 Human Pathway: HSA04145 84
47.2 Number of Hits: 45 84
47.3 Legend: 84

48 mRNA surveillance pathway **85**
48.1 Human Pathway: HSA03015 85
48.2 Number of Hits: 44 85
48.3 Legend: 85

49 Thyroid hormone signaling pathway **86**
49.1 Human Pathway: HSA04919 86
49.2 Number of Hits: 44 86
49.3 Legend: 86

50 Alcoholism **87**
50.1 Human Pathway: HSA05034 87
50.2 Number of Hits: 43 87
50.3 Legend: 87

51 Influenza A **88**
51.1 Human Pathway: HSA05164 88
51.2 Number of Hits: 43 88
51.3 Legend: 88

52 GABAergic synapse **89**
52.1 Human Pathway: HSA04727 89
52.2 Number of Hits: 42 89
52.3 Legend: 89

53 Hippo signaling pathway **90**
53.1 Human Pathway: HSA04390 90
53.2 Number of Hits: 42 90
53.3 Legend: 90

54 Fc gamma R-mediated phagocytosis **91**
54.1 Human Pathway: HSA04666 91
54.2 Number of Hits: 41 91
54.3 Legend: 91

55 RNA degradation **92**
55.1 Human Pathway: HSA03018 92
55.2 Number of Hits: 41 92
55.3 Legend: 92

56 Platelet activation **93**
56.1 Human Pathway: HSA04611 93
56.2 Number of Hits: 41 93
56.3 Legend: 93

57 Herpes simplex infection **94**
57.1 Human Pathway: HSA05168 94
57.2 Number of Hits: 41 94
57.3 Legend: 94

58 Biosynthesis of amino acids **95**
58.1 Human Pathway: HSA01230 95
58.2 Number of Hits: 40 95
58.3 Legend: 95

59 Choline metabolism in cancer **96**
59.1 Human Pathway: HSA05231 96
59.2 Number of Hits: 40 96
59.3 Legend: 96

60 Apoptosis **97**
60.1 Human Pathway: HSA04210 97
60.2 Number of Hits: 39 97
60.3 Legend: 97

61 Morphine addiction **98**
61.1 Human Pathway: HSA05032 98
61.2 Number of Hits: 39 98
61.3 Legend: 98

62 Insulin resistance **99**
62.1 Human Pathway: HSA04931 99
62.2 Number of Hits: 39 99
62.3 Legend: 99

63 Cholinergic synapse **100**
63.1 Human Pathway: HSA04725 100
63.2 Number of Hits: 39 100
63.3 Legend: 100

64 Hepatitis B **101**
64.1 Human Pathway: HSA05161 101
64.2 Number of Hits: 39 101
64.3 Legend: 101

65 Progesterone-mediated oocyte maturation **102**
65.1 Human Pathway: HSA04914 102
65.2 Number of Hits: 39 102
65.3 Legend: 102

66 Circadian entrainment **103**
66.1 Human Pathway: HSA04713 103
66.2 Number of Hits: 38 103
66.3 Legend: 103

67 T cell receptor signaling pathway **104**
67.1 Human Pathway: HSA04660 104
67.2 Number of Hits: 37 104
67.3 Legend: 104

68 Tuberculosis **105**
68.1 Human Pathway: HSA05152 105
68.2 Number of Hits: 37 105
68.3 Legend: 105

69 Lysosome **106**
69.1 Human Pathway: HSA04142 106
69.2 Number of Hits: 37 106
69.3 Legend: 106

70 Bacterial invasion of epithelial cells **107**
70.1 Human Pathway: HSA05100 107
70.2 Number of Hits: 37 107
70.3 Legend: 107

71 Shigellosis **108**
71.1 Human Pathway: HSA05131 108
71.2 Number of Hits: 36 108
71.3 Legend: 108

72 MicroRNAs in cancer **109**
72.1 Human Pathway: HSA05206 109
72.2 Number of Hits: 36 109
72.3 Legend: 110

73 Serotonergic synapse **110**
73.1 Human Pathway: HSA04726 110
73.2 Number of Hits: 36 110
73.3 Legend: 110

74 Longevity regulating pathway **111**
74.1 Human Pathway: HSA04211 111
74.2 Number of Hits: 35 111
74.3 Legend: 111

75 Hepatitis C **112**
75.1 Human Pathway: HSA05160 112
75.2 Number of Hits: 35 112
75.3 Legend: 112

76 Leukocyte transendothelial migration **113**
76.1 Human Pathway: HSA04670 113
76.2 Number of Hits: 35 113
76.3 Legend: 113

77 Synaptic vesicle cycle **114**
77.1 Human Pathway: HSA04721 114
77.2 Number of Hits: 35 114
77.3 Legend: 114

78 Phosphatidylinositol signaling system **115**
78.1 Human Pathway: HSA04070 115
78.2 Number of Hits: 35 115
78.3 Legend: 115

79 Signaling pathways regulating pluripotency of stem cells **116**
79.1 Human Pathway: HSA04550 116
79.2 Number of Hits: 35 116
79.3 Legend: 116

80 Peroxisome **117**
80.1 Human Pathway: HSA04146 117
80.2 Number of Hits: 34 117
80.3 Legend: 117

81 Calcium signaling pathway **118**
81.1 Human Pathway: HSA04020 118
81.2 Number of Hits: 34 118
81.3 Legend: 118

82 AGE-RAGE signaling pathway in diabetic complications **119**
82.1 Human Pathway: HSA04933 119
82.2 Number of Hits: 34 119
82.3 Legend: 119

83 Estrogen signaling pathway **120**
83.1 Human Pathway: HSA04915 120
83.2 Number of Hits: 34 120
83.3 Legend: 120

84 Glucagon signaling pathway **121**
84.1 Human Pathway: HSA04922 121
84.2 Number of Hits: 33 121
84.3 Legend: 121

85 HIF-1 signaling pathway **122**
85.1 Human Pathway: HSA04066 122
85.2 Number of Hits: 33 122
85.3 Legend: 122

86 Ribosome biogenesis in eukaryotes 123
86.1 Human Pathway: HSA03008 123
86.2 Number of Hits: 33 123
86.3 Legend: 124

87 Gap junction 124
87.1 Human Pathway: HSA04540 124
87.2 Number of Hits: 33 124
87.3 Legend: 125

88 Osteoclast differentiation 125
88.1 Human Pathway: HSA04380 125
88.2 Number of Hits: 32 125
88.3 Legend: 125

89 Valine, leucine and isoleucine degradation 126
89.1 Human Pathway: HSA00280 126
89.2 Number of Hits: 32 126
89.3 Legend: 127

90 Salmonella infection 127
90.1 Human Pathway: HSA05132 127
90.2 Number of Hits: 32 127
90.3 Legend: 128

91 Epithelial cell signaling in Helicobacter pylori infection 128
91.1 Human Pathway: HSA05120 128
91.2 Number of Hits: 31 128
91.3 Legend: 129

92 Toxoplasmosis 129
92.1 Human Pathway: HSA05145 129
92.2 Number of Hits: 31 129
92.3 Legend: 129

93 ErbB signaling pathway 130
93.1 Human Pathway: HSA04012 130
93.2 Number of Hits: 31 130
93.3 Legend: 130

94 GnRH signaling pathway **131**
94.1 Human Pathway: HSA04912 131
94.2 Number of Hits: 31 131
94.3 Legend: 131

95 Vascular smooth muscle contraction **132**
95.1 Human Pathway: HSA04270 132
95.2 Number of Hits: 31 132
95.3 Legend: 132

96 Glycolysis / Gluconeogenesis **133**
96.1 Human Pathway: HSA00010 133
96.2 Number of Hits: 30 133
96.3 Legend: 134

97 Renal cell carcinoma **134**
97.1 Human Pathway: HSA05211 134
97.2 Number of Hits: 30 134
97.3 Legend: 134

98 Aminoacyl-tRNA biosynthesis **135**
98.1 Human Pathway: HSA00970 135
98.2 Number of Hits: 29 135
98.3 Legend: 135

99 Basal transcription factors **136**
99.1 Human Pathway: HSA03022 136
99.2 Number of Hits: 28 136
99.3 Legend: 136

100 Proteasome **137**
100.1 Human Pathway: HSA03050 137
100.2 Number of Hits: 28 137
100.3 Legend: 137

101 Fc epsilon RI signaling pathway **138**
101.1 Human Pathway: HSA04664 138
101.2 Number of Hits: 28 138
101.3 Legend: 138

102 Chagas disease (American trypanosomiasis) 139
102.1 Human Pathway: HSA05142 139
102.2 Number of Hits: 28 139
102.3 Legend: 139

103 Adherens junction 140
103.1 Human Pathway: HSA04520 140
103.2 Number of Hits: 28 140
103.3 Legend: 140

104 Long-term potentiation 141
104.1 Human Pathway: HSA04720 141
104.2 Number of Hits: 28 141
104.3 Legend: 141

105 B cell receptor signaling pathway 142
105.1 Human Pathway: HSA04662 142
105.2 Number of Hits: 28 142
105.3 Legend: 142

106 Pancreatic cancer 143
106.1 Human Pathway: HSA05212 143
106.2 Number of Hits: 27 143
106.3 Legend: 143

107 Prolactin signaling pathway 144
107.1 Human Pathway: HSA04917 144
107.2 Number of Hits: 27 144
107.3 Legend: 144

108 Inflammatory mediator regulation of TRP channels 145
108.1 Human Pathway: HSA04750 145
108.2 Number of Hits: 27 145
108.3 Legend: 145

109 Glycerophospholipid metabolism 146
109.1 Human Pathway: HSA00564 146
109.2 Number of Hits: 27 146
109.3 Legend: 146

110 Long-term depression **147**
110.1 Human Pathway: HSA04730 147
110.2 Number of Hits: 26 147
110.3 Legend: 147

111 Central carbon metabolism in cancer **148**
111.1 Human Pathway: HSA05230 148
111.2 Number of Hits: 26 148
111.3 Legend: 148

112 Melanogenesis **149**
112.1 Human Pathway: HSA04916 149
112.2 Number of Hits: 26 149
112.3 Legend: 149

113 Transcriptional misregulation in cancer **150**
113.1 Human Pathway: HSA05202 150
113.2 Number of Hits: 26 150
113.3 Legend: 150

114 Dilated cardiomyopathy **151**
114.1 Human Pathway: HSA05414 151
114.2 Number of Hits: 26 151
114.3 Legend: 151

115 Inositol phosphate metabolism **152**
115.1 Human Pathway: HSA00562 152
115.2 Number of Hits: 26 152
115.3 Legend: 152

116 Adipocytokine signaling pathway **153**
116.1 Human Pathway: HSA04920 153
116.2 Number of Hits: 25 153
116.3 Legend: 153

117 TGF-beta signaling pathway **154**
117.1 Human Pathway: HSA04350 154
117.2 Number of Hits: 25 154
117.3 Legend: 154

118 TNF signaling pathway **155**
118.1 Human Pathway: HSA04668 155
118.2 Number of Hits: 25 155
118.3 Legend: 155

119 Hypertrophic cardiomyopathy (HCM) **156**
119.1 Human Pathway: HSA05410 156
119.2 Number of Hits: 25 156
119.3 Legend: 156

120 Measles **157**
120.1 Human Pathway: HSA05162 157
120.2 Number of Hits: 25 157
120.3 Legend: 158

121 Fatty acid metabolism **159**
121.1 Human Pathway: HSA01212 159
121.2 Number of Hits: 25 159
121.3 Legend: 160

122 Colorectal cancer **160**
122.1 Human Pathway: HSA05210 160
122.2 Number of Hits: 24 160
122.3 Legend: 160

123 Toll-like receptor signaling pathway **161**
123.1 Human Pathway: HSA04620 161
123.2 Number of Hits: 24 161
123.3 Legend: 161

124 Pancreatic secretion **162**
124.1 Human Pathway: HSA04972 162
124.2 Number of Hits: 24 162
124.3 Legend: 162

125 VEGF signaling pathway **163**
125.1 Human Pathway: HSA04370 163
125.2 Number of Hits: 24 163
125.3 Legend: 163

126 Longevity regulating pathway - multiple species **164**
126.1 Human Pathway: HSA04213 164
126.2 Number of Hits: 24 164
126.3 Legend: 164

127 Cardiac muscle contraction **165**
127.1 Human Pathway: HSA04260 165
127.2 Number of Hits: 24 165
127.3 Legend: 165

128 EGFR tyrosine kinase inhibitor resistance **166**
128.1 Human Pathway: HSA01521 166
128.2 Number of Hits: 24 166
128.3 Legend: 166

129 Nucleotide excision repair **167**
129.1 Human Pathway: HSA03420 167
129.2 Number of Hits: 23 167
129.3 Legend: 167

130 Natural killer cell mediated cytotoxicity **168**
130.1 Human Pathway: HSA04650 168
130.2 Number of Hits: 23 168
130.3 Legend: 168

131 Arrhythmogenic right ventricular cardiomyopathy (ARVC) **169**
131.1 Human Pathway: HSA05412 169
131.2 Number of Hits: 23 169
131.3 Legend: 169

132 Chronic myeloid leukemia **170**
132.1 Human Pathway: HSA05220 170
132.2 Number of Hits: 23 170
132.3 Legend: 170

133 Prostate cancer **171**
133.1 Human Pathway: HSA05215 171
133.2 Number of Hits: 23 171
133.3 Legend: 171

134 Amino sugar and nucleotide sugar metabolism **172**
134.1 Human Pathway: HSA00520 172
134.2 Number of Hits: 23 172
134.3 Legend: 173

135 Amyotrophic lateral sclerosis (ALS) **173**
135.1 Human Pathway: HSA05014 173
135.2 Number of Hits: 22 173
135.3 Legend: 173

136 Vibrio cholerae infection **174**
136.1 Human Pathway: HSA05110 174
136.2 Number of Hits: 22 174
136.3 Legend: 174

137 SNARE interactions in vesicular transport **175**
137.1 Human Pathway: HSA04130 175
137.2 Number of Hits: 22 175
137.3 Legend: 175

138 Pathogenic Escherichia coli infection **176**
138.1 Human Pathway: HSA05130 176
138.2 Number of Hits: 22 176
138.3 Legend: 176

139 Endocrine and other factor-regulated calcium reabsorption **177**
139.1 Human Pathway: HSA04961 177
139.2 Number of Hits: 21 177
139.3 Legend: 177

140 Insulin secretion **178**
140.1 Human Pathway: HSA04911 178
140.2 Number of Hits: 21 178
140.3 Legend: 178

141 Amphetamine addiction **179**
141.1 Human Pathway: HSA05031 179
141.2 Number of Hits: 21 179
141.3 Legend: 179

142 Small cell lung cancer **180**
142.1 Human Pathway: HSA05222 180
142.2 Number of Hits: 21 180
142.3 Legend: 180

143 Aldosterone synthesis and secretion **181**
143.1 Human Pathway: HSA04925 181
143.2 Number of Hits: 21 181
143.3 Legend: 181

144 Pyruvate metabolism **182**
144.1 Human Pathway: HSA00620 182
144.2 Number of Hits: 21 182
144.3 Legend: 182

145 Propanoate metabolism **183**
145.1 Human Pathway: HSA00640 183
145.2 Number of Hits: 21 183
145.3 Legend: 183

146 Acute myeloid leukemia **184**
146.1 Human Pathway: HSA05221 184
146.2 Number of Hits: 20 184
146.3 Legend: 184

147 Cysteine and methionine metabolism **185**
147.1 Human Pathway: HSA00270 185
147.2 Number of Hits: 20 185
147.3 Legend: 185

148 Lysine degradation **186**
148.1 Human Pathway: HSA00310 186
148.2 Number of Hits: 20 186
148.3 Legend: 186

149 Thyroid hormone synthesis **187**
149.1 Human Pathway: HSA04918 187
149.2 Number of Hits: 20 187
149.3 Legend: 187

150 N-Glycan biosynthesis **188**
150.1 Human Pathway: HSA00510 188
150.2 Number of Hits: 20 188
150.3 Legend: 189

151 Fatty acid degradation **189**
151.1 Human Pathway: HSA00071 189
151.2 Number of Hits: 19 189
151.3 Legend: 190

152 Jak-STAT signaling pathway **190**
152.1 Human Pathway: HSA04630 190
152.2 Number of Hits: 19 190
152.3 Legend: 190

153 Glycerolipid metabolism **191**
153.1 Human Pathway: HSA00561 191
153.2 Number of Hits: 19 191
153.3 Legend: 191

154 Glioma **192**
154.1 Human Pathway: HSA05214 192
154.2 Number of Hits: 19 192
154.3 Legend: 192

155 Glyoxylate and dicarboxylate metabolism **193**
155.1 Human Pathway: HSA00630 193
155.2 Number of Hits: 19 193
155.3 Legend: 193

156 Amoebiasis **194**
156.1 Human Pathway: HSA05146 194
156.2 Number of Hits: 19 194
156.3 Legend: 194

157 Arginine and proline metabolism **195**
157.1 Human Pathway: HSA00330 195
157.2 Number of Hits: 19 195
157.3 Legend: 195

158 Non-small cell lung cancer **196**
158.1 Human Pathway: HSA05223 196
158.2 Number of Hits: 19 196
158.3 Legend: 196

159 Cell adhesion molecules (CAMs) **197**
159.1 Human Pathway: HSA04514 197
159.2 Number of Hits: 19 197
159.3 Legend: 197

160 RNA polymerase **198**
160.1 Human Pathway: HSA03020 198
160.2 Number of Hits: 19 198
160.3 Legend: 198

161 Salivary secretion **199**
161.1 Human Pathway: HSA04970 199
161.2 Number of Hits: 18 199
161.3 Legend: 199

162 Glycine, serine and threonine metabolism **200**
162.1 Human Pathway: HSA00260 200
162.2 Number of Hits: 18 200
162.3 Legend: 200

163 p53 signaling pathway **201**
163.1 Human Pathway: HSA04115 201
163.2 Number of Hits: 18 201
163.3 Legend: 201

164 Gastric acid secretion **202**
164.1 Human Pathway: HSA04971 202
164.2 Number of Hits: 18 202
164.3 Legend: 202

165 PPAR signaling pathway **203**
165.1 Human Pathway: HSA03320 203
165.2 Number of Hits: 18 203
165.3 Legend: 203

166 Melanoma **204**
166.1 Human Pathway: HSA05218 204
166.2 Number of Hits: 18 204
166.3 Legend: 204

167 Citrate cycle (TCA cycle) **205**
167.1 Human Pathway: HSA00020 205
167.2 Number of Hits: 17 205
167.3 Legend: 205

168 Vasopressin-regulated water reabsorption **206**
168.1 Human Pathway: HSA04962 206
168.2 Number of Hits: 17 206
168.3 Legend: 206

169 Nicotine addiction **207**
169.1 Human Pathway: HSA05033 207
169.2 Number of Hits: 17 207
169.3 Legend: 207

170 Hedgehog signaling pathway **208**
170.1 Human Pathway: HSA04340 208
170.2 Number of Hits: 17 208
170.3 Legend: 208

171 beta-Alanine metabolism **209**
171.1 Human Pathway: HSA00410 209
171.2 Number of Hits: 17 209
171.3 Legend: 209

172 DNA replication **210**
172.1 Human Pathway: HSA03030 210
172.2 Number of Hits: 17 210
172.3 Legend: 210

173 Endometrial cancer **211**
173.1 Human Pathway: HSA05213 211
173.2 Number of Hits: 17 211
173.3 Legend: 211

174 Protein export **212**
174.1 Human Pathway: HSA03060 212
174.2 Number of Hits: 17 212
174.3 Legend: 212

175 Renin secretion **213**
175.1 Human Pathway: HSA04924 213
175.2 Number of Hits: 17 213
175.3 Legend: 213

176 Cocaine addiction **214**
176.1 Human Pathway: HSA05030 214
176.2 Number of Hits: 16 214
176.3 Legend: 214

177 Sphingolipid metabolism **215**
177.1 Human Pathway: HSA00600 215
177.2 Number of Hits: 16 215
177.3 Legend: 215

178 Cytosolic DNA-sensing pathway **216**
178.1 Human Pathway: HSA04623 216
178.2 Number of Hits: 16 216
178.3 Legend: 216

179 Alanine, aspartate and glutamate metabolism **217**
179.1 Human Pathway: HSA00250 217
179.2 Number of Hits: 16 217
179.3 Legend: 217

180 Other types of O-glycan biosynthesis **218**
180.1 Human Pathway: HSA00514 218
180.2 Number of Hits: 16 218
180.3 Legend: 218

181 Rheumatoid arthritis **219**
181.1 Human Pathway: HSA05323 219
181.2 Number of Hits: 15 219
181.3 Legend: 219

182 NF-kappa B signaling pathway **220**
182.1 Human Pathway: HSA04064 220
182.2 Number of Hits: 15 220
182.3 Legend: 220

183 Fructose and mannose metabolism **221**
183.1 Human Pathway: HSA00051 221
183.2 Number of Hits: 15 221
183.3 Legend: 221

184 Type II diabetes mellitus **222**
184.1 Human Pathway: HSA04930 222
184.2 Number of Hits: 15 222
184.3 Legend: 222

185 Regulation of lipolysis in adipocytes **223**
185.1 Human Pathway: HSA04923 223
185.2 Number of Hits: 15 223
185.3 Legend: 223

186 Pertussis **224**
186.1 Human Pathway: HSA05133 224
186.2 Number of Hits: 15 224
186.3 Legend: 224

187 Legionellosis **225**
187.1 Human Pathway: HSA05134 225
187.2 Number of Hits: 14 225
187.3 Legend: 225

188 Protein digestion and absorption **226**
188.1 Human Pathway: HSA04974 226
188.2 Number of Hits: 14 226
188.3 Legend: 226

189 Steroid biosynthesis **227**
189.1 Human Pathway: HSA00100 227
189.2 Number of Hits: 14 227
189.3 Legend: 227

190 NOD-like receptor signaling pathway **228**
190.1 Human Pathway: HSA04621 228
190.2 Number of Hits: 14 228
190.3 Legend: 228

191 ECM-receptor interaction **229**
191.1 Human Pathway: HSA04512 229
191.2 Number of Hits: 14 229
191.3 Legend: 229

192 Fatty acid elongation **230**
192.1 Human Pathway: HSA00062 230
192.2 Number of Hits: 14 230
192.3 Legend: 231

193 Butanoate metabolism **231**
193.1 Human Pathway: HSA00650 231
193.2 Number of Hits: 13 231
193.3 Legend: 231

194 Tryptophan metabolism **232**
194.1 Human Pathway: HSA00380 232
194.2 Number of Hits: 13 232
194.3 Legend: 232

195 Terpenoid backbone biosynthesis **233**
195.1 Human Pathway: HSA00900 233
195.2 Number of Hits: 13 233
195.3 Legend: 234

196 RIG-I-like receptor signaling pathway **234**
196.1 Human Pathway: HSA04622 234
196.2 Number of Hits: 13 234
196.3 Legend: 234

197 Leishmaniasis **235**
197.1 Human Pathway: HSA05140 235
197.2 Number of Hits: 13 235
197.3 Legend: 235

198 Platinum drug resistance **236**
198.1 Human Pathway: HSA01524 236
198.2 Number of Hits: 13 236
198.3 Legend: 236

199 Olfactory transduction **237**
199.1 Human Pathway: HSA04740 237
199.2 Number of Hits: 13 237
199.3 Legend: 237

200 Viral myocarditis **238**
200.1 Human Pathway: HSA05416 238
200.2 Number of Hits: 13 238
200.3 Legend: 238

201 Pentose phosphate pathway **239**
201.1 Human Pathway: HSA00030 239
201.2 Number of Hits: 13 239
201.3 Legend: 239

202 Nicotinate and nicotinamide metabolism **240**
202.1 Human Pathway: HSA00760 240
202.2 Number of Hits: 12 240
202.3 Legend: 240

203 Notch signaling pathway **241**
203.1 Human Pathway: HSA04330 241
203.2 Number of Hits: 12 241
203.3 Legend: 241

204 Prion diseases **242**
204.1 Human Pathway: HSA05020 242
204.2 Number of Hits: 12 242
204.3 Legend: 242

205 Systemic lupus erythematosus **243**
205.1 Human Pathway: HSA05322 243
205.2 Number of Hits: 12 243
205.3 Legend: 243

206 Aldosterone-regulated sodium reabsorption **244**
206.1 Human Pathway: HSA04960 244
206.2 Number of Hits: 12 244
206.3 Legend: 244

207 Ether lipid metabolism **245**
207.1 Human Pathway: HSA00565 245
207.2 Number of Hits: 12 245
207.3 Legend: 245

208 One carbon pool by folate **246**
208.1 Human Pathway: HSA00670 246
208.2 Number of Hits: 12 246
208.3 Legend: 246

209 Circadian rhythm **247**
209.1 Human Pathway: HSA04710 247
209.2 Number of Hits: 12 247
209.3 Legend: 247

210 Porphyrin and chlorophyll metabolism **248**
210.1 Human Pathway: HSA00860 248
210.2 Number of Hits: 12 248
210.3 Legend: 248

211 Galactose metabolism **249**
211.1 Human Pathway: HSA00052 249
211.2 Number of Hits: 12 249
211.3 Legend: 249

212 Starch and sucrose metabolism **250**
212.1 Human Pathway: HSA00500 250
212.2 Number of Hits: 12 250
212.3 Legend: 250

213 Regulation of autophagy **251**
213.1 Human Pathway: HSA04140 251
213.2 Number of Hits: 12 251
213.3 Legend: 251

214 Base excision repair **252**
214.1 Human Pathway: HSA03410 252
214.2 Number of Hits: 11 252
214.3 Legend: 252

215 Biosynthesis of unsaturated fatty acids **253**
215.1 Human Pathway: HSA01040 253
215.2 Number of Hits: 11 253
215.3 Legend: 253

216 2-Oxocarboxylic acid metabolism **254**
216.1 Human Pathway: HSA01210 254
216.2 Number of Hits: 11 254
216.3 Legend: 254

217 Bladder cancer **255**
217.1 Human Pathway: HSA05219 255
217.2 Number of Hits: 11 255
217.3 Legend: 255

218 Collecting duct acid secretion **256**
218.1 Human Pathway: HSA04966 256
218.2 Number of Hits: 11 256
218.3 Legend: 257

219 Glutathione metabolism **257**
219.1 Human Pathway: HSA00480 257
219.2 Number of Hits: 11 257
219.3 Legend: 258

220 Homologous recombination **258**
220.1 Human Pathway: HSA03440 258
220.2 Number of Hits: 11 258
220.3 Legend: 258

221 Drug metabolism - other enzymes **259**
221.1 Human Pathway: HSA00983 259
221.2 Number of Hits: 11 259
221.3 Legend: 259

222 Taste transduction **260**
222.1 Human Pathway: HSA04742 260
222.2 Number of Hits: 10 260
222.3 Legend: 260

223 Antigen processing and presentation **261**
223.1 Human Pathway: HSA04612 261
223.2 Number of Hits: 10 261
223.3 Legend: 261

224 Fat digestion and absorption **262**
224.1 Human Pathway: HSA04975 262
224.2 Number of Hits: 10 262
224.3 Legend: 262

225 Carbohydrate digestion and absorption **263**
225.1 Human Pathway: HSA04973 263
225.2 Number of Hits: 10 263
225.3 Legend: 263

226 Arginine biosynthesis **264**
226.1 Human Pathway: HSA00220 264
226.2 Number of Hits: 10 264
226.3 Legend: 264

227 Bile secretion **265**
227.1 Human Pathway: HSA04976 265
227.2 Number of Hits: 10 265
227.3 Legend: 265

228 Thyroid cancer **266**
228.1 Human Pathway: HSA05216 266
228.2 Number of Hits: 10 266
228.3 Legend: 266

229 Arachidonic acid metabolism **267**
229.1 Human Pathway: HSA00590 267
229.2 Number of Hits: 10 267
229.3 Legend: 267

230 Pentose and glucuronate interconversions **268**
230.1 Human Pathway: HSA00040 268
230.2 Number of Hits: 10 268
230.3 Legend: 268

231 Mucin type O-Glycan biosynthesis **270**
231.1 Human Pathway: HSA00512 270
231.2 Number of Hits: 9 270
231.3 Legend: 271

232 Basal cell carcinoma **271**
232.1 Human Pathway: HSA05217 271
232.2 Number of Hits: 9 271
232.3 Legend: 271

233 Mineral absorption **272**
233.1 Human Pathway: HSA04978 272
233.2 Number of Hits: 9 272
233.3 Legend: 272

234 Proximal tubule bicarbonate reclamation **273**
234.1 Human Pathway: HSA04964 273
234.2 Number of Hits: 9 273
234.3 Legend: 274

235 Mismatch repair **274**
235.1 Human Pathway: HSA03430 274
235.2 Number of Hits: 8 274
235.3 Legend: 275

236 Hippo signaling pathway -multiple species **275**
236.1 Human Pathway: HSA04392 275
236.2 Number of Hits: 8 275
236.3 Legend: 275

237 Fanconi anemia pathway **276**
237.1 Human Pathway: HSA03460 276
237.2 Number of Hits: 8 276
237.3 Legend: 277

238 Apoptosis - multiple species **277**
238.1 Human Pathway: HSA04215 277
238.2 Number of Hits: 8 277
238.3 Legend: 278

239 Dorso-ventral axis formation **278**
239.1 Human Pathway: HSA04320 278
239.2 Number of Hits: 8 278
239.3 Legend: 278

240 Selenocompound metabolism **279**
240.1 Human Pathway: HSA00450 279
240.2 Number of Hits: 8 279
240.3 Legend: 279

241 Histidine metabolism **280**
241.1 Human Pathway: HSA00340 280
241.2 Number of Hits: 7 280
241.3 Legend: 280

242 Retinol metabolism **281**
242.1 Human Pathway: HSA00830 281
242.2 Number of Hits: 7 281
242.3 Legend: 281

243 Ovarian steroidogenesis **282**
243.1 Human Pathway: HSA04913 282
243.2 Number of Hits: 7 282
243.3 Legend: 282

244 Folate biosynthesis **283**
244.1 Human Pathway: HSA00790 283
244.2 Number of Hits: 7 283
244.3 Legend: 283

245 Pantothenate and CoA biosynthesis **284**
245.1 Human Pathway: HSA00770 284
245.2 Number of Hits: 7 284
245.3 Legend: 284

246 Malaria **285**
246.1 Human Pathway: HSA05144 285
246.2 Number of Hits: 6 285
246.3 Legend: 285

247 Glycosaminoglycan biosynthesis - keratan sulfate **286**
247.1 Human Pathway: HSA00533 286
247.2 Number of Hits: 6 286
247.3 Legend: 286

248 Tyrosine metabolism **287**
248.1 Human Pathway: HSA00350 287
248.2 Number of Hits: 6 287
248.3 Legend: 287

249 Synthesis and degradation of ketone bodies **288**
249.1 Human Pathway: HSA00072 288
249.2 Number of Hits: 6 288
249.3 Legend: 288

250 Glycosphingolipid biosynthesis - lacto and neolacto series **290**
250.1 Human Pathway: HSA00601 290
250.2 Number of Hits: 6 290
250.3 Legend: 291

251 African trypanosomiasis **291**
251.1 Human Pathway: HSA05143 291
251.2 Number of Hits: 6 291
251.3 Legend: 291

252 Glycosaminoglycan biosynthesis - heparan sulfate / heparin **292**
252.1 Human Pathway: HSA00534 292
252.2 Number of Hits: 6 292
252.3 Legend: 292

253 Ascorbate and aldarate metabolism **293**
253.1 Human Pathway: HSA00053 293
253.2 Number of Hits: 5 293
253.3 Legend: 293

254 Maturity onset diabetes of the young 294
254.1 Human Pathway: HSA04950 294
254.2 Number of Hits: 5 294
254.3 Legend: 295

255 Sulfur metabolism 295
255.1 Human Pathway: HSA00920 295
255.2 Number of Hits: 5 295
255.3 Legend: 296

256 Type I diabetes mellitus 296
256.1 Human Pathway: HSA04940 296
256.2 Number of Hits: 5 296
256.3 Legend: 297

257 Cytokine-cytokine receptor interaction 297
257.1 Human Pathway: HSA04060 297
257.2 Number of Hits: 5 297
257.3 Legend: 297

258 Taurine and hypotaurine metabolism 298
258.1 Human Pathway: HSA00430 298
258.2 Number of Hits: 4 298
258.3 Legend: 298

259 Fatty acid biosynthesis 299
259.1 Human Pathway: HSA00061 299
259.2 Number of Hits: 4 299
259.3 Legend: 299

260 Inflammatory bowel disease (IBD) 300
260.1 Human Pathway: HSA05321 300
260.2 Number of Hits: 4 300
260.3 Legend: 300

261 Renin-angiotensin system 301
261.1 Human Pathway: HSA04614 301
261.2 Number of Hits: 4 301
261.3 Legend: 301

262 Glycosaminoglycan degradation **302**
262.1 Human Pathway: HSA00531 302
262.2 Number of Hits: 4 302
262.3 Legend: 302

263 ABC transporters **303**
263.1 Human Pathway: HSA02010 303
263.2 Number of Hits: 4 303
263.3 Legend: 304

264 Nitrogen metabolism **304**
264.1 Human Pathway: HSA00910 304
264.2 Number of Hits: 4 304
264.3 Legend: 305

265 Primary bile acid biosynthesis **307**
265.1 Human Pathway: HSA00120 307
265.2 Number of Hits: 3 307
265.3 Legend: 308

266 Glycosylphosphatidylinositol(GPI)-anchor biosynthesis **309**
266.1 Human Pathway: HSA00563 309
266.2 Number of Hits: 3 309
266.3 Legend: 310

267 Phototransduction **310**
267.1 Human Pathway: HSA04744 310
267.2 Number of Hits: 3 310
267.3 Legend: 311

268 alpha-Linolenic acid metabolism **311**
268.1 Human Pathway: HSA00592 311
268.2 Number of Hits: 3 311
268.3 Legend: 312

269 Other glycan degradation **312**
269.1 Human Pathway: HSA00511 312
269.2 Number of Hits: 3 312
269.3 Legend: 312

270 Sulfur relay system **313**
270.1 Human Pathway: HSA04122 313
270.2 Number of Hits: 3 313
270.3 Legend: 313

271 Glycosphingolipid biosynthesis - ganglio series **314**
271.1 Human Pathway: HSA00604 314
271.2 Number of Hits: 3 314
271.3 Legend: 315

272 Valine, leucine and isoleucine biosynthesis **315**
272.1 Human Pathway: HSA00290 315
272.2 Number of Hits: 2 315
272.3 Legend: 316

273 Ubiquinone and other terpenoid-quinone biosynthesis **317**
273.1 Human Pathway: HSA00130 317
273.2 Number of Hits: 2 317
273.3 Legend: 318

274 Metabolism of xenobiotics by cytochrome P450 **319**
274.1 Human Pathway: HSA00980 319
274.2 Number of Hits: 2 319
274.3 Legend: 319

275 Lysine biosynthesis **320**
275.1 Human Pathway: HSA00300 320
275.2 Number of Hits: 2 320
275.3 Legend: 320

276 Linoleic acid metabolism **321**
276.1 Human Pathway: HSA00591 321
276.2 Number of Hits: 2 321
276.3 Legend: 321

277 Glycosphingolipid biosynthesis - globo series **322**
277.1 Human Pathway: HSA00603 322
277.2 Number of Hits: 2 322
277.3 Legend: 323

278 Butirosin and neomycin biosynthesis **323**
278.1 Human Pathway: HSA00524 323
278.2 Number of Hits: 2 323
278.3 Legend: 323

279 Vitamin B6 metabolism **324**
279.1 Human Pathway: HSA00750 324
279.2 Number of Hits: 2 324
279.3 Legend: 324

280 Non-homologous end-joining **325**
280.1 Human Pathway: HSA03450 325
280.2 Number of Hits: 2 325
280.3 Legend: 325

281 Vitamin digestion and absorption **326**
281.1 Human Pathway: HSA04977 326
281.2 Number of Hits: 2 326
281.3 Legend: 326

282 Primary immunodeficiency **327**
282.1 Human Pathway: HSA05340 327
282.2 Number of Hits: 2 327
282.3 Legend: 327

283 Thiamine metabolism **328**
283.1 Human Pathway: HSA00730 328
283.2 Number of Hits: 2 328
283.3 Legend: 328

284 Glycosaminoglycan biosynthesis - chondroitin sulfate / dermatan sulfate **329**
284.1 Human Pathway: HSA00532 329
284.2 Number of Hits: 2 329
284.3 Legend: 329

285 Chemical carcinogenesis **331**
285.1 Human Pathway: HSA05204 331
285.2 Number of Hits: 2 331
285.3 Legend: 332

286 D-Glutamine and D-glutamate metabolism 332
286.1 Human Pathway: HSA00471 332
286.2 Number of Hits: 2 332
286.3 Legend: 332

287 Phenylalanine, tyrosine and tryptophan biosynthesis 333
287.1 Human Pathway: HSA00400 333
287.2 Number of Hits: 1 333
287.3 Legend: 333

288 Intestinal immune network for IgA production 334
288.1 Human Pathway: HSA04672 334
288.2 Number of Hits: 1 334
288.3 Legend: 334

289 Steroid hormone biosynthesis 335
289.1 Human Pathway: HSA00140 335
289.2 Number of Hits: 1 335
289.3 Legend: 335

290 Phenylalanine metabolism 336
290.1 Human Pathway: HSA00360 336
290.2 Number of Hits: 1 336
290.3 Legend: 336

291 Hematopoietic cell lineage 337
291.1 Human Pathway: HSA04640 337
291.2 Number of Hits: 1 337
291.3 Legend: 338

292 Lipoic acid metabolism 338
292.1 Human Pathway: HSA00785 338
292.2 Number of Hits: 1 338
292.3 Legend: 338

1 Pathways in cancer

1.1 Human Pathway: HSA05200

1.2 Number of Hits: 113

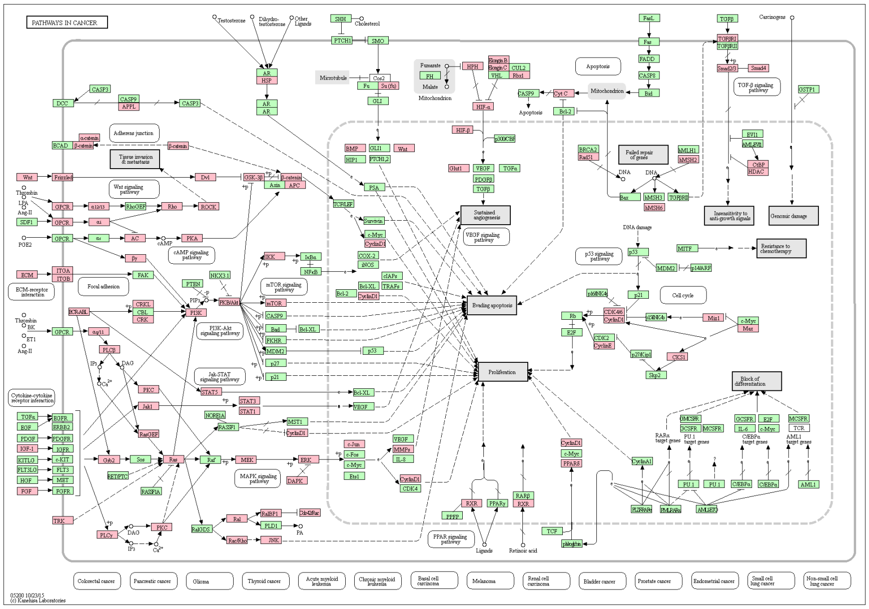

1.3 Legend:

RBH-Blast at 60% Identity + 50% Coverage
Green = Hit in *H. sapiens*
Red = Hit in *H. sapiens* and *T. californica*
White = Not in *H. sapiens*

2 Endocytosis

2.1 Human Pathway: HSA04144

2.2 Number of Hits: 108

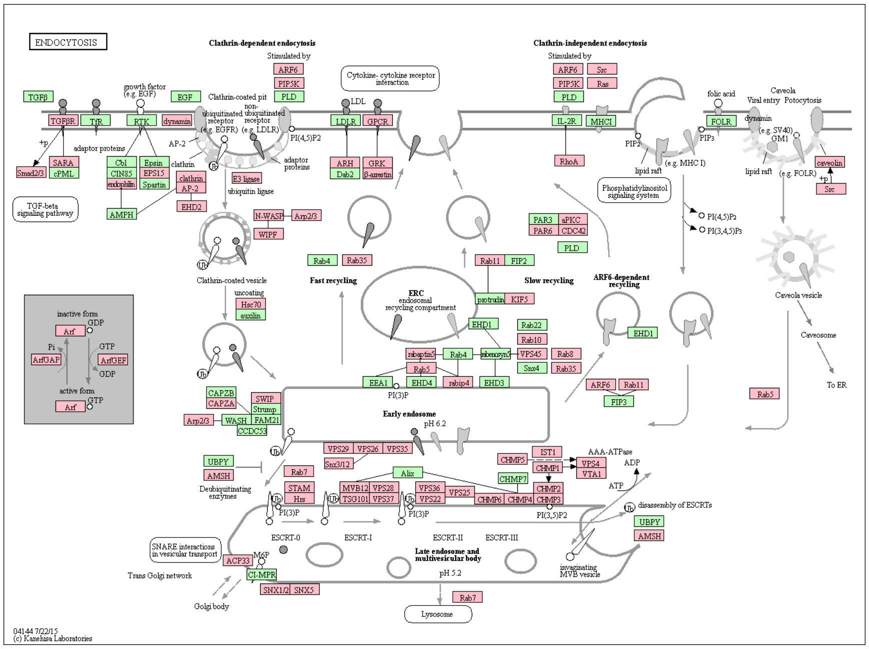

2.3 Legend:

RBH-Blast at 60% Identity + 50% Coverage
Green = Hit in *H. sapiens*
Red = Hit in *H. sapiens* and *T. californica*
White = Not in *H. sapiens*

3 MAPK signaling pathway

3.1 Human Pathway: HSA04010

3.2 Number of Hits: 90

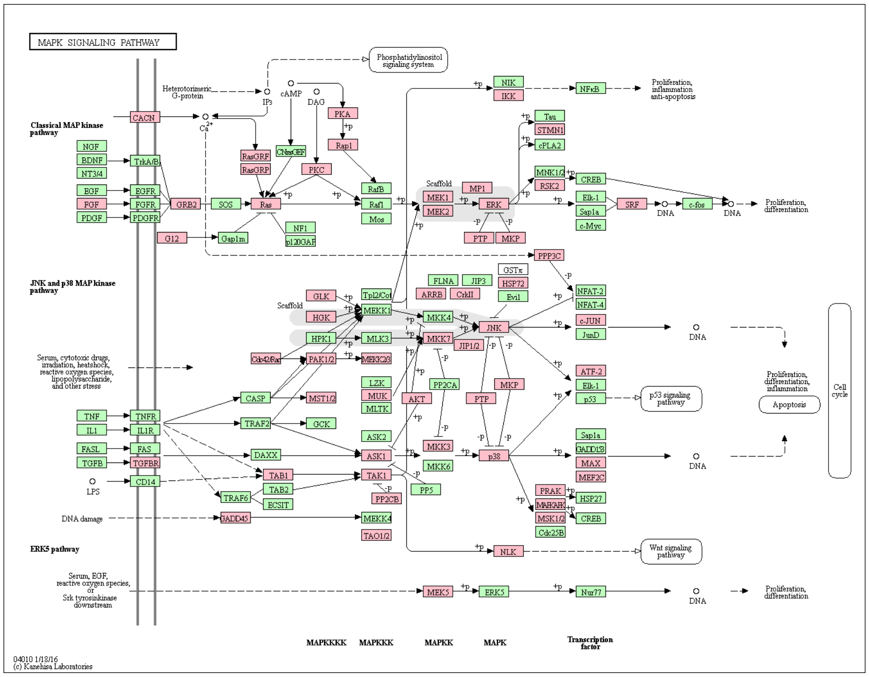

3.3 Legend:

RBH-Blast at 60% Identity + 50% Coverage
Green = Hit in *H. sapiens*
Red = Hit in *H. sapiens* and *T. californica*
White = Not in *H. sapiens*

4 PI3K-Akt signaling pathway

4.1 Human Pathway: HSA04151

4.2 Number of Hits: 86

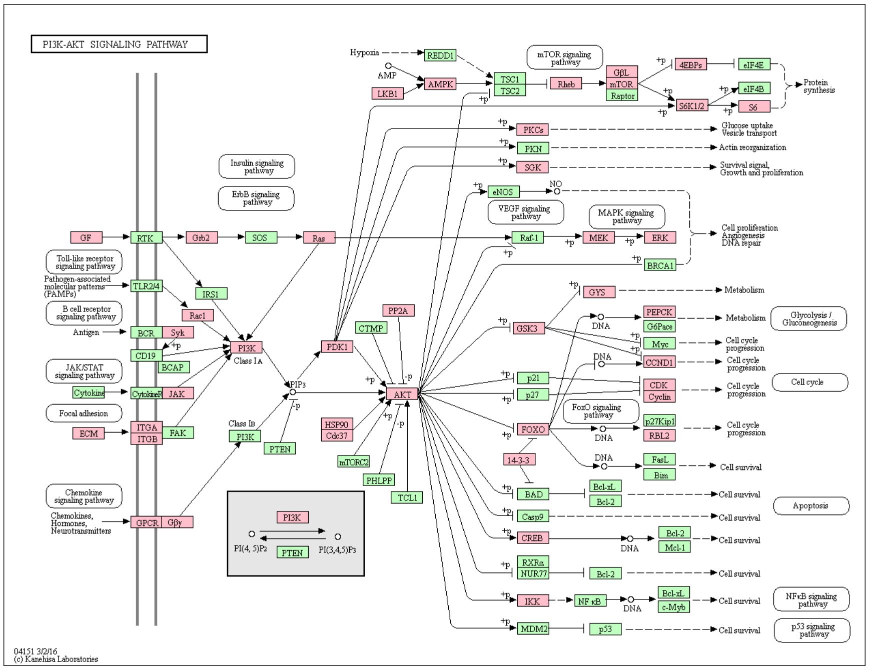

4.3 Legend:

RBH-Blast at 60% Identity + 50% Coverage
Green = Hit in *H. sapiens*
Red = Hit in *H. sapiens* and *T. californica*
White = Not in *H. sapiens*

5 Protein processing in endoplasmic reticulum

5.1 Human Pathway: HSA04141

5.2 Number of Hits: 83

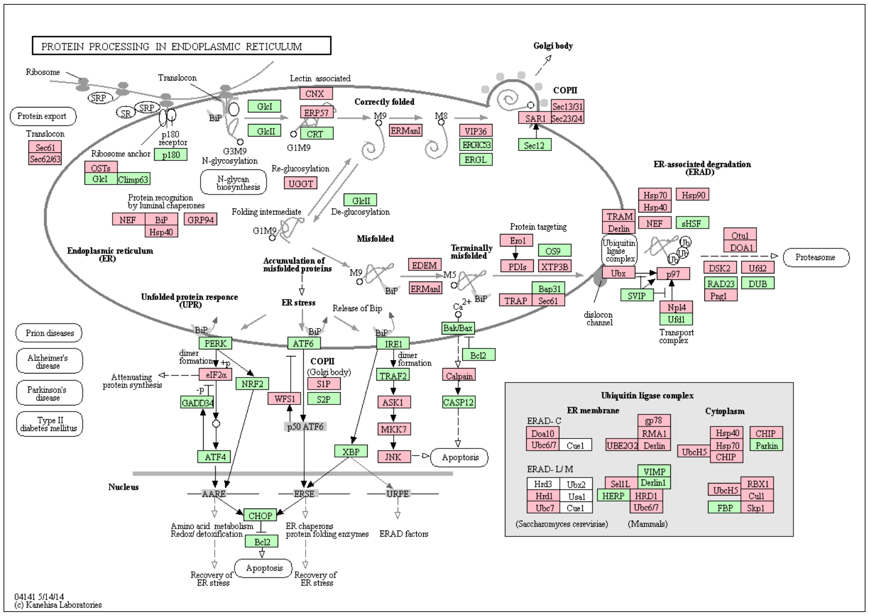

5.3 Legend:

RBH-Blast at 60% Identity + 50% Coverage
Green = Hit in *H. sapiens*
Red = Hit in *H. sapiens* and *T. californica*
White = Not in *H. sapiens*

6 Ribosome

6.1 Human Pathway: HSA03010

6.2 Number of Hits: 80

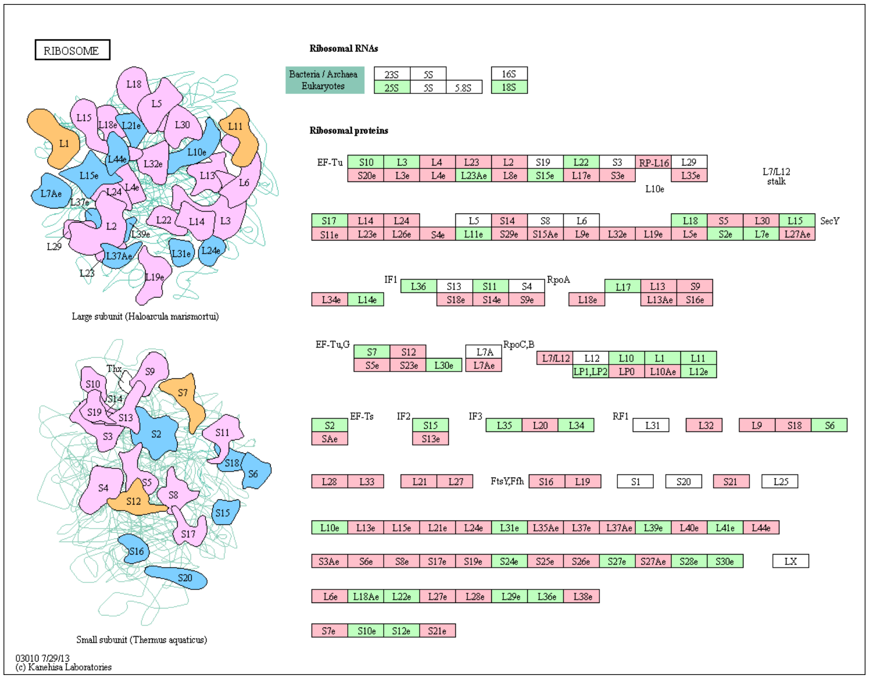

6.3 Legend:

RBH-Blast at 60% Identity + 50% Coverage
Green = Hit in *H. sapiens*
Red = Hit in *H. sapiens* and *T. californica*
White = Not in *H. sapiens*

7 Epstein-Barr virus infection

7.1 Human Pathway: HSA05169

7.2 Number of Hits: 76

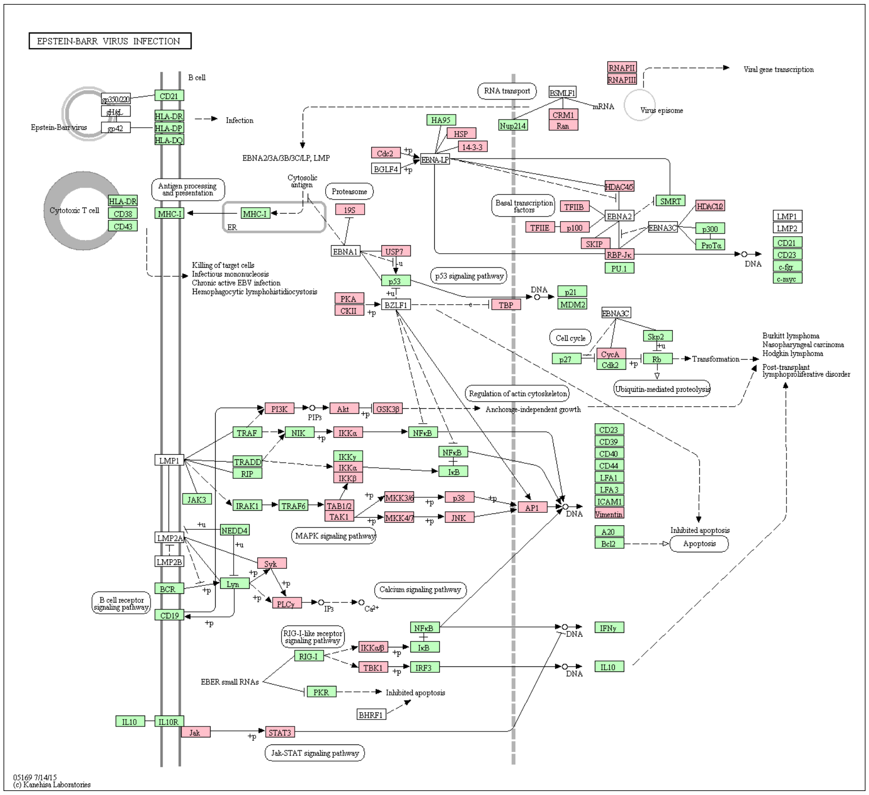

7.3 Legend:

RBH-Blast at 60% Identity + 50% Coverage
Green = Hit in *H. sapiens*
Red = Hit in *H. sapiens* and *T. californica*
White = Not in *H. sapiens*

8 Huntington's disease

8.1 Human Pathway: HSA05016

8.2 Number of Hits: 75

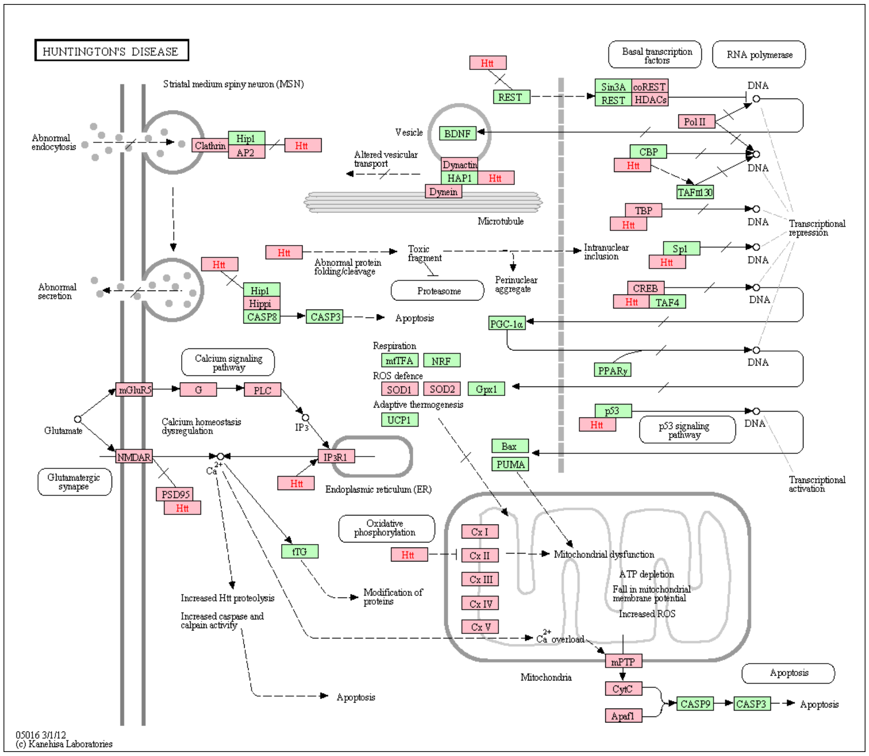

8.3 Legend:

RBH-Blast at 60% Identity + 50% Coverage
Green = Hit in *H. sapiens*
Red = Hit in *H. sapiens* and *T. californica*
White = Not in *H. sapiens*

9 Ubiquitin mediated proteolysis

9.1 Human Pathway: HSA04120

9.2 Number of Hits: 73

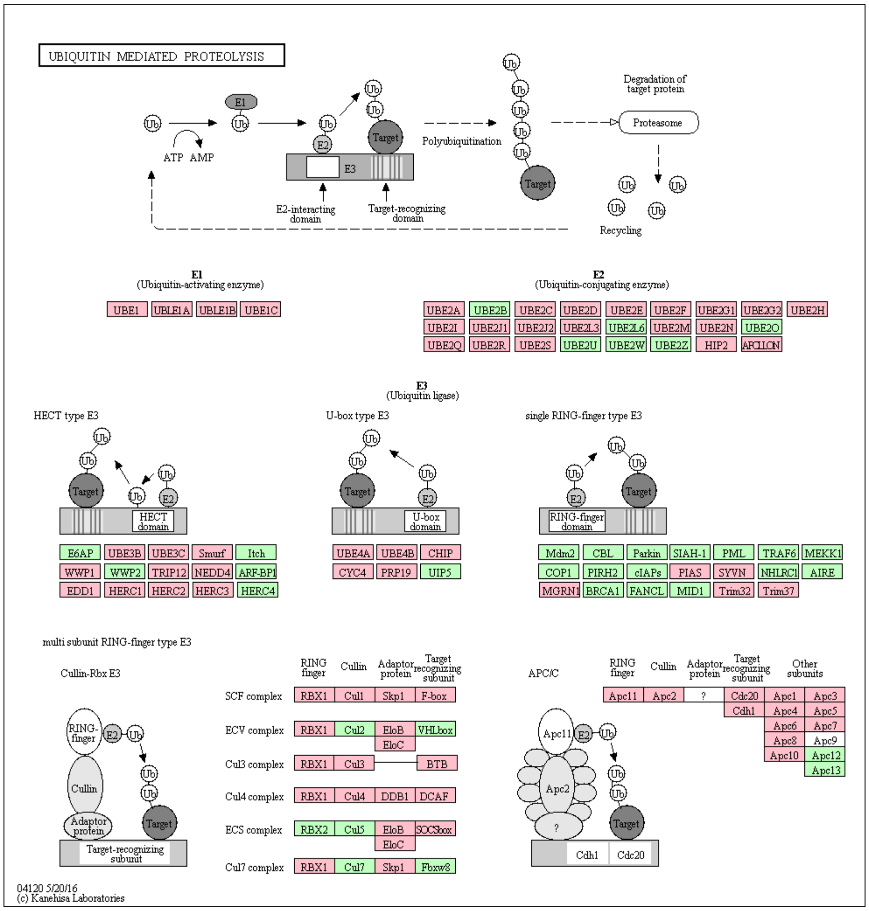

9.3 Legend:

RBH-Blast at 60% Identity + 50% Coverage
Green = Hit in *H. sapiens*
Red = Hit in *H. sapiens* and *T. californica*
White = Not in *H. sapiens*

10 Regulation of actin cytoskeleton

10.1 Human Pathway: HSA04810

10.2 Number of Hits: 72

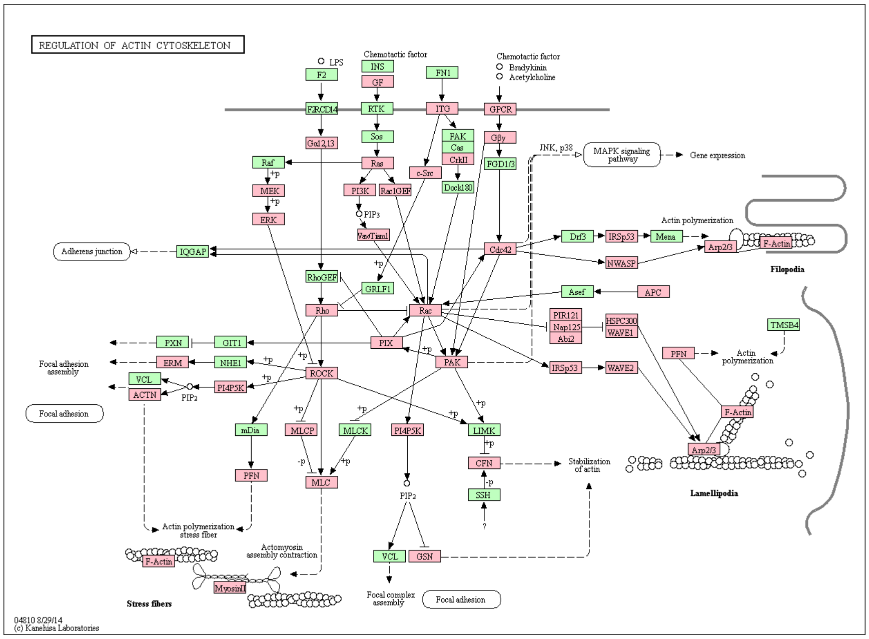

10.3 Legend:

RBH-Blast at 60% Identity + 50% Coverage
Green = Hit in *H. sapiens*
Red = Hit in *H. sapiens* and *T. californica*
White = Not in *H. sapiens*

11 HTLV-I infection

11.1 Human Pathway: HSA05166

11.2 Number of Hits: 72

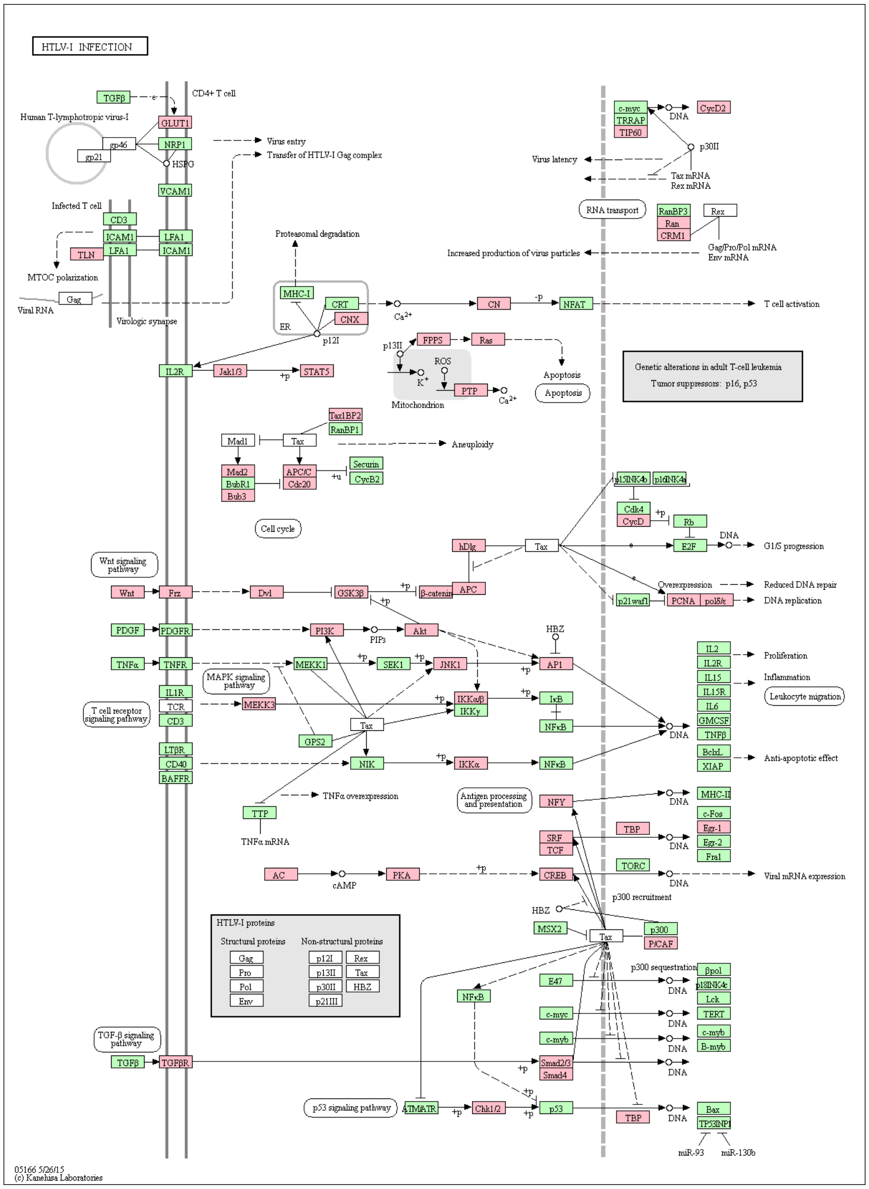

11.3 Legend:

RBH-Blast at 60% Identity + 50% Coverage
Green = Hit in *H. sapiens*
Red = Hit in *H. sapiens* and *T. californica*
White = Not in *H. sapiens*

---

12 Purine metabolism

12.1 Human Pathway: HSA00230

12.2 Number of Hits: 71

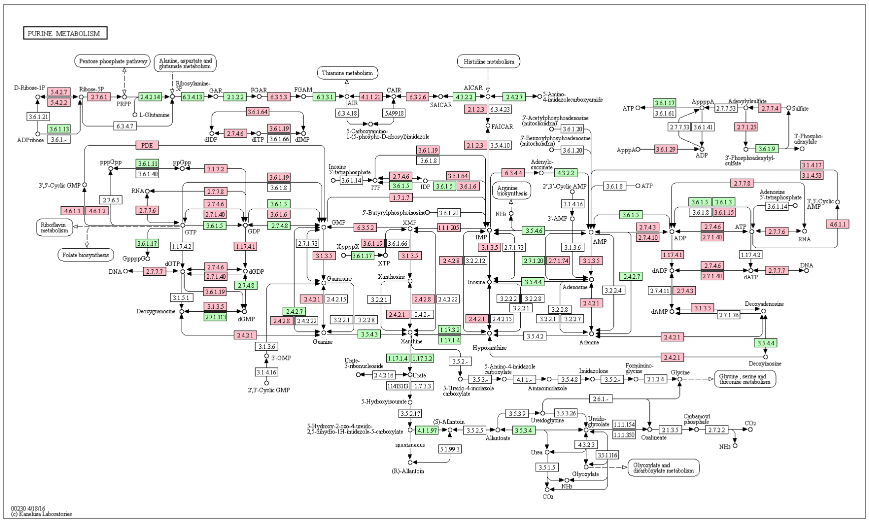

12.3 Legend:

RBH-Blast at 60% Identity + 50% Coverage
Green = Hit in *H. sapiens*
Red = Hit in *H. sapiens* and *T. californica*
White = Not in *H. sapiens*

---

13 Spliceosome

13.1 Human Pathway: HSA03040

13.2 Number of Hits: 71

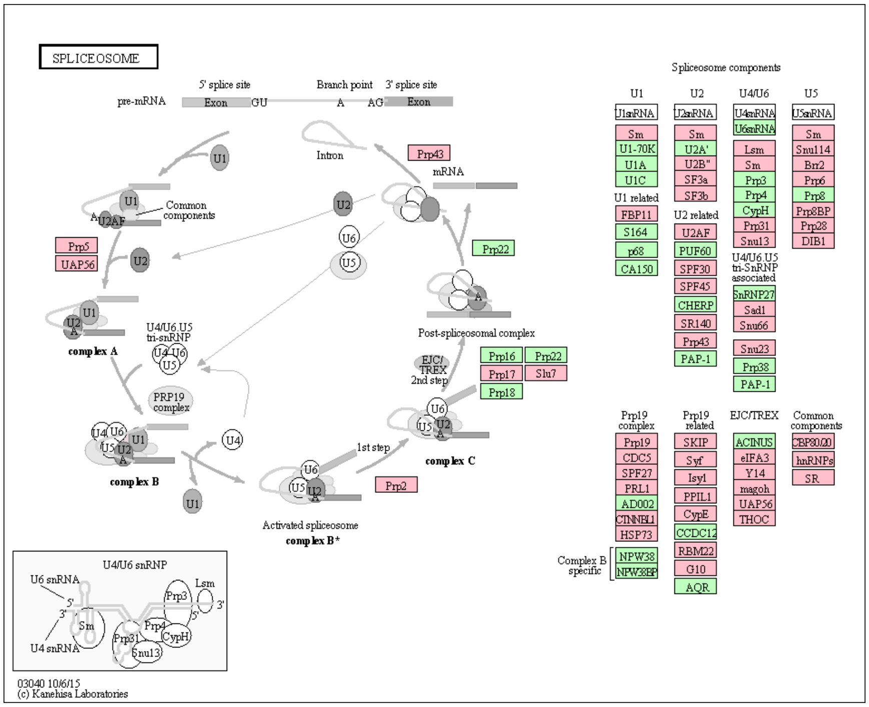

13.3 Legend:

RBH-Blast at 60% Identity + 50% Coverage
Green = Hit in *H. sapiens*
Red = Hit in *H. sapiens* and *T. californica*
White = Not in *H. sapiens*

14 Ras signaling pathway

14.1 Human Pathway: HSA04014

14.2 Number of Hits: 70

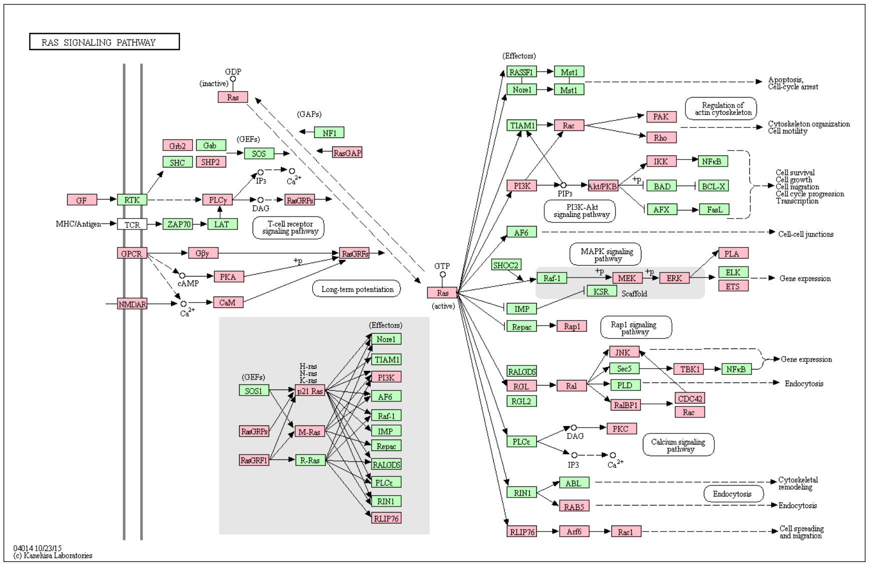

14.3 Legend:

RBH-Blast at 60% Identity + 50% Coverage
Green = Hit in *H. sapiens*
Red = Hit in *H. sapiens* and *T. californica*
White = Not in *H. sapiens*

15 RNA transport

15.1 Human Pathway: HSA03013

15.2 Number of Hits: 68

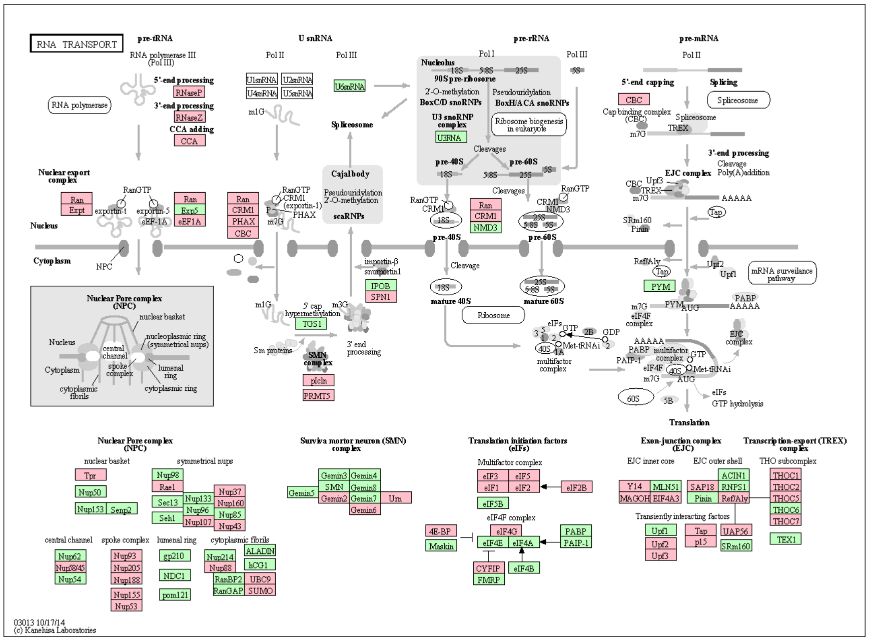

15.3 Legend:

RBH-Blast at 60% Identity + 50% Coverage
Green = Hit in *H. sapiens*
Red = Hit in *H. sapiens* and *T. californica*
White = Not in *H. sapiens*

16 Focal adhesion

16.1 Human Pathway: HSA04510

16.2 Number of Hits: 66

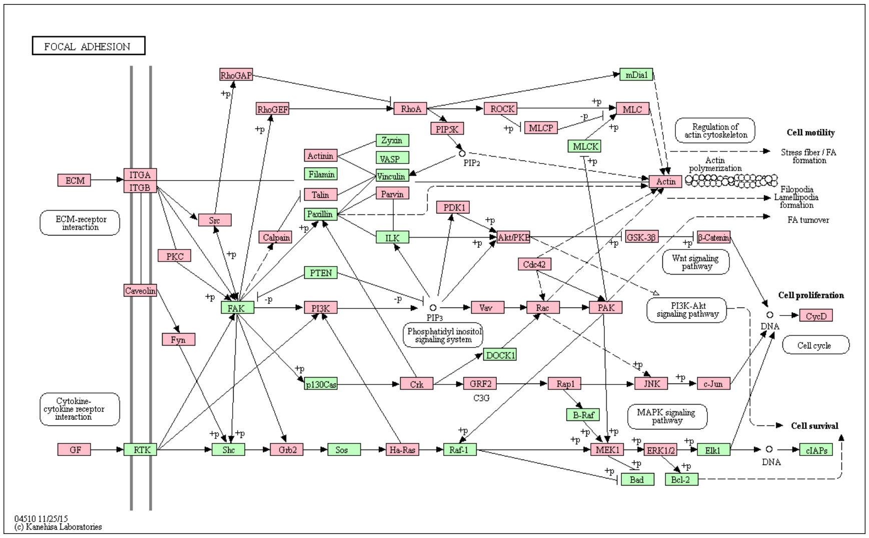

16.3 Legend:

RBH-Blast at 60% Identity + 50% Coverage
Green = Hit in *H. sapiens*
Red = Hit in *H. sapiens* and *T. californica*
White = Not in *H. sapiens*

17 Alzheimer's disease

17.1 Human Pathway: HSA05010

17.2 Number of Hits: 65

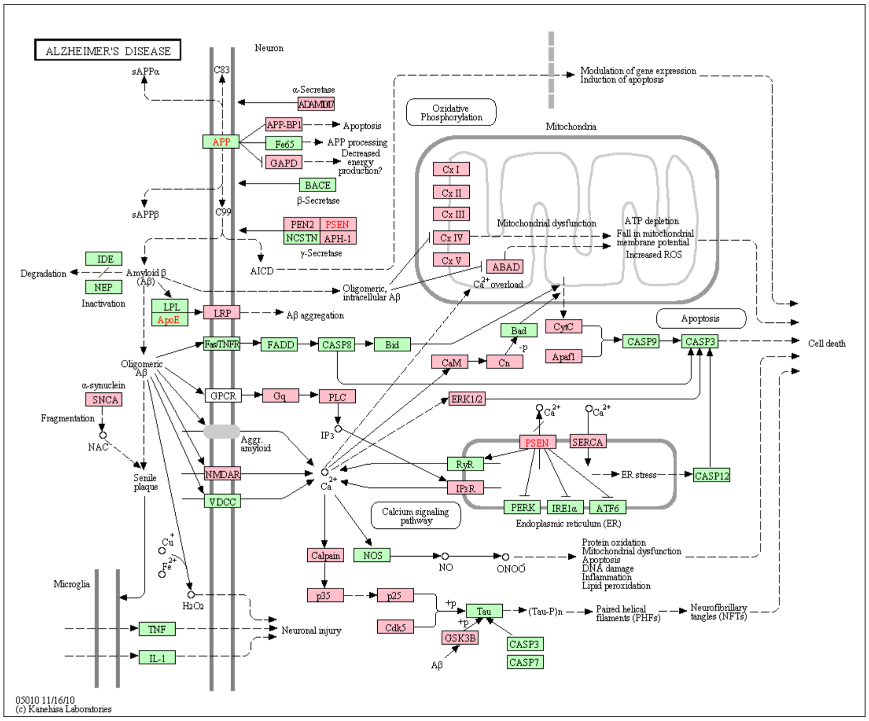

17.3 Legend:

RBH-Blast at 60% Identity + 50% Coverage
Green = Hit in *H. sapiens*
Red = Hit in *H. sapiens* and *T. californica*
White = Not in *H. sapiens*

18 Rap1 signaling pathway

18.1 Human Pathway: HSA04015

18.2 Number of Hits: 64

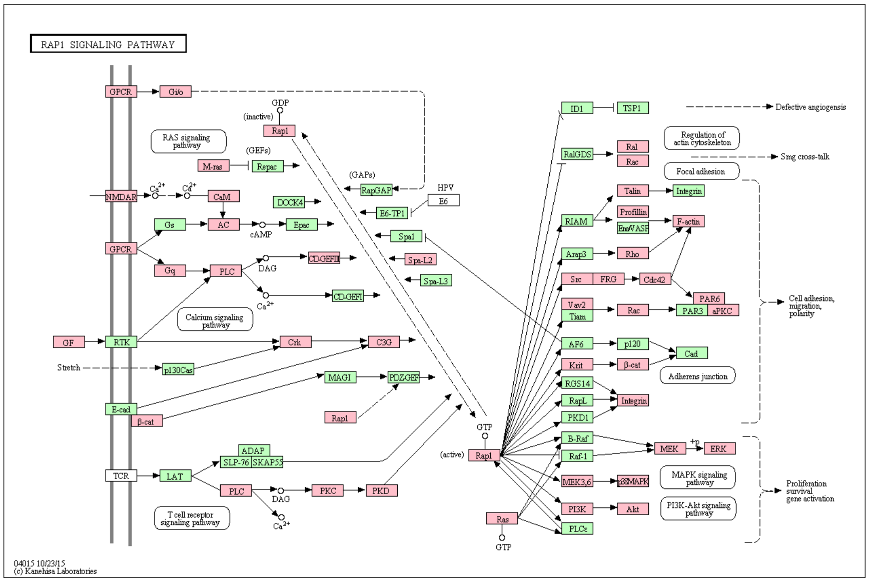

18.3 Legend:

RBH-Blast at 60% Identity + 50% Coverage
Green = Hit in *H. sapiens*
Red = Hit in *H. sapiens* and *T. californica*
White = Not in *H. sapiens*

19 Viral carcinogenesis

19.1 Human Pathway: HSA05203

19.2 Number of Hits: 61

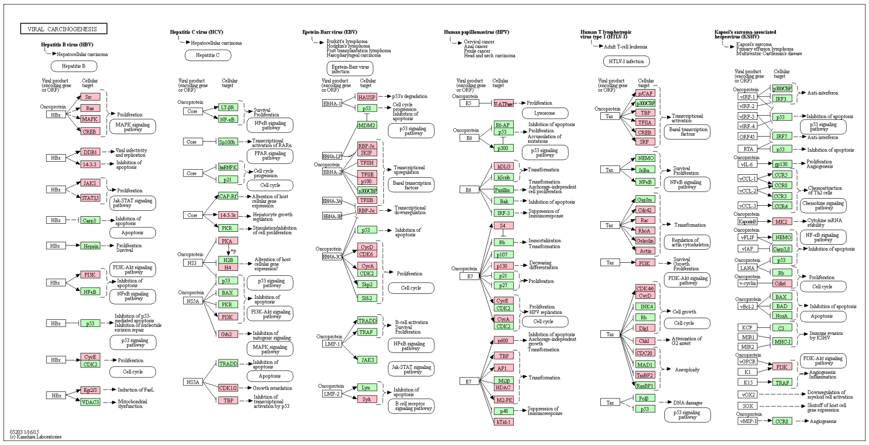

19.3 Legend:

RBH-Blast at 60% Identity + 50% Coverage
Green = Hit in *H. sapiens*
Red = Hit in *H. sapiens* and *T. californica*
White = Not in *H. sapiens*

20 Proteoglycans in cancer

20.1 Human Pathway: HSA05205

20.2 Number of Hits: 60

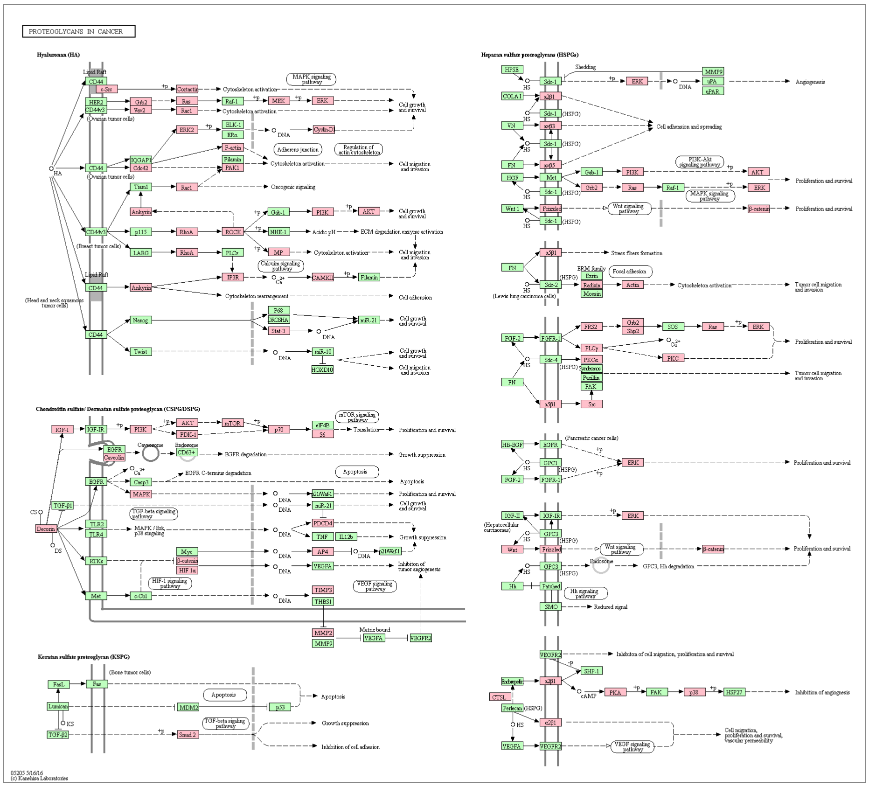

20.3 Legend:

RBH-Blast at 60% Identity + 50% Coverage
Green = Hit in *H. sapiens*
Red = Hit in *H. sapiens* and *T. californica*
White = Not in *H. sapiens*

21 mTOR signaling pathway

21.1 Human Pathway: HSA04150

21.2 Number of Hits: 60

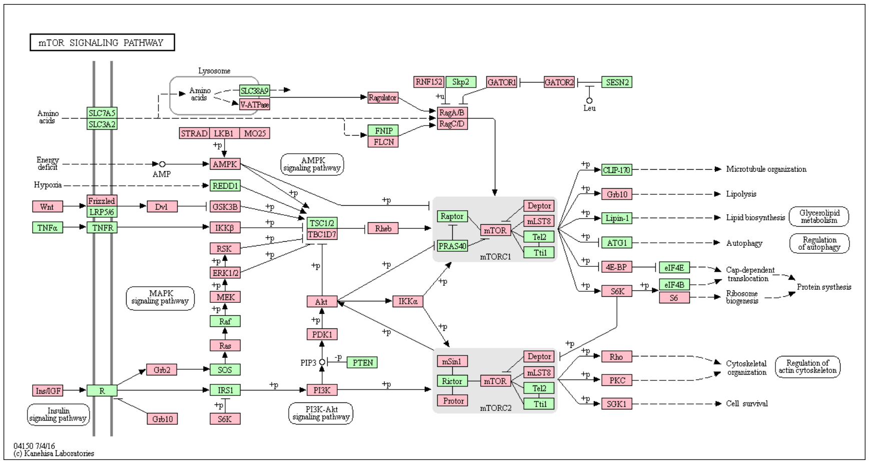

21.3 Legend:

RBH-Blast at 60% Identity + 50% Coverage
Green = Hit in *H. sapiens*
Red = Hit in *H. sapiens* and *T. californica*
White = Not in *H. sapiens*

22 Carbon metabolism

22.1 Human Pathway: HSA01200

22.2 Number of Hits: 60

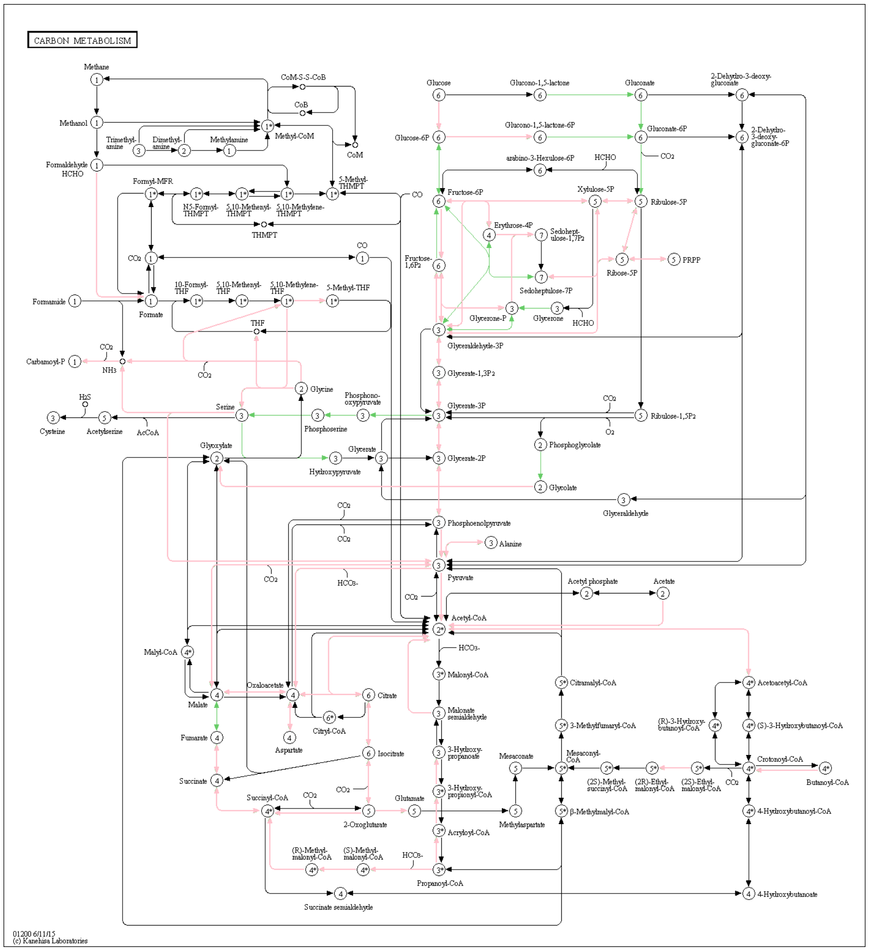

22.3 Legend:

RBH-Blast at 60% Identity + 50% Coverage
Green = Hit in *H. sapiens*
Red = Hit in *H. sapiens* and *T. californica*
White = Not in *H. sapiens*

23 Parkinson's disease

23.1 Human Pathway: HSA05012

23.2 Number of Hits: 58

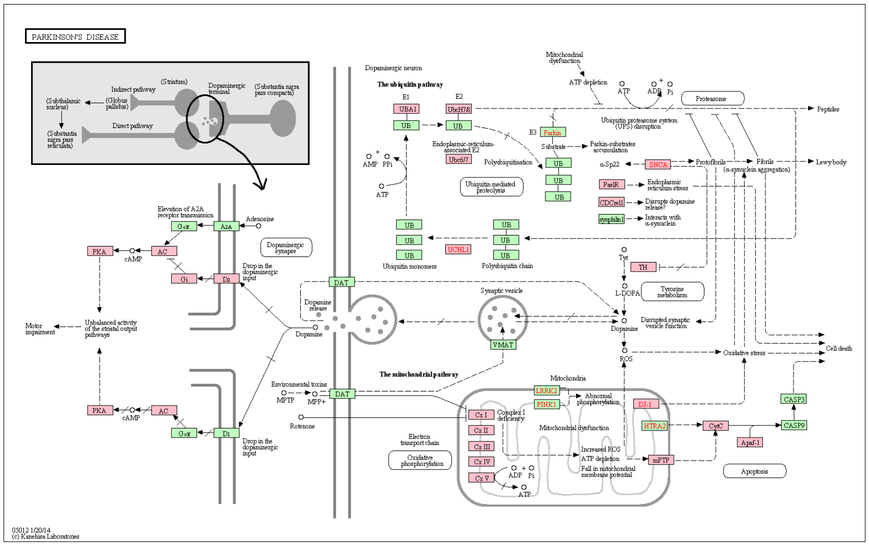

23.3 Legend:

RBH-Blast at 60% Identity + 50% Coverage
Green = Hit in *H. sapiens*
Red = Hit in *H. sapiens* and *T. californica*
White = Not in *H. sapiens*

24 Non-alcoholic fatty liver disease (NAFLD)

24.1 Human Pathway: HSA04932

24.2 Number of Hits: 57

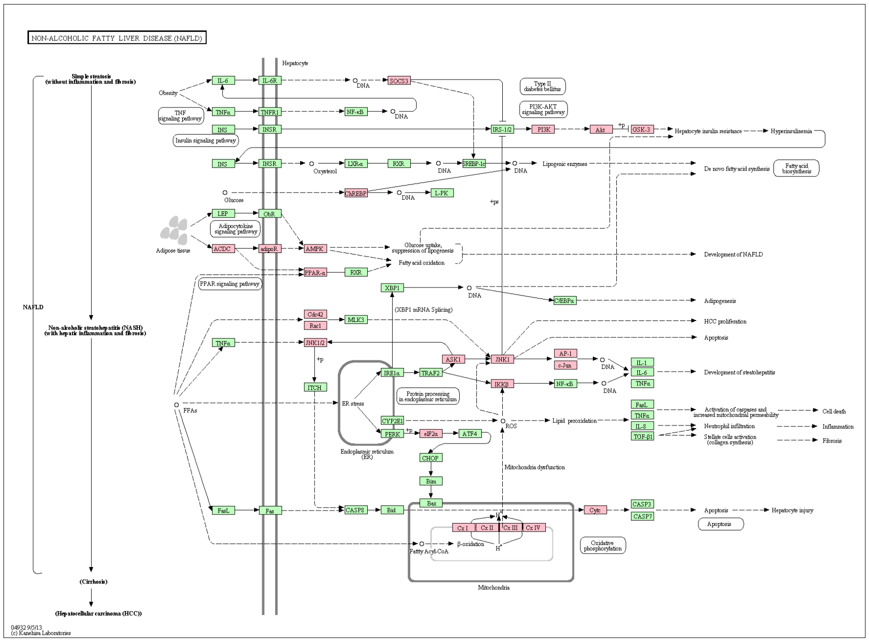

24.3 Legend:

RBH-Blast at 60% Identity + 50% Coverage
Green = Hit in *H. sapiens*
Red = Hit in *H. sapiens* and *T. californica*
White = Not in *H. sapiens*

25 Chemokine signaling pathway

25.1 Human Pathway: HSA04062

25.2 Number of Hits: 56

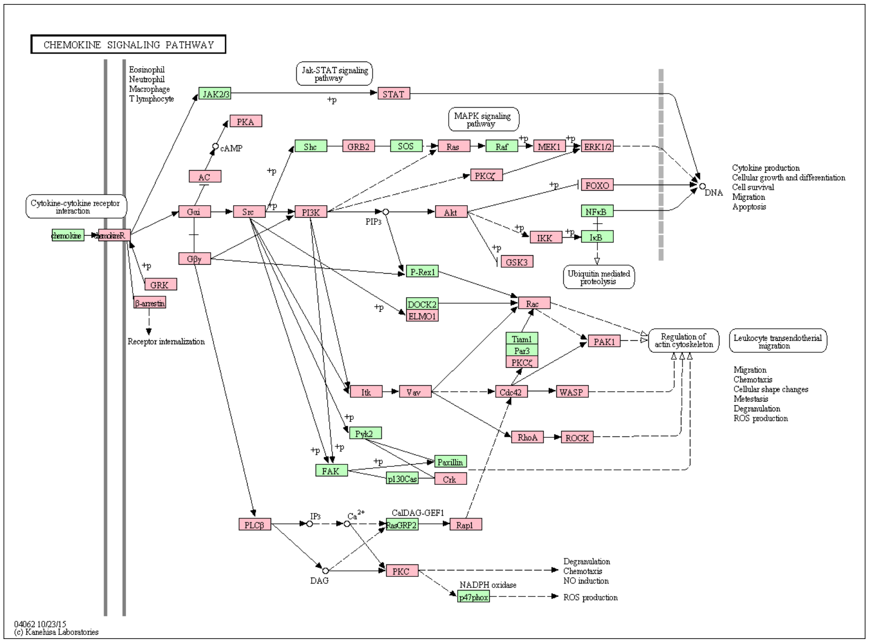

25.3 Legend:

RBH-Blast at 60% Identity + 50% Coverage
Green = Hit in *H. sapiens*
Red = Hit in *H. sapiens* and *T. californica*
White = Not in *H. sapiens*

26 Dopaminergic synapse

26.1 Human Pathway: HSA04728

26.2 Number of Hits: 56

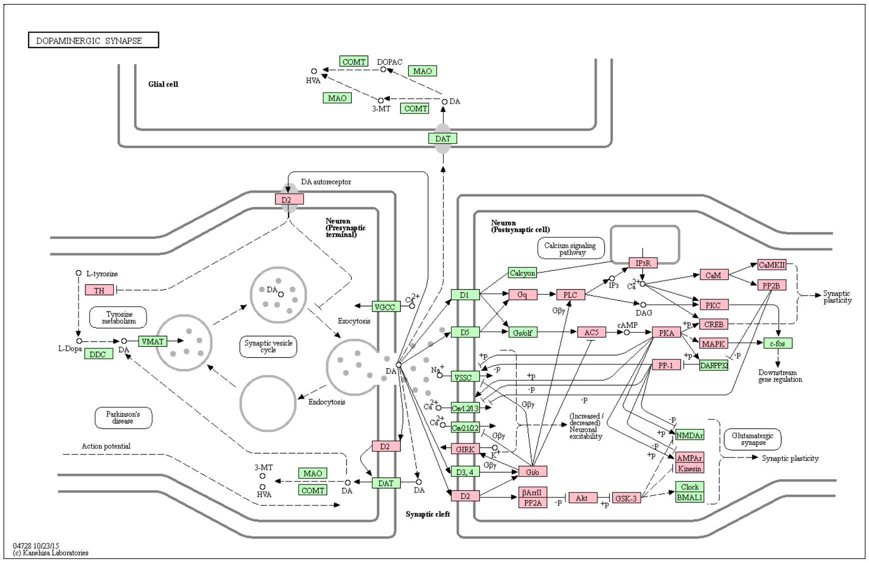

26.3 Legend:

RBH-Blast at 60% Identity + 50% Coverage
Green = Hit in *H. sapiens*
Red = Hit in *H. sapiens* and *T. californica*
White = Not in *H. sapiens*

27 Oxytocin signaling pathway

27.1 Human Pathway: HSA04921

27.2 Number of Hits: 56

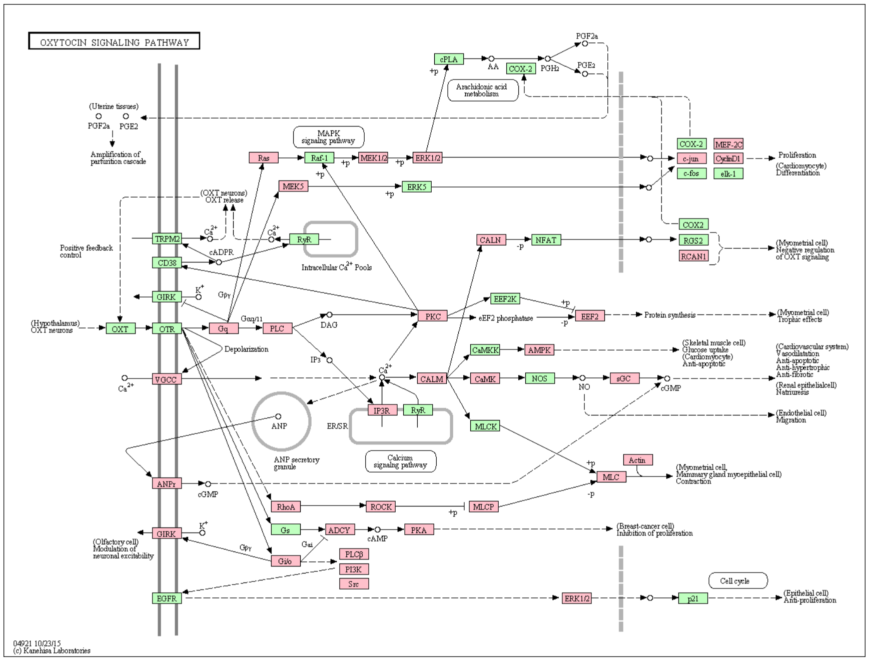

27.3 Legend:

RBH-Blast at 60% Identity + 50% Coverage
Green = Hit in *H. sapiens*
Red = Hit in *H. sapiens* and *T. californica*
White = Not in *H. sapiens*

28 Oxidative phosphorylation

28.1 Human Pathway: HSA00190

28.2 Number of Hits: 56

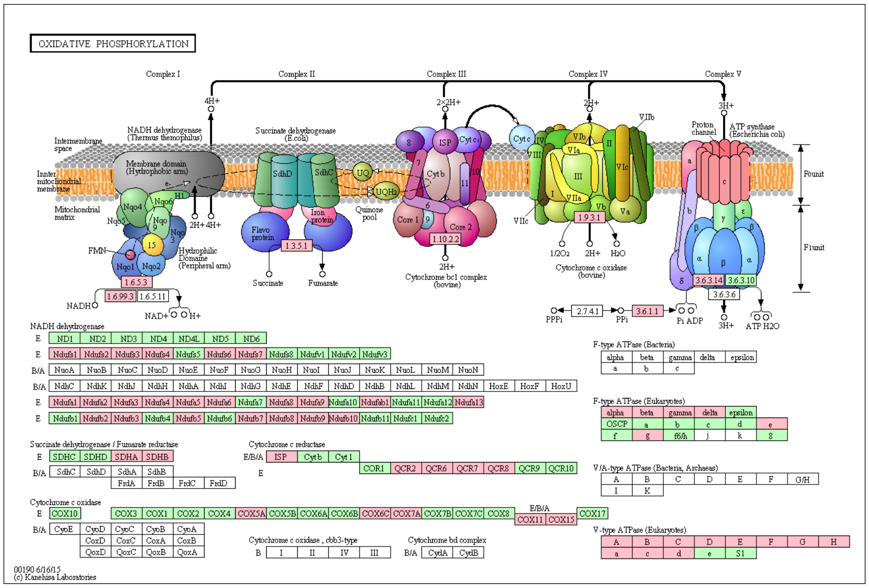

28.3 Legend:

RBH-Blast at 60% Identity + 50% Coverage
Green = Hit in *H. sapiens*
Red = Hit in *H. sapiens* and *T. californica*
White = Not in *H. sapiens*

29 cAMP signaling pathway

29.1 Human Pathway: HSA04024

29.2 Number of Hits: 54

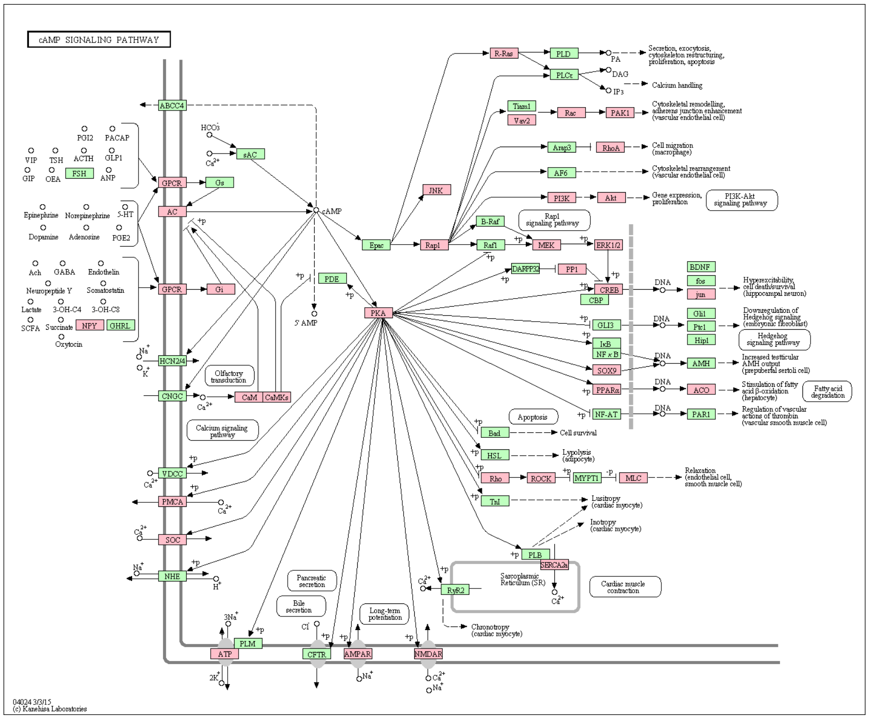

29.3 Legend:

RBH-Blast at 60% Identity + 50% Coverage
Green = Hit in *H. sapiens*
Red = Hit in *H. sapiens* and *T. californica*
White = Not in *H. sapiens*

30 Axon guidance

30.1 Human Pathway: HSA04360

30.2 Number of Hits: 53

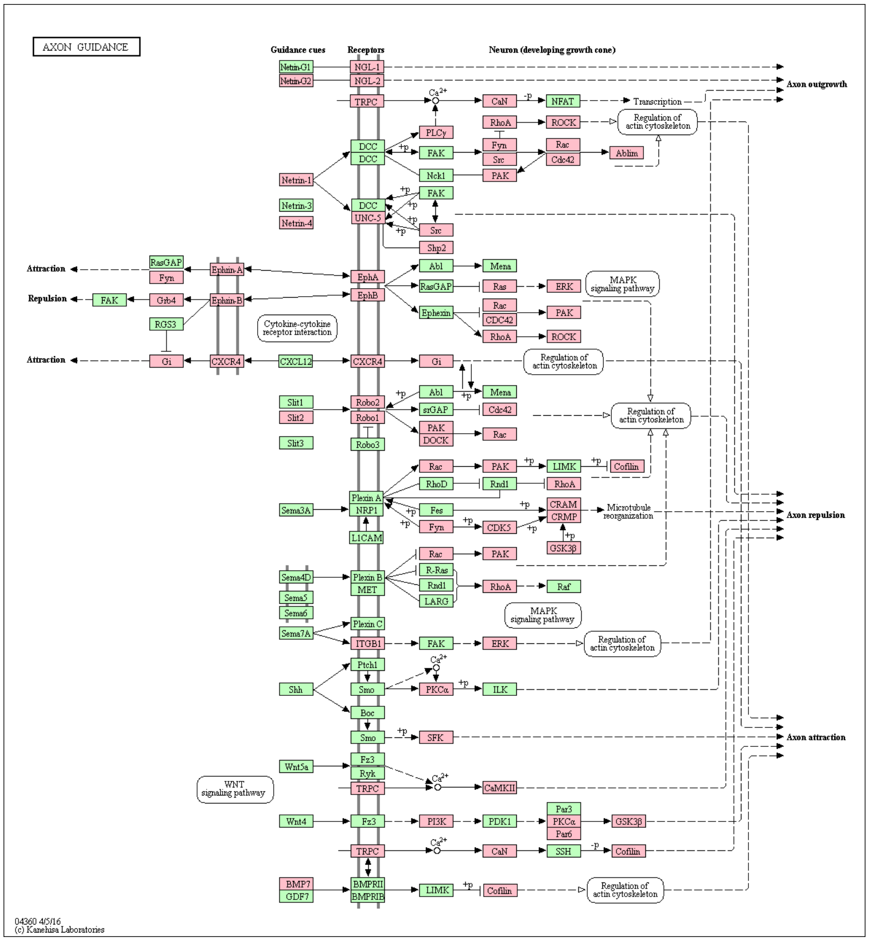

30.3 Legend:

RBH-Blast at 60% Identity + 50% Coverage
Green = Hit in *H. sapiens*
Red = Hit in *H. sapiens* and *T. californica*
White = Not in *H. sapiens*

31 Adrenergic signaling in cardiomyocytes

31.1 Human Pathway: HSA04261

31.2 Number of Hits: 52

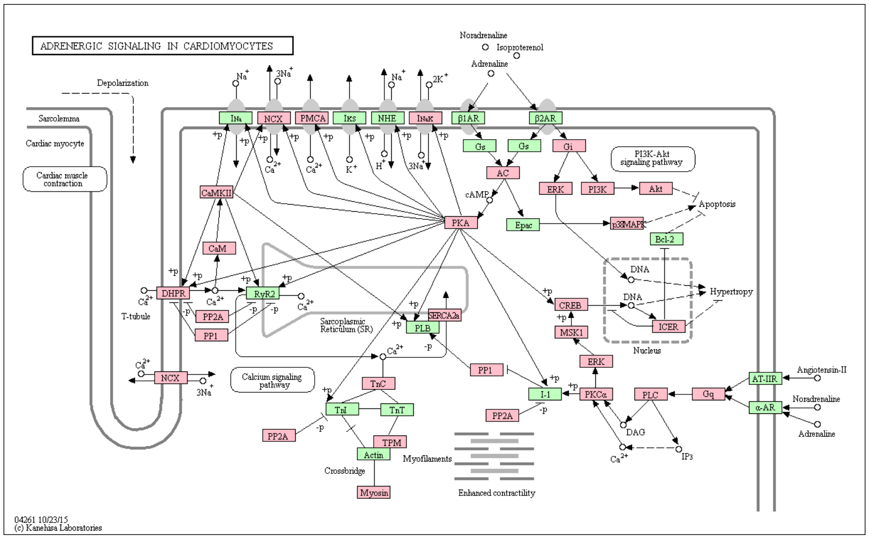

31.3 Legend:

RBH-Blast at 60% Identity + 50% Coverage
Green = Hit in *H. sapiens*
Red = Hit in *H. sapiens* and *T. californica*
White = Not in *H. sapiens*

32 FoxO signaling pathway

32.1 Human Pathway: HSA04068

32.2 Number of Hits: 51

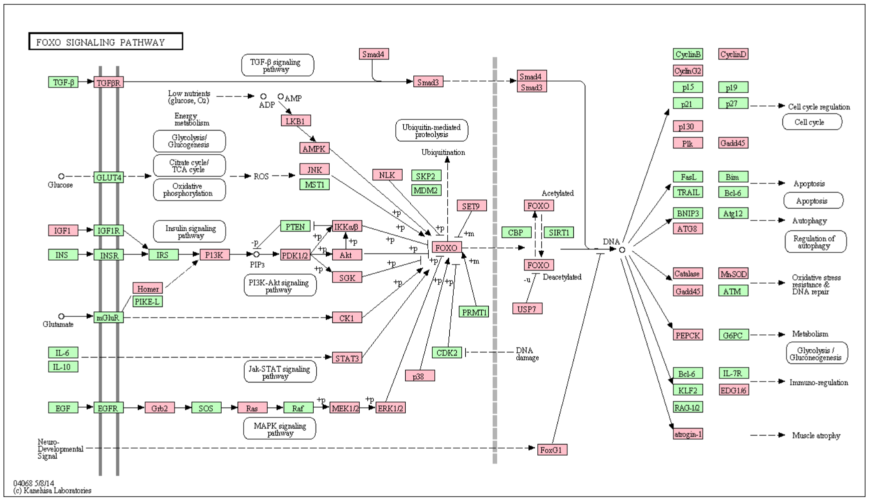

32.3 Legend:

RBH-Blast at 60% Identity + 50% Coverage
Green = Hit in *H. sapiens*
Red = Hit in *H. sapiens* and *T. californica*
White = Not in *H. sapiens*

33 Glutamatergic synapse

33.1 Human Pathway: HSA04724

33.2 Number of Hits: 50

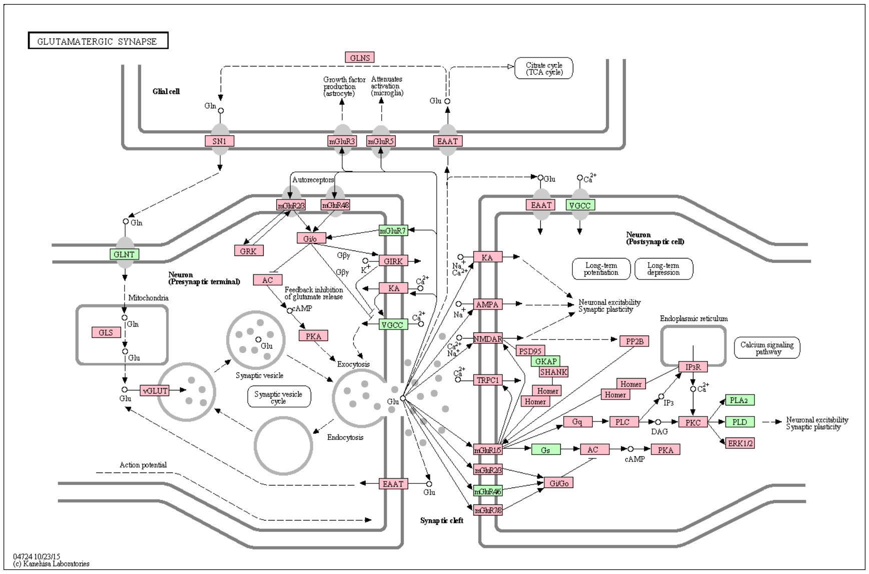

33.3 Legend:

RBH-Blast at 60% Identity + 50% Coverage
Green = Hit in *H. sapiens*
Red = Hit in *H. sapiens* and *T. californica*
White = Not in *H. sapiens*

34 cGMP-PKG signaling pathway

34.1 Human Pathway: HSA04022

34.2 Number of Hits: 50

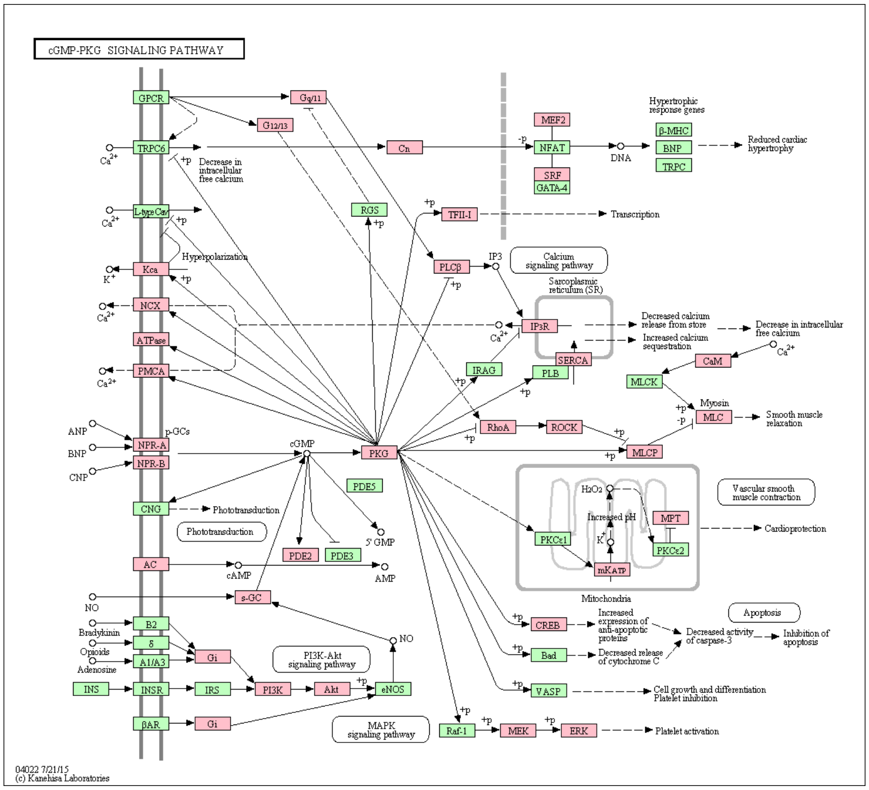

34.3 Legend:

RBH-Blast at 60% Identity + 50% Coverage
Green = Hit in *H. sapiens*
Red = Hit in *H. sapiens* and *T. californica*
White = Not in *H. sapiens*

35 AMPK signaling pathway

35.1 Human Pathway: HSA04152

35.2 Number of Hits: 50

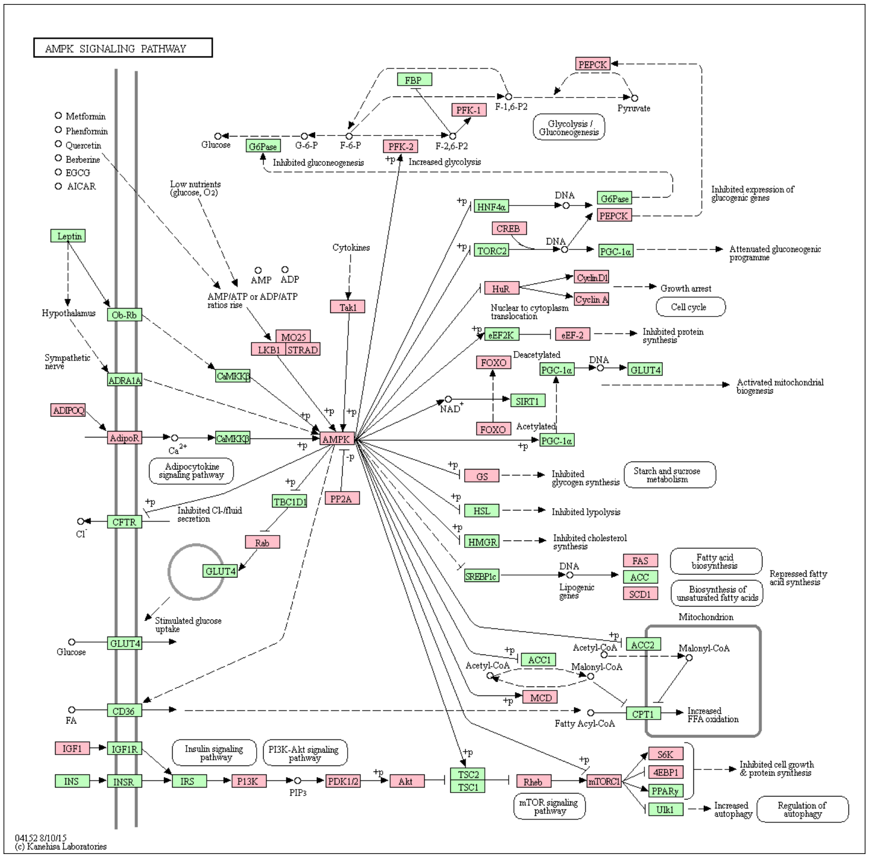

35.3 Legend:

RBH-Blast at 60% Identity + 50% Coverage
Green = Hit in *H. sapiens*
Red = Hit in *H. sapiens* and *T. californica*
White = Not in *H. sapiens*

36 Insulin signaling pathway

36.1 Human Pathway: HSA04910

36.2 Number of Hits: 50

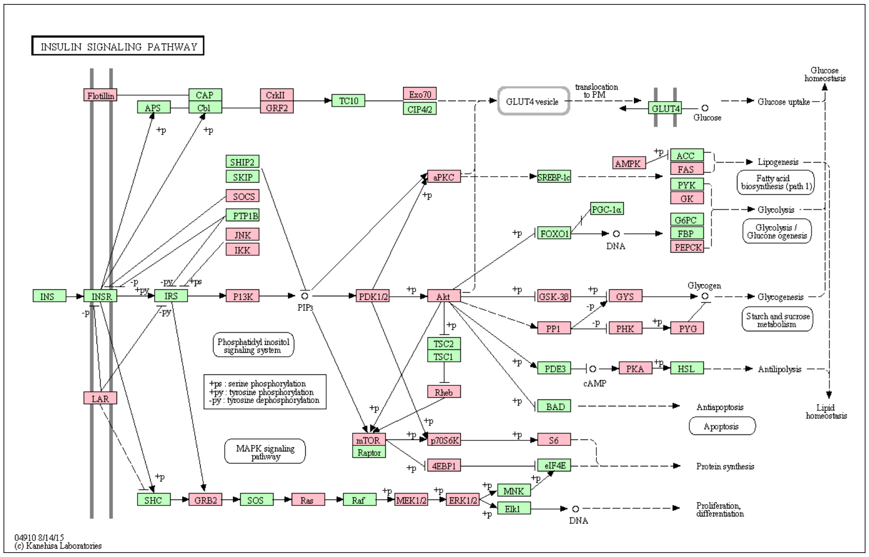

36.3 Legend:

RBH-Blast at 60% Identity + 50% Coverage
Green = Hit in *H. sapiens*
Red = Hit in *H. sapiens* and *T. californica*
White = Not in *H. sapiens*

37 Cell cycle

37.1 Human Pathway: HSA04110

37.2 Number of Hits: 50

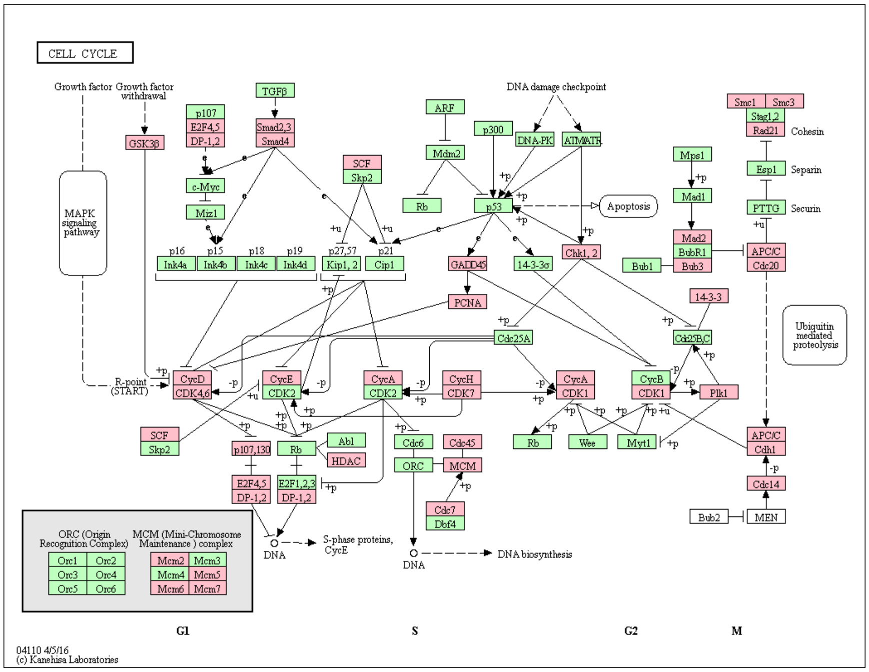

37.3 Legend:

RBH-Blast at 60% Identity + 50% Coverage
Green = Hit in *H. sapiens*
Red = Hit in *H. sapiens* and *T. californica*
White = Not in *H. sapiens*

38 Neurotrophin signaling pathway

38.1 Human Pathway: HSA04722

38.2 Number of Hits: 49

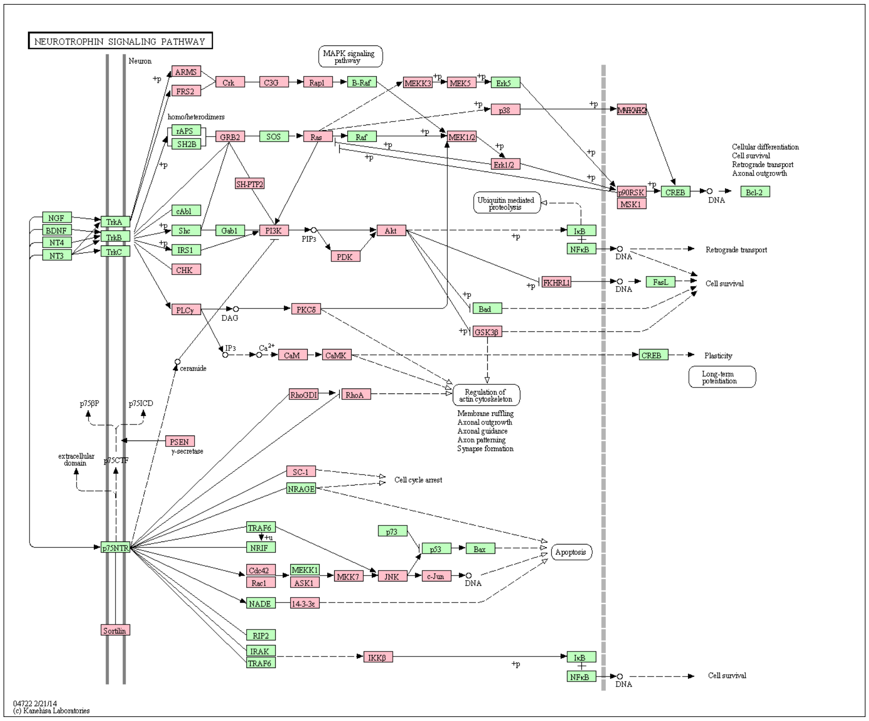

38.3 Legend:

RBH-Blast at 60% Identity + 50% Coverage
Green = Hit in *H. sapiens*
Red = Hit in *H. sapiens* and *T. californica*
White = Not in *H. sapiens*

39 Oocyte meiosis

39.1 Human Pathway: HSA04114

39.2 Number of Hits: 49

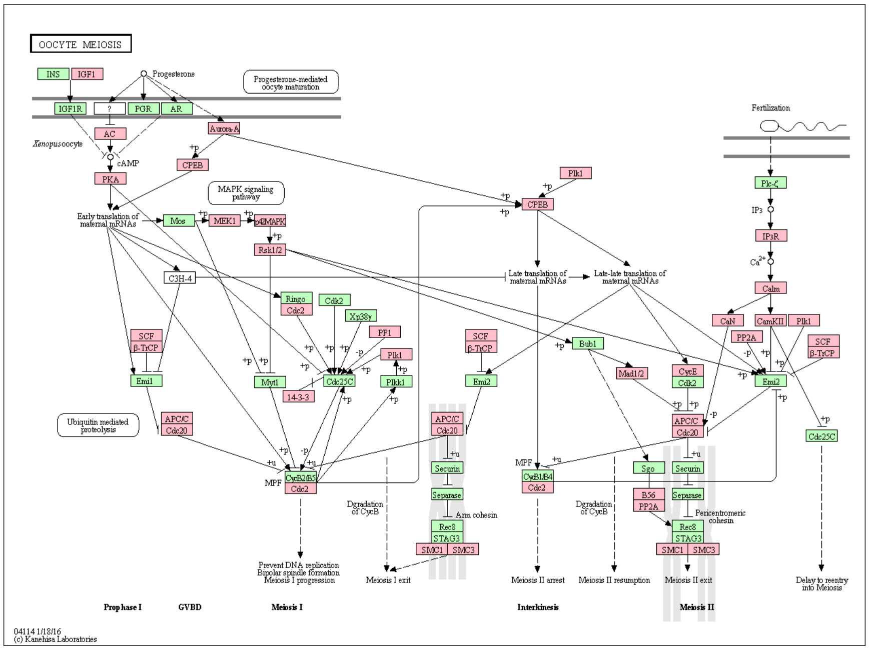

39.3 Legend:

RBH-Blast at 60% Identity + 50% Coverage
Green = Hit in *H. sapiens*
Red = Hit in *H. sapiens* and *T. californica*
White = Not in *H. sapiens*

40 Sphingolipid signaling pathway

40.1 Human Pathway: HSA04071

40.2 Number of Hits: 48

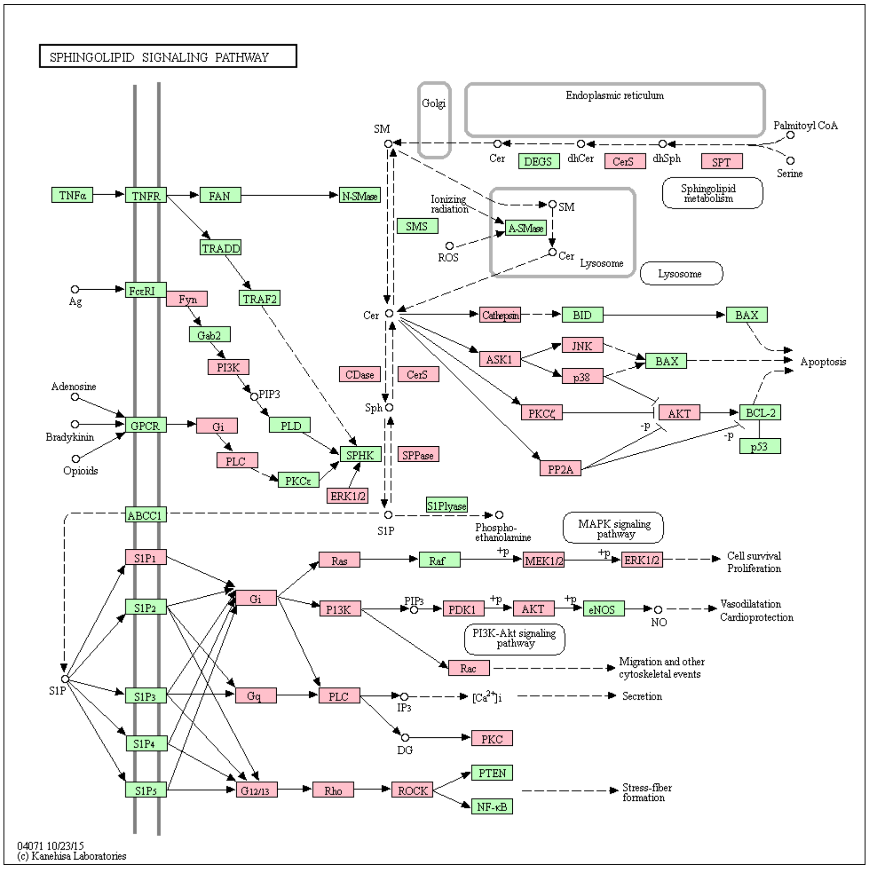

40.3 Legend:

RBH-Blast at 60% Identity + 50% Coverage
Green = Hit in *H. sapiens*
Red = Hit in *H. sapiens* and *T. californica*
White = Not in *H. sapiens*

41 Retrograde endocannabinoid signaling

41.1 Human Pathway: HSA04723

41.2 Number of Hits: 48

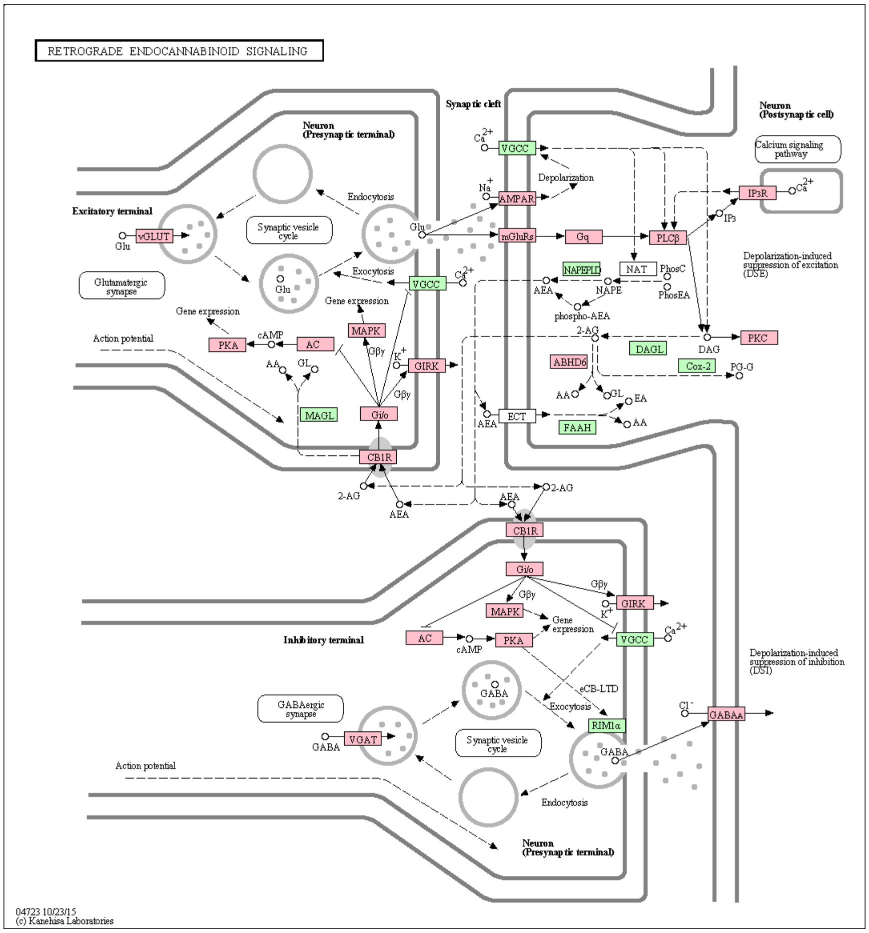

41.3 Legend:

RBH-Blast at 60% Identity + 50% Coverage
Green = Hit in *H. sapiens*
Red = Hit in *H. sapiens* and *T. californica*
White = Not in *H. sapiens*

42 Pyrimidine metabolism

42.1 Human Pathway: HSA00240

42.2 Number of Hits: 47

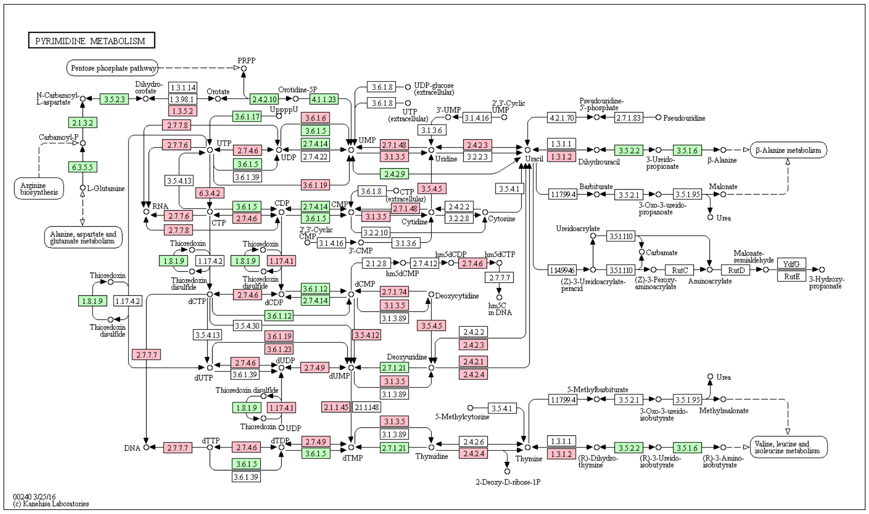

42.3 Legend:

RBH-Blast at 60% Identity + 50% Coverage
Green = Hit in *H. sapiens*
Red = Hit in *H. sapiens* and *T. californica*
White = Not in *H. sapiens*

43 Phospholipase D signaling pathway

43.1 Human Pathway: HSA04072

43.2 Number of Hits: 47

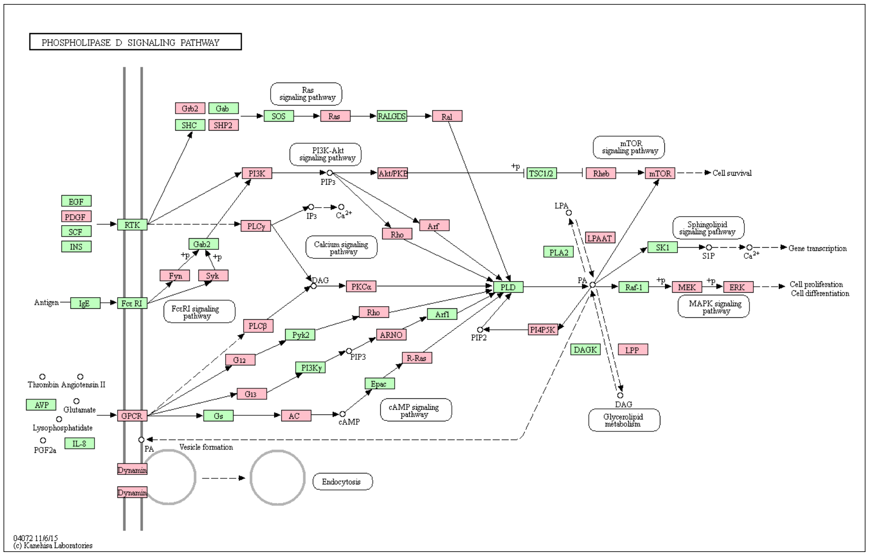

43.3 Legend:

RBH-Blast at 60% Identity + 50% Coverage
Green = Hit in *H. sapiens*
Red = Hit in *H. sapiens* and *T. californica*
White = Not in *H. sapiens*

44 Wnt signaling pathway

44.1 Human Pathway: HSA04310

44.2 Number of Hits: 47

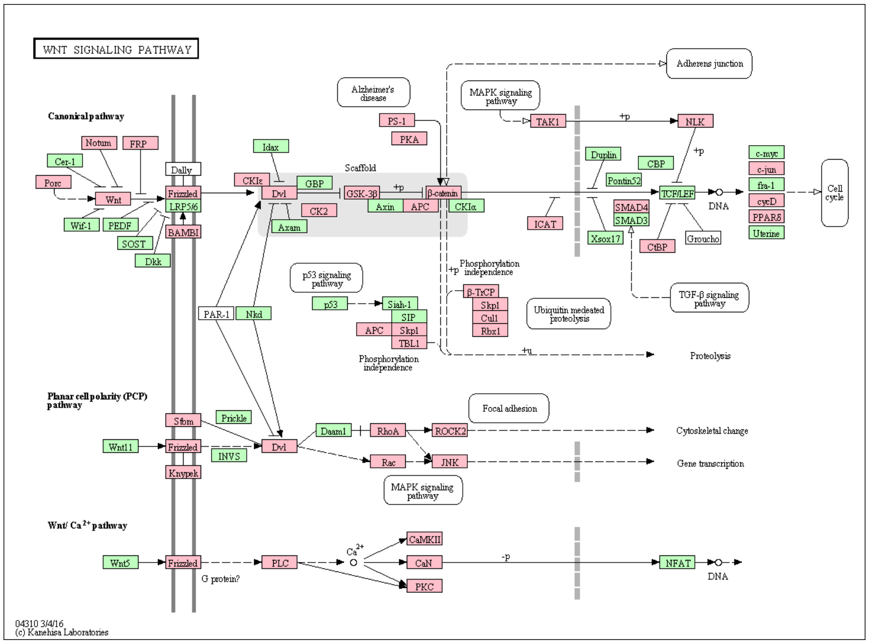

44.3 Legend:

RBH-Blast at 60% Identity + 50% Coverage
Green = Hit in *H. sapiens*
Red = Hit in *H. sapiens* and *T. californica*
White = Not in *H. sapiens*

45 Tight junction

45.1 Human Pathway: HSA04530

45.2 Number of Hits: 46

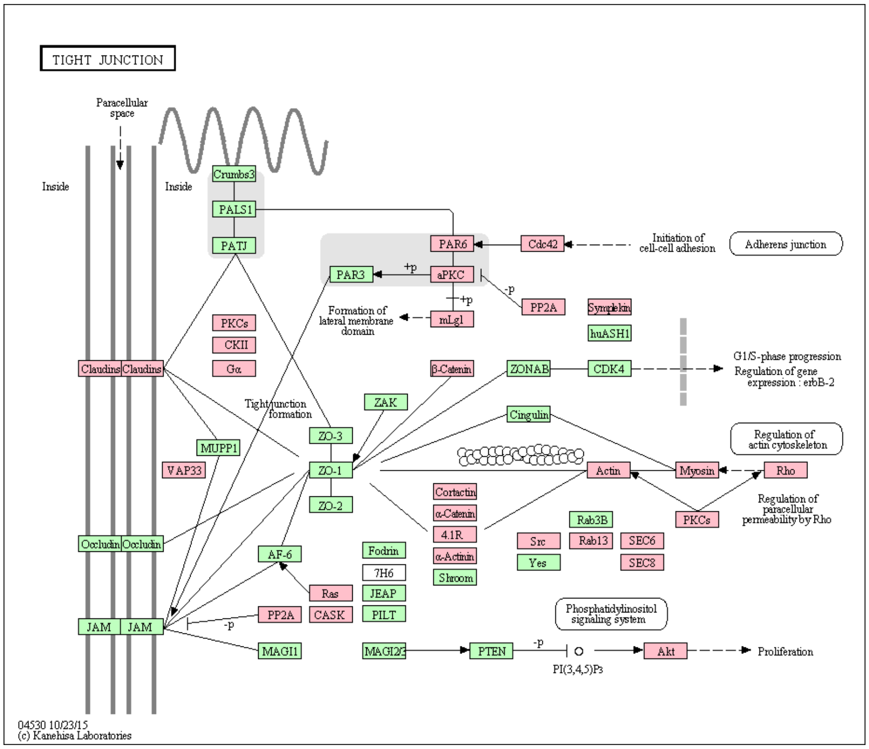

45.3 Legend:

RBH-Blast at 60% Identity + 50% Coverage
Green = Hit in *H. sapiens*
Red = Hit in *H. sapiens* and *T. californica*
White = Not in *H. sapiens*

46 Neuroactive ligand-receptor interaction

46.1 Human Pathway: HSA04080

46.2 Number of Hits: 45

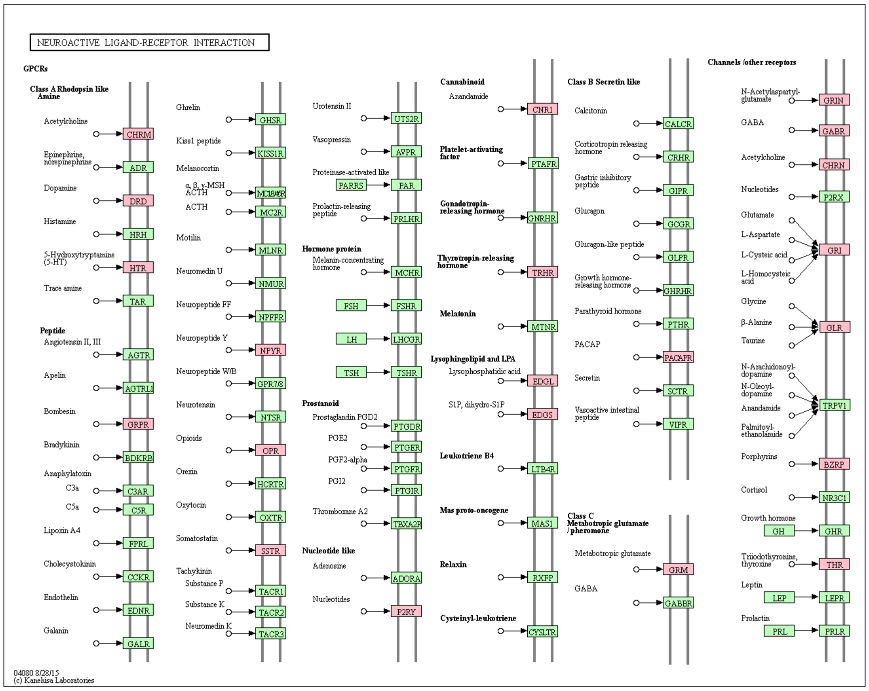

46.3 Legend:

RBH-Blast at 60% Identity + 50% Coverage
Green = Hit in *H. sapiens*
Red = Hit in *H. sapiens* and *T. californica*
White = Not in *H. sapiens*

47 Phagosome

47.1 Human Pathway: HSA04145

47.2 Number of Hits: 45

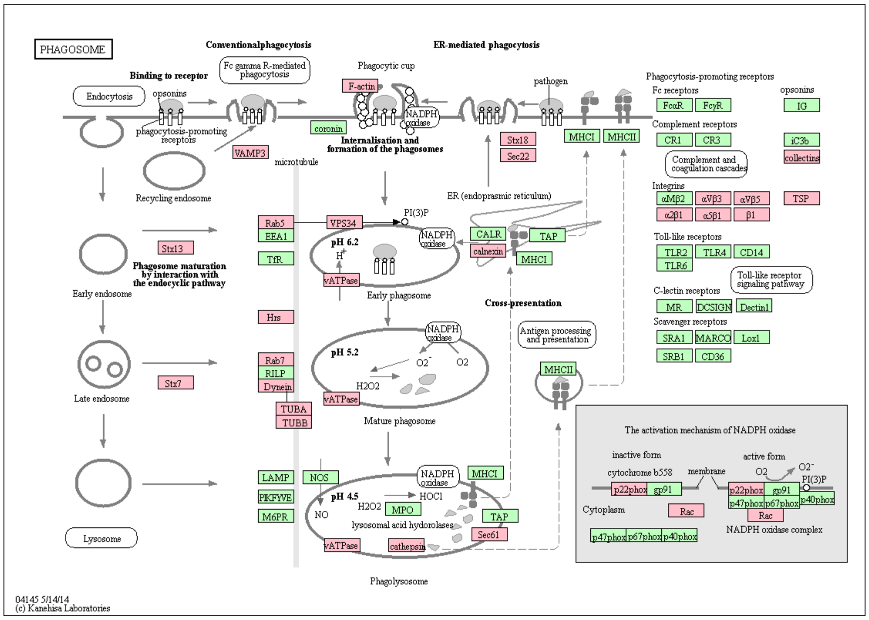

47.3 Legend:

RBH-Blast at 60% Identity + 50% Coverage
Green = Hit in *H. sapiens*
Red = Hit in *H. sapiens* and *T. californica*
White = Not in *H. sapiens*

48 mRNA surveillance pathway

48.1 Human Pathway: HSA03015

48.2 Number of Hits: 44

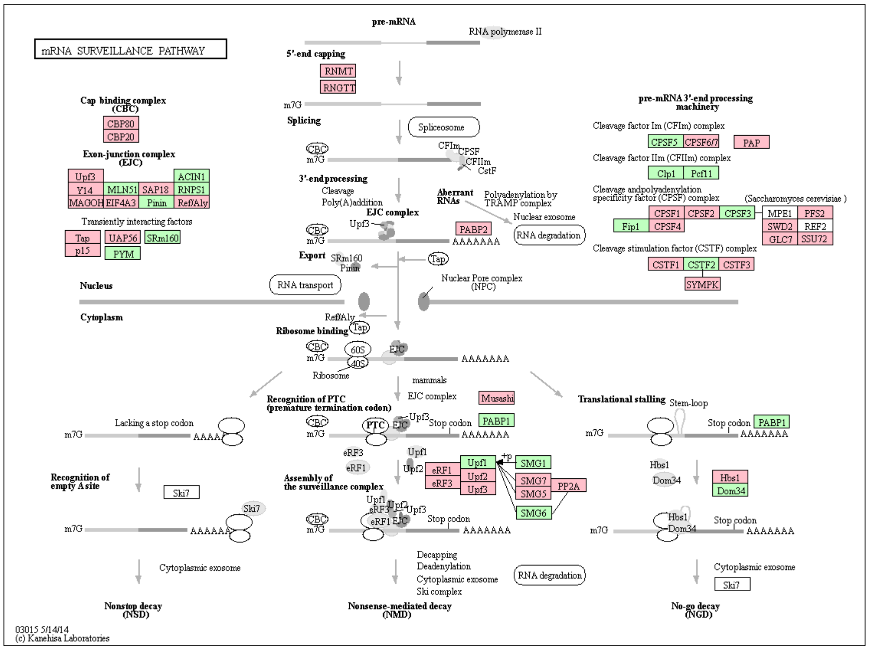

48.3 Legend:

RBH-Blast at 60% Identity + 50% Coverage
Green = Hit in *H. sapiens*
Red = Hit in *H. sapiens* and *T. californica*
White = Not in *H. sapiens*

49 Thyroid hormone signaling pathway

49.1 Human Pathway: HSA04919

49.2 Number of Hits: 44

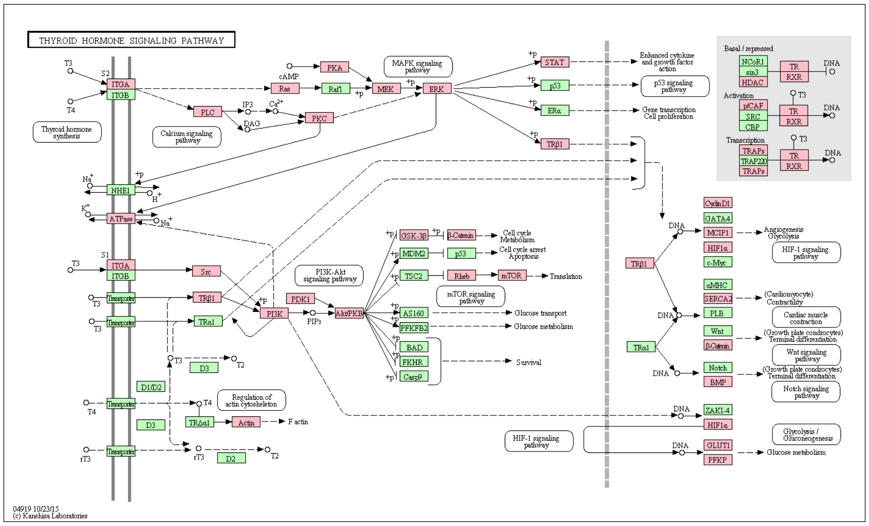

49.3 Legend:

RBH-Blast at 60% Identity + 50% Coverage
Green = Hit in *H. sapiens*
Red = Hit in *H. sapiens* and *T. californica*
White = Not in *H. sapiens*

50 Alcoholism

50.1 Human Pathway: HSA05034

50.2 Number of Hits: 43

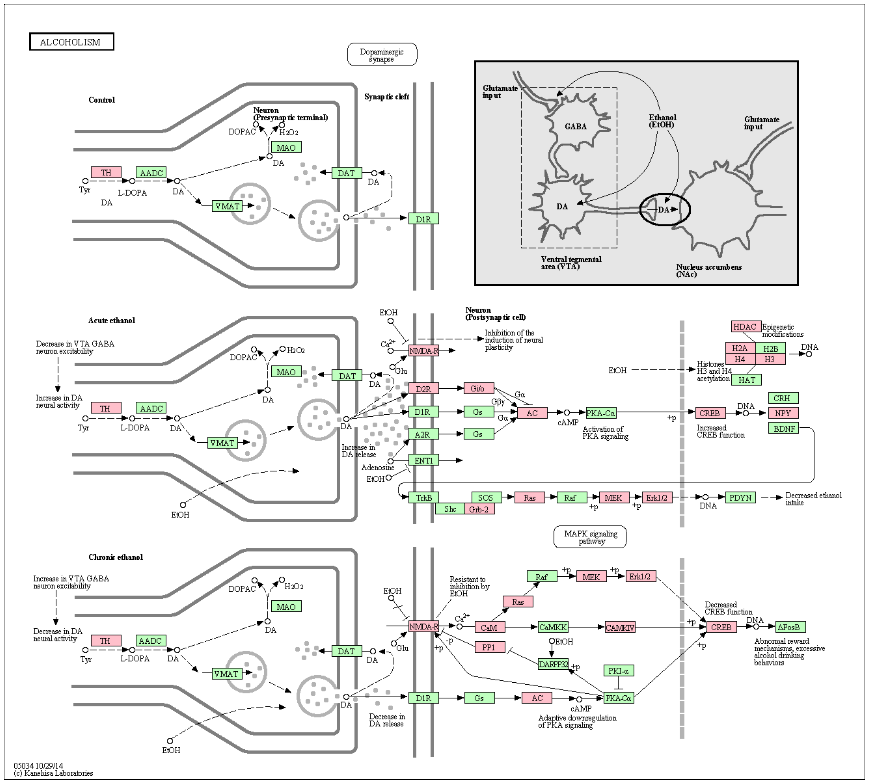

50.3 Legend:

RBH-Blast at 60% Identity + 50% Coverage
Green = Hit in *H. sapiens*
Red = Hit in *H. sapiens* and *T. californica*
White = Not in *H. sapiens*

51 Influenza A

51.1 Human Pathway: HSA05164

51.2 Number of Hits: 43

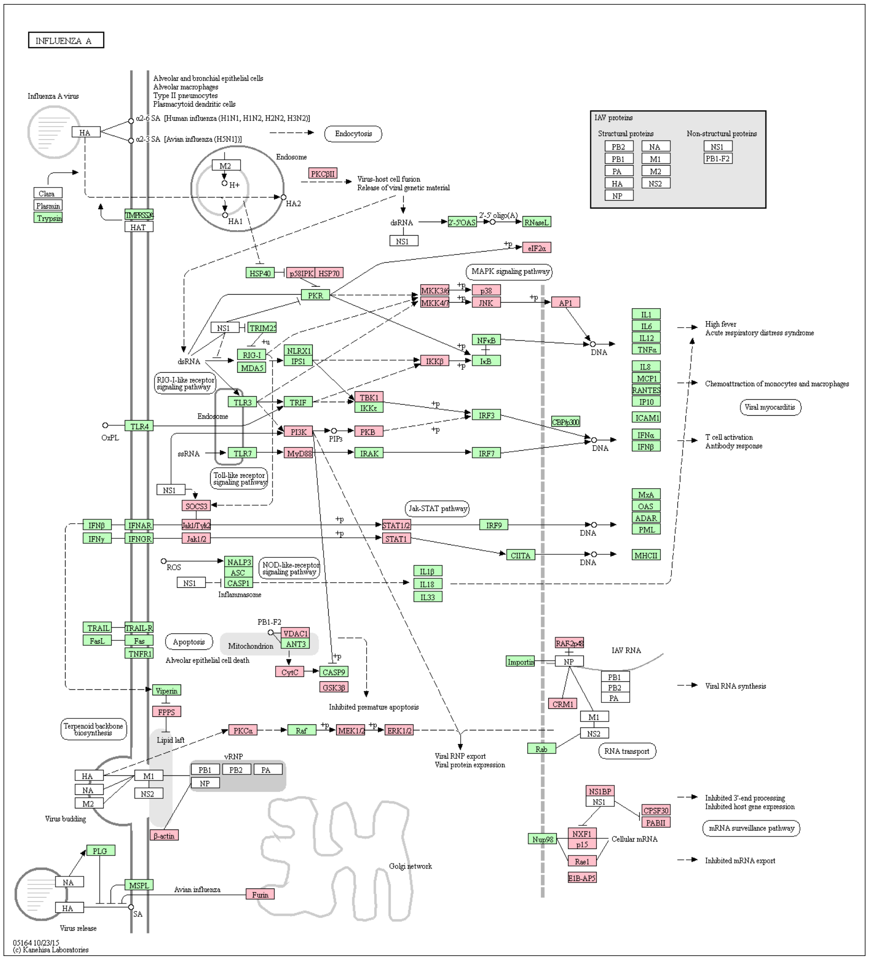

51.3 Legend:

RBH-Blast at 60% Identity + 50% Coverage
Green = Hit in *H. sapiens*
Red = Hit in *H. sapiens* and *T. californica*
White = Not in *H. sapiens*

52 GABAergic synapse

52.1 Human Pathway: HSA04727

52.2 Number of Hits: 42

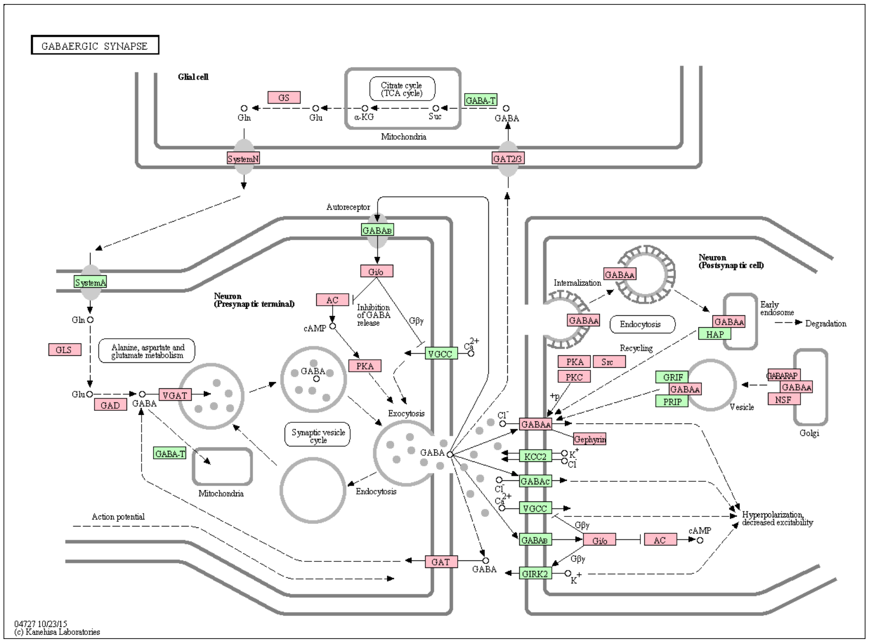

52.3 Legend:

RBH-Blast at 60% Identity + 50% Coverage
Green = Hit in *H. sapiens*
Red = Hit in *H. sapiens* and *T. californica*
White = Not in *H. sapiens*

53 Hippo signaling pathway

53.1 Human Pathway: HSA04390

53.2 Number of Hits: 42

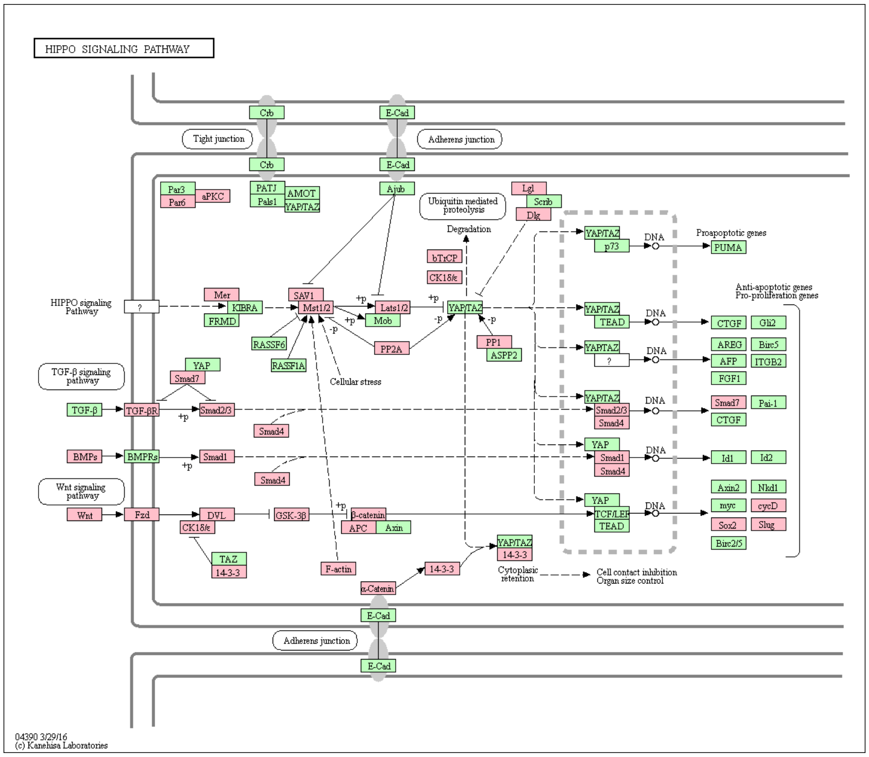

53.3 Legend:

RBH-Blast at 60% Identity + 50% Coverage
Green = Hit in *H. sapiens*
Red = Hit in *H. sapiens* and *T. californica*
White = Not in *H. sapiens*

54 Fc gamma R-mediated phagocytosis

54.1 Human Pathway: HSA04666

54.2 Number of Hits: 41

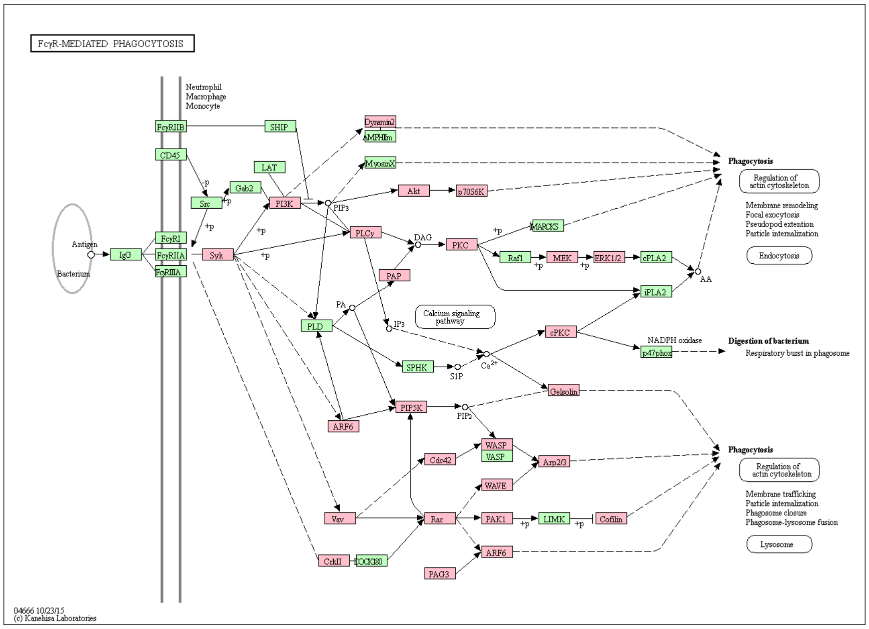

54.3 Legend:

RBH-Blast at 60% Identity + 50% Coverage
Green = Hit in *H. sapiens*
Red = Hit in *H. sapiens* and *T. californica*
White = Not in *H. sapiens*

55 RNA degradation

55.1 Human Pathway: HSA03018

55.2 Number of Hits: 41

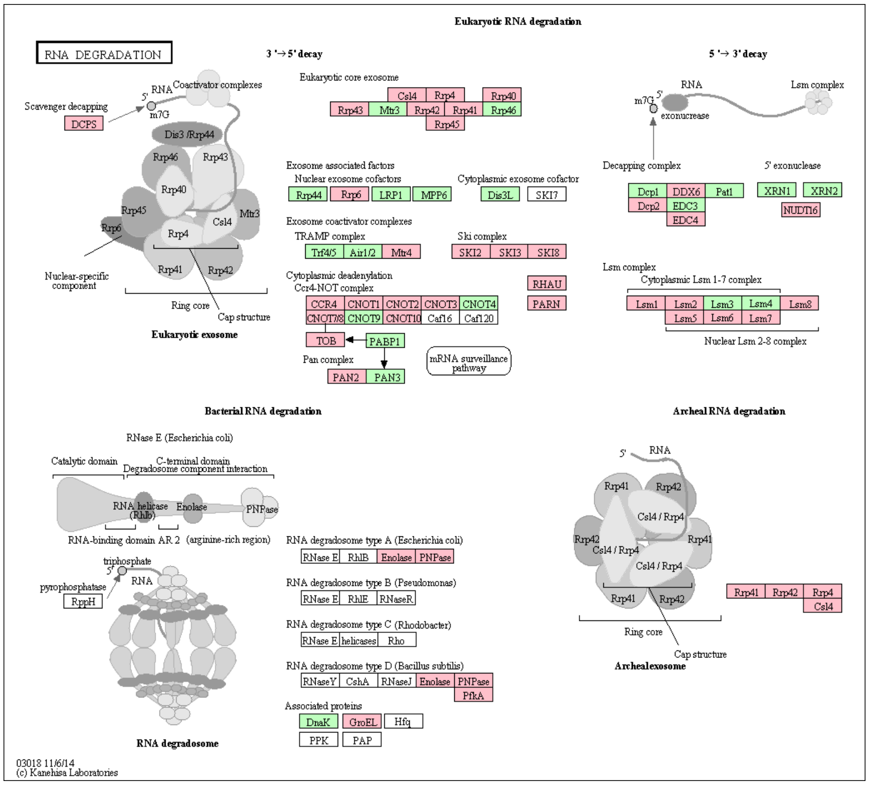

55.3 Legend:

RBH-Blast at 60% Identity + 50% Coverage
Green = Hit in *H. sapiens*
Red = Hit in *H. sapiens* and *T. californica*
White = Not in *H. sapiens*

56 Platelet activation

56.1 Human Pathway: HSA04611

56.2 Number of Hits: 41

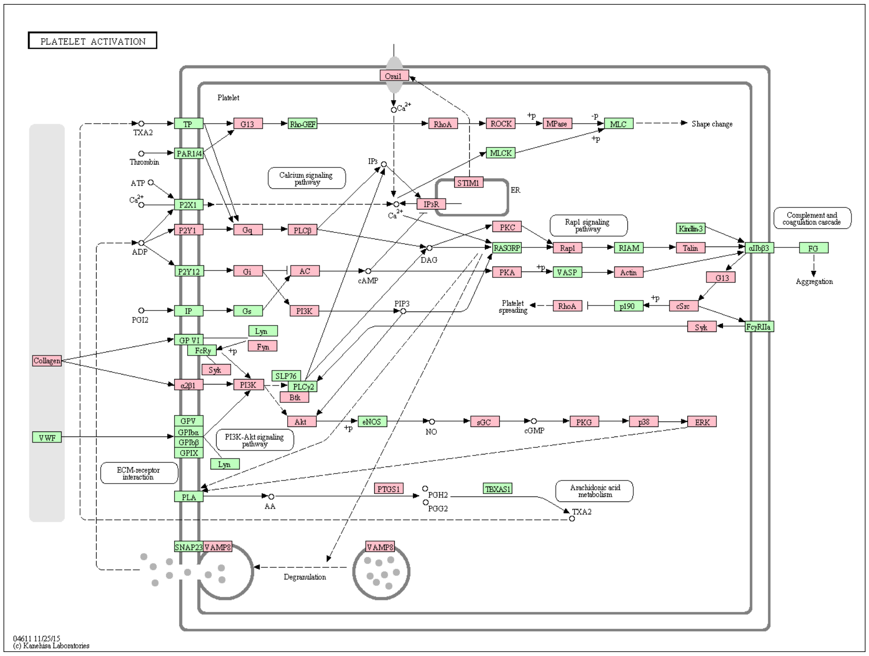

56.3 Legend:

RBH-Blast at 60% Identity + 50% Coverage
Green = Hit in *H. sapiens*
Red = Hit in *H. sapiens* and *T. californica*
White = Not in *H. sapiens*

57 Herpes simplex infection

57.1 Human Pathway: HSA05168

57.2 Number of Hits: 41

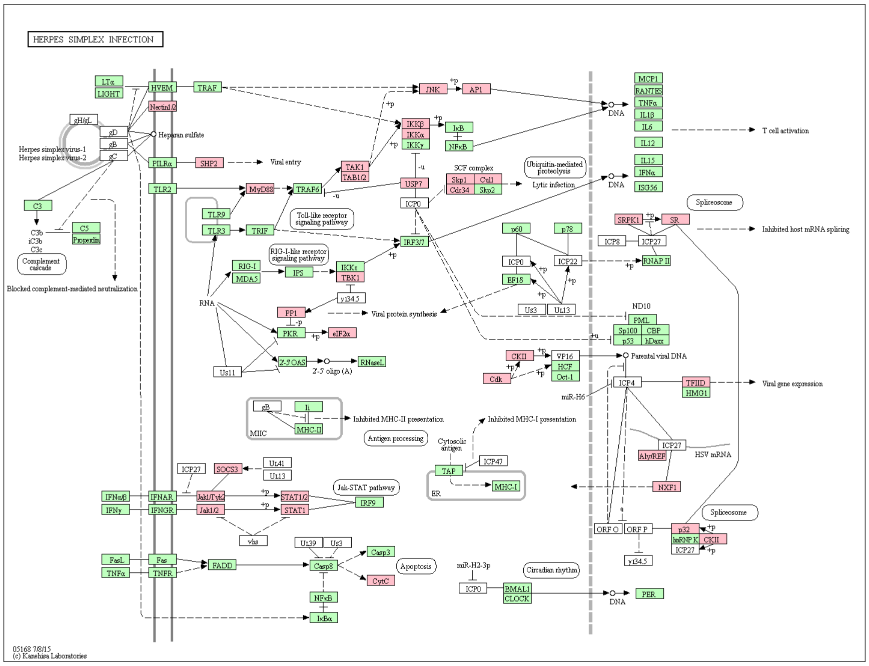

57.3 Legend:

RBH-Blast at 60% Identity + 50% Coverage
Green = Hit in *H. sapiens*
Red = Hit in *H. sapiens* and *T. californica*
White = Not in *H. sapiens*

58 Biosynthesis of amino acids

58.1 Human Pathway: HSA01230

58.2 Number of Hits: 40

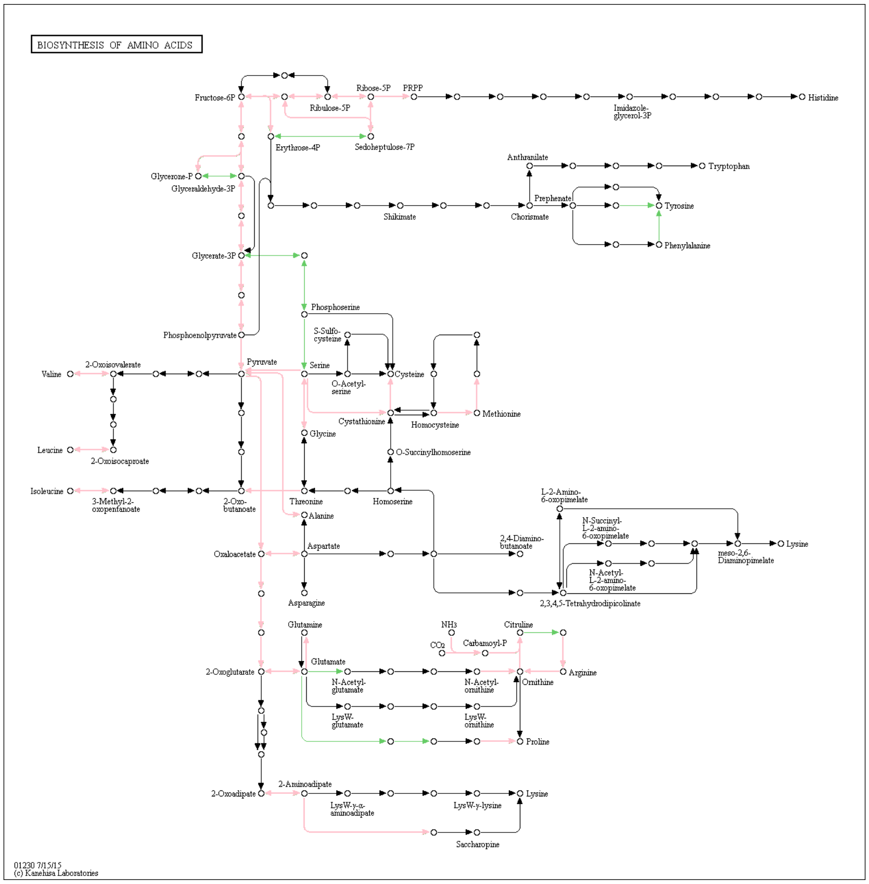

58.3 Legend:

RBH-Blast at 60% Identity + 50% Coverage
Green = Hit in *H. sapiens*
Red = Hit in *H. sapiens* and *T. californica*
White = Not in *H. sapiens*

59 Choline metabolism in cancer

59.1 Human Pathway: HSA05231

59.2 Number of Hits: 40

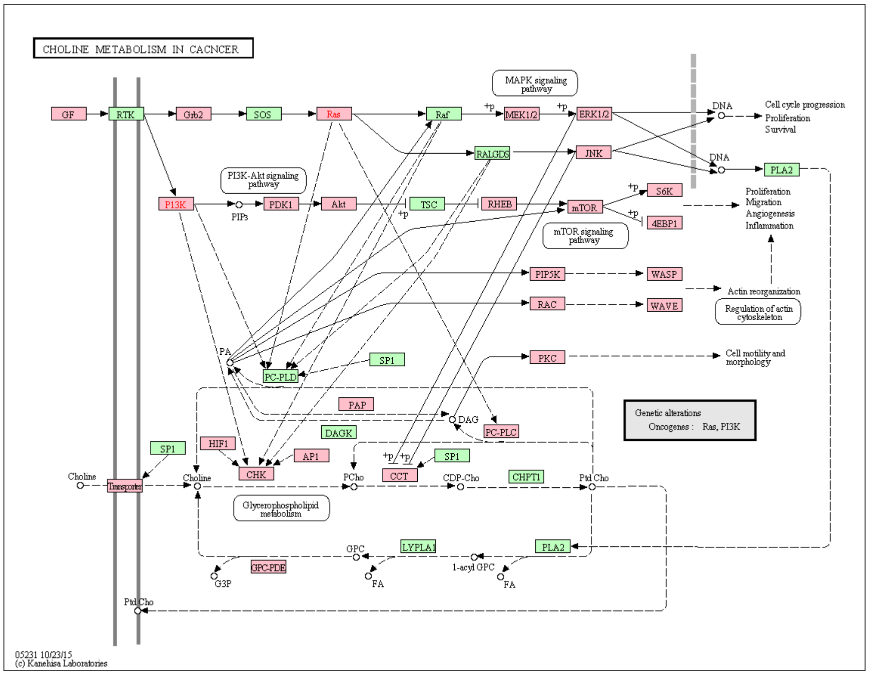

59.3 Legend:

RBH-Blast at 60% Identity + 50% Coverage
Green = Hit in *H. sapiens*
Red = Hit in *H. sapiens* and *T. californica*
White = Not in *H. sapiens*

60 Apoptosis

60.1 Human Pathway: HSA04210

60.2 Number of Hits: 39

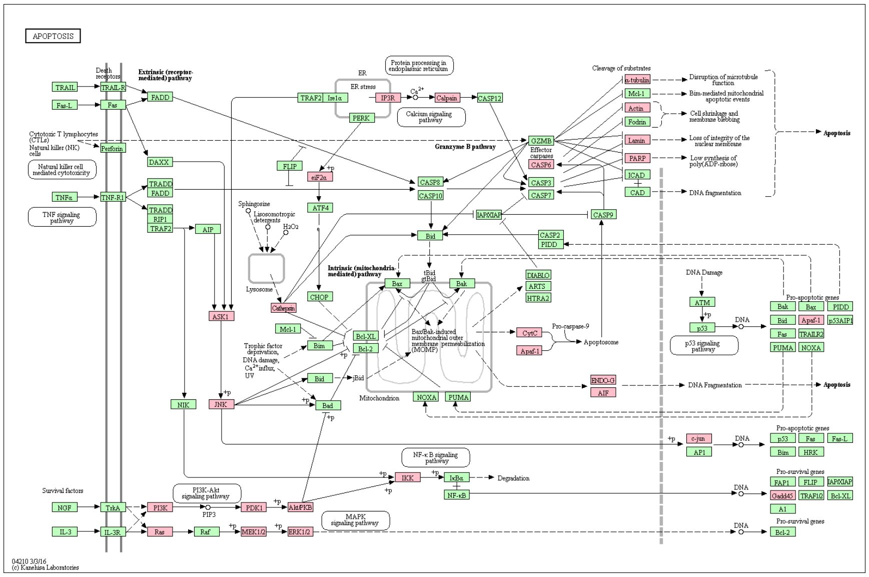

60.3 Legend:

RBH-Blast at 60% Identity + 50% Coverage
Green = Hit in *H. sapiens*
Red = Hit in *H. sapiens* and *T. californica*
White = Not in *H. sapiens*

61 Morphine addiction

61.1 Human Pathway: HSA05032

61.2 Number of Hits: 39

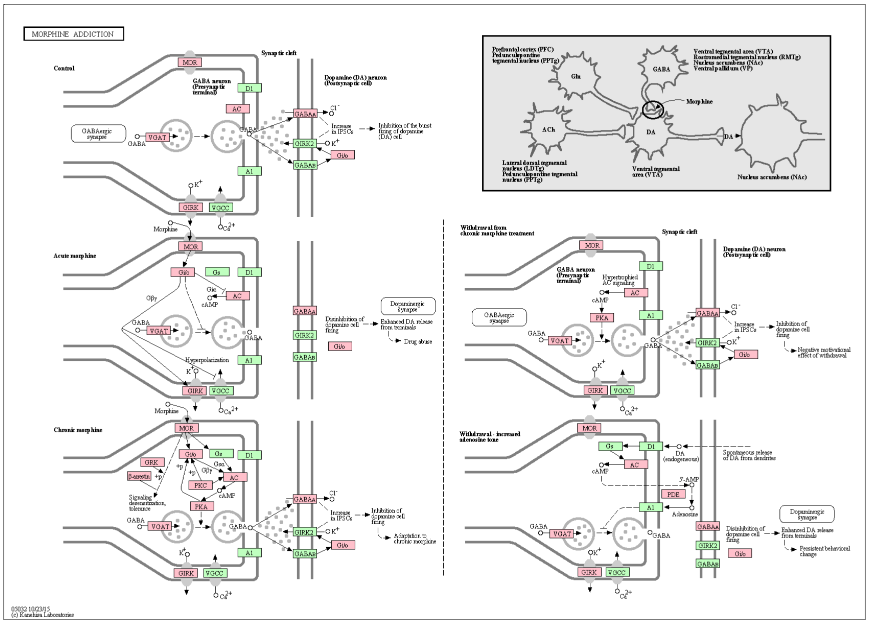

61.3 Legend:

RBH-Blast at 60% Identity + 50% Coverage
Green = Hit in *H. sapiens*
Red = Hit in *H. sapiens* and *T. californica*
White = Not in *H. sapiens*

62 Insulin resistance

62.1 Human Pathway: HSA04931

62.2 Number of Hits: 39

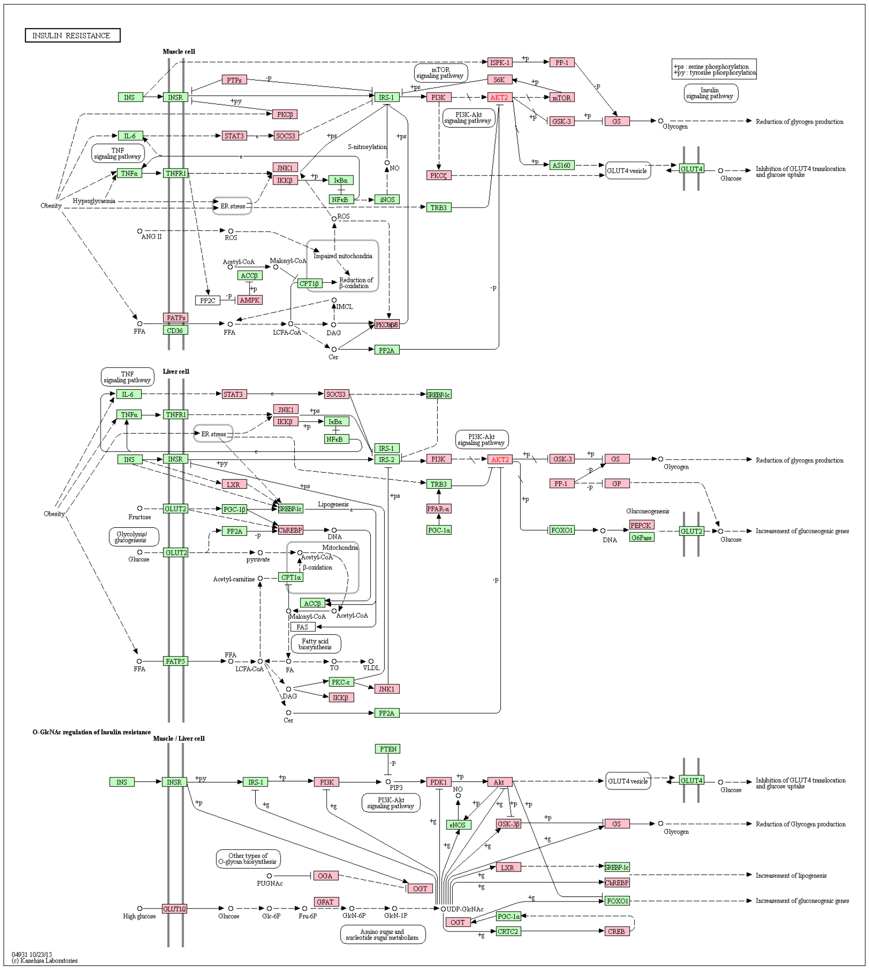

62.3 Legend:

RBH-Blast at 60% Identity + 50% Coverage
Green = Hit in *H. sapiens*
Red = Hit in *H. sapiens* and *T. californica*
White = Not in *H. sapiens*

63 Cholinergic synapse

63.1 Human Pathway: HSA04725

63.2 Number of Hits: 39

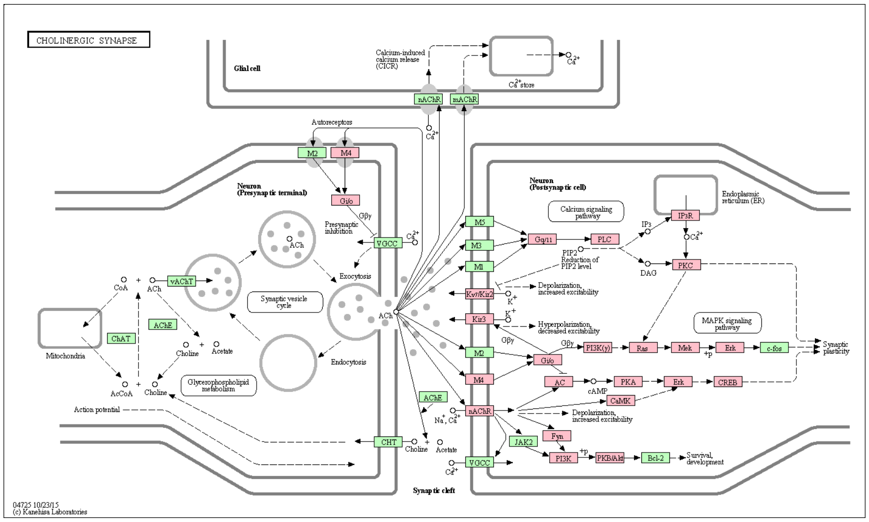

63.3 Legend:

RBH-Blast at 60% Identity + 50% Coverage
Green = Hit in *H. sapiens*
Red = Hit in *H. sapiens* and *T. californica*
White = Not in *H. sapiens*

64 Hepatitis B

64.1 Human Pathway: HSA05161

64.2 Number of Hits: 39

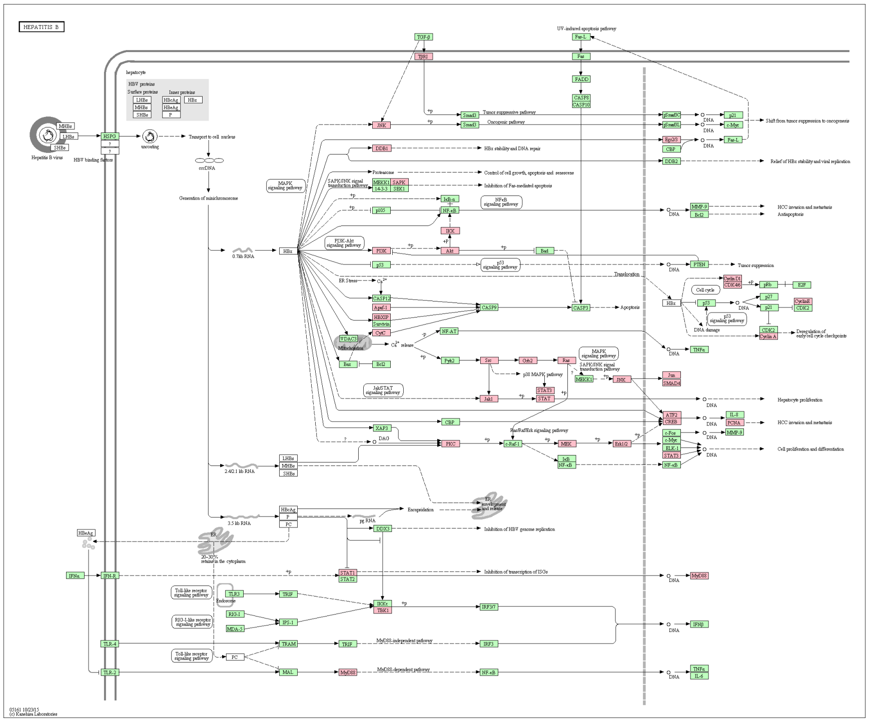

64.3 Legend:

RBH-Blast at 60% Identity + 50% Coverage
Green = Hit in *H. sapiens*
Red = Hit in *H. sapiens* and *T. californica*
White = Not in *H. sapiens*

65 Progesterone-mediated oocyte maturation

65.1 Human Pathway: HSA04914

65.2 Number of Hits: 39

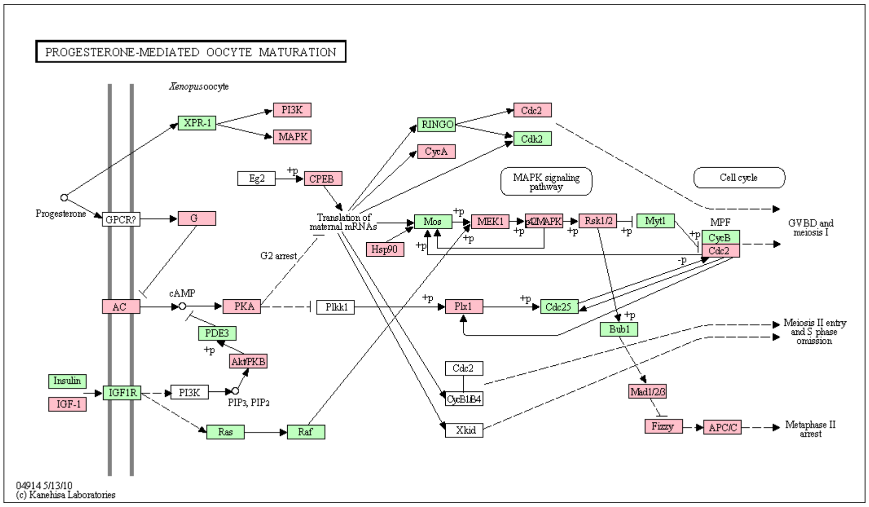

65.3 Legend:

RBH-Blast at 60% Identity + 50% Coverage
Green = Hit in *H. sapiens*
Red = Hit in *H. sapiens* and *T. californica*
White = Not in *H. sapiens*

66 Circadian entrainment

66.1 Human Pathway: HSA04713

66.2 Number of Hits: 38

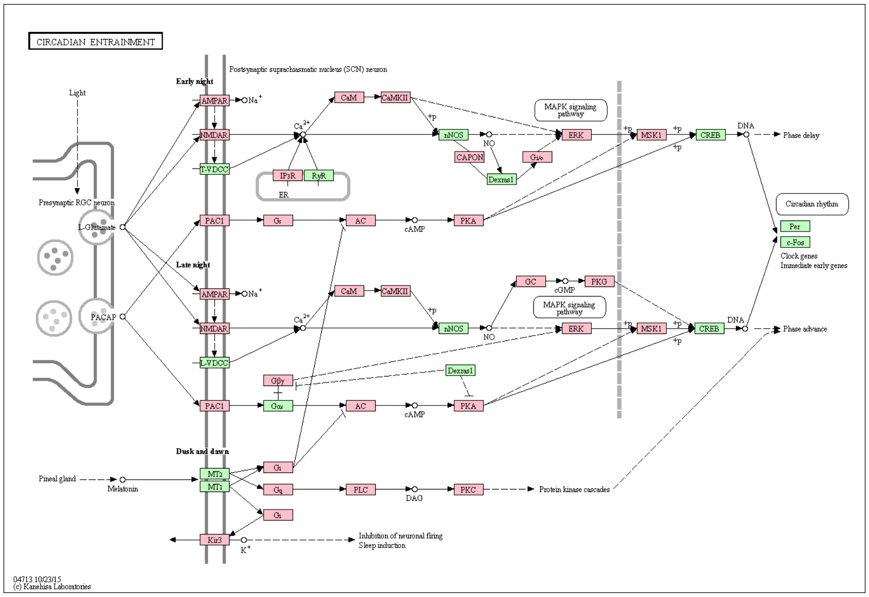

66.3 Legend:

RBH-Blast at 60% Identity + 50% Coverage
Green = Hit in *H. sapiens*
Red = Hit in *H. sapiens* and *T. californica*
White = Not in *H. sapiens*

67 T cell receptor signaling pathway

67.1 Human Pathway: HSA04660

67.2 Number of Hits: 37

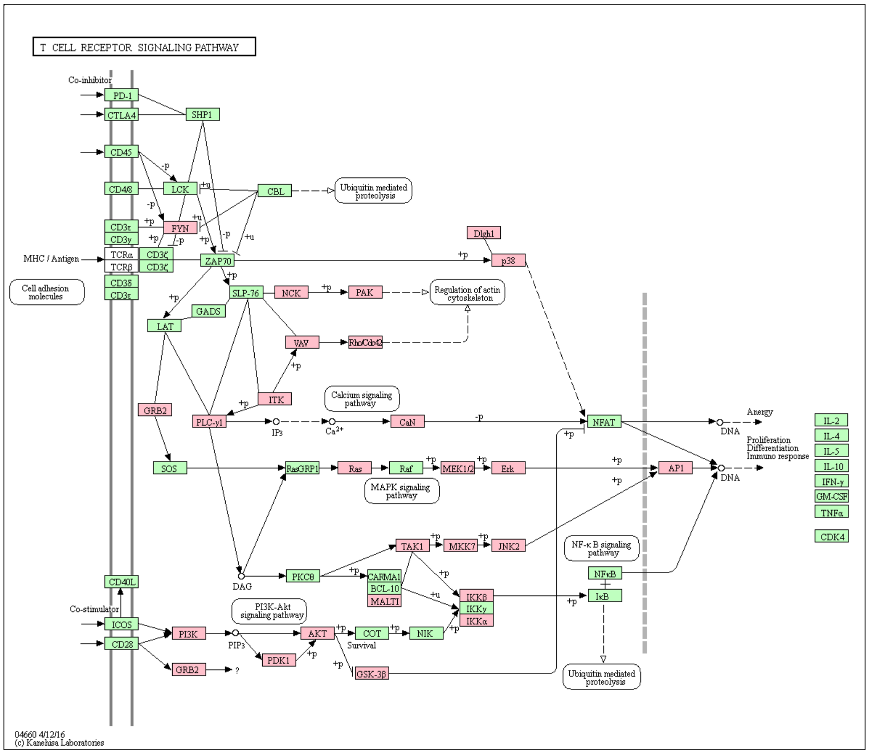

67.3 Legend:

RBH-Blast at 60% Identity + 50% Coverage
Green = Hit in *H. sapiens*
Red = Hit in *H. sapiens* and *T. californica*
White = Not in *H. sapiens*

68 Tuberculosis

68.1 Human Pathway: HSA05152

68.2 Number of Hits: 37

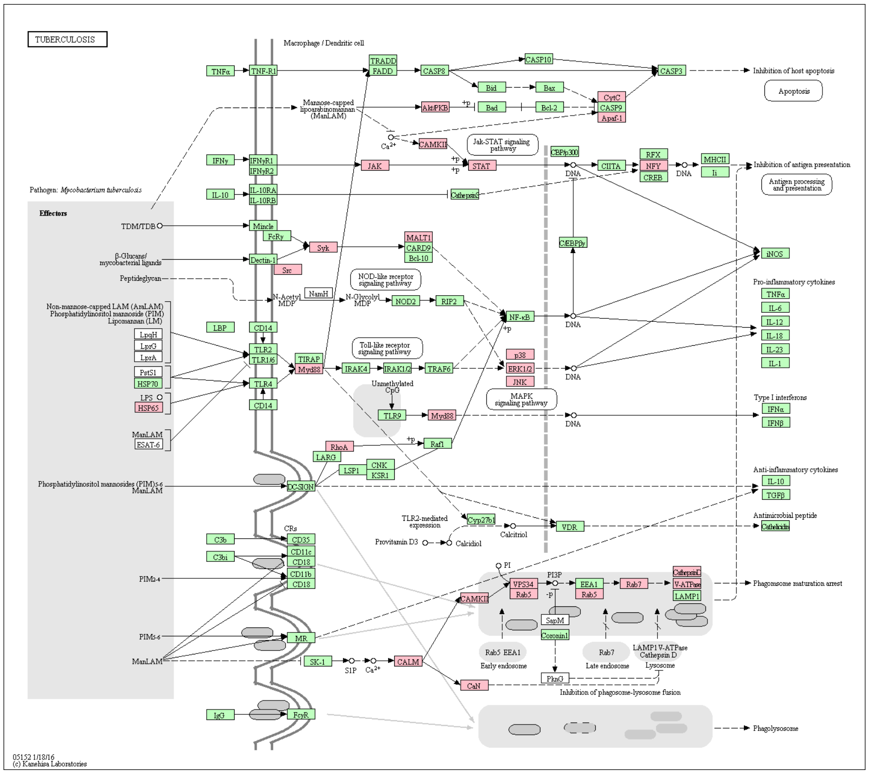

68.3 Legend:

RBH-Blast at 60% Identity + 50% Coverage
Green = Hit in *H. sapiens*
Red = Hit in *H. sapiens* and *T. californica*
White = Not in *H. sapiens*

69 Lysosome

69.1 Human Pathway: HSA04142

69.2 Number of Hits: 37

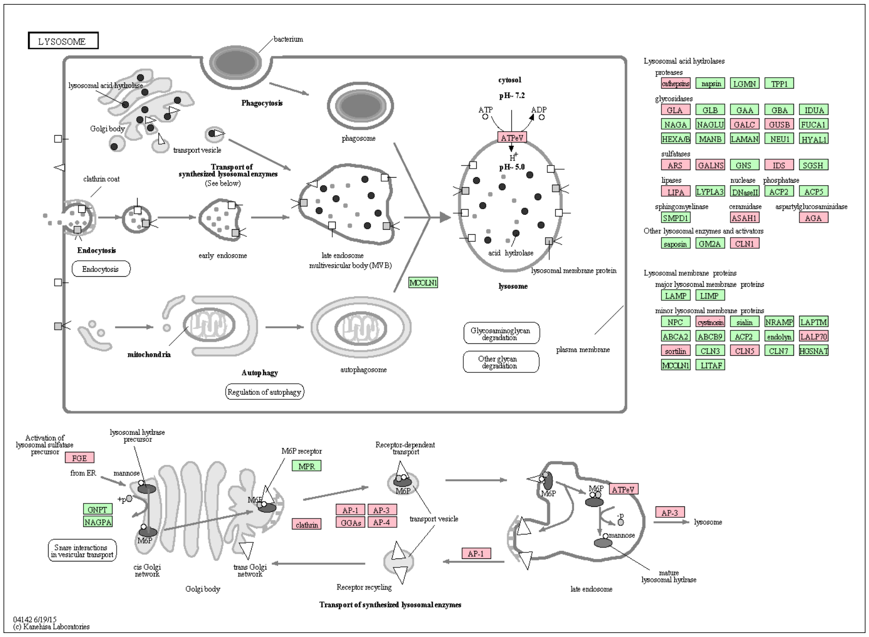

69.3 Legend:

RBH-Blast at 60% Identity + 50% Coverage
Green = Hit in *H. sapiens*
Red = Hit in *H. sapiens* and *T. californica*
White = Not in *H. sapiens*

70 Bacterial invasion of epithelial cells

70.1 Human Pathway: HSA05100

70.2 Number of Hits: 37

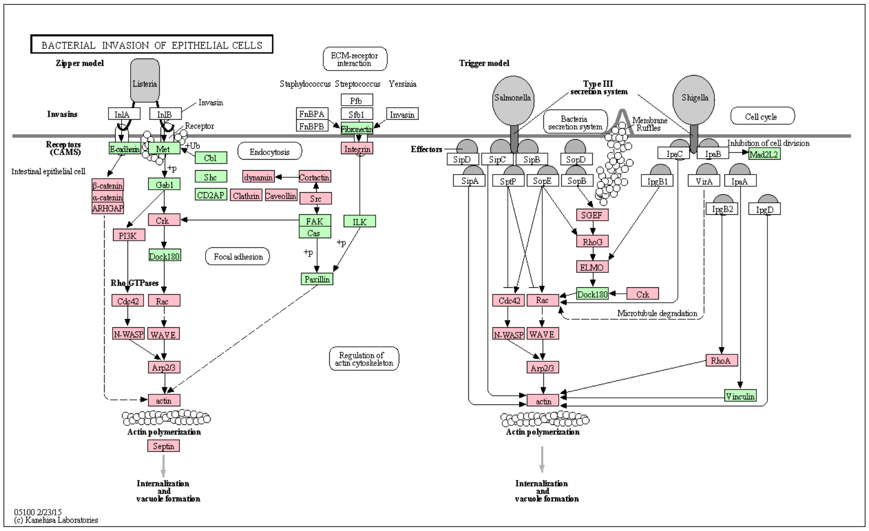

70.3 Legend:

RBH-Blast at 60% Identity + 50% Coverage
Green = Hit in *H. sapiens*
Red = Hit in *H. sapiens* and *T. californica*
White = Not in *H. sapiens*

71 Shigellosis

71.1 Human Pathway: HSA05131

71.2 Number of Hits: 36

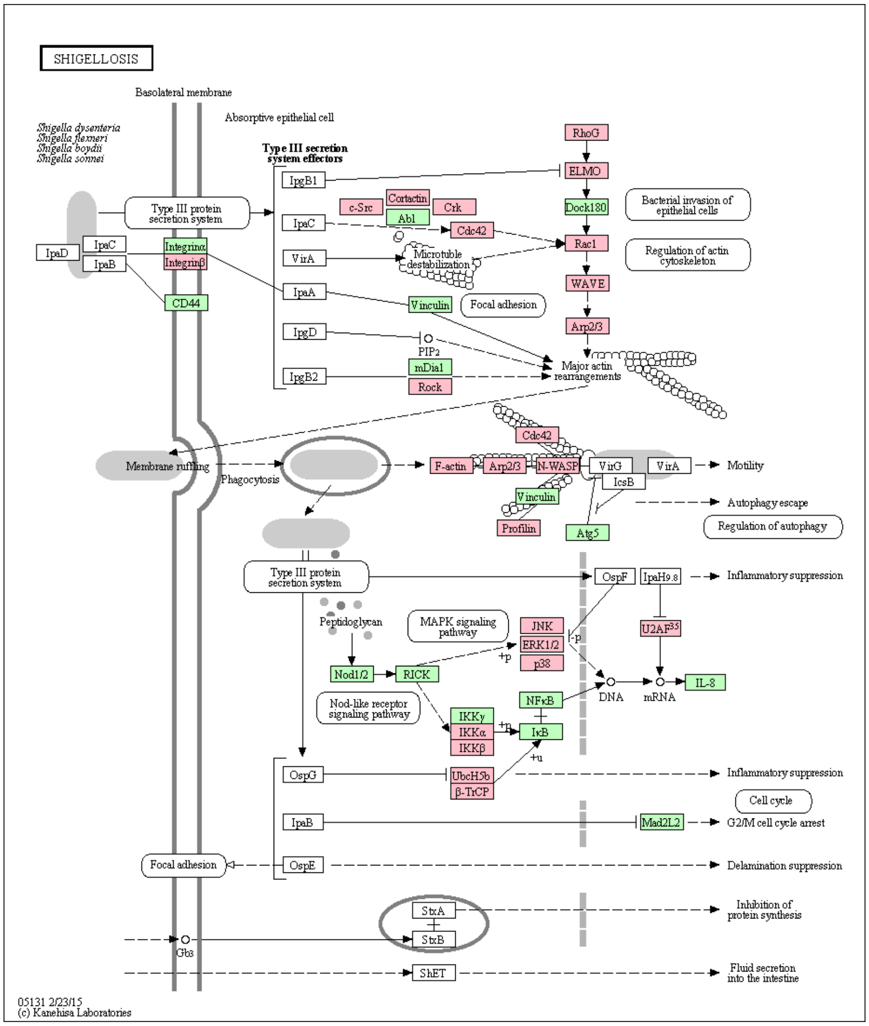

71.3 Legend:

RBH-Blast at 60% Identity + 50% Coverage
Green = Hit in *H. sapiens*
Red = Hit in *H. sapiens* and *T. californica*
White = Not in *H. sapiens*

72 MicroRNAs in cancer

72.1 Human Pathway: HSA05206

72.2 Number of Hits: 36

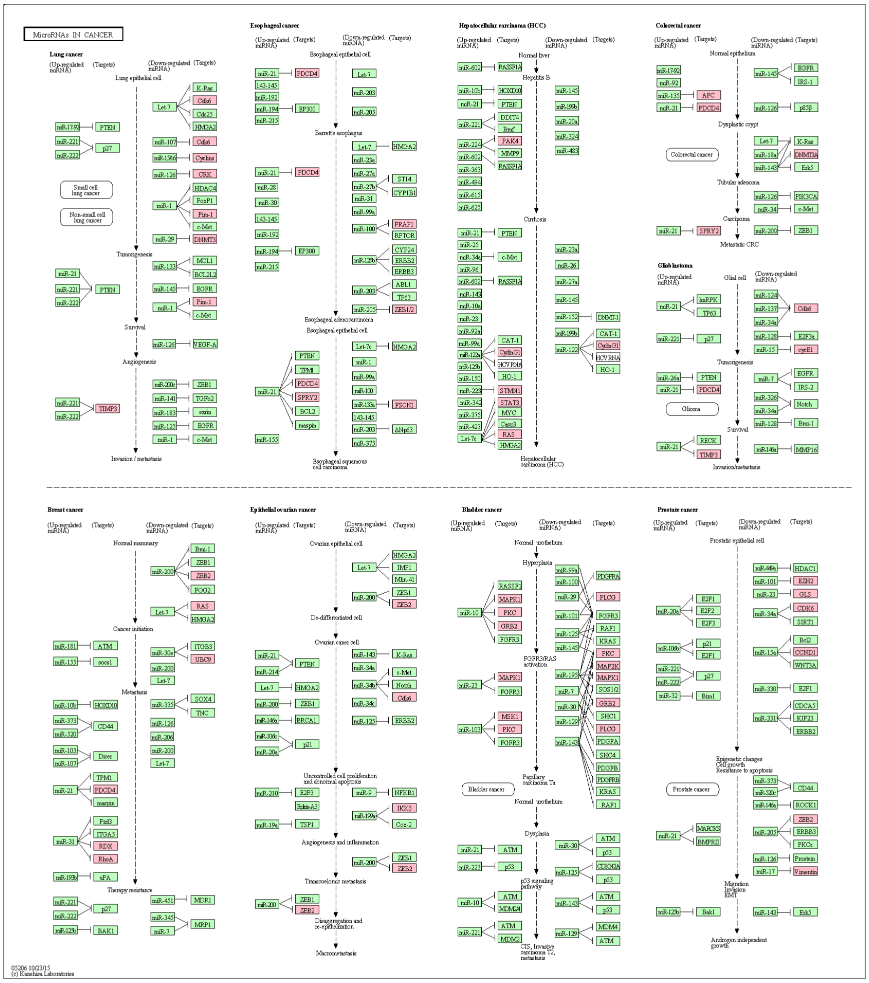

72.3 Legend:

RBH-Blast at 60% Identity + 50% Coverage
Green = Hit in *H. sapiens*
Red = Hit in *H. sapiens* and *T. californica*
White = Not in *H. sapiens*

---

73 Serotonergic synapse

73.1 Human Pathway: HSA04726

73.2 Number of Hits: 36

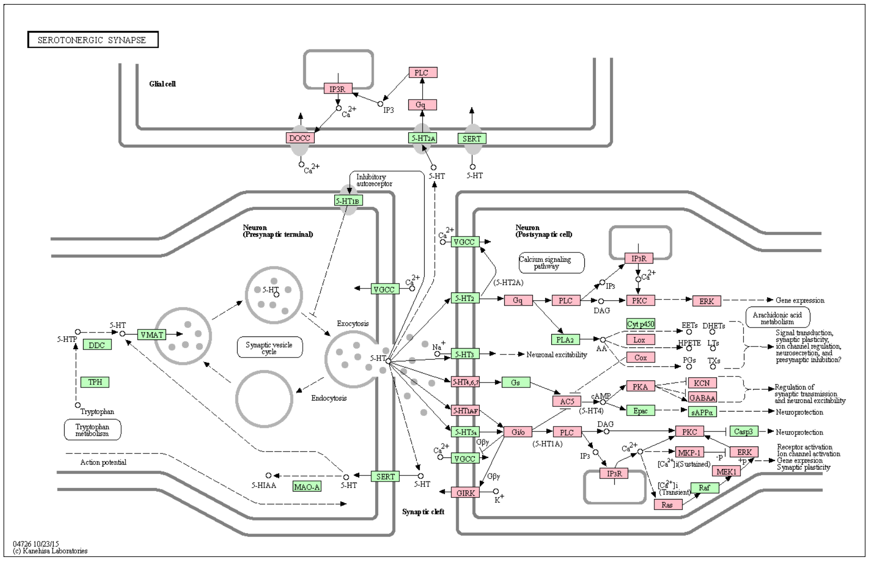

73.3 Legend:

RBH-Blast at 60% Identity + 50% Coverage
Green = Hit in *H. sapiens*
Red = Hit in *H. sapiens* and *T. californica*
White = Not in *H. sapiens*

---

74 Longevity regulating pathway

74.1 Human Pathway: HSA04211

74.2 Number of Hits: 35

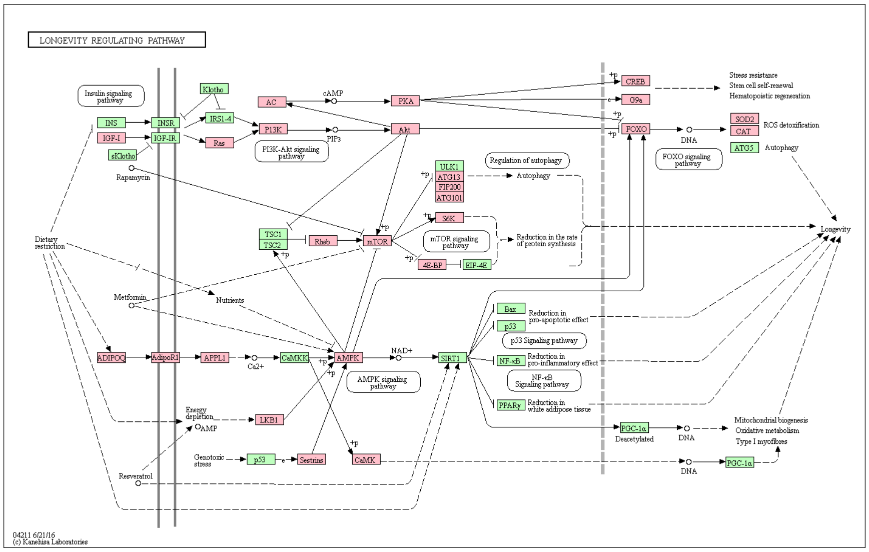

74.3 Legend:

RBH-Blast at 60% Identity + 50% Coverage
Green = Hit in *H. sapiens*
Red = Hit in *H. sapiens* and *T. californica*
White = Not in *H. sapiens*

75 Hepatitis C

75.1 Human Pathway: HSA05160

75.2 Number of Hits: 35

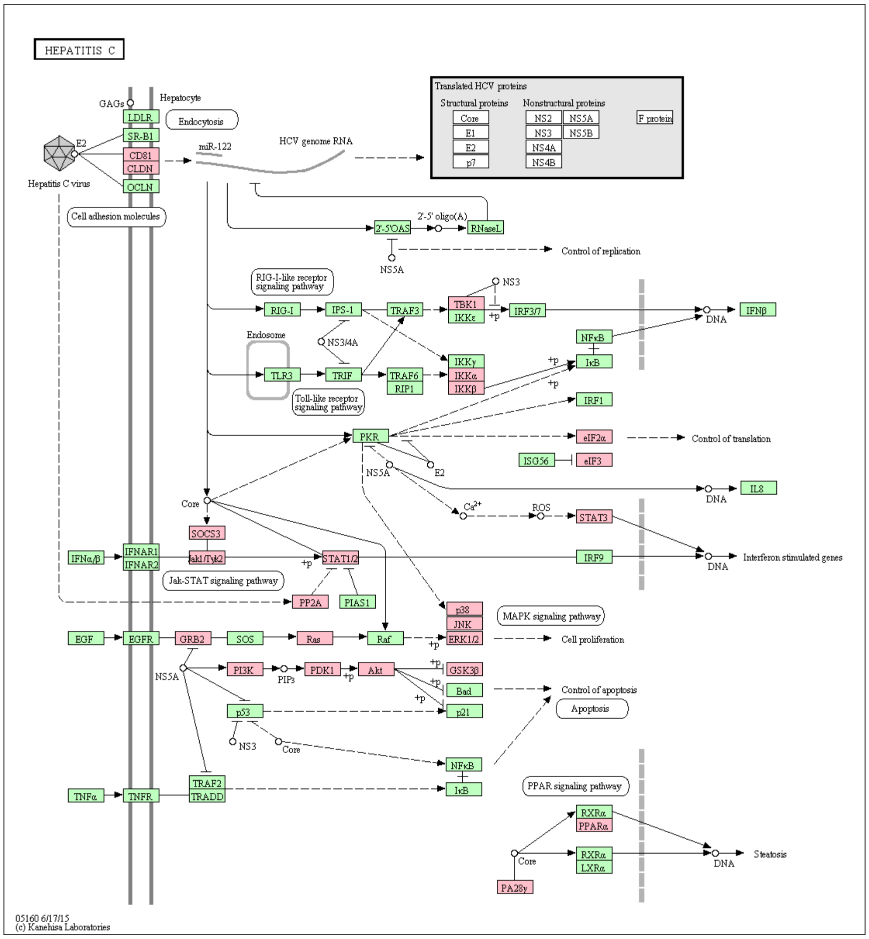

75.3 Legend:

RBH-Blast at 60% Identity + 50% Coverage
Green = Hit in *H. sapiens*
Red = Hit in *H. sapiens* and *T. californica*
White = Not in *H. sapiens*

76 Leukocyte transendothelial migration

76.1 Human Pathway: HSA04670

76.2 Number of Hits: 35

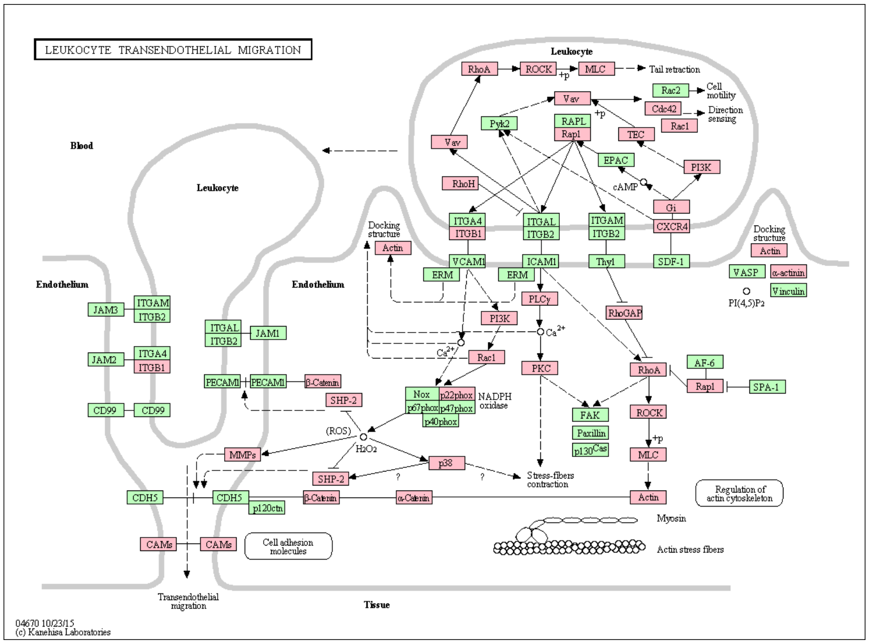

76.3 Legend:

RBH-Blast at 60% Identity + 50% Coverage
Green = Hit in *H. sapiens*
Red = Hit in *H. sapiens* and *T. californica*
White = Not in *H. sapiens*

77 Synaptic vesicle cycle

77.1 Human Pathway: HSA04721

77.2 Number of Hits: 35

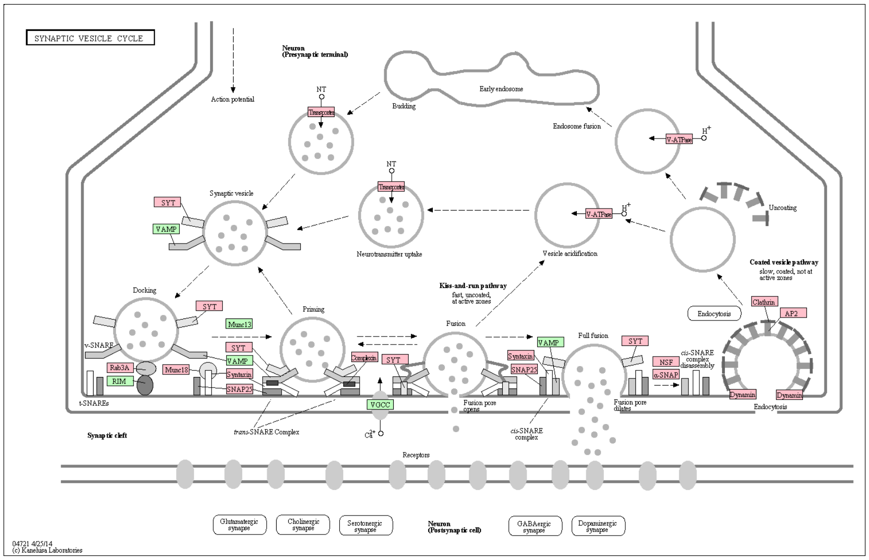

77.3 Legend:

RBH-Blast at 60% Identity + 50% Coverage
Green = Hit in *H. sapiens*
Red = Hit in *H. sapiens* and *T. californica*
White = Not in *H. sapiens*

78 Phosphatidylinositol signaling system

78.1 Human Pathway: HSA04070

78.2 Number of Hits: 35

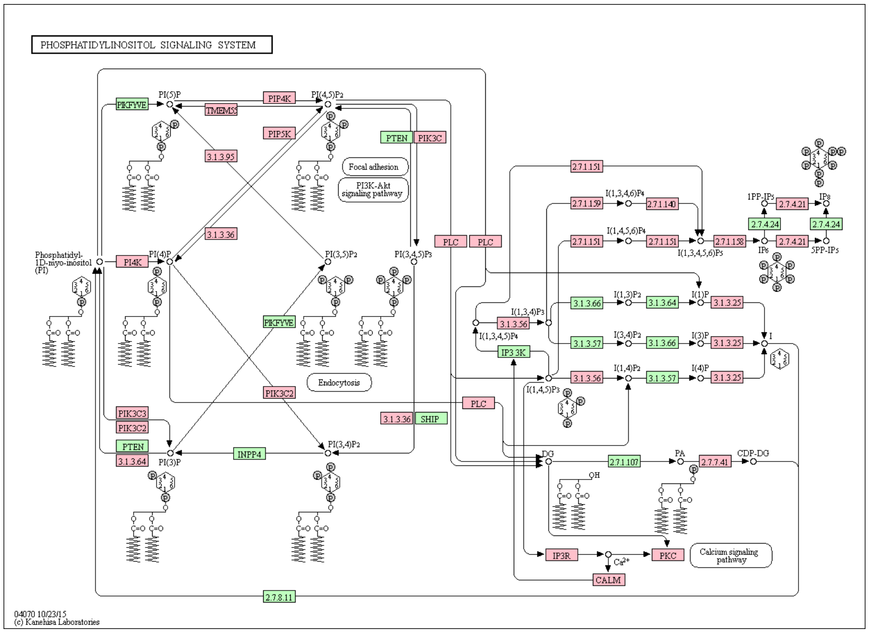

78.3 Legend:

RBH-Blast at 60% Identity + 50% Coverage
Green = Hit in *H. sapiens*
Red = Hit in *H. sapiens* and *T. californica*
White = Not in *H. sapiens*

79 Signaling pathways regulating pluripotency of stem cells

79.1 Human Pathway: HSA04550

79.2 Number of Hits: 35

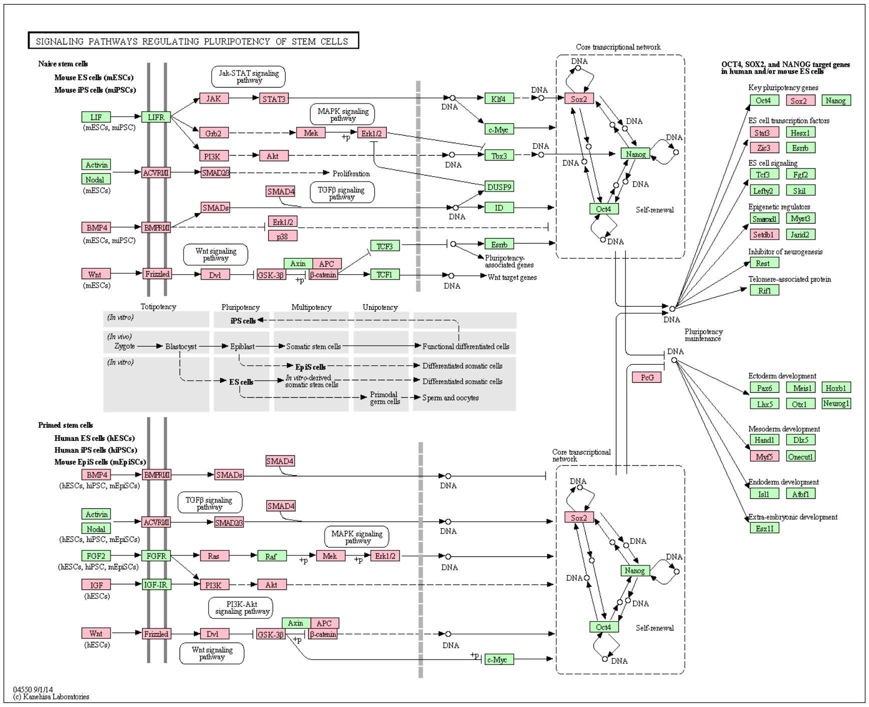

79.3 Legend:

RBH-Blast at 60% Identity + 50% Coverage
Green = Hit in *H. sapiens*
Red = Hit in *H. sapiens* and *T. californica*
White = Not in *H. sapiens*

80 Peroxisome

80.1 Human Pathway: HSA04146

80.2 Number of Hits: 34

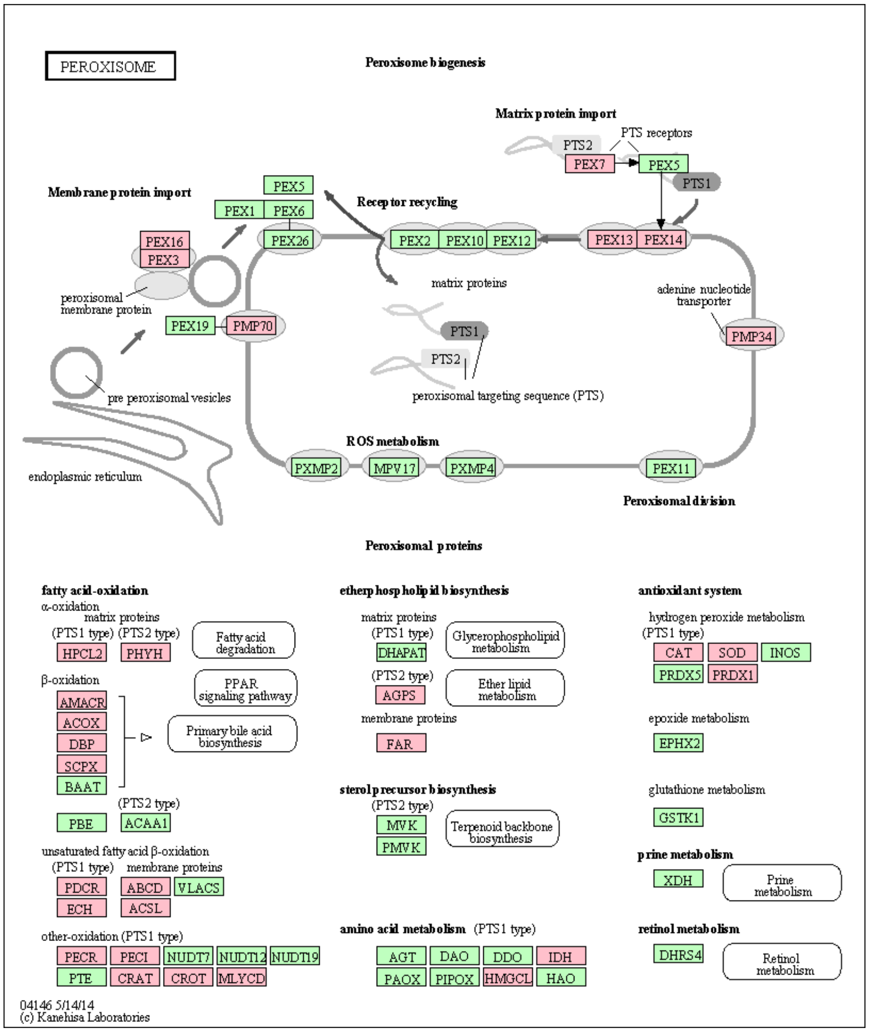

80.3 Legend:

RBH-Blast at 60% Identity + 50% Coverage
Green = Hit in *H. sapiens*
Red = Hit in *H. sapiens* and *T. californica*
White = Not in *H. sapiens*

81 Calcium signaling pathway

81.1 Human Pathway: HSA04020

81.2 Number of Hits: 34

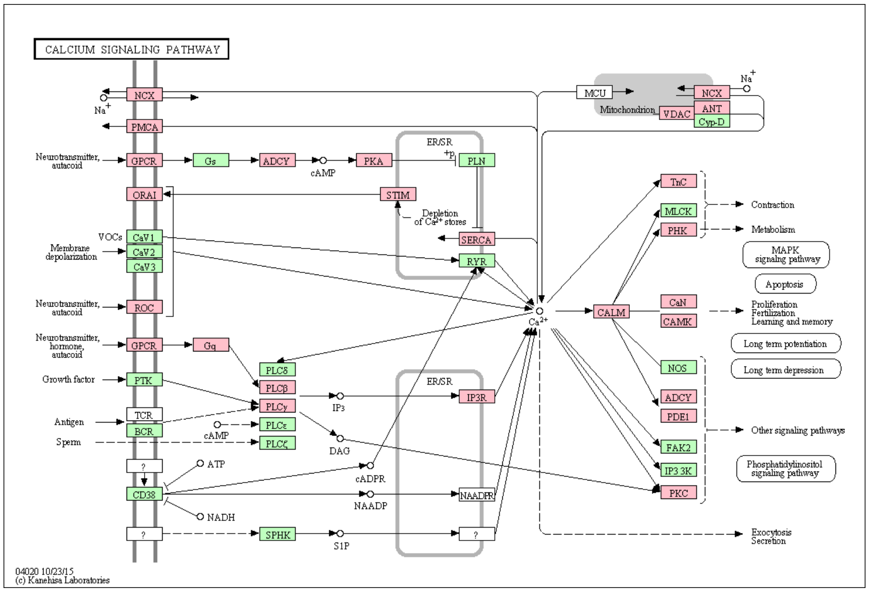

81.3 Legend:

RBH-Blast at 60% Identity + 50% Coverage
Green = Hit in *H. sapiens*
Red = Hit in *H. sapiens* and *T. californica*
White = Not in *H. sapiens*

82 AGE-RAGE signaling pathway in diabetic complications

82.1 Human Pathway: HSA04933

82.2 Number of Hits: 34

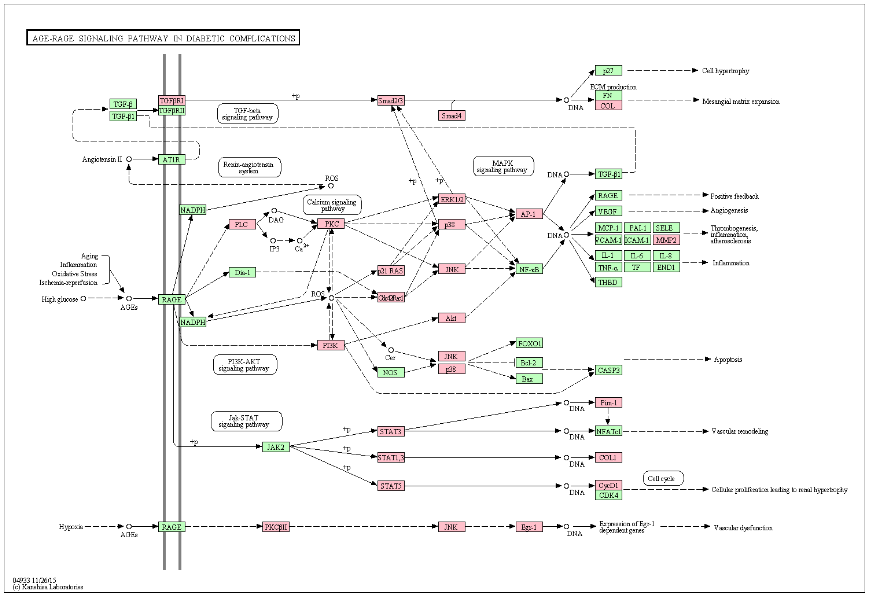

82.3 Legend:

RBH-Blast at 60% Identity + 50% Coverage
Green = Hit in *H. sapiens*
Red = Hit in *H. sapiens* and *T. californica*
White = Not in *H. sapiens*

83 Estrogen signaling pathway

83.1 Human Pathway: HSA04915

83.2 Number of Hits: 34

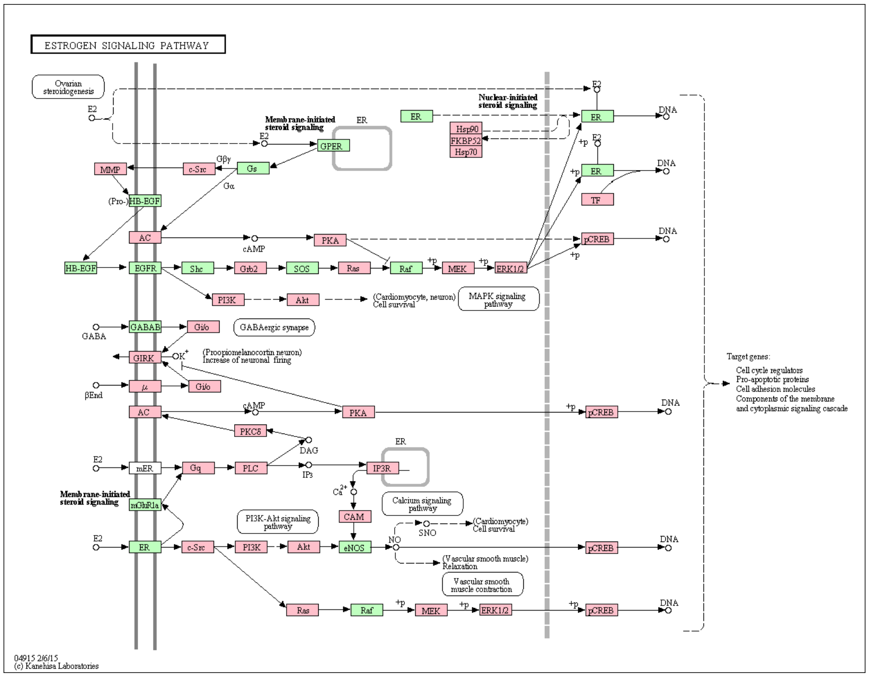

83.3 Legend:

RBH-Blast at 60% Identity + 50% Coverage
Green = Hit in *H. sapiens*
Red = Hit in *H. sapiens* and *T. californica*
White = Not in *H. sapiens*

84 Glucagon signaling pathway

84.1 Human Pathway: HSA04922

84.2 Number of Hits: 33

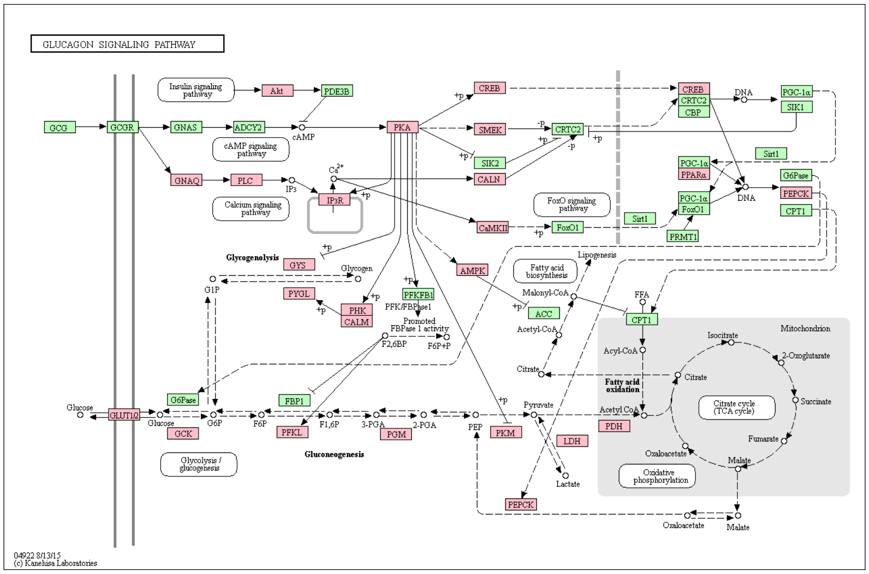

84.3 Legend:

RBH-Blast at 60% Identity + 50% Coverage
Green = Hit in *H. sapiens*
Red = Hit in *H. sapiens* and *T. californica*
White = Not in *H. sapiens*

85 HIF-1 signaling pathway

85.1 Human Pathway: HSA04066

85.2 Number of Hits: 33

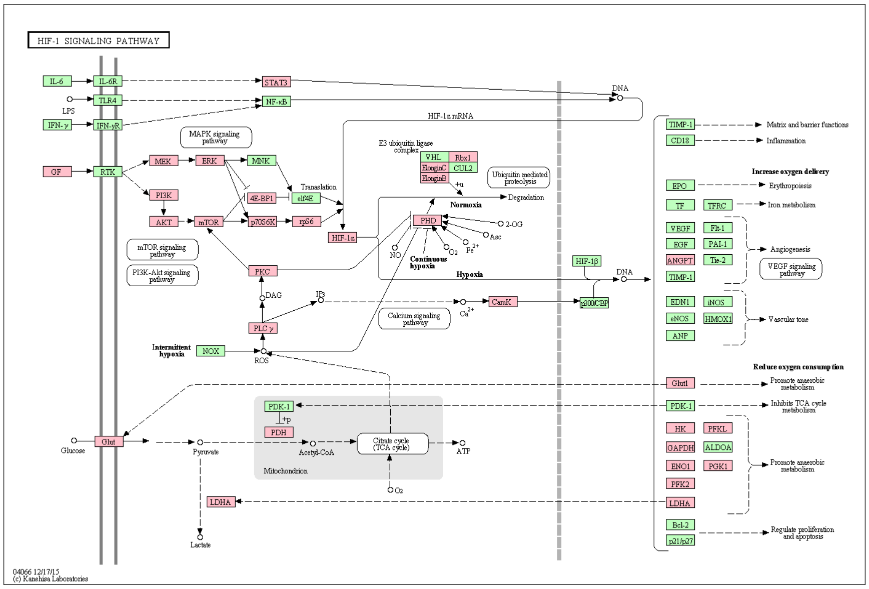

85.3 Legend:

RBH-Blast at 60% Identity + 50% Coverage
Green = Hit in *H. sapiens*
Red = Hit in *H. sapiens* and *T. californica*
White = Not in *H. sapiens*

86 Ribosome biogenesis in eukaryotes

86.1 Human Pathway: HSA03008

86.2 Number of Hits: 33

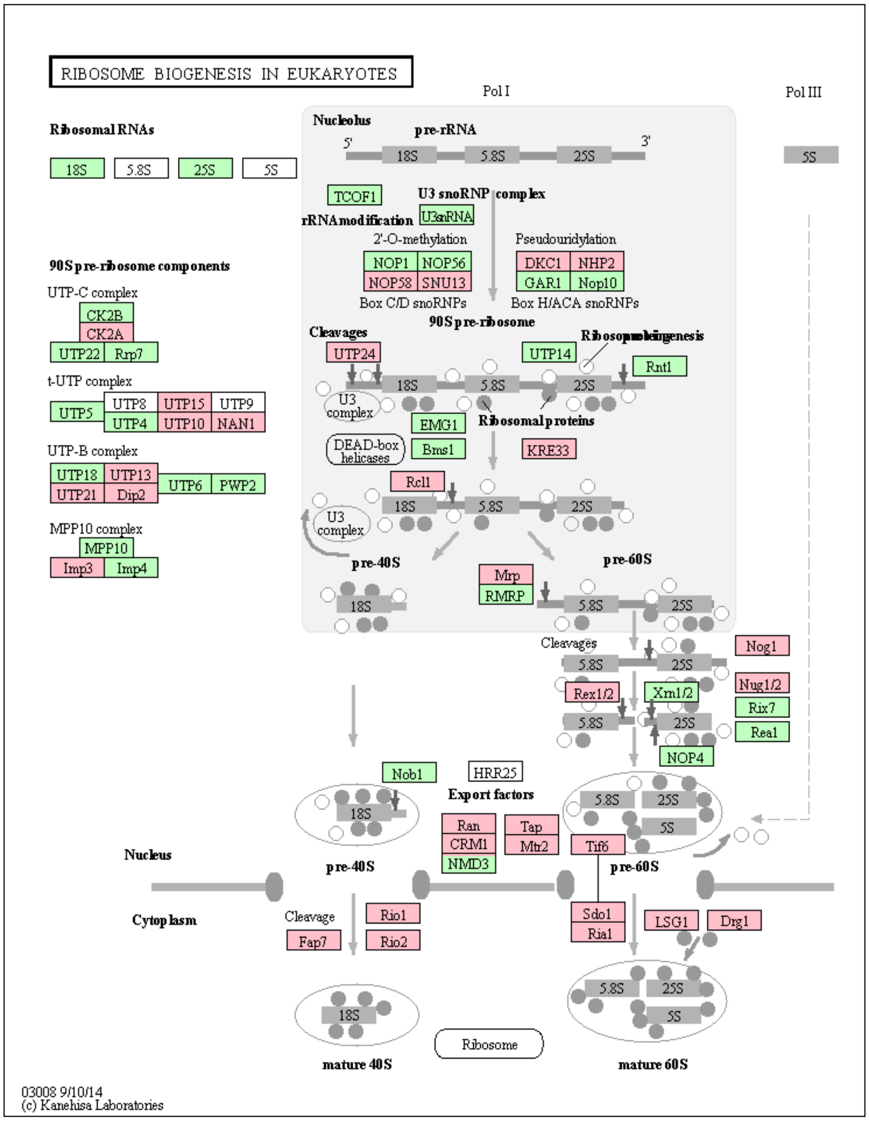

86.3 Legend:

RBH-Blast at 60% Identity + 50% Coverage
Green = Hit in *H. sapiens*
Red = Hit in *H. sapiens* and *T. californica*
White = Not in *H. sapiens*

---

87 Gap junction

87.1 Human Pathway: HSA04540

87.2 Number of Hits: 33

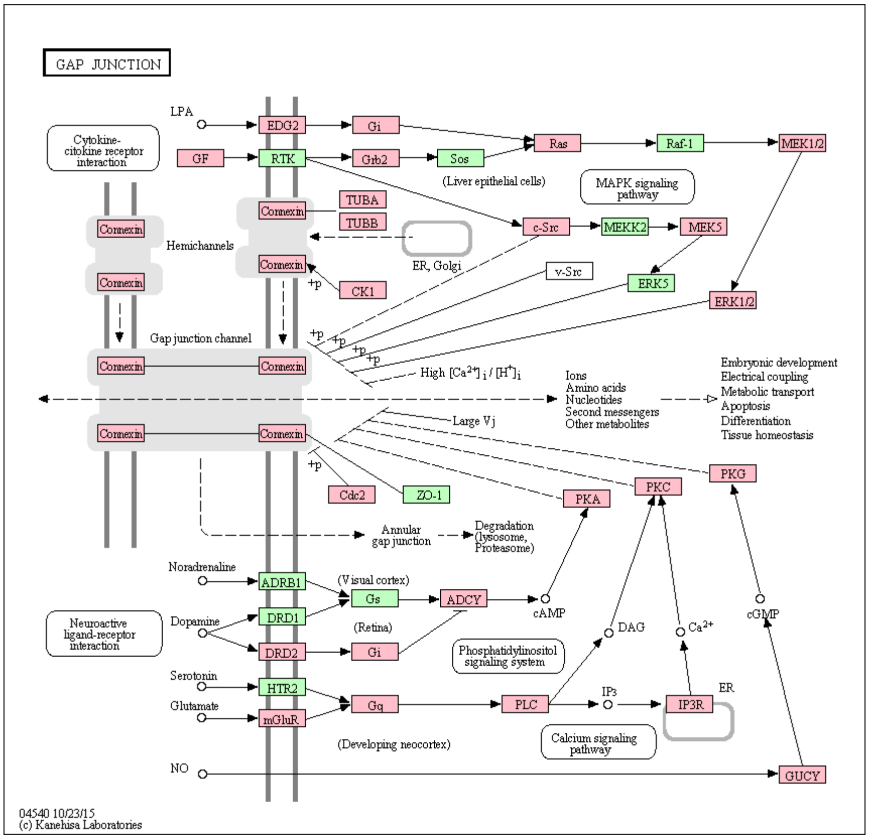

87.3 Legend:

RBH-Blast at 60% Identity + 50% Coverage
Green = Hit in *H. sapiens*
Red = Hit in *H. sapiens* and *T. californica*
White = Not in *H. sapiens*

88 Osteoclast differentiation

88.1 Human Pathway: HSA04380

88.2 Number of Hits: 32

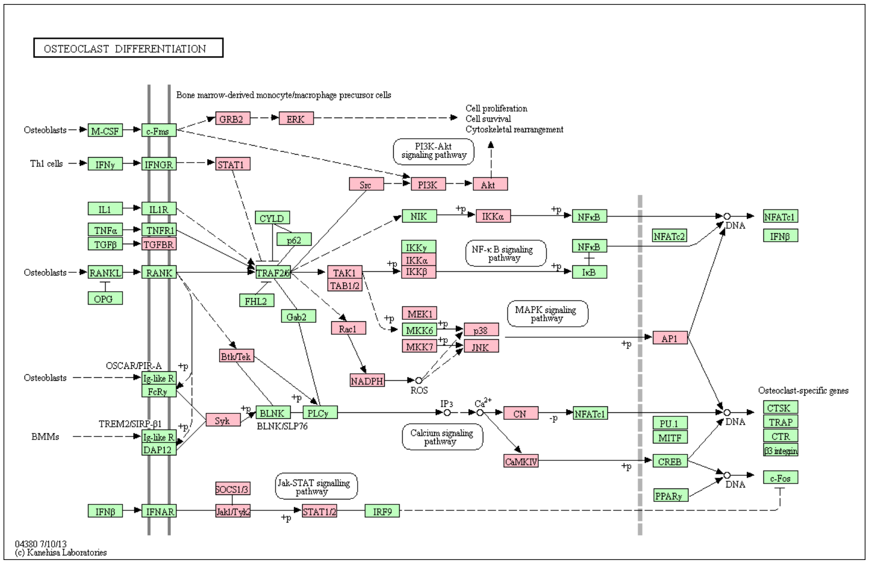

88.3 Legend:

RBH-Blast at 60% Identity + 50% Coverage
Green = Hit in *H. sapiens*
Red = Hit in *H. sapiens* and *T. californica*
White = Not in *H. sapiens*

89 Valine, leucine and isoleucine degradation

89.1 Human Pathway: HSA00280

89.2 Number of Hits: 32

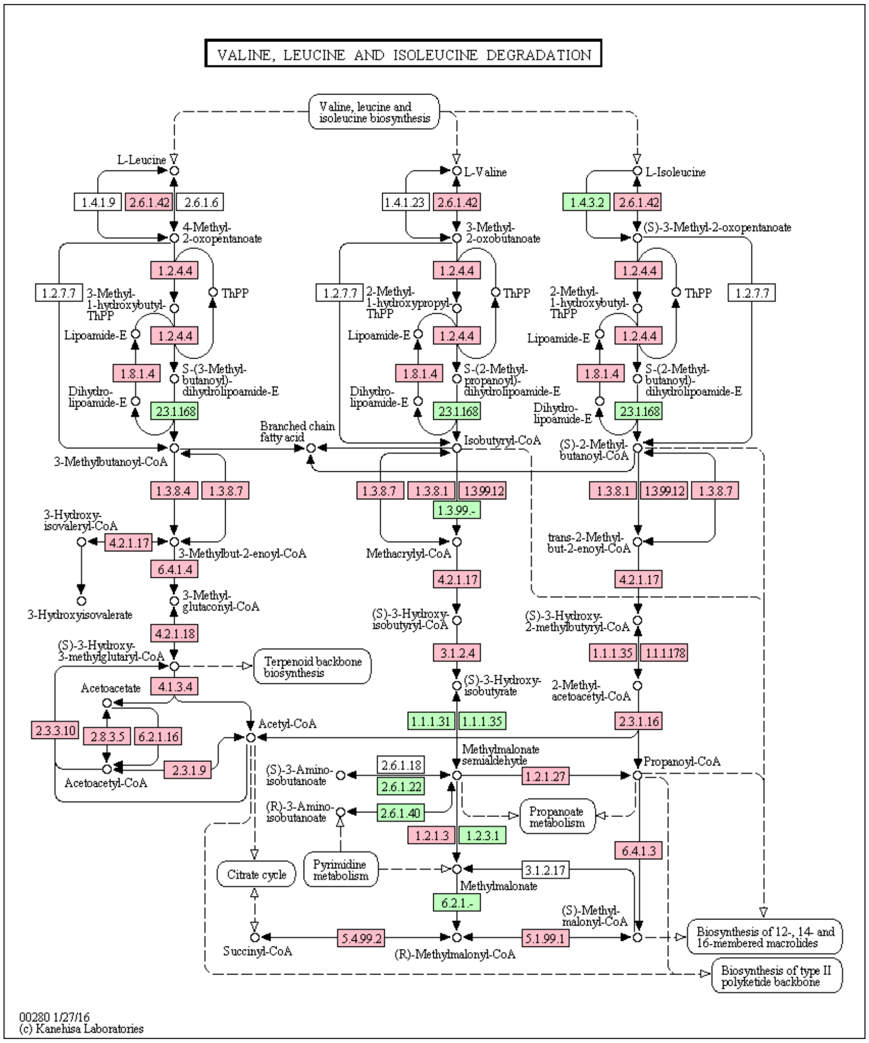

89.3 Legend:

RBH-Blast at 60% Identity + 50% Coverage
Green = Hit in *H. sapiens*
Red = Hit in *H. sapiens* and *T. californica*
White = Not in *H. sapiens*

---

90 Salmonella infection

90.1 Human Pathway: HSA05132

90.2 Number of Hits: 32

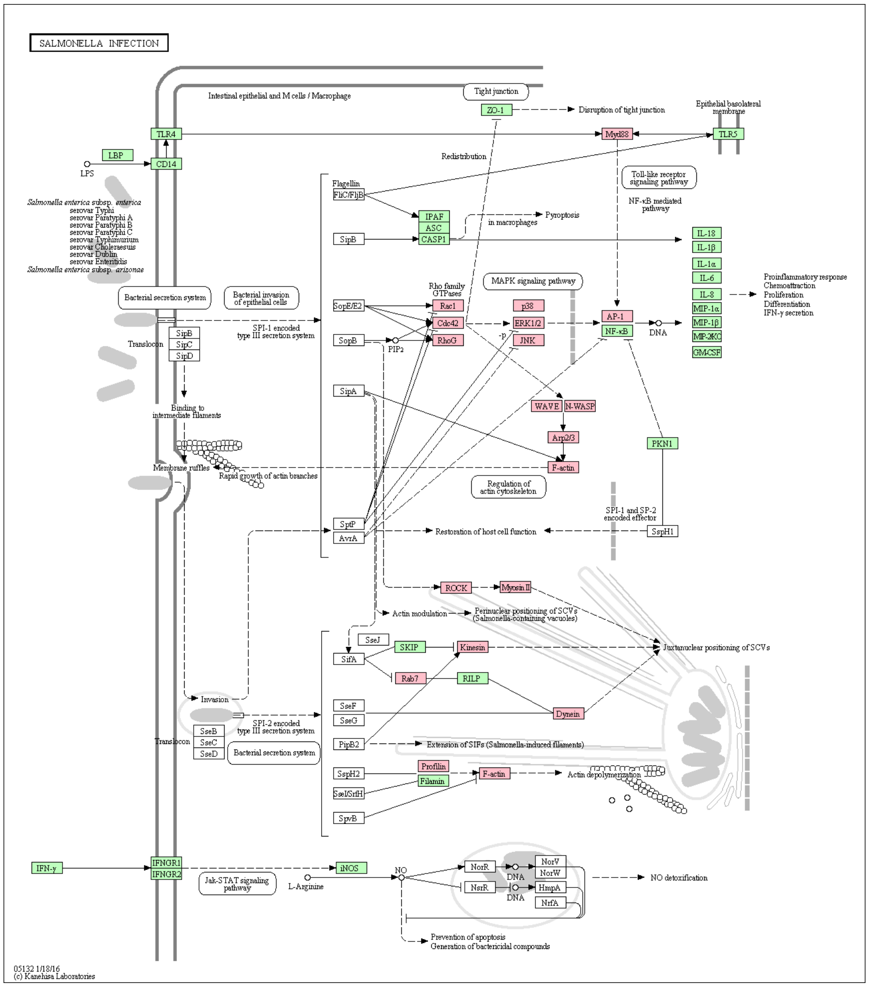

90.3 Legend:

RBH-Blast at 60% Identity + 50% Coverage
Green = Hit in *H. sapiens*
Red = Hit in *H. sapiens* and *T. californica*
White = Not in *H. sapiens*

---

91 Epithelial cell signaling in Helicobacter pylori infection

91.1 Human Pathway: HSA05120

91.2 Number of Hits: 31

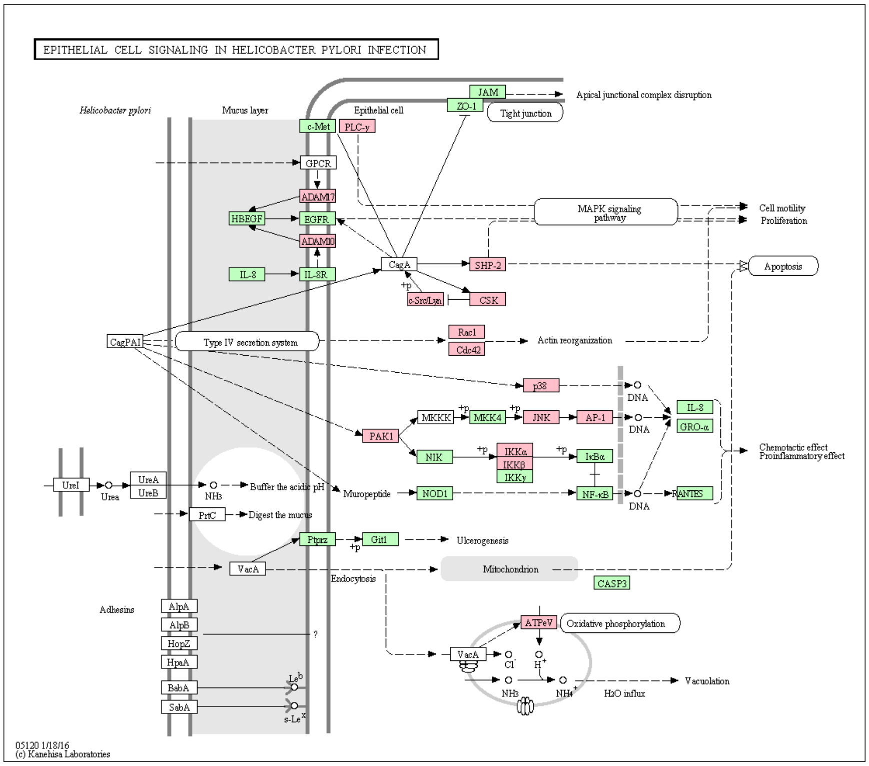

91.3 Legend:

RBH-Blast at 60% Identity + 50% Coverage
Green = Hit in *H. sapiens*
Red = Hit in *H. sapiens* and *T. californica*
White = Not in *H. sapiens*

92 Toxoplasmosis

92.1 Human Pathway: HSA05145

92.2 Number of Hits: 31

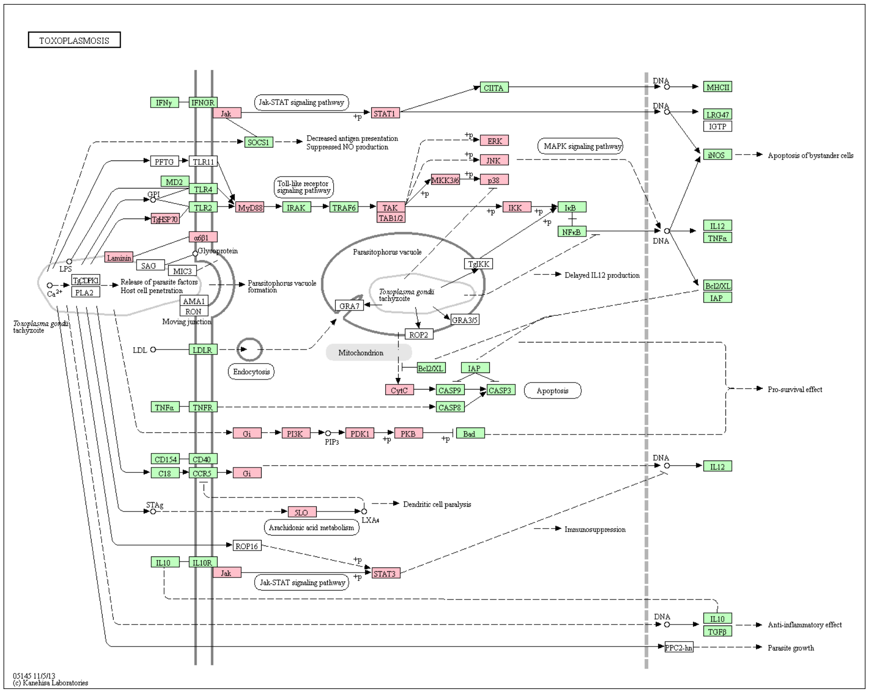

92.3 Legend:

RBH-Blast at 60% Identity + 50% Coverage
Green = Hit in *H. sapiens*
Red = Hit in *H. sapiens* and *T. californica*
White = Not in *H. sapiens*

93 ErbB signaling pathway

93.1 Human Pathway: HSA04012

93.2 Number of Hits: 31

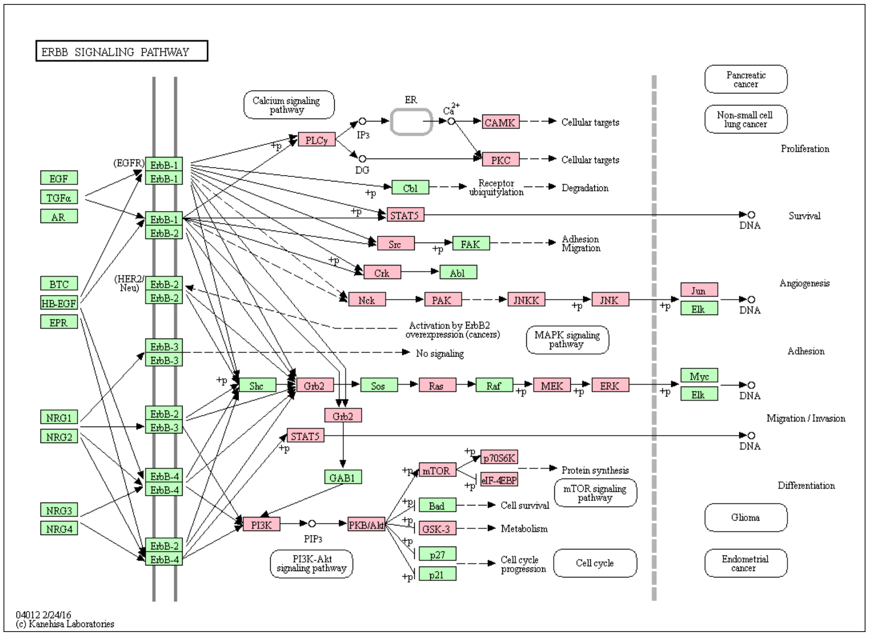

93.3 Legend:

RBH-Blast at 60% Identity + 50% Coverage
Green = Hit in *H. sapiens*
Red = Hit in *H. sapiens* and *T. californica*
White = Not in *H. sapiens*

94 GnRH signaling pathway

94.1 Human Pathway: HSA04912

94.2 Number of Hits: 31

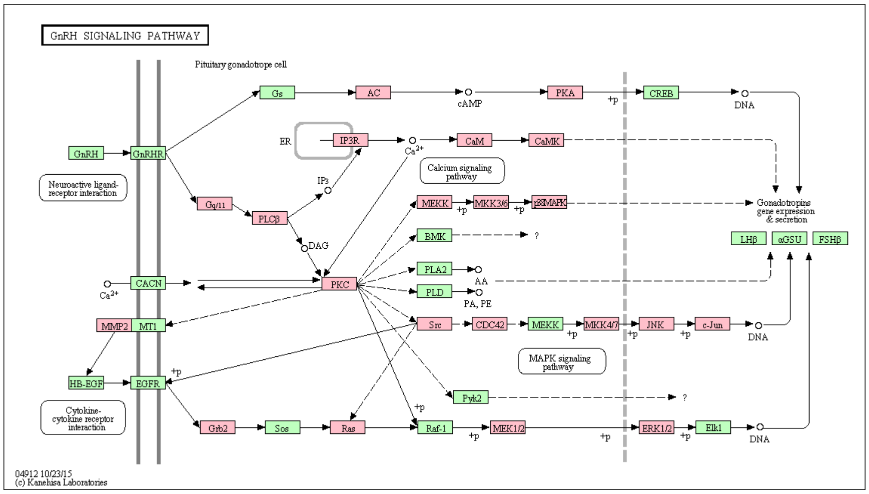

94.3 Legend:

RBH-Blast at 60% Identity + 50% Coverage
Green = Hit in *H. sapiens*
Red = Hit in *H. sapiens* and *T. californica*
White = Not in *H. sapiens*

95 Vascular smooth muscle contraction

95.1 Human Pathway: HSA04270

95.2 Number of Hits: 31

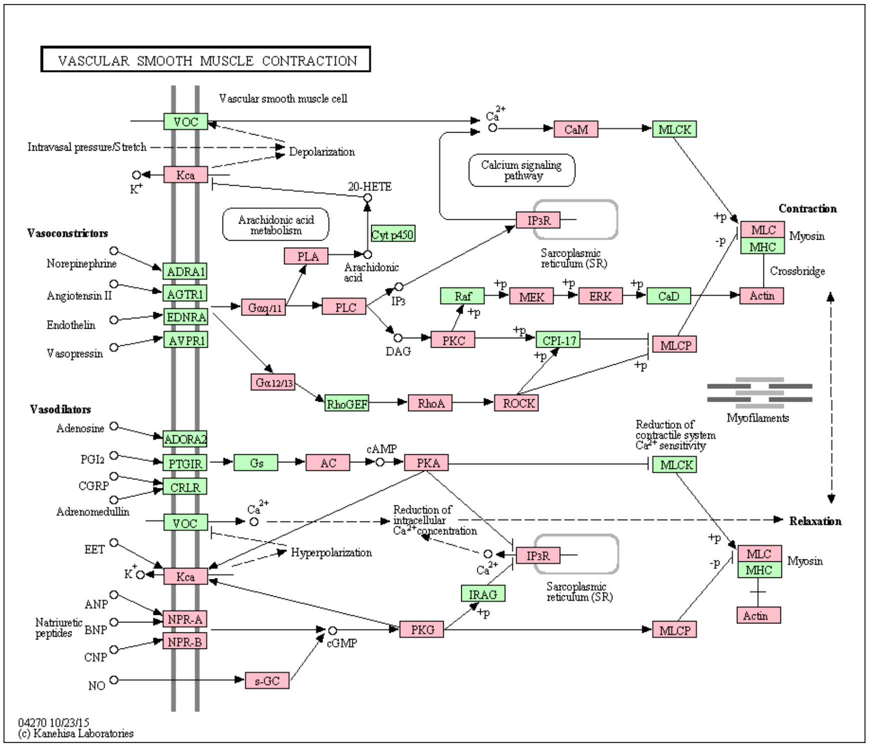

95.3 Legend:

RBH-Blast at 60% Identity + 50% Coverage
Green = Hit in *H. sapiens*
Red = Hit in *H. sapiens* and *T. californica*
White = Not in *H. sapiens*

96 Glycolysis / Gluconeogenesis

96.1 Human Pathway: HSA00010

96.2 Number of Hits: 30

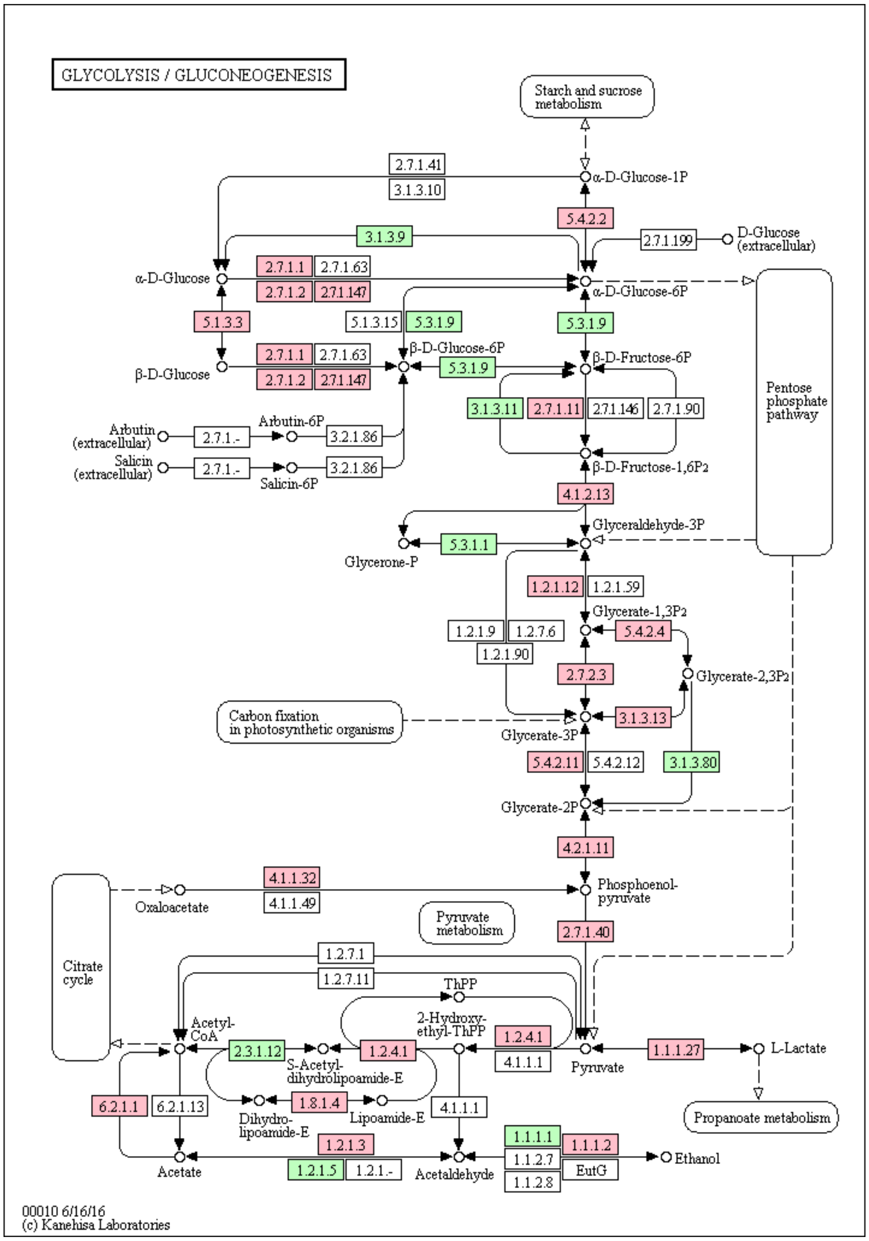

96.3 Legend:

RBH-Blast at 60% Identity + 50% Coverage
Green = Hit in *H. sapiens*
Red = Hit in *H. sapiens* and *T. californica*
White = Not in *H. sapiens*

97 Renal cell carcinoma

97.1 Human Pathway: HSA05211

97.2 Number of Hits: 30

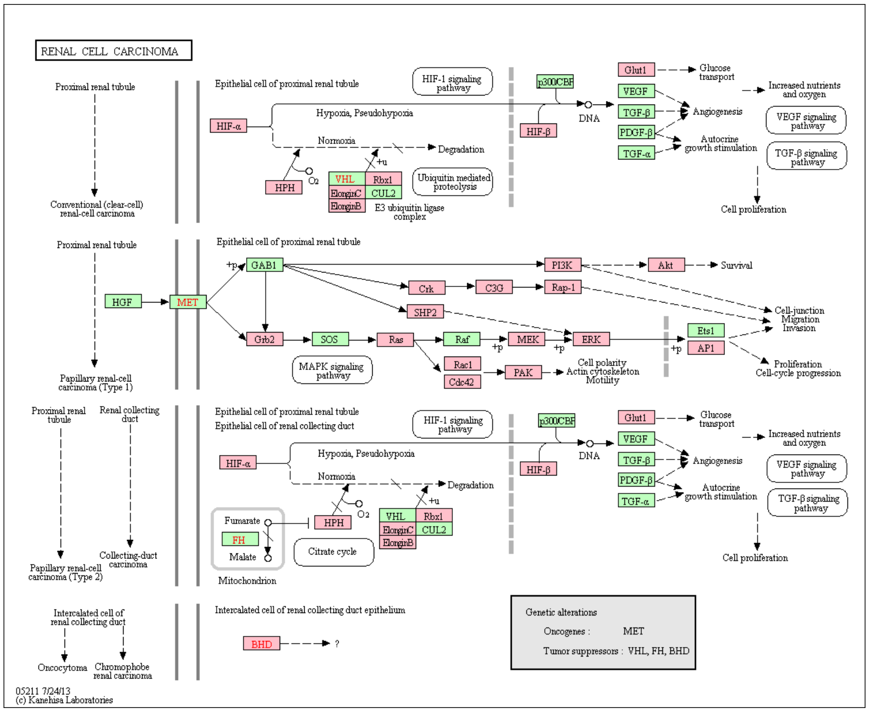

97.3 Legend:

RBH-Blast at 60% Identity + 50% Coverage
Green = Hit in *H. sapiens*
Red = Hit in *H. sapiens* and *T. californica*
White = Not in *H. sapiens*

98 Aminoacyl-tRNA biosynthesis

98.1 Human Pathway: HSA00970

98.2 Number of Hits: 29

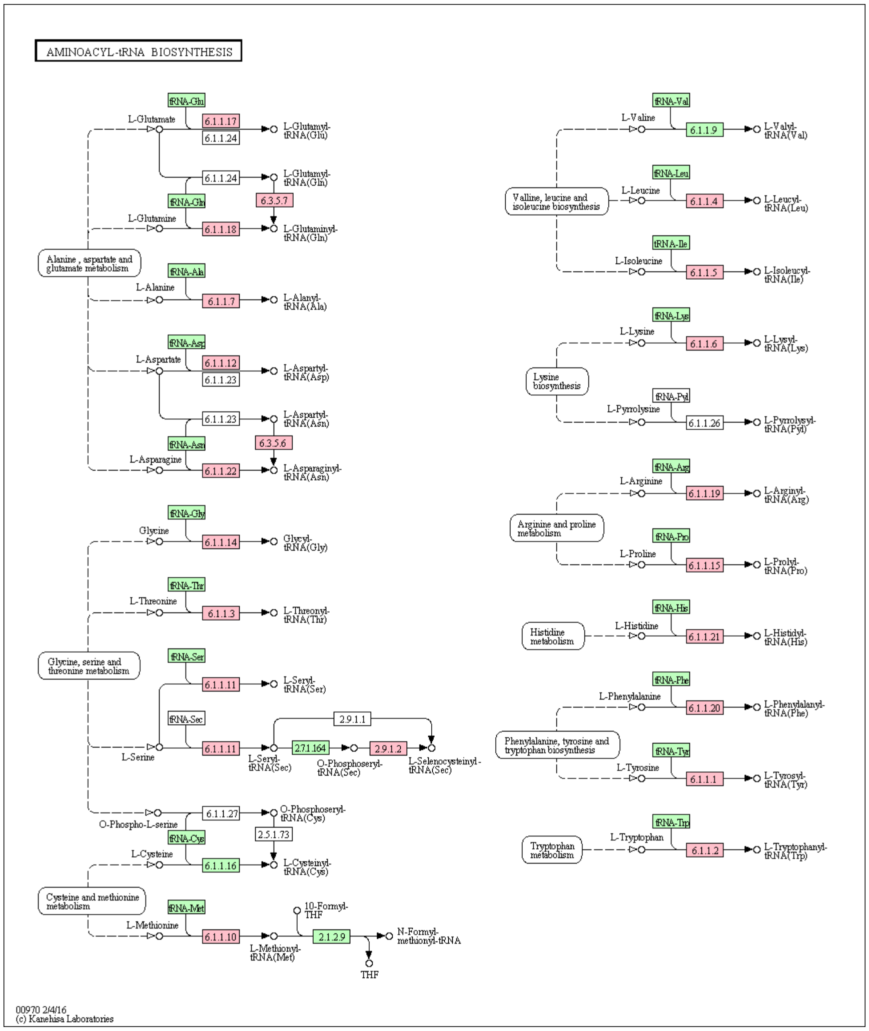

98.3 Legend:

RBH-Blast at 60% Identity + 50% Coverage
Green = Hit in *H. sapiens*
Red = Hit in *H. sapiens* and *T. californica*
White = Not in *H. sapiens*

99 Basal transcription factors

99.1 Human Pathway: HSA03022

99.2 Number of Hits: 28

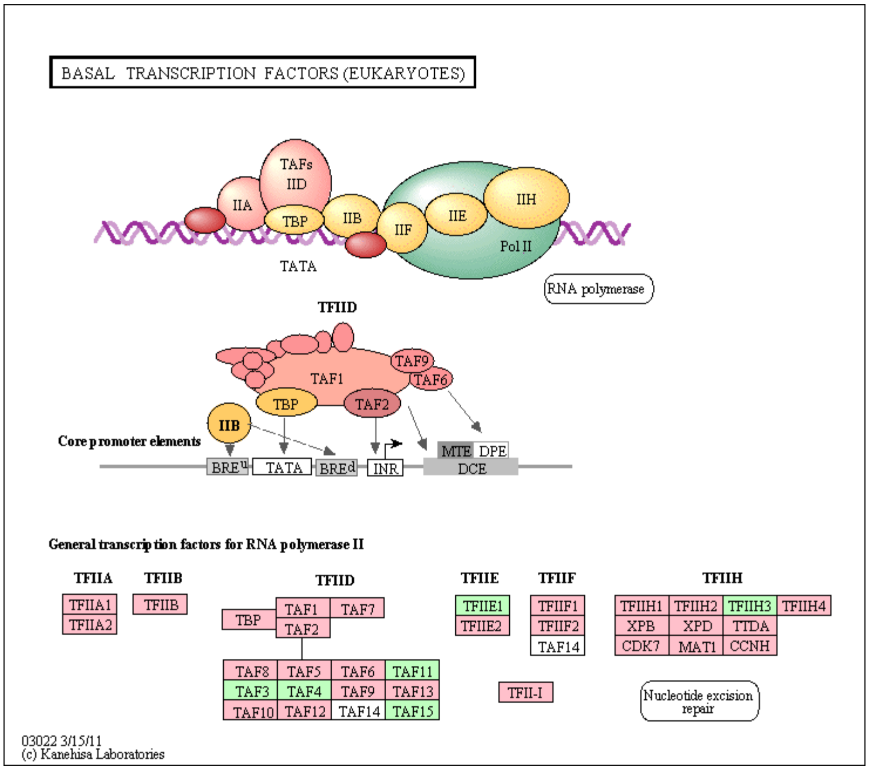

99.3 Legend:

RBH-Blast at 60% Identity + 50% Coverage
Green = Hit in *H. sapiens*
Red = Hit in *H. sapiens* and *T. californica*
White = Not in *H. sapiens*

100 Proteasome

100.1 Human Pathway: HSA03050

100.2 Number of Hits: 28

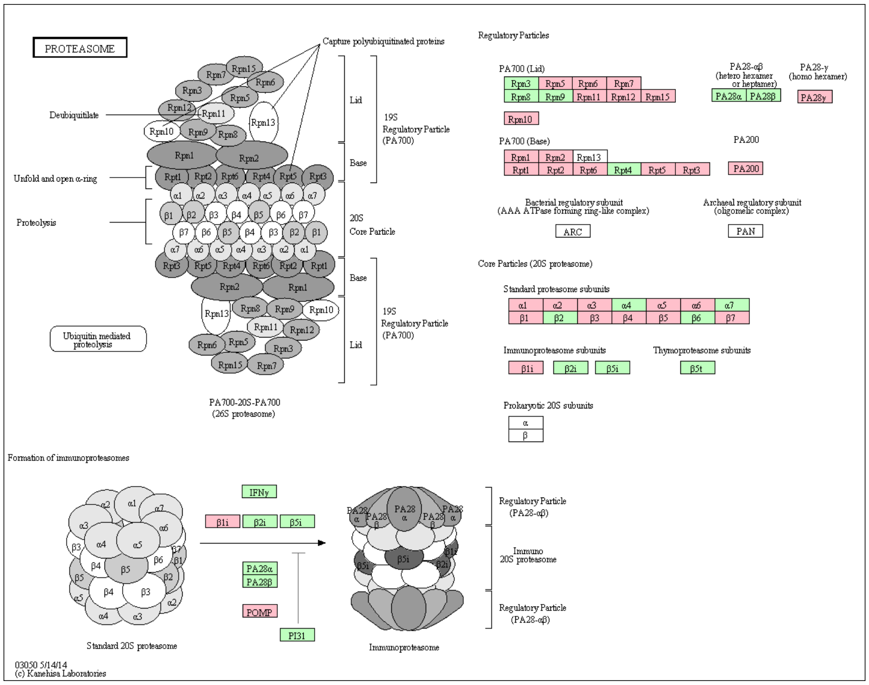

100.3 Legend:

RBH-Blast at 60% Identity + 50% Coverage
Green = Hit in *H. sapiens*
Red = Hit in *H. sapiens* and *T. californica*
White = Not in *H. sapiens*

101 Fc epsilon RI signaling pathway

101.1 Human Pathway: HSA04664

101.2 Number of Hits: 28

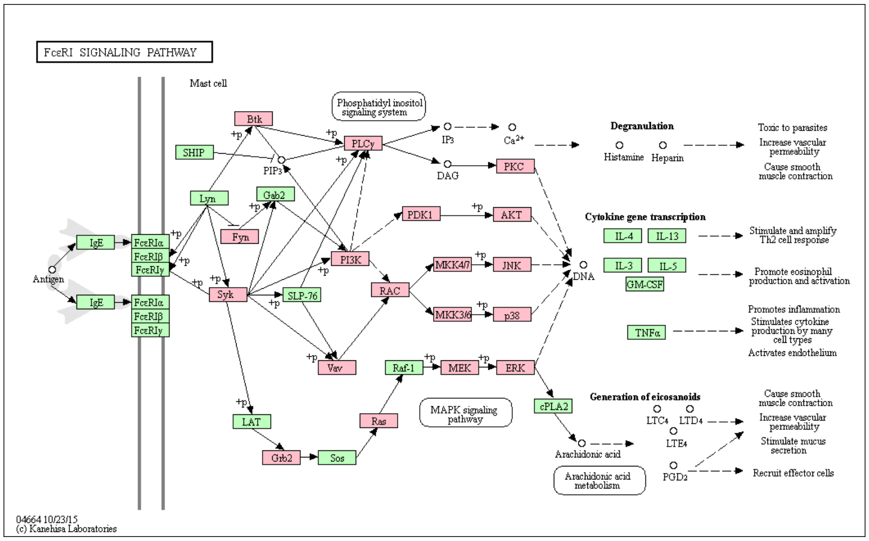

101.3 Legend:

RBH-Blast at 60% Identity + 50% Coverage
Green = Hit in *H. sapiens*
Red = Hit in *H. sapiens* and *T. californica*
White = Not in *H. sapiens*

102 Chagas disease (American trypanosomiasis)

102.1 Human Pathway: HSA05142

102.2 Number of Hits: 28

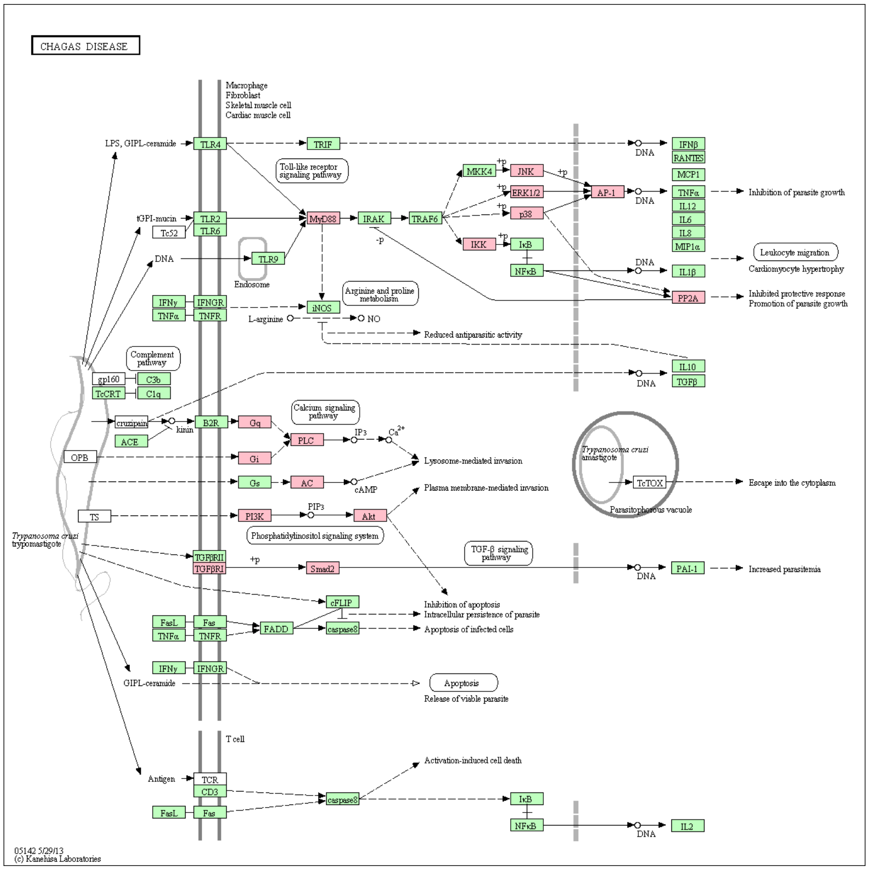

102.3 Legend:

RBH-Blast at 60% Identity + 50% Coverage
Green = Hit in *H. sapiens*
Red = Hit in *H. sapiens* and *T. californica*
White = Not in *H. sapiens*

103 Adherens junction

103.1 Human Pathway: HSA04520

103.2 Number of Hits: 28

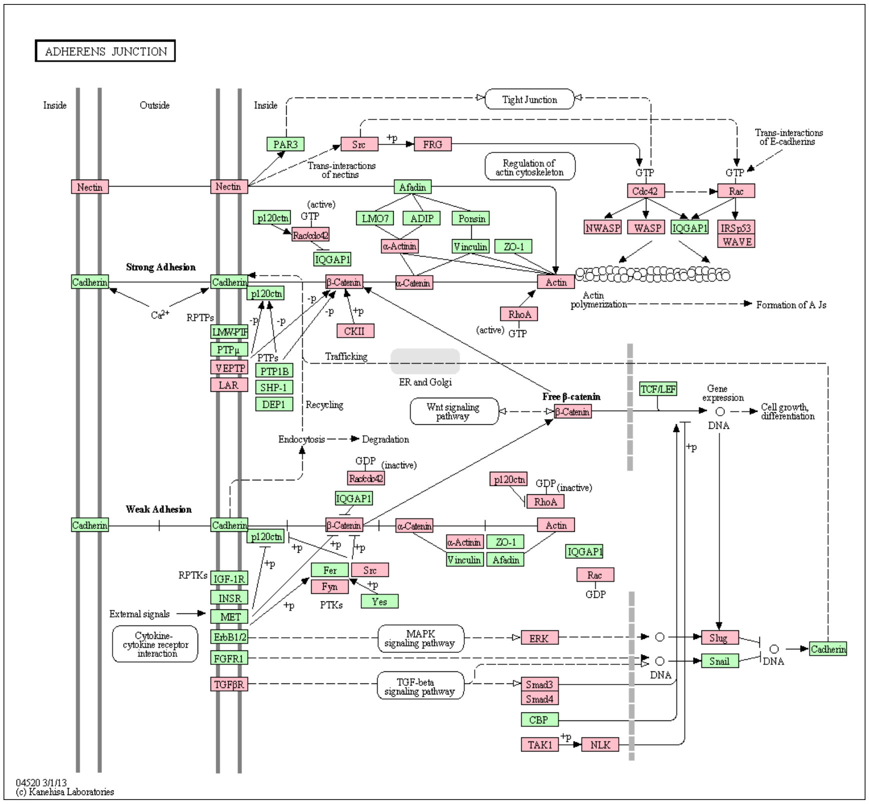

103.3 Legend:

RBH-Blast at 60% Identity + 50% Coverage
Green = Hit in *H. sapiens*
Red = Hit in *H. sapiens* and *T. californica*
White = Not in *H. sapiens*

104 Long-term potentiation

104.1 Human Pathway: HSA04720

104.2 Number of Hits: 28

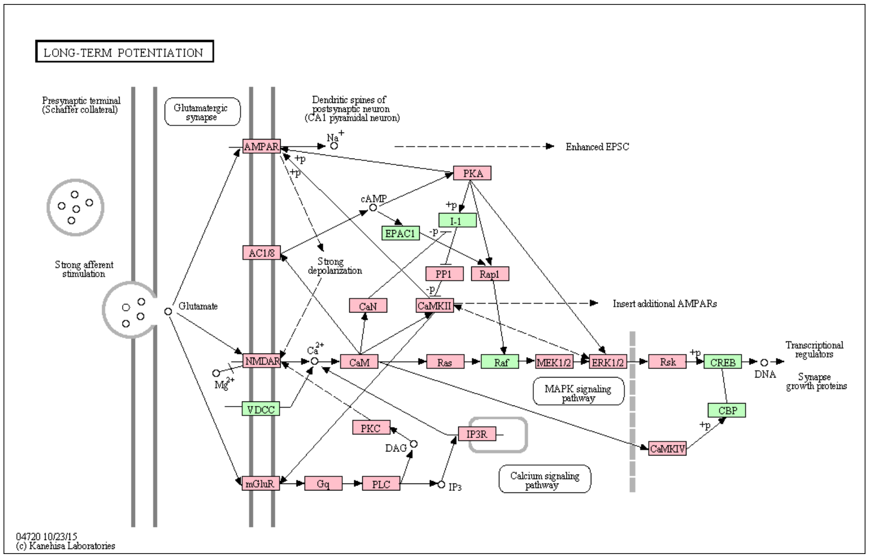

104.3 Legend:

RBH-Blast at 60% Identity + 50% Coverage
Green = Hit in *H. sapiens*
Red = Hit in *H. sapiens* and *T. californica*
White = Not in *H. sapiens*

105 B cell receptor signaling pathway

105.1 Human Pathway: HSA04662

105.2 Number of Hits: 28

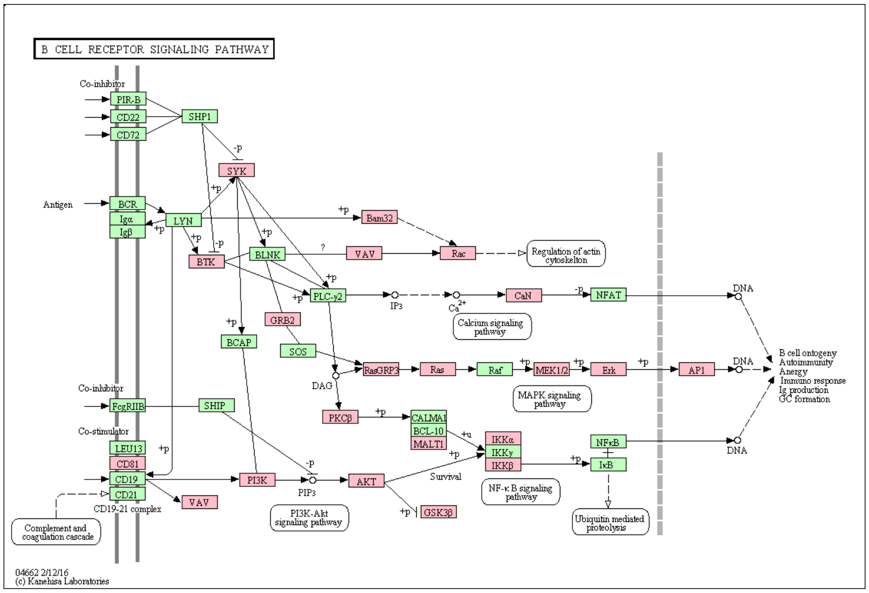

105.3 Legend:

RBH-Blast at 60% Identity + 50% Coverage
Green = Hit in *H. sapiens*
Red = Hit in *H. sapiens* and *T. californica*
White = Not in *H. sapiens*

106 Pancreatic cancer

106.1 Human Pathway: HSA05212

106.2 Number of Hits: 27

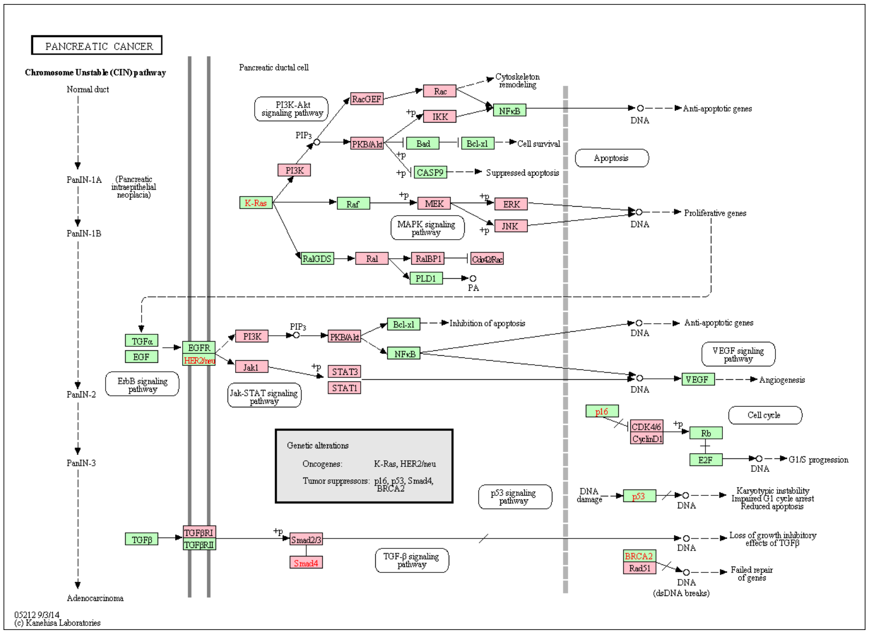

106.3 Legend:

RBH-Blast at 60% Identity + 50% Coverage
Green = Hit in *H. sapiens*
Red = Hit in *H. sapiens* and *T. californica*
White = Not in *H. sapiens*

107 Prolactin signaling pathway

107.1 Human Pathway: HSA04917

107.2 Number of Hits: 27

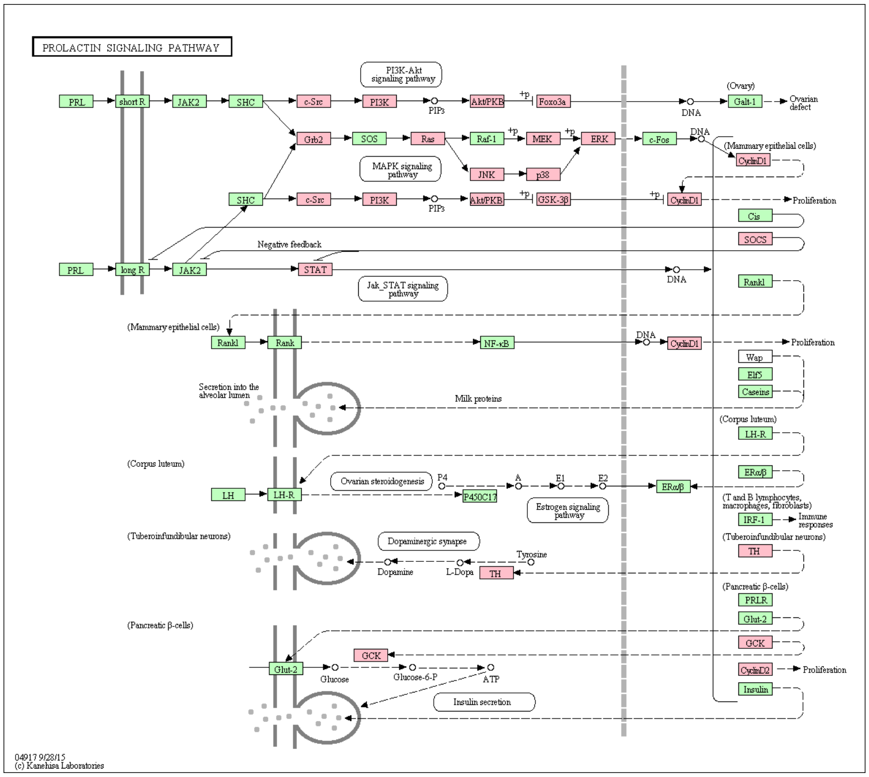

107.3 Legend:

RBH-Blast at 60% Identity + 50% Coverage
Green = Hit in *H. sapiens*
Red = Hit in *H. sapiens* and *T. californica*
White = Not in *H. sapiens*

108 Inflammatory mediator regulation of TRP channels

108.1 Human Pathway: HSA04750

108.2 Number of Hits: 27

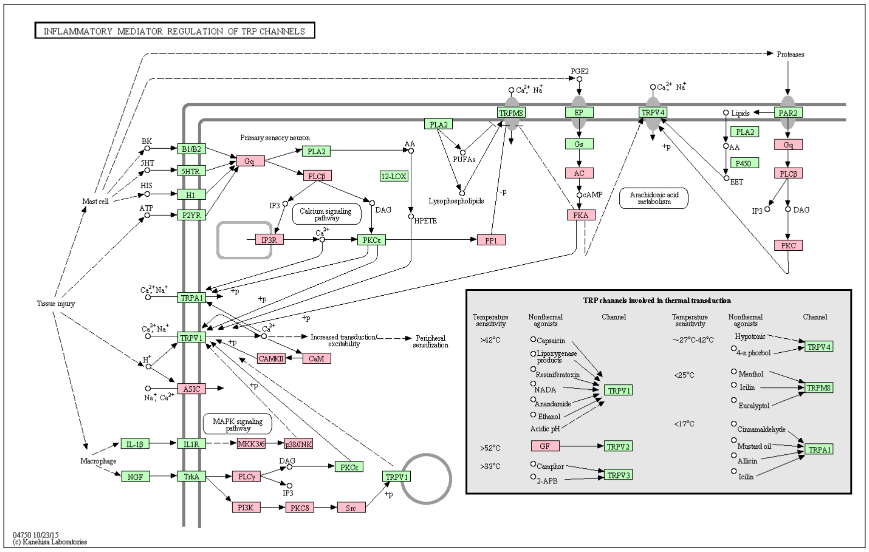

108.3 Legend:

RBH-Blast at 60% Identity + 50% Coverage
Green = Hit in *H. sapiens*
Red = Hit in *H. sapiens* and *T. californica*
White = Not in *H. sapiens*

109 Glycerophospholipid metabolism

109.1 Human Pathway: HSA00564

109.2 Number of Hits: 27

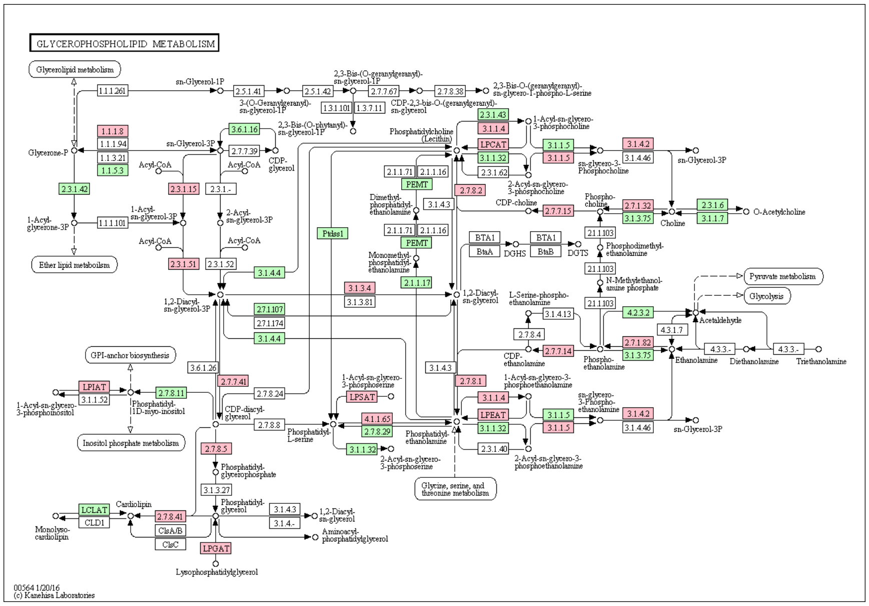

109.3 Legend:

RBH-Blast at 60% Identity + 50% Coverage
Green = Hit in *H. sapiens*
Red = Hit in *H. sapiens* and *T. californica*
White = Not in *H. sapiens*

110 Long-term depression

110.1 Human Pathway: HSA04730

110.2 Number of Hits: 26

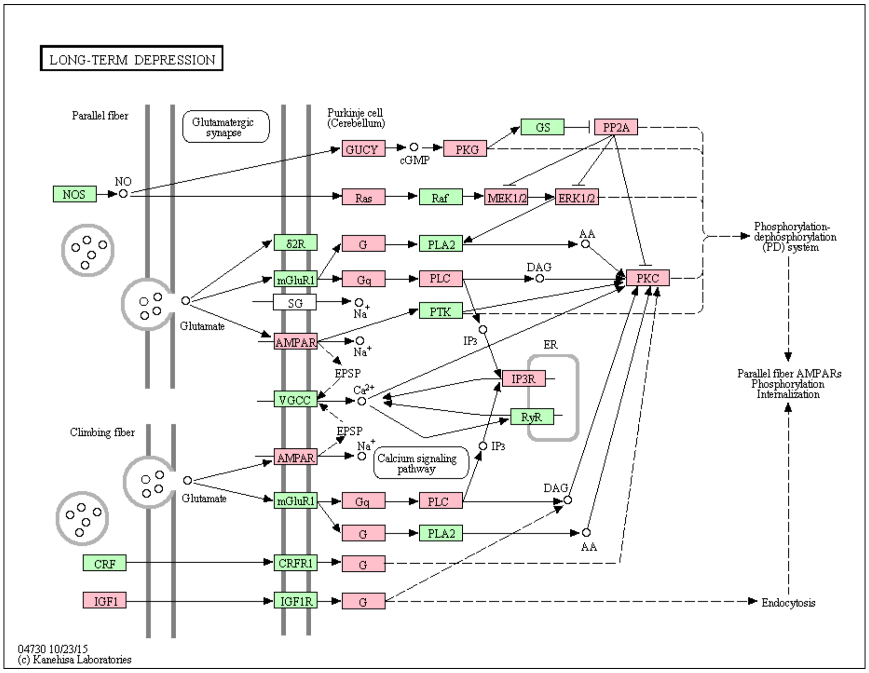

110.3 Legend:

RBH-Blast at 60% Identity + 50% Coverage
Green = Hit in *H. sapiens*
Red = Hit in *H. sapiens* and *T. californica*
White = Not in *H. sapiens*

111 Central carbon metabolism in cancer

111.1 Human Pathway: HSA05230

111.2 Number of Hits: 26

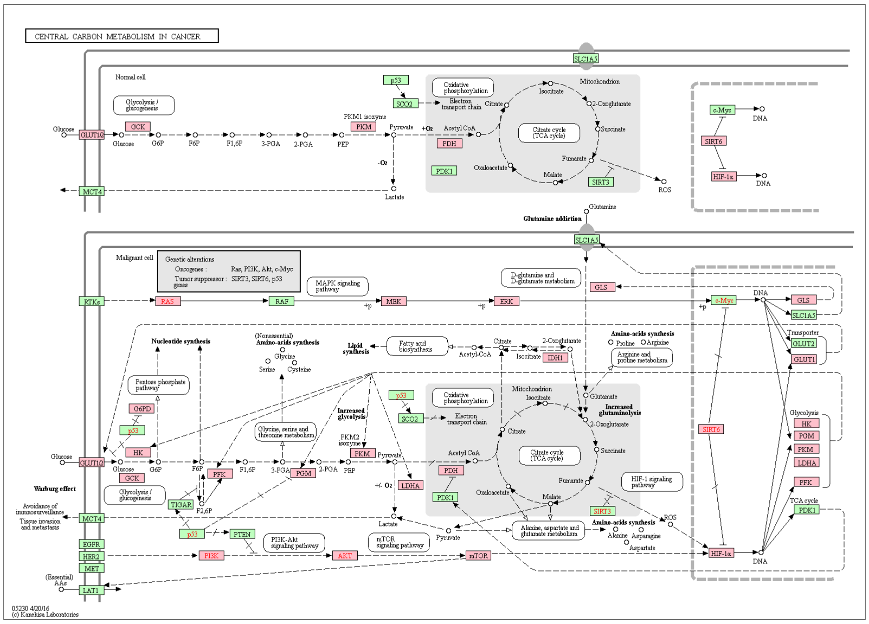

111.3 Legend:

RBH-Blast at 60% Identity + 50% Coverage
Green = Hit in *H. sapiens*
Red = Hit in *H. sapiens* and *T. californica*
White = Not in *H. sapiens*

112 Melanogenesis

112.1 Human Pathway: HSA04916

112.2 Number of Hits: 26

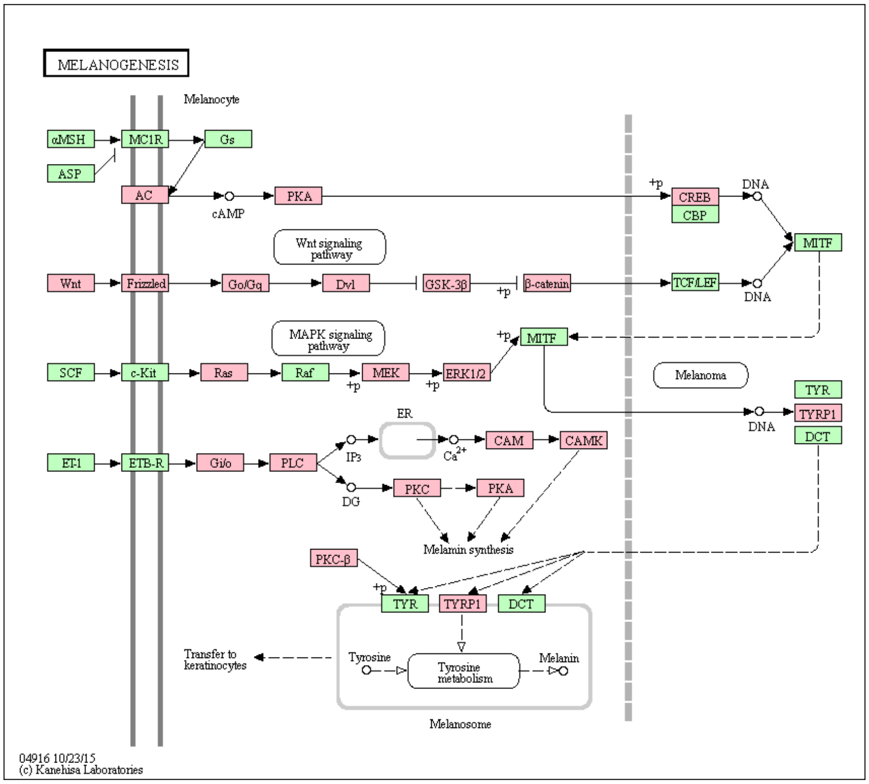

112.3 Legend:

RBH-Blast at 60% Identity + 50% Coverage
Green = Hit in *H. sapiens*
Red = Hit in *H. sapiens* and *T. californica*
White = Not in *H. sapiens*

113 Transcriptional misregulation in cancer

113.1 Human Pathway: HSA05202

113.2 Number of Hits: 26

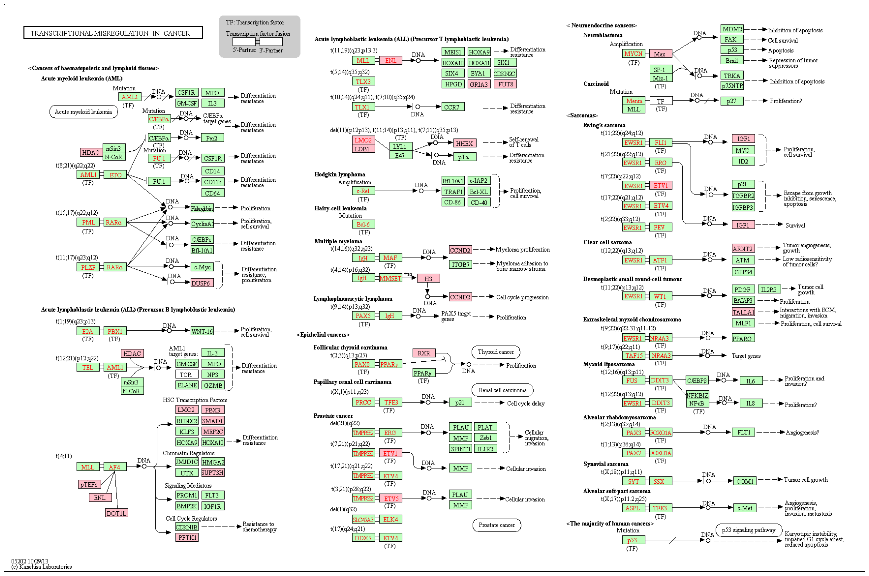

113.3 Legend:

RBH-Blast at 60% Identity + 50% Coverage
Green = Hit in *H. sapiens*
Red = Hit in *H. sapiens* and *T. californica*
White = Not in *H. sapiens*

114 Dilated cardiomyopathy

114.1 Human Pathway: HSA05414

114.2 Number of Hits: 26

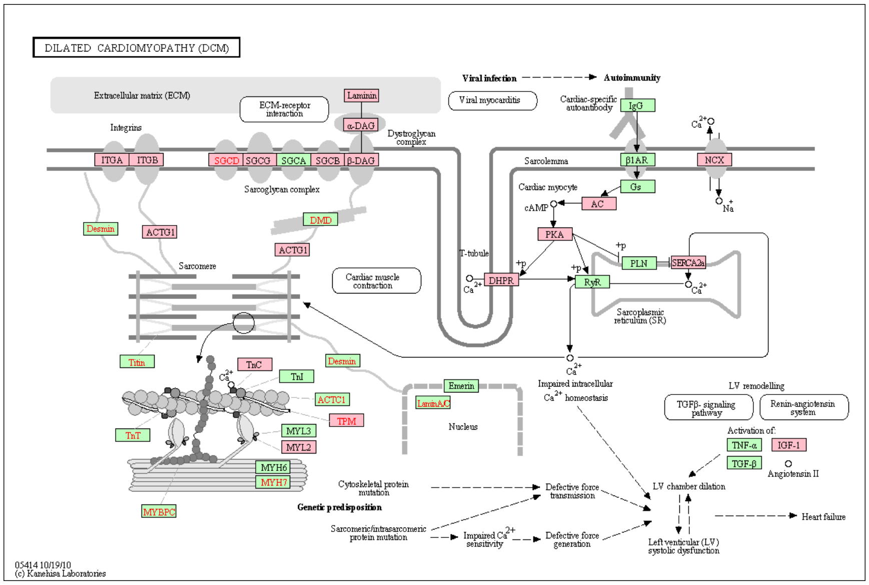

114.3 Legend:

RBH-Blast at 60% Identity + 50% Coverage
Green = Hit in *H. sapiens*
Red = Hit in *H. sapiens* and *T. californica*
White = Not in *H. sapiens*

115 Inositol phosphate metabolism

115.1 Human Pathway: HSA00562

115.2 Number of Hits: 26

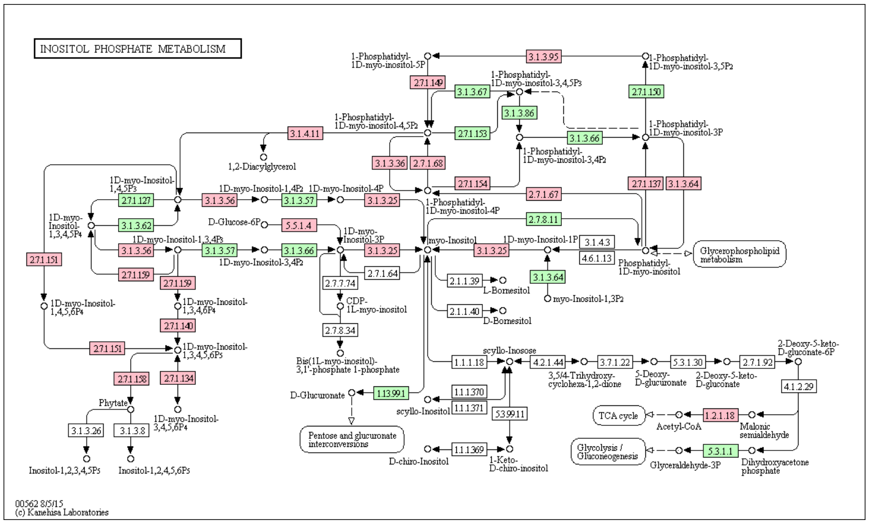

115.3 Legend:

RBH-Blast at 60% Identity + 50% Coverage
Green = Hit in *H. sapiens*
Red = Hit in *H. sapiens* and *T. californica*
White = Not in *H. sapiens*

116 Adipocytokine signaling pathway

116.1 Human Pathway: HSA04920

116.2 Number of Hits: 25

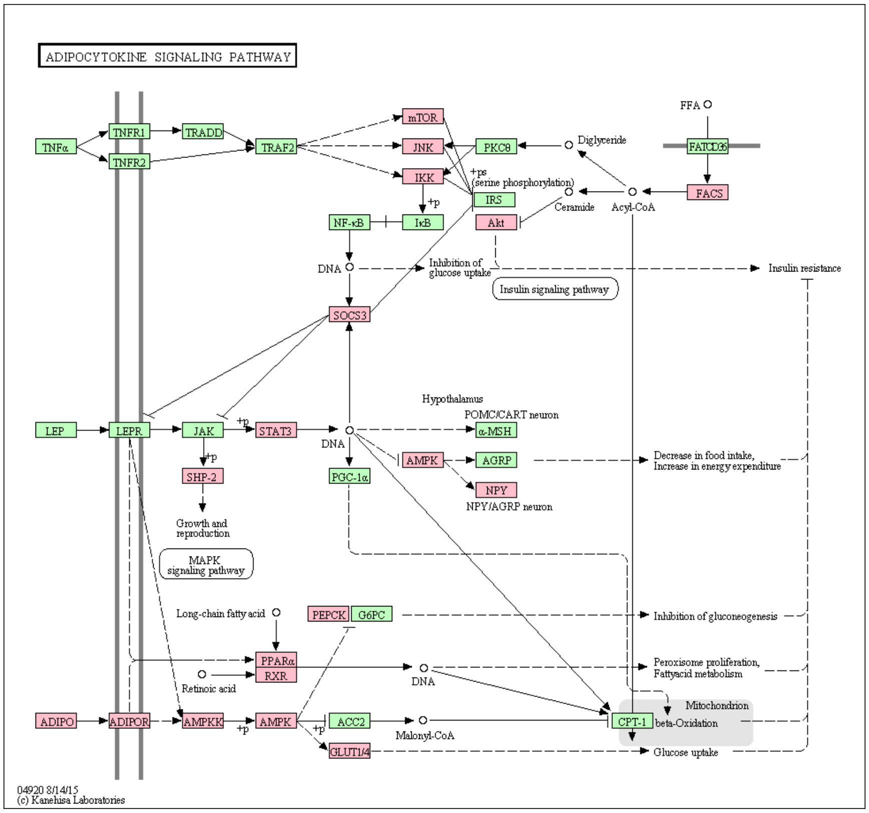

116.3 Legend:

RBH-Blast at 60% Identity + 50% Coverage
Green = Hit in *H. sapiens*
Red = Hit in *H. sapiens* and *T. californica*
White = Not in *H. sapiens*

117 TGF-beta signaling pathway

117.1 Human Pathway: HSA04350

117.2 Number of Hits: 25

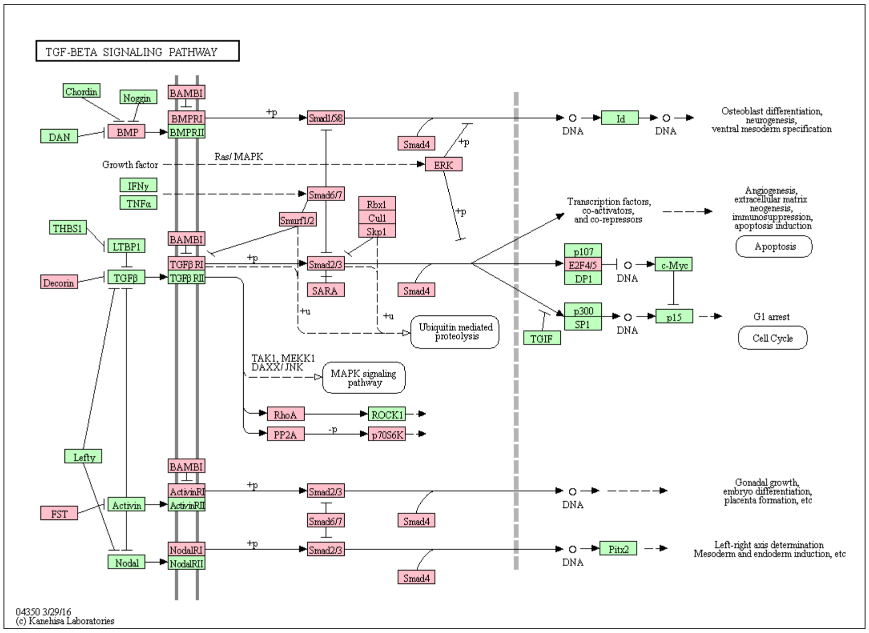

117.3 Legend:

RBH-Blast at 60% Identity + 50% Coverage
Green = Hit in *H. sapiens*
Red = Hit in *H. sapiens* and *T. californica*
White = Not in *H. sapiens*

118 TNF signaling pathway

118.1 Human Pathway: HSA04668

118.2 Number of Hits: 25

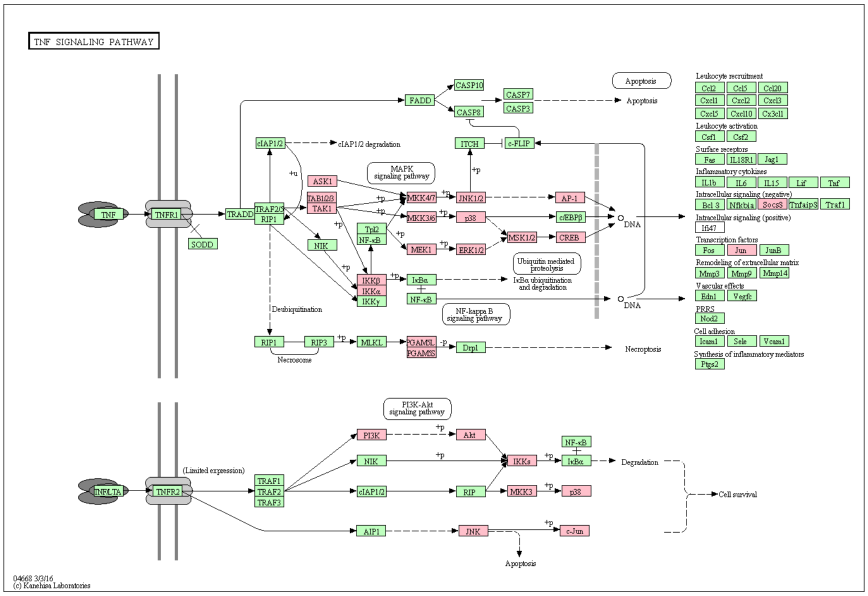

118.3 Legend:

RBH-Blast at 60% Identity + 50% Coverage
Green = Hit in *H. sapiens*
Red = Hit in *H. sapiens* and *T. californica*
White = Not in *H. sapiens*

119 Hypertrophic cardiomyopathy (HCM)

119.1 Human Pathway: HSA05410

119.2 Number of Hits: 25

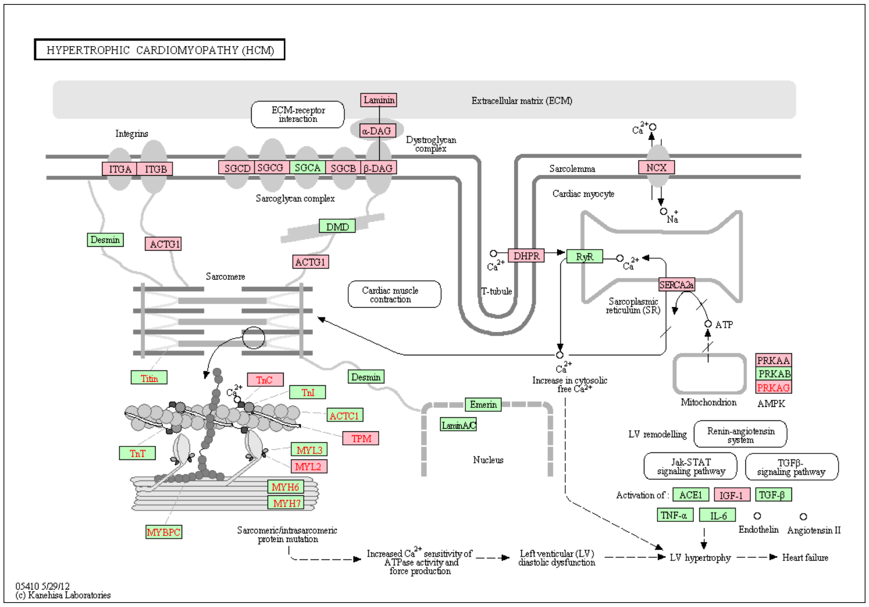

119.3 Legend:

RBH-Blast at 60% Identity + 50% Coverage
Green = Hit in *H. sapiens*
Red = Hit in *H. sapiens* and *T. californica*
White = Not in *H. sapiens*

120 Measles

120.1 Human Pathway: HSA05162

120.2 Number of Hits: 25

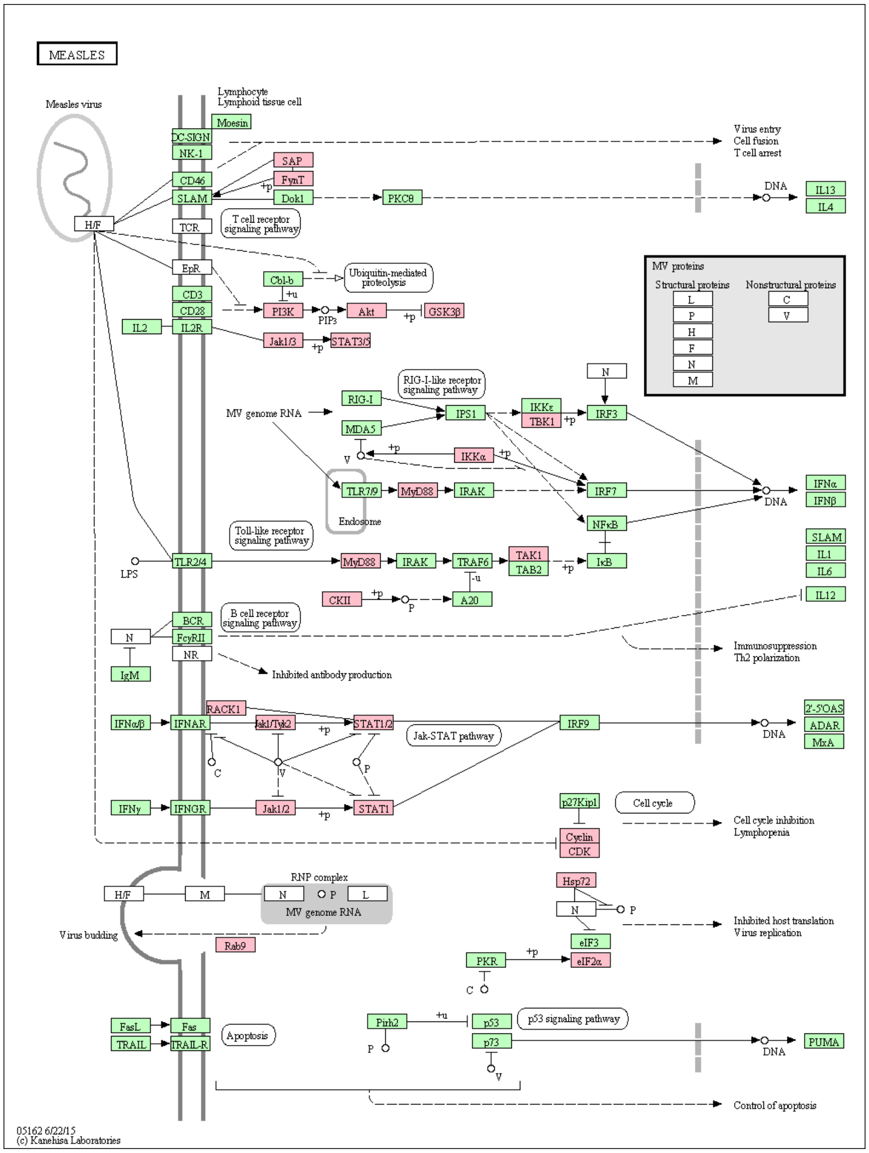

120.3 Legend:

RBH-Blast at 60% Identity + 50% Coverage
Green = Hit in *H. sapiens*
Red = Hit in *H. sapiens* and *T. californica*
White = Not in *H. sapiens*

---

121 Fatty acid metabolism

121.1 Human Pathway: HSA01212

121.2 Number of Hits: 25

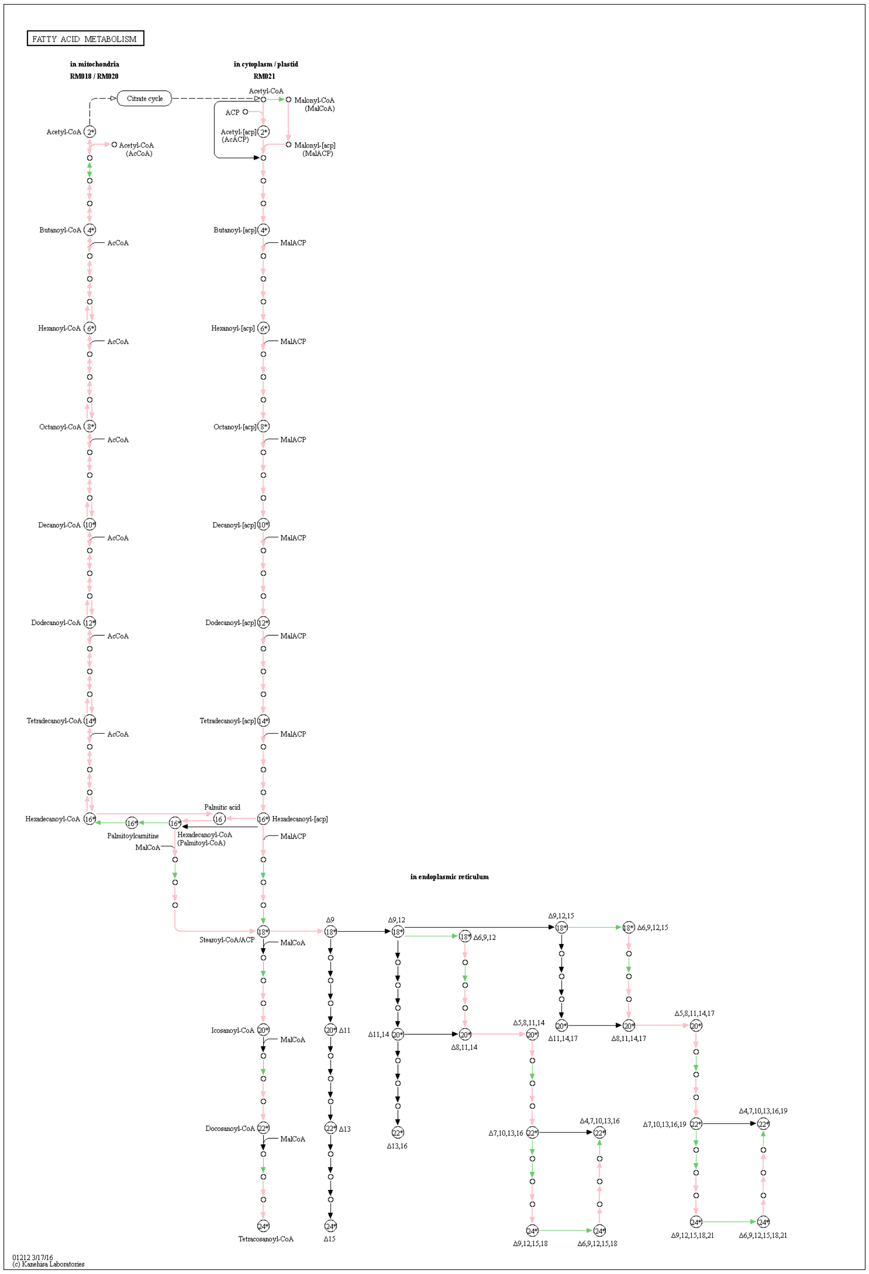

121.3 Legend:

RBH-Blast at 60% Identity + 50% Coverage
Green = Hit in *H. sapiens*
Red = Hit in *H. sapiens* and *T. californica*
White = Not in *H. sapiens*

---

122 Colorectal cancer

122.1 Human Pathway: HSA05210

122.2 Number of Hits: 24

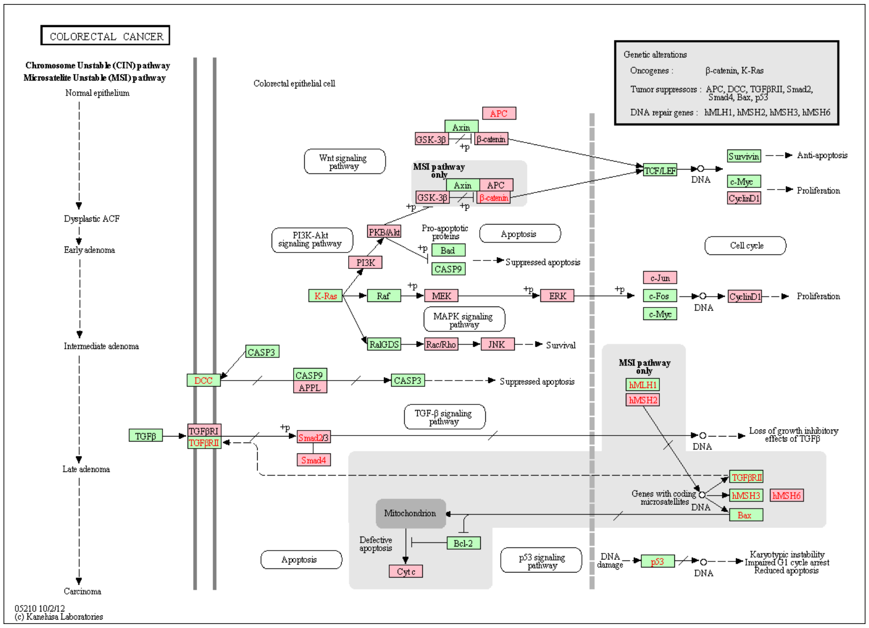

122.3 Legend:

RBH-Blast at 60% Identity + 50% Coverage
Green = Hit in *H. sapiens*
Red = Hit in *H. sapiens* and *T. californica*
White = Not in *H. sapiens*

---

123 Toll-like receptor signaling pathway

123.1 Human Pathway: HSA04620

123.2 Number of Hits: 24

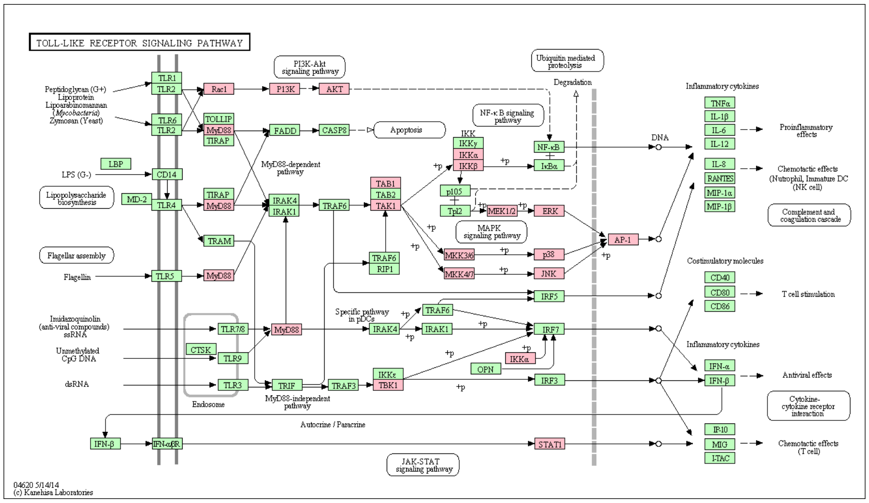

123.3 Legend:

RBH-Blast at 60% Identity + 50% Coverage
Green = Hit in *H. sapiens*
Red = Hit in *H. sapiens* and *T. californica*
White = Not in *H. sapiens*

124 Pancreatic secretion

124.1 Human Pathway: HSA04972

124.2 Number of Hits: 24

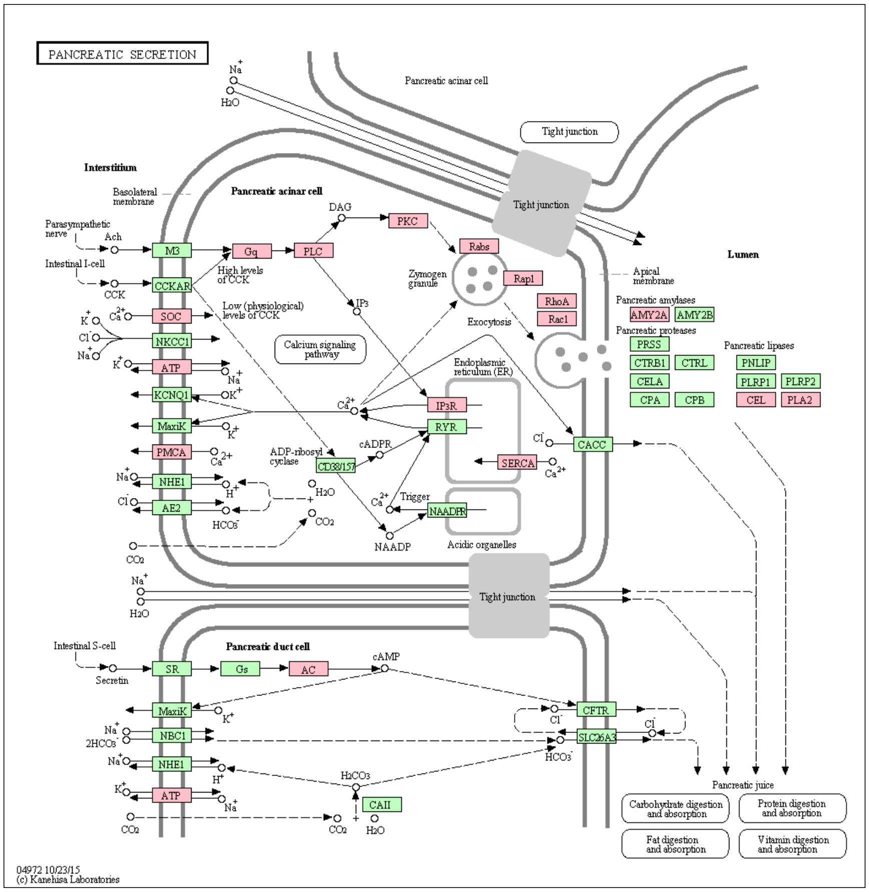

124.3 Legend:

RBH-Blast at 60% Identity + 50% Coverage
Green = Hit in *H. sapiens*
Red = Hit in *H. sapiens* and *T. californica*
White = Not in *H. sapiens*

125 VEGF signaling pathway

125.1 Human Pathway: HSA04370

125.2 Number of Hits: 24

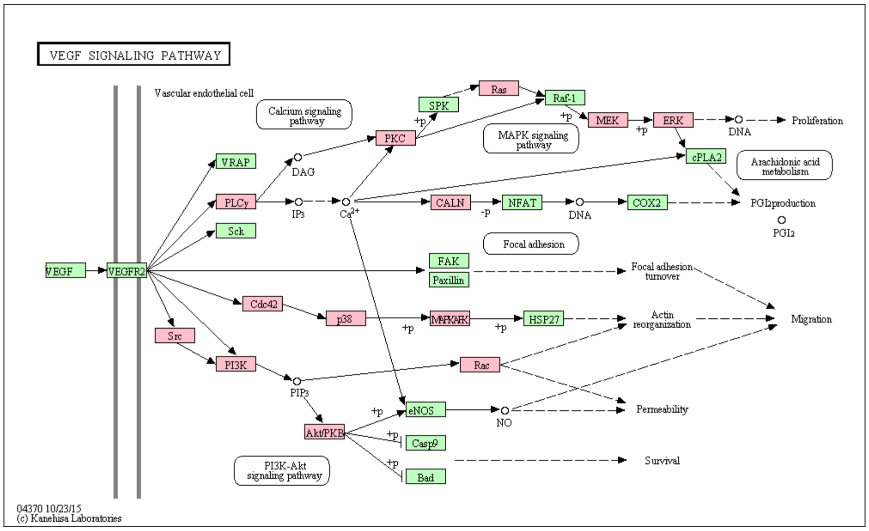

125.3 Legend:

RBH-Blast at 60% Identity + 50% Coverage
Green = Hit in *H. sapiens*
Red = Hit in *H. sapiens* and *T. californica*
White = Not in *H. sapiens*

126 Longevity regulating pathway - multiple species

126.1 Human Pathway: HSA04213

126.2 Number of Hits: 24

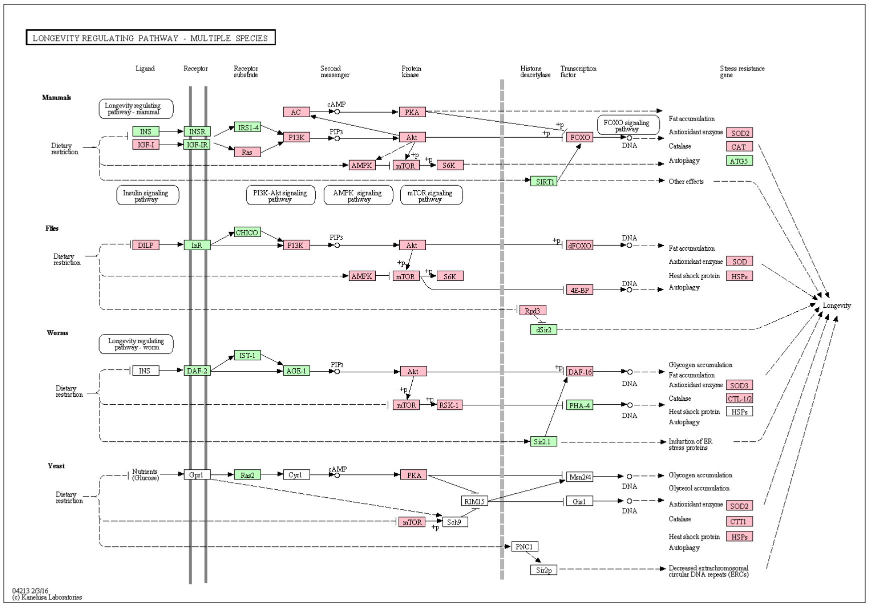

126.3 Legend:

RBH-Blast at 60% Identity + 50% Coverage
Green = Hit in *H. sapiens*
Red = Hit in *H. sapiens* and *T. californica*
White = Not in *H. sapiens*

127 Cardiac muscle contraction

127.1 Human Pathway: HSA04260

127.2 Number of Hits: 24

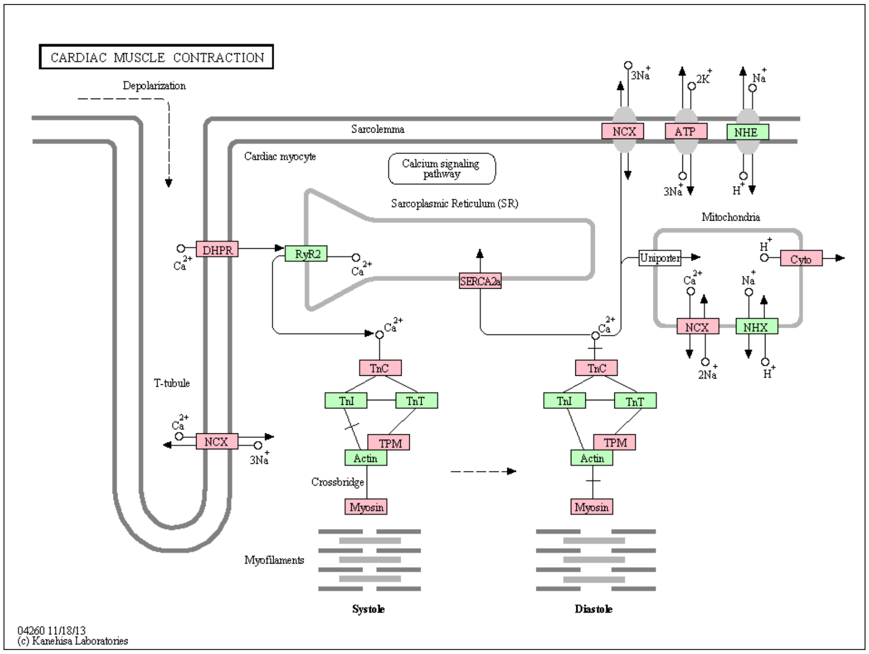

127.3 Legend:

RBH-Blast at 60% Identity + 50% Coverage
Green = Hit in *H. sapiens*
Red = Hit in *H. sapiens* and *T. californica*
White = Not in *H. sapiens*

128 EGFR tyrosine kinase inhibitor resistance

128.1 Human Pathway: HSA01521

128.2 Number of Hits: 24

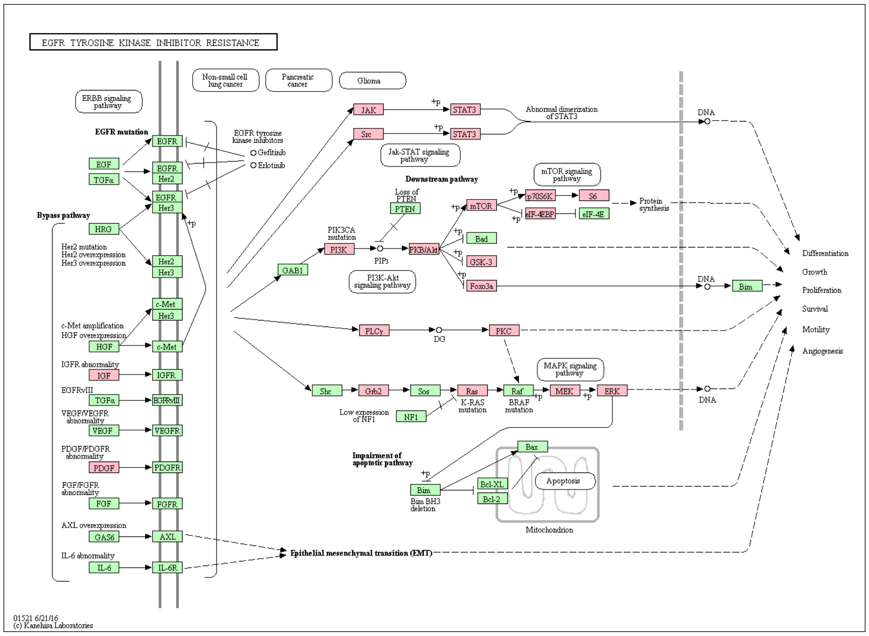

128.3 Legend:

RBH-Blast at 60% Identity + 50% Coverage
Green = Hit in *H. sapiens*
Red = Hit in *H. sapiens* and *T. californica*
White = Not in *H. sapiens*

129 Nucleotide excision repair

129.1 Human Pathway: HSA03420

129.2 Number of Hits: 23

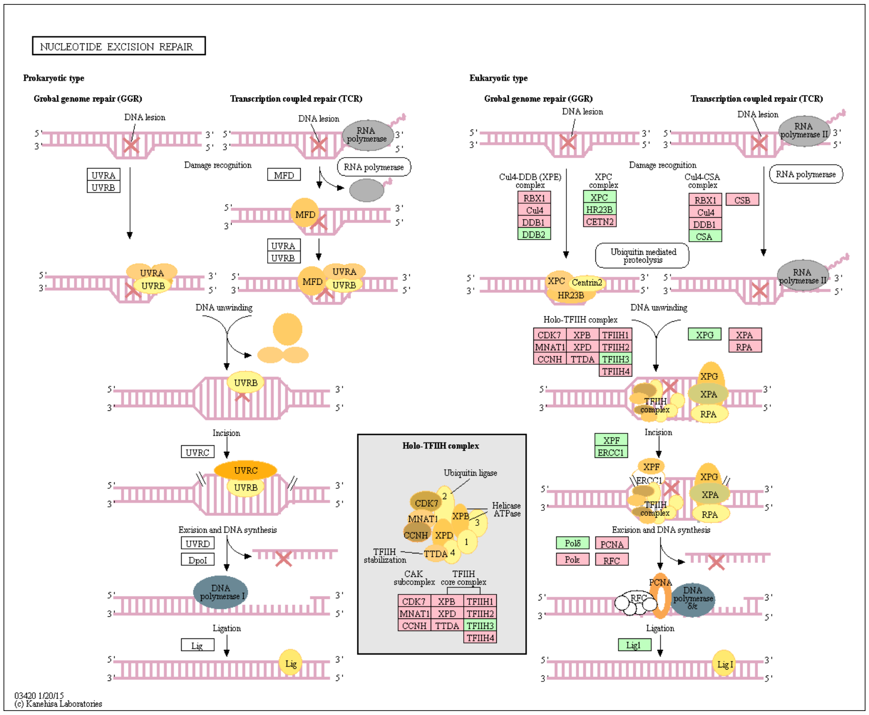

129.3 Legend:

RBH-Blast at 60% Identity + 50% Coverage
Green = Hit in *H. sapiens*
Red = Hit in *H. sapiens* and *T. californica*
White = Not in *H. sapiens*

130 Natural killer cell mediated cytotoxicity

130.1 Human Pathway: HSA04650

130.2 Number of Hits: 23

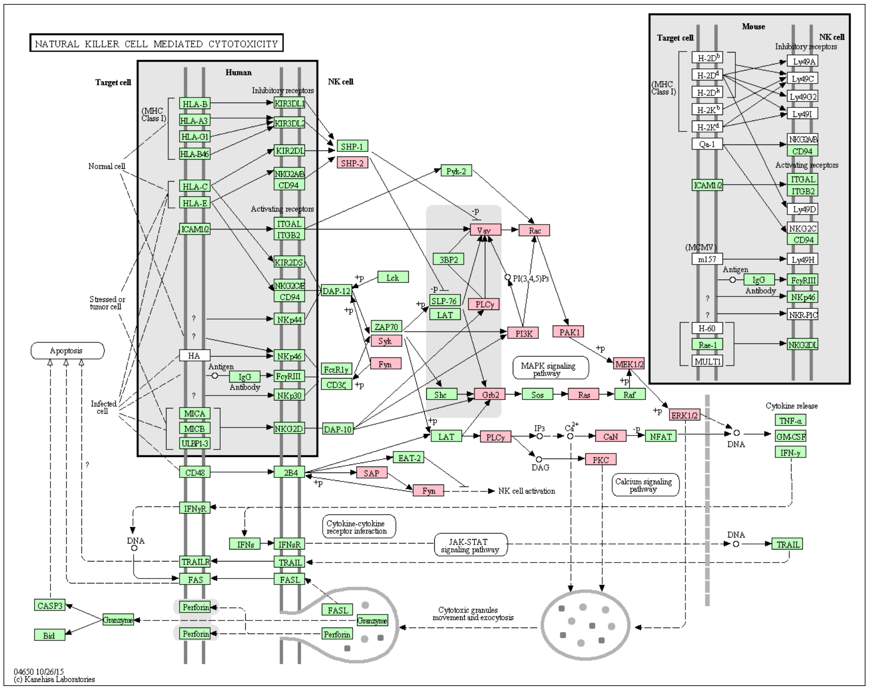

130.3 Legend:

RBH-Blast at 60% Identity + 50% Coverage
Green = Hit in *H. sapiens*
Red = Hit in *H. sapiens* and *T. californica*
White = Not in *H. sapiens*

131 Arrhythmogenic right ventricular cardiomyopathy (ARVC)

131.1 Human Pathway: HSA05412

131.2 Number of Hits: 23

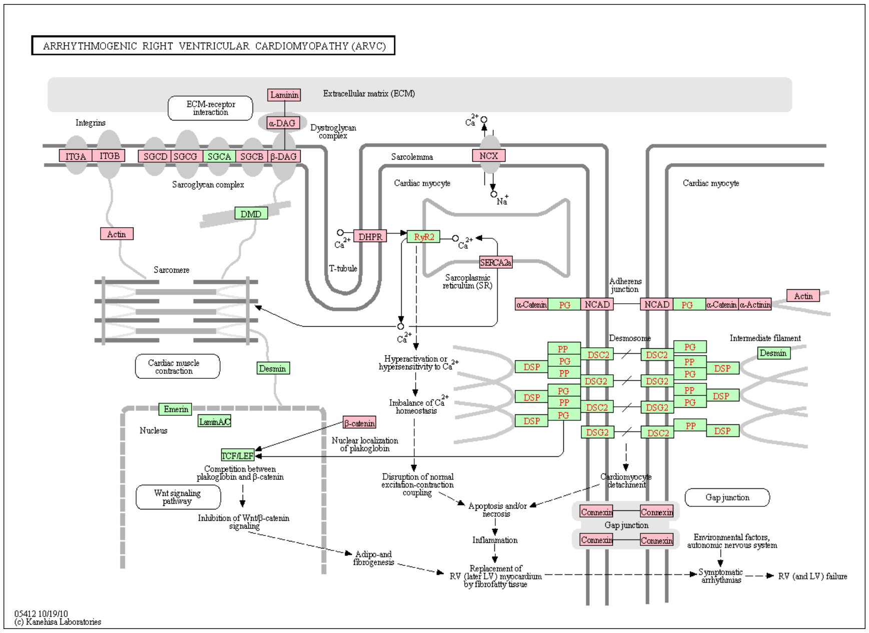

131.3 Legend:

RBH-Blast at 60% Identity + 50% Coverage
Green = Hit in *H. sapiens*
Red = Hit in *H. sapiens* and *T. californica*
White = Not in *H. sapiens*

132 Chronic myeloid leukemia

132.1 Human Pathway: HSA05220

132.2 Number of Hits: 23

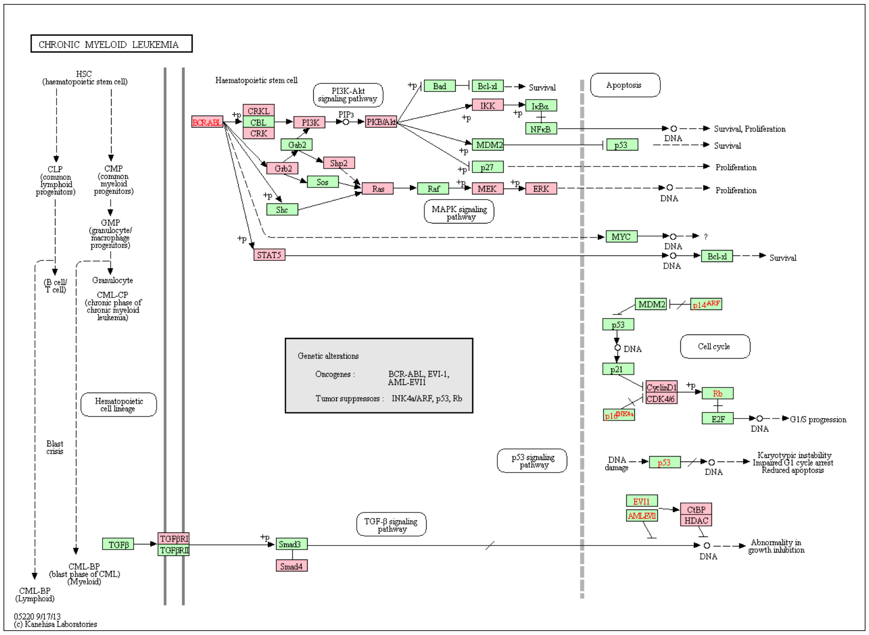

132.3 Legend:

RBH-Blast at 60% Identity + 50% Coverage
Green = Hit in *H. sapiens*
Red = Hit in *H. sapiens* and *T. californica*
White = Not in *H. sapiens*

133 Prostate cancer

133.1 Human Pathway: HSA05215

133.2 Number of Hits: 23

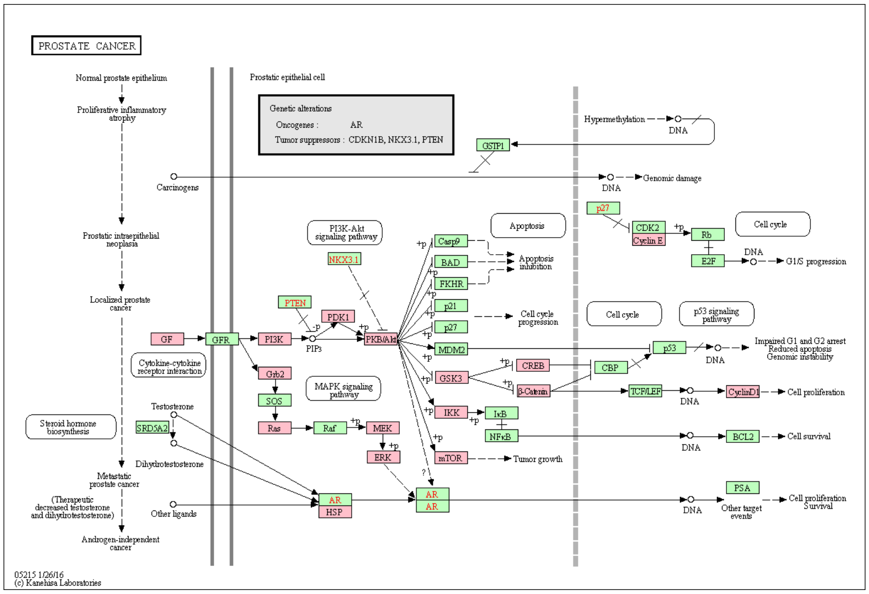

133.3 Legend:

RBH-Blast at 60% Identity + 50% Coverage
Green = Hit in *H. sapiens*
Red = Hit in *H. sapiens* and *T. californica*
White = Not in *H. sapiens*

134 Amino sugar and nucleotide sugar metabolism

134.1 Human Pathway: HSA00520

134.2 Number of Hits: 23

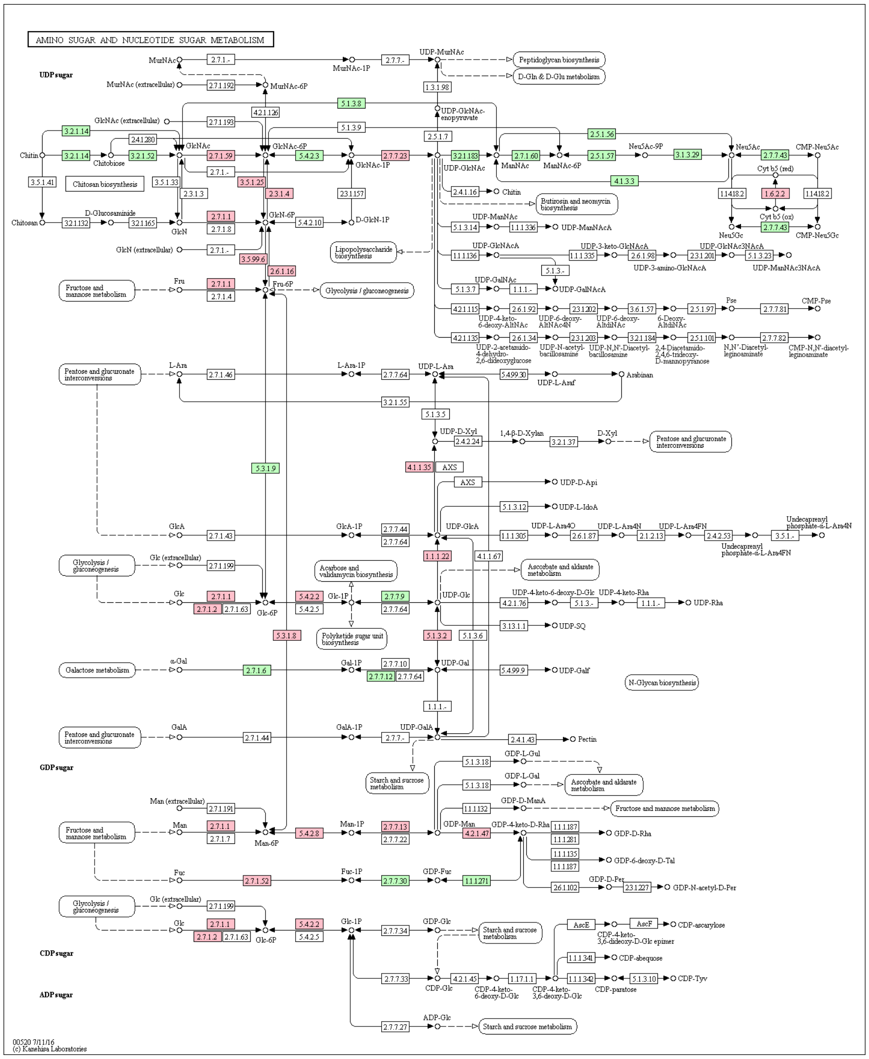

134.3 Legend:

RBH-Blast at 60% Identity + 50% Coverage
Green = Hit in *H. sapiens*
Red = Hit in *H. sapiens* and *T. californica*
White = Not in *H. sapiens*

---

135 Amyotrophic lateral sclerosis (ALS)

135.1 Human Pathway: HSA05014

135.2 Number of Hits: 22

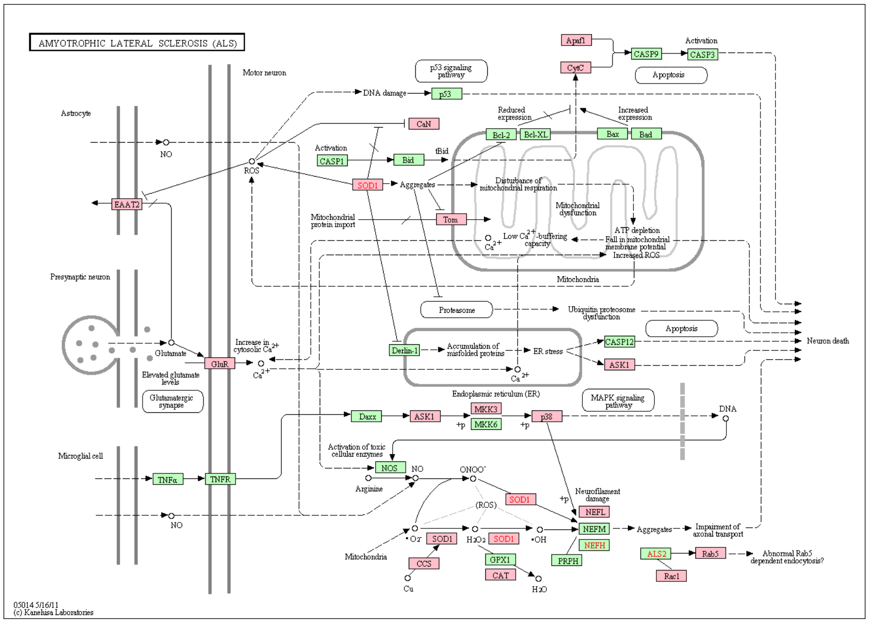

135.3 Legend:

RBH-Blast at 60% Identity + 50% Coverage
Green = Hit in *H. sapiens*
Red = Hit in *H. sapiens* and *T. californica*
White = Not in *H. sapiens*

---

136 Vibrio cholerae infection

136.1 Human Pathway: HSA05110

136.2 Number of Hits: 22

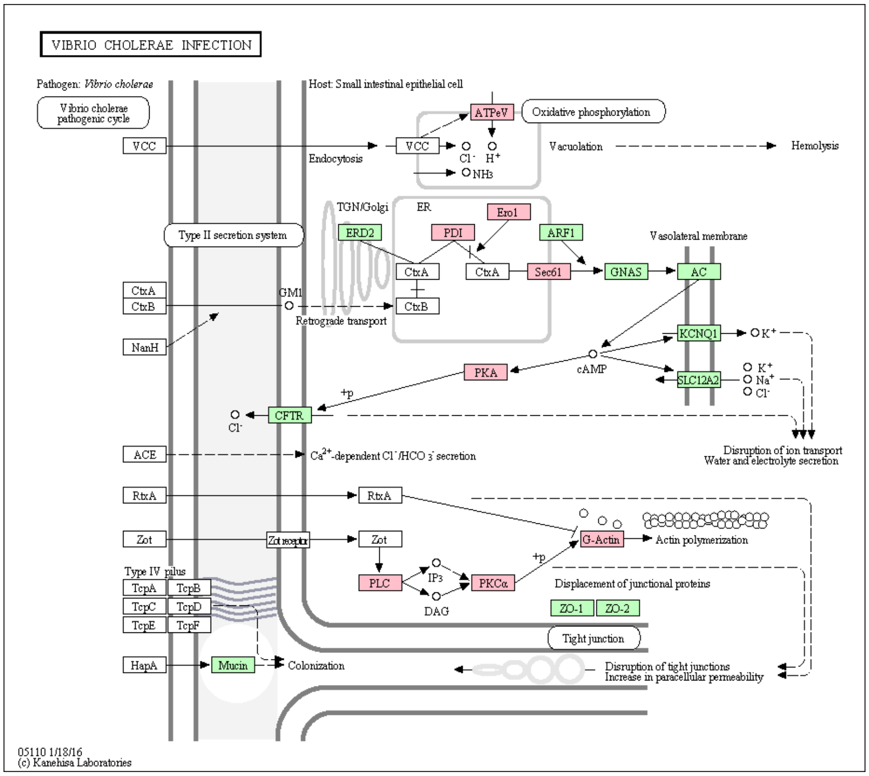

136.3 Legend:

RBH-Blast at 60% Identity + 50% Coverage
Green = Hit in *H. sapiens*
Red = Hit in *H. sapiens* and *T. californica*
White = Not in *H. sapiens*

137 SNARE interactions in vesicular transport

137.1 Human Pathway: HSA04130

137.2 Number of Hits: 22

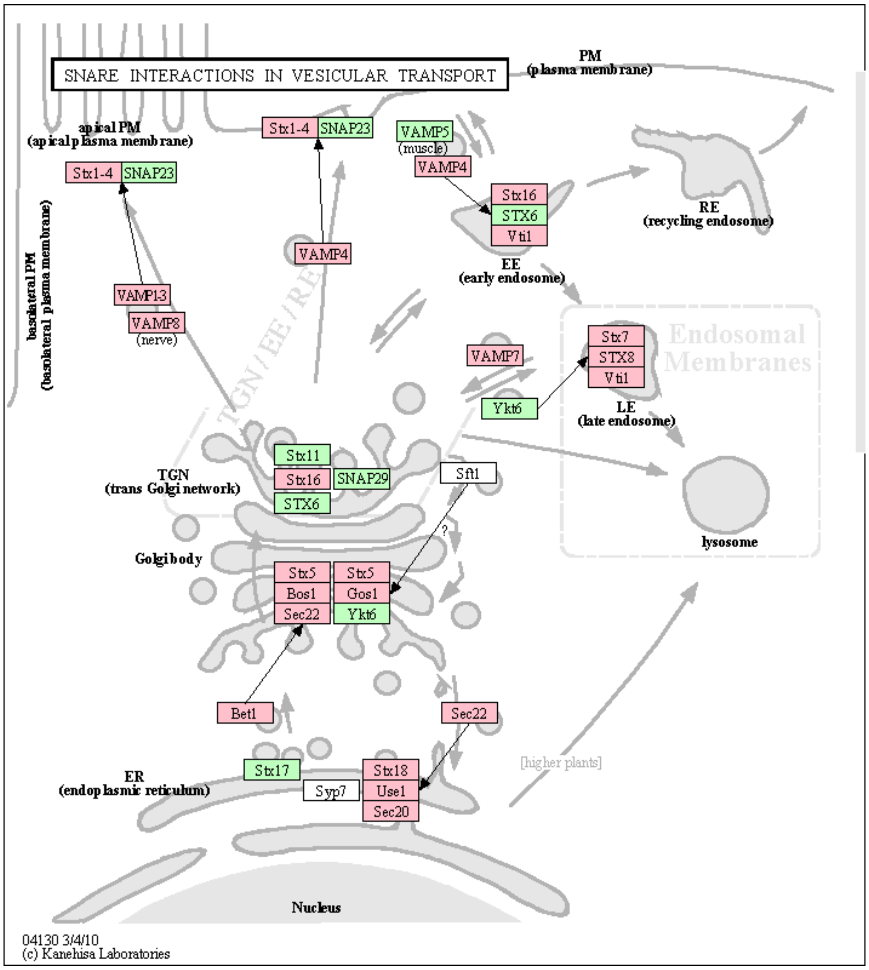

137.3 Legend:

RBH-Blast at 60% Identity + 50% Coverage
Green = Hit in *H. sapiens*
Red = Hit in *H. sapiens* and *T. californica*
White = Not in *H. sapiens*

138 Pathogenic Escherichia coli infection

138.1 Human Pathway: HSA05130

138.2 Number of Hits: 22

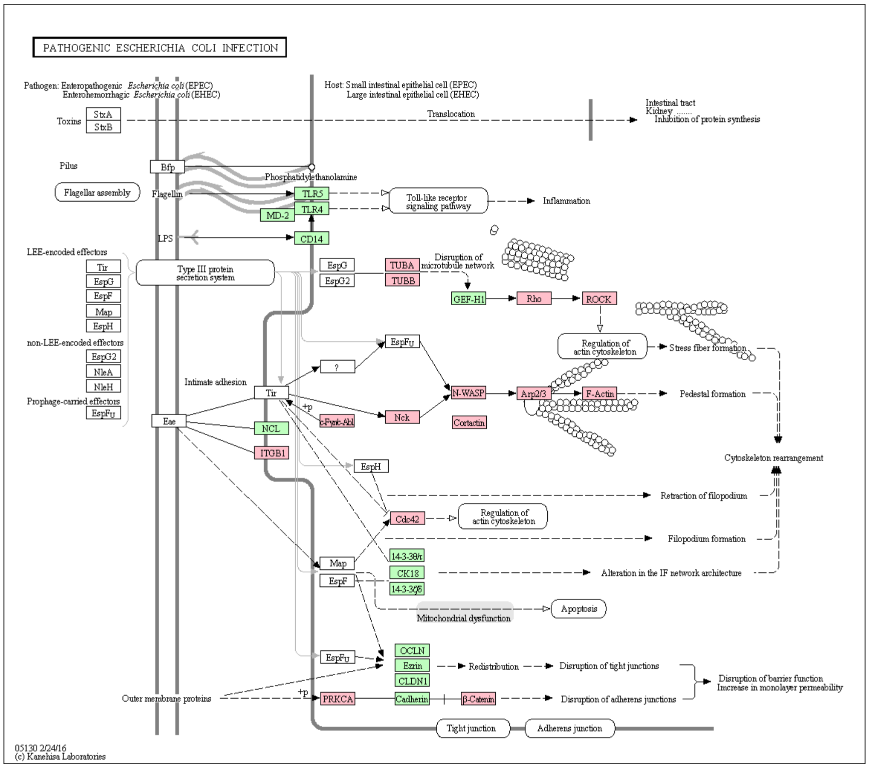

138.3 Legend:

RBH-Blast at 60% Identity + 50% Coverage
Green = Hit in *H. sapiens*
Red = Hit in *H. sapiens* and *T. californica*
White = Not in *H. sapiens*

139 Endocrine and other factor-regulated calcium reabsorption

139.1 Human Pathway: HSA04961

139.2 Number of Hits: 21

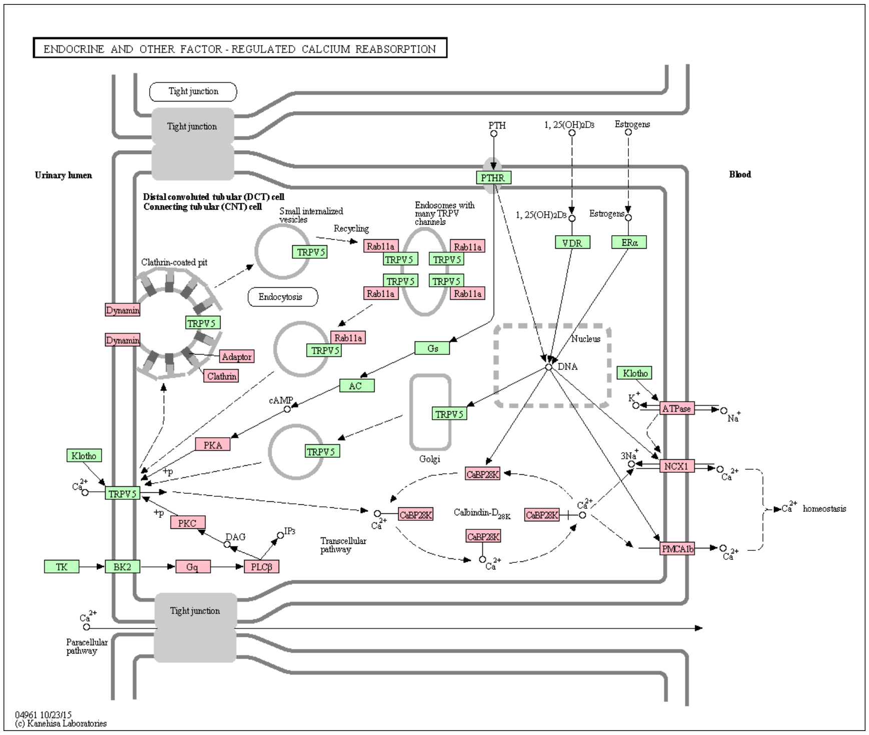

139.3 Legend:

RBH-Blast at 60% Identity + 50% Coverage
Green = Hit in *H. sapiens*
Red = Hit in *H. sapiens* and *T. californica*
White = Not in *H. sapiens*

140 Insulin secretion

140.1 Human Pathway: HSA04911

140.2 Number of Hits: 21

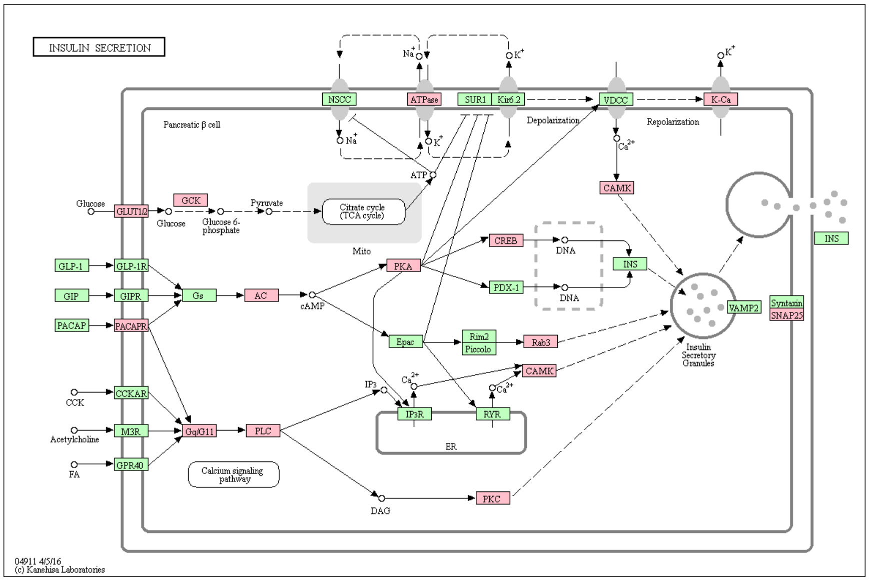

140.3 Legend:

RBH-Blast at 60% Identity + 50% Coverage
Green = Hit in *H. sapiens*
Red = Hit in *H. sapiens* and *T. californica*
White = Not in *H. sapiens*

141 Amphetamine addiction

141.1 Human Pathway: HSA05031

141.2 Number of Hits: 21

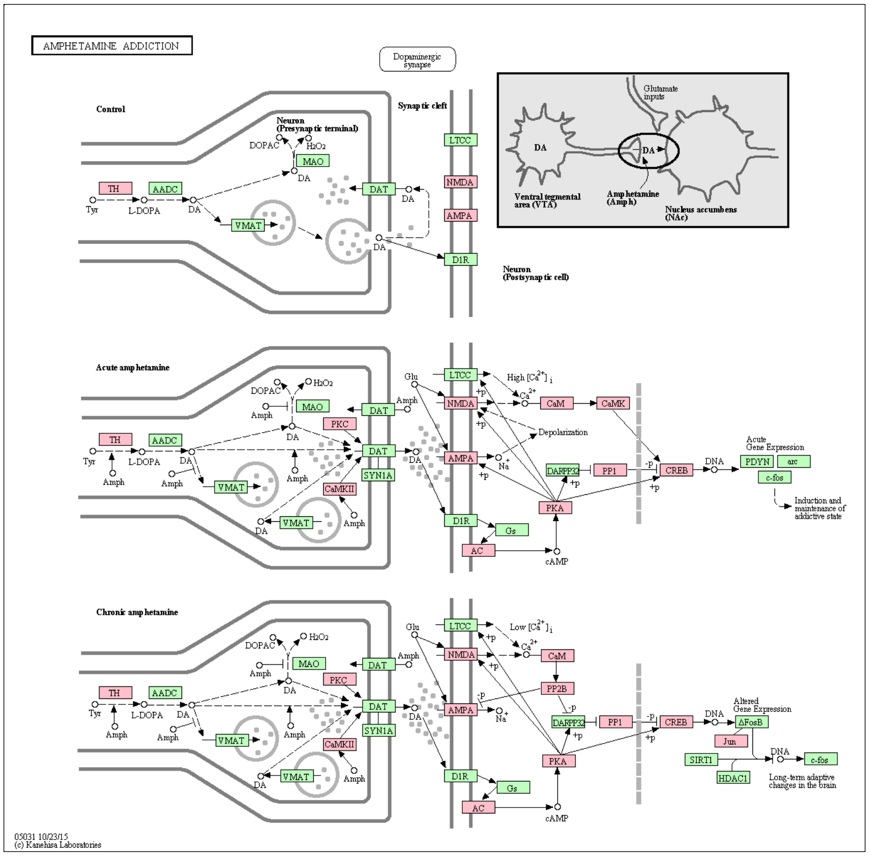

141.3 Legend:

RBH-Blast at 60% Identity + 50% Coverage
Green = Hit in *H. sapiens*
Red = Hit in *H. sapiens* and *T. californica*
White = Not in *H. sapiens*

142 Small cell lung cancer

142.1 Human Pathway: HSA05222

142.2 Number of Hits: 21

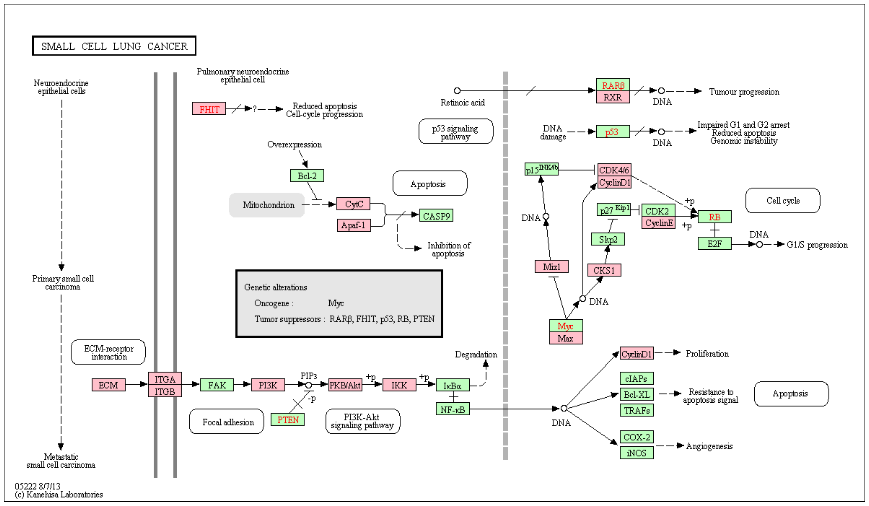

142.3 Legend:

RBH-Blast at 60% Identity + 50% Coverage
Green = Hit in *H. sapiens*
Red = Hit in *H. sapiens* and *T. californica*
White = Not in *H. sapiens*

143 Aldosterone synthesis and secretion

143.1 Human Pathway: HSA04925

143.2 Number of Hits: 21

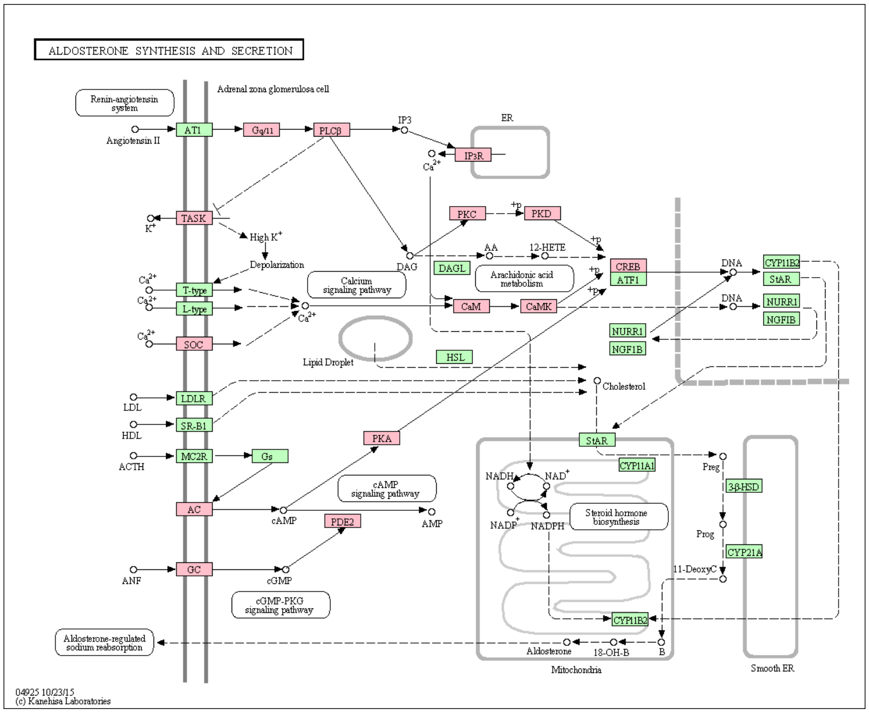

143.3 Legend:

RBH-Blast at 60% Identity + 50% Coverage
Green = Hit in *H. sapiens*
Red = Hit in *H. sapiens* and *T. californica*
White = Not in *H. sapiens*

144 Pyruvate metabolism

144.1 Human Pathway: HSA00620

144.2 Number of Hits: 21

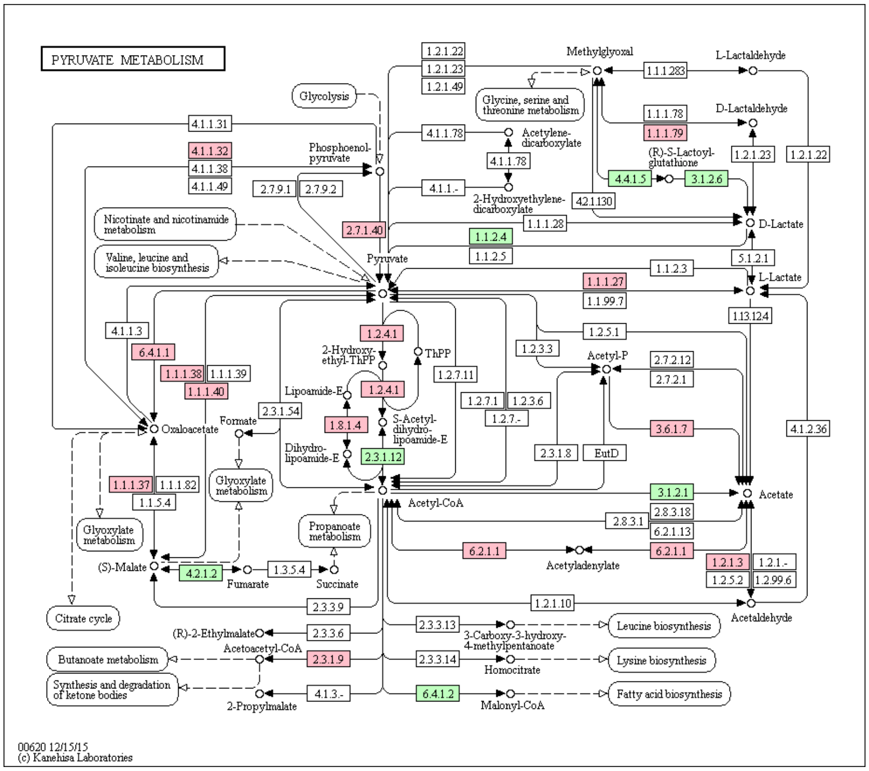

144.3 Legend:

RBH-Blast at 60% Identity + 50% Coverage
Green = Hit in *H. sapiens*
Red = Hit in *H. sapiens* and *T. californica*
White = Not in *H. sapiens*

145 Propanoate metabolism

145.1 Human Pathway: HSA00640

145.2 Number of Hits: 21

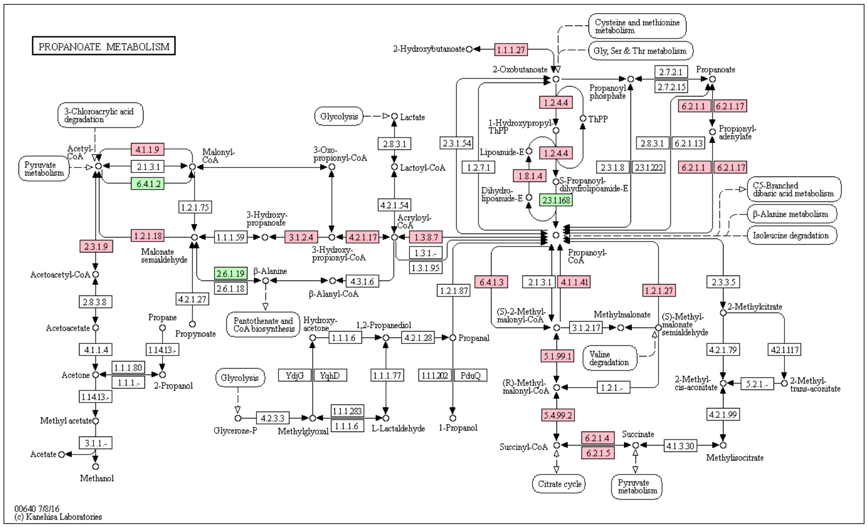

145.3 Legend:

RBH-Blast at 60% Identity + 50% Coverage
Green = Hit in *H. sapiens*
Red = Hit in *H. sapiens* and *T. californica*
White = Not in *H. sapiens*

146 Acute myeloid leukemia

146.1 Human Pathway: HSA05221

146.2 Number of Hits: 20

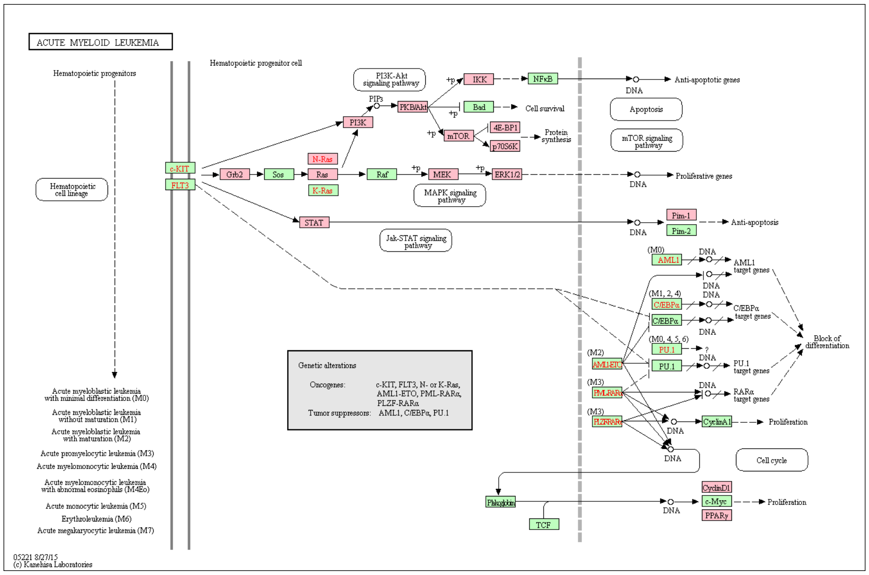

146.3 Legend:

RBH-Blast at 60% Identity + 50% Coverage
Green = Hit in *H. sapiens*
Red = Hit in *H. sapiens* and *T. californica*
White = Not in *H. sapiens*

147 Cysteine and methionine metabolism

147.1 Human Pathway: HSA00270

147.2 Number of Hits: 20

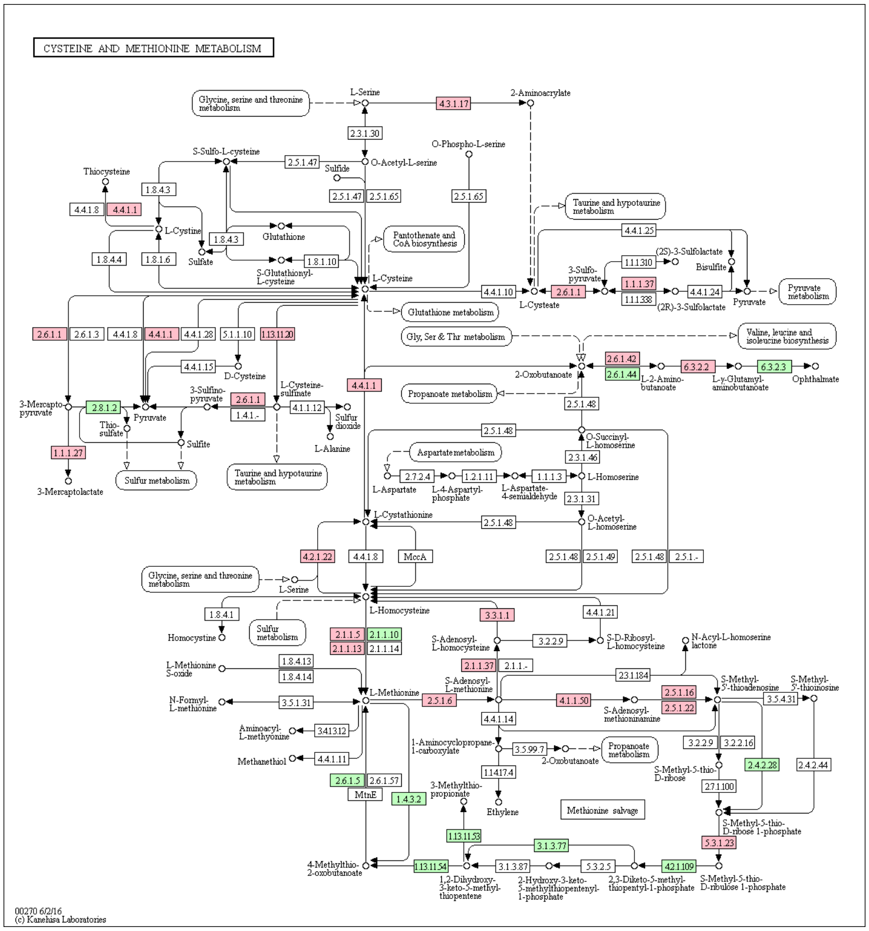

147.3 Legend:

RBH-Blast at 60% Identity + 50% Coverage
Green = Hit in *H. sapiens*
Red = Hit in *H. sapiens* and *T. californica*
White = Not in *H. sapiens*

148 Lysine degradation

148.1 Human Pathway: HSA00310

148.2 Number of Hits: 20

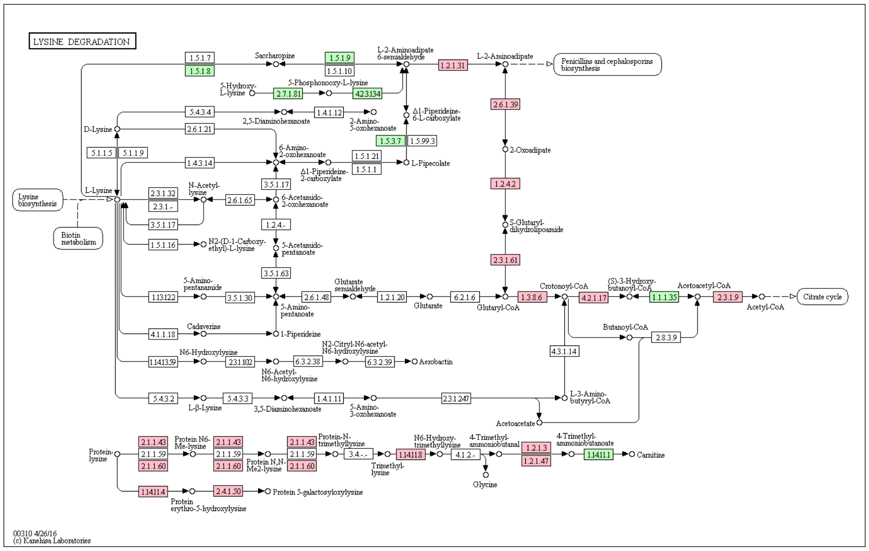

148.3 Legend:

RBH-Blast at 60% Identity + 50% Coverage
Green = Hit in *H. sapiens*
Red = Hit in *H. sapiens* and *T. californica*
White = Not in *H. sapiens*

149 Thyroid hormone synthesis

149.1 Human Pathway: HSA04918

149.2 Number of Hits: 20

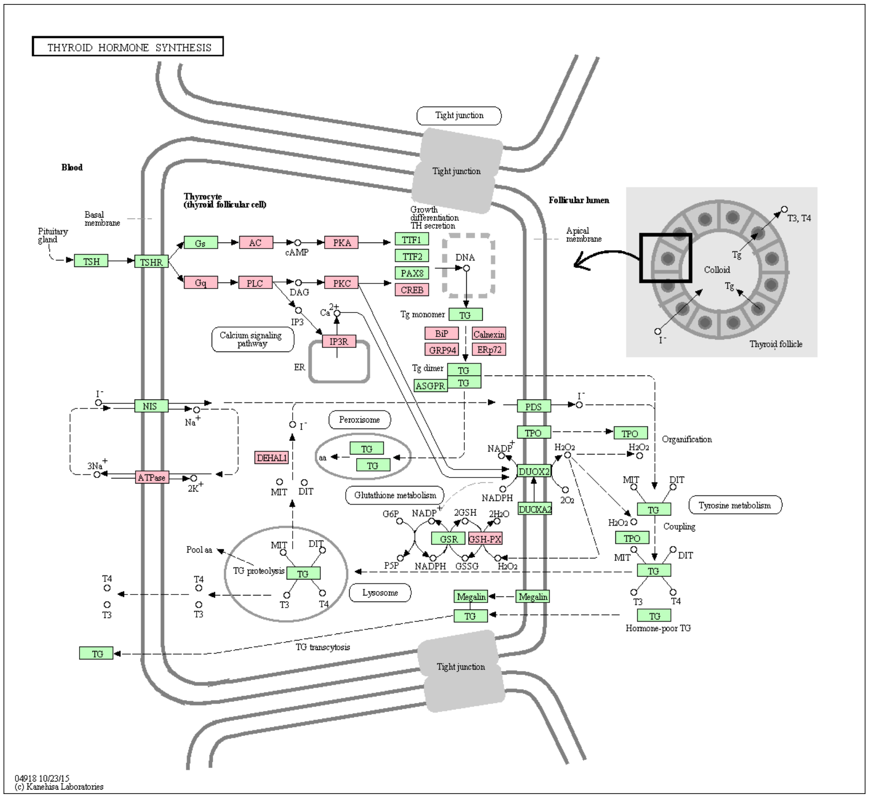

149.3 Legend:

RBH-Blast at 60% Identity + 50% Coverage
Green = Hit in *H. sapiens*
Red = Hit in *H. sapiens* and *T. californica*
White = Not in *H. sapiens*

150 N-Glycan biosynthesis

150.1 Human Pathway: HSA00510

150.2 Number of Hits: 20

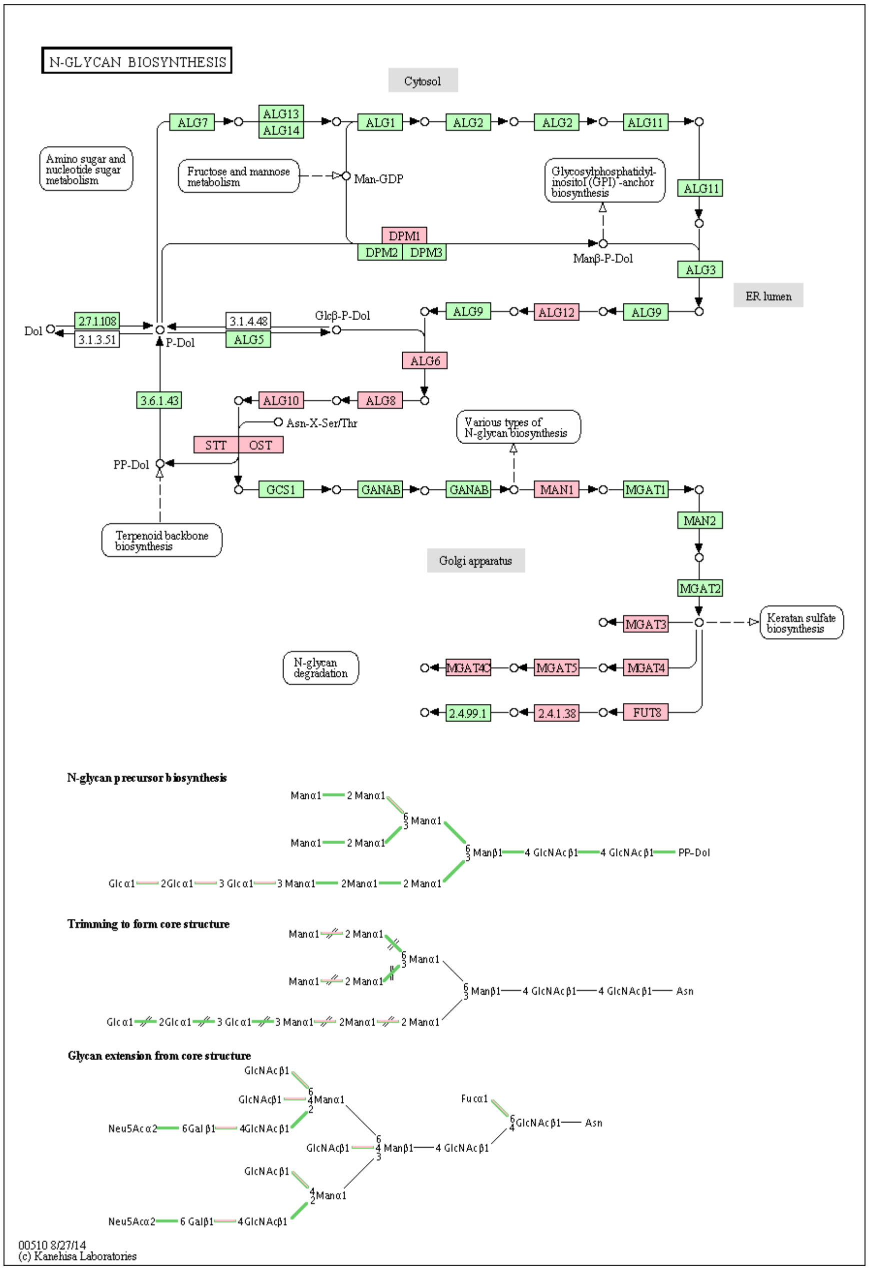

150.3 Legend:

RBH-Blast at 60% Identity + 50% Coverage
Green = Hit in *H. sapiens*
Red = Hit in *H. sapiens* and *T. californica*
White = Not in *H. sapiens*

---

151 Fatty acid degradation

151.1 Human Pathway: HSA00071

151.2 Number of Hits: 19

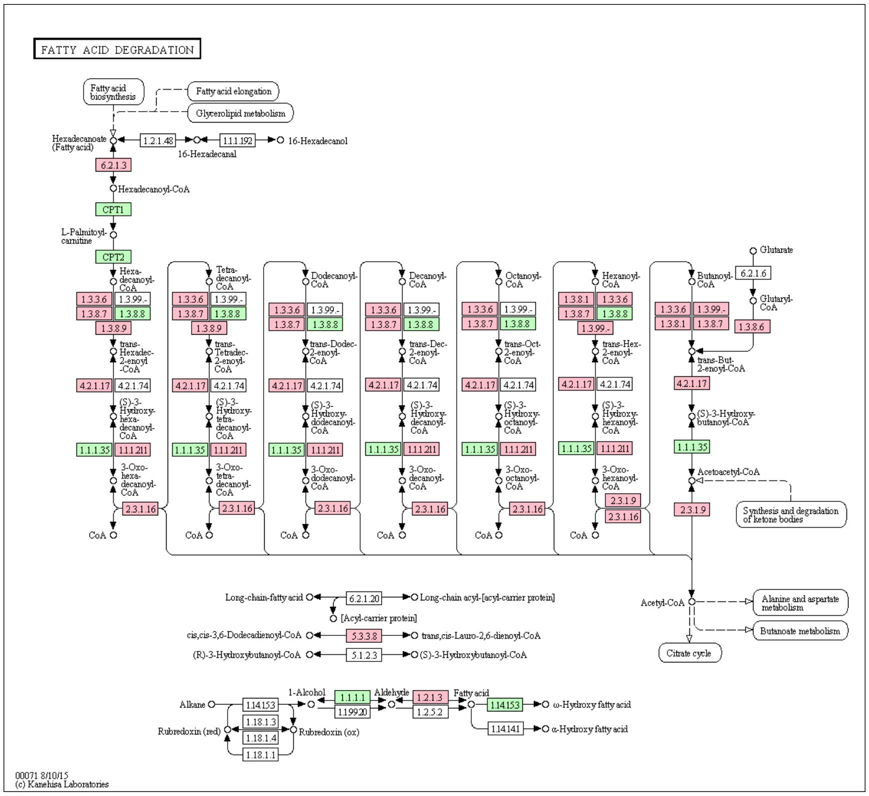

151.3 Legend:

RBH-Blast at 60% Identity + 50% Coverage
Green = Hit in *H. sapiens*
Red = Hit in *H. sapiens* and *T. californica*
White = Not in *H. sapiens*

---

152 Jak-STAT signaling pathway

152.1 Human Pathway: HSA04630

152.2 Number of Hits: 19

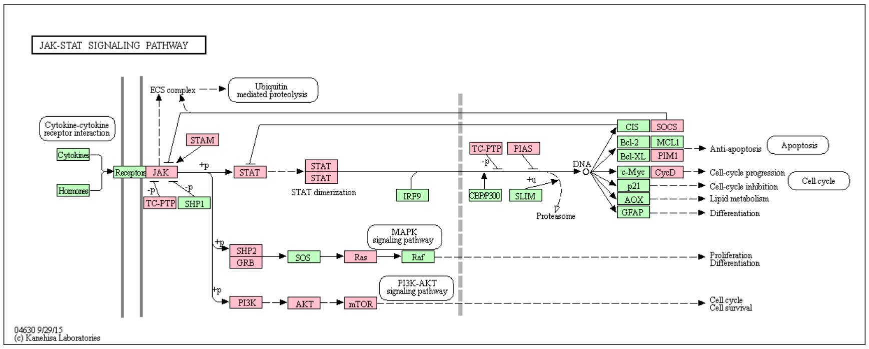

152.3 Legend:

RBH-Blast at 60% Identity + 50% Coverage
Green = Hit in *H. sapiens*
Red = Hit in *H. sapiens* and *T. californica*
White = Not in *H. sapiens*

---

153 Glycerolipid metabolism

153.1 Human Pathway: HSA00561

153.2 Number of Hits: 19

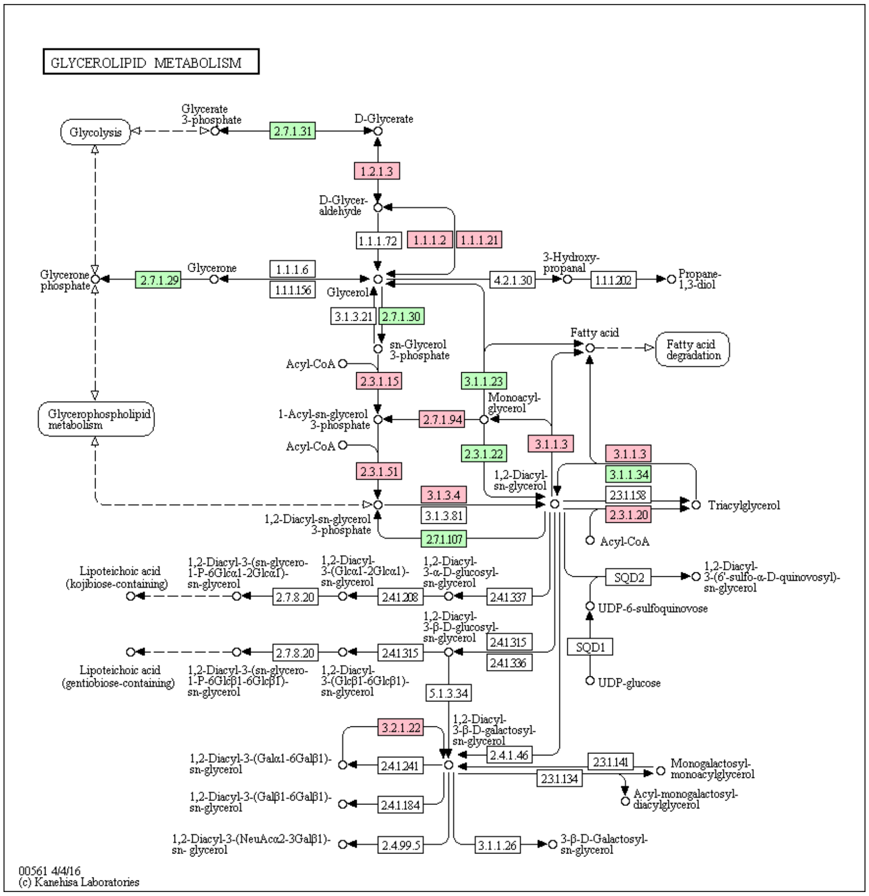

153.3 Legend:

RBH-Blast at 60% Identity + 50% Coverage
Green = Hit in *H. sapiens*
Red = Hit in *H. sapiens* and *T. californica*
White = Not in *H. sapiens*

154 Glioma

154.1 Human Pathway: HSA05214

154.2 Number of Hits: 19

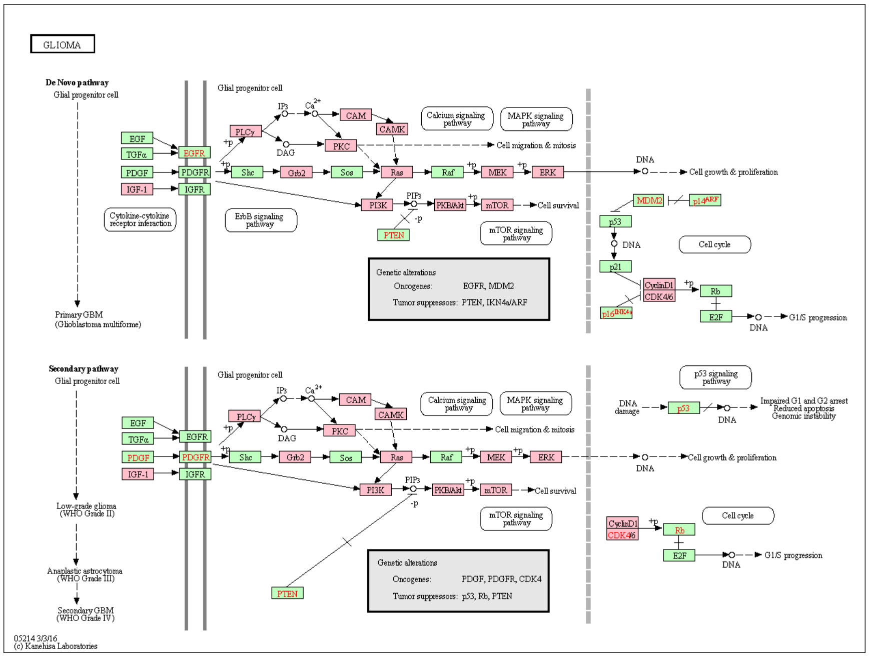

154.3 Legend:

RBH-Blast at 60% Identity + 50% Coverage
Green = Hit in *H. sapiens*
Red = Hit in *H. sapiens* and *T. californica*
White = Not in *H. sapiens*

155 Glyoxylate and dicarboxylate metabolism

155.1 Human Pathway: HSA00630

155.2 Number of Hits: 19

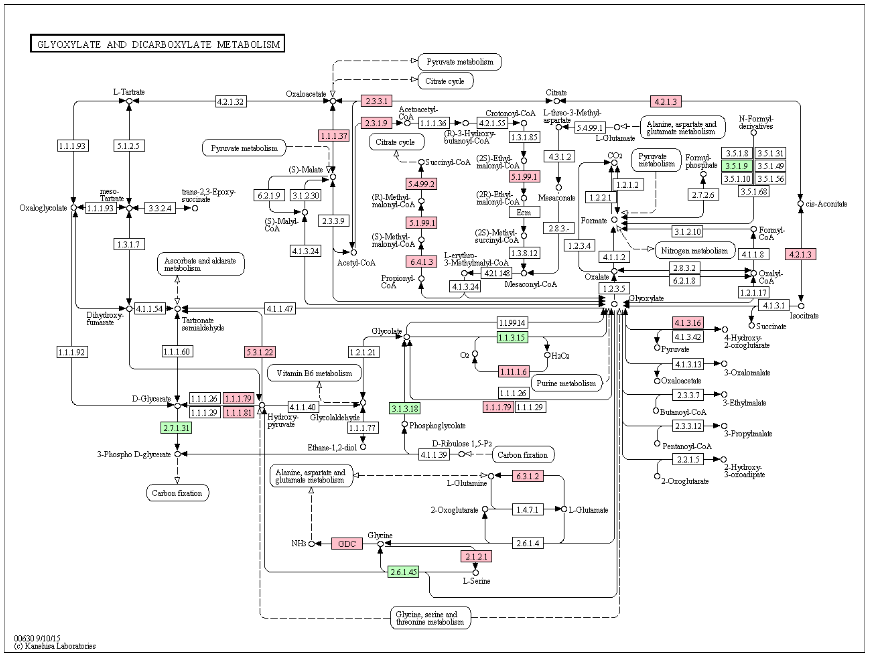

155.3 Legend:

RBH-Blast at 60% Identity + 50% Coverage
Green = Hit in *H. sapiens*
Red = Hit in *H. sapiens* and *T. californica*
White = Not in *H. sapiens*

156 Amoebiasis

156.1 Human Pathway: HSA05146

156.2 Number of Hits: 19

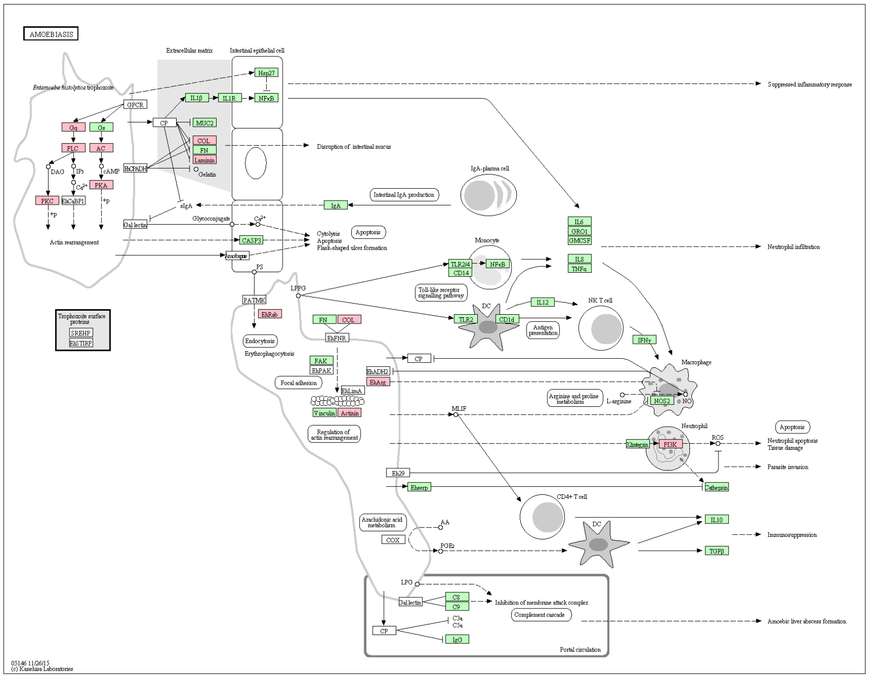

156.3 Legend:

RBH-Blast at 60% Identity + 50% Coverage
Green = Hit in *H. sapiens*
Red = Hit in *H. sapiens* and *T. californica*
White = Not in *H. sapiens*

157 Arginine and proline metabolism

157.1 Human Pathway: HSA00330

157.2 Number of Hits: 19

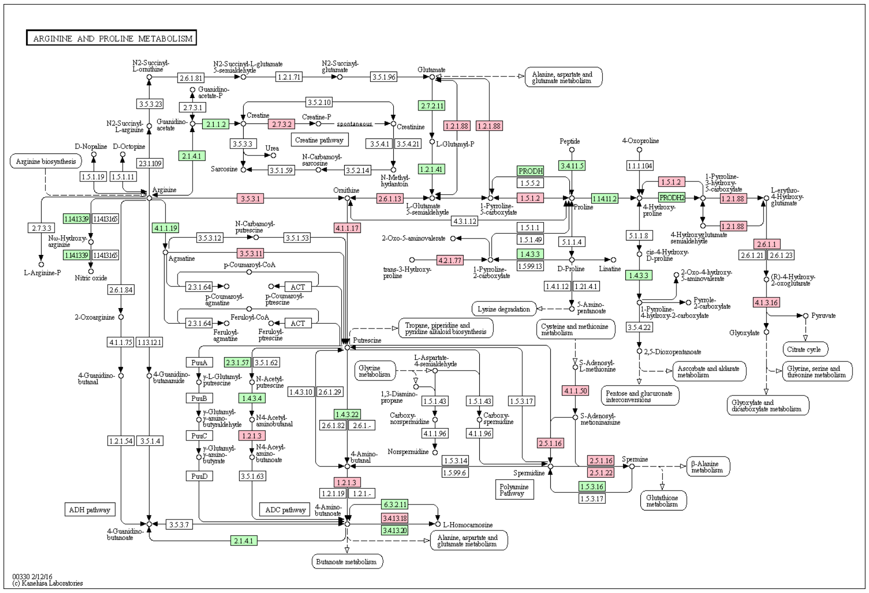

157.3 Legend:

RBH-Blast at 60% Identity + 50% Coverage
Green = Hit in *H. sapiens*
Red = Hit in *H. sapiens* and *T. californica*
White = Not in *H. sapiens*

158 Non-small cell lung cancer

158.1 Human Pathway: HSA05223

158.2 Number of Hits: 19

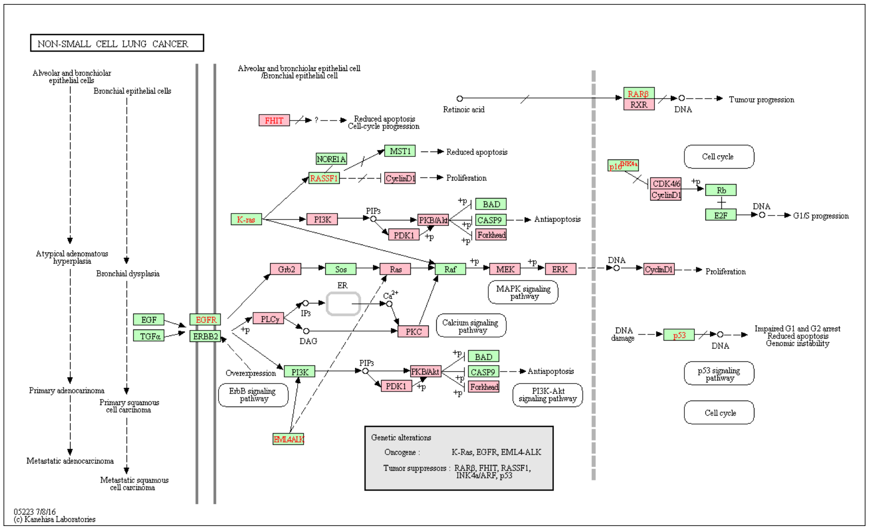

158.3 Legend:

RBH-Blast at 60% Identity + 50% Coverage
Green = Hit in *H. sapiens*
Red = Hit in *H. sapiens* and *T. californica*
White = Not in *H. sapiens*

159 Cell adhesion molecules (CAMs)

159.1 Human Pathway: HSA04514

159.2 Number of Hits: 19

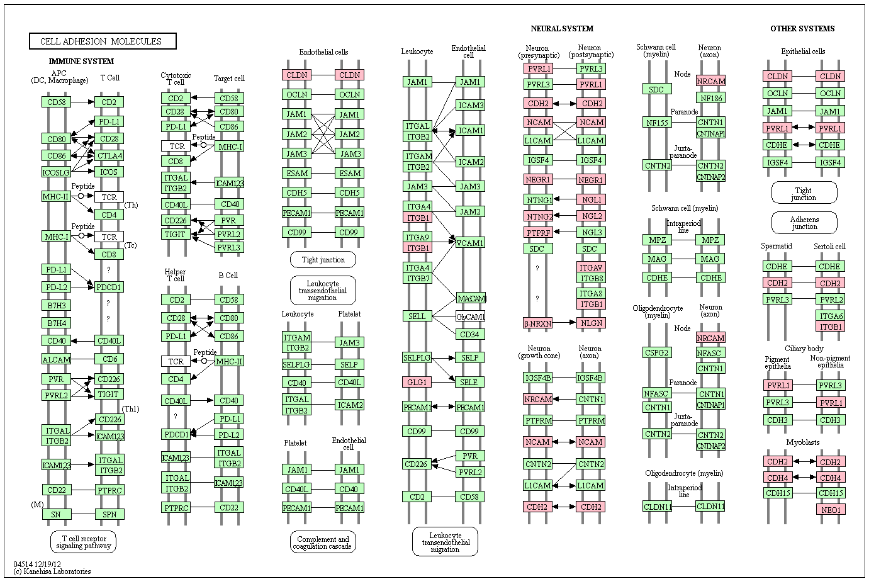

159.3 Legend:

RBH-Blast at 60% Identity + 50% Coverage
Green = Hit in *H. sapiens*
Red = Hit in *H. sapiens* and *T. californica*
White = Not in *H. sapiens*

160 RNA polymerase

160.1 Human Pathway: HSA03020

160.2 Number of Hits: 19

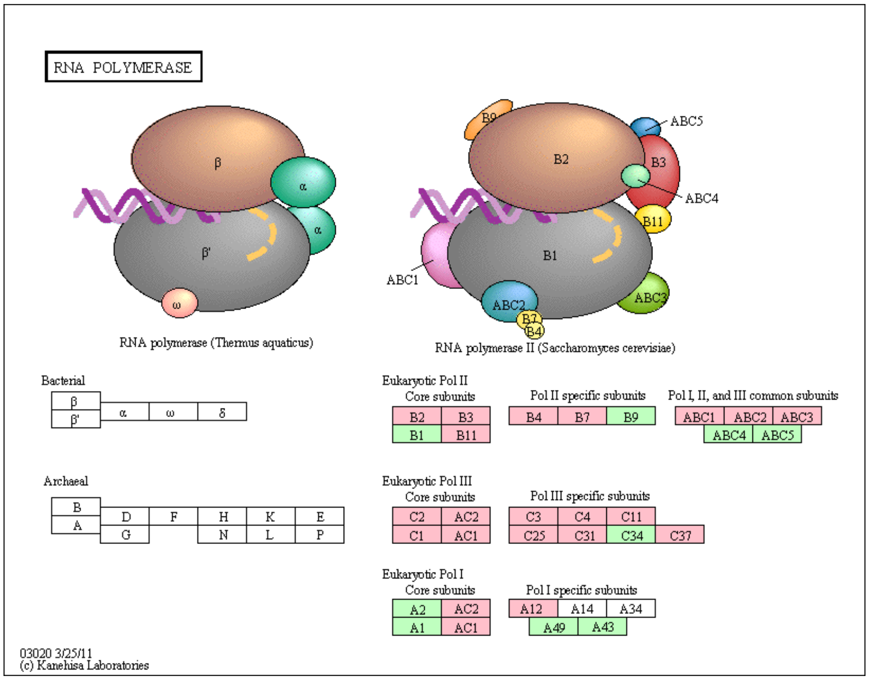

160.3 Legend:

RBH-Blast at 60% Identity + 50% Coverage
Green = Hit in *H. sapiens*
Red = Hit in *H. sapiens* and *T. californica*
White = Not in *H. sapiens*

161 Salivary secretion

161.1 Human Pathway: HSA04970

161.2 Number of Hits: 18

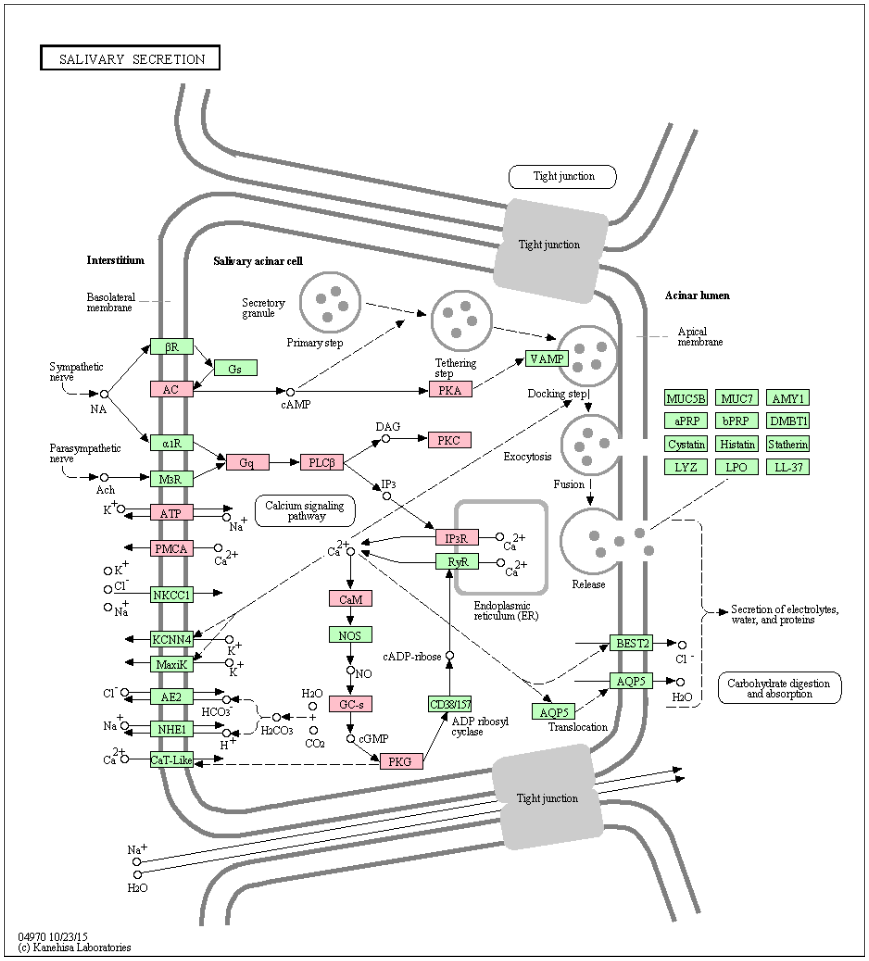

161.3 Legend:

RBH-Blast at 60% Identity + 50% Coverage
Green = Hit in *H. sapiens*
Red = Hit in *H. sapiens* and *T. californica*
White = Not in *H. sapiens*

162 Glycine, serine and threonine metabolism

162.1 Human Pathway: HSA00260

162.2 Number of Hits: 18

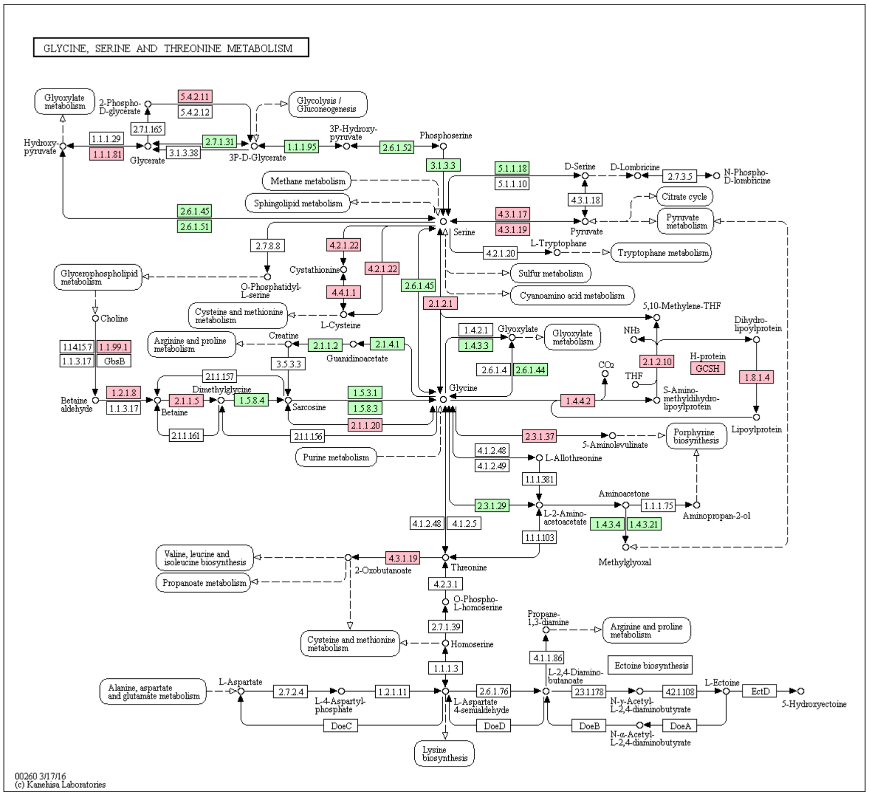

162.3 Legend:

RBH-Blast at 60% Identity + 50% Coverage
Green = Hit in *H. sapiens*
Red = Hit in *H. sapiens* and *T. californica*
White = Not in *H. sapiens*

163 p53 signaling pathway

163.1 Human Pathway: HSA04115

163.2 Number of Hits: 18

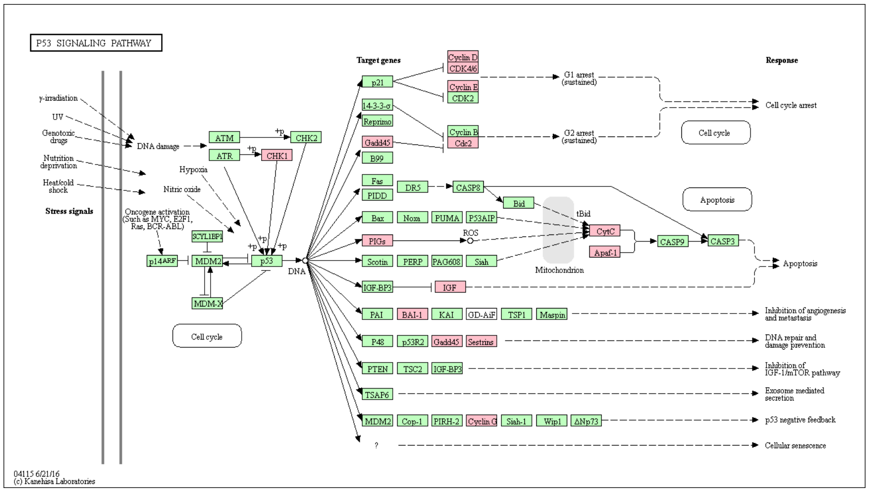

163.3 Legend:

RBH-Blast at 60% Identity + 50% Coverage
Green = Hit in *H. sapiens*
Red = Hit in *H. sapiens* and *T. californica*
White = Not in *H. sapiens*

164 Gastric acid secretion

164.1 Human Pathway: HSA04971

164.2 Number of Hits: 18

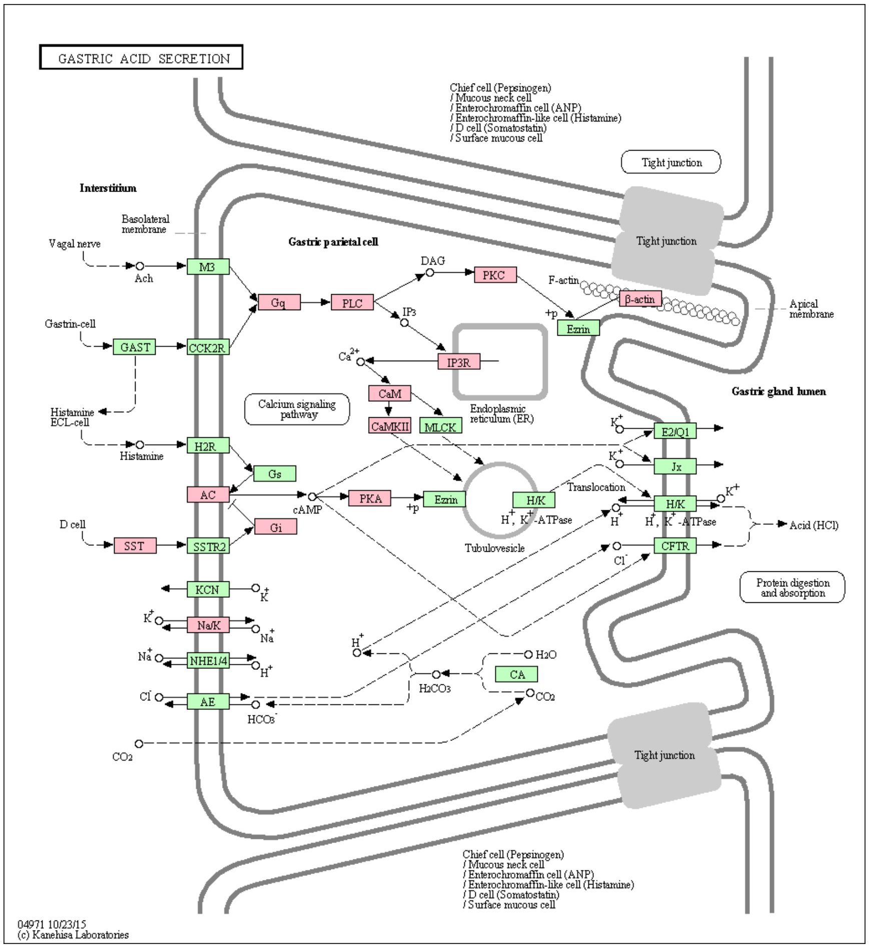

164.3 Legend:

RBH-Blast at 60% Identity + 50% Coverage
Green = Hit in *H. sapiens*
Red = Hit in *H. sapiens* and *T. californica*
White = Not in *H. sapiens*

165 PPAR signaling pathway

165.1 Human Pathway: HSA03320

165.2 Number of Hits: 18

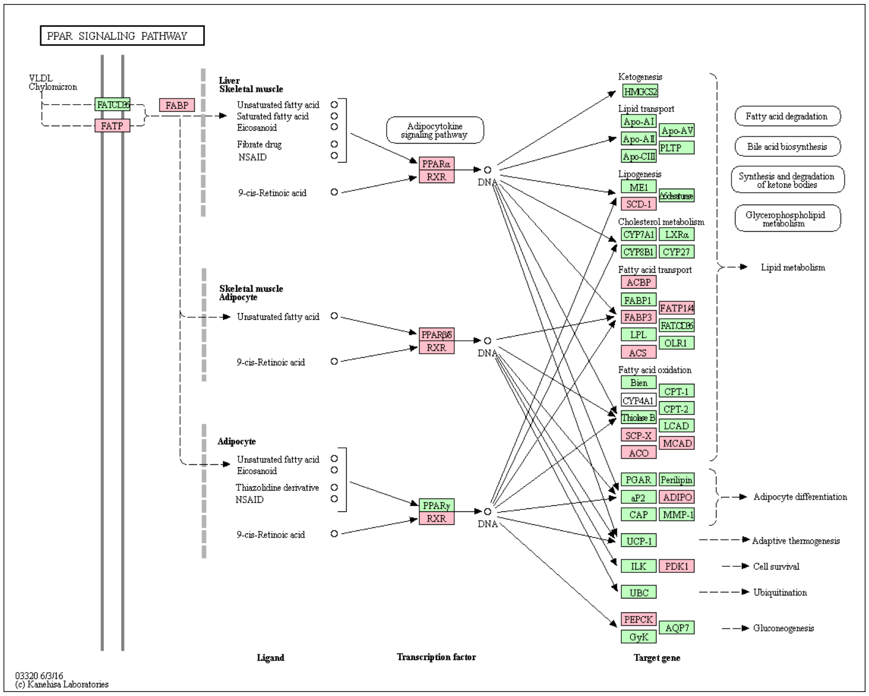

165.3 Legend:

RBH-Blast at 60% Identity + 50% Coverage
Green = Hit in *H. sapiens*
Red = Hit in *H. sapiens* and *T. californica*
White = Not in *H. sapiens*

166 Melanoma

166.1 Human Pathway: HSA05218

166.2 Number of Hits: 18

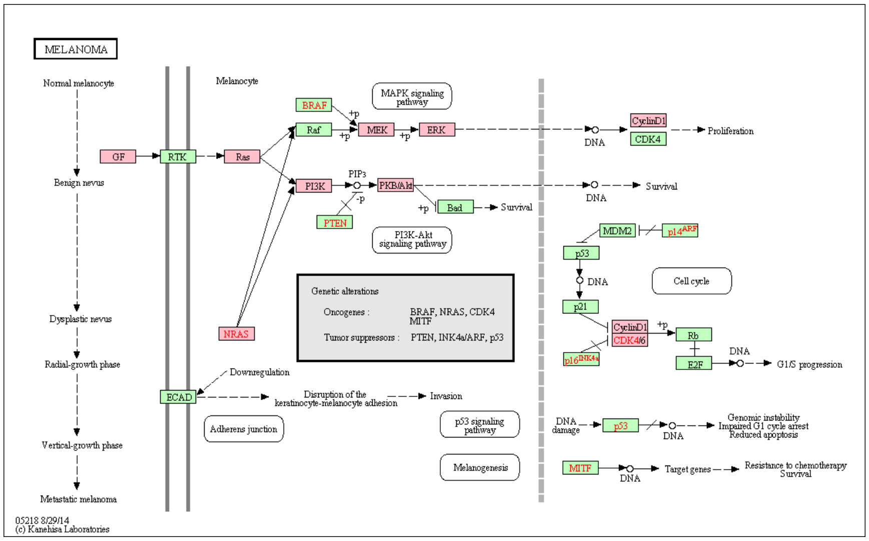

166.3 Legend:

RBH-Blast at 60% Identity + 50% Coverage
Green = Hit in *H. sapiens*
Red = Hit in *H. sapiens* and *T. californica*
White = Not in *H. sapiens*

167 Citrate cycle (TCA cycle)

167.1 Human Pathway: HSA00020

167.2 Number of Hits: 17

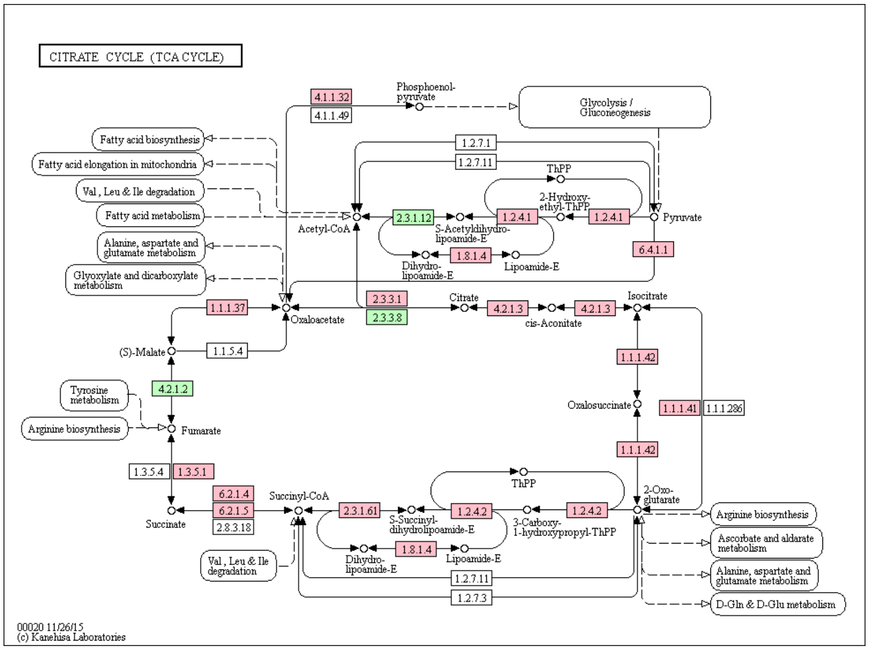

167.3 Legend:

RBH-Blast at 60% Identity + 50% Coverage
Green = Hit in *H. sapiens*
Red = Hit in *H. sapiens* and *T. californica*
White = Not in *H. sapiens*

168 Vasopressin-regulated water reabsorption

168.1 Human Pathway: HSA04962

168.2 Number of Hits: 17

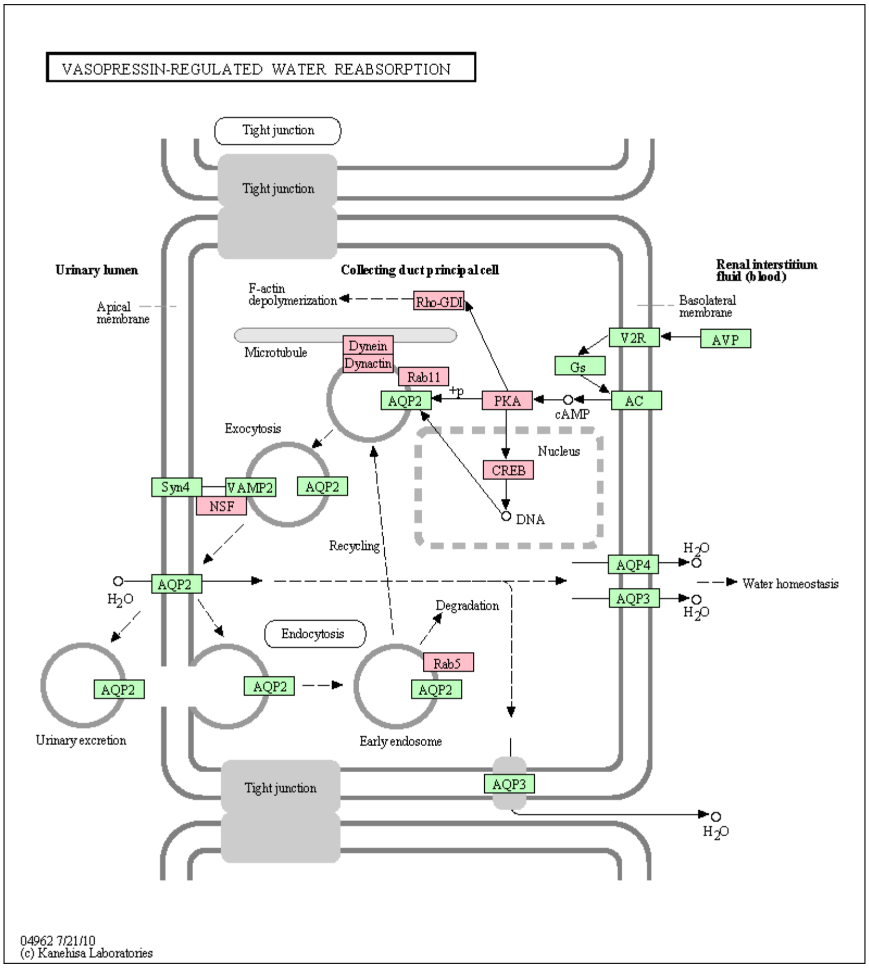

168.3 Legend:

RBH-Blast at 60% Identity + 50% Coverage
Green = Hit in *H. sapiens*
Red = Hit in *H. sapiens* and *T. californica*
White = Not in *H. sapiens*

169 Nicotine addiction

169.1 Human Pathway: HSA05033

169.2 Number of Hits: 17

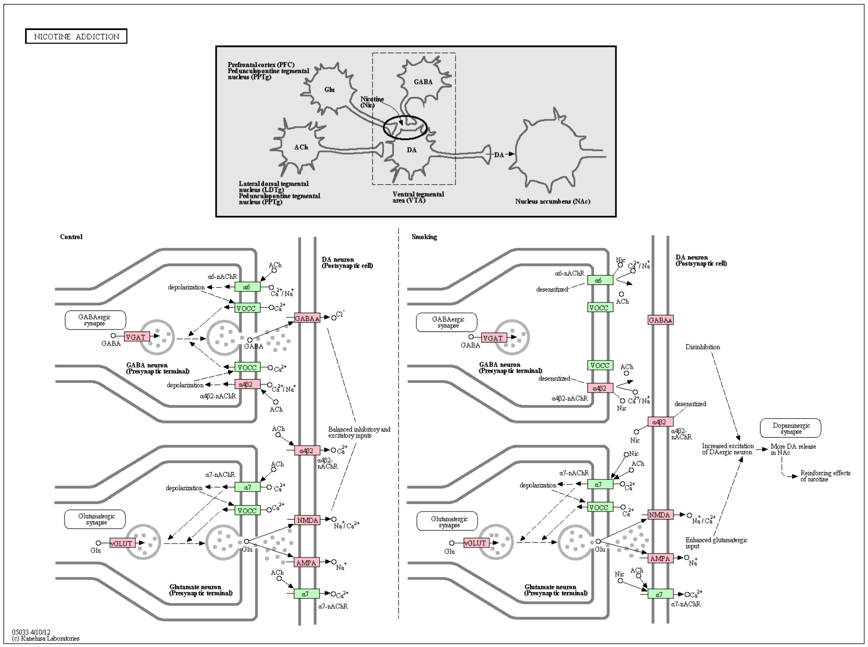

169.3 Legend:

RBH-Blast at 60% Identity + 50% Coverage
Green = Hit in *H. sapiens*
Red = Hit in *H. sapiens* and *T. californica*
White = Not in *H. sapiens*

170 Hedgehog signaling pathway

170.1 Human Pathway: HSA04340

170.2 Number of Hits: 17

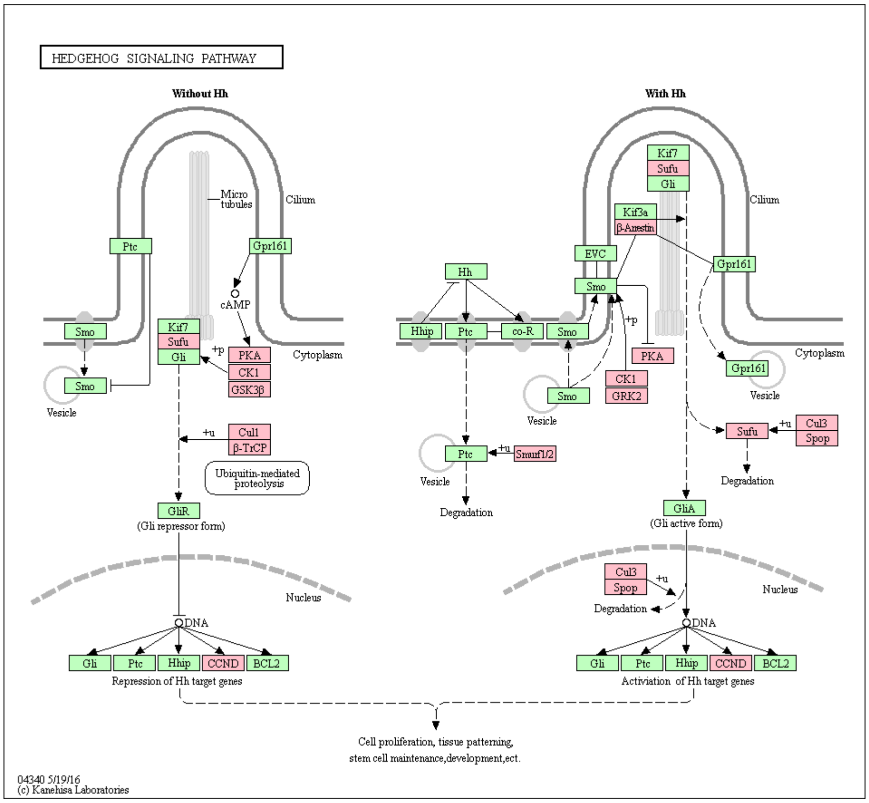

170.3 Legend:

RBH-Blast at 60% Identity + 50% Coverage
Green = Hit in *H. sapiens*
Red = Hit in *H. sapiens* and *T. californica*
White = Not in *H. sapiens*

171 beta-Alanine metabolism

171.1 Human Pathway: HSA00410

171.2 Number of Hits: 17

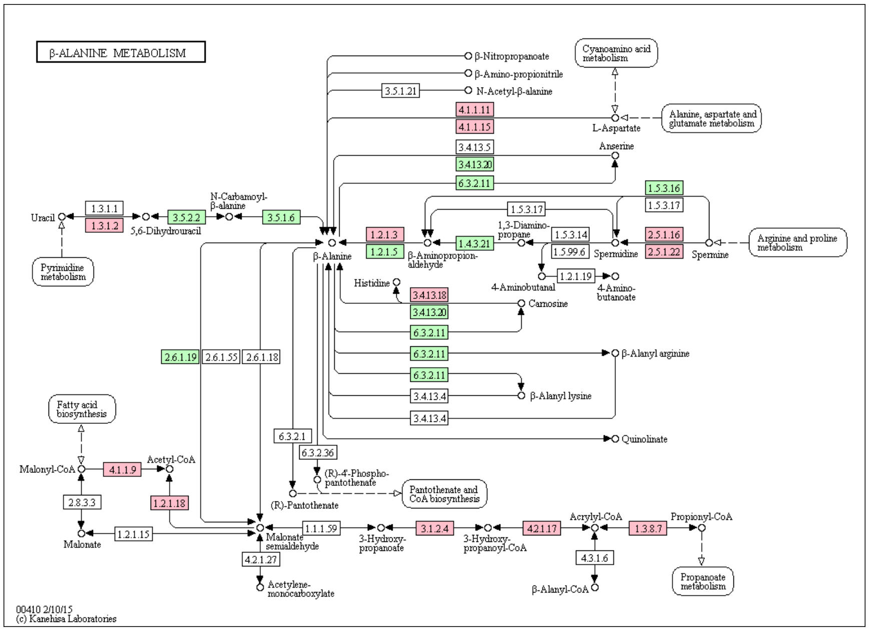

171.3 Legend:

RBH-Blast at 60% Identity + 50% Coverage
Green = Hit in *H. sapiens*
Red = Hit in *H. sapiens* and *T. californica*
White = Not in *H. sapiens*

172 DNA replication

172.1 Human Pathway: HSA03030

172.2 Number of Hits: 17

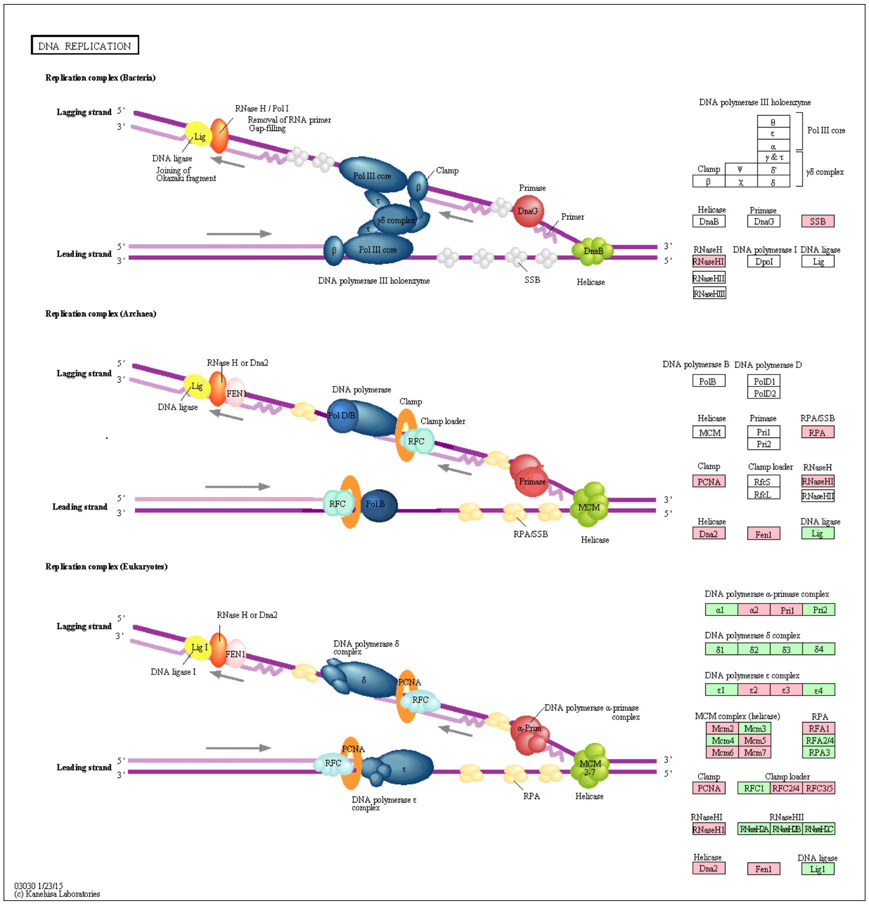

172.3 Legend:

RBH-Blast at 60% Identity + 50% Coverage
Green = Hit in *H. sapiens*
Red = Hit in *H. sapiens* and *T. californica*
White = Not in *H. sapiens*

173 Endometrial cancer

173.1 Human Pathway: HSA05213

173.2 Number of Hits: 17

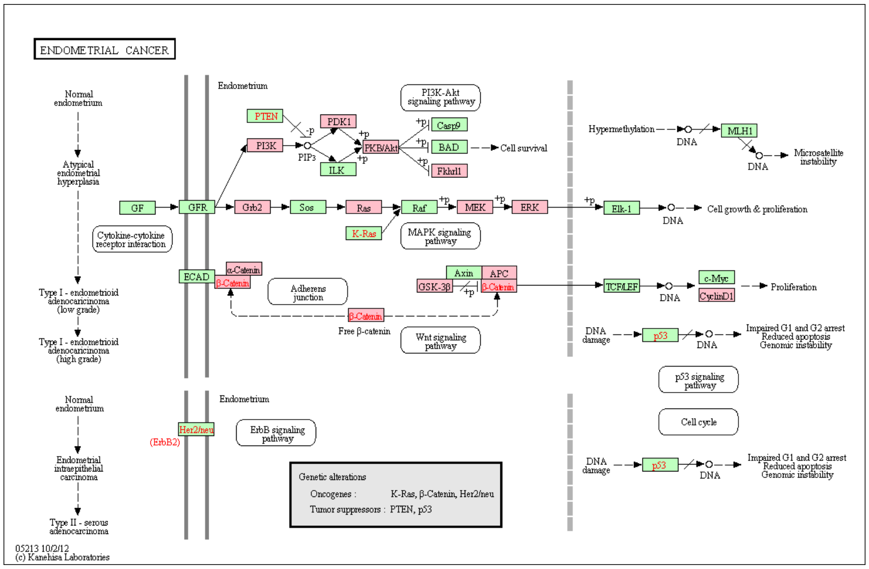

173.3 Legend:

RBH-Blast at 60% Identity + 50% Coverage
Green = Hit in *H. sapiens*
Red = Hit in *H. sapiens* and *T. californica*
White = Not in *H. sapiens*

174 Protein export

174.1 Human Pathway: HSA03060

174.2 Number of Hits: 17

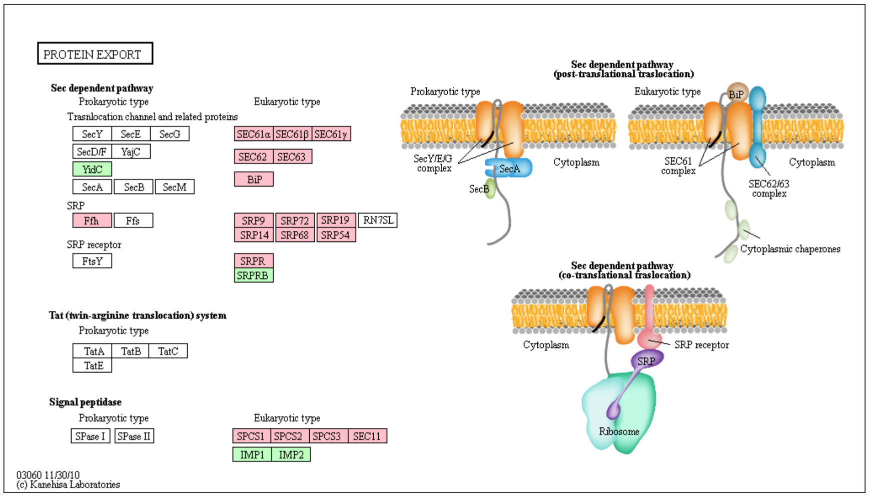

174.3 Legend:

RBH-Blast at 60% Identity + 50% Coverage
Green = Hit in *H. sapiens*
Red = Hit in *H. sapiens* and *T. californica*
White = Not in *H. sapiens*

175 Renin secretion

175.1 Human Pathway: HSA04924

175.2 Number of Hits: 17

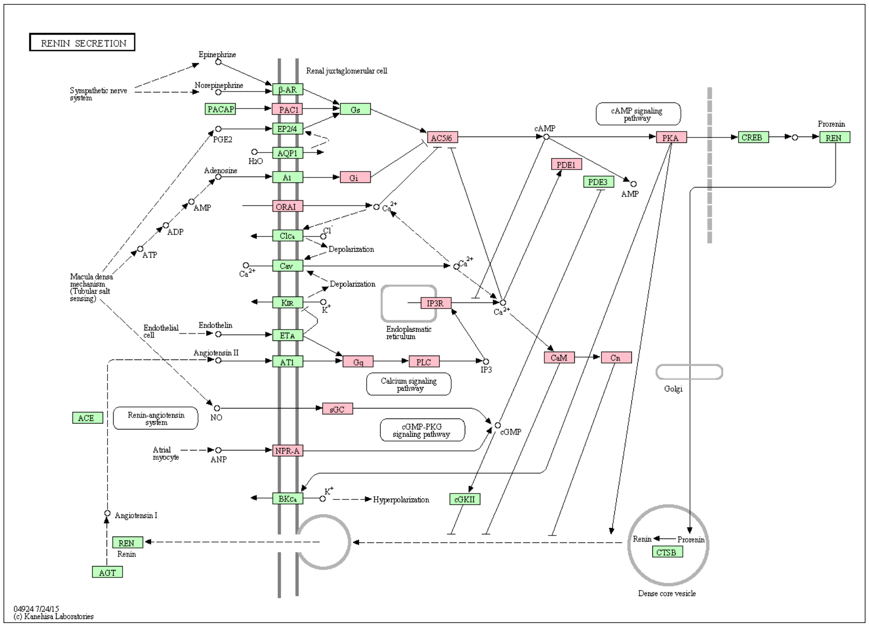

175.3 Legend:

RBH-Blast at 60% Identity + 50% Coverage
Green = Hit in *H. sapiens*
Red = Hit in *H. sapiens* and *T. californica*
White = Not in *H. sapiens*

176 Cocaine addiction

176.1 Human Pathway: HSA05030

176.2 Number of Hits: 16

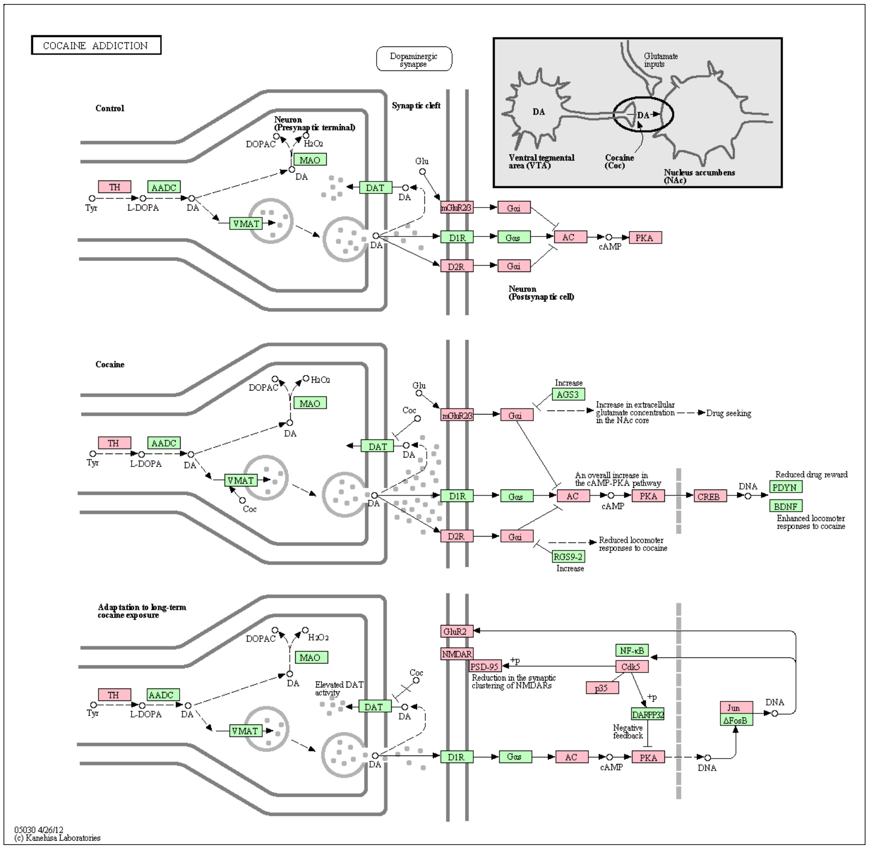

176.3 Legend:

RBH-Blast at 60% Identity + 50% Coverage
Green = Hit in *H. sapiens*
Red = Hit in *H. sapiens* and *T. californica*
White = Not in *H. sapiens*

177 Sphingolipid metabolism

177.1 Human Pathway: HSA00600

177.2 Number of Hits: 16

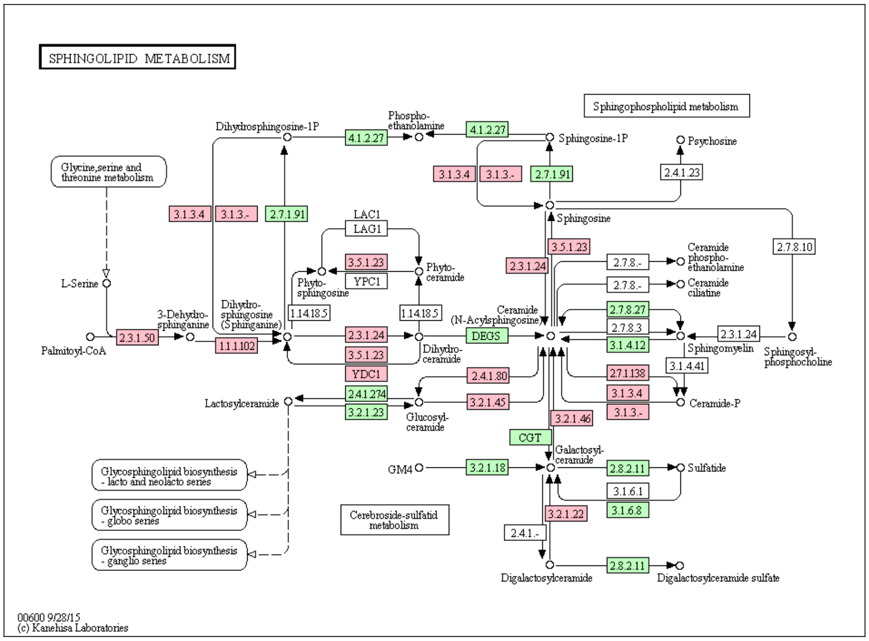

177.3 Legend:

RBH-Blast at 60% Identity + 50% Coverage
Green = Hit in *H. sapiens*
Red = Hit in *H. sapiens* and *T. californica*
White = Not in *H. sapiens*

178 Cytosolic DNA-sensing pathway

178.1 Human Pathway: HSA04623

178.2 Number of Hits: 16

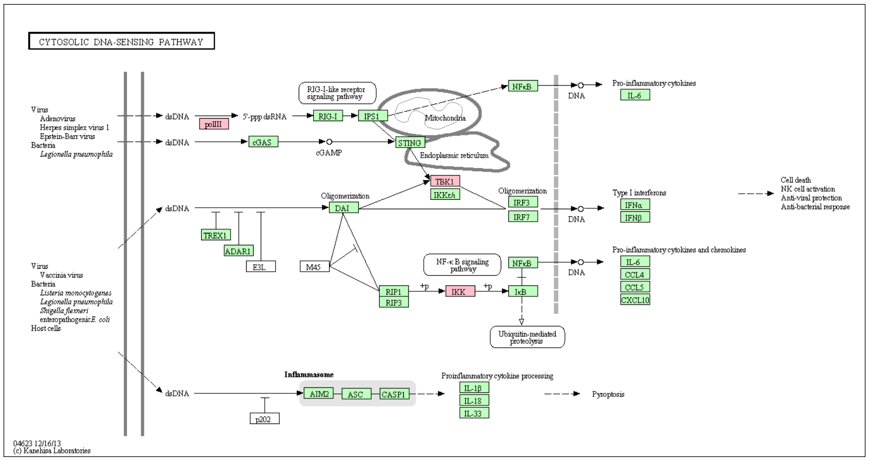

178.3 Legend:

RBH-Blast at 60% Identity + 50% Coverage
Green = Hit in *H. sapiens*
Red = Hit in *H. sapiens* and *T. californica*
White = Not in *H. sapiens*

179 Alanine, aspartate and glutamate metabolism

179.1 Human Pathway: HSA00250

179.2 Number of Hits: 16

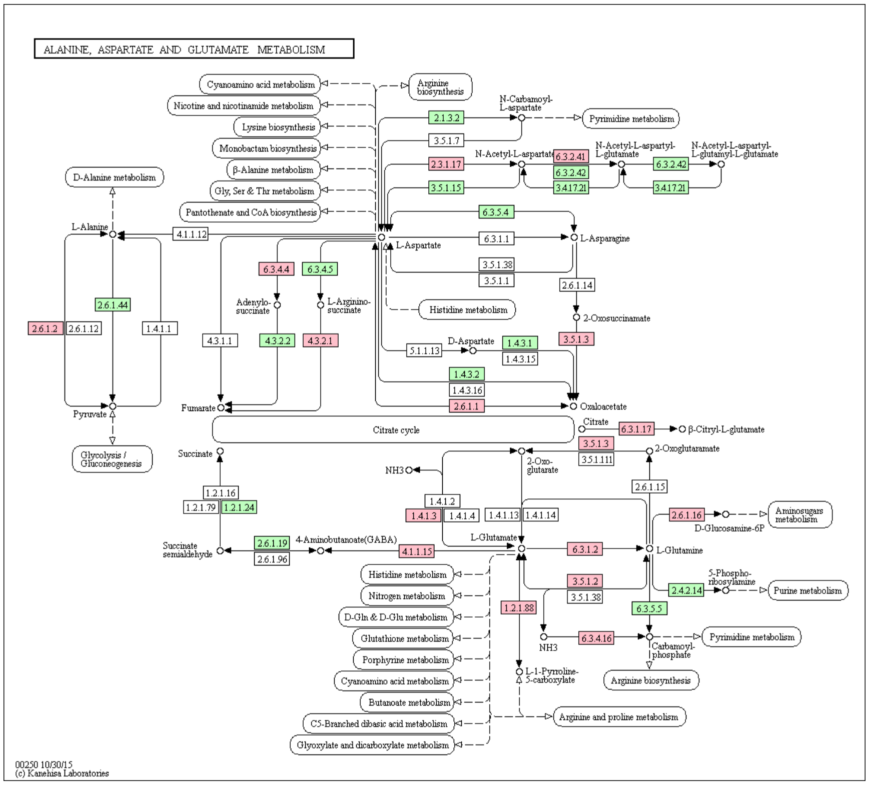

179.3 Legend:

RBH-Blast at 60% Identity + 50% Coverage
Green = Hit in *H. sapiens*
Red = Hit in *H. sapiens* and *T. californica*
White = Not in *H. sapiens*

180 Other types of O-glycan biosynthesis

180.1 Human Pathway: HSA00514

180.2 Number of Hits: 16

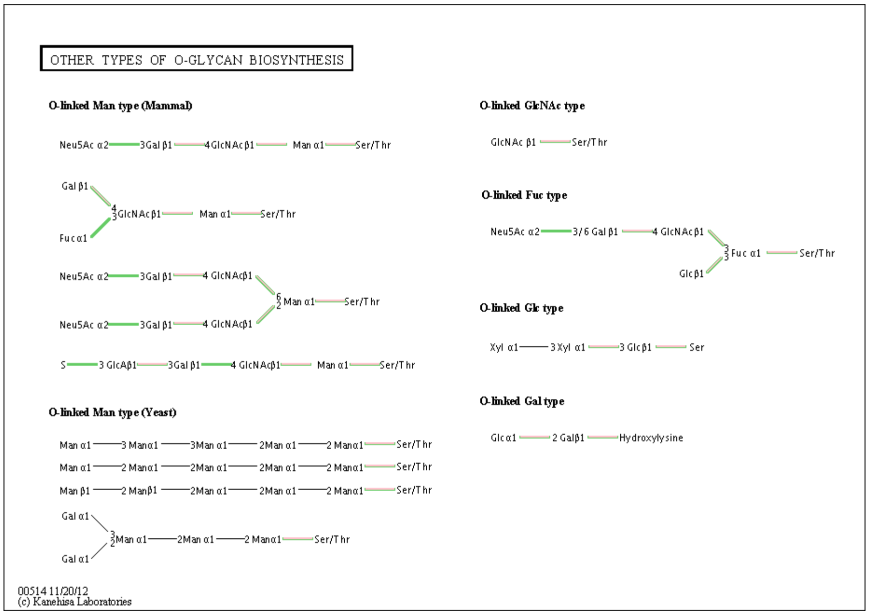

180.3 Legend:

RBH-Blast at 60% Identity + 50% Coverage
Green = Hit in *H. sapiens*
Red = Hit in *H. sapiens* and *T. californica*
White = Not in *H. sapiens*

181 Rheumatoid arthritis

181.1 Human Pathway: HSA05323

181.2 Number of Hits: 15

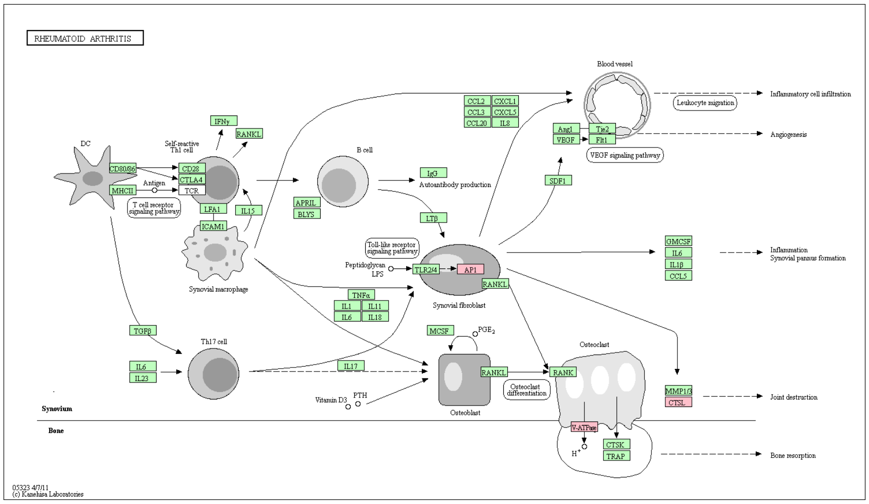

181.3 Legend:

RBH-Blast at 60% Identity + 50% Coverage
Green = Hit in *H. sapiens*
Red = Hit in *H. sapiens* and *T. californica*
White = Not in *H. sapiens*

182 NF-kappa B signaling pathway

182.1 Human Pathway: HSA04064

182.2 Number of Hits: 15

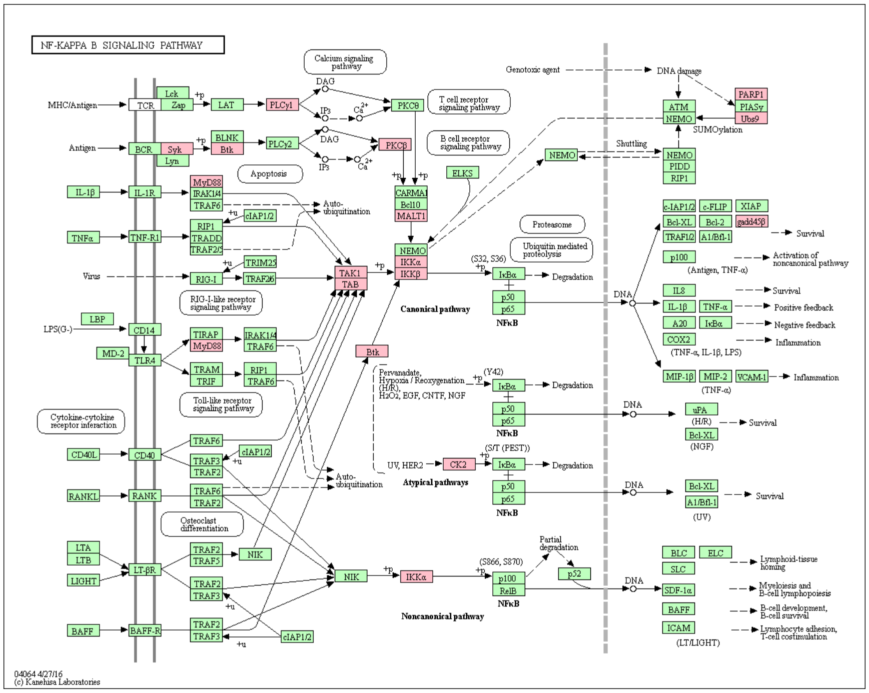

182.3 Legend:

RBH-Blast at 60% Identity + 50% Coverage
Green = Hit in *H. sapiens*
Red = Hit in *H. sapiens* and *T. californica*
White = Not in *H. sapiens*

183 Fructose and mannose metabolism

183.1 Human Pathway: HSA00051

183.2 Number of Hits: 15

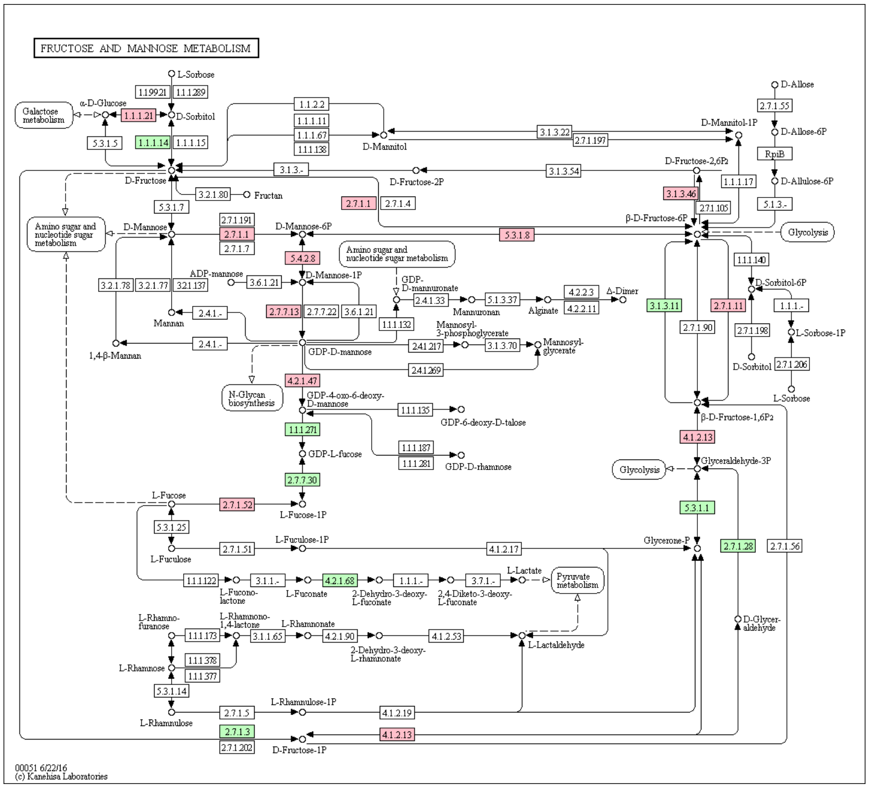

183.3 Legend:

RBH-Blast at 60% Identity + 50% Coverage
Green = Hit in *H. sapiens*
Red = Hit in *H. sapiens* and *T. californica*
White = Not in *H. sapiens*

184 Type II diabetes mellitus

184.1 Human Pathway: HSA04930

184.2 Number of Hits: 15

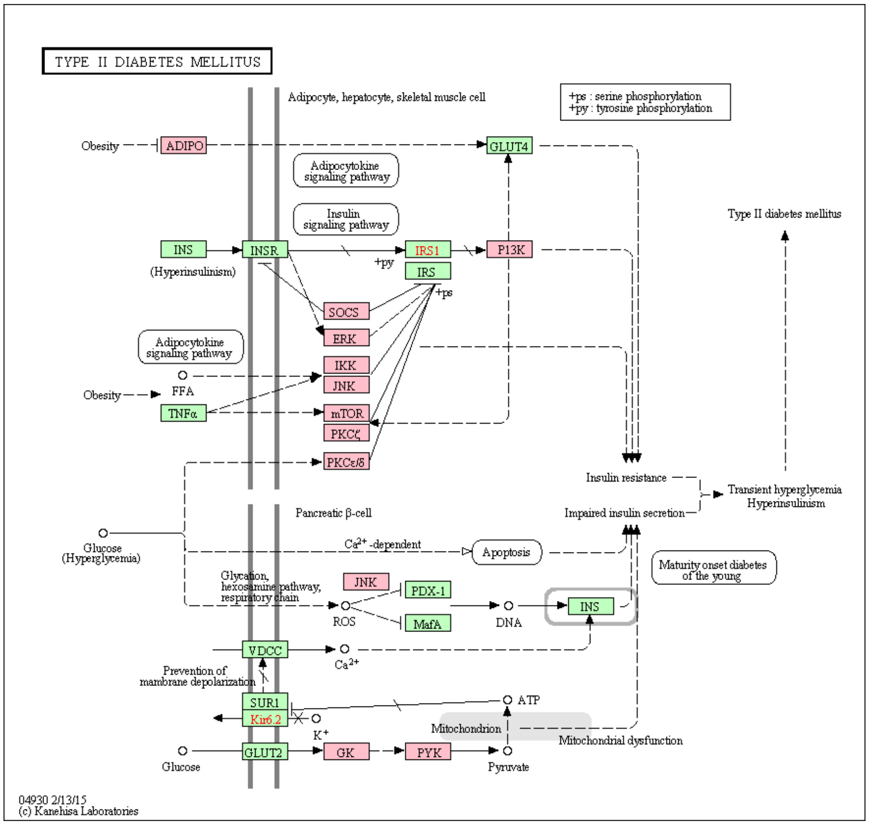

184.3 Legend:

RBH-Blast at 60% Identity + 50% Coverage
Green = Hit in *H. sapiens*
Red = Hit in *H. sapiens* and *T. californica*
White = Not in *H. sapiens*

185 Regulation of lipolysis in adipocytes

185.1 Human Pathway: HSA04923

185.2 Number of Hits: 15

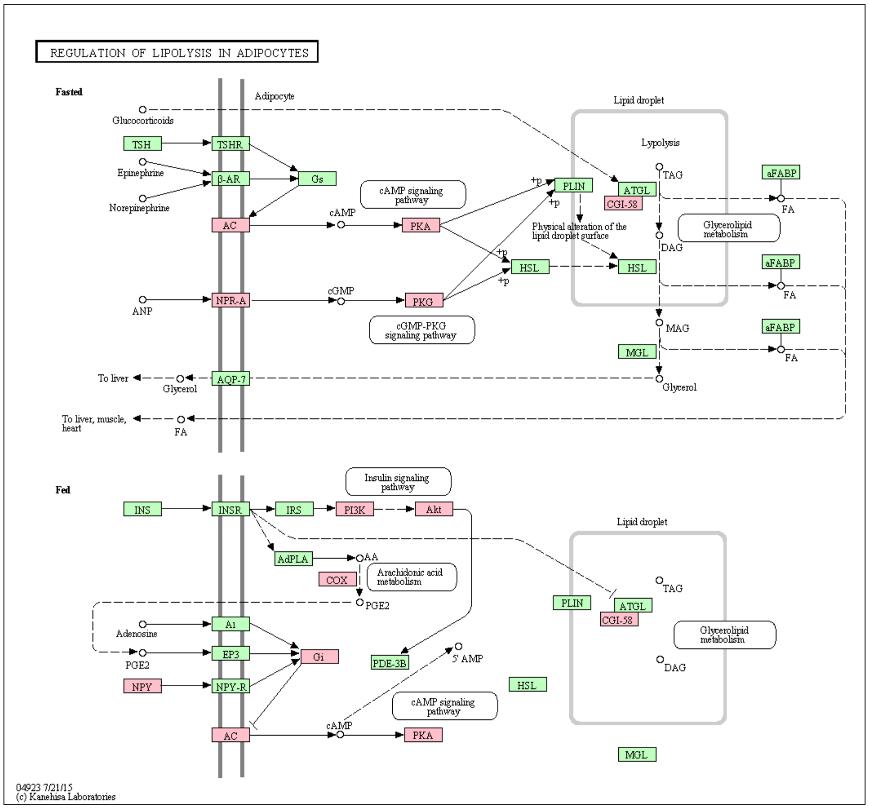

185.3 Legend:

RBH-Blast at 60% Identity + 50% Coverage
Green = Hit in *H. sapiens*
Red = Hit in *H. sapiens* and *T. californica*
White = Not in *H. sapiens*

186 Pertussis

186.1 Human Pathway: HSA05133

186.2 Number of Hits: 15

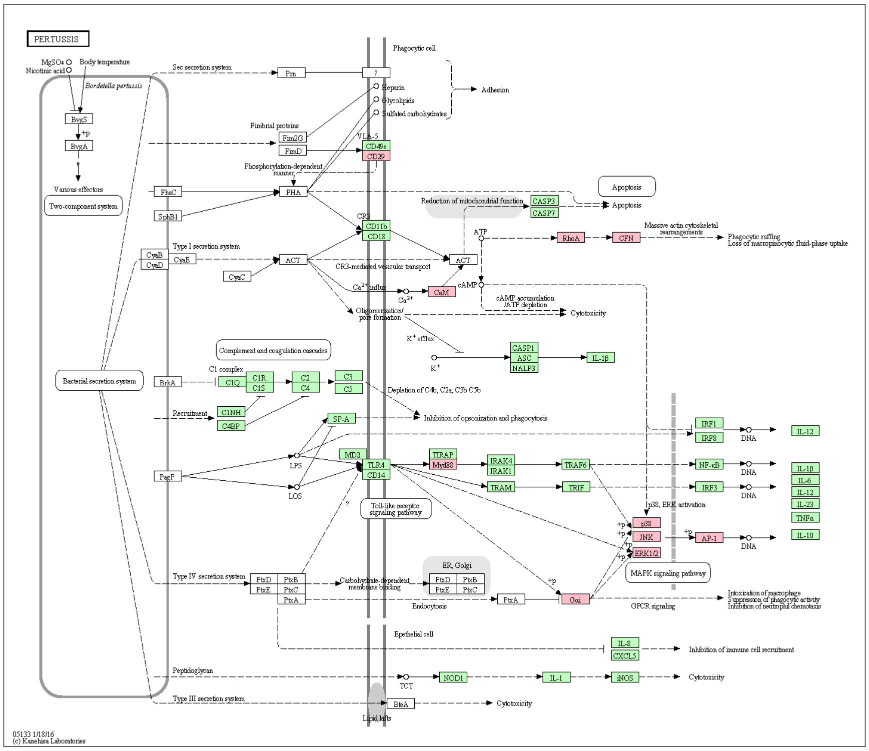

186.3 Legend:

RBH-Blast at 60% Identity + 50% Coverage
Green = Hit in *H. sapiens*
Red = Hit in *H. sapiens* and *T. californica*
White = Not in *H. sapiens*

187 Legionellosis

187.1 Human Pathway: HSA05134

187.2 Number of Hits: 14

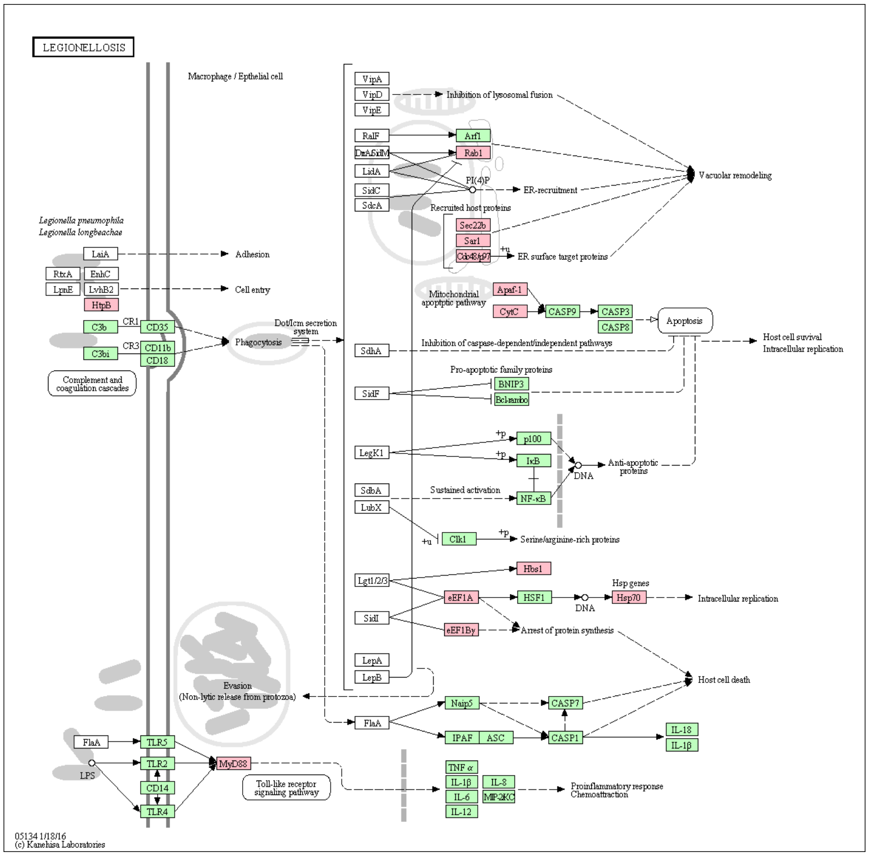

187.3 Legend:

RBH-Blast at 60% Identity + 50% Coverage
Green = Hit in *H. sapiens*
Red = Hit in *H. sapiens* and *T. californica*
White = Not in *H. sapiens*

188 Protein digestion and absorption

188.1 Human Pathway: HSA04974

188.2 Number of Hits: 14

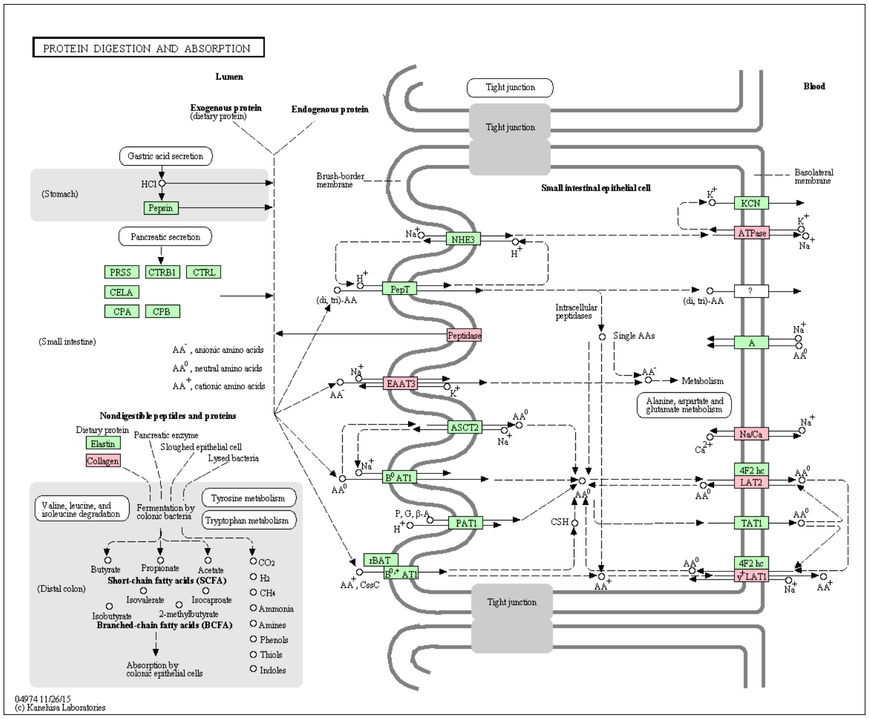

188.3 Legend:

RBH-Blast at 60% Identity + 50% Coverage
Green = Hit in *H. sapiens*
Red = Hit in *H. sapiens* and *T. californica*
White = Not in *H. sapiens*

189 Steroid biosynthesis

189.1 Human Pathway: HSA00100

189.2 Number of Hits: 14

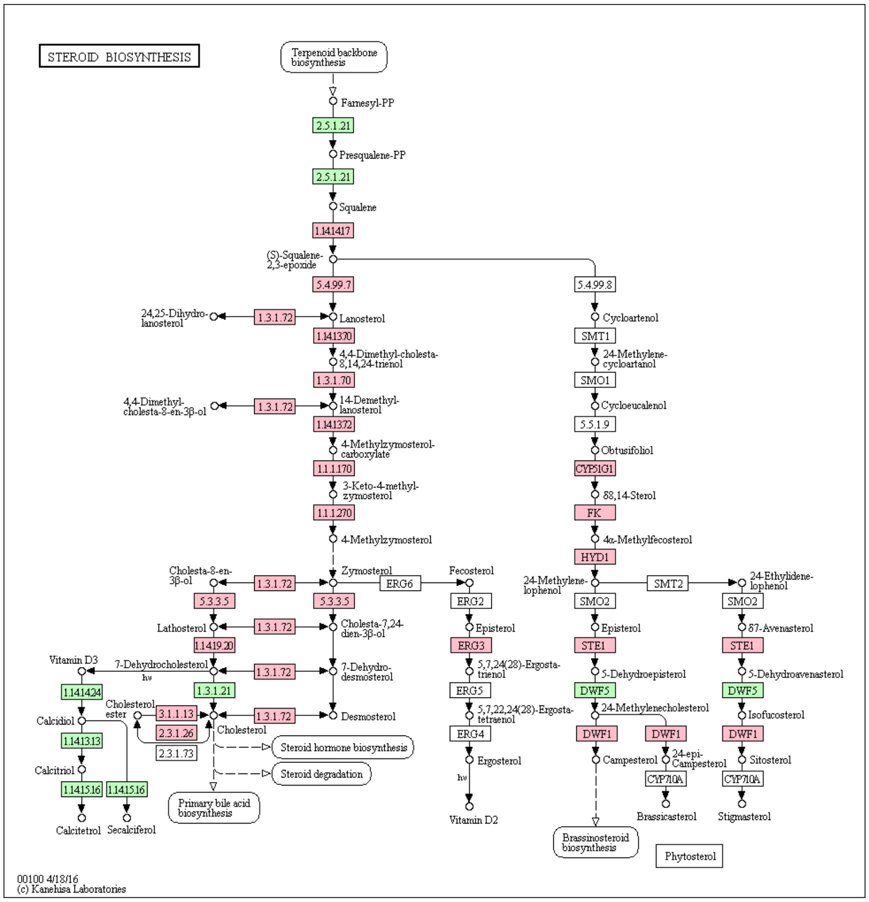

189.3 Legend:

RBH-Blast at 60% Identity + 50% Coverage
Green = Hit in *H. sapiens*
Red = Hit in *H. sapiens* and *T. californica*
White = Not in *H. sapiens*

190 NOD-like receptor signaling pathway

190.1 Human Pathway: HSA04621

190.2 Number of Hits: 14

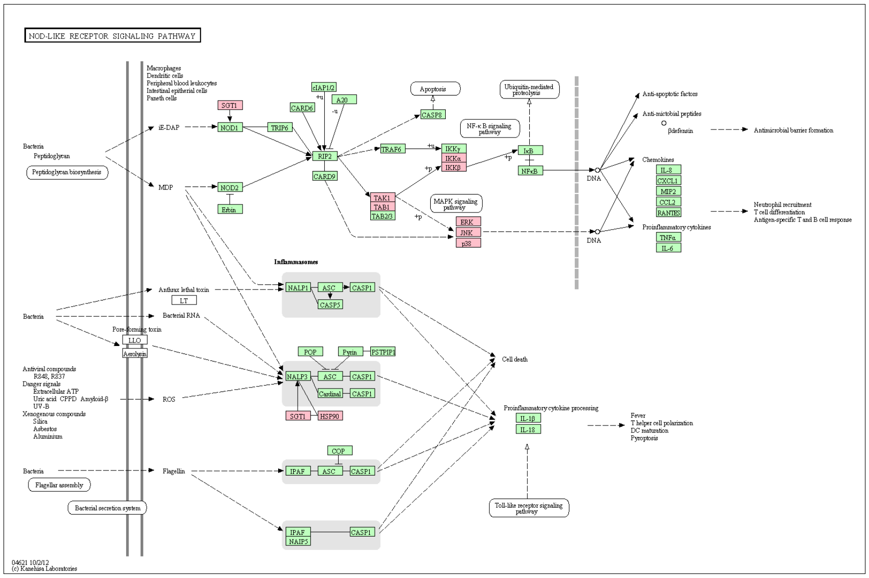

190.3 Legend:

RBH-Blast at 60% Identity + 50% Coverage
Green = Hit in *H. sapiens*
Red = Hit in *H. sapiens* and *T. californica*
White = Not in *H. sapiens*

191 ECM-receptor interaction

191.1 Human Pathway: HSA04512

191.2 Number of Hits: 14

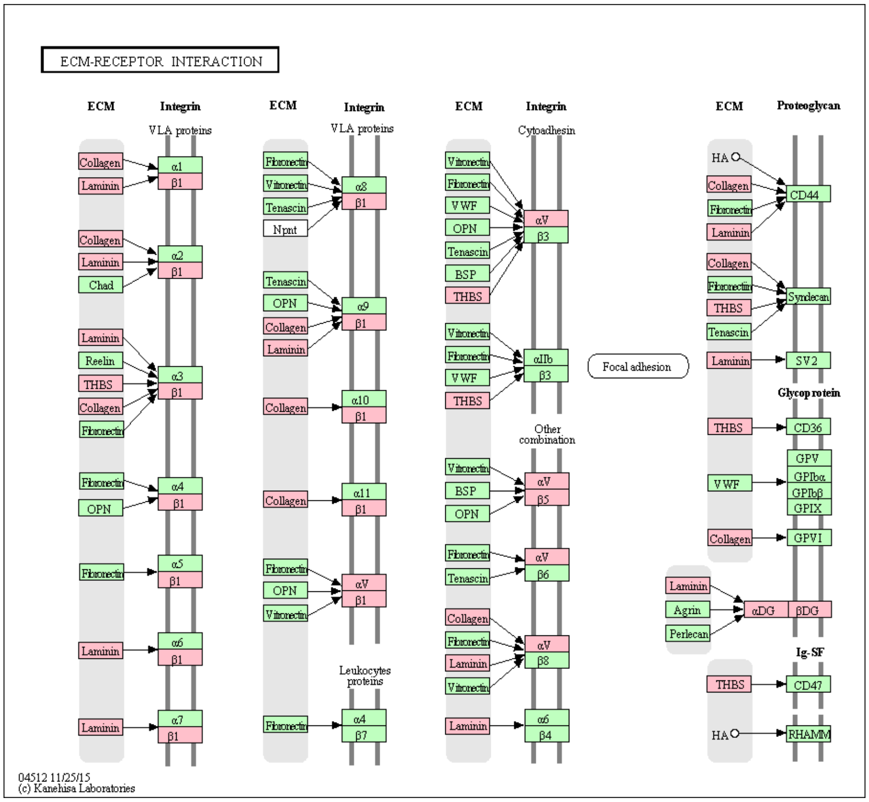

191.3 Legend:

RBH-Blast at 60% Identity + 50% Coverage
Green = Hit in *H. sapiens*
Red = Hit in *H. sapiens* and *T. californica*
White = Not in *H. sapiens*

192 Fatty acid elongation

192.1 Human Pathway: HSA00062

192.2 Number of Hits: 14

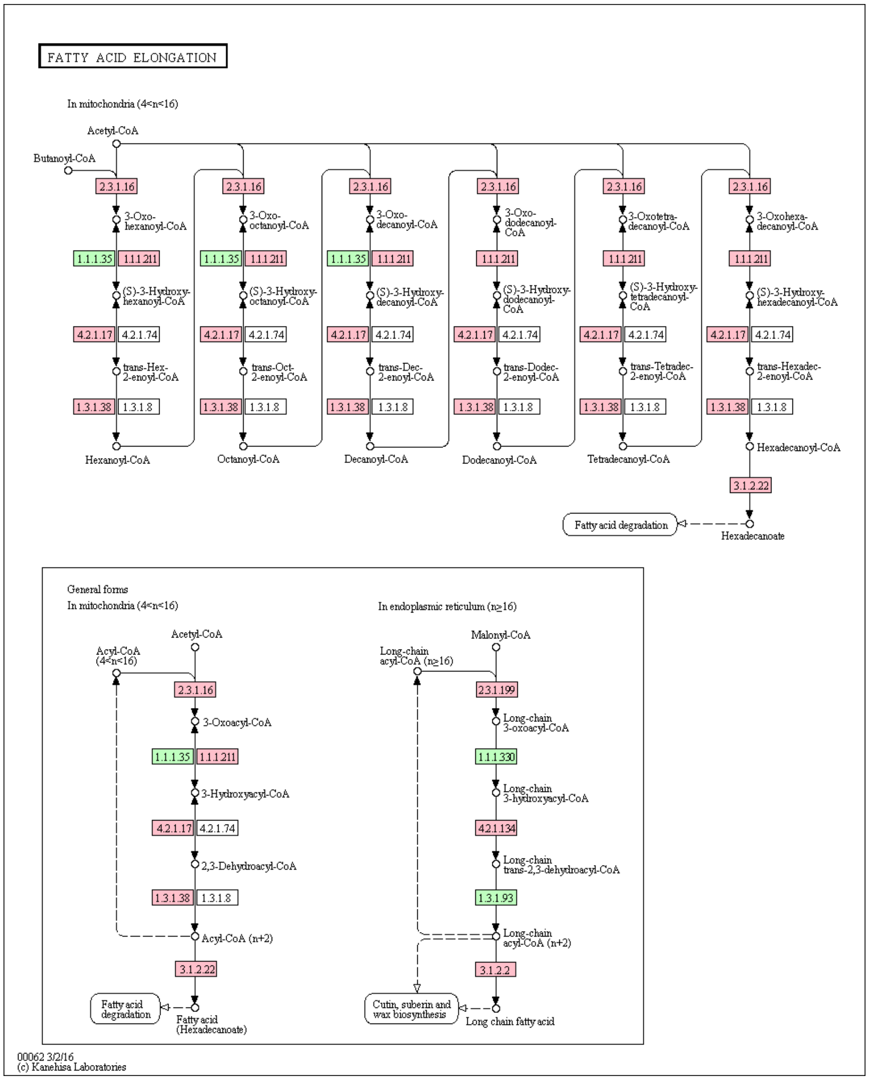

192.3 Legend:

RBH-Blast at 60% Identity + 50% Coverage
Green = Hit in *H. sapiens*
Red = Hit in *H. sapiens* and *T. californica*
White = Not in *H. sapiens*

193 Butanoate metabolism

193.1 Human Pathway: HSA00650

193.2 Number of Hits: 13

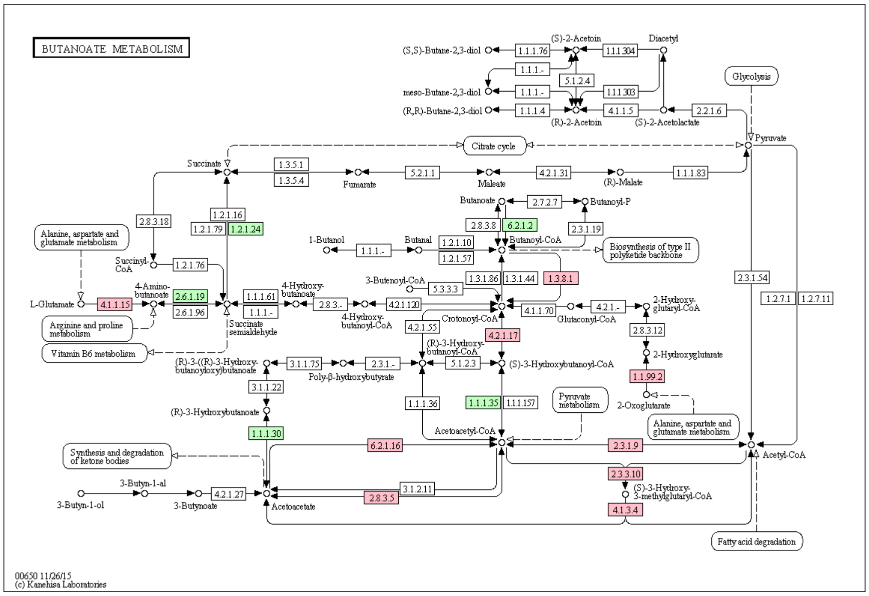

193.3 Legend:

RBH-Blast at 60% Identity + 50% Coverage
Green = Hit in *H. sapiens*
Red = Hit in *H. sapiens* and *T. californica*
White = Not in *H. sapiens*

194 Tryptophan metabolism

194.1 Human Pathway: HSA00380

194.2 Number of Hits: 13

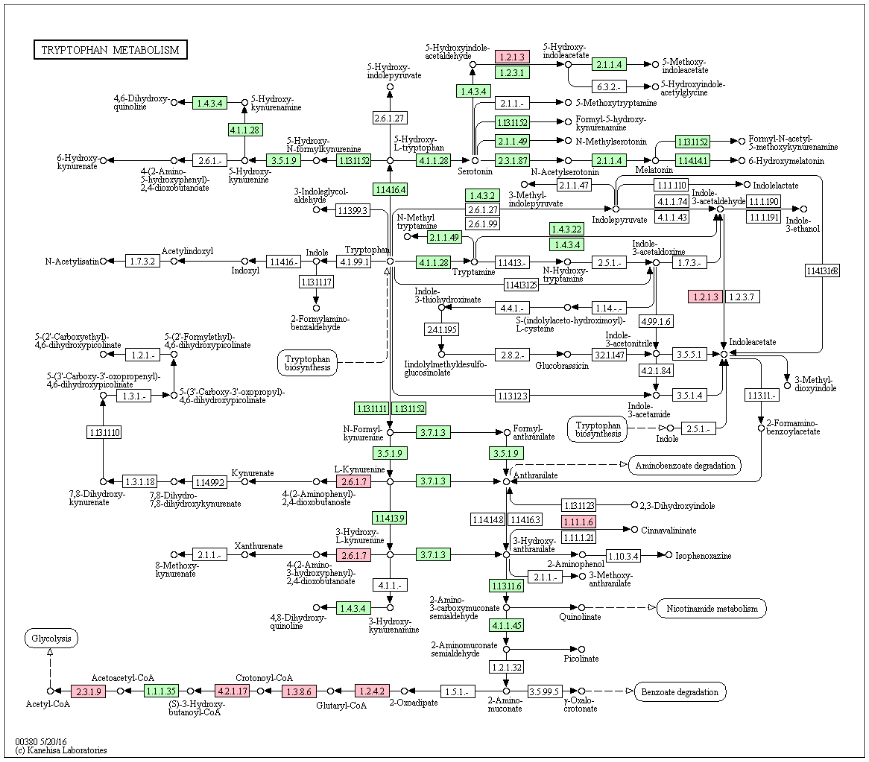

194.3 Legend:

RBH-Blast at 60% Identity + 50% Coverage
Green = Hit in *H. sapiens*
Red = Hit in *H. sapiens* and *T. californica*
White = Not in *H. sapiens*

195 Terpenoid backbone biosynthesis

195.1 Human Pathway: HSA00900

195.2 Number of Hits: 13

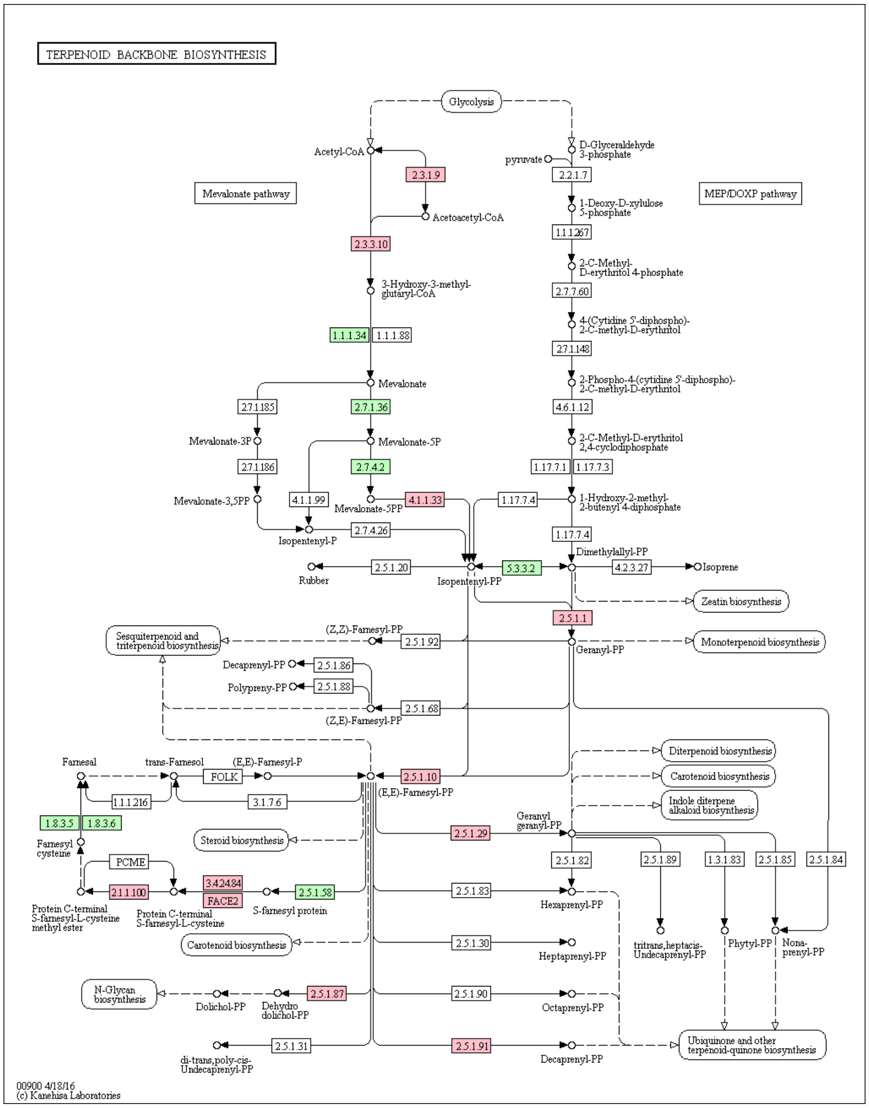

195.3 Legend:

RBH-Blast at 60% Identity + 50% Coverage
Green = Hit in *H. sapiens*
Red = Hit in *H. sapiens* and *T. californica*
White = Not in *H. sapiens*

---

196 RIG-I-like receptor signaling pathway

196.1 Human Pathway: HSA04622

196.2 Number of Hits: 13

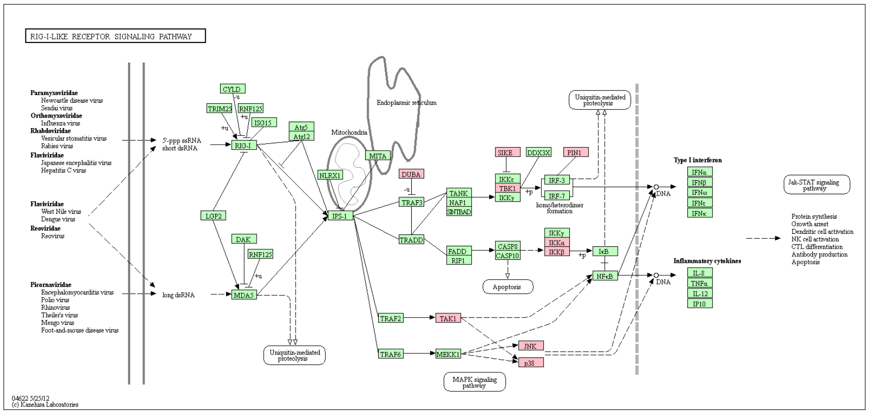

196.3 Legend:

RBH-Blast at 60% Identity + 50% Coverage
Green = Hit in *H. sapiens*
Red = Hit in *H. sapiens* and *T. californica*
White = Not in *H. sapiens*

---

197 Leishmaniasis

197.1 Human Pathway: HSA05140

197.2 Number of Hits: 13

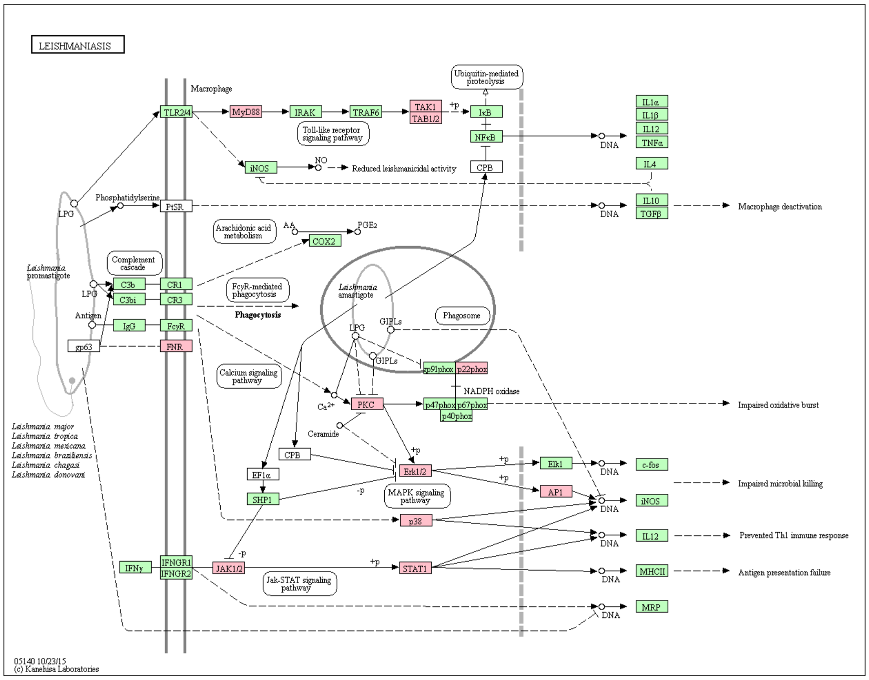

197.3 Legend:

RBH-Blast at 60% Identity + 50% Coverage
Green = Hit in *H. sapiens*
Red = Hit in *H. sapiens* and *T. californica*
White = Not in *H. sapiens*

198 Platinum drug resistance

198.1 Human Pathway: HSA01524

198.2 Number of Hits: 13

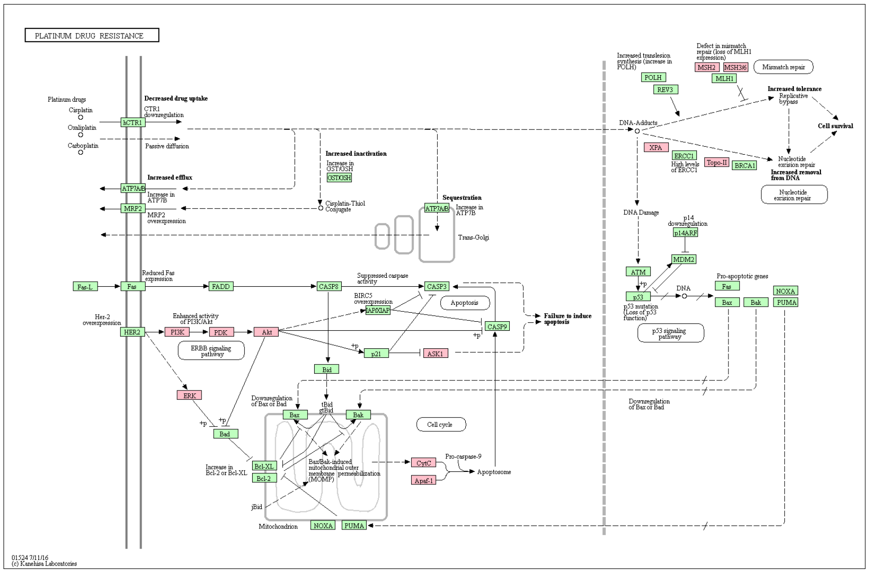

198.3 Legend:

RBH-Blast at 60% Identity + 50% Coverage
Green = Hit in *H. sapiens*
Red = Hit in *H. sapiens* and *T. californica*
White = Not in *H. sapiens*

199 Olfactory transduction

199.1 Human Pathway: HSA04740

199.2 Number of Hits: 13

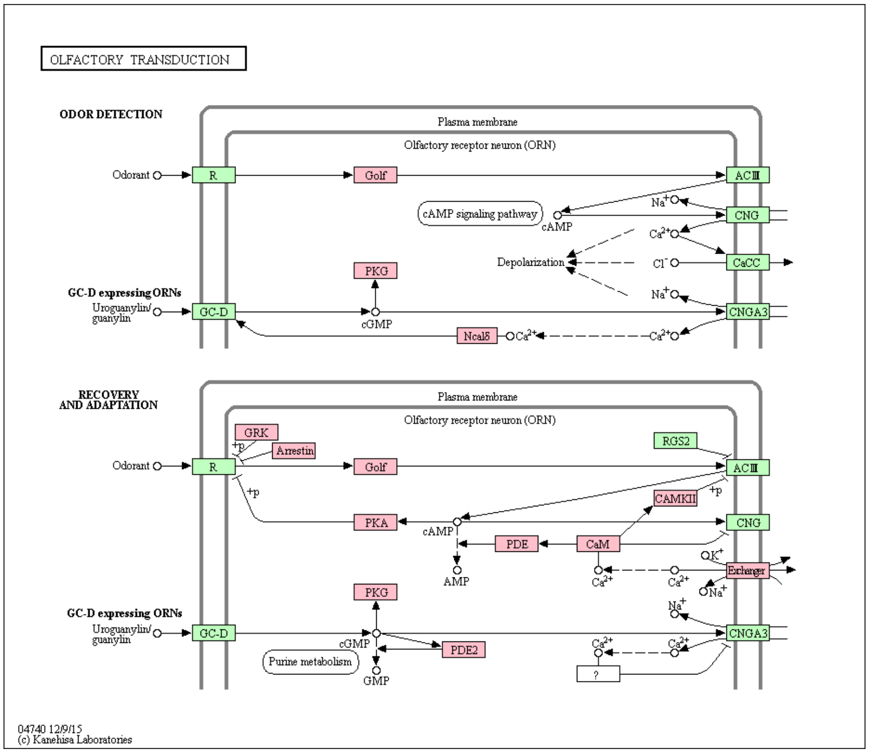

199.3 Legend:

RBH-Blast at 60% Identity + 50% Coverage
Green = Hit in *H. sapiens*
Red = Hit in *H. sapiens* and *T. californica*
White = Not in *H. sapiens*

200 Viral myocarditis

200.1 Human Pathway: HSA05416

200.2 Number of Hits: 13

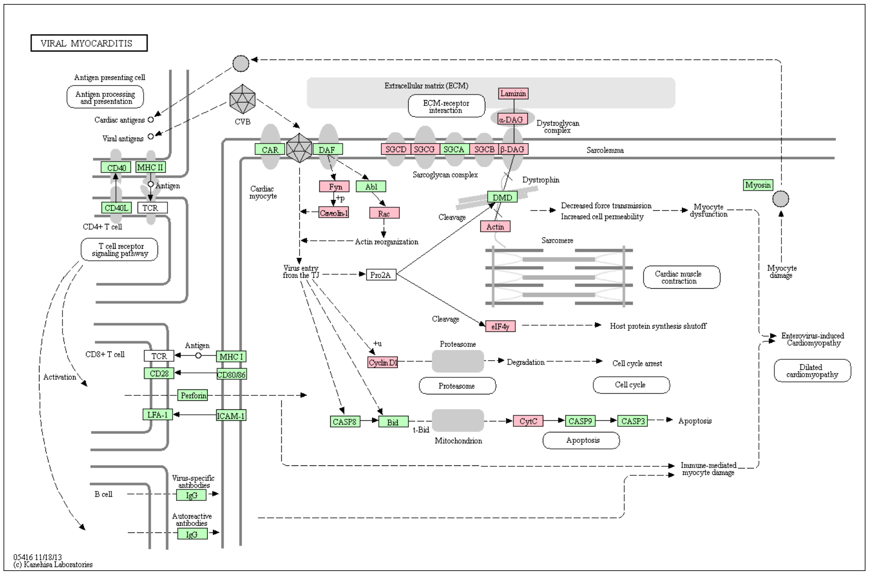

200.3 Legend:

RBH-Blast at 60% Identity + 50% Coverage
Green = Hit in *H. sapiens*
Red = Hit in *H. sapiens* and *T. californica*
White = Not in *H. sapiens*

201 Pentose phosphate pathway

201.1 Human Pathway: HSA00030

201.2 Number of Hits: 13

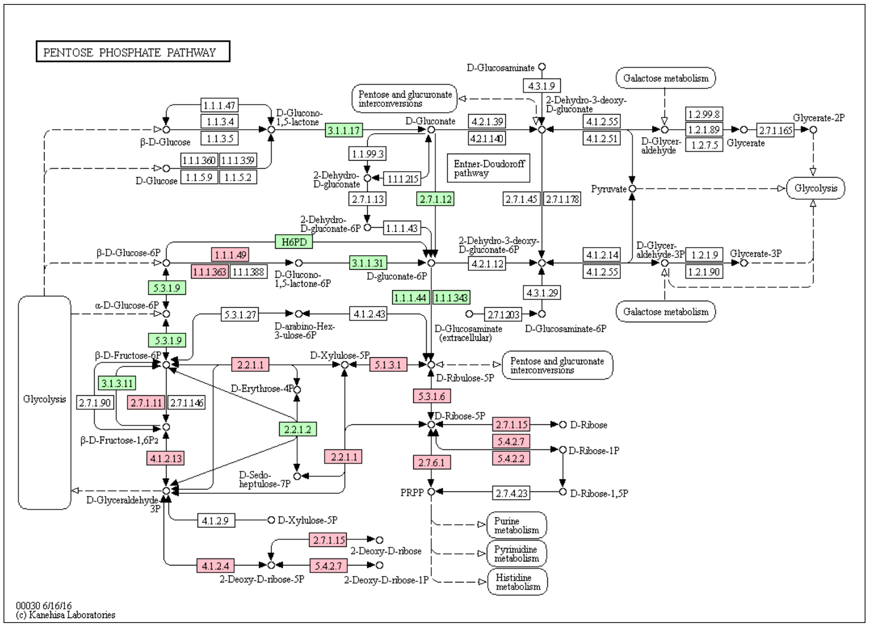

201.3 Legend:

RBH-Blast at 60% Identity + 50% Coverage
Green = Hit in *H. sapiens*
Red = Hit in *H. sapiens* and *T. californica*
White = Not in *H. sapiens*

202 Nicotinate and nicotinamide metabolism

202.1 Human Pathway: HSA00760

202.2 Number of Hits: 12

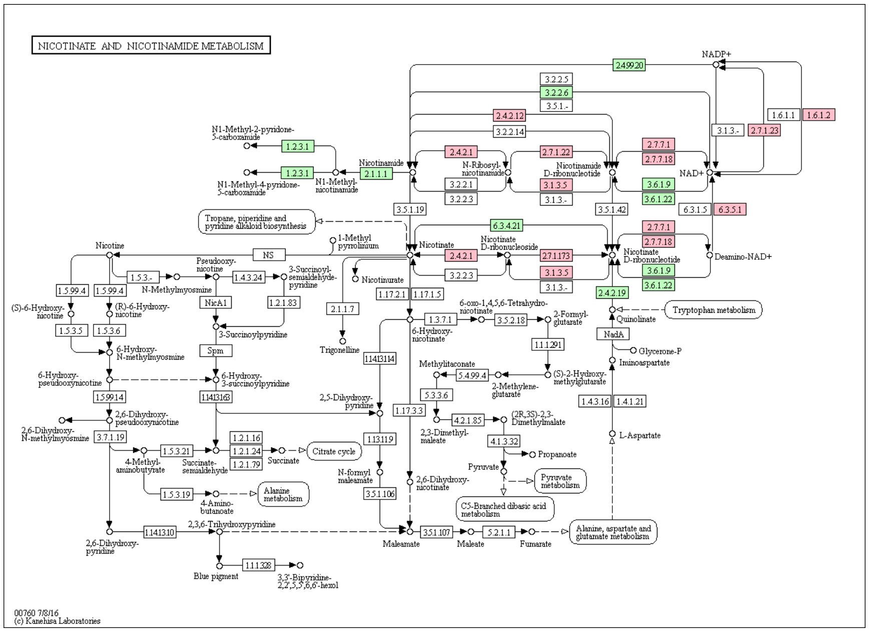

202.3 Legend:

RBH-Blast at 60% Identity + 50% Coverage
Green = Hit in *H. sapiens*
Red = Hit in *H. sapiens* and *T. californica*
White = Not in *H. sapiens*

203 Notch signaling pathway

203.1 Human Pathway: HSA04330

203.2 Number of Hits: 12

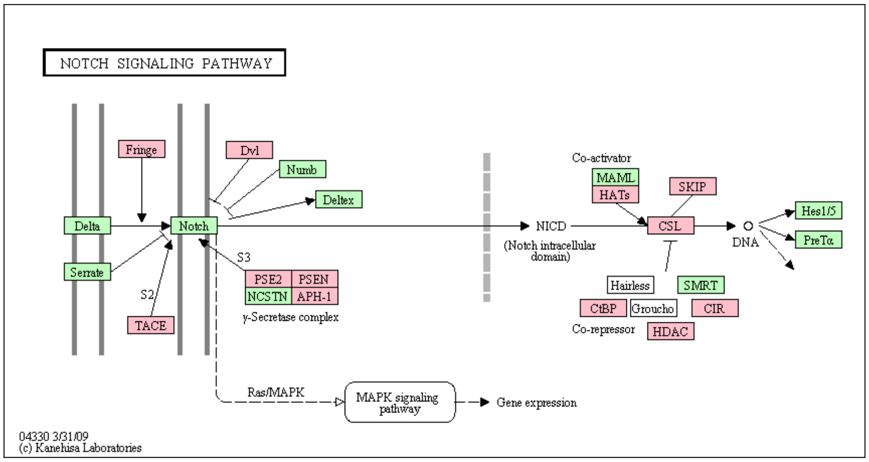

203.3 Legend:

RBH-Blast at 60% Identity + 50% Coverage
Green = Hit in *H. sapiens*
Red = Hit in *H. sapiens* and *T. californica*
White = Not in *H. sapiens*

204 Prion diseases

204.1 Human Pathway: HSA05020

204.2 Number of Hits: 12

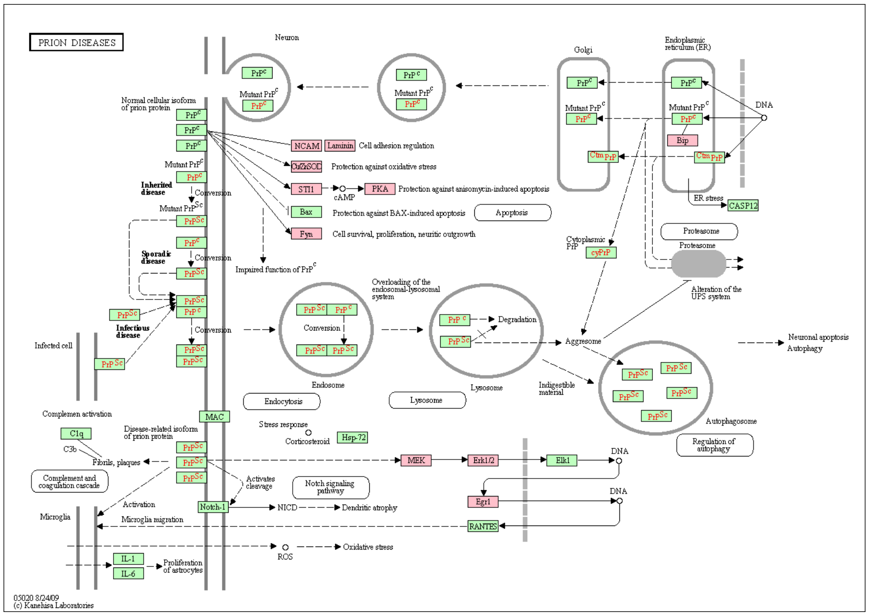

204.3 Legend:

RBH-Blast at 60% Identity + 50% Coverage
Green = Hit in *H. sapiens*
Red = Hit in *H. sapiens* and *T. californica*
White = Not in *H. sapiens*

205 Systemic lupus erythematosus

205.1 Human Pathway: HSA05322

205.2 Number of Hits: 12

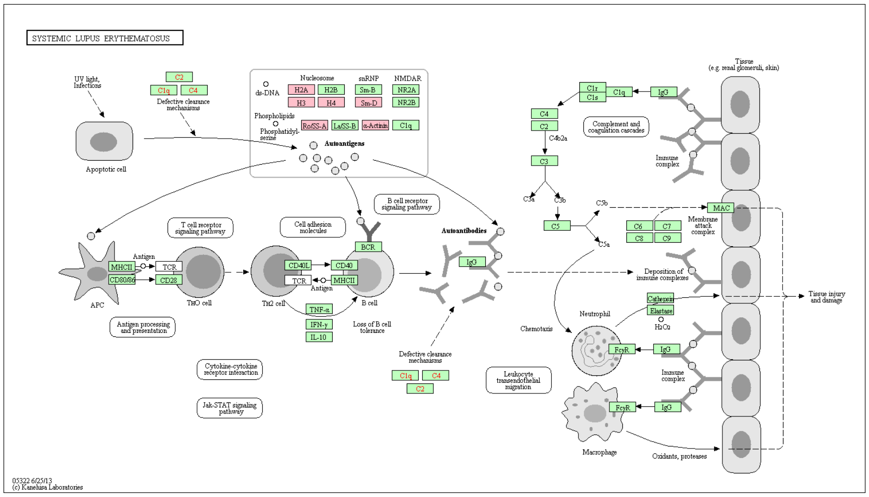

205.3 Legend:

RBH-Blast at 60% Identity + 50% Coverage
Green = Hit in *H. sapiens*
Red = Hit in *H. sapiens* and *T. californica*
White = Not in *H. sapiens*

206 Aldosterone-regulated sodium reabsorption

206.1 Human Pathway: HSA04960

206.2 Number of Hits: 12

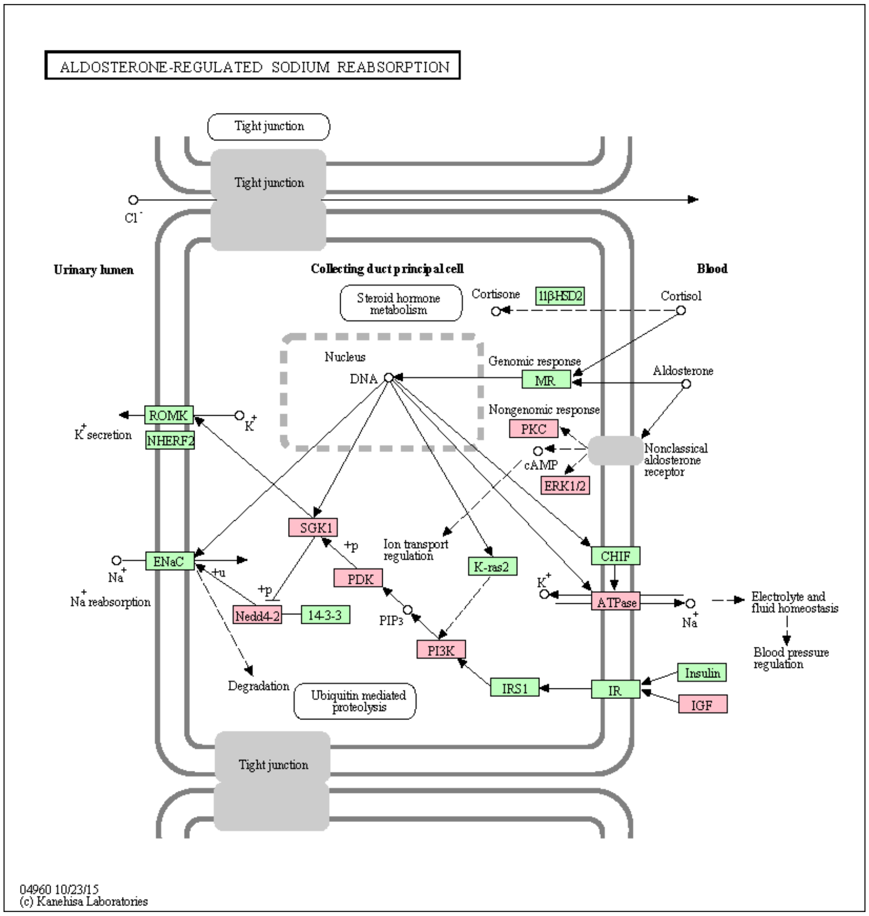

206.3 Legend:

RBH-Blast at 60% Identity + 50% Coverage
Green = Hit in *H. sapiens*
Red = Hit in *H. sapiens* and *T. californica*
White = Not in *H. sapiens*

207 Ether lipid metabolism

207.1 Human Pathway: HSA00565

207.2 Number of Hits: 12

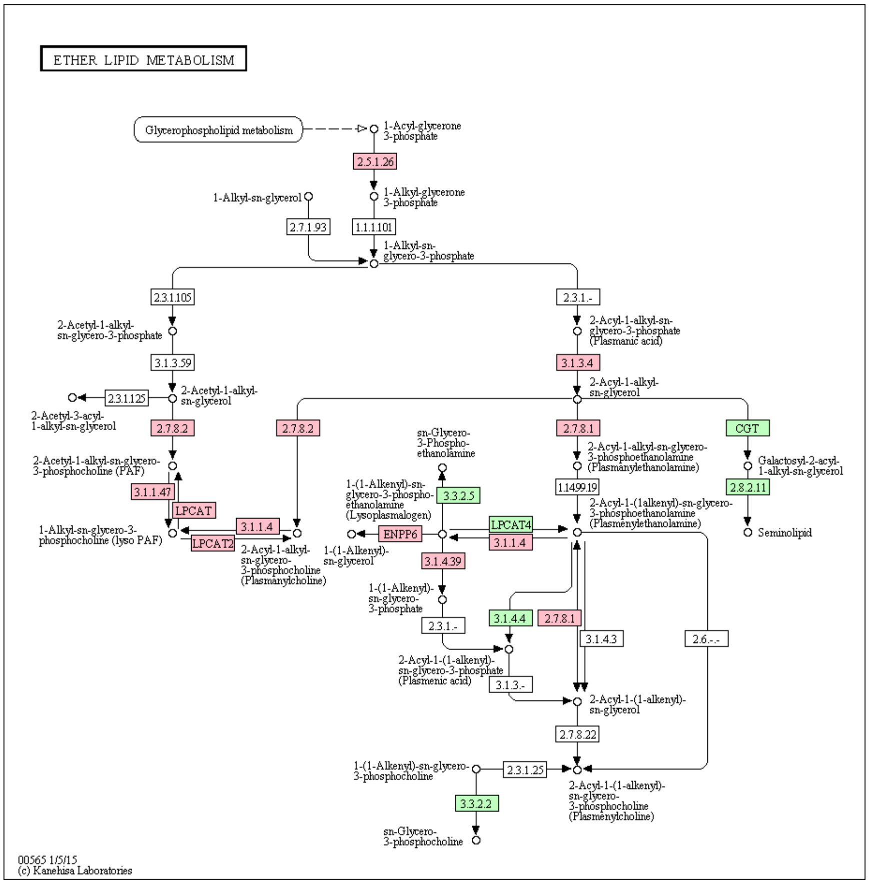

207.3 Legend:

RBH-Blast at 60% Identity + 50% Coverage
Green = Hit in *H. sapiens*
Red = Hit in *H. sapiens* and *T. californica*
White = Not in *H. sapiens*

208 One carbon pool by folate

208.1 Human Pathway: HSA00670

208.2 Number of Hits: 12

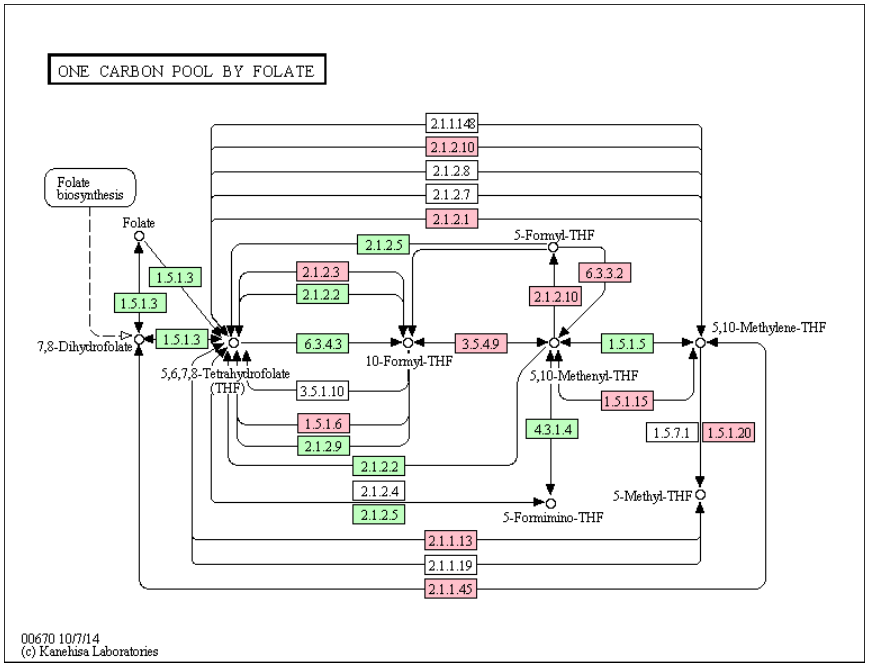

208.3 Legend:

RBH-Blast at 60% Identity + 50% Coverage
Green = Hit in *H. sapiens*
Red = Hit in *H. sapiens* and *T. californica*
White = Not in *H. sapiens*

209 Circadian rhythm

209.1 Human Pathway: HSA04710

209.2 Number of Hits: 12

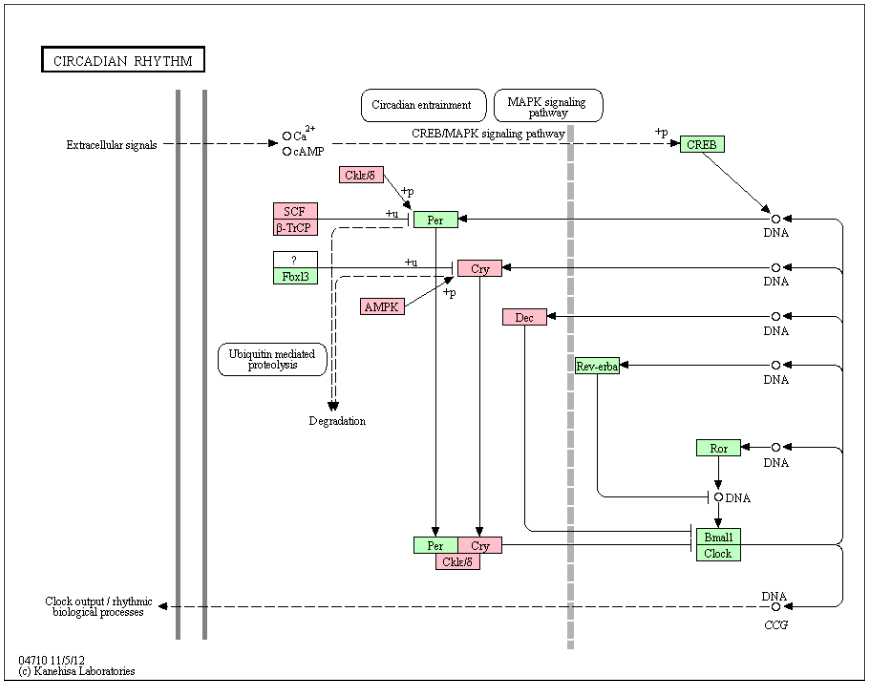

209.3 Legend:

RBH-Blast at 60% Identity + 50% Coverage
Green = Hit in *H. sapiens*
Red = Hit in *H. sapiens* and *T. californica*
White = Not in *H. sapiens*

210 Porphyrin and chlorophyll metabolism

210.1 Human Pathway: HSA00860

210.2 Number of Hits: 12

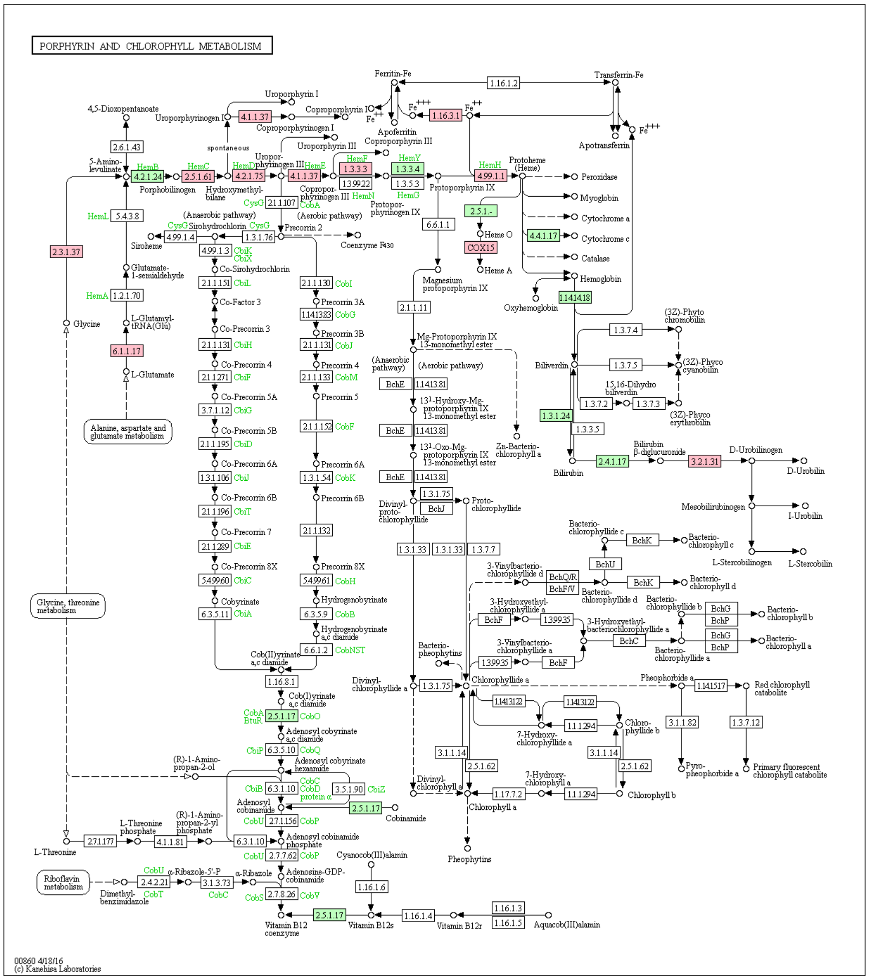

210.3 Legend:

RBH-Blast at 60% Identity + 50% Coverage
Green = Hit in *H. sapiens*
Red = Hit in *H. sapiens* and *T. californica*
White = Not in *H. sapiens*

211 Galactose metabolism

211.1 Human Pathway: HSA00052

211.2 Number of Hits: 12

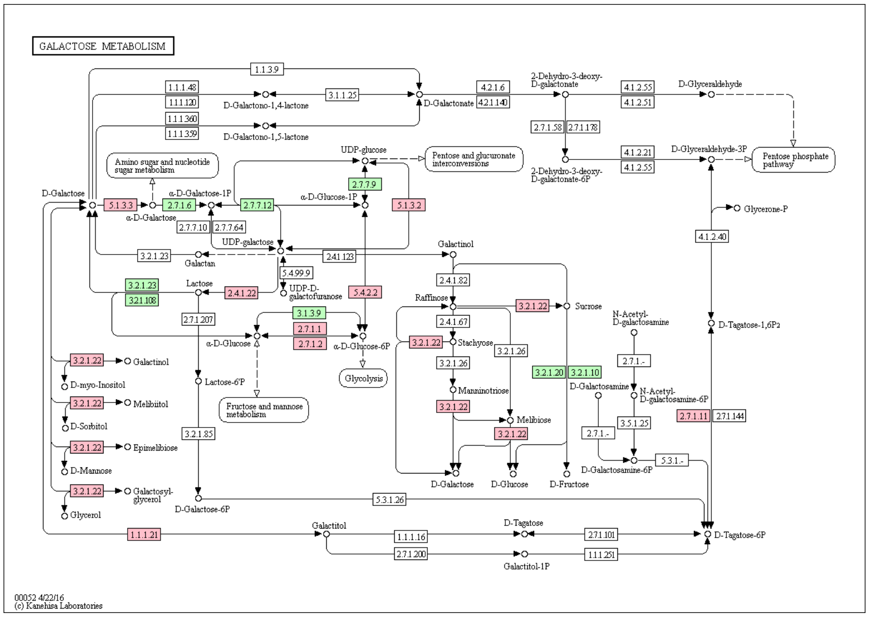

211.3 Legend:

RBH-Blast at 60% Identity + 50% Coverage
Green = Hit in *H. sapiens*
Red = Hit in *H. sapiens* and *T. californica*
White = Not in *H. sapiens*

212 Starch and sucrose metabolism

212.1 Human Pathway: HSA00500

212.2 Number of Hits: 12

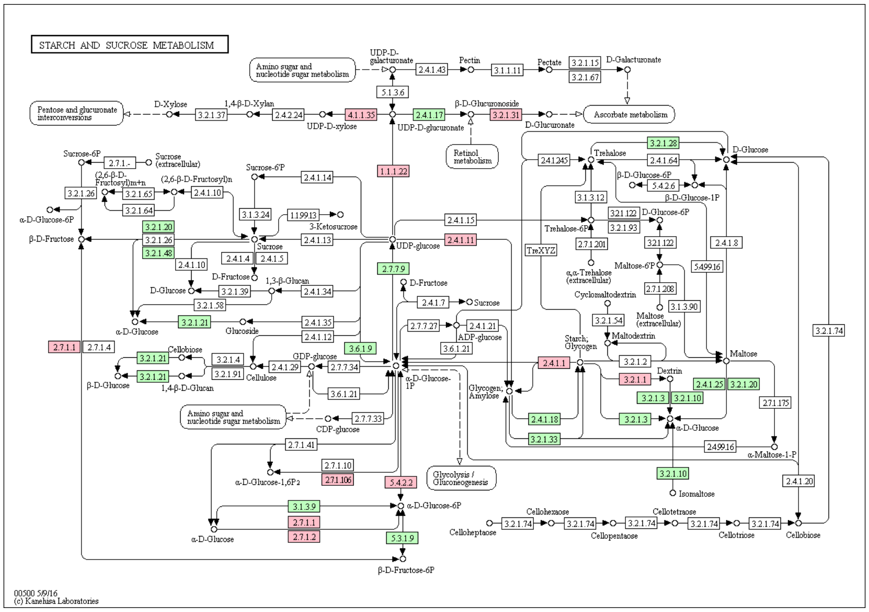

212.3 Legend:

RBH-Blast at 60% Identity + 50% Coverage
Green = Hit in *H. sapiens*
Red = Hit in *H. sapiens* and *T. californica*
White = Not in *H. sapiens*

213 Regulation of autophagy

213.1 Human Pathway: HSA04140

213.2 Number of Hits: 12

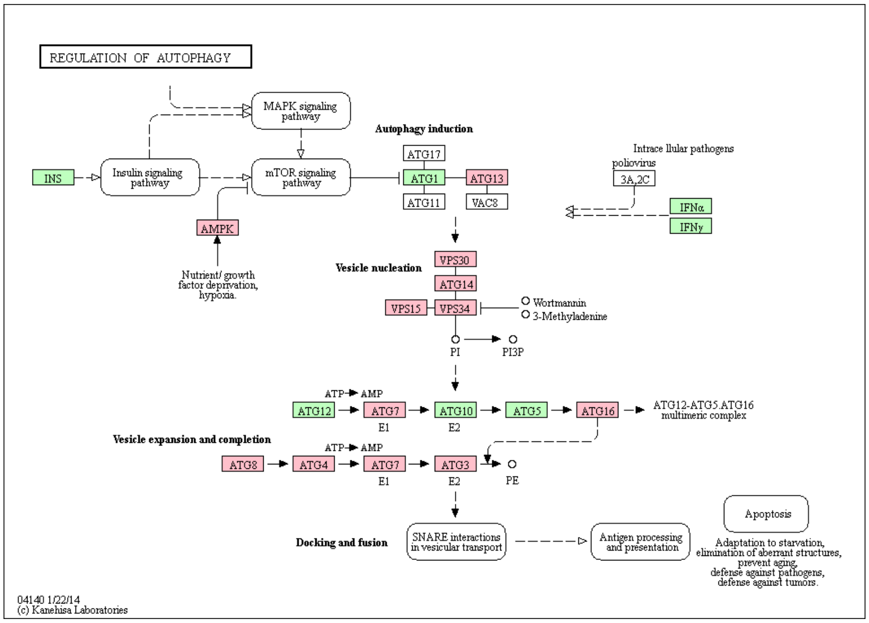

213.3 Legend:

RBH-Blast at 60% Identity + 50% Coverage
Green = Hit in *H. sapiens*
Red = Hit in *H. sapiens* and *T. californica*
White = Not in *H. sapiens*

214 Base excision repair

214.1 Human Pathway: HSA03410

214.2 Number of Hits: 11

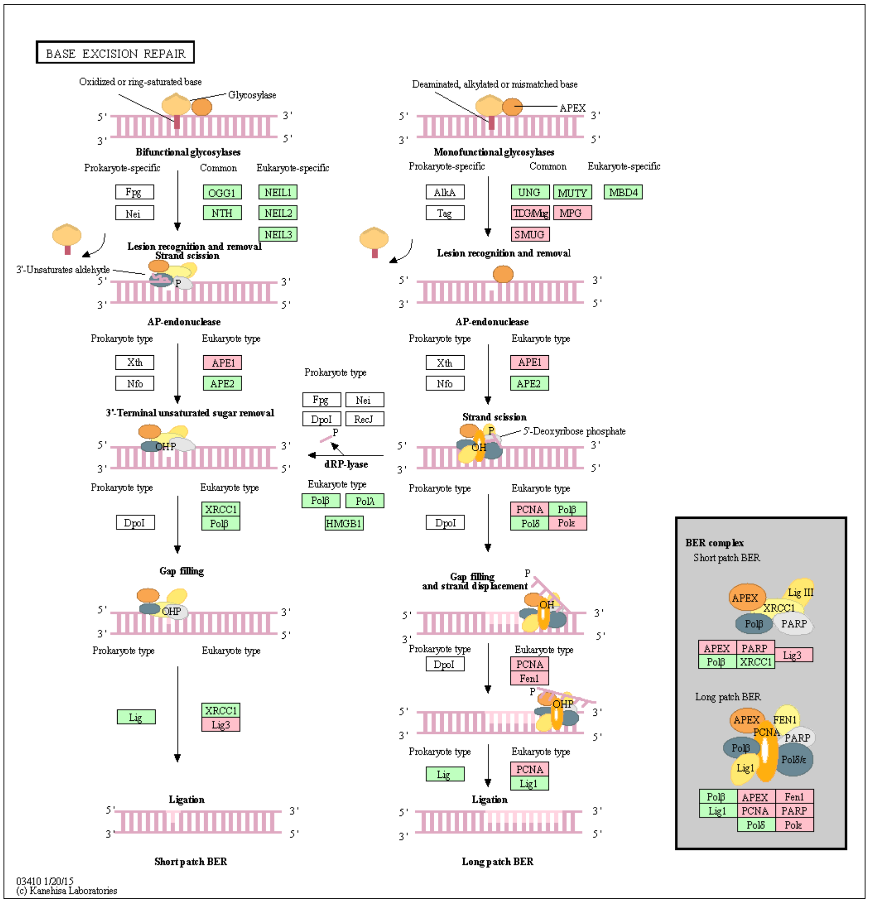

214.3 Legend:

RBH-Blast at 60% Identity + 50% Coverage
Green = Hit in *H. sapiens*
Red = Hit in *H. sapiens* and *T. californica*
White = Not in *H. sapiens*

215 Biosynthesis of unsaturated fatty acids

215.1 Human Pathway: HSA01040

215.2 Number of Hits: 11

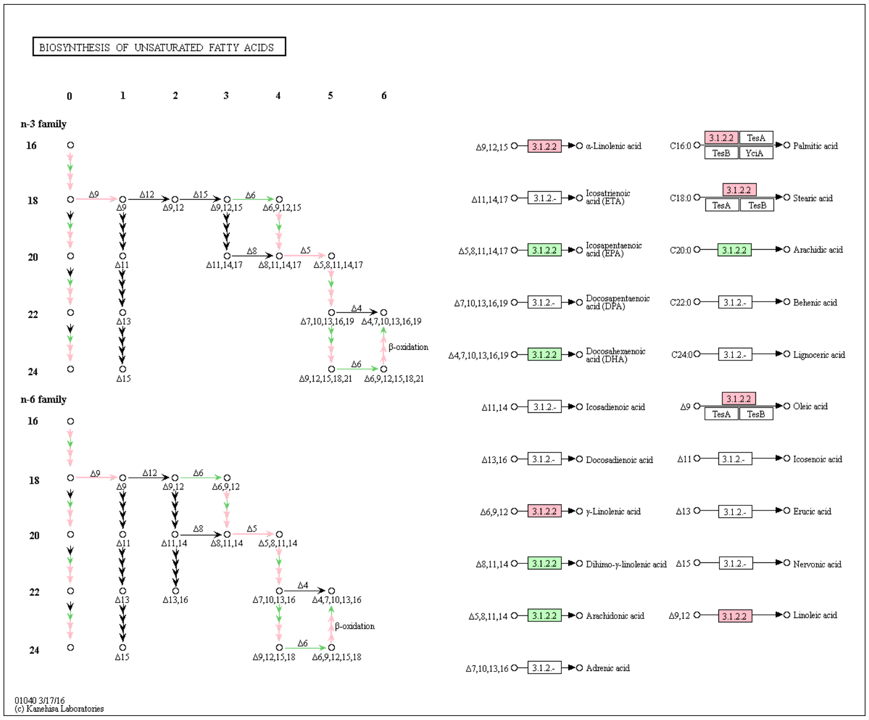

215.3 Legend:

RBH-Blast at 60% Identity + 50% Coverage
Green = Hit in *H. sapiens*
Red = Hit in *H. sapiens* and *T. californica*
White = Not in *H. sapiens*

216 2-Oxocarboxylic acid metabolism

216.1 Human Pathway: HSA01210

216.2 Number of Hits: 11

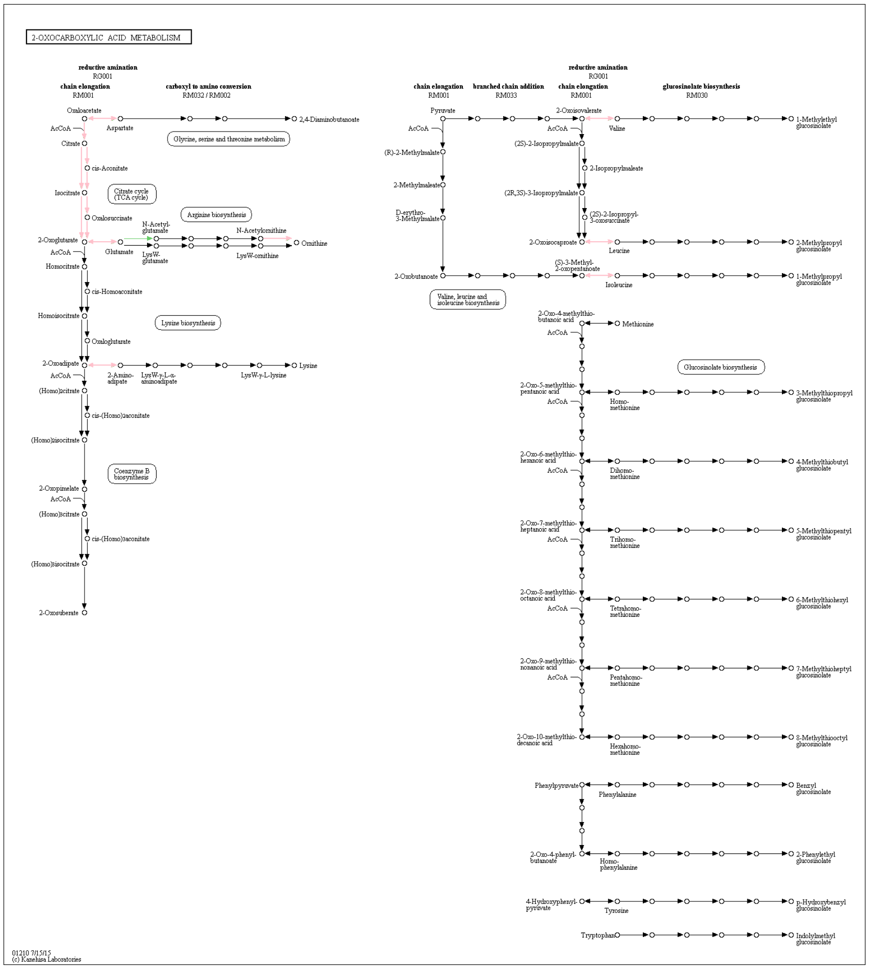

216.3 Legend:

RBH-Blast at 60% Identity + 50% Coverage
Green = Hit in *H. sapiens*
Red = Hit in *H. sapiens* and *T. californica*
White = Not in *H. sapiens*

217 Bladder cancer

217.1 Human Pathway: HSA05219

217.2 Number of Hits: 11

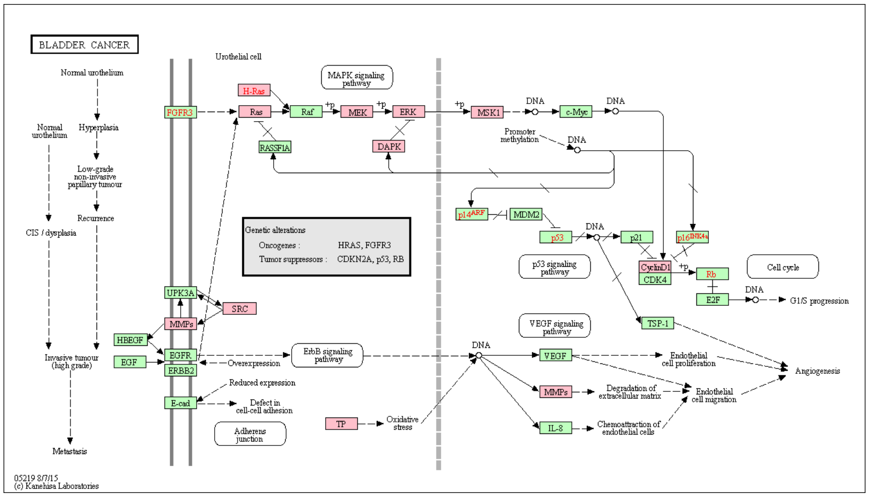

217.3 Legend:

RBH-Blast at 60% Identity + 50% Coverage
Green = Hit in *H. sapiens*
Red = Hit in *H. sapiens* and *T. californica*
White = Not in *H. sapiens*

218 Collecting duct acid secretion

218.1 Human Pathway: HSA04966

218.2 Number of Hits: 11

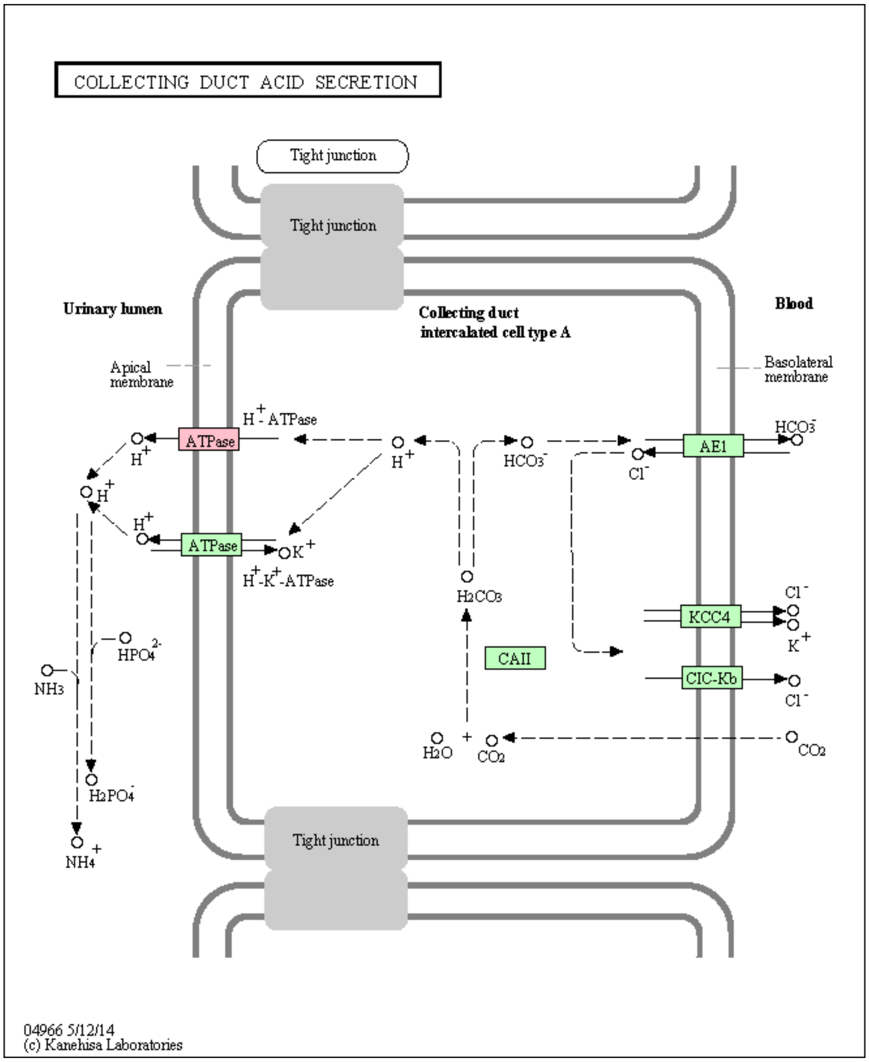

218.3 Legend:

RBH-Blast at 60% Identity + 50% Coverage
Green = Hit in *H. sapiens*
Red = Hit in *H. sapiens* and *T. californica*
White = Not in *H. sapiens*

---

219 Glutathione metabolism

219.1 Human Pathway: HSA00480

219.2 Number of Hits: 11

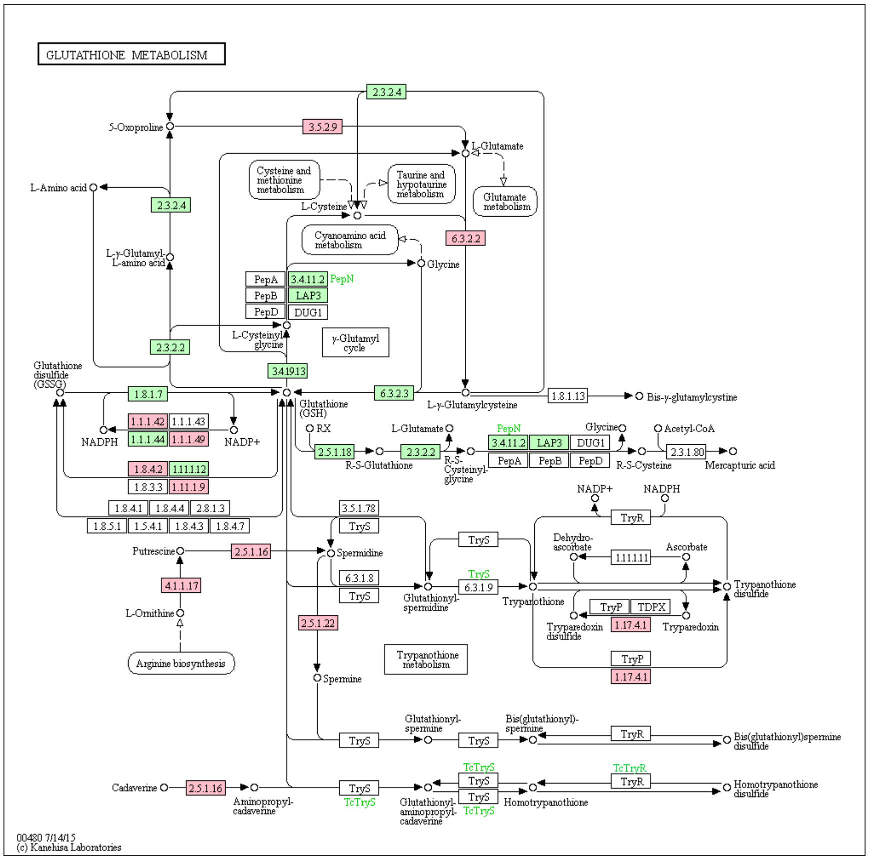

219.3 Legend:

RBH-Blast at 60% Identity + 50% Coverage
Green = Hit in *H. sapiens*
Red = Hit in *H. sapiens* and *T. californica*
White = Not in *H. sapiens*

220 Homologous recombination

220.1 Human Pathway: HSA03440

220.2 Number of Hits: 11

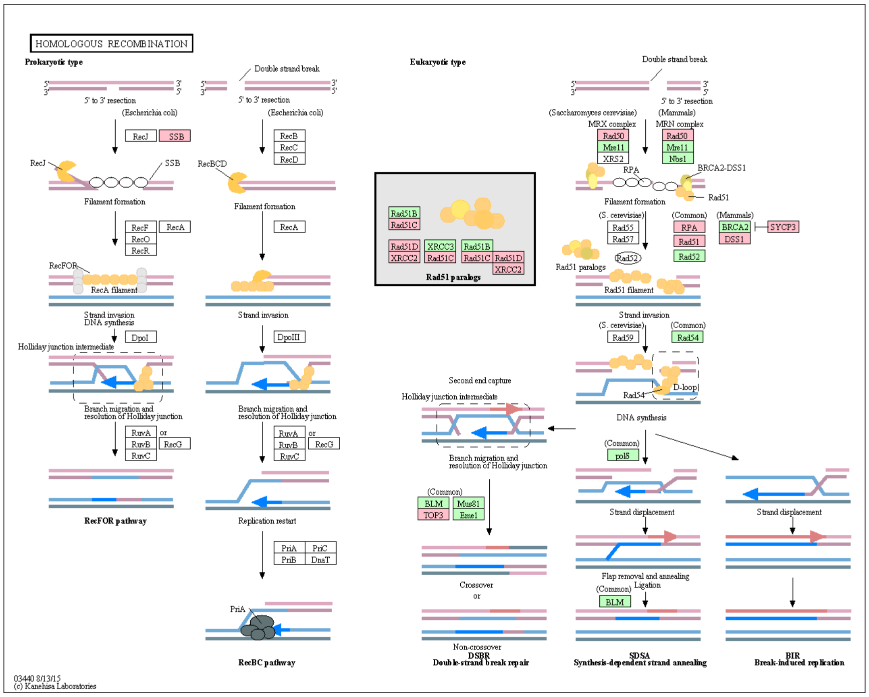

220.3 Legend:

RBH-Blast at 60% Identity + 50% Coverage
Green = Hit in *H. sapiens*
Red = Hit in *H. sapiens* and *T. californica*
White = Not in *H. sapiens*

221 Drug metabolism - other enzymes

221.1 Human Pathway: HSA00983

221.2 Number of Hits: 11

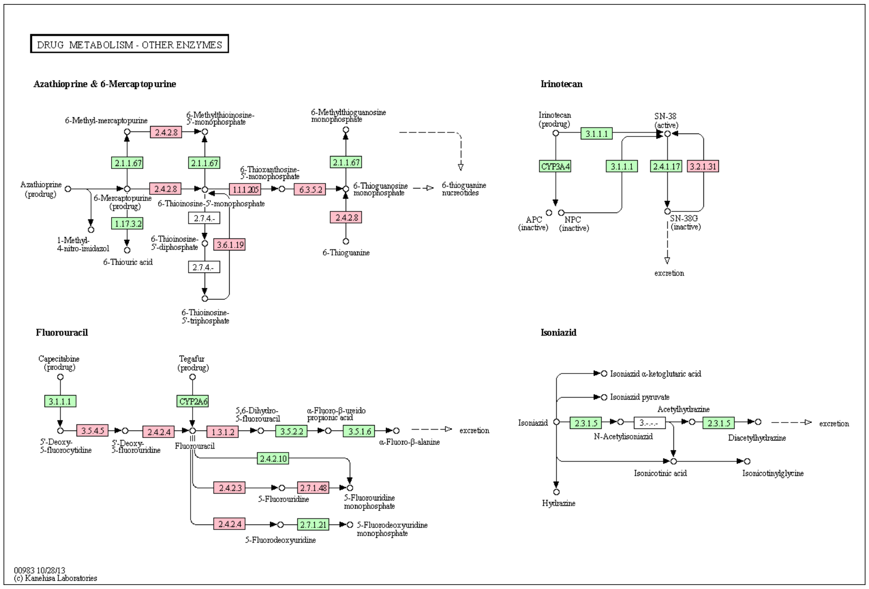

221.3 Legend:

RBH-Blast at 60% Identity + 50% Coverage
Green = Hit in *H. sapiens*
Red = Hit in *H. sapiens* and *T. californica*
White = Not in *H. sapiens*

222 Taste transduction

222.1 Human Pathway: HSA04742

222.2 Number of Hits: 10

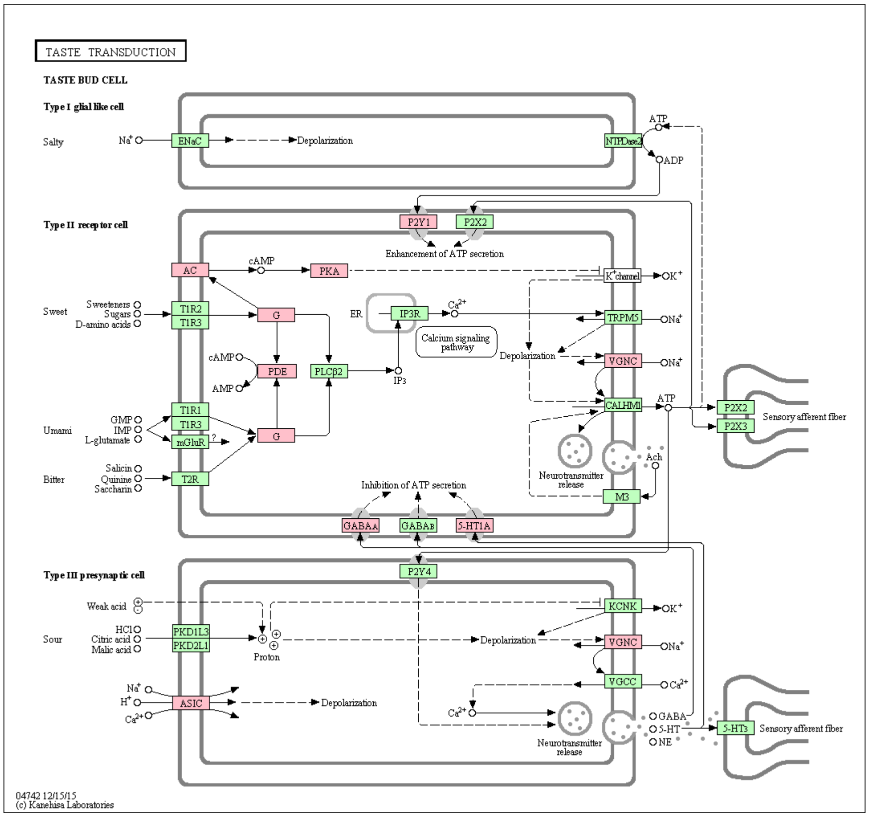

222.3 Legend:

RBH-Blast at 60% Identity + 50% Coverage
Green = Hit in *H. sapiens*
Red = Hit in *H. sapiens* and *T. californica*
White = Not in *H. sapiens*

223 Antigen processing and presentation

223.1 Human Pathway: HSA04612

223.2 Number of Hits: 10

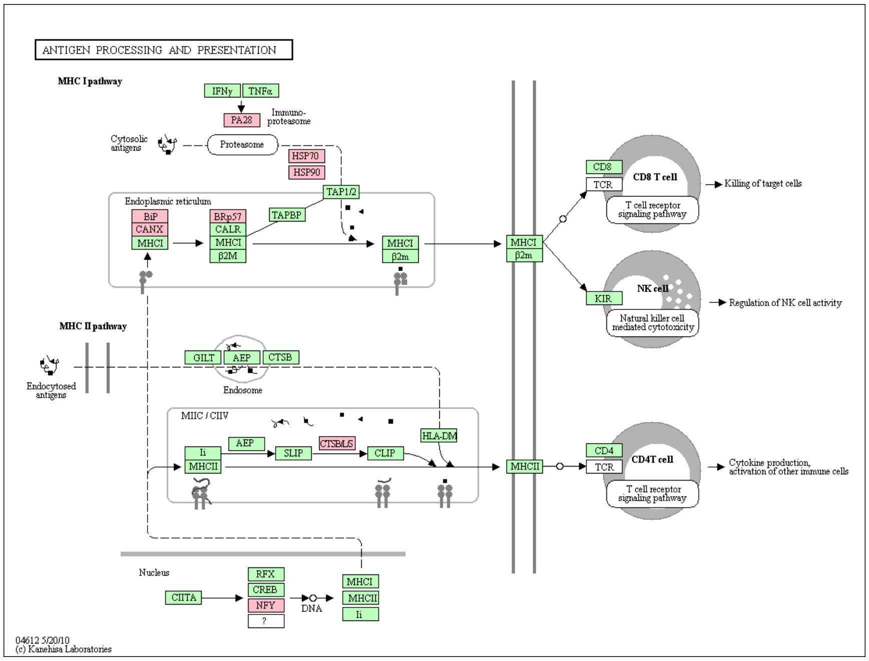

223.3 Legend:

RBH-Blast at 60% Identity + 50% Coverage
Green = Hit in *H. sapiens*
Red = Hit in *H. sapiens* and *T. californica*
White = Not in *H. sapiens*

224 Fat digestion and absorption

224.1 Human Pathway: HSA04975

224.2 Number of Hits: 10

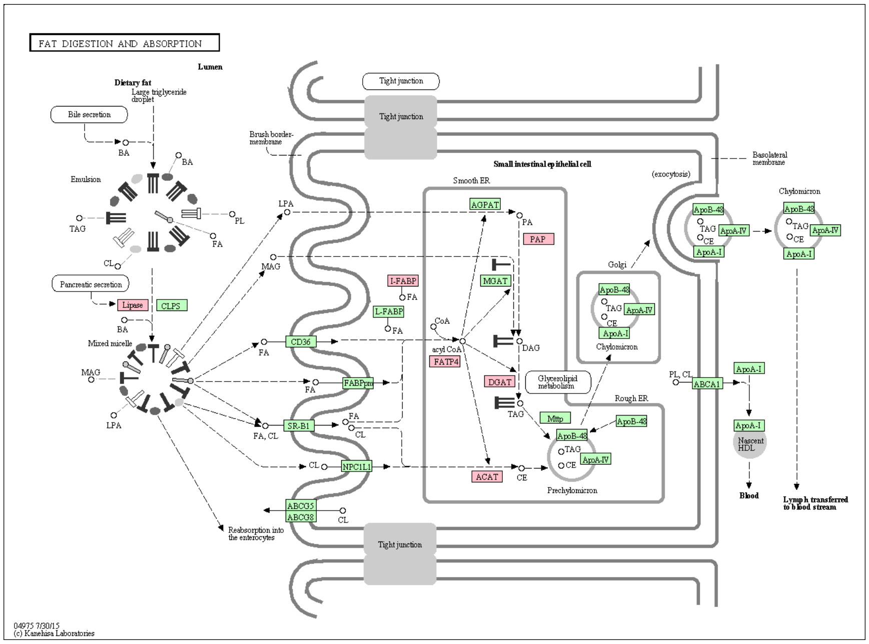

224.3 Legend:

RBH-Blast at 60% Identity + 50% Coverage
Green = Hit in *H. sapiens*
Red = Hit in *H. sapiens* and *T. californica*
White = Not in *H. sapiens*

225 Carbohydrate digestion and absorption

225.1 Human Pathway: HSA04973

225.2 Number of Hits: 10

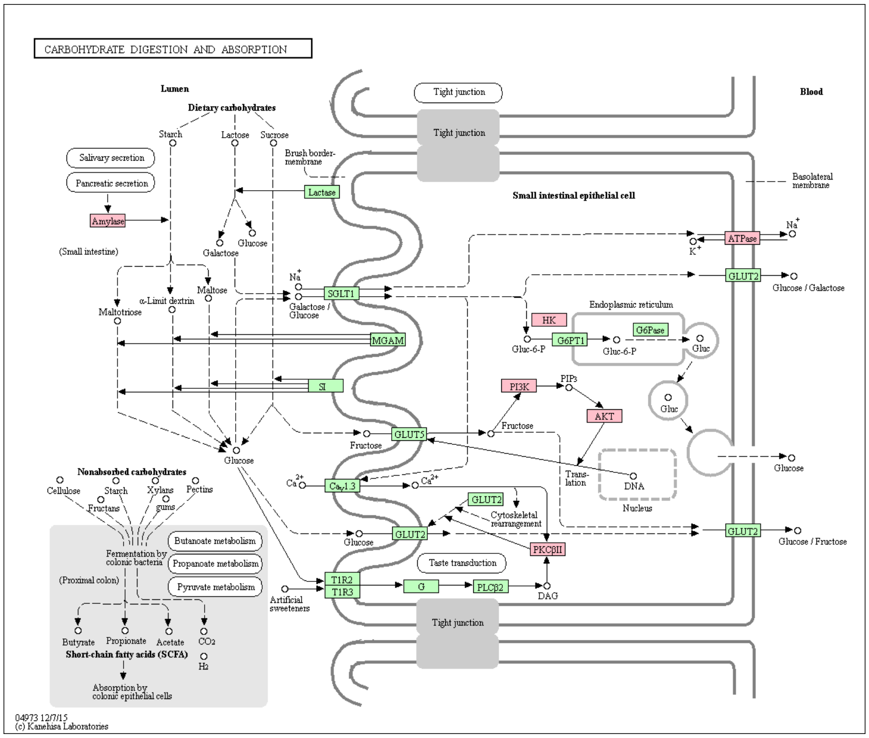

225.3 Legend:

RBH-Blast at 60% Identity + 50% Coverage
Green = Hit in *H. sapiens*
Red = Hit in *H. sapiens* and *T. californica*
White = Not in *H. sapiens*

226 Arginine biosynthesis

226.1 Human Pathway: HSA00220

226.2 Number of Hits: 10

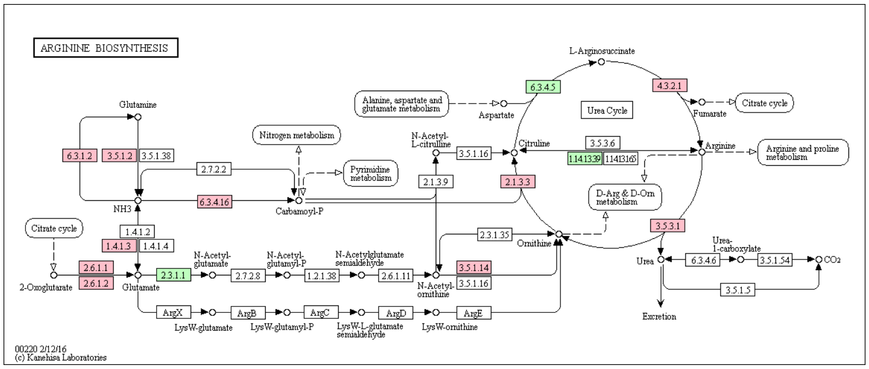

226.3 Legend:

RBH-Blast at 60% Identity + 50% Coverage
Green = Hit in *H. sapiens*
Red = Hit in *H. sapiens* and *T. californica*
White = Not in *H. sapiens*

227 Bile secretion

227.1 Human Pathway: HSA04976

227.2 Number of Hits: 10

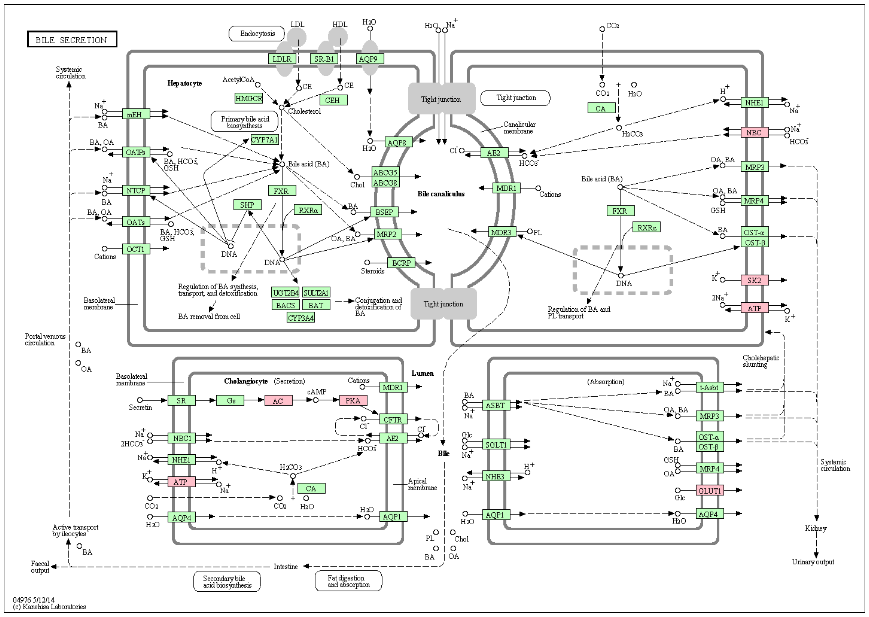

227.3 Legend:

RBH-Blast at 60% Identity + 50% Coverage
Green = Hit in *H. sapiens*
Red = Hit in *H. sapiens* and *T. californica*
White = Not in *H. sapiens*

228 Thyroid cancer

228.1 Human Pathway: HSA05216

228.2 Number of Hits: 10

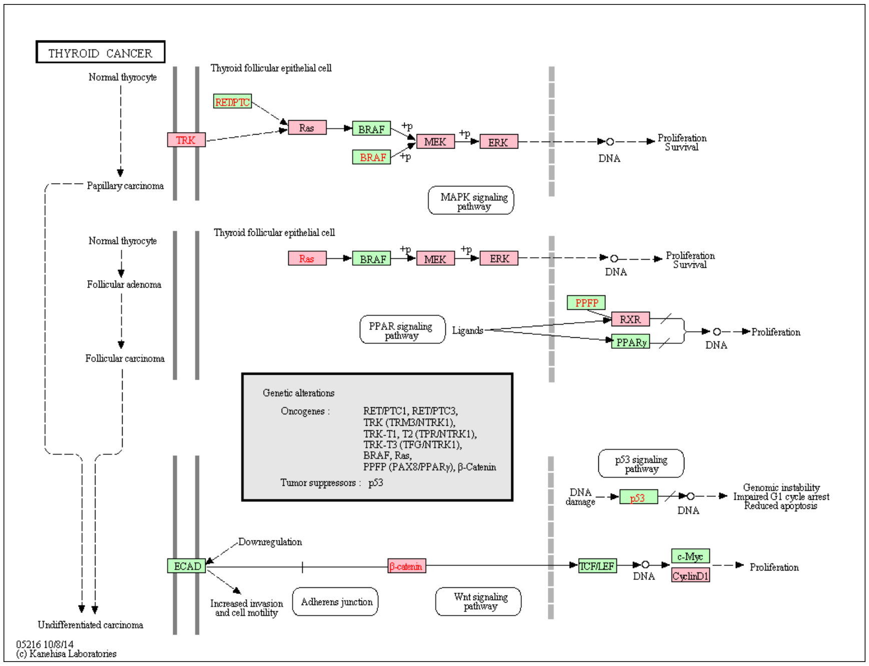

228.3 Legend:

RBH-Blast at 60% Identity + 50% Coverage
Green = Hit in *H. sapiens*
Red = Hit in *H. sapiens* and *T. californica*
White = Not in *H. sapiens*

229 Arachidonic acid metabolism

229.1 Human Pathway: HSA00590

229.2 Number of Hits: 10

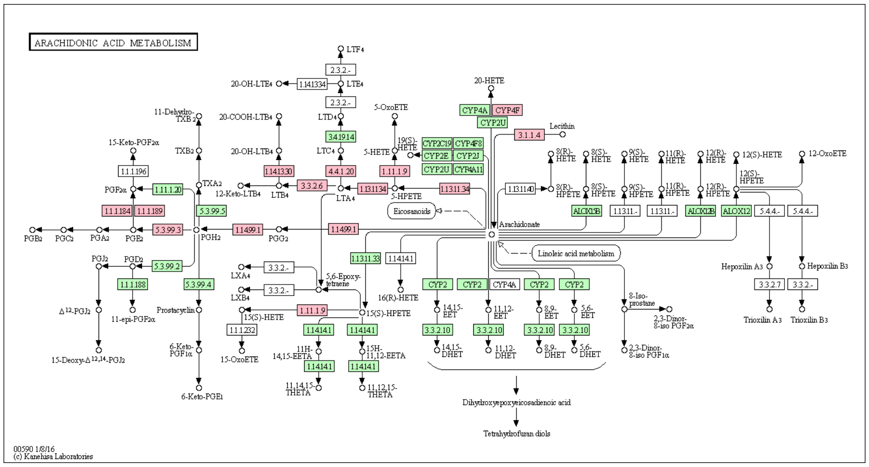

229.3 Legend:

RBH-Blast at 60% Identity + 50% Coverage
Green = Hit in *H. sapiens*
Red = Hit in *H. sapiens* and *T. californica*
White = Not in *H. sapiens*

230 Pentose and glucuronate interconversions

230.1 Human Pathway: HSA00040

230.2 Number of Hits: 10

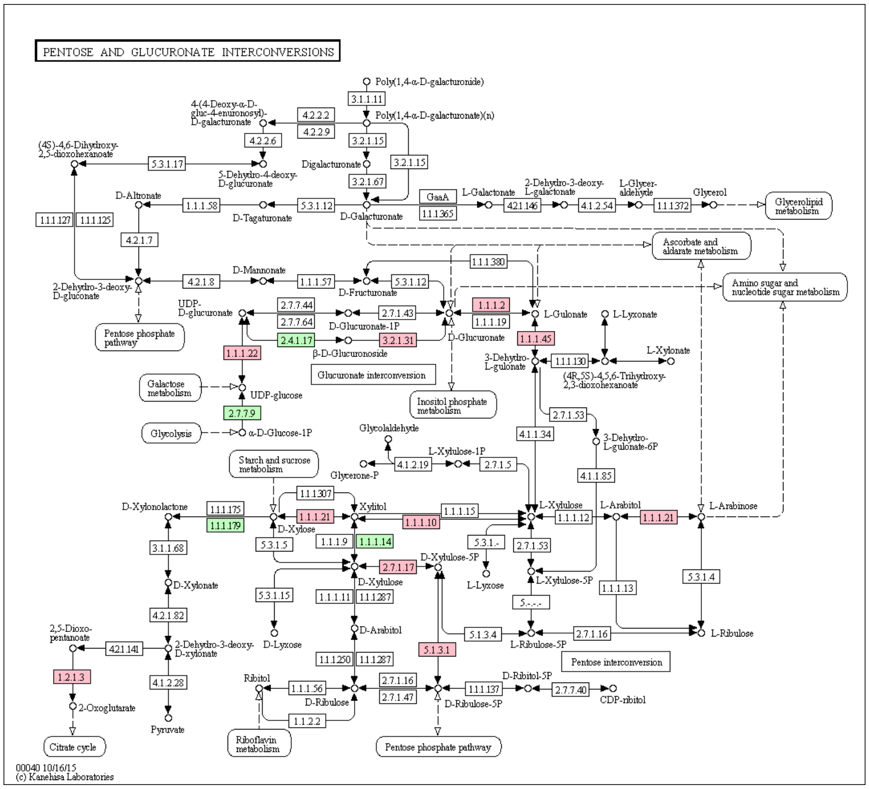

230.3 Legend:

RBH-Blast at 60% Identity + 50% Coverage
Green = Hit in *H. sapiens*
Red = Hit in *H. sapiens* and *T. californica*
White = Not in *H. sapiens*

231 Mucin type O-Glycan biosynthesis

231.1 Human Pathway: HSA00512

231.2 Number of Hits: 9

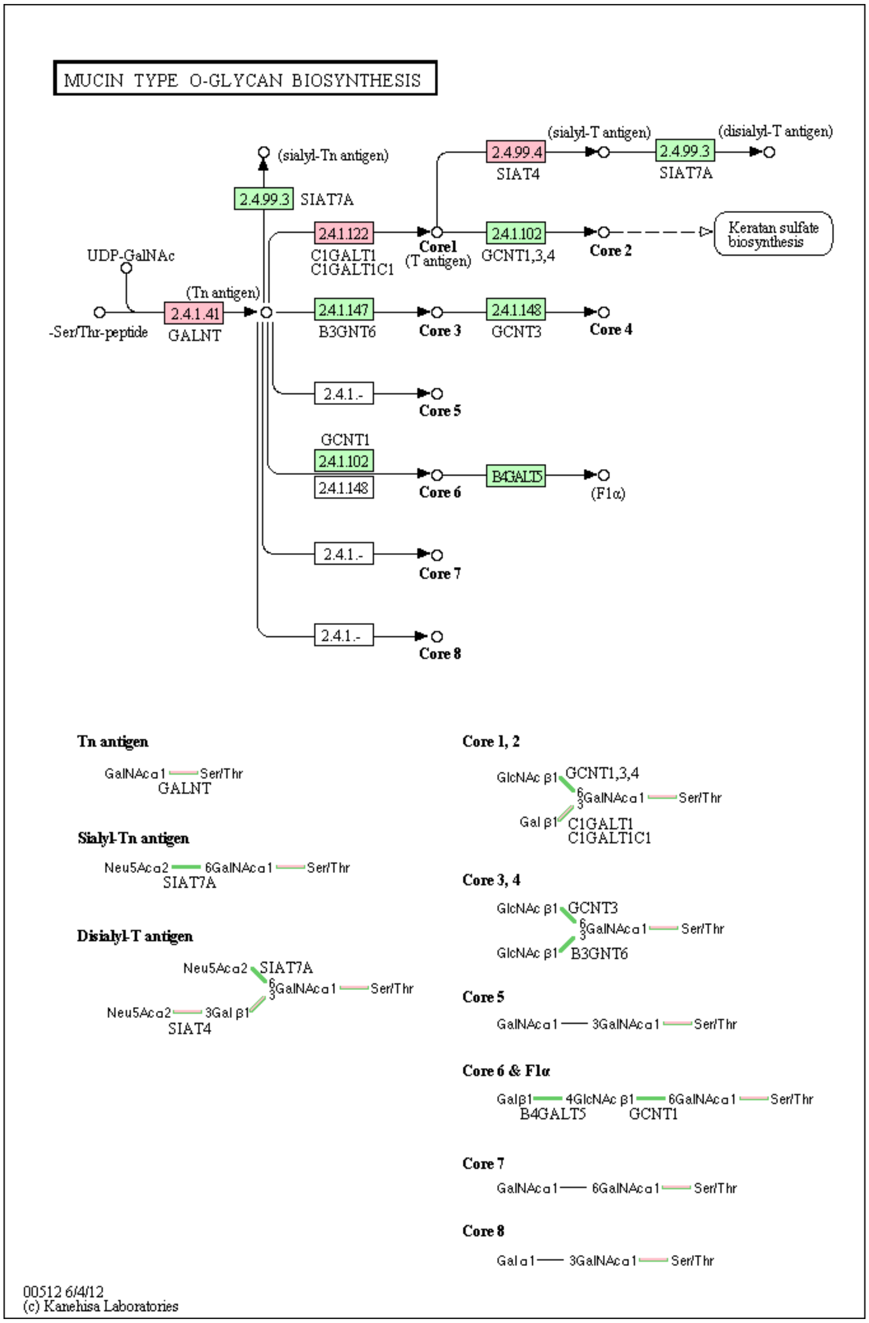

231.3 Legend:

RBH-Blast at 60% Identity + 50% Coverage
Green = Hit in *H. sapiens*
Red = Hit in *H. sapiens* and *T. californica*
White = Not in *H. sapiens*

---

232 Basal cell carcinoma

232.1 Human Pathway: HSA05217

232.2 Number of Hits: 9

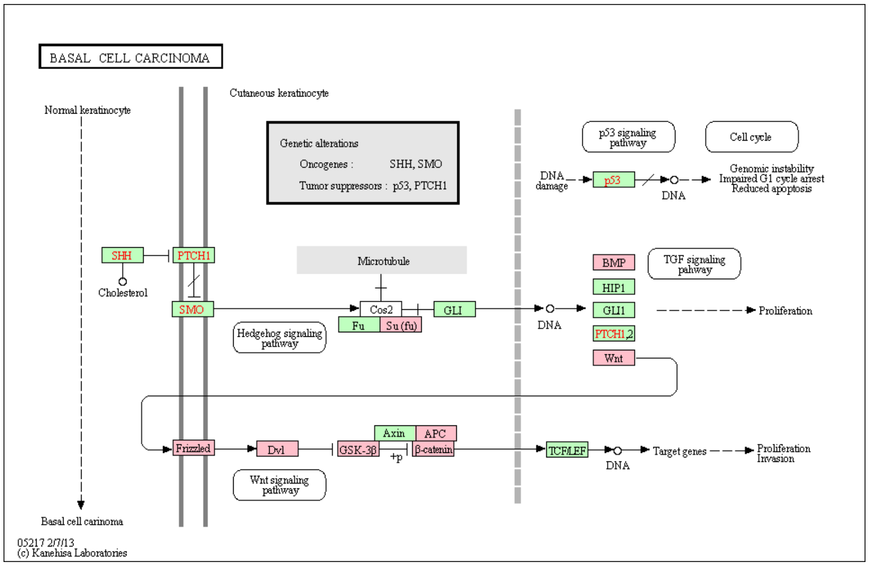

232.3 Legend:

RBH-Blast at 60% Identity + 50% Coverage
Green = Hit in *H. sapiens*
Red = Hit in *H. sapiens* and *T. californica*
White = Not in *H. sapiens*

---

233 Mineral absorption

233.1 Human Pathway: HSA04978

233.2 Number of Hits: 9

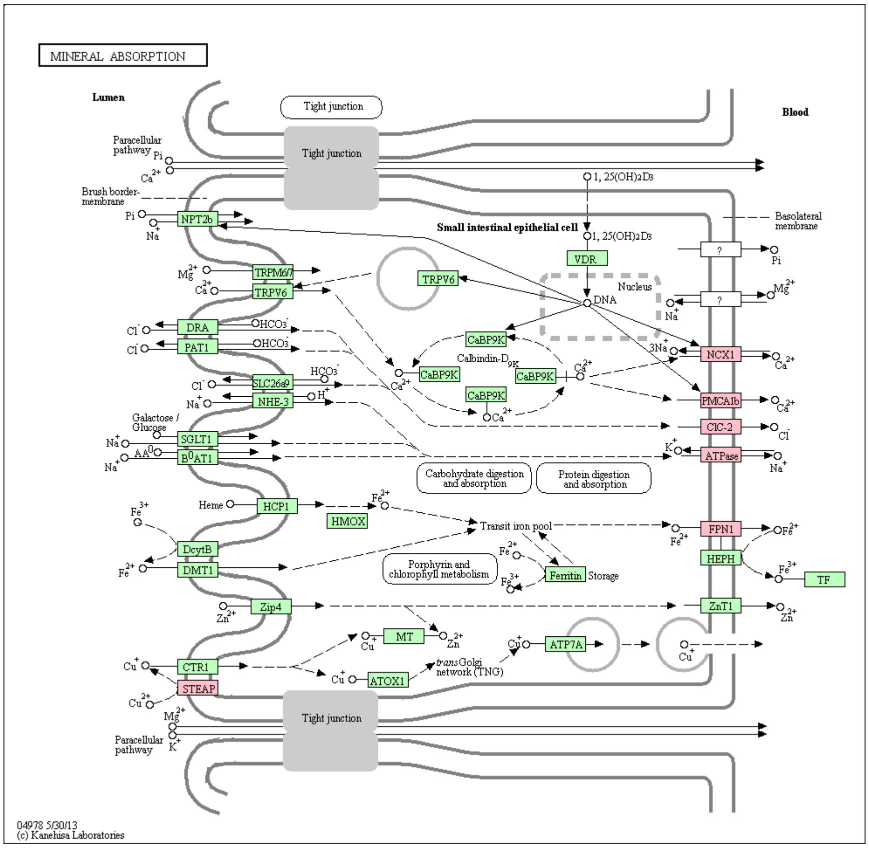

233.3 Legend:

RBH-Blast at 60% Identity + 50% Coverage
Green = Hit in *H. sapiens*
Red = Hit in *H. sapiens* and *T. californica*
White = Not in *H. sapiens*

234 Proximal tubule bicarbonate reclamation

234.1 Human Pathway: HSA04964

234.2 Number of Hits: 9

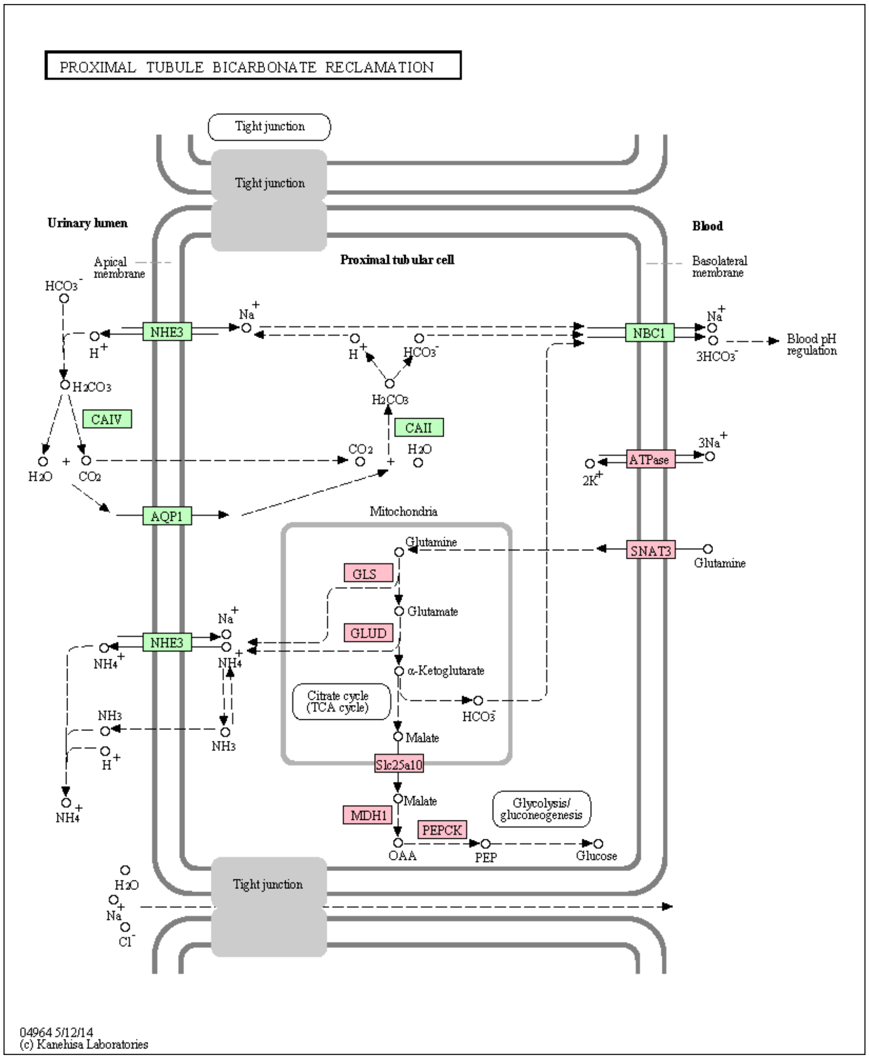

234.3 Legend:

RBH-Blast at 60% Identity + 50% Coverage
Green = Hit in *H. sapiens*
Red = Hit in *H. sapiens* and *T. californica*
White = Not in *H. sapiens*

---

235 Mismatch repair

235.1 Human Pathway: HSA03430

235.2 Number of Hits: 8

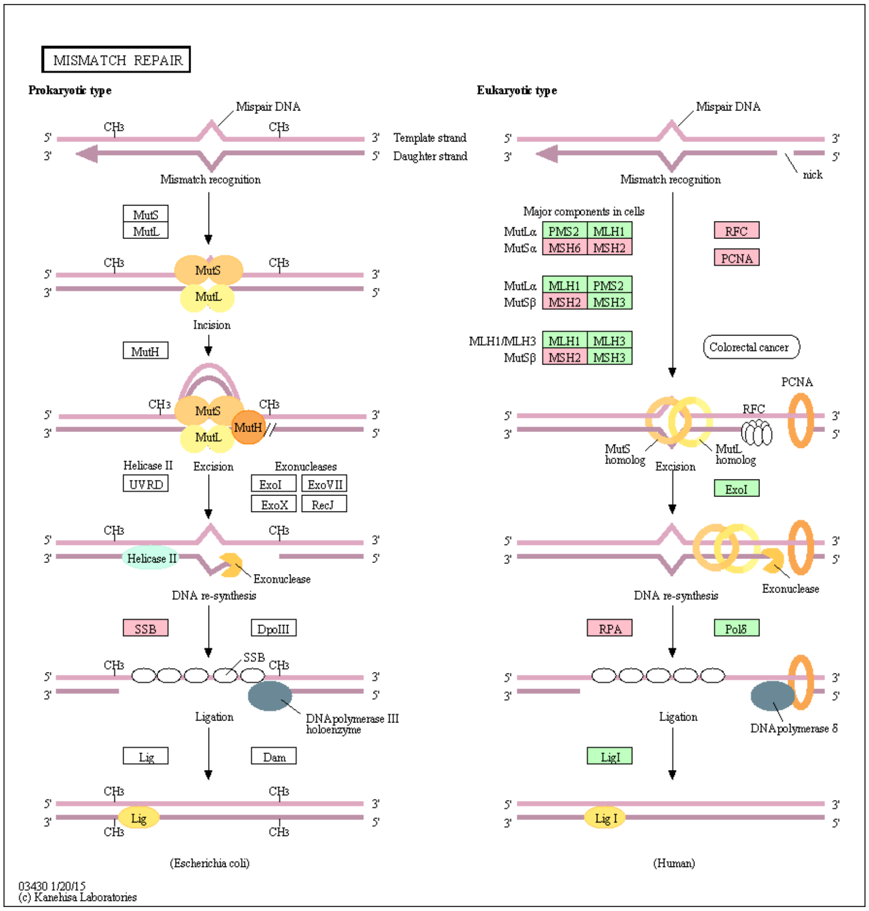

235.3 Legend:

RBH-Blast at 60% Identity + 50% Coverage
Green = Hit in *H. sapiens*
Red = Hit in *H. sapiens* and *T. californica*
White = Not in *H. sapiens*

---

236 Hippo signaling pathway -multiple species

236.1 Human Pathway: HSA04392

236.2 Number of Hits: 8

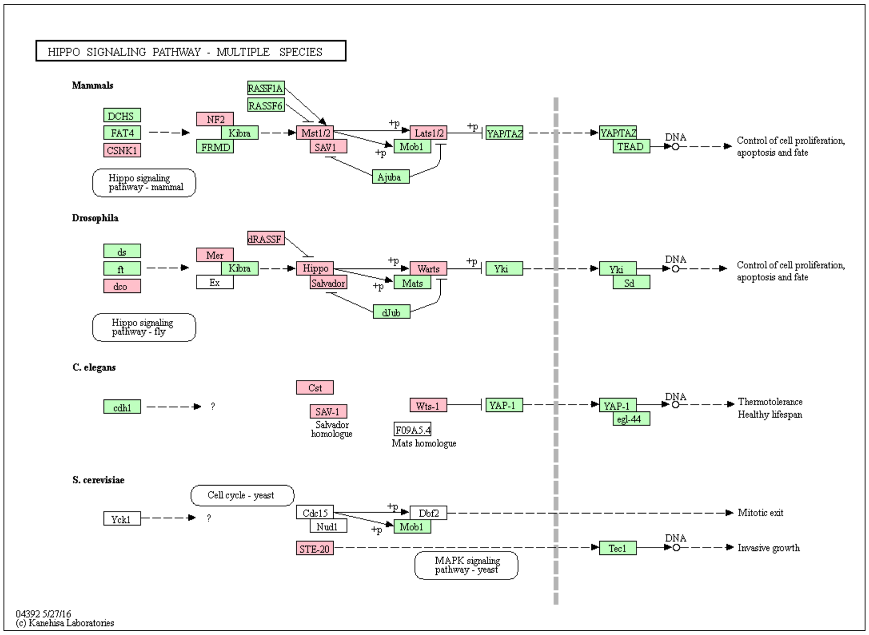

236.3 Legend:

RBH-Blast at 60% Identity + 50% Coverage
Green = Hit in *H. sapiens*
Red = Hit in *H. sapiens* and *T. californica*
White = Not in *H. sapiens*

---

237 Fanconi anemia pathway

237.1 Human Pathway: HSA03460

237.2 Number of Hits: 8

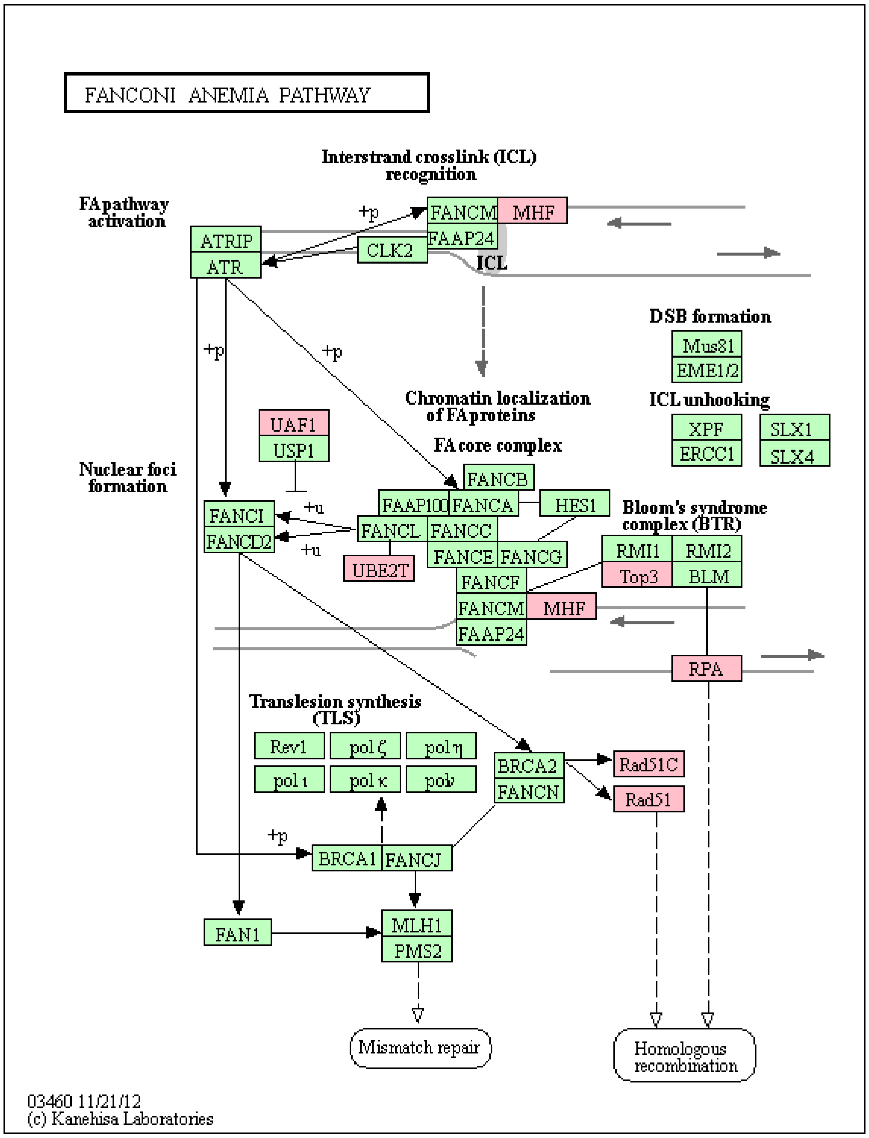

237.3 Legend:

RBH-Blast at 60% Identity + 50% Coverage
Green = Hit in *H. sapiens*
Red = Hit in *H. sapiens* and *T. californica*
White = Not in *H. sapiens*

---

238 Apoptosis - multiple species

238.1 Human Pathway: HSA04215

238.2 Number of Hits: 8

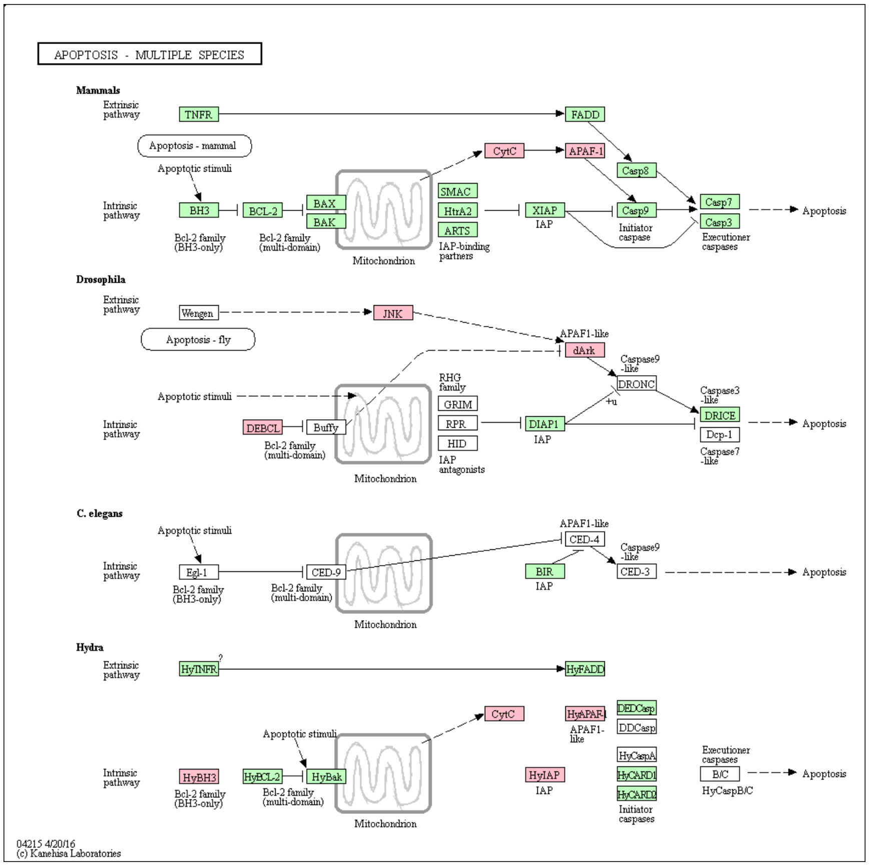

238.3 Legend:

RBH-Blast at 60% Identity + 50% Coverage
Green = Hit in *H. sapiens*
Red = Hit in *H. sapiens* and *T. californica*
White = Not in *H. sapiens*

---

239 Dorso-ventral axis formation

239.1 Human Pathway: HSA04320

239.2 Number of Hits: 8

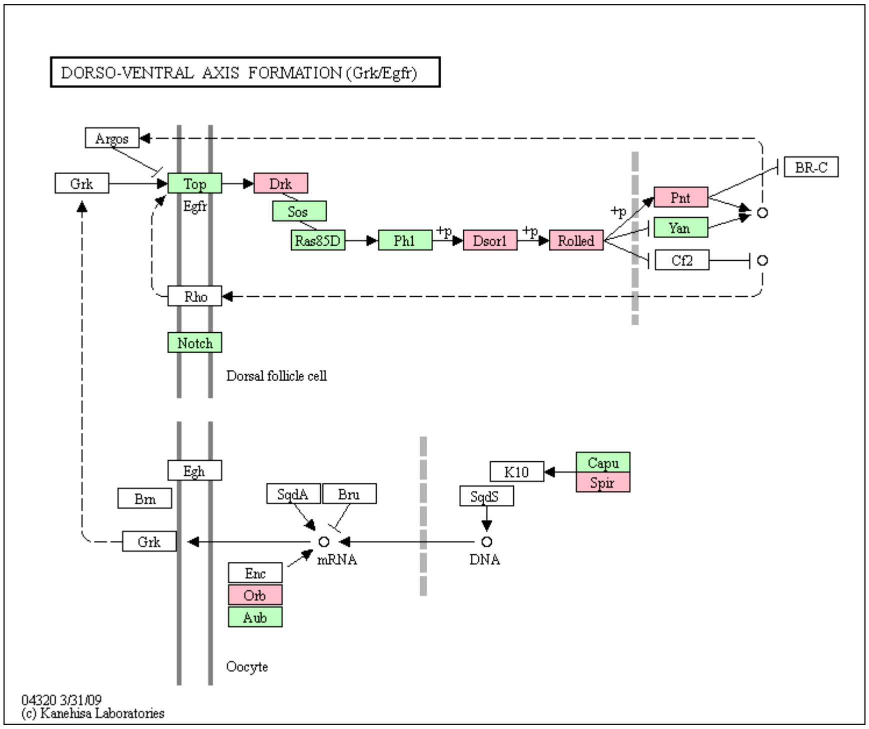

239.3 Legend:

RBH-Blast at 60% Identity + 50% Coverage
Green = Hit in *H. sapiens*
Red = Hit in *H. sapiens* and *T. californica*
White = Not in *H. sapiens*

240 Selenocompound metabolism

240.1 Human Pathway: HSA00450

240.2 Number of Hits: 8

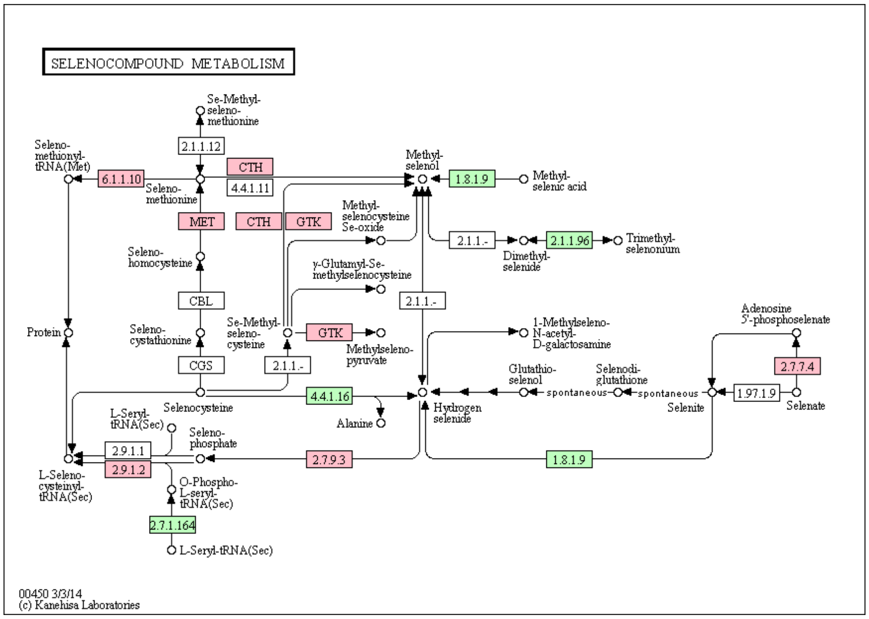

240.3 Legend:

RBH-Blast at 60% Identity + 50% Coverage
Green = Hit in *H. sapiens*
Red = Hit in *H. sapiens* and *T. californica*
White = Not in *H. sapiens*

241 Histidine metabolism

241.1 Human Pathway: HSA00340

241.2 Number of Hits: 7

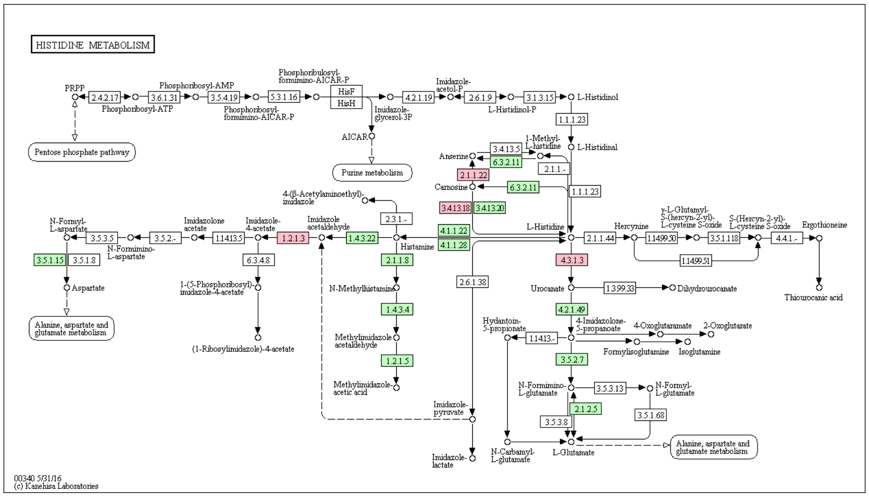

241.3 Legend:

RBH-Blast at 60% Identity + 50% Coverage
Green = Hit in *H. sapiens*
Red = Hit in *H. sapiens* and *T. californica*
White = Not in *H. sapiens*

242 Retinol metabolism

242.1 Human Pathway: HSA00830

242.2 Number of Hits: 7

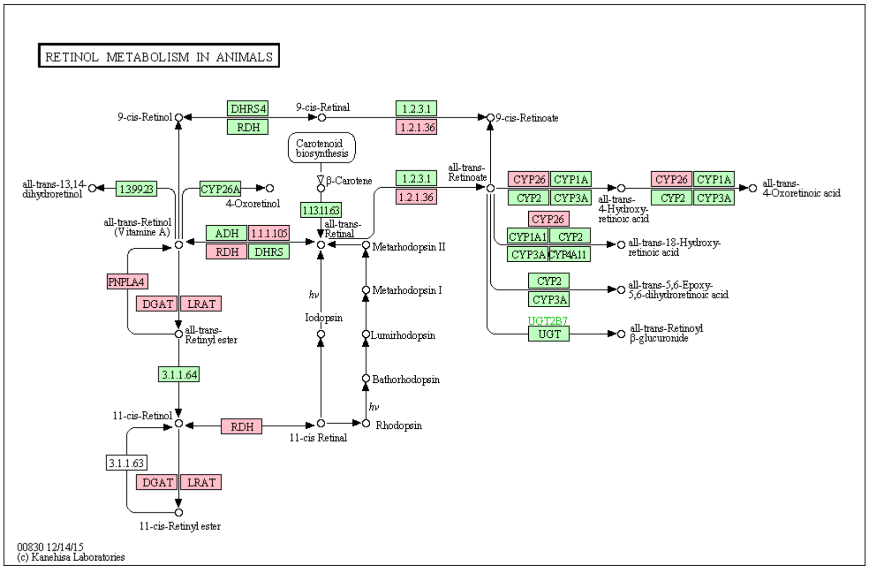

242.3 Legend:

RBH-Blast at 60% Identity + 50% Coverage
Green = Hit in *H. sapiens*
Red = Hit in *H. sapiens* and *T. californica*
White = Not in *H. sapiens*

243 Ovarian steroidogenesis

243.1 Human Pathway: HSA04913

243.2 Number of Hits: 7

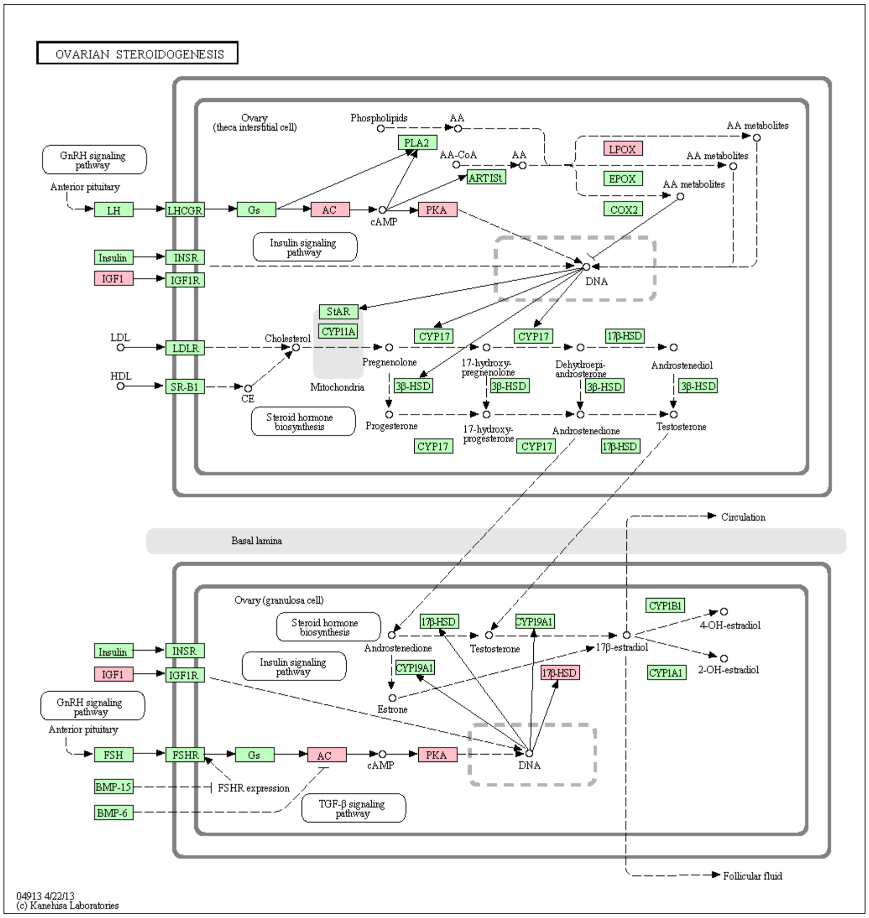

243.3 Legend:

RBH-Blast at 60% Identity + 50% Coverage
Green = Hit in *H. sapiens*
Red = Hit in *H. sapiens* and *T. californica*
White = Not in *H. sapiens*

244 Folate biosynthesis

244.1 Human Pathway: HSA00790

244.2 Number of Hits: 7

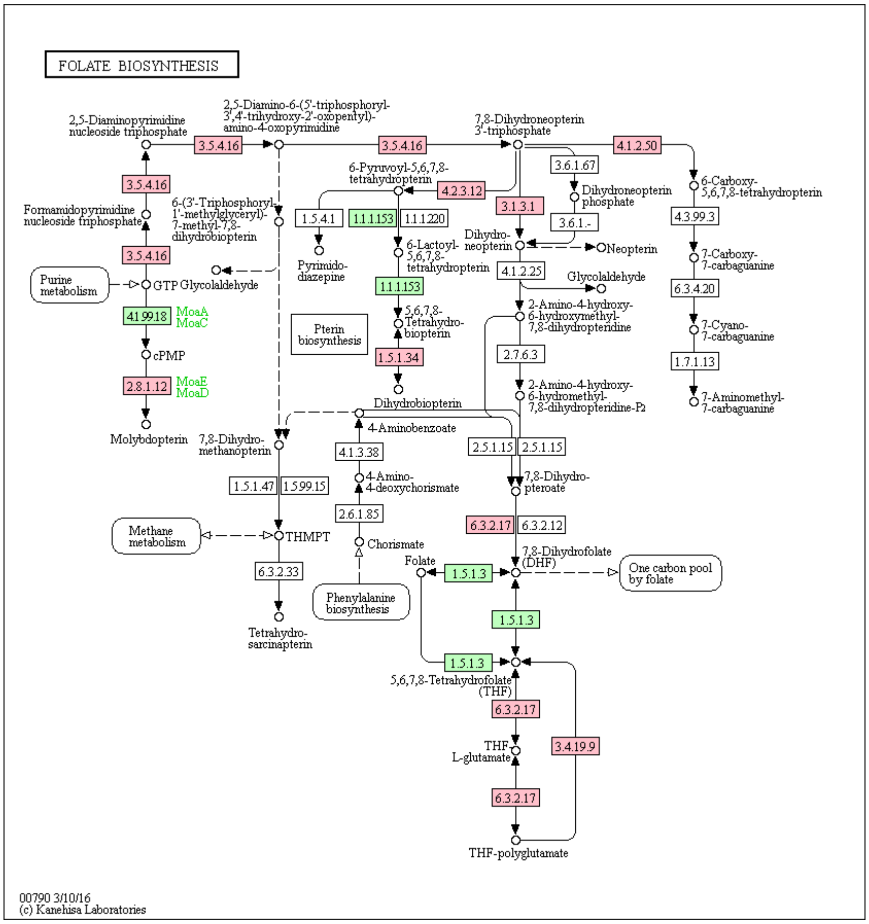

244.3 Legend:

RBH-Blast at 60% Identity + 50% Coverage
Green = Hit in *H. sapiens*
Red = Hit in *H. sapiens* and *T. californica*
White = Not in *H. sapiens*

245 Pantothenate and CoA biosynthesis

245.1 Human Pathway: HSA00770

245.2 Number of Hits: 7

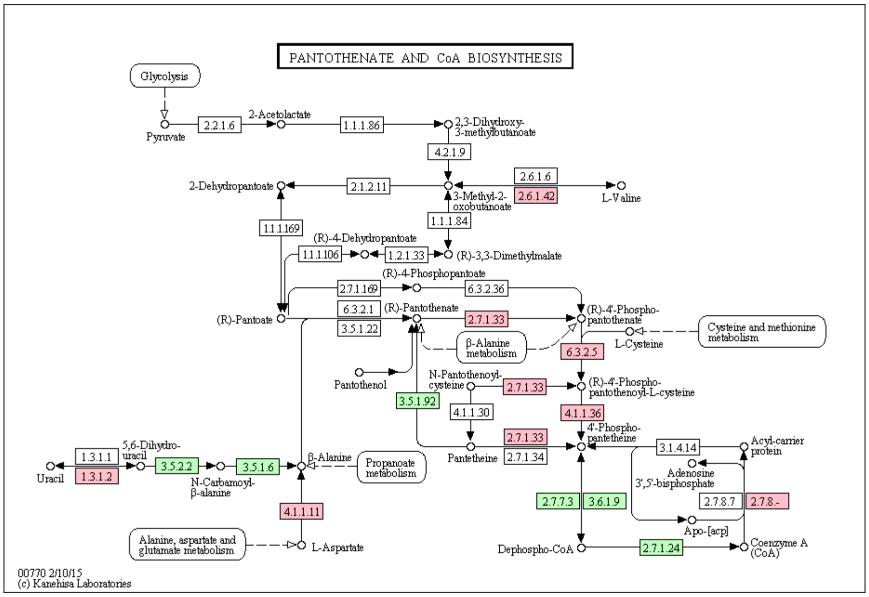

245.3 Legend:

RBH-Blast at 60% Identity + 50% Coverage
Green = Hit in *H. sapiens*
Red = Hit in *H. sapiens* and *T. californica*
White = Not in *H. sapiens*

246 Malaria

246.1 Human Pathway: HSA05144

246.2 Number of Hits: 6

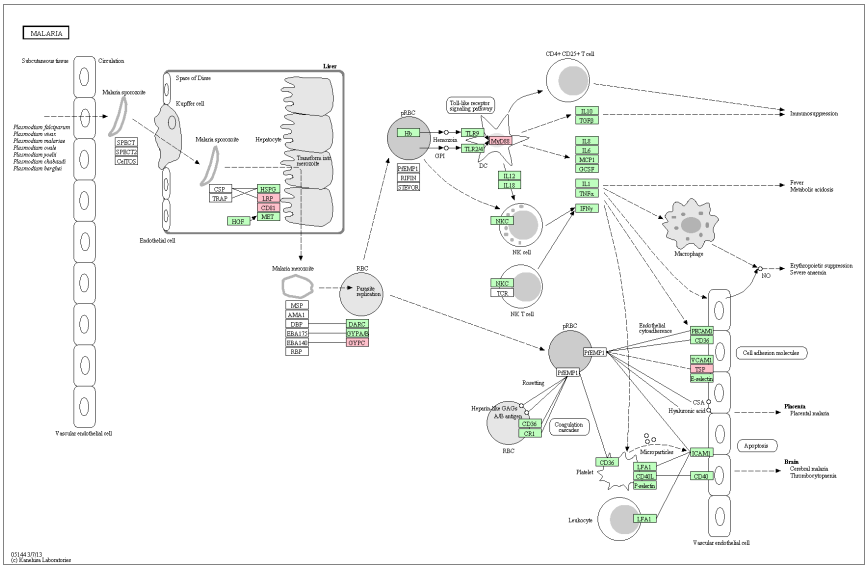

246.3 Legend:

RBH-Blast at 60% Identity + 50% Coverage
Green = Hit in *H. sapiens*
Red = Hit in *H. sapiens* and *T. californica*
White = Not in *H. sapiens*

247 Glycosaminoglycan biosynthesis - keratan sulfate

247.1 Human Pathway: HSA00533

247.2 Number of Hits: 6

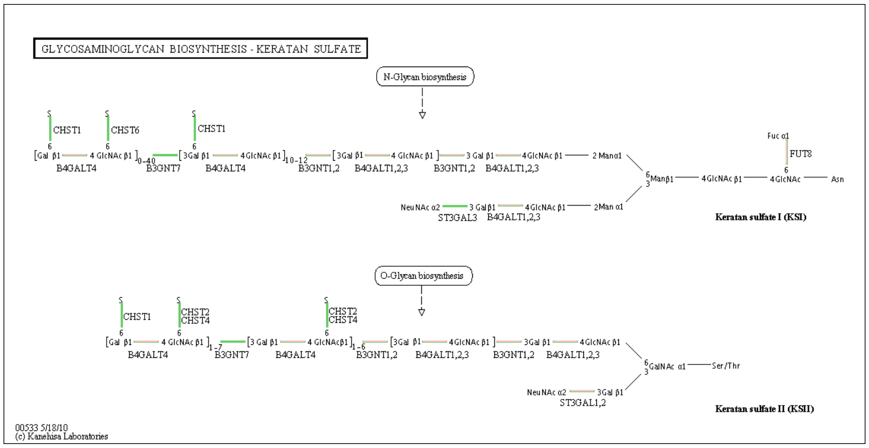

247.3 Legend:

RBH-Blast at 60% Identity + 50% Coverage
Green = Hit in *H. sapiens*
Red = Hit in *H. sapiens* and *T. californica*
White = Not in *H. sapiens*

248 Tyrosine metabolism

248.1 Human Pathway: HSA00350

248.2 Number of Hits: 6

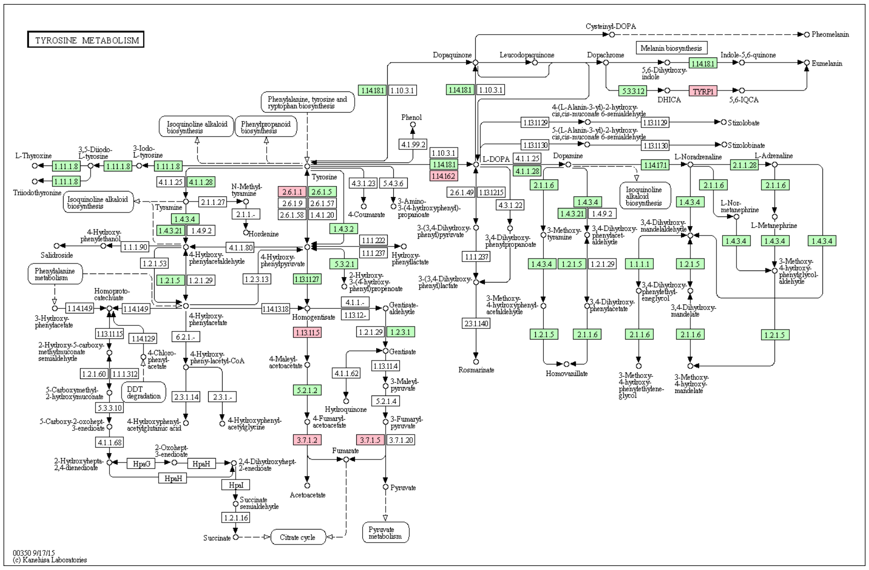

248.3 Legend:

RBH-Blast at 60% Identity + 50% Coverage
Green = Hit in *H. sapiens*
Red = Hit in *H. sapiens* and *T. californica*
White = Not in *H. sapiens*

249 Synthesis and degradation of ketone bodies

249.1 Human Pathway: HSA00072

249.2 Number of Hits: 6

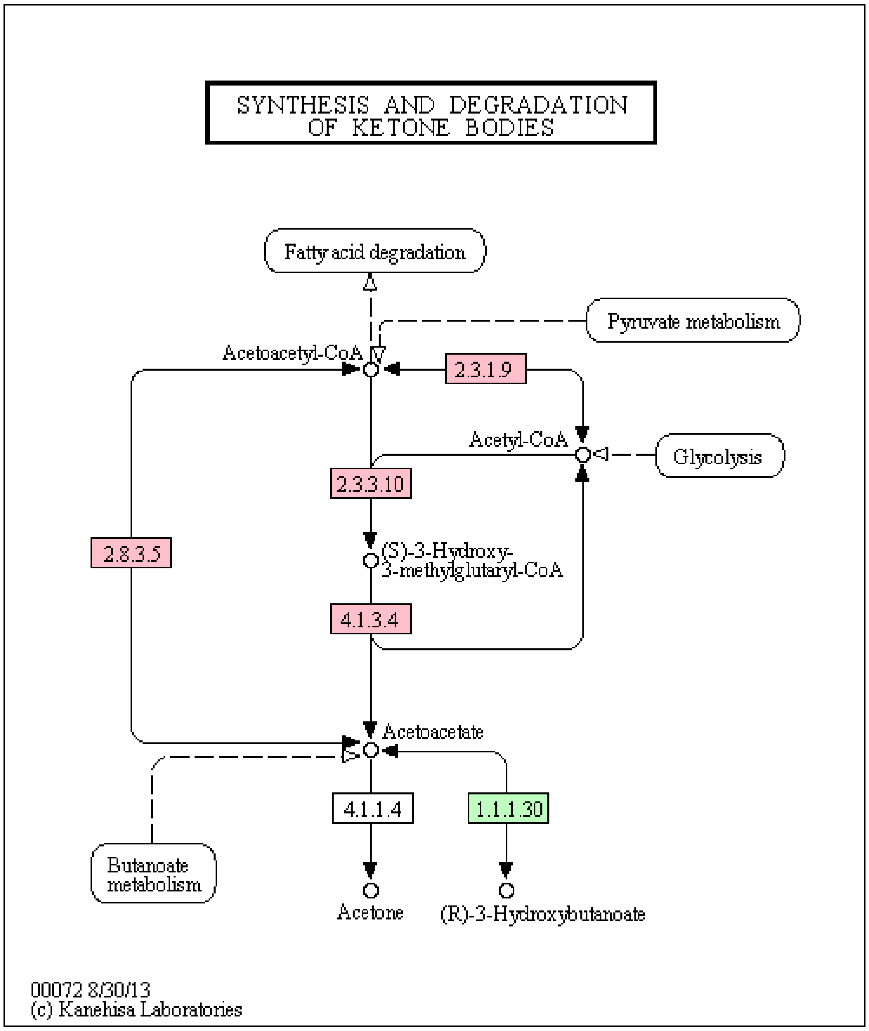

249.3 Legend:

RBH-Blast at 60% Identity + 50% Coverage
Green = Hit in *H. sapiens*
Red = Hit in *H. sapiens* and *T. californica*
White = Not in *H. sapiens*

250 Glycosphingolipid biosynthesis - lacto and neolacto series

250.1 Human Pathway: HSA00601

250.2 Number of Hits: 6

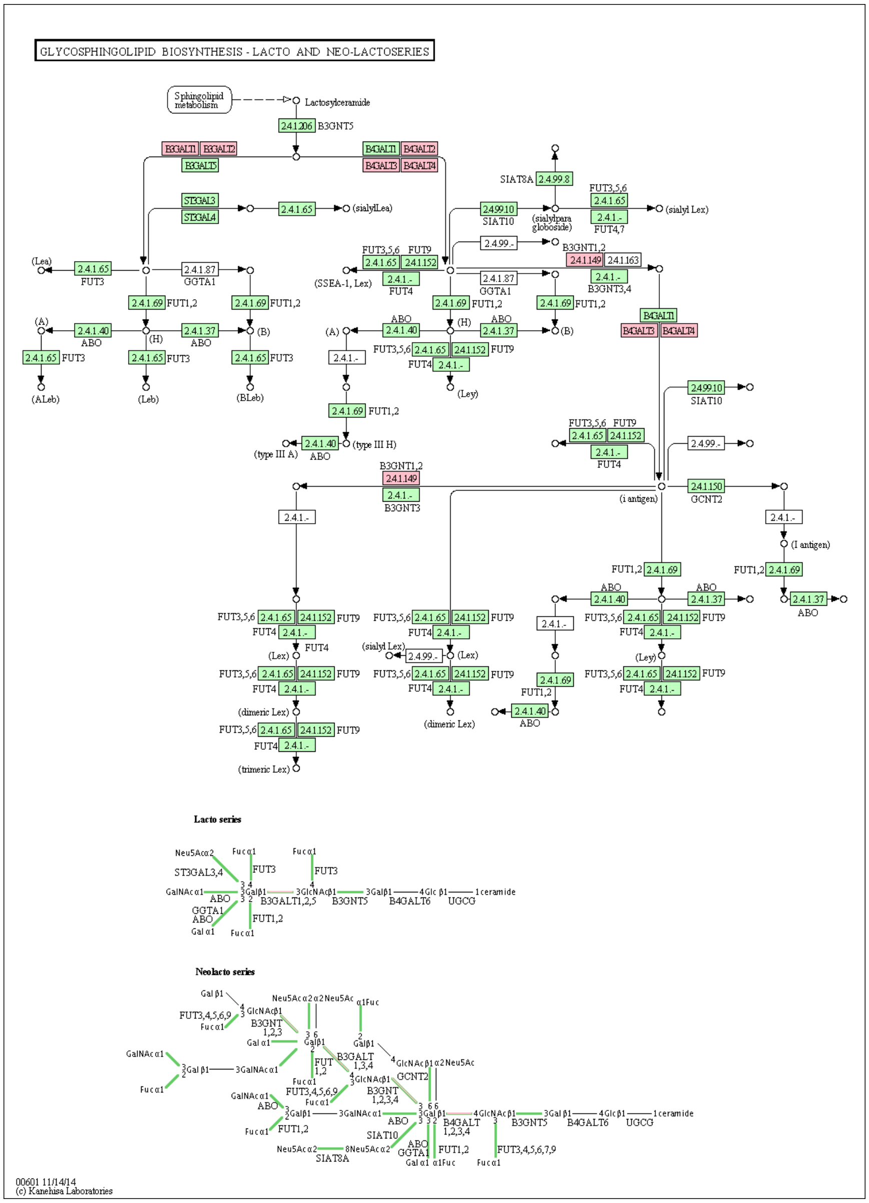

250.3 Legend:

RBH-Blast at 60% Identity + 50% Coverage
Green = Hit in *H. sapiens*
Red = Hit in *H. sapiens* and *T. californica*
White = Not in *H. sapiens*

---

251 African trypanosomiasis

251.1 Human Pathway: HSA05143

251.2 Number of Hits: 6

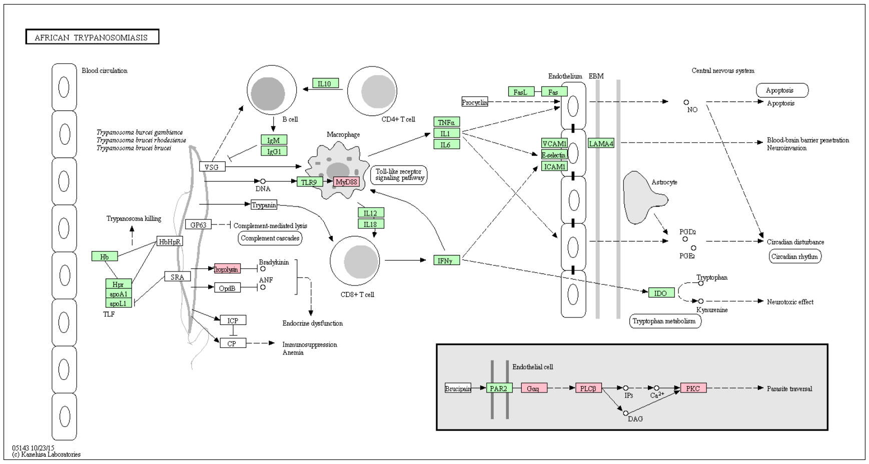

251.3 Legend:

RBH-Blast at 60% Identity + 50% Coverage
Green = Hit in *H. sapiens*
Red = Hit in *H. sapiens* and *T. californica*
White = Not in *H. sapiens*

---

252 Glycosaminoglycan biosynthesis - heparan sulfate / heparin

252.1 Human Pathway: HSA00534

252.2 Number of Hits: 6

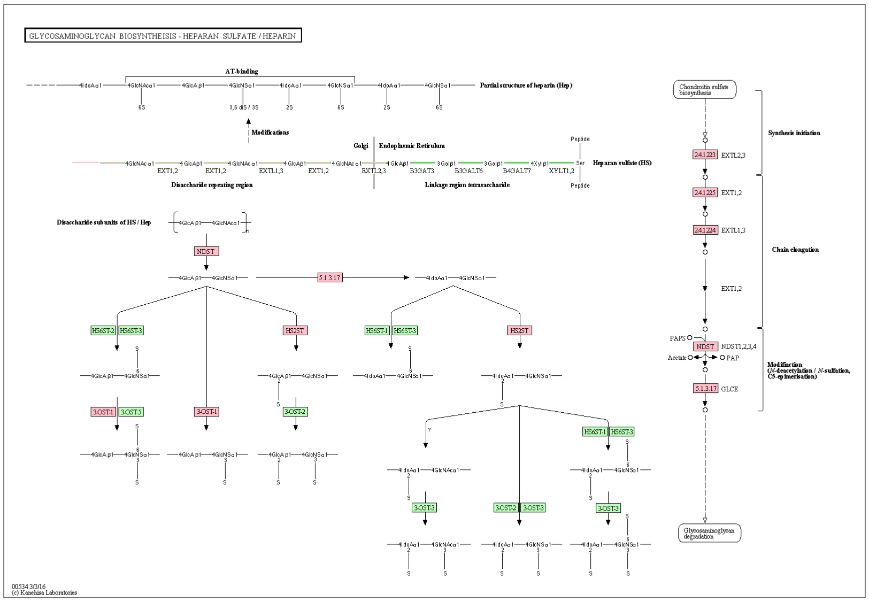

252.3 Legend:

RBH-Blast at 60% Identity + 50% Coverage
Green = Hit in *H. sapiens*
Red = Hit in *H. sapiens* and *T. californica*
White = Not in *H. sapiens*

253 Ascorbate and aldarate metabolism

253.1 Human Pathway: HSA00053

253.2 Number of Hits: 5

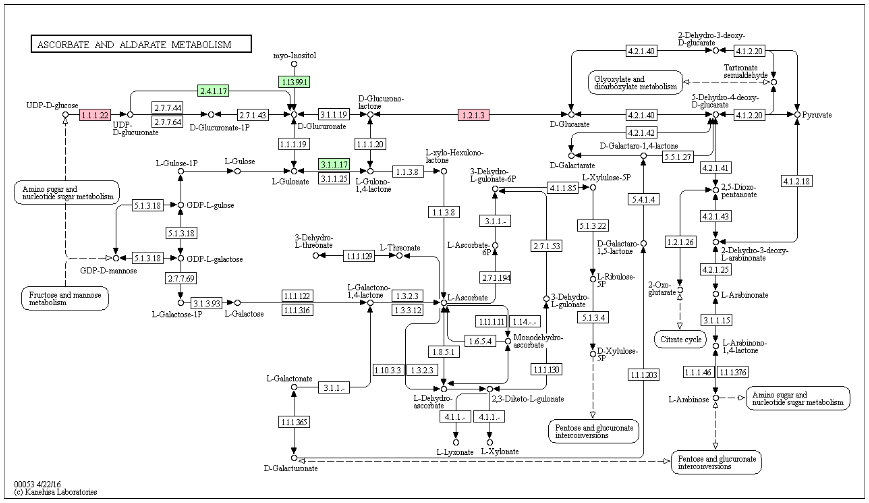

253.3 Legend:

RBH-Blast at 60% Identity + 50% Coverage
Green = Hit in *H. sapiens*
Red = Hit in *H. sapiens* and *T. californica*
White = Not in *H. sapiens*

254 Maturity onset diabetes of the young

254.1 Human Pathway: HSA04950

254.2 Number of Hits: 5

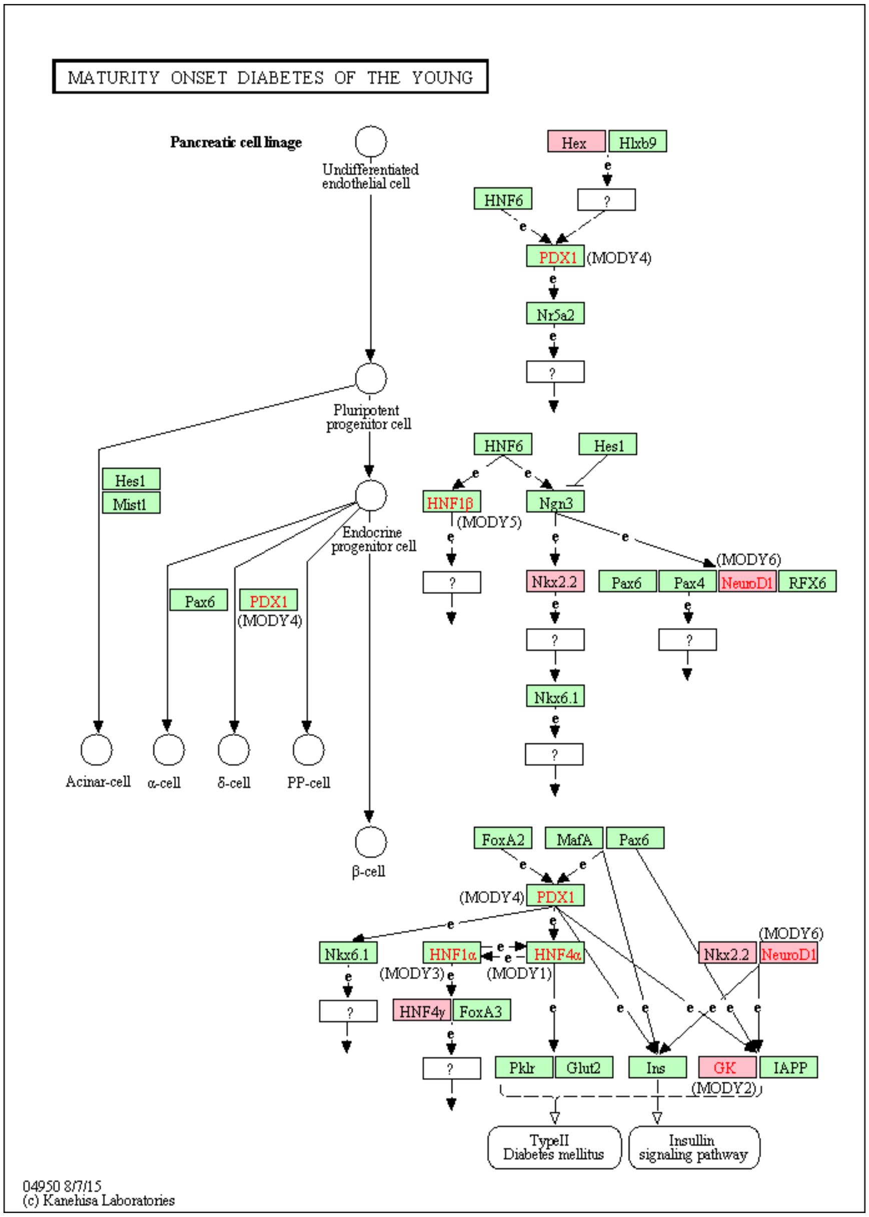

254.3 Legend:

RBH-Blast at 60% Identity + 50% Coverage
Green = Hit in *H. sapiens*
Red = Hit in *H. sapiens* and *T. californica*
White = Not in *H. sapiens*

---

255 Sulfur metabolism

255.1 Human Pathway: HSA00920

255.2 Number of Hits: 5

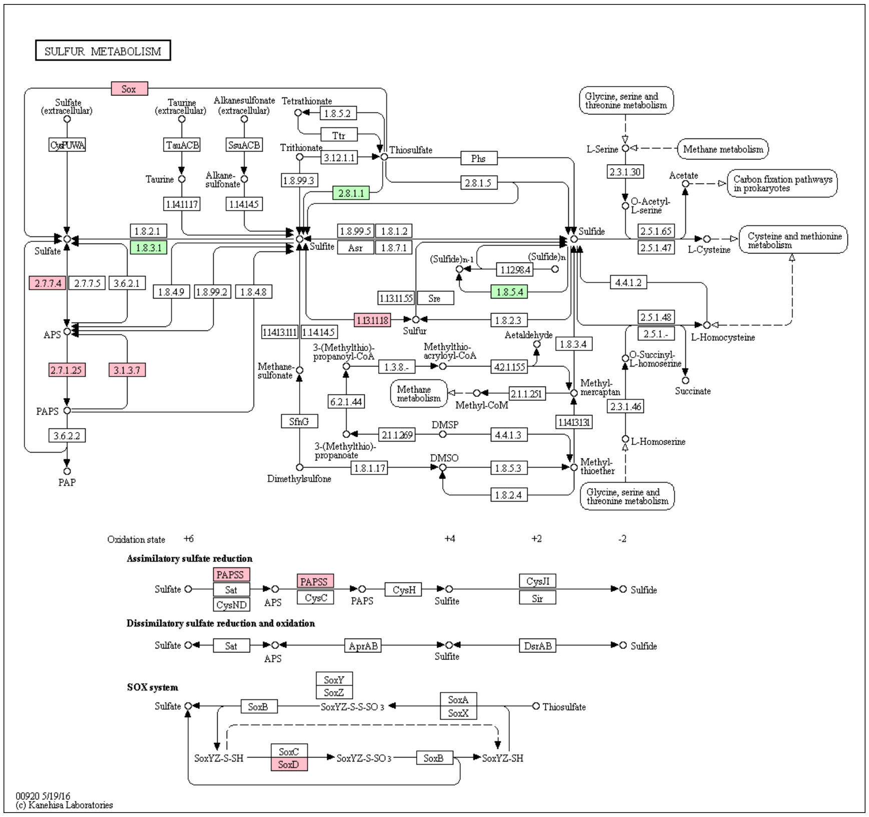

255.3 Legend:

RBH-Blast at 60% Identity + 50% Coverage
Green = Hit in *H. sapiens*
Red = Hit in *H. sapiens* and *T. californica*
White = Not in *H. sapiens*

---

256 Type I diabetes mellitus

256.1 Human Pathway: HSA04940

256.2 Number of Hits: 5

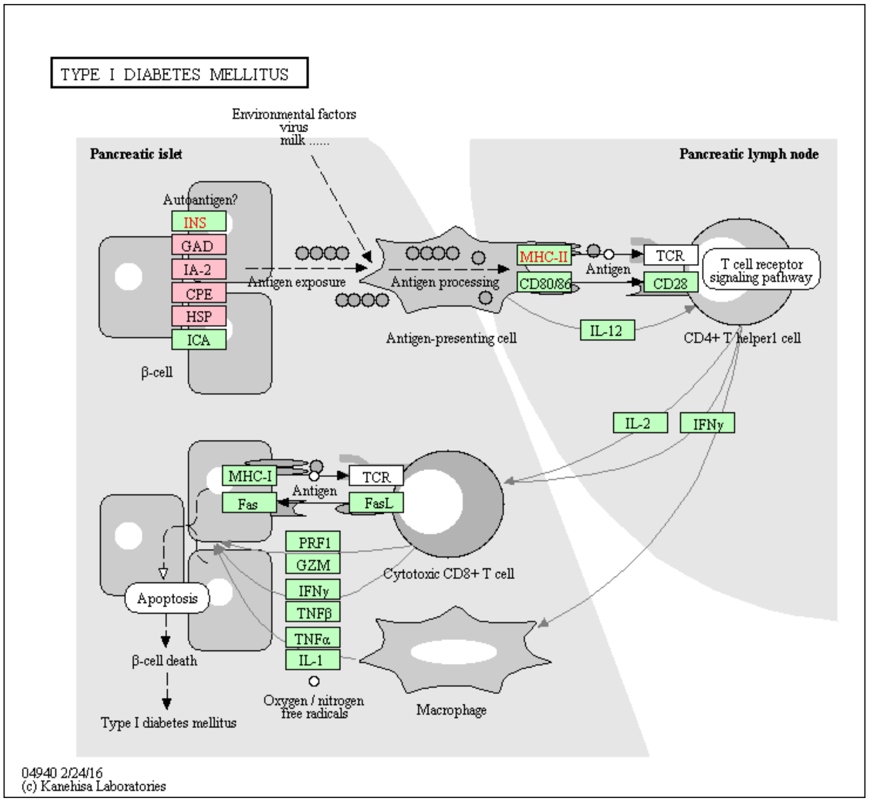

256.3 Legend:

RBH-Blast at 60% Identity + 50% Coverage
Green = Hit in *H. sapiens*
Red = Hit in *H. sapiens* and *T. californica*
White = Not in *H. sapiens*

---

257 Cytokine-cytokine receptor interaction

257.1 Human Pathway: HSA04060

257.2 Number of Hits: 5

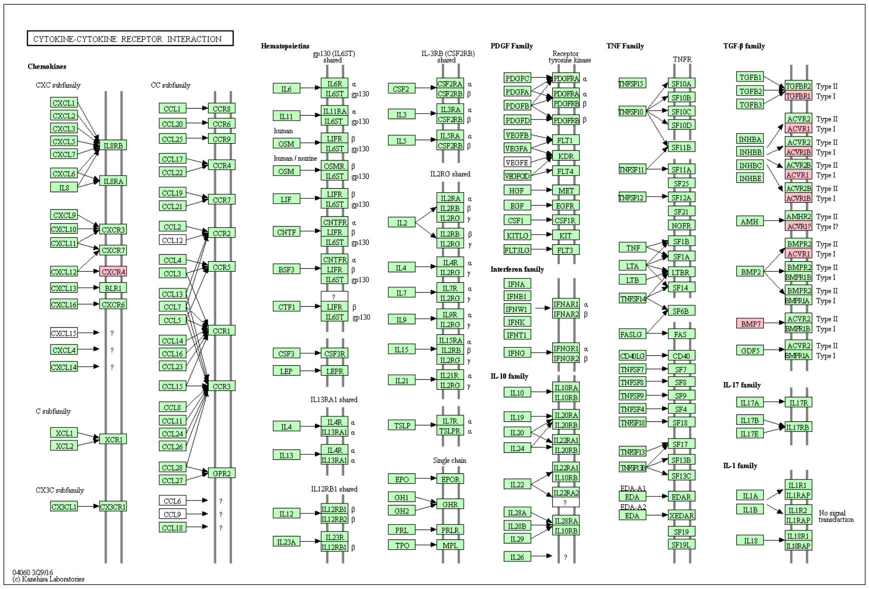

257.3 Legend:

RBH-Blast at 60% Identity + 50% Coverage
Green = Hit in *H. sapiens*
Red = Hit in *H. sapiens* and *T. californica*
White = Not in *H. sapiens*

---

258 Taurine and hypotaurine metabolism

258.1 Human Pathway: HSA00430

258.2 Number of Hits: 4

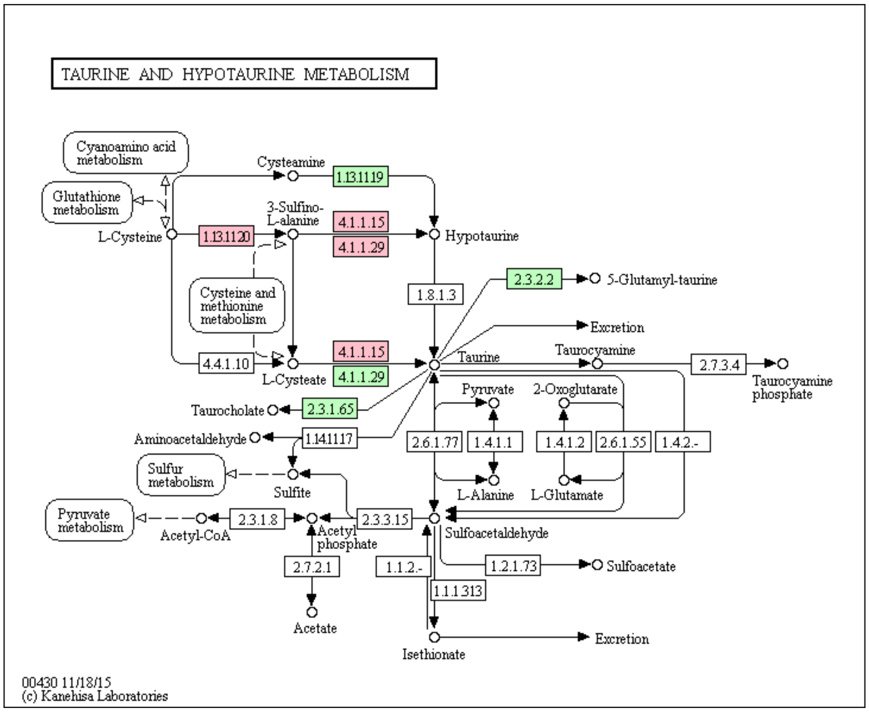

258.3 Legend:

RBH-Blast at 60% Identity + 50% Coverage
Green = Hit in *H. sapiens*
Red = Hit in *H. sapiens* and *T. californica*
White = Not in *H. sapiens*

259 Fatty acid biosynthesis

259.1 Human Pathway: HSA00061

259.2 Number of Hits: 4

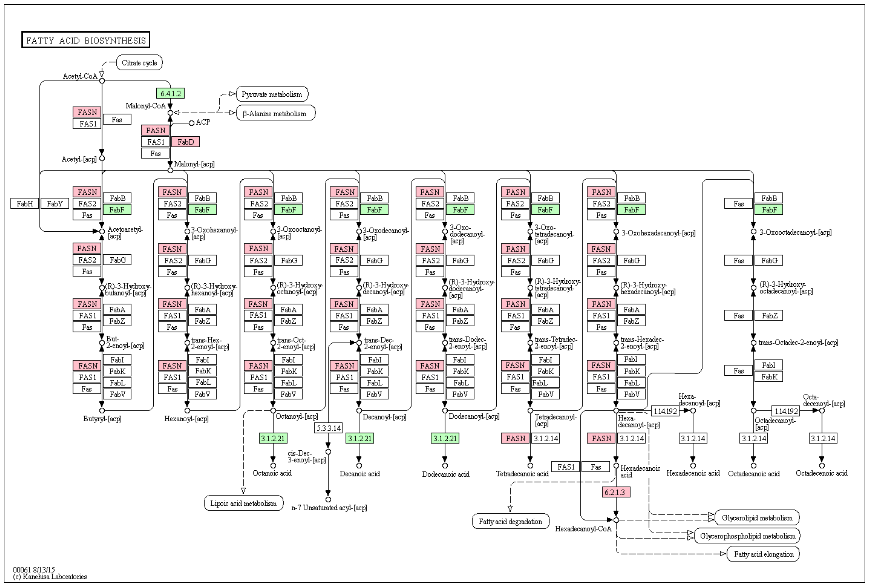

259.3 Legend:

RBH-Blast at 60% Identity + 50% Coverage
Green = Hit in *H. sapiens*
Red = Hit in *H. sapiens* and *T. californica*
White = Not in *H. sapiens*

260 Inflammatory bowel disease (IBD)

260.1 Human Pathway: HSA05321

260.2 Number of Hits: 4

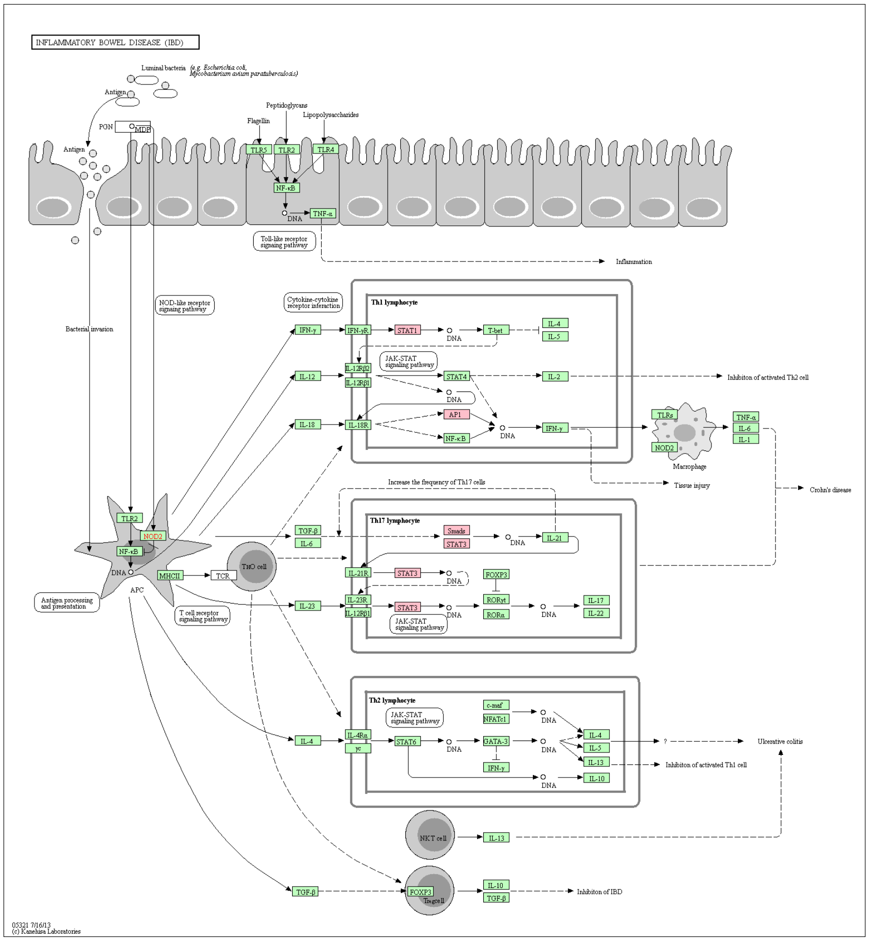

260.3 Legend:

RBH-Blast at 60% Identity + 50% Coverage
Green = Hit in *H. sapiens*
Red = Hit in *H. sapiens* and *T. californica*
White = Not in *H. sapiens*

261 Renin-angiotensin system

261.1 Human Pathway: HSA04614

261.2 Number of Hits: 4

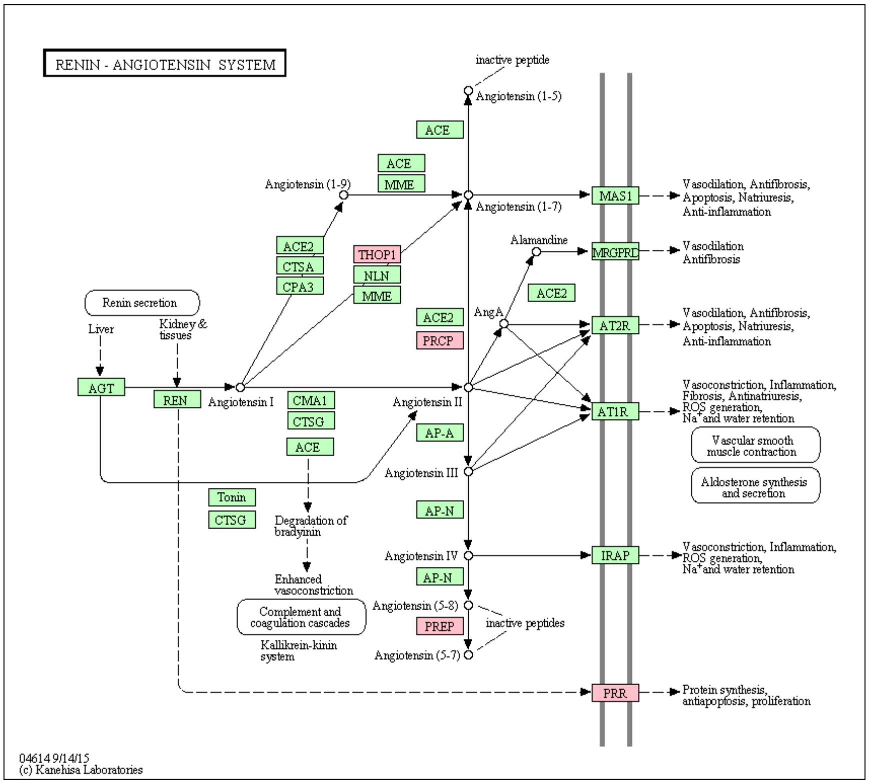

261.3 Legend:

RBH-Blast at 60% Identity + 50% Coverage
Green = Hit in *H. sapiens*
Red = Hit in *H. sapiens* and *T. californica*
White = Not in *H. sapiens*

262 Glycosaminoglycan degradation

262.1 Human Pathway: HSA00531

262.2 Number of Hits: 4

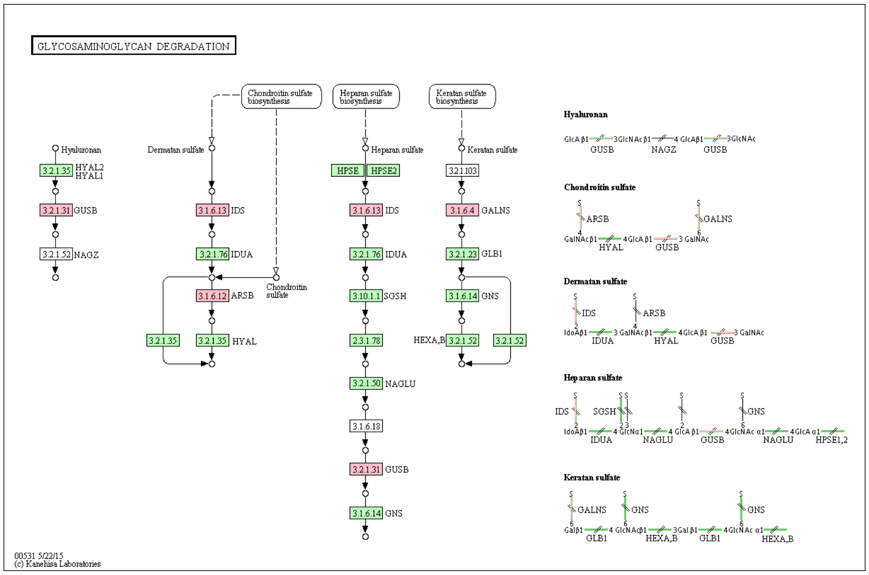

262.3 Legend:

RBH-Blast at 60% Identity + 50% Coverage
Green = Hit in *H. sapiens*
Red = Hit in *H. sapiens* and *T. californica*
White = Not in *H. sapiens*

263 ABC transporters

263.1 Human Pathway: HSA02010

263.2 Number of Hits: 4

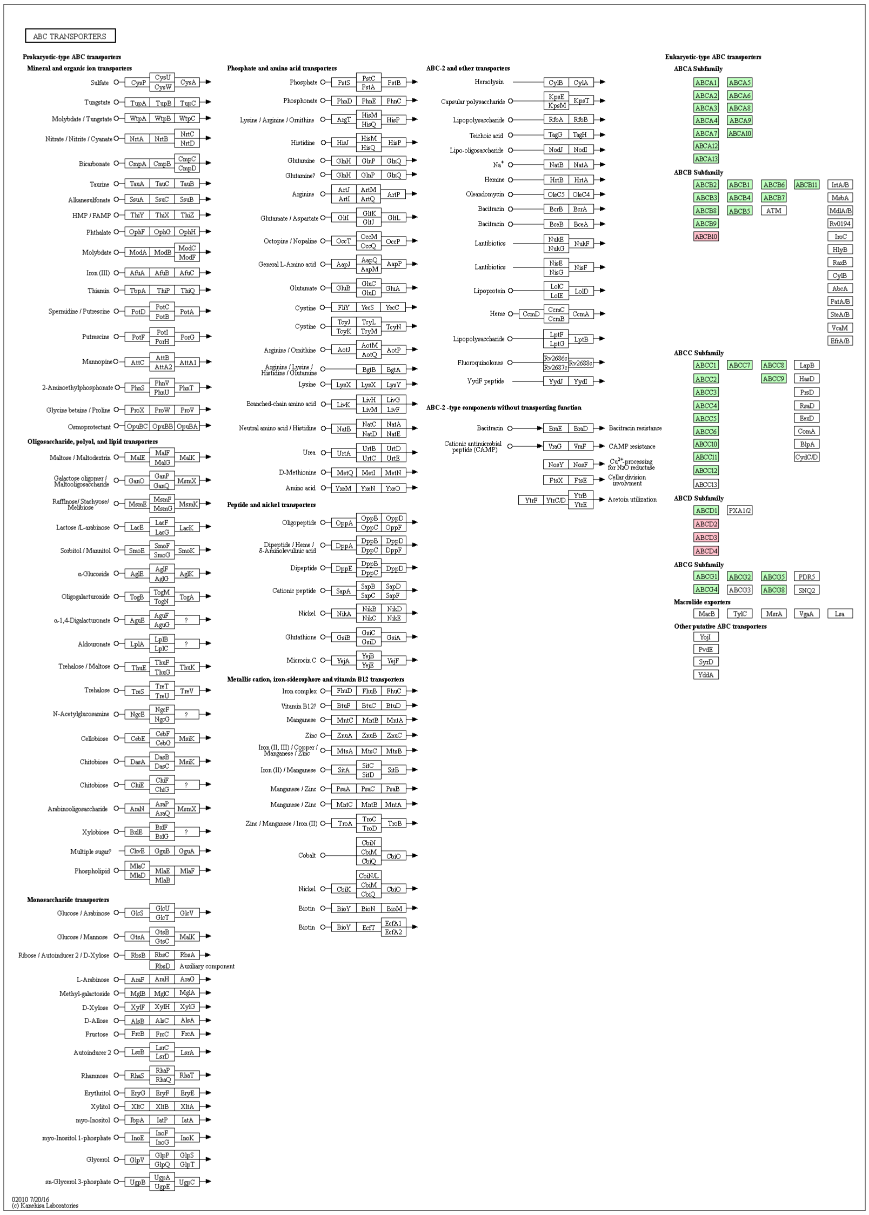

263.3 Legend:

RBH-Blast at 60% Identity + 50% Coverage
Green = Hit in *H. sapiens*
Red = Hit in *H. sapiens* and *T. californica*
White = Not in *H. sapiens*

264 Nitrogen metabolism

264.1 Human Pathway: HSA00910

264.2 Number of Hits: 4

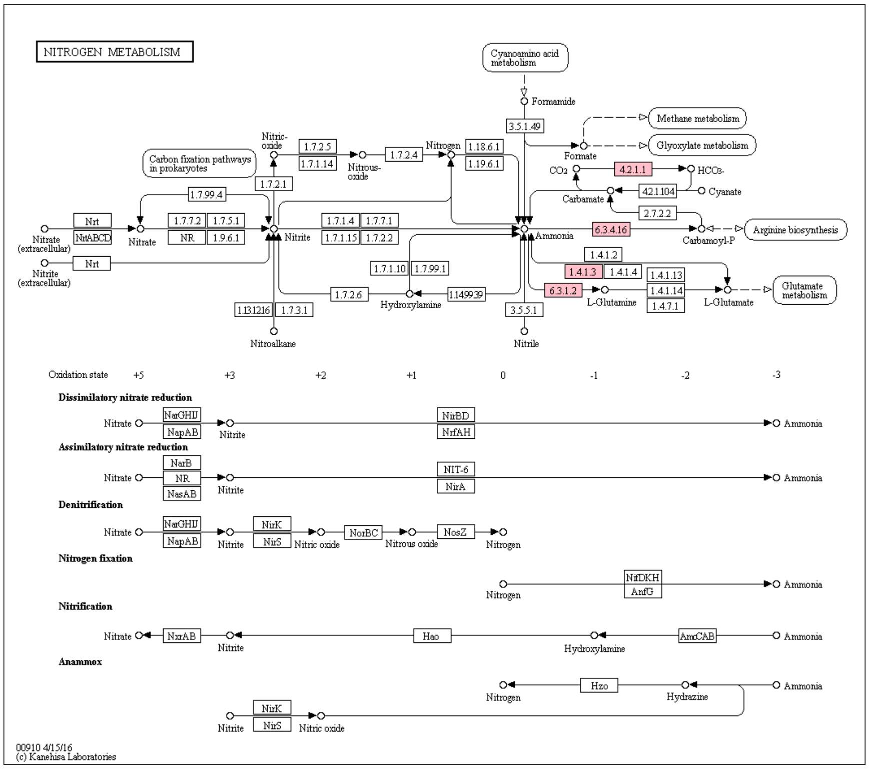

264.3 Legend:

RBH-Blast at 60% Identity + 50% Coverage
Green = Hit in *H. sapiens*
Red = Hit in *H. sapiens* and *T. californica*
White = Not in *H. sapiens*

265 Primary bile acid biosynthesis

265.1 Human Pathway: HSA00120

265.2 Number of Hits: 3

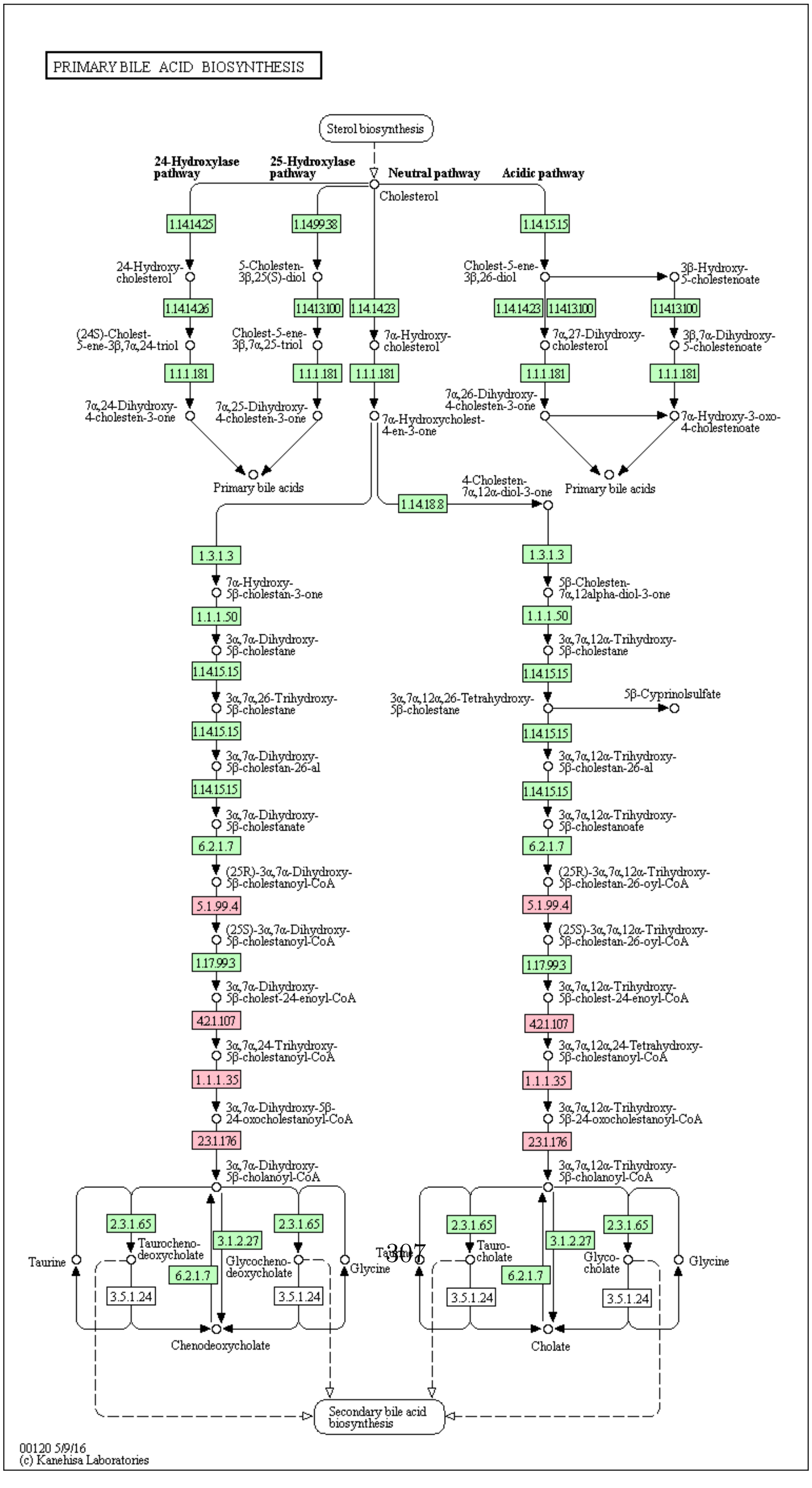

265.3 Legend:

RBH-Blast at 60% Identity + 50% Coverage
Green = Hit in *H. sapiens*
Red = Hit in *H. sapiens* and *T. californica*
White = Not in *H. sapiens*

266 Glycosylphosphatidylinositol(GPI)-anchor biosynthesis

266.1 Human Pathway: HSA00563

266.2 Number of Hits: 3

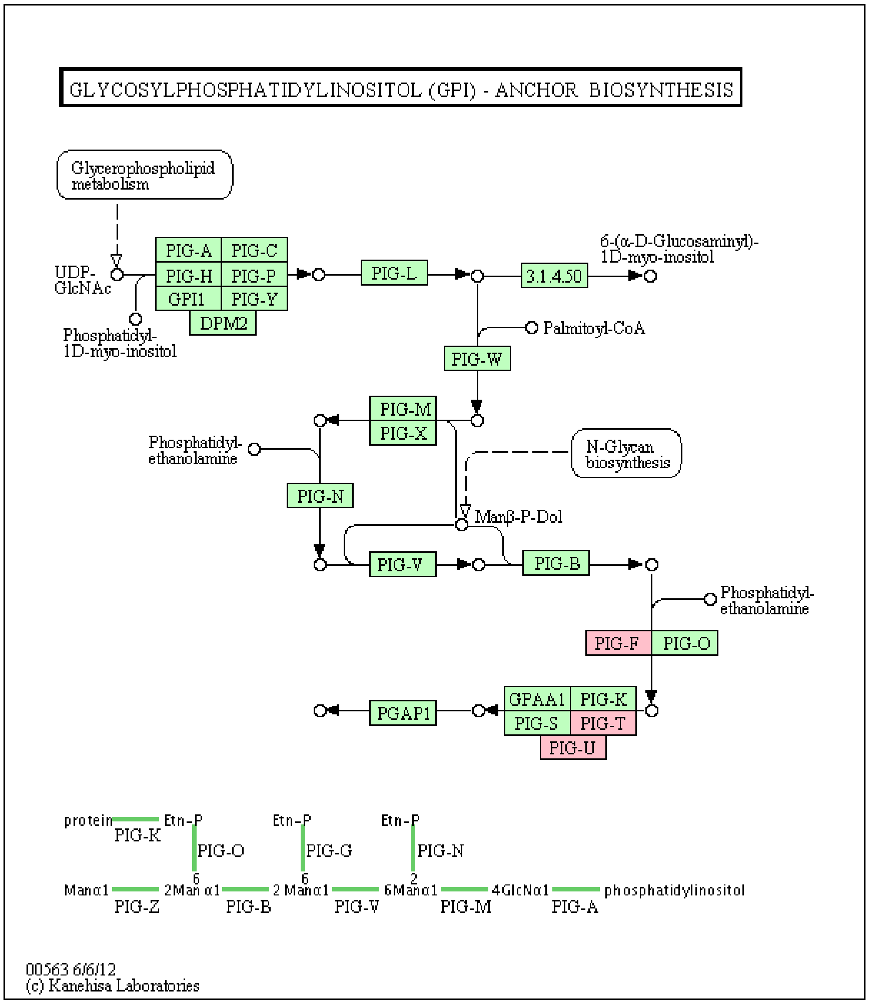

266.3 Legend:

RBH-Blast at 60% Identity + 50% Coverage
Green = Hit in *H. sapiens*
Red = Hit in *H. sapiens* and *T. californica*
White = Not in *H. sapiens*

---

267 Phototransduction

267.1 Human Pathway: HSA04744

267.2 Number of Hits: 3

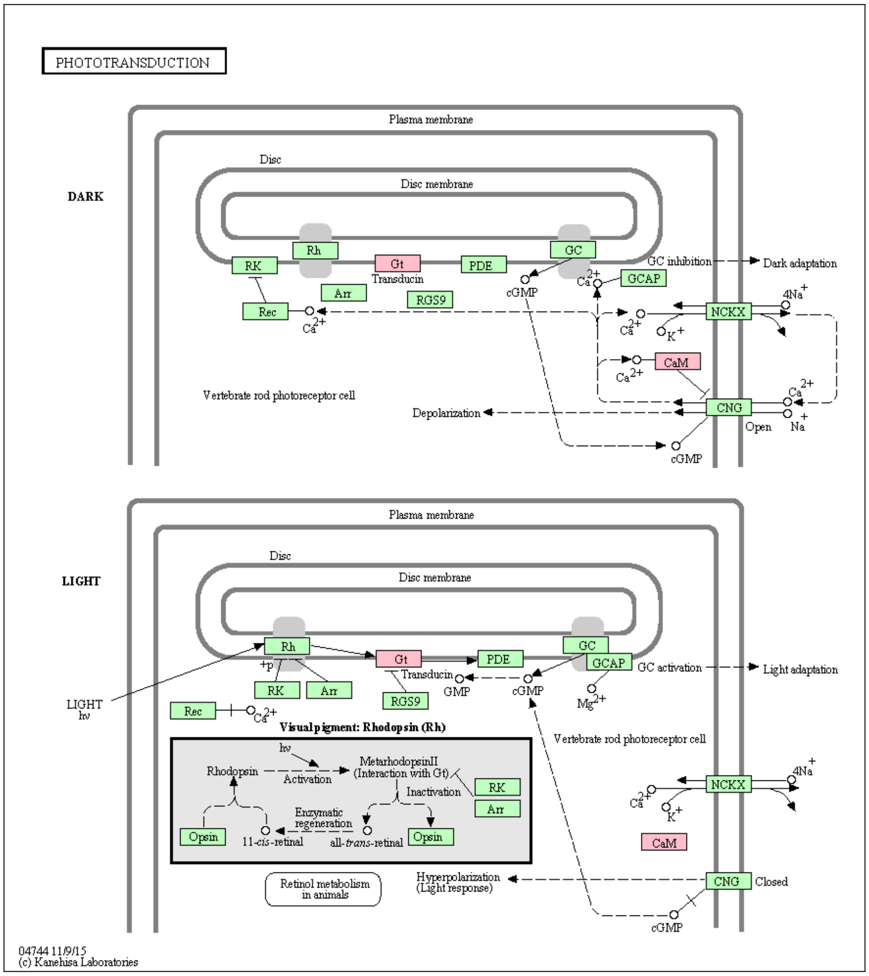

267.3 Legend:

RBH-Blast at 60% Identity + 50% Coverage
Green = Hit in *H. sapiens*
Red = Hit in *H. sapiens* and *T. californica*
White = Not in *H. sapiens*

---

268 alpha-Linolenic acid metabolism

268.1 Human Pathway: HSA00592

268.2 Number of Hits: 3

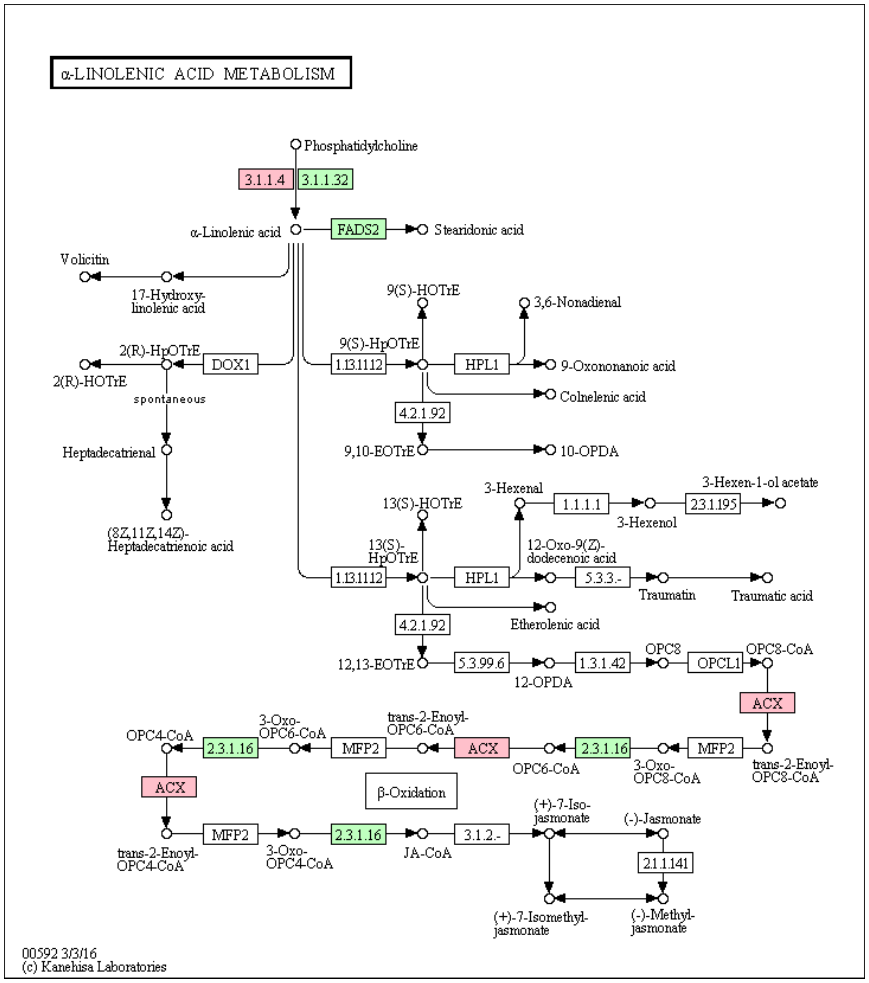

268.3 Legend:

RBH-Blast at 60% Identity + 50% Coverage
Green = Hit in *H. sapiens*
Red = Hit in *H. sapiens* and *T. californica*
White = Not in *H. sapiens*

---

269 Other glycan degradation

269.1 Human Pathway: HSA00511

269.2 Number of Hits: 3

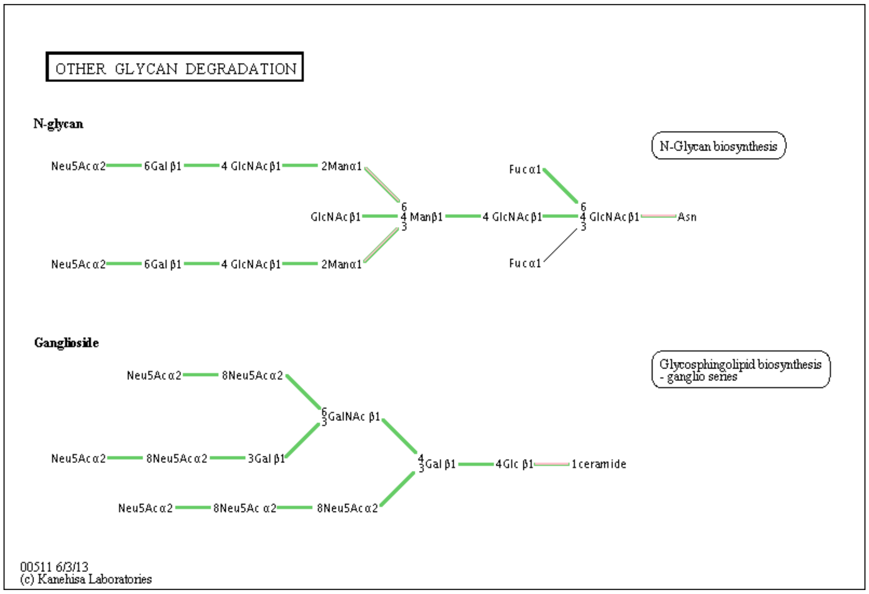

269.3 Legend:

RBH-Blast at 60% Identity + 50% Coverage
Green = Hit in *H. sapiens*
Red = Hit in *H. sapiens* and *T. californica*
White = Not in *H. sapiens*

---

270 Sulfur relay system

270.1 Human Pathway: HSA04122

270.2 Number of Hits: 3

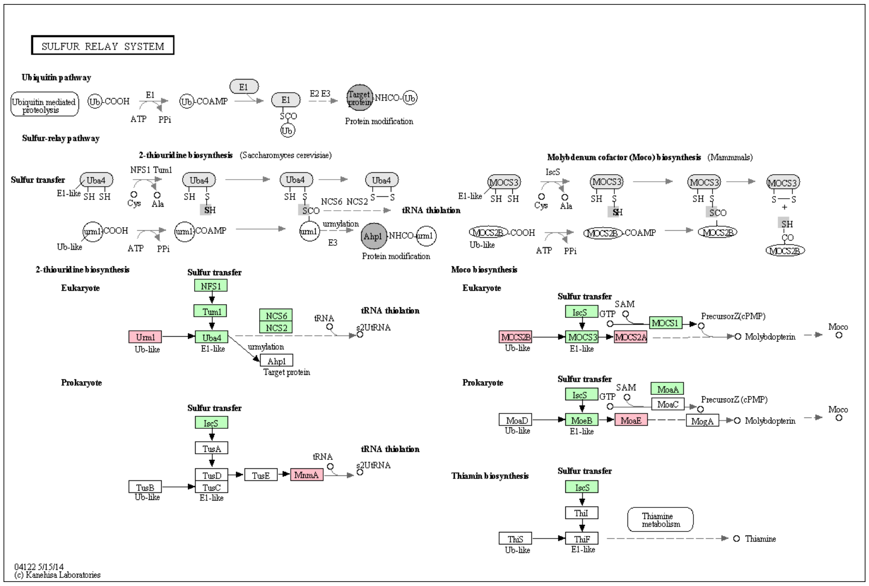

270.3 Legend:

RBH-Blast at 60% Identity + 50% Coverage
Green = Hit in *H. sapiens*
Red = Hit in *H. sapiens* and *T. californica*
White = Not in *H. sapiens*

271 Glycosphingolipid biosynthesis - ganglio series

271.1 Human Pathway: HSA00604

271.2 Number of Hits: 3

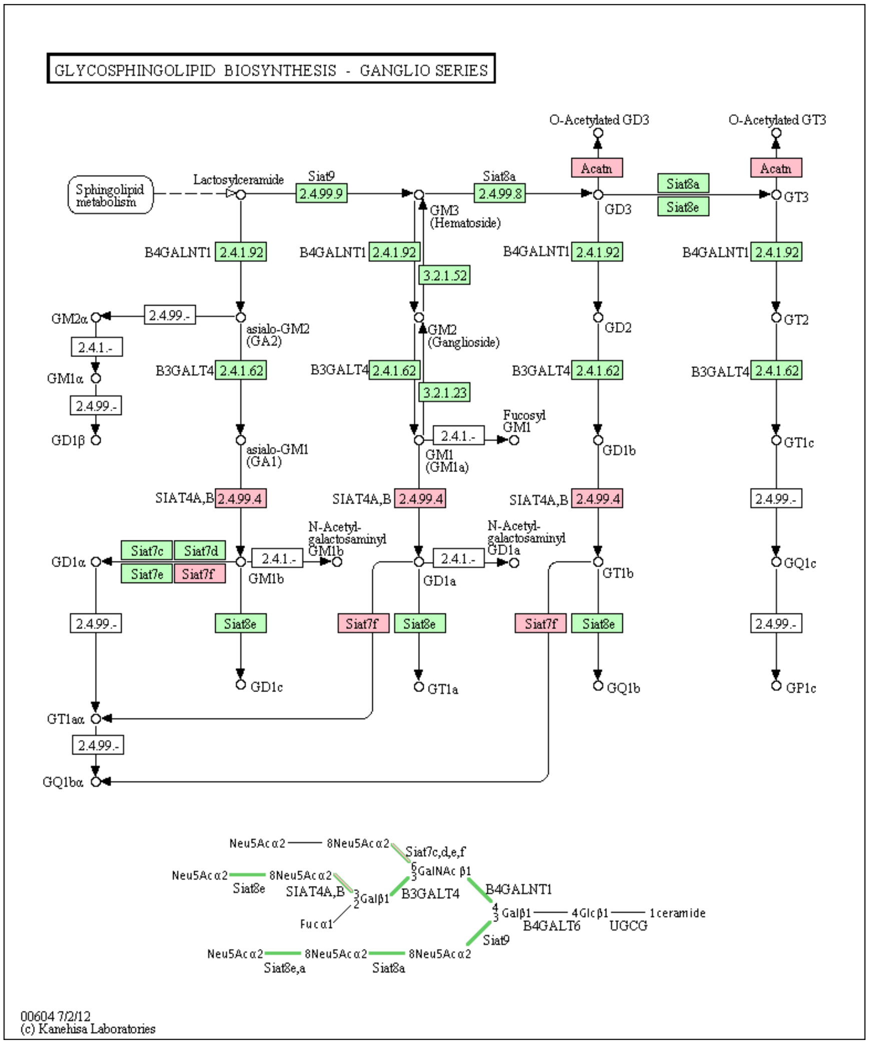

271.3 Legend:

RBH-Blast at 60% Identity + 50% Coverage
Green = Hit in *H. sapiens*
Red = Hit in *H. sapiens* and *T. californica*
White = Not in *H. sapiens*

---

272 Valine, leucine and isoleucine biosynthesis

272.1 Human Pathway: HSA00290

272.2 Number of Hits: 2

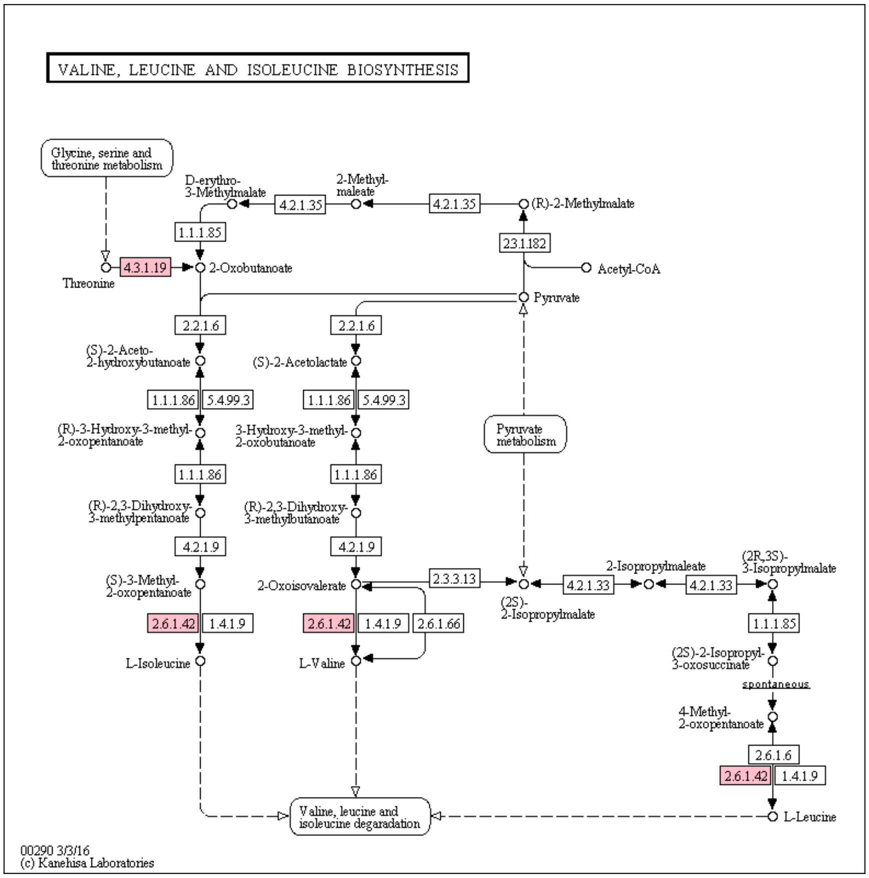

272.3 Legend:

RBH-Blast at 60% Identity + 50% Coverage
Green = Hit in *H. sapiens*
Red = Hit in *H. sapiens* and *T. californica*
White = Not in *H. sapiens*

273 Ubiquinone and other terpenoid-quinone biosynthesis

273.1 Human Pathway: HSA00130

273.2 Number of Hits: 2

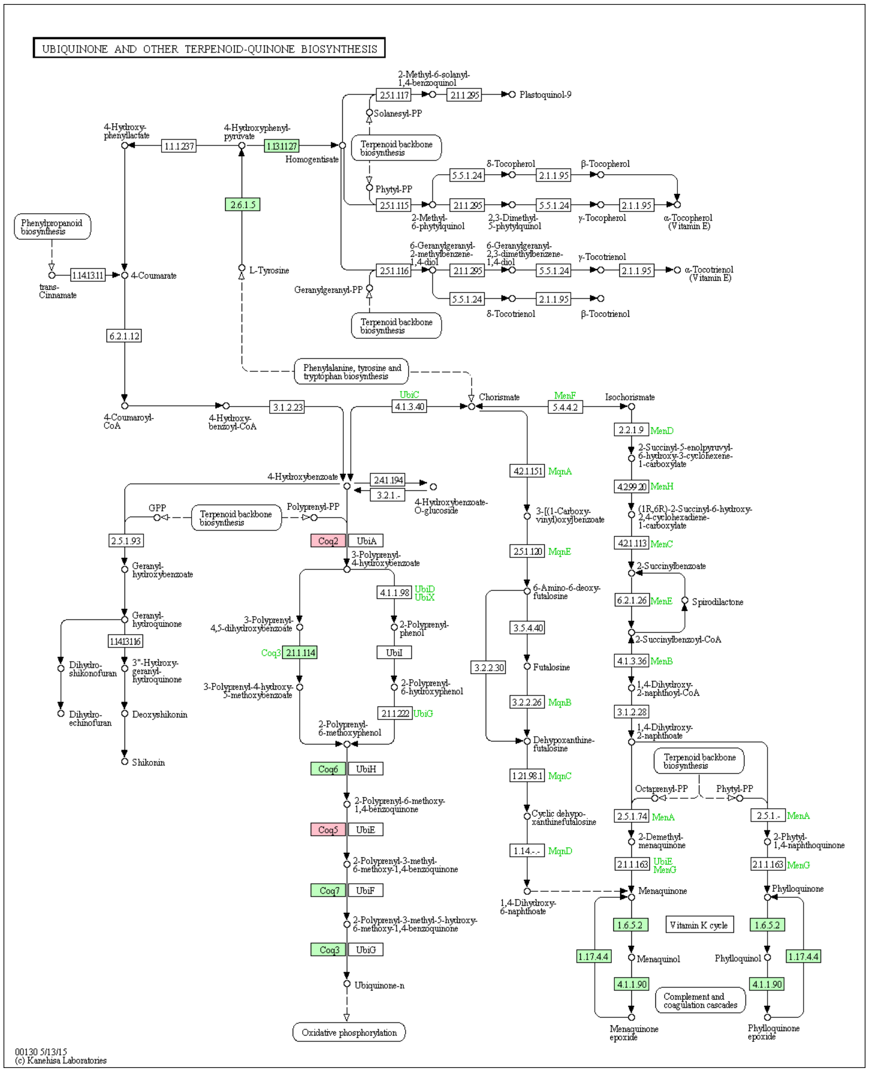

273.3 Legend:

RBH-Blast at 60% Identity + 50% Coverage
Green = Hit in *H. sapiens*
Red = Hit in *H. sapiens* and *T. californica*
White = Not in *H. sapiens*

274 Metabolism of xenobiotics by cytochrome P450

274.1 Human Pathway: HSA00980

274.2 Number of Hits: 2

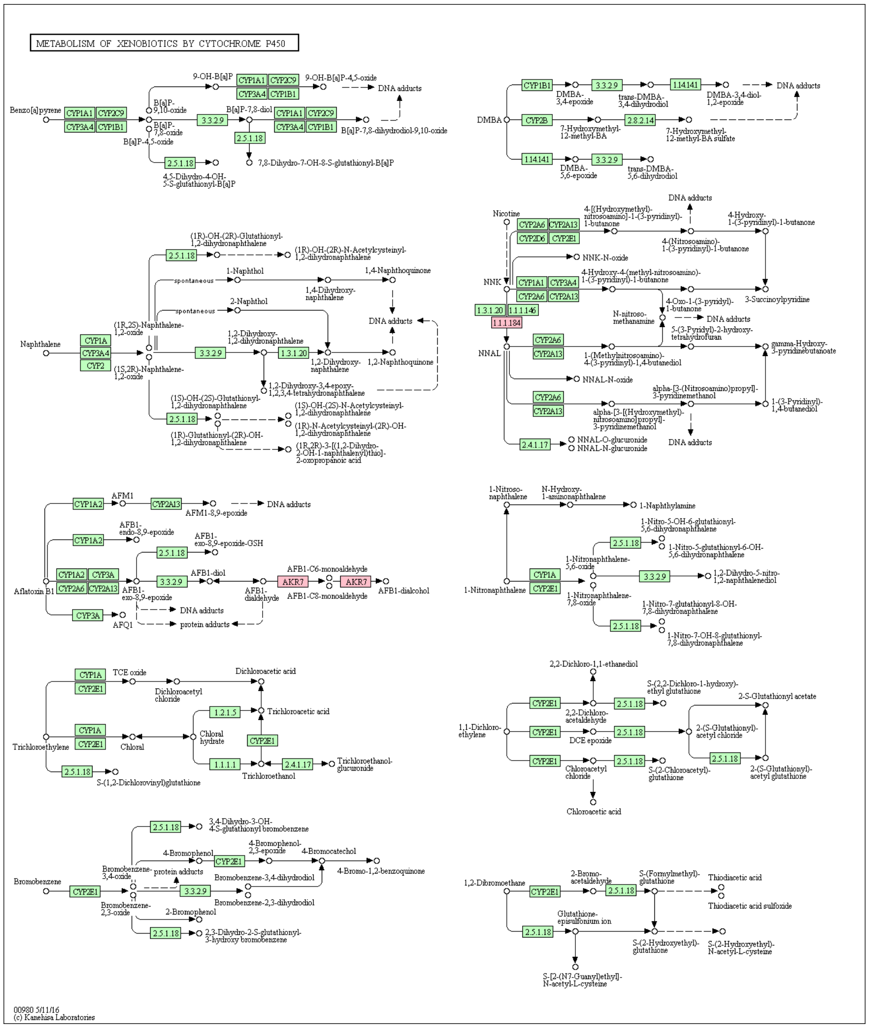

274.3 Legend:

RBH-Blast at 60% Identity + 50% Coverage
Green = Hit in *H. sapiens*
Red = Hit in *H. sapiens* and *T. californica*
White = Not in *H. sapiens*

275 Lysine biosynthesis

275.1 Human Pathway: HSA00300

275.2 Number of Hits: 2

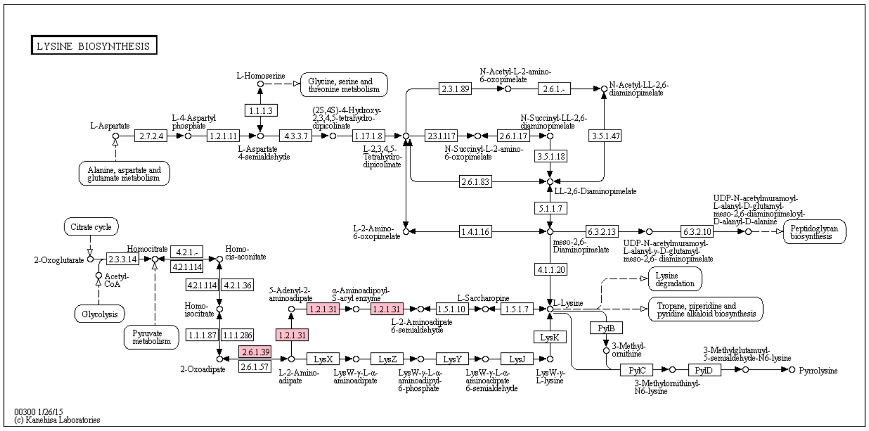

275.3 Legend:

RBH-Blast at 60% Identity + 50% Coverage
Green = Hit in *H. sapiens*
Red = Hit in *H. sapiens* and *T. californica*
White = Not in *H. sapiens*

276 Linoleic acid metabolism

276.1 Human Pathway: HSA00591

276.2 Number of Hits: 2

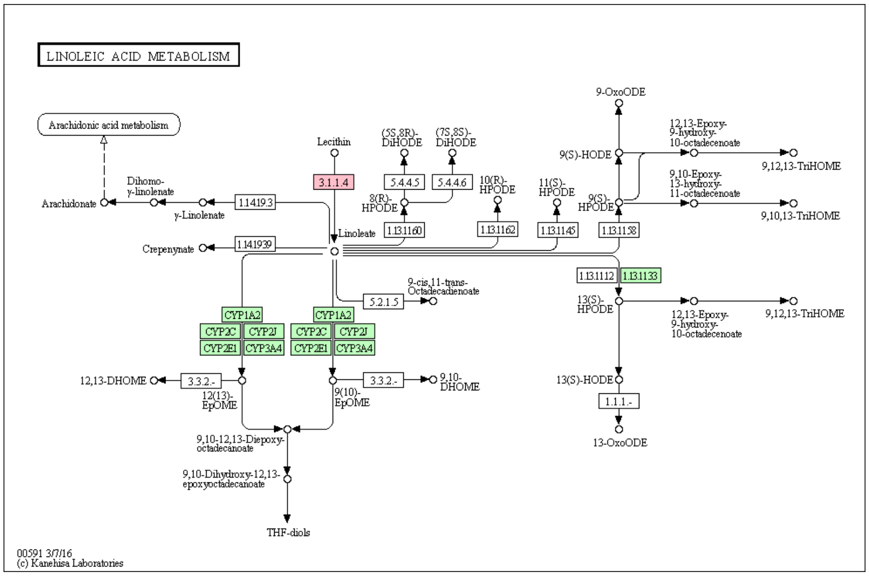

276.3 Legend:

RBH-Blast at 60% Identity + 50% Coverage
Green = Hit in *H. sapiens*
Red = Hit in *H. sapiens* and *T. californica*
White = Not in *H. sapiens*

277 Glycosphingolipid biosynthesis - globo series

277.1 Human Pathway: HSA00603

277.2 Number of Hits: 2

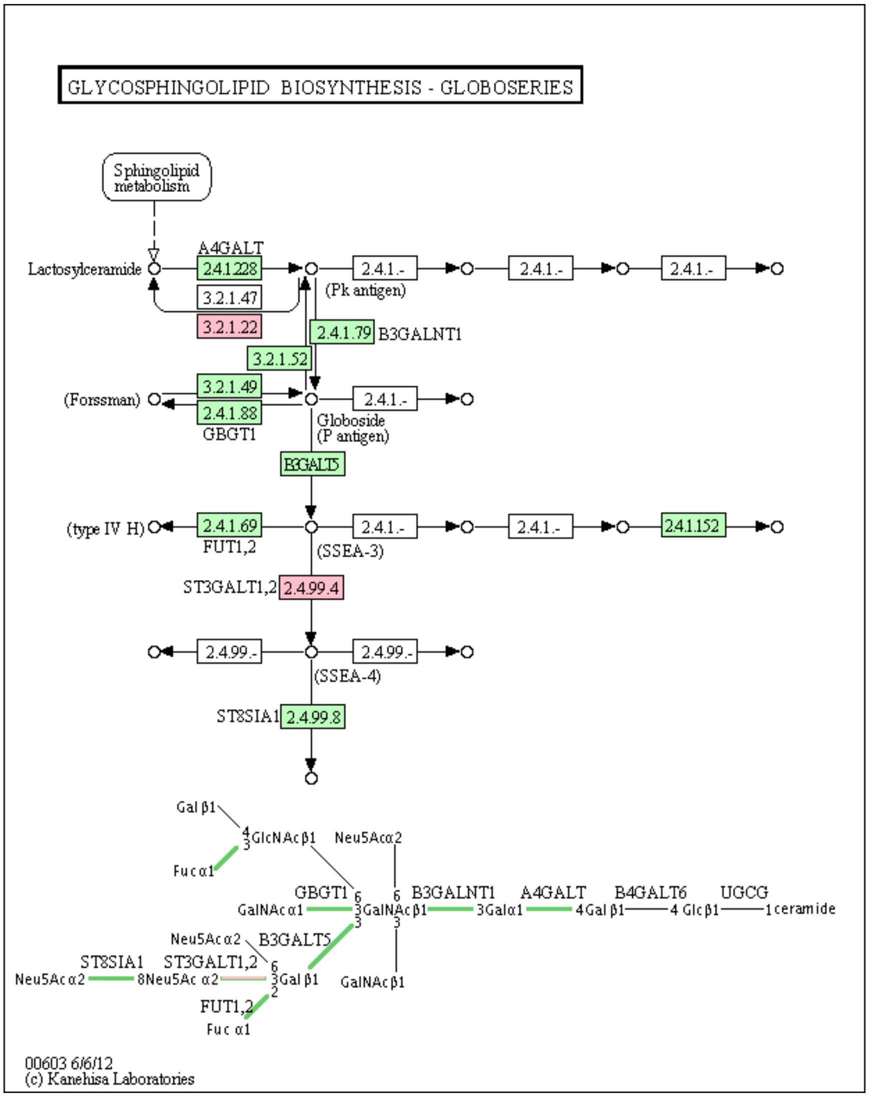

277.3 Legend:

RBH-Blast at 60% Identity + 50% Coverage
Green = Hit in *H. sapiens*
Red = Hit in *H. sapiens* and *T. californica*
White = Not in *H. sapiens*

---

278 Butirosin and neomycin biosynthesis

278.1 Human Pathway: HSA00524

278.2 Number of Hits: 2

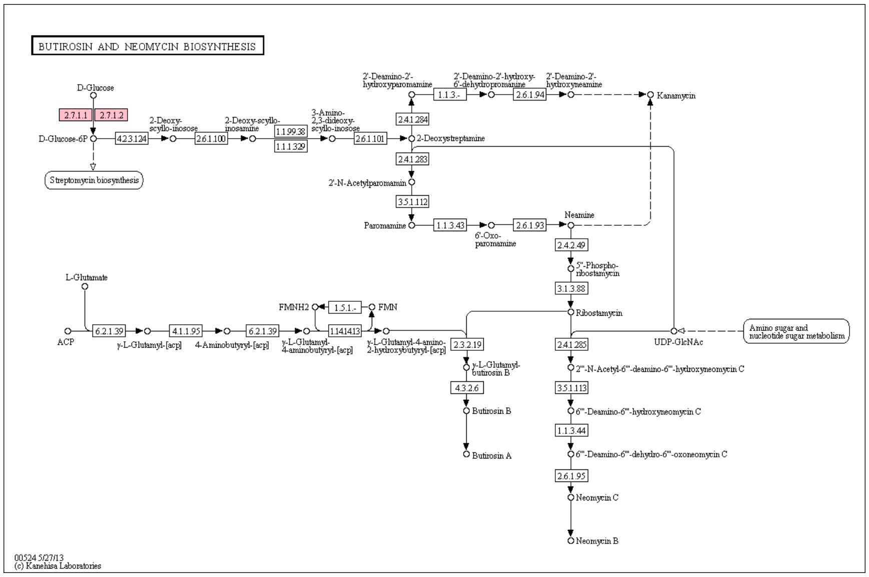

278.3 Legend:

RBH-Blast at 60% Identity + 50% Coverage
Green = Hit in *H. sapiens*
Red = Hit in *H. sapiens* and *T. californica*
White = Not in *H. sapiens*

---

279 Vitamin B6 metabolism

279.1 Human Pathway: HSA00750

279.2 Number of Hits: 2

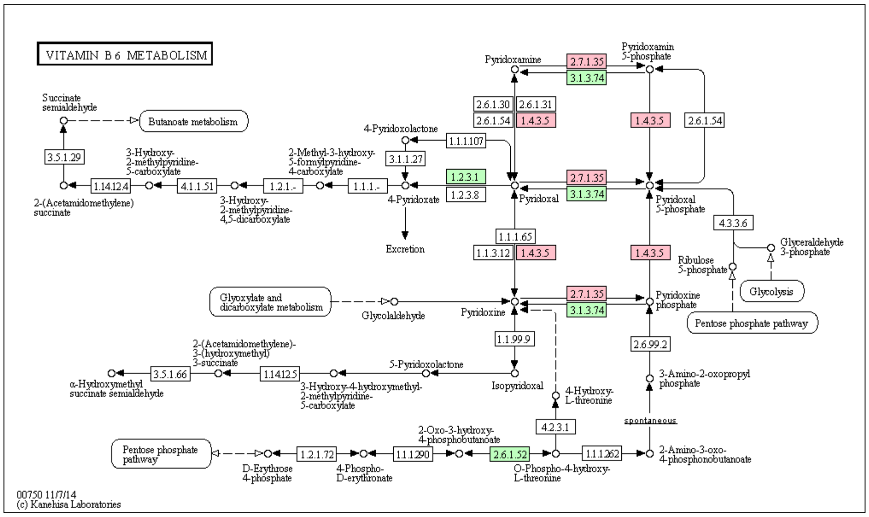

279.3 Legend:

RBH-Blast at 60% Identity + 50% Coverage
Green = Hit in *H. sapiens*
Red = Hit in *H. sapiens* and *T. californica*
White = Not in *H. sapiens*

280 Non-homologous end-joining

280.1 Human Pathway: HSA03450

280.2 Number of Hits: 2

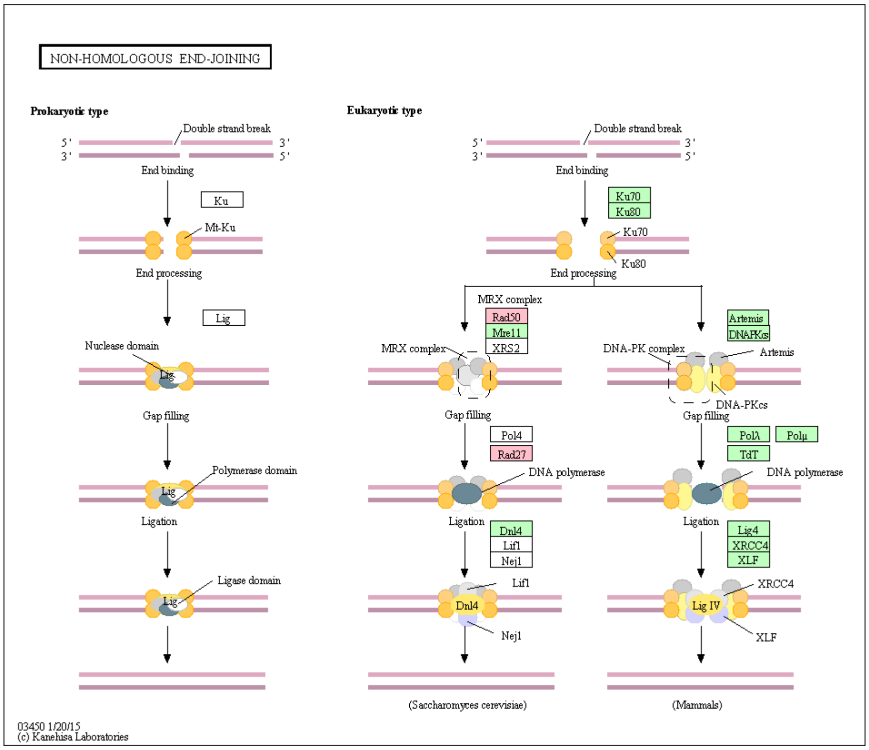

280.3 Legend:

RBH-Blast at 60% Identity + 50% Coverage
Green = Hit in *H. sapiens*
Red = Hit in *H. sapiens* and *T. californica*
White = Not in *H. sapiens*

281 Vitamin digestion and absorption

281.1 Human Pathway: HSA04977

281.2 Number of Hits: 2

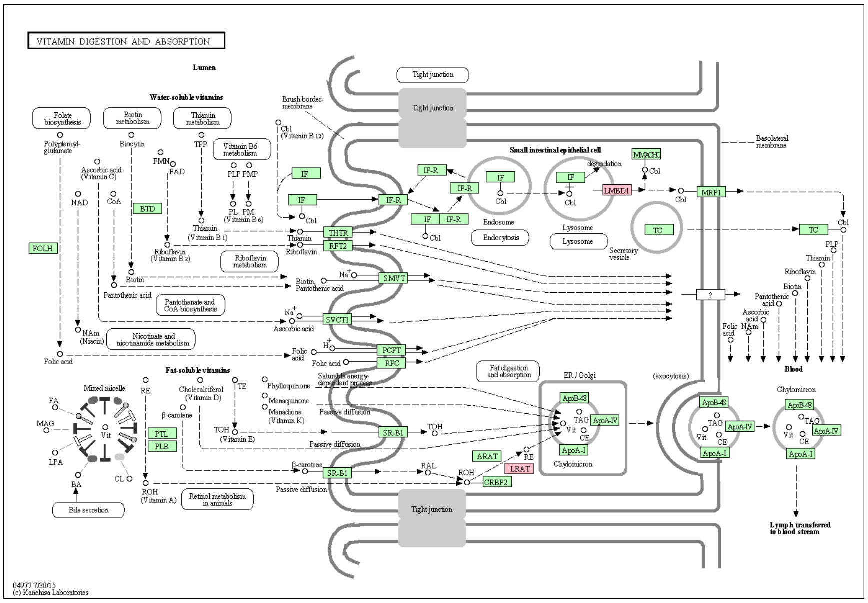

281.3 Legend:

RBH-Blast at 60% Identity + 50% Coverage
Green = Hit in *H. sapiens*
Red = Hit in *H. sapiens* and *T. californica*
White = Not in *H. sapiens*

282 Primary immunodeficiency

282.1 Human Pathway: HSA05340

282.2 Number of Hits: 2

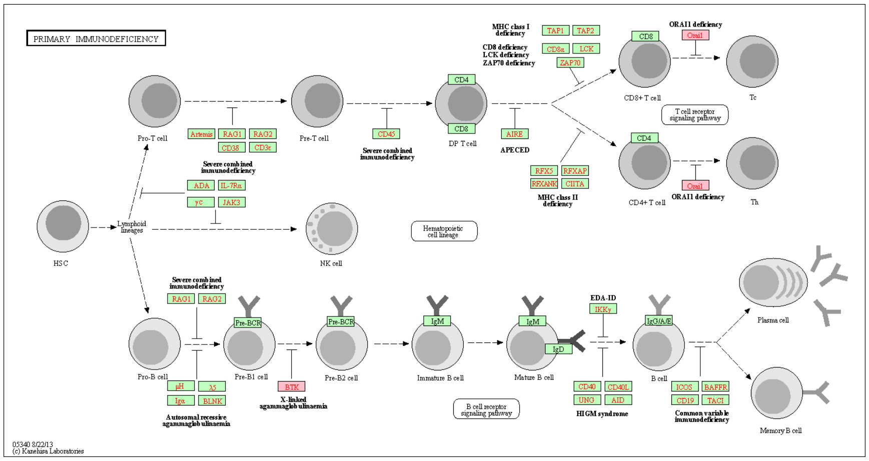

282.3 Legend:

RBH-Blast at 60% Identity + 50% Coverage
Green = Hit in *H. sapiens*
Red = Hit in *H. sapiens* and *T. californica*
White = Not in *H. sapiens*

283 Thiamine metabolism

283.1 Human Pathway: HSA00730

283.2 Number of Hits: 2

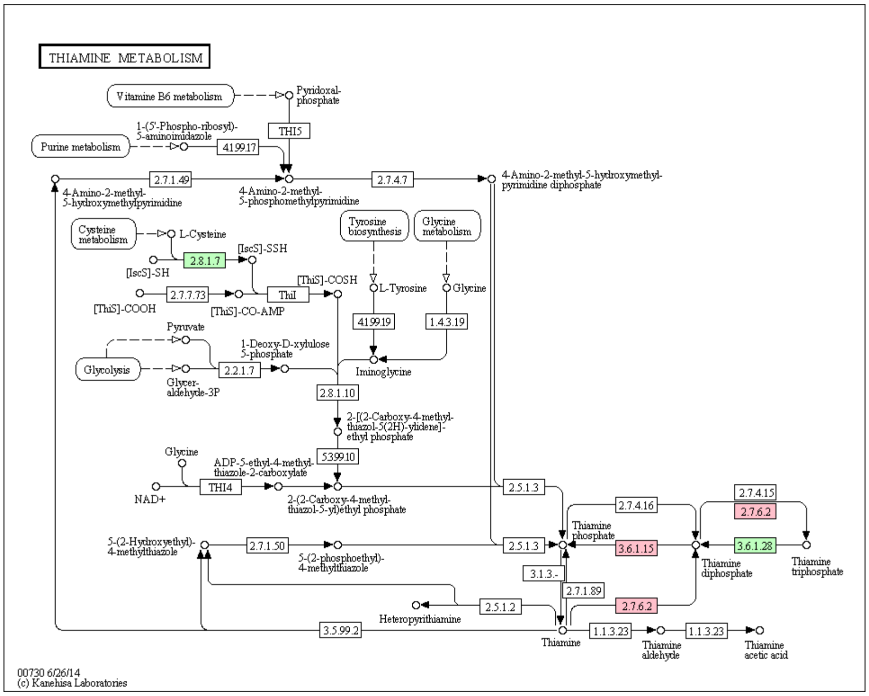

283.3 Legend:

RBH-Blast at 60% Identity + 50% Coverage
Green = Hit in *H. sapiens*
Red = Hit in *H. sapiens* and *T. californica*
White = Not in *H. sapiens*

284 Glycosaminoglycan biosynthesis - chondroitin sulfate / dermatan sulfate

284.1 Human Pathway: HSA00532

284.2 Number of Hits: 2

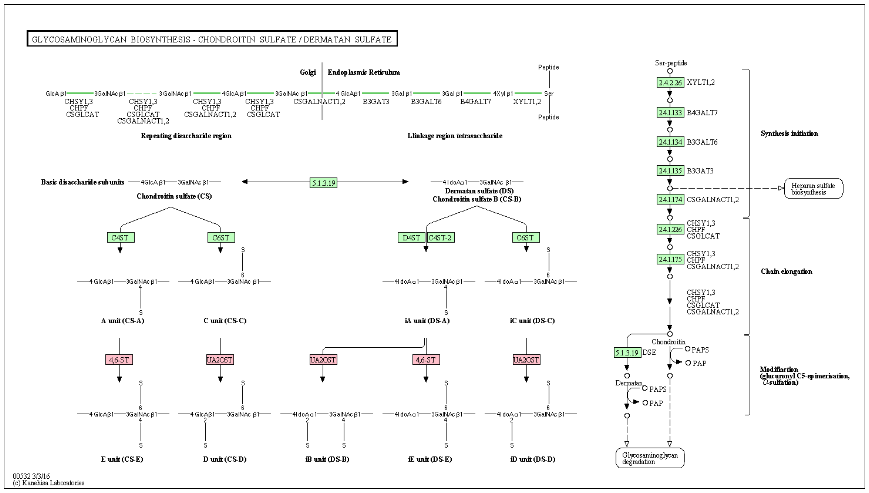

284.3 Legend:

RBH-Blast at 60% Identity + 50% Coverage
Green = Hit in *H. sapiens*
Red = Hit in *H. sapiens* and *T. californica*
White = Not in *H. sapiens*

285 Chemical carcinogenesis

285.1 Human Pathway: HSA05204

285.2 Number of Hits: 2

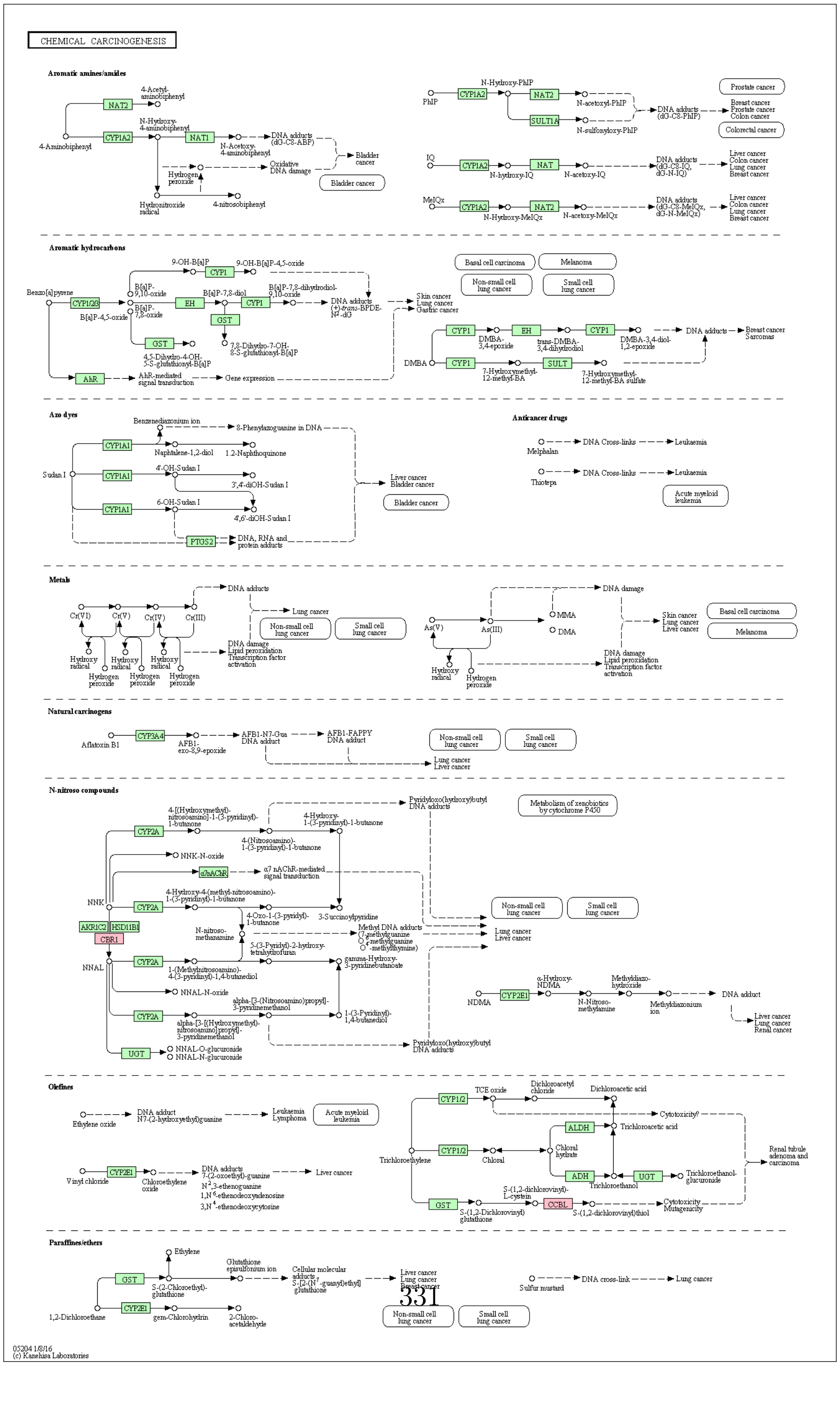

285.3 Legend:

RBH-Blast at 60% Identity + 50% Coverage
Green = Hit in *H. sapiens*
Red = Hit in *H. sapiens* and *T. californica*
White = Not in *H. sapiens*

---

286 D-Glutamine and D-glutamate metabolism

286.1 Human Pathway: HSA00471

286.2 Number of Hits: 2

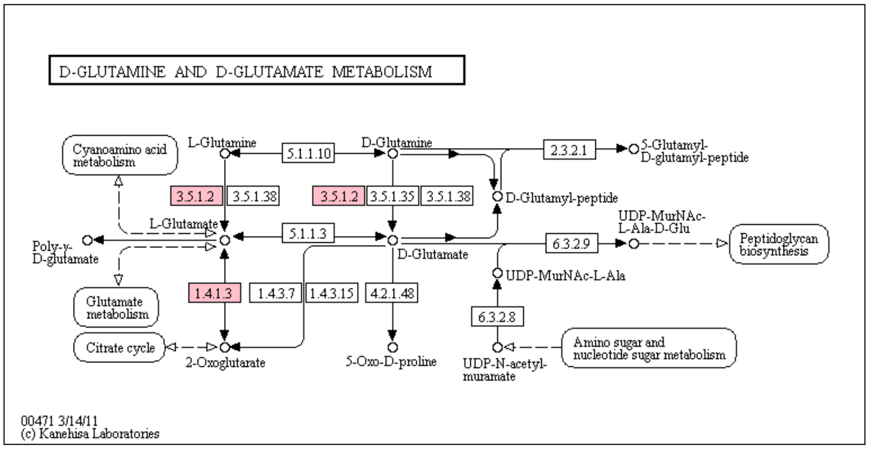

286.3 Legend:

RBH-Blast at 60% Identity + 50% Coverage
Green = Hit in *H. sapiens*
Red = Hit in *H. sapiens* and *T. californica*
White = Not in *H. sapiens*

---

287 Phenylalanine, tyrosine and tryptophan biosynthesis

287.1 Human Pathway: HSA00400

287.2 Number of Hits: 1

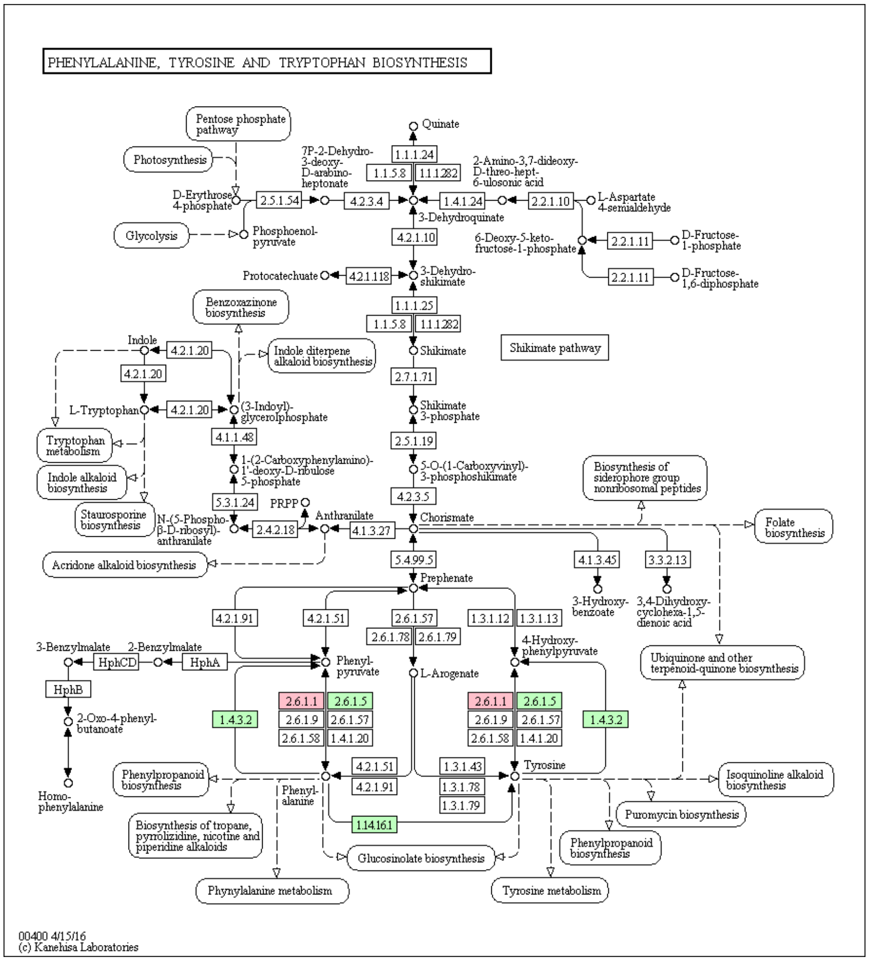

287.3 Legend:

RBH-Blast at 60% Identity + 50% Coverage
Green = Hit in *H. sapiens*
Red = Hit in *H. sapiens* and *T. californica*
White = Not in *H. sapiens*

288 Intestinal immune network for IgA production

288.1 Human Pathway: HSA04672

288.2 Number of Hits: 1

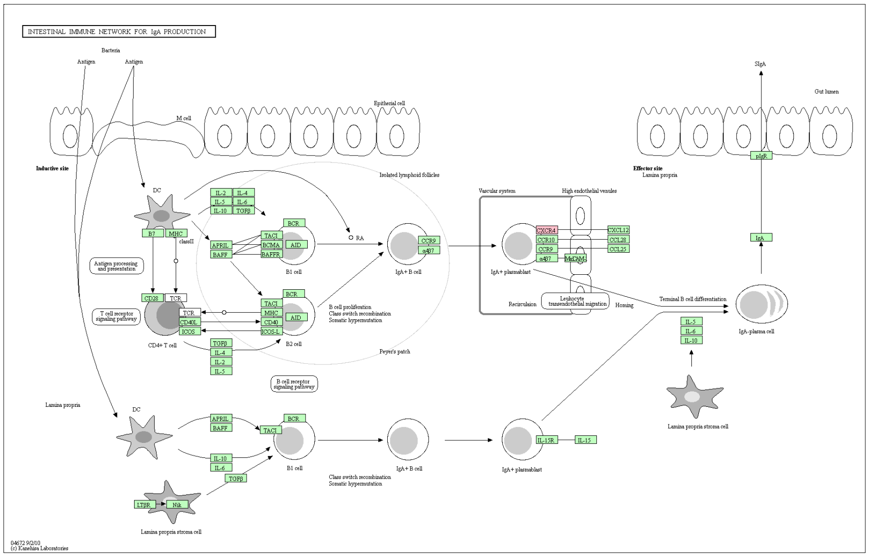

288.3 Legend:

RBH-Blast at 60% Identity + 50% Coverage
Green = Hit in *H. sapiens*
Red = Hit in *H. sapiens* and *T. californica*
White = Not in *H. sapiens*

289 Steroid hormone biosynthesis

289.1 Human Pathway: HSA00140

289.2 Number of Hits: 1

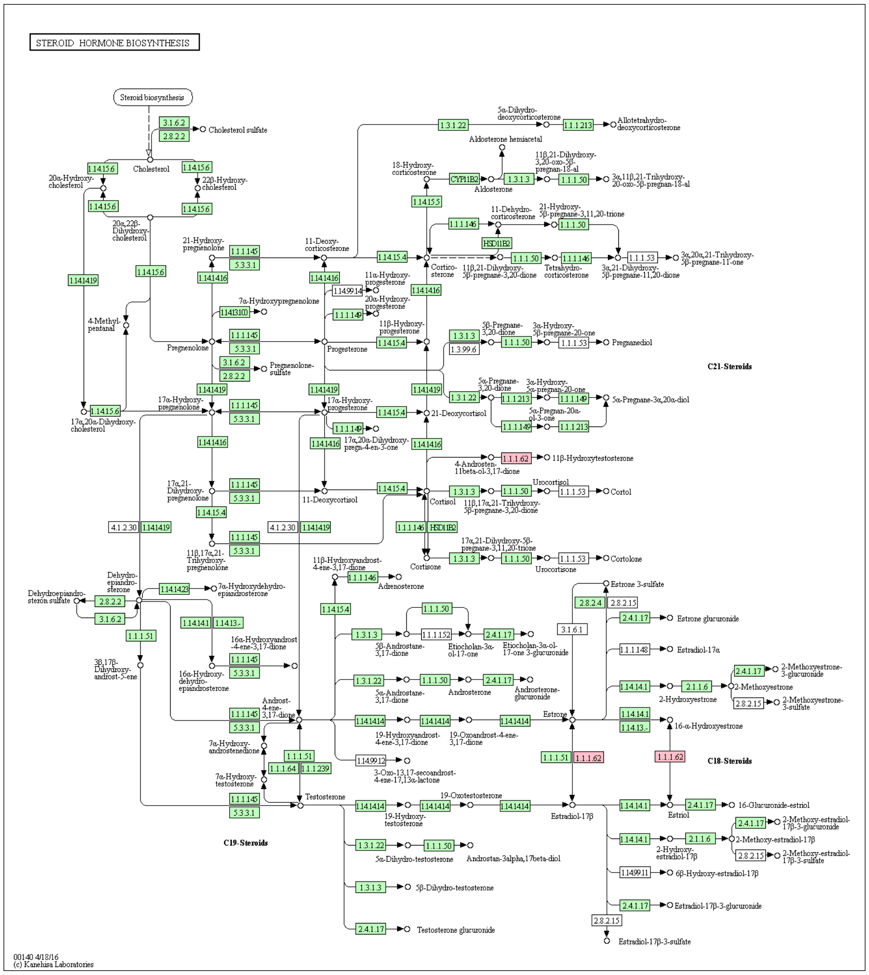

289.3 Legend:

RBH-Blast at 60% Identity + 50% Coverage
Green = Hit in *H. sapiens*
Red = Hit in *H. sapiens* and *T. californica*
White = Not in *H. sapiens*

290 Phenylalanine metabolism

290.1 Human Pathway: HSA00360

290.2 Number of Hits: 1

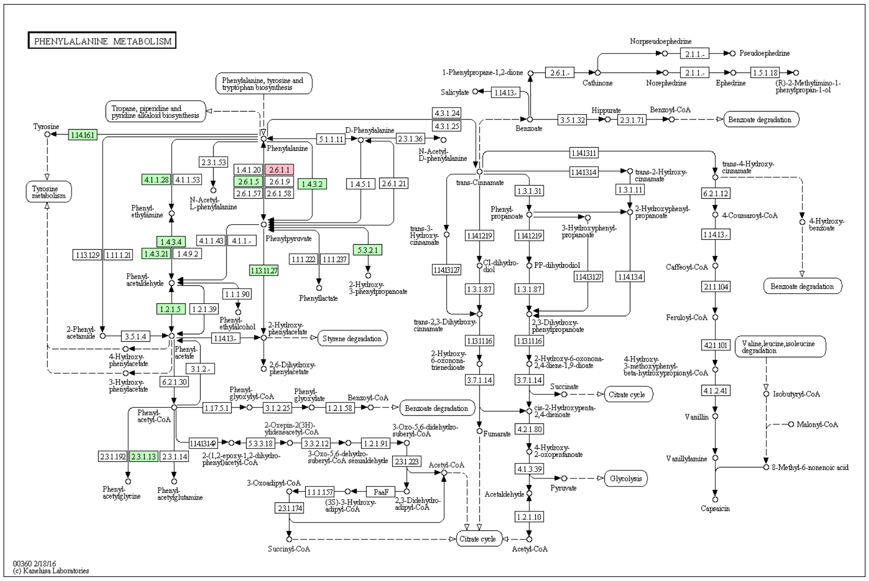

290.3 Legend:

RBH-Blast at 60% Identity + 50% Coverage
Green = Hit in *H. sapiens*
Red = Hit in *H. sapiens* and *T. californica*
White = Not in *H. sapiens*

291 Hematopoietic cell lineage

291.1 Human Pathway: HSA04640

291.2 Number of Hits: 1

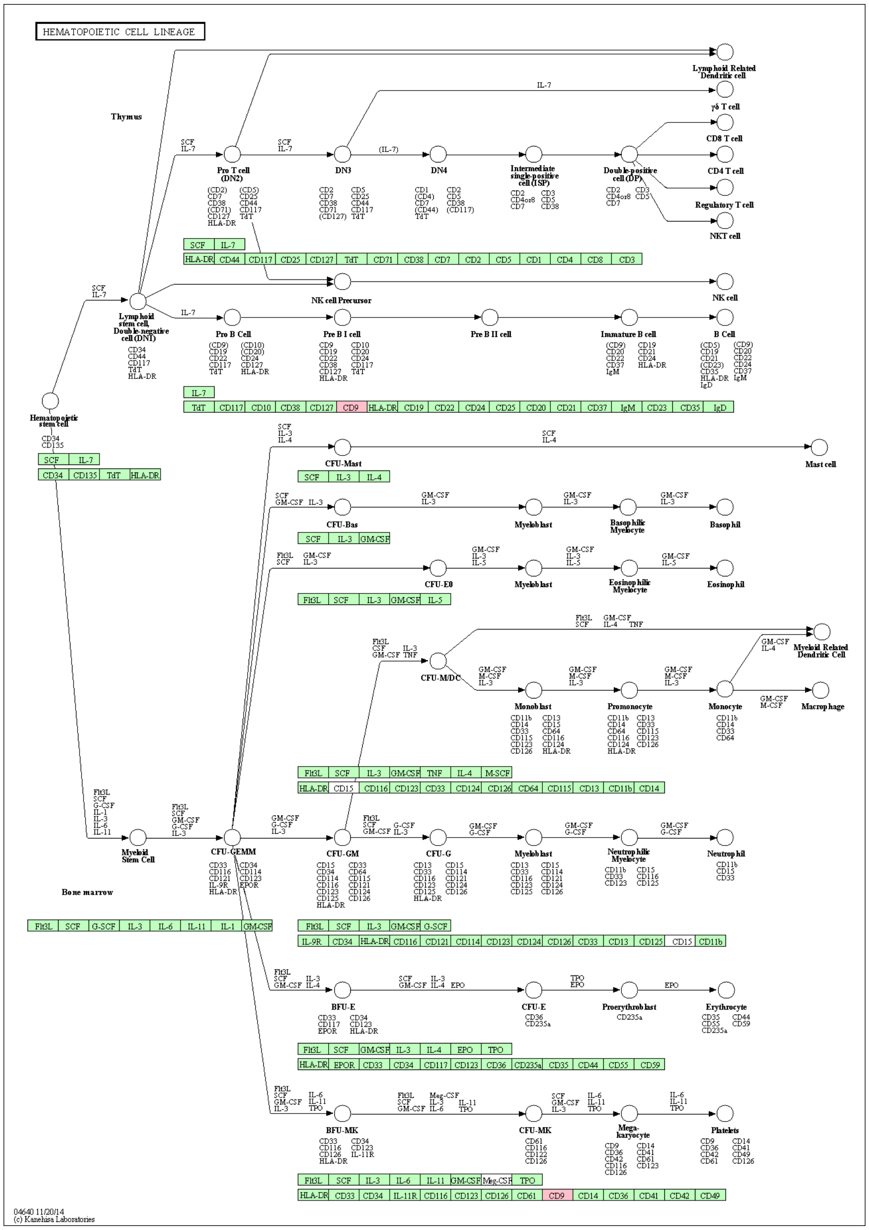

291.3 Legend:

RBH-Blast at 60% Identity + 50% Coverage
Green = Hit in *H. sapiens*
Red = Hit in *H. sapiens* and *T. californica*
White = Not in *H. sapiens*

292 Lipoic acid metabolism

292.1 Human Pathway: HSA00785

292.2 Number of Hits: 1

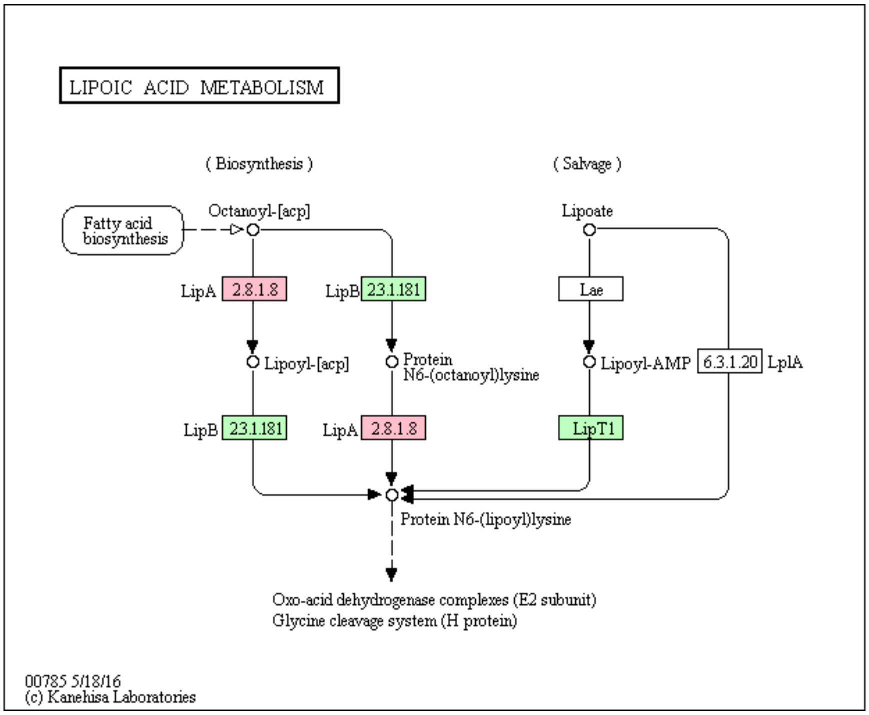

292.3 Legend:

RBH-Blast at 60% Identity + 50% Coverage
Green = Hit in *H. sapiens*
Red = Hit in *H. sapiens* and *T. californica*
White = Not in *H. sapiens*
