# Supplementary material for: Draft de novo transcriptome assembly and proteome characterization of the electric lobe of Tetronarce californica: a molecular tool for the study of cholinergic neurotransmission in the electric organ
Source: BMC Genomics. 2017 Aug 14;18:611. doi: 10.1186/s12864-017-3890-4 (PMC5557070; doi:10.1186/s12864-017-3890-4)
Supplement: Supplementary file 21 — KEGG Analysis. Intersection of H. sapiens With C. milli (set 13). See Additional file 3: TableS07 for details. (PDF 11366 kb) [file 12864_2017_3890_MOESM21_ESM.pdf]

Intersection of *H. sapiens* With *C. milli*

Set13: Reverse-Blast-Hit (RBH)-Blast at 60% Identity + 50% Coverage

Rodolfo Aramayo

April 30, 2017

Contents

1 Pathways in cancer **39**
- 1.1 Human Pathway: HSA05200 39
- 1.2 Number of Hits: 180 39
- 1.3 Legend: 39

2 MAPK signaling pathway **40**
- 2.1 Human Pathway: HSA04010 40
- 2.2 Number of Hits: 118 40
- 2.3 Legend: 40

3 Endocytosis **41**
- 3.1 Human Pathway: HSA04144 41
- 3.2 Number of Hits: 117 41
- 3.3 Legend: 41

4 PI3K-Akt signaling pathway **42**
- 4.1 Human Pathway: HSA04151 42
- 4.2 Number of Hits: 114 42
- 4.3 Legend: 42

5 HTLV-I infection **43**
- 5.1 Human Pathway: HSA05166 43
- 5.2 Number of Hits: 109 43
- 5.3 Legend: 44

6 Ras signaling pathway **44**
6.1 Human Pathway: HSA04014 44
6.2 Number of Hits: 102 44
6.3 Legend: 44

7 Rap1 signaling pathway **45**
7.1 Human Pathway: HSA04015 45
7.2 Number of Hits: 102 45
7.3 Legend: 45

8 Neuroactive ligand-receptor interaction **46**
8.1 Human Pathway: HSA04080 46
8.2 Number of Hits: 100 46
8.3 Legend: 46

9 Regulation of actin cytoskeleton **47**
9.1 Human Pathway: HSA04810 47
9.2 Number of Hits: 98 47
9.3 Legend: 47

10 cAMP signaling pathway **48**
10.1 Human Pathway: HSA04024 48
10.2 Number of Hits: 97 48
10.3 Legend: 48

11 Proteoglycans in cancer **49**
11.1 Human Pathway: HSA05205 49
11.2 Number of Hits: 96 49
11.3 Legend: 49

12 Protein processing in endoplasmic reticulum **50**
12.1 Human Pathway: HSA04141 50
12.2 Number of Hits: 95 50
12.3 Legend: 50

13 mTOR signaling pathway **51**
13.1 Human Pathway: HSA04150 51
13.2 Number of Hits: 94 51
13.3 Legend: 51

14 Purine metabolism **52**
14.1 Human Pathway: HSA00230 52
14.2 Number of Hits: 94 52
14.3 Legend: 52

15 Huntington's disease **53**
15.1 Human Pathway: HSA05016 53
15.2 Number of Hits: 93 53
15.3 Legend: 53

16 Epstein-Barr virus infection **54**
16.1 Human Pathway: HSA05169 54
16.2 Number of Hits: 89 54
16.3 Legend: 54

17 Axon guidance **55**
17.1 Human Pathway: HSA04360 55
17.2 Number of Hits: 86 55
17.3 Legend: 55

18 Viral carcinogenesis **56**
18.1 Human Pathway: HSA05203 56
18.2 Number of Hits: 82 56
18.3 Legend: 56

19 Ubiquitin mediated proteolysis **57**
19.1 Human Pathway: HSA04120 57
19.2 Number of Hits: 80 57
19.3 Legend: 57

20 Ribosome **58**
20.1 Human Pathway: HSA03010 58
20.2 Number of Hits: 80 58
20.3 Legend: 58

21 Focal adhesion **59**
21.1 Human Pathway: HSA04510 59
21.2 Number of Hits: 79 59
21.3 Legend: 59

22 Spliceosome **60**
22.1 Human Pathway: HSA03040 60
22.2 Number of Hits: 78 60
22.3 Legend: 60

23 Alzheimer's disease **61**
23.1 Human Pathway: HSA05010 61
23.2 Number of Hits: 77 61
23.3 Legend: 61

24 Oxytocin signaling pathway **62**
24.1 Human Pathway: HSA04921 62
24.2 Number of Hits: 76 62
24.3 Legend: 62

25 Wnt signaling pathway **63**
25.1 Human Pathway: HSA04310 63
25.2 Number of Hits: 73 63
25.3 Legend: 63

26 Calcium signaling pathway **64**
26.1 Human Pathway: HSA04020 64
26.2 Number of Hits: 72 64
26.3 Legend: 64

27 Chemokine signaling pathway **65**
27.1 Human Pathway: HSA04062 65
27.2 Number of Hits: 72 65
27.3 Legend: 65

28 Signaling pathways regulating pluripotency of stem cells **66**
28.1 Human Pathway: HSA04550 66
28.2 Number of Hits: 72 66
28.3 Legend: 66

29 FoxO signaling pathway **67**
29.1 Human Pathway: HSA04068 67
29.2 Number of Hits: 72 67
29.3 Legend: 67

30 RNA transport **68**
30.1 Human Pathway: HSA03013 68
30.2 Number of Hits: 71 68
30.3 Legend: 68

31 Hippo signaling pathway **69**
31.1 Human Pathway: HSA04390 69
31.2 Number of Hits: 71 69
31.3 Legend: 69

32 Sphingolipid signaling pathway **70**
32.1 Human Pathway: HSA04071 70
32.2 Number of Hits: 70 70
32.3 Legend: 70

33 Adrenergic signaling in cardiomyocytes **71**
33.1 Human Pathway: HSA04261 71
33.2 Number of Hits: 70 71
33.3 Legend: 71

34 cGMP-PKG signaling pathway **72**
34.1 Human Pathway: HSA04022 72
34.2 Number of Hits: 70 72
34.3 Legend: 72

35 Phospholipase D signaling pathway **73**
35.1 Human Pathway: HSA04072 73
35.2 Number of Hits: 69 73
35.3 Legend: 73

36 Dopaminergic synapse **74**
36.1 Human Pathway: HSA04728 74
36.2 Number of Hits: 69 74
36.3 Legend: 74

37 MicroRNAs in cancer **75**
37.1 Human Pathway: HSA05206 75
37.2 Number of Hits: 67 75
37.3 Legend: 75

38 Insulin signaling pathway **76**
38.1 Human Pathway: HSA04910 76
38.2 Number of Hits: 66 76
38.3 Legend: 76

39 Neurotrophin signaling pathway **77**
39.1 Human Pathway: HSA04722 77
39.2 Number of Hits: 65 77
39.3 Legend: 77

40 Parkinson's disease **78**
40.1 Human Pathway: HSA05012 78
40.2 Number of Hits: 64 78
40.3 Legend: 78

41 Retrograde endocannabinoid signaling **79**
41.1 Human Pathway: HSA04723 79
41.2 Number of Hits: 64 79
41.3 Legend: 79

42 AMPK signaling pathway **80**
42.1 Human Pathway: HSA04152 80
42.2 Number of Hits: 64 80
42.3 Legend: 80

43 Thyroid hormone signaling pathway **81**
43.1 Human Pathway: HSA04919 81
43.2 Number of Hits: 64 81
43.3 Legend: 81

44 Cell cycle **82**
44.1 Human Pathway: HSA04110 82
44.2 Number of Hits: 63 82
44.3 Legend: 82

45 Carbon metabolism **83**
45.1 Human Pathway: HSA01200 83
45.2 Number of Hits: 62 83
45.3 Legend: 83

46 Glutamatergic synapse **84**
46.1 Human Pathway: HSA04724 84
46.2 Number of Hits: 62 84
46.3 Legend: 84

47 Non-alcoholic fatty liver disease (NAFLD) **85**
47.1 Human Pathway: HSA04932 85
47.2 Number of Hits: 61 85
47.3 Legend: 85

48 Oxidative phosphorylation **86**
48.1 Human Pathway: HSA00190 86
48.2 Number of Hits: 60 86
48.3 Legend: 86

49 Tight junction **87**
49.1 Human Pathway: HSA04530 87
49.2 Number of Hits: 60 87
49.3 Legend: 87

50 Alcoholism **88**
50.1 Human Pathway: HSA05034 88
50.2 Number of Hits: 57 88
50.3 Legend: 88

51 Pyrimidine metabolism **89**
51.1 Human Pathway: HSA00240 89
51.2 Number of Hits: 56 89
51.3 Legend: 89

52 Cholinergic synapse **90**
52.1 Human Pathway: HSA04725 90
52.2 Number of Hits: 56 90
52.3 Legend: 90

53 Platelet activation **91**
53.1 Human Pathway: HSA04611 91
53.2 Number of Hits: 55 91
53.3 Legend: 91

54 Serotonergic synapse **92**
54.1 Human Pathway: HSA04726 92
54.2 Number of Hits: 54 92
54.3 Legend: 92

55 Oocyte meiosis **93**
55.1 Human Pathway: HSA04114 93
55.2 Number of Hits: 53 93
55.3 Legend: 93

56 Leukocyte transendothelial migration **94**
56.1 Human Pathway: HSA04670 94
56.2 Number of Hits: 53 94
56.3 Legend: 94

57 Progesterone-mediated oocyte maturation **95**
57.1 Human Pathway: HSA04914 95
57.2 Number of Hits: 52 95
57.3 Legend: 95

58 Hepatitis C **96**
58.1 Human Pathway: HSA05160 96
58.2 Number of Hits: 52 96
58.3 Legend: 96

59 Apoptosis **97**
59.1 Human Pathway: HSA04210 97
59.2 Number of Hits: 52 97
59.3 Legend: 97

60 Circadian entrainment **98**
60.1 Human Pathway: HSA04713 98
60.2 Number of Hits: 51 98
60.3 Legend: 98

61 Lysosome **99**
61.1 Human Pathway: HSA04142 99
61.2 Number of Hits: 51 99
61.3 Legend: 99

62 Melanogenesis **100**
62.1 Human Pathway: HSA04916 100
62.2 Number of Hits: 51 100
62.3 Legend: 100

63 Hepatitis B **101**
63.1 Human Pathway: HSA05161 101
63.2 Number of Hits: 51 101
63.3 Legend: 101

64 Phagosome **102**
64.1 Human Pathway: HSA04145 102
64.2 Number of Hits: 51 102
64.3 Legend: 102

65 Gap junction **103**
65.1 Human Pathway: HSA04540 103
65.2 Number of Hits: 51 103
65.3 Legend: 103

66 Phosphatidylinositol signaling system **104**
66.1 Human Pathway: HSA04070 104
66.2 Number of Hits: 50 104
66.3 Legend: 104

67 Estrogen signaling pathway **105**
67.1 Human Pathway: HSA04915 105
67.2 Number of Hits: 50 105
67.3 Legend: 105

68 Morphine addiction **106**
68.1 Human Pathway: HSA05032 106
68.2 Number of Hits: 50 106
68.3 Legend: 106

69 Choline metabolism in cancer **107**
69.1 Human Pathway: HSA05231 107
69.2 Number of Hits: 50 107
69.3 Legend: 107

70 Glucagon signaling pathway **108**
70.1 Human Pathway: HSA04922 108
70.2 Number of Hits: 49 108
70.3 Legend: 108

71 Transcriptional misregulation in cancer **109**
71.1 Human Pathway: HSA05202 109
71.2 Number of Hits: 48 109
71.3 Legend: 109

72 Fc gamma R-mediated phagocytosis **110**
72.1 Human Pathway: HSA04666 110
72.2 Number of Hits: 48 110
72.3 Legend: 110

73 GABAergic synapse **111**
73.1 Human Pathway: HSA04727 111
73.2 Number of Hits: 48 111
73.3 Legend: 111

74 T cell receptor signaling pathway **112**
74.1 Human Pathway: HSA04660 112
74.2 Number of Hits: 48 112
74.3 Legend: 112

75 Tuberculosis **113**
75.1 Human Pathway: HSA05152 113
75.2 Number of Hits: 47 113
75.3 Legend: 113

76 AGE-RAGE signaling pathway in diabetic complications **114**
76.1 Human Pathway: HSA04933 114
76.2 Number of Hits: 47 114
76.3 Legend: 114

77 GnRH signaling pathway **115**
77.1 Human Pathway: HSA04912 115
77.2 Number of Hits: 47 115
77.3 Legend: 115

78 Vascular smooth muscle contraction **116**
78.1 Human Pathway: HSA04270 116
78.2 Number of Hits: 46 116
78.3 Legend: 116

79 Influenza A **117**
79.1 Human Pathway: HSA05164 117
79.2 Number of Hits: 46 117
79.3 Legend: 117

80 Insulin resistance **118**
80.1 Human Pathway: HSA04931 118
80.2 Number of Hits: 46 118
80.3 Legend: 118

81 mRNA surveillance pathway **119**
81.1 Human Pathway: HSA03015 119
81.2 Number of Hits: 46 119
81.3 Legend: 119

82 Herpes simplex infection **120**
82.1 Human Pathway: HSA05168 120
82.2 Number of Hits: 45 120
82.3 Legend: 120

83 Peroxisome **121**
83.1 Human Pathway: HSA04146 121
83.2 Number of Hits: 45 121
83.3 Legend: 121

84 RNA degradation **122**
84.1 Human Pathway: HSA03018 122
84.2 Number of Hits: 45 122
84.3 Legend: 122

85 Longevity regulating pathway **123**
85.1 Human Pathway: HSA04211 123
85.2 Number of Hits: 44 123
85.3 Legend: 123

86 Osteoclast differentiation **124**
86.1 Human Pathway: HSA04380 124
86.2 Number of Hits: 43 124
86.3 Legend: 124

87 HIF-1 signaling pathway **125**
87.1 Human Pathway: HSA04066 125
87.2 Number of Hits: 42 125
87.3 Legend: 125

88 Adherens junction **126**
88.1 Human Pathway: HSA04520 126
88.2 Number of Hits: 42 126
88.3 Legend: 126

89 Long-term depression **127**
89.1 Human Pathway: HSA04730 127
89.2 Number of Hits: 41 127
89.3 Legend: 127

90 Synaptic vesicle cycle **128**
90.1 Human Pathway: HSA04721 128
90.2 Number of Hits: 41 128
90.3 Legend: 128

91 Prolactin signaling pathway **129**
91.1 Human Pathway: HSA04917 129
91.2 Number of Hits: 41 129
91.3 Legend: 129

92 Glycerophospholipid metabolism **130**
92.1 Human Pathway: HSA00564 130
92.2 Number of Hits: 41 130
92.3 Legend: 130

93 Inflammatory mediator regulation of TRP channels **131**
93.1 Human Pathway: HSA04750 131
93.2 Number of Hits: 40 131
93.3 Legend: 131

94 Long-term potentiation **132**
94.1 Human Pathway: HSA04720 132
94.2 Number of Hits: 40 132
94.3 Legend: 132

95 Chagas disease (American trypanosomiasis) **133**
95.1 Human Pathway: HSA05142 133
95.2 Number of Hits: 40 133
95.3 Legend: 133

96 Prostate cancer **134**
96.1 Human Pathway: HSA05215 134
96.2 Number of Hits: 40 134
96.3 Legend: 134

97 ErbB signaling pathway **135**
97.1 Human Pathway: HSA04012 135
97.2 Number of Hits: 40 135
97.3 Legend: 135

98 Biosynthesis of amino acids **136**
98.1 Human Pathway: HSA01230 136
98.2 Number of Hits: 40 136
98.3 Legend: 136

99 Ribosome biogenesis in eukaryotes **137**
99.1 Human Pathway: HSA03008 137
99.2 Number of Hits: 39 137
99.3 Legend: 138

100 Bacterial invasion of epithelial cells **138**
100.1 Human Pathway: HSA05100 138
100.2 Number of Hits: 39 138
100.3 Legend: 138

101 Toxoplasmosis **139**
101.1 Human Pathway: HSA05145 139
101.2 Number of Hits: 38 139
101.3 Legend: 139

102 Epithelial cell signaling in Helicobacter pylori infection 140
102.1 Human Pathway: HSA05120 140
102.2 Number of Hits: 38 140
102.3 Legend: 140

103 Inositol phosphate metabolism 141
103.1 Human Pathway: HSA00562 141
103.2 Number of Hits: 38 141
103.3 Legend: 141

104 Renal cell carcinoma 142
104.1 Human Pathway: HSA05211 142
104.2 Number of Hits: 38 142
104.3 Legend: 142

105 EGFR tyrosine kinase inhibitor resistance 143
105.1 Human Pathway: HSA01521 143
105.2 Number of Hits: 37 143
105.3 Legend: 143

106 Dilated cardiomyopathy 144
106.1 Human Pathway: HSA05414 144
106.2 Number of Hits: 36 144
106.3 Legend: 144

107 Chronic myeloid leukemia 145
107.1 Human Pathway: HSA05220 145
107.2 Number of Hits: 36 145
107.3 Legend: 145

108 Measles 146
108.1 Human Pathway: HSA05162 146
108.2 Number of Hits: 36 146
108.3 Legend: 147

109 Pancreatic secretion 147
109.1 Human Pathway: HSA04972 147
109.2 Number of Hits: 35 147
109.3 Legend: 148

110 Pancreatic cancer **148**
110.1 Human Pathway: HSA05212 148
110.2 Number of Hits: 35 148
110.3 Legend: 148

111 Salmonella infection **149**
111.1 Human Pathway: HSA05132 149
111.2 Number of Hits: 35 149
111.3 Legend: 149

112 Shigellosis **150**
112.1 Human Pathway: HSA05131 150
112.2 Number of Hits: 34 150
112.3 Legend: 150

113 TGF-beta signaling pathway **151**
113.1 Human Pathway: HSA04350 151
113.2 Number of Hits: 34 151
113.3 Legend: 151

114 Longevity regulating pathway - multiple species **152**
114.1 Human Pathway: HSA04213 152
114.2 Number of Hits: 34 152
114.3 Legend: 152

115 B cell receptor signaling pathway **153**
115.1 Human Pathway: HSA04662 153
115.2 Number of Hits: 34 153
115.3 Legend: 153

116 Fc epsilon RI signaling pathway **154**
116.1 Human Pathway: HSA04664 154
116.2 Number of Hits: 34 154
116.3 Legend: 154

117 Hypertrophic cardiomyopathy (HCM) **155**
117.1 Human Pathway: HSA05410 155
117.2 Number of Hits: 34 155
117.3 Legend: 155

118 Fatty acid metabolism **156**
118.1 Human Pathway: HSA01212 156
118.2 Number of Hits: 33 156
118.3 Legend: 157

119 Melanoma **157**
119.1 Human Pathway: HSA05218 157
119.2 Number of Hits: 33 157
119.3 Legend: 157

120 Thyroid hormone synthesis **158**
120.1 Human Pathway: HSA04918 158
120.2 Number of Hits: 32 158
120.3 Legend: 158

121 Central carbon metabolism in cancer **159**
121.1 Human Pathway: HSA05230 159
121.2 Number of Hits: 32 159
121.3 Legend: 159

122 N-Glycan biosynthesis **160**
122.1 Human Pathway: HSA00510 160
122.2 Number of Hits: 32 160
122.3 Legend: 161

123 Aminoacyl-tRNA biosynthesis **162**
123.1 Human Pathway: HSA00970 162
123.2 Number of Hits: 32 162
123.3 Legend: 162

124 Arrhythmogenic right ventricular cardiomyopathy (ARVC) **163**
124.1 Human Pathway: HSA05412 163
124.2 Number of Hits: 32 163
124.3 Legend: 163

125 TNF signaling pathway **164**
125.1 Human Pathway: HSA04668 164
125.2 Number of Hits: 32 164
125.3 Legend: 164

126 Vibrio cholerae infection **165**
126.1 Human Pathway: HSA05110 165
126.2 Number of Hits: 31 165
126.3 Legend: 165

127 Basal cell carcinoma **166**
127.1 Human Pathway: HSA05217 166
127.2 Number of Hits: 31 166
127.3 Legend: 166

128 Colorectal cancer **167**
128.1 Human Pathway: HSA05210 167
128.2 Number of Hits: 31 167
128.3 Legend: 167

129 Natural killer cell mediated cytotoxicity **168**
129.1 Human Pathway: HSA04650 168
129.2 Number of Hits: 31 168
129.3 Legend: 168

130 Gastric acid secretion **169**
130.1 Human Pathway: HSA04971 169
130.2 Number of Hits: 31 169
130.3 Legend: 169

131 Adipocytokine signaling pathway **170**
131.1 Human Pathway: HSA04920 170
131.2 Number of Hits: 31 170
131.3 Legend: 170

132 Acute myeloid leukemia **171**
132.1 Human Pathway: HSA05221 171
132.2 Number of Hits: 31 171
132.3 Legend: 171

133 Salivary secretion **172**
133.1 Human Pathway: HSA04970 172
133.2 Number of Hits: 30 172
133.3 Legend: 172

134 Jak-STAT signaling pathway 173
134.1 Human Pathway: HSA04630 173
134.2 Number of Hits: 30 173
134.3 Legend: 173

135 Glioma 174
135.1 Human Pathway: HSA05214 174
135.2 Number of Hits: 30 174
135.3 Legend: 174

136 Insulin secretion 175
136.1 Human Pathway: HSA04911 175
136.2 Number of Hits: 30 175
136.3 Legend: 175

137 Cardiac muscle contraction 176
137.1 Human Pathway: HSA04260 176
137.2 Number of Hits: 30 176
137.3 Legend: 176

138 Renin secretion 177
138.1 Human Pathway: HSA04924 177
138.2 Number of Hits: 29 177
138.3 Legend: 177

139 Endometrial cancer 178
139.1 Human Pathway: HSA05213 178
139.2 Number of Hits: 29 178
139.3 Legend: 178

140 Glycolysis / Gluconeogenesis 179
140.1 Human Pathway: HSA00010 179
140.2 Number of Hits: 29 179
140.3 Legend: 180

141 VEGF signaling pathway 180
141.1 Human Pathway: HSA04370 180
141.2 Number of Hits: 29 180
141.3 Legend: 180

142 Non-small cell lung cancer **181**
142.1 Human Pathway: HSA05223 181
142.2 Number of Hits: 29 181
142.3 Legend: 181

143 Small cell lung cancer **182**
143.1 Human Pathway: HSA05222 182
143.2 Number of Hits: 29 182
143.3 Legend: 182

144 Nicotine addiction **183**
144.1 Human Pathway: HSA05033 183
144.2 Number of Hits: 28 183
144.3 Legend: 183

145 Toll-like receptor signaling pathway **184**
145.1 Human Pathway: HSA04620 184
145.2 Number of Hits: 28 184
145.3 Legend: 184

146 Arginine and proline metabolism **185**
146.1 Human Pathway: HSA00330 185
146.2 Number of Hits: 28 185
146.3 Legend: 185

147 Aldosterone synthesis and secretion **186**
147.1 Human Pathway: HSA04925 186
147.2 Number of Hits: 28 186
147.3 Legend: 186

148 Basal transcription factors **187**
148.1 Human Pathway: HSA03022 187
148.2 Number of Hits: 28 187
148.3 Legend: 187

149 Valine, leucine and isoleucine degradation **188**
149.1 Human Pathway: HSA00280 188
149.2 Number of Hits: 27 188
149.3 Legend: 189

150 Nucleotide excision repair **189**
150.1 Human Pathway: HSA03420 189
150.2 Number of Hits: 27 189
150.3 Legend: 189

151 p53 signaling pathway **190**
151.1 Human Pathway: HSA04115 190
151.2 Number of Hits: 27 190
151.3 Legend: 190

152 Amphetamine addiction **191**
152.1 Human Pathway: HSA05031 191
152.2 Number of Hits: 27 191
152.3 Legend: 191

153 Sphingolipid metabolism **192**
153.1 Human Pathway: HSA00600 192
153.2 Number of Hits: 27 192
153.3 Legend: 192

154 Amino sugar and nucleotide sugar metabolism **193**
154.1 Human Pathway: HSA00520 193
154.2 Number of Hits: 27 193
154.3 Legend: 194

155 Proteasome **194**
155.1 Human Pathway: HSA03050 194
155.2 Number of Hits: 27 194
155.3 Legend: 194

156 Amoebiasis **195**
156.1 Human Pathway: HSA05146 195
156.2 Number of Hits: 26 195
156.3 Legend: 195

157 Cysteine and methionine metabolism **196**
157.1 Human Pathway: HSA00270 196
157.2 Number of Hits: 26 196
157.3 Legend: 196

158 Glycerolipid metabolism **197**
158.1 Human Pathway: HSA00561 197
158.2 Number of Hits: 26 197
158.3 Legend: 197

159 Regulation of lipolysis in adipocytes **198**
159.1 Human Pathway: HSA04923 198
159.2 Number of Hits: 26 198
159.3 Legend: 198

160 Cell adhesion molecules (CAMs) **199**
160.1 Human Pathway: HSA04514 199
160.2 Number of Hits: 26 199
160.3 Legend: 199

161 Taste transduction **200**
161.1 Human Pathway: HSA04742 200
161.2 Number of Hits: 25 200
161.3 Legend: 200

162 PPAR signaling pathway **201**
162.1 Human Pathway: HSA03320 201
162.2 Number of Hits: 25 201
162.3 Legend: 201

163 Amyotrophic lateral sclerosis (ALS) **202**
163.1 Human Pathway: HSA05014 202
163.2 Number of Hits: 25 202
163.3 Legend: 202

164 Type II diabetes mellitus **203**
164.1 Human Pathway: HSA04930 203
164.2 Number of Hits: 25 203
164.3 Legend: 203

165 Bile secretion **204**
165.1 Human Pathway: HSA04976 204
165.2 Number of Hits: 24 204
165.3 Legend: 204

166 Pyruvate metabolism **205**
166.1 Human Pathway: HSA00620 205
166.2 Number of Hits: 24 205
166.3 Legend: 205

167 Notch signaling pathway **206**
167.1 Human Pathway: HSA04330 206
167.2 Number of Hits: 24 206
167.3 Legend: 206

168 Cocaine addiction **207**
168.1 Human Pathway: HSA05030 207
168.2 Number of Hits: 23 207
168.3 Legend: 207

169 Pathogenic Escherichia coli infection **208**
169.1 Human Pathway: HSA05130 208
169.2 Number of Hits: 23 208
169.3 Legend: 208

170 Glycine, serine and threonine metabolism **209**
170.1 Human Pathway: HSA00260 209
170.2 Number of Hits: 23 209
170.3 Legend: 209

171 Protein digestion and absorption **210**
171.1 Human Pathway: HSA04974 210
171.2 Number of Hits: 22 210
171.3 Legend: 210

172 NF-kappa B signaling pathway **211**
172.1 Human Pathway: HSA04064 211
172.2 Number of Hits: 22 211
172.3 Legend: 211

173 Alanine, aspartate and glutamate metabolism **212**
173.1 Human Pathway: HSA00250 212
173.2 Number of Hits: 20 212
173.3 Legend: 212

174 Hedgehog signaling pathway **213**
174.1 Human Pathway: HSA04340 213
174.2 Number of Hits: 20 213
174.3 Legend: 213

175 Glyoxylate and dicarboxylate metabolism **214**
175.1 Human Pathway: HSA00630 214
175.2 Number of Hits: 20 214
175.3 Legend: 214

176 Propanoate metabolism **215**
176.1 Human Pathway: HSA00640 215
176.2 Number of Hits: 20 215
176.3 Legend: 215

177 SNARE interactions in vesicular transport **216**
177.1 Human Pathway: HSA04130 216
177.2 Number of Hits: 20 216
177.3 Legend: 216

178 Dorso-ventral axis formation **217**
178.1 Human Pathway: HSA04320 217
178.2 Number of Hits: 20 217
178.3 Legend: 217

179 beta-Alanine metabolism **218**
179.1 Human Pathway: HSA00410 218
179.2 Number of Hits: 20 218
179.3 Legend: 218

180 Lysine degradation **219**
180.1 Human Pathway: HSA00310 219
180.2 Number of Hits: 20 219
180.3 Legend: 219

181 Pertussis **220**
181.1 Human Pathway: HSA05133 220
181.2 Number of Hits: 20 220
181.3 Legend: 220

182 Platinum drug resistance **221**
182.1 Human Pathway: HSA01524 221
182.2 Number of Hits: 20 221
182.3 Legend: 221

183 Fatty acid degradation **222**
183.1 Human Pathway: HSA00071 222
183.2 Number of Hits: 19 222
183.3 Legend: 222

184 DNA replication **223**
184.1 Human Pathway: HSA03030 223
184.2 Number of Hits: 19 223
184.3 Legend: 223

185 Citrate cycle (TCA cycle) **224**
185.1 Human Pathway: HSA00020 224
185.2 Number of Hits: 19 224
185.3 Legend: 224

186 Vasopressin-regulated water reabsorption **225**
186.1 Human Pathway: HSA04962 225
186.2 Number of Hits: 19 225
186.3 Legend: 225

187 NOD-like receptor signaling pathway **226**
187.1 Human Pathway: HSA04621 226
187.2 Number of Hits: 19 226
187.3 Legend: 226

188 Rheumatoid arthritis **227**
188.1 Human Pathway: HSA05323 227
188.2 Number of Hits: 19 227
188.3 Legend: 227

189 Mucin type O-Glycan biosynthesis **229**
189.1 Human Pathway: HSA00512 229
189.2 Number of Hits: 19 229
189.3 Legend: 230

190 Protein export **230**
190.1 Human Pathway: HSA03060 230
190.2 Number of Hits: 18 230
190.3 Legend: 230

191 Other types of O-glycan biosynthesis **231**
191.1 Human Pathway: HSA00514 231
191.2 Number of Hits: 18 231
191.3 Legend: 231

192 Endocrine and other factor-regulated calcium reabsorption **232**
192.1 Human Pathway: HSA04961 232
192.2 Number of Hits: 18 232
192.3 Legend: 232

193 Bladder cancer **233**
193.1 Human Pathway: HSA05219 233
193.2 Number of Hits: 18 233
193.3 Legend: 233

194 Leishmaniasis **234**
194.1 Human Pathway: HSA05140 234
194.2 Number of Hits: 18 234
194.3 Legend: 234

195 Tryptophan metabolism **235**
195.1 Human Pathway: HSA00380 235
195.2 Number of Hits: 18 235
195.3 Legend: 235

196 RNA polymerase **236**
196.1 Human Pathway: HSA03020 236
196.2 Number of Hits: 18 236
196.3 Legend: 236

197 Steroid biosynthesis **237**
197.1 Human Pathway: HSA00100 237
197.2 Number of Hits: 17 237
197.3 Legend: 237

198 ABC transporters **238**
198.1 Human Pathway: HSA02010 238
198.2 Number of Hits: 17 238
198.3 Legend: 239

199 Olfactory transduction **239**
199.1 Human Pathway: HSA04740 239
199.2 Number of Hits: 17 239
199.3 Legend: 239

200 RIG-I-like receptor signaling pathway **240**
200.1 Human Pathway: HSA04622 240
200.2 Number of Hits: 17 240
200.3 Legend: 240

201 Fatty acid elongation **241**
201.1 Human Pathway: HSA00062 241
201.2 Number of Hits: 17 241
201.3 Legend: 242

202 Glutathione metabolism **242**
202.1 Human Pathway: HSA00480 242
202.2 Number of Hits: 17 242
202.3 Legend: 243

203 Aldosterone-regulated sodium reabsorption **243**
203.1 Human Pathway: HSA04960 243
203.2 Number of Hits: 16 243
203.3 Legend: 244

204 Systemic lupus erythematosus **244**
204.1 Human Pathway: HSA05322 244
204.2 Number of Hits: 16 244
204.3 Legend: 244

205 Carbohydrate digestion and absorption **245**
205.1 Human Pathway: HSA04973 245
205.2 Number of Hits: 16 245
205.3 Legend: 245

206 Fructose and mannose metabolism 246
206.1 Human Pathway: HSA00051 246
206.2 Number of Hits: 16 246
206.3 Legend: 246

207 ECM-receptor interaction 247
207.1 Human Pathway: HSA04512 247
207.2 Number of Hits: 16 247
207.3 Legend: 247

208 Cytosolic DNA-sensing pathway 248
208.1 Human Pathway: HSA04623 248
208.2 Number of Hits: 15 248
208.3 Legend: 248

209 Collecting duct acid secretion 249
209.1 Human Pathway: HSA04966 249
209.2 Number of Hits: 15 249
209.3 Legend: 250

210 Legionellosis 250
210.1 Human Pathway: HSA05134 250
210.2 Number of Hits: 15 250
210.3 Legend: 251

211 Glycosaminoglycan biosynthesis - heparan sulfate / heparin 251
211.1 Human Pathway: HSA00534 251
211.2 Number of Hits: 15 251
211.3 Legend: 251

212 Starch and sucrose metabolism 252
212.1 Human Pathway: HSA00500 252
212.2 Number of Hits: 15 252
212.3 Legend: 252

213 Retinol metabolism 253
213.1 Human Pathway: HSA00830 253
213.2 Number of Hits: 15 253
213.3 Legend: 253

214 Phototransduction **254**
214.1 Human Pathway: HSA04744 254
214.2 Number of Hits: 15 254
214.3 Legend: 254

215 Ether lipid metabolism **255**
215.1 Human Pathway: HSA00565 255
215.2 Number of Hits: 15 255
215.3 Legend: 255

216 Base excision repair **256**
216.1 Human Pathway: HSA03410 256
216.2 Number of Hits: 14 256
216.3 Legend: 256

217 Regulation of autophagy **257**
217.1 Human Pathway: HSA04140 257
217.2 Number of Hits: 14 257
217.3 Legend: 257

218 Ovarian steroidogenesis **258**
218.1 Human Pathway: HSA04913 258
218.2 Number of Hits: 14 258
218.3 Legend: 258

219 Biosynthesis of unsaturated fatty acids **259**
219.1 Human Pathway: HSA01040 259
219.2 Number of Hits: 14 259
219.3 Legend: 259

220 Mineral absorption **260**
220.1 Human Pathway: HSA04978 260
220.2 Number of Hits: 14 260
220.3 Legend: 260

221 Fanconi anemia pathway **261**
221.1 Human Pathway: HSA03460 261
221.2 Number of Hits: 14 261
221.3 Legend: 262

222 Pentose phosphate pathway **262**
222.1 Human Pathway: HSA00030 262
222.2 Number of Hits: 14 262
222.3 Legend: 262

223 Arachidonic acid metabolism **263**
223.1 Human Pathway: HSA00590 263
223.2 Number of Hits: 14 263
223.3 Legend: 263

224 Drug metabolism - other enzymes **264**
224.1 Human Pathway: HSA00983 264
224.2 Number of Hits: 14 264
224.3 Legend: 264

225 Fat digestion and absorption **265**
225.1 Human Pathway: HSA04975 265
225.2 Number of Hits: 13 265
225.3 Legend: 265

226 Porphyrin and chlorophyll metabolism **266**
226.1 Human Pathway: HSA00860 266
226.2 Number of Hits: 13 266
226.3 Legend: 266

227 Thyroid cancer **267**
227.1 Human Pathway: HSA05216 267
227.2 Number of Hits: 13 267
227.3 Legend: 267

228 Galactose metabolism **268**
228.1 Human Pathway: HSA00052 268
228.2 Number of Hits: 13 268
228.3 Legend: 268

229 Arginine biosynthesis **269**
229.1 Human Pathway: HSA00220 269
229.2 Number of Hits: 13 269
229.3 Legend: 269

230 Chemical carcinogenesis **271**
230.1 Human Pathway: HSA05204 271
230.2 Number of Hits: 12 271
230.3 Legend: 272

231 Circadian rhythm **272**
231.1 Human Pathway: HSA04710 272
231.2 Number of Hits: 12 272
231.3 Legend: 272

232 Tyrosine metabolism **273**
232.1 Human Pathway: HSA00350 273
232.2 Number of Hits: 12 273
232.3 Legend: 273

233 Nicotinate and nicotinamide metabolism **274**
233.1 Human Pathway: HSA00760 274
233.2 Number of Hits: 12 274
233.3 Legend: 274

234 One carbon pool by folate **275**
234.1 Human Pathway: HSA00670 275
234.2 Number of Hits: 12 275
234.3 Legend: 275

235 Histidine metabolism **276**
235.1 Human Pathway: HSA00340 276
235.2 Number of Hits: 12 276
235.3 Legend: 276

236 Butanoate metabolism **277**
236.1 Human Pathway: HSA00650 277
236.2 Number of Hits: 12 277
236.3 Legend: 277

237 Cytokine-cytokine receptor interaction **278**
237.1 Human Pathway: HSA04060 278
237.2 Number of Hits: 11 278
237.3 Legend: 278

238 Proximal tubule bicarbonate reclamation **279**
238.1 Human Pathway: HSA04964 279
238.2 Number of Hits: 11 279
238.3 Legend: 280

239 Homologous recombination **280**
239.1 Human Pathway: HSA03440 280
239.2 Number of Hits: 11 280
239.3 Legend: 280

240 Hippo signaling pathway -multiple species **281**
240.1 Human Pathway: HSA04392 281
240.2 Number of Hits: 11 281
240.3 Legend: 281

241 Prion diseases **282**
241.1 Human Pathway: HSA05020 282
241.2 Number of Hits: 11 282
241.3 Legend: 282

242 Apoptosis - multiple species **283**
242.1 Human Pathway: HSA04215 283
242.2 Number of Hits: 11 283
242.3 Legend: 283

243 Glycosylphosphatidylinositol(GPI)-anchor biosynthesis **284**
243.1 Human Pathway: HSA00563 284
243.2 Number of Hits: 10 284
243.3 Legend: 285

244 Antigen processing and presentation **285**
244.1 Human Pathway: HSA04612 285
244.2 Number of Hits: 10 285
244.3 Legend: 285

245 Maturity onset diabetes of the young **286**
245.1 Human Pathway: HSA04950 286
245.2 Number of Hits: 10 286
245.3 Legend: 287

246 Glycosaminoglycan biosynthesis - chondroitin sulfate / dermatan sulfate 287
246.1 Human Pathway: HSA00532 287
246.2 Number of Hits: 10 287
246.3 Legend: 287

247 Terpenoid backbone biosynthesis 288
247.1 Human Pathway: HSA00900 288
247.2 Number of Hits: 10 288
247.3 Legend: 289

248 Inflammatory bowel disease (IBD) 289
248.1 Human Pathway: HSA05321 289
248.2 Number of Hits: 10 289
248.3 Legend: 290

249 Viral myocarditis 290
249.1 Human Pathway: HSA05416 290
249.2 Number of Hits: 9 290
249.3 Legend: 290

250 2-Oxocarboxylic acid metabolism 291
250.1 Human Pathway: HSA01210 291
250.2 Number of Hits: 9 291
250.3 Legend: 291

251 Metabolism of xenobiotics by cytochrome P450 292
251.1 Human Pathway: HSA00980 292
251.2 Number of Hits: 9 292
251.3 Legend: 292

252 Selenocompound metabolism 293
252.1 Human Pathway: HSA00450 293
252.2 Number of Hits: 9 293
252.3 Legend: 293

253 Pantothenate and CoA biosynthesis 294
253.1 Human Pathway: HSA00770 294
253.2 Number of Hits: 9 294
253.3 Legend: 294

254 Mismatch repair **295**
254.1 Human Pathway: HSA03430 295
254.2 Number of Hits: 9 295
254.3 Legend: 295

255 Glycosphingolipid biosynthesis - lacto and neolacto series **296**
255.1 Human Pathway: HSA00601 296
255.2 Number of Hits: 9 296
255.3 Legend: 297

256 Fatty acid biosynthesis **297**
256.1 Human Pathway: HSA00061 297
256.2 Number of Hits: 8 297
256.3 Legend: 297

257 Drug metabolism - cytochrome P450 **298**
257.1 Human Pathway: HSA00982 298
257.2 Number of Hits: 8 298
257.3 Legend: 298

258 Steroid hormone biosynthesis **299**
258.1 Human Pathway: HSA00140 299
258.2 Number of Hits: 8 299
258.3 Legend: 299

259 Pentose and glucuronate interconversions **300**
259.1 Human Pathway: HSA00040 300
259.2 Number of Hits: 7 300
259.3 Legend: 300

260 Ascorbate and aldarate metabolism **301**
260.1 Human Pathway: HSA00053 301
260.2 Number of Hits: 7 301
260.3 Legend: 301

261 Glycosphingolipid biosynthesis - ganglio series **302**
261.1 Human Pathway: HSA00604 302
261.2 Number of Hits: 7 302
261.3 Legend: 303

262 African trypanosomiasis **303**
262.1 Human Pathway: HSA05143 303
262.2 Number of Hits: 7 303
262.3 Legend: 303

263 Phenylalanine metabolism **304**
263.1 Human Pathway: HSA00360 304
263.2 Number of Hits: 6 304
263.3 Legend: 304

264 Renin-angiotensin system **305**
264.1 Human Pathway: HSA04614 305
264.2 Number of Hits: 6 305
264.3 Legend: 305

265 Vitamin digestion and absorption **306**
265.1 Human Pathway: HSA04977 306
265.2 Number of Hits: 6 306
265.3 Legend: 306

266 Glycosaminoglycan degradation **307**
266.1 Human Pathway: HSA00531 307
266.2 Number of Hits: 6 307
266.3 Legend: 307

267 Folate biosynthesis **308**
267.1 Human Pathway: HSA00790 308
267.2 Number of Hits: 6 308
267.3 Legend: 308

268 Primary immunodeficiency **309**
268.1 Human Pathway: HSA05340 309
268.2 Number of Hits: 6 309
268.3 Legend: 309

269 Nitrogen metabolism **310**
269.1 Human Pathway: HSA00910 310
269.2 Number of Hits: 6 310
269.3 Legend: 310

270 Glycosaminoglycan biosynthesis - keratan sulfate 311
270.1 Human Pathway: HSA00533 311
270.2 Number of Hits: 5 311
270.3 Legend: 311

271 Type I diabetes mellitus 312
271.1 Human Pathway: HSA04940 312
271.2 Number of Hits: 5 312
271.3 Legend: 312

272 Other glycan degradation 313
272.1 Human Pathway: HSA00511 313
272.2 Number of Hits: 5 313
272.3 Legend: 313

273 Sulfur relay system 314
273.1 Human Pathway: HSA04122 314
273.2 Number of Hits: 5 314
273.3 Legend: 314

274 Taurine and hypotaurine metabolism 315
274.1 Human Pathway: HSA00430 315
274.2 Number of Hits: 5 315
274.3 Legend: 315

275 Primary bile acid biosynthesis 317
275.1 Human Pathway: HSA00120 317
275.2 Number of Hits: 5 317
275.3 Legend: 318

276 Malaria 318
276.1 Human Pathway: HSA05144 318
276.2 Number of Hits: 5 318
276.3 Legend: 318

277 alpha-Linolenic acid metabolism 319
277.1 Human Pathway: HSA00592 319
277.2 Number of Hits: 4 319
277.3 Legend: 319

278 Non-homologous end-joining **320**
278.1 Human Pathway: HSA03450 320
278.2 Number of Hits: 4 320
278.3 Legend: 320

279 Synthesis and degradation of ketone bodies **321**
279.1 Human Pathway: HSA00072 321
279.2 Number of Hits: 4 321
279.3 Legend: 321

280 Ubiquinone and other terpenoid-quinone biosynthesis **322**
280.1 Human Pathway: HSA00130 322
280.2 Number of Hits: 4 322
280.3 Legend: 323

281 Sulfur metabolism **323**
281.1 Human Pathway: HSA00920 323
281.2 Number of Hits: 4 323
281.3 Legend: 324

282 Thiamine metabolism **324**
282.1 Human Pathway: HSA00730 324
282.2 Number of Hits: 3 324
282.3 Legend: 324

283 Butirosin and neomycin biosynthesis **325**
283.1 Human Pathway: HSA00524 325
283.2 Number of Hits: 3 325
283.3 Legend: 325

284 Vitamin B6 metabolism **326**
284.1 Human Pathway: HSA00750 326
284.2 Number of Hits: 2 326
284.3 Legend: 326

285 Lysine biosynthesis **327**
285.1 Human Pathway: HSA00300 327
285.2 Number of Hits: 2 327
285.3 Legend: 327

286 Glycosphingolipid biosynthesis - globo series **328**
286.1 Human Pathway: HSA00603 328
286.2 Number of Hits: 2 328
286.3 Legend: 329

287 Linoleic acid metabolism **329**
287.1 Human Pathway: HSA00591 329
287.2 Number of Hits: 2 329
287.3 Legend: 329

288 D-Glutamine and D-glutamate metabolism **330**
288.1 Human Pathway: HSA00471 330
288.2 Number of Hits: 2 330
288.3 Legend: 330

289 Valine, leucine and isoleucine biosynthesis **331**
289.1 Human Pathway: HSA00290 331
289.2 Number of Hits: 2 331
289.3 Legend: 331

290 Lipoic acid metabolism **332**
290.1 Human Pathway: HSA00785 332
290.2 Number of Hits: 1 332
290.3 Legend: 332

291 Biotin metabolism **333**
291.1 Human Pathway: HSA00780 333
291.2 Number of Hits: 1 333
291.3 Legend: 334

292 Riboflavin metabolism **334**
292.1 Human Pathway: HSA00740 334
292.2 Number of Hits: 1 334
292.3 Legend: 334

293 Autoimmune thyroid disease **335**
293.1 Human Pathway: HSA05320 335
293.2 Number of Hits: 1 335
293.3 Legend: 335

294 Phenylalanine, tyrosine and tryptophan biosynthesis 336
294.1 Human Pathway: HSA00400 336
294.2 Number of Hits: 1 336
294.3 Legend: 336

295 D-Arginine and D-ornithine metabolism 337
295.1 Human Pathway: HSA00472 337
295.2 Number of Hits: 1 337
295.3 Legend: 337

296 Caffeine metabolism 338
296.1 Human Pathway: HSA00232 338
296.2 Number of Hits: 1 338
296.3 Legend: 338

297 Hematopoietic cell lineage 339
297.1 Human Pathway: HSA04640 339
297.2 Number of Hits: 1 339
297.3 Legend: 340

1 Pathways in cancer

1.1 Human Pathway: HSA05200

1.2 Number of Hits: 180

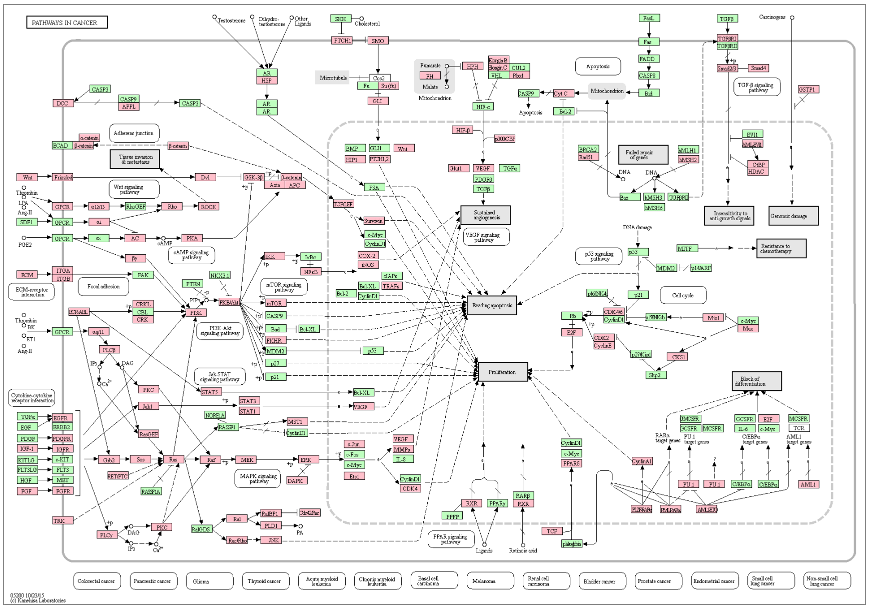

1.3 Legend:

RBH-Blast at 60% Identity + 50% Coverage
Green = Hit in *H. sapiens*
Red = Hit in *H. sapiens* and *C. milli*
White = Not in *H. sapiens*

2 MAPK signaling pathway

2.1 Human Pathway: HSA04010

2.2 Number of Hits: 118

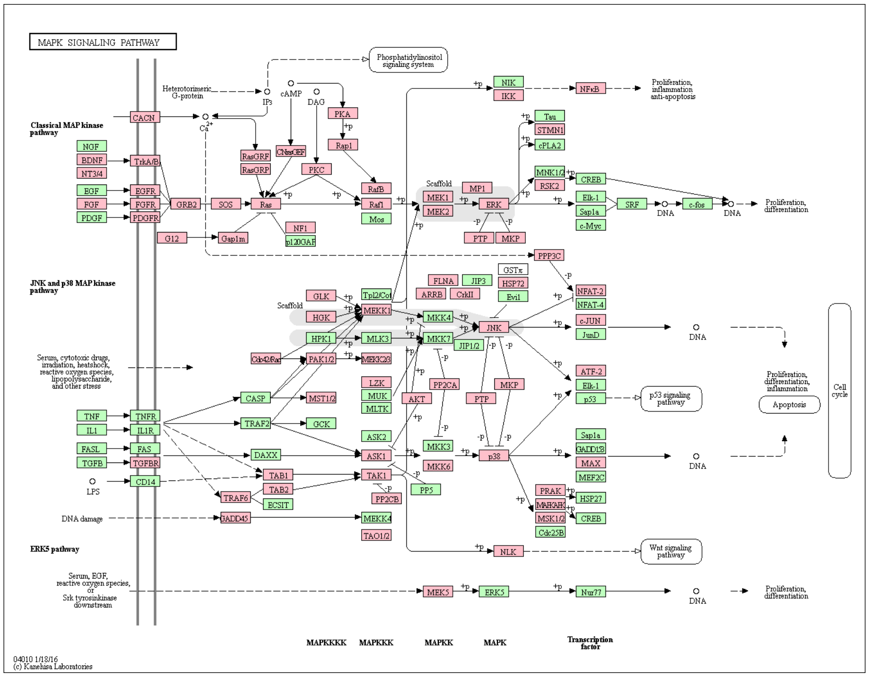

2.3 Legend:

RBH-Blast at 60% Identity + 50% Coverage
Green = Hit in *H. sapiens*
Red = Hit in *H. sapiens* and *C. milli*
White = Not in *H. sapiens*

3 Endocytosis

3.1 Human Pathway: HSA04144

3.2 Number of Hits: 117

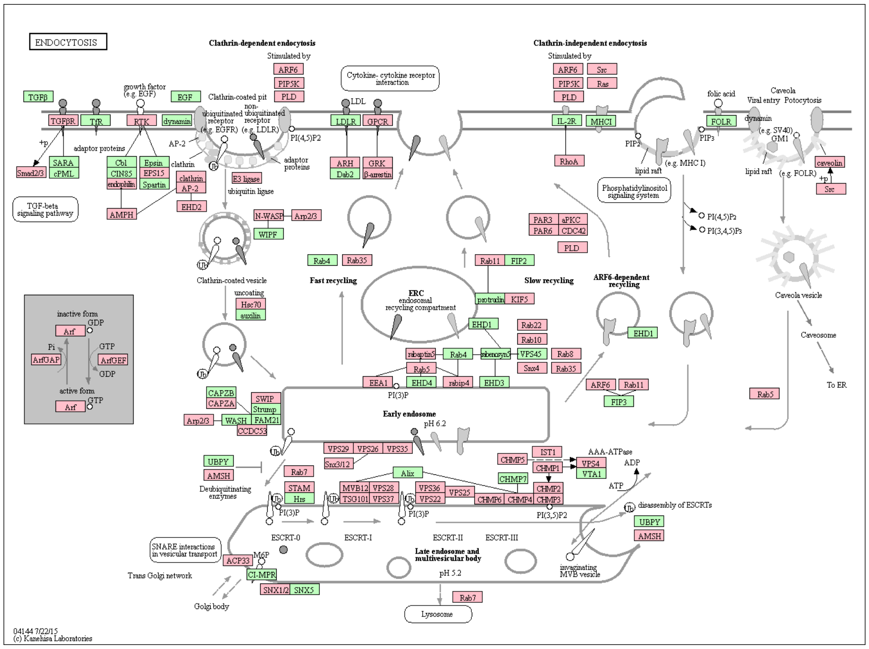

3.3 Legend:

RBH-Blast at 60% Identity + 50% Coverage
Green = Hit in *H. sapiens*
Red = Hit in *H. sapiens* and *C. milli*
White = Not in *H. sapiens*

4 PI3K-Akt signaling pathway

4.1 Human Pathway: HSA04151

4.2 Number of Hits: 114

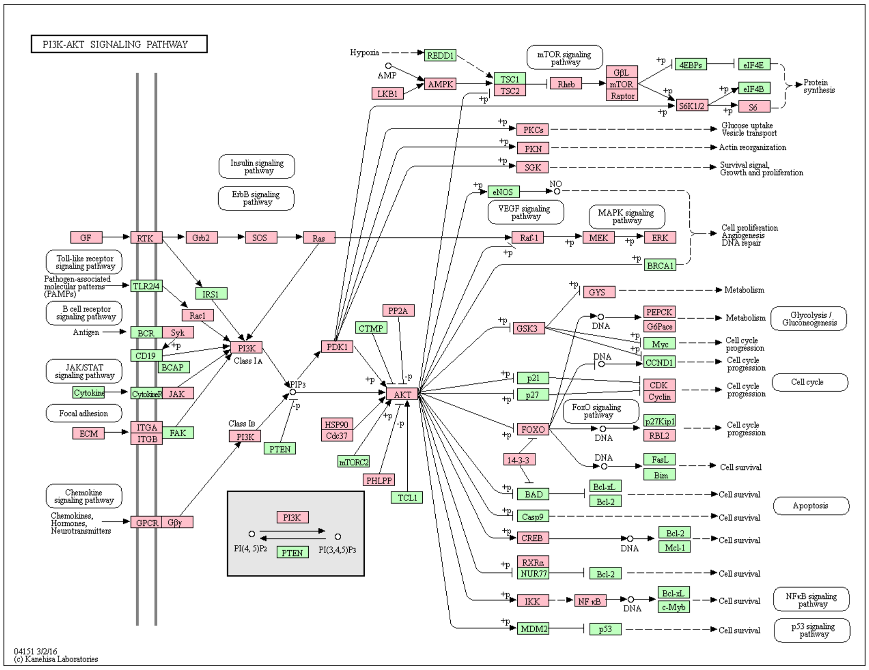

4.3 Legend:

RBH-Blast at 60% Identity + 50% Coverage
Green = Hit in *H. sapiens*
Red = Hit in *H. sapiens* and *C. milli*
White = Not in *H. sapiens*

5 HTLV-I infection

5.1 Human Pathway: HSA05166

5.2 Number of Hits: 109

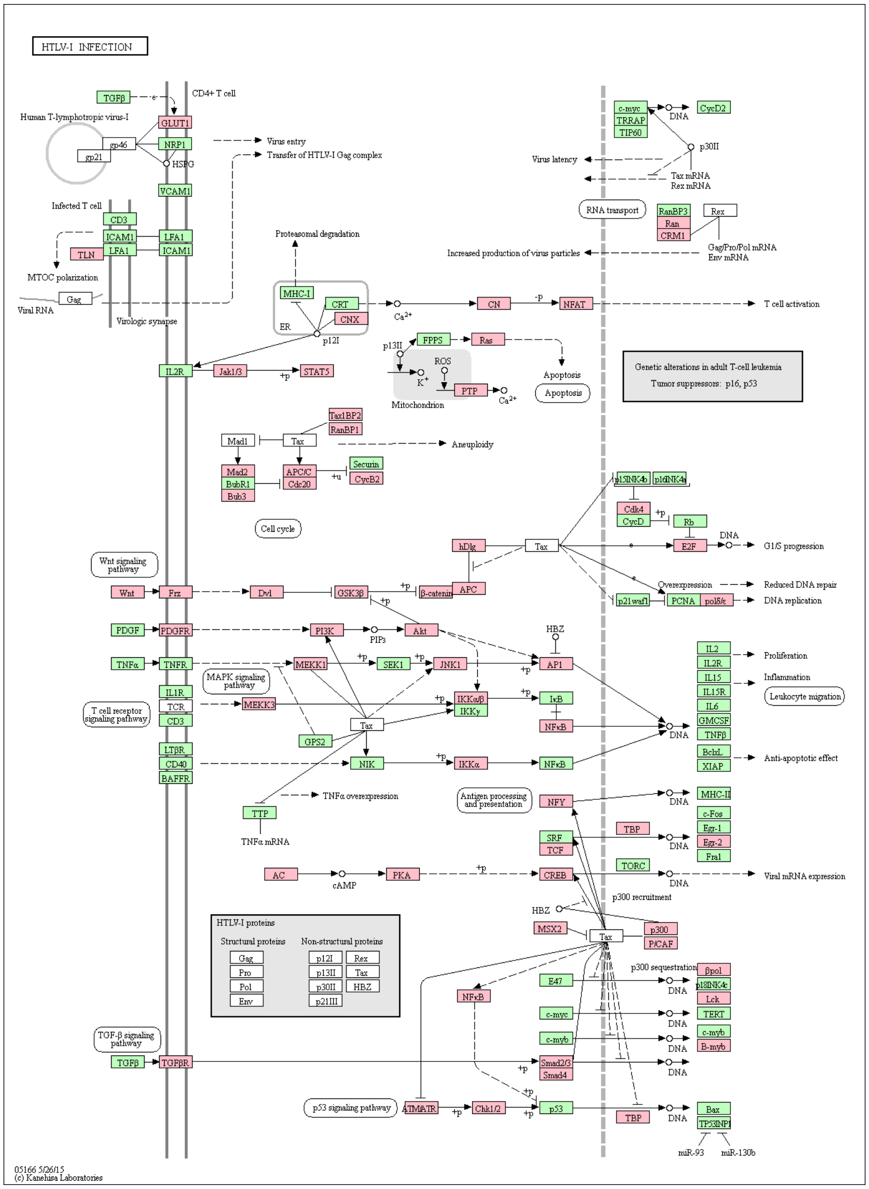

5.3 Legend:

RBH-Blast at 60% Identity + 50% Coverage
Green = Hit in *H. sapiens*
Red = Hit in *H. sapiens* and *C. milli*
White = Not in *H. sapiens*

---

6 Ras signaling pathway

6.1 Human Pathway: HSA04014

6.2 Number of Hits: 102

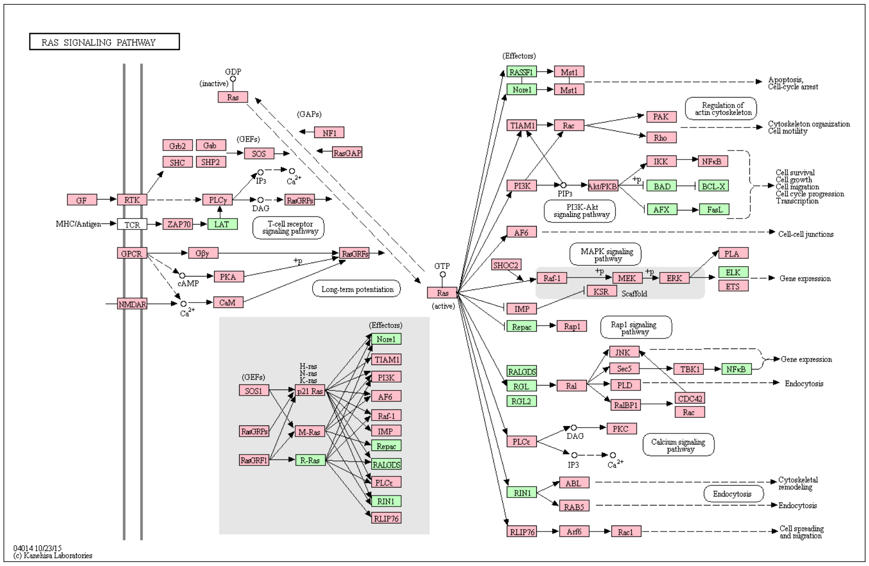

6.3 Legend:

RBH-Blast at 60% Identity + 50% Coverage
Green = Hit in *H. sapiens*
Red = Hit in *H. sapiens* and *C. milli*
White = Not in *H. sapiens*

---

7 Rap1 signaling pathway

7.1 Human Pathway: HSA04015

7.2 Number of Hits: 102

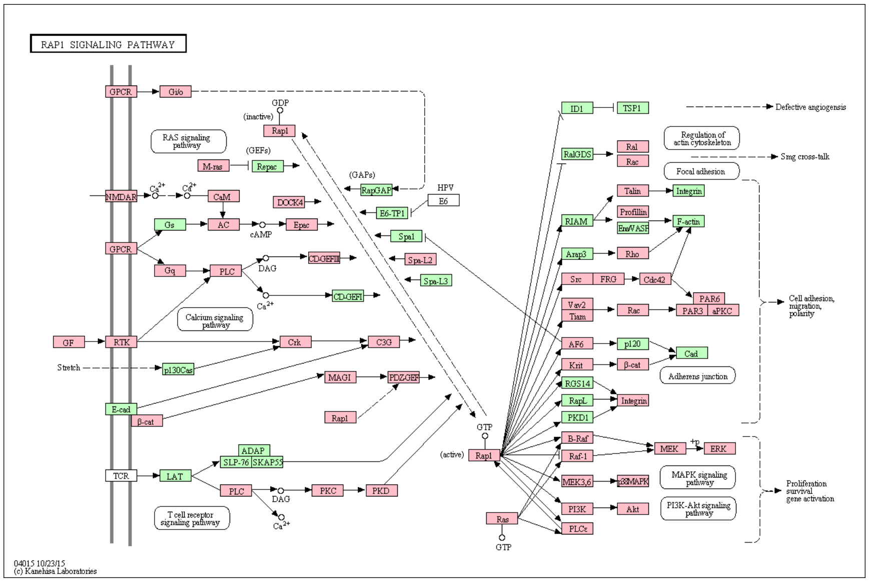

7.3 Legend:

RBH-Blast at 60% Identity + 50% Coverage
Green = Hit in *H. sapiens*
Red = Hit in *H. sapiens* and *C. milli*
White = Not in *H. sapiens*

8 Neuroactive ligand-receptor interaction

8.1 Human Pathway: HSA04080

8.2 Number of Hits: 100

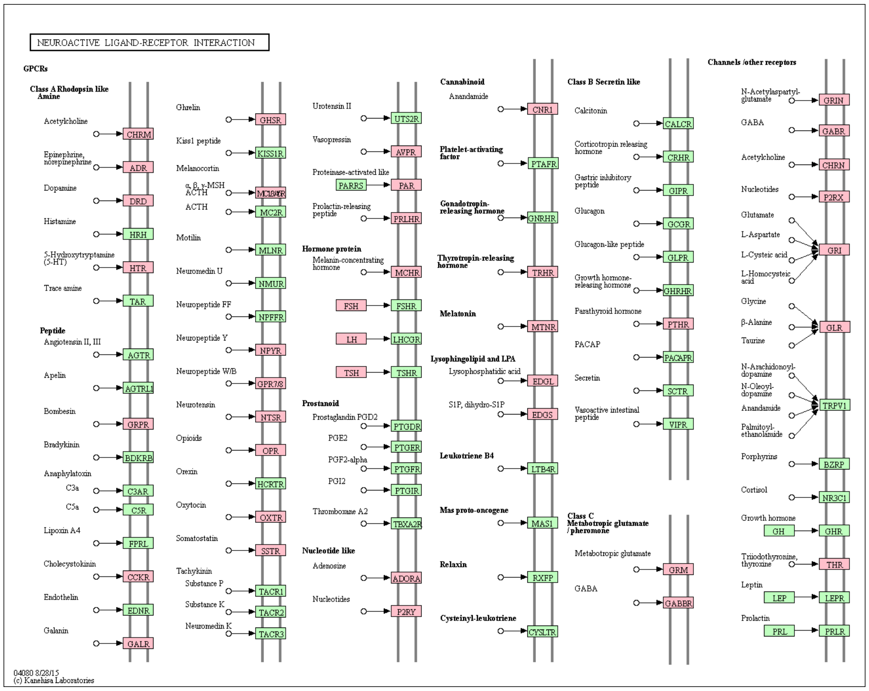

8.3 Legend:

RBH-Blast at 60% Identity + 50% Coverage
Green = Hit in *H. sapiens*
Red = Hit in *H. sapiens* and *C. milli*
White = Not in *H. sapiens*

9 Regulation of actin cytoskeleton

9.1 Human Pathway: HSA04810

9.2 Number of Hits: 98

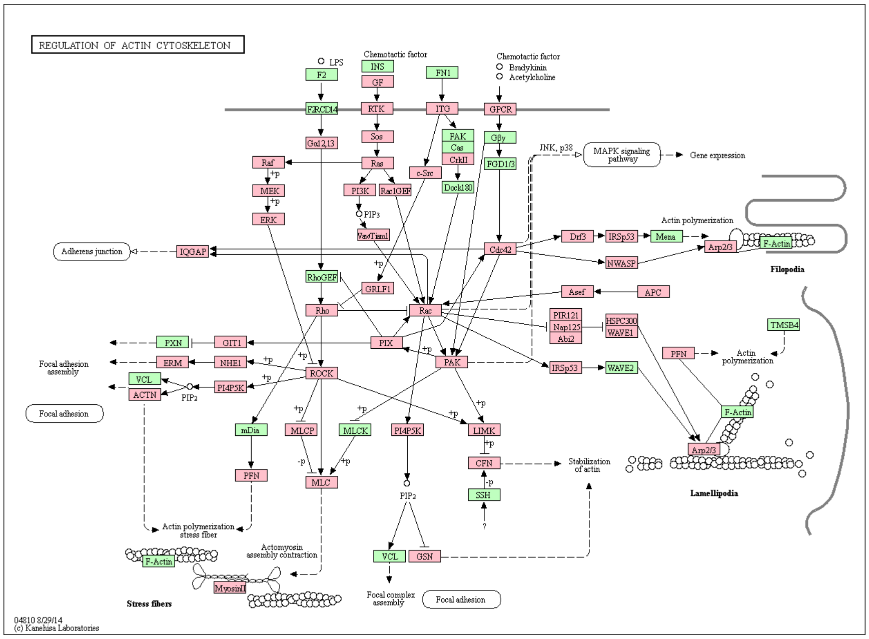

9.3 Legend:

RBH-Blast at 60% Identity + 50% Coverage
Green = Hit in *H. sapiens*
Red = Hit in *H. sapiens* and *C. milli*
White = Not in *H. sapiens*

10 cAMP signaling pathway

10.1 Human Pathway: HSA04024

10.2 Number of Hits: 97

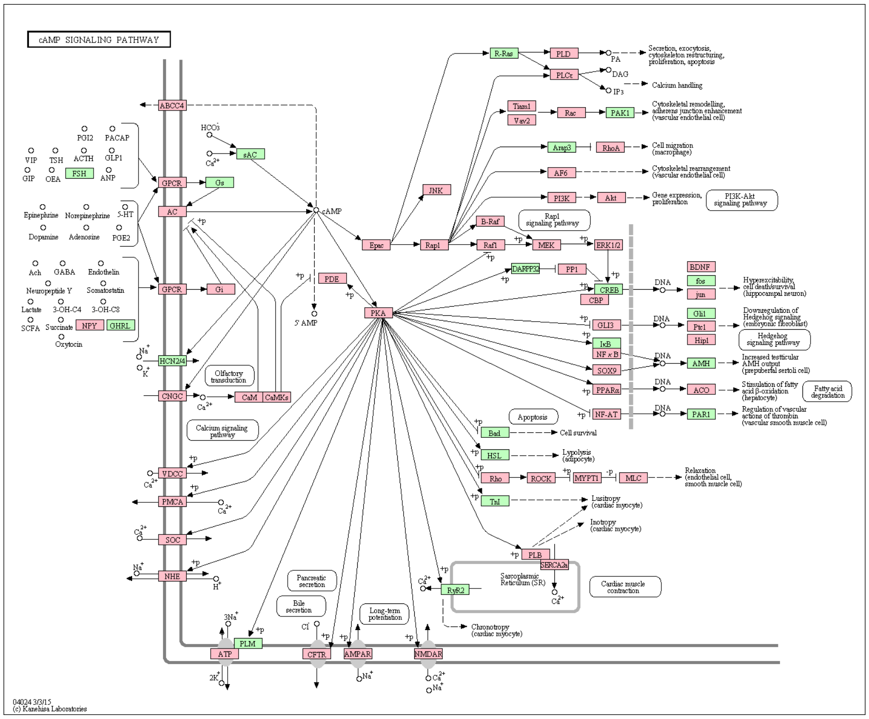

10.3 Legend:

RBH-Blast at 60% Identity + 50% Coverage
Green = Hit in *H. sapiens*
Red = Hit in *H. sapiens* and *C. milli*
White = Not in *H. sapiens*

11 Proteoglycans in cancer

11.1 Human Pathway: HSA05205

11.2 Number of Hits: 96

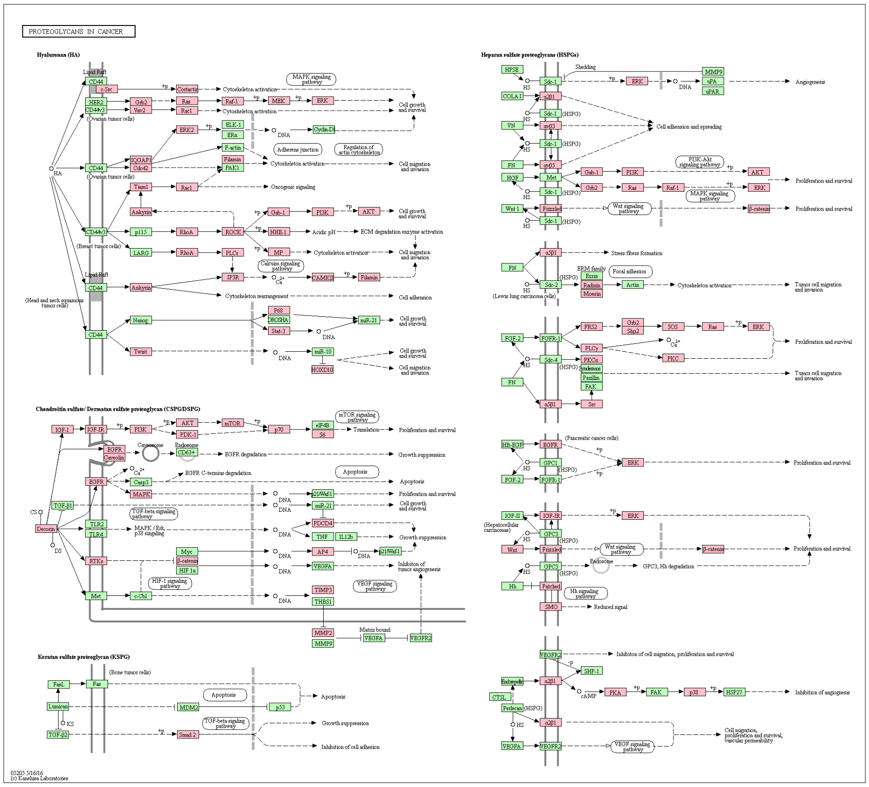

11.3 Legend:

RBH-Blast at 60% Identity + 50% Coverage
Green = Hit in *H. sapiens*
Red = Hit in *H. sapiens* and *C. milli*
White = Not in *H. sapiens*

12 Protein processing in endoplasmic reticulum

12.1 Human Pathway: HSA04141

12.2 Number of Hits: 95

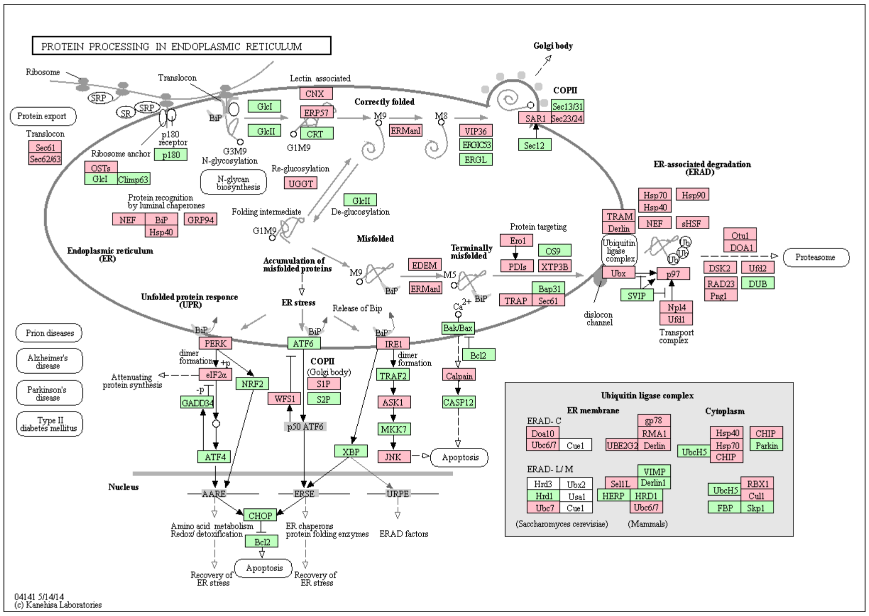

12.3 Legend:

RBH-Blast at 60% Identity + 50% Coverage
Green = Hit in *H. sapiens*
Red = Hit in *H. sapiens* and *C. milli*
White = Not in *H. sapiens*

13 mTOR signaling pathway

13.1 Human Pathway: HSA04150

13.2 Number of Hits: 94

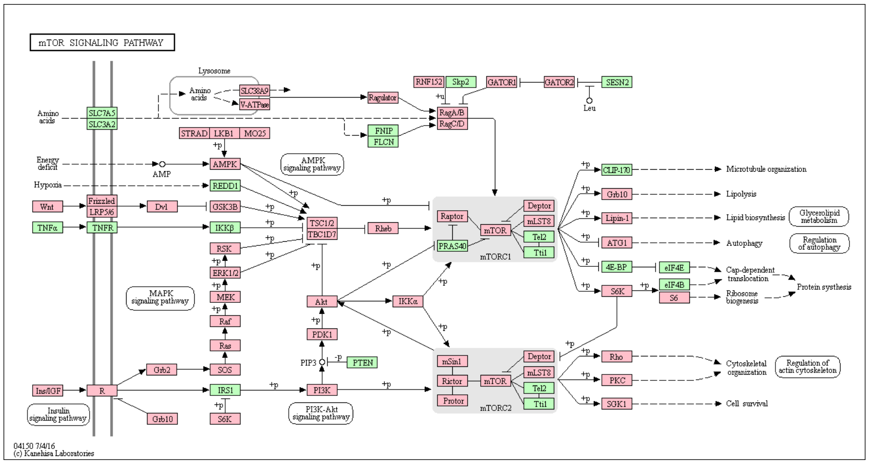

13.3 Legend:

RBH-Blast at 60% Identity + 50% Coverage
Green = Hit in *H. sapiens*
Red = Hit in *H. sapiens* and *C. milli*
White = Not in *H. sapiens*

14 Purine metabolism

14.1 Human Pathway: HSA00230

14.2 Number of Hits: 94

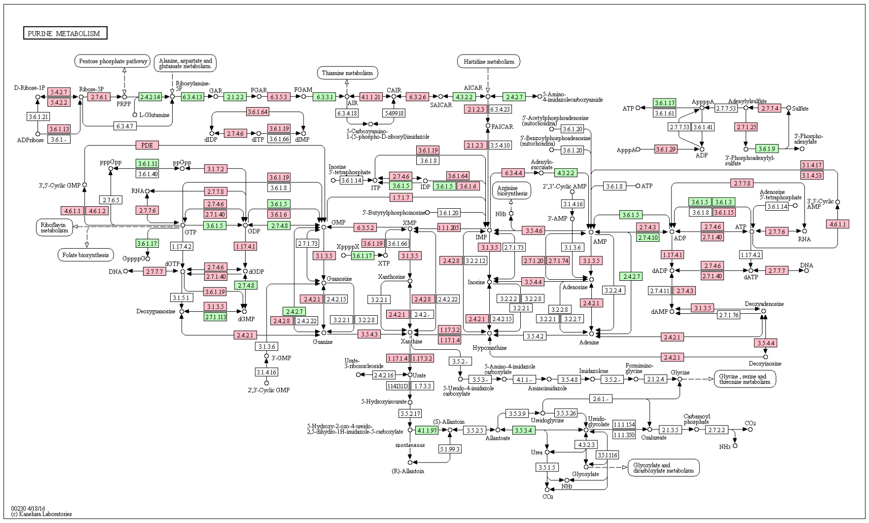

14.3 Legend:

RBH-Blast at 60% Identity + 50% Coverage
Green = Hit in *H. sapiens*
Red = Hit in *H. sapiens* and *C. milli*
White = Not in *H. sapiens*

15 Huntington's disease

15.1 Human Pathway: HSA05016

15.2 Number of Hits: 93

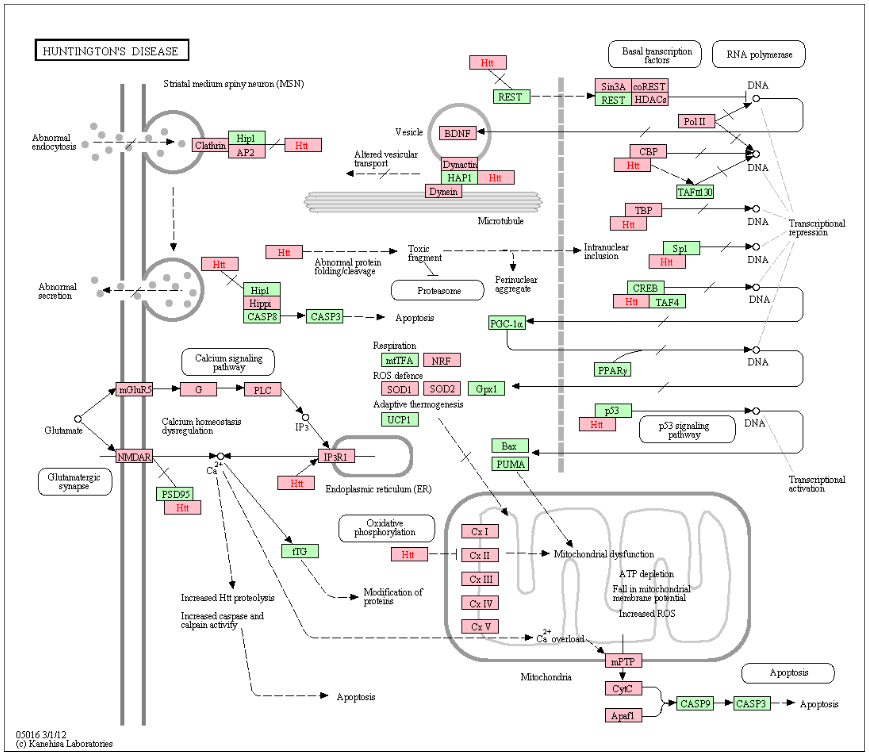

15.3 Legend:

RBH-Blast at 60% Identity + 50% Coverage
Green = Hit in *H. sapiens*
Red = Hit in *H. sapiens* and *C. milli*
White = Not in *H. sapiens*

16 Epstein-Barr virus infection

16.1 Human Pathway: HSA05169

16.2 Number of Hits: 89

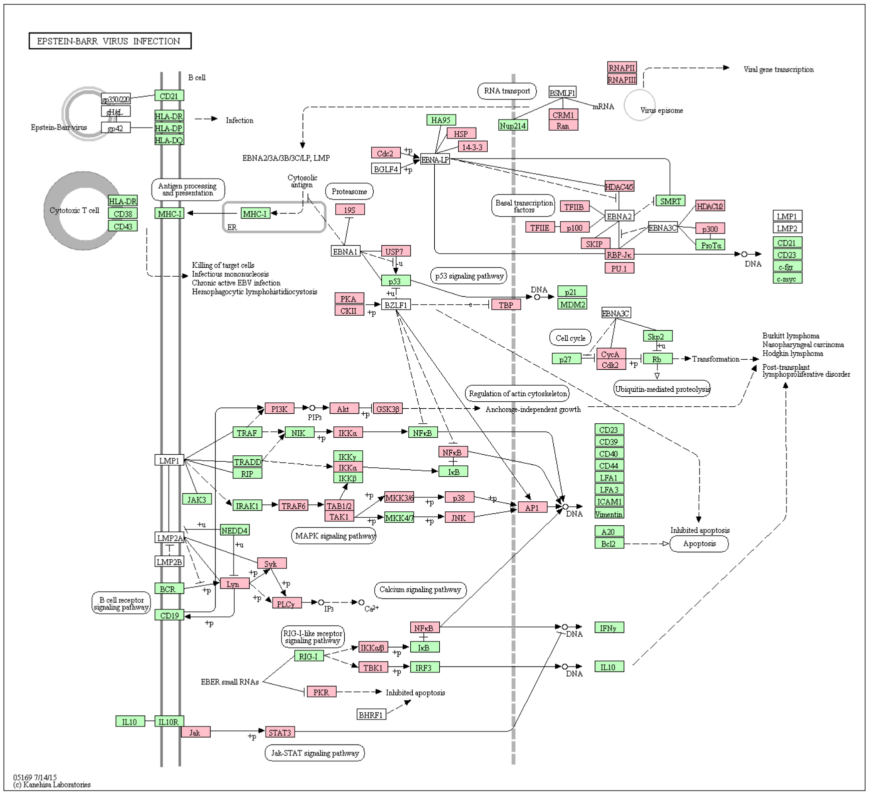

16.3 Legend:

RBH-Blast at 60% Identity + 50% Coverage
Green = Hit in *H. sapiens*
Red = Hit in *H. sapiens* and *C. milli*
White = Not in *H. sapiens*

17 Axon guidance

17.1 Human Pathway: HSA04360

17.2 Number of Hits: 86

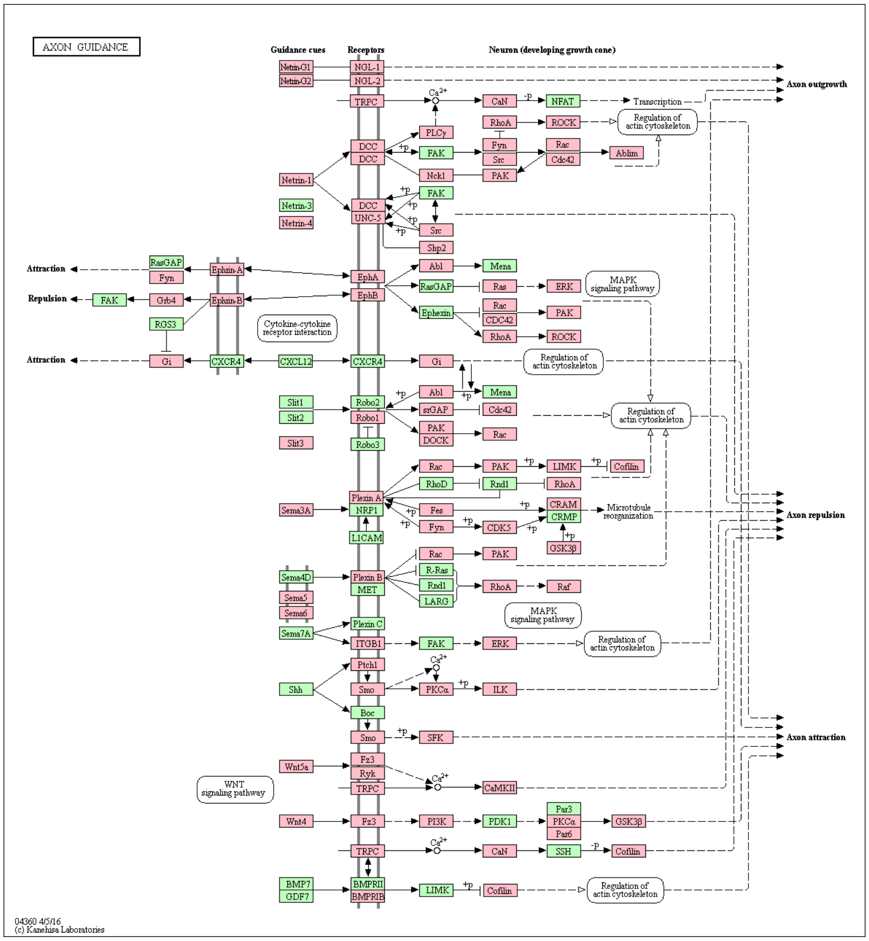

17.3 Legend:

RBH-Blast at 60% Identity + 50% Coverage
Green = Hit in *H. sapiens*
Red = Hit in *H. sapiens* and *C. milli*
White = Not in *H. sapiens*

18 Viral carcinogenesis

18.1 Human Pathway: HSA05203

18.2 Number of Hits: 82

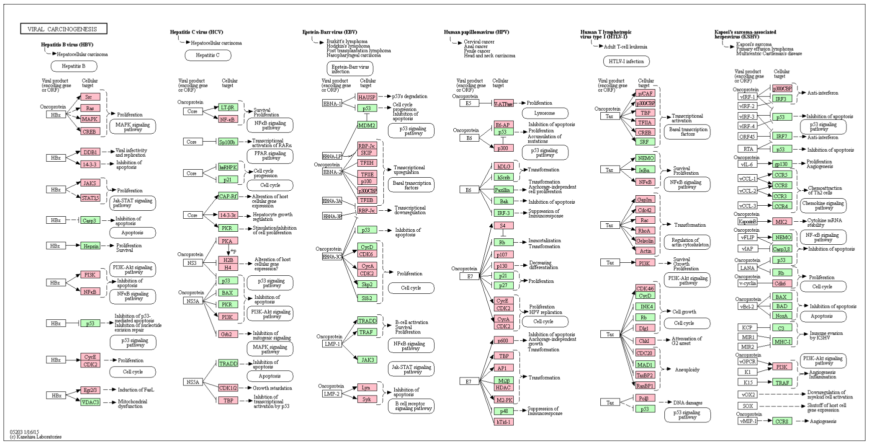

18.3 Legend:

RBH-Blast at 60% Identity + 50% Coverage
Green = Hit in *H. sapiens*
Red = Hit in *H. sapiens* and *C. milli*
White = Not in *H. sapiens*

19 Ubiquitin mediated proteolysis

19.1 Human Pathway: HSA04120

19.2 Number of Hits: 80

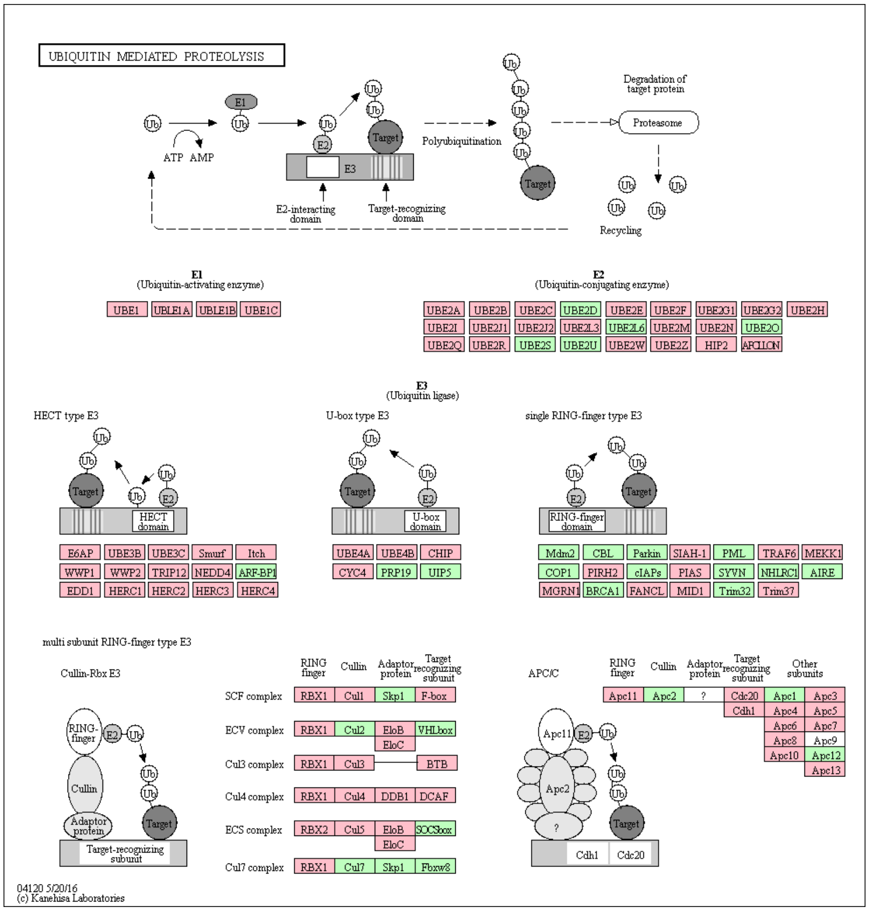

19.3 Legend:

RBH-Blast at 60% Identity + 50% Coverage
Green = Hit in *H. sapiens*
Red = Hit in *H. sapiens* and *C. milli*
White = Not in *H. sapiens*

20 Ribosome

20.1 Human Pathway: HSA03010

20.2 Number of Hits: 80

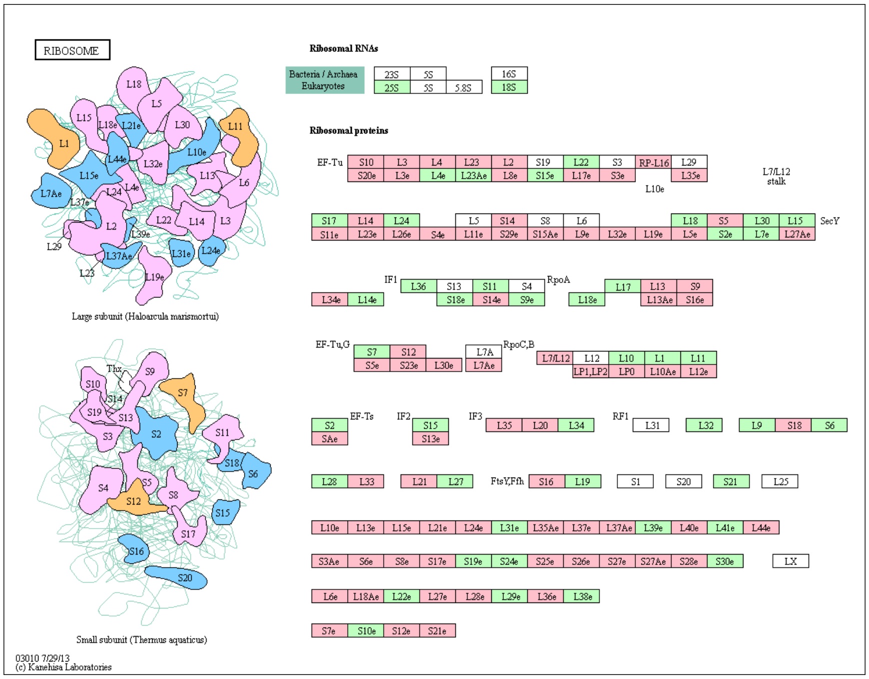

20.3 Legend:

RBH-Blast at 60% Identity + 50% Coverage
Green = Hit in *H. sapiens*
Red = Hit in *H. sapiens* and *C. milli*
White = Not in *H. sapiens*

21 Focal adhesion

21.1 Human Pathway: HSA04510

21.2 Number of Hits: 79

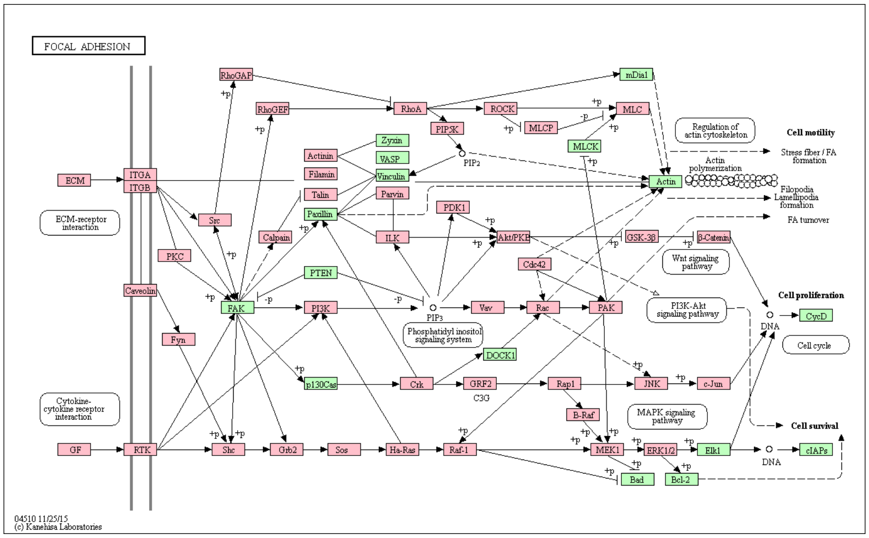

21.3 Legend:

RBH-Blast at 60% Identity + 50% Coverage
Green = Hit in *H. sapiens*
Red = Hit in *H. sapiens* and *C. milli*
White = Not in *H. sapiens*

22 Spliceosome

22.1 Human Pathway: HSA03040

22.2 Number of Hits: 78

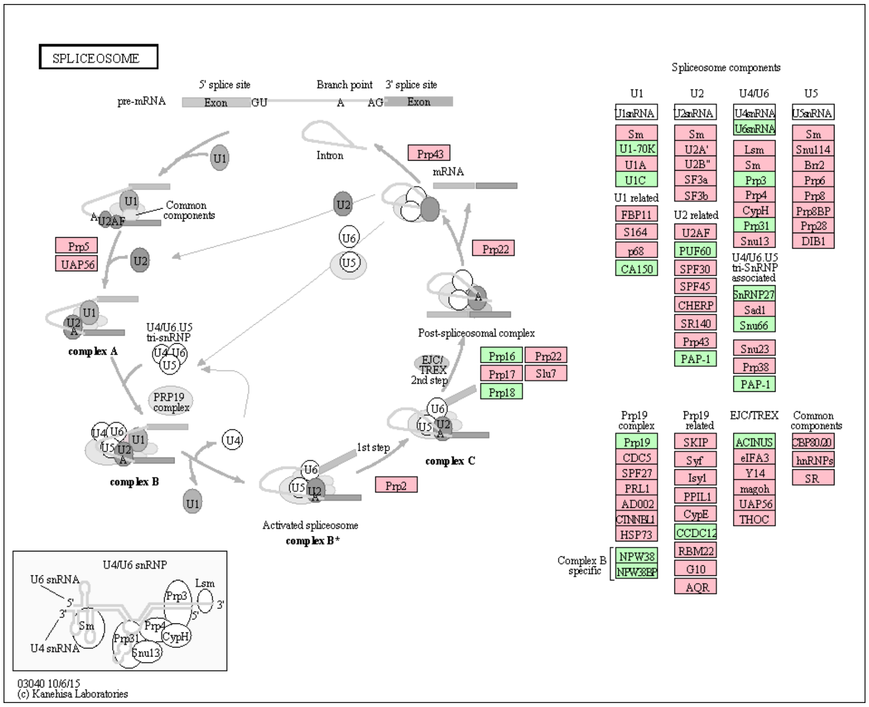

22.3 Legend:

RBH-Blast at 60% Identity + 50% Coverage
Green = Hit in *H. sapiens*
Red = Hit in *H. sapiens* and *C. milli*
White = Not in *H. sapiens*

23 Alzheimer's disease

23.1 Human Pathway: HSA05010

23.2 Number of Hits: 77

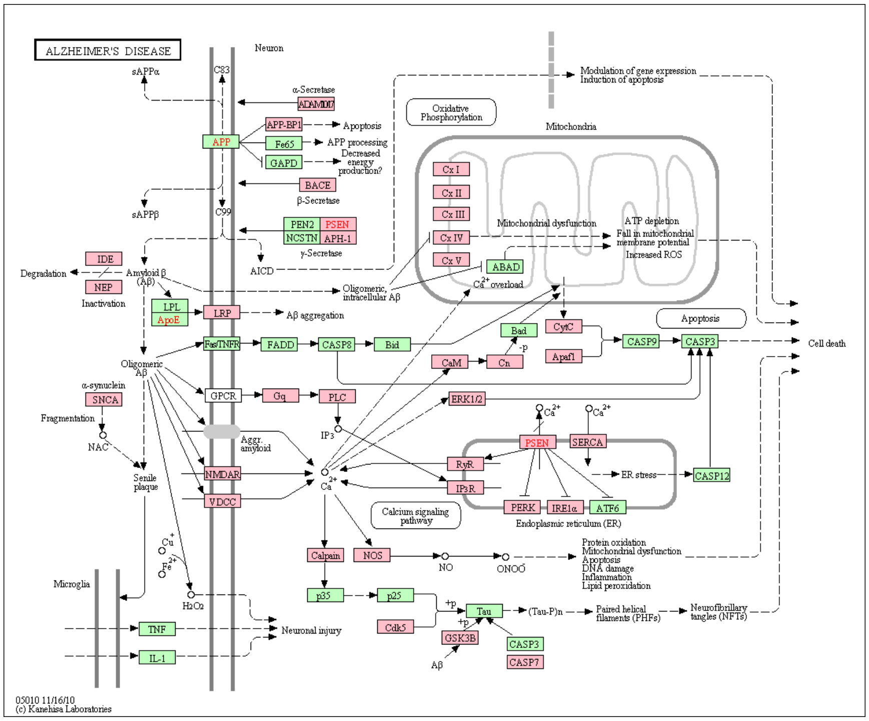

23.3 Legend:

RBH-Blast at 60% Identity + 50% Coverage
Green = Hit in *H. sapiens*
Red = Hit in *H. sapiens* and *C. milli*
White = Not in *H. sapiens*

24 Oxytocin signaling pathway

24.1 Human Pathway: HSA04921

24.2 Number of Hits: 76

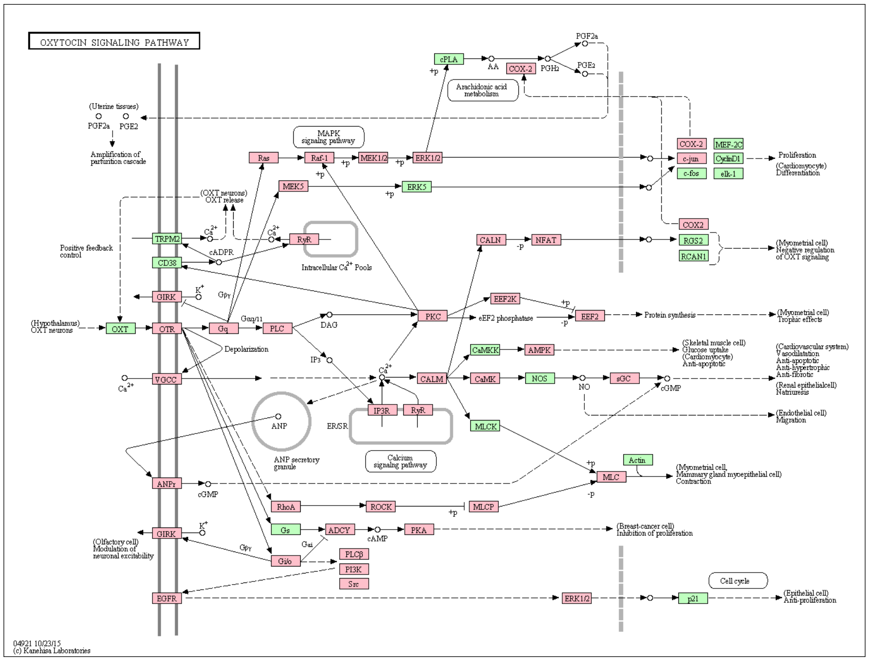

24.3 Legend:

RBH-Blast at 60% Identity + 50% Coverage
Green = Hit in *H. sapiens*
Red = Hit in *H. sapiens* and *C. milli*
White = Not in *H. sapiens*

25 Wnt signaling pathway

25.1 Human Pathway: HSA04310

25.2 Number of Hits: 73

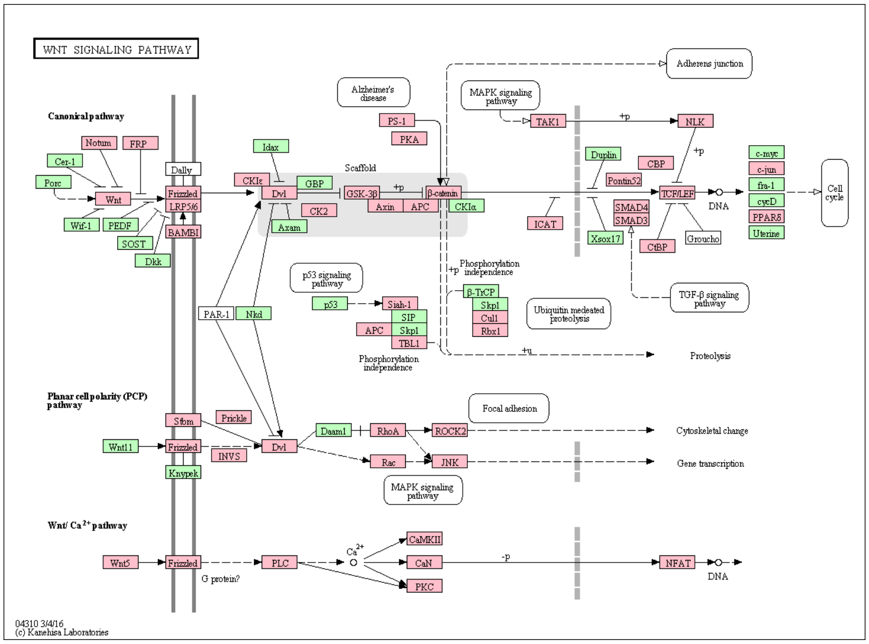

25.3 Legend:

RBH-Blast at 60% Identity + 50% Coverage
Green = Hit in *H. sapiens*
Red = Hit in *H. sapiens* and *C. milli*
White = Not in *H. sapiens*

26 Calcium signaling pathway

26.1 Human Pathway: HSA04020

26.2 Number of Hits: 72

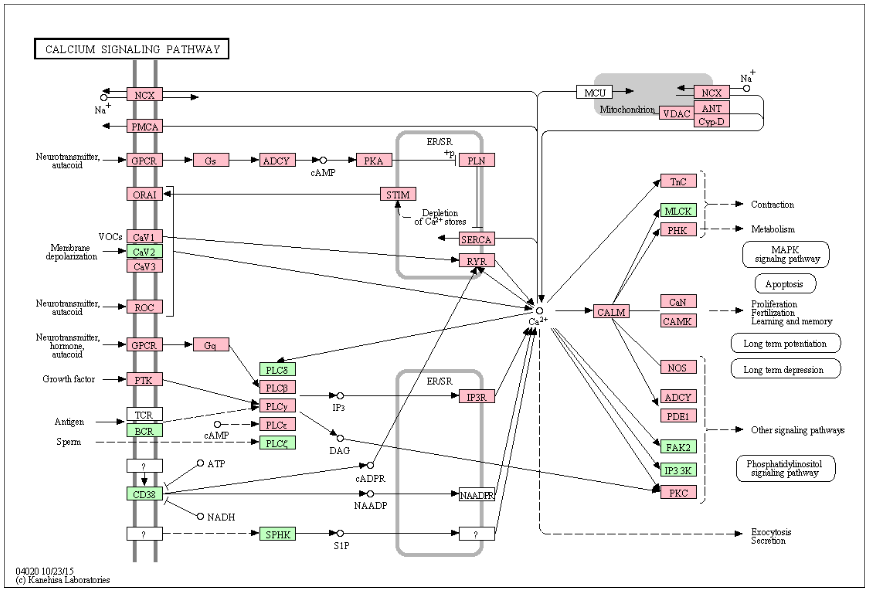

26.3 Legend:

RBH-Blast at 60% Identity + 50% Coverage
Green = Hit in *H. sapiens*
Red = Hit in *H. sapiens* and *C. milli*
White = Not in *H. sapiens*

27 Chemokine signaling pathway

27.1 Human Pathway: HSA04062

27.2 Number of Hits: 72

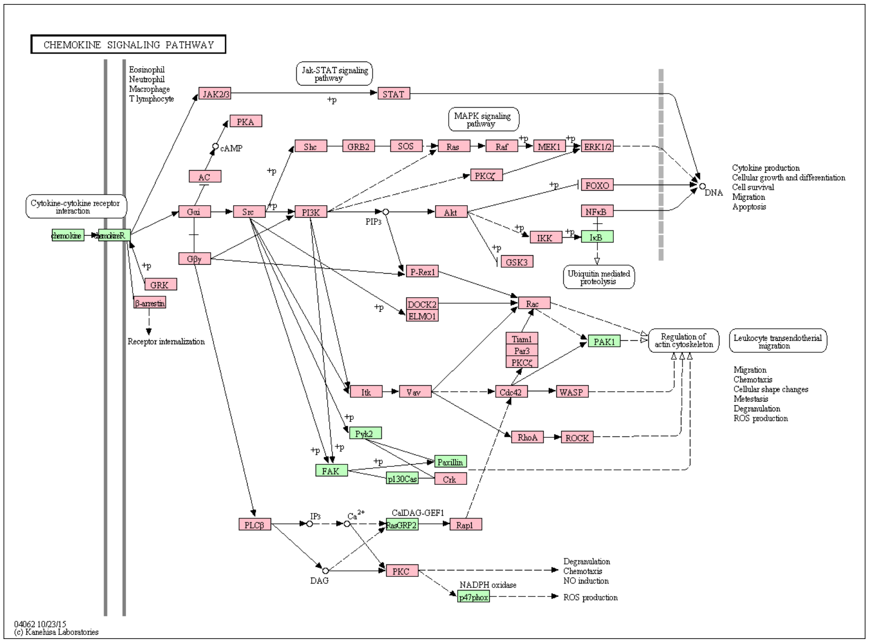

27.3 Legend:

RBH-Blast at 60% Identity + 50% Coverage
Green = Hit in *H. sapiens*
Red = Hit in *H. sapiens* and *C. milli*
White = Not in *H. sapiens*

28 Signaling pathways regulating pluripotency of stem cells

28.1 Human Pathway: HSA04550

28.2 Number of Hits: 72

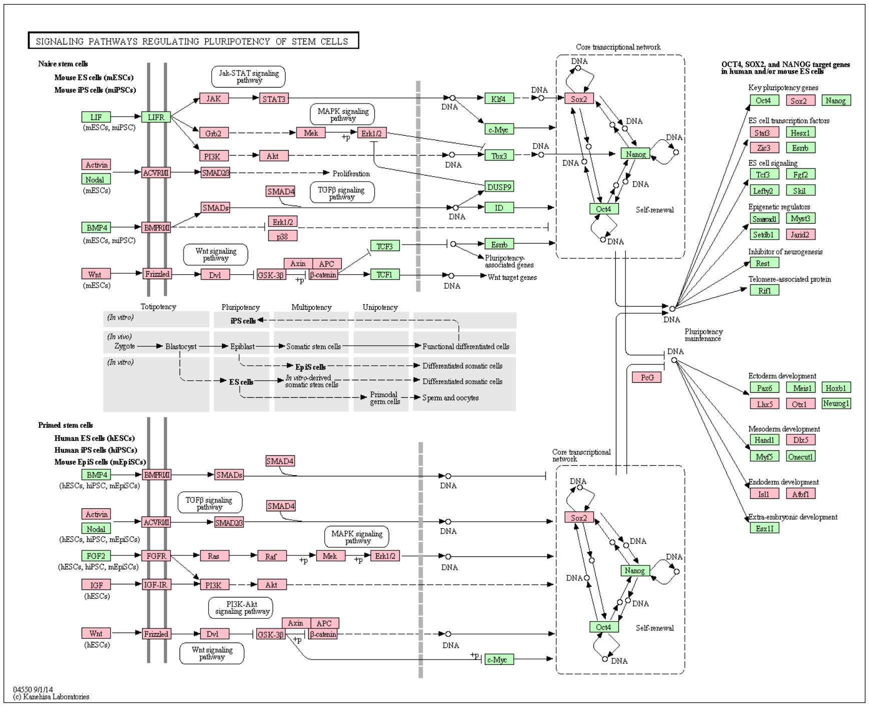

28.3 Legend:

RBH-Blast at 60% Identity + 50% Coverage
Green = Hit in *H. sapiens*
Red = Hit in *H. sapiens* and *C. milli*
White = Not in *H. sapiens*

29 FoxO signaling pathway

29.1 Human Pathway: HSA04068

29.2 Number of Hits: 72

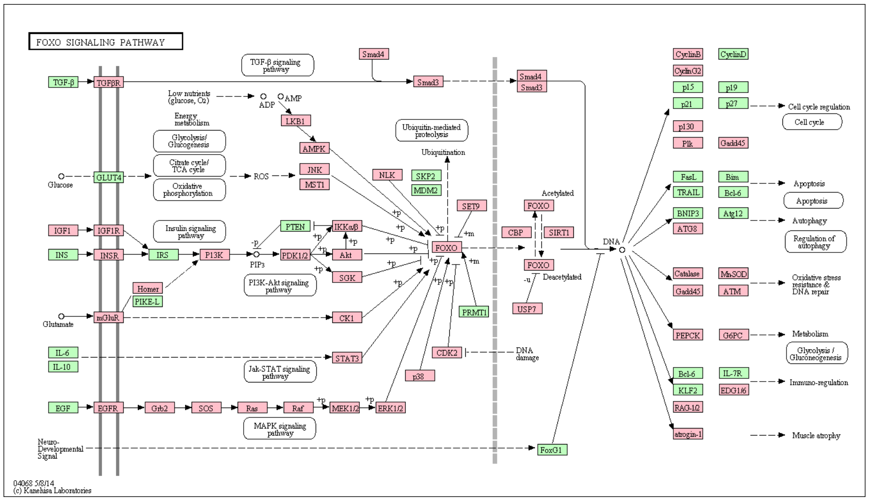

29.3 Legend:

RBH-Blast at 60% Identity + 50% Coverage
Green = Hit in *H. sapiens*
Red = Hit in *H. sapiens* and *C. milli*
White = Not in *H. sapiens*

30 RNA transport

30.1 Human Pathway: HSA03013

30.2 Number of Hits: 71

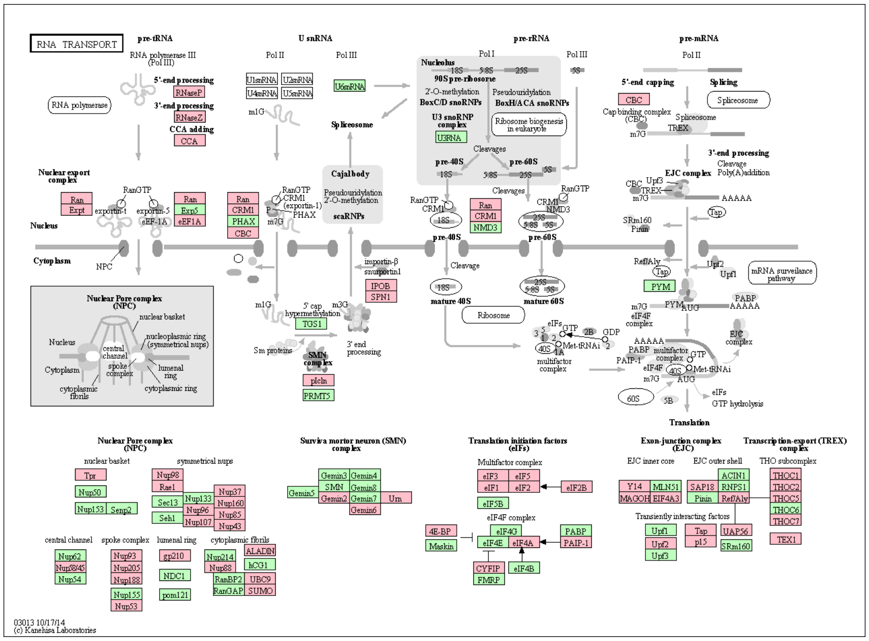

30.3 Legend:

RBH-Blast at 60% Identity + 50% Coverage
Green = Hit in *H. sapiens*
Red = Hit in *H. sapiens* and *C. milli*
White = Not in *H. sapiens*

31 Hippo signaling pathway

31.1 Human Pathway: HSA04390

31.2 Number of Hits: 71

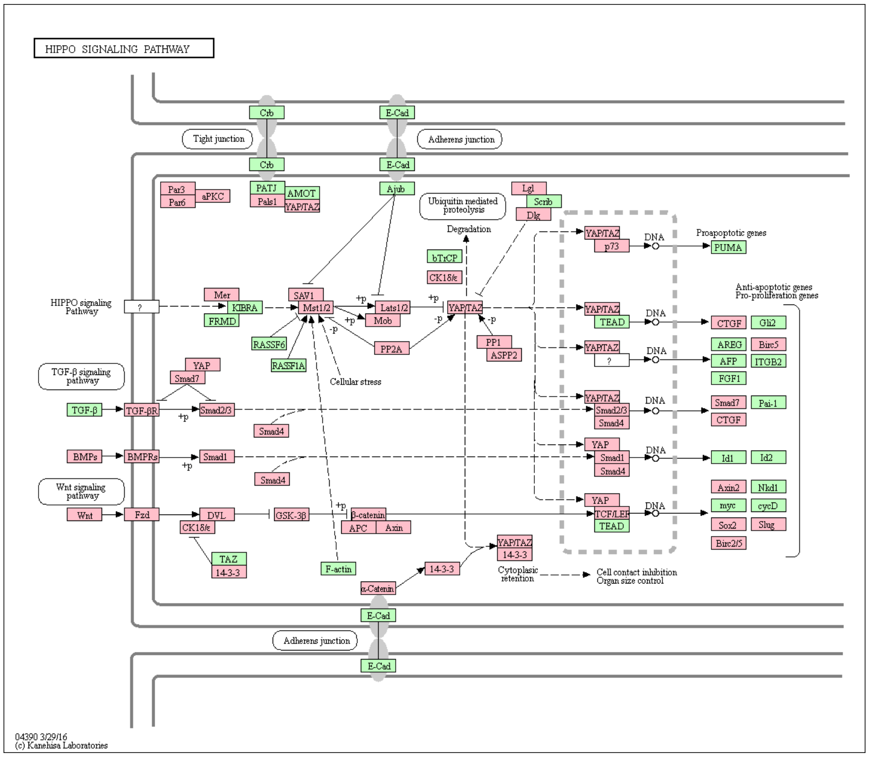

31.3 Legend:

RBH-Blast at 60% Identity + 50% Coverage
Green = Hit in *H. sapiens*
Red = Hit in *H. sapiens* and *C. milli*
White = Not in *H. sapiens*

32 Sphingolipid signaling pathway

32.1 Human Pathway: HSA04071

32.2 Number of Hits: 70

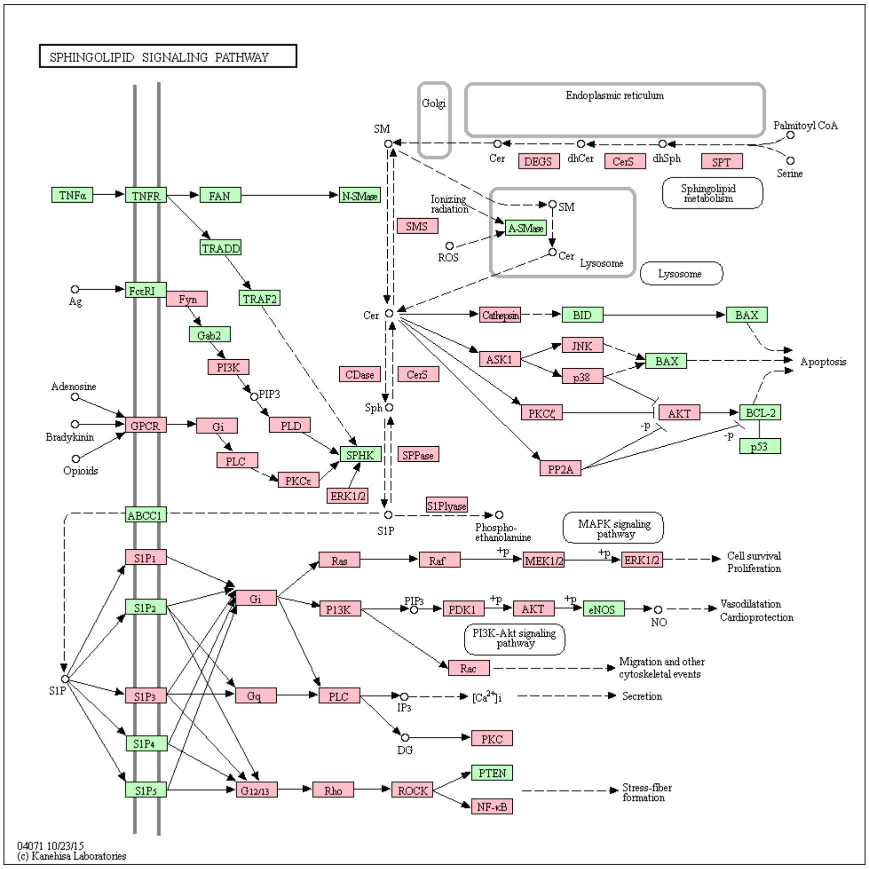

32.3 Legend:

RBH-Blast at 60% Identity + 50% Coverage
Green = Hit in *H. sapiens*
Red = Hit in *H. sapiens* and *C. milli*
White = Not in *H. sapiens*

33 Adrenergic signaling in cardiomyocytes

33.1 Human Pathway: HSA04261

33.2 Number of Hits: 70

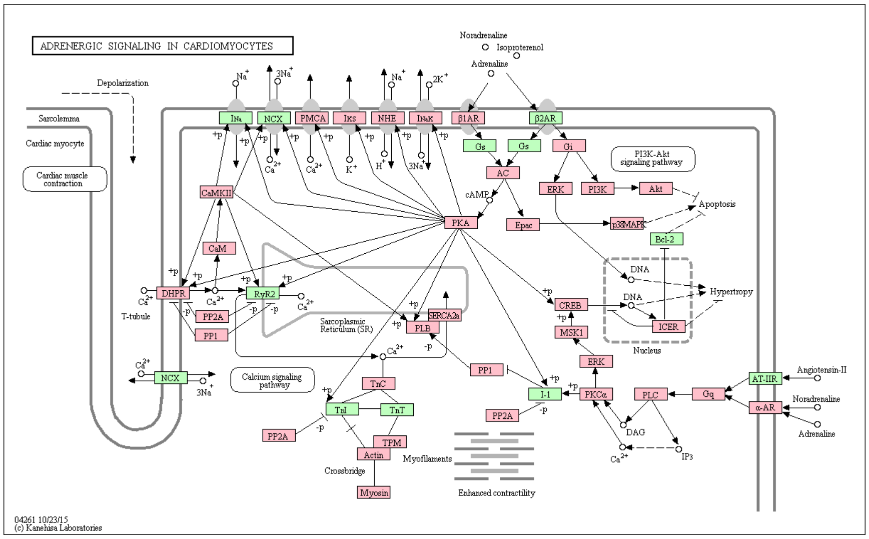

33.3 Legend:

RBH-Blast at 60% Identity + 50% Coverage
Green = Hit in *H. sapiens*
Red = Hit in *H. sapiens* and *C. milli*
White = Not in *H. sapiens*

34 cGMP-PKG signaling pathway

34.1 Human Pathway: HSA04022

34.2 Number of Hits: 70

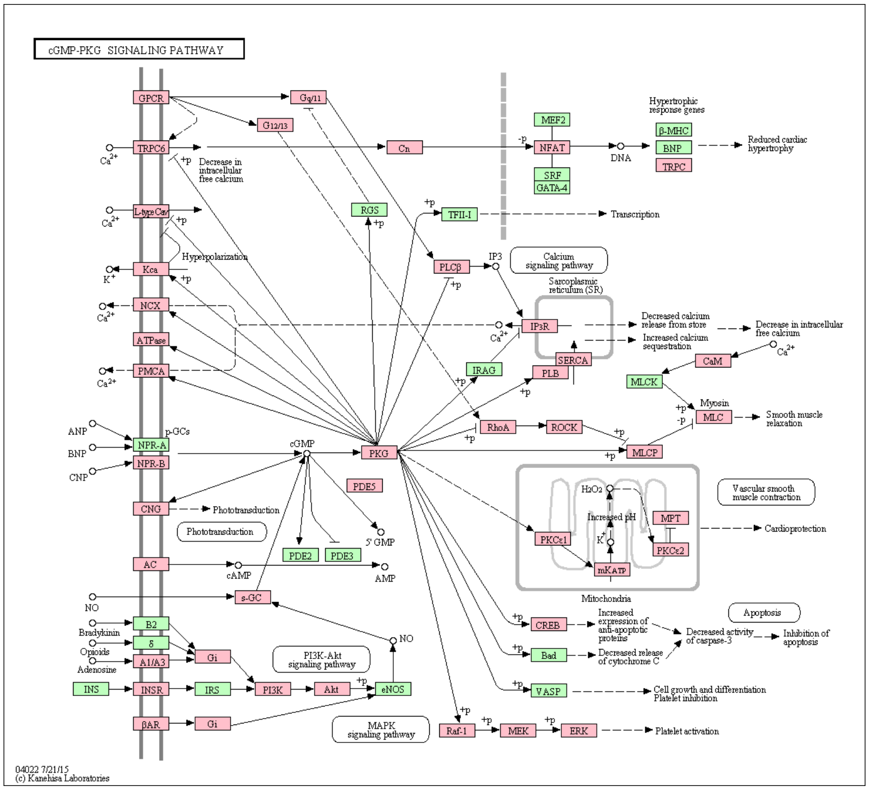

34.3 Legend:

RBH-Blast at 60% Identity + 50% Coverage
Green = Hit in *H. sapiens*
Red = Hit in *H. sapiens* and *C. milli*
White = Not in *H. sapiens*

35 Phospholipase D signaling pathway

35.1 Human Pathway: HSA04072

35.2 Number of Hits: 69

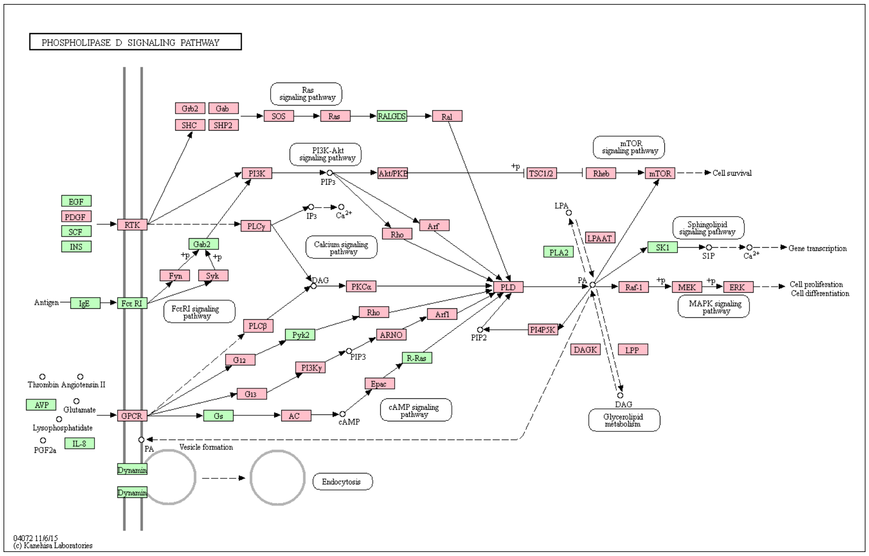

35.3 Legend:

RBH-Blast at 60% Identity + 50% Coverage
Green = Hit in *H. sapiens*
Red = Hit in *H. sapiens* and *C. milli*
White = Not in *H. sapiens*

36 Dopaminergic synapse

36.1 Human Pathway: HSA04728

36.2 Number of Hits: 69

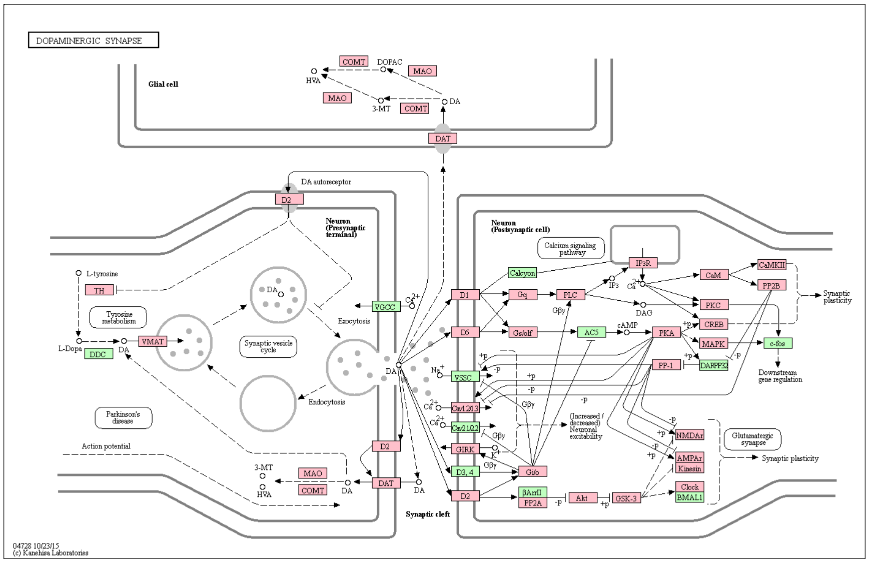

36.3 Legend:

RBH-Blast at 60% Identity + 50% Coverage
Green = Hit in *H. sapiens*
Red = Hit in *H. sapiens* and *C. milli*
White = Not in *H. sapiens*

37 MicroRNAs in cancer

37.1 Human Pathway: HSA05206

37.2 Number of Hits: 67

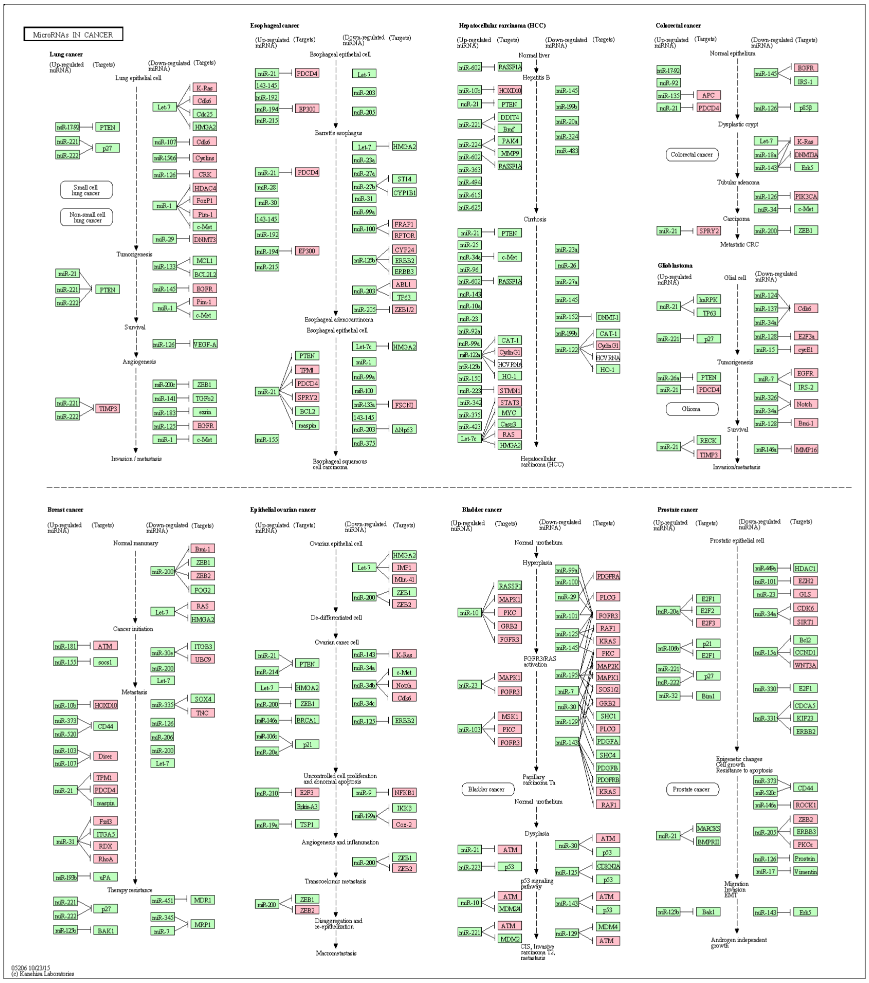

37.3 Legend:

RBH-Blast at 60% Identity + 50% Coverage
Green = Hit in *H. sapiens*
Red = Hit in *H. sapiens* and *C. milli*
White = Not in *H. sapiens*

38 Insulin signaling pathway

38.1 Human Pathway: HSA04910

38.2 Number of Hits: 66

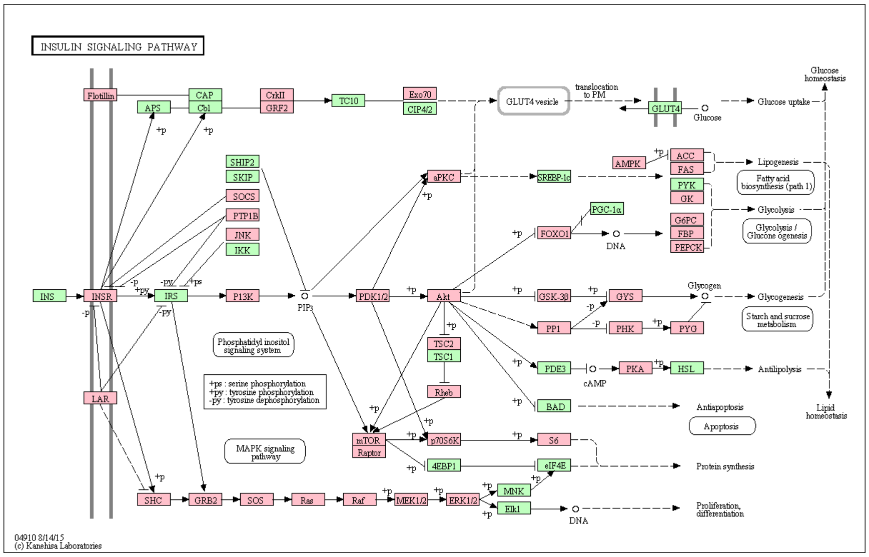

38.3 Legend:

RBH-Blast at 60% Identity + 50% Coverage
Green = Hit in *H. sapiens*
Red = Hit in *H. sapiens* and *C. milli*
White = Not in *H. sapiens*

39 Neurotrophin signaling pathway

39.1 Human Pathway: HSA04722

39.2 Number of Hits: 65

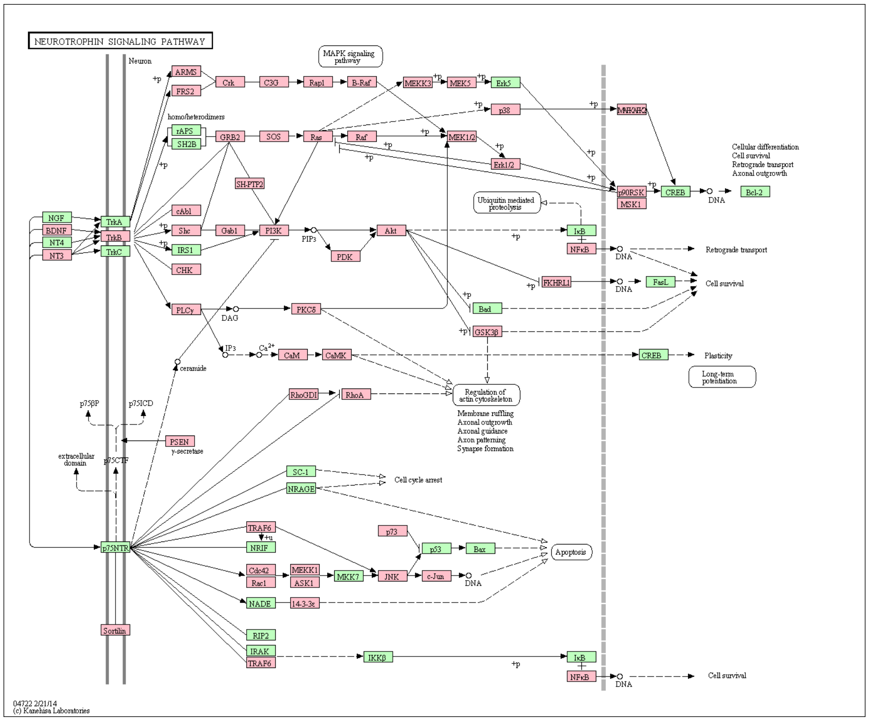

39.3 Legend:

RBH-Blast at 60% Identity + 50% Coverage
Green = Hit in *H. sapiens*
Red = Hit in *H. sapiens* and *C. milli*
White = Not in *H. sapiens*

40 Parkinson's disease

40.1 Human Pathway: HSA05012

40.2 Number of Hits: 64

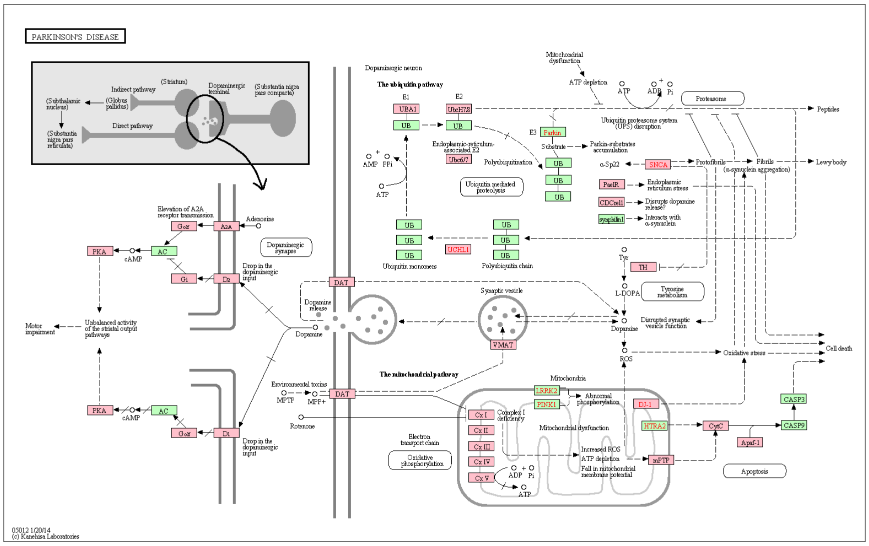

40.3 Legend:

RBH-Blast at 60% Identity + 50% Coverage
Green = Hit in *H. sapiens*
Red = Hit in *H. sapiens* and *C. milli*
White = Not in *H. sapiens*

41 Retrograde endocannabinoid signaling

41.1 Human Pathway: HSA04723

41.2 Number of Hits: 64

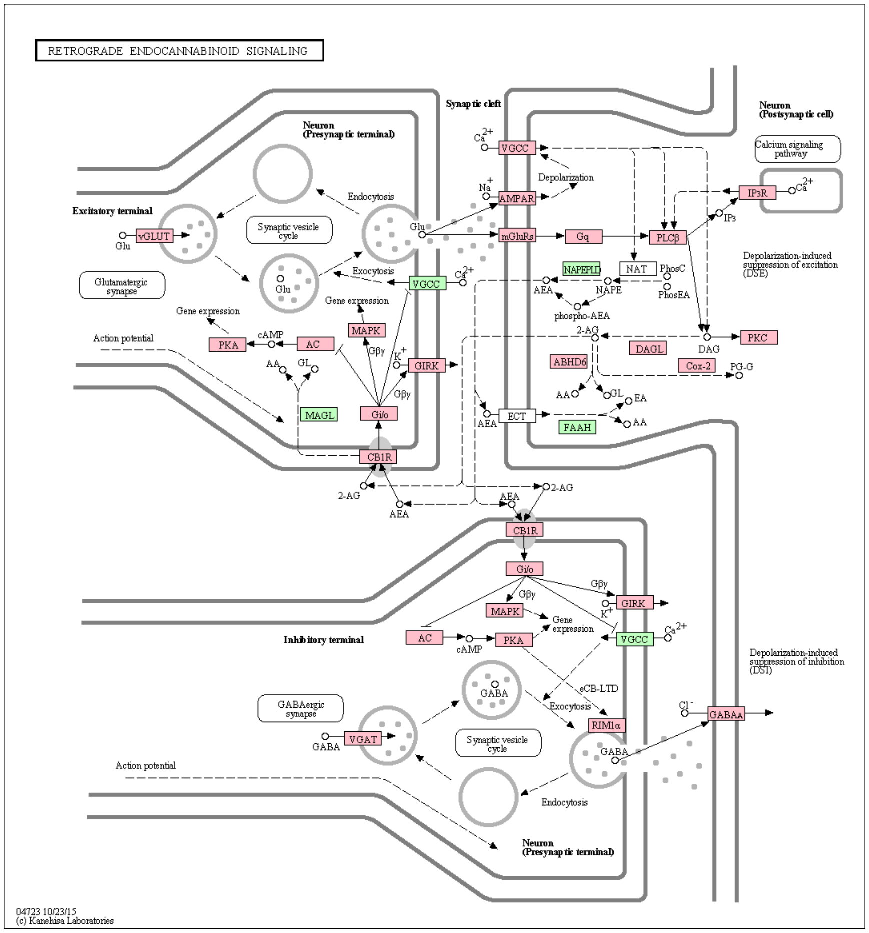

41.3 Legend:

RBH-Blast at 60% Identity + 50% Coverage
Green = Hit in *H. sapiens*
Red = Hit in *H. sapiens* and *C. milli*
White = Not in *H. sapiens*

42 AMPK signaling pathway

42.1 Human Pathway: HSA04152

42.2 Number of Hits: 64

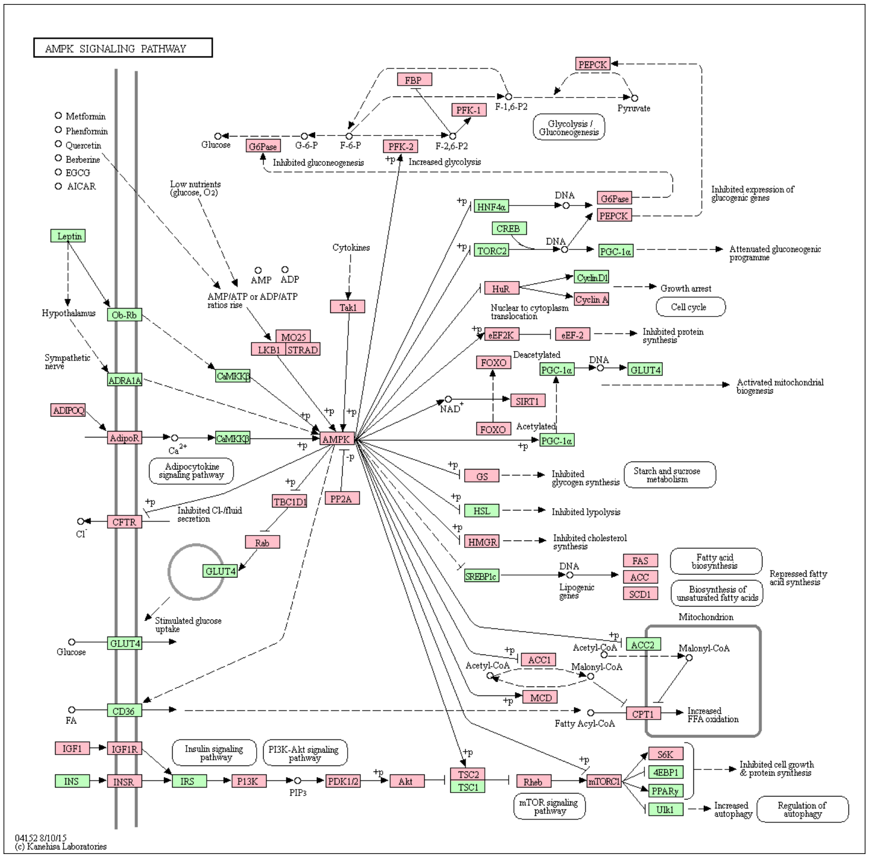

42.3 Legend:

RBH-Blast at 60% Identity + 50% Coverage
Green = Hit in *H. sapiens*
Red = Hit in *H. sapiens* and *C. milli*
White = Not in *H. sapiens*

43 Thyroid hormone signaling pathway

43.1 Human Pathway: HSA04919

43.2 Number of Hits: 64

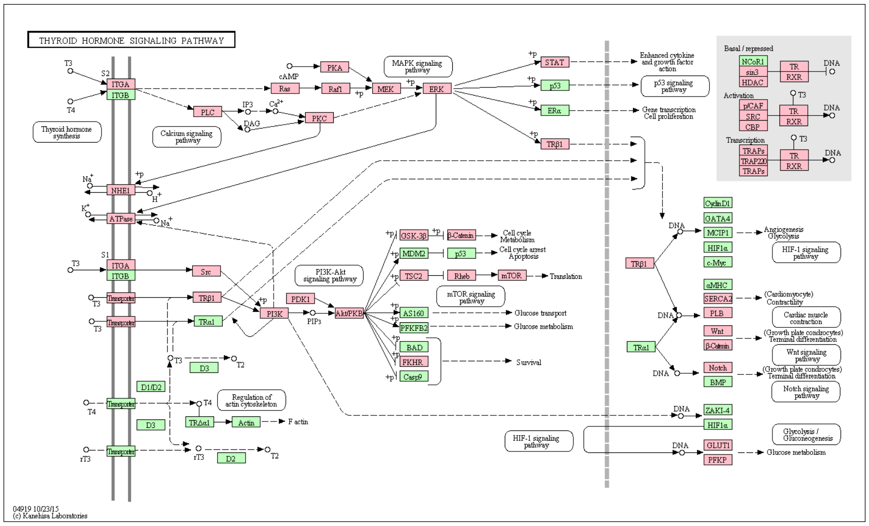

43.3 Legend:

RBH-Blast at 60% Identity + 50% Coverage
Green = Hit in *H. sapiens*
Red = Hit in *H. sapiens* and *C. milli*
White = Not in *H. sapiens*

44 Cell cycle

44.1 Human Pathway: HSA04110

44.2 Number of Hits: 63

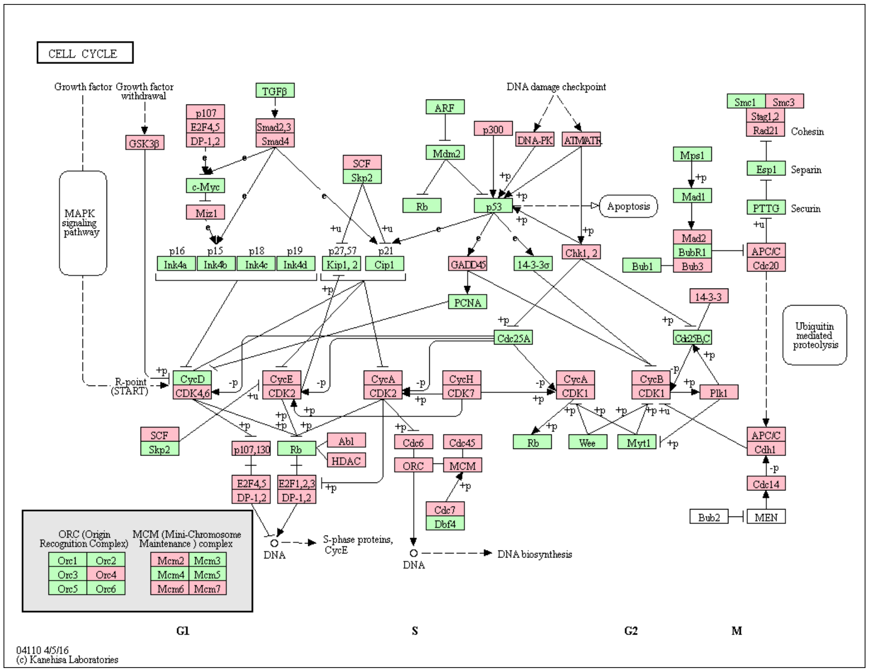

44.3 Legend:

RBH-Blast at 60% Identity + 50% Coverage
Green = Hit in *H. sapiens*
Red = Hit in *H. sapiens* and *C. milli*
White = Not in *H. sapiens*

45 Carbon metabolism

45.1 Human Pathway: HSA01200

45.2 Number of Hits: 62

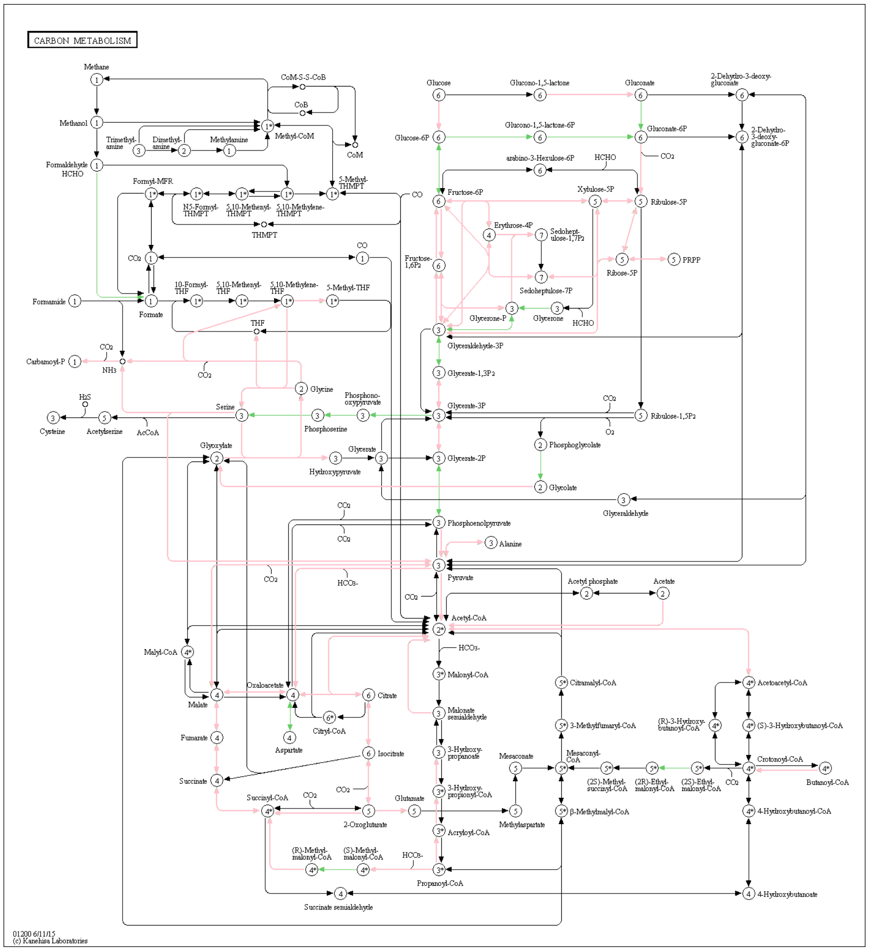

45.3 Legend:

RBH-Blast at 60% Identity + 50% Coverage
Green = Hit in *H. sapiens*
Red = Hit in *H. sapiens* and *C. milli*
White = Not in *H. sapiens*

46 Glutamatergic synapse

46.1 Human Pathway: HSA04724

46.2 Number of Hits: 62

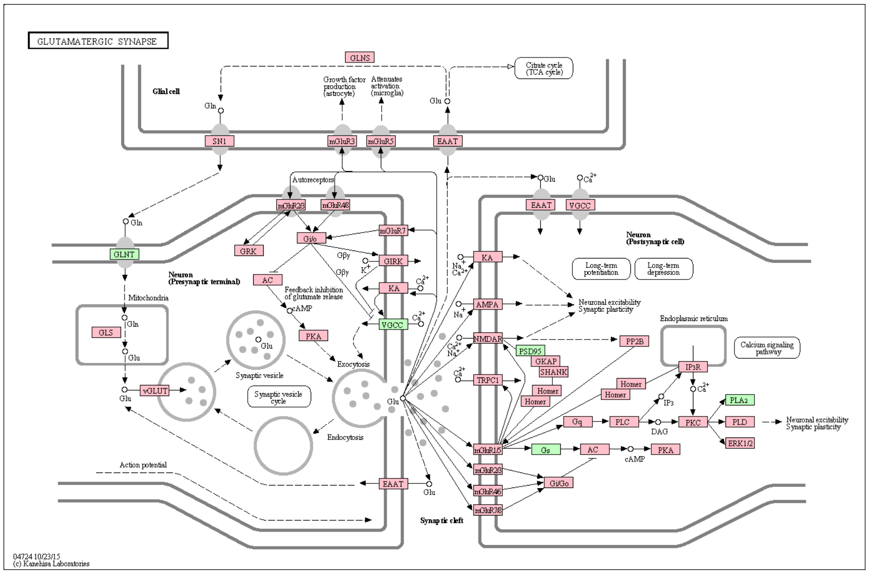

46.3 Legend:

RBH-Blast at 60% Identity + 50% Coverage
Green = Hit in *H. sapiens*
Red = Hit in *H. sapiens* and *C. milli*
White = Not in *H. sapiens*

47 Non-alcoholic fatty liver disease (NAFLD)

47.1 Human Pathway: HSA04932

47.2 Number of Hits: 61

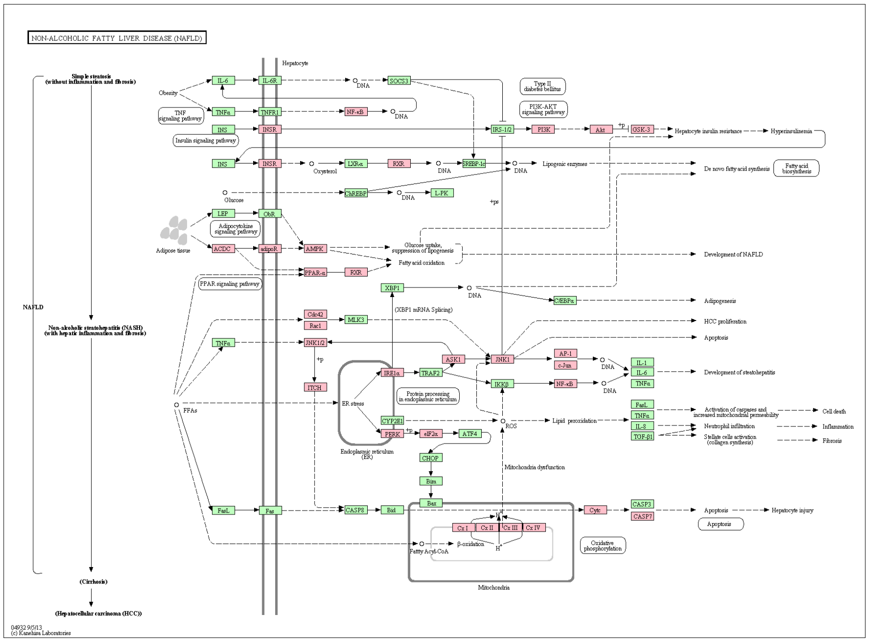

47.3 Legend:

RBH-Blast at 60% Identity + 50% Coverage
Green = Hit in *H. sapiens*
Red = Hit in *H. sapiens* and *C. milli*
White = Not in *H. sapiens*

48 Oxidative phosphorylation

48.1 Human Pathway: HSA00190

48.2 Number of Hits: 60

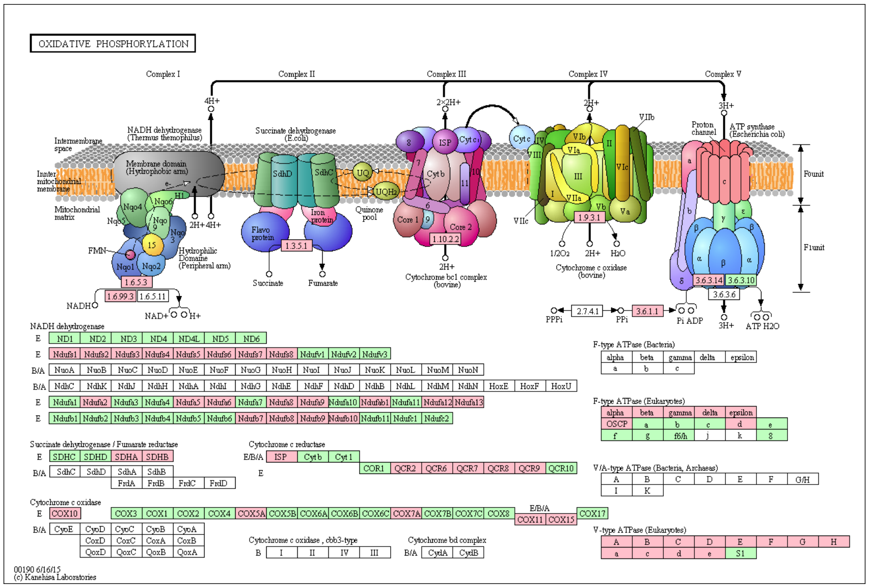

48.3 Legend:

RBH-Blast at 60% Identity + 50% Coverage
Green = Hit in *H. sapiens*
Red = Hit in *H. sapiens* and *C. milli*
White = Not in *H. sapiens*

49 Tight junction

49.1 Human Pathway: HSA04530

49.2 Number of Hits: 60

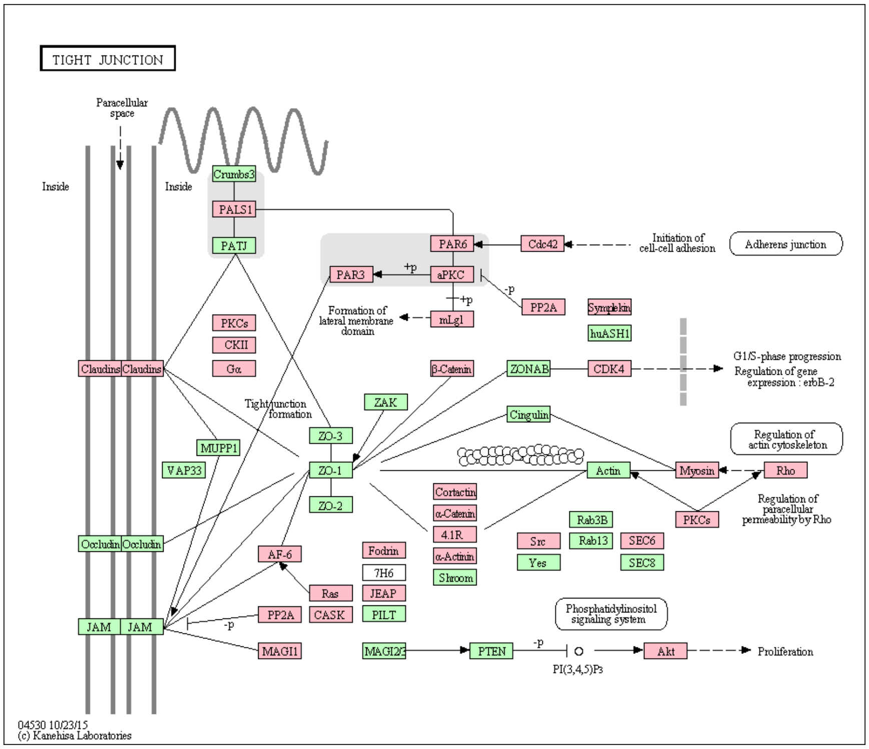

49.3 Legend:

RBH-Blast at 60% Identity + 50% Coverage
Green = Hit in *H. sapiens*
Red = Hit in *H. sapiens* and *C. milli*
White = Not in *H. sapiens*

50 Alcoholism

50.1 Human Pathway: HSA05034

50.2 Number of Hits: 57

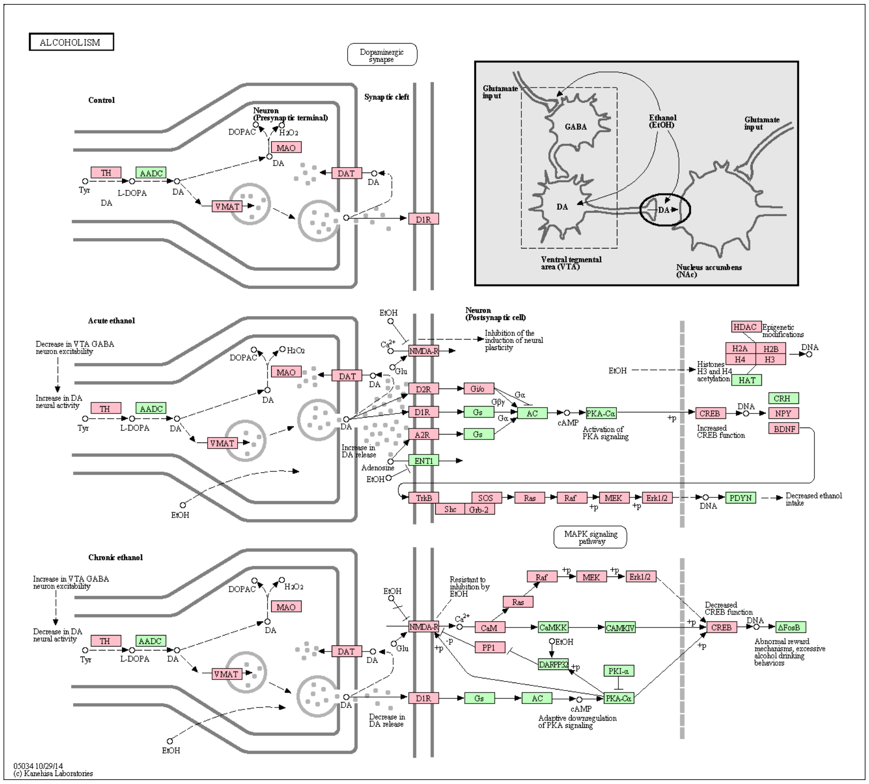

50.3 Legend:

RBH-Blast at 60% Identity + 50% Coverage
Green = Hit in *H. sapiens*
Red = Hit in *H. sapiens* and *C. milli*
White = Not in *H. sapiens*

51 Pyrimidine metabolism

51.1 Human Pathway: HSA00240

51.2 Number of Hits: 56

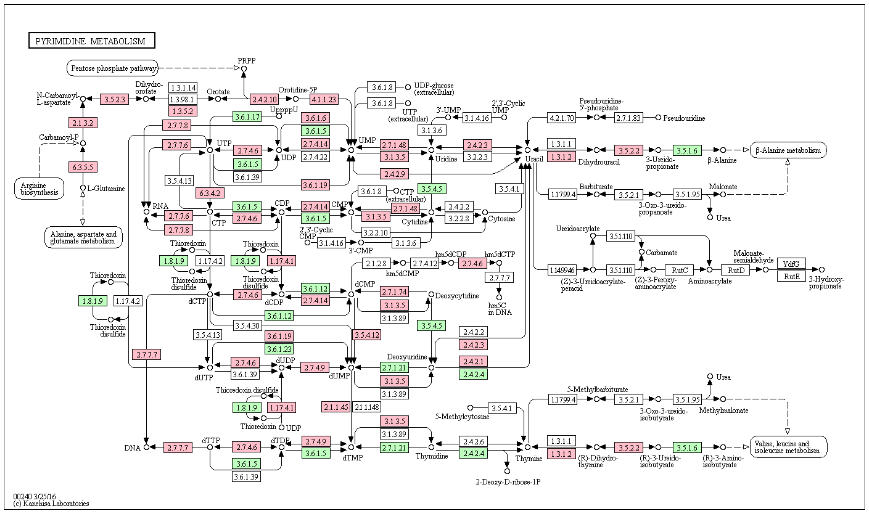

51.3 Legend:

RBH-Blast at 60% Identity + 50% Coverage
Green = Hit in *H. sapiens*
Red = Hit in *H. sapiens* and *C. milli*
White = Not in *H. sapiens*

52 Cholinergic synapse

52.1 Human Pathway: HSA04725

52.2 Number of Hits: 56

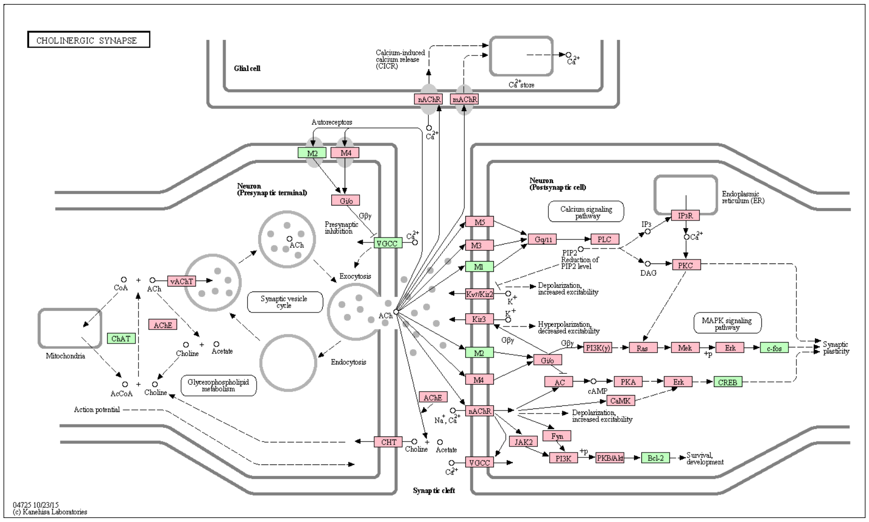

52.3 Legend:

RBH-Blast at 60% Identity + 50% Coverage
Green = Hit in *H. sapiens*
Red = Hit in *H. sapiens* and *C. milli*
White = Not in *H. sapiens*

53 Platelet activation

53.1 Human Pathway: HSA04611

53.2 Number of Hits: 55

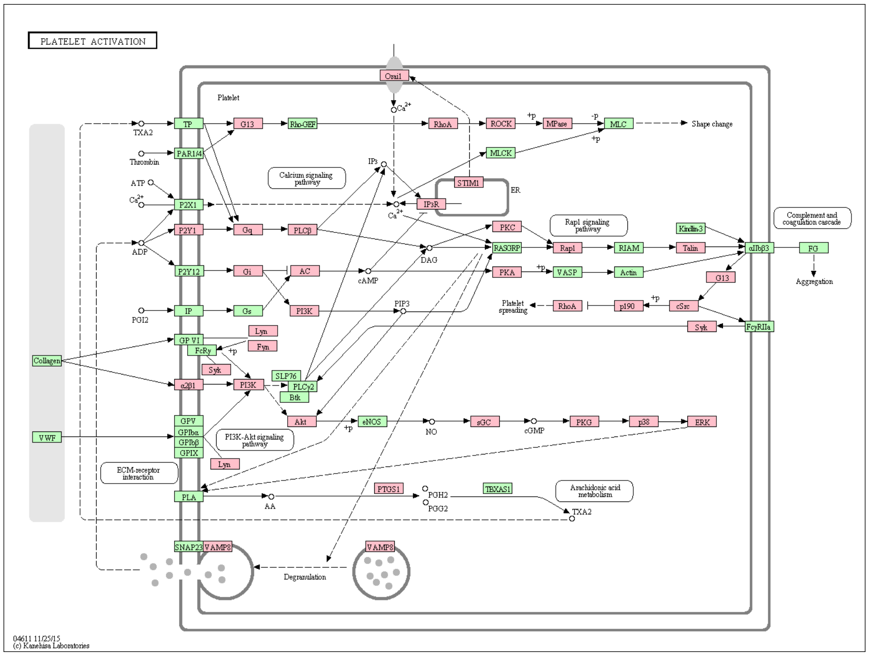

53.3 Legend:

RBH-Blast at 60% Identity + 50% Coverage
Green = Hit in *H. sapiens*
Red = Hit in *H. sapiens* and *C. milli*
White = Not in *H. sapiens*

54 Serotonergic synapse

54.1 Human Pathway: HSA04726

54.2 Number of Hits: 54

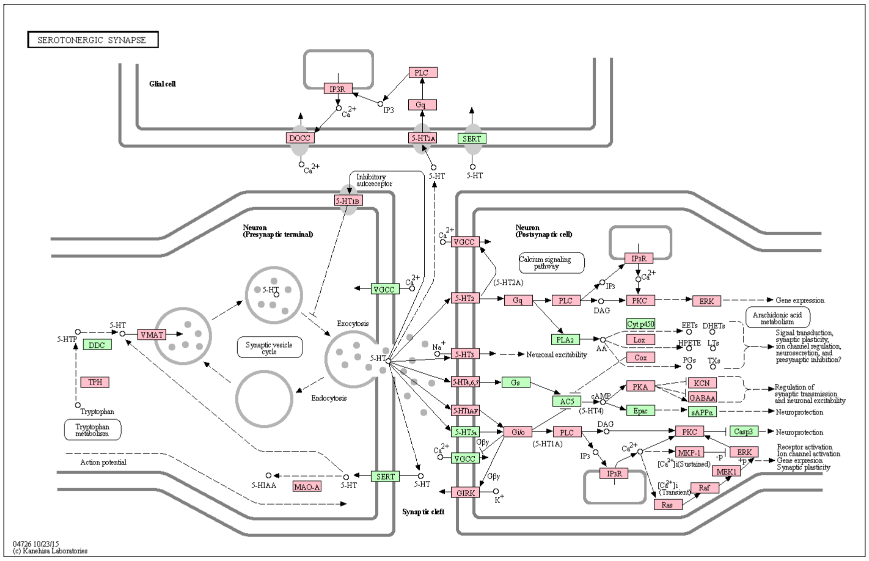

54.3 Legend:

RBH-Blast at 60% Identity + 50% Coverage
Green = Hit in *H. sapiens*
Red = Hit in *H. sapiens* and *C. milli*
White = Not in *H. sapiens*

55 Oocyte meiosis

55.1 Human Pathway: HSA04114

55.2 Number of Hits: 53

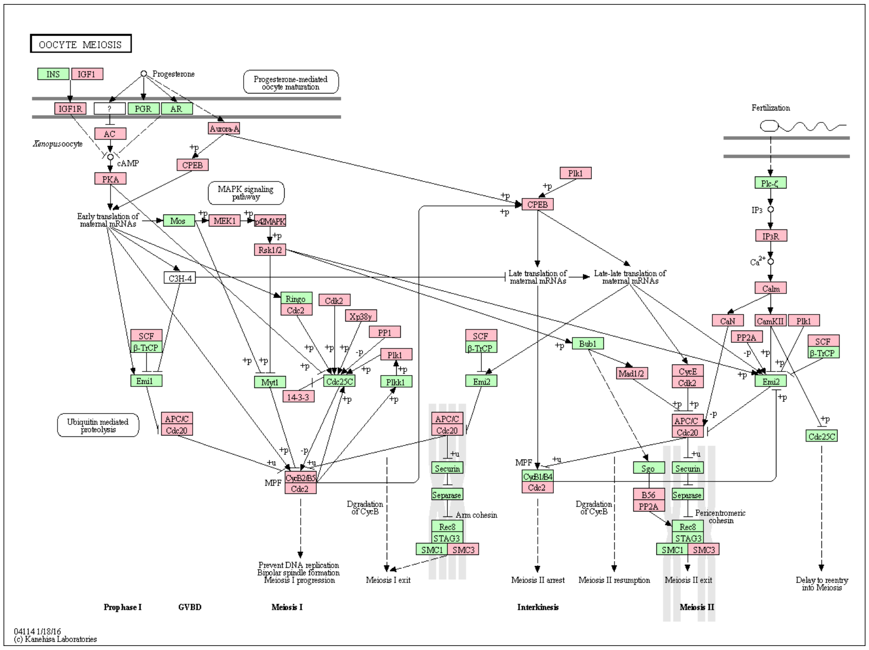

55.3 Legend:

RBH-Blast at 60% Identity + 50% Coverage
Green = Hit in *H. sapiens*
Red = Hit in *H. sapiens* and *C. milli*
White = Not in *H. sapiens*

56 Leukocyte transendothelial migration

56.1 Human Pathway: HSA04670

56.2 Number of Hits: 53

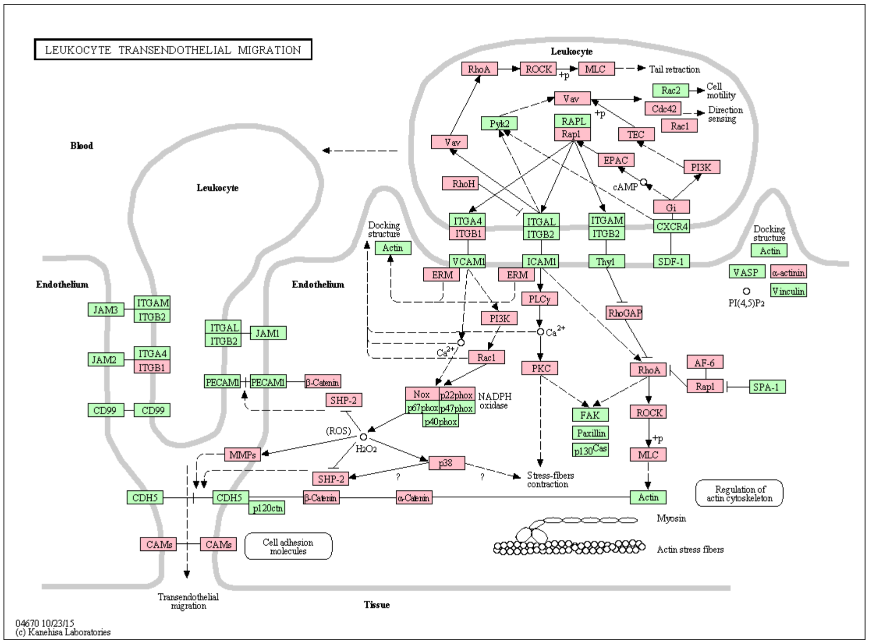

56.3 Legend:

RBH-Blast at 60% Identity + 50% Coverage
Green = Hit in *H. sapiens*
Red = Hit in *H. sapiens* and *C. milli*
White = Not in *H. sapiens*

57 Progesterone-mediated oocyte maturation

57.1 Human Pathway: HSA04914

57.2 Number of Hits: 52

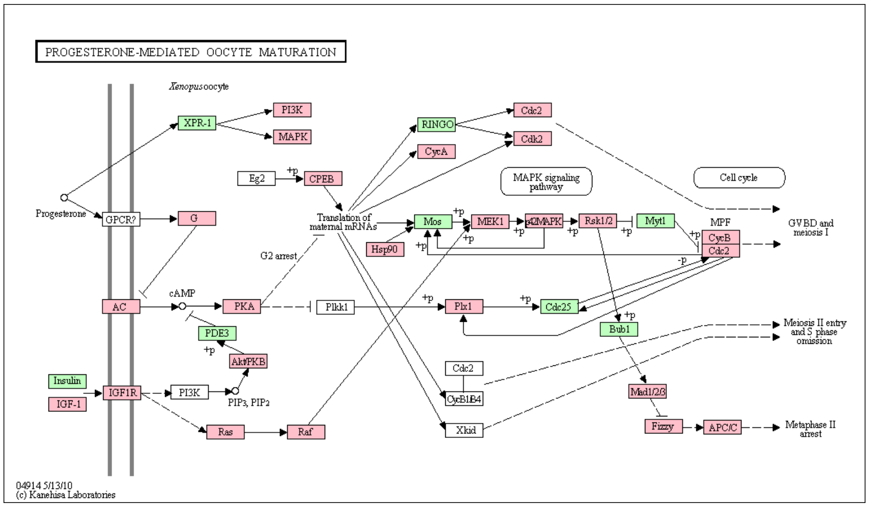

57.3 Legend:

RBH-Blast at 60% Identity + 50% Coverage
Green = Hit in *H. sapiens*
Red = Hit in *H. sapiens* and *C. milli*
White = Not in *H. sapiens*

58 Hepatitis C

58.1 Human Pathway: HSA05160

58.2 Number of Hits: 52

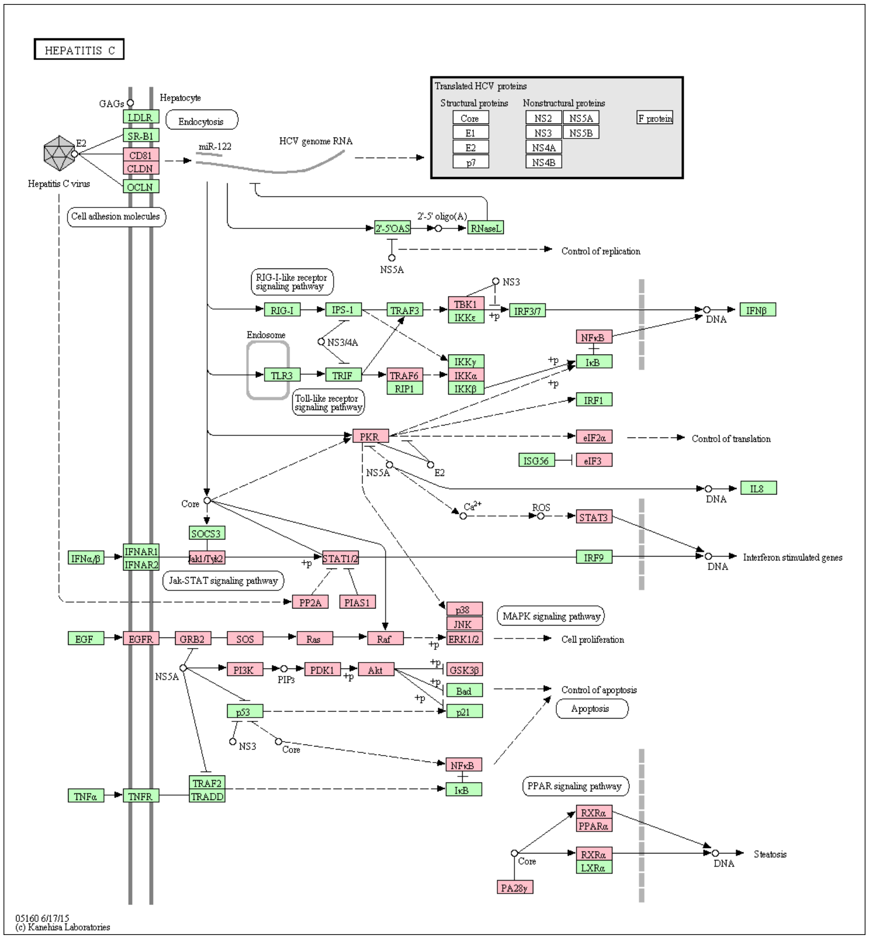

58.3 Legend:

RBH-Blast at 60% Identity + 50% Coverage
Green = Hit in *H. sapiens*
Red = Hit in *H. sapiens* and *C. milli*
White = Not in *H. sapiens*

59 Apoptosis

59.1 Human Pathway: HSA04210

59.2 Number of Hits: 52

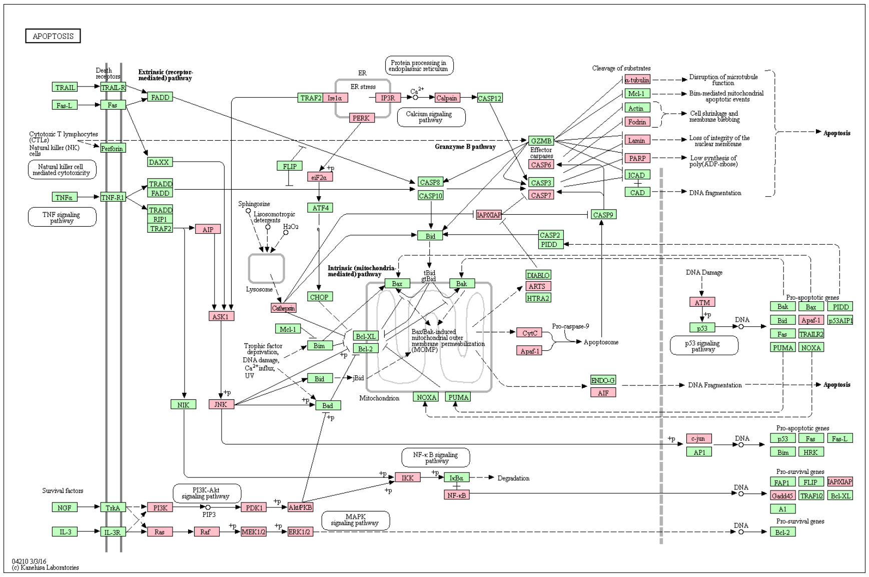

59.3 Legend:

RBH-Blast at 60% Identity + 50% Coverage
Green = Hit in *H. sapiens*
Red = Hit in *H. sapiens* and *C. milli*
White = Not in *H. sapiens*

60 Circadian entrainment

60.1 Human Pathway: HSA04713

60.2 Number of Hits: 51

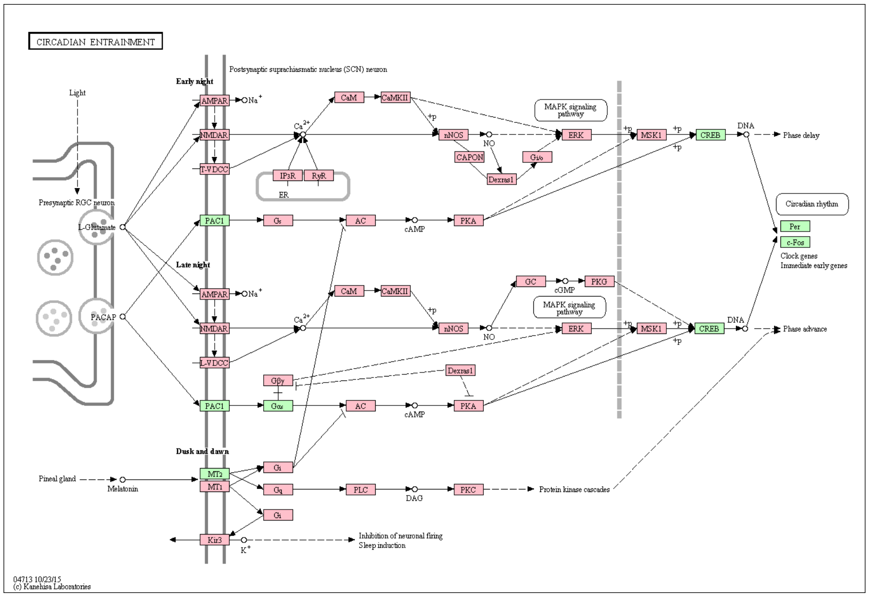

60.3 Legend:

RBH-Blast at 60% Identity + 50% Coverage
Green = Hit in *H. sapiens*
Red = Hit in *H. sapiens* and *C. milli*
White = Not in *H. sapiens*

61 Lysosome

61.1 Human Pathway: HSA04142

61.2 Number of Hits: 51

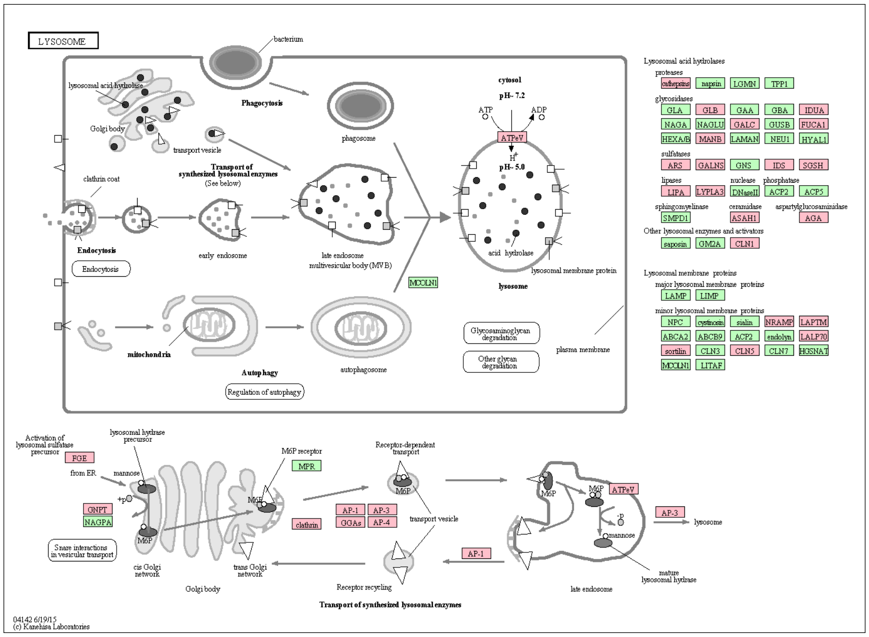

61.3 Legend:

RBH-Blast at 60% Identity + 50% Coverage
Green = Hit in *H. sapiens*
Red = Hit in *H. sapiens* and *C. milli*
White = Not in *H. sapiens*

62 Melanogenesis

62.1 Human Pathway: HSA04916

62.2 Number of Hits: 51

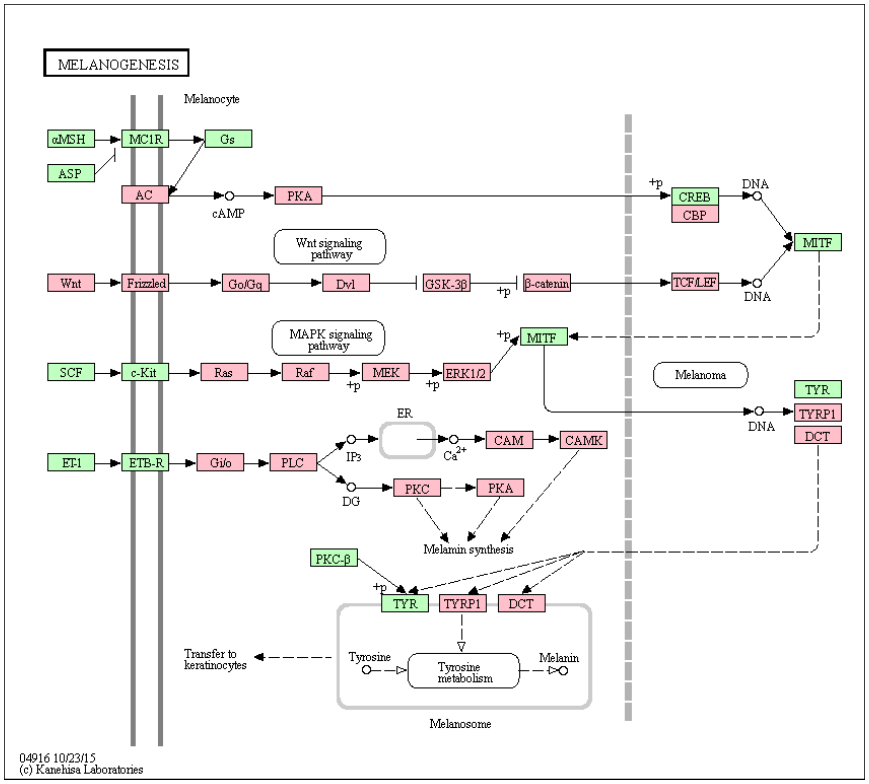

62.3 Legend:

RBH-Blast at 60% Identity + 50% Coverage
Green = Hit in *H. sapiens*
Red = Hit in *H. sapiens* and *C. milli*
White = Not in *H. sapiens*

63 Hepatitis B

63.1 Human Pathway: HSA05161

63.2 Number of Hits: 51

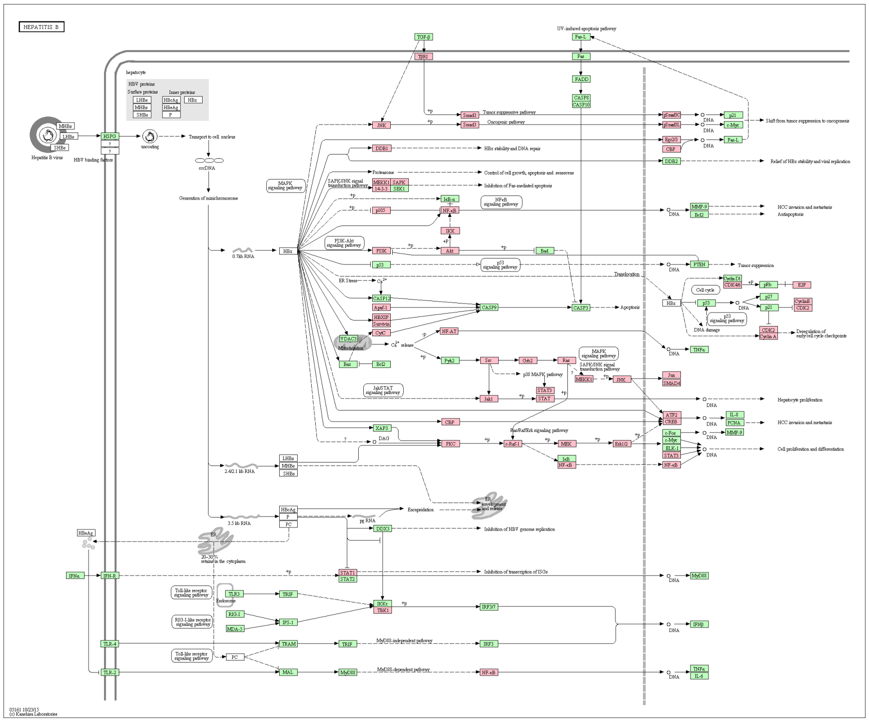

63.3 Legend:

RBH-Blast at 60% Identity + 50% Coverage
Green = Hit in *H. sapiens*
Red = Hit in *H. sapiens* and *C. milli*
White = Not in *H. sapiens*

64 Phagosome

64.1 Human Pathway: HSA04145

64.2 Number of Hits: 51

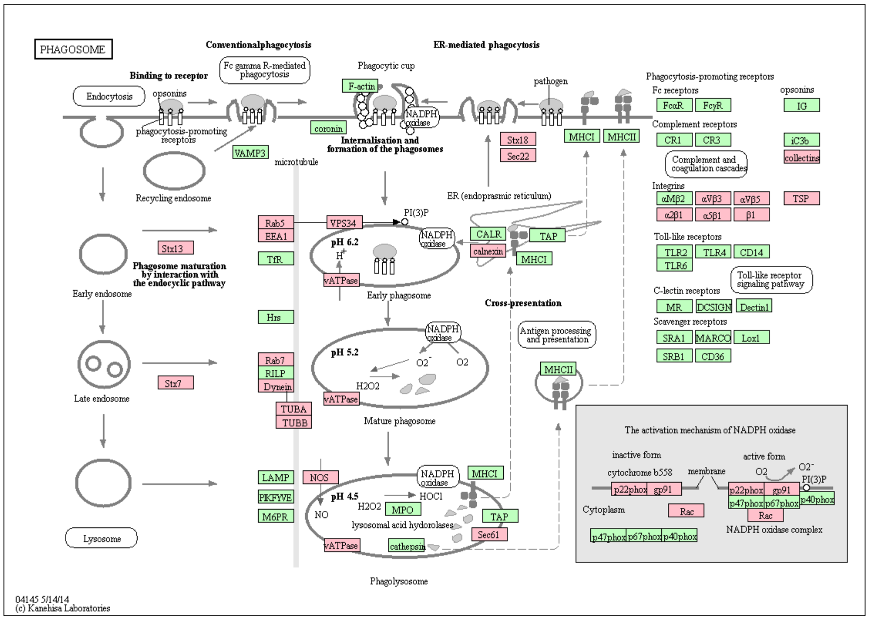

64.3 Legend:

RBH-Blast at 60% Identity + 50% Coverage
Green = Hit in *H. sapiens*
Red = Hit in *H. sapiens* and *C. milli*
White = Not in *H. sapiens*

65 Gap junction

65.1 Human Pathway: HSA04540

65.2 Number of Hits: 51

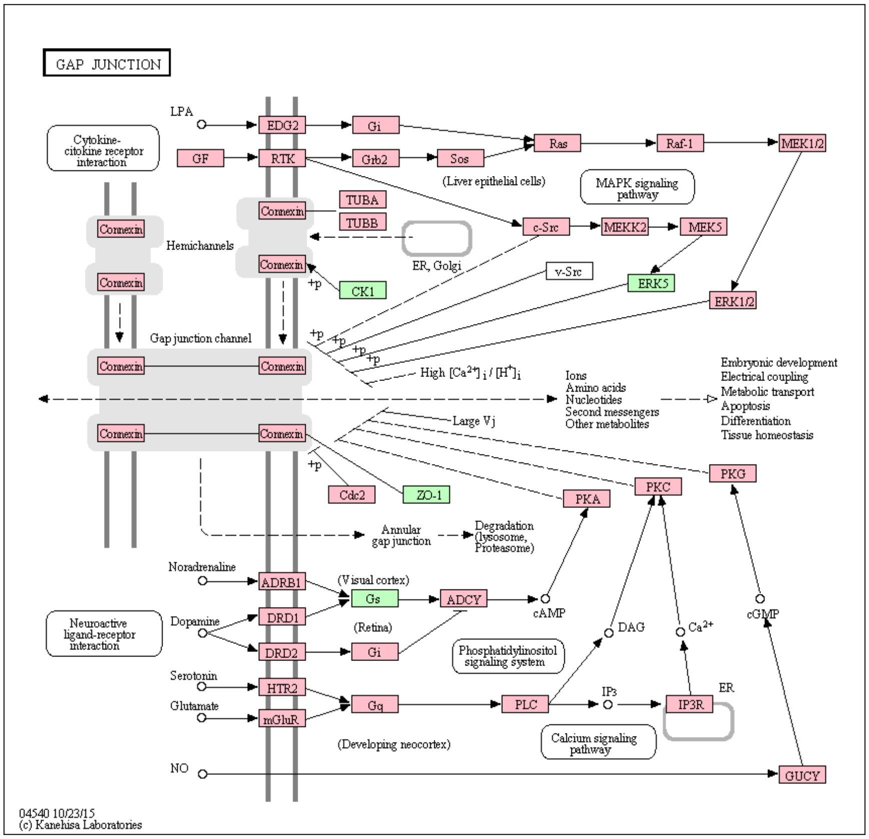

65.3 Legend:

RBH-Blast at 60% Identity + 50% Coverage
Green = Hit in *H. sapiens*
Red = Hit in *H. sapiens* and *C. milli*
White = Not in *H. sapiens*

66 Phosphatidylinositol signaling system

66.1 Human Pathway: HSA04070

66.2 Number of Hits: 50

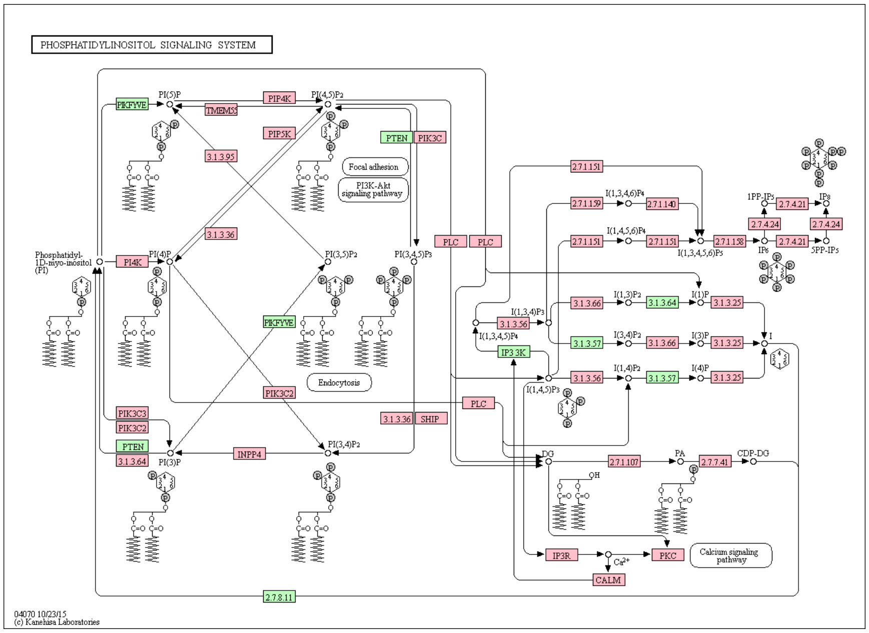

66.3 Legend:

RBH-Blast at 60% Identity + 50% Coverage
Green = Hit in *H. sapiens*
Red = Hit in *H. sapiens* and *C. milli*
White = Not in *H. sapiens*

67 Estrogen signaling pathway

67.1 Human Pathway: HSA04915

67.2 Number of Hits: 50

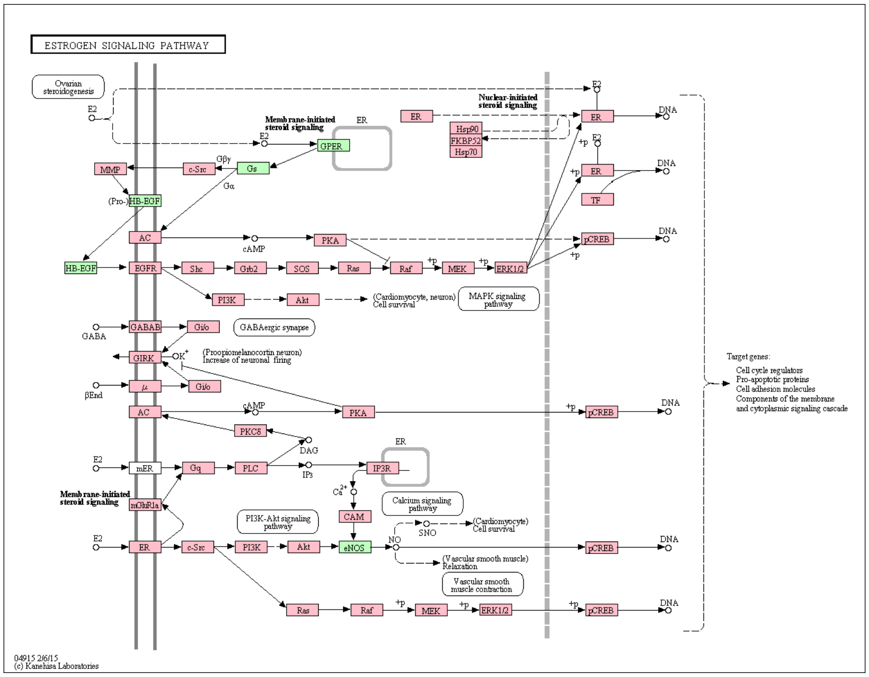

67.3 Legend:

RBH-Blast at 60% Identity + 50% Coverage
Green = Hit in *H. sapiens*
Red = Hit in *H. sapiens* and *C. milli*
White = Not in *H. sapiens*

68 Morphine addiction

68.1 Human Pathway: HSA05032

68.2 Number of Hits: 50

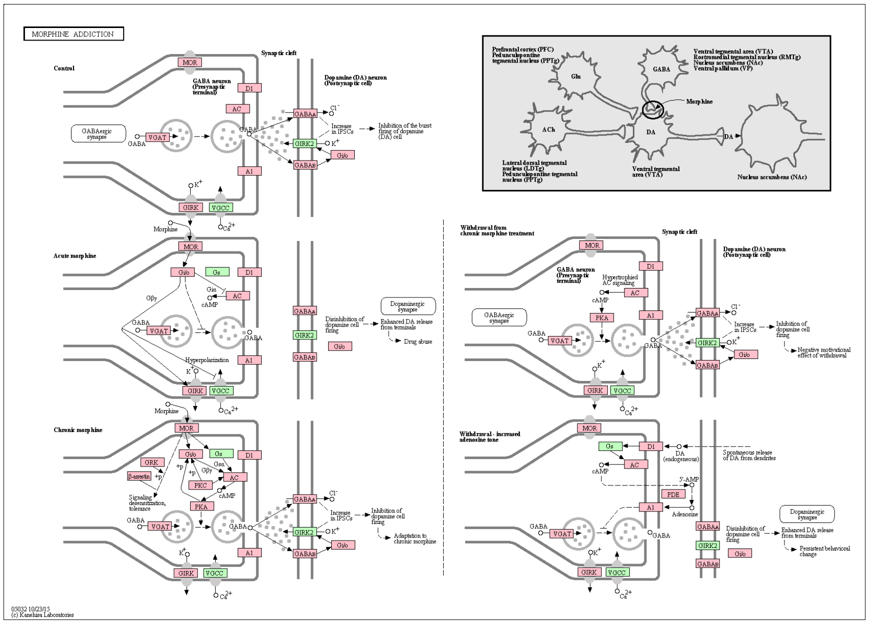

68.3 Legend:

RBH-Blast at 60% Identity + 50% Coverage
Green = Hit in *H. sapiens*
Red = Hit in *H. sapiens* and *C. milli*
White = Not in *H. sapiens*

69 Choline metabolism in cancer

69.1 Human Pathway: HSA05231

69.2 Number of Hits: 50

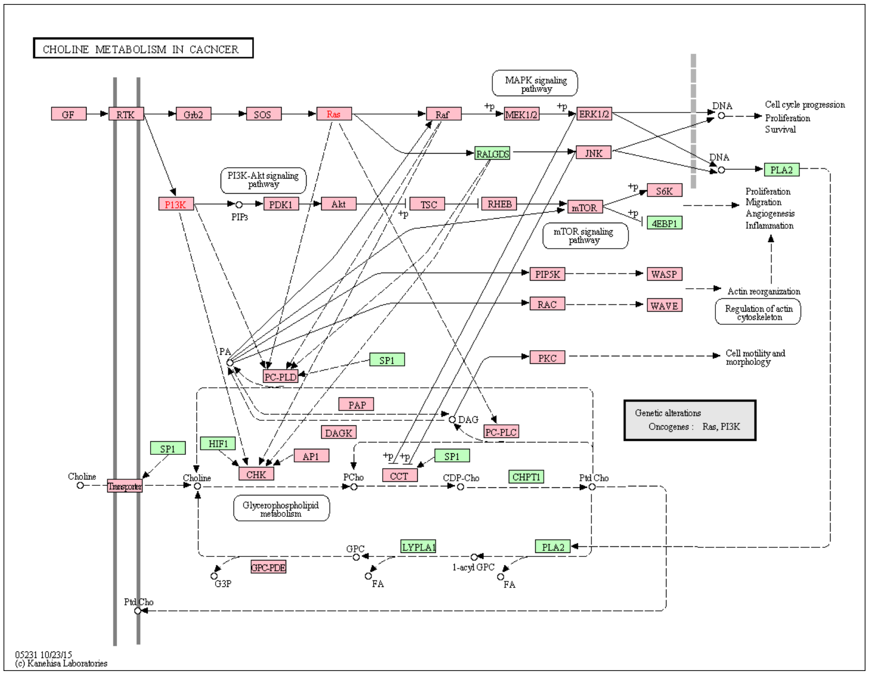

69.3 Legend:

RBH-Blast at 60% Identity + 50% Coverage
Green = Hit in *H. sapiens*
Red = Hit in *H. sapiens* and *C. milli*
White = Not in *H. sapiens*

70 Glucagon signaling pathway

70.1 Human Pathway: HSA04922

70.2 Number of Hits: 49

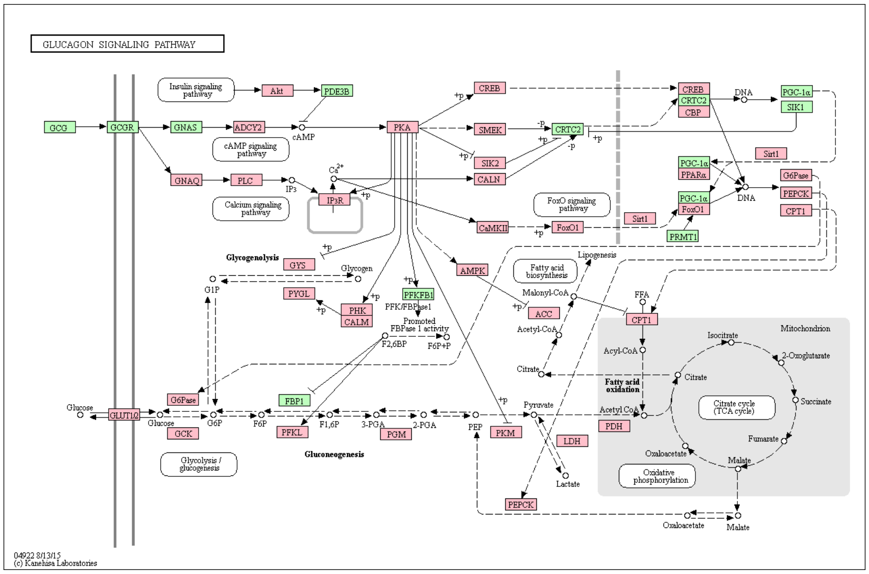

70.3 Legend:

RBH-Blast at 60% Identity + 50% Coverage
Green = Hit in *H. sapiens*
Red = Hit in *H. sapiens* and *C. milli*
White = Not in *H. sapiens*

71 Transcriptional misregulation in cancer

71.1 Human Pathway: HSA05202

71.2 Number of Hits: 48

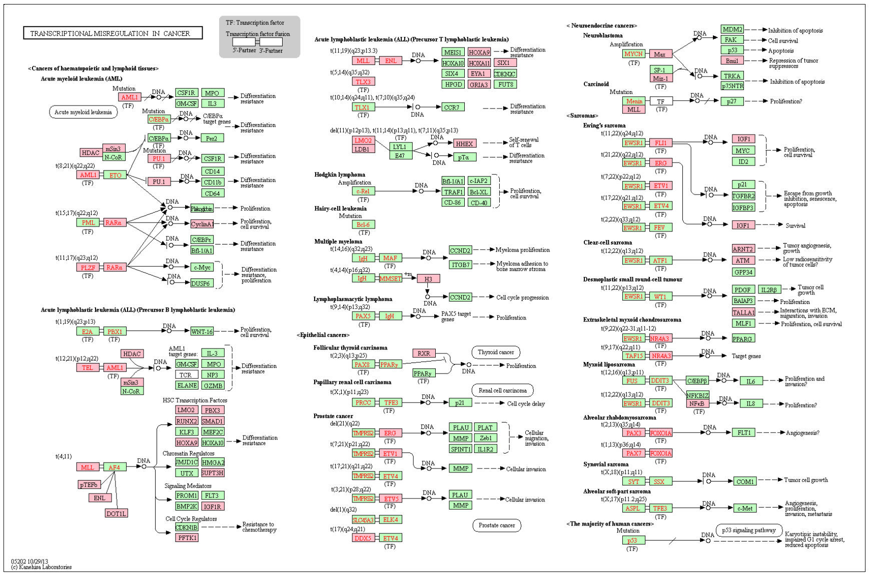

71.3 Legend:

RBH-Blast at 60% Identity + 50% Coverage
Green = Hit in *H. sapiens*
Red = Hit in *H. sapiens* and *C. milli*
White = Not in *H. sapiens*

72 Fc gamma R-mediated phagocytosis

72.1 Human Pathway: HSA04666

72.2 Number of Hits: 48

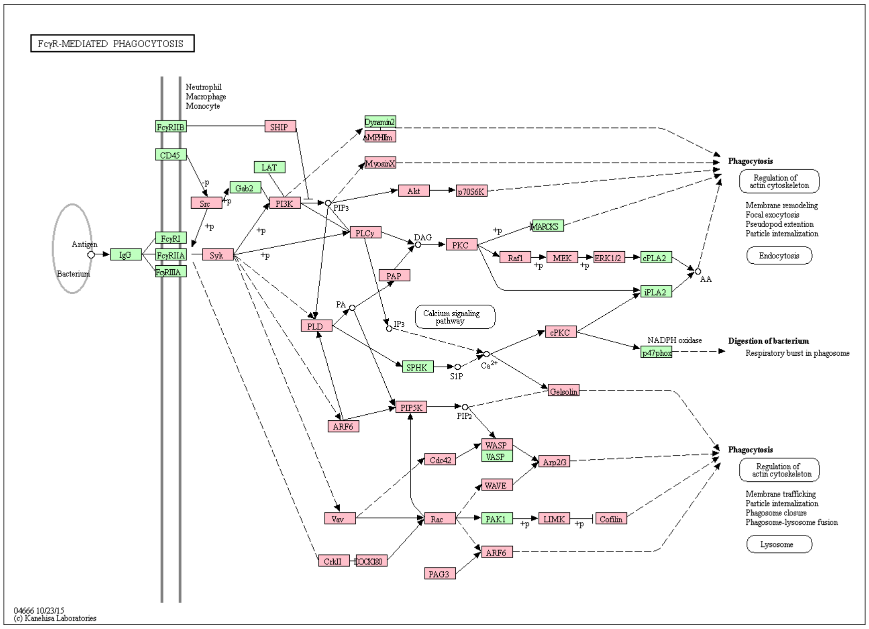

72.3 Legend:

RBH-Blast at 60% Identity + 50% Coverage
Green = Hit in *H. sapiens*
Red = Hit in *H. sapiens* and *C. milli*
White = Not in *H. sapiens*

73 GABAergic synapse

73.1 Human Pathway: HSA04727

73.2 Number of Hits: 48

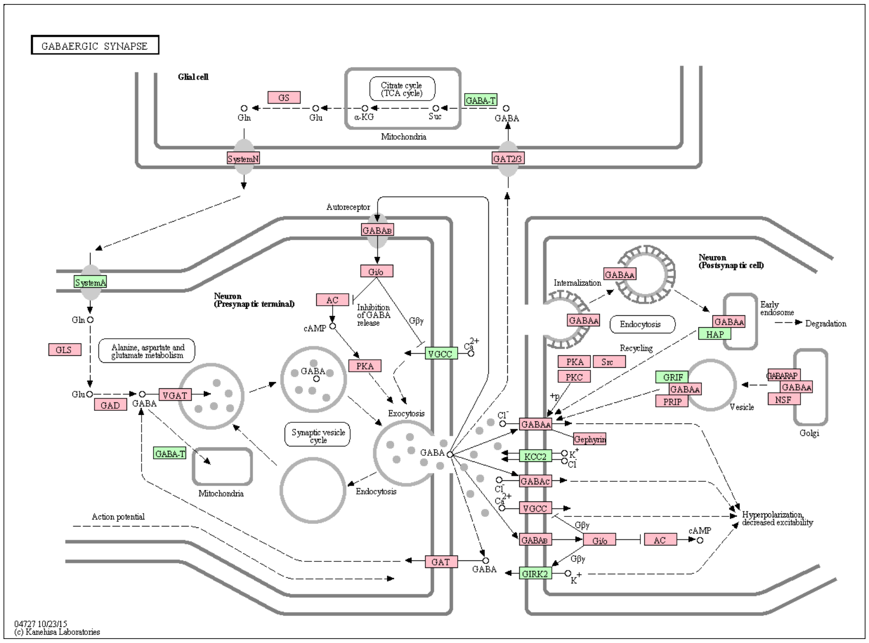

73.3 Legend:

RBH-Blast at 60% Identity + 50% Coverage
Green = Hit in *H. sapiens*
Red = Hit in *H. sapiens* and *C. milli*
White = Not in *H. sapiens*

74 T cell receptor signaling pathway

74.1 Human Pathway: HSA04660

74.2 Number of Hits: 48

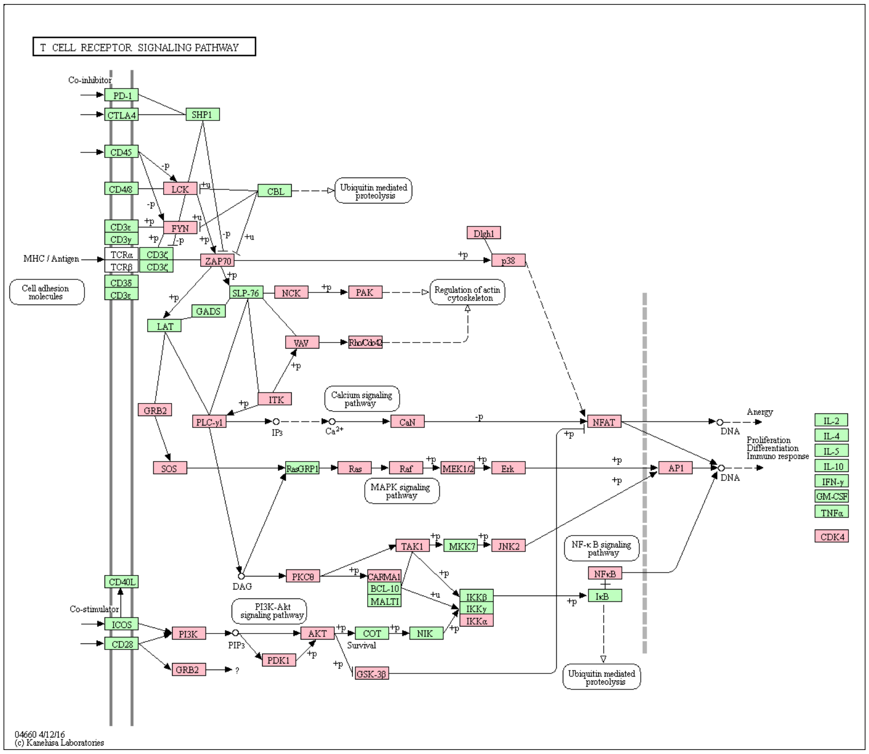

74.3 Legend:

RBH-Blast at 60% Identity + 50% Coverage
Green = Hit in *H. sapiens*
Red = Hit in *H. sapiens* and *C. milli*
White = Not in *H. sapiens*

75 Tuberculosis

75.1 Human Pathway: HSA05152

75.2 Number of Hits: 47

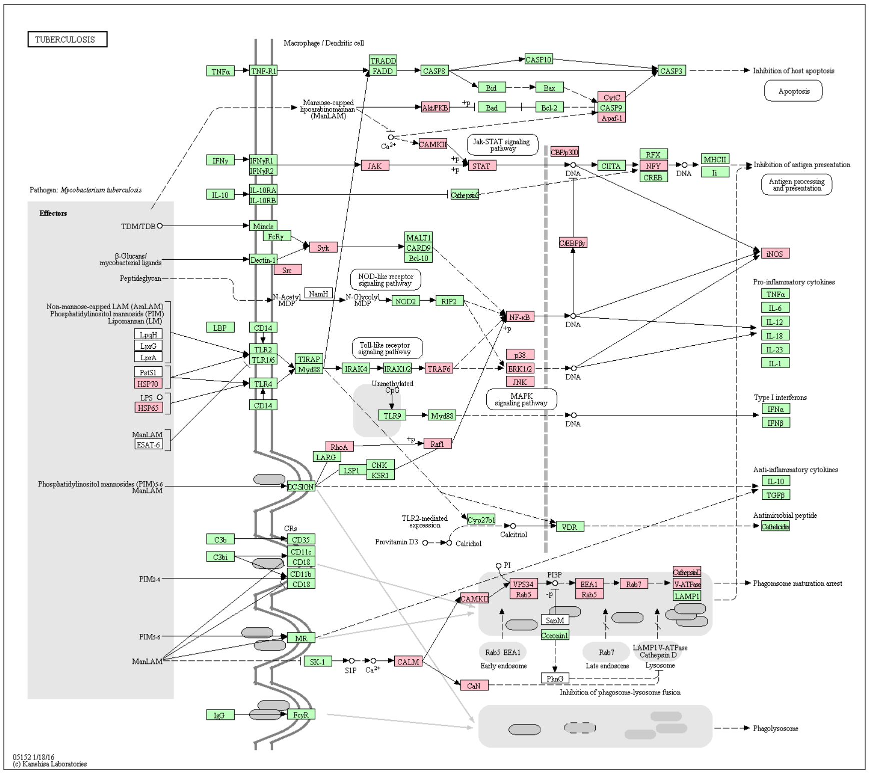

75.3 Legend:

RBH-Blast at 60% Identity + 50% Coverage
Green = Hit in *H. sapiens*
Red = Hit in *H. sapiens* and *C. milli*
White = Not in *H. sapiens*

76 AGE-RAGE signaling pathway in diabetic complications

76.1 Human Pathway: HSA04933

76.2 Number of Hits: 47

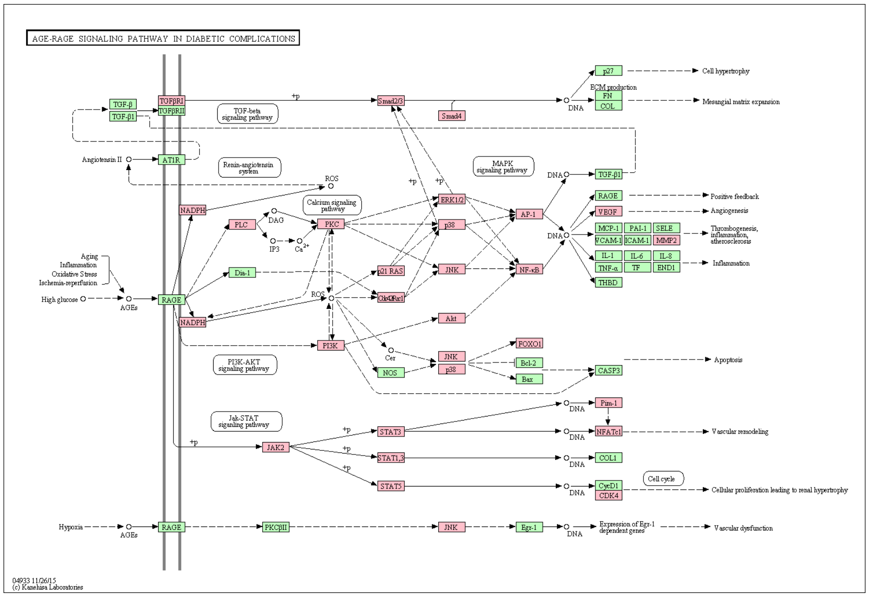

76.3 Legend:

RBH-Blast at 60% Identity + 50% Coverage
Green = Hit in *H. sapiens*
Red = Hit in *H. sapiens* and *C. milli*
White = Not in *H. sapiens*

77 GnRH signaling pathway

77.1 Human Pathway: HSA04912

77.2 Number of Hits: 47

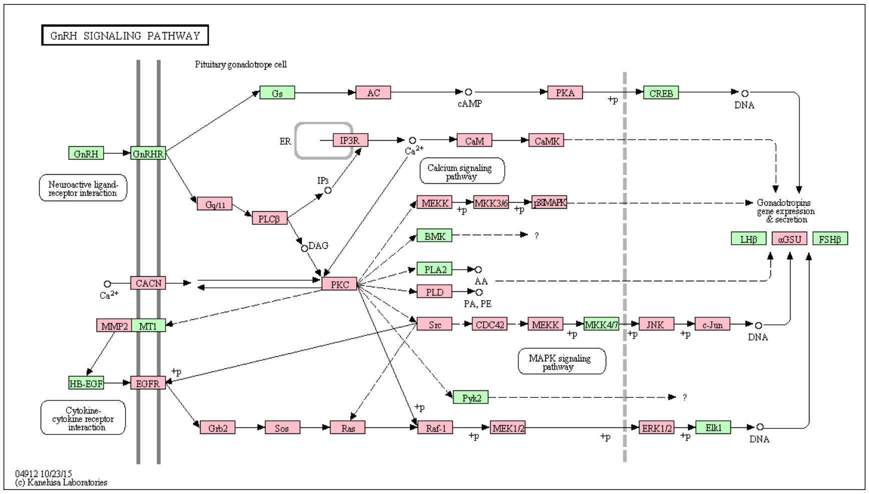

77.3 Legend:

RBH-Blast at 60% Identity + 50% Coverage
Green = Hit in *H. sapiens*
Red = Hit in *H. sapiens* and *C. milli*
White = Not in *H. sapiens*

78 Vascular smooth muscle contraction

78.1 Human Pathway: HSA04270

78.2 Number of Hits: 46

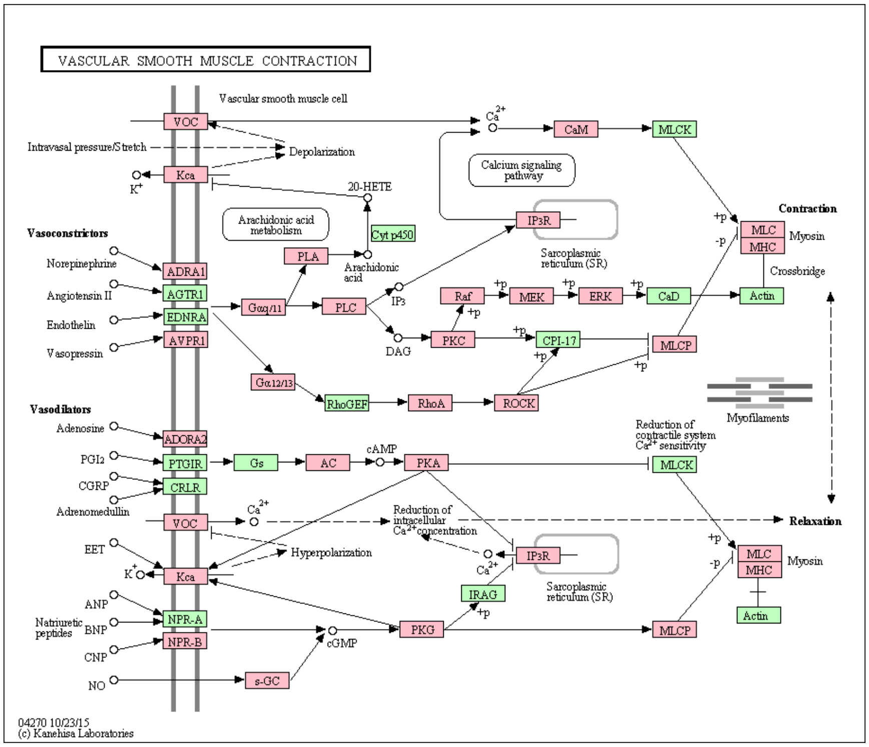

78.3 Legend:

RBH-Blast at 60% Identity + 50% Coverage
Green = Hit in *H. sapiens*
Red = Hit in *H. sapiens* and *C. milli*
White = Not in *H. sapiens*

79 Influenza A

79.1 Human Pathway: HSA05164

79.2 Number of Hits: 46

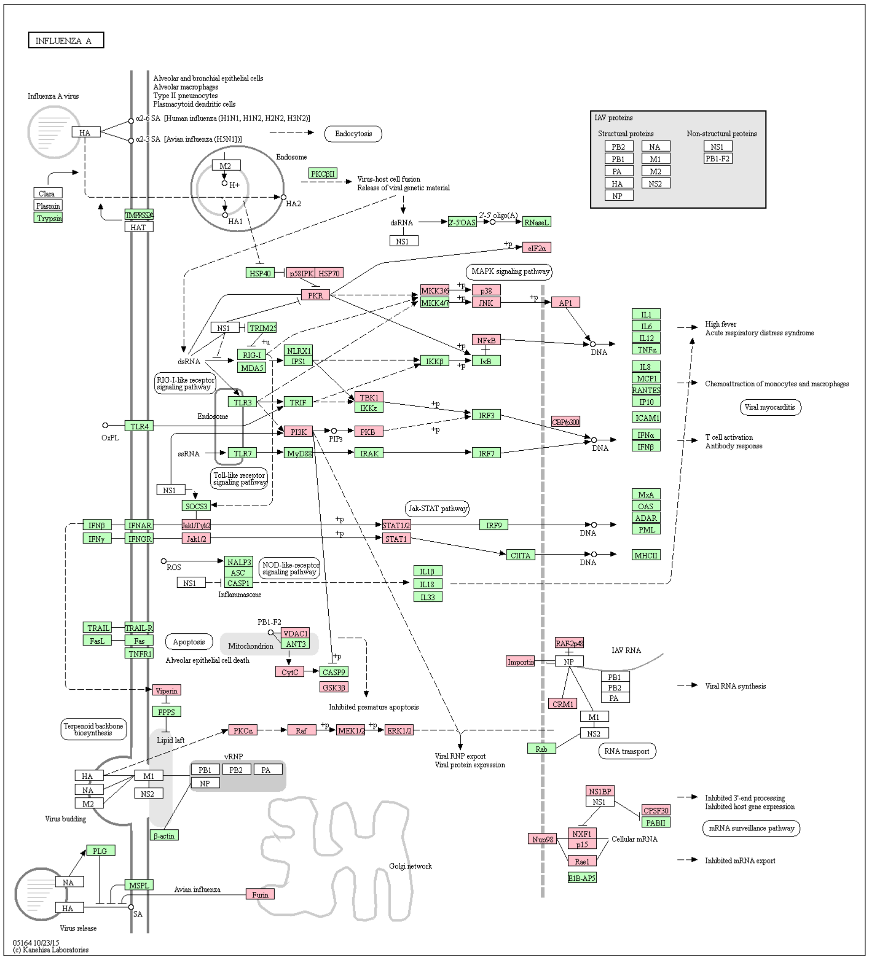

79.3 Legend:

RBH-Blast at 60% Identity + 50% Coverage
Green = Hit in *H. sapiens*
Red = Hit in *H. sapiens* and *C. milli*
White = Not in *H. sapiens*

80 Insulin resistance

80.1 Human Pathway: HSA04931

80.2 Number of Hits: 46

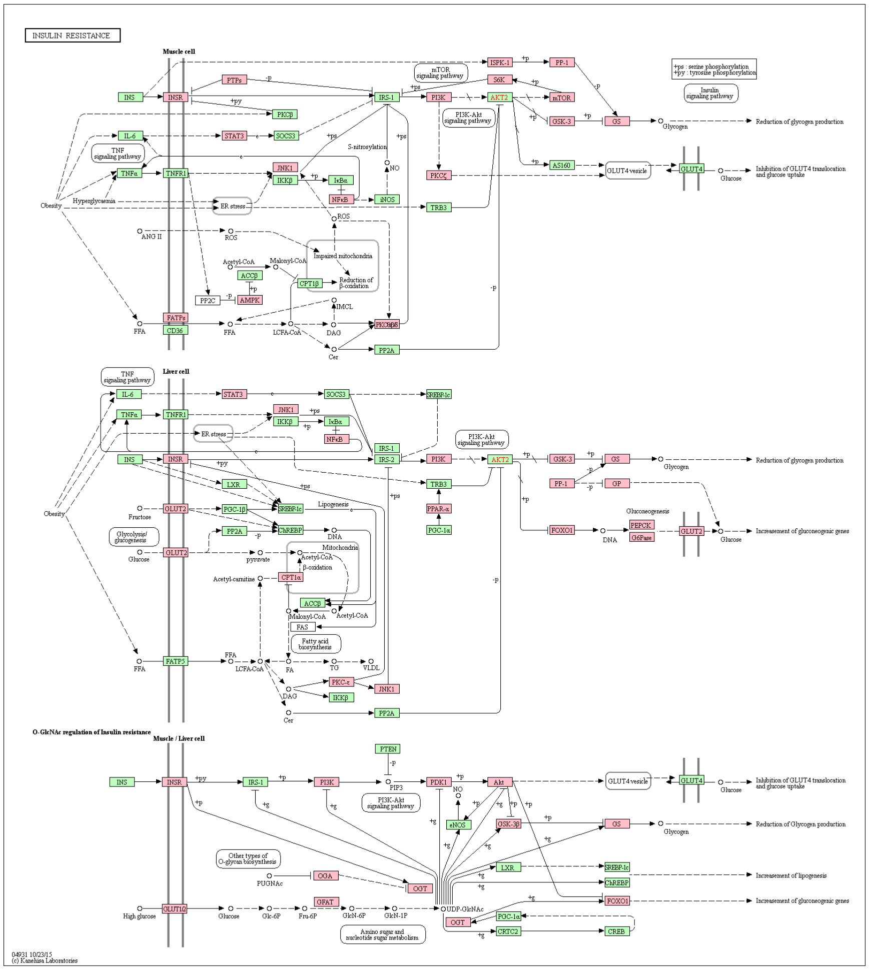

80.3 Legend:

RBH-Blast at 60% Identity + 50% Coverage
Green = Hit in *H. sapiens*
Red = Hit in *H. sapiens* and *C. milli*
White = Not in *H. sapiens*

81 mRNA surveillance pathway

81.1 Human Pathway: HSA03015

81.2 Number of Hits: 46

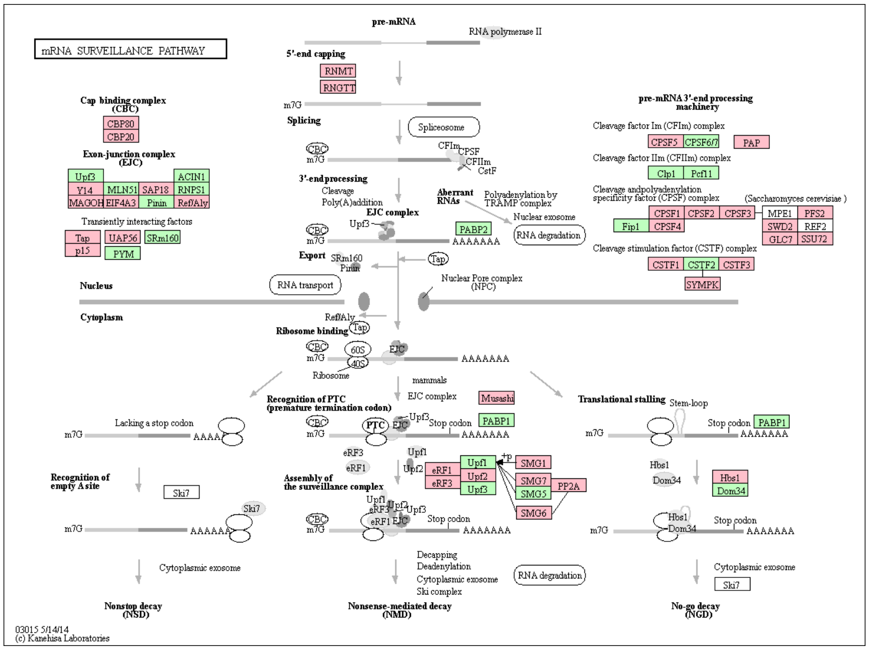

81.3 Legend:

RBH-Blast at 60% Identity + 50% Coverage
Green = Hit in *H. sapiens*
Red = Hit in *H. sapiens* and *C. milli*
White = Not in *H. sapiens*

82 Herpes simplex infection

82.1 Human Pathway: HSA05168

82.2 Number of Hits: 45

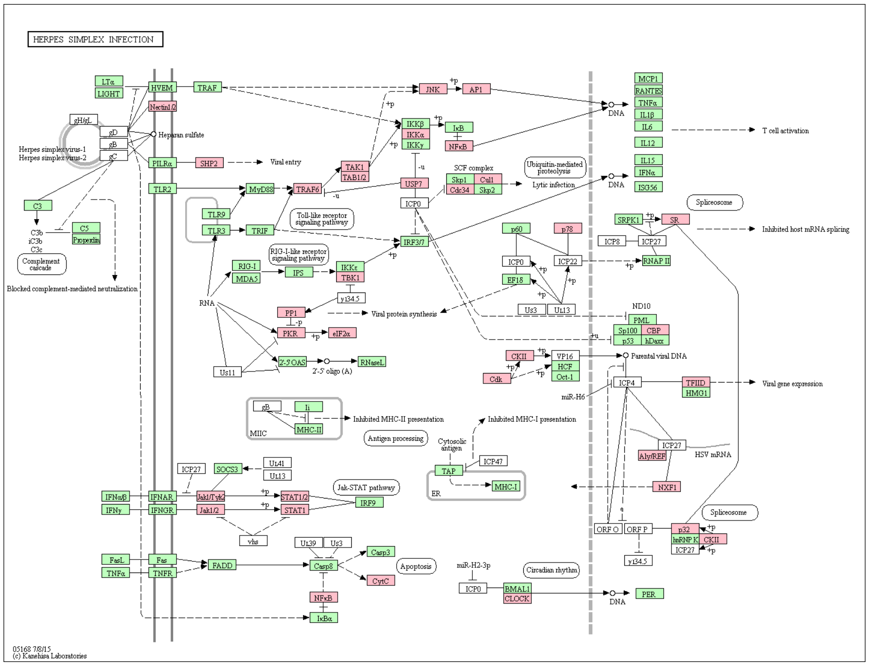

82.3 Legend:

RBH-Blast at 60% Identity + 50% Coverage
Green = Hit in *H. sapiens*
Red = Hit in *H. sapiens* and *C. milli*
White = Not in *H. sapiens*

83 Peroxisome

83.1 Human Pathway: HSA04146

83.2 Number of Hits: 45

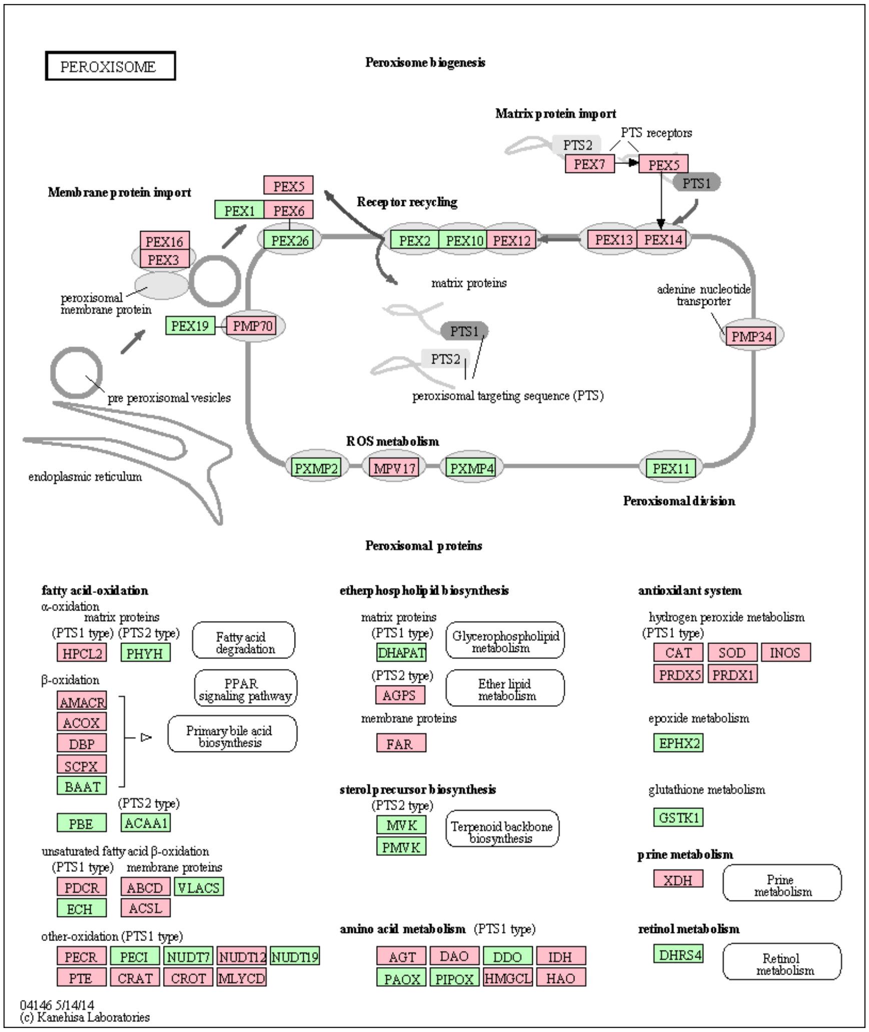

83.3 Legend:

RBH-Blast at 60% Identity + 50% Coverage
Green = Hit in *H. sapiens*
Red = Hit in *H. sapiens* and *C. milli*
White = Not in *H. sapiens*

84 RNA degradation

84.1 Human Pathway: HSA03018

84.2 Number of Hits: 45

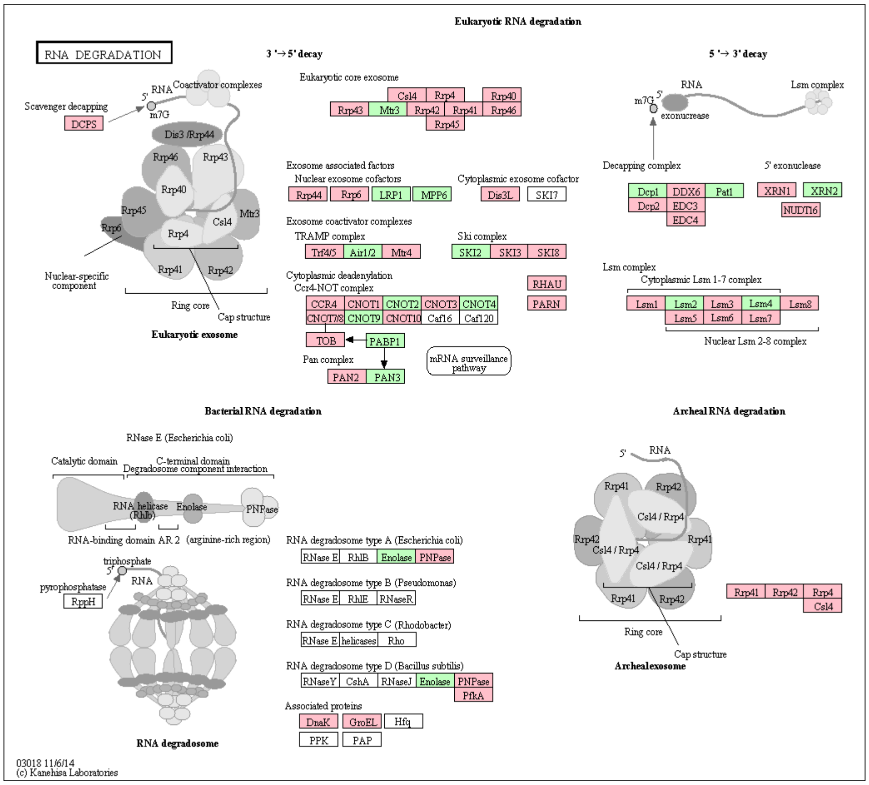

84.3 Legend:

RBH-Blast at 60% Identity + 50% Coverage
Green = Hit in *H. sapiens*
Red = Hit in *H. sapiens* and *C. milli*
White = Not in *H. sapiens*

85 Longevity regulating pathway

85.1 Human Pathway: HSA04211

85.2 Number of Hits: 44

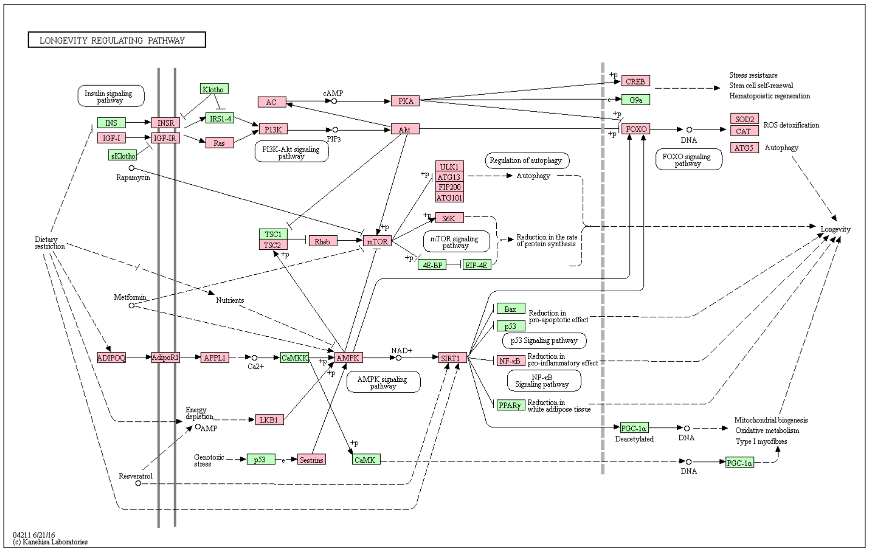

85.3 Legend:

RBH-Blast at 60% Identity + 50% Coverage
Green = Hit in *H. sapiens*
Red = Hit in *H. sapiens* and *C. milli*
White = Not in *H. sapiens*

86 Osteoclast differentiation

86.1 Human Pathway: HSA04380

86.2 Number of Hits: 43

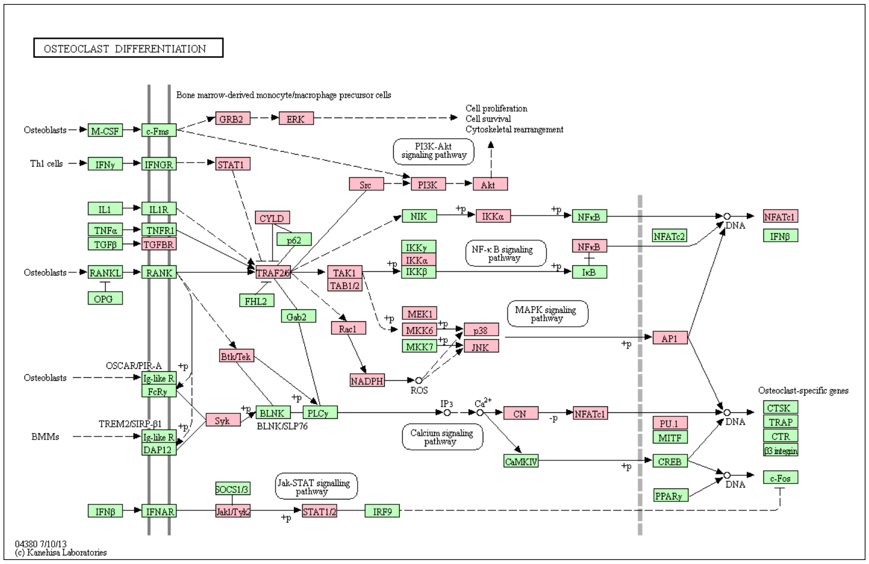

86.3 Legend:

RBH-Blast at 60% Identity + 50% Coverage
Green = Hit in *H. sapiens*
Red = Hit in *H. sapiens* and *C. milli*
White = Not in *H. sapiens*

87 HIF-1 signaling pathway

87.1 Human Pathway: HSA04066

87.2 Number of Hits: 42

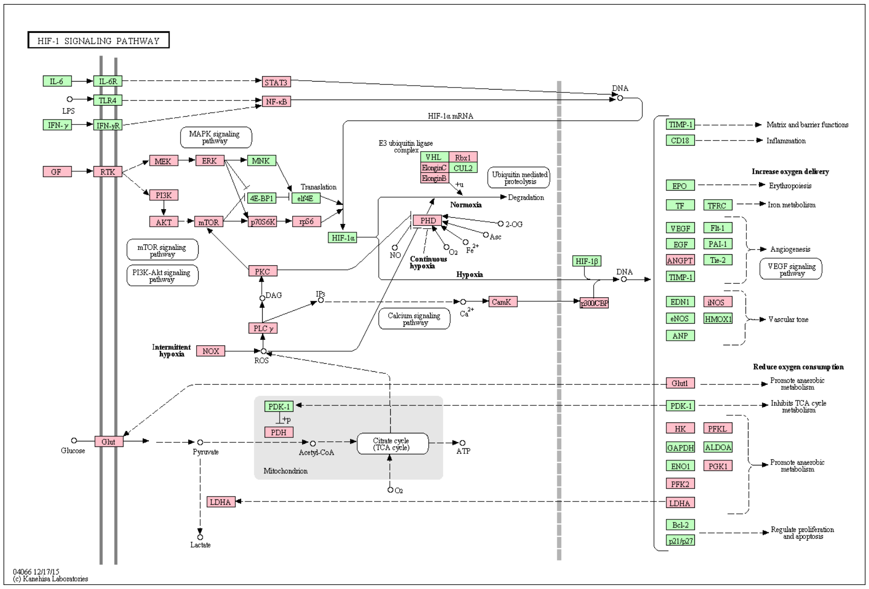

87.3 Legend:

RBH-Blast at 60% Identity + 50% Coverage
Green = Hit in *H. sapiens*
Red = Hit in *H. sapiens* and *C. milli*
White = Not in *H. sapiens*

88 Adherens junction

88.1 Human Pathway: HSA04520

88.2 Number of Hits: 42

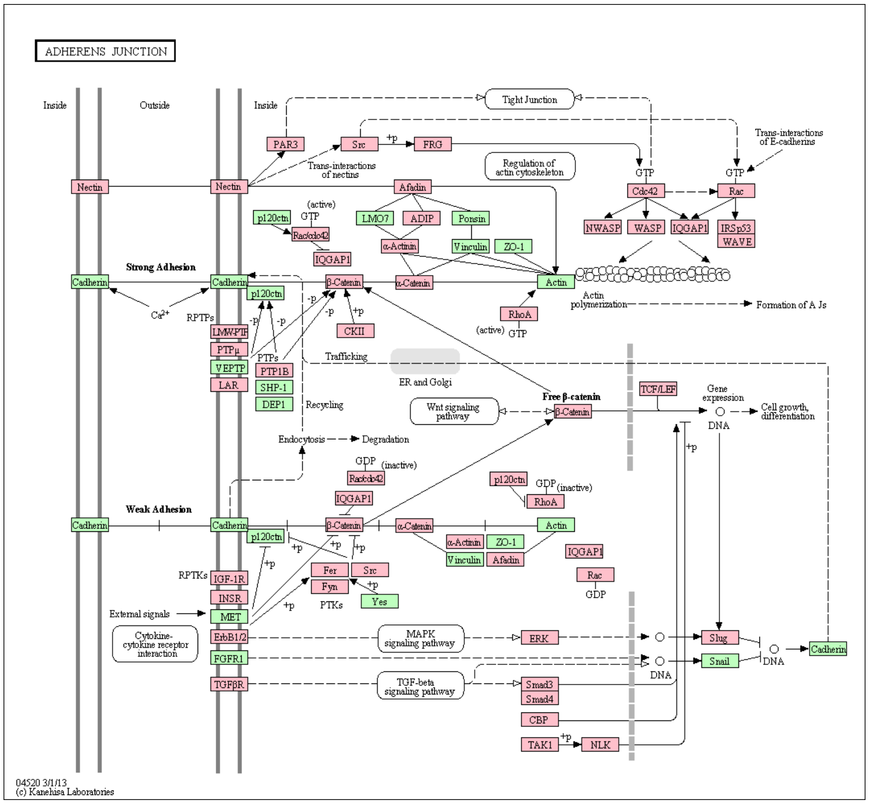

88.3 Legend:

RBH-Blast at 60% Identity + 50% Coverage
Green = Hit in *H. sapiens*
Red = Hit in *H. sapiens* and *C. milli*
White = Not in *H. sapiens*

89 Long-term depression

89.1 Human Pathway: HSA04730

89.2 Number of Hits: 41

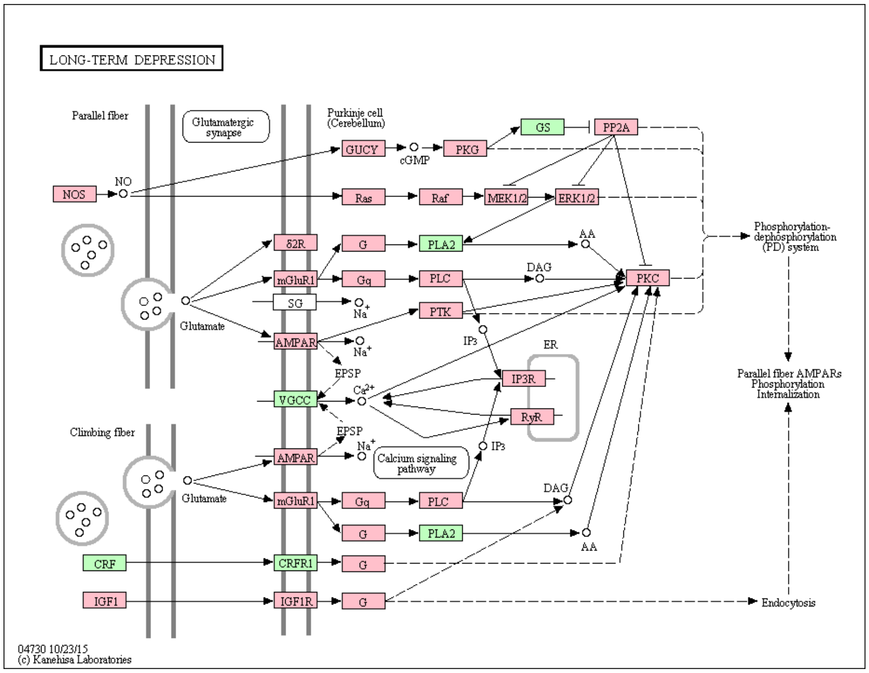

89.3 Legend:

RBH-Blast at 60% Identity + 50% Coverage
Green = Hit in *H. sapiens*
Red = Hit in *H. sapiens* and *C. milli*
White = Not in *H. sapiens*

---

90 Synaptic vesicle cycle

90.1 Human Pathway: HSA04721

90.2 Number of Hits: 41

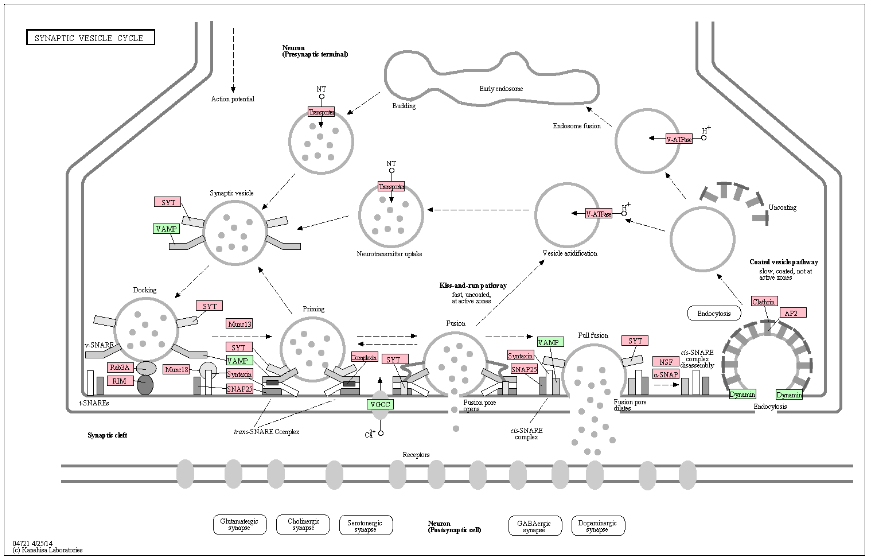

90.3 Legend:

RBH-Blast at 60% Identity + 50% Coverage
Green = Hit in *H. sapiens*
Red = Hit in *H. sapiens* and *C. milli*
White = Not in *H. sapiens*

91 Prolactin signaling pathway

91.1 Human Pathway: HSA04917

91.2 Number of Hits: 41

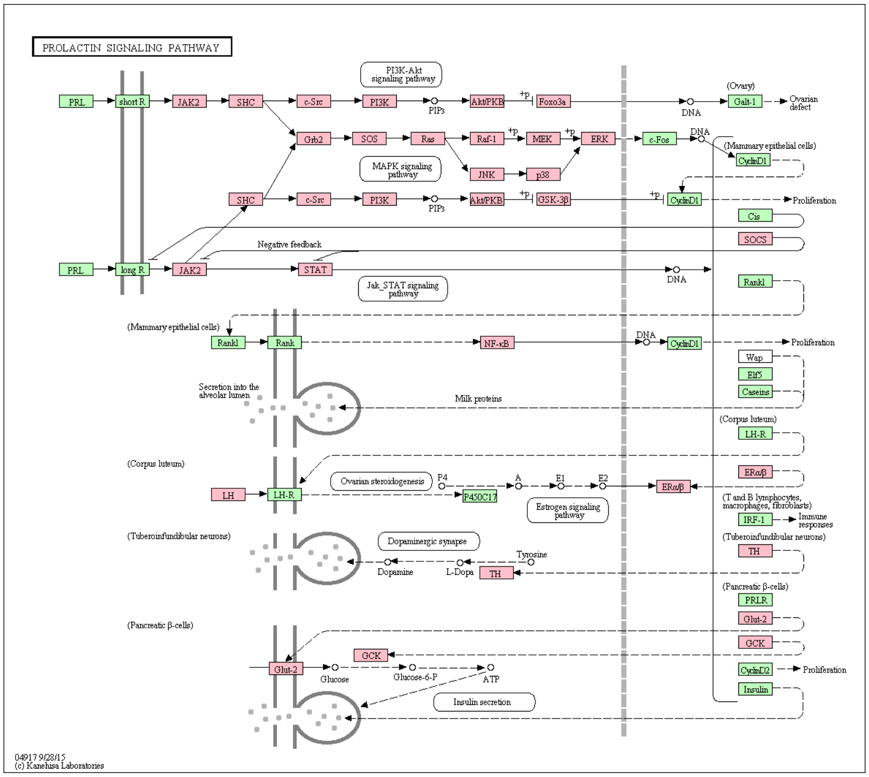

91.3 Legend:

RBH-Blast at 60% Identity + 50% Coverage
Green = Hit in *H. sapiens*
Red = Hit in *H. sapiens* and *C. milli*
White = Not in *H. sapiens*

92 Glycerophospholipid metabolism

92.1 Human Pathway: HSA00564

92.2 Number of Hits: 41

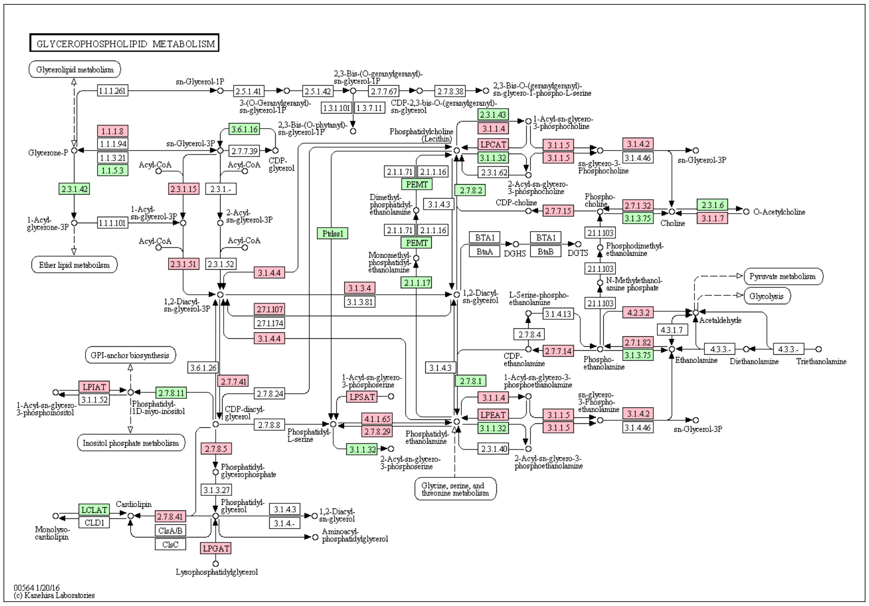

92.3 Legend:

RBH-Blast at 60% Identity + 50% Coverage
Green = Hit in *H. sapiens*
Red = Hit in *H. sapiens* and *C. milli*
White = Not in *H. sapiens*

93 Inflammatory mediator regulation of TRP channels

93.1 Human Pathway: HSA04750

93.2 Number of Hits: 40

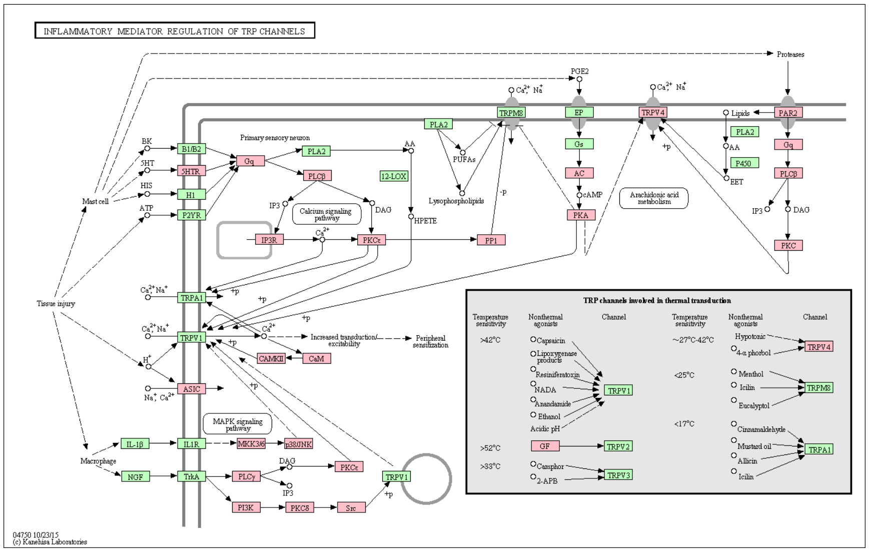

93.3 Legend:

RBH-Blast at 60% Identity + 50% Coverage
Green = Hit in *H. sapiens*
Red = Hit in *H. sapiens* and *C. milli*
White = Not in *H. sapiens*

94 Long-term potentiation

94.1 Human Pathway: HSA04720

94.2 Number of Hits: 40

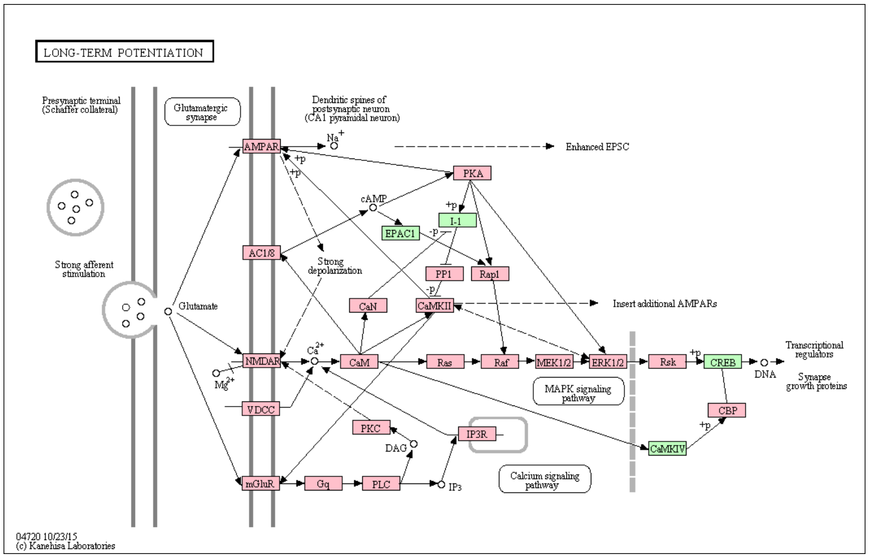

94.3 Legend:

RBH-Blast at 60% Identity + 50% Coverage
Green = Hit in *H. sapiens*
Red = Hit in *H. sapiens* and *C. milli*
White = Not in *H. sapiens*

95 Chagas disease (American trypanosomiasis)

95.1 Human Pathway: HSA05142

95.2 Number of Hits: 40

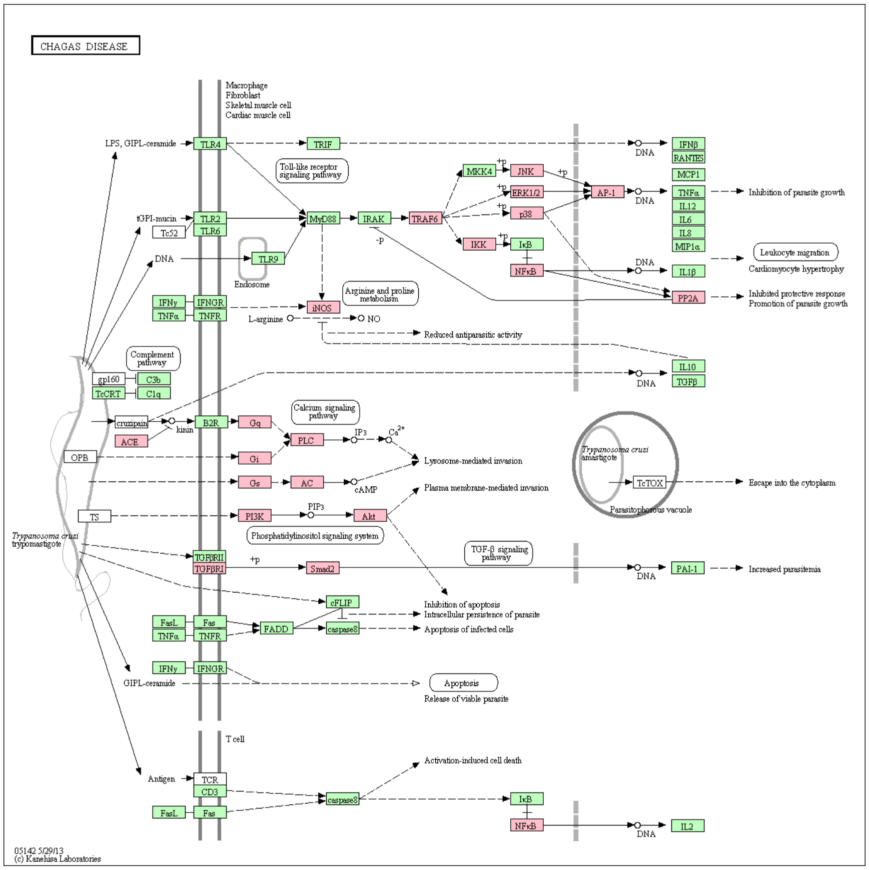

95.3 Legend:

RBH-Blast at 60% Identity + 50% Coverage
Green = Hit in *H. sapiens*
Red = Hit in *H. sapiens* and *C. milli*
White = Not in *H. sapiens*

96 Prostate cancer

96.1 Human Pathway: HSA05215

96.2 Number of Hits: 40

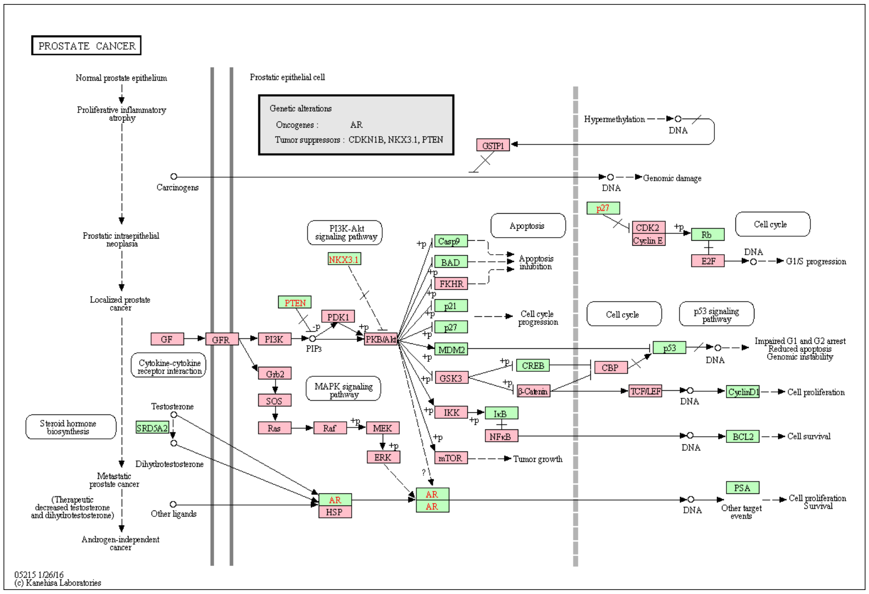

96.3 Legend:

RBH-Blast at 60% Identity + 50% Coverage
Green = Hit in *H. sapiens*
Red = Hit in *H. sapiens* and *C. milli*
White = Not in *H. sapiens*

97 ErbB signaling pathway

97.1 Human Pathway: HSA04012

97.2 Number of Hits: 40

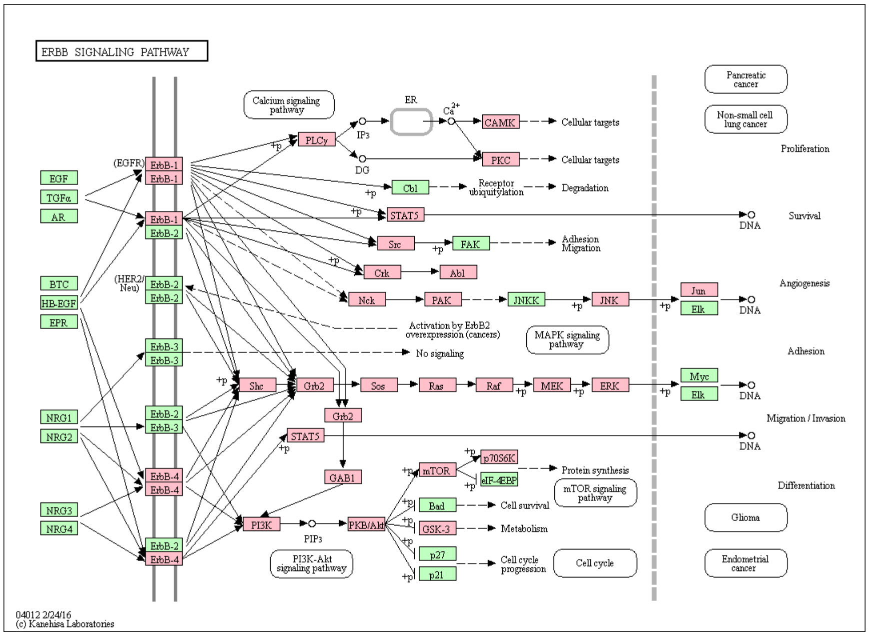

97.3 Legend:

RBH-Blast at 60% Identity + 50% Coverage
Green = Hit in *H. sapiens*
Red = Hit in *H. sapiens* and *C. milli*
White = Not in *H. sapiens*

98 Biosynthesis of amino acids

98.1 Human Pathway: HSA01230

98.2 Number of Hits: 40

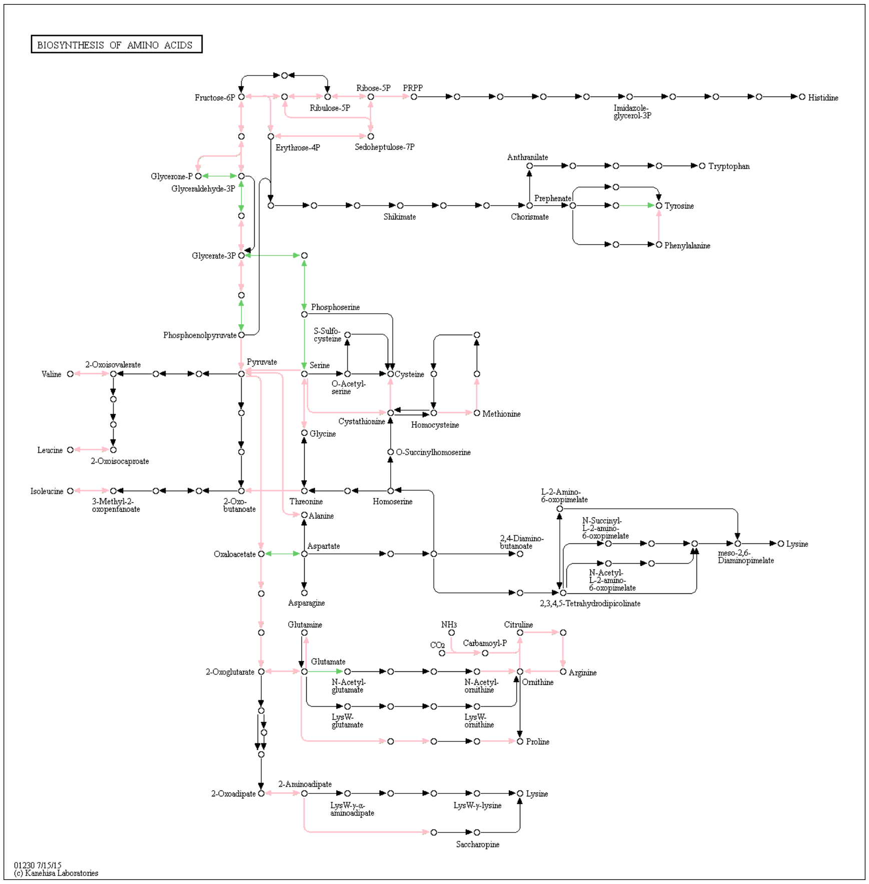

98.3 Legend:

RBH-Blast at 60% Identity + 50% Coverage
Green = Hit in *H. sapiens*
Red = Hit in *H. sapiens* and *C. milli*
White = Not in *H. sapiens*

99 Ribosome biogenesis in eukaryotes

99.1 Human Pathway: HSA03008

99.2 Number of Hits: 39

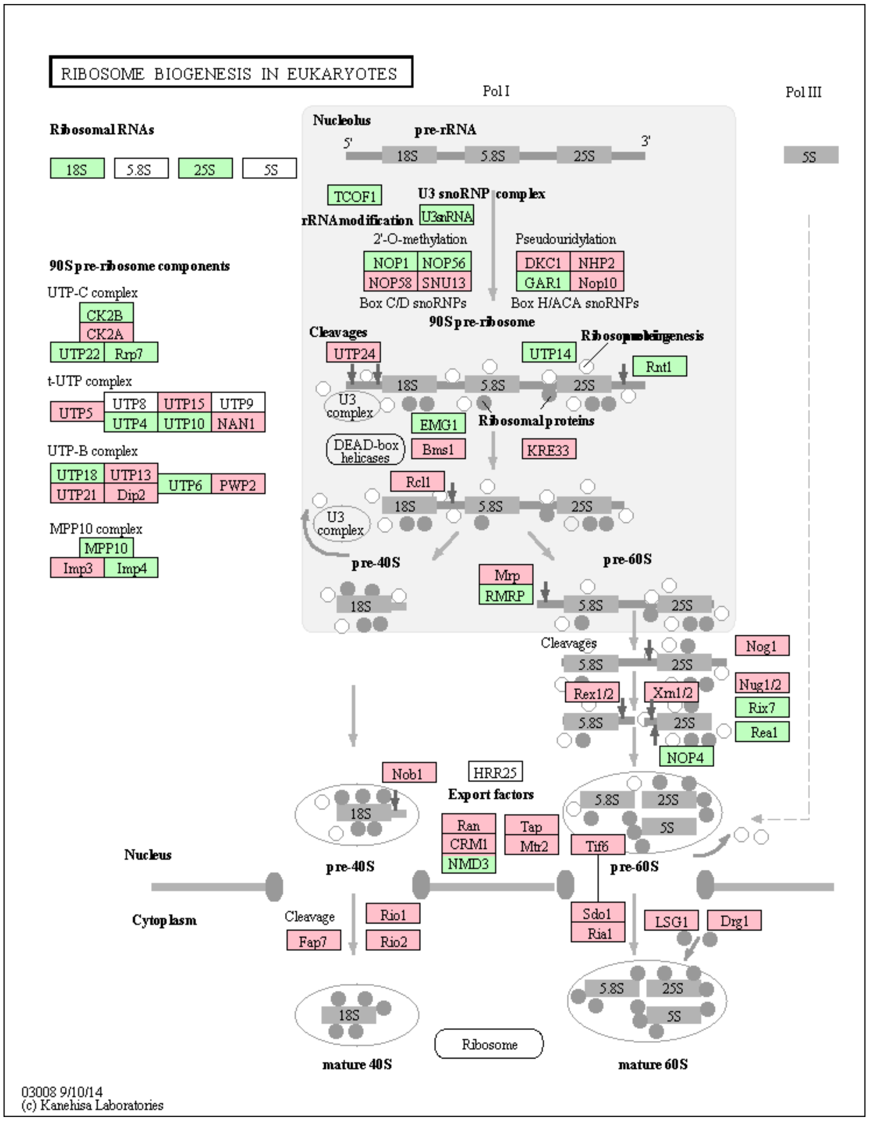

99.3 Legend:

RBH-Blast at 60% Identity + 50% Coverage
Green = Hit in *H. sapiens*
Red = Hit in *H. sapiens* and *C. milli*
White = Not in *H. sapiens*

100 Bacterial invasion of epithelial cells

100.1 Human Pathway: HSA05100

100.2 Number of Hits: 39

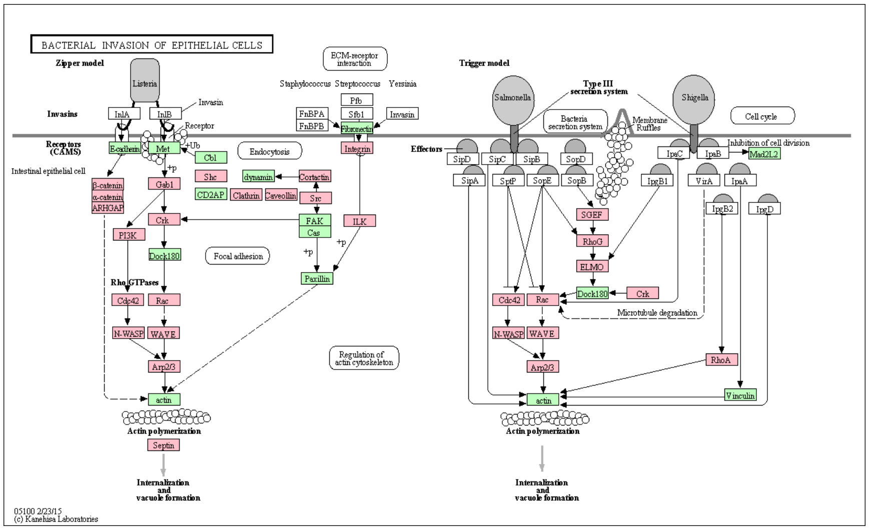

100.3 Legend:

RBH-Blast at 60% Identity + 50% Coverage
Green = Hit in *H. sapiens*
Red = Hit in *H. sapiens* and *C. milli*
White = Not in *H. sapiens*

101 Toxoplasmosis

101.1 Human Pathway: HSA05145

101.2 Number of Hits: 38

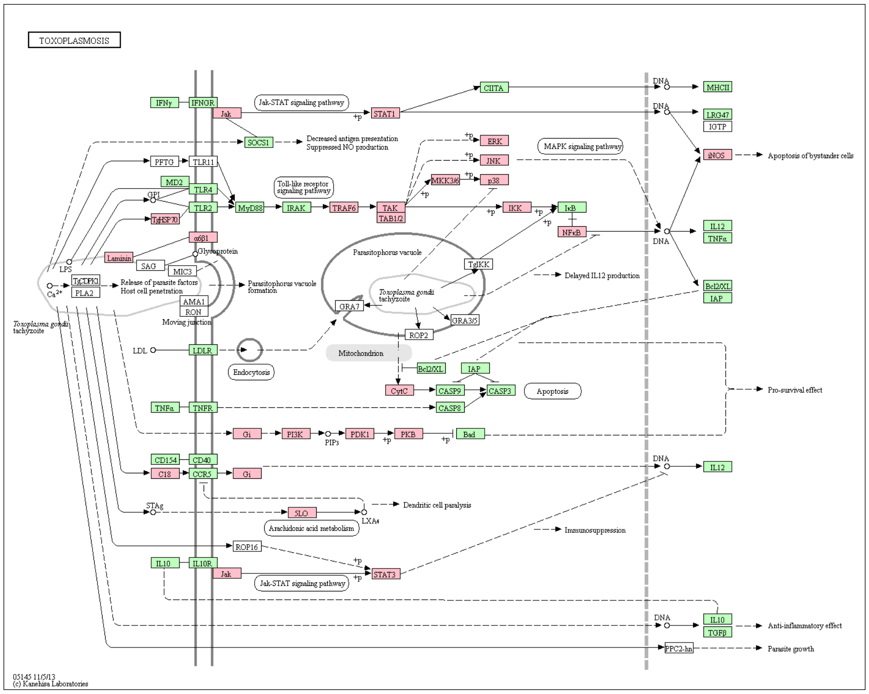

101.3 Legend:

RBH-Blast at 60% Identity + 50% Coverage
Green = Hit in *H. sapiens*
Red = Hit in *H. sapiens* and *C. milli*
White = Not in *H. sapiens*

102 Epithelial cell signaling in Helicobacter pylori infection

102.1 Human Pathway: HSA05120

102.2 Number of Hits: 38

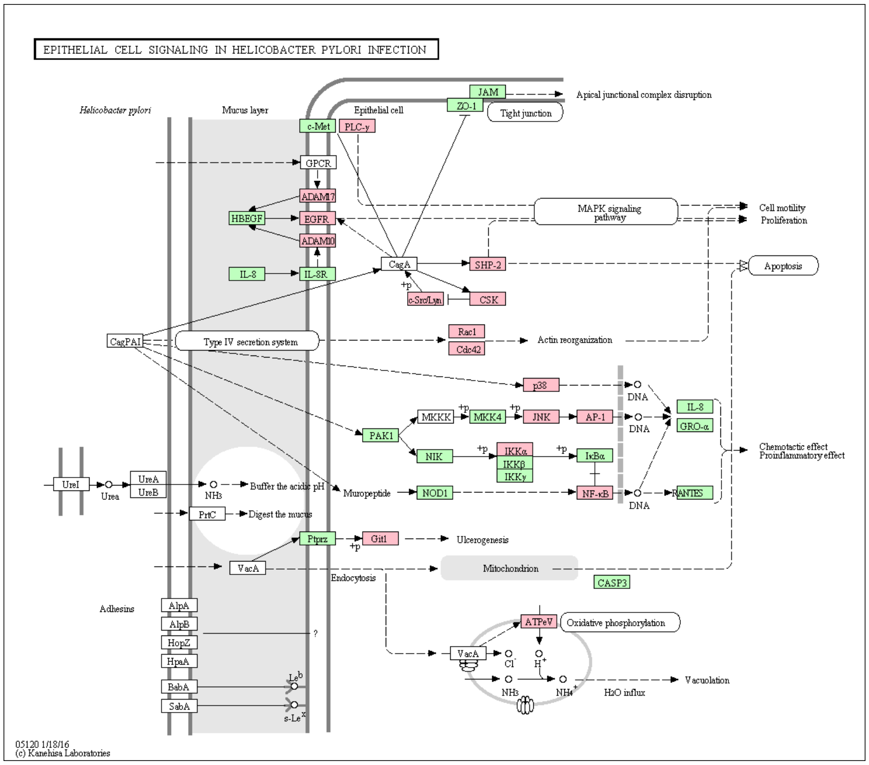

102.3 Legend:

RBH-Blast at 60% Identity + 50% Coverage
Green = Hit in *H. sapiens*
Red = Hit in *H. sapiens* and *C. milli*
White = Not in *H. sapiens*

103 Inositol phosphate metabolism

103.1 Human Pathway: HSA00562

103.2 Number of Hits: 38

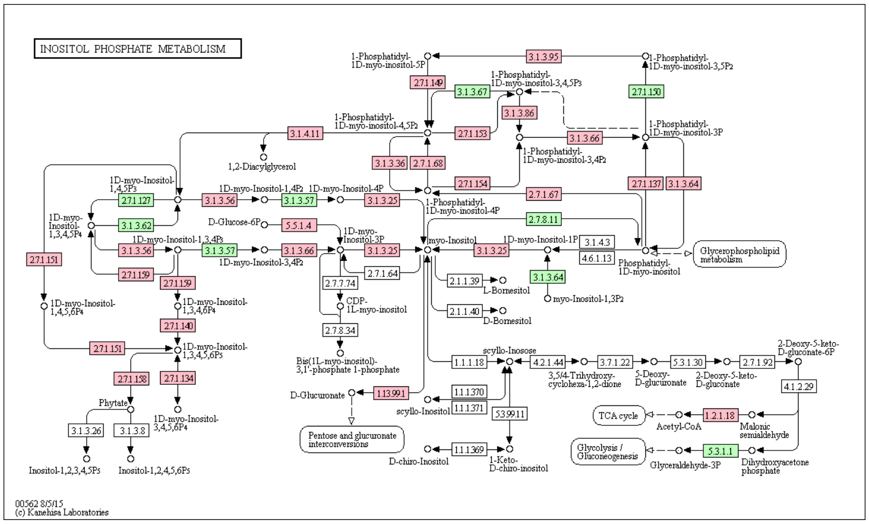

103.3 Legend:

RBH-Blast at 60% Identity + 50% Coverage
Green = Hit in *H. sapiens*
Red = Hit in *H. sapiens* and *C. milli*
White = Not in *H. sapiens*

104 Renal cell carcinoma

104.1 Human Pathway: HSA05211

104.2 Number of Hits: 38

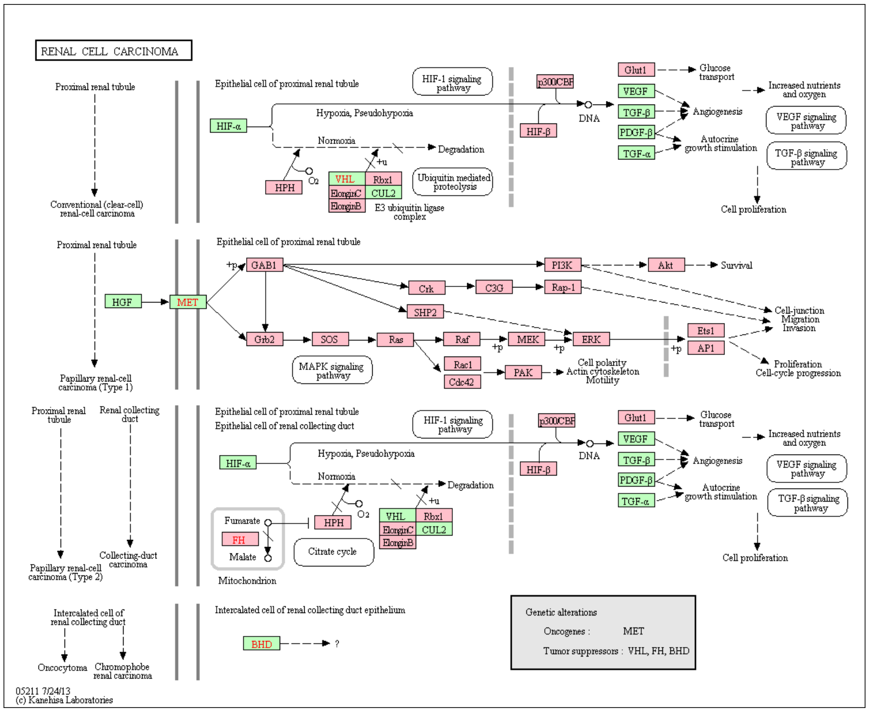

104.3 Legend:

RBH-Blast at 60% Identity + 50% Coverage
Green = Hit in *H. sapiens*
Red = Hit in *H. sapiens* and *C. milli*
White = Not in *H. sapiens*

105 EGFR tyrosine kinase inhibitor resistance

105.1 Human Pathway: HSA01521

105.2 Number of Hits: 37

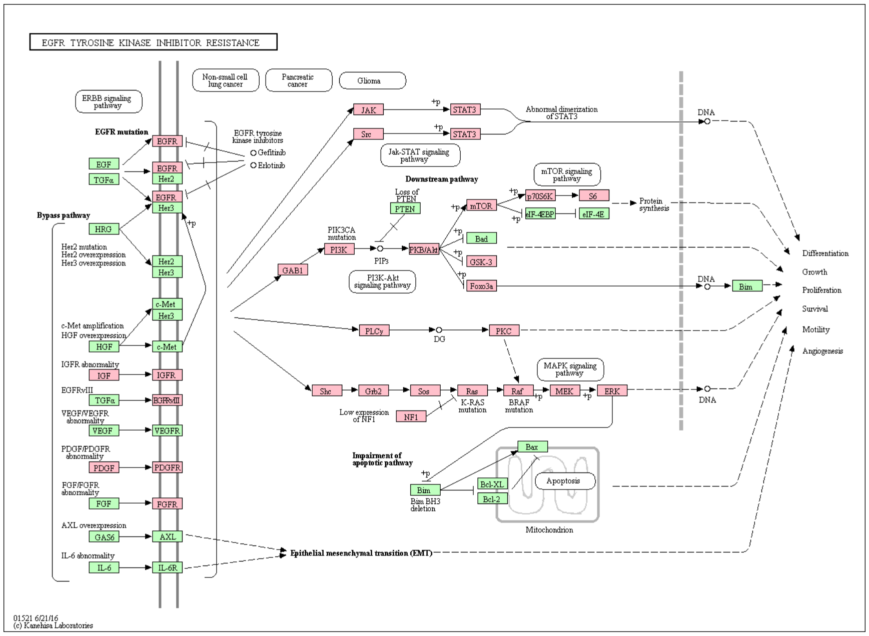

105.3 Legend:

RBH-Blast at 60% Identity + 50% Coverage
Green = Hit in *H. sapiens*
Red = Hit in *H. sapiens* and *C. milli*
White = Not in *H. sapiens*

106 Dilated cardiomyopathy

106.1 Human Pathway: HSA05414

106.2 Number of Hits: 36

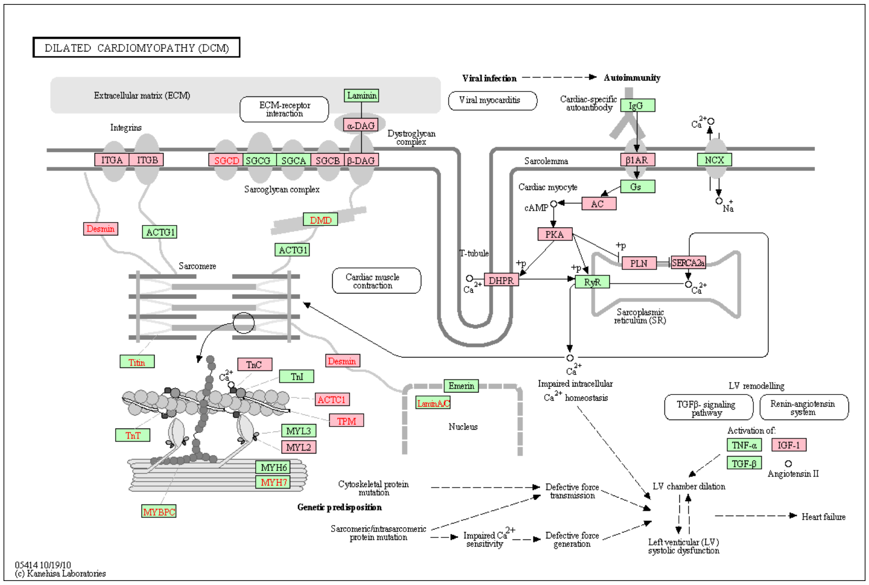

106.3 Legend:

RBH-Blast at 60% Identity + 50% Coverage
Green = Hit in *H. sapiens*
Red = Hit in *H. sapiens* and *C. milli*
White = Not in *H. sapiens*

107 Chronic myeloid leukemia

107.1 Human Pathway: HSA05220

107.2 Number of Hits: 36

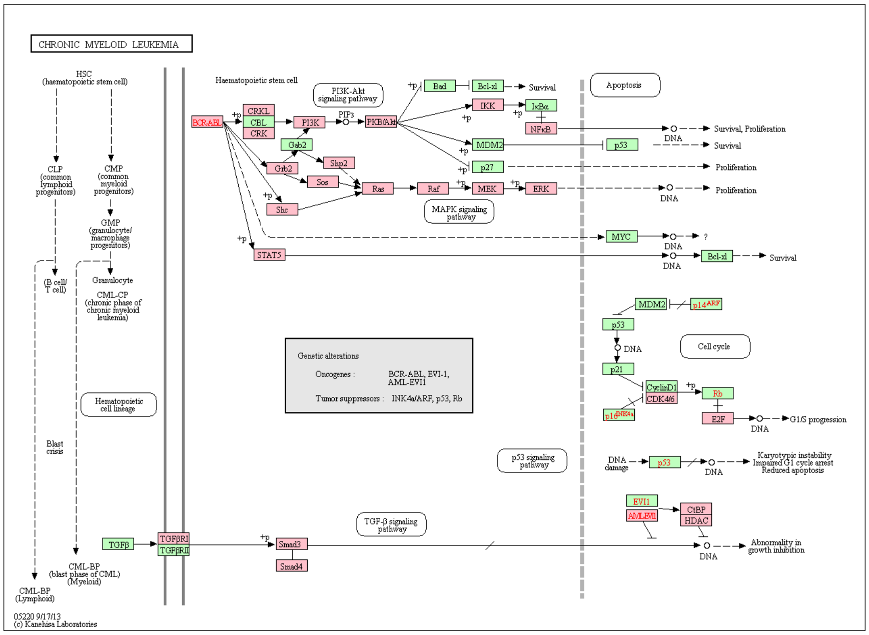

107.3 Legend:

RBH-Blast at 60% Identity + 50% Coverage
Green = Hit in *H. sapiens*
Red = Hit in *H. sapiens* and *C. milli*
White = Not in *H. sapiens*

108 Measles

108.1 Human Pathway: HSA05162

108.2 Number of Hits: 36

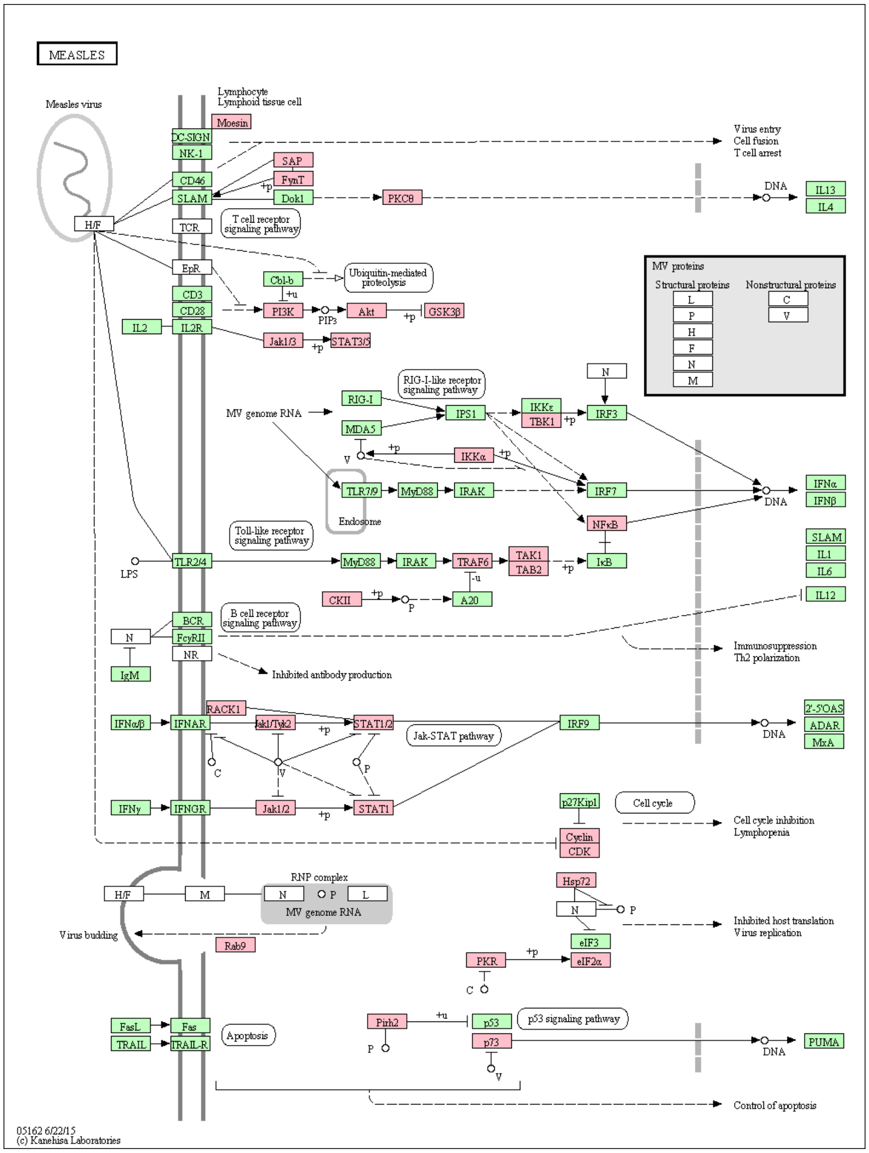

108.3 Legend:

RBH-Blast at 60% Identity + 50% Coverage
Green = Hit in *H. sapiens*
Red = Hit in *H. sapiens* and *C. milli*
White = Not in *H. sapiens*

109 Pancreatic secretion

109.1 Human Pathway: HSA04972

109.2 Number of Hits: 35

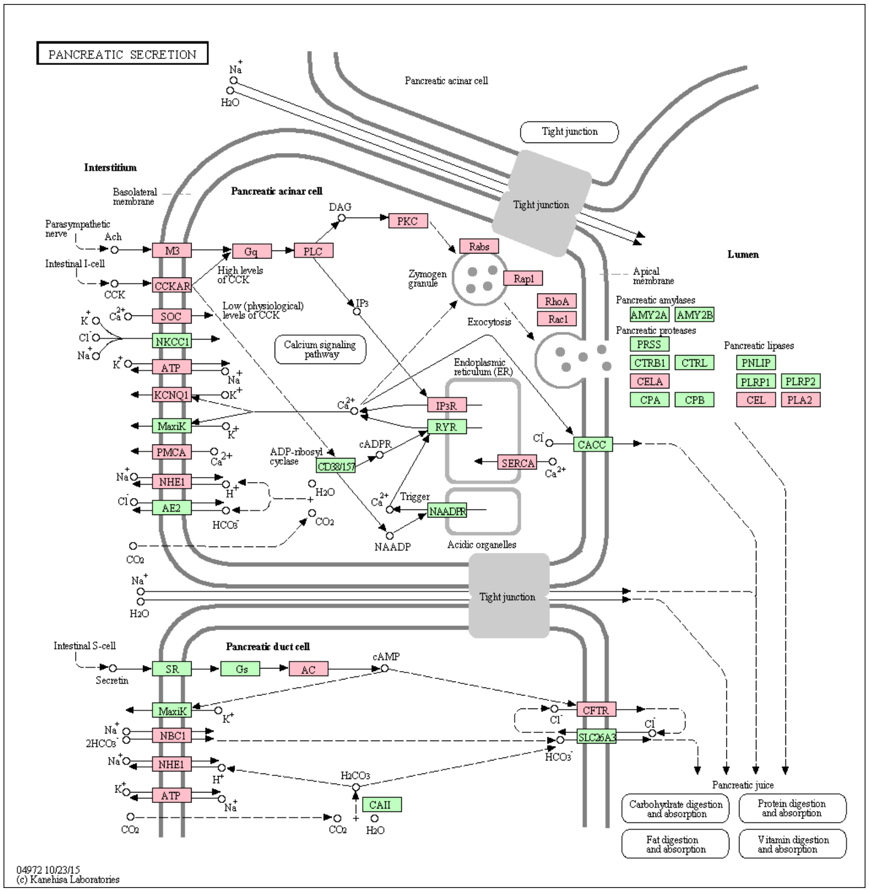

109.3 Legend:

RBH-Blast at 60% Identity + 50% Coverage
Green = Hit in *H. sapiens*
Red = Hit in *H. sapiens* and *C. milli*
White = Not in *H. sapiens*

---

110 Pancreatic cancer

110.1 Human Pathway: HSA05212

110.2 Number of Hits: 35

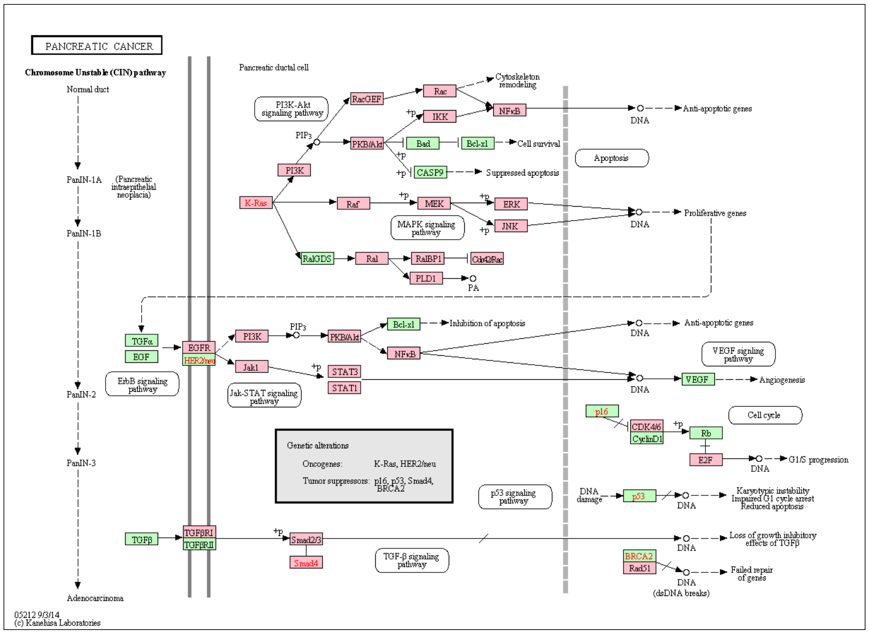

110.3 Legend:

RBH-Blast at 60% Identity + 50% Coverage
Green = Hit in *H. sapiens*
Red = Hit in *H. sapiens* and *C. milli*
White = Not in *H. sapiens*

---

111 Salmonella infection

111.1 Human Pathway: HSA05132

111.2 Number of Hits: 35

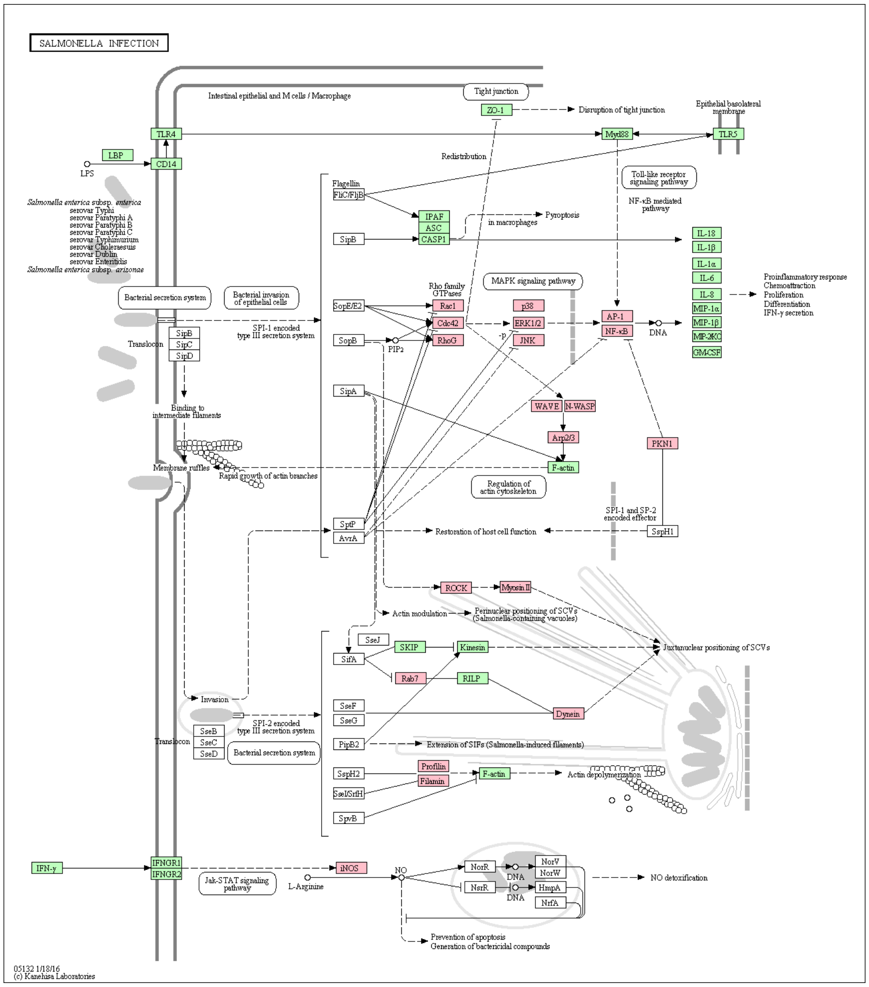

111.3 Legend:

RBH-Blast at 60% Identity + 50% Coverage
Green = Hit in *H. sapiens*
Red = Hit in *H. sapiens* and *C. milli*
White = Not in *H. sapiens*

112 Shigellosis

112.1 Human Pathway: HSA05131

112.2 Number of Hits: 34

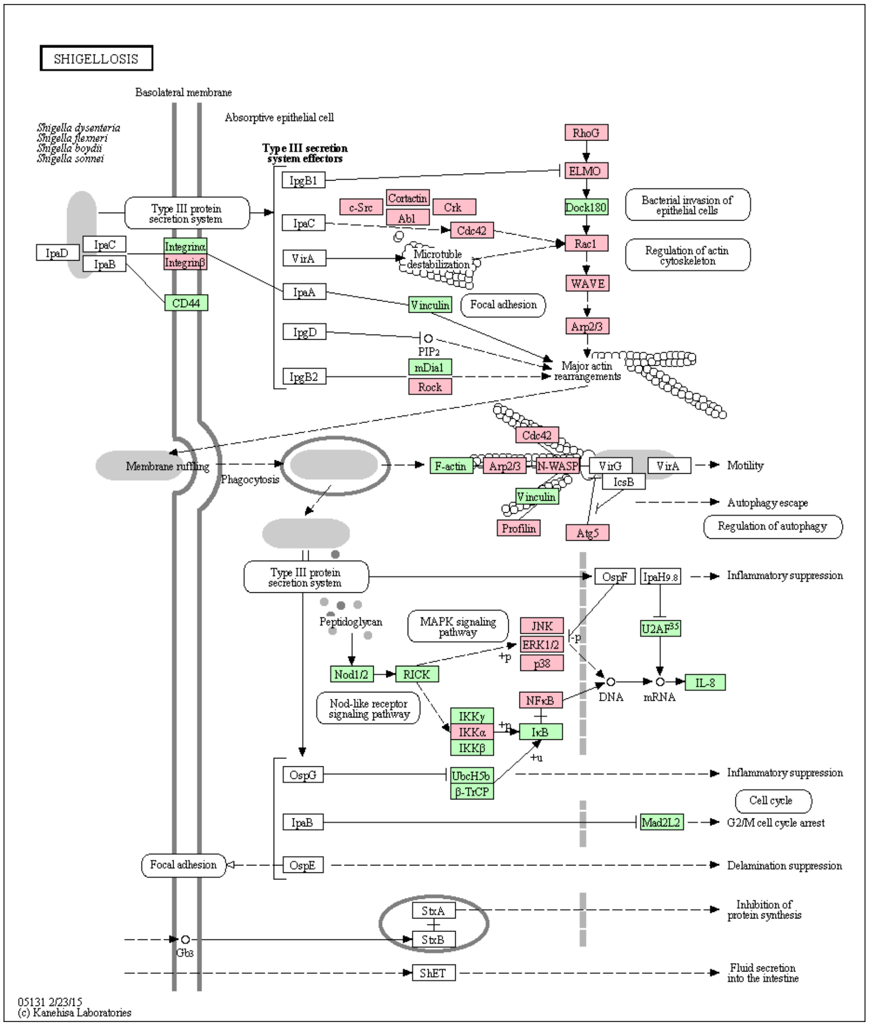

112.3 Legend:

RBH-Blast at 60% Identity + 50% Coverage
Green = Hit in *H. sapiens*
Red = Hit in *H. sapiens* and *C. milli*
White = Not in *H. sapiens*

113 TGF-beta signaling pathway

113.1 Human Pathway: HSA04350

113.2 Number of Hits: 34

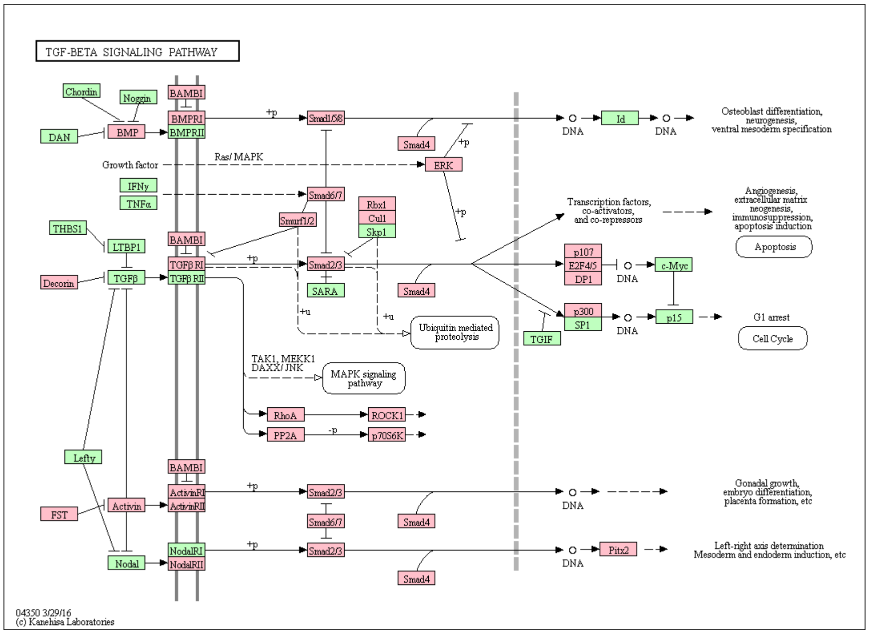

113.3 Legend:

RBH-Blast at 60% Identity + 50% Coverage
Green = Hit in *H. sapiens*
Red = Hit in *H. sapiens* and *C. milli*
White = Not in *H. sapiens*

114 Longevity regulating pathway - multiple species

114.1 Human Pathway: HSA04213

114.2 Number of Hits: 34

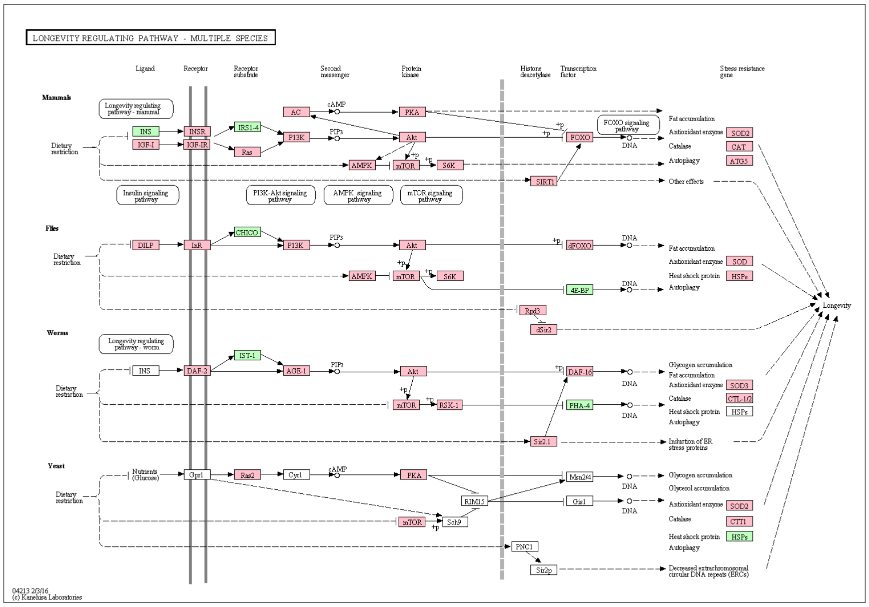

114.3 Legend:

RBH-Blast at 60% Identity + 50% Coverage
Green = Hit in *H. sapiens*
Red = Hit in *H. sapiens* and *C. milli*
White = Not in *H. sapiens*

115 B cell receptor signaling pathway

115.1 Human Pathway: HSA04662

115.2 Number of Hits: 34

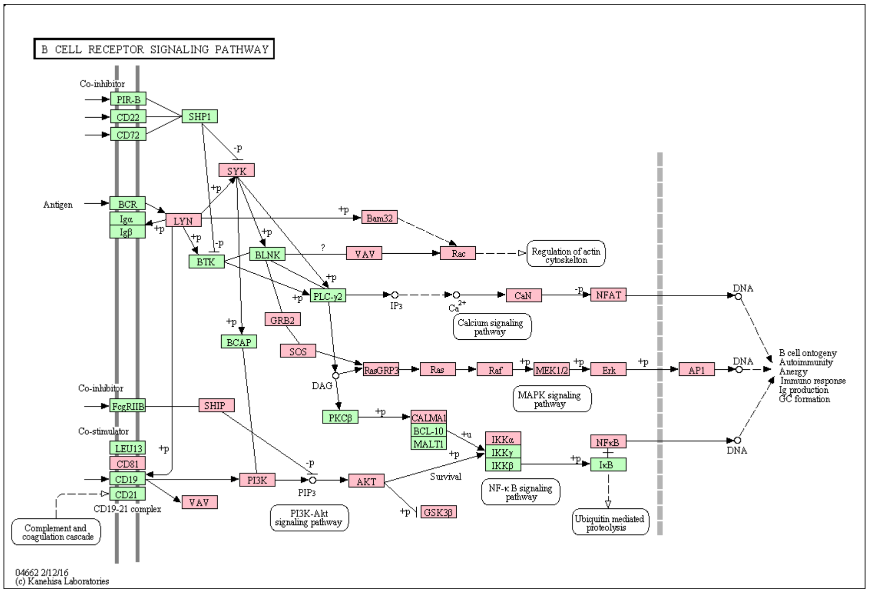

115.3 Legend:

RBH-Blast at 60% Identity + 50% Coverage
Green = Hit in *H. sapiens*
Red = Hit in *H. sapiens* and *C. milli*
White = Not in *H. sapiens*

116 Fc epsilon RI signaling pathway

116.1 Human Pathway: HSA04664

116.2 Number of Hits: 34

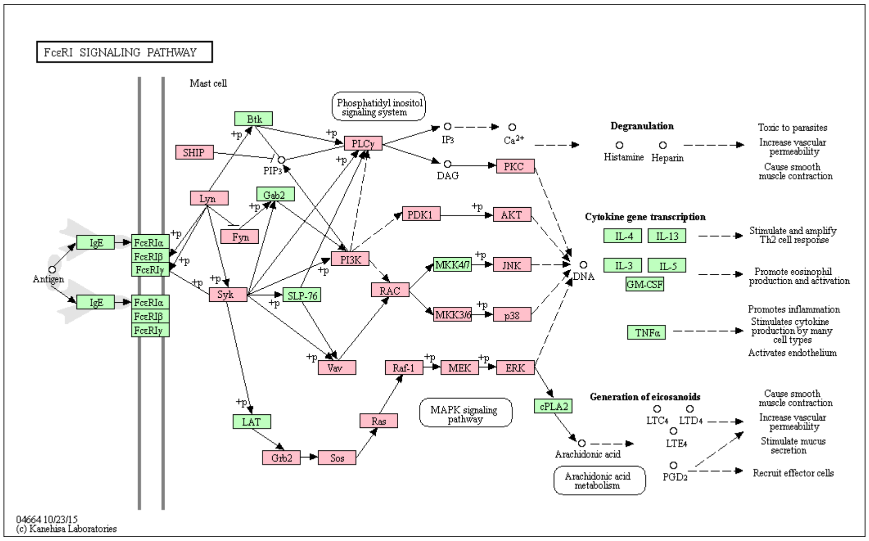

116.3 Legend:

RBH-Blast at 60% Identity + 50% Coverage
Green = Hit in *H. sapiens*
Red = Hit in *H. sapiens* and *C. milli*
White = Not in *H. sapiens*

117 Hypertrophic cardiomyopathy (HCM)

117.1 Human Pathway: HSA05410

117.2 Number of Hits: 34

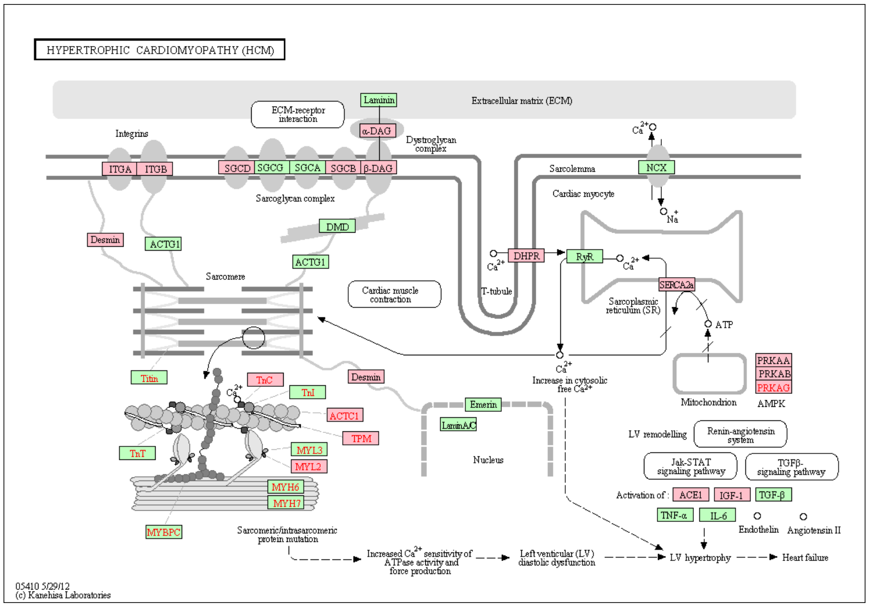

117.3 Legend:

RBH-Blast at 60% Identity + 50% Coverage
Green = Hit in *H. sapiens*
Red = Hit in *H. sapiens* and *C. milli*
White = Not in *H. sapiens*

118 Fatty acid metabolism

118.1 Human Pathway: HSA01212

118.2 Number of Hits: 33

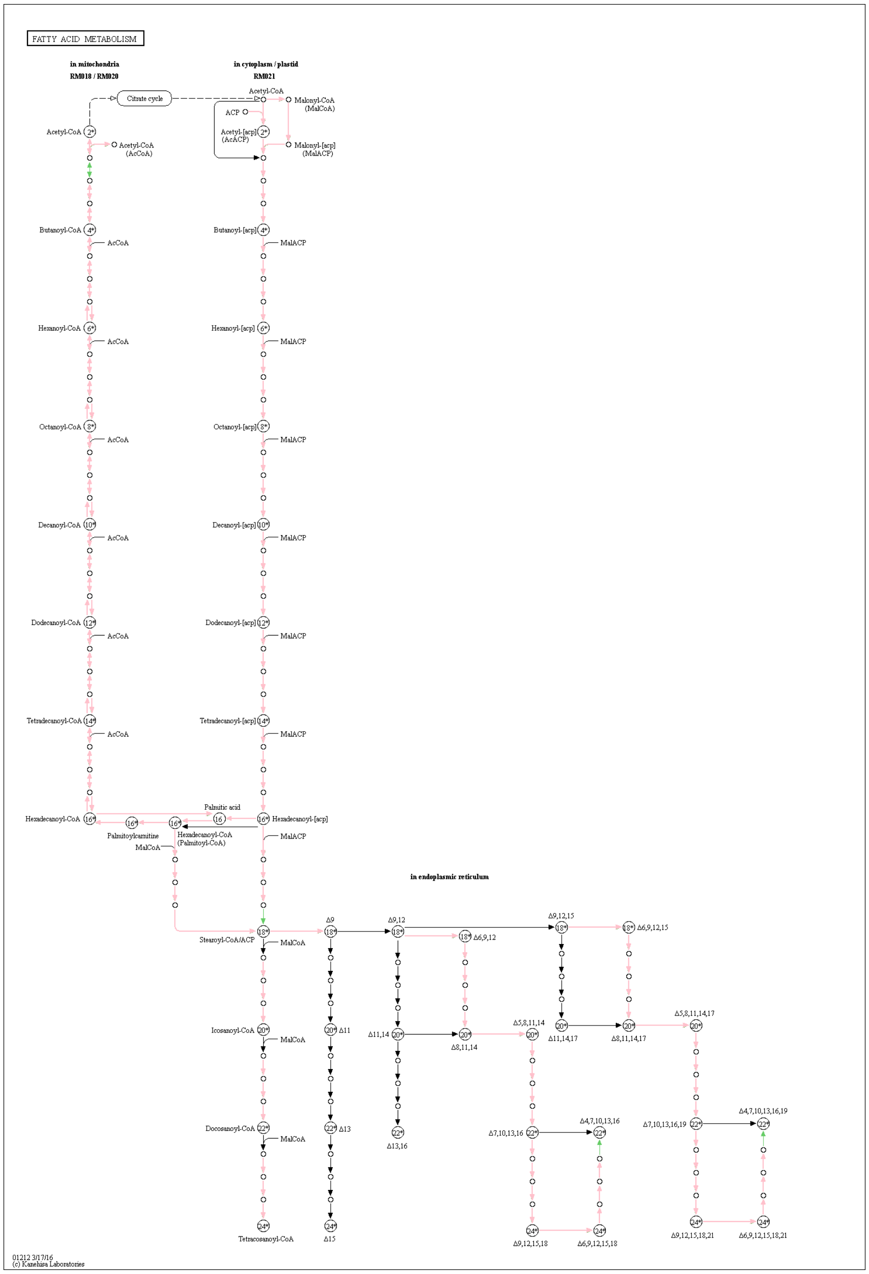

118.3 Legend:

RBH-Blast at 60% Identity + 50% Coverage
Green = Hit in *H. sapiens*
Red = Hit in *H. sapiens* and *C. milli*
White = Not in *H. sapiens*

---

119 Melanoma

119.1 Human Pathway: HSA05218

119.2 Number of Hits: 33

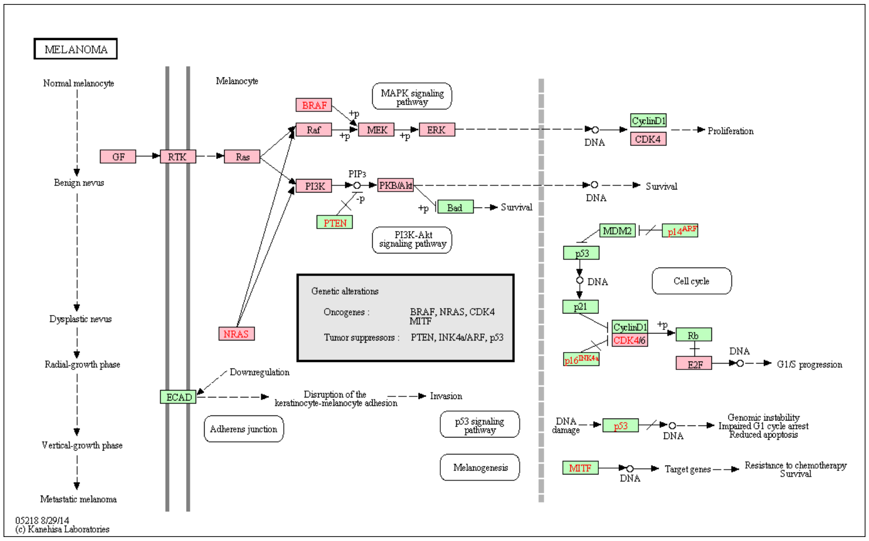

119.3 Legend:

RBH-Blast at 60% Identity + 50% Coverage
Green = Hit in *H. sapiens*
Red = Hit in *H. sapiens* and *C. milli*
White = Not in *H. sapiens*

---

120 Thyroid hormone synthesis

120.1 Human Pathway: HSA04918

120.2 Number of Hits: 32

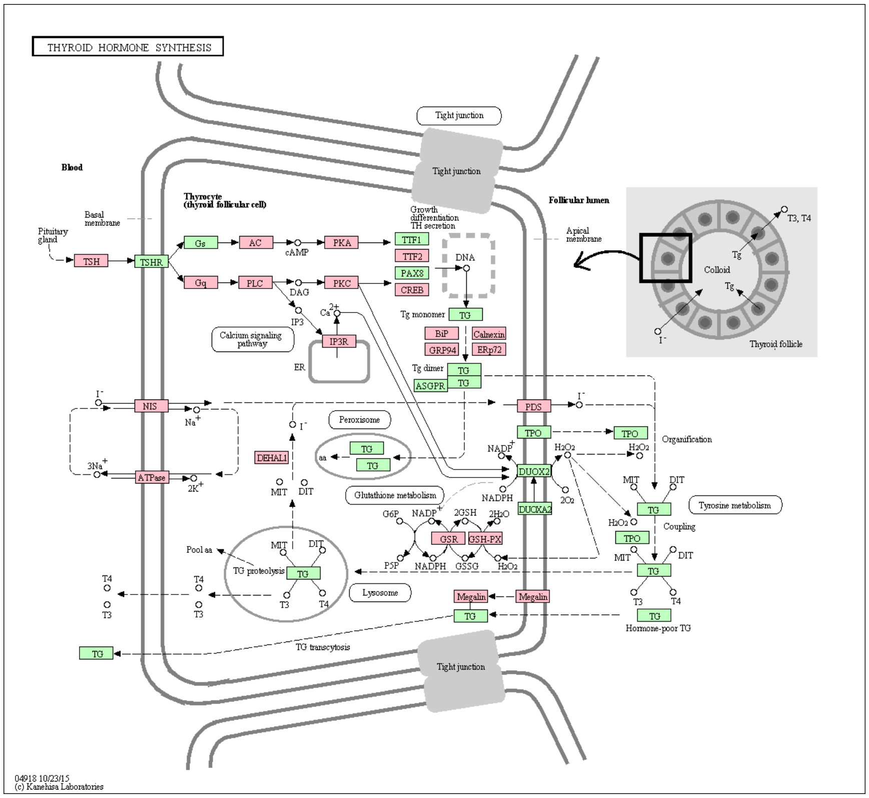

120.3 Legend:

RBH-Blast at 60% Identity + 50% Coverage
Green = Hit in *H. sapiens*
Red = Hit in *H. sapiens* and *C. milli*
White = Not in *H. sapiens*

121 Central carbon metabolism in cancer

121.1 Human Pathway: HSA05230

121.2 Number of Hits: 32

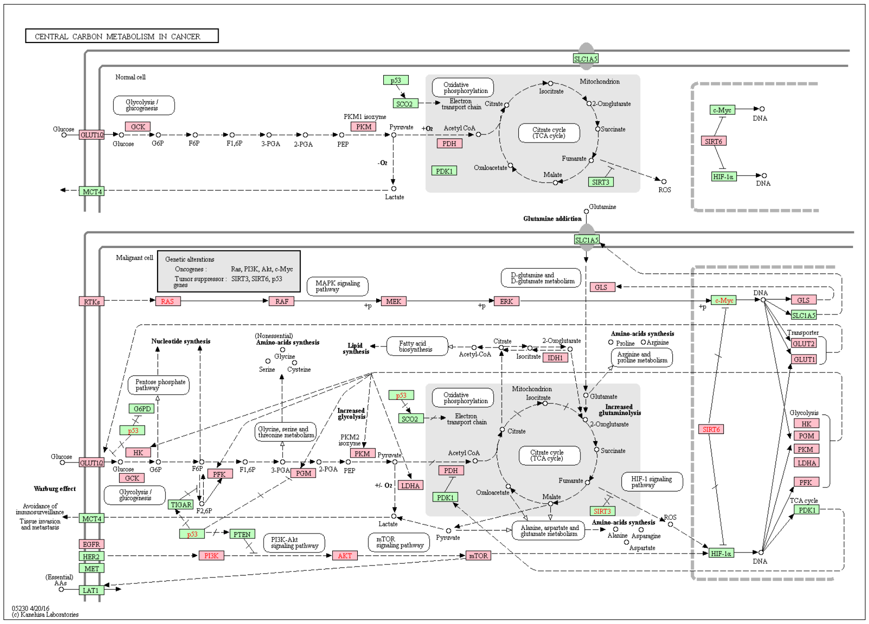

121.3 Legend:

RBH-Blast at 60% Identity + 50% Coverage
Green = Hit in *H. sapiens*
Red = Hit in *H. sapiens* and *C. milli*
White = Not in *H. sapiens*

122 N-Glycan biosynthesis

122.1 Human Pathway: HSA00510

122.2 Number of Hits: 32

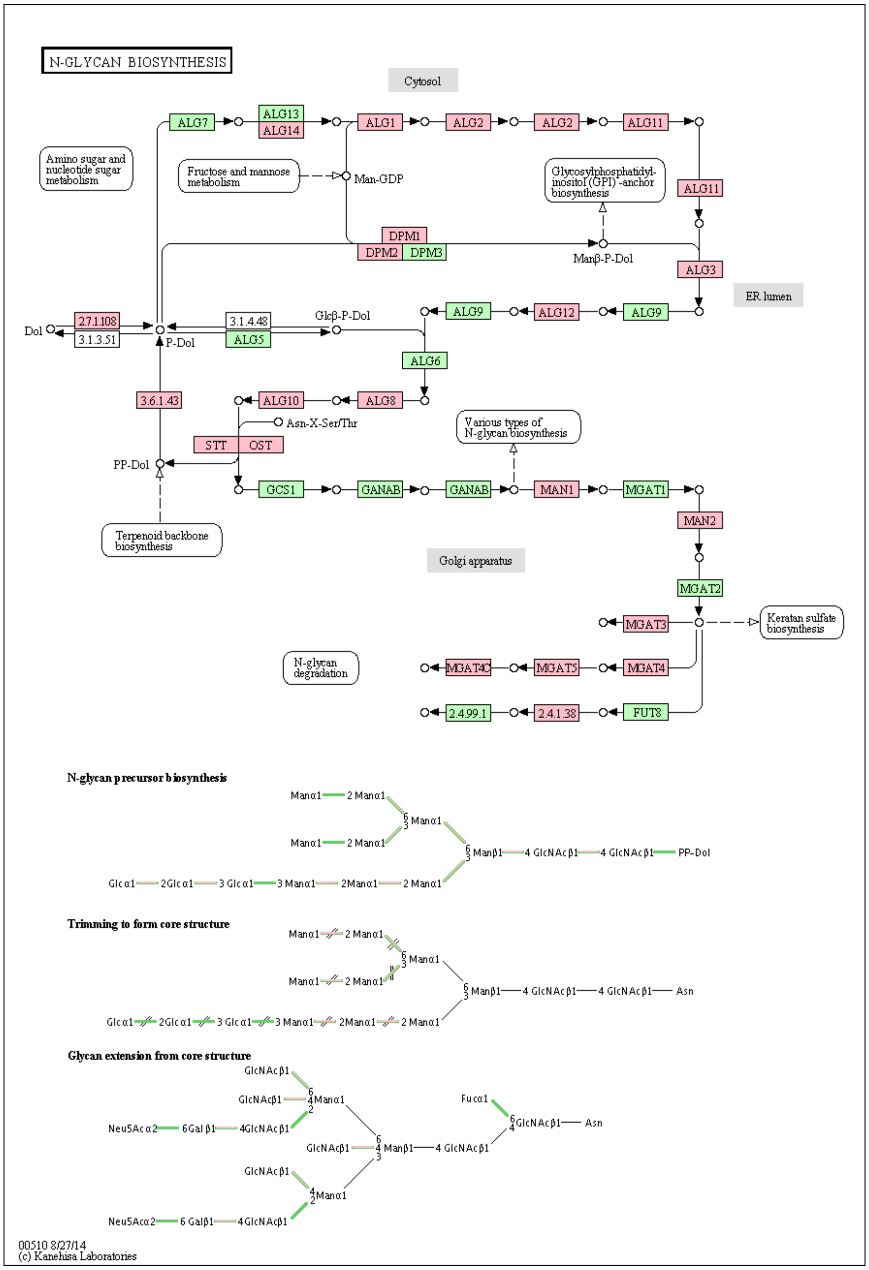

122.3 Legend:

RBH-Blast at 60% Identity + 50% Coverage
Green = Hit in *H. sapiens*
Red = Hit in *H. sapiens* and *C. milli*
White = Not in *H. sapiens*

123 Aminoacyl-tRNA biosynthesis

123.1 Human Pathway: HSA00970

123.2 Number of Hits: 32

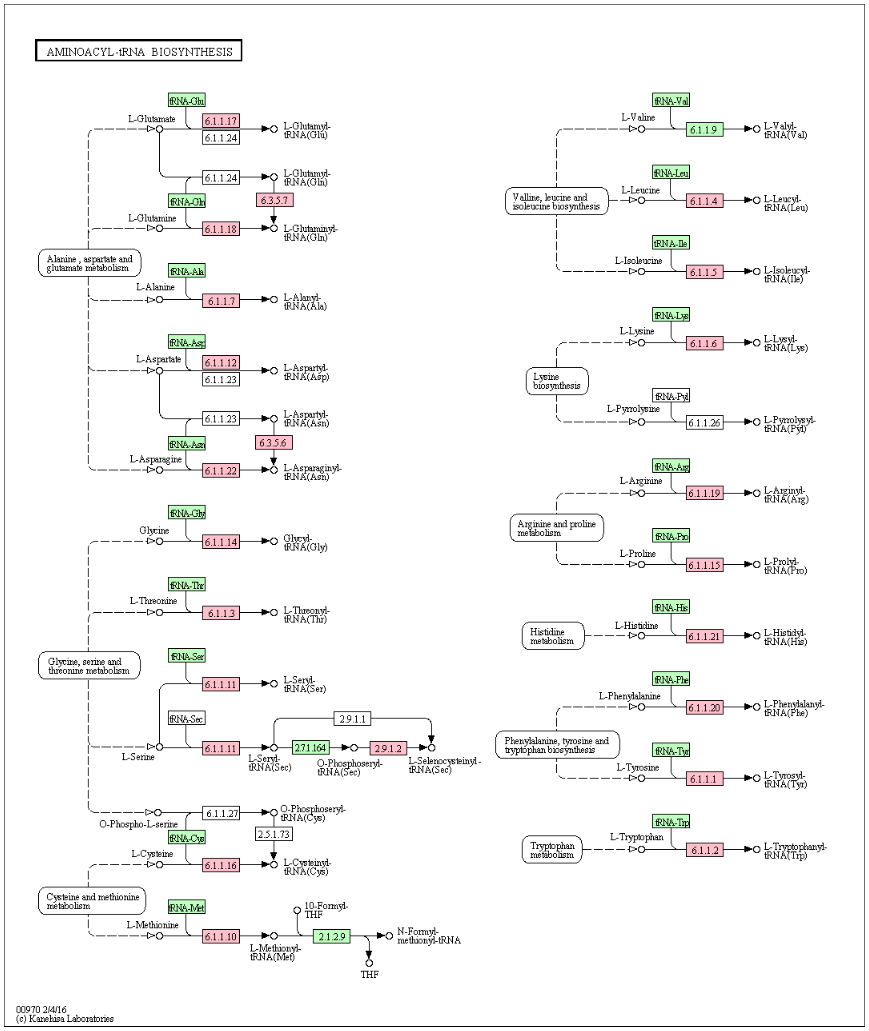

123.3 Legend:

RBH-Blast at 60% Identity + 50% Coverage
Green = Hit in *H. sapiens*
Red = Hit in *H. sapiens* and *C. milli*
White = Not in *H. sapiens*

124 Arrhythmogenic right ventricular cardiomyopathy (ARVC)

124.1 Human Pathway: HSA05412

124.2 Number of Hits: 32

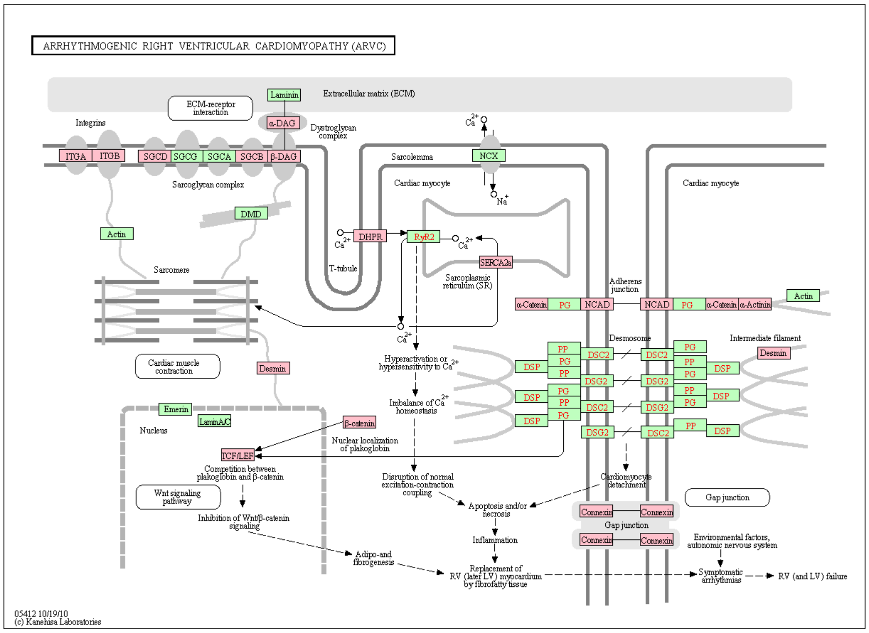

124.3 Legend:

RBH-Blast at 60% Identity + 50% Coverage
Green = Hit in *H. sapiens*
Red = Hit in *H. sapiens* and *C. milli*
White = Not in *H. sapiens*

125 TNF signaling pathway

125.1 Human Pathway: HSA04668

125.2 Number of Hits: 32

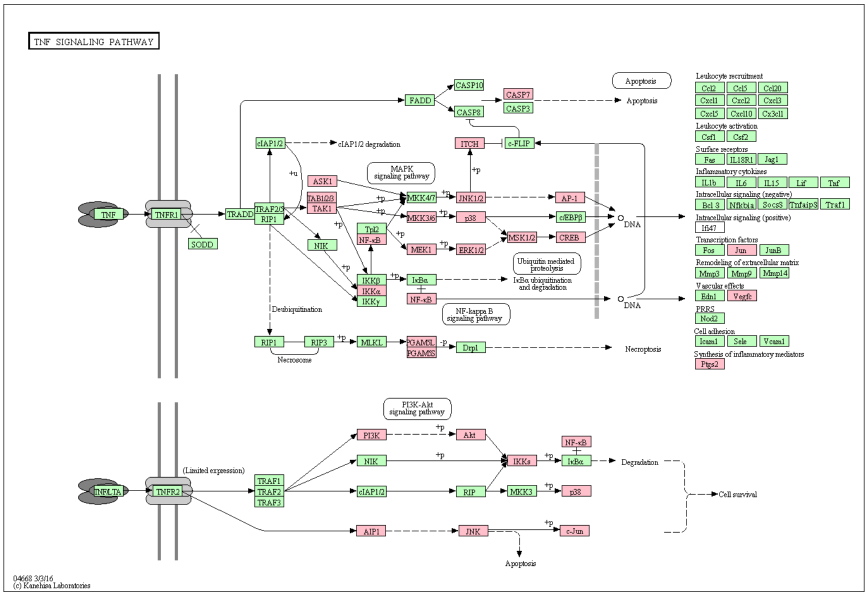

125.3 Legend:

RBH-Blast at 60% Identity + 50% Coverage
Green = Hit in *H. sapiens*
Red = Hit in *H. sapiens* and *C. milli*
White = Not in *H. sapiens*

126 Vibrio cholerae infection

126.1 Human Pathway: HSA05110

126.2 Number of Hits: 31

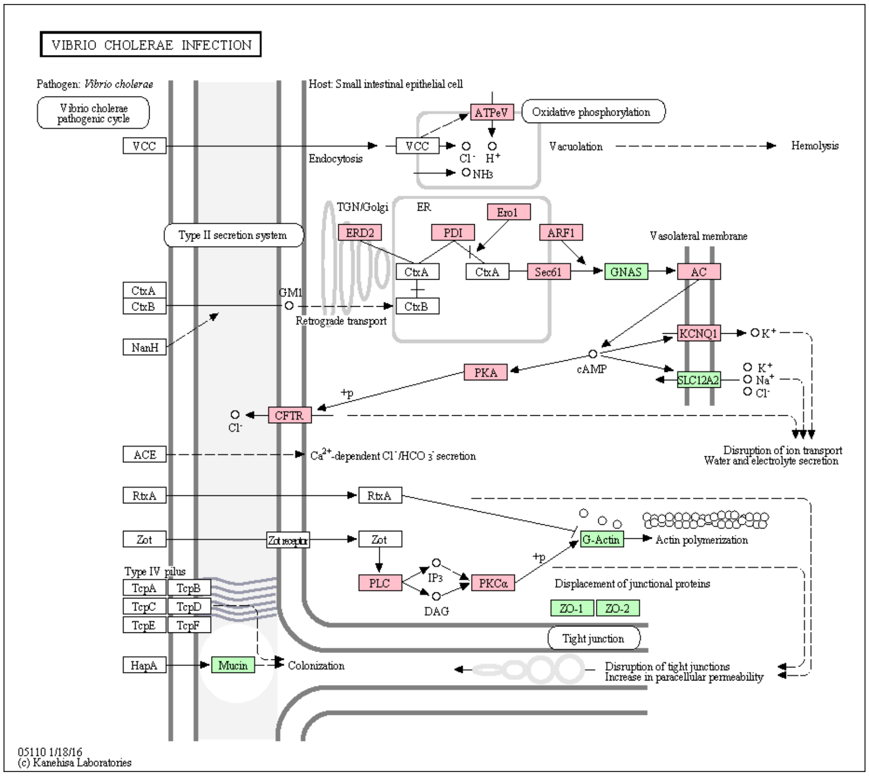

126.3 Legend:

RBH-Blast at 60% Identity + 50% Coverage
Green = Hit in *H. sapiens*
Red = Hit in *H. sapiens* and *C. milli*
White = Not in *H. sapiens*

127 Basal cell carcinoma

127.1 Human Pathway: HSA05217

127.2 Number of Hits: 31

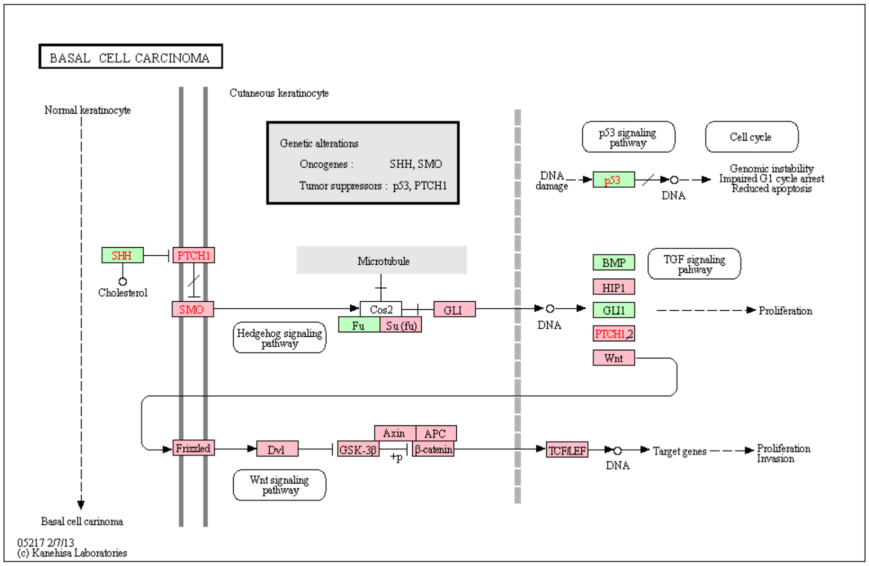

127.3 Legend:

RBH-Blast at 60% Identity + 50% Coverage
Green = Hit in *H. sapiens*
Red = Hit in *H. sapiens* and *C. milli*
White = Not in *H. sapiens*

128 Colorectal cancer

128.1 Human Pathway: HSA05210

128.2 Number of Hits: 31

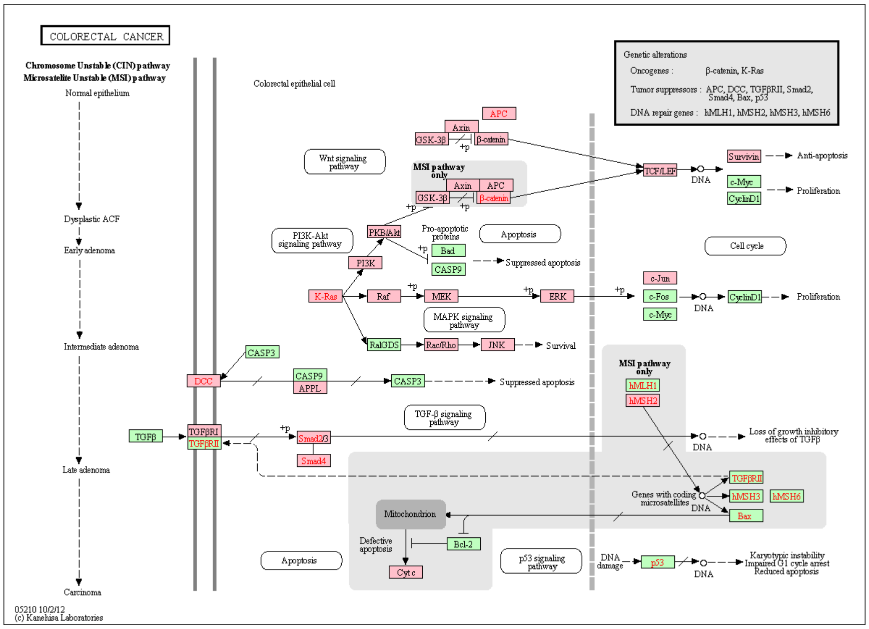

128.3 Legend:

RBH-Blast at 60% Identity + 50% Coverage
Green = Hit in *H. sapiens*
Red = Hit in *H. sapiens* and *C. milli*
White = Not in *H. sapiens*

129 Natural killer cell mediated cytotoxicity

129.1 Human Pathway: HSA04650

129.2 Number of Hits: 31

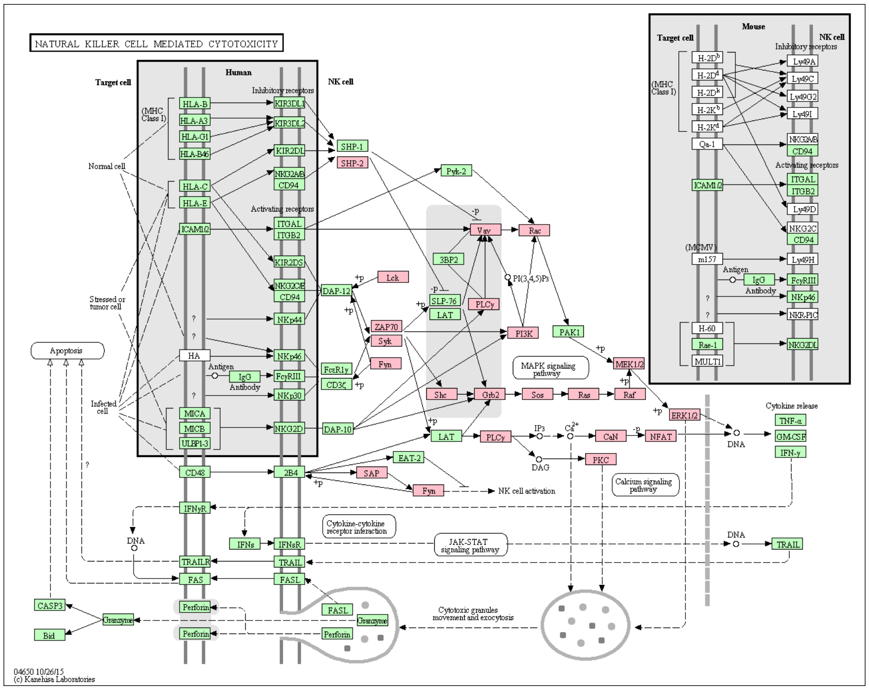

129.3 Legend:

RBH-Blast at 60% Identity + 50% Coverage
Green = Hit in *H. sapiens*
Red = Hit in *H. sapiens* and *C. milli*
White = Not in *H. sapiens*

130 Gastric acid secretion

130.1 Human Pathway: HSA04971

130.2 Number of Hits: 31

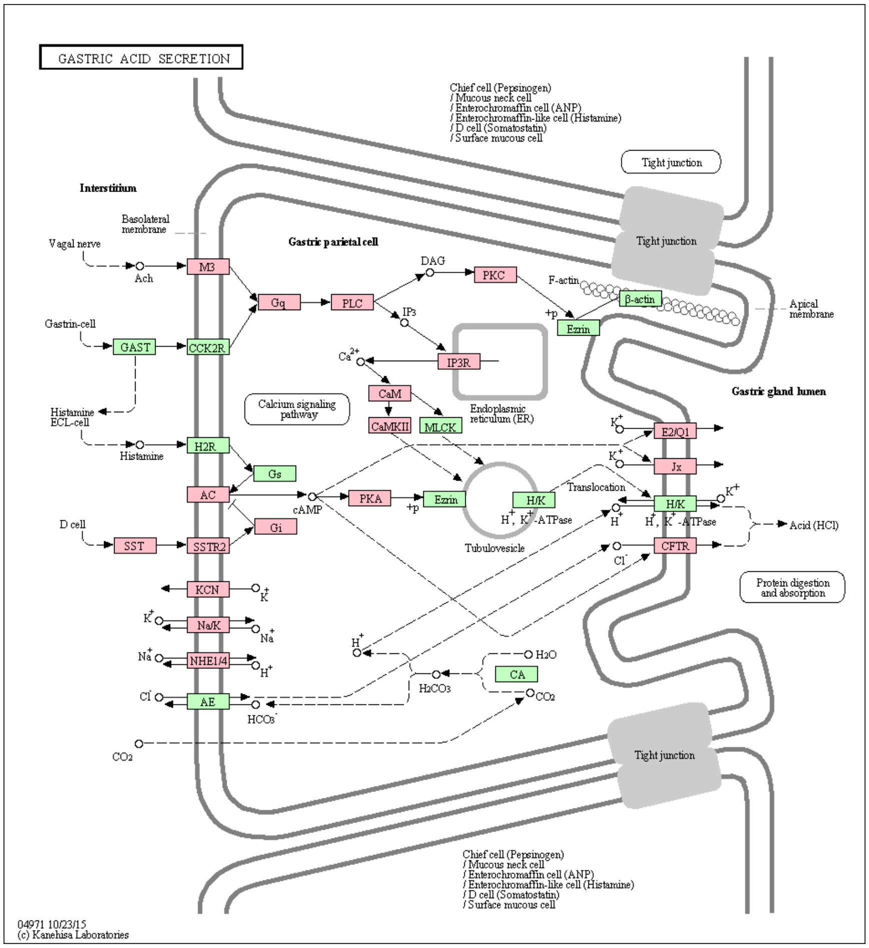

130.3 Legend:

RBH-Blast at 60% Identity + 50% Coverage
Green = Hit in *H. sapiens*
Red = Hit in *H. sapiens* and *C. milli*
White = Not in *H. sapiens*

131 Adipocytokine signaling pathway

131.1 Human Pathway: HSA04920

131.2 Number of Hits: 31

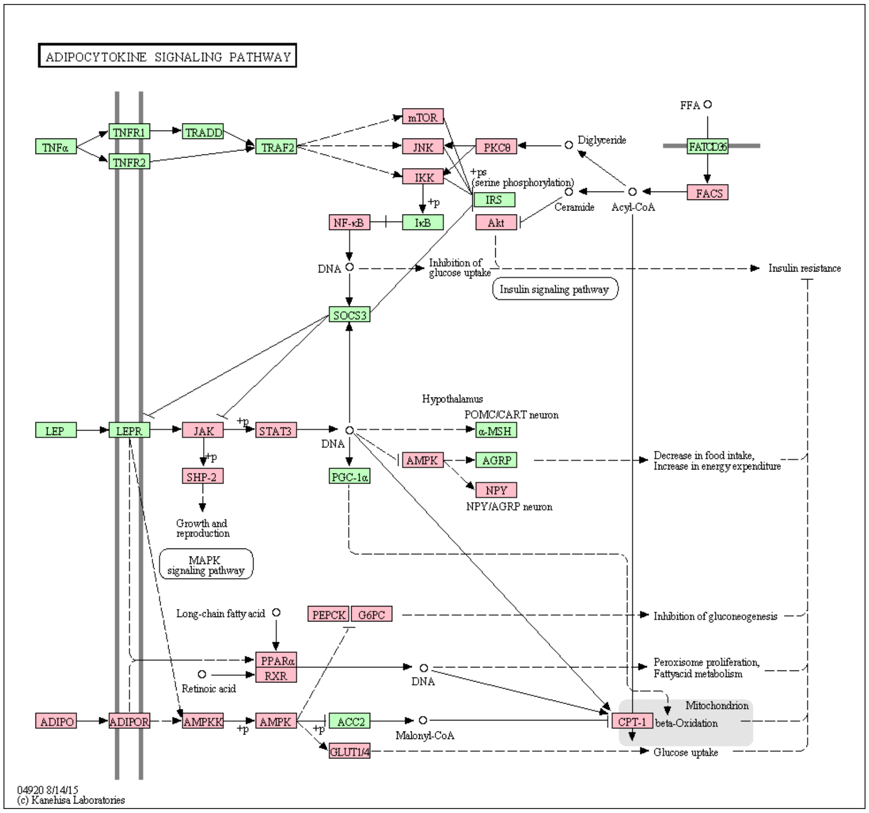

131.3 Legend:

RBH-Blast at 60% Identity + 50% Coverage
Green = Hit in *H. sapiens*
Red = Hit in *H. sapiens* and *C. milli*
White = Not in *H. sapiens*

132 Acute myeloid leukemia

132.1 Human Pathway: HSA05221

132.2 Number of Hits: 31

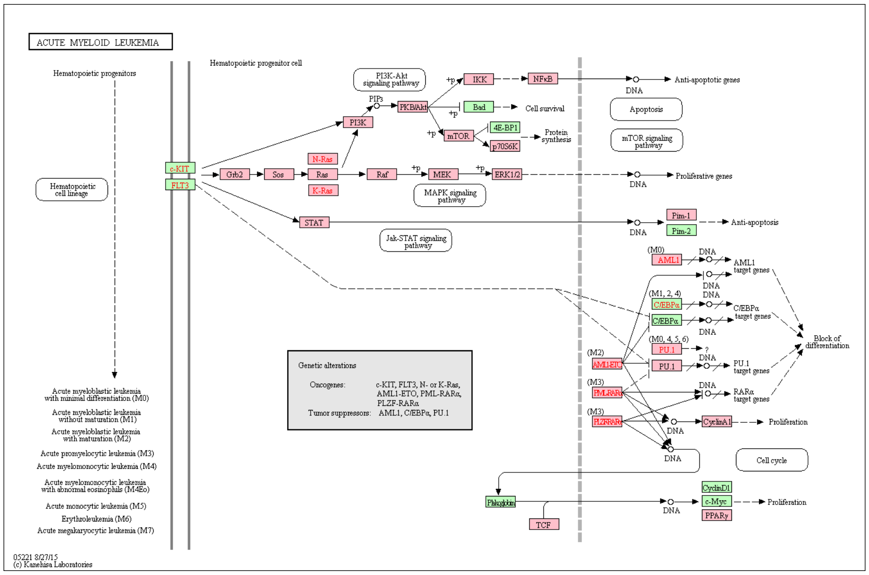

132.3 Legend:

RBH-Blast at 60% Identity + 50% Coverage
Green = Hit in *H. sapiens*
Red = Hit in *H. sapiens* and *C. milli*
White = Not in *H. sapiens*

133 Salivary secretion

133.1 Human Pathway: HSA04970

133.2 Number of Hits: 30

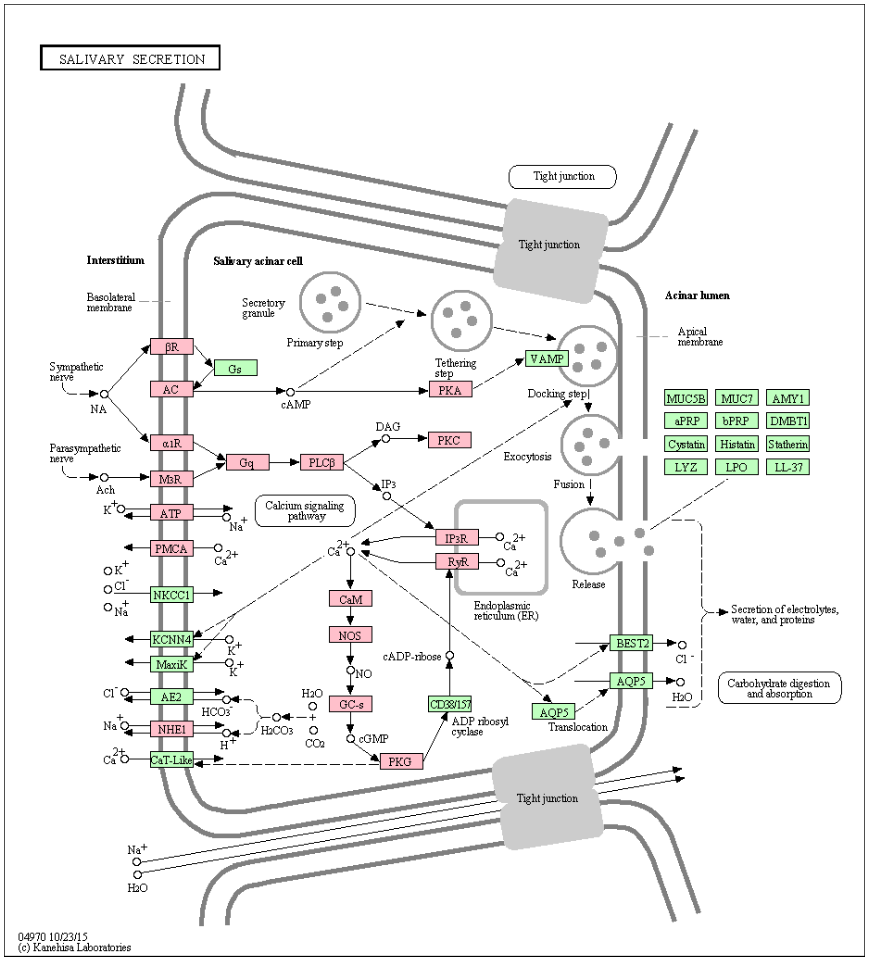

133.3 Legend:

RBH-Blast at 60% Identity + 50% Coverage
Green = Hit in *H. sapiens*
Red = Hit in *H. sapiens* and *C. milli*
White = Not in *H. sapiens*

134 Jak-STAT signaling pathway

134.1 Human Pathway: HSA04630

134.2 Number of Hits: 30

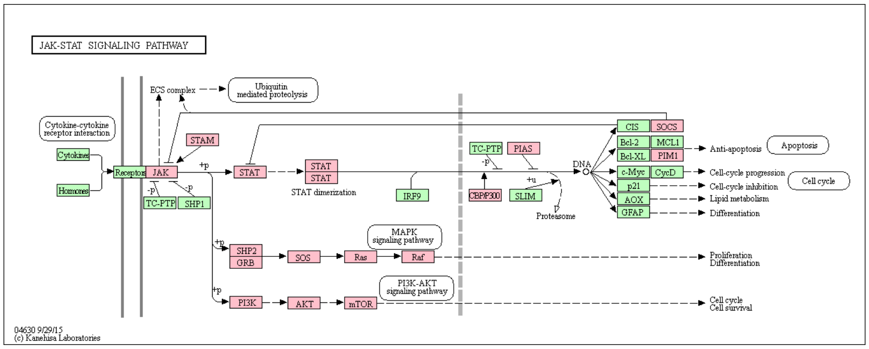

134.3 Legend:

RBH-Blast at 60% Identity + 50% Coverage
Green = Hit in *H. sapiens*
Red = Hit in *H. sapiens* and *C. milli*
White = Not in *H. sapiens*

135 Glioma

135.1 Human Pathway: HSA05214

135.2 Number of Hits: 30

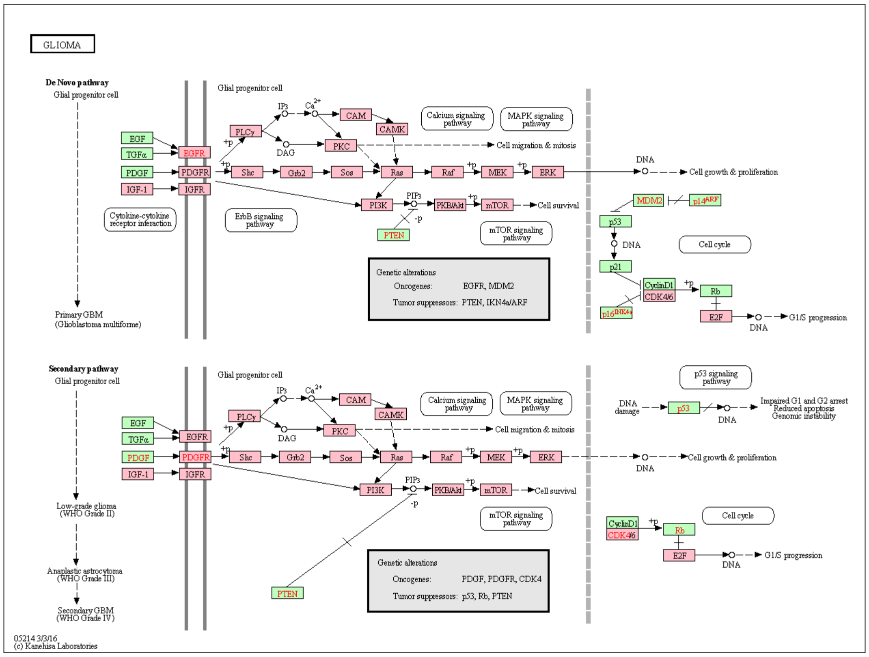

135.3 Legend:

RBH-Blast at 60% Identity + 50% Coverage
Green = Hit in *H. sapiens*
Red = Hit in *H. sapiens* and *C. milli*
White = Not in *H. sapiens*

136 Insulin secretion

136.1 Human Pathway: HSA04911

136.2 Number of Hits: 30

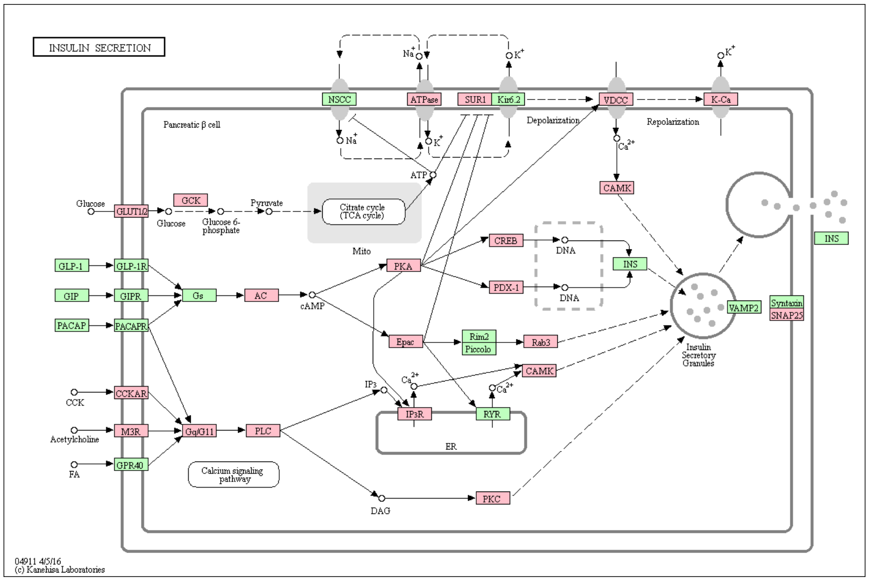

136.3 Legend:

RBH-Blast at 60% Identity + 50% Coverage
Green = Hit in *H. sapiens*
Red = Hit in *H. sapiens* and *C. milli*
White = Not in *H. sapiens*

137 Cardiac muscle contraction

137.1 Human Pathway: HSA04260

137.2 Number of Hits: 30

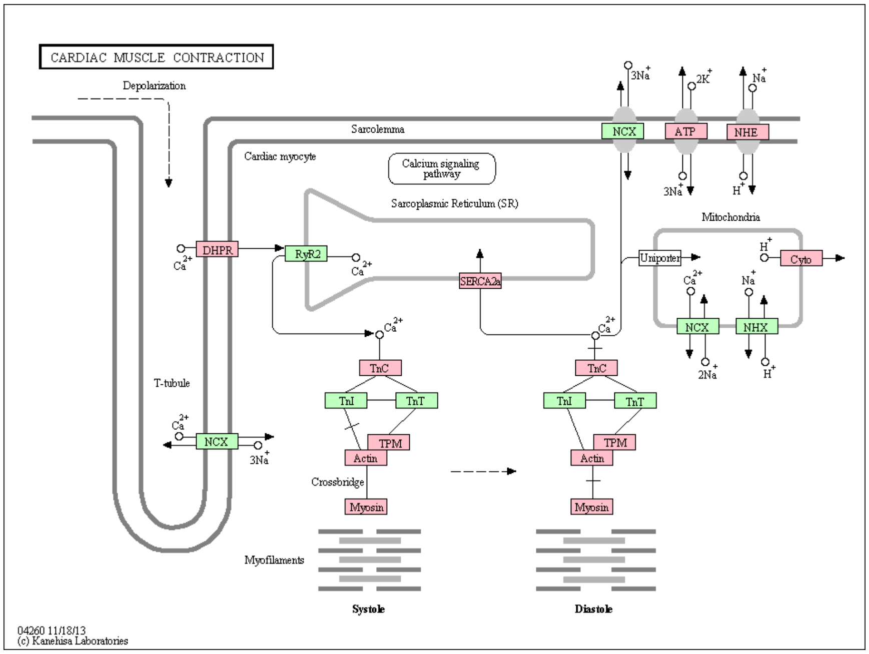

137.3 Legend:

RBH-Blast at 60% Identity + 50% Coverage
Green = Hit in *H. sapiens*
Red = Hit in *H. sapiens* and *C. milli*
White = Not in *H. sapiens*

138 Renin secretion

138.1 Human Pathway: HSA04924

138.2 Number of Hits: 29

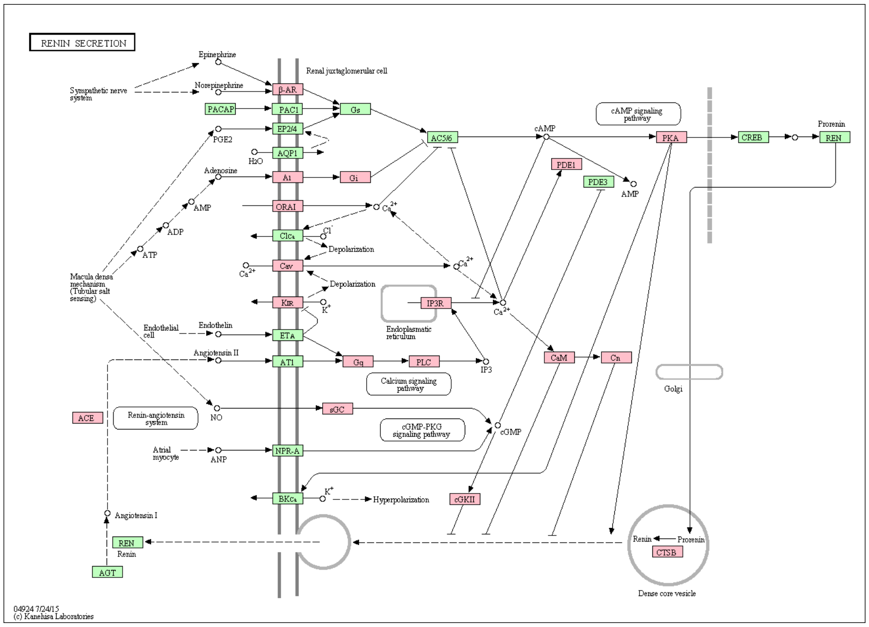

138.3 Legend:

RBH-Blast at 60% Identity + 50% Coverage
Green = Hit in *H. sapiens*
Red = Hit in *H. sapiens* and *C. milli*
White = Not in *H. sapiens*

139 Endometrial cancer

139.1 Human Pathway: HSA05213

139.2 Number of Hits: 29

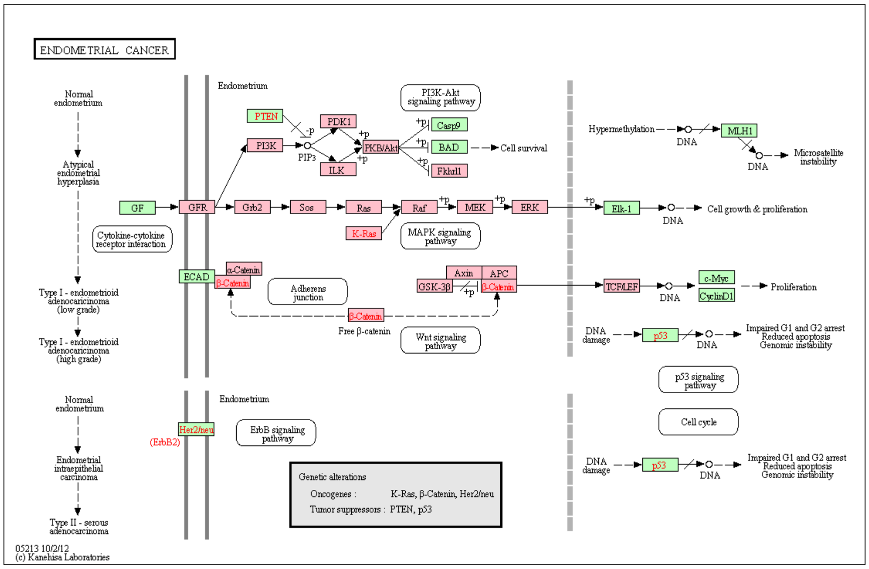

139.3 Legend:

RBH-Blast at 60% Identity + 50% Coverage
Green = Hit in *H. sapiens*
Red = Hit in *H. sapiens* and *C. milli*
White = Not in *H. sapiens*

140 Glycolysis / Gluconeogenesis

140.1 Human Pathway: HSA00010

140.2 Number of Hits: 29

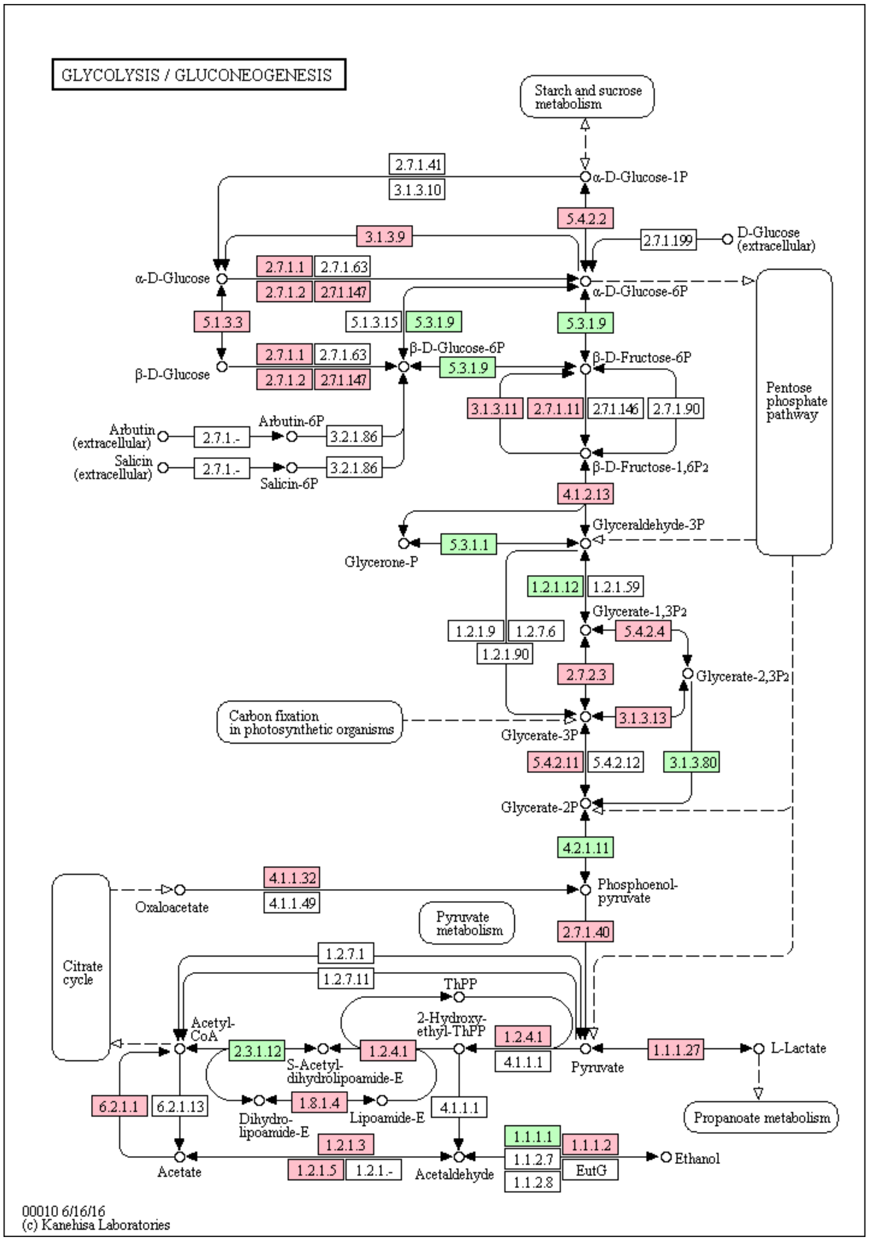

140.3 Legend:

RBH-Blast at 60% Identity + 50% Coverage
Green = Hit in *H. sapiens*
Red = Hit in *H. sapiens* and *C. milli*
White = Not in *H. sapiens*

141 VEGF signaling pathway

141.1 Human Pathway: HSA04370

141.2 Number of Hits: 29

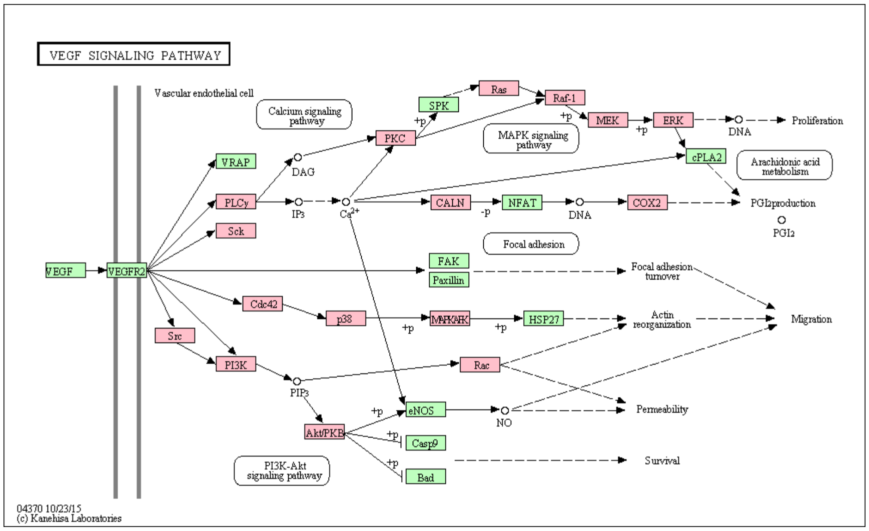

141.3 Legend:

RBH-Blast at 60% Identity + 50% Coverage
Green = Hit in *H. sapiens*
Red = Hit in *H. sapiens* and *C. milli*
White = Not in *H. sapiens*

142 Non-small cell lung cancer

142.1 Human Pathway: HSA05223

142.2 Number of Hits: 29

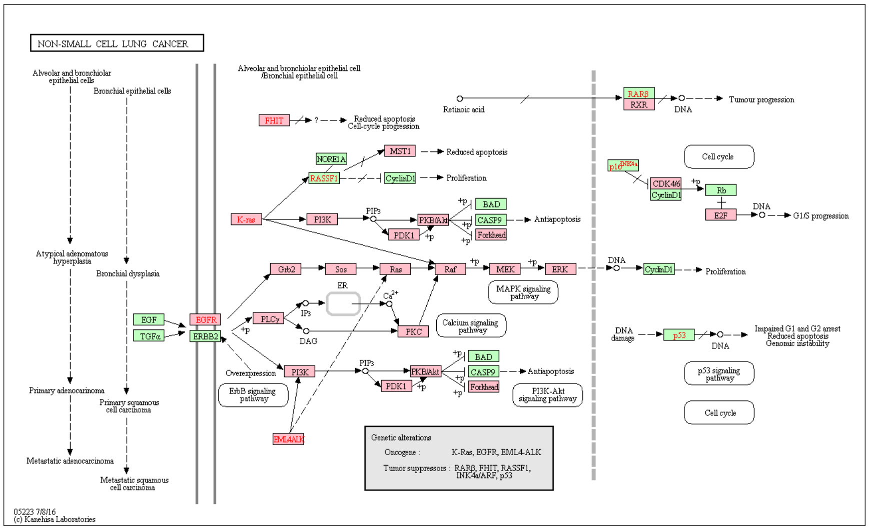

142.3 Legend:

RBH-Blast at 60% Identity + 50% Coverage
Green = Hit in *H. sapiens*
Red = Hit in *H. sapiens* and *C. milli*
White = Not in *H. sapiens*

143 Small cell lung cancer

143.1 Human Pathway: HSA05222

143.2 Number of Hits: 29

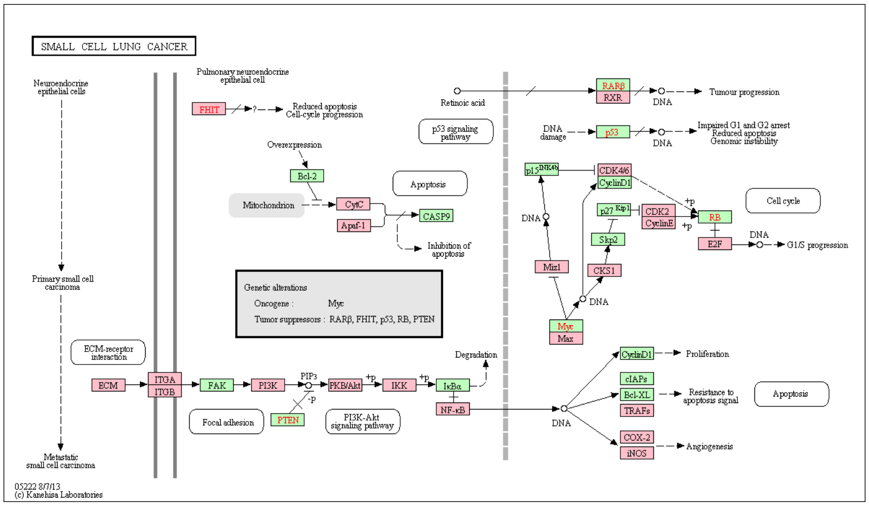

143.3 Legend:

RBH-Blast at 60% Identity + 50% Coverage
Green = Hit in *H. sapiens*
Red = Hit in *H. sapiens* and *C. milli*
White = Not in *H. sapiens*

---

144 Nicotine addiction

144.1 Human Pathway: HSA05033

144.2 Number of Hits: 28

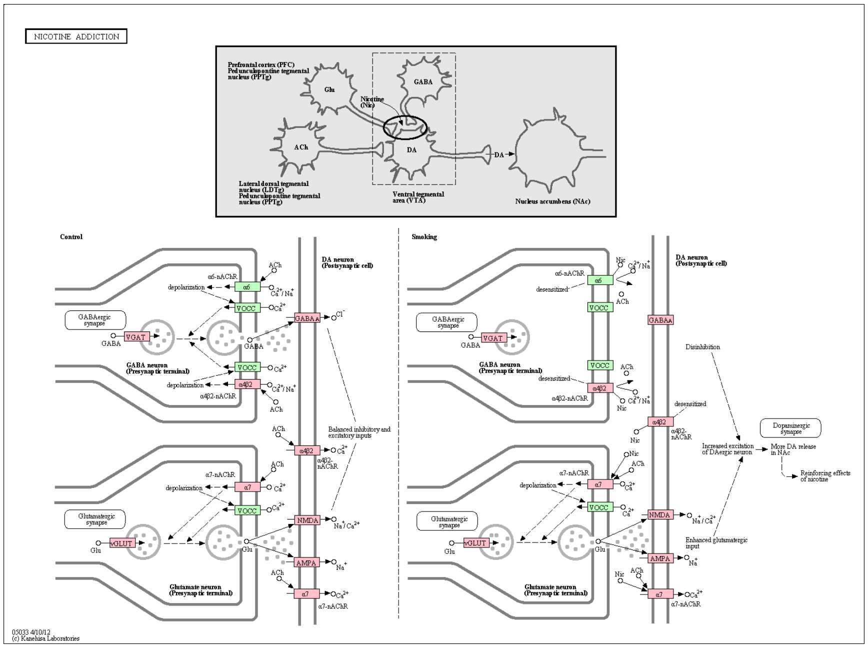

144.3 Legend:

RBH-Blast at 60% Identity + 50% Coverage
Green = Hit in *H. sapiens*
Red = Hit in *H. sapiens* and *C. milli*
White = Not in *H. sapiens*

145 Toll-like receptor signaling pathway

145.1 Human Pathway: HSA04620

145.2 Number of Hits: 28

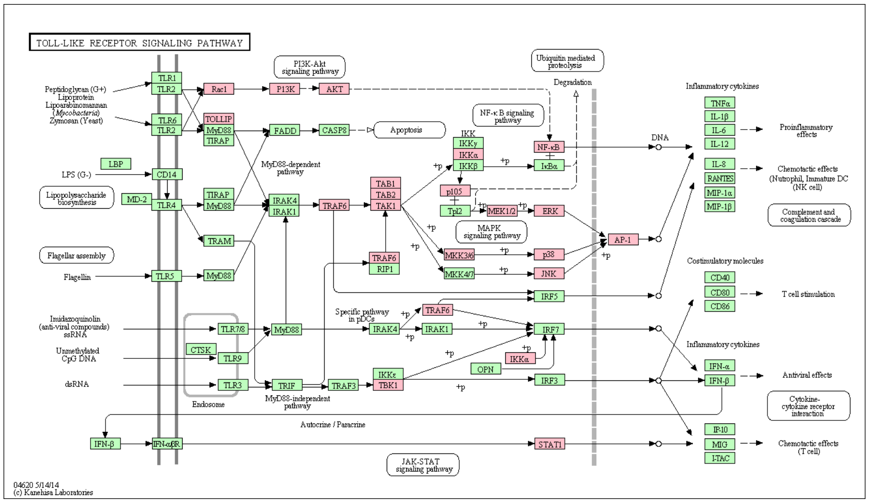

145.3 Legend:

RBH-Blast at 60% Identity + 50% Coverage
Green = Hit in *H. sapiens*
Red = Hit in *H. sapiens* and *C. milli*
White = Not in *H. sapiens*

146 Arginine and proline metabolism

146.1 Human Pathway: HSA00330

146.2 Number of Hits: 28

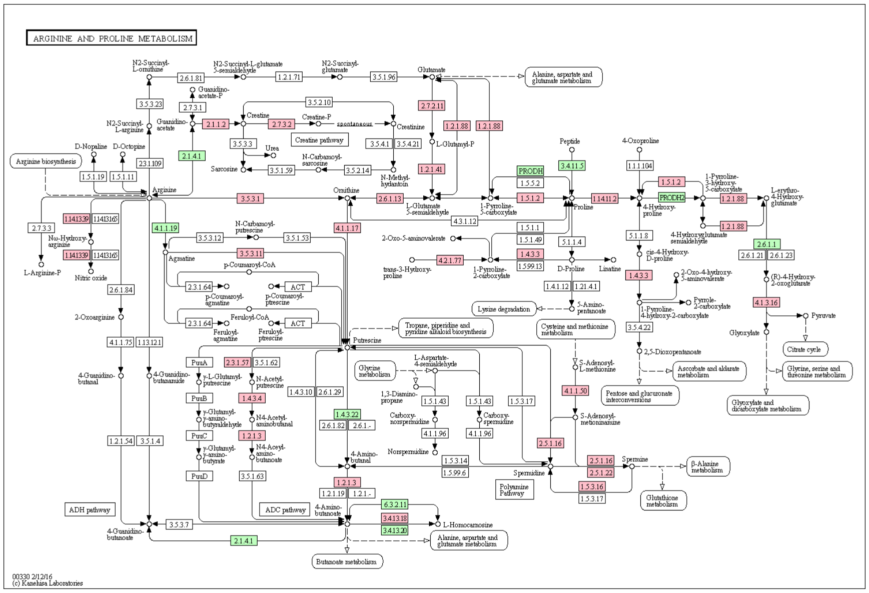

146.3 Legend:

RBH-Blast at 60% Identity + 50% Coverage
Green = Hit in *H. sapiens*
Red = Hit in *H. sapiens* and *C. milli*
White = Not in *H. sapiens*

147 Aldosterone synthesis and secretion

147.1 Human Pathway: HSA04925

147.2 Number of Hits: 28

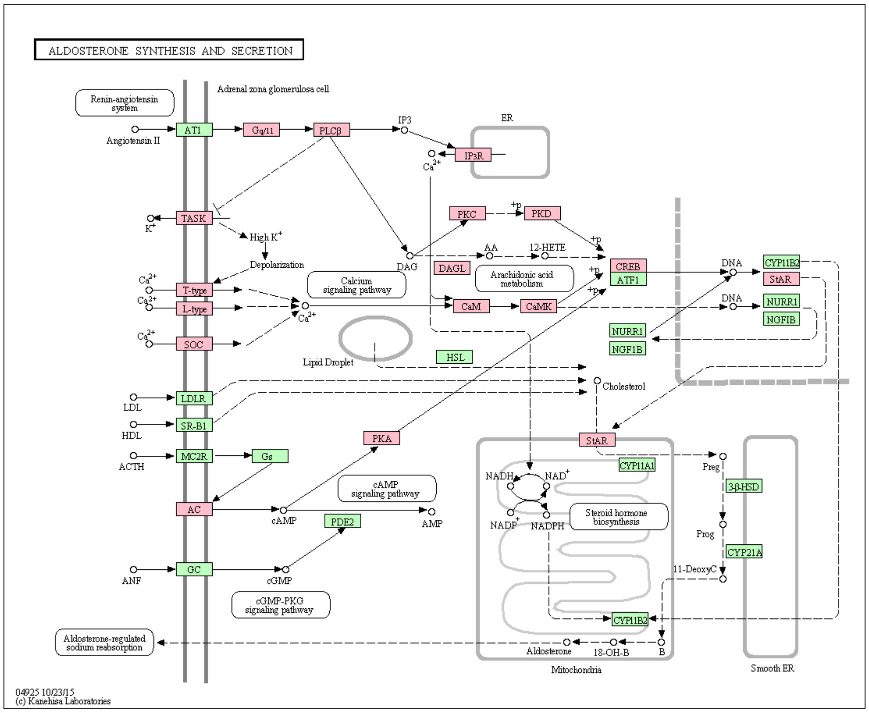

147.3 Legend:

RBH-Blast at 60% Identity + 50% Coverage
Green = Hit in *H. sapiens*
Red = Hit in *H. sapiens* and *C. milli*
White = Not in *H. sapiens*

148 Basal transcription factors

148.1 Human Pathway: HSA03022

148.2 Number of Hits: 28

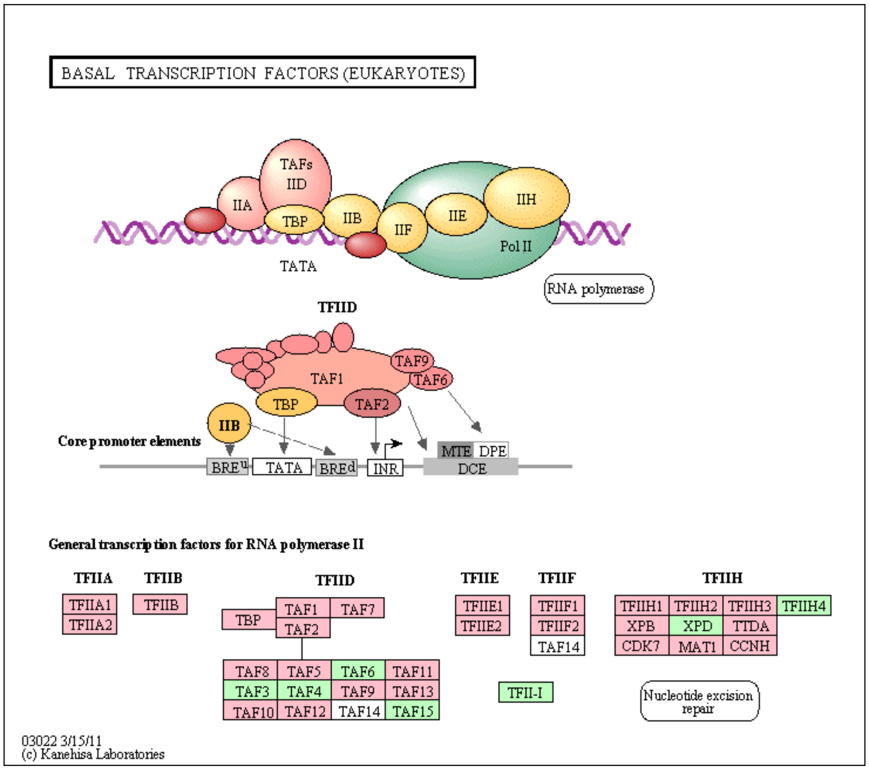

148.3 Legend:

RBH-Blast at 60% Identity + 50% Coverage
Green = Hit in *H. sapiens*
Red = Hit in *H. sapiens* and *C. milli*
White = Not in *H. sapiens*

149 Valine, leucine and isoleucine degradation

149.1 Human Pathway: HSA00280

149.2 Number of Hits: 27

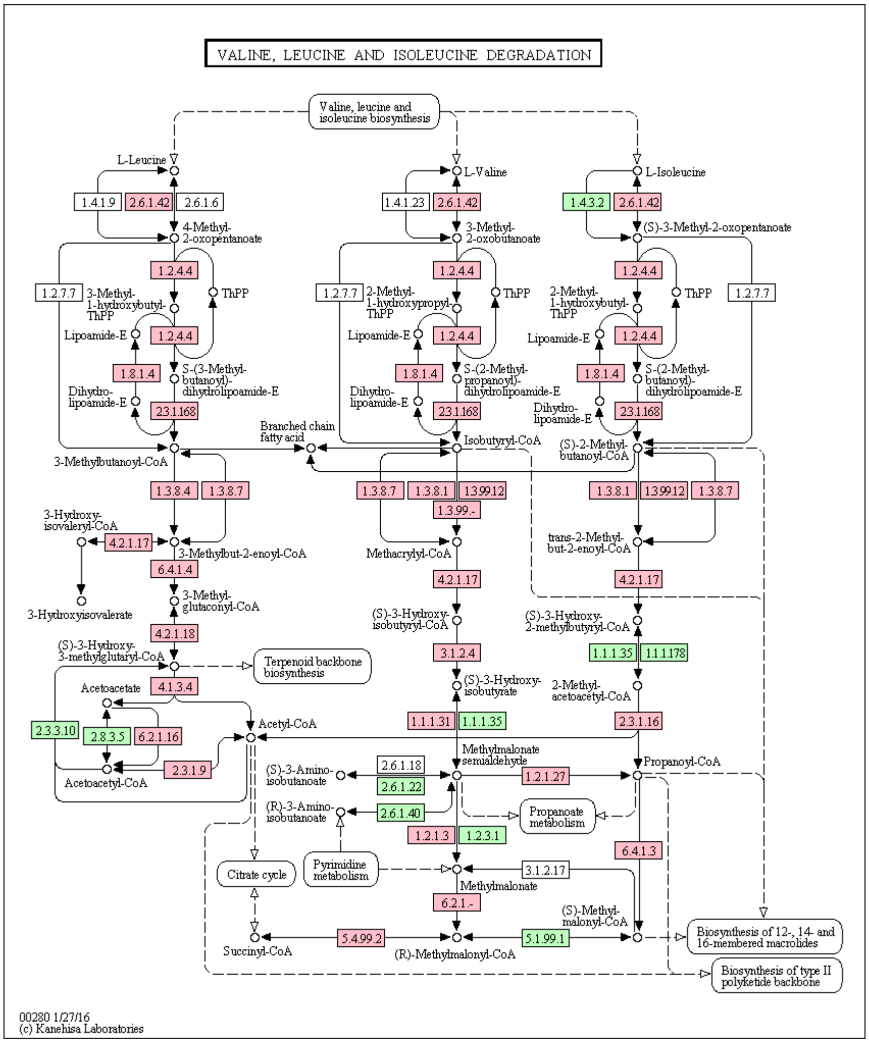

149.3 Legend:

RBH-Blast at 60% Identity + 50% Coverage
Green = Hit in *H. sapiens*
Red = Hit in *H. sapiens* and *C. milli*
White = Not in *H. sapiens*

150 Nucleotide excision repair

150.1 Human Pathway: HSA03420

150.2 Number of Hits: 27

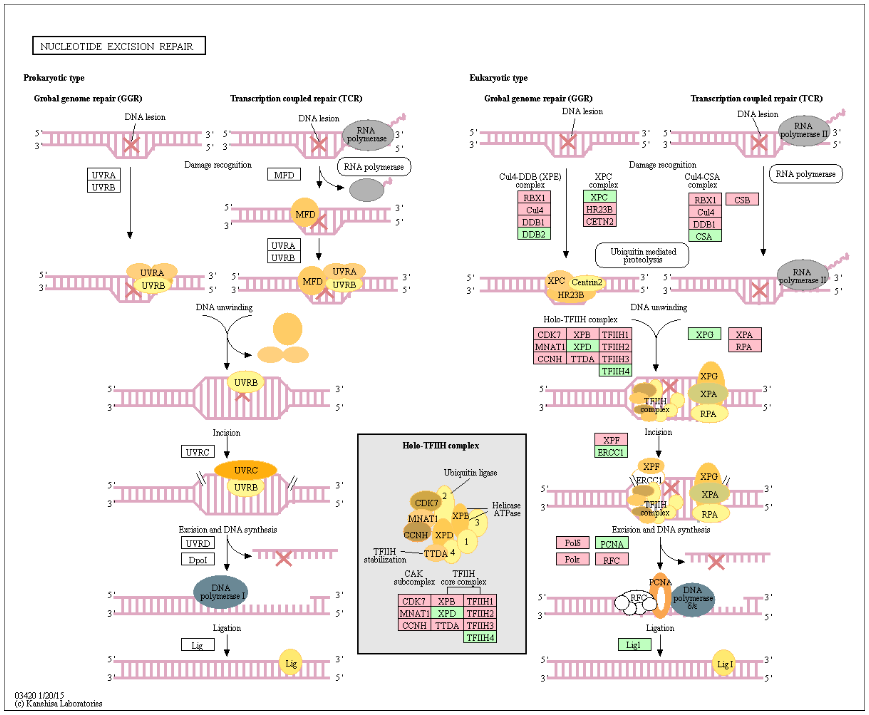

150.3 Legend:

RBH-Blast at 60% Identity + 50% Coverage
Green = Hit in *H. sapiens*
Red = Hit in *H. sapiens* and *C. milli*
White = Not in *H. sapiens*

151 p53 signaling pathway

151.1 Human Pathway: HSA04115

151.2 Number of Hits: 27

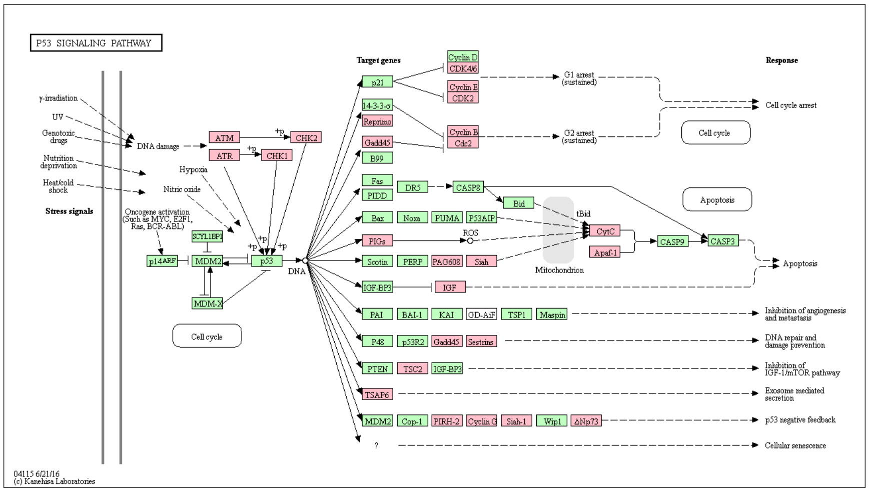

151.3 Legend:

RBH-Blast at 60% Identity + 50% Coverage
Green = Hit in *H. sapiens*
Red = Hit in *H. sapiens* and *C. milli*
White = Not in *H. sapiens*

152 Amphetamine addiction

152.1 Human Pathway: HSA05031

152.2 Number of Hits: 27

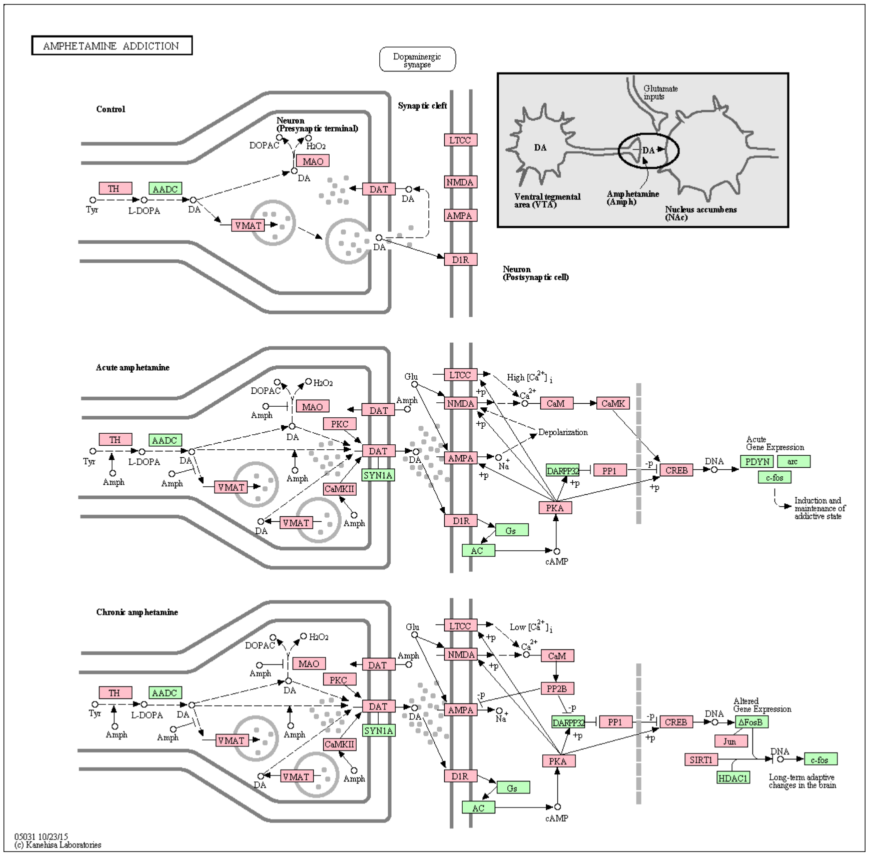

152.3 Legend:

RBH-Blast at 60% Identity + 50% Coverage
Green = Hit in *H. sapiens*
Red = Hit in *H. sapiens* and *C. milli*
White = Not in *H. sapiens*

153 Sphingolipid metabolism

153.1 Human Pathway: HSA00600

153.2 Number of Hits: 27

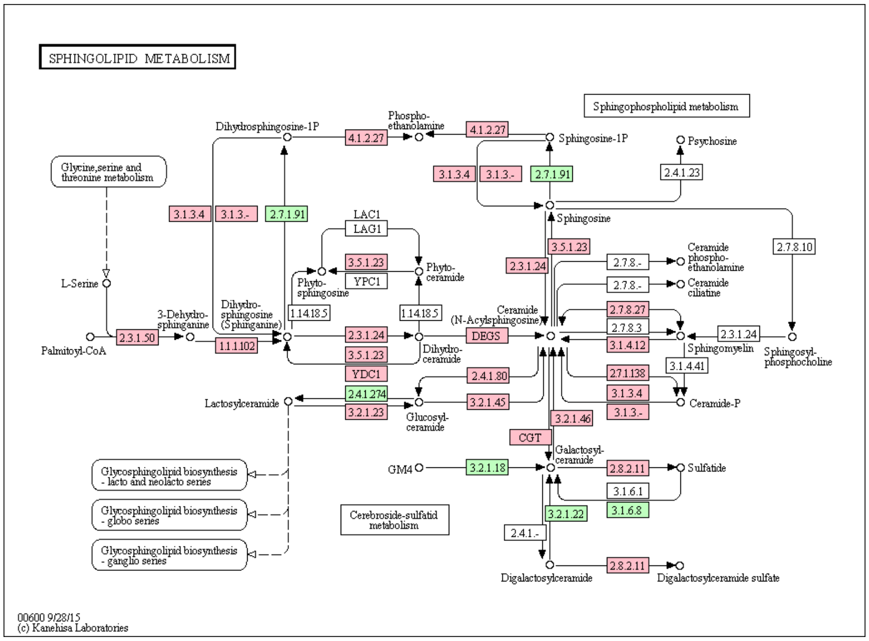

153.3 Legend:

RBH-Blast at 60% Identity + 50% Coverage
Green = Hit in *H. sapiens*
Red = Hit in *H. sapiens* and *C. milli*
White = Not in *H. sapiens*

154 Amino sugar and nucleotide sugar metabolism

154.1 Human Pathway: HSA00520

154.2 Number of Hits: 27

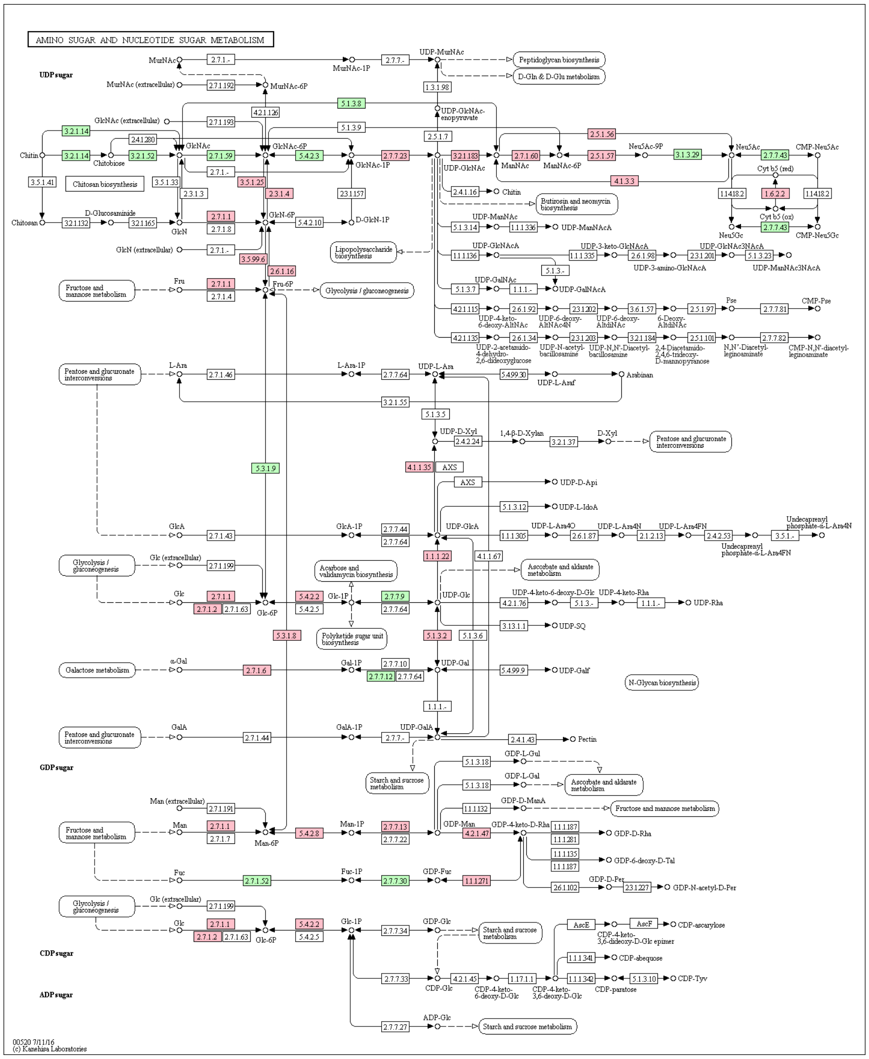

154.3 Legend:

RBH-Blast at 60% Identity + 50% Coverage
Green = Hit in *H. sapiens*
Red = Hit in *H. sapiens* and *C. milli*
White = Not in *H. sapiens*

---

155 Proteasome

155.1 Human Pathway: HSA03050

155.2 Number of Hits: 27

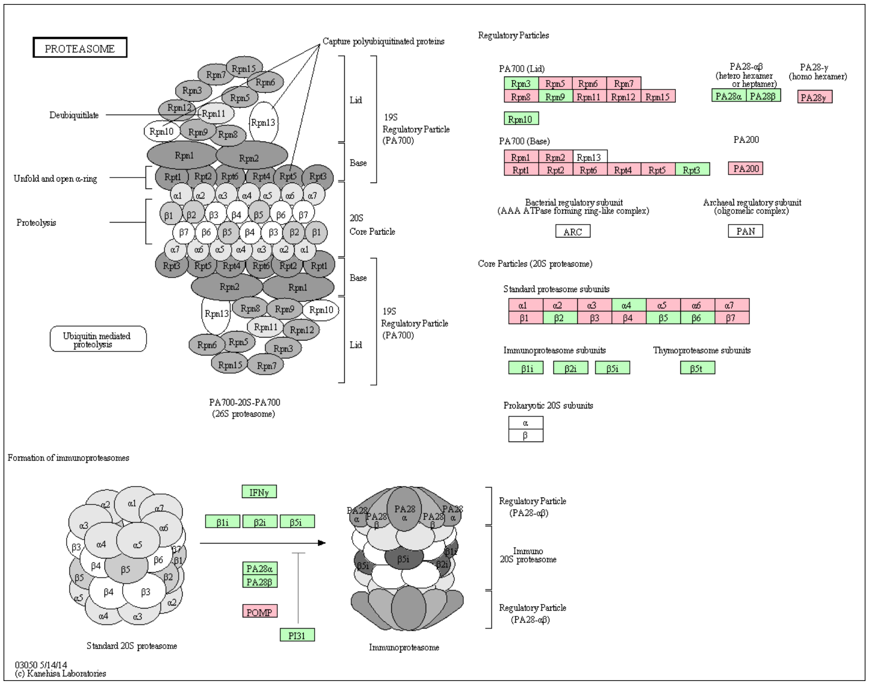

155.3 Legend:

RBH-Blast at 60% Identity + 50% Coverage
Green = Hit in *H. sapiens*
Red = Hit in *H. sapiens* and *C. milli*
White = Not in *H. sapiens*

---

156 Amoebiasis

156.1 Human Pathway: HSA05146

156.2 Number of Hits: 26

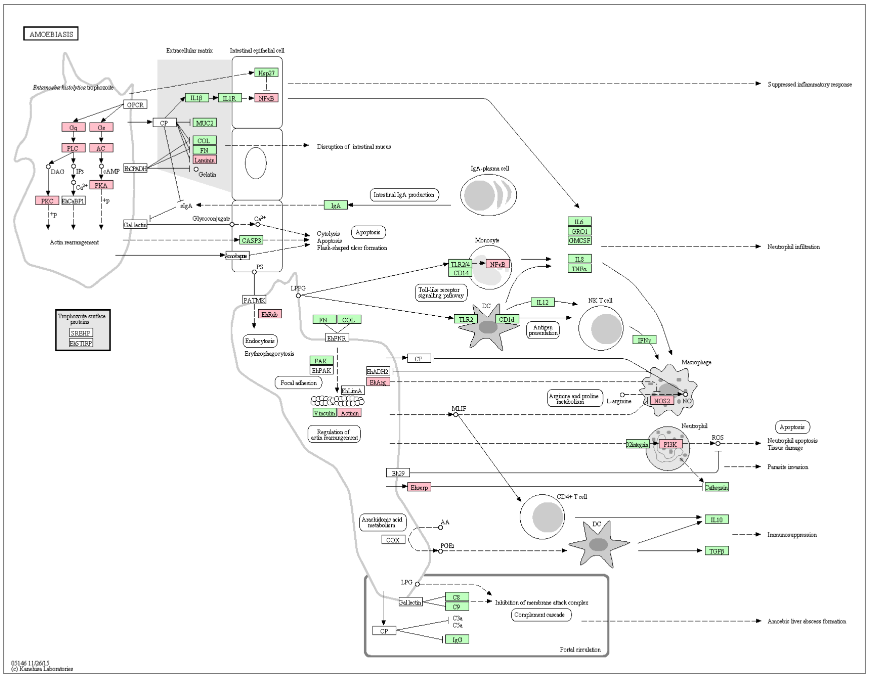

156.3 Legend:

RBH-Blast at 60% Identity + 50% Coverage
Green = Hit in *H. sapiens*
Red = Hit in *H. sapiens* and *C. milli*
White = Not in *H. sapiens*

157 Cysteine and methionine metabolism

157.1 Human Pathway: HSA00270

157.2 Number of Hits: 26

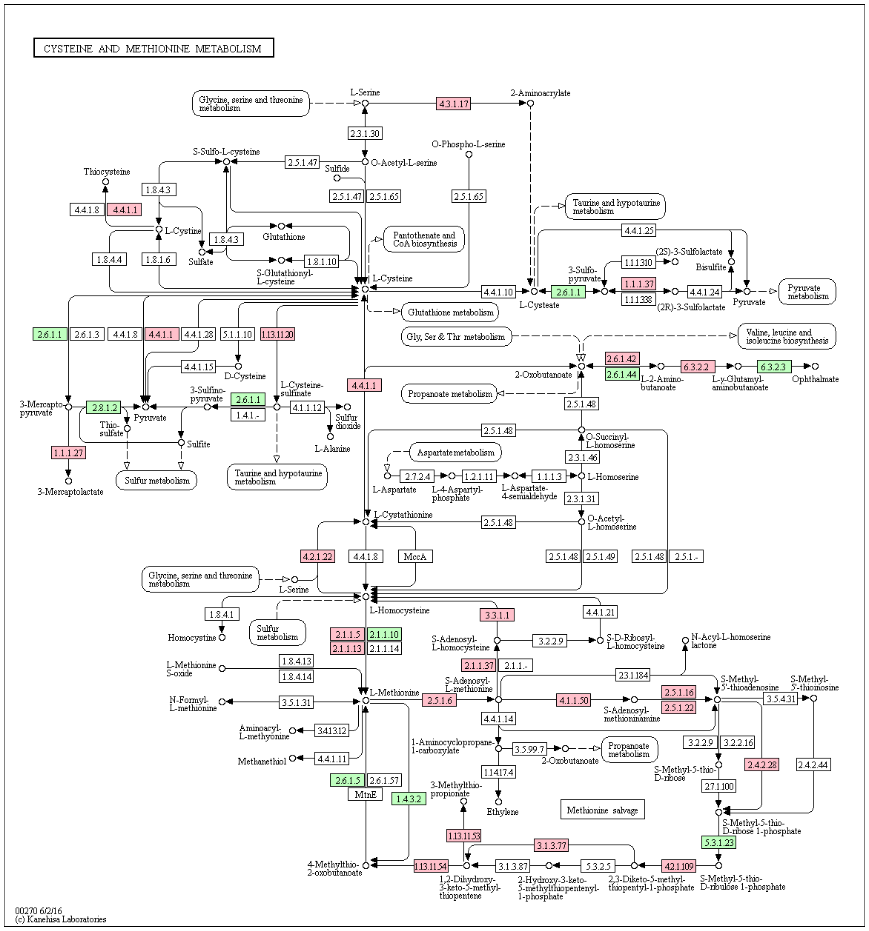

157.3 Legend:

RBH-Blast at 60% Identity + 50% Coverage
Green = Hit in *H. sapiens*
Red = Hit in *H. sapiens* and *C. milli*
White = Not in *H. sapiens*

158 Glycerolipid metabolism

158.1 Human Pathway: HSA00561

158.2 Number of Hits: 26

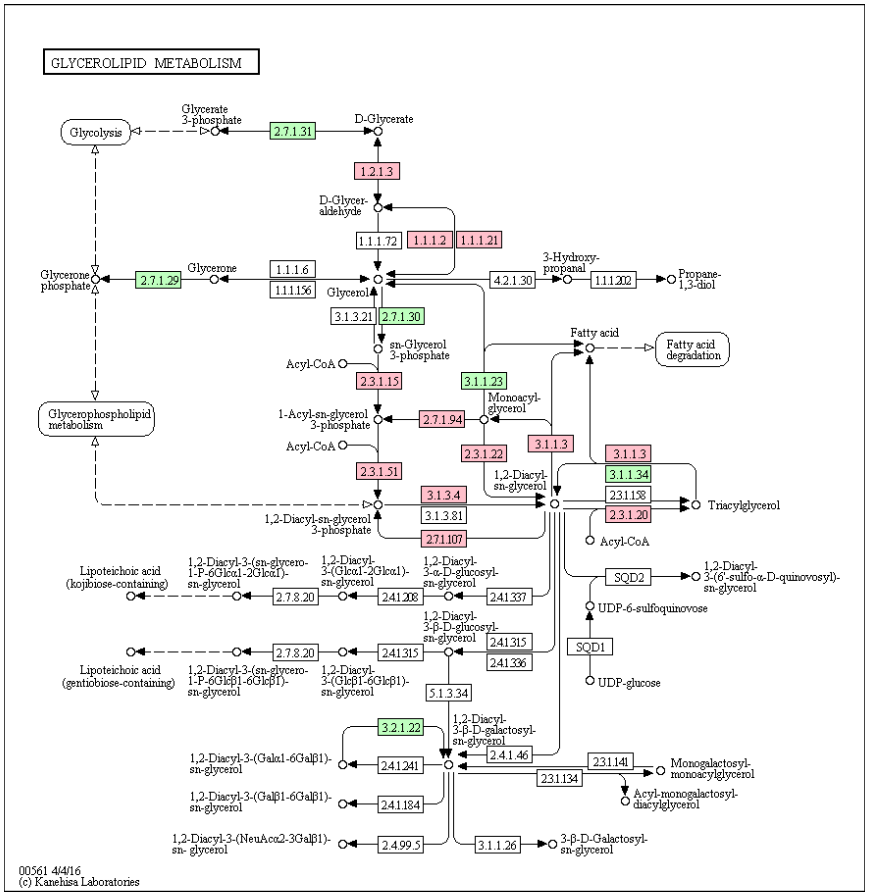

158.3 Legend:

RBH-Blast at 60% Identity + 50% Coverage
Green = Hit in *H. sapiens*
Red = Hit in *H. sapiens* and *C. milli*
White = Not in *H. sapiens*

159 Regulation of lipolysis in adipocytes

159.1 Human Pathway: HSA04923

159.2 Number of Hits: 26

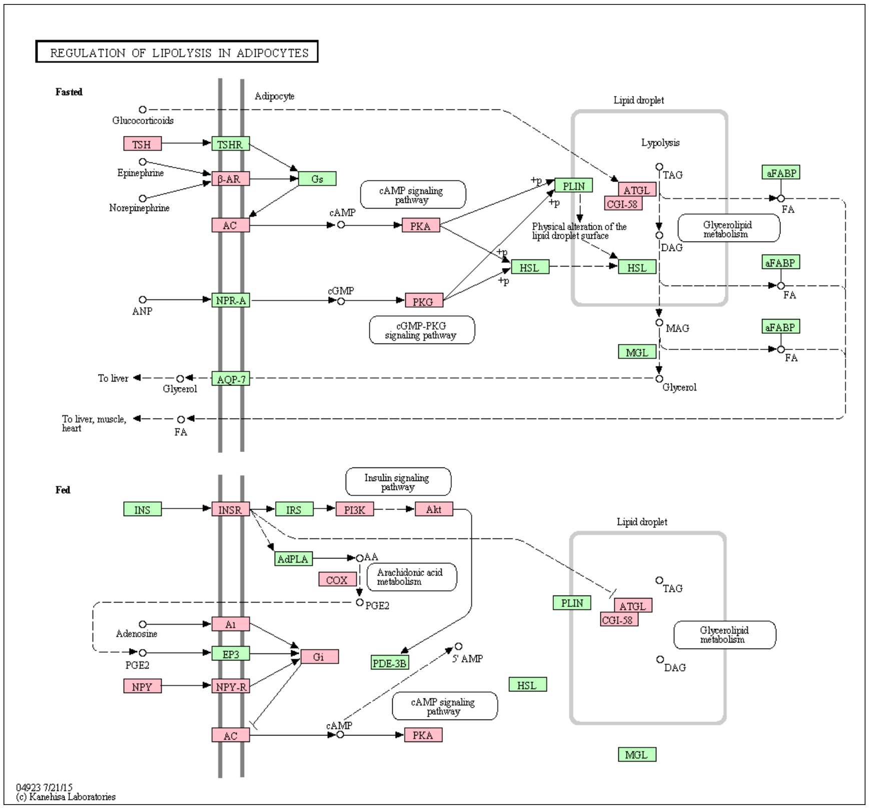

159.3 Legend:

RBH-Blast at 60% Identity + 50% Coverage
Green = Hit in *H. sapiens*
Red = Hit in *H. sapiens* and *C. milli*
White = Not in *H. sapiens*

160 Cell adhesion molecules (CAMs)

160.1 Human Pathway: HSA04514

160.2 Number of Hits: 26

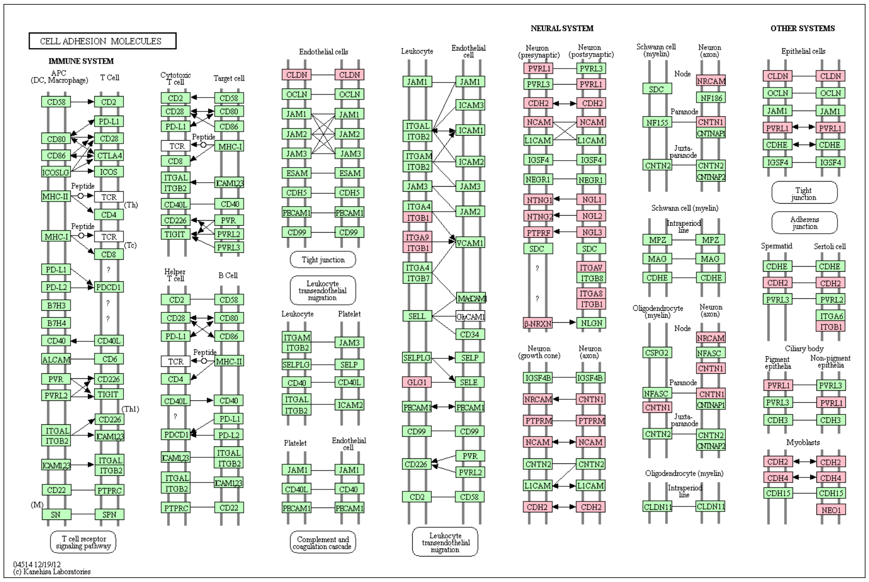

160.3 Legend:

RBH-Blast at 60% Identity + 50% Coverage
Green = Hit in *H. sapiens*
Red = Hit in *H. sapiens* and *C. milli*
White = Not in *H. sapiens*

161 Taste transduction

161.1 Human Pathway: HSA04742

161.2 Number of Hits: 25

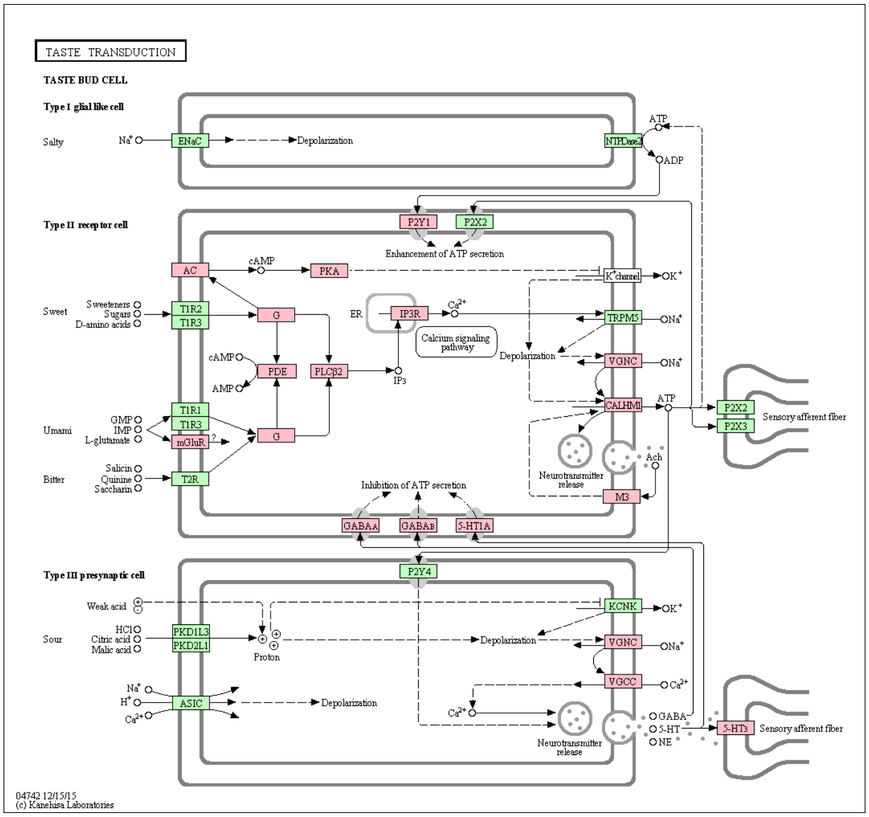

161.3 Legend:

RBH-Blast at 60% Identity + 50% Coverage
Green = Hit in *H. sapiens*
Red = Hit in *H. sapiens* and *C. milli*
White = Not in *H. sapiens*

162 PPAR signaling pathway

162.1 Human Pathway: HSA03320

162.2 Number of Hits: 25

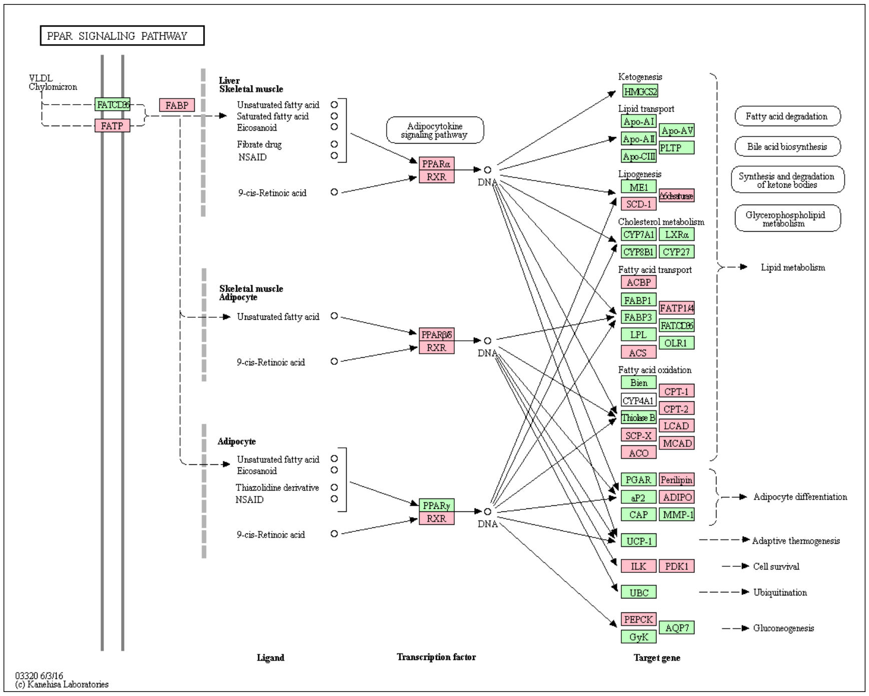

162.3 Legend:

RBH-Blast at 60% Identity + 50% Coverage
Green = Hit in *H. sapiens*
Red = Hit in *H. sapiens* and *C. milli*
White = Not in *H. sapiens*

163 Amyotrophic lateral sclerosis (ALS)

163.1 Human Pathway: HSA05014

163.2 Number of Hits: 25

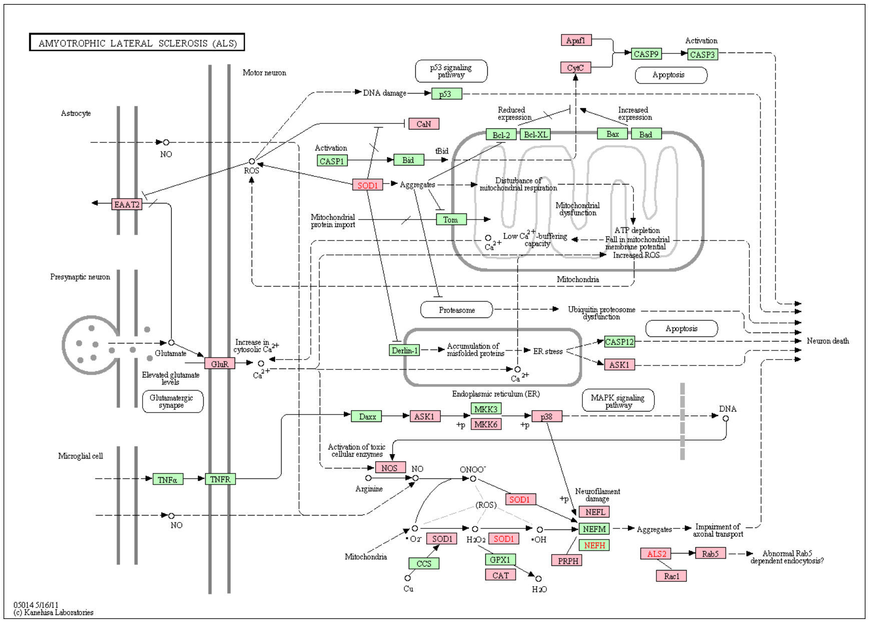

163.3 Legend:

RBH-Blast at 60% Identity + 50% Coverage
Green = Hit in *H. sapiens*
Red = Hit in *H. sapiens* and *C. milli*
White = Not in *H. sapiens*

164 Type II diabetes mellitus

164.1 Human Pathway: HSA04930

164.2 Number of Hits: 25

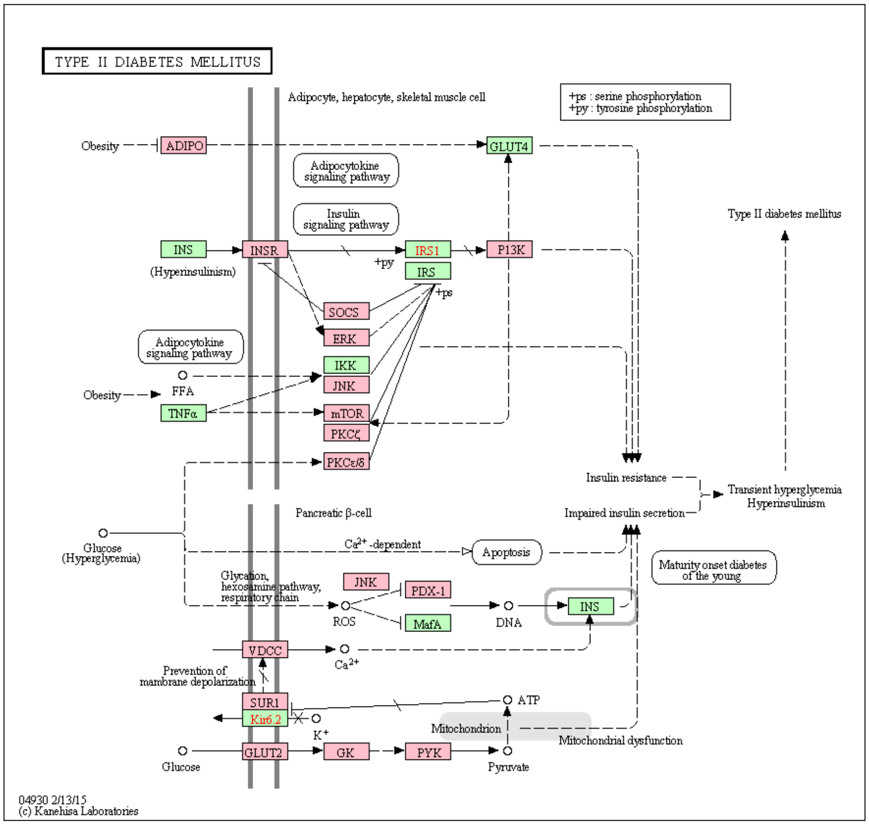

164.3 Legend:

RBH-Blast at 60% Identity + 50% Coverage
Green = Hit in *H. sapiens*
Red = Hit in *H. sapiens* and *C. milli*
White = Not in *H. sapiens*

165 Bile secretion

165.1 Human Pathway: HSA04976

165.2 Number of Hits: 24

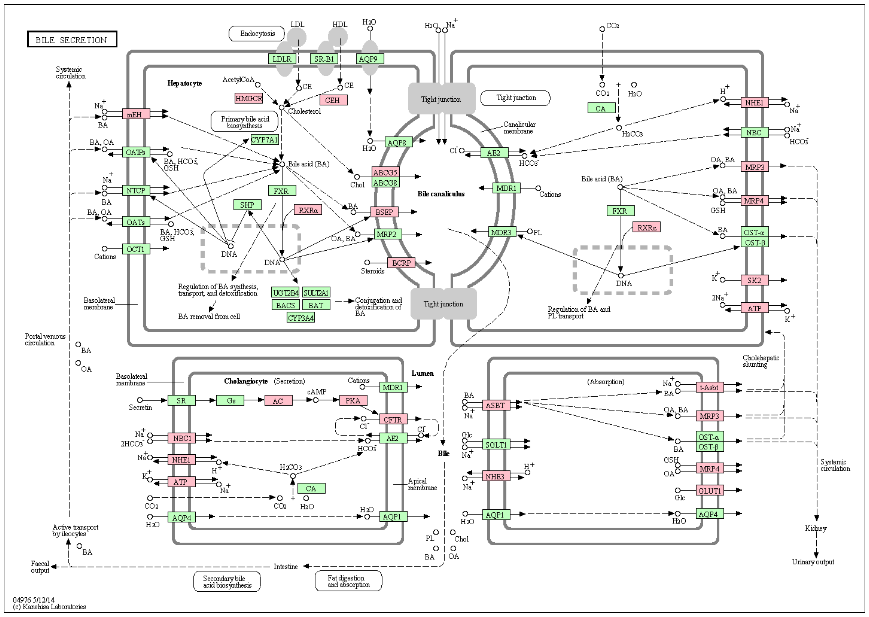

165.3 Legend:

RBH-Blast at 60% Identity + 50% Coverage
Green = Hit in *H. sapiens*
Red = Hit in *H. sapiens* and *C. milli*
White = Not in *H. sapiens*

166 Pyruvate metabolism

166.1 Human Pathway: HSA00620

166.2 Number of Hits: 24

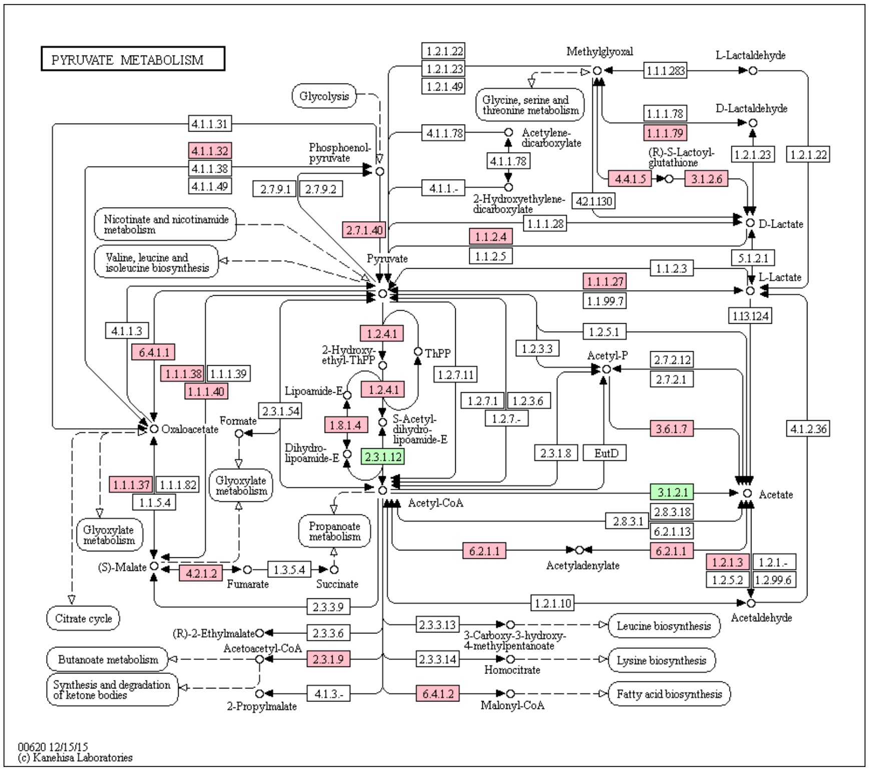

166.3 Legend:

RBH-Blast at 60% Identity + 50% Coverage
Green = Hit in *H. sapiens*
Red = Hit in *H. sapiens* and *C. milli*
White = Not in *H. sapiens*

167 Notch signaling pathway

167.1 Human Pathway: HSA04330

167.2 Number of Hits: 24

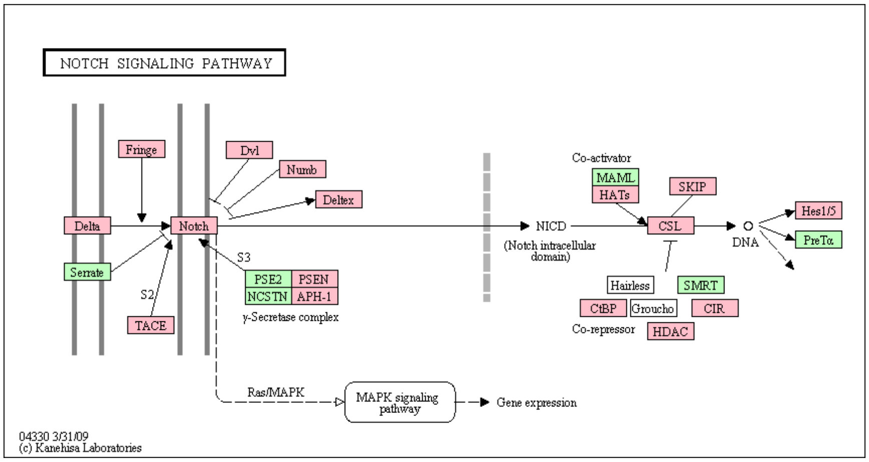

167.3 Legend:

RBH-Blast at 60% Identity + 50% Coverage
Green = Hit in *H. sapiens*
Red = Hit in *H. sapiens* and *C. milli*
White = Not in *H. sapiens*

168 Cocaine addiction

168.1 Human Pathway: HSA05030

168.2 Number of Hits: 23

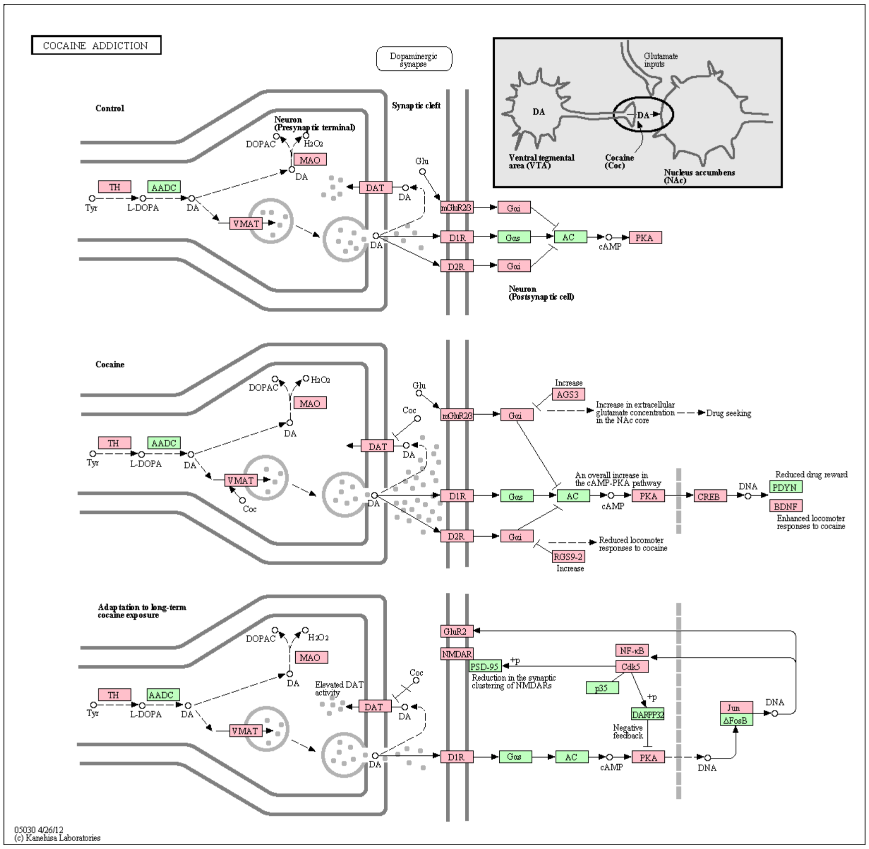

168.3 Legend:

RBH-Blast at 60% Identity + 50% Coverage
Green = Hit in *H. sapiens*
Red = Hit in *H. sapiens* and *C. milli*
White = Not in *H. sapiens*

169 Pathogenic Escherichia coli infection

169.1 Human Pathway: HSA05130

169.2 Number of Hits: 23

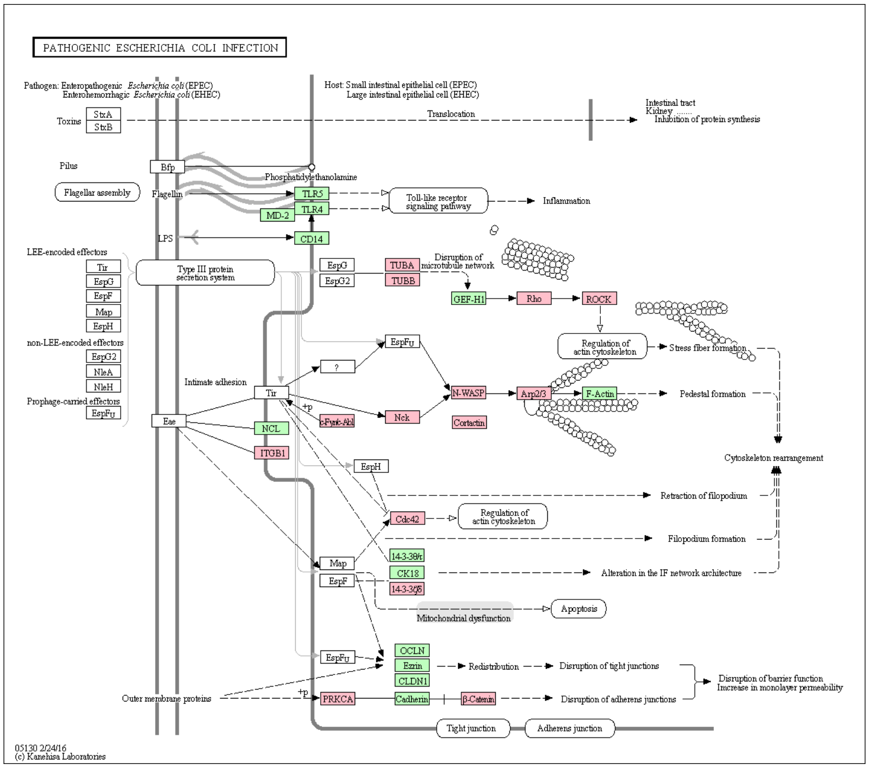

169.3 Legend:

RBH-Blast at 60% Identity + 50% Coverage
Green = Hit in *H. sapiens*
Red = Hit in *H. sapiens* and *C. milli*
White = Not in *H. sapiens*

170 Glycine, serine and threonine metabolism

170.1 Human Pathway: HSA00260

170.2 Number of Hits: 23

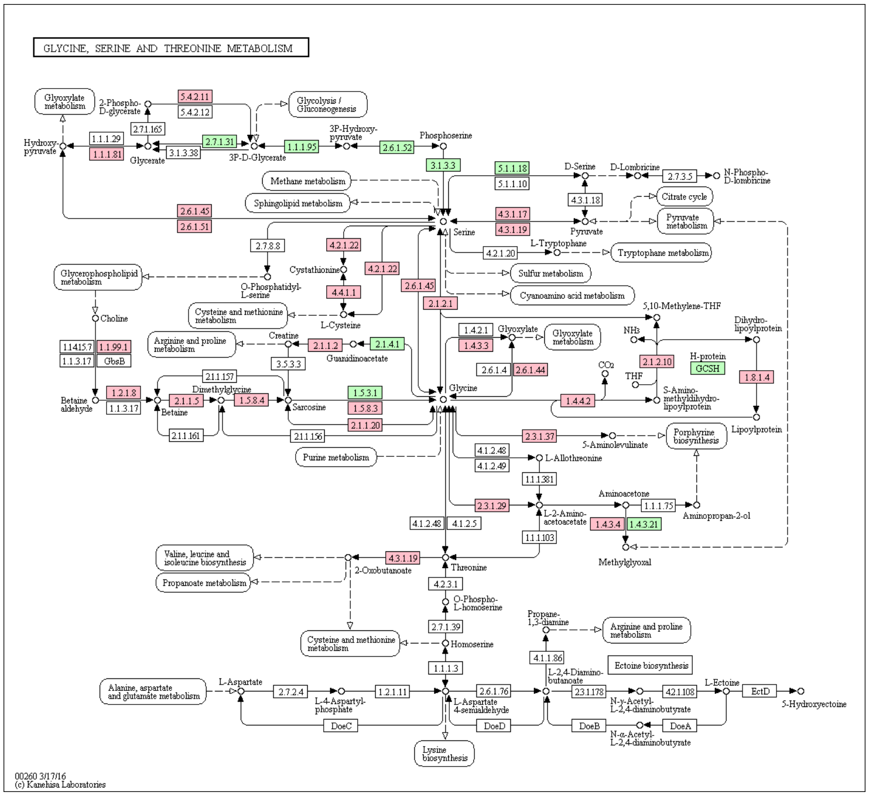

170.3 Legend:

RBH-Blast at 60% Identity + 50% Coverage
Green = Hit in *H. sapiens*
Red = Hit in *H. sapiens* and *C. milli*
White = Not in *H. sapiens*

171 Protein digestion and absorption

171.1 Human Pathway: HSA04974

171.2 Number of Hits: 22

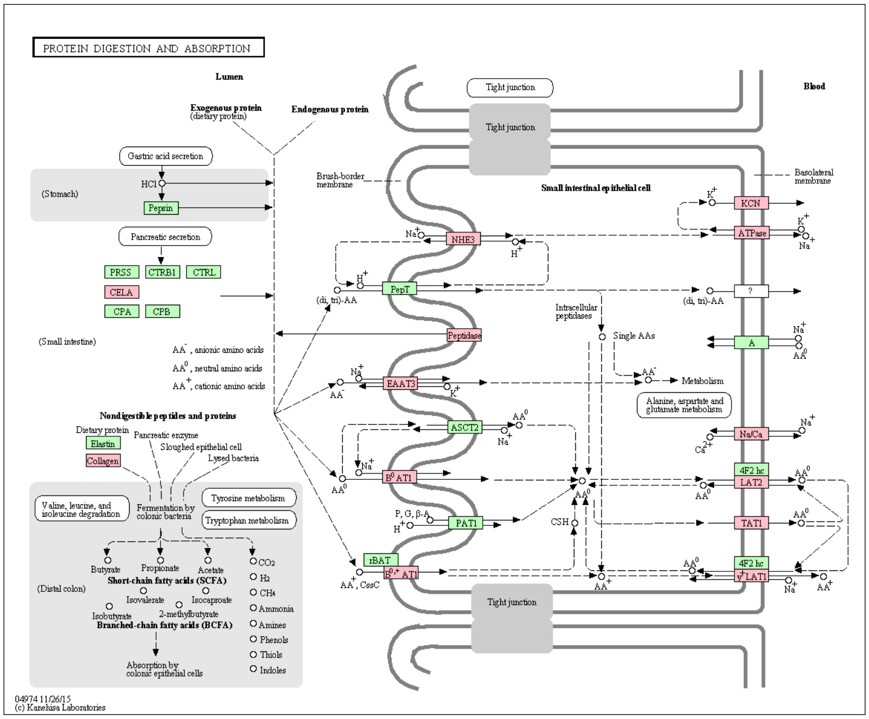

171.3 Legend:

RBH-Blast at 60% Identity + 50% Coverage
Green = Hit in *H. sapiens*
Red = Hit in *H. sapiens* and *C. milli*
White = Not in *H. sapiens*

172 NF-kappa B signaling pathway

172.1 Human Pathway: HSA04064

172.2 Number of Hits: 22

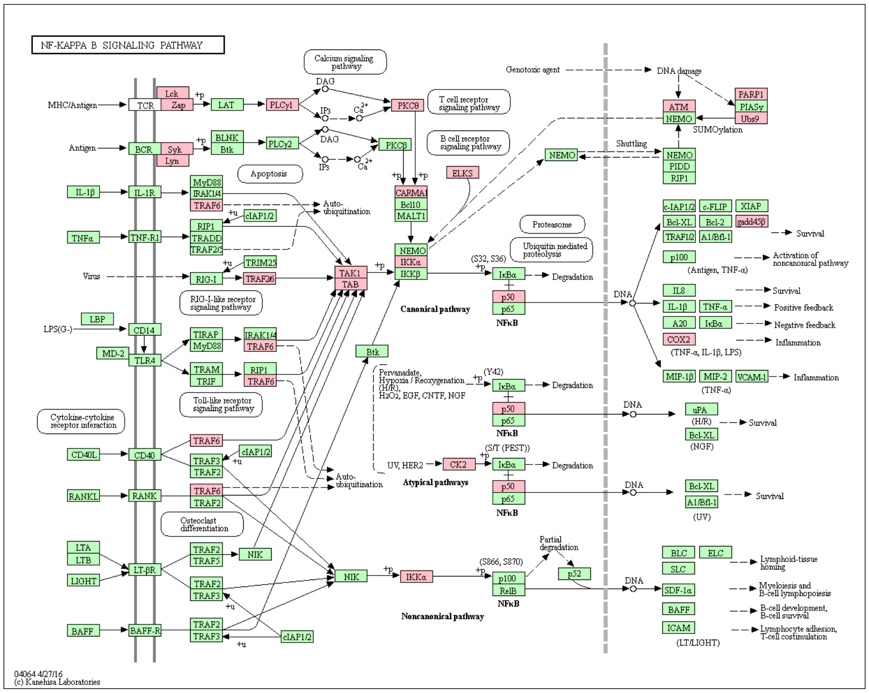

172.3 Legend:

RBH-Blast at 60% Identity + 50% Coverage
Green = Hit in *H. sapiens*
Red = Hit in *H. sapiens* and *C. milli*
White = Not in *H. sapiens*

173 Alanine, aspartate and glutamate metabolism

173.1 Human Pathway: HSA00250

173.2 Number of Hits: 20

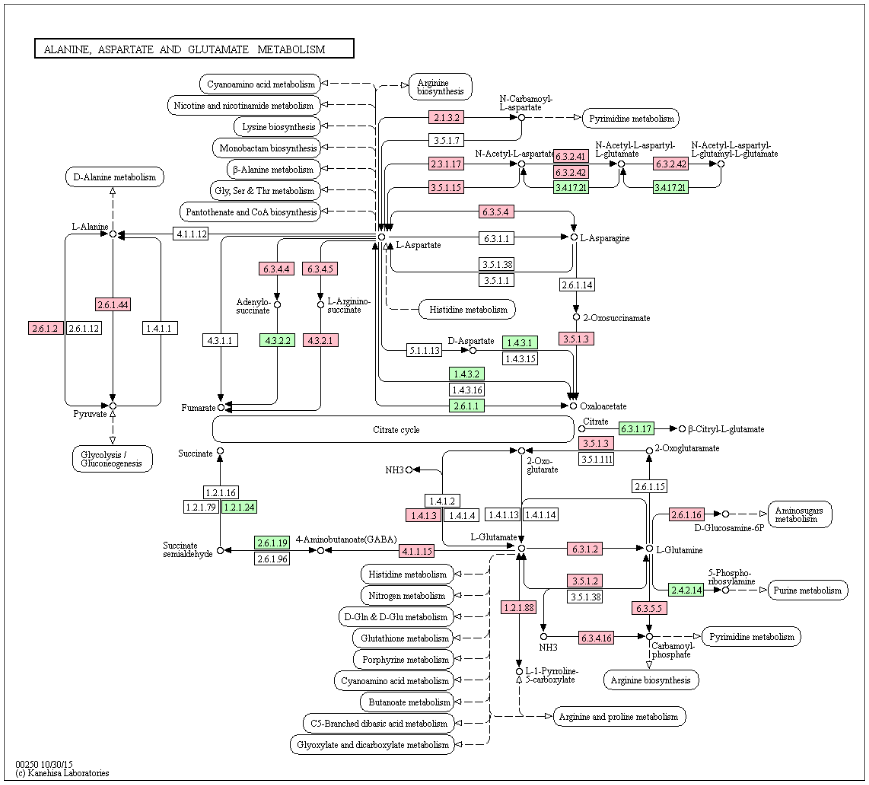

173.3 Legend:

RBH-Blast at 60% Identity + 50% Coverage
Green = Hit in *H. sapiens*
Red = Hit in *H. sapiens* and *C. milli*
White = Not in *H. sapiens*

174 Hedgehog signaling pathway

174.1 Human Pathway: HSA04340

174.2 Number of Hits: 20

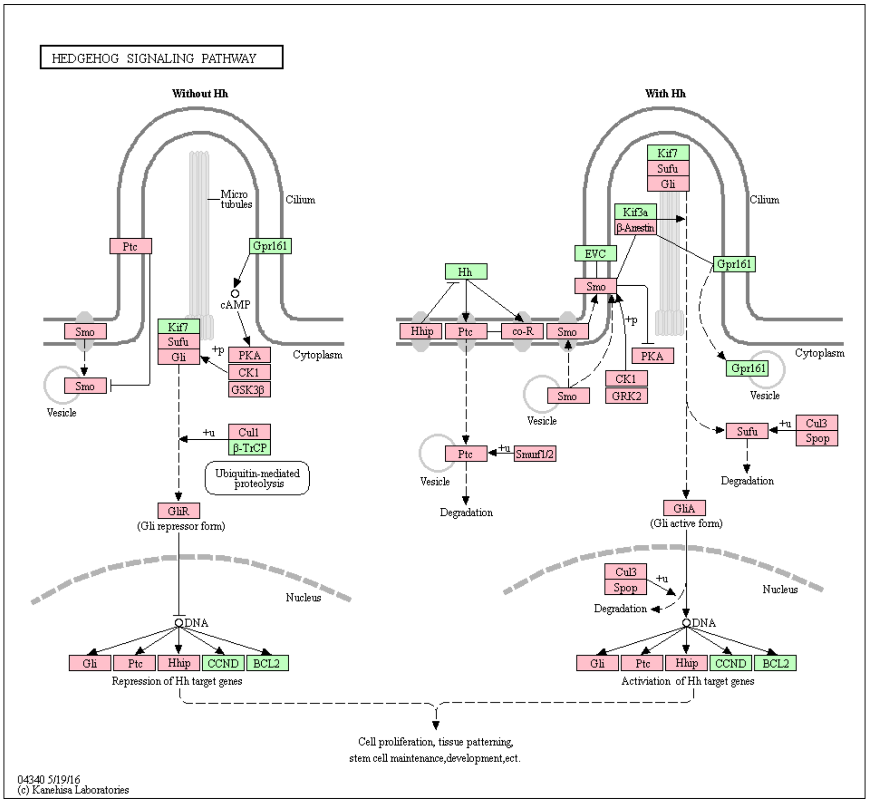

174.3 Legend:

RBH-Blast at 60% Identity + 50% Coverage
Green = Hit in *H. sapiens*
Red = Hit in *H. sapiens* and *C. milli*
White = Not in *H. sapiens*

175 Glyoxylate and dicarboxylate metabolism

175.1 Human Pathway: HSA00630

175.2 Number of Hits: 20

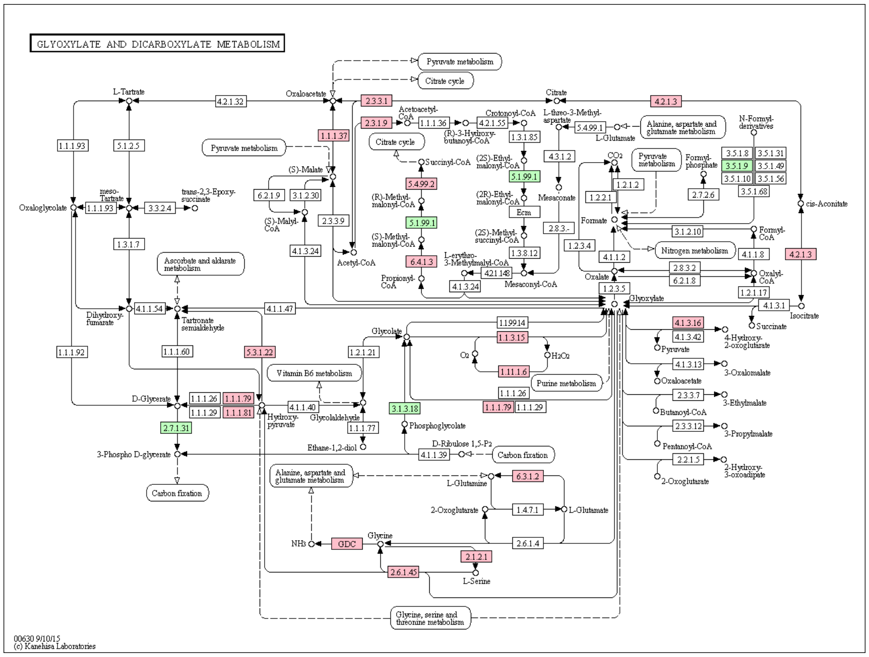

175.3 Legend:

RBH-Blast at 60% Identity + 50% Coverage
Green = Hit in *H. sapiens*
Red = Hit in *H. sapiens* and *C. milli*
White = Not in *H. sapiens*

176 Propanoate metabolism

176.1 Human Pathway: HSA00640

176.2 Number of Hits: 20

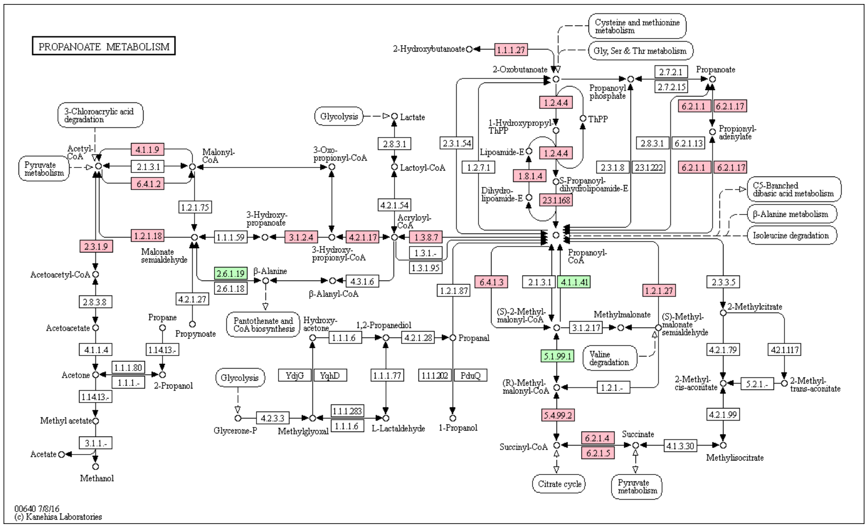

176.3 Legend:

RBH-Blast at 60% Identity + 50% Coverage
Green = Hit in *H. sapiens*
Red = Hit in *H. sapiens* and *C. milli*
White = Not in *H. sapiens*

177 SNARE interactions in vesicular transport

177.1 Human Pathway: HSA04130

177.2 Number of Hits: 20

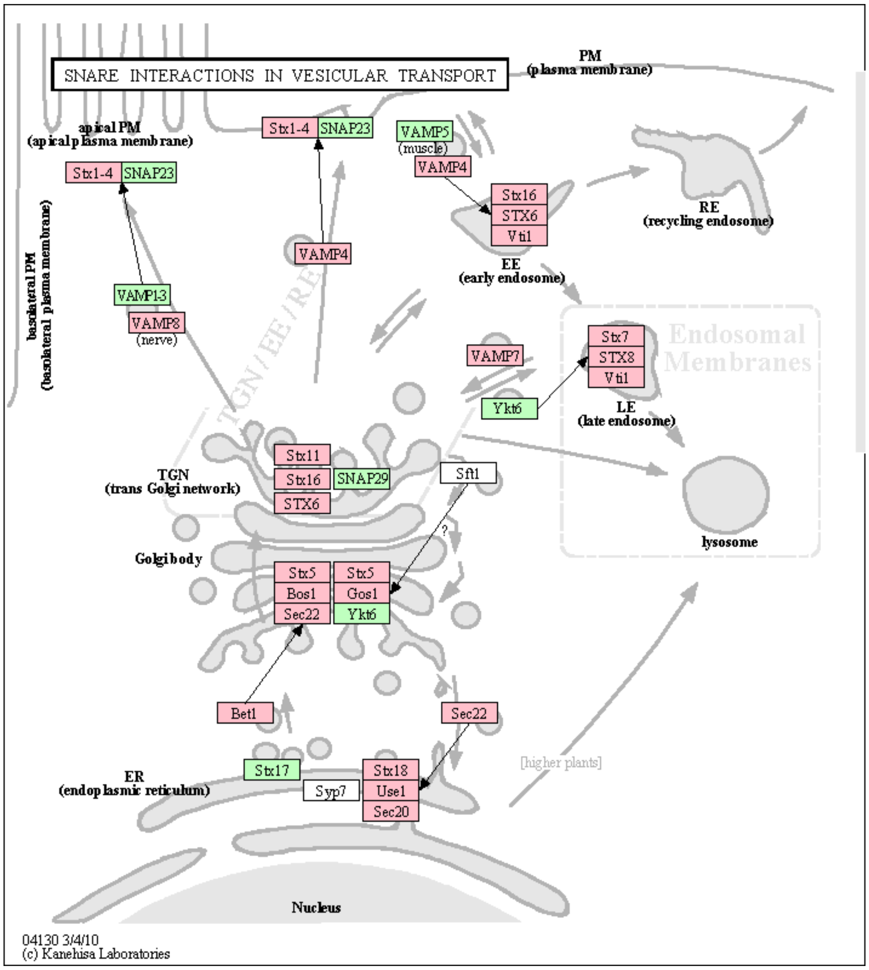

177.3 Legend:

RBH-Blast at 60% Identity + 50% Coverage
Green = Hit in *H. sapiens*
Red = Hit in *H. sapiens* and *C. milli*
White = Not in *H. sapiens*

178 Dorso-ventral axis formation

178.1 Human Pathway: HSA04320

178.2 Number of Hits: 20

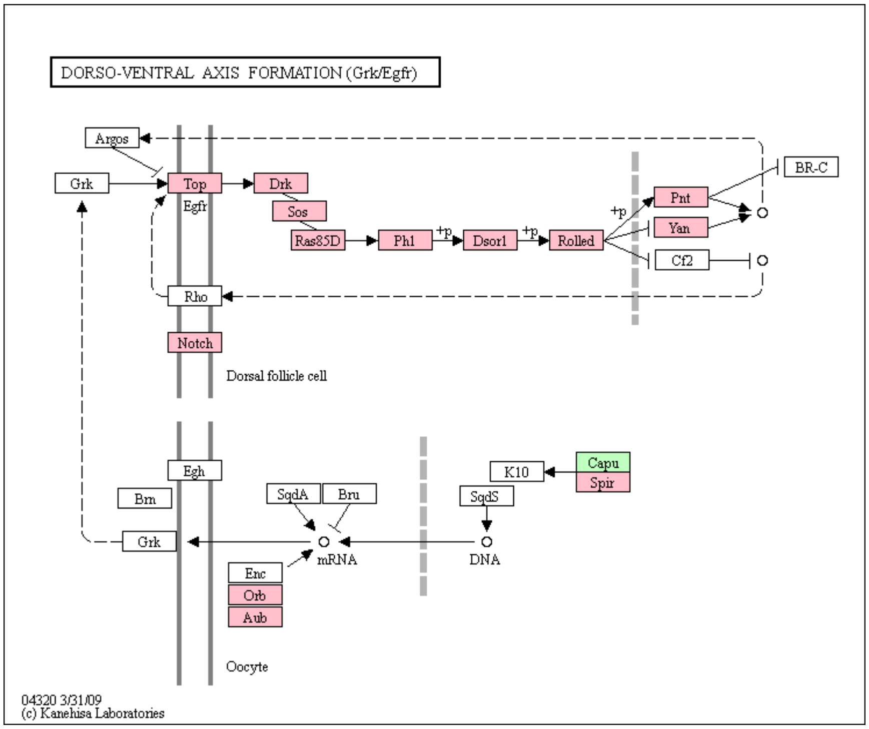

178.3 Legend:

RBH-Blast at 60% Identity + 50% Coverage
Green = Hit in *H. sapiens*
Red = Hit in *H. sapiens* and *C. milli*
White = Not in *H. sapiens*

179 beta-Alanine metabolism

179.1 Human Pathway: HSA00410

179.2 Number of Hits: 20

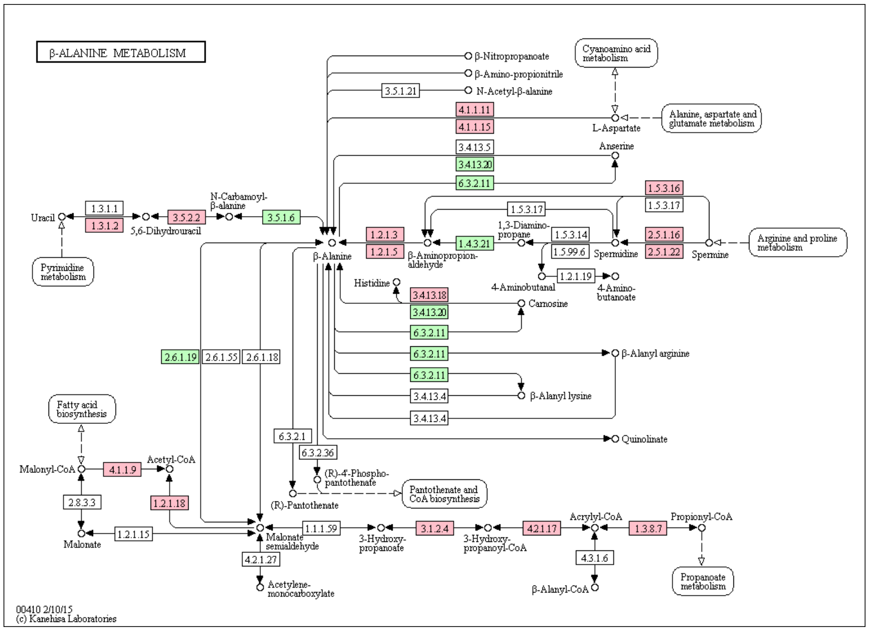

179.3 Legend:

RBH-Blast at 60% Identity + 50% Coverage
Green = Hit in *H. sapiens*
Red = Hit in *H. sapiens* and *C. milli*
White = Not in *H. sapiens*

180 Lysine degradation

180.1 Human Pathway: HSA00310

180.2 Number of Hits: 20

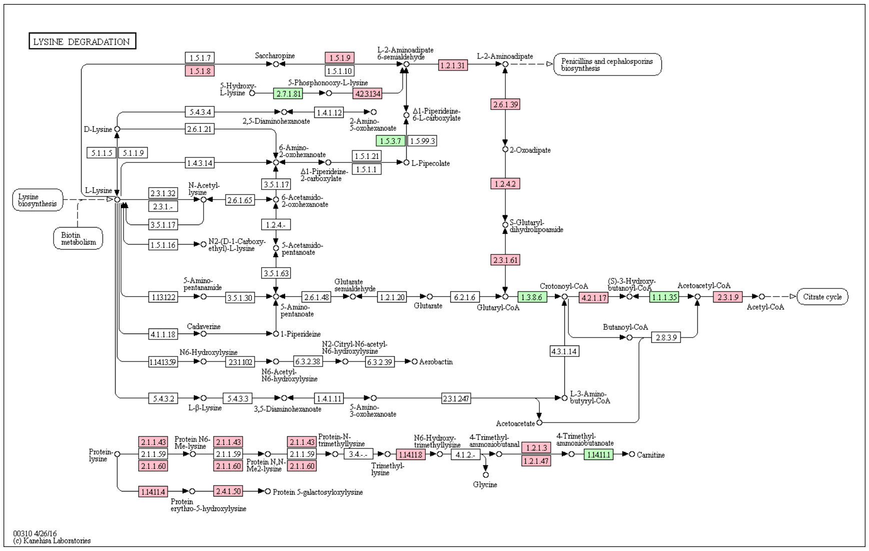

180.3 Legend:

RBH-Blast at 60% Identity + 50% Coverage
Green = Hit in *H. sapiens*
Red = Hit in *H. sapiens* and *C. milli*
White = Not in *H. sapiens*

181 Pertussis

181.1 Human Pathway: HSA05133

181.2 Number of Hits: 20

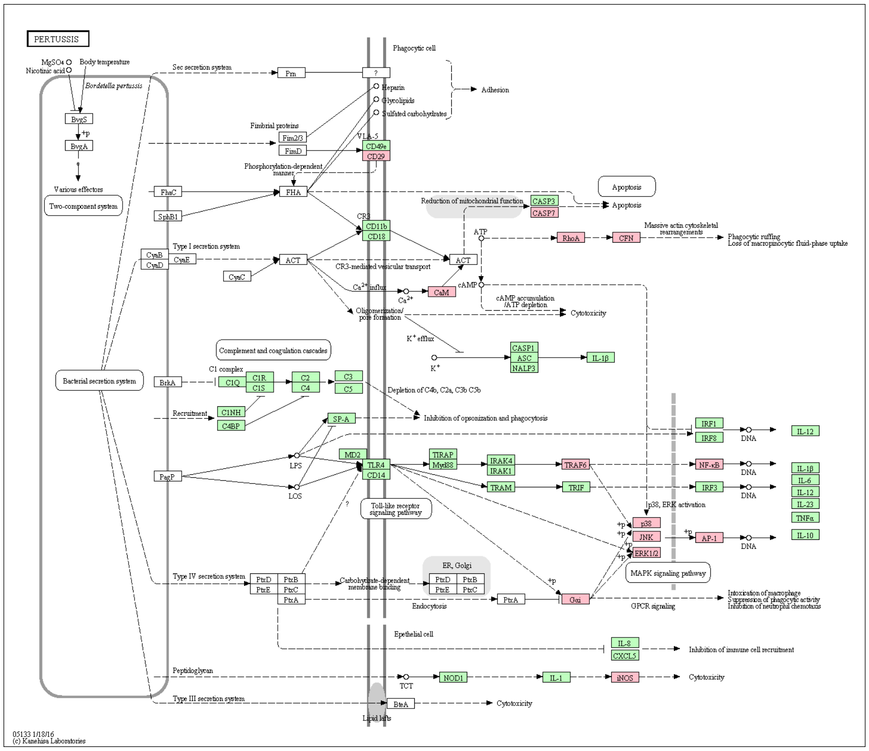

181.3 Legend:

RBH-Blast at 60% Identity + 50% Coverage
Green = Hit in *H. sapiens*
Red = Hit in *H. sapiens* and *C. milli*
White = Not in *H. sapiens*

182 Platinum drug resistance

182.1 Human Pathway: HSA01524

182.2 Number of Hits: 20

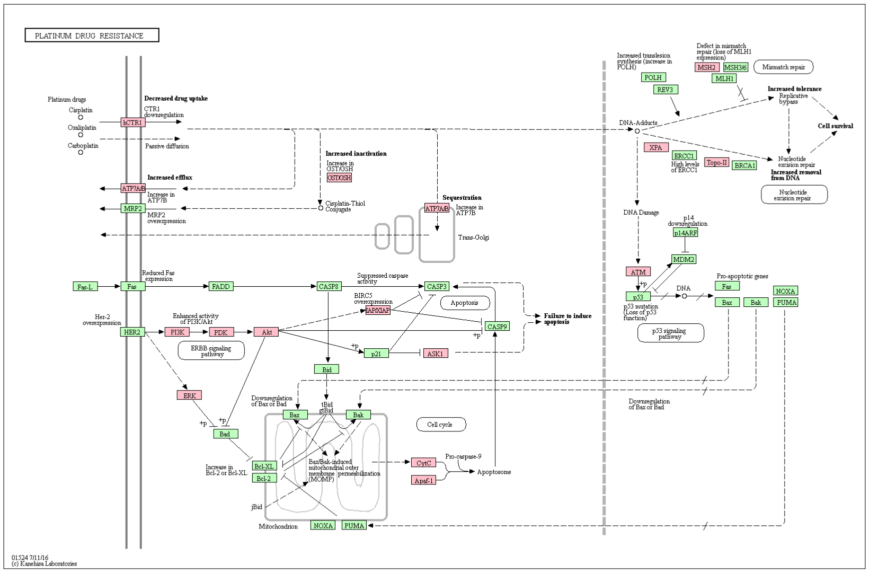

182.3 Legend:

RBH-Blast at 60% Identity + 50% Coverage
Green = Hit in *H. sapiens*
Red = Hit in *H. sapiens* and *C. milli*
White = Not in *H. sapiens*

183 Fatty acid degradation

183.1 Human Pathway: HSA00071

183.2 Number of Hits: 19

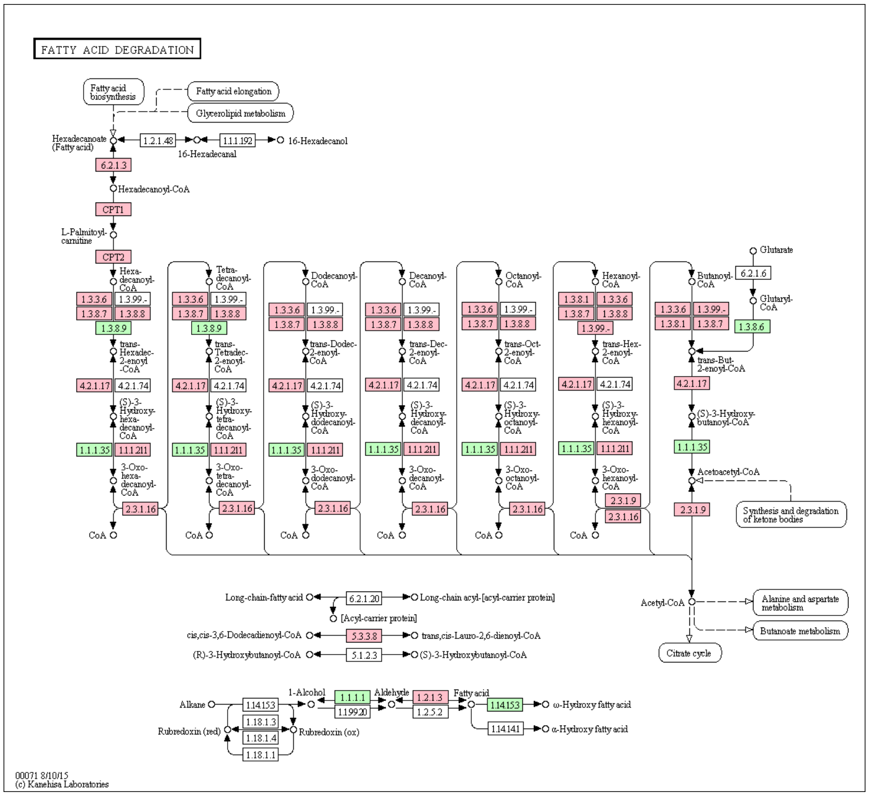

183.3 Legend:

RBH-Blast at 60% Identity + 50% Coverage
Green = Hit in *H. sapiens*
Red = Hit in *H. sapiens* and *C. milli*
White = Not in *H. sapiens*

184 DNA replication

184.1 Human Pathway: HSA03030

184.2 Number of Hits: 19

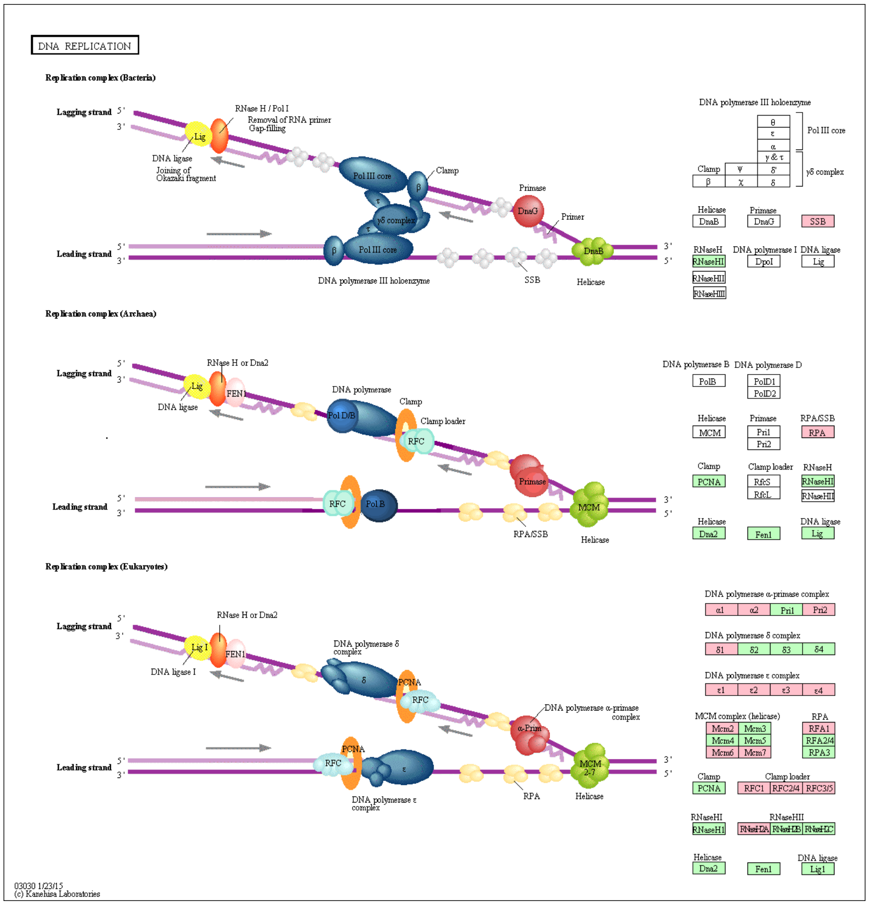

184.3 Legend:

RBH-Blast at 60% Identity + 50% Coverage
Green = Hit in *H. sapiens*
Red = Hit in *H. sapiens* and *C. milli*
White = Not in *H. sapiens*

185 Citrate cycle (TCA cycle)

185.1 Human Pathway: HSA00020

185.2 Number of Hits: 19

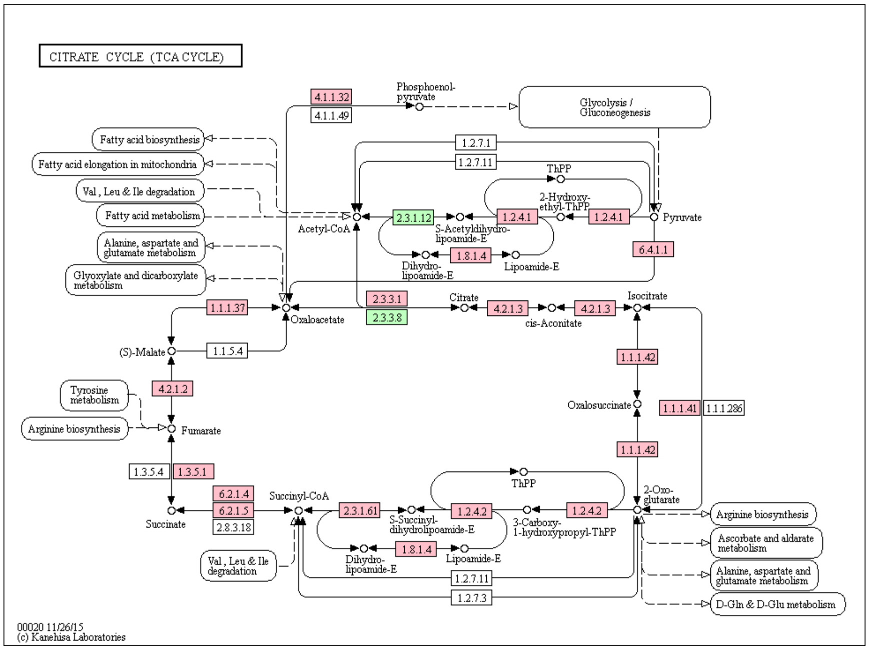

185.3 Legend:

RBH-Blast at 60% Identity + 50% Coverage
Green = Hit in *H. sapiens*
Red = Hit in *H. sapiens* and *C. milli*
White = Not in *H. sapiens*

186 Vasopressin-regulated water reabsorption

186.1 Human Pathway: HSA04962

186.2 Number of Hits: 19

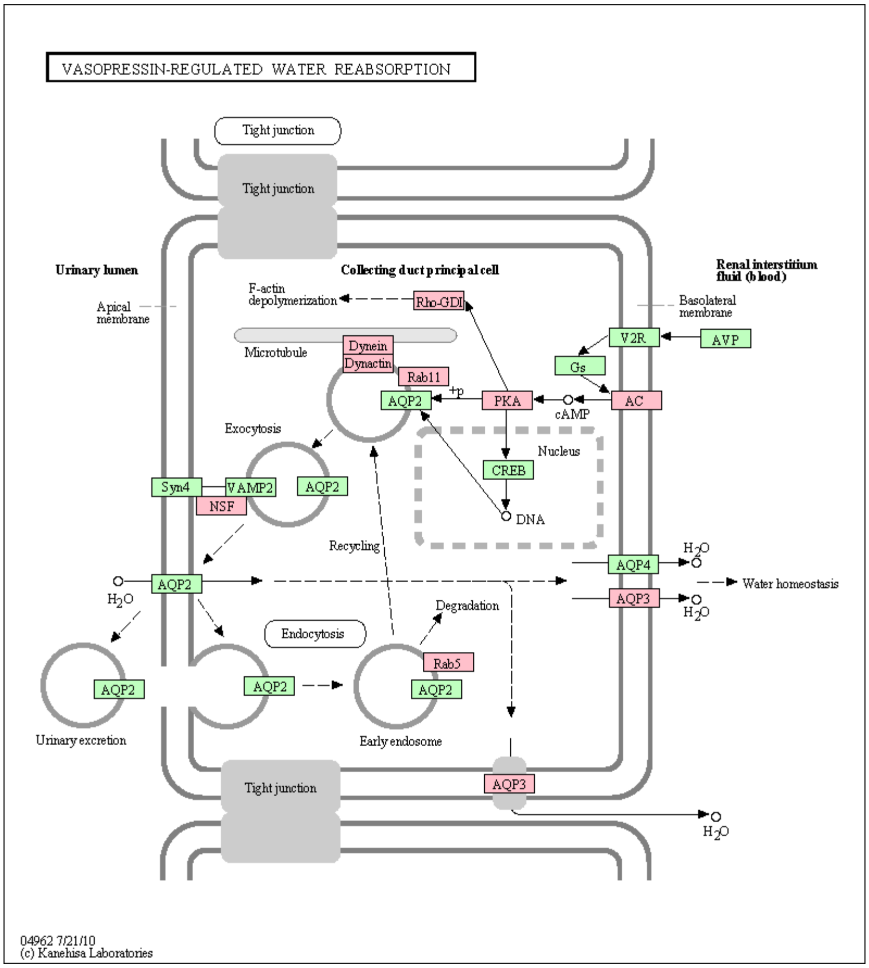

186.3 Legend:

RBH-Blast at 60% Identity + 50% Coverage
Green = Hit in *H. sapiens*
Red = Hit in *H. sapiens* and *C. milli*
White = Not in *H. sapiens*

187 NOD-like receptor signaling pathway

187.1 Human Pathway: HSA04621

187.2 Number of Hits: 19

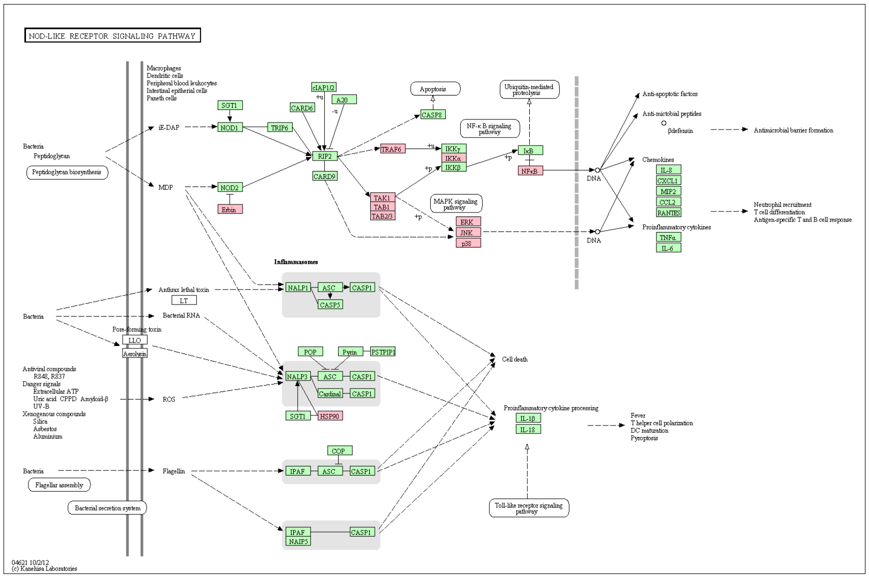

187.3 Legend:

RBH-Blast at 60% Identity + 50% Coverage
Green = Hit in *H. sapiens*
Red = Hit in *H. sapiens* and *C. milli*
White = Not in *H. sapiens*

188 Rheumatoid arthritis

188.1 Human Pathway: HSA05323

188.2 Number of Hits: 19

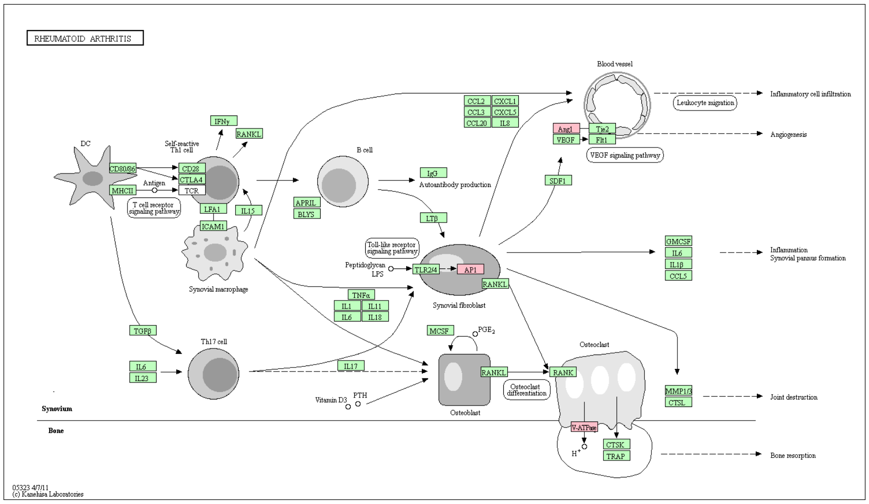

188.3 Legend:

RBH-Blast at 60% Identity + 50% Coverage
Green = Hit in *H. sapiens*
Red = Hit in *H. sapiens* and *C. milli*
White = Not in *H. sapiens*

189 Mucin type O-Glycan biosynthesis

189.1 Human Pathway: HSA00512

189.2 Number of Hits: 19

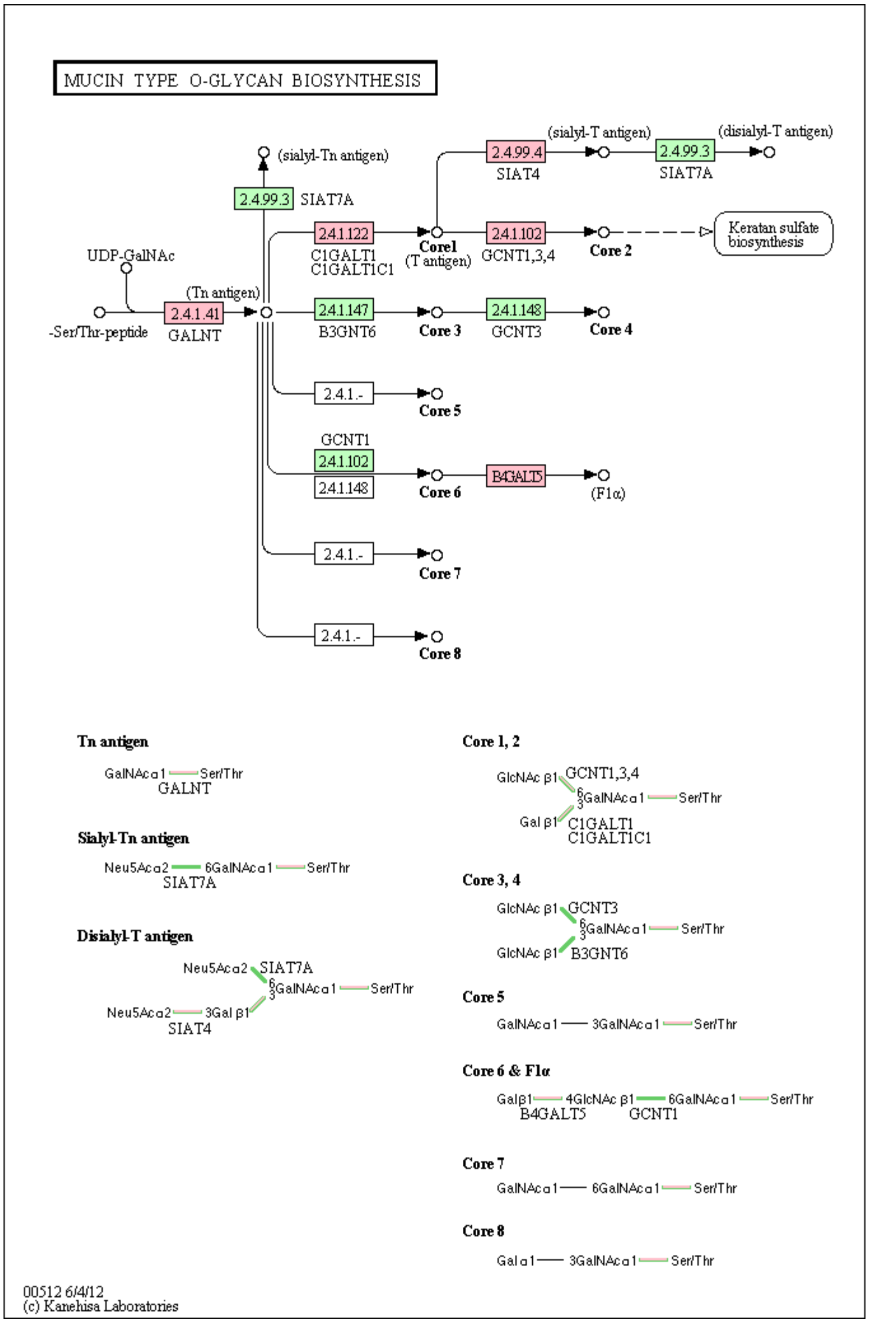

189.3 Legend:

RBH-Blast at 60% Identity + 50% Coverage
Green = Hit in *H. sapiens*
Red = Hit in *H. sapiens* and *C. milli*
White = Not in *H. sapiens*

190 Protein export

190.1 Human Pathway: HSA03060

190.2 Number of Hits: 18

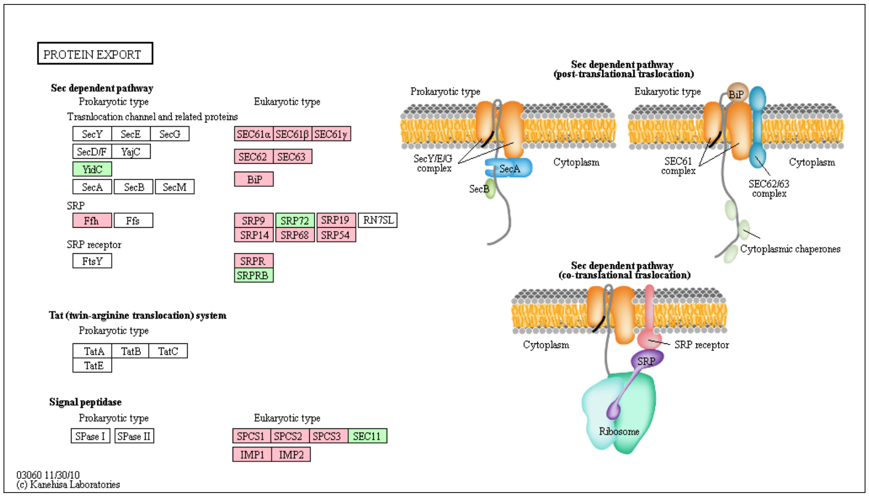

190.3 Legend:

RBH-Blast at 60% Identity + 50% Coverage
Green = Hit in *H. sapiens*
Red = Hit in *H. sapiens* and *C. milli*
White = Not in *H. sapiens*

191 Other types of O-glycan biosynthesis

191.1 Human Pathway: HSA00514

191.2 Number of Hits: 18

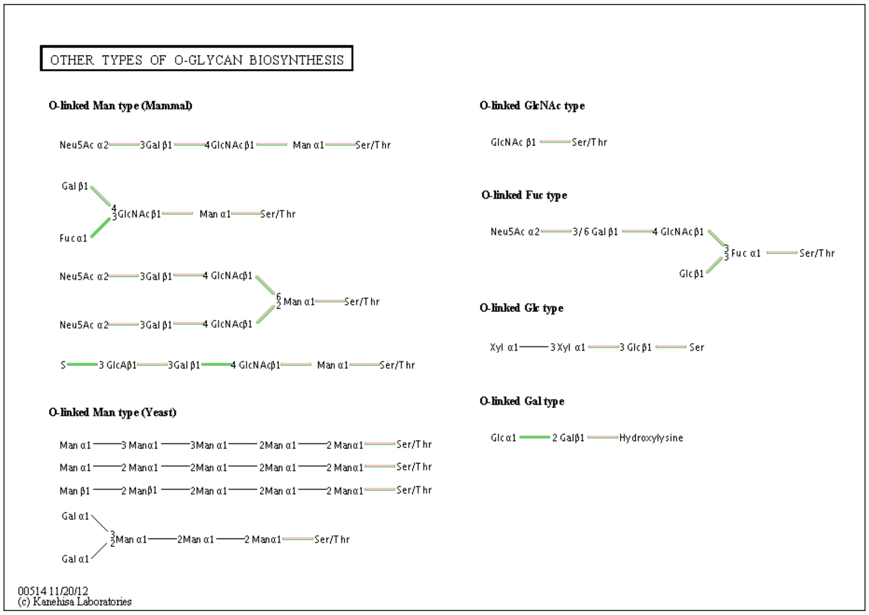

191.3 Legend:

RBH-Blast at 60% Identity + 50% Coverage
Green = Hit in *H. sapiens*
Red = Hit in *H. sapiens* and *C. milli*
White = Not in *H. sapiens*

192 Endocrine and other factor-regulated calcium reabsorption

192.1 Human Pathway: HSA04961

192.2 Number of Hits: 18

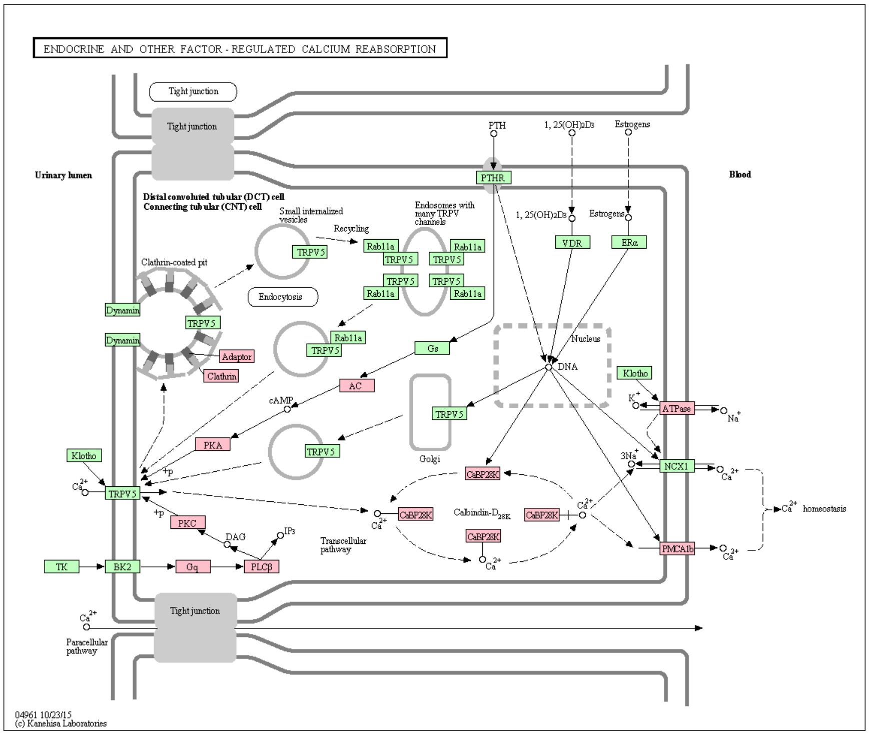

192.3 Legend:

RBH-Blast at 60% Identity + 50% Coverage
Green = Hit in *H. sapiens*
Red = Hit in *H. sapiens* and *C. milli*
White = Not in *H. sapiens*

193 Bladder cancer

193.1 Human Pathway: HSA05219

193.2 Number of Hits: 18

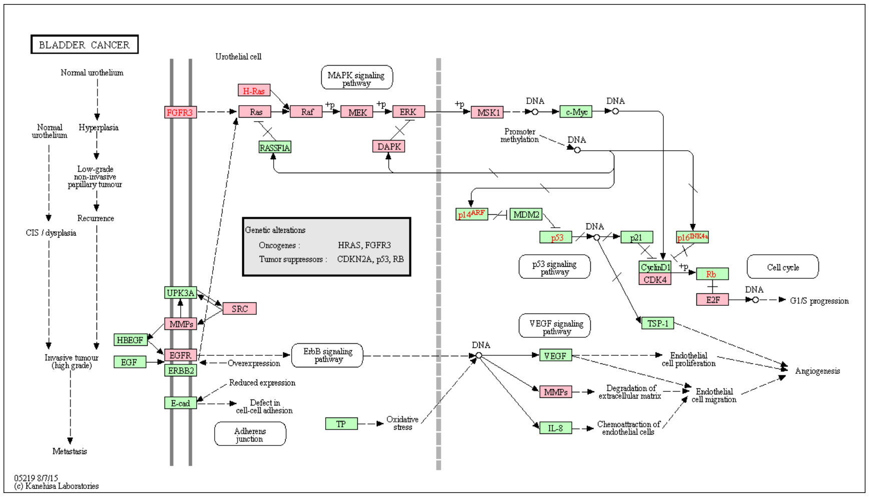

193.3 Legend:

RBH-Blast at 60% Identity + 50% Coverage
Green = Hit in *H. sapiens*
Red = Hit in *H. sapiens* and *C. milli*
White = Not in *H. sapiens*

194 Leishmaniasis

194.1 Human Pathway: HSA05140

194.2 Number of Hits: 18

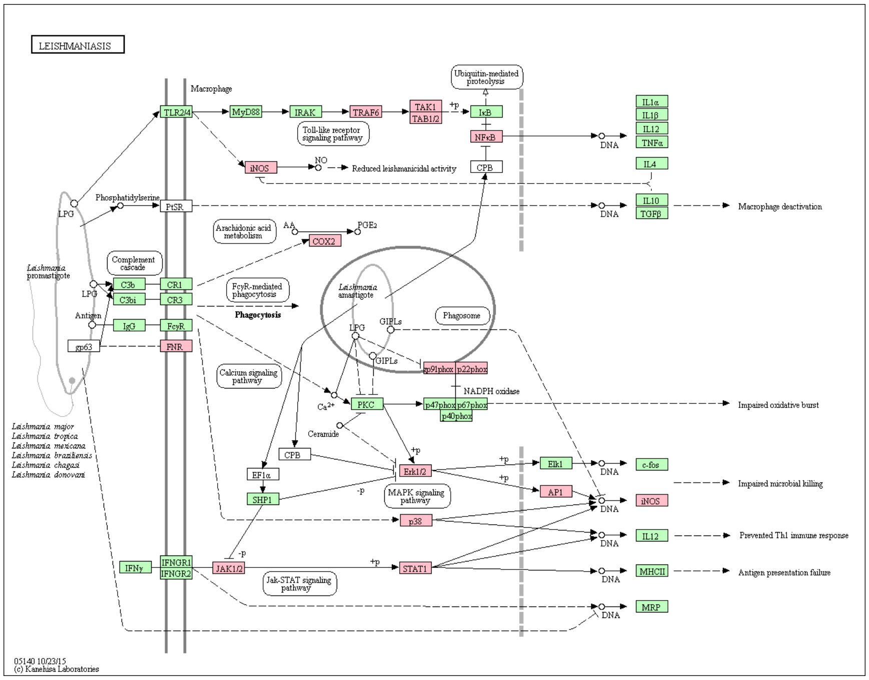

194.3 Legend:

RBH-Blast at 60% Identity + 50% Coverage
Green = Hit in *H. sapiens*
Red = Hit in *H. sapiens* and *C. milli*
White = Not in *H. sapiens*

195 Tryptophan metabolism

195.1 Human Pathway: HSA00380

195.2 Number of Hits: 18

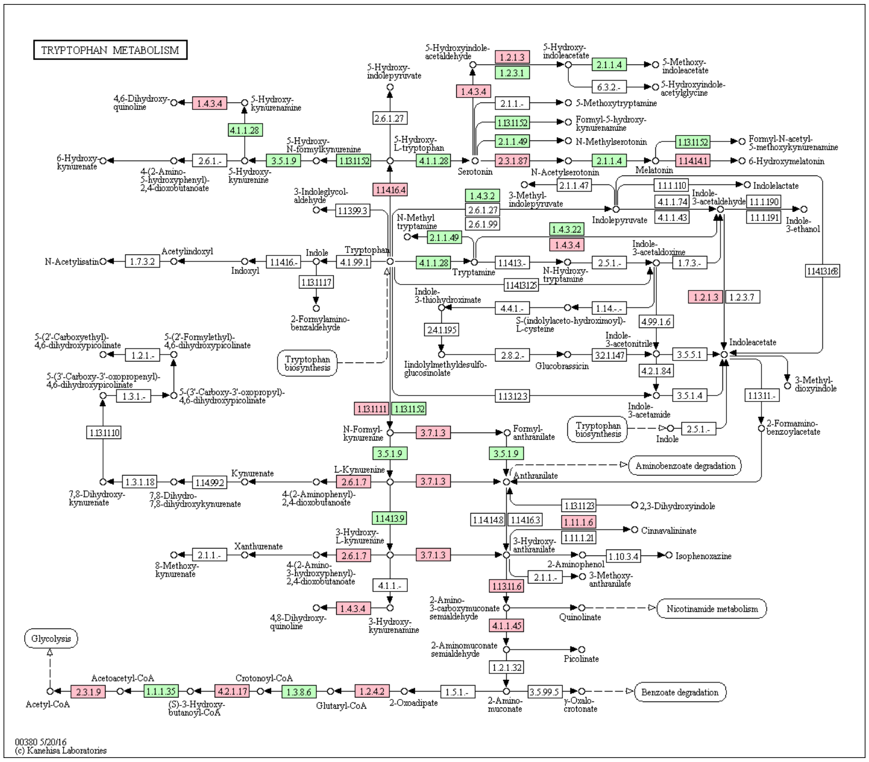

195.3 Legend:

RBH-Blast at 60% Identity + 50% Coverage
Green = Hit in *H. sapiens*
Red = Hit in *H. sapiens* and *C. milli*
White = Not in *H. sapiens*

196 RNA polymerase

196.1 Human Pathway: HSA03020

196.2 Number of Hits: 18

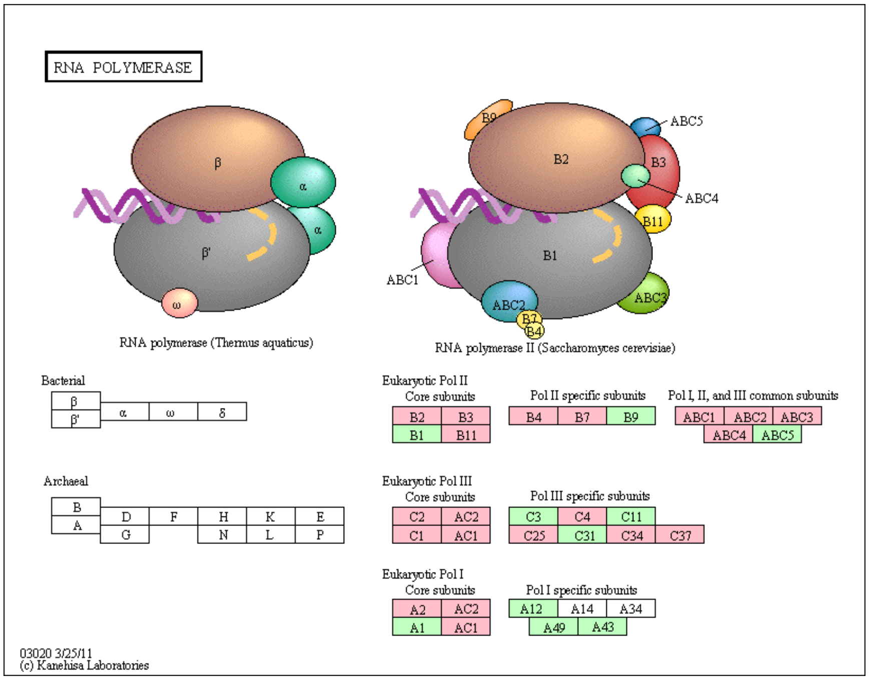

196.3 Legend:

RBH-Blast at 60% Identity + 50% Coverage
Green = Hit in *H. sapiens*
Red = Hit in *H. sapiens* and *C. milli*
White = Not in *H. sapiens*

197 Steroid biosynthesis

197.1 Human Pathway: HSA00100

197.2 Number of Hits: 17

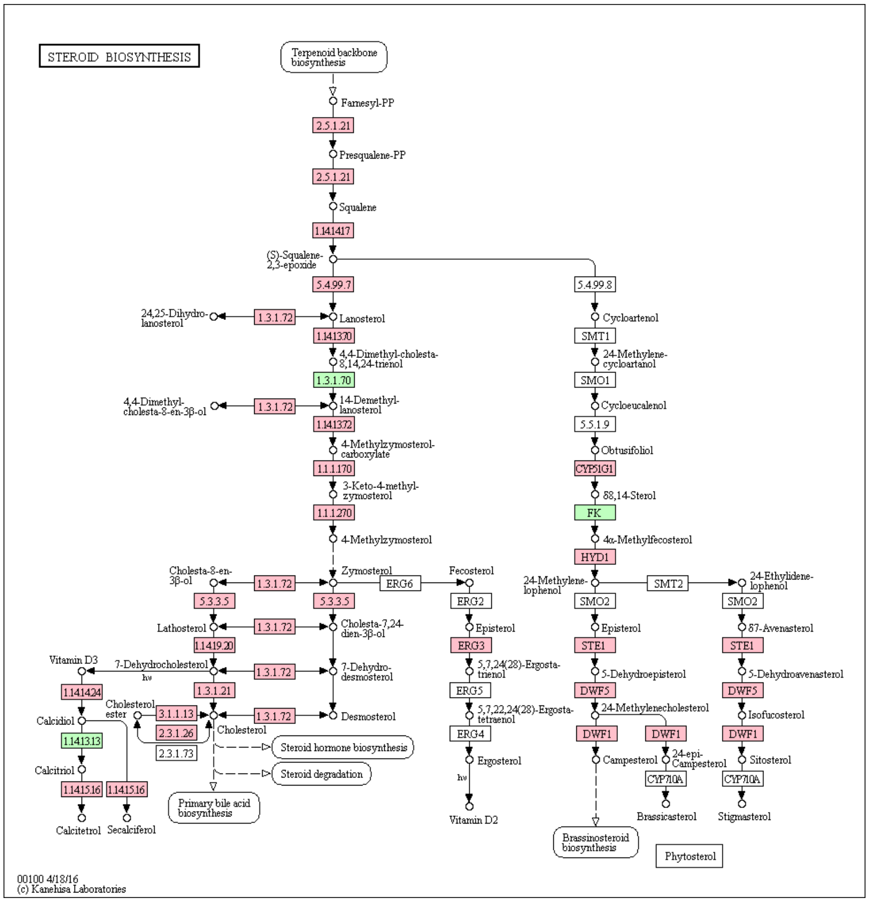

197.3 Legend:

RBH-Blast at 60% Identity + 50% Coverage
Green = Hit in *H. sapiens*
Red = Hit in *H. sapiens* and *C. milli*
White = Not in *H. sapiens*

198 ABC transporters

198.1 Human Pathway: HSA02010

198.2 Number of Hits: 17

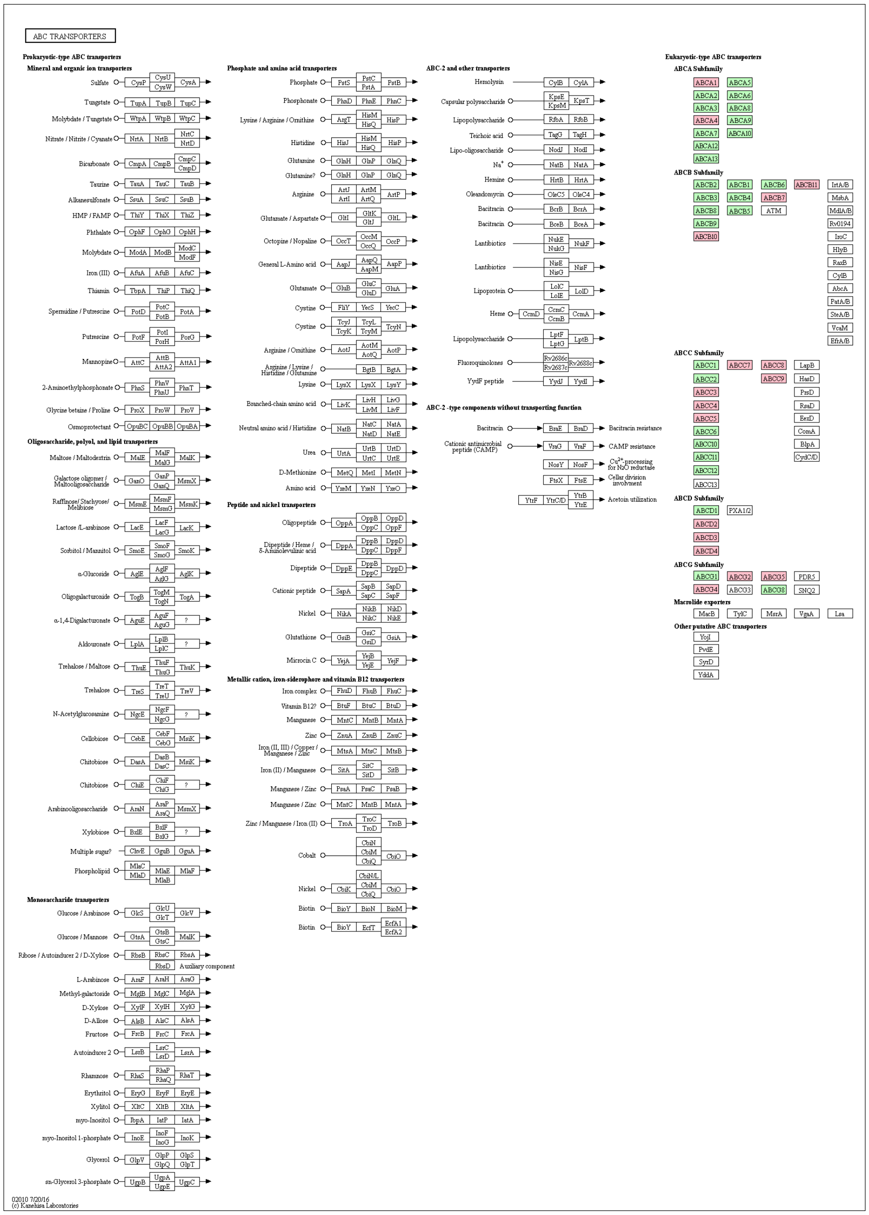

198.3 Legend:

RBH-Blast at 60% Identity + 50% Coverage
Green = Hit in *H. sapiens*
Red = Hit in *H. sapiens* and *C. milli*
White = Not in *H. sapiens*

199 Olfactory transduction

199.1 Human Pathway: HSA04740

199.2 Number of Hits: 17

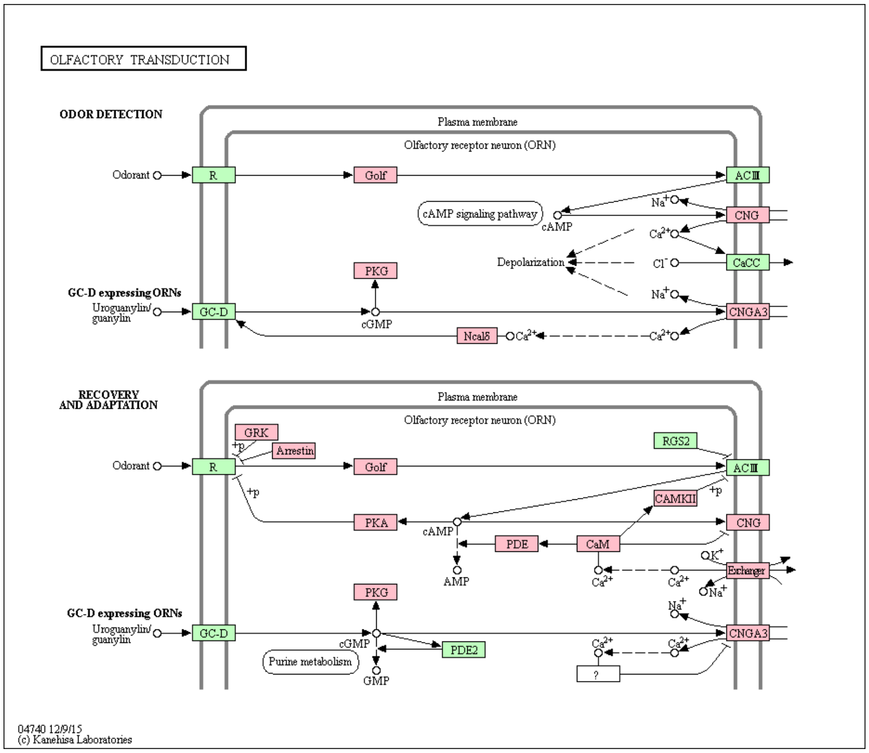

199.3 Legend:

RBH-Blast at 60% Identity + 50% Coverage
Green = Hit in *H. sapiens*
Red = Hit in *H. sapiens* and *C. milli*
White = Not in *H. sapiens*

200 RIG-I-like receptor signaling pathway

200.1 Human Pathway: HSA04622

200.2 Number of Hits: 17

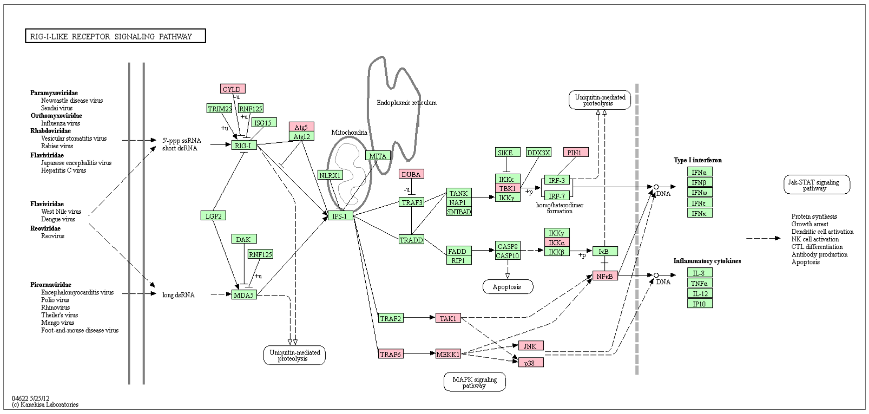

200.3 Legend:

RBH-Blast at 60% Identity + 50% Coverage
Green = Hit in *H. sapiens*
Red = Hit in *H. sapiens* and *C. milli*
White = Not in *H. sapiens*

201 Fatty acid elongation

201.1 Human Pathway: HSA00062

201.2 Number of Hits: 17

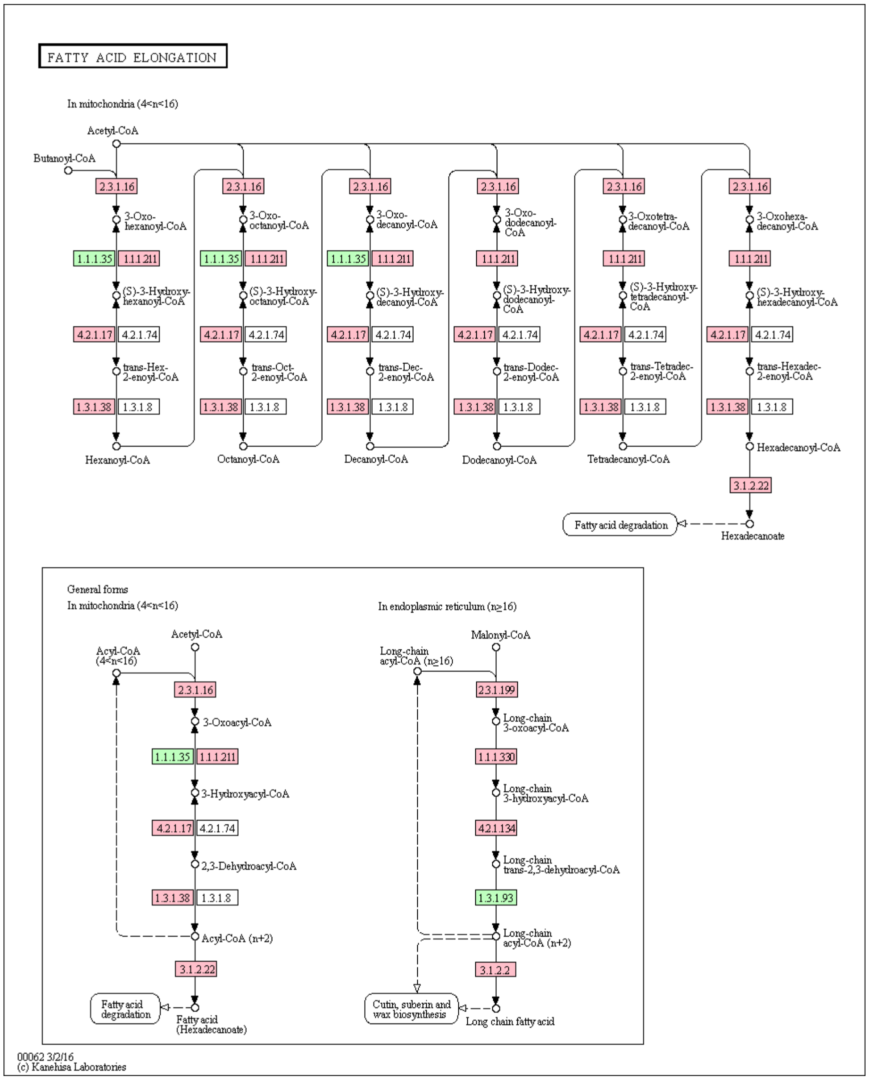

201.3 Legend:

RBH-Blast at 60% Identity + 50% Coverage
Green = Hit in *H. sapiens*
Red = Hit in *H. sapiens* and *C. milli*
White = Not in *H. sapiens*

202 Glutathione metabolism

202.1 Human Pathway: HSA00480

202.2 Number of Hits: 17

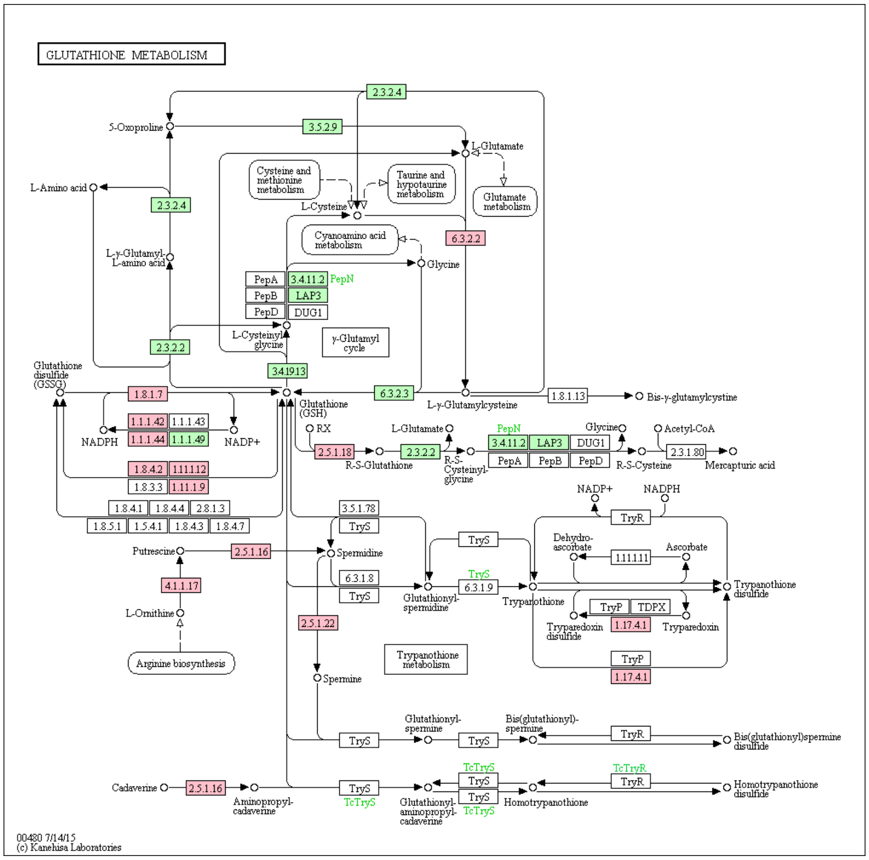

202.3 Legend:

RBH-Blast at 60% Identity + 50% Coverage
Green = Hit in *H. sapiens*
Red = Hit in *H. sapiens* and *C. milli*
White = Not in *H. sapiens*

---

203 Aldosterone-regulated sodium reabsorption

203.1 Human Pathway: HSA04960

203.2 Number of Hits: 16

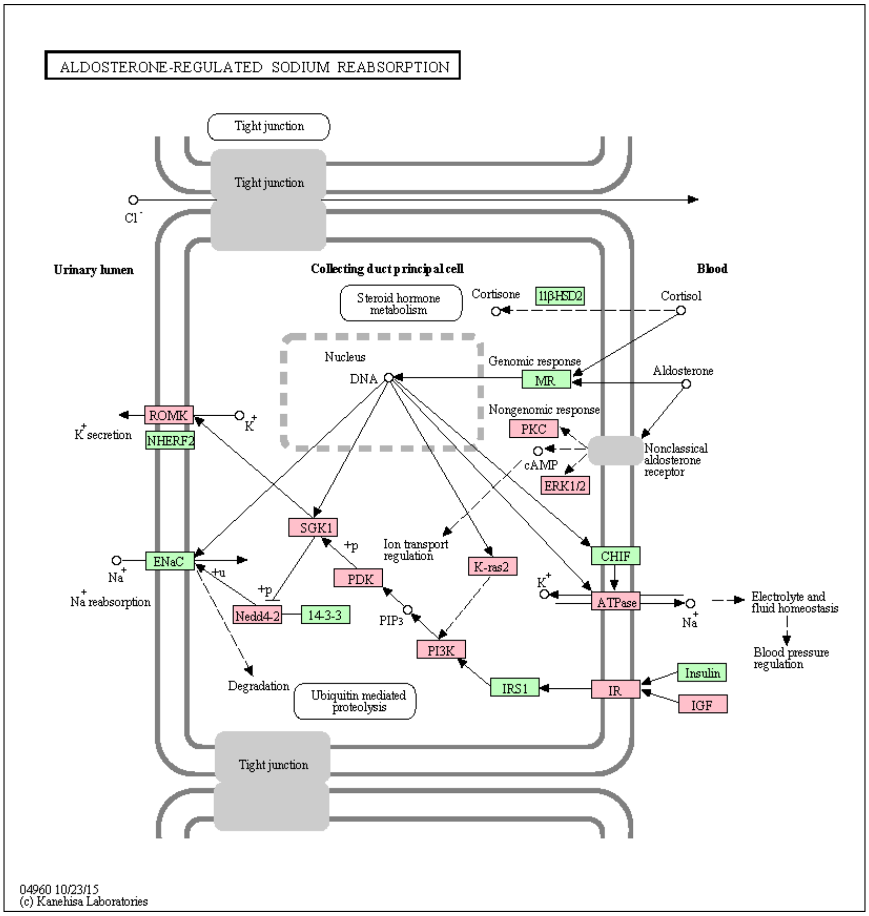

203.3 Legend:

RBH-Blast at 60% Identity + 50% Coverage
Green = Hit in *H. sapiens*
Red = Hit in *H. sapiens* and *C. milli*
White = Not in *H. sapiens*

---

204 Systemic lupus erythematosus

204.1 Human Pathway: HSA05322

204.2 Number of Hits: 16

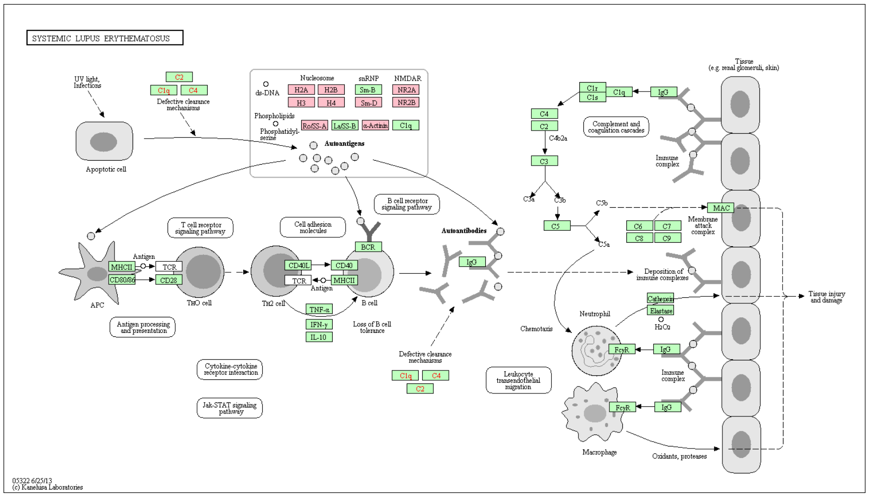

204.3 Legend:

RBH-Blast at 60% Identity + 50% Coverage
Green = Hit in *H. sapiens*
Red = Hit in *H. sapiens* and *C. milli*
White = Not in *H. sapiens*

---

205 Carbohydrate digestion and absorption

205.1 Human Pathway: HSA04973

205.2 Number of Hits: 16

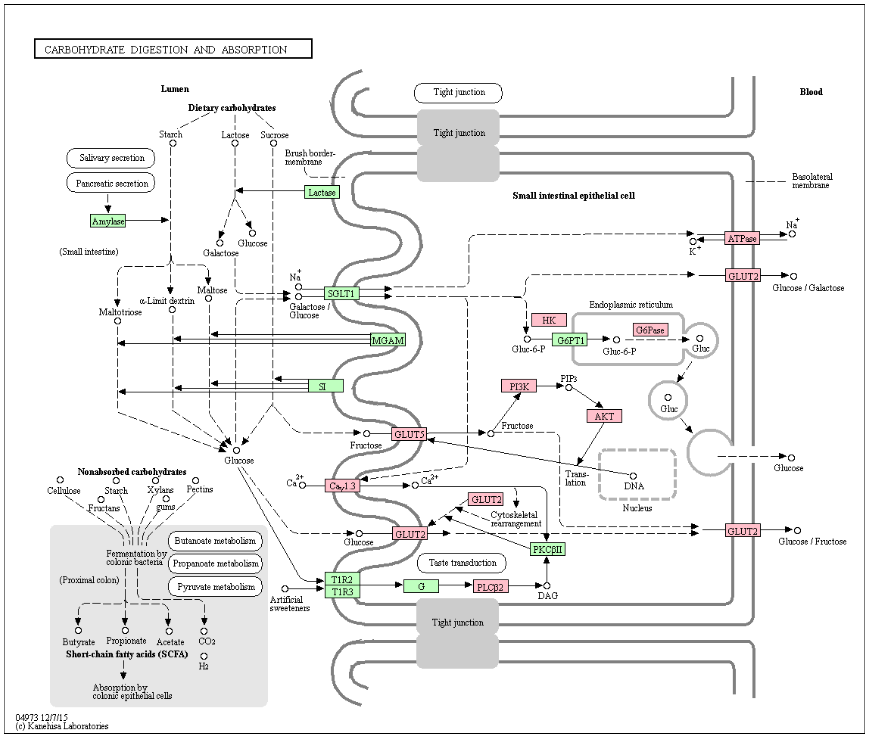

205.3 Legend:

RBH-Blast at 60% Identity + 50% Coverage
Green = Hit in *H. sapiens*
Red = Hit in *H. sapiens* and *C. milli*
White = Not in *H. sapiens*

206 Fructose and mannose metabolism

206.1 Human Pathway: HSA00051

206.2 Number of Hits: 16

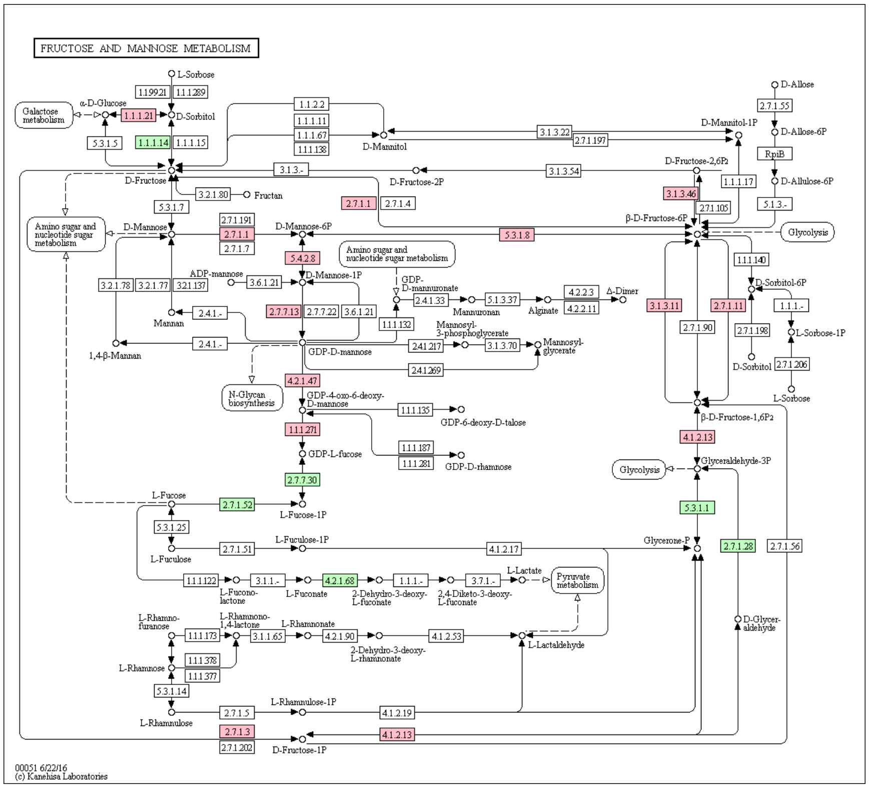

206.3 Legend:

RBH-Blast at 60% Identity + 50% Coverage
Green = Hit in *H. sapiens*
Red = Hit in *H. sapiens* and *C. milli*
White = Not in *H. sapiens*

207 ECM-receptor interaction

207.1 Human Pathway: HSA04512

207.2 Number of Hits: 16

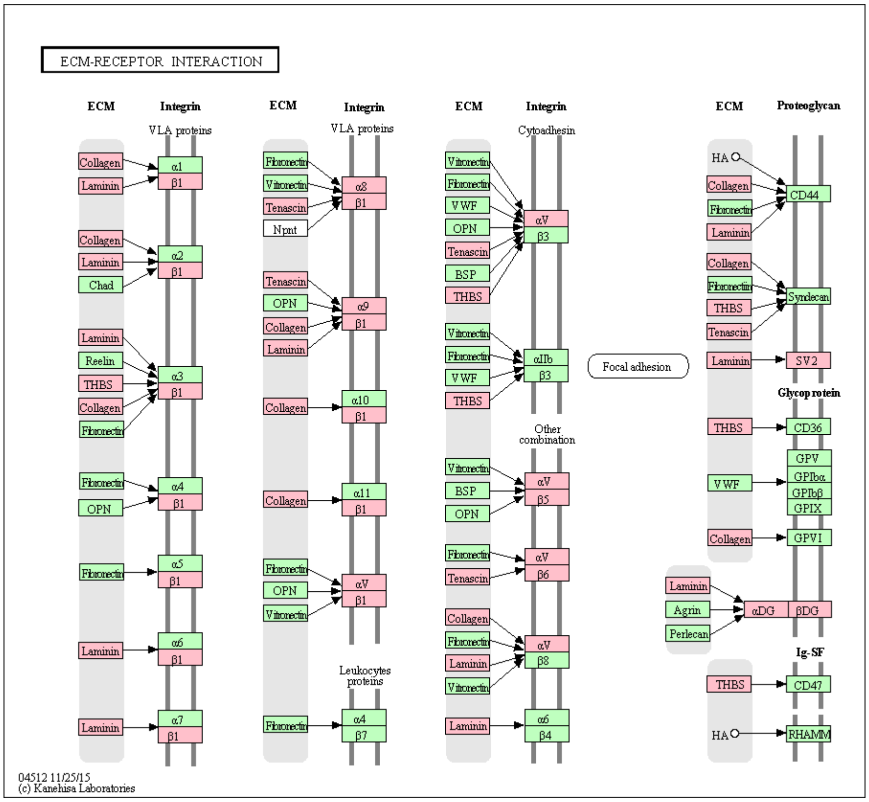

207.3 Legend:

RBH-Blast at 60% Identity + 50% Coverage
Green = Hit in *H. sapiens*
Red = Hit in *H. sapiens* and *C. milli*
White = Not in *H. sapiens*

208 Cytosolic DNA-sensing pathway

208.1 Human Pathway: HSA04623

208.2 Number of Hits: 15

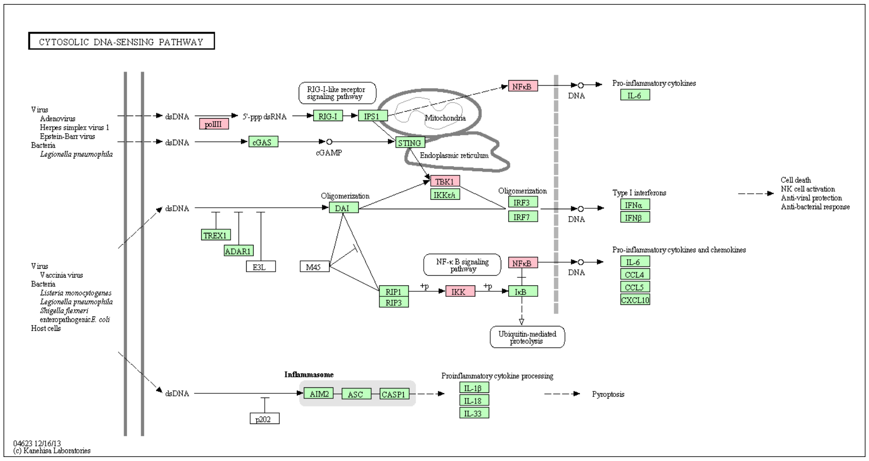

208.3 Legend:

RBH-Blast at 60% Identity + 50% Coverage
Green = Hit in *H. sapiens*
Red = Hit in *H. sapiens* and *C. milli*
White = Not in *H. sapiens*

209 Collecting duct acid secretion

209.1 Human Pathway: HSA04966

209.2 Number of Hits: 15

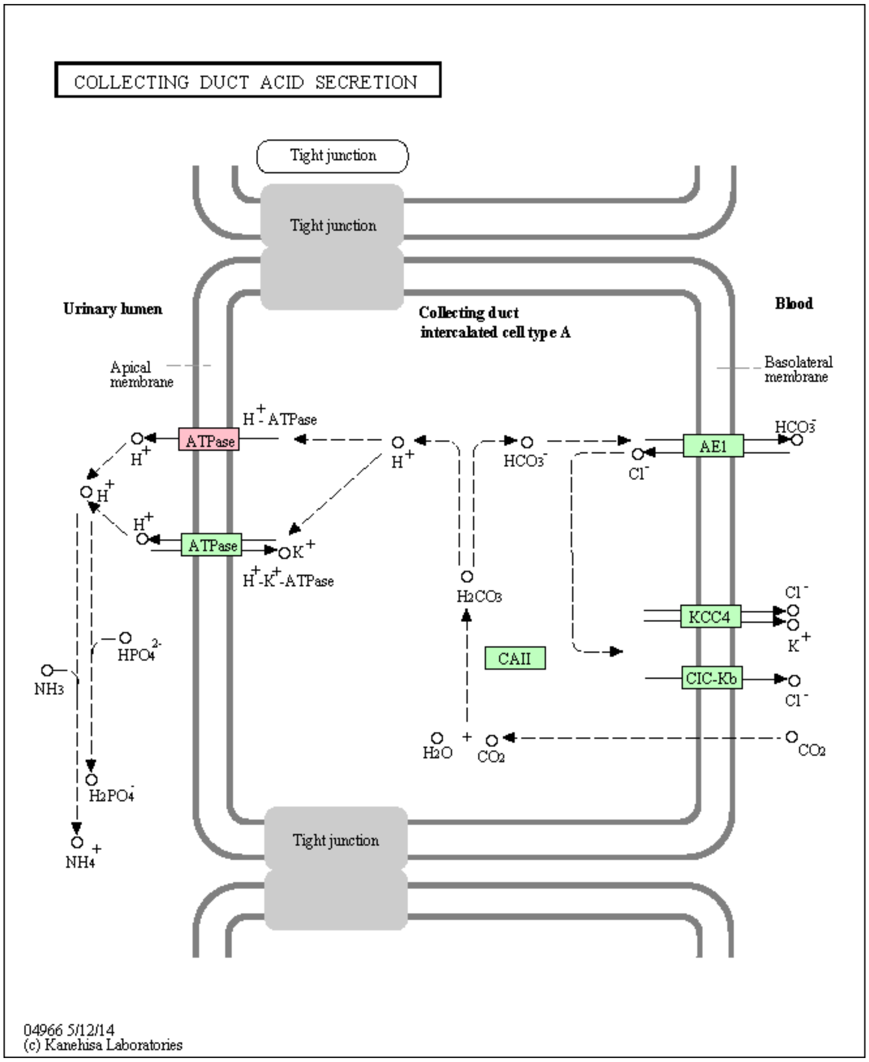

209.3 Legend:

RBH-Blast at 60% Identity + 50% Coverage
Green = Hit in *H. sapiens*
Red = Hit in *H. sapiens* and *C. milli*
White = Not in *H. sapiens*

---

210 Legionellosis

210.1 Human Pathway: HSA05134

210.2 Number of Hits: 15

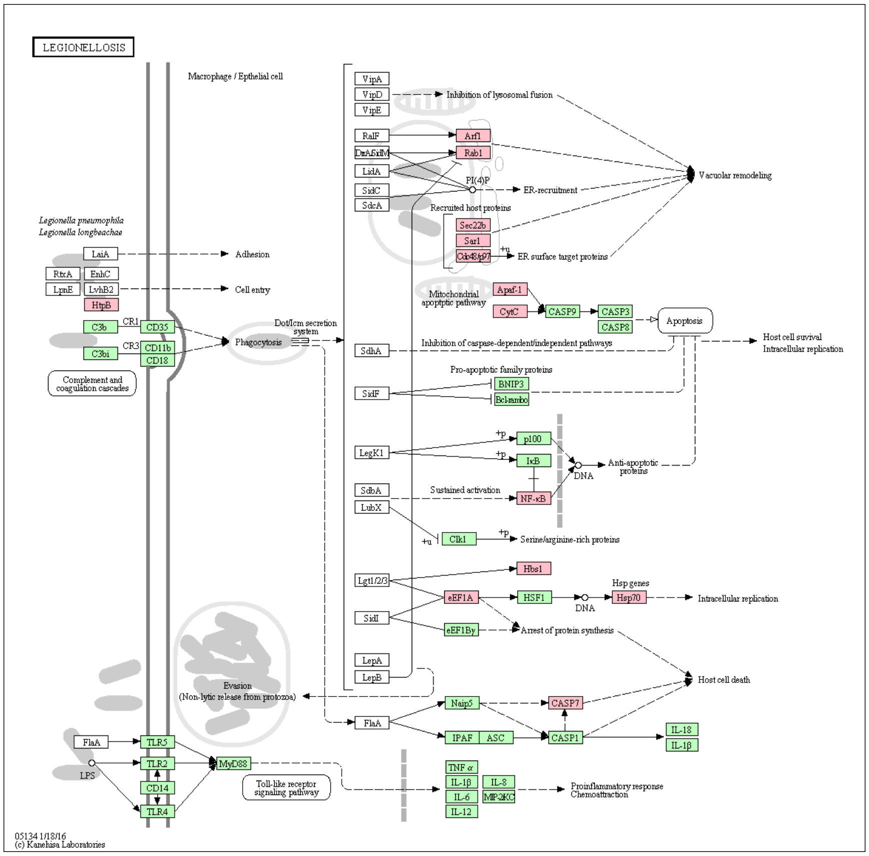

210.3 Legend:

RBH-Blast at 60% Identity + 50% Coverage
Green = Hit in *H. sapiens*
Red = Hit in *H. sapiens* and *C. milli*
White = Not in *H. sapiens*

211 Glycosaminoglycan biosynthesis - heparan sulfate / heparin

211.1 Human Pathway: HSA00534

211.2 Number of Hits: 15

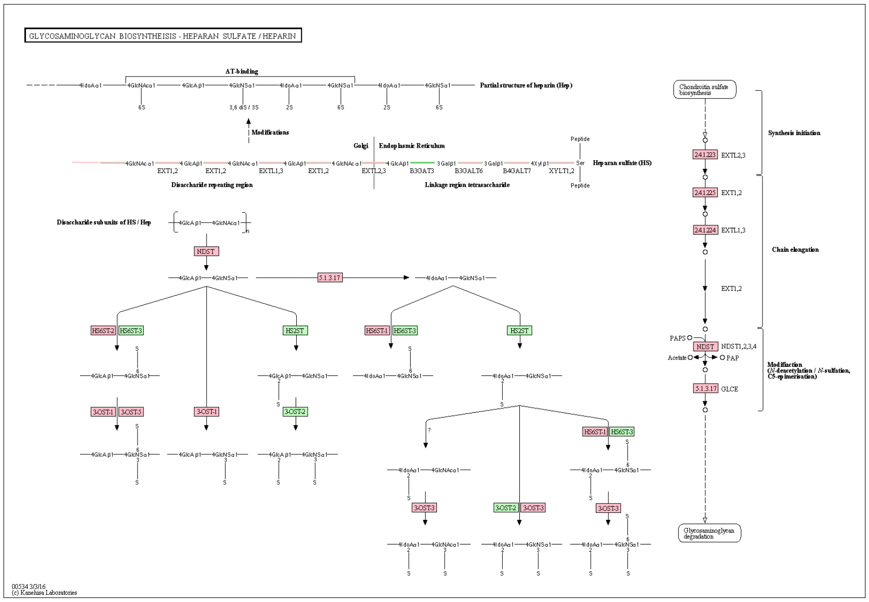

211.3 Legend:

RBH-Blast at 60% Identity + 50% Coverage
Green = Hit in *H. sapiens*
Red = Hit in *H. sapiens* and *C. milli*
White = Not in *H. sapiens*

212 Starch and sucrose metabolism

212.1 Human Pathway: HSA00500

212.2 Number of Hits: 15

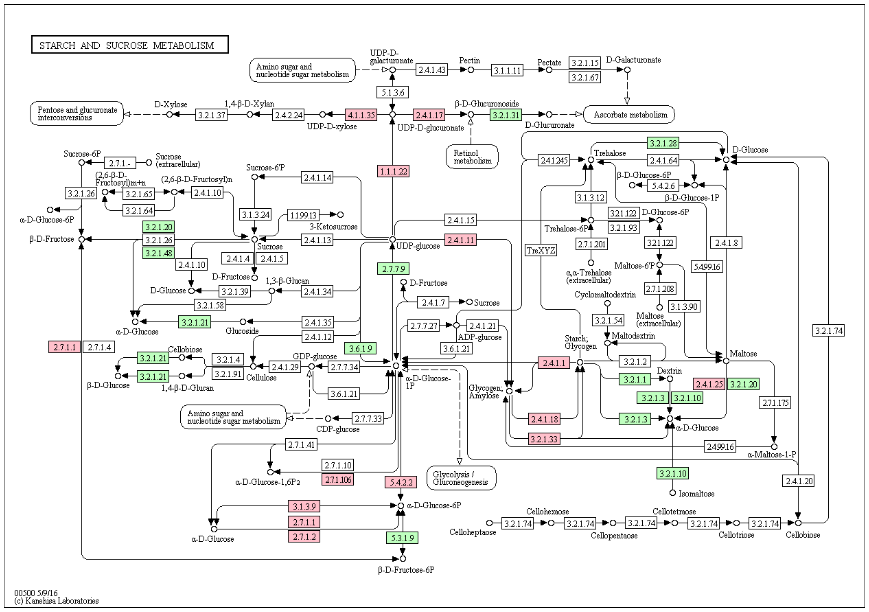

212.3 Legend:

RBH-Blast at 60% Identity + 50% Coverage
Green = Hit in *H. sapiens*
Red = Hit in *H. sapiens* and *C. milli*
White = Not in *H. sapiens*

213 Retinol metabolism

213.1 Human Pathway: HSA00830

213.2 Number of Hits: 15

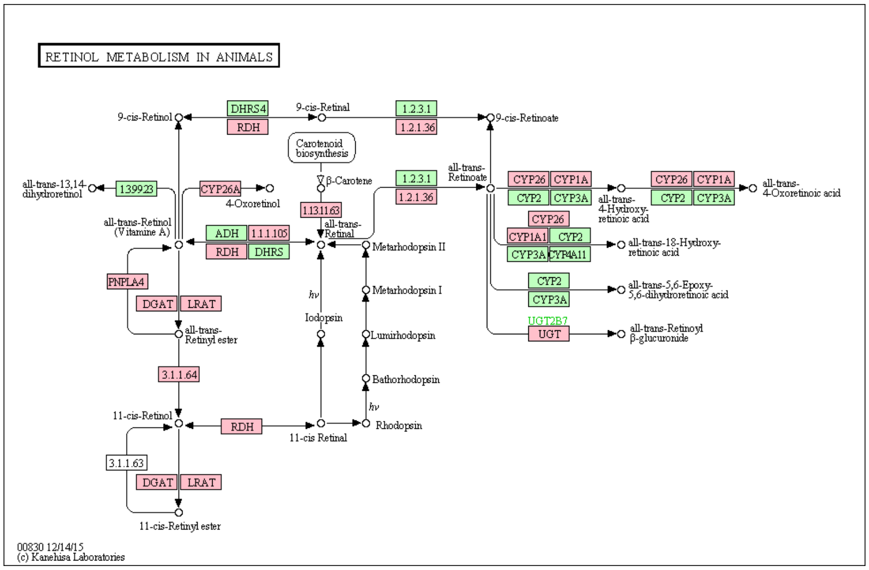

213.3 Legend:

RBH-Blast at 60% Identity + 50% Coverage
Green = Hit in *H. sapiens*
Red = Hit in *H. sapiens* and *C. milli*
White = Not in *H. sapiens*

214 Phototransduction

214.1 Human Pathway: HSA04744

214.2 Number of Hits: 15

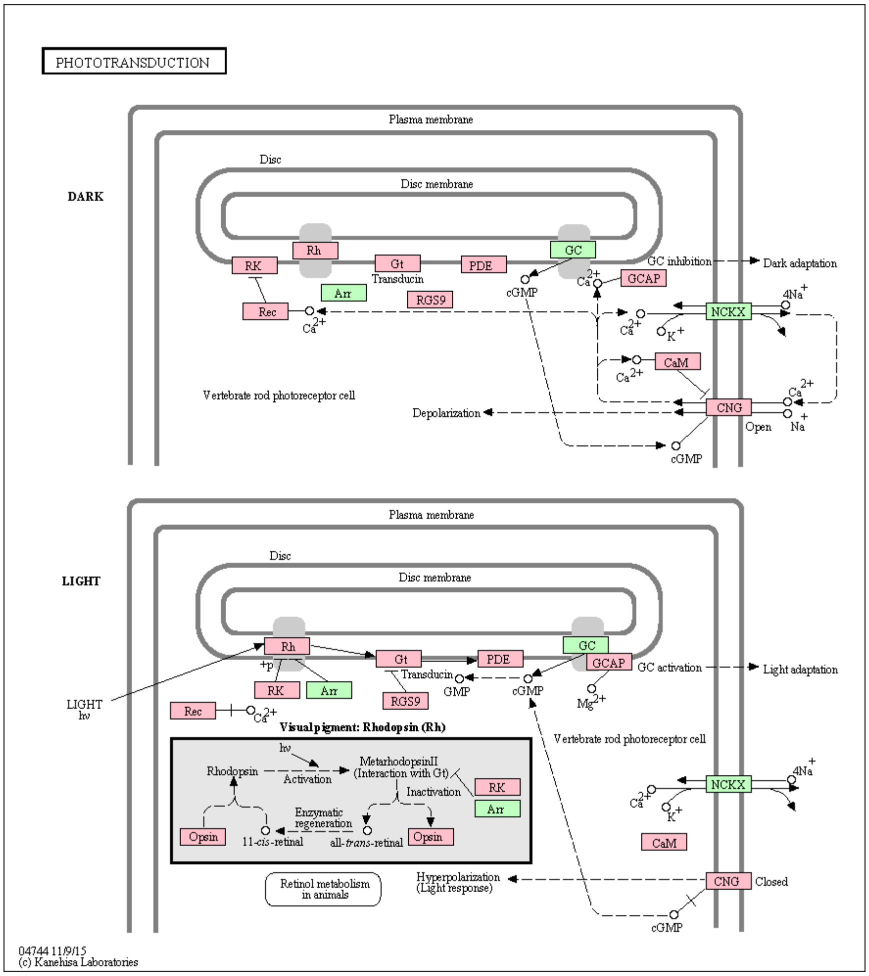

214.3 Legend:

RBH-Blast at 60% Identity + 50% Coverage
Green = Hit in *H. sapiens*
Red = Hit in *H. sapiens* and *C. milli*
White = Not in *H. sapiens*

215 Ether lipid metabolism

215.1 Human Pathway: HSA00565

215.2 Number of Hits: 15

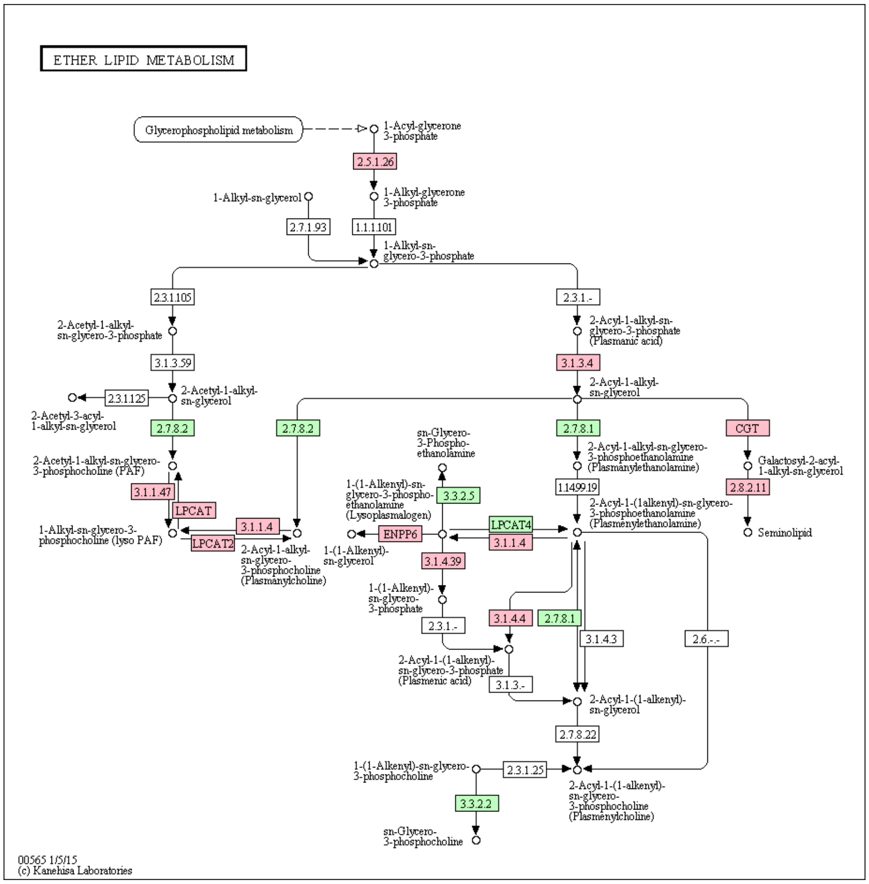

215.3 Legend:

RBH-Blast at 60% Identity + 50% Coverage
Green = Hit in *H. sapiens*
Red = Hit in *H. sapiens* and *C. milli*
White = Not in *H. sapiens*

216 Base excision repair

216.1 Human Pathway: HSA03410

216.2 Number of Hits: 14

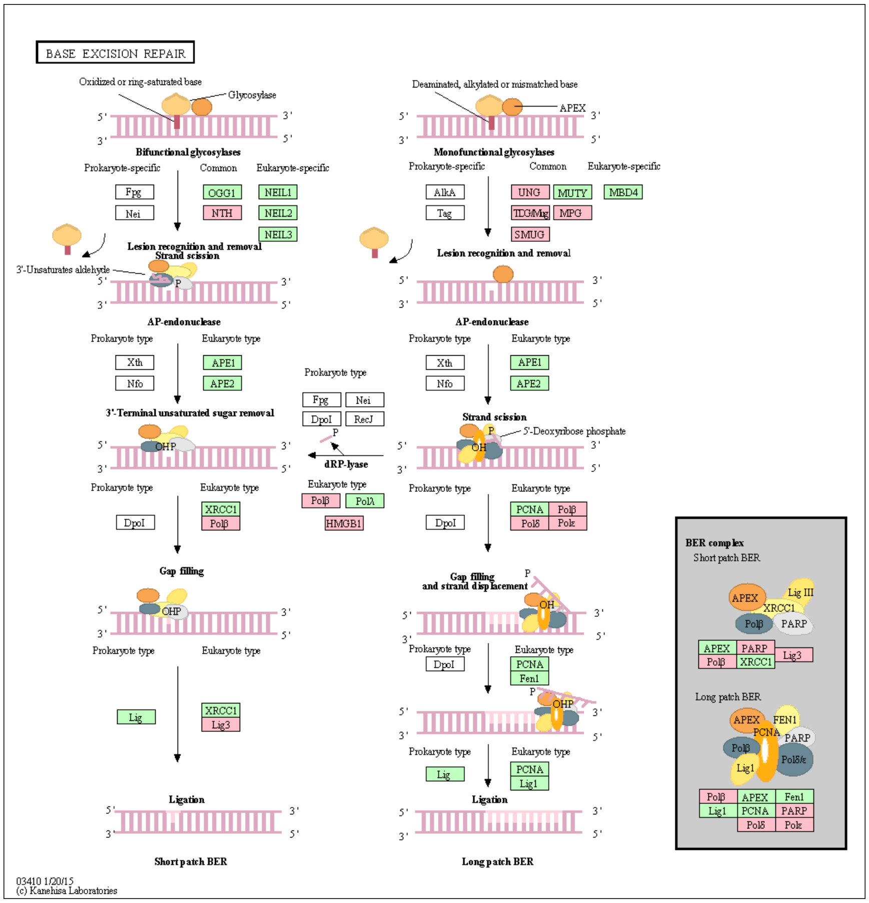

216.3 Legend:

RBH-Blast at 60% Identity + 50% Coverage
Green = Hit in *H. sapiens*
Red = Hit in *H. sapiens* and *C. milli*
White = Not in *H. sapiens*

217 Regulation of autophagy

217.1 Human Pathway: HSA04140

217.2 Number of Hits: 14

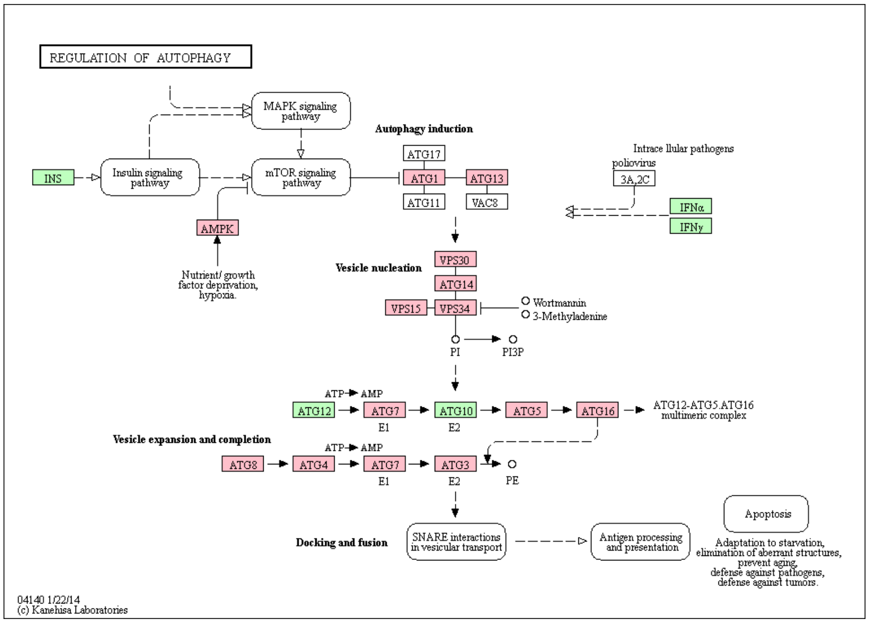

217.3 Legend:

RBH-Blast at 60% Identity + 50% Coverage
Green = Hit in *H. sapiens*
Red = Hit in *H. sapiens* and *C. milli*
White = Not in *H. sapiens*

218 Ovarian steroidogenesis

218.1 Human Pathway: HSA04913

218.2 Number of Hits: 14

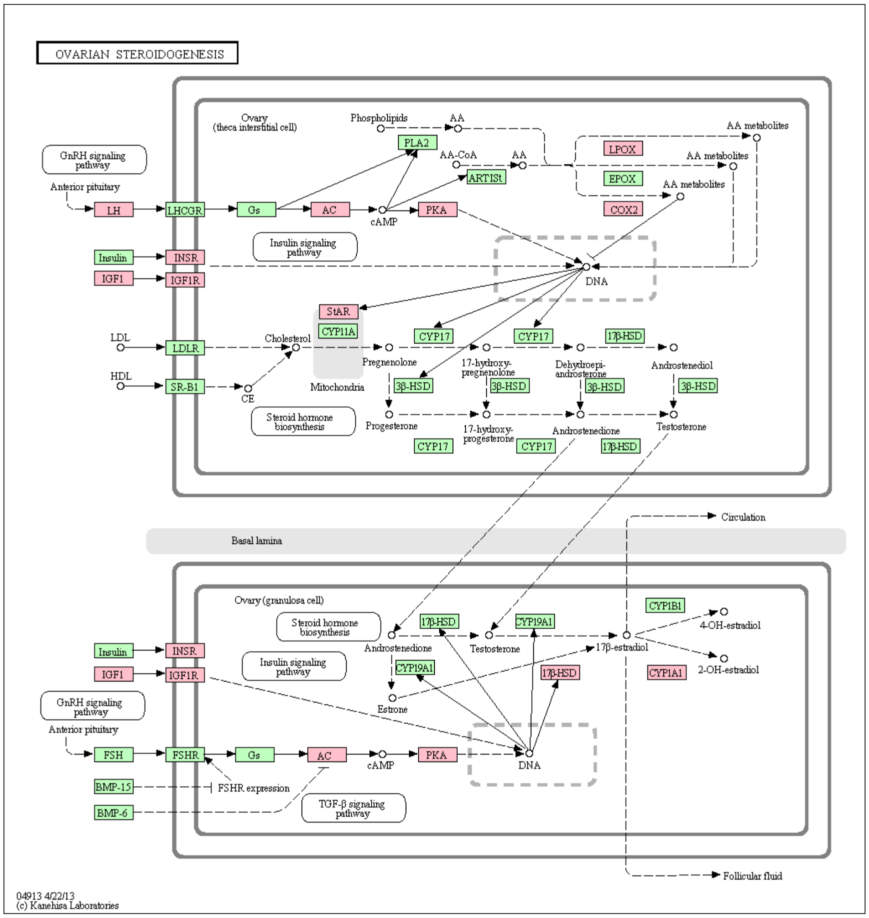

218.3 Legend:

RBH-Blast at 60% Identity + 50% Coverage
Green = Hit in *H. sapiens*
Red = Hit in *H. sapiens* and *C. milli*
White = Not in *H. sapiens*

219 Biosynthesis of unsaturated fatty acids

219.1 Human Pathway: HSA01040

219.2 Number of Hits: 14

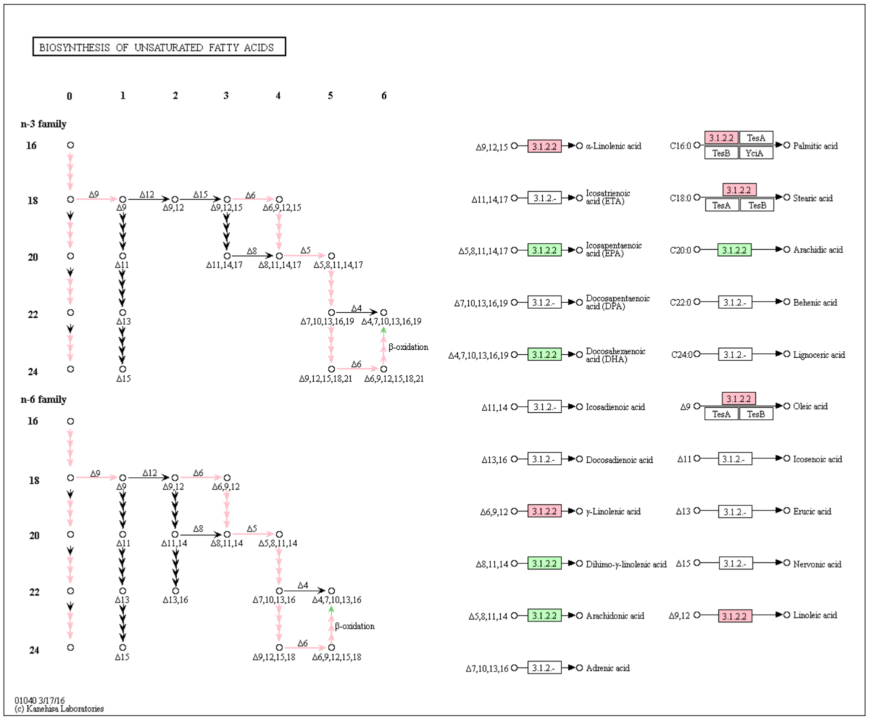

219.3 Legend:

RBH-Blast at 60% Identity + 50% Coverage
Green = Hit in *H. sapiens*
Red = Hit in *H. sapiens* and *C. milli*
White = Not in *H. sapiens*

220 Mineral absorption

220.1 Human Pathway: HSA04978

220.2 Number of Hits: 14

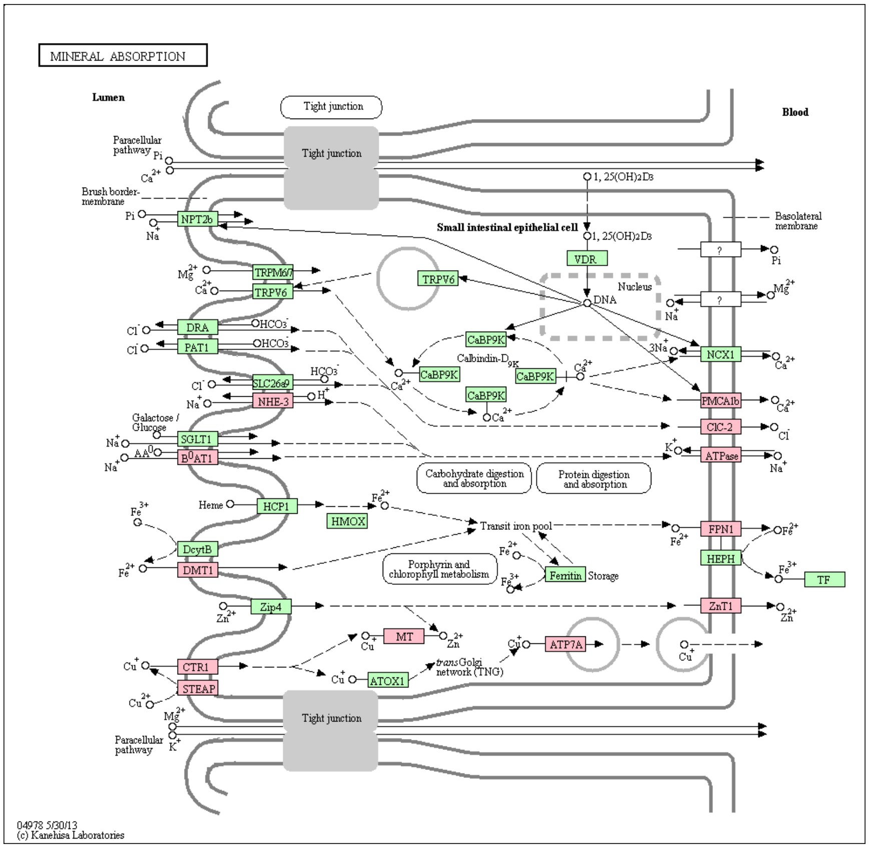

220.3 Legend:

RBH-Blast at 60% Identity + 50% Coverage
Green = Hit in *H. sapiens*
Red = Hit in *H. sapiens* and *C. milli*
White = Not in *H. sapiens*

221 Fanconi anemia pathway

221.1 Human Pathway: HSA03460

221.2 Number of Hits: 14

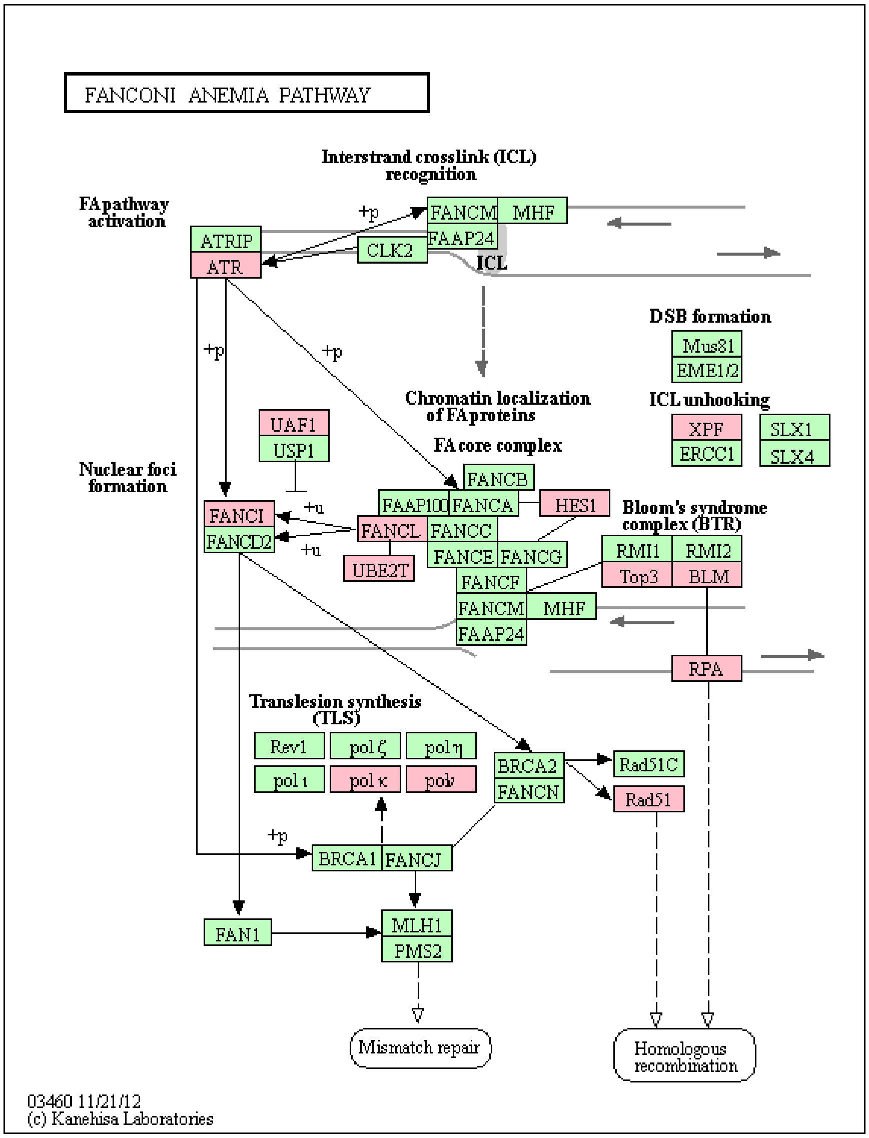

221.3 Legend:

RBH-Blast at 60% Identity + 50% Coverage
Green = Hit in *H. sapiens*
Red = Hit in *H. sapiens* and *C. milli*
White = Not in *H. sapiens*

---

222 Pentose phosphate pathway

222.1 Human Pathway: HSA00030

222.2 Number of Hits: 14

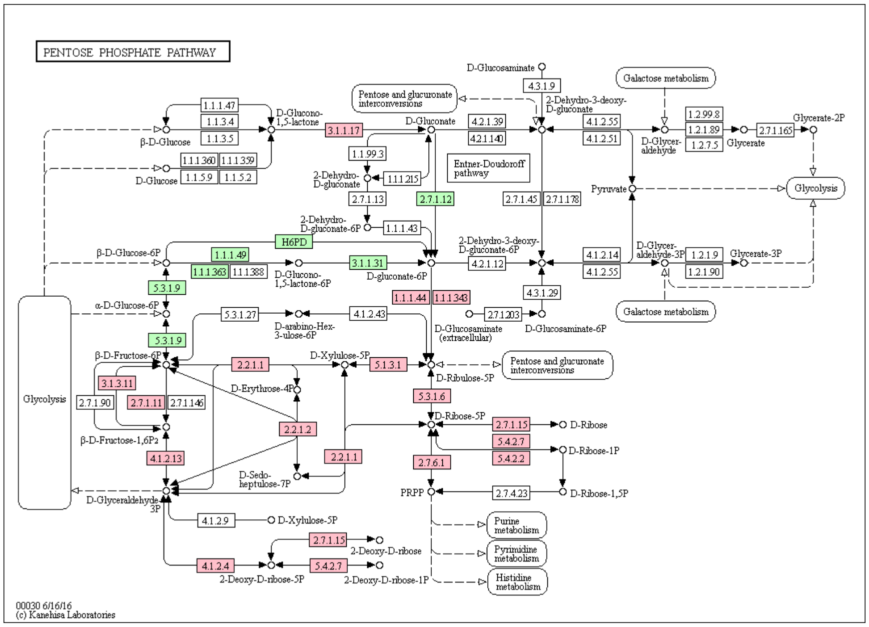

222.3 Legend:

RBH-Blast at 60% Identity + 50% Coverage
Green = Hit in *H. sapiens*
Red = Hit in *H. sapiens* and *C. milli*
White = Not in *H. sapiens*

---

223 Arachidonic acid metabolism

223.1 Human Pathway: HSA00590

223.2 Number of Hits: 14

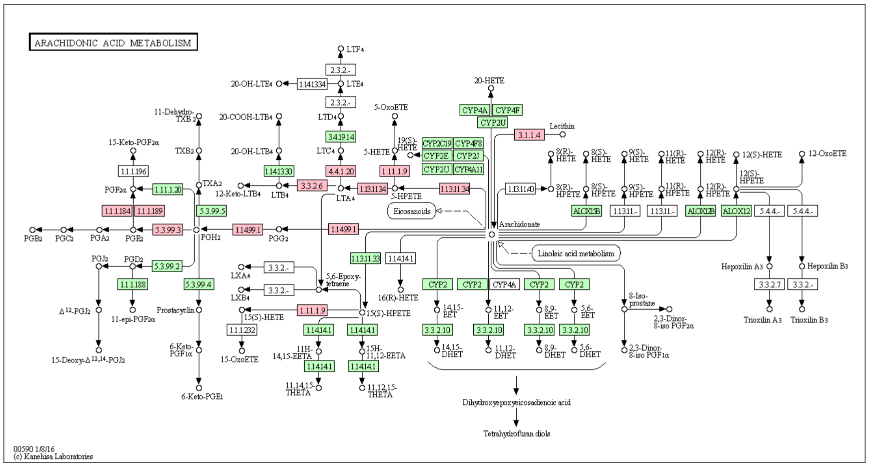

223.3 Legend:

RBH-Blast at 60% Identity + 50% Coverage
Green = Hit in *H. sapiens*
Red = Hit in *H. sapiens* and *C. milli*
White = Not in *H. sapiens*

224 Drug metabolism - other enzymes

224.1 Human Pathway: HSA00983

224.2 Number of Hits: 14

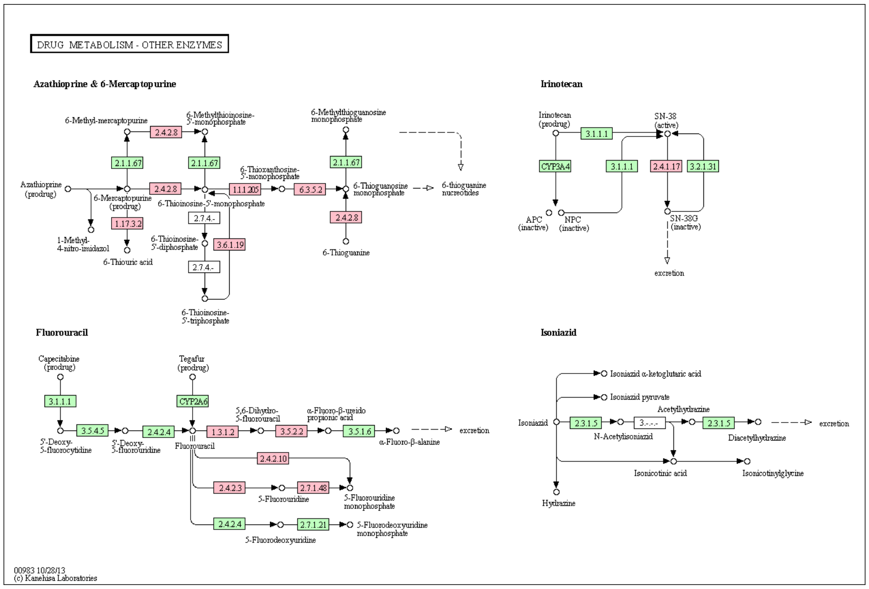

224.3 Legend:

RBH-Blast at 60% Identity + 50% Coverage
Green = Hit in *H. sapiens*
Red = Hit in *H. sapiens* and *C. milli*
White = Not in *H. sapiens*

225 Fat digestion and absorption

225.1 Human Pathway: HSA04975

225.2 Number of Hits: 13

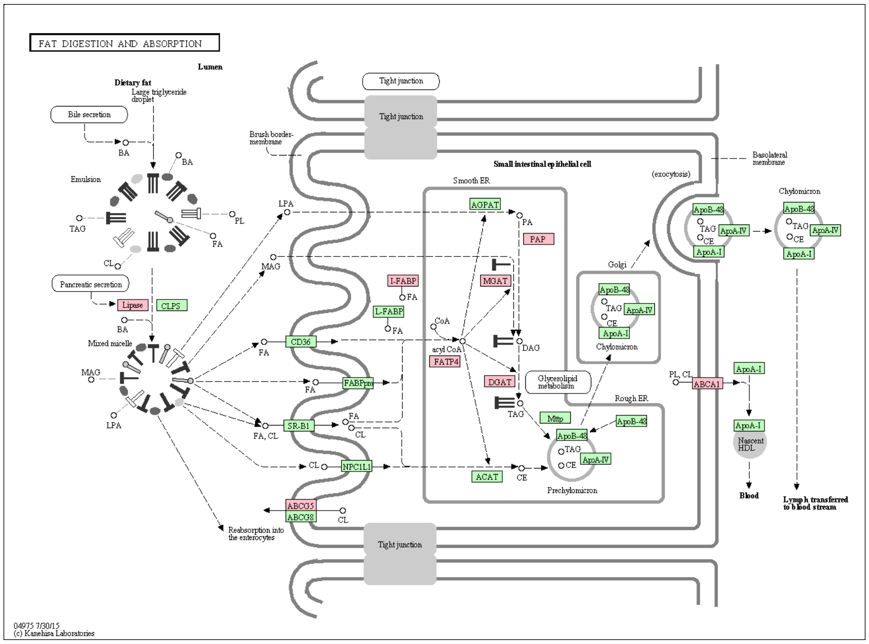

225.3 Legend:

RBH-Blast at 60% Identity + 50% Coverage
Green = Hit in *H. sapiens*
Red = Hit in *H. sapiens* and *C. milli*
White = Not in *H. sapiens*

226 Porphyrin and chlorophyll metabolism

226.1 Human Pathway: HSA00860

226.2 Number of Hits: 13

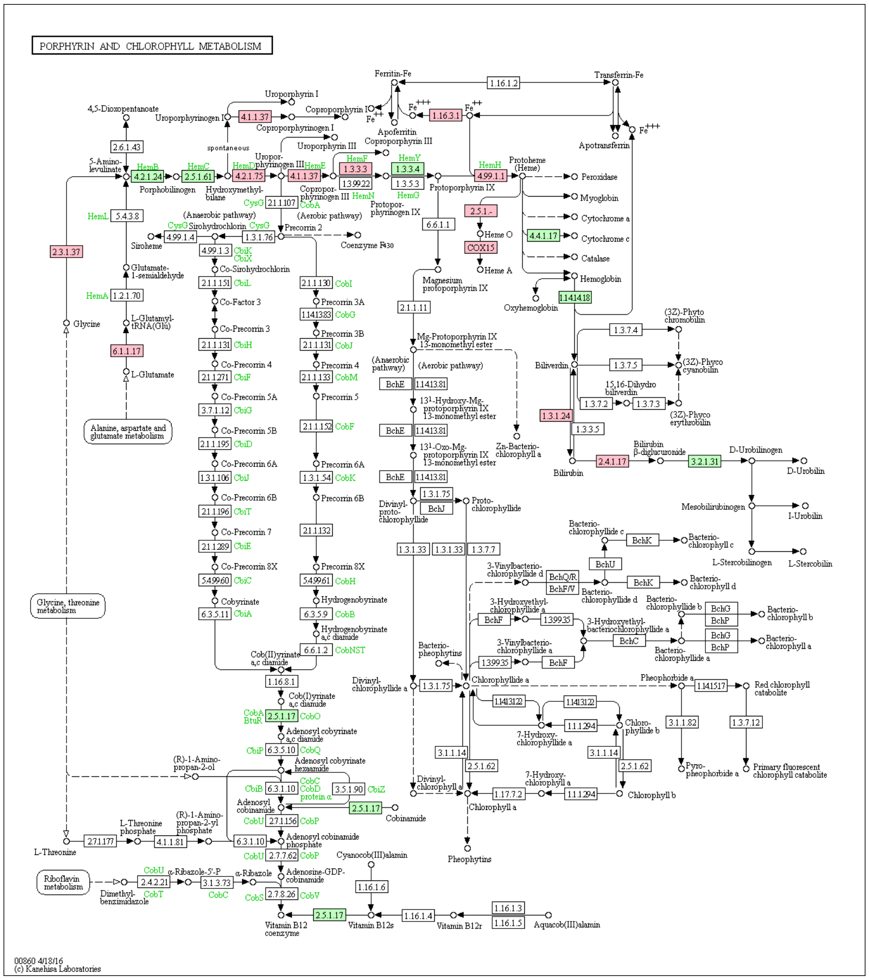

226.3 Legend:

RBH-Blast at 60% Identity + 50% Coverage
Green = Hit in *H. sapiens*
Red = Hit in *H. sapiens* and *C. milli*
White = Not in *H. sapiens*

227 Thyroid cancer

227.1 Human Pathway: HSA05216

227.2 Number of Hits: 13

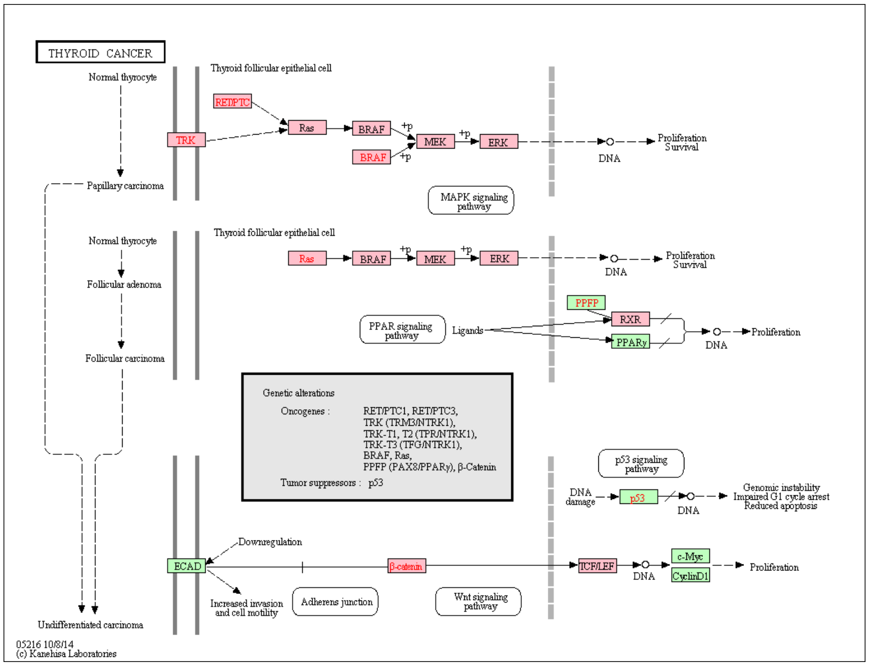

227.3 Legend:

RBH-Blast at 60% Identity + 50% Coverage
Green = Hit in *H. sapiens*
Red = Hit in *H. sapiens* and *C. milli*
White = Not in *H. sapiens*

228 Galactose metabolism

228.1 Human Pathway: HSA00052

228.2 Number of Hits: 13

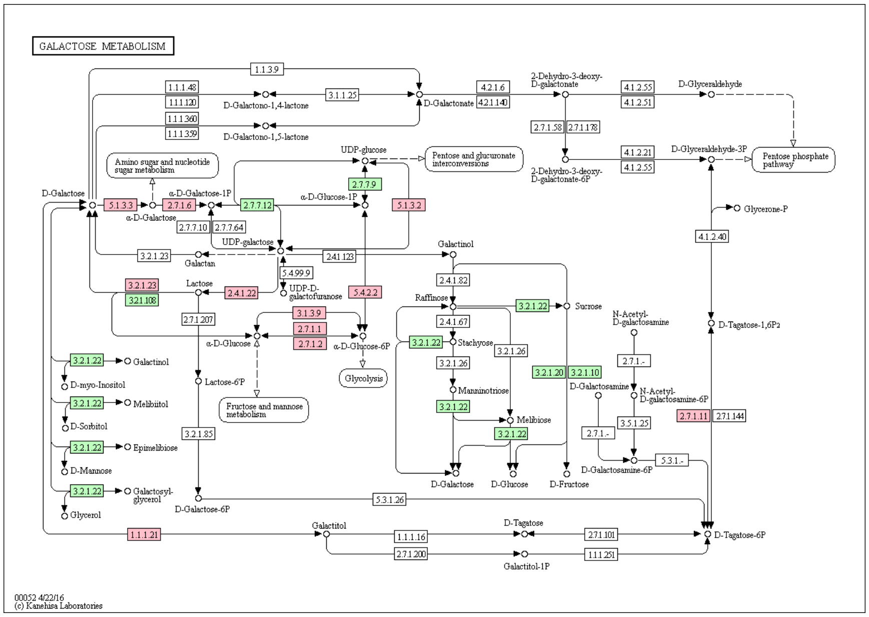

228.3 Legend:

RBH-Blast at 60% Identity + 50% Coverage
Green = Hit in *H. sapiens*
Red = Hit in *H. sapiens* and *C. milli*
White = Not in *H. sapiens*

229 Arginine biosynthesis

229.1 Human Pathway: HSA00220

229.2 Number of Hits: 13

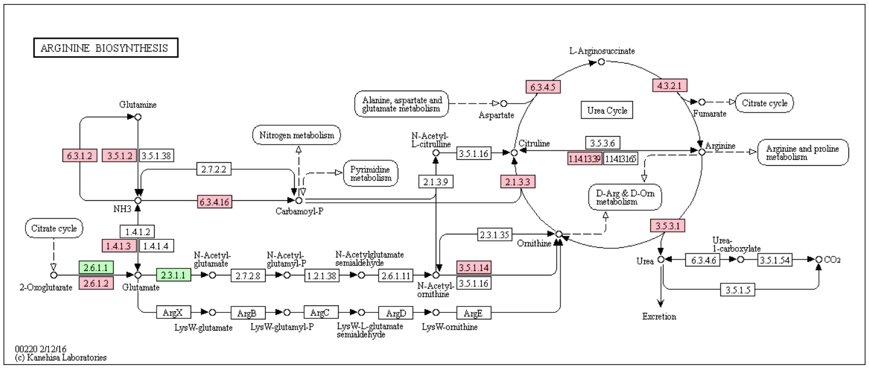

229.3 Legend:

RBH-Blast at 60% Identity + 50% Coverage
Green = Hit in *H. sapiens*
Red = Hit in *H. sapiens* and *C. milli*
White = Not in *H. sapiens*

230 Chemical carcinogenesis

230.1 Human Pathway: HSA05204

230.2 Number of Hits: 12

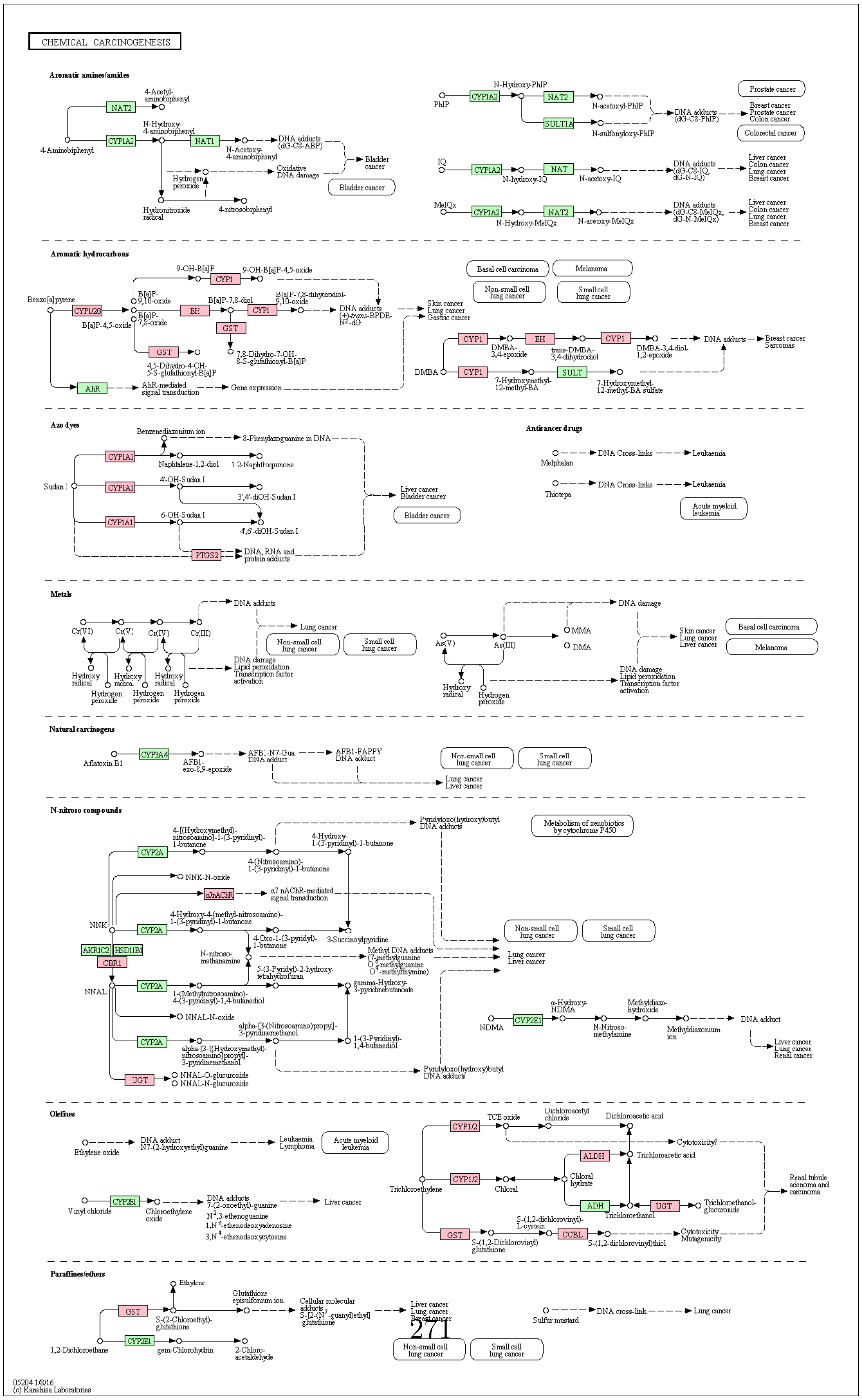

230.3 Legend:

RBH-Blast at 60% Identity + 50% Coverage
Green = Hit in *H. sapiens*
Red = Hit in *H. sapiens* and *C. milli*
White = Not in *H. sapiens*

231 Circadian rhythm

231.1 Human Pathway: HSA04710

231.2 Number of Hits: 12

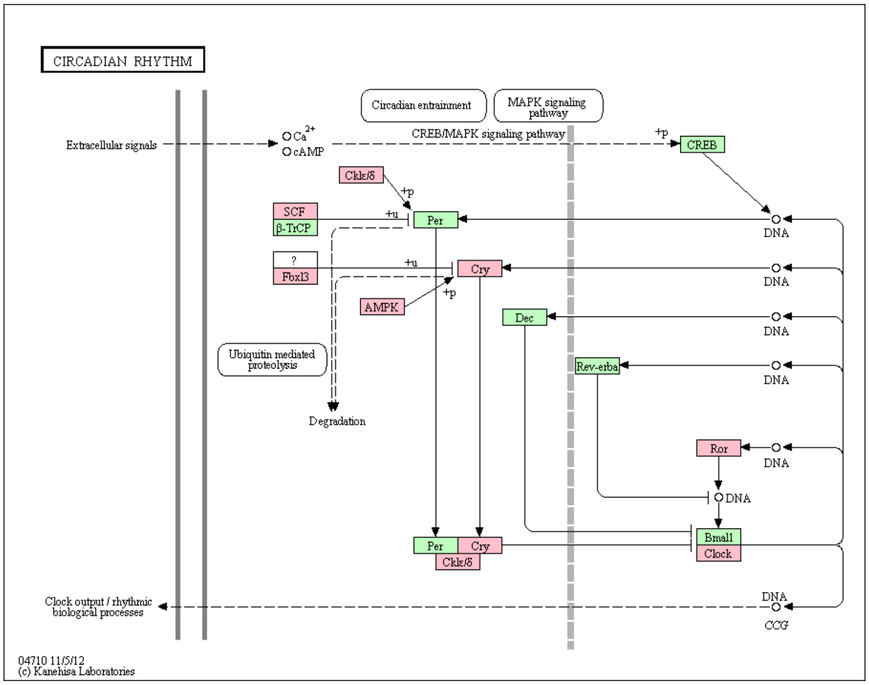

231.3 Legend:

RBH-Blast at 60% Identity + 50% Coverage
Green = Hit in *H. sapiens*
Red = Hit in *H. sapiens* and *C. milli*
White = Not in *H. sapiens*

232 Tyrosine metabolism

232.1 Human Pathway: HSA00350

232.2 Number of Hits: 12

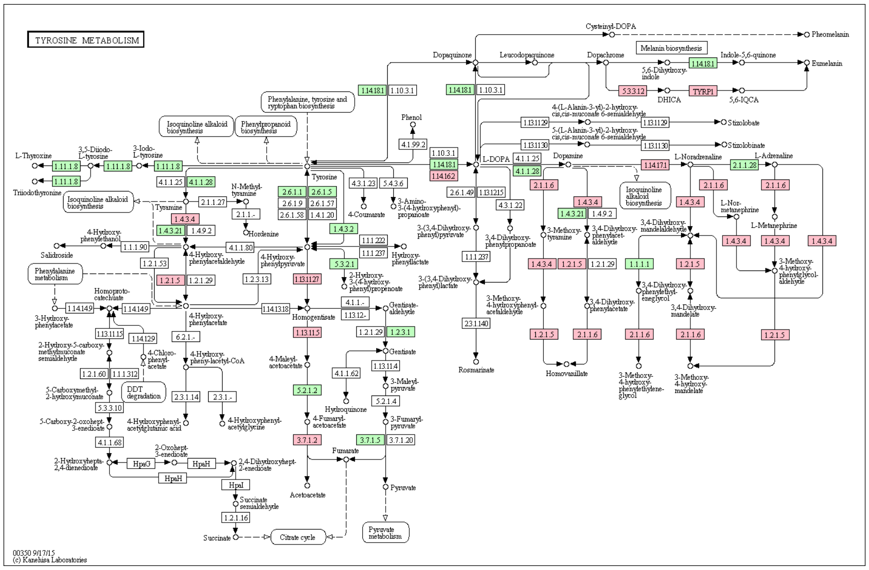

232.3 Legend:

RBH-Blast at 60% Identity + 50% Coverage
Green = Hit in *H. sapiens*
Red = Hit in *H. sapiens* and *C. milli*
White = Not in *H. sapiens*

233 Nicotinate and nicotinamide metabolism

233.1 Human Pathway: HSA00760

233.2 Number of Hits: 12

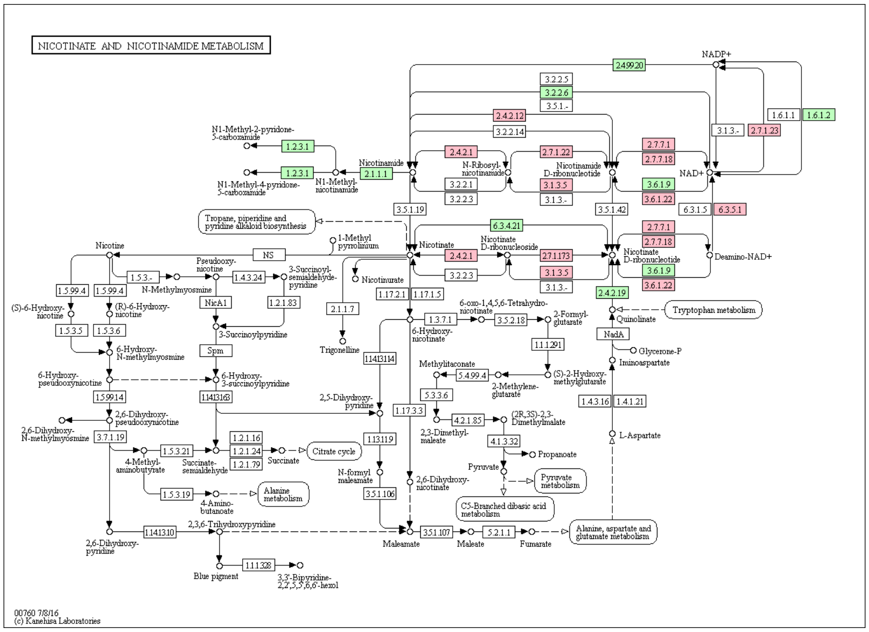

233.3 Legend:

RBH-Blast at 60% Identity + 50% Coverage
Green = Hit in *H. sapiens*
Red = Hit in *H. sapiens* and *C. milli*
White = Not in *H. sapiens*

234 One carbon pool by folate

234.1 Human Pathway: HSA00670

234.2 Number of Hits: 12

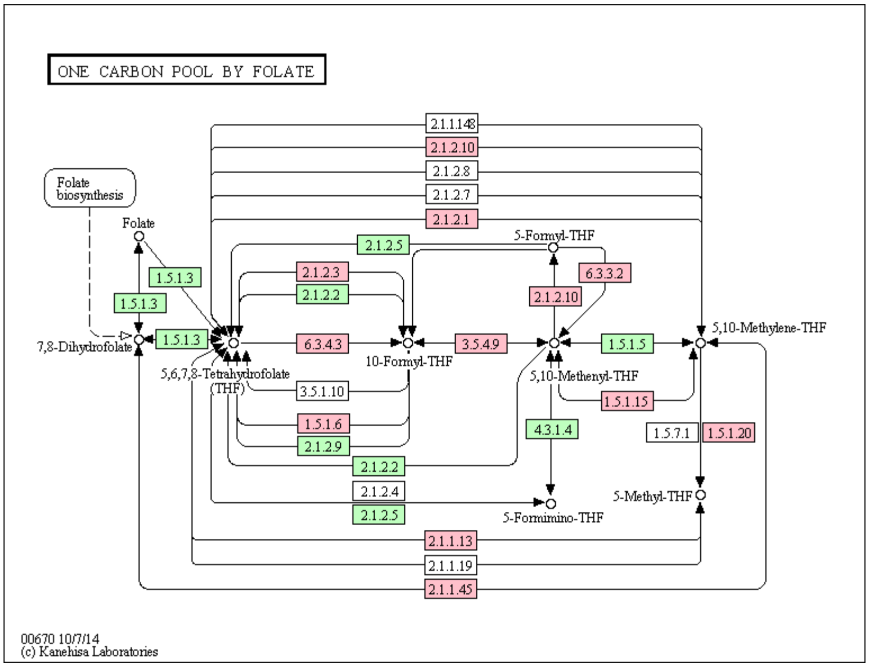

234.3 Legend:

RBH-Blast at 60% Identity + 50% Coverage
Green = Hit in *H. sapiens*
Red = Hit in *H. sapiens* and *C. milli*
White = Not in *H. sapiens*

235 Histidine metabolism

235.1 Human Pathway: HSA00340

235.2 Number of Hits: 12

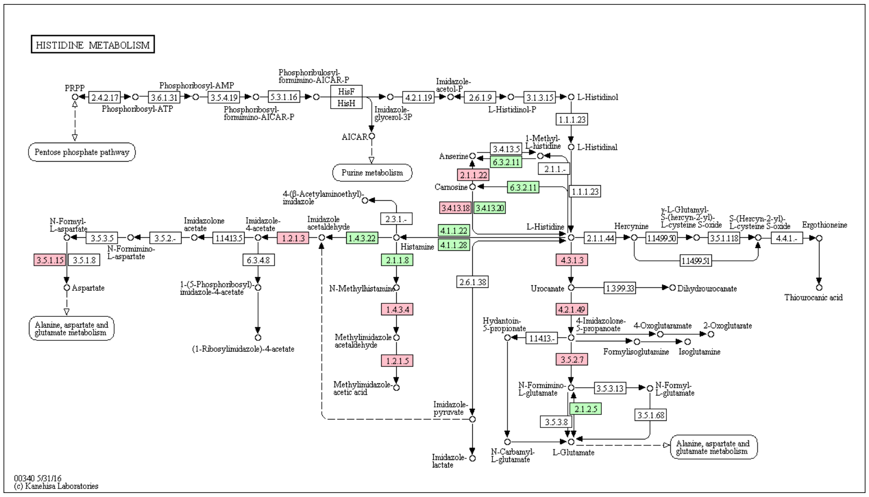

235.3 Legend:

RBH-Blast at 60% Identity + 50% Coverage
Green = Hit in *H. sapiens*
Red = Hit in *H. sapiens* and *C. milli*
White = Not in *H. sapiens*

236 Butanoate metabolism

236.1 Human Pathway: HSA00650

236.2 Number of Hits: 12

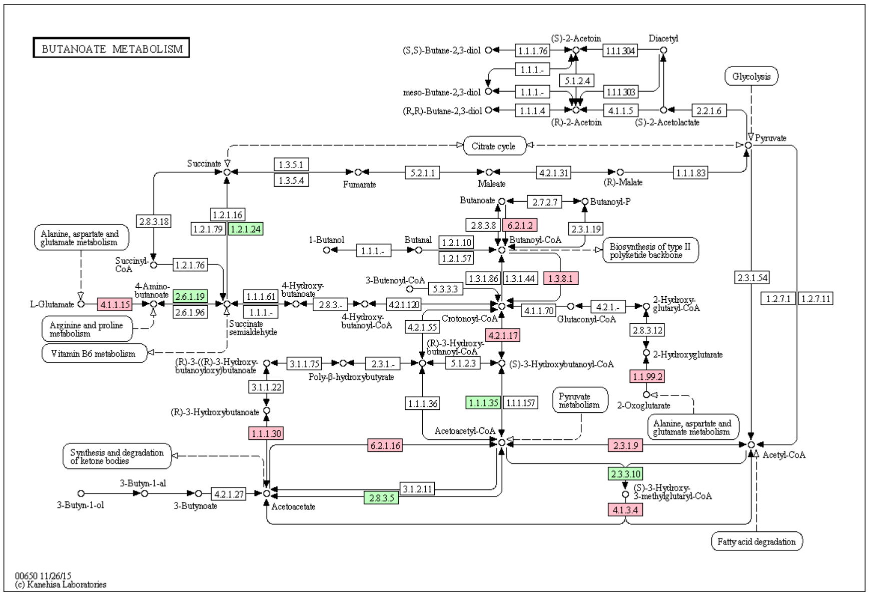

236.3 Legend:

RBH-Blast at 60% Identity + 50% Coverage
Green = Hit in *H. sapiens*
Red = Hit in *H. sapiens* and *C. milli*
White = Not in *H. sapiens*

237 Cytokine-cytokine receptor interaction

237.1 Human Pathway: HSA04060

237.2 Number of Hits: 11

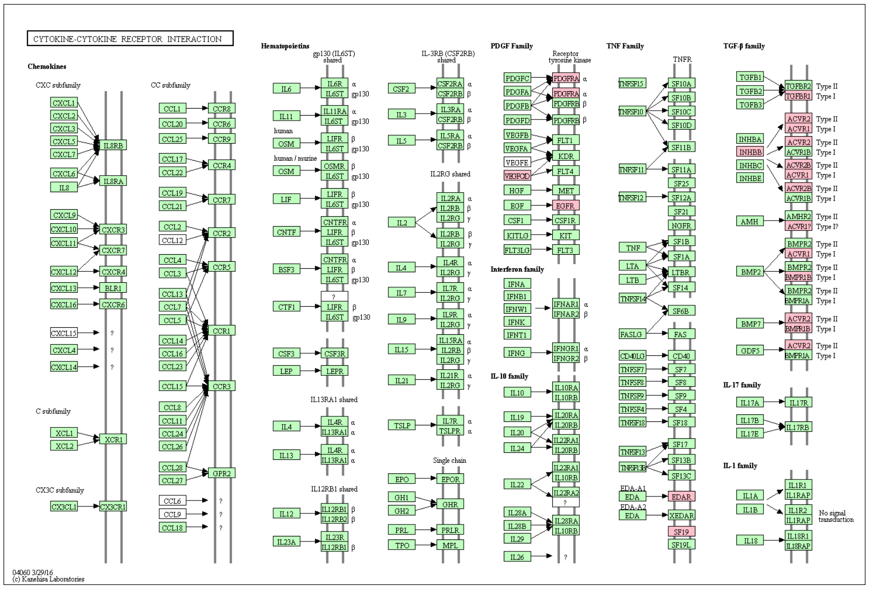

237.3 Legend:

RBH-Blast at 60% Identity + 50% Coverage
Green = Hit in *H. sapiens*
Red = Hit in *H. sapiens* and *C. milli*
White = Not in *H. sapiens*

238 Proximal tubule bicarbonate reclamation

238.1 Human Pathway: HSA04964

238.2 Number of Hits: 11

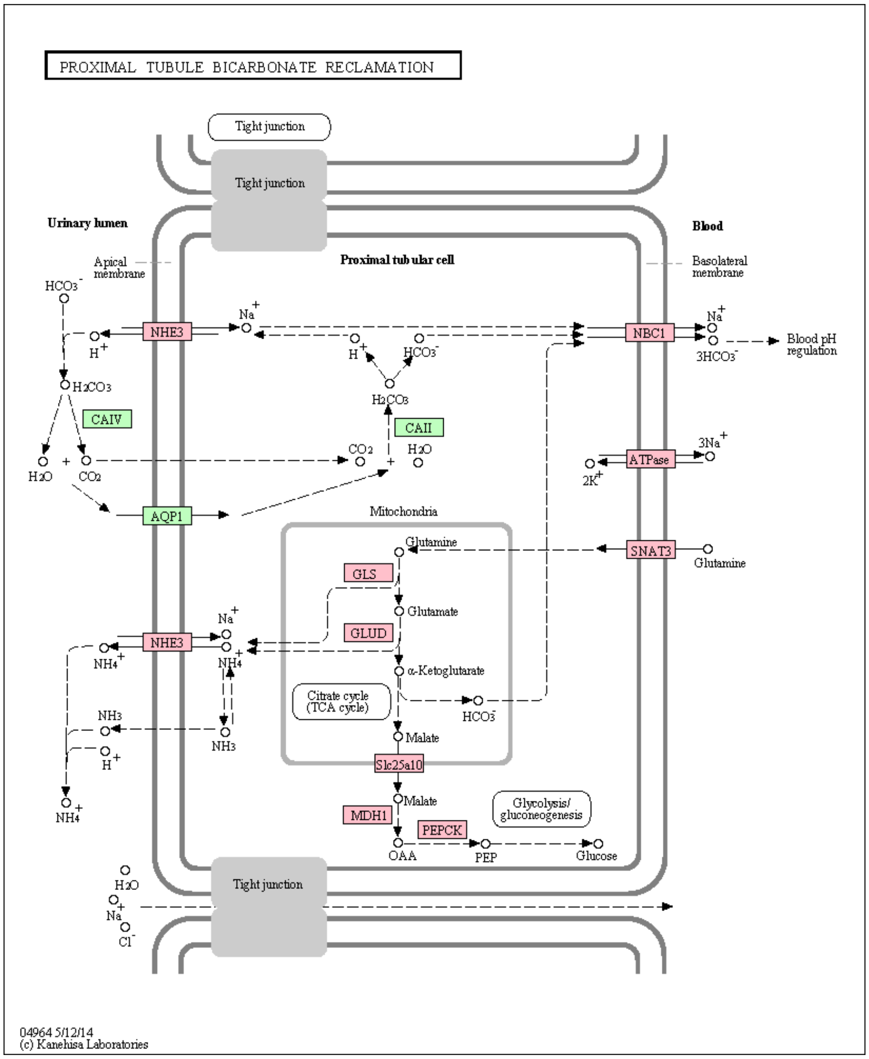

238.3 Legend:

RBH-Blast at 60% Identity + 50% Coverage
Green = Hit in *H. sapiens*
Red = Hit in *H. sapiens* and *C. milli*
White = Not in *H. sapiens*

239 Homologous recombination

239.1 Human Pathway: HSA03440

239.2 Number of Hits: 11

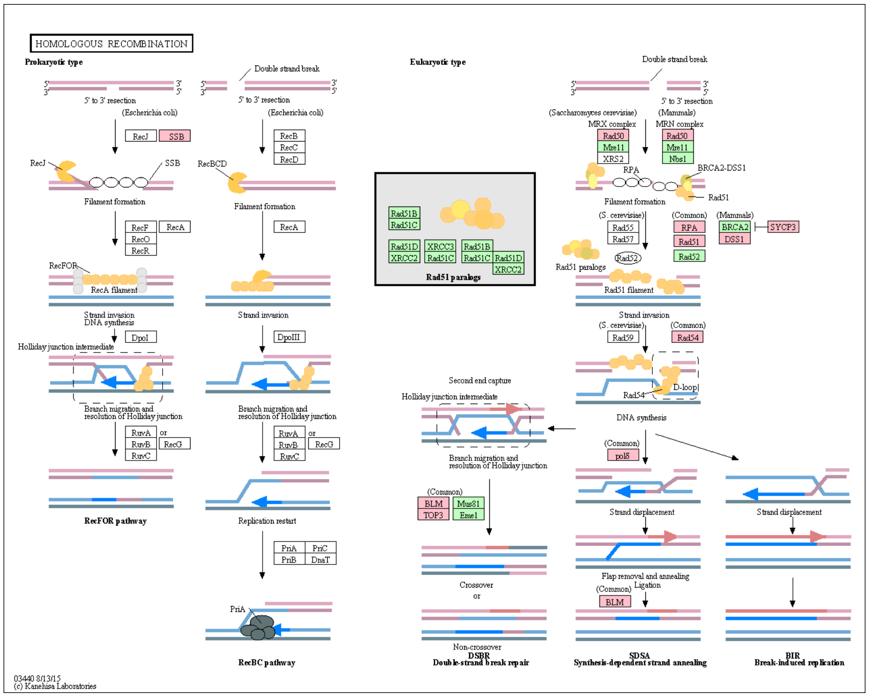

239.3 Legend:

RBH-Blast at 60% Identity + 50% Coverage
Green = Hit in *H. sapiens*
Red = Hit in *H. sapiens* and *C. milli*
White = Not in *H. sapiens*

240 Hippo signaling pathway -multiple species

240.1 Human Pathway: HSA04392

240.2 Number of Hits: 11

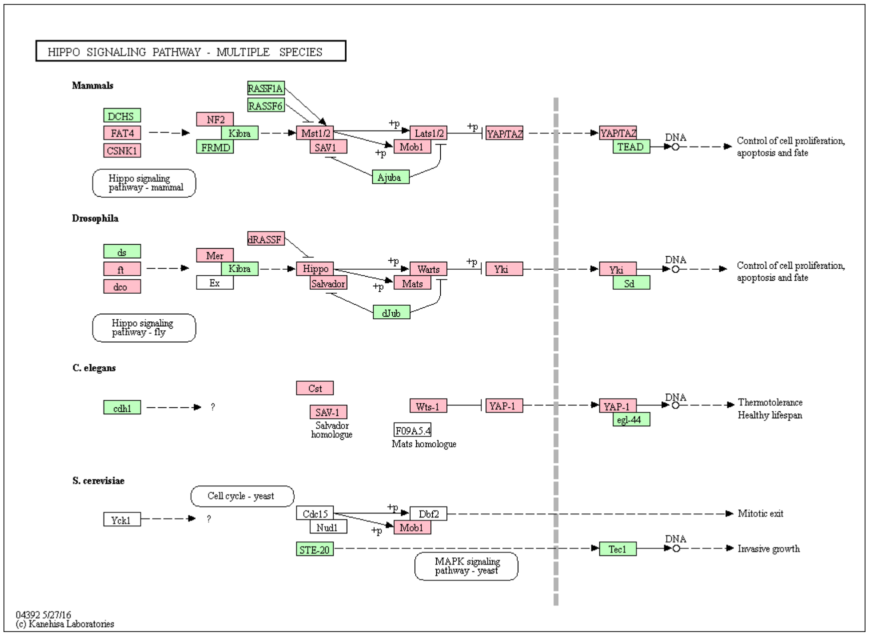

240.3 Legend:

RBH-Blast at 60% Identity + 50% Coverage
Green = Hit in *H. sapiens*
Red = Hit in *H. sapiens* and *C. milli*
White = Not in *H. sapiens*

241 Prion diseases

241.1 Human Pathway: HSA05020

241.2 Number of Hits: 11

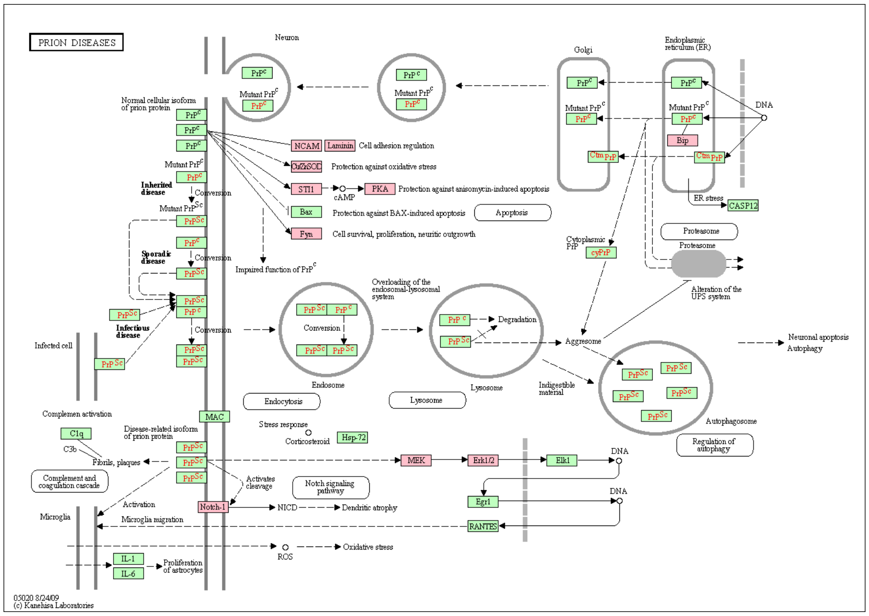

241.3 Legend:

RBH-Blast at 60% Identity + 50% Coverage
Green = Hit in *H. sapiens*
Red = Hit in *H. sapiens* and *C. milli*
White = Not in *H. sapiens*

242 Apoptosis - multiple species

242.1 Human Pathway: HSA04215

242.2 Number of Hits: 11

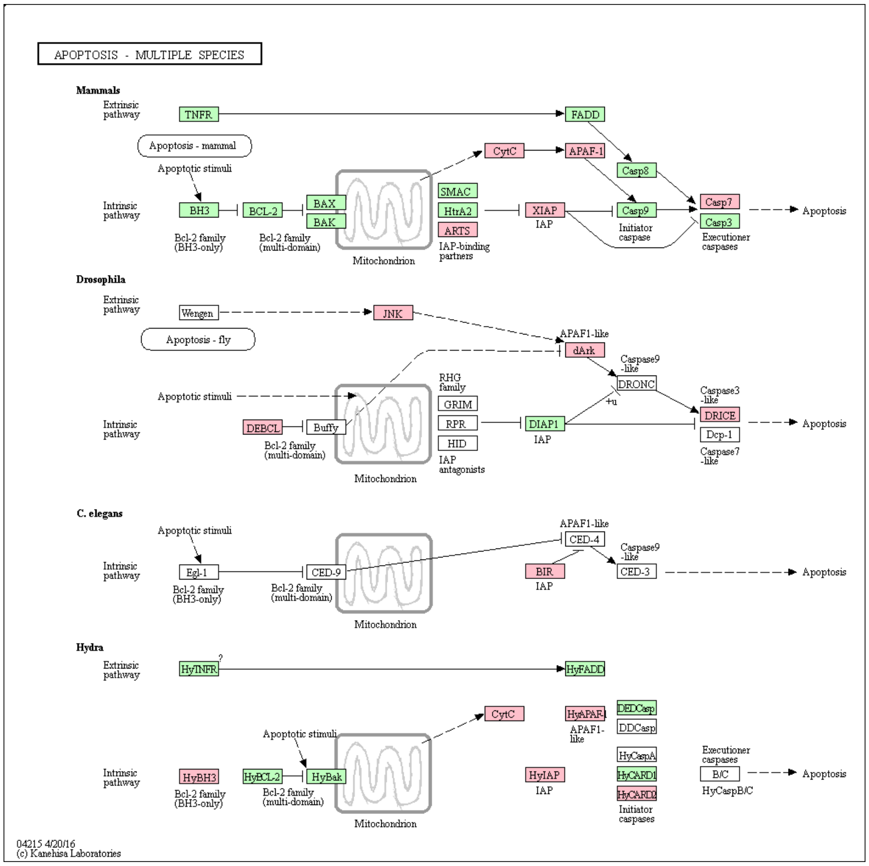

242.3 Legend:

RBH-Blast at 60% Identity + 50% Coverage
Green = Hit in *H. sapiens*
Red = Hit in *H. sapiens* and *C. milli*
White = Not in *H. sapiens*

243 Glycosylphosphatidylinositol(GPI)-anchor biosynthesis

243.1 Human Pathway: HSA00563

243.2 Number of Hits: 10

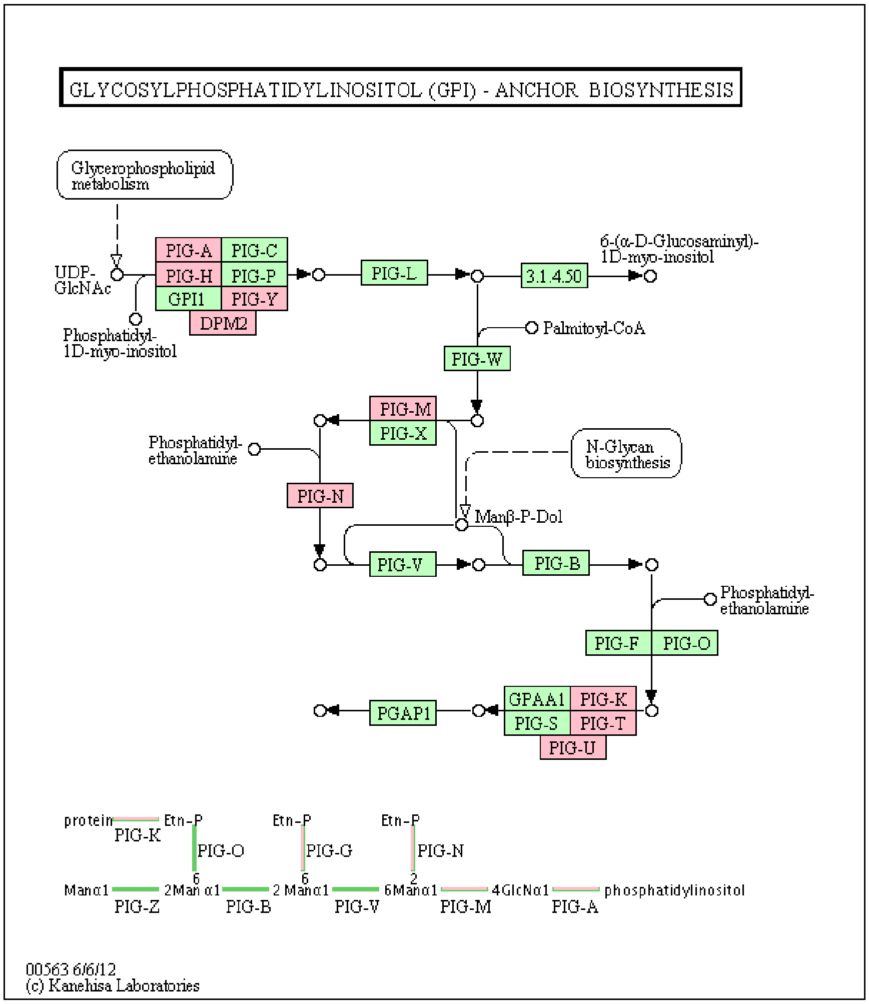

243.3 Legend:

RBH-Blast at 60% Identity + 50% Coverage
Green = Hit in *H. sapiens*
Red = Hit in *H. sapiens* and *C. milli*
White = Not in *H. sapiens*

244 Antigen processing and presentation

244.1 Human Pathway: HSA04612

244.2 Number of Hits: 10

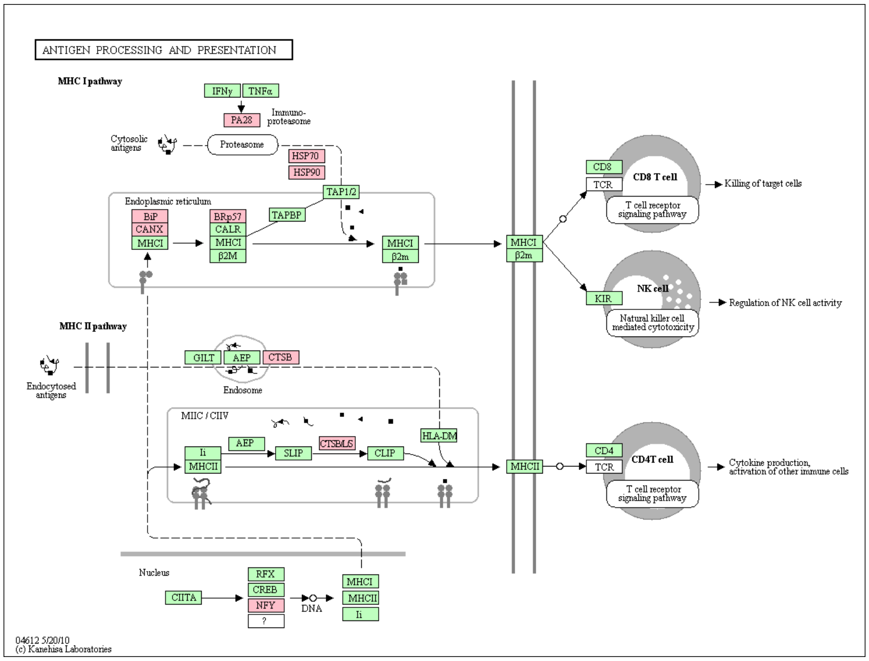

244.3 Legend:

RBH-Blast at 60% Identity + 50% Coverage
Green = Hit in *H. sapiens*
Red = Hit in *H. sapiens* and *C. milli*
White = Not in *H. sapiens*

245 Maturity onset diabetes of the young

245.1 Human Pathway: HSA04950

245.2 Number of Hits: 10

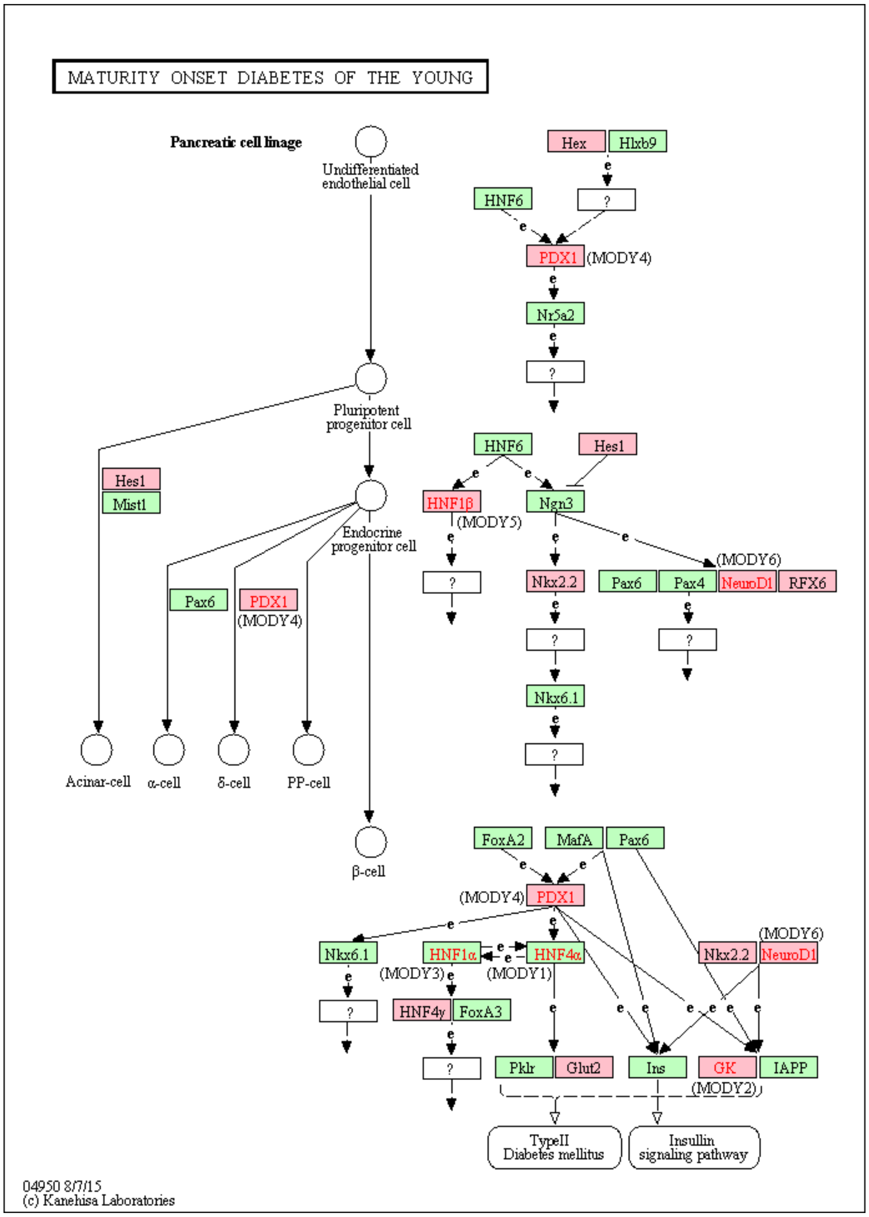

245.3 Legend:

RBH-Blast at 60% Identity + 50% Coverage
Green = Hit in *H. sapiens*
Red = Hit in *H. sapiens* and *C. milli*
White = Not in *H. sapiens*

---

246 Glycosaminoglycan biosynthesis - chondroitin sulfate / dermatan sulfate

246.1 Human Pathway: HSA00532

246.2 Number of Hits: 10

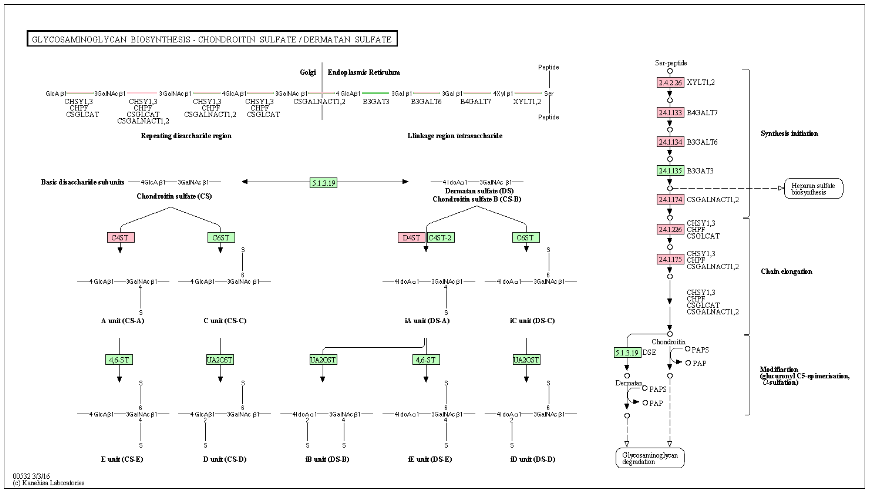

246.3 Legend:

RBH-Blast at 60% Identity + 50% Coverage
Green = Hit in *H. sapiens*
Red = Hit in *H. sapiens* and *C. milli*
White = Not in *H. sapiens*

---

247 Terpenoid backbone biosynthesis

247.1 Human Pathway: HSA00900

247.2 Number of Hits: 10

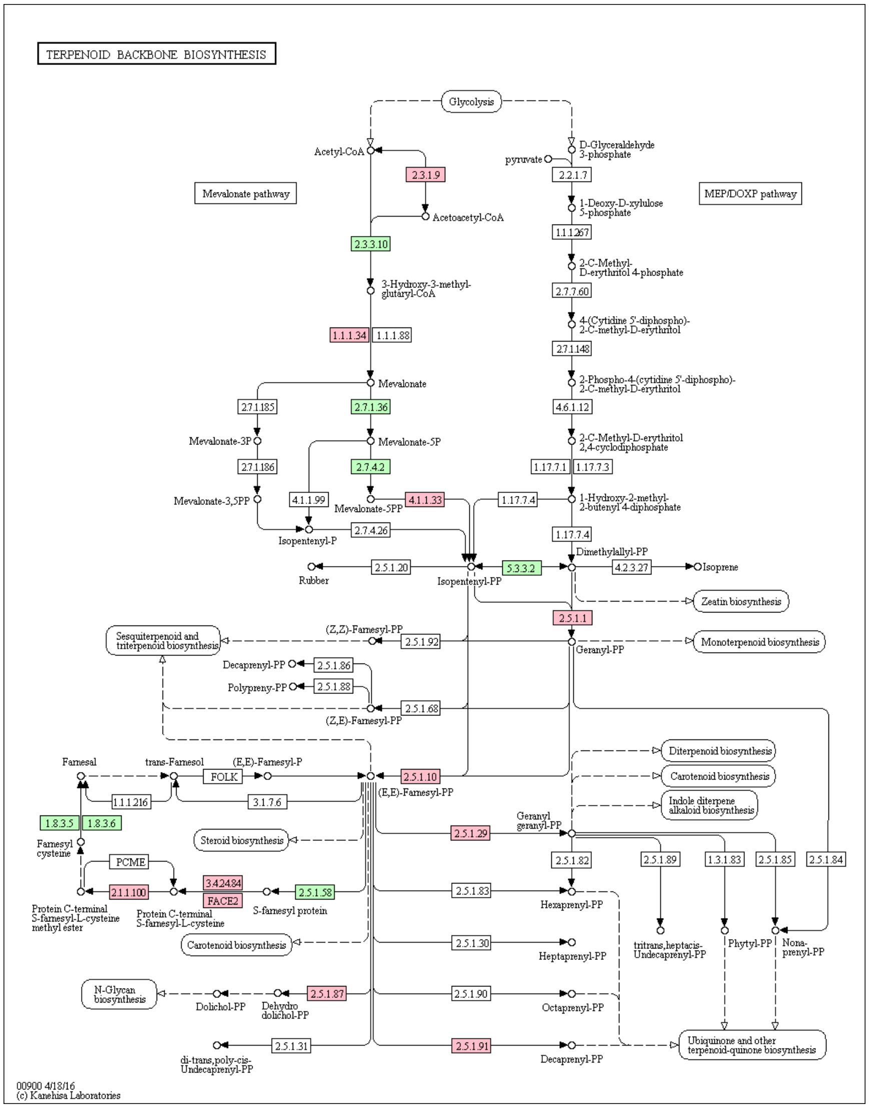

247.3 Legend:

RBH-Blast at 60% Identity + 50% Coverage
Green = Hit in *H. sapiens*
Red = Hit in *H. sapiens* and *C. milli*
White = Not in *H. sapiens*

---

248 Inflammatory bowel disease (IBD)

248.1 Human Pathway: HSA05321

248.2 Number of Hits: 10

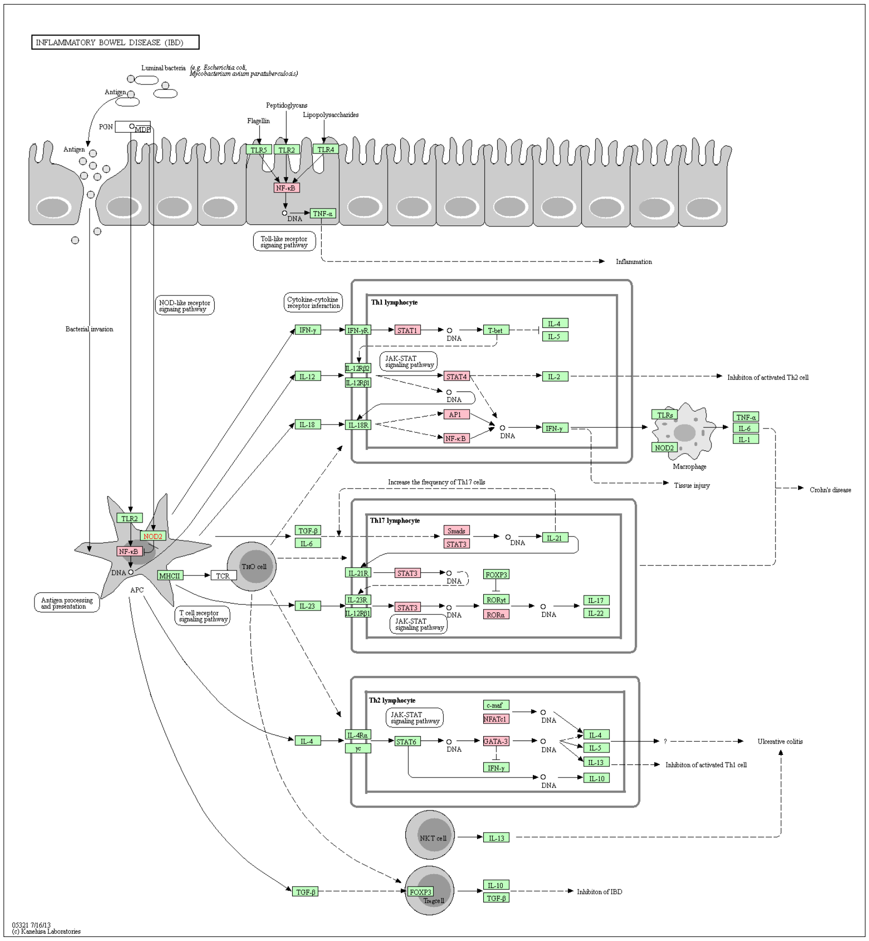

248.3 Legend:

RBH-Blast at 60% Identity + 50% Coverage
Green = Hit in *H. sapiens*
Red = Hit in *H. sapiens* and *C. milli*
White = Not in *H. sapiens*

---

249 Viral myocarditis

249.1 Human Pathway: HSA05416

249.2 Number of Hits: 9

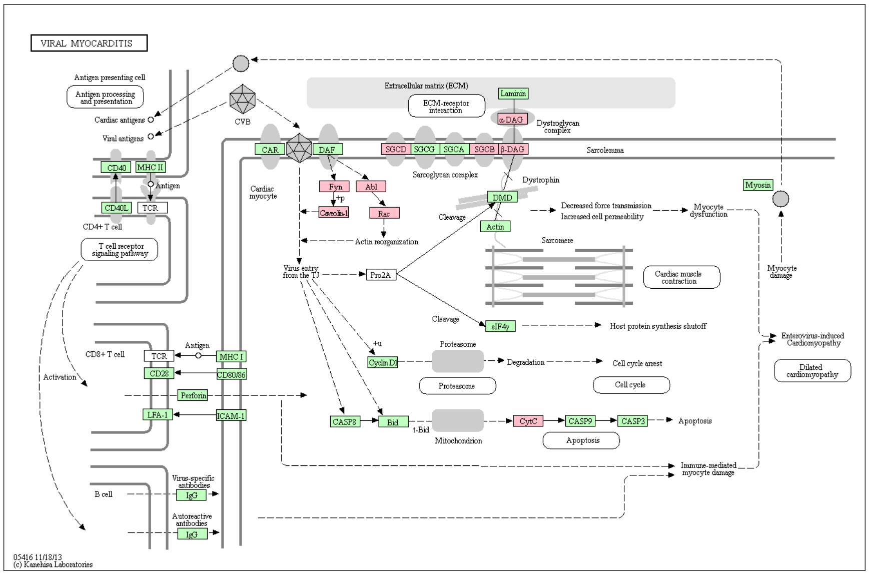

249.3 Legend:

RBH-Blast at 60% Identity + 50% Coverage
Green = Hit in *H. sapiens*
Red = Hit in *H. sapiens* and *C. milli*
White = Not in *H. sapiens*

---

250 2-Oxocarboxylic acid metabolism

250.1 Human Pathway: HSA01210

250.2 Number of Hits: 9

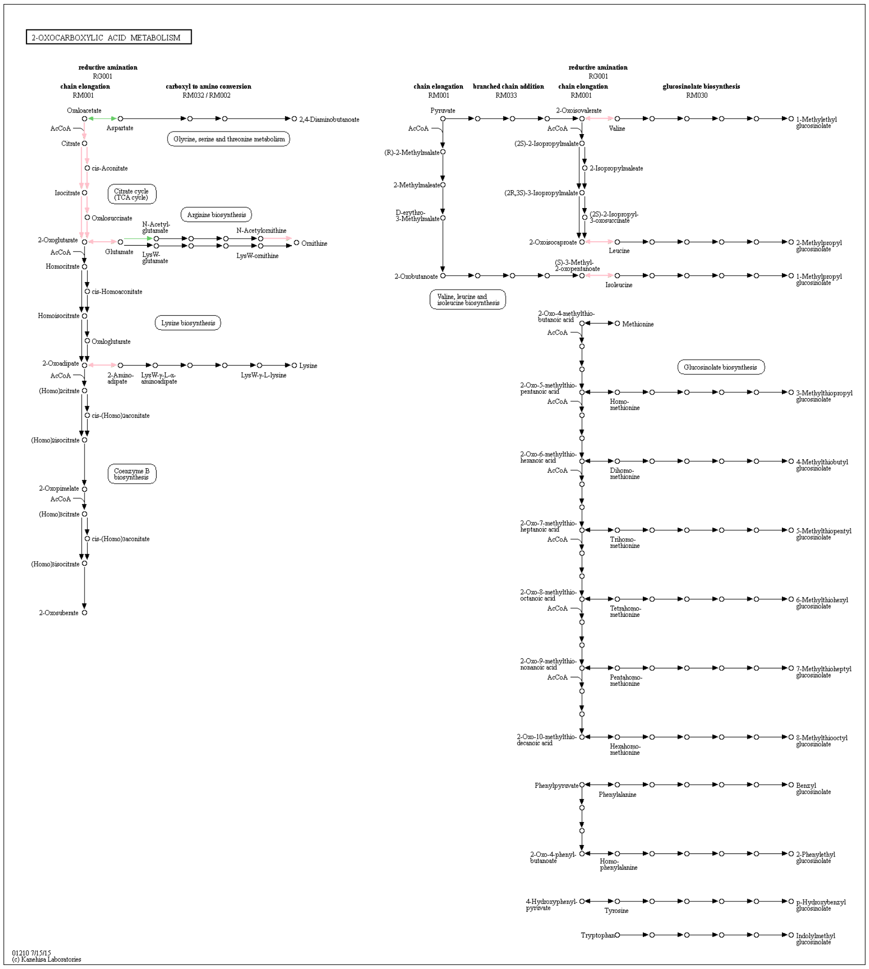

250.3 Legend:

RBH-Blast at 60% Identity + 50% Coverage
Green = Hit in *H. sapiens*
Red = Hit in *H. sapiens* and *C. milli*
White = Not in *H. sapiens*

251 Metabolism of xenobiotics by cytochrome P450

251.1 Human Pathway: HSA00980

251.2 Number of Hits: 9

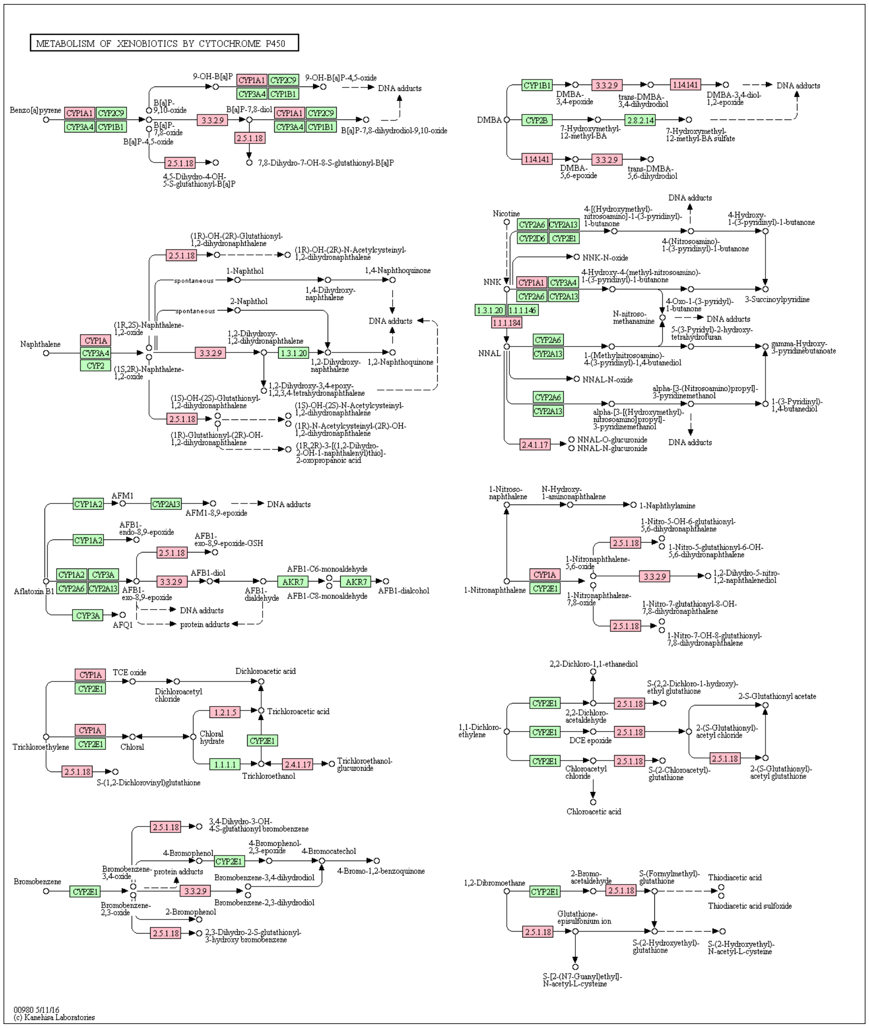

251.3 Legend:

RBH-Blast at 60% Identity + 50% Coverage
Green = Hit in *H. sapiens*
Red = Hit in *H. sapiens* and *C. milli*
White = Not in *H. sapiens*

252 Selenocompound metabolism

252.1 Human Pathway: HSA00450

252.2 Number of Hits: 9

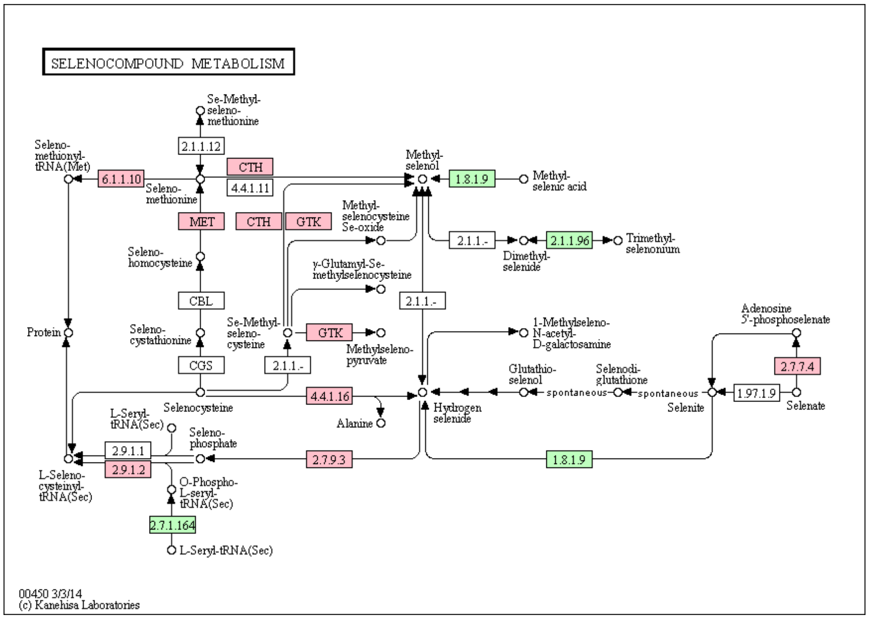

252.3 Legend:

RBH-Blast at 60% Identity + 50% Coverage
Green = Hit in *H. sapiens*
Red = Hit in *H. sapiens* and *C. milli*
White = Not in *H. sapiens*

253 Pantothenate and CoA biosynthesis

253.1 Human Pathway: HSA00770

253.2 Number of Hits: 9

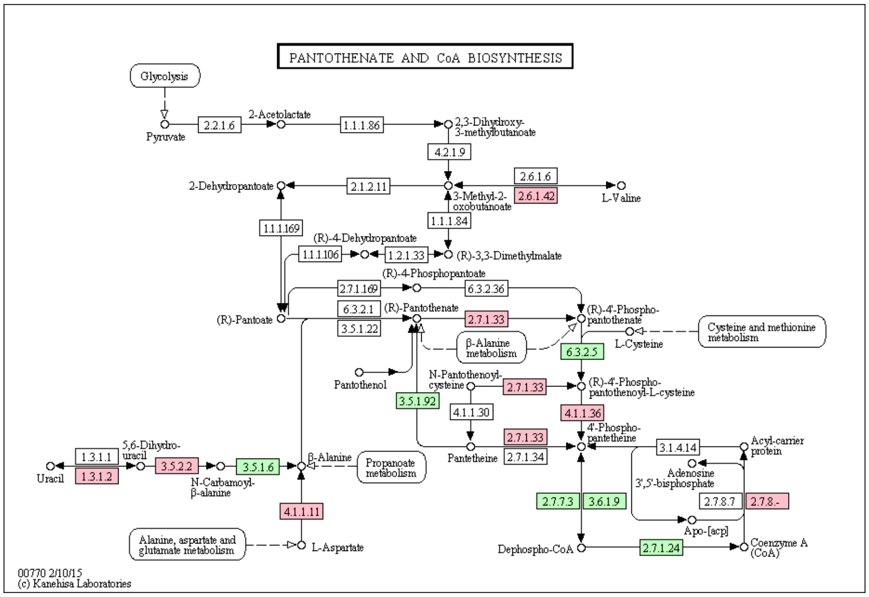

253.3 Legend:

RBH-Blast at 60% Identity + 50% Coverage
Green = Hit in *H. sapiens*
Red = Hit in *H. sapiens* and *C. milli*
White = Not in *H. sapiens*

254 Mismatch repair

254.1 Human Pathway: HSA03430

254.2 Number of Hits: 9

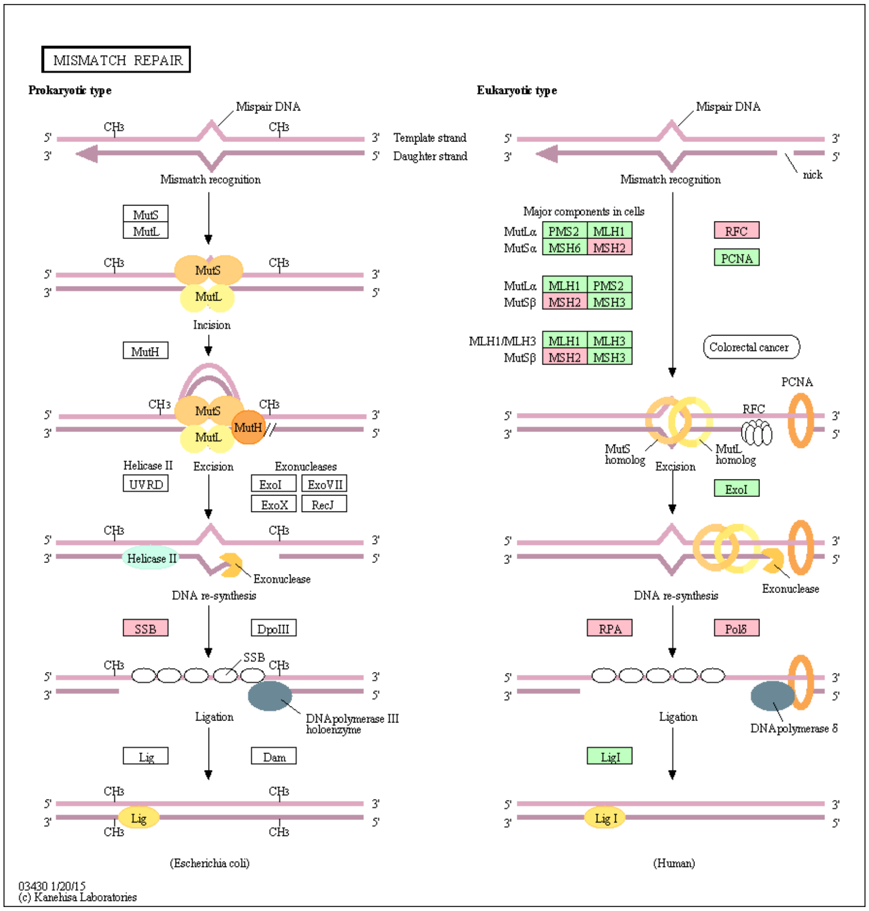

254.3 Legend:

RBH-Blast at 60% Identity + 50% Coverage
Green = Hit in *H. sapiens*
Red = Hit in *H. sapiens* and *C. milli*
White = Not in *H. sapiens*

255 Glycosphingolipid biosynthesis - lacto and neolacto series

255.1 Human Pathway: HSA00601

255.2 Number of Hits: 9

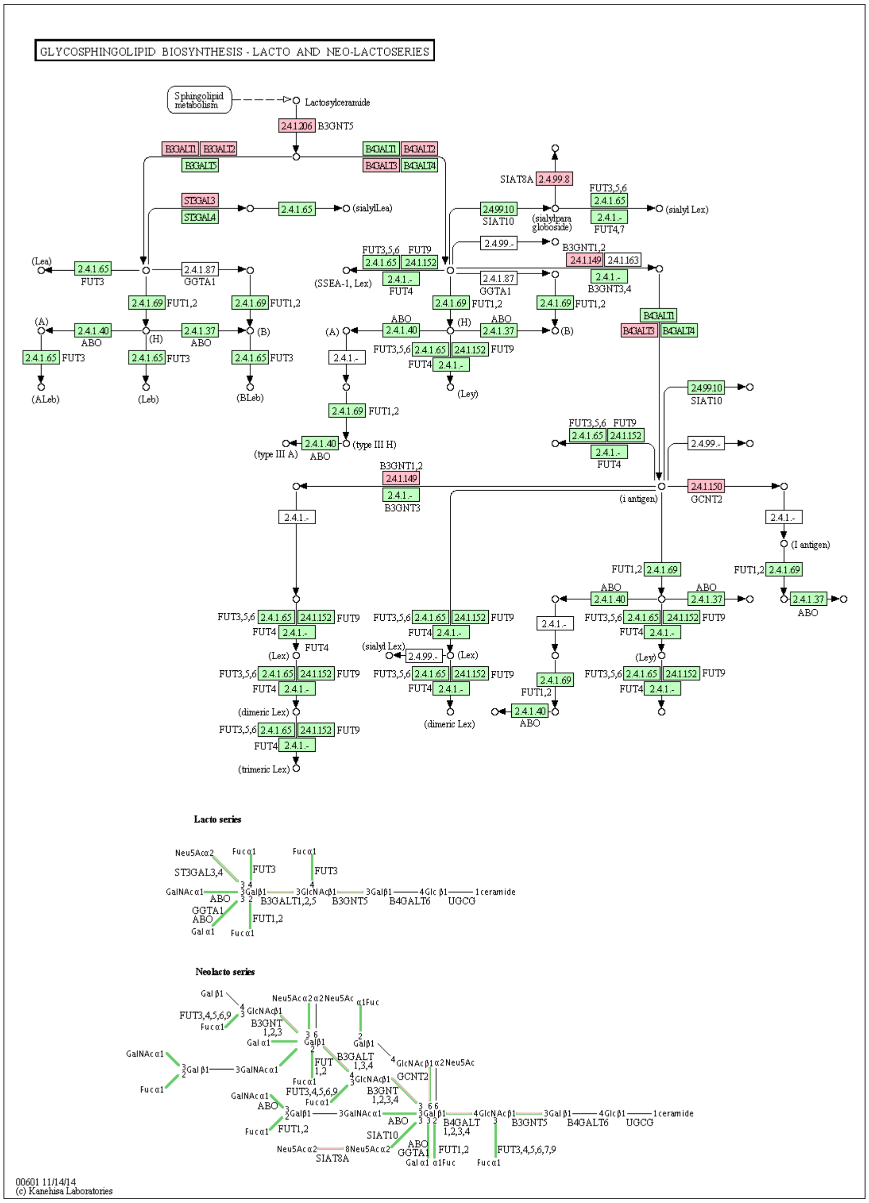

255.3 Legend:

RBH-Blast at 60% Identity + 50% Coverage
Green = Hit in *H. sapiens*
Red = Hit in *H. sapiens* and *C. milli*
White = Not in *H. sapiens*

256 Fatty acid biosynthesis

256.1 Human Pathway: HSA00061

256.2 Number of Hits: 8

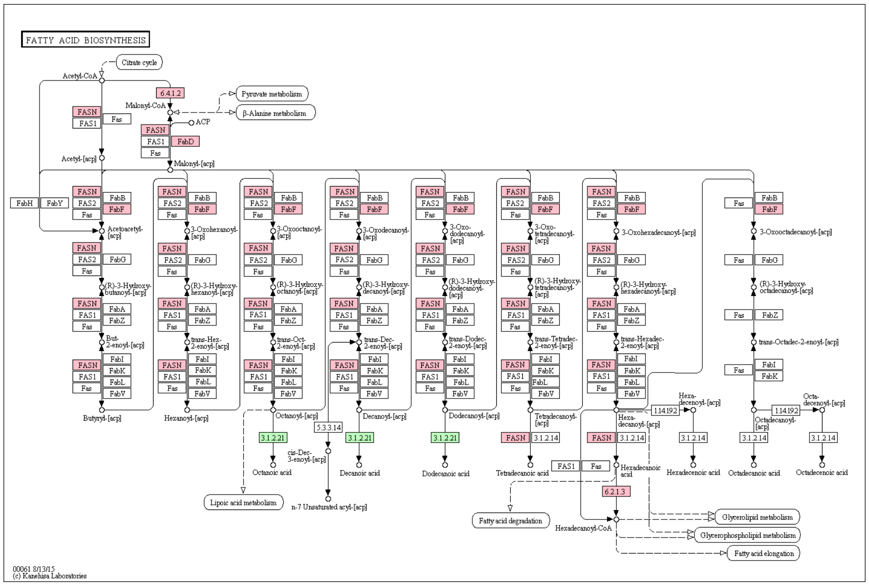

256.3 Legend:

RBH-Blast at 60% Identity + 50% Coverage
Green = Hit in *H. sapiens*
Red = Hit in *H. sapiens* and *C. milli*
White = Not in *H. sapiens*

257 Drug metabolism - cytochrome P450

257.1 Human Pathway: HSA00982

257.2 Number of Hits: 8

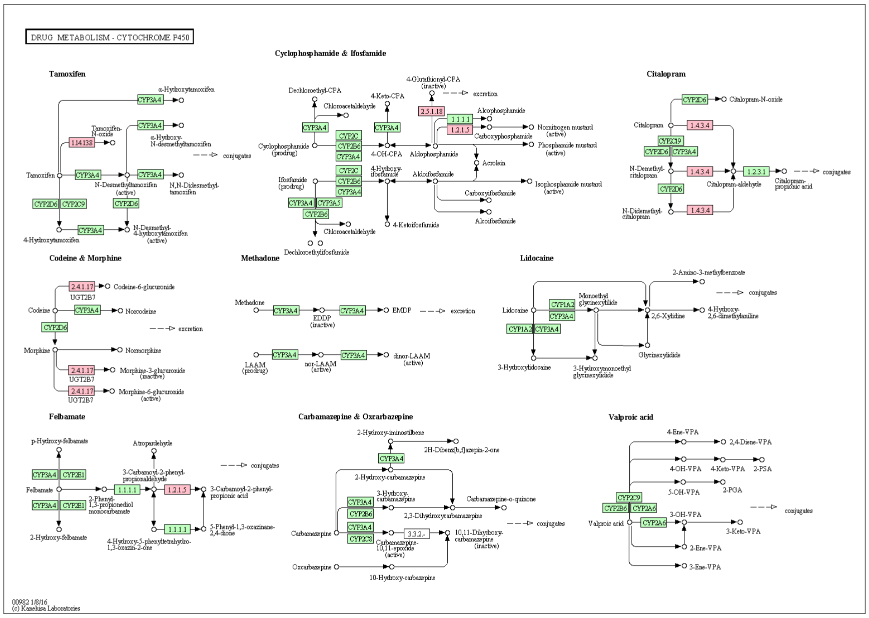

257.3 Legend:

RBH-Blast at 60% Identity + 50% Coverage
Green = Hit in *H. sapiens*
Red = Hit in *H. sapiens* and *C. milli*
White = Not in *H. sapiens*

258 Steroid hormone biosynthesis

258.1 Human Pathway: HSA00140

258.2 Number of Hits: 8

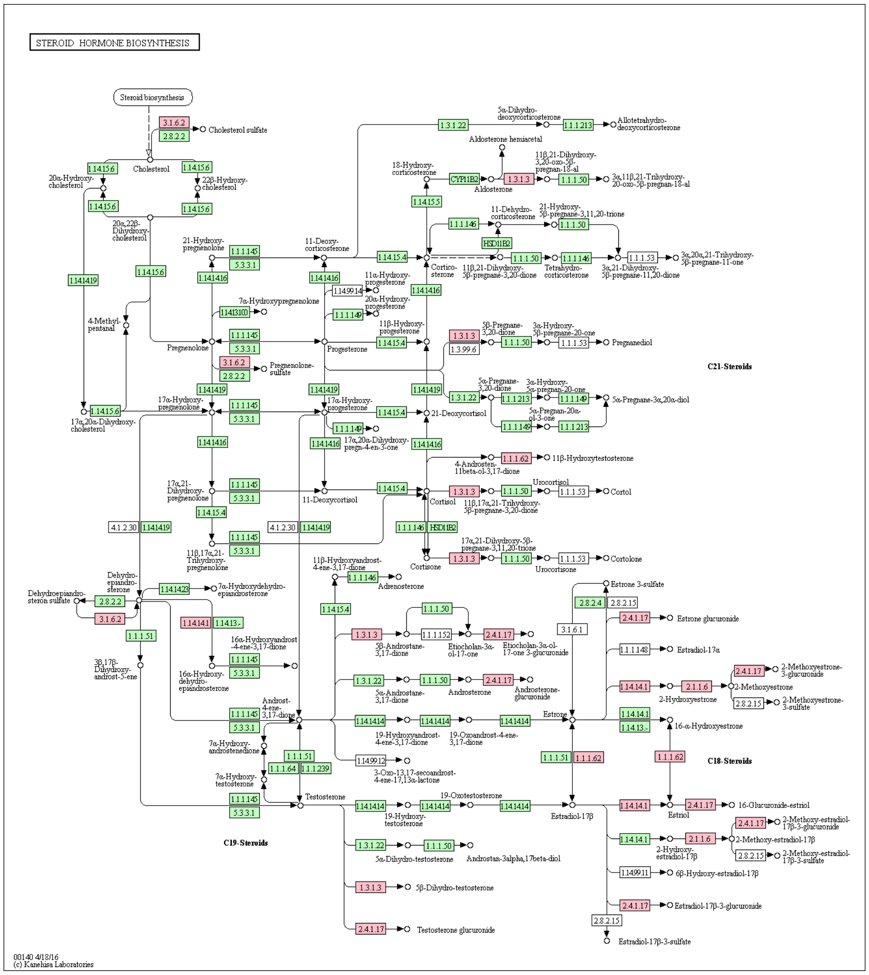

258.3 Legend:

RBH-Blast at 60% Identity + 50% Coverage
Green = Hit in *H. sapiens*
Red = Hit in *H. sapiens* and *C. milli*
White = Not in *H. sapiens*

259 Pentose and glucuronate interconversions

259.1 Human Pathway: HSA00040

259.2 Number of Hits: 7

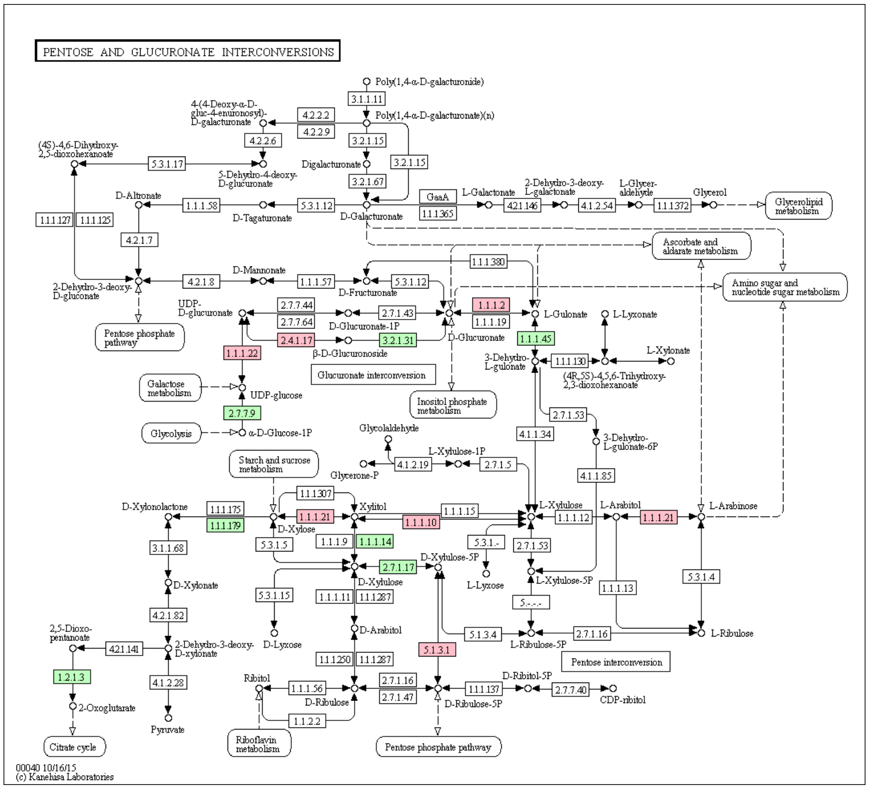

259.3 Legend:

RBH-Blast at 60% Identity + 50% Coverage
Green = Hit in *H. sapiens*
Red = Hit in *H. sapiens* and *C. milli*
White = Not in *H. sapiens*

260 Ascorbate and aldarate metabolism

260.1 Human Pathway: HSA00053

260.2 Number of Hits: 7

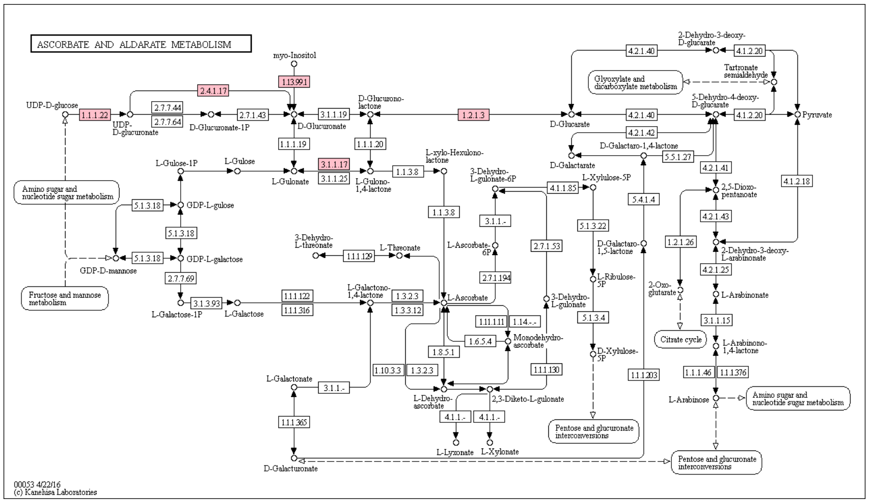

260.3 Legend:

RBH-Blast at 60% Identity + 50% Coverage
Green = Hit in *H. sapiens*
Red = Hit in *H. sapiens* and *C. milli*
White = Not in *H. sapiens*

---

261 Glycosphingolipid biosynthesis - ganglio series

261.1 Human Pathway: HSA00604

261.2 Number of Hits: 7

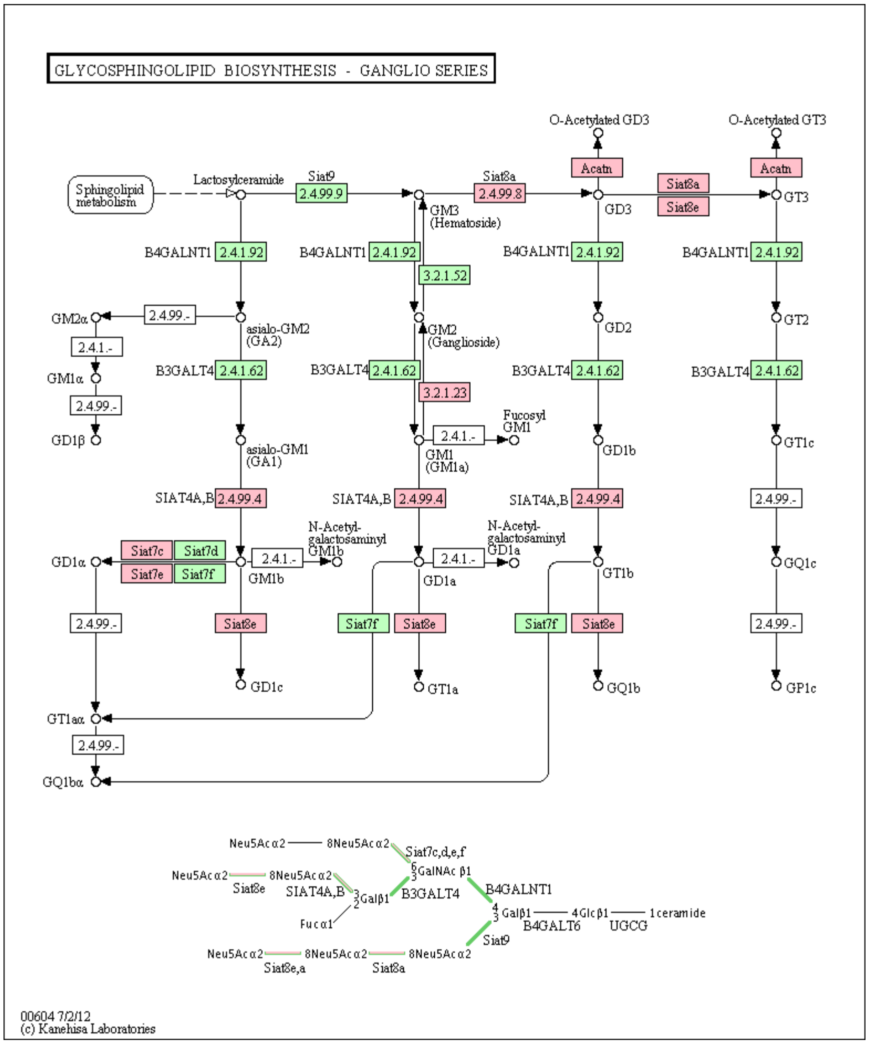

261.3 Legend:

RBH-Blast at 60% Identity + 50% Coverage
Green = Hit in *H. sapiens*
Red = Hit in *H. sapiens* and *C. milli*
White = Not in *H. sapiens*

---

262 African trypanosomiasis

262.1 Human Pathway: HSA05143

262.2 Number of Hits: 7

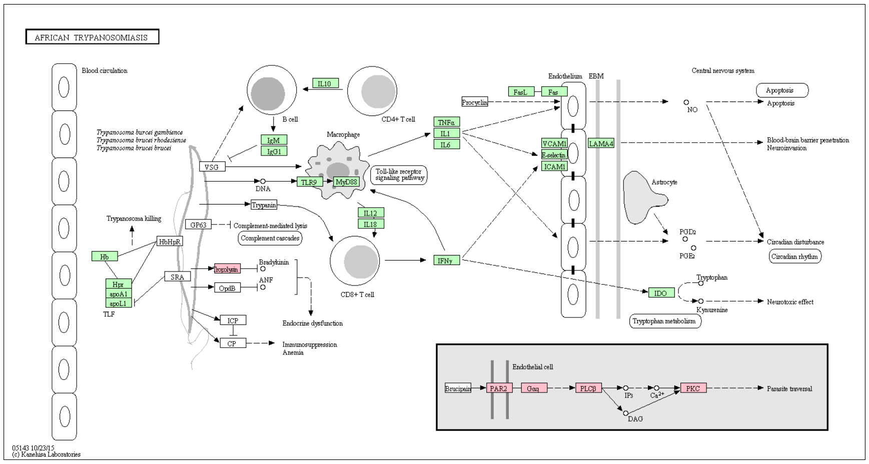

262.3 Legend:

RBH-Blast at 60% Identity + 50% Coverage
Green = Hit in *H. sapiens*
Red = Hit in *H. sapiens* and *C. milli*
White = Not in *H. sapiens*

---

263 Phenylalanine metabolism

263.1 Human Pathway: HSA00360

263.2 Number of Hits: 6

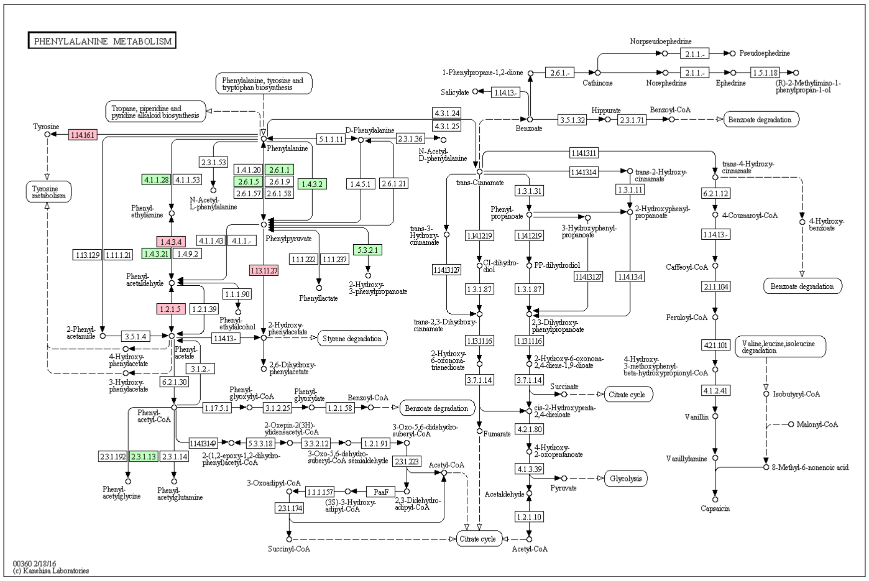

263.3 Legend:

RBH-Blast at 60% Identity + 50% Coverage
Green = Hit in *H. sapiens*
Red = Hit in *H. sapiens* and *C. milli*
White = Not in *H. sapiens*

264 Renin-angiotensin system

264.1 Human Pathway: HSA04614

264.2 Number of Hits: 6

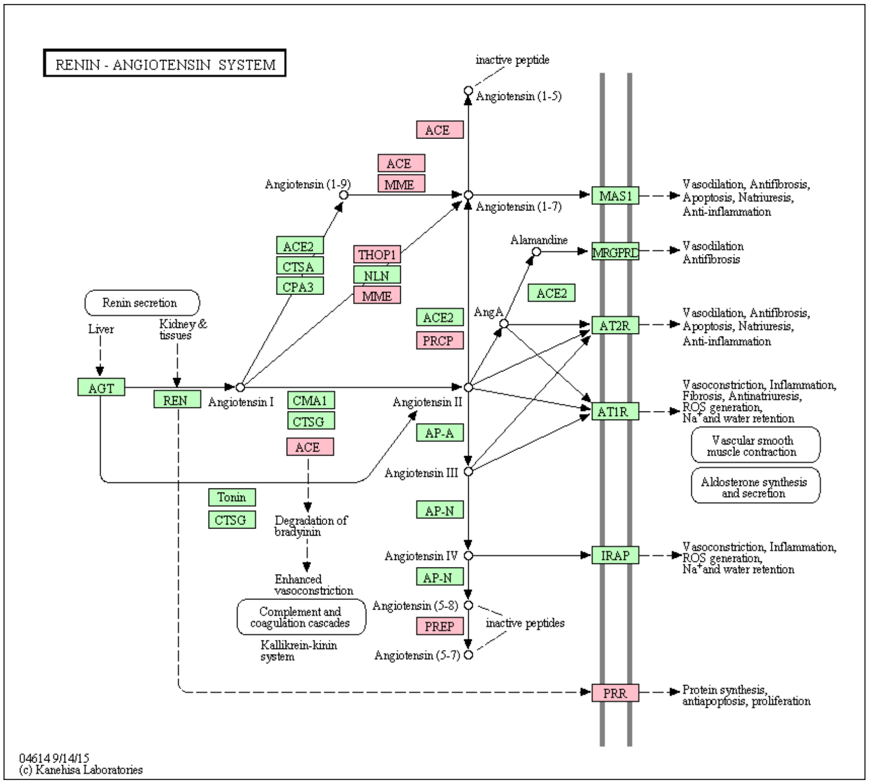

264.3 Legend:

RBH-Blast at 60% Identity + 50% Coverage
Green = Hit in *H. sapiens*
Red = Hit in *H. sapiens* and *C. milli*
White = Not in *H. sapiens*

---

265 Vitamin digestion and absorption

265.1 Human Pathway: HSA04977

265.2 Number of Hits: 6

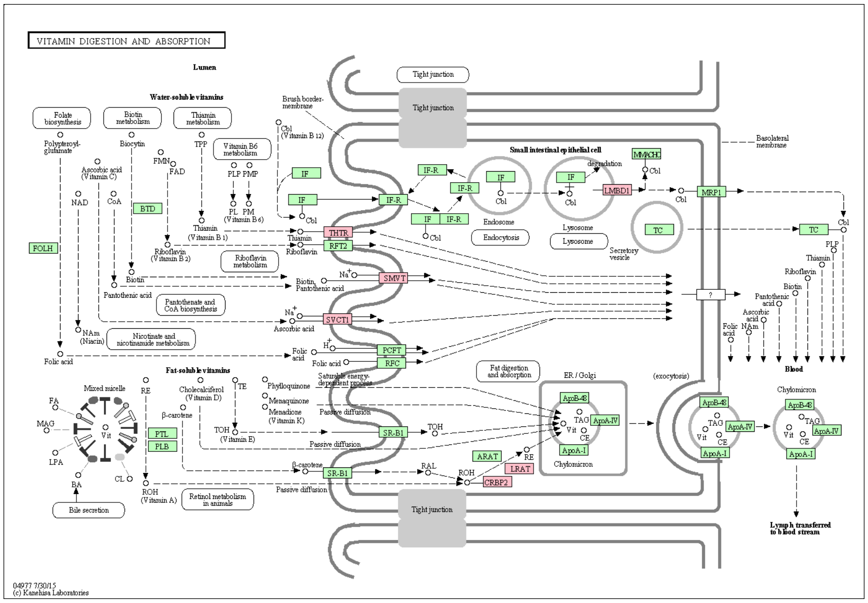

265.3 Legend:

RBH-Blast at 60% Identity + 50% Coverage
Green = Hit in *H. sapiens*
Red = Hit in *H. sapiens* and *C. milli*
White = Not in *H. sapiens*

266 Glycosaminoglycan degradation

266.1 Human Pathway: HSA00531

266.2 Number of Hits: 6

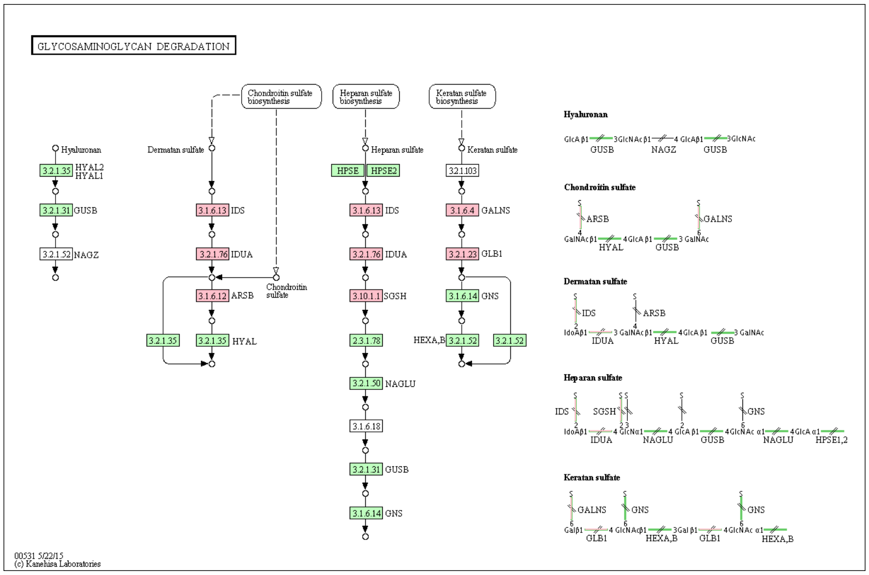

266.3 Legend:

RBH-Blast at 60% Identity + 50% Coverage
Green = Hit in *H. sapiens*
Red = Hit in *H. sapiens* and *C. milli*
White = Not in *H. sapiens*

267 Folate biosynthesis

267.1 Human Pathway: HSA00790

267.2 Number of Hits: 6

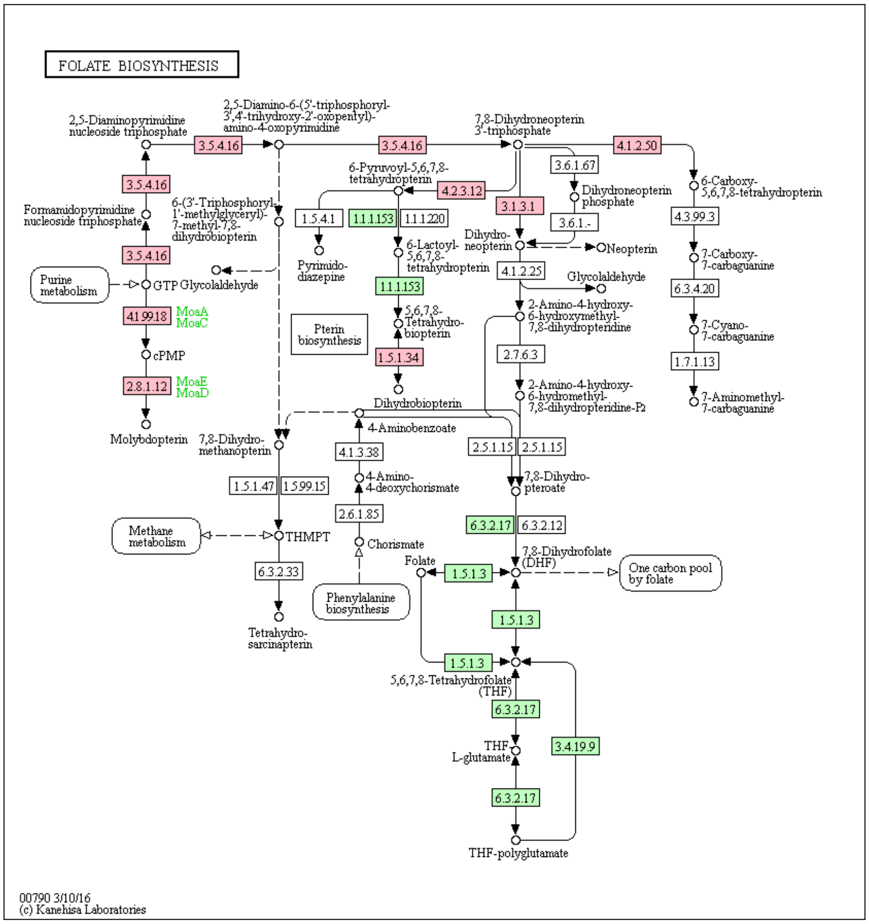

267.3 Legend:

RBH-Blast at 60% Identity + 50% Coverage
Green = Hit in *H. sapiens*
Red = Hit in *H. sapiens* and *C. milli*
White = Not in *H. sapiens*

268 Primary immunodeficiency

268.1 Human Pathway: HSA05340

268.2 Number of Hits: 6

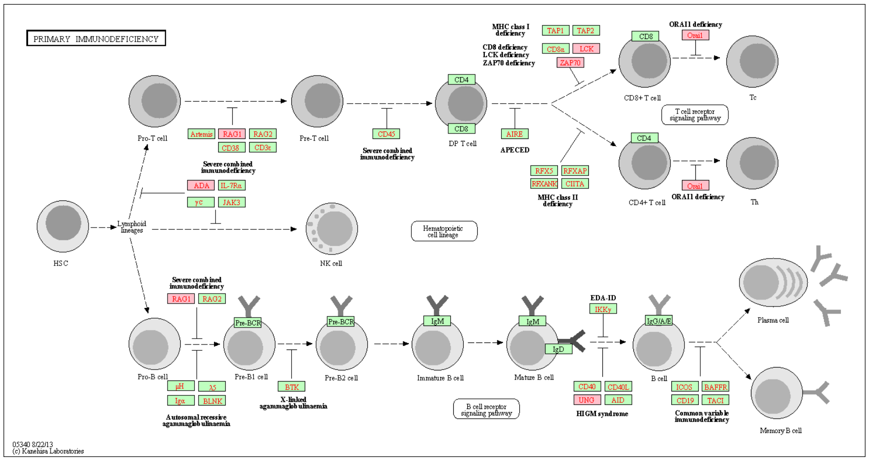

268.3 Legend:

RBH-Blast at 60% Identity + 50% Coverage
Green = Hit in *H. sapiens*
Red = Hit in *H. sapiens* and *C. milli*
White = Not in *H. sapiens*

269 Nitrogen metabolism

269.1 Human Pathway: HSA00910

269.2 Number of Hits: 6

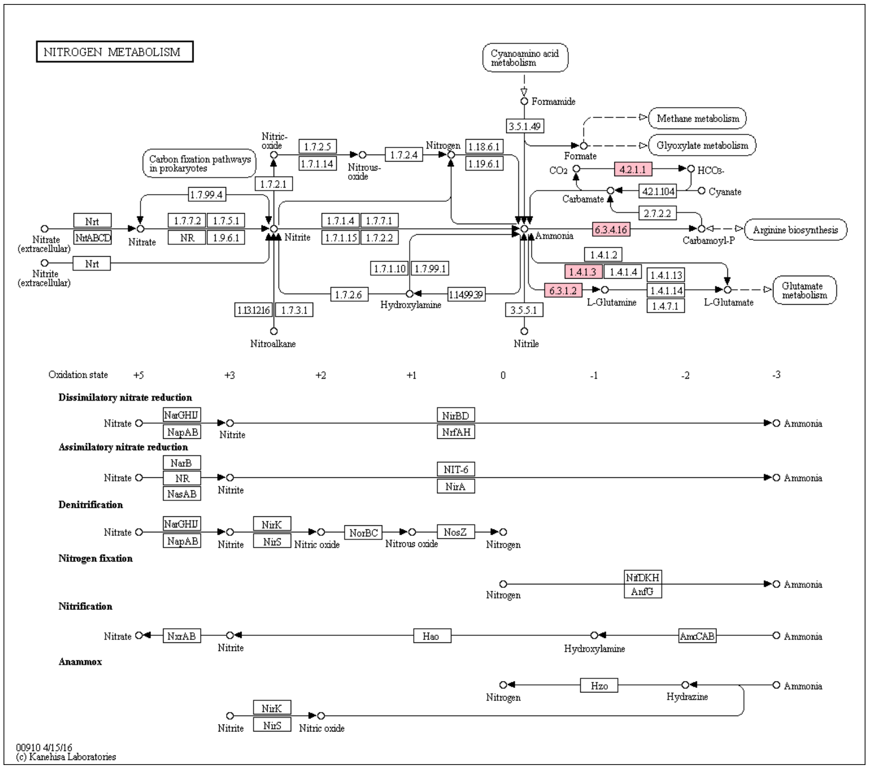

269.3 Legend:

RBH-Blast at 60% Identity + 50% Coverage
Green = Hit in *H. sapiens*
Red = Hit in *H. sapiens* and *C. milli*
White = Not in *H. sapiens*

270 Glycosaminoglycan biosynthesis - keratan sulfate

270.1 Human Pathway: HSA00533

270.2 Number of Hits: 5

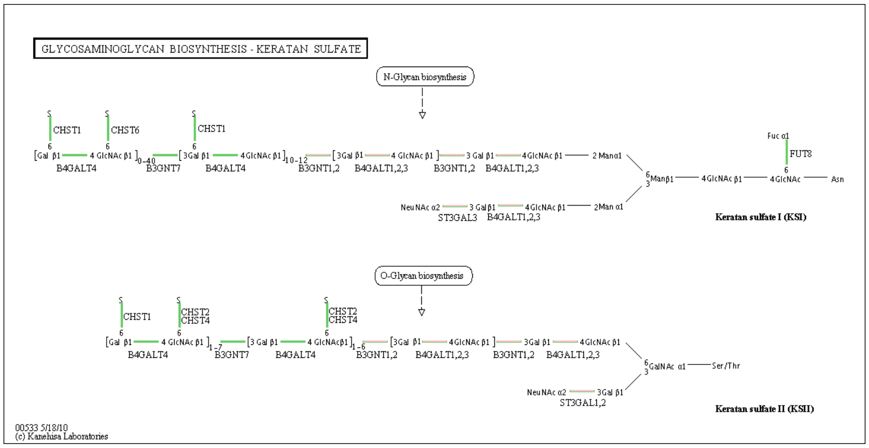

270.3 Legend:

RBH-Blast at 60% Identity + 50% Coverage
Green = Hit in *H. sapiens*
Red = Hit in *H. sapiens* and *C. milli*
White = Not in *H. sapiens*

271 Type I diabetes mellitus

271.1 Human Pathway: HSA04940

271.2 Number of Hits: 5

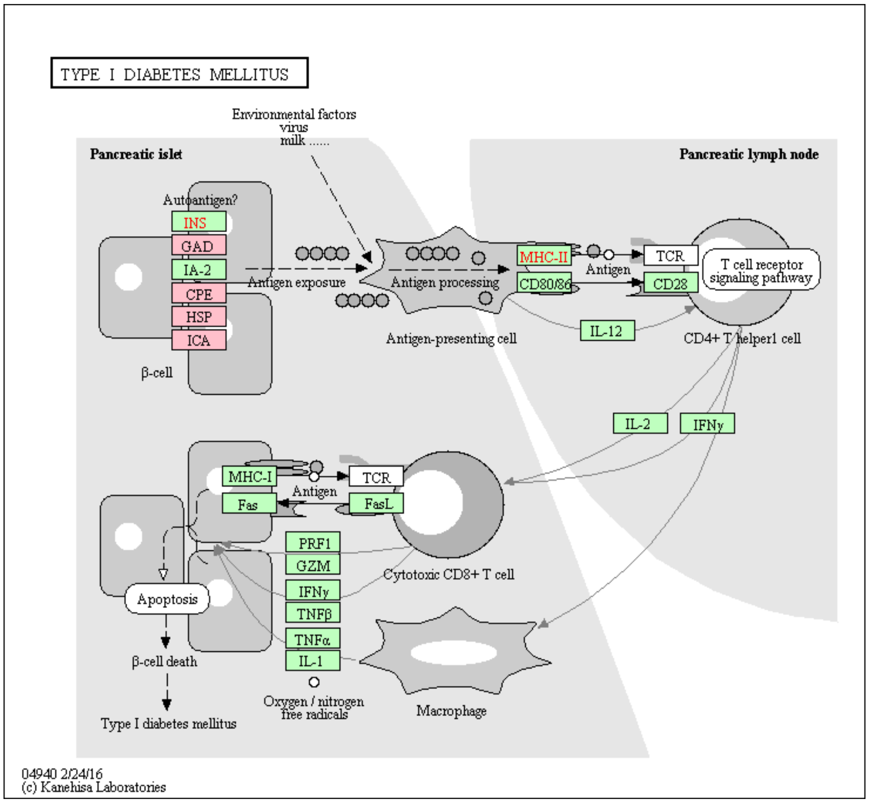

271.3 Legend:

RBH-Blast at 60% Identity + 50% Coverage
Green = Hit in *H. sapiens*
Red = Hit in *H. sapiens* and *C. milli*
White = Not in *H. sapiens*

272 Other glycan degradation

272.1 Human Pathway: HSA00511

272.2 Number of Hits: 5

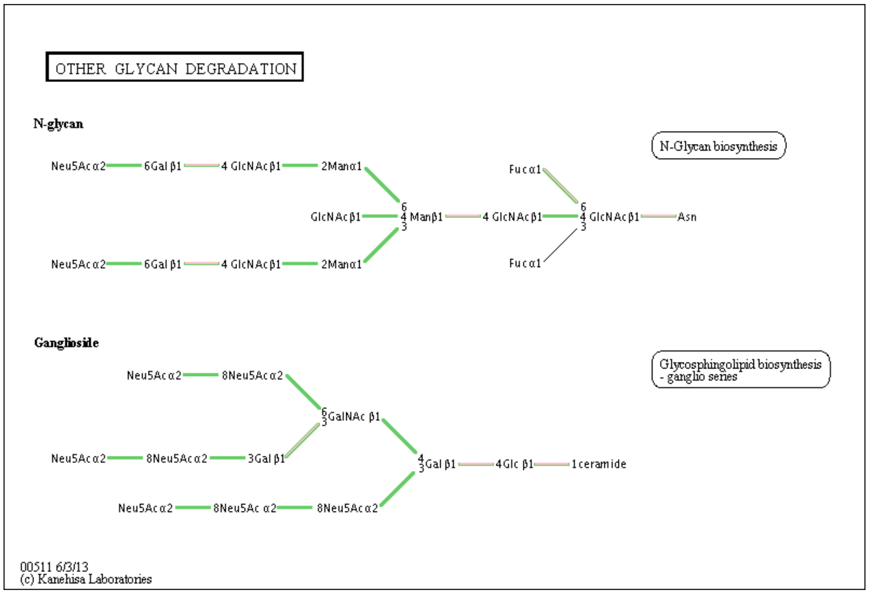

272.3 Legend:

RBH-Blast at 60% Identity + 50% Coverage
Green = Hit in *H. sapiens*
Red = Hit in *H. sapiens* and *C. milli*
White = Not in *H. sapiens*

273 Sulfur relay system

273.1 Human Pathway: HSA04122

273.2 Number of Hits: 5

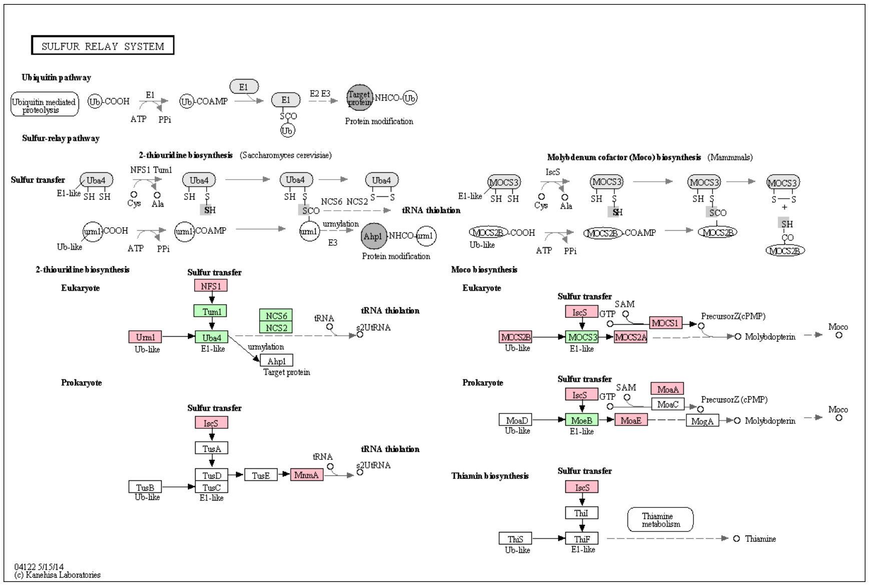

273.3 Legend:

RBH-Blast at 60% Identity + 50% Coverage
Green = Hit in *H. sapiens*
Red = Hit in *H. sapiens* and *C. milli*
White = Not in *H. sapiens*

274 Taurine and hypotaurine metabolism

274.1 Human Pathway: HSA00430

274.2 Number of Hits: 5

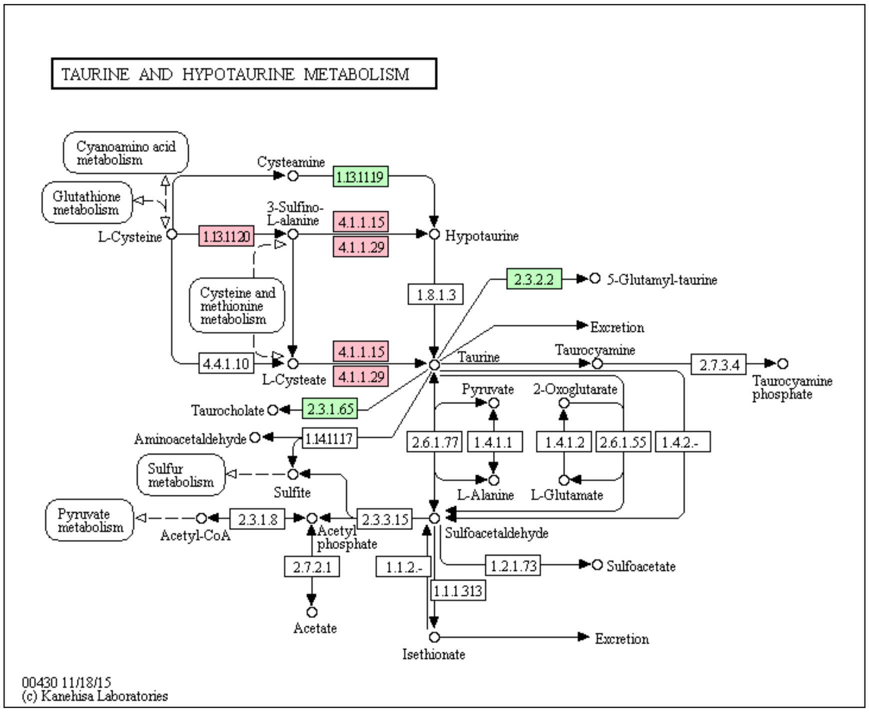

274.3 Legend:

RBH-Blast at 60% Identity + 50% Coverage
Green = Hit in *H. sapiens*
Red = Hit in *H. sapiens* and *C. milli*
White = Not in *H. sapiens*

275 Primary bile acid biosynthesis

275.1 Human Pathway: HSA00120

275.2 Number of Hits: 5

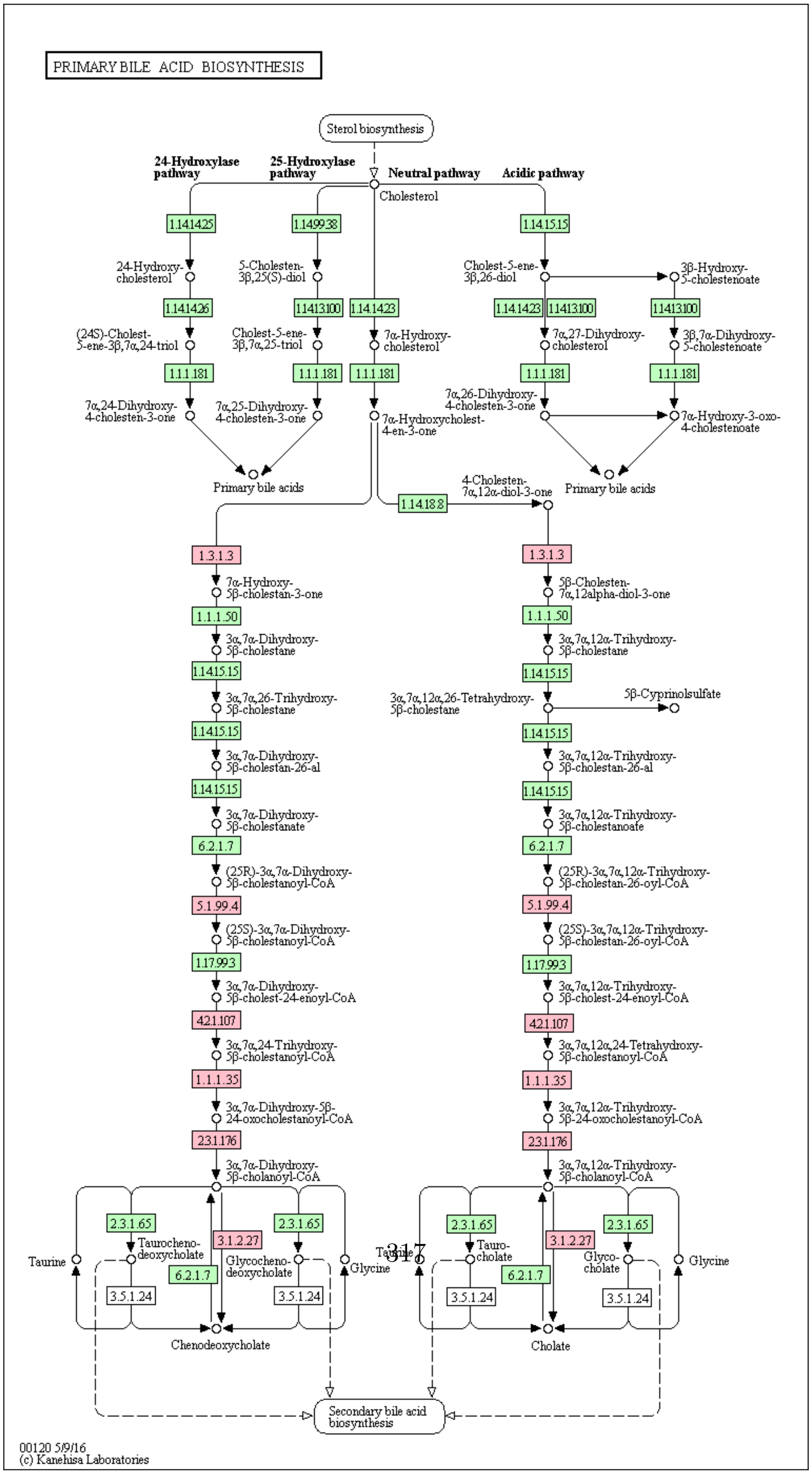

275.3 Legend:

RBH-Blast at 60% Identity + 50% Coverage
Green = Hit in *H. sapiens*
Red = Hit in *H. sapiens* and *C. milli*
White = Not in *H. sapiens*

---

276 Malaria

276.1 Human Pathway: HSA05144

276.2 Number of Hits: 5

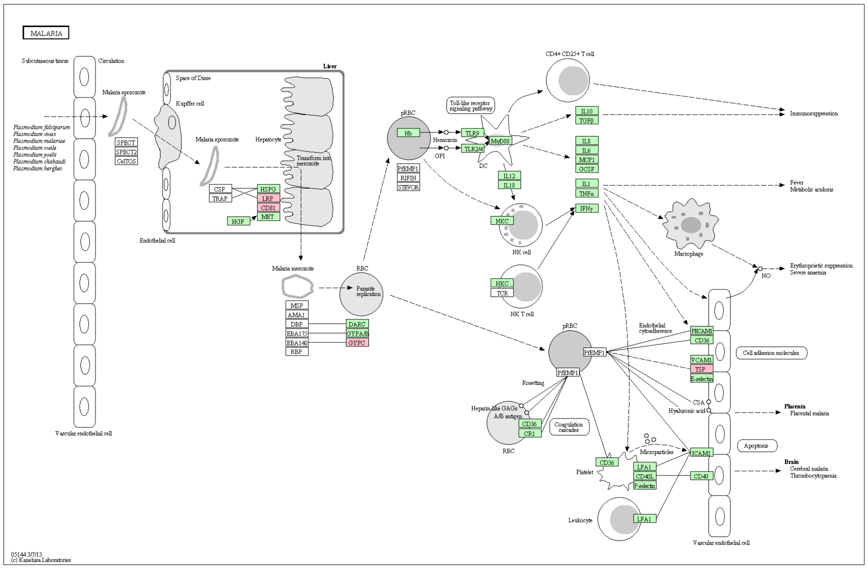

276.3 Legend:

RBH-Blast at 60% Identity + 50% Coverage
Green = Hit in *H. sapiens*
Red = Hit in *H. sapiens* and *C. milli*
White = Not in *H. sapiens*

---

277 alpha-Linolenic acid metabolism

277.1 Human Pathway: HSA00592

277.2 Number of Hits: 4

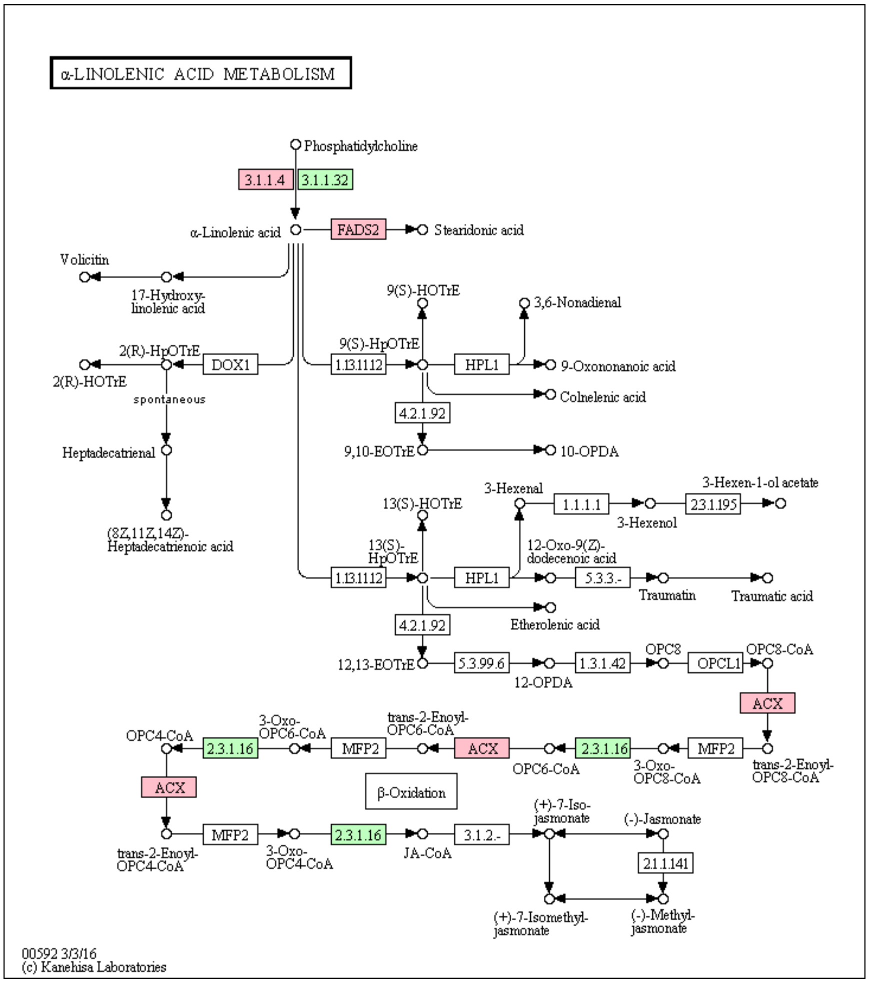

277.3 Legend:

RBH-Blast at 60% Identity + 50% Coverage
Green = Hit in *H. sapiens*
Red = Hit in *H. sapiens* and *C. milli*
White = Not in *H. sapiens*

278 Non-homologous end-joining

278.1 Human Pathway: HSA03450

278.2 Number of Hits: 4

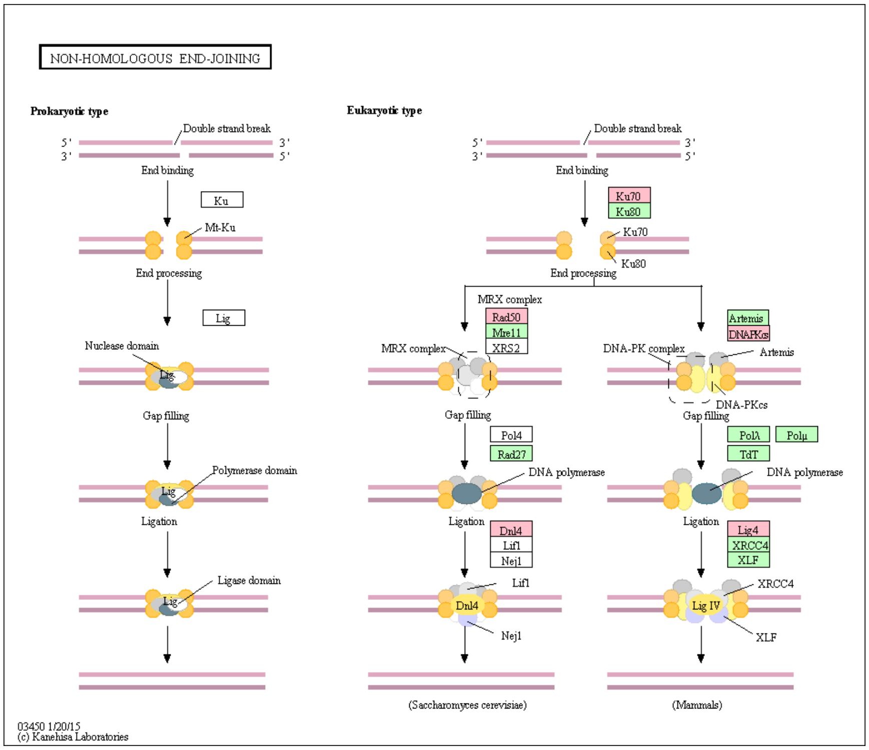

278.3 Legend:

RBH-Blast at 60% Identity + 50% Coverage
Green = Hit in *H. sapiens*
Red = Hit in *H. sapiens* and *C. milli*
White = Not in *H. sapiens*

279 Synthesis and degradation of ketone bodies

279.1 Human Pathway: HSA00072

279.2 Number of Hits: 4

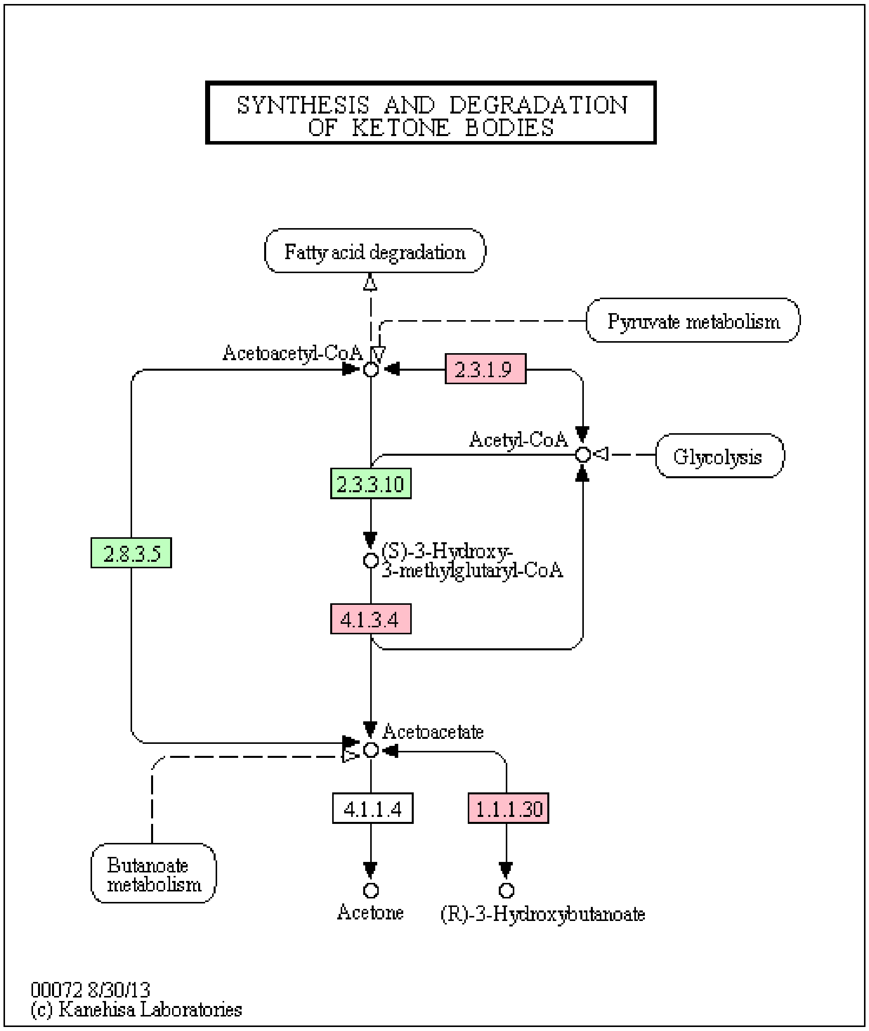

279.3 Legend:

RBH-Blast at 60% Identity + 50% Coverage
Green = Hit in *H. sapiens*
Red = Hit in *H. sapiens* and *C. milli*
White = Not in *H. sapiens*

280 Ubiquinone and other terpenoid-quinone biosynthesis

280.1 Human Pathway: HSA00130

280.2 Number of Hits: 4

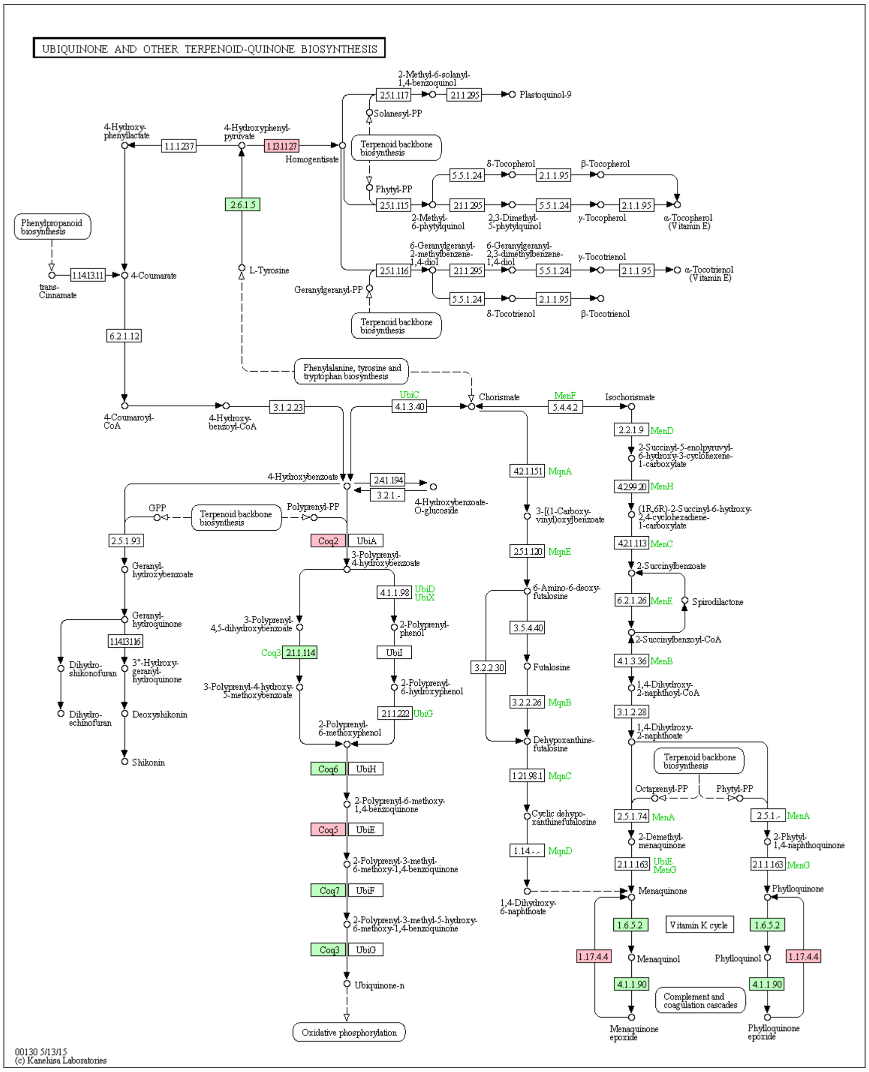

280.3 Legend:

RBH-Blast at 60% Identity + 50% Coverage
Green = Hit in *H. sapiens*
Red = Hit in *H. sapiens* and *C. milli*
White = Not in *H. sapiens*

---

281 Sulfur metabolism

281.1 Human Pathway: HSA00920

281.2 Number of Hits: 4

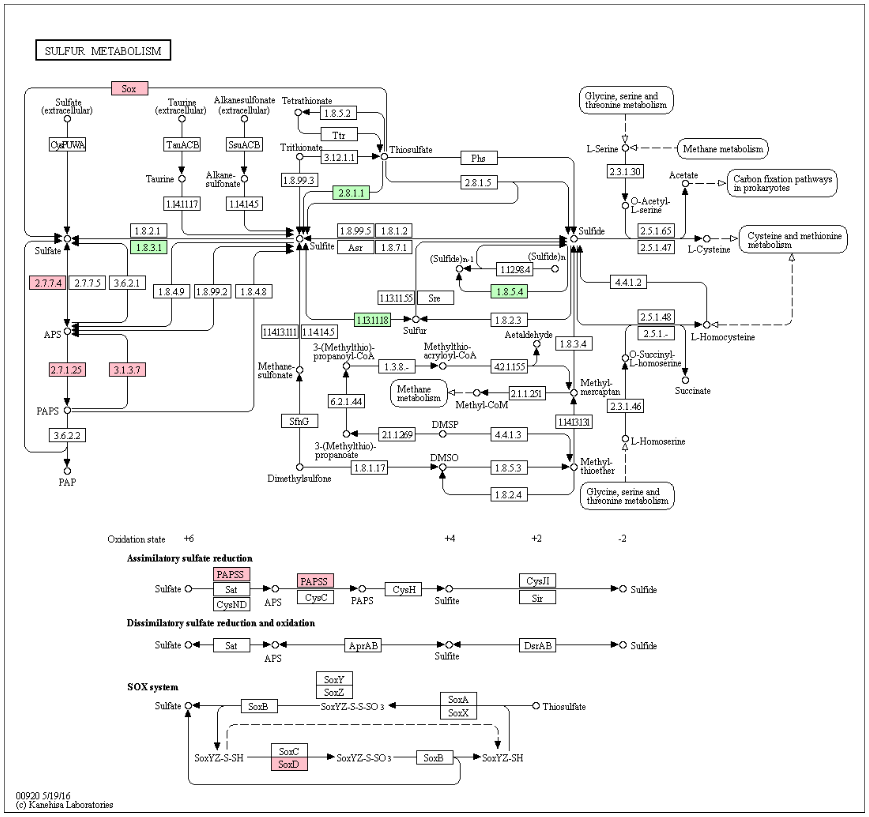

281.3 Legend:

RBH-Blast at 60% Identity + 50% Coverage
Green = Hit in *H. sapiens*
Red = Hit in *H. sapiens* and *C. milli*
White = Not in *H. sapiens*

282 Thiamine metabolism

282.1 Human Pathway: HSA00730

282.2 Number of Hits: 3

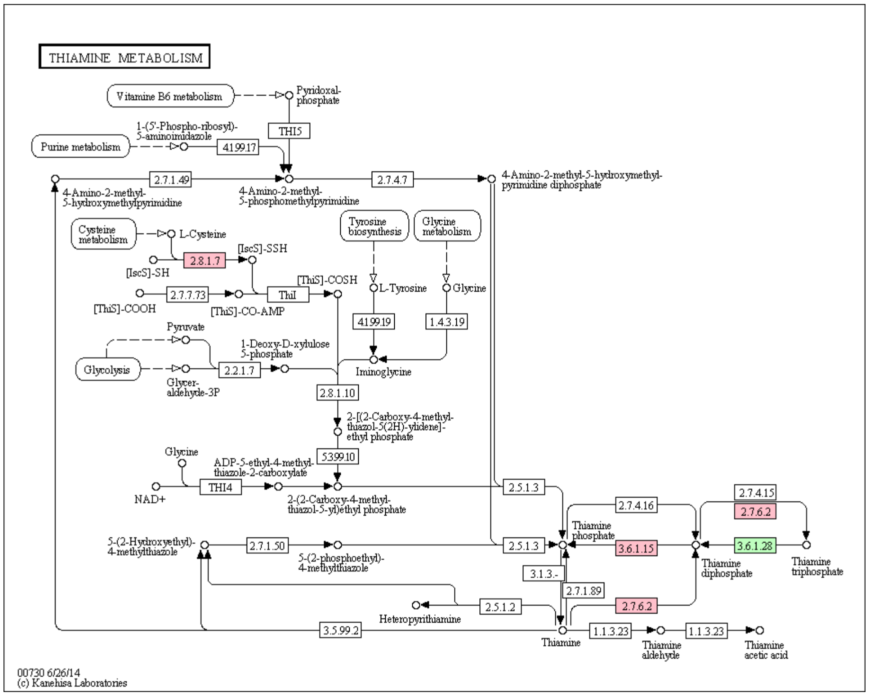

282.3 Legend:

RBH-Blast at 60% Identity + 50% Coverage
Green = Hit in *H. sapiens*
Red = Hit in *H. sapiens* and *C. milli*
White = Not in *H. sapiens*

283 Butirosin and neomycin biosynthesis

283.1 Human Pathway: HSA00524

283.2 Number of Hits: 3

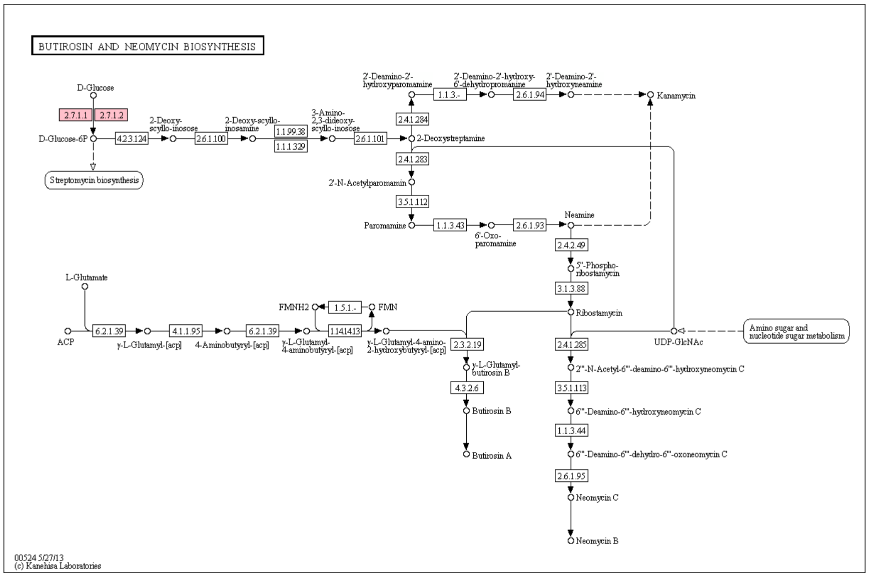

283.3 Legend:

RBH-Blast at 60% Identity + 50% Coverage
Green = Hit in *H. sapiens*
Red = Hit in *H. sapiens* and *C. milli*
White = Not in *H. sapiens*

284 Vitamin B6 metabolism

284.1 Human Pathway: HSA00750

284.2 Number of Hits: 2

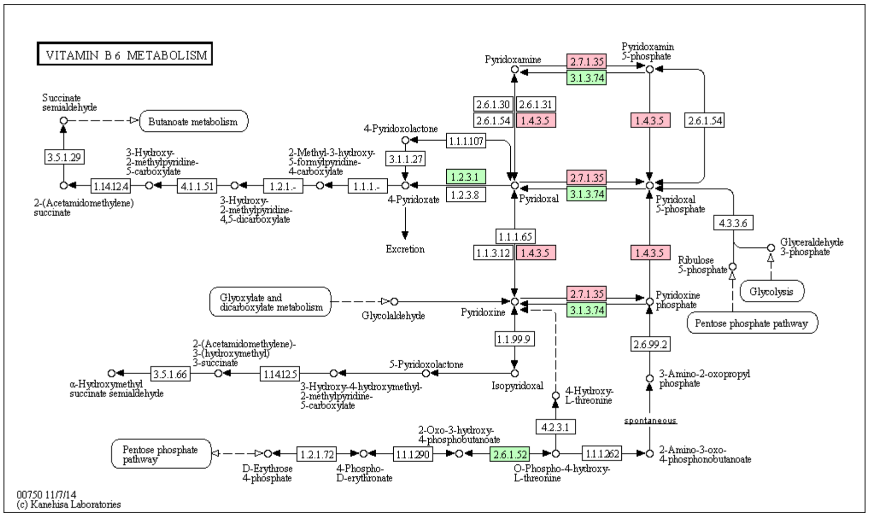

284.3 Legend:

RBH-Blast at 60% Identity + 50% Coverage
Green = Hit in *H. sapiens*
Red = Hit in *H. sapiens* and *C. milli*
White = Not in *H. sapiens*

285 Lysine biosynthesis

285.1 Human Pathway: HSA00300

285.2 Number of Hits: 2

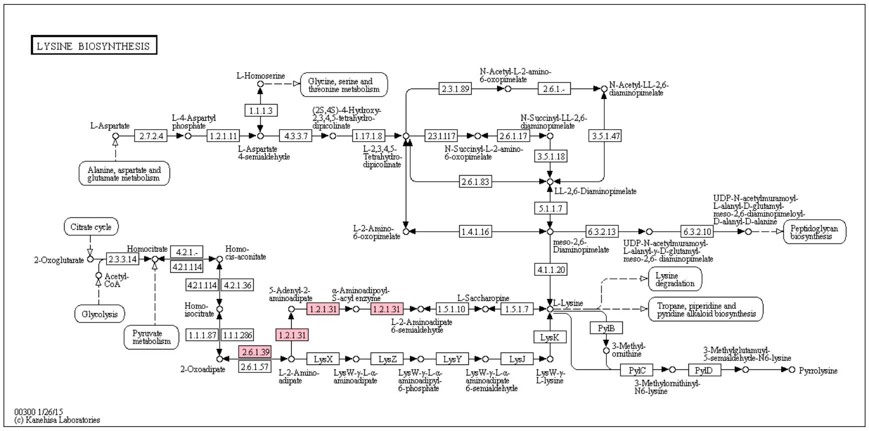

285.3 Legend:

RBH-Blast at 60% Identity + 50% Coverage
Green = Hit in *H. sapiens*
Red = Hit in *H. sapiens* and *C. milli*
White = Not in *H. sapiens*

286 Glycosphingolipid biosynthesis - globo series

286.1 Human Pathway: HSA00603

286.2 Number of Hits: 2

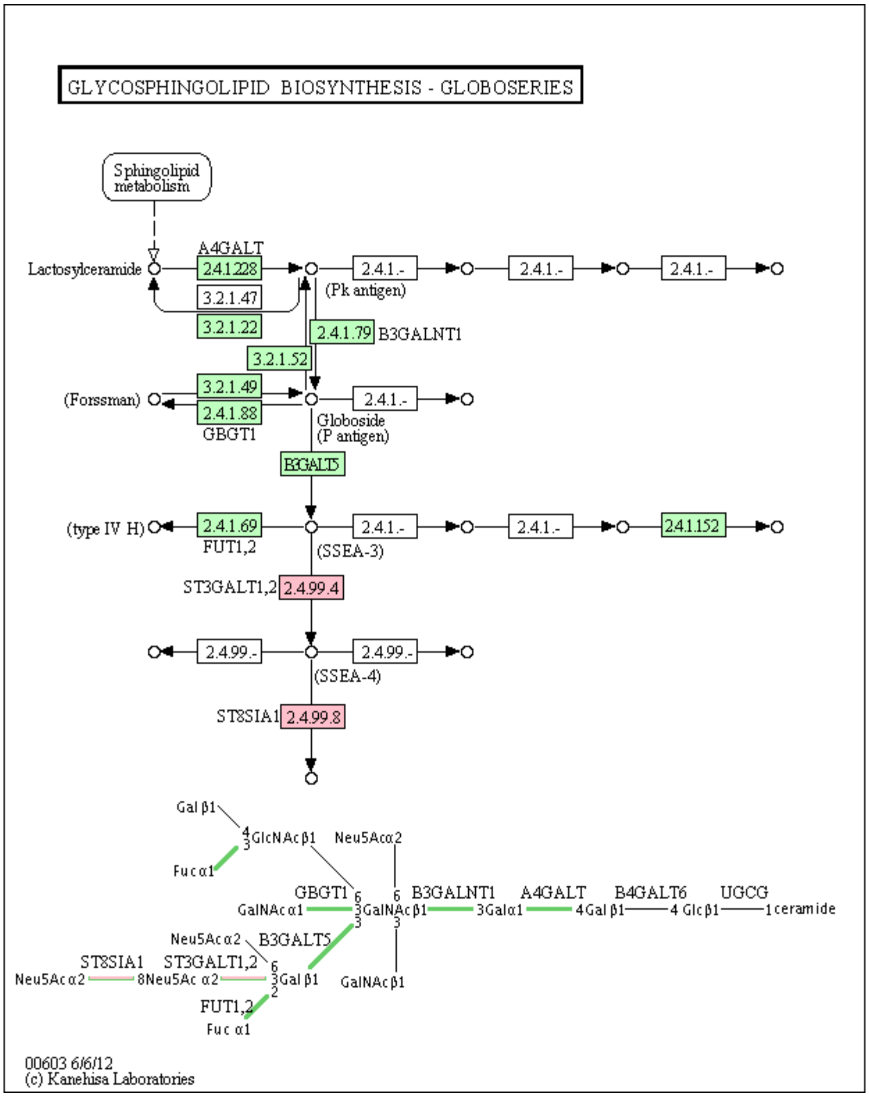

286.3 Legend:

RBH-Blast at 60% Identity + 50% Coverage
Green = Hit in *H. sapiens*
Red = Hit in *H. sapiens* and *C. milli*
White = Not in *H. sapiens*

---

287 Linoleic acid metabolism

287.1 Human Pathway: HSA00591

287.2 Number of Hits: 2

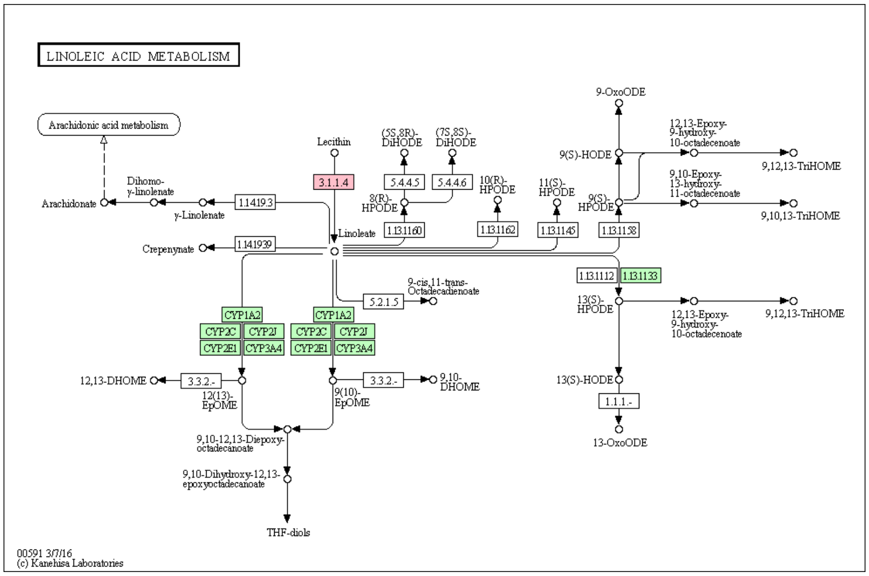

287.3 Legend:

RBH-Blast at 60% Identity + 50% Coverage
Green = Hit in *H. sapiens*
Red = Hit in *H. sapiens* and *C. milli*
White = Not in *H. sapiens*

---

288 D-Glutamine and D-glutamate metabolism

288.1 Human Pathway: HSA00471

288.2 Number of Hits: 2

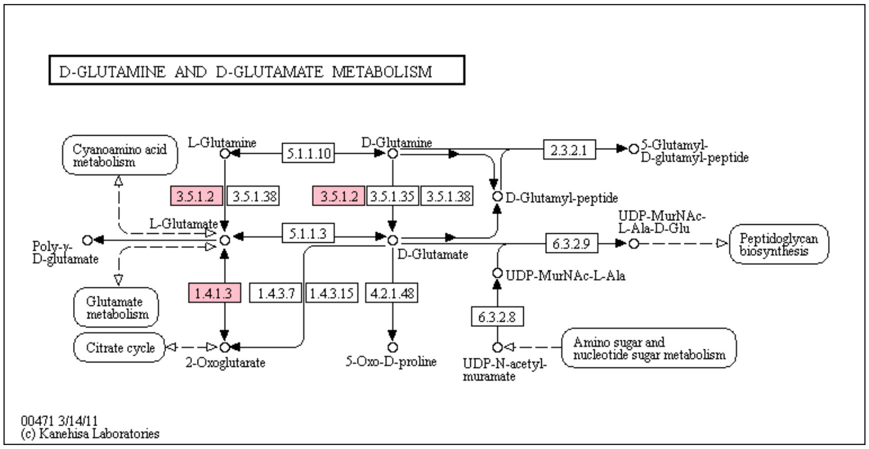

288.3 Legend:

RBH-Blast at 60% Identity + 50% Coverage
Green = Hit in *H. sapiens*
Red = Hit in *H. sapiens* and *C. milli*
White = Not in *H. sapiens*

289 Valine, leucine and isoleucine biosynthesis

289.1 Human Pathway: HSA00290

289.2 Number of Hits: 2

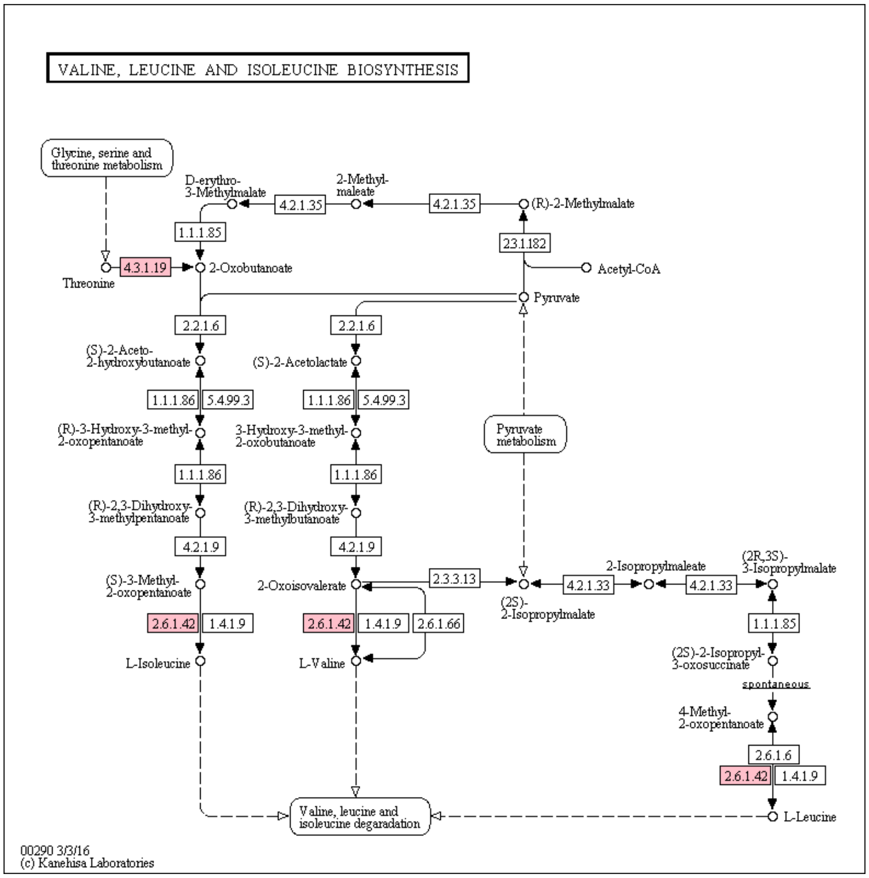

289.3 Legend:

RBH-Blast at 60% Identity + 50% Coverage
Green = Hit in *H. sapiens*
Red = Hit in *H. sapiens* and *C. milli*
White = Not in *H. sapiens*

290 Lipoic acid metabolism

290.1 Human Pathway: HSA00785

290.2 Number of Hits: 1

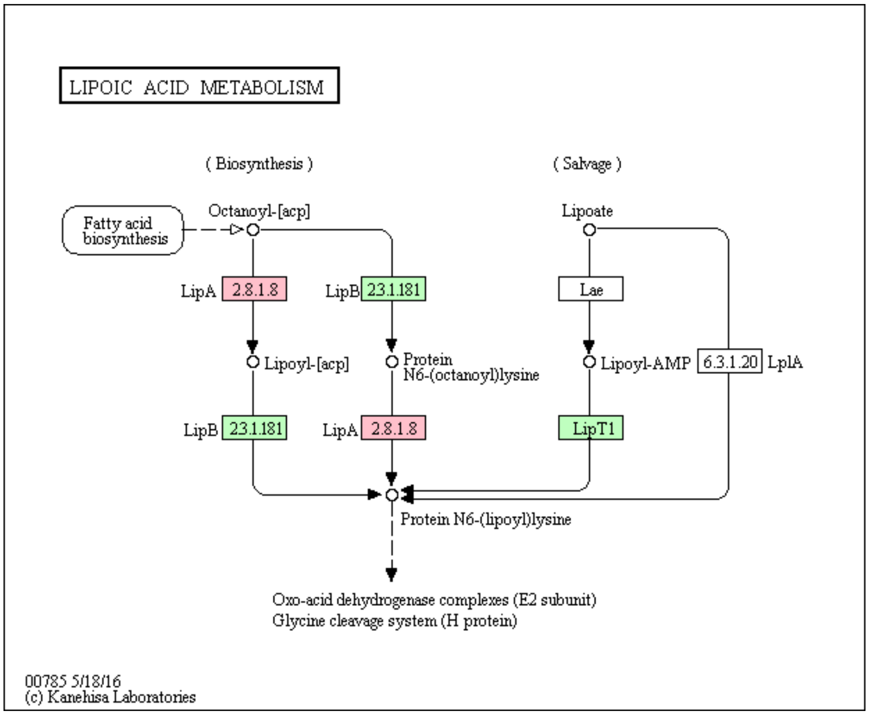

290.3 Legend:

RBH-Blast at 60% Identity + 50% Coverage
Green = Hit in *H. sapiens*
Red = Hit in *H. sapiens* and *C. milli*
White = Not in *H. sapiens*

291 Biotin metabolism

291.1 Human Pathway: HSA00780

291.2 Number of Hits: 1

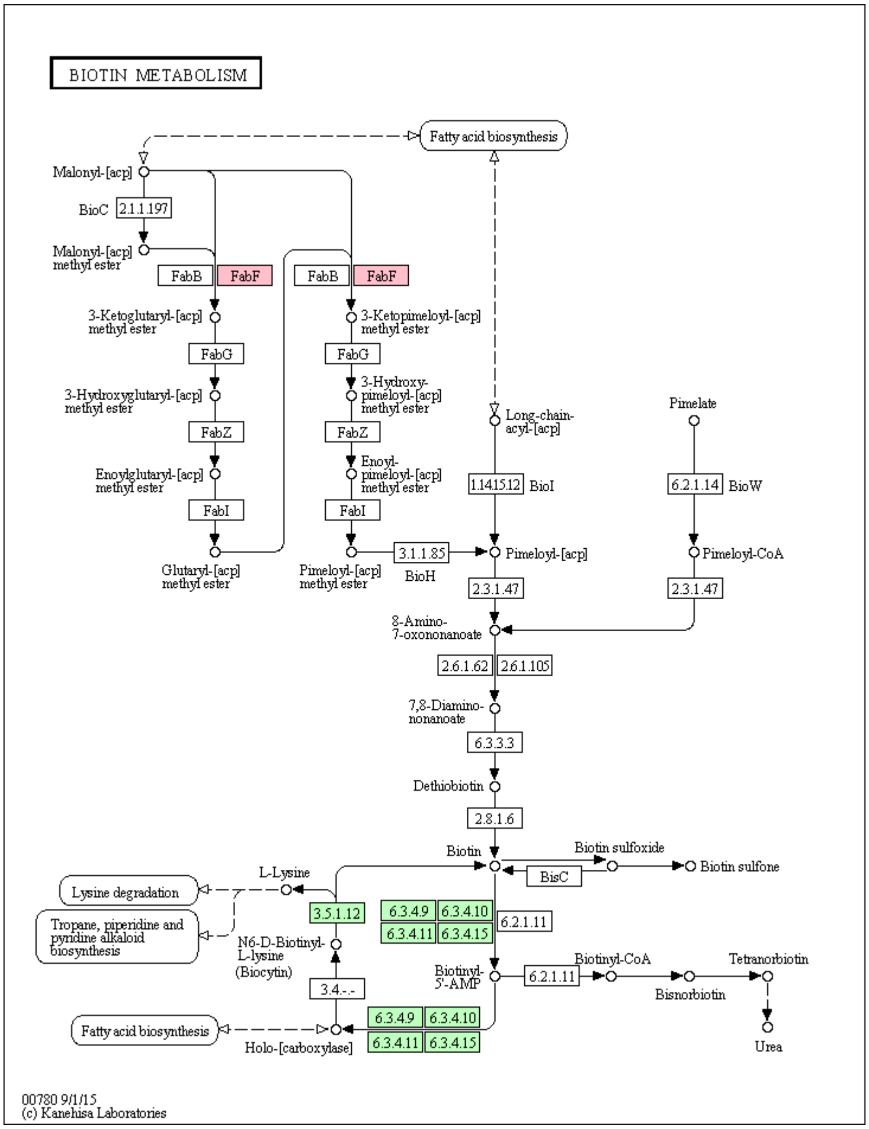

291.3 Legend:

RBH-Blast at 60% Identity + 50% Coverage
Green = Hit in *H. sapiens*
Red = Hit in *H. sapiens* and *C. milli*
White = Not in *H. sapiens*

292 Riboflavin metabolism

292.1 Human Pathway: HSA00740

292.2 Number of Hits: 1

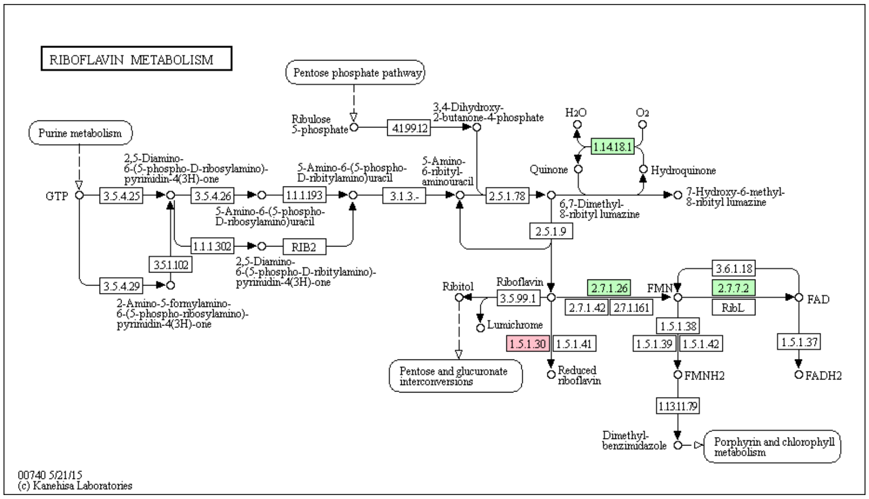

292.3 Legend:

RBH-Blast at 60% Identity + 50% Coverage
Green = Hit in *H. sapiens*
Red = Hit in *H. sapiens* and *C. milli*
White = Not in *H. sapiens*

293 Autoimmune thyroid disease

293.1 Human Pathway: HSA05320

293.2 Number of Hits: 1

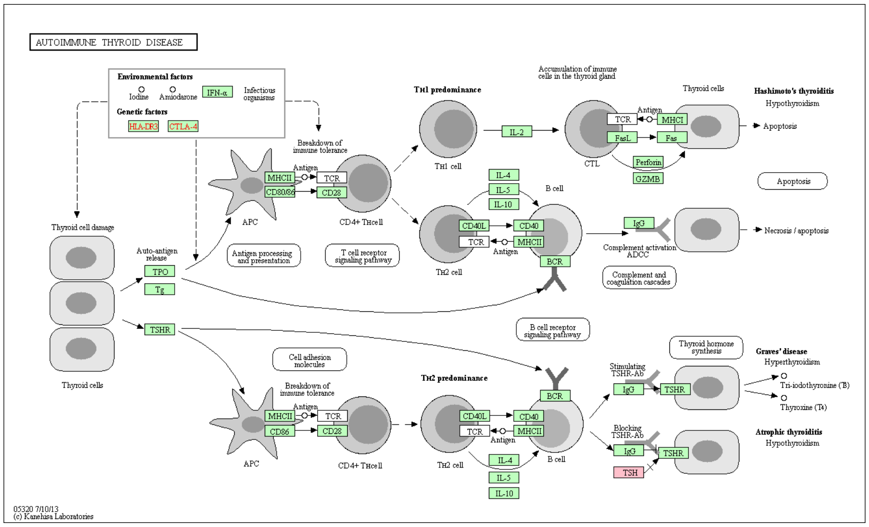

293.3 Legend:

RBH-Blast at 60% Identity + 50% Coverage
Green = Hit in *H. sapiens*
Red = Hit in *H. sapiens* and *C. milli*
White = Not in *H. sapiens*

294 Phenylalanine, tyrosine and tryptophan biosynthesis

294.1 Human Pathway: HSA00400

294.2 Number of Hits: 1

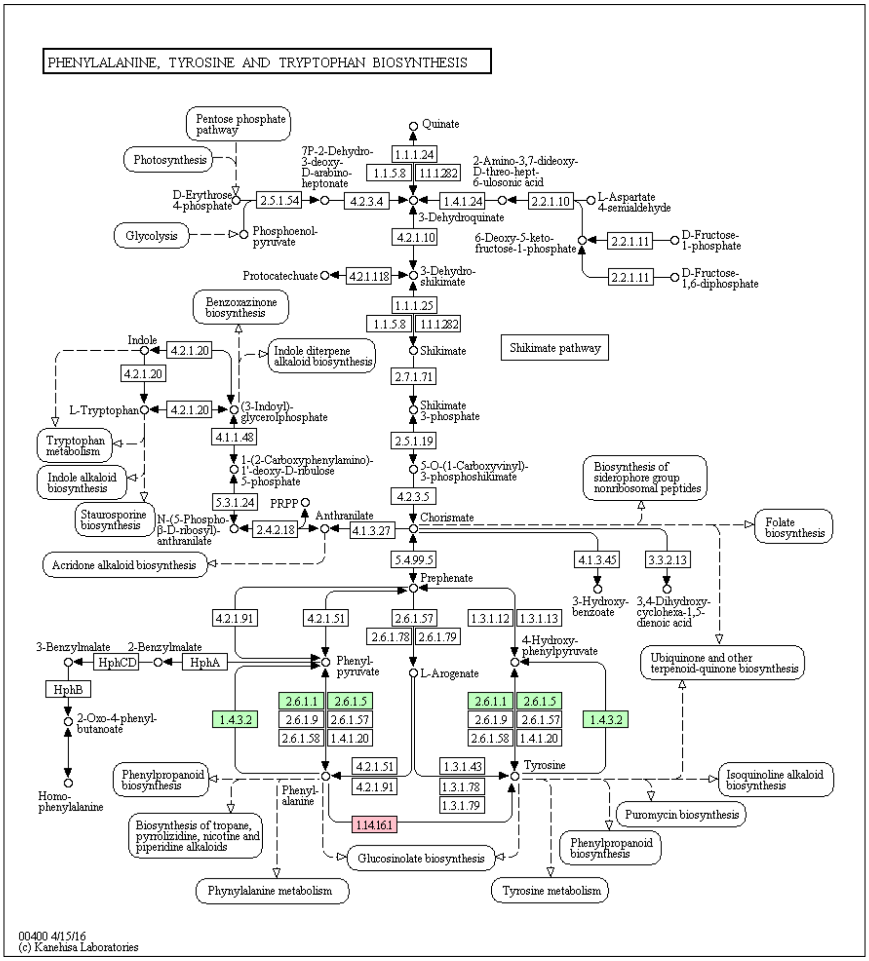

294.3 Legend:

RBH-Blast at 60% Identity + 50% Coverage
Green = Hit in *H. sapiens*
Red = Hit in *H. sapiens* and *C. milli*
White = Not in *H. sapiens*

295 D-Arginine and D-ornithine metabolism

295.1 Human Pathway: HSA00472

295.2 Number of Hits: 1

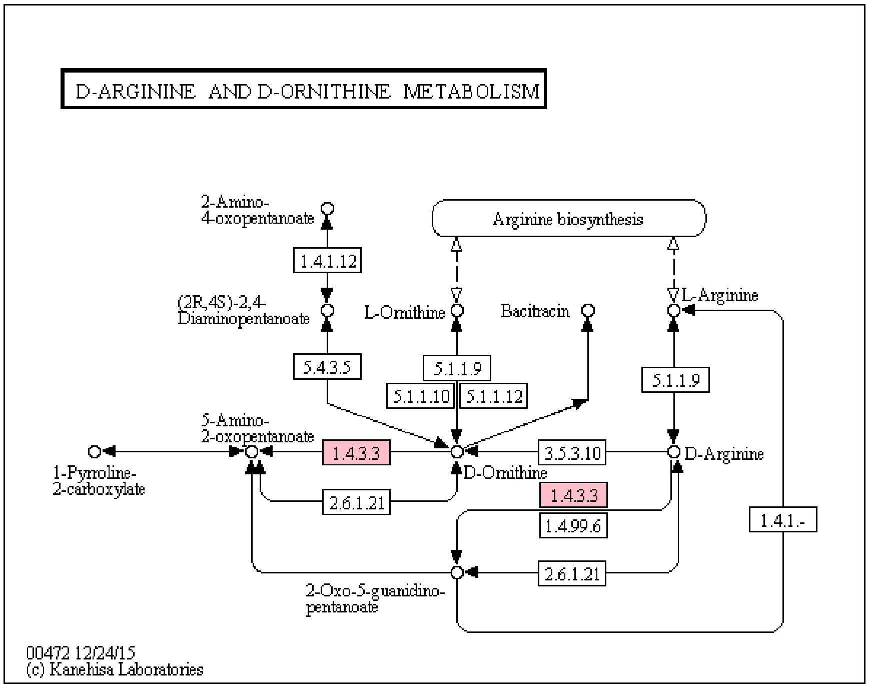

295.3 Legend:

RBH-Blast at 60% Identity + 50% Coverage
Green = Hit in *H. sapiens*
Red = Hit in *H. sapiens* and *C. milli*
White = Not in *H. sapiens*

296 Caffeine metabolism

296.1 Human Pathway: HSA00232

296.2 Number of Hits: 1

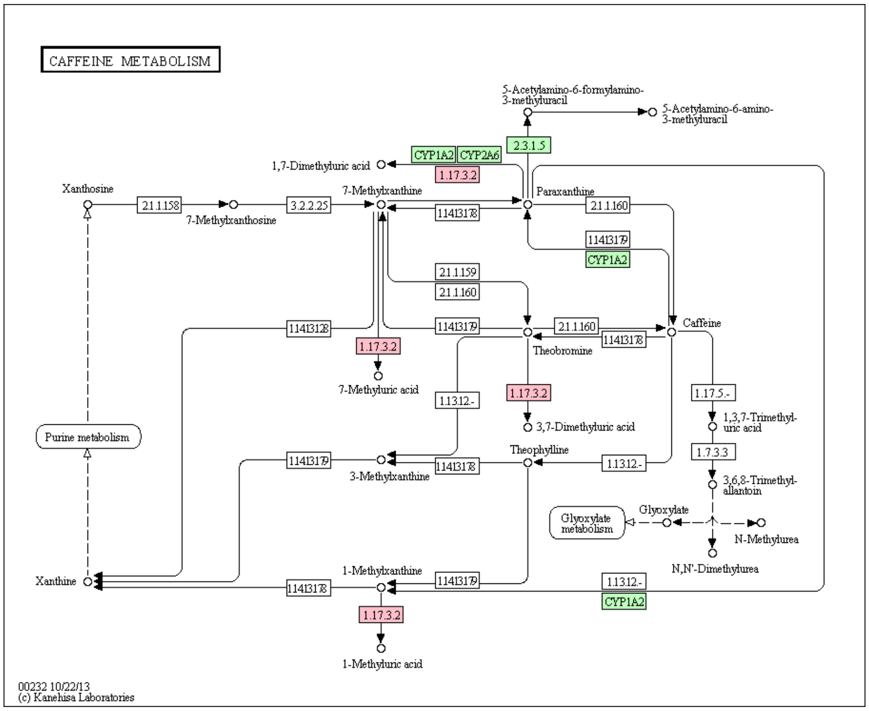

296.3 Legend:

RBH-Blast at 60% Identity + 50% Coverage
Green = Hit in *H. sapiens*
Red = Hit in *H. sapiens* and *C. milli*
White = Not in *H. sapiens*

297 Hematopoietic cell lineage

297.1 Human Pathway: HSA04640

297.2 Number of Hits: 1

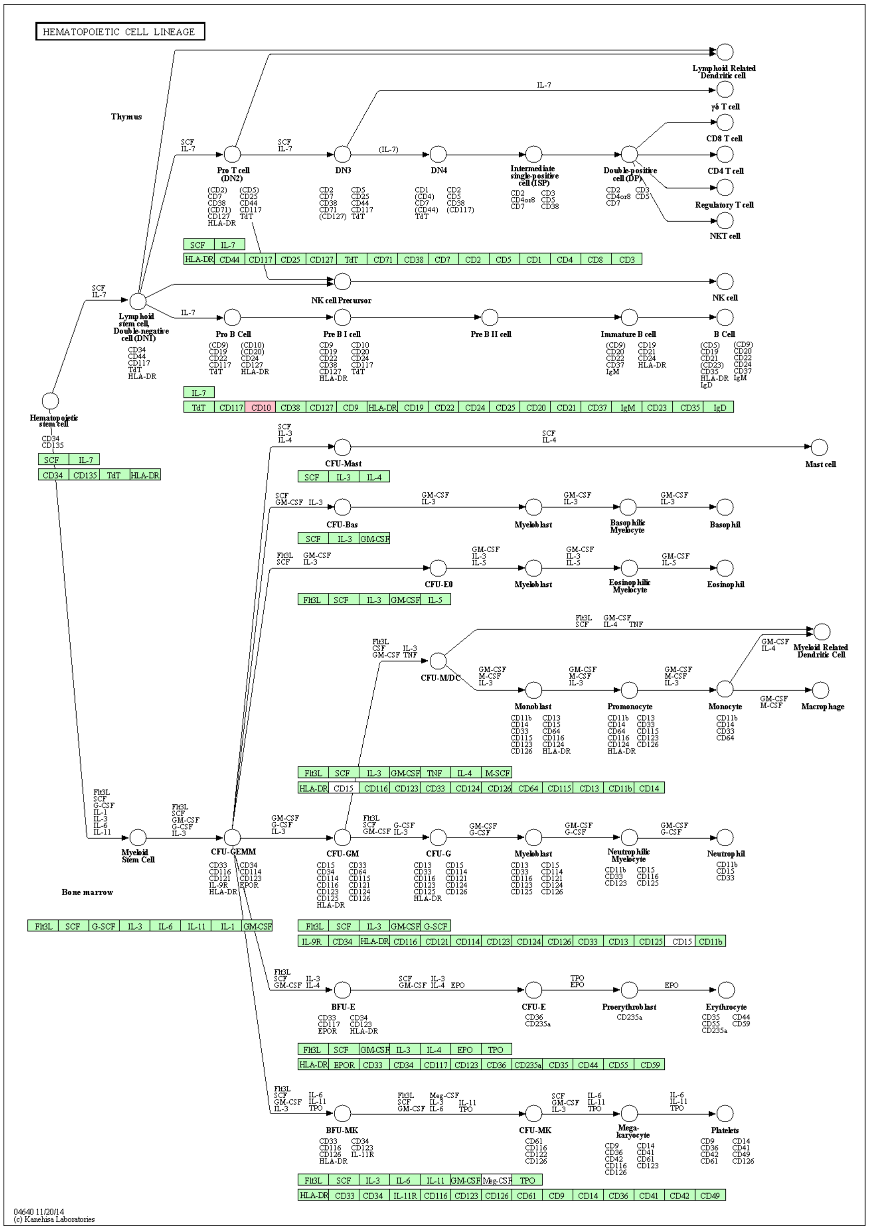

297.3 Legend:

RBH-Blast at 60% Identity + 50% Coverage
Green = Hit in *H. sapiens*
Red = Hit in *H. sapiens* and *C. milli*
White = Not in *H. sapiens*
